# The Concept of Neuroglia ‐ the State of the Art Circa 1900

**DOI:** 10.1002/glia.24678

**Published:** 2025-02-04

**Authors:** Helmut Kettenmann, Bilge Ugursu, Bruce R. Ransom, Christian Steinhäuser

**Affiliations:** ^1^ Department of Neuroscience Shenzhen University of Advanced Technology Shenzhen China; ^2^ Max‐Delbrück‐Center for Molecular Medicine in the Helmholtz Association Berlin Germany; ^3^ Chinese University of Hong Kong Sha Tin Hong Kong; ^4^ Institute of Cellular Neurosciences I Medical Faculty, University of Bonn Bonn Germany

**Keywords:** Held, Lenhossek, neuroglia, Retzius, Weigert

## Abstract

Glial cells were first defined by Rudolf Virchow in 1856. About 40 years later, glial research had developed into a field distinct from the mainstream study of neurons as the central elements governing brain function. By that time, substantial knowledge about the properties of glial cells had accumulated, exemplified by five important publications by four distinguished investigators: Gustav Retzius, Michael von Lenhossek, Carl Weigert, and Hans Held. These treatises broadly summarized what was known about glial cells, comparing findings from leeches to humans. Practically speaking, these articles represent the foundation of our current knowledge. All five contributions were published in German, which at the time was one of the dominant languages for scientific exchange. This article summarizes and comments on their findings and thus provides insight into what was known about glial cells at that time. More importantly, in the Supporting Information, we provide English translations and original scans of these five publications, making them accessible to an international readership.

## Main Points

- Retzius, Lenhossek, Weigert, Held illuminate glial research circa 1900.
- English translations provided.
- They analyze glia during vertebrate evolution, including human, and relate it to development.
- They compare grey and white matter, glial relationships to vessels, brain surface.

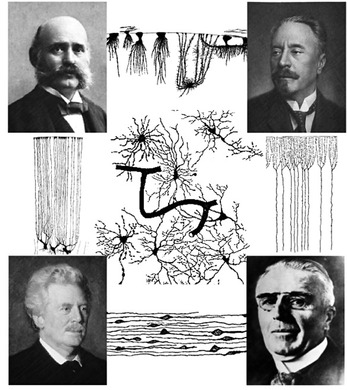

## 1 Introduction

Rudolf Virchow defined neuroglia as a population of cells distinct from neurons in the central nervous system in 1856 (Virchow 1856). Two years later, he published the first image of what he called a neuroglial cell (Virchow 1858). These cells did not resemble typical macroglial cells but instead appeared similar to activated microglia. Inspired by this novel concept, almost all prominent neuroscientists not only focused on neurons but also studied neuroglial cells. This was an international effort by the European community, spearheaded by Camillo Golgi in Italy, Ramón y Cajal in Spain, Louis‐Antoine Ranvier in France, Gustav Retzius in Sweden, and Albert von Kölliker in Germany. These scientists exchanged ideas and concepts, often engaging in disputes.

Almost 40 years after the definition of neuroglia, three summarizing accounts on the concept of neuroglia were published between 1893 and 1895. The first consisted of two overview articles: “Studies on Ependyma and Neuroglia” in 1893 (*Studien über Ependym und Neuroglia*) and “The Neuroglia of the Brain in Humans and Mammals” (*Die Neuroglia des Gehirns bei Menschen und bei Säugethieren*) in 1894, both by Gustav Retzius in his “Biological Investigations” (*Biologische Untersuchungen*), a series of articles he published acting as his own publisher. In 1895, another publication appeared as a large chapter on the “Support Cells of the Spinal Cord” (*Die Stützzellen des Rückenmarkes*) in the textbook “The Detailed Structure of the Nervous System in the Light of the Newest Research” (*Der feinere Bau des Nervensystems im Lichte neuester Forschungen*) by Michael (Mihály) von Lenhossek (Lenhossek 1895). In the same year, Carl Weigert published his book, “On the Knowledge of the Normal Human Neuroglia” (*Über die Kenntnis der normalen menschlichen Neuroglia*), as part of the proceedings of the Medical Association in Frankfurt/Main.

These three authors summarized the knowledge of glial cells at the time and frequently referenced one another. In 1903, a fourth author, Hans Held, published a book on neuroglia titled “The Structure of the Neuroglia” (*Über den Bau der Neuroglia*), as part of the proceedings of the Royal Saxonian Society of Science (Held 1903). We have also included this publication in our survey, besides the four mentioned above, as it provides additional insight about the knowledge available at that time.

While the term astrocyte was coined by Lenhossek in his publication, the distinction between the three major classes of central nervous system glial cells—namely, astrocytes, oligodendrocytes, and microglia—was not yet made. This critical distinction was finally achieved by Pío del Río‐Hortega, and reported in his remarkable series of four articles published in Spanish in 1919. These articles have since been made available to the international community as English translations (Sierra et al. 2016).

In the historical period under consideration, “circa 1900”, papers on glial cells could still be easily overlooked. For his overview account on neuroglia in 1903, Held cited a total of 85 papers. We have added all references from these five publications as a supplement, which provides all major references related to glial cells at that time—a total of 158. In 2023 alone, 4183 publications appear in PubMed with “astrocyte” in the title or abstract, 1926 with “oligodendrocyte”, and 5145 with “microglia”! Similarly a search on “astrocyte” but without specifying a particular year, PubMed finds an astonishing 80,000+ articles. Thus, in our era, glial research has reached a point where no single person could reasonably oversee the entire field of glial research.

The five historic accounts under consideration were published in German, including the articles by Gustav Retzius. One may wonder why the Swedish scientist Retzius published in German. He provides an explanation in the introduction to *Anatomical Investigations*, written in 1872 (Retzius 1872): “I publish these in a language that is not my mother tongue, based on the insight that such highly specialized scientific studies must target a larger audience to have an impact greater than a publication in Swedish would achieve. I chose the German language since the science of anatomy is now best cultivated in Germany, and therefore the terminology is best developed in this language. If there should be language errors in my reports, may they be excused, as I wrote them myself in the German language.” In a similar vein, since scientific literature is now predominately communicated in English, we find Rezius's rationale compelling and recommend his argument, substituting ‘English’ for ‘German’, to non‐native English‐speaking students.

We have now translated these historic publications into English. You will find both the translations and the scanned originals in the supplement. In translating these articles, we have chosen to compromise between readability and originality. Often, a single sentence in German would span almost half a page, meaning that by the time one reached the end of the sentence, the beginning would have been forgotten. Hence, we sometimes divided individual German sentences into two or even more sentences in English. We have also added comments to better clarify the content. In addition, we have cut out the figures from the tables of Retzius, Held, and Weigert and inserted them into the translated text wherever they were cited. This makes it possible to see the referenced figures alongside the text, as opposed to looking for them elsewhere in the article. Of course, the original tables can still be found in the scanned originals.

In what follows, we will present the highlights, controversies, and insights of these scientific accounts from their time. These four authors presented their own findings but always placed them in the context of existing literature and extensively cited their peers. Clearly, there must have been significant intellectual exchange, mainly on the European continent, at that time. In this pre‐email, pre‐internet era, scientific meetings and lab visits were critically important, despite the fact that traveling was more cumbersome than it is today. On the other hand, the community of neuroscientists was smaller compared to today, making it more manageable to maintain an overview.

## 2 The Big Controversy: Neuroglia Versus Neuroglial Cells

**FIGURE 1:**
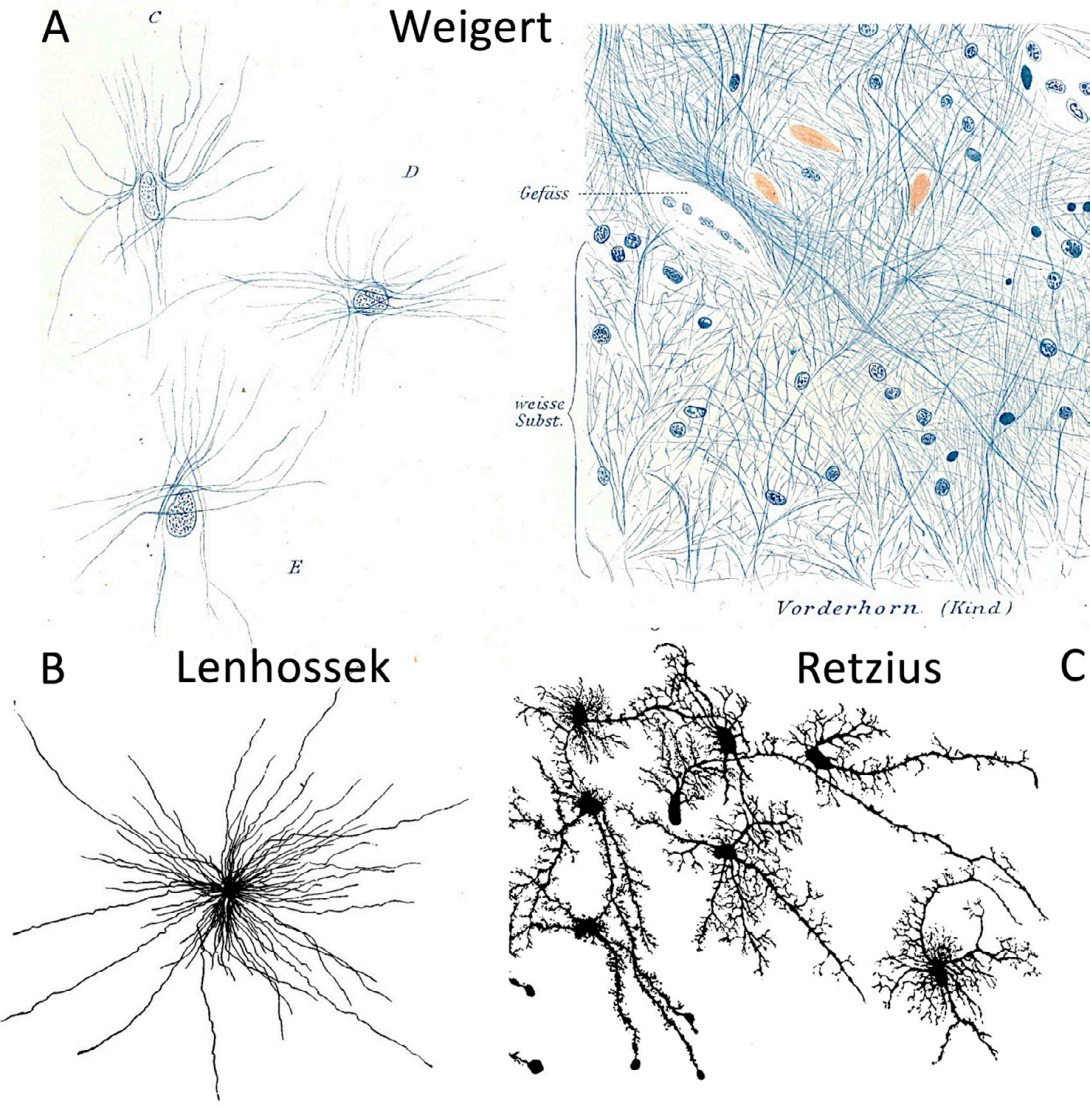
Neuroglia versus neuroglial cells. (A). Image from Weigert (1895) with free nuclei and glial fibers drawn isolated (left, origin not specified, from tab. I, Figure 1) or in a tissue context from (right, dorsal horn of a new‐born child, from tab. II, Figure 1). (B) Image from Lenhossek (1905), (fig. 20). Spider cell from the spinal cord of a three month old child. (C) Image from Retzius (1894), (tab. I, Figure 2). Two Tangential section of the cortical surface of a 6 1/2 month old human fetus) with neuroglial cells.

To visualize neuroglia, different staining techniques were applied. These techniques involved the use of chemical reagents and the resulting chemical reactions following tissue fixation. The limitations of these techniques led to a fundamental controversy regarding the nature of neuroglia. Carl Weigert developed a staining method that labeled fibers and nuclei in human brain tissue. He claimed that this fiber structure represented neuroglia. He postulated that the nuclei of glial cells were embedded in a matrix and that the glial fibers were structures independent of cells. According to his concept, the fiber structure constituted neuroglia. Independent of the scientific conclusions, Weigert's drawings of the images he saw are beautiful (Figure 1A).

In contrast, Retzius, Lenhossek, and Held relied on the silver impregnation technique developed by Camillo Golgi in 1871 and proposed the concept that glial cells are encapsulated by a cell membrane, with the nucleus residing in the soma and surrounded by protoplasm, and that the glial fibers are embedded in the processes originating from the cell body. Thus, they supported the concept that a glial cell is an independent entity. They described in very vivid language and with astonishing morphological precision what they had seen under the microscope, and the images are excellent two‐dimensional reproductions. The multi‐branched, bushy structure of astrocytes can be clearly recognized (Figure 1B,C).

In today's view, Weigert's concept is considered erroneous, as clearly stated by Lenhossek. The Golgi technique, however, also has its disadvantages, as pointed out by Weigert. It only selectively stains a subpopulation of cells, and this selectivity remains an enigma to this day. In contrast, the Weigert technique appears to label the entire neuroglial fiber structure. Thus, Weigert's account has value in that it describes the fiber system in all major brain regions and indicates that what he defined as neuroglia is present in all regions of the brain.

## 3 Lenhossek and Retzius Provide a Clear Picture of Macroglial Cells

Another feature of the Golgi stain is that it predominantly labels macroglial cells (besides neurons), namely astrocytes and oligodendrocytes, but not microglia. In fact, a staining technique that reliably labeled microglia would not be available until Hortega's discovery of such a technique, approximately 25 years later. Thus, this initial period of glial studies focused on macroglia.

Lenhossek's chapter focused on the spinal cord, but essentially all the major features of glial cells are similar in other areas of the central nervous system. He defined glia as “highly ramified, star‐shaped, delicate structures that are widely distributed in gray and white matter. These cells form the support structure of the spinal cord; they are not the main structure, but are the only elements described as ‘Neuroglia.’ ‘Neuroglia’ is not actually a separate tissue, but highly ramified, star‐shaped cells embedded in the brain and spinal cord tissue. They are intermingled with the nerve cells in gray matter and with the nerve fibers in white matter, thereby forming a support system. Moreover, there are no separate glial fibers, but only extensions from glial cells, similar to how dendrites are extensions of nerve cells. The astrocytes are completely independent elements and, like neurons, are units by themselves.”

Retzius comments on the development: “The first developing support cells, the ependymal cells, play only a transitory role. In particular, in higher vertebrates, they later regress or remain in a rudimentary state. In the meantime, the neuroglial cells develop from the same type of germ cell. First, they acquire the peculiar, long radial shape of the ependymal cells, but soon transform into the different types of neuroglial cells proper.”

There was no consensus about glial cell terminology at this time. Lenhossek, Retzius, and Held alternatively used the terms glial cells, Deiters cells, spider cells, or astrocytes to refer to the same type of cells. Lenhossek advocated for the term astrocytes:“The term ‘spider cells’ introduced by Jastrowitz has become fashionable and describes their features. However, Gierke's argument should be considered: nobody has seen spiders with as many legs as these cells have processes. I must confess that this name for astrocytes is, among the German terms, the better one.”

## 4 Glial Cells Are Recognized as Support Cells

Pedro Ramón, Santiago Ramón y Cajal, and C.L. Sala made functional definitions of nerve and glial cells. As summarized by Lenhossek: “These different features mirror the functional differences between these two cell types: the nerve cells represent the functional elements for the multiple nervous features embedded in their protoplasm, while the astrocytes represent elements without internal movement, fulfilling supportive functions or forming structures.” Likewise, Held stated: “Thus, not only are the protoplasmic extensions of the nerve cells relevant for nourishing the rest of the nerve cell, but also the glia, which, on one hand, determines the lymphatic spaces with its marginal side and, on the other hand, contacts the free areas of the nerve cell protoplasm. It can thus be considered a peculiar feeder of the nervous substance besides its function as a support structure.”

It was also evident at that time that glial cells in white and gray matter may have distinct functions. Lenhossek recognized that “the astrocytes of the white matter all belong to the category of typical Langstrahler. What was most obvious to me is that they are not much different from the astrocytes in gray matter. This is surprising because they serve … another purpose compared to gray matter. In gray matter, the nerve cells and the support cells are intermingled. The complex, irregular processes of the nerve cells and their spiky cell bodies are enwrapped by astrocyte processes, forming a basket‐like structure. In white matter, they behave very differently. Here, the astrocytes and their processes form a kind of regular meshwork that circumvents the longitudinal fibers.”

As they had not yet differentiated between astrocytes and oligodendrocytes, he might have been referring to oligodendrocytes. Held states in this context: “For this first function of the glial cells (nutrition), I would still like to mention the studies by Wlassak (1898), who attributes to the primary and secondary glia an importance as storage and transfer structures providing substances for the myelin cover from the blood.” Thus, Held perceives that glia not only transfer nutrients but also store them. It is also interesting that glia were believed to support myelin. At the time, it was not yet known that oligodendrocytes form myelin. This was only recognized many decades later, in the 1960s. Today we know that the network of macroglial cells, astrocytes and oligodendrocytes, are essential elements for providing control of metabolic support to neurons. Astrocytes are connected to the blood system via endfeet and neuronal activity controls this energy transfer, ultimately by alteration of blood flow into nutritive capillaries (Mishra et al. 2024). Astrocytes also contain glycogen which is broken down and released as the neuronal nutrient lactate during neural activity (Brown and Ransom 2007). This macroglial network is interconnected by gap junctions and oligodendrocytes also play an essential role in the energy support of axons in white matter (Nave, Asadollahi, and Sasmita 2023; Lee et al. 2012).

At that time, electrophysiology was restricted to detect neuronal activity and physiological studies on glial cells were not performed before the 1960's. Today we know that astrocytes, in particular, closely interact with neurons at the synaptic level, sense neuronal activity by expressing neurotransmitter receptors and feed‐back on the neuronal network (Volterra and Steinhäuser 2004; Bergles, Jabs, and Steinhäuser 2010). Recent work shows that specialized astrocytes mediate glutamatergic transmission (de Ceglia et al. 2023). Also, microglia express transmitter receptors (Pocock and Kettenmann 2007) and accumulating evidence indicates that they are involved in synaptic pruning (Kettenmann, Kirchhoff, and Verkhratsky 2013; Sierra, Paolicelli, and Kettenmann 2019).

## 5 On the Developmental Origin of Neuroglia

It was the consensus among most scientists before 1900 that glial cells were not mesodermal but ectodermal in origin. It should be noted that this specifically refers to macroglial cells, namely astrocytes and oligodendrocytes. As summarized by Lenhossek: “The supportive cells originate from the same *Anlage* as the nerve cells. This does not mean that they are considered nervous in the physiological sense. Only the origin is common, while with regard to functional aspects, there is differentiation into neurocytes, that is, cells characterized by outgrowth of a nerve and related to nervous functions. These elements, which send a rough process to the periphery of the spinal cord, have features of epithelial cells. This is the ependymal stage of astrocytes. Hence, astrocytes, which develop in this fashion, undergo three developmental stages: first, the stage of the ependymal cell; then, through eccentric dislocation of the cell body, the stage of radial astroblasts; and from this stage, they finally develop into spider cells by the atrophy of the process. It should be mentioned that degeneration of the ependymal structure is only found in higher vertebrates, while in lower vertebrates, the ependymium plays a lifelong and important role.”

**FIGURE 2:**
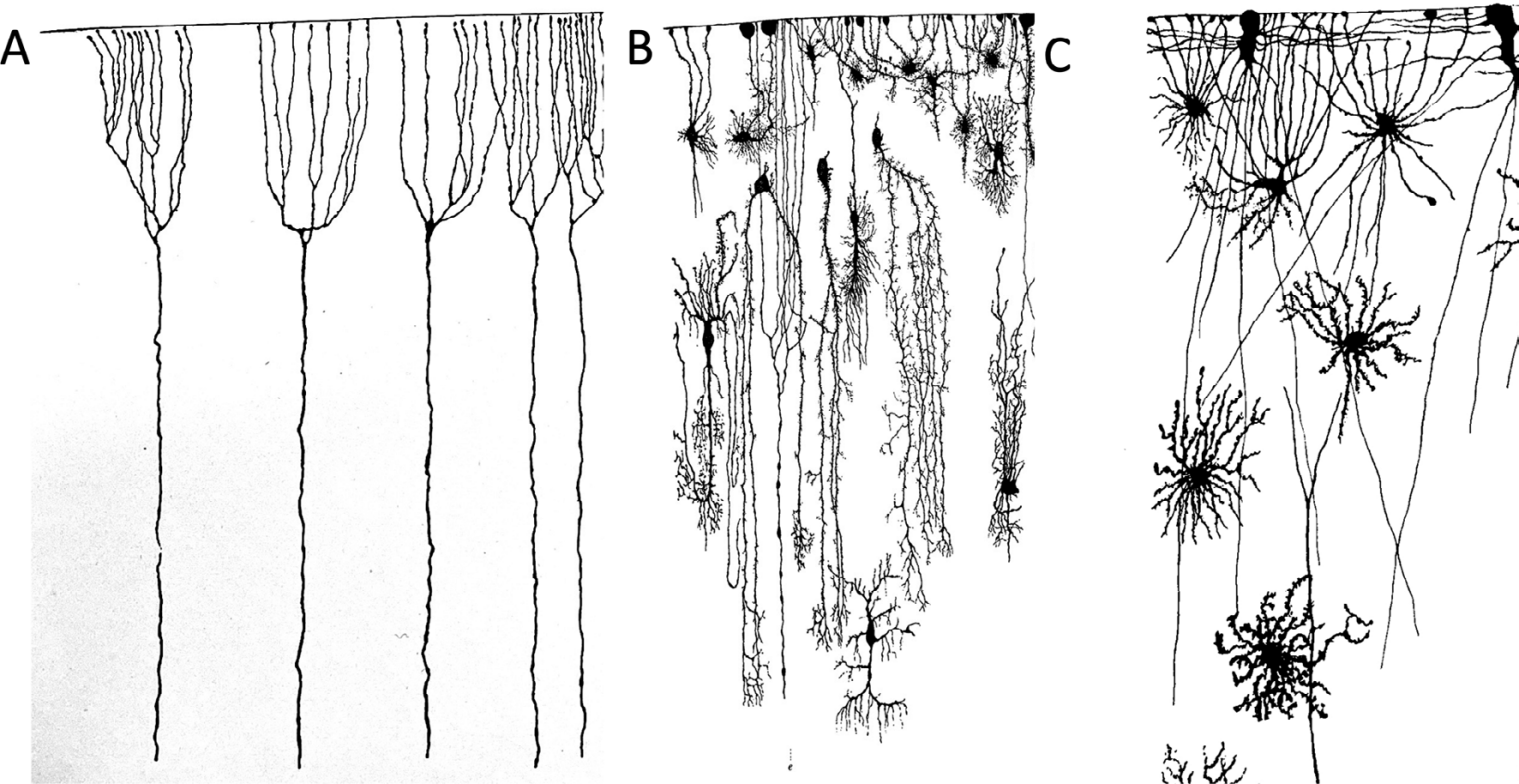
Development from ependymal/radial cells to glial cells. (A) Vertical section of the cerebral cortex (frontal lobe) of a 19.5 cm long human embryo. Outer ends of the ependymal/radial cells (from Retzius 1894, tab. I, Figure 4). (B) Vertical section of the cerebral cortex of a 6 1/2 old human fetus. Different types of neuroglial cells in their natural position in the cortex (Retzius 1994, tab. I, Figure 1). (C) Border region of the pial sheath from a cross section of optic nerve from a 11 cm. long cat fetus (Retzius 1894, tab. IV, Figure 3).

There was also recognition that not all glial cells derive from these radial cells. From today's perspective, the elongated precursor cells of astrocytes are referred to as radial glia (embryonic stage) or radial glia‐like cells (postnatal stage), while ependymal cells are regarded as differentiated, postmitotic glial cells. Lenhossek argued that “the number of spider cells in the human spinal cord is far too large for them to have descended from the earlier radial cells, which are much less frequent. I would like to propose that for many of the spider cells, particularly those in the gray matter, the complicated developmental mode has been cainogenetically replaced by a much shorter and simpler one in which they do not pass through the radial fiber stage. Instead, they develop in the inner layers of the cord or in the more peripheral regions where they are later found, originating from germ cells as small, initially process‐less cells, and transforming into typical astrocytes surrounded by many processes. Regardless of whether the astrocytes develop through one mode or the other, there is no difference in their outer appearance. The key point is that all are derived from the same descendants. Both forms—those developing from radial cells and those originating directly—are ectodermal elements.” Retzius compared the different forms of neuroglial cells that emerged from these radial cells, emphasized the morphological uniformity of the latter and added careful anatomical explanations to his extraordinarily delicate drawings (Figure 2).

Today it is evident that the early progenitor cells which give rise to neurons, but also to the macroglial cells share many properties with astrocytes and that these are quite distinct in humans (Namba and Huttner 2024). It has also become evident the microglial cells have an origin distinct from macroglial cells (Greter and Merad 2013).

## 6 Studies on Human Neuroglia

These early researchers obviously had good access to fresh human material, including human embryos, brains of children of different ages, and adults even up to 70 years of age. Hans Held even analyzed brains from young, executed males in their twenties—a practice that is considered unethical today. The Weigert study focused exclusively on humans since his staining technique was human‐specific. Retzius devoted one of his two chapters to human brains at different developmental stages.

As described in the section on evolution, in the early embryonic stages of humans, radial cells span from the dorsal to the ventral surface. It was evident that these cells disappear during later embryonic development. In a 6 ½‐month‐old fetus, Retzius found cells typical of astrocytes in the cortex. He demonstrated that these cells contact blood vessels through the formation of endfeet. He analyzed several embryonic stages, including newborns, a few‐month‐old children, and up to 5 years of age. He stated: “Already at the end of the first year and continuing in the following years, the neuroglial cells of the cerebral cortex acquire their final, terminated shape.”

He described and depicted a large variety of morphological forms at these different stages. As shown below, Retzius developed a complex terminology to describe the different morphological subtypes of neuroglial cells. An interesting finding was that he detected these forms in a 1‐year‐old child and found the same pattern at the ages of 5, 8, 17, 33, and 42 years, indicating that cell diversity neither increased nor decreased from childhood to middle age. He even found morphological diversity in a 70‐year‐old man. However, he also noted subtle changes at later ages. For a 42‐year‐old woman, he stated: “Striking, in particular, are the thick, coarse‐armed Sternstrahler, which we have not found in such a succinct form in younger brains. Finally, one finds at the blood vessels a sometimes‐occurring unusual form: the cell body is attached to the vessel sheath, but the processes do not surround it; instead, they project in contrast to the brain substance.” For the 70‐year‐old man, he found corresponding conditions but also subtle differences: “What is obvious in studies of neuroglia of the cerebral cortex of elderly individuals is the stiffening of the processes of the elements at the surface. They appear more stretched, coarser and more equipped with less branches.”

Retzius compared that diversity to other mammals, in particular dogs, rabbits, and cats, ranging from 1 month of age to adulthood. He found a similar diversity, with some variations, such as a delayed development in rabbits compared to humans. He extended his studies to other brain regions, such as the hippocampus, cerebellum, medulla oblongata, and pituitary gland. He studied the optic nerve and retina only in embryos and during very early postnatal stages. Based on his human studies, he concluded: “From the description above, it is evident that the glial cells of the brain partially undergo a substantial change in morphology during development. Their substance must, therefore, not be rigid during the fetal period, but rather flexible and transformable, despite the fact that later they are rather stable, particularly with respect to the processes. This stabilization of the glial elements serves to provide a proper support system for the nervous elements; it does not exclude the possibility that glial cells may have other functions since there is living protoplasm around their nuclei. In particular, there could exist influences of glial cells related to the nutrition of the organ, on the composition of the extracellular fluid, and influences of the glial elements on development during the fetal phase. I would like to mention these possibilities, despite the fact that there is not yet proof.”

In the last decades, neuroscience research has moved from these early human studies to rodents as models for brain function like memory formation or brain disease. In the meantime, we have mouse models for any type of brain disease or dysfunction including Alzheimer's disease, epilepsy, stroke and psychiatric diseases like schizophrenia or autism. Yet these models have also shown that there are limitations to understand the complex functions and dysfunctions of the nervous system. Thus, recent research efforts are targeted to develop models, which more resemble the human situation. Glial cells generated from human induced pluripotent stem cells might be a route to move closer to humans (Hasselmann and Blurton‐Jones 2020) and combining this with human organoids promise to create novel models closer to the human situation (Summers et al. 2024). In few cases, glial cells can be investigated in living human tissue, for example, in specimens from epilepsy surgery. These studies have shown that human and rodent glial cells have many similar functional properties (Bedner et al. 2020).

## 7 Studies on the Evolution of Vertebrate Glia

**FIGURE 3:**
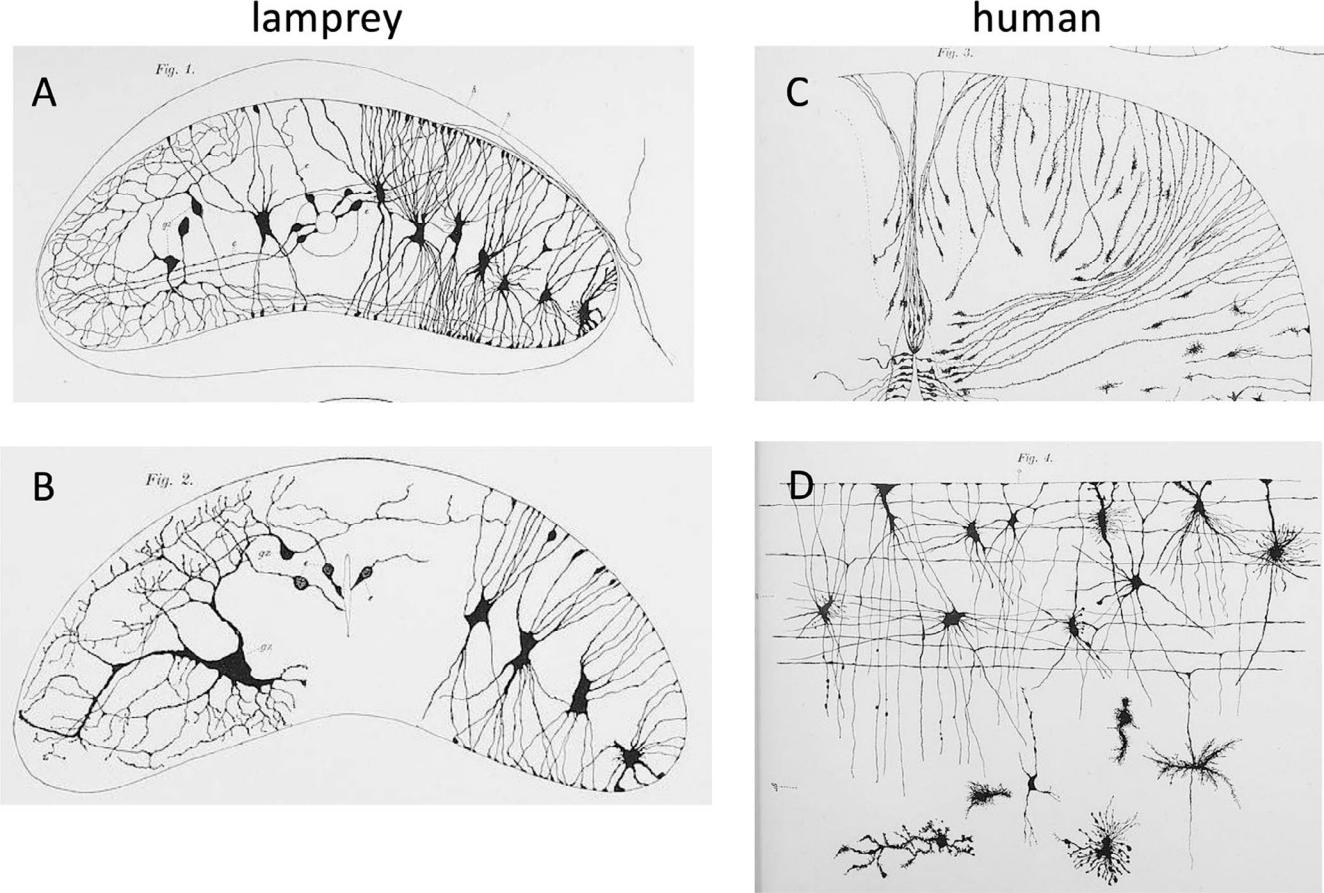
Evolution of glial cells. (A) Cross‐section from the posterior part of the spinal cord of a 4 cm long Petromyzon (lamprey). At the right from the central canal are seven neuroglial cells and on the left of it is a cell illustrated in a stained mode. Around the central canal are five ependymal cells present; − gz, three ganglion cells with their branched processes; − s, sensible nerve roots; − h; contour of the outer sheath (Retzius 1893, tab. V, Figure 1). (B) Cross‐section from the posterior part of the spinal cord of an adult long Petromyzon. On the right are four neuroglial cells, at the central canal three ependymal cells illustrated; gz, two ganglion cells, one small and a large with their branched processes are shown in the left half of the cord. (Retzius 1893, tab. V, Figure 2). (C) Cross section from the spinal cord of a 15 cm long human embryo (neck region). Ependymal and neuroglial cells (Retzius 1893, tab. XI, Figure 3). (D) Region from a vertical, longitudinal section of the ventral root of a 26 cm long fetus. Neuroglial cells in different variants. o—surface of the cord (Retzius 1893, tab. XIII, Figure 4).

Gustav Retzius systematically studied glial cells in all classes of vertebrates. The most primitive ones were jawless fish, specifically the genus *Myxine* (hagfish) and *Petromyzon* (lamprey). These animals were studied by several researchers in parallel, obtaining similar results, including von Lenhossek and Fridtjof Nansen (1861–1930), the latter being better known for his polar expeditions. In these animals, radial cells span from the central canal of the spinal cord to the surface, and this arrangement is found in both larvae and adults. Retzius has documented some examples in his detailed artistic drawings (Figure 3A,B). The cell body of these radial cells is always close to the central canal. Glial cells are also found in the central nervous system, which can send bilateral processes to the dorsal and ventral surfaces. They differ from radial cells with respect to their cell bodies. In higher vertebrates, including humans, such an arrangement is only found in early developmental stages (Figure 3C).

For his study of the development of bony fish, Retzius obtained salmon eggs from a fish farm and raised the animals. In their immature state, their spinal cords are also dominated by radial cells, and this is also the case in amphibians, such as frogs. The development in birds was extensively studied in chickens due to their easy availability, with Golgi and Cajal making major contributions. Among mammals, mice, rats, cats, dogs, and humans were well studied. At early embryonic stages, cells with features of radial cells dominate, including in humans. In humans, Retzius and Lenhossek found that glial cells appeared at later embryonic stages and already had a morphological appearance resembling more mature glial cells. Retzius depicted in his beautiful reconstructions the delicate, elongated structures of the processes of these cells. It can also be well recognized in these drawings that the glial processes from different cells contact each other. As we know today, this is the morphological basis for the formation of the gap junction‐coupled glial syncytium (Figure 3D).

At later stages, the radial cells disappear. The conclusion was that in mammals, radial cells play only a transitory role and are then replaced by glial cells. This led them to conclude that primitive vertebrates remain in a state reflecting early development, while higher vertebrates, particularly mammals, differentiate into a state dominated by glial cells and no longer by radial cells. Held stated this clearly: “In amphioxus, the glial cells remain at this first stage. In the other classes of vertebrates, however, it is typical only for the first stage of brain development. The second stage leads to the secondary cellular glia and its further progeny, which contains, besides the pure and later reduced ependymal cells, transformed ependymal cells and, furthermore, astrocytes. The third stage comprises the development of the glial fibers, which occurs within the tissue of the above‐described purely cellular glia and results in the formation of a secondary cellular‐fibrous neuroglia.”

## 8 Identification of Neuroglia in Invertebrates

**FIGURE 4:**
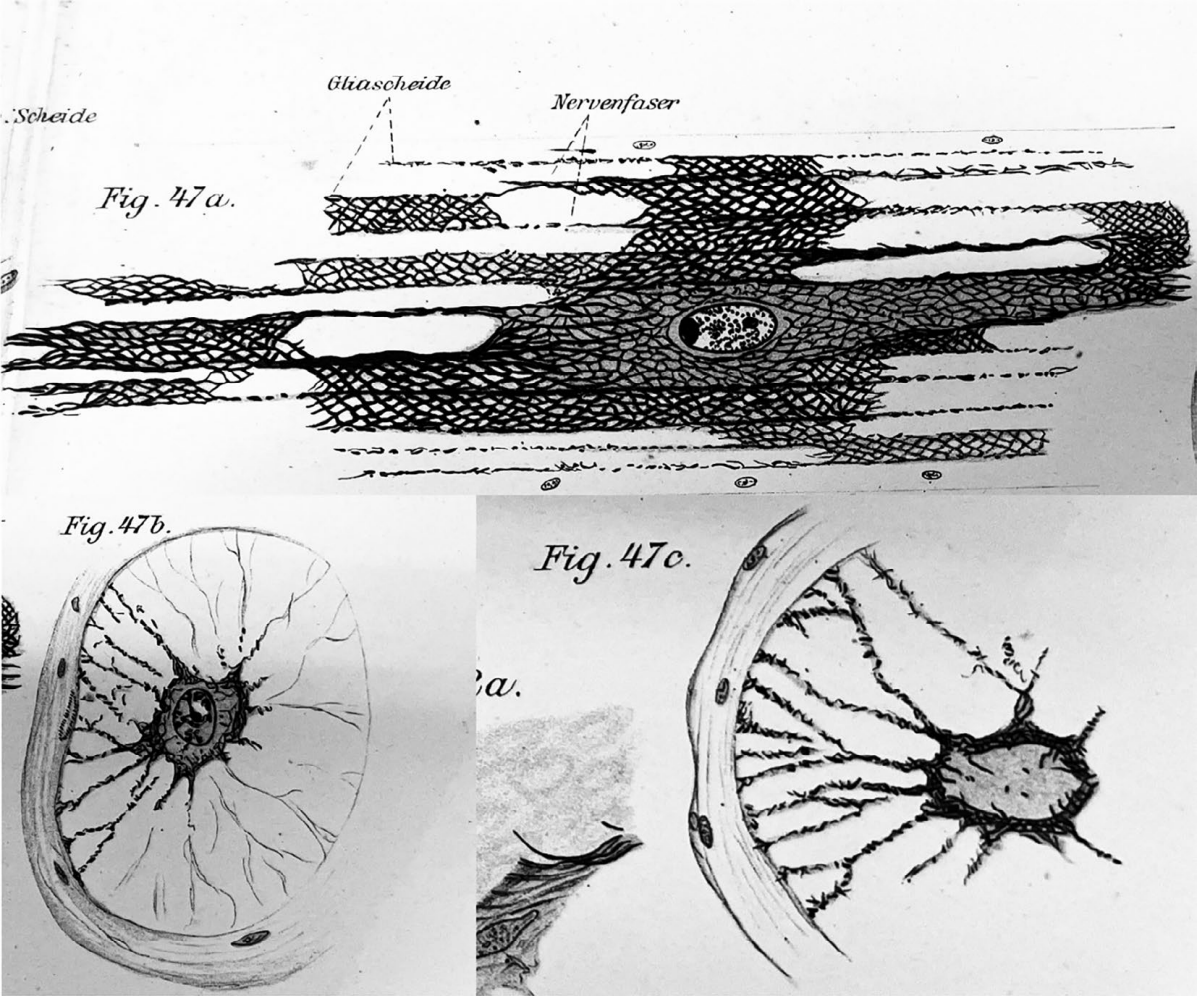
Glial cells from a leech. Longitudinal section and two cross‐sections of a leech connective. Glial cell and its extension to the glial sheath. 'a' from a stretched, 'b' and 'c' from a contracted animal. Alcohol‐chloroform‐acetic acid. Alaun hematoxylin staining according to Heidenhain. (Held, 1905, tab. III, fig. 47).

Held also studied and described glial cells in the leech. The pictures he provides clearly show the leech glial cells, which many years later were studied by Kuffler and Potter (1964). From his carful drawings, it is obvious that he had identified those star‐like cells as neuroglial (as an example, see Figure 4). These are a few of his statements: “I describe some observations on *Hirudo officinalis*. … This mesh‐like glial sheath, as shown in fig. 47a–c, is the long growth differentiation product of a single cell located at the center of the connective… If I compare the nerve fibers of the connective with the white matter of a vertebrate, it appears that what is formed here (in the leech) by one single cell is, in the vertebrate, the fibrous product of a sum of smaller glial cells. … While there (in the leech) a giant glial cell enwraps many ganglion cells, in vertebrates many small glial cells, as accompanying cells, provide the Golgi nets for the corresponding ganglion cells. … The star cells of the ganglia packages provide an outer glial cover for the corresponding ganglion cells, according to Apathy. … They seem to me less as supporting elements for the ganglion cells, but rather as feeding ones.”

## 9 Glial Cells Form the Border Structure of the Nervous Tissue and Isolate Vessels by Endfeet

**FIGURE 5:**
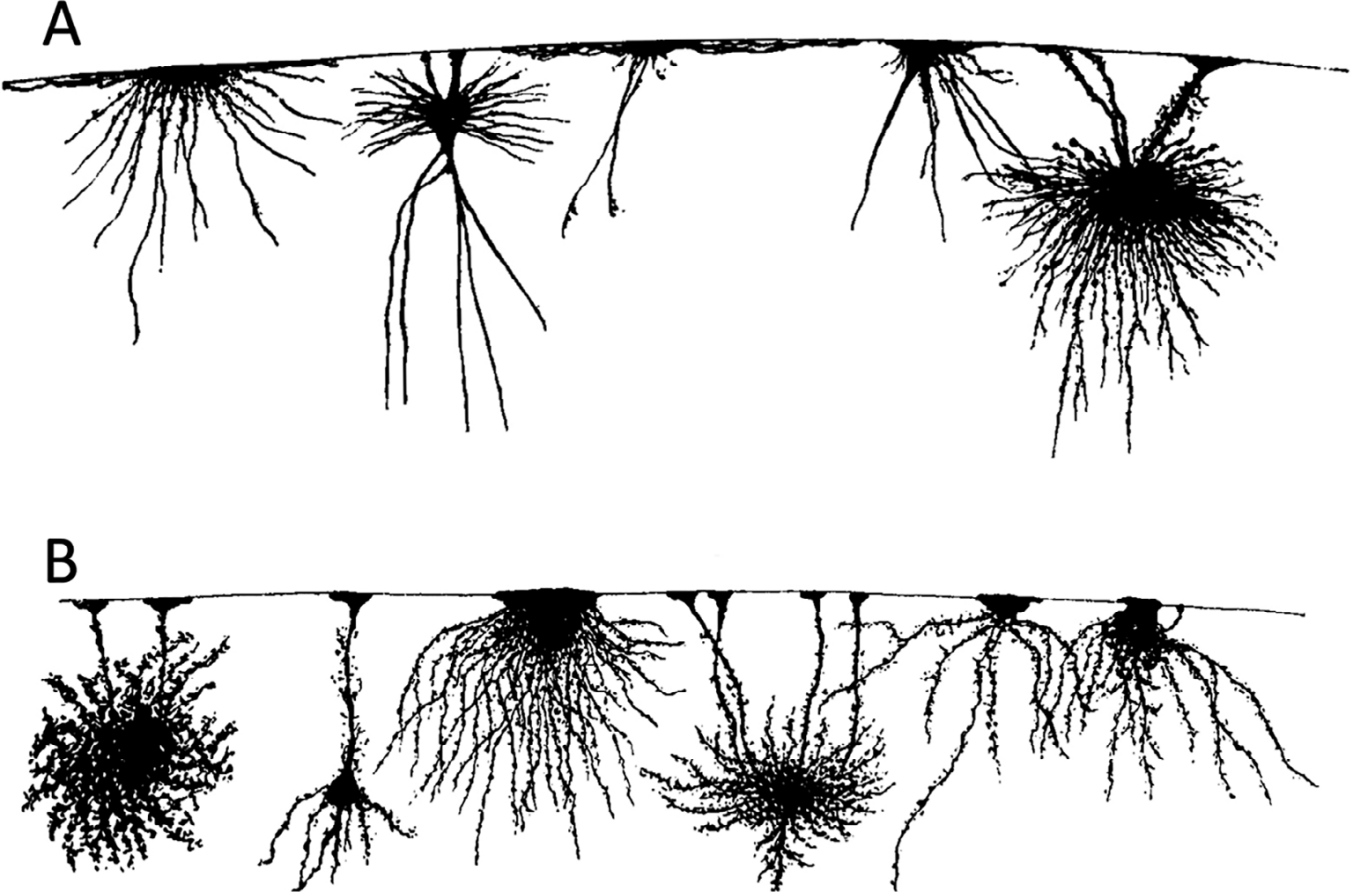
Glial cells form border structure of the nervous system. (A) Vertical section of gyri from the vertex of the cerebrum of an adult (old) dog (from Retzius 1894, tab. VI, Figure 6). (B) Vertical section at the vertex of the cerebrum of an adult (old) rabbit (from Retzius 1894, tab. VIII, Figure 4).

It was evident to these researchers that glial cells form a border membrane both toward the surface of the central nervous system and against the vessels. Lenhossek stated: “It should be noted here that all processes of the spider cells which reach the surface of the cord form small, roundish, or foot‐formed swellings.” These structures are today known as astrocytic endfeet. Held concluded with respect to the endfoot: “I conclude that this membrane of the glial foot is not simply a protoplasmic rest or a transformed substance, but a product of the glial cell with the same relevance as the glial fibers.” Lenhossek further noted: “The complex of these nodules forms the actual surface of the organ. All these fibers, both the radial processes from deeper layer cells or the tangential processes of superficially located peri‐ependymal astrocytes, have thickenings (bulbs) at the end. These bulbs form a mosaic at the surface and actually form a thin and complete border membrane, a kind of cuticula (*Membrana limitans meningea*, His), which covers the ectodermal spinal cord (and isolates it) completely against the pia mater.” Retzius provided several detailed pictures demonstrating that glial cells often have a bushy appearance and form border structures with their endfeet (Figure 5A,B).

**FIGURE 6:**
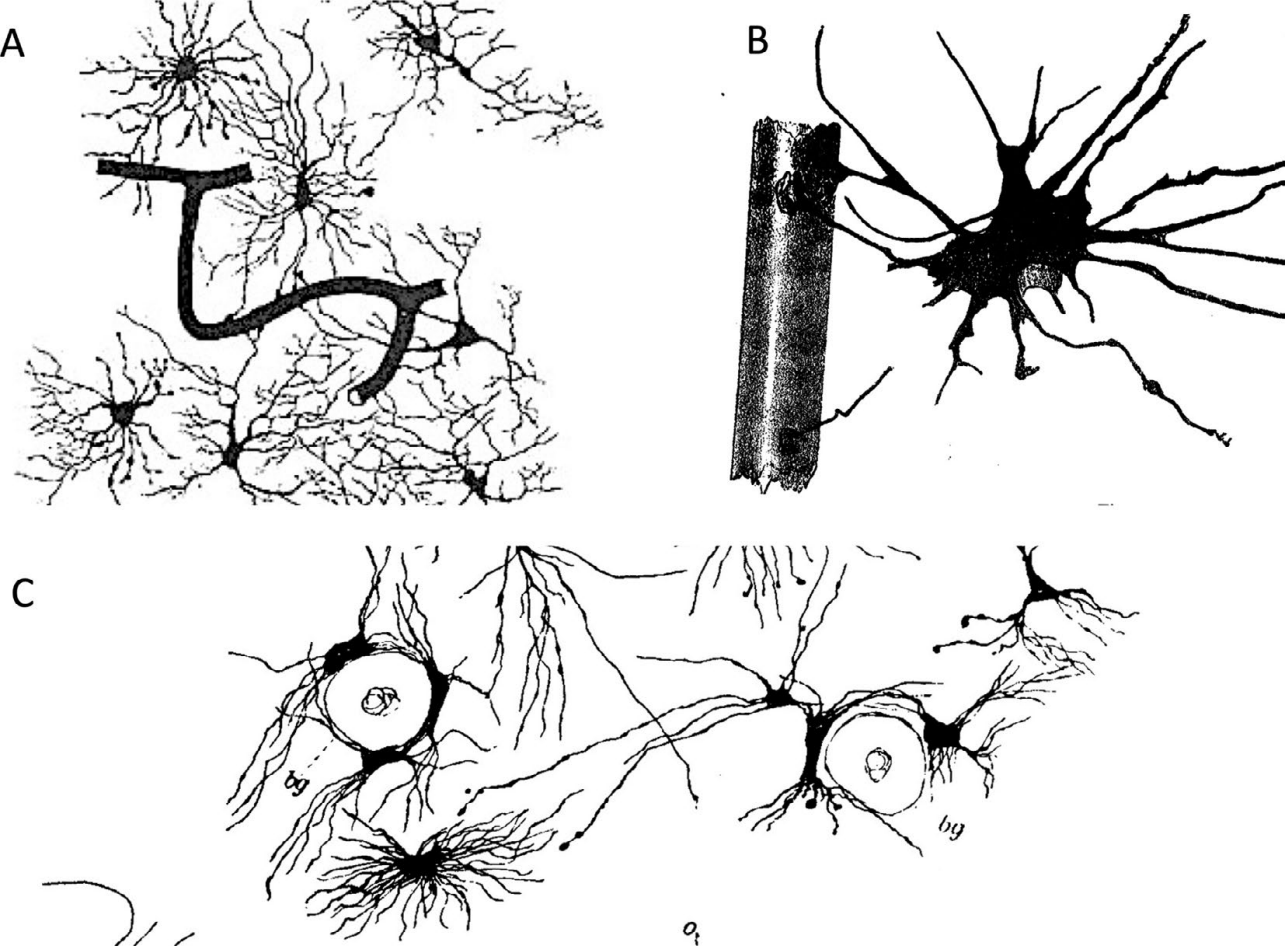
Glial cells from endfeet at blood vessels. (A) Section from the cortex of a 6 1/2 month old human fetus (about 2.5 mm) below the surface; a blood vessel with attached neuroglial cells (Retzius 1994, tab. I, Figure 3). (B) Glial cell from the spinal cord of a few week‐old dog (Held 1905, Text fig. 20c). (C) Optic nerve from a 45 cm, long human fetus; − bg, white matter with cross‐cut blood vessel (Retzius 1894, tab. XIII, Figure 2).

Another border forms around blood vessels and it was clearly recognized that glial cells form defined structures by which they contact the vessels. Retzius and Held provided clear evidence in their delicate drawings. They showed with high resolution that the endings of glial processes touch blood vessels (Figure 6). Lenhossek specifically mentioned the astrocytic endfeet facing the vessels: “If one analyzes, in Golgi preparations, the relationship of the spider cells to the blood vessels, one finds that the former has a similar relationship to the walls of the canals containing larger vessels as to the surface of the cord. If a blood vessel gets in the way of a spider cell, the processes of the latter do not circumvent it, but terminate at the vessel with a thickening similar to what is observed on the surface of the spinal cord. Since processes of the astrocytes come from all directions toward the blood vessel, they form a delicate tube‐like cuticular membrane by which the substance of the spinal cord is separated from the blood vessel.”

Held also commented on that issue: “Thus, the final connections of the neuroglia with the blood vessels of the gray or white matter are mediated by glial endfeet surrounding the vessel, and they are similar to those at the superficial border layer. … In summation, all these endfeet form a special ensheathment around the blood vessel and its adventitious cover, namely the *M. Lim. Perivascularis*, which, according to my observations, represents a complete border including its adventitious sheath between the vessel tube and the central nervous system proper.”

Held also commented on the development of the vascular system: “… in a rabbit embryo. It shows that the continuous ingrowth of the first vessels into the brain simultaneously results in the progressive formation of a *M. limitans* from the deeper layers of the border haze. It separates and isolates, similar to the *M. limitans* at the outer surface, the intruding vessel containing connective tissue from the genuine epithelial mass of the brain. I would like to name this border membrane *M. limitans perivascularis*, which is located in the interior of the brain tube and formed from branches of the spongioblasts. It enwraps the individual vessels as a tube‐like septum and is continuously connected at the superficial entry zone with the border membrane of the brain, the *M. limitans superficialis*.” These histological observations, and how they were interpreted, clearly anticipate our current perspectives about the blood brain barrier. In fact, it seems almost “unscholarly” not to recognize how perceptively early histologists recognized that form likely served function.

## 10 First Thoughts About the Glymphatic System

Recent work from Maiken Nedergaard's group has described a critical waste clearance pathway in the central nervous system, the glymphatic system, facilitated importantly by astrocytes (Jessen et al. 2015). Specifically, the aquaporin‐4 water channels on the astrocytic endfeet play a central role in regulating cerebrospinal fluid flow through the brain's interstitial spaces (Jessen et al. 2015; Bohr et al. 2022). Nedergaard's research demonstrated that this clearance process predominantly occurs during sleep because of a significant expansion of brain extracellular space that is sleep‐related (Hablitz et al. 2020). This facilitates the efficient removal of waste products such as amyloid‐beta, which has significant relevance to Alzheimer's disease. While questions remain about this complex process it is relevant to note how clairvoyant the early histologists were about such possibilities.

Held already discussed this issue in his publication from 1905. He stated: “Two questions remain open, which are of great importance for the fluid movement within the brain and its special spaces, the Robin and the His space. The first question is whether these two spaces in deeper layers of the brain, i.e., in the region of fine capillaries, are connected with each other or not after following the blood vessel to this point. … The other question is whether the space between the pial membrane and the *Membrana limitans Gliae* is connected with lymphatic vessels of the pia mater.”

At the end of this chapter, he concluded: “Possibly the pulsation of the vessels triggers a flow within the fluid of the perivascular glial structure and could promote the drainage to the subarachnoid lymphatic spaces. … It will remain for future investigations to determine to what extent glia participate in the substance movement within its protoplasm or its meshwork. It needs to be determined to what extent the longitudinal drainage via the extramarginal spaces, namely the His and Virchow‐Robinson space, serves the efflux of the lymphatic brain fluid to the outside.”

Held also addressed the issue of the possibility of fluid exchange across the glial boundary. “I will address the lymphatic vessels in the interior of the central nervous system. … One cannot consider the clear holes or canals in which the blood vessels of the brain or spinal cord are embedded as natural wide spaces for the circulation of lymphatic fluid. The border membrane of the glia (if preserved) is very closely pressed on the vessel or its adventitious sheath. Due to certain fixations, the entire arrangement of radial fibers, endfeet, and border membrane may be destroyed.”

Thus, his observation did not support such a fluid exchange. Yet he did not make a final conclusion and stated: “I remain cautious on the importance of virtual lymphatic clefts in this context. In any case, from those I distinguish clefts between the vessel adventitia and the *Membrana limitans Gliae*, and I assume that these are fluid pathways which are filled and emptied from the adventitious vessel sheath since the border membrane of the glia prevents diffusion into the tissue of the central substance or at least restricts it.”

To determine the tightness of the glial border membrane, he injected ink: “By injecting a thin China ink solution into the lower surface of the *M. limitans superficialis*, I have tried to determine their permeability and closeness. … It was everywhere trapped below the border membrane and along the glial endfeet of the ascending glial fibers while it nowhere permeated the border membrane.”

While he could not solve the issue with the technologies available to him at the time, he deserves credit for addressing the issue of fluid exchange between the brain and periphery.

## 11 The Filled Net—Reference to Myelinating Cells?

Held described a so‐called filled net, which is associated with glial cells, and he referred to the corpus callosum. It could potentially be the myelin structure. The cells in the pictures he refers to look like oligodendrocytes. This is his description: “As I have shown in figs. 16 and 17 from the white matter of the rabbit cerebellum, the filled net is connected to several glial cells with their protoplasm being slightly stained in a longitudinal section showing the nerve fibers with glial sheath and glial (Ranvier) nodes. … Furthermore, I refer to fig. 14, which illustrates the formation of a part of a glial sheath from a glial cell in the white matter of the human cortex …”

## 12 Do Glial Cells Anastomize?

Held provided a discussion on this controversial issue: “As formation of neuroglia, I consider the general and interdependent relationship of the neuroglial cells among each other. The question arises whether the processes of the neuroglial cells are interconnected among each other or only cross over, be they fiber‐containing, fiber‐poor, or consisting only of nude, partially protoplasmic‐covered glial fibers. …It is remarkable that the views on that question have recently changed to the opposite. Von Kölliker earlier postulated a general net‐type connection of the glial cells and considered it a general reticulum of the central nervous system that penetrates, like a delicate skeleton, the entire white and gray matter. While Fromann and Gierke supported that view, Deiters and Golgi, and later Ranvier and Weigert, objected to an anastomosis of glial cells, resulting in the dominance of the doctrine of a neuroglial felt. I refer in this context to the statement of von Kölliker describing the insertions of the nerve fibers of the white matter into the deceptive meshes of the glial net. Also, Erik Müller reports, in the sense of Deiters and Golgi, that in lower vertebrates, the processes of neuroglial cells only cross each other or only attach to the cell body of other glial cells in ridge‐like elevations or grooves. … With this, I refer to a finding obtained by J. Hardesty, in which he describes glia as a syncytium of ectodermal nature. I would like to add that I independently came to the same opinion based on my observations.”

Today it is well established that the macroglial cells, particularly the astrocytes, form a syncytium and are interconnected with gap junctions forming channels mediated by the protein family of connexins (Kettenmann and Ransom 2013).

## 13 Glial Response to Injury

There is an interesting statement by Held: “It may remain undecided whether the expansion and growth of glia is a secondary event after the nerve cells are damaged, or whether their activity is primary and results in a suffocation of the nervous substance.”

The publications introduced here are less focused on brain diseases and more on features of glial cells in the healthy brain, including during development and vertebrate evolution. It was a large book chapter written by Alzheimer (1910), which exclusively focused on the role of glial cells in different brain diseases. This account was also published in German (Alzheimer 1910).

## 14 Diversity of Glial Cells

Von Kölliker was the first to distinguish two variants of glial cells, which he called short beamer (*Kurzstrahler*) and long beamer (*Langstrahler*), with the former having shorter, strongly branched processes, and the latter very long, sparsely divided processes. The *Kurzstrahler* may represent astrocytes since they are predominantly in the gray matter, and their shape is described as star‐like with processes in all directions. Moreover, they are attached to the blood vessels, a feature of astrocytes. The *Langstrahler* are predominantly found in the white matter and may represent both astrocytes and oligodendrocytes; they are more elongated and often strongly flattened. These types of cells were described in many mammals, including rabbits and humans.

**FIGURE 7:**
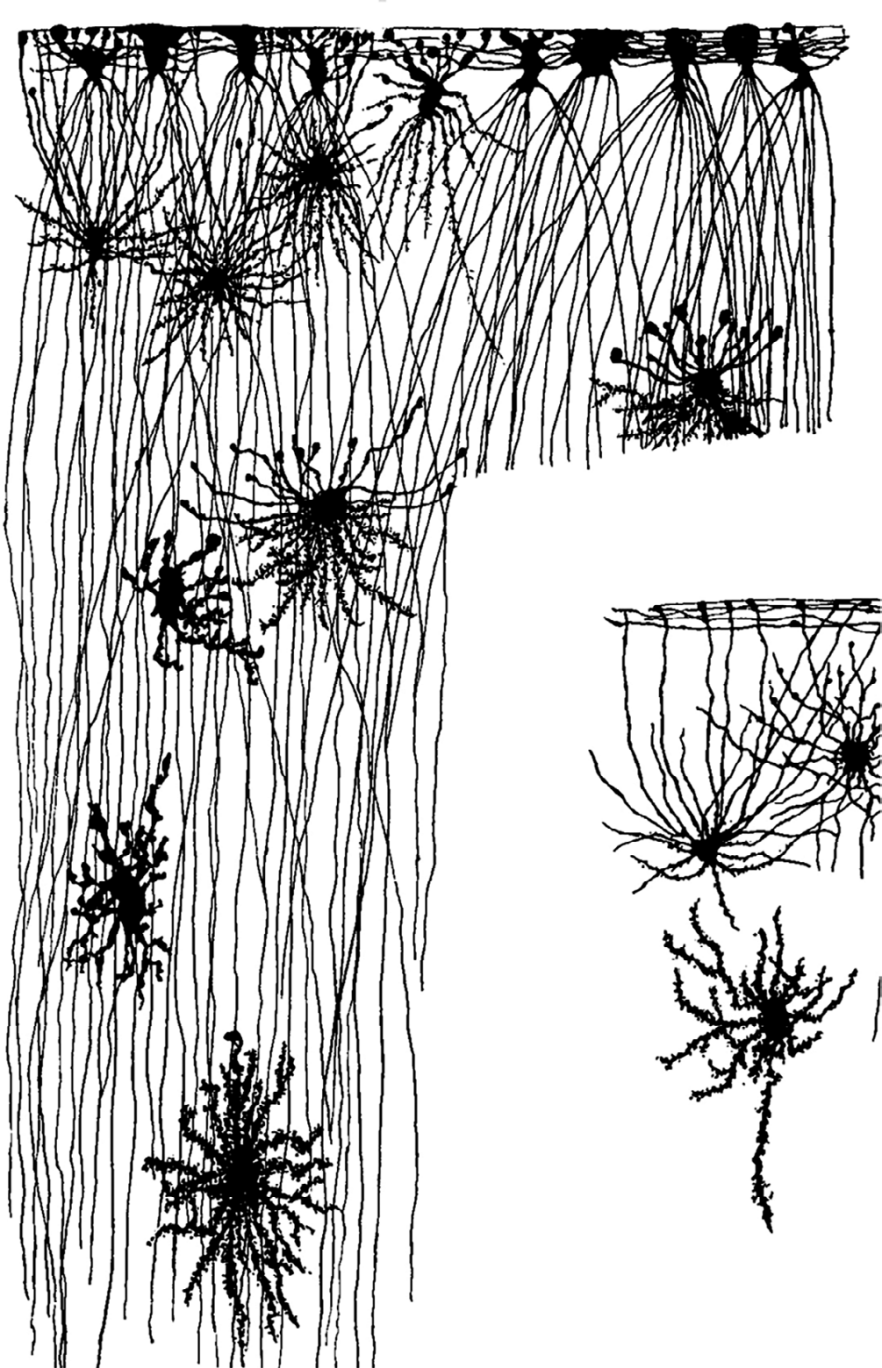
Different types of glial cells. Vertical section of one cortical gyrus of the frontal lobe from a 1‐year‐old child. Schwanzstrahler, Sternstrahler, and Fusssternstrahler. (from Retzius 1894, tab. IV, Figures 1 and 4).

Retzius was trying to extend the terminology describing the different forms of neuroglial cells, which he found in the human cortex. He distinguished *Sternstrahler* (star projector) and *Schwanzstrahler* (tail projector), depending on whether their processes project in all directions or predominantly in one direction. Among the *Sternstrahler* (star projector), he further distinguished *Kurzsternstrahler* (short star projector) and *Langsternstrahler* (long star projector). In addition, he found cells below the surface sending a number of processes to the surface, which terminated there with knots and feet. They also occured in the vicinity of blood vessels to which they attached with conic swollen feet, and he termed them *Fuss‐Sternstrahler* (foot‐star projector). Cells which projected like a sandglass in two directions, he named *Doppelschwanzstrahler* (double tail projector), and finally, cells spreading in a plane he called *Flächenstrahler* (plane projector). He found such cells in different layers of the cortex and also in the spinal cord. This glial diversity is nicely visible in the microscopic reconstructions by Retzius. His drawings also indicate many potential contact sites between extensions of different cells, where gap junctions may be formed and connect the cells into functional networks (Figure 7).

It was also clear at that time that there are special glial cells, such as the Bergmann glia, also termed Golgi epithelial cells, in the cerebellum and the Müller cells in the retina. They were also described by Retzius in humans and mammals.

Today, modern transcriptomics, proteomics technologies and electrophysiology provide unprecedented tools to unravel the diversity of glial cells. Two recent consensus publications have provided an overview of astrocyte (Escartin et al. 2021) and microglial diversity and nomenclature (Paolicelli et al. 2022).

## 15 Phagocytic Neuroglia

Today we know that microglial cells are the professional phagocytes of the brain, yet there are also reports on the capacity of astrocytes to phagocytose. Held referred to work by Nissl, who commented on phagocytosis: “I mention the observation by Nissl that the glial cells, besides their function as providing nutrients for the nervous substance, also have phagocytic properties similar to the body leukocytes, which never enter the cerebral cortex. This latter observation by Nissl seems to me crucial since, first, I have never observed clearly identified leukocytes in the brain and spinal cord within the border membrane. I agree with Nissl that those contrary claims are based on a confusion with glial cells.”

At that time, microglial cells were not yet recognized as a distinct type of glial cell, that would wait till the work of Río‐Hortega. Consequently, their role as the professional phagocytes of the brain would not be established till decades later. It has become evident in the last two decades that microglial cells not only play a role in the pathologic brain, but that synaptic stripping by these cells is essential for brain development and brain plasticity (Sierra, Paolicelli, and Kettenmann 2019). Their role as the “synaptic stripper” has led to a recent explosion of microglial research.

## 16 Summary of the Contents of the Five Books/Chapters

### 16.1 Gustav Retzius “Studien über Ependym and Neuroglia, 1893”

In this chapter, Retzius provided a comparative study of the classes of vertebrates, including jawless, primitive fish like the cyclostomes (hagfish), moving on to bony fish, amphibians, birds, mammals, and finally humans. He demonstrated that the arrangement of what he called “ependymal cells” is a universal phenomenon, present not only in embryos but also in young animals and adult lower animals. These ependymal cells extend from the ventricles, specifically the central canal, to the surface of the brain and spinal cord. That these ependymal cells, which show a fixed habitus and often have lateral branches, form a support system was quite evident to him. Today we call these precursor cells radial glia or radial glia‐like cells.

**FIGURE 8:**
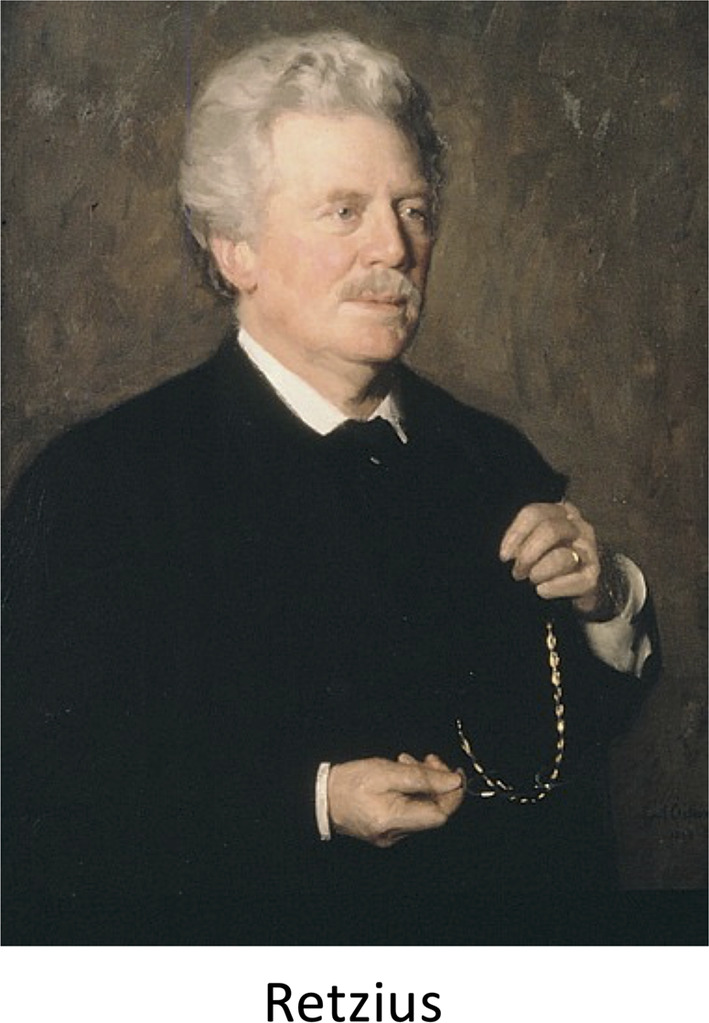
Portrait of Retzius. Gustav (or Magnus Gustaf) Retzius was born in Stockholm in 1842. He received his Medical education first in Uppsala University starting in 1860, and the continued at the Karolinska Institute where he received his Medical degree in 1869. He received his scientific training with Axel Key (1832‐1901) who had previously also worked with Virchow in Berlin. As an assistant to Key their first common publication already focused on connective tissue and the brain (Key and Retzius 1875). He became ordinary professor at the Karolinska in 1889, but resigned a year later due to conflicts with his peers at the institute. Due to his marriage to the woman rights activist Anna Hierta (1841‐1924), the daughter of the well‐established publisher Lars Johan Hierta (1801‐1872), he also got in contact with journalism. After his resignement, he continued his scientific studies as an independent scientist. All his scientific studies were published in his own series of proceedings, the Biologische Untersuchungen (Biological Investigations) in which he served as editor and author at the same time. Also, the two publications on glial cells in 1894 and 1895 which are translated here, were published in this series, His studies on the histology of the nervous system were also focusing on neurons describing for instance the Cajal‐Retzius cells in development. He also analyzed the gross‐anatomy of the brain and his two volume book on 'Das Menschenhirn' (the human brain) (Retzius 1896) was a landmark publication. His last study on glial cells was published in 1921, two years after his death and his wife fostered that last publication (Retzius 1921). *Source*: Wikipedia.

He also demonstrated that these first developing radial support cells play only a transitory role, particularly in higher vertebrates, as they later recede or remain in a rudimentary state. In the meantime, the neuroglial cells develop from the same type of germ cell. Initially, they acquire the peculiar, long radial morphology, but soon they transform into the distinct forms of the neuroglial cells proper Figure 8.

### 16.2 Gustav Retzius “Die Neuroglia des Gehirns beim Menschen und bei Säugetieren, 1894”

In this chapter, Retzius provided a detailed description of different regions of the human brain and compared them to other mammals, predominantly cats, but also dogs and rabbits. He provided excellent images of Golgi‐stained glial cells from these brain regions. These include the cerebral cortex, brain ganglia, medulla oblongata, insula Reili, cerebellum, pituitary, optic nerve, and retina. Based on the diverse morphology, he introduced a nomenclature distinguishing different glial cell types. He found that this diversity of morphological phenotypes is common across the mammalian species he investigated. He also compared different developmental stages in humans, from early and late fetal stages to early postnatal periods, such as a 2.5‐month‐old and a 2‐year‐old child. He compared this to the adult stage, analyzing the brain of a 17‐year‐old male, a 42‐year‐old female, and a 70‐year‐old male. He stated: “Already at the end of the first year and continuing in the following years, the neuroglial cells of the cerebral cortex acquire their final, terminated shape.” He also compared this human material to different developmental stages in cats, dogs, and rabbits. The value of this publication lies particularly in the excellent images of these Golgi‐stained cells.

### 16.3 Michael von Lenhossek “Bau des Nervensystems, 1895”

**FIGURE 9:**
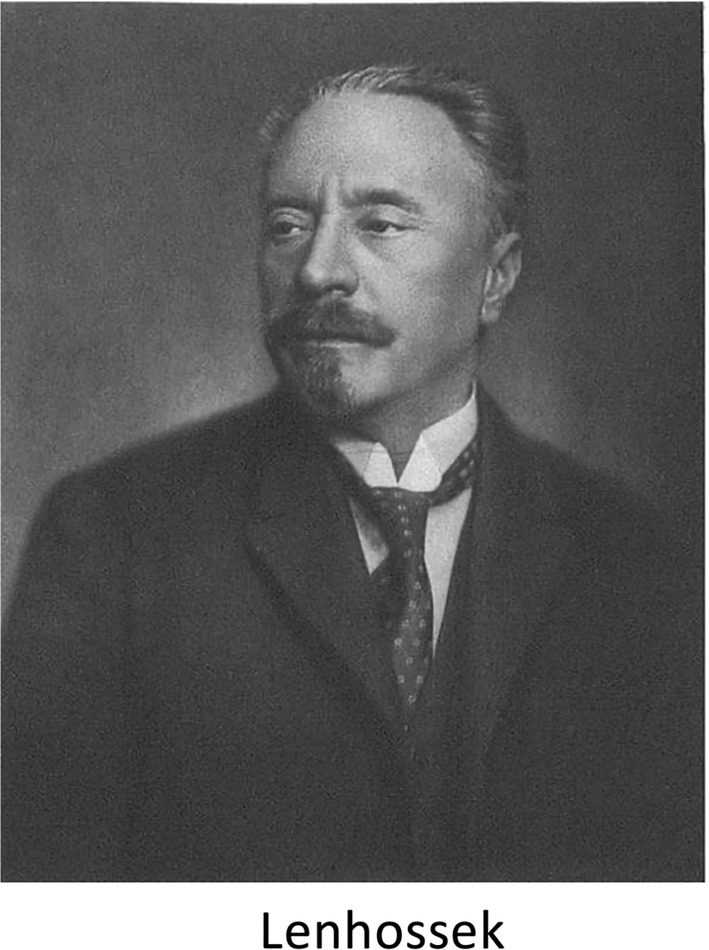
Portrait of Lenhossek. Mihály (von) Lenhossék was born in 1863 in Pest, Hungary as the son of the well‐known anatomist József von Lenhossék (1818‐1888). He studied medicine at the University of Budapest. He was assistant at the Institute of Anatomy headed by his father and became deputy director after his father died in 1888. In 1889, he moved to Basel as prosector of the Anatomical Institute. In 1893 he moved to Würzburg to join Albert von Köllikers (1817‐1905) Department of Anatomy. Von Kölliker at that time was the leading histologist in Germany and even beyond and there Lenhossek studies focused on the histology of the nervous system. His textbook on the histology of the nervous system published in 1893 and as new edtion in 1895 was a landmark publication. He adopted the German version of his first name and published as Michael von Lenhossek. It is quite remarkable that this textbook contained a very large chapter focusing on glial cells which is now translated in this article. Lenhosske had intensly studied glial cells in the human spinal cord. In 1895 he moved to Tübingen and returned to Budapest in 1899 where he served until his retirement in 1934. He died three years later in Budapest. *Source*: Wikipedia.

Lenhossek summarized his studies in a presentation, On the Knowledge of the Neuroglia of the Human Spinal Cord (*Zur Kenntnis der Neuroglia des menschlichen Rückenmarkes*), delivered at the (German) Anatomical Society (*Verhandlung der Anatomischen Gesellschaft auf der fünften Versammlung*) in Munic, held from May 18–20, 1891. Retzius must have attended this lecture, as he frequently cited it. Lenhossek's chapter focused on the spinal cord but also discussed issues beyond humans. He provided an excellent account of the distribution, function, and development of glial cells in the spinal cord. He concluded from his investigations Figure 9:

1. “The support system of the cord consists of cells, including ependymal cells and variably branched support cells (glial cells). These elements are all of ectodermal origin, similar to nerve cells, and are generated within the spinal cord itself.
2. The arrangement of these support cells shows differences across different animals when examining the fully developed cord in higher and lower vertebrates. However, these differences have natural explanations when considering the developmental processes of the cord. A highly interesting fact is that the simpler type of support system in lower vertebrates shows similar conditions to those of higher forms as they pass through transient embryonic stages. Thus, the arrangement of the support cells in the cord convincingly demonstrates a common organization of vertebrates, with phylogenetic developmental stages being repeated in the individual development of higher forms.”

He also coined the term “astrocyte” in this publication. He stated: “I therefore propose to name all supportive cells of the nervous system in general as supportive cells or intermediate cells, *Spongiocytes*, and the common form in higher vertebrates as spider cells or ‘astrocytes’ and use the term neuroglia only *cum grano salis* as long as the concept is not finally approved.”

### 16.4 Carl Weigert “Beiträge zur Kenntnis der normalen menschlichen Neuroglia, 1895”

**FIGURE 10:**
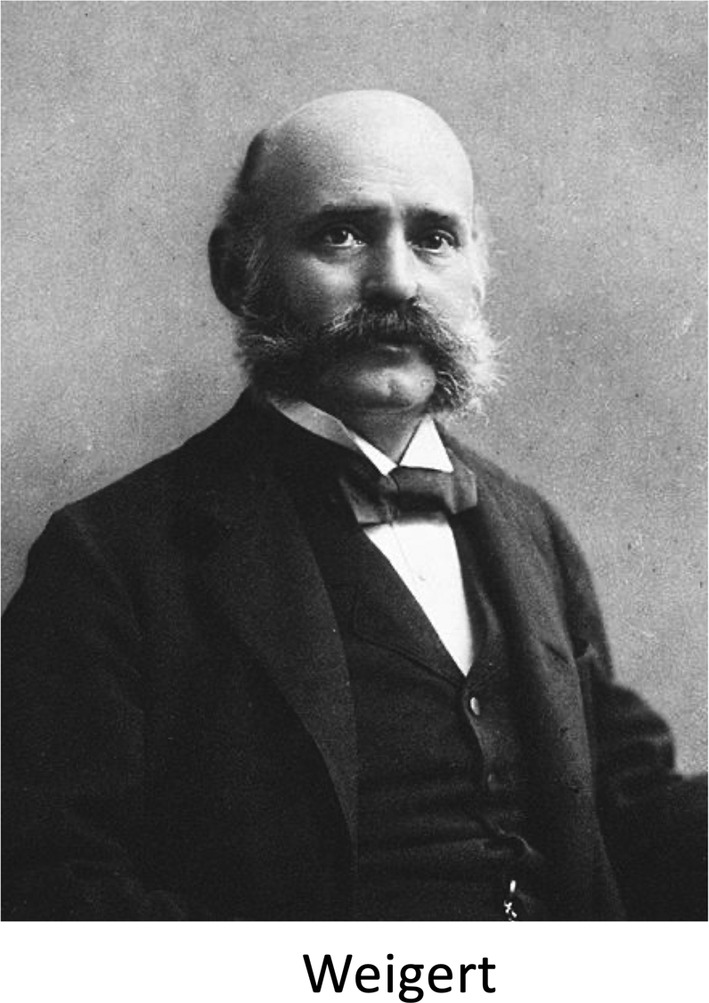
Portrait of Weigert. Carl Weigert was born in Münsterberg in Silesia in 1845. He studied medicine in Breslau, Vienna and Berlin where he received his MD in 1866. He started as an assistant with the weel‐known anatomist Wilhelm Waldeyer (1836‐1921) in Breslau, later with Julius Cohnheim (1839‐1884) and followed him to Leipzig in 1878 at the Pathological Institute. After Cohnheims death, he was not appointed as his successor due to his jewish origin. He therefore moved to the Senckenberg Foundation in Frankfurt and became the director of the Pathological Institute and stayed there until his death in 1904. He had a passion for staining techniques and developed the first stain for bacteria using anilin dyes in 1875 (Weigert 1875). This led him to studies on infections focusing on tuberculosis and pocks. A milestone was the development of the myelin stain with iron hemetoxilin in 1891 (Weigert 1891) and Weigert became a pioneer of nervous system staining technologies besides Camillo Golgi (1843‐1926) and Franz Nissl (1860‐1919). He also developed a staining method which labeled glial fibers in the nervous system and he summarized his findings on the human nervous system in his 1895 publication which is translated in this article (Weigert 1895). *Source*: Wikipedia.

Weigert summarized his studies on the human brain. An important aspect of his publication is the development of his specific staining method. According to him, he optimized it over approximately 6 years to provide a labeling method that visualized what he considered the neuroglial structures. His technique did not label any neuronal structures but rather fibers, which he considered reflective of the neuroglial structure of the brain. Additionally, the nuclei of glial cells were stained Figure 10. He stated: “Among the nuclei are those which, according to the current view, can only be regarded as nuclei of glial cells because they are located in regions where, as far as we know, ganglion cells do not occur, for example, in the white matter of the spinal cord. There are two main types of these nuclei: larger vesicular nuclei with granular‐looking chromatin and smaller ones in which the chromatin is a homogeneous dark mass.”

He argued that his labeling method was specific since he assumed that these fibers possess a distinct chemical nature. The fibers had the following features:

1. The fibers are more or less straight, or they run in rigidly curved bends. They are never tightly curled.
2. The fibers appear quite solid; they are not hollow.
3. The fibers are quite smooth; they do not have a “granular texture” or circumscribed buds or thickenings.
4. Just like varicosities, neuroglial fibers do not acquire moss‐like or other attachments.
5. Finally, these fibers never exhibit any of the conical or flask‐like extensions so often described with the Golgi method.

His staining method was specific to human material. He described the distribution of these fibers in separate chapters entitled: spinal cord, medulla oblongata, pons, pedunculus cerebri, quadruplet bodies, pineal gland, cerebellum, cerebrum, gyrus hippocampi, corpus callosum and fornix, optic nerve and chiasm, corpora mammillaria, thalamus, corpus striatum and capsula.

He also added a chapter on the physiological functions of neuroglia. He stated: “It is safe to say that neuroglia serves a space‐filling function … A correspondingly smaller or larger number of neuroglial fibers are always observed to fill the vacant space.” His conclusion that “neuroglia serves a space‐filling function” was frequently repeated in textbooks and elsewhere. This may have inadvertently tempered enthusiasm for neuroglial research if that modest function was the most that could be said about these cells.

Then he claimed that “Golgi's notion that dendrites have protoplasmic nutritional processes precisely because they are associated with “neuroglial cells” cannot be correct. Golgi's argument is only valid if the “extensions” of Deiters' cells are considered to be true protoplasmic cell processes. But we can now confidently confirm that Deiters' cell “extensions” are not protoplasmic extensions and indeed that they are not cell extensions at all. Consequently, the concept that these fibers might be important for neuroglial cell metabolism and, moreover, that they may be indirectly involved in ganglion cell metabolism must definitely be dismissed.”

### 16.5 Hans Held “Bau der Neuroglia, 1905”

Held is known to the neuroscience community as he was the first to describe the largest synapse in the vertebrate nervous system, the Calyx of Held (Held 1893) Figure 11.

**FIGURE 11:**
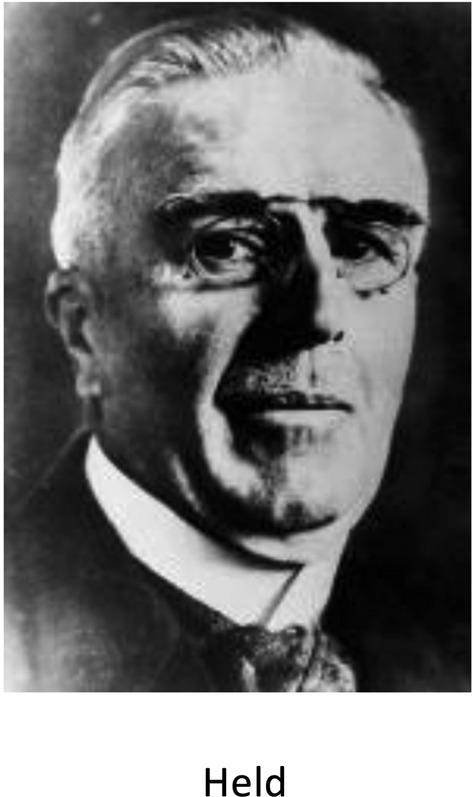
Portrait of Held. Hans Held, born 1866 in Neukloster, in the northern east of Germany, studied Medicine in Rostock and Leipzig finishing with his doctoral degree in 1891. In 1899 he was extraordinary professor, in 1917 full professor at the Institute of Anatomy and Histology at the University of Leipzig. Held was a very active teacher offering several courses every year mainly on the histology of the nervous system, but also on general histology and anatomy between 1892 to 1914. He served as the Dean of Medicine in 1920/21 and as President of the university in 1922/23. He is well‐known for his studies on the cellular mechanism of auditory processing and he identified the giant synapse in the central auditory pathway, termed after him as the Calyx of Held (Held 1893). His studies on the structure of the grey and white matter triggered his interest in glial cells (Held 1902). He summarized his studies on glial cells in the publication which is translated in this article. He retired in 1934 and died in Leipzig in 1942. *Source*: https://archiv.saw‐leipzig.de/saw‐archive/personen/hans‐held.

At the beginning of his chapter, he provided a definition of neuroglia: “They are distinct by the fact that all of their processes are homogeneous and stable and never transform into nerve fibers. In contrast, they partially form an ensheathment around the nerve fibers and the nerve cells. Second, they neither produce nor accumulate Nissl bodies in their protoplasm. Third, they largely produce a felt of characteristic neuroglia fibers, which serves to stabilize the nervous elements of the central nervous system. Fourth, they enable the formation of border membranes which are responsible for the complete enclosure of the central nervous system in the true sense of the word.”

He clearly supported the concept by Retzius and Lenhossek and dismissed the astrocyte concept proposed by Weigert: “My data, described above, demonstrate that astrocytes are radial‐fibered glial cells and their own glial fibers are embedded in the protoplasm or enwrapped by a cell membrane. Not only is the cell body enwrapped, but so is the onset of the process. Weigert, with respect to the astrocyte definition, claims that their images are mirage, showing no distinction between glial fibers and glial cell protoplasm, and the former being simply its processes. In my opinion, this reproach should be amended to the effect that the glial fibers are intracellular within the protoplasm of the cell body and its branching processes. I must thus accuse Weigert of having overstepped the limits of his method.”

His studies were focused on the central nervous system of humans, which he had studied in newborns or children only a few months old and in adults in their early to mid‐twenties. Moreover, he compared the human findings to rabbits and dogs and partially to the spinal cords of calves and cattle.

## Author Contributions

**Helmut Kettenmann:** provided the concept, translated the German texts and wrote the first draft of the manuscript. **Bilge Ugursu, Bruce R. Ransom and Christian Steinhäuser:** proofreading.

## Conflicts of Interest

The authors declare no conflicts of interest.

## Supporting information


**Data S1.** Scanned original publication by Retzius, Volume V.

**Data S2.** Translated text by Rezius, Volume V with single figures inserted into the text.

**Data S3.** Scanned original publication by Retzius, Volume VI.

**Data S4.** Translated text by Rezius, Volume VI with single figures inserted into the text.

**Data S5.** Scanned original publication by Lenhossek.

**Data S6.** Translated text by Lenhossek.

**Data S7.** Scanned original publication by Weigert.

**Data S8.** Translated text by Weigert with single figures inserted into the text.

**Data S9.** Scanned original publication by Held.

**Data S10.** Translated text by Held with single figures inserted into the text.

**Data S11.** Reference list of citations from the articles by Retzius, Lenhossek, Weigert and Held and combined into one list either sorted alphabetically or chronologically.

## Data Availability

The data that support the findings of this study are available from the corresponding author upon reasonable request.
