# Supplementary material for: The Concept of Neuroglia ‐ the State of the Art Circa 1900
Source: Glia. 2025 Feb 4;73(5):890–904. doi: 10.1002/glia.24678 (PMC11920685; doi:10.1002/glia.24678)
Supplement: Supplementary file 1 — Data S1. Scanned original publication by Retzius, Volume V. [file GLIA-73-890-s002.pdf]

2.

STUDIEN ÜBER
EPENDYM UND NEUROGLIA.

Taf. V—XIII.

Als ich vor zwei Jahren eine vorläufige Mittheilung über die Ependymzellen der Centralorgane[1] veröffentlichte, war es meine Absicht, dasselbe Thema bald nachher in eingehenderer Weise zu behandeln. Auf dem im Mai desselben Jahres abgehaltenen Anatomencongresse in München legte indessen VON LENHOSSÉK[2] seine Untersuchungen »Zur Kenntnis der Neuroglia des menschlichen Rückenmarkes» vor, durch welche diese Frage in so vortrefflicher Weise dargestellt wurde, dass in prinzipieller Hinsicht kaum etwas Wesentliches hinzuzufügen war. Ich legte deshalb zu jener Zeit die betreffende Arbeit zur Seite.

Oft sind mir indessen bei meinen Studien über den Bau der Centralorgane Bilder vorgekommen, die über das Ependym und die Neuroglia erläuternd waren. Da dieselben auch Thiere, wie z. B. die Knochenfische, betreffen, welche in dieser Beziehung bisher wenig oder fast nicht behandelt worden sind, so habe ich mich entschlossen, von meinen bei verschiedenen Gelegenheiten gemachten Abbildungen eine Reihe zu veröffentlichen. Dieselben können vielleicht dazu beitragen, die noch nicht allgemein angenommenen neuen Lehren zu stützen und einige Lücken auszufüllen. Ich beabsichtige deshalb keineswegs, eine zusammenfassende Darstellung, sondern nur eine Reihe erläuternder Beiträge mitzutheilen. Unter diesen Umständen ist hier für eine ausführliche geschichtliche Darstellung nicht der Platz; eine solche ist ja schon mehrmals von anderer Seite geliefert worden. Ich werde deshalb die »vorgolgische» Zeit nicht berühren, sondern nur einige wichtigere Data der späteren Geschichte der Frage hervorheben. Was die vorgolgische Zeit betrifft, so findet man z. B. in der gründlichen Arbeit von GIERKE[3] die hierauf bezüglichen Angaben und Ansichten früherer Forscher, wie DEITERS, BOLL, JASTROWITZ RANVIER u. A., berücksichtigt.

Mit GOLGI's epochemachenden Arbeiten begann aber eine neue Aera in unseren Kenntnissen in der Nervenhistologie. Auch in Betreff der Lehre von dem Ependym und der Neuroglia ging er voran, und durch seine und WEIGERT's neuen Methoden erhielten wir Mittel, um auf diesem Gebiete siegreich vorwärts schreiten zu können. In Betreff des Ependyms wusste man zwar (STIEDA, REISSNER u. A.), dass wenigstens bei gewissen Wirbelthieren im Rückenmark die Ependymzellen fadenähnliche Fortsätze radiirend nach der Oberfläche, v. A. aber nach der vorderen und hinteren Furche, hin senden. Aber erst durch GOLGI erhielt diese Thatsache ihre volle Bestätigung und Erweiterung. In seinem grossen Werke über die feinere Anatomie der nervösen Centralorgane[4] giebt er zwar nur eine vorläufige, aber doch offenbar auf eingehende Untersuchungen gestützte Darstellung, die später von verschie-

[1] GUSTAF RETZIUS, Zur Kenntniss der Ependymzellen der Centralorgane. Verhandl. d. Biolog. Vereins in Stockholm. Bd 3, 1890—91 (15 März 1891).

[2] MICH. VON LENHOSSÉK, Zur Kenntnis der Neuroglia des menschlichen Rückenmarks. Verhandl. d. Anatomischen Gesellschaft auf d. fünften Versamml. in München, 18—20 Mai 1891.

[3] H. GIERKE, Die Stützsubstanz des Centralnervensystems. Archiv f. mikrosk. Anatomie. Bd 25, 1885.

[4] CAMILLO GOLGI, Sulla fina anatomia degli organi centrali del sistema nervoso, Reggio-Emilia, 1885. Ich habe oben die vom Verf. besorgte deutsche Uebersetzung benutzt, die im Anatom. Anzeiger, Jahrg. V, N:o 13 & 14, Juli 1890 veröffentlicht worden ist.

denen Seiten bestätigt wurde. Bei Hühnerembryonen in verschiedenen Stadien (4. bis 12. Bebrütungstage) sah er das Epithel des Centralkanals einen unmittelbaren und beträchtlichen Antheil an der Bildung der interstitiellen Substanz in jedem seiner Theile nehmen, und zwar nicht nur der grauen Substanz in ihrer ganzen Ausdehnung, sondern auch der weissen Substanz der Vorder- und Seitenstränge sowohl wie der Hinterstränge, d. h. von dem Centralkanal an bis an die äusserste Begrenzung des Rückenmarks unmittelbar unter der Pia mater. Die einzelnen Cylinderepithelzellen des Centralkanales erstrecken sich nämlich radiär durch die ganze Schnittfläche des Rückenmarks und erreichen die äusserste Peripherie dieses Organs unter der Pia. Hier enden die fadenförmigen Ausläufer der einzelnen Cylinderzellen, indem sie manchmal eine konische Anschwellung, manchmal eine geringe Verbreiterung bilden, mit der die genannten Fäden, sei es an der Pia mater, sei es an den dort vorhandenen Gefässwänden, sich ansetzen. Auf diesem langen Verlaufe zeigen die mehr oder weniger starken Fäden, welche die periphere Fortsetzung des Körpers der einzelnen Epithelzellen darstellen, gewöhnlich Verzweigungen. Dieselben sind manchmal spärlich, häufiger aber sind sie zahlreich und kompliciert. Die sekundären Verzweigungen gelangen nun ihrerseits zum Theil an die Peripherie des Marks, wo sie in der angegebenen Weise enden, zum Theil verlieren sie sich während ihres Verlaufes, zum Theil setzen sie sich an die Gefässwände an. Es entsteht durch diese zierliche und dichte Ausstrahlung von Fasern in gewisser Weise ein zusammenhängendes Gewebe zwischen dem Centrum und der Peripherie des Markes. GOLGI äussert ferner, dass er sich durch die gewonnenen Ergebnisse für berechtigt halte »ohne weiteres anzunehmen, dass das interstitielle Stroma der Nervencentren zu den Geweben gehört, welche ihren Ursprung von dem äusseren Keimblatt oder Hornblatt nehmen», und er fügt am Ende des betreffenden Capitels Folgendes hinzu: »Aus dieser Beschreibung geht klar hervor, dass der epitheliale Theil des Rückenmarks, welcher zweifellos von dem äusseren Keimblatte abstammt, einen direkten Antheil an der Bildung des zwischen den nervösen Elementen liegenden Gewebes (der Neuroglia) nimmt. Durch welche Reihe embryologischer, chemischer und histologischer Thatsachen», sagt er, »ich mich für berechtigt halte, auch die Zellen der Neuroglia im eigentlichen Sinne dem Epithel gleichzustellen, das werde ich, wie ich bereits gesagt habe, in einer anderen Arbeit darthun.»

Meines Wissens ist diese vom Verfasser versprochene Arbeit bisher nicht erschienen. Die angeführte vorläufige Mittheilung legt aber seine wichtigen Angaben und Ansichten deutlich und klar dar.

Der Forscher, der das in Rede stehende Thema zunächst behandelte, war FRIDTJOF NANSEN[1], und zwar that er dies bei *Amphioxus* und *Myxine*. Beim ersteren Thiere sah er die epithelialen Cylinderzellen, die den Centralkanal umgeben, mit ihren äusseren, theilweise in Bündeln verlaufenden Fortsätzen die »weisse» Substanz durchsetzen und sich mittelst ihrer Enden mit der Rückenmarkshülle verbinden. Keine Neurogliazellen von der Art wie bei Myxine sind hier gefunden worden. »Deshalb dürfen wir annehmen», sagt NANSEN, »dass diese Epithelialzellen die wirklichen Neurogliazellen des Amphioxus darstellen und dass diese Neuroglia als das erste Stadium der fraglichen Elemente bei den Wirbelthieren aufzufassen ist. Bei *Myxine* sind dagegen zwei Arten von derartigen Elementen vorhanden, nämlich erstens *Epithelzellen,* die den Centralkanal umgeben und an der dorsalen und ventralen Seite des Markes bis zur Oberfläche empordringen sowie auch in den lateralen Partien vorkommen, obschon NANSEN hier ihre äusseren Enden nicht nachweisen konnte, und zweitens die *Neurogliazellen,* die in der grauen Substanz in grosser Anzahl auftreten und einen kleinen Zellkörper haben, der oft mit zahlreichen, wenig verzweigten Fortsätzen versehen ist, die bis an die dorsale oder die ventrale, zuweilen bis an beide Hüllen zugleich reichen und dort endigen. Mittelst der Golgi'schen Methode lassen sie sich schön darstellen. Die Fortsätze dieser Zellen anastomosiren mit einander nicht. An den Seiten des Kanales giebt es Uebergangsformen zwischen den beiden Zellenarten. »Ich glaube», sagt NANSEN, »dass wir wohl berechtigt sind, zu sagen, dass die Neurogliazellen einen ektodermalen Ursprung haben und aus den Epithelzellen hervorgehen, die den Centralkanal umgeben».

Nachdem die vorliegende Frage danach von den italienischen Forschern FALZACAPPA und MAGINI behandelt worden war, wurde sie, ebenfalls mit der Golgi'schen Methode von RAMÓN Y CAJAL und VON KÖLLIKER angegriffen und besprochen. CAJAL[2] bestätigte bei Hühnerembryonen die Angaben von GOLGI; er beschrieb zuerst die verschieden-

[1] FRIDTJOF NANSEN, The Structure and Combination of the Histological Elements of the Central Nervous System. Bergens Museums Aarsberetning for 1886. Bergen 1887.

[2] S. RAMÓN Y CAJAL, Sur l'origine et les ramifications des fibres nerveuses de la moelle embryonaire. Anatom. Anzeiger, V. Jahrg. N:o 4. Febr. 1890.

artige Disposition der Ependymzellen in der vorderen und hinteren Commissur sowie in den Seitenpartien des Rückenmarkes genauer und lieferte eine gute Abbildung des Rückenmark-Querschnittes des Hühnerembryos v. 9. Bebrütungstage; er erwähnt an den Rändern der einzelnen Ependymfäden kleine stachelige Aeste und eine zuweilen vorkommende Verzweigung des peripherischen Endes derselben, sowie konische Verdickungen dieser Enden unter der Pia mater, und er hatte sogar gesehen, dass das innere Ende sich zuweilen in ein in den Centralkanal einschiessendes, feines und langes Haar verlängert. Vom 8. Bebrütungstage an, sagt Cajal, bemerkt man zwischen den Ependymelementen stets gewisse Gebilde, die kürzer sind und den Centralkanal nicht erreichen; sie rühren von einer Dislocation und vielleicht von einer Proliferation der anderen Ependymelemente her und stellen die Vorstufen der Spinnenzellen dar. Je älter der Embryo ist, um so kürzer und verzweigter erscheinen die die Seitenpartien des Rückenmarks radiär durchziehenden Ependymfäden. Die Neuroglia- oder Spinnenzellen sind im Rückenmarke der Hühnerembryonen vom 9. oder 10. Tage an erkennbar; sie treten zuerst im Vorderhorn auf, bald danach findet man sie in der weissen Substanz der Stränge und zuletzt in den Hinterhörnern. Die Spinnenzellen sind nichts Anderes als dislocirte und sehr umgewandelte Ependymelemente; vom 7. bis 14. Tage trifft man unter ihnen alle Uebergänge sowohl in Betreff der Form wie der Lage. Man bemerkt sogar im Marke erwachsener Säugethiere Neurogliazellen, an denen man einen centralen Faden (Ependymfortsatz) weit durch die graue Substanz hindurch verfolgen kann und die noch einen oder mehrere peripherisch radiirende Fortsätze besitzen. Im Grosshirn und Kleinhirn sind orientirte Neurogliazellen nicht selten: die Radialzellen der Molekularschicht des letzteren stellen ein gutes Beispiel davon dar. Die leucocytische Herkunft der Neurogliazellen ist nicht annehmbar. Diese Zellen anastomosiren übrigens nicht mit einander, sondern sie sind vollständig selbstständige Elemente. — In einigen seiner folgenden Arbeiten bespricht Cajal ausserdem gelegentlich die Ependym- und Neurogliazellen anderer Partien der Centralorgane, z. B. die der Kleinhirn- und Grosshirnrinde.

In seiner Arbeit über das Rückenmark bespricht auch von Kölliker[1] die Neurogliafrage. Das Mark junger Embryonen besitzt, sagt er, anfänglich nur einerlei Gliazellen, die nichts Anderes sind, als die sogenannten Epithelzellen des Centralkanals, die mit ihren Ausläufern radienartig das ganze Mark durchziehen und an der Oberfläche desselben dicht an der Pia mit grösseren oder kleineren Verbreiterungen enden. Hierbei zeigen die längeren dieser Elemente, die alle nur *einen* Kern dicht am Centralkanale besitzen, in ihren äusseren Theilen spitzwinklige Verästelungen und viele Seitenästchen, so dass der Anschein eines Netzes erzeugt wird, ohne dass ein solches wirklich vorhanden wäre. Das erste Auftreten dieser Gliazellen, das besonders His und auch Vignal verfolgt haben, ist bei jungen Embryonen leicht zu sehen und führt auf einen Theil der Zellen der Medullarplatte zurück, die zu Faserzellen auswachsen und zugleich Seitenausläufer treiben. Das ursprüngliche Verhalten der Gliazellen erhält sich längere Zeit, und v. Kölliker fand es noch bei einem Schafembryo von 9 Cm. Länge und einem Schweineembryo von 10 Cm. Länge. Bei älteren Embryonen und nach der Geburt erhalten sich diejenigen dieser Elemente vollständig, die nach dem Grunde der ventralen Spalte des Markes und nach der dorsalen Mittellinie gehen; dagegen werden die anderen, bis auf die um den Centralkanal gelegenen Theile, nach und nach undeutlich, und es treten in allen Theilen des Markes eine Menge neuer Gliazellen, die bekannten sternförmigen Elemente auf, die sich unzweifelhaft allmählich aus den indifferenten Zellen der Markanlage entwickeln, welche, so lange dieses Organ nicht ausgebildet ist, in Form rundlicher Zellen in grosser Menge in der weissen und grauen Substanz zwischen den nervösen Elementen vorhanden sind und später sich nicht mehr nachweisen lassen.

M. von Lenhossék[2] stellte etwa gleichzeitig mit Cajal Untersuchungen über die Entwicklung des Rückenmarks des Hühnerembryos an. Er beschrieb die Entwicklung der Ependymzellen oder »Radiärfasern», die sich schon am dritten Tage imprägniren lassen; dieselben sind von Anfang an mit zahlreichen, ganz minimalen, unter rechtem Winkel abgehenden Fädchen und Unregelmässigkeiten besetzt, die in einer späteren Phase grössere Entfaltung gewinnen, indess nur an deren innerem, der grauen Substanz angehörendem Abschnitt vorhanden sind. Theilungen treten früh auf, und zwar erfolgen sie aussen, im Bereich der weissen Belegschicht, oder nahe derselben, und erscheinen zuerst in Form einfacher dichotomischer Spaltungen, um sich allmählig complicirter zu gestalten. Die

[1] A. von Kölliker, Zur feineren Anatomie des centralen Nervensystems. Zweiter Beitrag: Das Rückenmark. Zeitschr. f. wissensch. Zoologie. Bd 51, 1890.

[2] Mich. von Lenhossék, Zur ersten Entstehung der Nervenzellen und Nervenfasern bei dem Vogelembryo. Mittheil. aus dem anatom. Institut im Vesalianum zu Basel, 1890.

centralen Fortsätze sind stets ungetheilt. Die Zellen der Bodenplatte sind anfangs ansehnlich verbreitert, schrumpfen aber allmählig zusammen, um sich vom 8. Tage an dem Typus der übrigen Radiärfasern anzuschliessen. Eine Complication dieses einfachen Stützsystems leitet sich am 6. Tage durch das Auftreten der Deiters'schen Zellen ein, die zunächst in der Umgebung des Centralkanales auftauchen und nichts Anderes als herausgerückte, ihres centralen Ausläufers verlustig gewordene, mit ihren peripheren Theilen hingegen bis zur Oberfläche des Markes vordringende Radiärzellen darstellen. Am 12. Tage findet man bereits zahlreiche freie Neurogliazellen, doch erscheinen sie nun schon zum grossen Theile von etwas abweichender, charakteristischer Form und erreichen mit ihrem äusseren Ausläufer bisweilen nicht mehr die Peripherie. »Diese später entstandene Sorte von Neurogliazellen geht wohl», sagt von Lenhossék, »nicht mehr aus einer Umwandlung und Herausrückung der primitiven Radiärzellen, sondern direct aus den Mitosen der Keimschichte hervor.»

Oyarzun[1] beschrieb im Vorderhirn der Amphibien (Frosch, Triton, Salamander) nach Behandlung desselben mittelst der Golgi'schen Methode reichlich verzweigte Ependymzellen, und *ich*[2] lieferte eine kurze Darstellung von der Gestalt und Anordnung dieser Zellen im Gehirn und Rückenmark bei verschiedenen Wirbelthieren (Hecht, Frosch, Kaninchen, Katze). In einer zu derselben Zeit veröffentlichten Abhandlung[3] besprach ich auch die Neurogliazellen der äusseren Gross- und Kleinhirnrindenschicht der Säugethiere, welche letztgenannten Zellen kurz vorher auch von Martinotti[4] behandelt wurden. Ich beschrieb u. A. im Rückenmarke von Katzenembryonen das nähere Verhalten der Ependymzellen vorn und hinten (im vorderen und hinteren »Keilstück» oder Ependymkeil), sowie in den Seitenpartien. Aus meiner in Bruchstücken gegebenen Darstellung zog ich den Schluss, »dass die neulich von mehreren Forschern im Rückenmark und Gehirn beschriebene Anordnung der Ependymzellen eine allgemeine Erscheinung zu sein scheint. Nicht nur bei Embryonen, sondern auch bei jungen Thieren und bei niederen Thieren im erwachsenen Zustande lassen sich in grosser Ausdehnung solche Zellen nachweisen, welche von den Ventrikelflächen, resp. vom Centralkanal, bis zur Oberfläche des Gehirns und Rückenmarks ziehen, obwohl hier und da während der Entwicklung die äusseren Enden derselben verkümmern können und dann nicht mehr darzulegen sind. Dass diese Ependymzellen, welche im Ganzen einen starren Habitus zeigen und oft Seitenäste abgeben, eine Art Stützgewebe des centralen Nervengewebes darstellen, liegt auf der Hand». In Betreff des Verhaltens dieser Ependymzellen zu den echten Neurogliazellen schloss ich mich der von Kölliker ausgedrückten Ansicht an. »Die beiden Zellenarten zeigen nämlich einen anffallend differenten Typus. Die Herkunft und Entwicklung der wirklichen Neurogliazellen ist aber bisjetzt nicht hinreichend bekannt.»

In seiner Arbeit über das Rückenmark und das Kleinhirn besprach Van Gehuchten[5] gelegentlich auch die Ependym- und Neurogliazellen. In Betreff der ersteren bestätigte er die Angaben Golgi's und lieferte aus der Medulla oblongata eines fast ausgetragenen Kaninchenjungen ein Querschnittsbild mit reichlichem Ependym. Die Neurogliazellen der weissen und grauen Substanz haben zahlreiche Fortsätze, zeigen aber nicht immer dieselben Charaktere. Sehr oft erstreckt sich einer der Fortsätze, welcher dicker ist, vom Zellenkörper weit hinaus; dies ist gewöhnlich am hinteren Theil des Centralkanales der Fall, wo sich die Neurogliazellen im dorsalen Medianseptum bis zur Oberfläche des Rückenmarkes erstrecken; mehrere in der Nähe dieses Septum belegene Zellen schlagen sogar eine gegen die vorigen senkrechte Richtung ein und senden einen ihrer Fortsätze bis in die Substantia Rolandi hinein. Diese Zellen sind unzweifelhaft Ependymzellen, die in irgend einer Weise ihre Verbindung mit dem Centralkanal verloren haben. Van Gehuchten beschrieb auch die Bergmann'schen Faserzellen der Kleinhirnrinde und lieferte Abbildungen von ihnen.

In demselben Jahre erschien eine Abhandlung von Lachi[6] über die Entwicklung der Neuroglia. Er unterschied betreffs derselben zwei Perioden. In der ersten, die bis zum 8. oder 9. Tage der Bebrütung dauert, ist

[1] A. Oyarzun, Ueber den feineren Bau des Vorderhirns der Amphibien. Arch. f. mikrosk. Anatomie. Bd 35, 1890.

[2] Gustaf Retzius, Zur Kenntniss der Ependymzellen der Centralorgane. Verhandl. d. Biolog. Vereins in Stockholm. Bd 3, (1890—91); 15 März 1891.

[3] Gustaf Retzius, Ueber den Bau der Oberflächenschicht der Grosshirnrinde beim Menschen und bei den Säugethieren, ebenda.

[4] Carlo Martinotti, Beitrag zum Studium der Hirnrinde und dem Centralursprung der Nerven. Internat. Monatschrift f. Anat. und Phys. Bd 7, 1890.

[5] A. Van Gehuchten. La structure des centres nerveux. La Moelle épinière et le cervelet. La Cellule, t. 7; dép. l. 20 avril 1891.

[6] Pilade Lachi, Contributo alla istogenesi della Nevroglia nel midollo spinale del pollo. Atti della Società toscana di scienze naturali, resid. in Pisa. Memorie. Vol. 11, 1891.

die Neuroglia ausschliesslich durch Spongioblasten ektodermalen Ursprungs repräsentirt. In der zweiten, vom 8. oder 9. Tage an bis in die ersten Tage »nach der Geburt», treten Elemente mesenchymalen Ursprungs, zuerst in der weissen und dann auch in der grauen Substanz, hinzu; die neuen mesenchymalen Elemente vermehren sich vielfach durch indirecte Theilung und senden gegen das Ende der Bebrütung Fortsätze aus, die für die Neurogliazellen charakteristisch sind. Vom 21. Tage an zeigen sich noch andere Elemente vasculären Ursprungs, sei es Endothelzellen oder Leucocyten.

Indessen hatte noch im Frühjahr desselben Jahres, auf dem Anatomencongresse in München, von Lenhossék[1] seinen schon oben angeführten Vortrag über die Neuroglia des *menschlichen* Rückenmarkes gehalten, welcher Vortrag bald nachher in den Verhandlungen der Anatom. Gesellschaft erschien. Es ist leider nicht möglich, hier ein vollständiges Referat dieser inhaltreichen und genauen Darstellung zu liefern; ich muss mich deshalb auf die Andeutung der wichtigeren Angaben derselben beschränken. Von Lenhossék studirte die Verhältnisse bei menschlichen Embryonen; am vollkommensten gelang ihm die Imprägnation eines 14 Cm. langen Embryos, weshalb seine Schilderung hauptsächlich auf dem Studium dieses Objectes beruht. Das Stützgerüst des Markes besitzt einen exquisit faserigen Bau: das reiche System langer, dünner, von den inneren Theilen des Markes nach der Peripherie ausstrahlender Fasern. Befestigt wird das Gefüge dieser Fasern durch eine grosse Anzahl minimaler seitlicher Aestchen und Anhängsel, durch welche sie sich wohl zu einem geschlossenen Gerüst verhäkeln, ohne aber dabei mehr als einen einfachen Contakt aufzuweisen. Ihren Ausgangspunkt haben diese Fasern in besonderen Zellen, die theils als Ependymzellen am Centralkanale stehen, theils als Deiters'sche Zellen oder Neurogliazellen in einer bestimmten Anordnung über die graue und, in etwas geringerer Zahl, über die weisse Substanz vertheilt sind. Die Vertheilung der auf diesem Entwicklungsstadium mit länglich-spindelförmig oder elliptisch gestalteten und mit zahlreichen feinen, starren, kurzen Fäserchen besetzten Zellenkörpern versehenen Elemente ist in den verschiedenen Regionen verschieden. Die Vorderhörner scheinen an Gliazellen am ärmsten zu sein; gegen die Hinterhörner hin nimmt ihre Menge allmählig zu; innerhalb der weissen Substanz ist ihre Anzahl eine viel geringere als innerhalb der grauen. Alle diese Zellen sind nun ohne Frage gleichen Ursprungs; sie entstammen dem Ektoderm durch Vermittelung der in der innersten Schicht des Medullarrohres befindlichen Mitosen und gelangen durch successives Herauswandern an ihren definitiven Platz. »Ich vermisse», sagt v. Lenhossék, »vollständig bei dem menschlichen Foetus, selbst in viel vorgerückteren Stadien, anderweitige Neurogliaelemente als die eben geschilderten, sei es solche, die zu den Blutgefässen Beziehungen erkennen lassen, sei es die, die Charaktere von Bindegewebszellen zur Schau tragen würden, und so erscheint das Stützgewebe des menschlichen Rückenmarkes wenigstens bis in sehr späte Entwicklungsstufen hinein ausschliesslich als eine Bildung des äusseren Keimblattes.» Die radiären sog. »Pialsepta» erweisen sich als die zu grösseren Bündeln zusammengefassten peripheren Fortsätze tiefer gelegener Neurogliazellen. Im ausgebildeten Rückenmarke scheint derselbe Plan noch als Grundtypus, allerdings unter Hinzutritt ausgiebiger Complikationen, bis zu einem gewissen Grade in Geltung zu bleiben; die Hauptveränderungen bestehen in der viel ansehnlicheren Ausbreitung der von dem Zellkörper ausgehenden, zarten, sekundären Aestchen; der periphere Fortsatz verliert deshalb allmählig seine Bedeutung; die früher so häufig länglichen, spindelförmigen Zellen nehmen mehr und mehr einen spinnenförmigen Charakter an. Obgleich es sich im Grunde genommen überall um die gleiche Zellensorte handelt, bringt v. Lenhossék im Interesse der Darstellung die Zellen in drei Kategorien: 1) Ependymzellen, 2) Zellen der grauen und 3) Zellen der weissen Substanz. Die Ependymzellen haben gewöhnlich einen spindelförmigen Zellenkörper mit einer freien centralen cuticularen Platte, von deren Mitte beim 23 Cm. langen Embryo — bei dem 14 Cm. langen Embryo vermisste v. Lenhossék diese Stiftchen vollständig — je eine intensiv geschwärzte, starre, oft auffallend lange, in der Regel hakenförmig umgebogene Borste hervorragt, welche Borsten jedenfalls nicht als Flimmerhaare zu betrachten sind. Die Schilderung des feinen, stets bis zur Peripherie vordringenden Fortsatzes erfordert je nach den Regionen eine gesonderte Betrachtung. In der Gegend der vorderen Commissur zeigen die Ependymzellen eine meridianartige oder tonnenförmige Anordnung, indem ihre peripheren Enden nach der vorderen Längsfissur convergiren; die mittleren sind sagittal; stets sind sie ungetheilt und in der Regel etwas wellenförmig verlaufend, sowie stärker als die übrigen Fasern; gewöhnlich sind

[1] Mich. von Lenhossék, Zur Kenntnis der Neuroglia des menschlichen Rückenmarkes. Verhandl. d. Anatom. Gesellschaft auf der fünften Versamml. in München, 18—20 Mai 1891.

sie glatt und nur an ihrem Anfangstheil zuweilen mit zarten Seitenästchen besetzt. Am Uebergang zwischen der vorderen und der seitlichen Wand des Centralkanales folgt eine spärliche Zellengattung, deren ebenfalls bogenförmig medianwärts gewendete Ausläufer sich durch eine reichliche Theilung von allen anderen Ependymzellen auszeichnen; die 6—8 zarten, divergirenden Endästchen finden an der vorderen Fissur der zugekehrten Partie der Vorderstränge ihr stets dreieckig verdicktes Ende. Eine derartige Verästelung kommt hier auch beim Hühnerembryo (4—5. Tag) vor. Die Anordnung der seitlichen Ependymzellen ist eine sehr typische, indem sie radiär ausstrahlen; die meisten beschreiben einen mässigen Bogen, dessen Concavität bei denen der ventralen Hälfte nach vorn, bei denen der dorsalen nach hinten gewendet ist; in der Mitte dazwischen sind sie annähernd geradlinig. Ihr Gang ist ein zart welliger, vielleicht in Folge der Härtung; fast constant ist eine gabelige Theilung im Bereich der weissen Substanz vorhanden; die Enden zeigen die terminalen Knötchen. Zwischen den seitlichen und den hinteren, das sog. Septum posticum bildenden Fasern findet sich ein beträchtlicher Zwischenraum, indem das ganze, den Hinterhörnern und Hintersträngen entsprechende Gebiet der Ependymfasern entbehrt. Von Lenhossék beschreibt dann die eigenthümliche Reduction des dorsalen Theils des Centralkanales, der einem sagittalen Spalt ähnlich wird und durch Anlöthung seiner Flächen verschwindet, wobei die hinten in der Mittellinie befindlichen Zellen zwischen ihnen allmählig vorwärts rücken; hierbei verlieren eine Anzahl von diesen seitlichen Zellen ihren Ependymcharakter und wandeln sich in Deiters'sche Zellen um; sie behalten indessen oft ihre ursprüngliche Stellung, d. h. sie verbleiben senkrecht auf die sagittale Medianebene gestellt: das sind nun die früheren Ependymfasern der Hinterhörner und Hinterstränge. Die hintersten, relativ dicken, stets ungetheilten und etwas zackig verlaufenden Ependymzellen zeigen eine streng mediane Lage und dicht gedrängte bündelartige Anordnung. Das Septum posticum der Autoren ist kein Fortsatz der Pia mater, sondern es ist ependymal und gehört eigentlich der Rückenmarksubstanz an; das Mark ist dorsal ohne Frage ungetheilt und zeigt nur eine schwache Furche. Im Zusammanhange betrachtet erscheint die Zahl der Ependymzellen im Verhältniss zu den übrigen Elementen des Stützsystems unbedeutend.

Was die *Neurogliazellen der grauen Substanz* betrifft, so sind sie im Bereich der Substantia gelat. centralis zahlreich; sie haben eine plumpe, rundliche Form mit relativ reichem Faserbesatz, der eine dem Centralkanal concentrische Anordnung und eine glattrandige Beschaffenheit darbietet. Viele Neurogliazellen der grauen Substanz sind ausser dem peripherischen noch mit einem centralen Fortsatz versehen, der in der Regel kurz, zuweilen aber länger ist und gegen den Centralkanal hin zieht, ohne ihn zu erreichen. In den peripherischen Partien der grauen Substanz, namentlich im Gebiet zwischen dem Vorder- und dem Hinterhorn, kommen quer zur radiären Ausstrahlung der Fasern gelagerte, spindelförmige Elemente vor, deren beide in Fortsätze auslaufende Enden bogenförmig die Richtung nach der Peripherie einschlagen, die indessen nur das eine erreicht. Von Lenhossék beschreibt dann genau den eigenthümlichen Verlauf der Neurogliazellen der Hinterhörner und speciell der Substantia gelat. Rolandi, deren Eigenart hauptsächlich in der besonderen Beschaffenheit ihrer zahlreichen Neurogliazellen und Fasern, v. A. in deren pelzigem Charakter begründet ist. Die *Neurogliazellen der weissen* Substanz sind zwar in dieser Periode noch in relativ geringer Zahl vorhanden, zeigen jedoch eine grosse Mannigfaltigkeit der Formen; er theilt sie in vier Typen ein. *Typus 1,* der einfachste, findet sich v. A. in den Hintersträngen, wo ihm alle Zellen angehören; die Zelle ist länglich, radiär gestellt, entbehrt fast durchgehends eines centralen Ausläufers und ist nur mit einem einzigen peripherischen ausgestattet, der stets ungetheilt, glattrandig und etwas geschlängelt nach aussen zieht; die in den Vorder- und Seitensträngen befindlichen Exemplare dieser Zellenart sind dagegen mit zarten sekundären Aestchen versehen. *Typus 2* zeigt 2—5 oder mehr peripherische Fortsätze, die schon getrennt von der Zelle entspringen, und zwar von deren peripherischem Pol und in büschelförmiger, divergirender Anordnung; sie sind relativ stark, in der Regel glatt, doch auch oft varicös, wellig verlaufend und unterliegen oft noch weiterer Theilung; ein kurzer centraler Fortsatz gehört nicht zu den Seltenheiten. *Typus 3* besteht aus spindelförmigen Zellen, deren beide Enden in je einen Fortsatz auslaufen, welche Fortsätze bogenförmig nach der Peripherie hin ziehen und sich noch weiter theilen. *Typus 4* ist das Attribut der der vorderen Fissur zugekehrten Abtheilung der Vorderstränge und durch die reichliche, buschige Verästelung ihrer nach der Peripherie hin ziehenden, weiter getheilten Fortsätze charakterisirt. — Alle diese Zellentypen erfahren in ihrer weiteren Entwicklung eine Umwandlung, indem sich der spindelförmige Körper zu einer mehr gleichmässigen spinnenförmigen Figur reducirt und der Verlauf der Fortsätze mehr gerade wird. Die Neurogliazellen ordnen sich mehr und mehr einem gemeinsamen Typus unter; die Zahl der Aeste bleibt aber verschieden.

Ramón y Cajal[1] beschrieb das Ependym und die Neuroglia im Rückenmark und Gehirn der *Reptilien* (Lacerta) und im Gehirn der *Amphibien* (Rana) und *Vögel*. Pedro Ramón[2] gab ebenfalls eine Darstellung dieser Elemente im *Reptil*gehirn und Cl. Sala[3] im Rückenmark der *Amphibien* (Rana). Ich[4] bestätigte die Angaben Nansen's über die Neurogliazellen des Rückenmarks von *Myxine*.

Dann untersuchte M. von Lenhossék[5] die Neuroglia im Rückenmark der Pristiurusembryonen. Zwar vermochte er hier keine gelungenen Bilder der Ependymzellen zu erhalten, die übrigen Neurogliazellen aber, zumal diejenigen in der ventralen Markhälfte, wurden imprägnirt, und zwar mit kaffebrauner Färbung. Die kleinen eckigen Zellen sind nur mit Hauptfortsätzen ausgestattet; alle Ausläufer, deren es 5—6 oder noch mehr geben kann, ziehen nach der Oberfläche des Markes, wobei sie einen stark divergirenden Verlauf einschlagen, so dass die Ausbreitung einer einzigen Zelle fast eine ganze Hälfte des Markes spinnenartig zu umspannen vermag. Die Ausläufer sind von derber, steifer Beschaffenheit und endigen an der Oberfläche mit je einer kolbigen Verdickung. Sie nähern sich bis zu einem gewissen Grade dem Typus der Zellen der Cyclostomen.

Endlich hat von Lenhossék in seiner im vorigen Jahre erschienenen umfassenden Darstellung vom feineren Bau des Nervensystems[6] die Neuroglia des Rückenmarks in übersichtlicher Weise behandelt. Diese Darstellung schliesst sich im allgemeinen seinen früheren Mittheilungen an, doch enthält sie einige neue Angaben, z. B. über die Verhältnisse bei Petromyzon und Raja.

Da ich jetzt zu meinen eigenen Befunden übergehe, kann ich so gerne von vorn herein meinen Standpunkt in Betreff der Neurogliafrage angeben. Meine Ansichten stimmen in allem Wesentlichen mit denen von Golgi, Nansen, Ramón y Cajal und von Lenhossék überein. Deshalb brauche ich nach der obigen historischen Einleitung keine eingehendere Beschreibung zu liefern. Ich werde mich daher auf eine kürzere Darstellung meiner Befunde beschränken und hauptsächlich eine Erklärung der betreffenden Abbildungen geben.

---

1. Ependym und Neuroglia bei den Cyclostomen.

Taf. V—VII.

Die Verhältnisse bei Amphioxus bei Seite lassend, weil ich in dieser Hinsicht den Angaben von Nansen, Rohde und von Lenhossék nichts hinzuzufügen habe, fange ich mit den Cyclostomen an. In Betreff der *Myxine* haben meine fortgesetzten Untersuchungen über Ependym und Neuroglia des Rückenmarkes die von mir schon vorher bestätigten Befunde Nansen's noch weiter bekräftigt. Im Gehirn dieses Thieres bekam ich zwar auch eine Färbung dieser Elemente (s. u.). Ich werde aber hier nicht die Myxine besprechen, sondern sogleich zu den Verhältnissen bei *Petromyzon* übergehen. Schon in meiner Darstellung vom Nervensystem der Myxine (Biol. Unters. II, 1891) erwähnte ich, dass die mit der Golgi'schen Methode gewonnenen Präparate vom Rückenmark des Petromyzon denen von Myxine sehr ähnlich sind, dies »sowohl in Betreff der Ganglienzellen und ihrer Fortsätze wie auch der Neurogliazellen». Es war meine Absicht, gleichzeitig eine Abbildung dieser Präparate zu veröffentlichen, die angefertigte Figur (die hier unten mitgetheilte Fig. 1 d. Taf. V) war aber verlegt worden. Seitdem hat von Lenhossék im vorigen Jahre in seiner eben

[1] S. Ramón y Cajal, Pequeñas Contribuciones al Conocimiento del sistema nervioso. Trab. d. Laborat. histol. de la facultad de medicina de Barcelona. Aug. 1891.
[2] Pedro Ramón, El encéfalo de los Reptiles. Trab. d. Laborat. de histología de la facultad de medicina de Zaragoza. Sept. 1891.
[3] Cl. Sala, Estructura de la médula espinal de los batracios. Trab. del Laborat. de histología de la facultad de medicina de Barcelona. Febr. 1892.
[4] Gustaf Retzius, Zur Kenntniss des Nervensystems von Myxine glutinosa. Biol. Unters. N. F. II,3. 1892.
[5] M. von Lenhossék, Beobachtungen an den Spinalganglien und dem Rückenmark von Pristiurusembryonen. Anatom. Anzeiger, 7. Jahrg., 1892.
[6] M. von Lenhossék, Der feinere Bau des Nervensystems im Lichte neuester Forschungen. Fortschritte der Medicin, 1892.

erwähnten Arbeit in »Fortschritte der Medicin» eine gute Abbildung des Petromyzon-Rückenmarkes veröffentlicht. »Die Ependymfasern», sagt er in der Beschreibung, »erscheinen glatt, zart und sind sehr spärlich; dagegen finden wir zahlreiche Gliazellen, die sich aber nicht gleichmässig auf den ganzen Querschnitt vertheilen, sondern sich mit ihrem Zellkörper auf die einem Streifen ähnliche graue Substanz beschränken. Sie zeichnen sich alle durch enorme Verästelung aus, wobei es sich nicht um secundäre, sondern mit geringen Ausnahmen um lauter primäre, die Peripherie erreichende Aeste handelt. Jede Zelle lässt sowohl an die ventrale wie an die dorsale Fläche des Markes ein Buschwerk von Zweigen herantreten (im Gegensatz zu den Abbildungen von Nansen und Retzius, wo sie nur mit einseitiger Ausbreitung dargestellt sind), die am seitlichsten gelegenen auch an die laterale Kante des Markes. Die medialen Aeste der zu beiden Seiten der Mittellinie befindlichen Zellen kreuzen sich vielfach vor und hinter dem Centralkanal.» Diese seine Darstellung stimmt zwar vollständig mit meinen Befunden bei Petromyzon überein. In Betreff der von Nansen und *mir* vom Myxine-Rückenmark angegebenen ist sie jedoch nicht ganz exact. Sowohl Nansen (Pl. XI, Fig. 103) u. s. w., wie *ich* (Fig. J, S. 51, Biol. Unt. II) habe auch Neurogliazellen abgebildet, deren Fortsätze nicht einseitig nach der Peripherie des Markes, sondern sowohl nach der dorsalen, wie nach der ventralen Fläche derselben verlaufen und die Oberfläche erreichen, gerade wie es von Lenhossék bei Petromyzon abbildet. In dem Texte sagt Nansen ferner: »The same neuroglia cells sends often, at the same time, processes to the dorsal side as well as to the ventral side of the spinal cord.» In meinem Texte sage ich betreffs solcher Zellen: »Zuweilen gehen auch, wie Nansen gezeigt hat, nach entgegengesetzter Richtung feine Fasern aus, und zuweilen reichen dieselben wie ein Büschel bis an die Oberfläche.» Indessen liegt in der That in dieser Hinsicht eine Differenz zwischen Myxine und Petromyzon vor, indem bei Myxine nur ein Theil der Neurogliazellen Fortsätze nach beiden Flächen, sowohl nach der dorsalen, wie nach der ventralen schickt; ein grosser Theil sendet nur nach einer Seite solche Fortsätze aus. Bei Petromyzon hingegen scheint dies letztere ein ausserordentlich seltenes Vorkommniss zu sein: die Neurogliazellen schicken fast constant bilateral Fortsätze aus, welche die ventrale und dorsale Fläche erreichen.

Ich untersuchte vom Petromyzon fluviatilis theils Larven (Ammocoetes) von verschiedener Grösse (5—20 Cm. lange Exemplare), theils umgewandelte Junge (von 25—30 Cm. Länge), theils auch erwachsene Individuen. Im Schwanztheil der letzteren bekam ich mit der schnellen Golgi'schen Methode mehrmals gute Färbung der Neuroglia und der Ganglienzellen des Markes. Vor Allem aber erhielt ich gute Präparate bei Larvenformen in verschiedenen Stadien und bei neulich umgewandelten Individuen. Hier war auch im Gehirn oft eine gute Färbung der Elemente vorhanden. Auf das Verhalten der Ganglienzellen und Nervenfasern gehe ich indessen diesmal nicht ein, da dies nicht in dem Plane dieser Mittheilung liegt. In den Figuren habe ich jedoch eine Anzahl von Ganglienzellen wiedergegeben, um den verschiedenen Typus derselben und der Neurogliazellen zum Vergleich darzulegen. In Betreff der Structurverhältnisse des Ependyms und der Neuroglia finde ich bei den untersuchten Larvenstadien und erwachsenen Exemplaren keine bemerkenswerthen Unterschiede, weshalb ich hier keine besondere Beschreibung von ihnen zu geben brauche.

Wie von Lenhossék hervorhebt, befinden sich die Zellenkörper der *Neurogliazellen* in dem abgeplatteten, aber dorsal convexen, ventral concaven Rückenmarke von Petromyzon in dem inneren schmalen Bande, das der grauen Substanz des Markes entspricht. Die Zellenkörper (Taf. V, Fig. 1—4) liegen jedoch in etwas verschiedener Höhe, sind im Ganzen klein und haben eine verschiedene, aber in der Regel unregelmässig polygonale Gestalt. Von ihnen entspringt nun in der Regel nach zwei Richtungen hin, dorsal- und ventralwärts, je ein Büschel von steifen, mehr oder weniger schmalen, im Allgemeinen glatten, hier und da aber auch etwas gezahnten oder varicösen Fortsätzen, die bald einzeln direct vom Zellenkörper, bald aber von einem dickeren Aste desselben ausgehen. Diese Fortsätze spreizen mehr oder weniger aus einander und ziehen steif oder ein wenig geschlängelt und meistens ungetheilt, hier und da aber auch dichotomisch verzweigt, nach der dorsalen und ventralen Oberfläche des Markes, wo sie mit knopfförmiger oder konischer, etwas verschieden grosser Verdickung endigen. Wenn mehrere nebeneinander liegende Neurogliazellen gefärbt sind, was oft in den Präparaten zu sehen ist, kreuzen sich ihre Fortsätze in meistens spitzen Winkeln (Fig. 1, 4); hierdurch entsteht auf dem Querschnitt das eigenthümliche Bild eines Strickwerkes, das, von den Knotenpunkten (Zellenkörpern) ausgehend, gewissermassen die dorsale und die ventrale Oberfläche des Markes mit einander verbindet. Die Ausbreitung der Fortsätze ist nicht platt, und sie geschieht auch nicht in einer Ebene, sondern nach verschiedenen Seiten hin, wovon man sich am besten an Flächenansichten des nach

der Golgi'schen Methode gefärbten Markes überzeugen kann. Die Gestalt dieser Neurogliazellen ist zwar sehr charakteristisch und typisch; sie wechselt jedoch, wie aus den beigegebenen Figuren hervorgeht, recht sehr, dies sowohl in Betreff der Grösse und Form des Zellenkörpers, wie auch der Anzahl und des Verlaufes der Fortsätze. In den vorderen Partien des Markes, wo die dicken Müllerschen Fasern vorhanden sind (Taf. V, Fig. 4), biegen die Fortsätze auf verschiedenen, zuweilen recht verwickelten Bahnen um und ziehen zwischen den Fasern gegen die ventrale Oberfläche hinaus. Am lateralen Rande des Markes sind die Fortsätze kurz und spreizen, wie auch von Lenhossék hervorhebt, mehr oder weniger radiirend, gewissermassen sonnenschirmartig aus einander, indem sie sich hier nicht nur zu zwei Büscheln anordnen, sondern auch lateral vom Zellenkörper entspringen und ebensowohl die abgerundete Kante des Markes, wie die dorsale und ventrale Fläche erreichen (Taf. V, Fig. 1, 2, 4).

Die *Ependymzellen* des Rückenmarkes von Petromyzon haben einen von den Neurogliazellen ganz differenten Typus. Sie zeigen eine echt embryonale Gestalt, treten aber wenig hervor und färben sich nach der Golgi'schen Methode nur hier und da. Am ehesten werden die Ependymzellen gefärbt, die dorsalwärts vom Centralkanal liegen; ihre Zellenkörper und der nächste Theil des peripheren Fortsatzes treten am schärfsten hervor. In anderen Fällen kommen aber auch lateral ziehende Ependymzellen zum Vorschein. Aus solchen Präparaten (Taf. V, Fig. 1, 2, 4 *e*) geht hervor, dass die Ependymzellen, wie Nansen bei Myxine und von Lenhossék bei Petromyzon dargestellt haben, mit ihren schmalen kernfürenden Zellenkörpern rings um den Centralkanal angeordnet sind und je einen feinen, meistens unverzweigten und wenig knotigen Fortsatz in wenig gewundenem Verlaufe nach der Peripherie des Markes schicken; im Allgemeinen ist es schwer, diesen Fortsatz bis zur Oberfläche zu verfolgen, hier und da gelingt es jedoch; haarartige, in den Centralkanal einschiessende Anhängsel traf ich am centralen Ende der Ependymzellen des Markes nicht an.

Uebergangs- oder Zwischenformen der Ependym- und Neurogliazellen sah ich hier nie, ebenso wenig bei Myxine, so dass ich kaum die Gründe zu verstehen vermag, die Nansen für die Entstehung der Neurogliazellen des Markes aus den Ependymzellen als beweisend hervorgehoben hat; jedenfalls ist diese Relation bei den höheren Thieren viel deutlicher; ich bezweifle indessen nicht, dass ein näheres Studium der embryonalen Entwicklung des Rückenmarkes auch bei den Cyclostomen eine derartige Zusammengehörigkeit darlegen wird.

Als ich dann, zu meinem Erstaunen, im verlängerten Marke und Gehirn des Petromyzon einen ganz anderen Typus des Stützgerüstes entdeckte, den ich weiter unten beschreiben werde, suchte ich in der Uebergangspartie zwischen der Medulla spinalis und oblongata die Uebergangsformen der Elemente zu finden. In der Fig. 1 der Taf. VI habe ich einen Querschnitt des vordersten Endes des Rückenmarkes wiedergegeben. Das ganze Mark zeigt sonst den oben beschriebenen Typus, und erst im vordersten Theil, wo die bandartige Gestalt des Markes in eine etwas abgeplattet-cylindrische übergeht, erhalten die Neurogliazellen eine modificirte Beschaffenheit. Wie aus der angeführten Figur hervorgeht, sind die Zellenkörper nicht mehr alle auf einen Streifen zusammengeführt, sondern sie finden sich theils in der Umgebung des Centralkanales, theils auch weiter nach aussen hin, sogar in der Nähe der Oberfläche. Die in der Umgebung oder eigentlich zu beiden Seiten des Centralkanales befindlichen Neurogliazellen haben im Ganzen den für das übrige Mark charakteristischen Typus bewahrt, nur sind in Folge der veränderten Gestalt des Markes die Fortsätze, um die dorsale und ventrale Oberfläche des Markes zu erreichen, viel länger geworden. Weiter nach aussen hin trifft man aber Elemente an, die verschiedenartig gestaltet sind und grösstentheils ihre Fortsätze nur nach einer Fläche des Markes schicken; bald liegen die Zellenkörper in der Nähe der Oberfläche, bald mehr von ihr entfernt. Im Ganzen ähneln diese Neurogliazellen in auffallendem Grade den entsprechenden Elementen der weissen Marksubstanz der höheren Thiere. Was die Ependymzellen betrifft, so kommen sie selten zum Vorschein; in der Fig. 1 der Taf. VI ist nur eine solche Zelle abgebildet, sie zeigt aber denselben Typus wie im übrigen Marke.

Ein wenig nach vorn von dieser Gegend des Markes, an dessen allervorderstem Ende, treten nun die beschriebenen Formen der Neurogliazellen sehr zurück; man trifft sie noch in der Umgebung des Centralkanales an (Fig. 1 der Taf. VII); hier haben sie sich gewissermassen concentrisch um ihn angeordnet; ich konnte ihre Fortsätze nicht mehr bis zur Oberfläche verfolgen. Dagegen ist eine ganz neue Art von Stützelementen aufgetreten, die in verschiedener Entfernung vom Centralkanal nach aussen hin einen spindelförmigen, mit zahlreichen kurzen Aestchen ausgerüsteten Zellenkörper zeigen; von diesen geht in radiirender Richtung ein peripherischer Fortsatz aus, der sich bald wiederholt dichotomisch theilt; hierdurch entsteht ein dendritisches Büschel feiner, etwas knotig gezackter und wellig verlaufender Fäserchen, die in konisch angeordneter Ausbreitung gegen die Peripherie des Markes hin streben

und seine Oberfläche erreichen, wo sie mit je einem Knötchen endigen. Die eigentlichen Ependymzellen konnte ich hier nicht gefärbt bekommen.

Als ich jetzt zu der näheren Untersuchung der *Medulla oblongata* überging, fand ich, wie es in Fig. 2 der Taf. VI wiedergegeben ist, in dem ganzen Umfang dieses Gehirntheiles nur die eben beschriebene Art der Stützelemente. Die meisten derselben erwiesen sich aber als echte Ependymzellen, indem die kernführenden, schmal spindelförmigen Zellenkörper sich in der nächsten Umgebung des Centralkanales befinden, einen centralen Fortsatz bis zu diesem Kanal schicken und zuweilen sogar eine haarartige Verlängerung in seine Höhle hineinsenden, den anderen peripherischen Fortsatz dagegen radiär nach aussen richten, um nach mehrfacher dichotomischer Verästelung und unter etwas geschlängeltem, wellenförmigem Verlaufe bis zur Oberfläche des Markes zu gelangen, wo sie knopfförmig endigen. Alle diese peripheren Aeste sind ausserdem mit kurzen und dichten Zacken oder Knötchen versehen. Zwischen diesen echten Ependymzellen sind hier und da einzelne Elemente eingestreut, deren Zellenkörper nicht den Centralkanal erreichen, sondern etwas nach aussen davon liegen, mehr dick spindelförmig sind und sich mit moosigen Aestchen versehen zeigen. Diese Zellen, von denen ich einige Exemplare in der Fig. 2 der Taf. VI abgebildet habe, entsprechen vollständig den in der Fig. 1 der Taf. VII beschriebenen Elementen. In der Medulla oblongata konnte ich dagegen keine solche, für das eigentliche Rückenmark der Cyclostomen so charakteristische Neurogliaelemente finden.

Auch in dem übrigen *Gehirn* des Petromyzon war dies der Fall. Ueberall traf ich ependymale Elemente, deren peripherischer Fortsatz unter spitzwinkliger dichotomischer Verzweigung radiär nach aussen verlief. Zwar vermochte ich die Aestchen nicht bis zur Peripherie zu verfolgen; ihre ganze Anordnung deutete aber auf einen derartigen Verlauf hin. In der Fig. 4 der Taf. VII habe ich bei *e, e* einige solche Ependymzellen abgebildet und nebenbei einige Ganglienzellen verschiedenen Calibers eingezeichnet, um die zwischen den beiden Elementen vorhandenen Differenzen und die Uebereinstimmung der Anordnung derselben in gewisser Hinsicht darzulegen. In den Fig. 2 und 3 der Taf. VII habe ich noch zwei Partien aus dem Gehirn wiedergegeben, wo die centralen Theile der Ependymzellen zu sehen sind. Nicht nur die Verästelung der mit feinen Seitenästchen reichlich besetzten peripheren Fortsätze ist hier schön zu erkennen, sondern auch die Gestalt der meistens flaschenförmigen Zellenkörper, die mit einer centralen sog. cuticularen Platte versehen sind, aus der Haaranhängsel verschiedener Art hervorragen. An einigen Zellen sieht man nur ein einziges langes Haar, an anderen sind zwei vorhanden, an anderen drei oder mehr, so dass Zellen vorkommen, die einen wahren »Flimmerbesatz» tragen. Ob diese Haare wirklich während des Lebens flimmern, ist mir nicht bekannt. Jedenfalls ist aber das Vorkommen von einhaarigen und mehrhaarigen Zellen neben einander für die Erklärung der sonst gewöhnlich, v. A. am Centralkanal des Rückenmarks anderer Wirbelthiere vorkommenden, einhaarigen Ependymzellen von einem gewissen Interesse.

Durch die hier gelieferte Darstellung ist gezeigt worden, dass schon bei einem Cyclostomen ganz verschiedene Typen des Stützgewebes vorhanden sind, indem die für das eigentliche Rückenmark dieser Thiere charakteristischen Neurogliazellen in der Medulla oblongata und im Gehirn dem Typus der Ependymzellen weichen, der besonders bei Amphibien und Reptilien der allgemein vorkommende ist. Im Gehirn von Petromyzon habe ich in der That, wie in der Medulla oblongata, keine andere Art von Neurogliaelemente angetroffen, v. A. nicht die für das Rückenmark typischen.

2. **Bei Knochenfischen.**

Taf. VIII.

Bei den Teleostiern wurde mittelst der früheren Methoden theilweise die peripherische Fortsetzung der Ependymzellen beobachtet (Stieda, Mauthner) und mittelst der Golgi'schen Methode erhielt ich in der Medulla oblongata und den Grosshirnganglien des erwachsenen Hechtes solche Zellen, welche von den centralen Höhlen bis zur Peripherie hin radiär verlaufen.

In der neueren Zeit scheint indessen diese Thiergruppe mittelst der Golgi'schen Methode nicht bearbeitet worden zu sein. Es ist lange meine Absicht gewesen, diese Lücke auszufüllen und zugleich die Knorpelfische in den Kreiss meiner Untersuchungen zu ziehen. Versuche, die ich bei erwachsenen Exemplaren und beinahe aus-

getragenen Foetus von Acanthias vor einigen Jahren mittelst der Chromsilbermethode ausführte, gaben keine erläuternden Resultate, und kleinere Embryonen standen mir damals nicht zur Verfügung. Im vorigen Jahre hat nun, wie oben erwähnt worden ist, von Lenhossék seine interessanten Untersuchungen an Pristiurusembryonen veröffentlicht, so dass bei den Knorpelfischen die wichtigsten hierauf bezüglichen Verhältnisse bekannt sind. Ich wendete mich deshalb in erster Linie zu den *Knochenfischen.* Da es hierbei v. A. gilt, die embryonalen Verhältnisse zu untersuchen, so habe ich mir eine Anzahl von Eiern und ausgeschlüpften Jungen eines Teleostiers, *Salmo Salar,* von einer Fischzuchtanstalt bei Elfkarleby in Schweden verschafft und die Jungen lange am Leben erhalten, um sie in verschiedenen Stadien zu untersuchen. Zuerst mislangen die Färbungsversuche nach der Golgi'schen Methode. Nachdem ich aber die erste Einwirkungsdauer der Chromosmium-Mischung bis auf einen Tag herabgesetzt und nach Cajal's Vorschrift eine doppelte Behandlung angewandt hatte, bekam ich eine Reihe schöner Färbungen sowohl vom Ependym, wie von den nervösen Elementen des Rückenmarks und des Gehirns. Was die letzteren betrifft, so werde ich sie in einer folgenden Abtheilung dieses Bandes beschreiben, und ich beschränke mich daher hier zunächst auf die Verhältnisse des Stützgewebes.

Ich habe eine grosse Anzahl von Lachsjungen von 16 mm. Länge (noch im Ei) bis auf 30 mm. Länge, (einen Monat nach dem Ausschlüpfen) untersucht. Bei allen waren die Verhältnisse so übereinstimmend, dass ich nur eine übersichtliche Darstellung liefern werde.

Im ganzen *Rückenmark* der jungen Lachsen bekommt man in Querschnitten rings um den entweder mehr breit oder schmal spaltförmigen, in sagittaler Richtung jedenfalls verlängerten Centralkanal, die in gefärbtem Zustande kastanienbraunen oder mehr geschwärzten Ependymzellen scharf hervortretend. In den Fig. 1—3 der Taf. VIII habe ich drei solche Querschnitte, in denen die gewöhnlich vorkommenden Formen zusammengestellt sind, wiedergegeben. Oft färben sich in jedem Schnitte nur einige Ependymzellen, und dieselben sind daher in ihrem ganzen Umfange leicht zu verfolgen; wenn, wie es hin und wieder der Fall ist, eine grössere Anzahl gefärbt vorliegt, lassen sie sich schwerer von einander unterscheiden.

In dem hintersten Ende des Markes sind die Verhältnisse am primitivsten; hier haben die Ependymzellen noch den Charakter echter, schmaler Cylinderzellen bewahrt, die vom Centralkanal bis zur Peripherie radiär angeordnet sind; die Zellen tragen keine oder sehr wenige Seitenästchen. Etwas weiter nach vorn hin verändert sich der Typus der Zellen in der Hinsicht, dass ihr peripherischer Fortsatz eine Menge feiner, kurzer, seitlicher Aestchen abgiebt; das centrale Ende enthält den länglich-spindelförmigen Kern und stellt mithin den Zellenkörper dar, der in der Regel etwas zugespitzt die Höhle des Centralkanales erreicht. In der Fig. 1 der Taf. VIII ist ein solcher Querschnitt abgebildet; vier Ependymzellen sind darin wiedergegeben und zum Vergleich noch vier Ganglienzellen eingezeichnet. Der in Fig. 1 dargestellte Typus der Ependymzellen herrscht nun im ganzen Rückenmarke vor und stellt die gewöhnliche Form derselben dar; von diesem Typus lassen sich alle übrigen Formen ableiten, die eigentlich nur Variationen von ihm sind. In den Fig. 2 und 3 der Taf. VIII sind sowohl die mehr regulären Formen, wie eine Anzahl von Modificationen des Typus wiedergegeben. Die meisten dieser Zellen tragen ihren Zellenkörper radiär gegen den Centralkanal gestellt. Der ovale oder schmal spindel- oder zuweilen fast stabförmige Kern ist von verschiedener Grösse; in Uebereinstimmung damit wechselt auch die Grösse des Zellenkörpers; seine Form richtet sich ebenfalls im Ganzen nach der des Kerns; zuweilen liegt der den Kern beherbergende Körper mit seiner Hauptmasse neben dem Kanal, und er zeigt dann eine dreieckige Form (rechts in der Fig. 2 der Tafel VIII). Der Zellenkörper ist an seiner Oberfläche entweder glatt oder etwas gezackt und uneben. Von seinem äusseren Ende entspringt der einzige periphere Fortsatz; derselbe ist zwar von etwas verschiedener Dicke, im Ganzen aber recht dick; er zieht geradlinig oder etwas geschlängelt radiär nach der Oberfläche des Markes hin. Gleich nach dem Abgang vom Zellenkörper zeigt er gewöhnlich einige rechtwinklige, stärkere Zweige, die verschieden weit hinausragen; nach aussen hin aber verändert sich die Beschaffenheit der Aeste, indem sie fein, knotig, sogar moosartig werden und sehr gedrängt liegen. Hierdurch entsteht das eigenthümliche Aussehen der peripheren Fortsätze, das in den beigegebenen Figuren in mehreren Variationen wiedergegeben ist. Die Anzahl der seitlichen Aestchen unterliegt indessen sehr dem Wechsel; sie kann in einzelnen Fällen ziemlich gering sein (links unten in der Fig. 3 der Taf. VIII), in anderen können die Aestchen aber massenhaft, sogar bartartig auftreten, wie einige Ependymzellen in derselben Figur zeigen. Wie ich oben hervorgehoben habe, ist der periphere Fortsatz, von diesen feinen Aestchen abgesehen, in der Regel ungetheilt; es kommt aber vor, dass er auch etwas stärkere Seitenäste abgiebt,

und in einzelnen Fällen kann er sich auch in 2—3 Hauptzweige theilen, die dann getrennt nach der Peripherie verlaufen. Auf der weiter unten folgenden Tafel, die die Nervenzellen wiedergiebt, ist eine solche getheilte Ependymzelle dargestellt.

In der Mittelpartie vorn und hinten sind etwas verschiedene Verhältnisse vorhanden. Die Ependymzellen zeigen hier oft eine geknickte Gestalt, indem der Zellenkörper sagittal steht, der periphere Fortsatz aber rechtwinklig nach aussen hin umbiegt (Fig. 1, 2 der Tafel VIII). In der hinteren sagittalen Mittellinie ist die Anordnung der Ependymzellen noch eigenthümlicher. Viele Zellen reichen mit ihren Zellenkörpern nicht bis zum Centralkanal hinan, sondern sie zeigen dieselben mehr oder weniger senkrecht gegen das sog. »Septum posticum» gestellt. Es liegt also hier, wie die Fig. 3 der Taf. VIII wiedergiebt, gewissermassen eine derjenigen ähnliche Anordnung vor, die v. A. von Lenhossék beim Menschen beschrieben hat. Offenbar ist hier, wie dort, die frühere hintere Spalte des Centralkanales geschlossen worden; die Ependymzellen und zu echten Neurogliazellen gewordenen Stützzellen stehen aber noch gegen das »Septum» gerichtet; in der Mittellinie, im »Septum», findet man jedoch lange, schmale, wenig verzweigte Zellen (Fig. 3 der Taf. VIII und unten auf der Tafel XIV über den Nervenzellen des Markes), deren Kerne eigenthümlicher Weise noch in der äussersten Peripherie liegen; wahrscheinlich haben sie ihre centralen Fortsätze von hinten in das Septum und bei dem Verschluss der Spalte des Kanales sodann nach vorn hin geschickt.

Wie man aus den Figuren sieht, bilden die hier geschilderten Stützzellen des Rückenmarks ein gegen die Ganglienzellen sehr contrastirendes, robustes, buschiges Zellenelement von sehr charakteristischer Form. Innere haarartige Anhängsel oder gar Flimmerhaare, die in den Kanal hineinragen, habe ich bei den jungen Lachsen nicht gesehen. Bei älteren Individuen von Salmo, Gobius, Gasterosteus und Anguilla traf ich im Rückenmarke Ependymzellen von demselben Typus an; nur zeigte der periphere Fortsatz oft eine stärkere, wiederholte, sogar büschelartige Verzweigung.

Anderweitige zu diesem Stützsystem gehörige Elemente habe ich im Rückenmarke nicht gefunden. In der Medulla oblongata und im Gehirn verändert sich der Typus der Ependymzellen, indem dieselben viel schmäler und schlanker werden und sehr wenige Aestchen abgeben; hin und wieder theilt sich aber der periphere Fortsatz auf dem Wege nach der Oberfläche hin, und dies sogar wiederholt dichotomisch. In der Fig. 4 *a, b* der Tafel VIII habe ich eine Partie eines Querschnittes des Grosshirns dargestellt; die eigentlichen Ependymzellen sind hier leicht zu erkennen; dagegen ist es in der That recht schwer zu bestimmen, welche von den Zellen, deren Zellenkörper nicht bis zum Hirnventrikel hinanreichen, als Ependymzellen oder als Ganglienzellen aufzufassen sind.

3. Bei den Amphibien.

Taf. IX.

Ependym und Neuroglia bei den Amphibien sind schon mehrmals der Gegenstand von Untersuchungen gewesen. Schon Reissner scheint hier die nach aussen hin ziehenden Fortsätze der Ependymzellen gesehen zu haben. Mittelst der Golgi'schen Methode haben Oyarzun, *ich*, Ramón y Cajal und Cl. Sala das in Rede stehende Thema genauer untersucht.

Ich habe diesmal hauptsächlich Larven von *Salamandra maculata* und *Rana temporaria* sowie junge Individuen der letzteren Thierart untersucht. Meine bei diesen Untersuchungen erhaltenen Befunde stimmen mit den bei früheren gewonnenen so gut überein, dass ich eine Beschreibung derselben nicht für nöthig erachte. Ich weise deshalb nur auf die Figuren hin (Taf. IX). Die Fig. 1 stellt einen Querschnitt des Rückenmarkes einer 2.5 cm. langen Salamanderlarve dar; die Ependym- resp. Neurogliazellen (d. h. diejenigen Zellen, welche ihren Körper nach aussen vom Centralkanal haben) zeigen den bekannten Typus mit büschelförmigem, oft schon in der grauen Substanz verzweigtem peripherem Fortsatz, dessen Zweige einen nach aussen hin radiirenden Verlauf zeigen; zum Vergleich habe ich in der Figur noch einige Ganglienzellen abgebildet. Die Fig. 2 stellt die laterale Partie der Medulla oblongata einer 2.5 cm. langen Salamanderlarve dar; der Typus der Ependymzellen ist derselbe wie im Rückenmark, nur tritt die konisch gestaltete Verzweigung noch früher ein. In Fig. 3 ist eine Partie einer Grosshirnhemisphäre abgebildet; die Ependymzellen zeigen die von Oyarzun, *mir* und Ramón y Cajal bei den Amphibien beschriebene Gestalt. Die

Fig. 4 giebt den Querschnitt des Rückenmarks von einer jungen, ganz umgewandelten Rana wieder; die Anordnung und Beschaffenheit der Zellen stimmt hier ganz mit den Verhältnissen bei der Salamanderlarve überein, ebenso auch mit der schönen Figur, die Cl. Sala vom Rückenmarke des erwachsenen Frosches giebt; hervorzuheben ist v. A. das Verhalten der Neurogliazellen in der vorderen Fissur und zu dem »Septum posticum». Die Fig. 5 stellt die Form der Ependymzellen in dem Querschnitt der Medulla oblongata dar; hier ist derselbe Typus vorhanden. In Fig. 7 sieht man die obere-mediale Partie eines Querschnitts der Grosshirnhemisphäre (Vorderhirn) eines jungen Frosches; der Typus der Ependymzellen ist derselbe, nur trifft ihre Verzweigung hier etwas weiter nach aussen ein; zum Vergleich ist eine Anzahl der Ganglienzellen in der Figur abgebildet. In der Fig. 6 habe ich eine Partie des Mittelhirns eines jungen Frosches dargestellt; hier haben die Ependymzellen einen anderen Typus, indem ihr langer peripherischer Fortsatz fast gerade nach aussen hin zieht, ohne dichotomische Theilungen einzugehen; dagegen schicken sie rechts zahlreiche kleine moosige Aestchen nach den Seiten hin aus, was besonders gegen das äussere Ende hin der Fall ist. Hier habe ich ebenfalls eine Anzahl der in verschiedener Weise angeordneten Ganglienzellen in die Figur eingetragen.

Bei den Amphibien herrscht also im Allgemeinen, sowohl im Rückenmark wie in der Medulla oblongata und im Vorderhirn, derselbe Typus von Ependym- resp. Neurogliazellen, nämlich derjenige, wo der periphere Fortsatz nach aussen hin mit konisch gestalteter, reichlicher Verästelung versehen ist. Es ist derselbe Typus, den man zuerst in der Medulla oblongata und im Gehirn von Petromyzon antrifft.

Anderartige Neurogliazellen als die oben beschriebenen habe ich bei den Amphibien nicht gefunden.

4. Bei Vögeln.

Taf. VIII, Fig. 5—8.

Für die Untersuchung des Ependyms und der Neuroglia bei den Reptilien stand mir kein passendes Material zu Gebote; durch die trefflichen Arbeiten von Ramón y Cajal und Pedro Ramón kennt man aber die in Rede stehende Frage schon in ihren wesentlichen Zügen. Was die Vögel betrifft, so ist sie auch v. A. durch Golgi, Ramón y Cajal und v. Lenhossék zu einem gewissen Abschluss gebracht worden, so dass es sich hier kaum lohnt, auf sie näher einzugehen, zumal meine Befunde mit denen der genannten Forscher ganz übereinstimmen. Aus meinen zahlreichen Präparaten vom Rückenmarke der Hühnerembryonen theile ich deshalb nur ein Bild mit (Fig. 5 der Taf. VIII); dasselbe giebt den Querschnitt eines achttägigen Embryos wieder. Man wundert sich vielleicht, dass das Mark noch in diesem Stadium so primitive Verhältnisse aufweist, indem es nur wenig weiter entwickelt erscheint, als das von v. Lenhossék vom viertägigen Embryo abgebildete. Dies erklärt sich indessen dadurch, dass, wie ich gefunden habe, der distalste Theil des Markes sich gesetzmässig später entwickelt als das übrige Mark, worüber ich mich in einem folgenden Capitel etwas ausführlicher äussern werde. Wie die Fig. 5 zeigt, stellt der Centralkanal noch eine lange, sagittale Spalte dar, und der vordere und hintere Ependymkeil sind noch sehr kurz und primitiv. Die Stützzellen reichen sämmtlich vom Centralkanal bis zur Oberfläche; nur die am vorderen und dem antero-lateralen Umfang befindlichen sind noch am äusseren Ende verzweigt; keine eigentlichen Neurogliazellen sind sichtbar; die »weisse» Substanz bildet eine dünne (durch eine punktirte Linie in der Figur angegebene Mantelschicht, die hinten und vorn fehlt. In älteren Stadien des Markes treten dann die von Golgi, Cajal und v. Lenhossék sowie auch von Lachi beschriebenen Neurogliazellen auf. In der Fig. 6 der Taf. VIII habe ich (vom 14-tägigen Embryo) einige solche Zellen in dem bekannten Entwickelungsstadium abgebildet, wo die Zellenkörper nach aussen vom Centralkanal, aber von ihm etwas entfernt liegen und den peripheren Fortsatz, gleich den echten Ependymzellen, nach der Oberfläche hin schicken. Zum Vergleich habe ich endlich aus dem Vorderhirn des 17-tägigen Embryos einige Ependymzellen abgebildet, bei denen keine Verzweigung des peripheren Fortsatzes vorhanden ist. Ich theile diese Bruchstücke nur als Beispiele mit.

5. Bei Säugethieren.

Taf. X.

Ich habe in dieser Beziehung *Maus, Ratte, Katze, Hund* und *Mensch* untersucht, und zwar v. A. das embryonale und junge Rückenmark, das Kleinhirn und das Grosshirn.

Im Rückenmarke der untersuchten Säugethiere zeigten Ependym und Neuroglia im Grossen und Ganzen so übereinstimmende Verhältnisse, dass es in der That nicht nöthig ist, für jedes Thier eine besondere Darstellung zu geben.

Das jüngste Stadium, in dem ich bei Säugethieren gute Färbung des Stützgerüstes im Rückenmarke bekommen habe, ist der 27 mm. lange Katzenembryo. Ich habe schon vor zwei Jahren eine Figur und Beschreibung von einem Stadium geliefert, das diesem sehr nahe steht (v. 30 mm. langen Embryo), füge aber den auf der Taf. X mitgetheilten Figuren noch eine Abbildung desselben bei (Fig. 1). Man sieht hier den langen, spaltförmigen Querschnitt des Centralkanales, den vorderen und hinteren Ependymkeil und die die ganzen lateralen Felder radiär durchziehenden Ependymzellen, deren periphere Fortsätze sich nur in den vorderen und antero-lateralen Regionen am äusseren Ende verzweigen, um an der Oberfläche knotig verdickt zu endigen. Die früher von mir beschriebene hakenförmige Umbiegung der äussersten Enden kommt zwar in verschiedenen Präparaten vor, scheint aber keine constante Bildung zu sein; vielleicht rührt sie von einer Schrumpfung der Oberfläche bei der Erhärtung her. Noch ist hier keine Art von Neurogliazellen nachweisbar; wohl aber sind einige radiäre Neurogliazellen im Halsmarke vorhanden.

Von den folgenden Stadien habe ich die besten Bilder bei Hundeembryonen erhalten. In der Fig. 3 theile ich die eine Hälfte eines Querschnitts aus der Halsregion eines 12 cm. langen Hundeembryos mit. Das Bild ähnelt in mancher Hinsicht dem neulich von v. Lenhossék vom 14 cm. langen menschlichen Embryo gelieferten (Fortschritte d. Medicin, 1892, Taf. 1). Man sieht u. A. den vorderen und den hinteren Ependymkeil, von denen der letzte schon weit ventralwärts vorgedrungen ist, während gleichzeitig die dorsale Spalte des Centralkanales sich obliterirt zeigt. Die ganze übrige Fläche des Schnittes ist theils von echten, d. h. vom Centralkanal bis zur Oberfläche reichenden Ependymzellen, theils von anderen Zellen durchsponnen, deren Zellenkörper in verschiedener Entfernung vom Centralkanal liegen, den peripheren Fortsatz aber in gleicher Weise wie die eigentlichen Ependymzellen nach der Oberfläche hin senden, wo sie diesen ähnlich endigen. In der antero-lateralen Region theilen sich diese Fortsätze gewöhnlich, wenn sie in die Längsstränge eintreten, oder auch kurz vorher; die Aeste biegen sich dabei oft, der Oberfläche parallel, eine Strecke um, ehe sie wieder radiär nach aussen hin ziehen; auf diese Weise entsteht hier an der Grenze des Strangmantels ein der Oberfläche tangential verlaufender Streifen, der aber nur die anterolaterale Region einnimmt; nach innen von diesem Streifen liegen ausserdem eigenthümlich gestaltete Zellen, deren längliche Körper in der Regel mit ihrer Längsaxe der Markoberfläche parallel gelagert sind; von den beiden Enden dieser Zellenkörper entspringt je ein Fortsatz, der sich bogenförmig nach aussen hin umbiegt und, gewöhnlich noch weiter dichotomisch getheilt, durch den Strangmantel nach der Oberfläche zieht, um dort knopfförmig zu endigen. Die Zellenkörper aller dieser Zellen sind gewöhnlich mit kurzen und feinen Aestchen versehen, und dies ist auch mit den Fortsätsen der Fall, weshalb sie oft ein gezacktes oder gar moosiges Aussehen erhalten. Wenn man nun alle diese Zellen vergleicht, so findet man, dass sie einander sehr ähnlich sind. Sie stellen offenbar verschiedene Modificationen einer und derselben Zellengattung dar, die verschiedene Stufen eines Entwicklungsverlaufes repräsentiren, eventuell auf denselben bleiben. Wenn man den Namen *Ependymzellen*, wie es gewöhnlich geschieht, für die Zellen beibehält, deren kernführender Körper am Centralkanal liegt und die den peripheren Fortsatz bis zur Oberfläche senden, so lässt sich mit Recht, wie Cajal, von Lenhossék, von Kölliker u. A. es thun, der Name *Neuroglia* für alle übrigen Stützzellen anwenden; nur ist dabei hervorzuheben, dass die beiden Namen keine principiell verschiedenen Zellengattungen, sondern nur Modificationen derselben Stützelemente bezeichnen. Gerade in diesem Stadium der Entwicklung sind die Uebergangsformen zwischen beiden Zellengattungen so zahlreich vorhanden, dass man bei genauerer Betrachtung kaum länger einen Zweifel über ihre Zusammengehörigkeit hegen kann. Unter

den radiären Neurogliazellen giebt es hier und da Exemplare, deren Zellenkörper beinahe bis zum Centralkanal reichen und den Ependymzellen frappant ähnlich sind; und unter den äusseren Neurogliazellen, deren Zellenkörper an der inneren Grenze der Strangregion liegen, giebt es viele, die einen kürzeren oder längeren Fortsatz radiär nach innen hin schicken; in der Strangregion selbst giebt es noch keine kernführenden Körper der Neurogliazellen. Wie v. A. von Lenhossék hervorgehoben hat, biegen die neben der vorderen Fissur des Markes befindlichen Neurogliazellen ihre Fortsätze nach ihr um; gerade diese Zellen sind am meisten verästelt.

In der hinteren Region des Querschnitts herrscht nun, wie von Lenhossék genau beschrieben hat, ein abweichendes Verhalten. Es sind hier keine eigentlichen Ependymzellen vorhanden, sondern nur radiäre Neurogliazellen, die ihre ovalen oder spindelförmigen, mit feinen moosähnlichen Fortsätzen versehenen Zellenkörper senkrecht oder etwas schief gegen die sagittale Mittellinie, das »Septum posticum autorum», kehren, im Allgemeinen aber in einiger, etwas verschieden grosser Entfernung davon belegen sind; nach aussen hin schicken sie ihren peripheren Fortsatz, der nach hinten hin etwas bogenförmig umbiegt und fast immer unverzweigt die Oberfläche erreicht und dort knopfförmig endigt; die hintersten machen sogar eine S-förmige Umbiegung, wie es von Lenhossék beim Menschen so eingehend und genau dargestellt hat. Die Erklärung, die dieser Forscher von der Entstehung jener Neuroglia-Anordnung in der hinteren Region des Markes gegeben hat, ist höchst plausibel: in Folge der Obliteration der hinteren Spalte des Centralkanales verschwinden die früheren eigentlichen Ependymzellen, und von ihnen restiren nunmehr nur radiäre Neurogliazellen, deren gegen das »Septum» senkrechte Anordnung indessen noch auf ihre Entstehung hinweist. Ferner hat von Lenhossék nachgewiesen, dass das sog. Septum nicht pialer Natur ist, sondern der Marksubstanz selbst angehört; es besteht aus Ependymzellen, dem hinteren Ependymkeil, dessen Zellen bei der Verlöthung der dorsalen Spalte des Centralkanales ventral vorrücken und zu dem Schluss und der Obliteration der Spalte wesentlich beitragen. Diese Erklärung ist offenbar zutreffend und lässt sich bei den Säugethier- und Menschenembryonen leicht bestätigen.

Was die Zellen des »Septum» betrifft, so gilt auch für die Säuger die von von Lenhossék beim Menschenembryo gegebene Darstellung; am Dorsalende des Centralkanales drängen sich die Zellenenden von beiden Seiten eng zusammen; dahinter biegen sie sich beiderseits etwas nach aussen, und nur die in der Mittellinie befindlichen verlaufen gerade in sagittaler Richtung; dann biegen sich die seitlichen Zellen wieder nach innen, um im dorsalen Theil des Septum als schmales, gedrungenes Bündel bis zu der seichten dorsalen Fissur zu verlaufen.

Im vorderen Ependymkeil sind die Ependymzellen kurz, aber verhältnismässig dick und in der Regel unverzweigt, jedoch mit etwas gezackten Rändern versehen und in den Seitentheilen tonnenartig gebogen, wogegen sie in der Mitte gerade verlaufen; am Grunde der ventralen Fissur endigen sie mit gewöhnlich sehr kleinen Knöpfen.

Stützelemente anderer Art als die soeben beschriebenen habe ich in diesem Stadium des Hunderückenmarkes nicht gefunden.

In der Fig. 4 der Taf. X habe ich noch eine Abbildung des grössten Theils eines Querschnitts von der Lendenregion desselben 14 cm. langen Hundembryos mitgetheilt. Es sind im Ganzen so übereinstimmende Verhältnisse vorhanden, dass ich auf eine nähere Beschreibung derselben verzichten kann. Noch weiter unten in der Lendenregion war die Anordnung und Beschaffenheit der Stützelemente viel primitiver (Fig. 2 der Tafel X), indem hier nur Ependymzellen vorhanden waren; keine Neurogliaelemente waren entstanden; die dorsale Spalte war nicht verlöthet.

Es ist nicht meine Absicht, diesmal die weitere Entwicklung der Stützelemente des Rückenmarks der Säuger zu verfolgen, da die Darstellung durch die vielen Einzelheiten gar zu sehr in die Breite gezogen werden und noch eine Menge von Figuren dazu nöthig sein würde. Das Angeführte reicht hin, um in Uebereinstimmung mit von Lenhossék's Darstellung das Wesentlichste hervorzuheben. Ich komme bei der Beschreibung der Verhältnisse beim Menschen auf dieses Thema zurück.

Was das Ependym und die Neuroglia des *Gehirns* betrifft, so habe ich theils schon früher (Verhandl. d. Biol. Vereins in Stockholm, Bd 3, 1891) diese Frage gelegentlich berührt und die Hauptformen der beiden Zellenarten aus der Grosshirnrinde des jungen Hundes und auch die Bergmann'schen Faserzellen der Kleinhirnrinde des Menschen etc. abgebildet, theils den zur obigen Abhandlung gehörigen Figuren aus der Grosshirnrinde noch einige typische Ependym- und Neurogliazellen beigefügt. Diese Zellenformen sind auch schon längst von anderen Forschern,

wie GOLGI, MARTINOTTI, CAJAL, VON KÖLLIKER, VAN GEHUCHTEN u. A., beschrieben und abgebildet worden. Eine eingehende Schilderung ihres ganzen Entwicklungsmodus wäre aber noch immer von besonderem Interesse; es ist jedoch diesmal nicht meine Absicht, diese Frage zu behandeln.

6. Beim Menschen.

Taf. XI—XIII.

Von jüngeren menschlichen Embryonen habe ich nur einmal so frisches Material bekommen, dass es sich nach der Golgi'schen Methode färben liess. Dasselbe war von einem 3 cm. langen Embryo, in dessen Rückenmark sich hier und da sowohl das Ependym und die Neuroglia, wie auch Ganglienzellen und Collaterale schön färbten. Die Verschliessung der dorsalen Spalte des Centralkanales war in den verschiedenen Regionen des Markes verschieden weit vorgeschritten. In der Fig. 1 der Taf. XI habe ich ein Stadium abgebildet, wo diese Verschliessung schon ziemlich weit avancirt ist; in der Fig. 2 derselben Tafel ist ein anderes Stadium wiedergegeben, wo die Spalte noch weiter dorsalwärts reicht und das sog. Septum kürzer ist. In der Fig. 1 sieht man über das ganze Feld Ependymzellen gestreut, die senkrecht gegen die Höhle des Centralkanales, resp. auch gegen die verlöthete dorsale Spalte, stehen und von dort aus ihre äusseren Fortsätze radiär gegen die Peripherie hin senden; in den ventralen Theilen biegen sie sich, wie VON LENHOSSÉK betont hat und wie es auch bei anderen Säugern (Hund etc.) der Fall ist, zugleich etwas ventralwärts, in den dorsalen Theilen etwas dorsalwärts; nur in der Grenzgegend zwischen der dorsalen und der ventralen Partie ziehen die Ependymfasern gerade lateralwärts. Die Zellenkörper der Ependymzellen sind in Allgemeinen schmal und erweitern sich nur dort, wo der Kern liegt; vom inneren freien Ende sieht man hier und da ein haarartiges, oft etwas gekrümmtes Anhängsel in den Centralkanal hineinschiessen; ich bemerke dies, weil VON LENHOSSÉK solche Haare erst in viel späteren Stadien gefunden hat. Der periphere Fortsatz verläuft geschlängelt nach aussen hin; er ist noch glatt oder nur wenig knotig und gezackt. In der anterolateralen Region theilt er sich an der Grenze der Stränge dichotomisch, und die beiden Aeste können sich noch weiter theilen; sie ziehen in mehr oder weniger grossem Winkel von einander fort und biegen sich dann nach aussen hin, um an der Oberfläche knopfförmig oder mit konischer Verdickung zu endigen. Die Fortsätze der zu beiden Seiten des ventralen Ependymkeils befindlichen Ependymzellen biegen sich, wie v. LENHOSSÉK hervorgehoben hat, medialwärts gegen die ventrale Fissur um und theilen sich dabei reichlich. Die beiderseits von der dorsalen Spalte des Centralkanals befindlichen Ependymzellen reichen mit ihrem centralen Ende bei offener Spalte (Fig. 2 der Taf. XI) bis zur Höhle hinan; nach dem Verschluss derselben (Fig. 1 ders. Taf.) sieht man noch einige bis an sie hinan reichen; hier sind aber schon mehrere nunmehr als Neurogliazellen zu bezeichnende Zellen mit spindelförmigem moosigem Zellkörper und nach der Peripherie strebendem, in der Regel ungetheiltem Fortsatz zu beiden Seiten des Septum posticum vorhanden, die es nicht erreichen; die Fortsätze dieser Zellen werden von den Collateralen des Hinterstrangs (Fig. 1 der Taf. XI) gekreuzt; Neurogliazellen derselben Art sind auch in der vorderen Hälfte des Rückenmarkes vorhanden; Neurogliazellen von anderer Beschaffenheit habe ich in diesem Stadium nicht angetroffen; in der Strangregion (»der weissen Substanz») waren keine Zellenkörper nachweisbar.

Die beiden Ependymkeile verhalten sich so, wie oben beim Hunde und früher von v. LENHOSSÉK näher beschrieben worden ist. Hinsichtlich des dorsalen Keils ist ein Vergleich der Fig. 1 und 2 d. Taf. XI lehrreich, indem die letztere Figur ein früheres, die Fig. 1 ein späteres Stadium darstellt. Der ventrale Keil zeigt ein typisches Verhalten; nur will ich bemerken, dass in ihm einzelne Fasern eine Theilung darbieten.

Von den folgenden Stadien der Entwicklung habe ich das Rückenmark eines 15 cm. langen menschlichen Embryos gewählt. Dies Stadium (Fig. 3 der Taf. XI) zeigt eine auffallend höhere Entwicklung als das von v. LENHOSSÉK bei dem 14 cm. langen Embryo dargestellte. Vor Allem ist hervorzuheben, dass das ganze laterale Ependym in der Ausbildung zurückgeblieben und die Neuroglia schon weit entwickelt ist. Die Zellenkörper der Ependymzellen haben zwar ihre Beschaffenheit ziemlich bewahrt, ihre peripheren Fortsätze aber zeigen ein verändertes Aussehen, indem sie bald grob gekörnt, bald äusserst fein sind und nicht mehr regelmässig radiär, sondern buchtig verlaufen und bald verschwinden; die neben dem ventralen Ependymkeil befindlichen Zellen lassen sich, obwohl

von sehr feinem Caliber, noch hier und da bis zur ventralen Fissur verfolgen; die mehr lateral belegenen ziehen in verschiedenen Richtungen und biegen sich oft sogar wieder medialwärts um, wonach sie dem Anscheine nach verschwinden; nie konnte ich nunmehr diese Fasern bis zur Strangsubstanz verfolgen, sondern sie endigten in der Regel unweit des Centralkanales. Die beiden Ependymkeile zeigten die v. A. von von Lenhossék beschriebene Beschaffenheit; in dem ventralen Keile biegen sich die peripheren, dicken und fein gezahnten Fortsätze oft in mehreren grossen Bögen um, wie die bei stärkerer Vergrösserung gezeichnete Fig. 4 der Taf. XI deutlicher zeigt. Die von von Lenhossék geschilderte mediale Umbiegung der centralen Enden der Ependymzellen des dorsalen Keiles ist in der Fig. 3 scharf ausgeprägt, wie auch die danach folgende Verbreiterung und dann wieder die pinselförmige Verengerung des hinteren Theils des Keiles aus dieser Figur hervorgeht.

Lange radiäre Neurogliazellen sind vorn und v. A. hinten reichlich vorhanden und zeigen die oben beim Hundeembryo und früher von von Lenhossék beim Menschenembryo geschilderte Beschaffenheit und Anordnung. Ihr Verlauf vor, hinter und in dem Hinterhorn, resp. in der Substantia gelatinosa Rolandi ist von dem genannten Forscher so genau beschrieben worden, dass ich nur auf seine Darstellung hinzuweisen brauche.

Was die übrigen Neurogliazellen betrifft, so findet man, wie oben angedeutet wurde, in der antero-lateralen Region schon eine weit avancirte Entwicklung derselben, und zwar sowohl in der »grauen», wie in der »weissen» Substanz. Die neben der ventralen Fissur befindlichen Zellen besitzen schon ihre spätere Beschaffenheit; die Zellenkörper sind unregelmässig und zeigen nur ausnahmsweise einen centralen Fortsatz, wogegen in der Regel zahlreiche periphere Fortsätze bis zur Oberfläche ziehen, wo sie verdickt endigen. In der übrigen antero-lateralen Region sind in der »weissen» Substanz (Strangsubstanz) ebenfalls zahlreiche Neurogliazellen verschiedener Gestalt vorhanden; die meisten sind einfach oder wenig verzweigt; in der Fig. 3 der Tafel XI habe ich eine Reihe der gewöhnlicheren Formen wiedergegeben, weshalb ich auf diese Figur hinweise. Die in der grauen Substanz befindlichen Neurogliazellen sind im Allgemeinen ziemlich klein und mit moosigen und zottigen Aestchen versehen; hier und da trifft man den noch vorhandenen peripheren Fortsatz, zuweilen auch einen kurzen centralen.

Von den folgenden Stadien wählte ich das Rückenmark des *26 Cm. langen* menschlichen Embryo aus, weil ich bei diesem eine schöne Färbung der Neurogliaelemente bekam. Auf den Taf. XII und XIII habe ich einige Abbildungen von Partien des Quer- und Längsschnitts dieses Rückenmarkes mitgetheilt. Ich finde aber nicht, dass es nöthig ist, eine detaillirte Beschreibung der Verhältnisse zu geben, da ich das, was v. Lenhossék darüber gesagt hat, in allem Wesentlichen bestätigen kann. Indessen will ich einige Punkte hervorheben, in welchen meine Ansichten etwas von den seinigen abweichen. Was zuerst die Ependymzellen betrifft, so finde ich, wie schon oben beim 15 cm. langen Embryo bemerkt wurde, dass dieselben zum grössten Theile verkümmert sind. Ventral- und dorsalwärts sind sie zwar noch ziemlich erhalten; eine ventrale Keilfigur ist aber nicht mehr nachweisbar; sternförmige Deiters'sche oder Spinnenzellen sind hier aufgetreten; Nervenfasern und Ganglienzellenfortsätze sind vielfach durch diese vordere Commissur hervorgedrungen, und die Ependymzellen sind gewissermassen aus einander geschoben; sie lassen sich aber noch grösstentheils bis zur Fissur verfolgen; die meisten gelangen dahin auf verwickelten Bahnen; ihre central hervorragenden Haarausläufer sind oft schön nachweisbar.

Die Ependymzellen des dorsalen Ependymkeiles biegen ihre peripheren Fortsätze, bevor dieselben in das schmale Septum eintreten und in ihm fast geraden Weges oder nur wenig geschlängelt nach hinten ziehen, oft stark lateralwärts um.

Die lateralen Ependymzellen färben sich recht ungerne; ihre peripheren Fortsätze sind in der Regel fein, varicös und in ihrem Verlaufe unregelmässig; dieselben biegen sich oft um und entziehen sich dem Blicke. von Lenhossék hegt die Ansicht, dass sie nicht gerne verschwinden können; er meint nämlich, dass das, was auf so vorgeschrittener Stufe da ist, auch später erhalten bleibt; ich kann zwar nicht beweisen, das die Ependymfasern »atrophiren» und ganz verschwinden, doch werden sie nicht nur spärlicher, sondern auch im Verlaufe unregelmässiger, indem sie sogar nicht selten medialwärts umbiegen, bevor sie sich dem Auge entziehen. Darin stimmen aber alle Forscher überein, dass diese Fasern keine bedeutende Rolle mehr spielen, sondern als embryonale Rudimente zu betrachten sind.

Die *Neurogliazellen* sind dagegen in diesem Stadium zu einem hohen Grade von Entwicklung gelangt. In der Umgebung des Centralkanales (Taf. XII, Fig. 1) sind die Deiters'schen oder Spinnenzellen in schöner Ausbildung vorhanden; ihre Fortsätze sind auf weite Strecken zu verfolgen. In der *grauen* Substanz (Taf. XIII, Fig.

1 *gr*) trifft man eine grosse Menge von Zellen zerstreut an, die sehr verschiedene Formen darbieten; im Allgemeinen haben sie viele feine Fortsätze, die den Zellen ein moosiges Aussehen geben; oft sind die Ausläufer varicös, sogar mit dicken, plumpen Knoten versehen, oder auch tragen sie zahlreiche Seitenästchen. Hier und da sieht man, dass die Fortsätze sich an den naheliegenden Blutgefässscheiden mit konischen Enden ansetzen, wie es Golgi längst beschrieben hat (Taf. XII, Fig. 1 bei *bl, bl,* wo Spinnenzellen sich in dieser Weise verhalten). Den nach der Oberfläche ziehenden peripheren Fortsatz vermisse ich in diesem Stadium bei den Neurogliazellen der grauen Substanz oft. In der *Substantia gelatinosa Rolandi* sind die Zellen, wie von Lenhossék sie beschreibt, im Allgemeinen langgestreckt und moosig; es kommen aber auch viele kurze und dicke Zellen vor, die eine wechselnde Gestalt darbieten (Taf. XIII, Fig. 3).

Die Neurogliazellen *der weissen* Substanz (Taf. XIII, Fig. 1 *w* und Fig. 2) bieten ebenfalls, wie von Lenhossék geschildert hat, verschiedene Formen dar. Es ist fast unmöglich, von ihnen eine allgemeine Beschreibung zu liefern, und ich muss daher auf die angeführten Figuren verweisen. Im Ganzen ist der verschieden gestaltete Zellenkörper klein, unregelmässig eckig und mit dichten, feinen Ausläufern versehen, die in allen Richtungen ausstrahlen; vor Allem ziehen aber von ihm ein oder mehrere dickere und mehr gestreckt verlaufende, sogar steife Fortsätze centralwärts, und ebenso ein, zwei oder mehr dickere steife Fortsätze nach der Peripherie hin, entweder ungetheilt, oder, in der Regel, verzweigt die Oberfläche erreichend und dort mit je einer kleineren oder grösseren konischen Verdickung endigend. Die Zellenkörper liegen in verschiedener Tiefe unter der Oberfläche; viele findet man dicht unter derselben, oder sogar an ihr selbst; hierdurch entstehen, wie aus den Figuren hervorgeht, eigenthümlich gestaltete, aber sehr wechselnde Formen. Im Ganzen lassen sich zwar die von von Lenhossék beschriebenen 4 Typen nachweisen; es kommt aber eine grosse Anzahl vor, die sich nicht ohne Zwang unter diese Typen einreihen lassen. Der Wechsel ist nämlich auffallend gross, wie schon ein Blick auf die angeführten Figuren lehrt und auch von Lenhossék selbst betont hat.

Aus der obigen Darstellung geht indessen schon zur Genüge hervor, dass ich, v. A. in Uebereinstimmung mit Golgi, Cajal und von Lenhossék, die Stützelemente des centralen Nervensystems der Wirbelthiere von Anfang an für eine besondere Gattung von Zellenelementen halte. In der That hat ja auch His auf Grund seiner tiefgehenden und ausgedehnten Untersuchungen schon vor Jahren bewiesen, dass die Stützelemente aus einer bestimmten Art embryonaler Zellen, den Spongioblasten, sich entwickeln.

Die sich zuerst entwickelnden Stützzellen, die Ependymzellen, spielen indessen, besonders bei den höheren Wirbelthieren, nur vorübergehend eine grössere Rolle; sie treten nachher zurück oder bleiben auf rudimentärem Standpunkt stehen. Unterdessen haben sich, offenbar aus derselben Art von Keimzellen, die Neurogliazellen entwickelt, die zuerst die eigenthümliche lange radiäre, den Ependymzellen ähnelnde Gestalt annehmen, bald aber sich in die verschiedenen Formen der eigentlichen Neurogliazellen umwandeln, die in den einzelnen Regionen der grauen und weissen Substanz einen differenten, obwohl mannigfaltig wechselnden Typus erhalten.

Tafel V.

Ependym und Neuroglia

im Rückenmarke von Petromyzon.

Fig. 1. Querschnitt vom *hinteren* Theil des Rückenmarks eines 4 Cm. langen *Petromyzon.* Rechts vom Centralkanal sind sieben *Neurogliazellen* und links von ihm eine solche Zelle in gefärbtem Zustande dargestellt. Ringsum den Centralkanal sind fünf Ependymzellen vorhanden; — *gz,* drei Ganglienzellen mit ihren verästelten Fortsätzen; — *s* sensible Nervenwurzel; — *h,* Contour der äusseren Scheide.

Fig. 2. Querschnitt vom hinteren Theil des Rückenmarks des erwachsenen *Petromyzon.* Rechts sind vier *Neurogliazellen,* am Centralkanal drei *Ependymzellen* dargestellt; — *gz,* zwei Ganglienzellen, eine kleine und eine grosse, mit ihren verästelten Fortsätzen in der linken Markhälfte wiedergegeben.

Fig. 3. Querschnitt vom hinteren Theil des Rückenmarks eines 40 Cm. langen *Petromyzon.* Zwei *Neurogliazellen* und fünf Ganglienzellen sind hier dargestellt.

Fig. 4. Querschnitt vom vorderen, nicht weit hinter dem Kopfe belegenen Theil des Rückenmarks eines 14 Cm. langen *Ammocoetes.* Acht *Neurogliazellen* und eine *Ependymzelle (e)* sind wiedergegeben. Eine Anzahl Querschnitte Müller'scher Fasern von verschiedenem Caliber sind durch Contouren *(m)* angegeben; die Fortsätze der Neurogliazellen schmiegen sich zwischen dieselben.

Sämmtliche Figuren sind nach Golgi'schen Präparaten bei der Anwendung von Vér. Obj. 6 und Ocul. 3 (eingeschob. Tubus) gezeichnet.

Fig. 1.

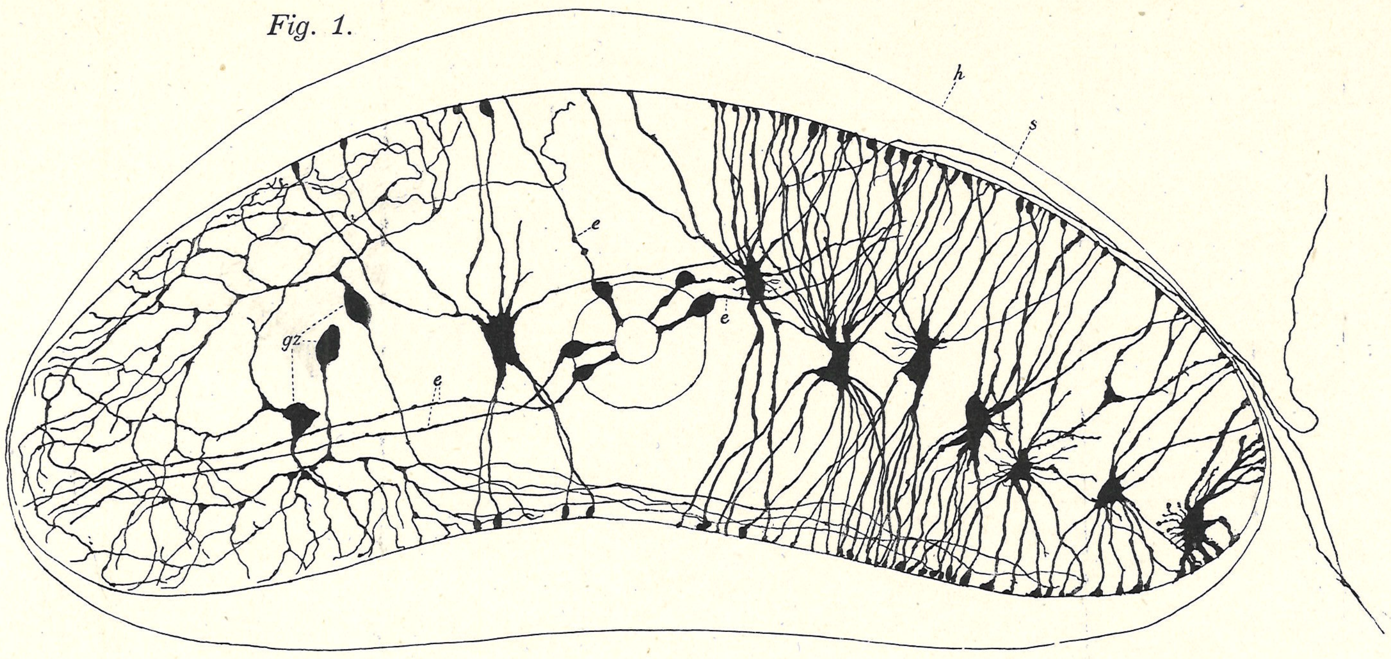

Fig. 2.

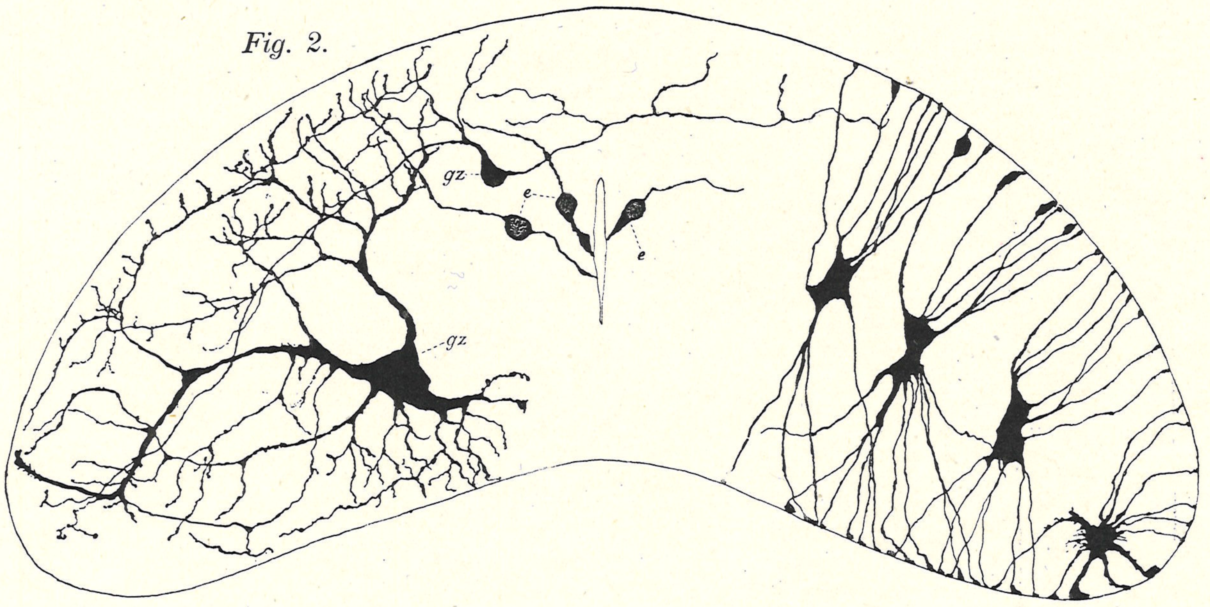

Fig. 3.

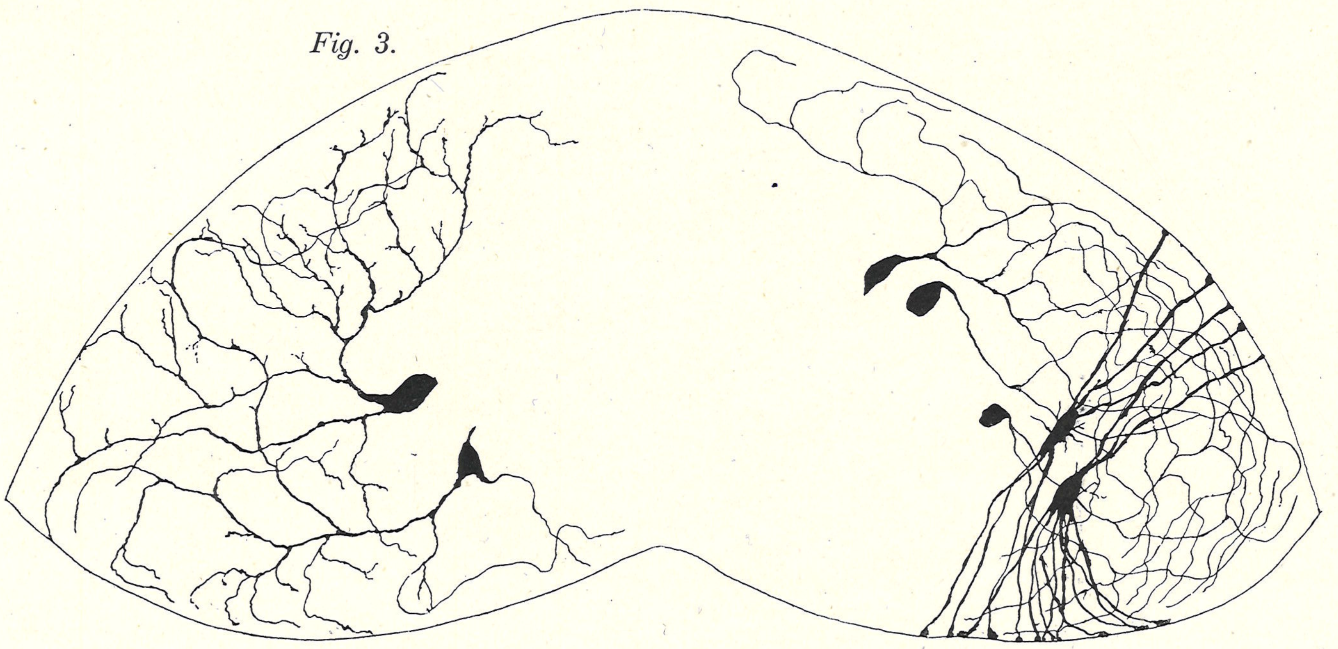

Fig. 4.

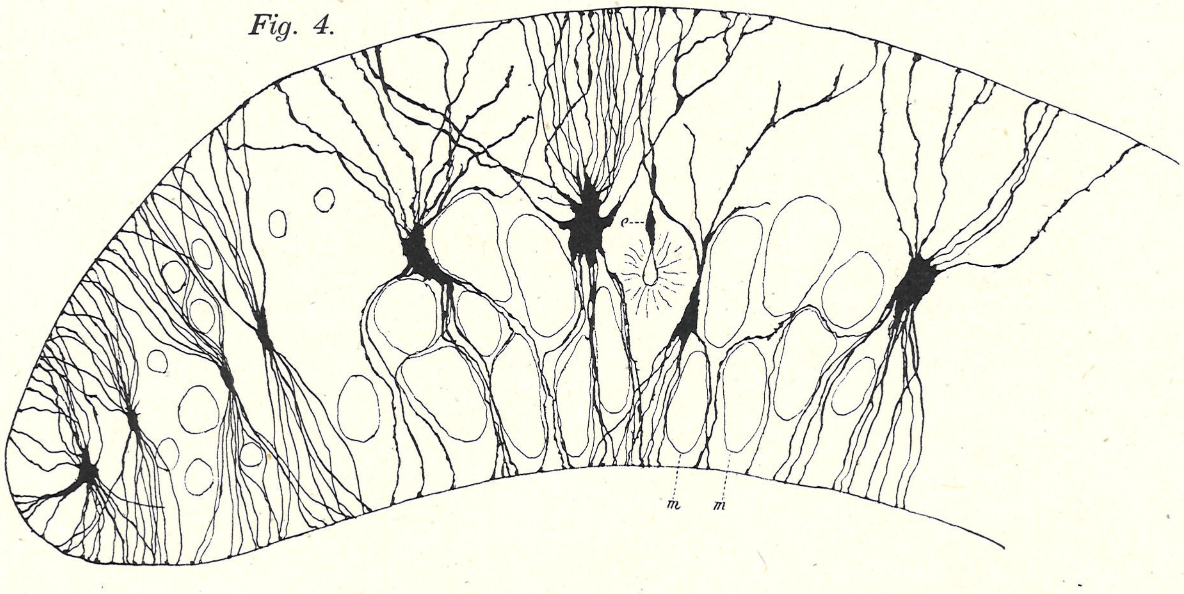

Gez. v. Gustaf Retzius. Typogr. Central-Tryckeriet. Fototypi v. J. Cederquist.

Tafel VI.

Ependym und Neuroglia

der Med. spinalis und oblongata des Petromyzon.

Fig. 1. Querschnitt des vordersten Endes vom *Rückenmark* des *Petromyzon.* Verschiedene Formen von *Neurogliazellen;* — *e,* eine *Ependymzelle;* — *m* Querschnitte der Müller'schen Fasern.

Fig. 2. Querschnitt der *Medulla oblongata* des *Petromyzon. Ependymzellen* und *Neurogliazellen.*

Beide Figuren sind nach Golgi'schen Präparaten bei Vér. Obj. 6 und Ocul. 1 (eingeschob. Tubus) gezeichnet.

Fig. 1.

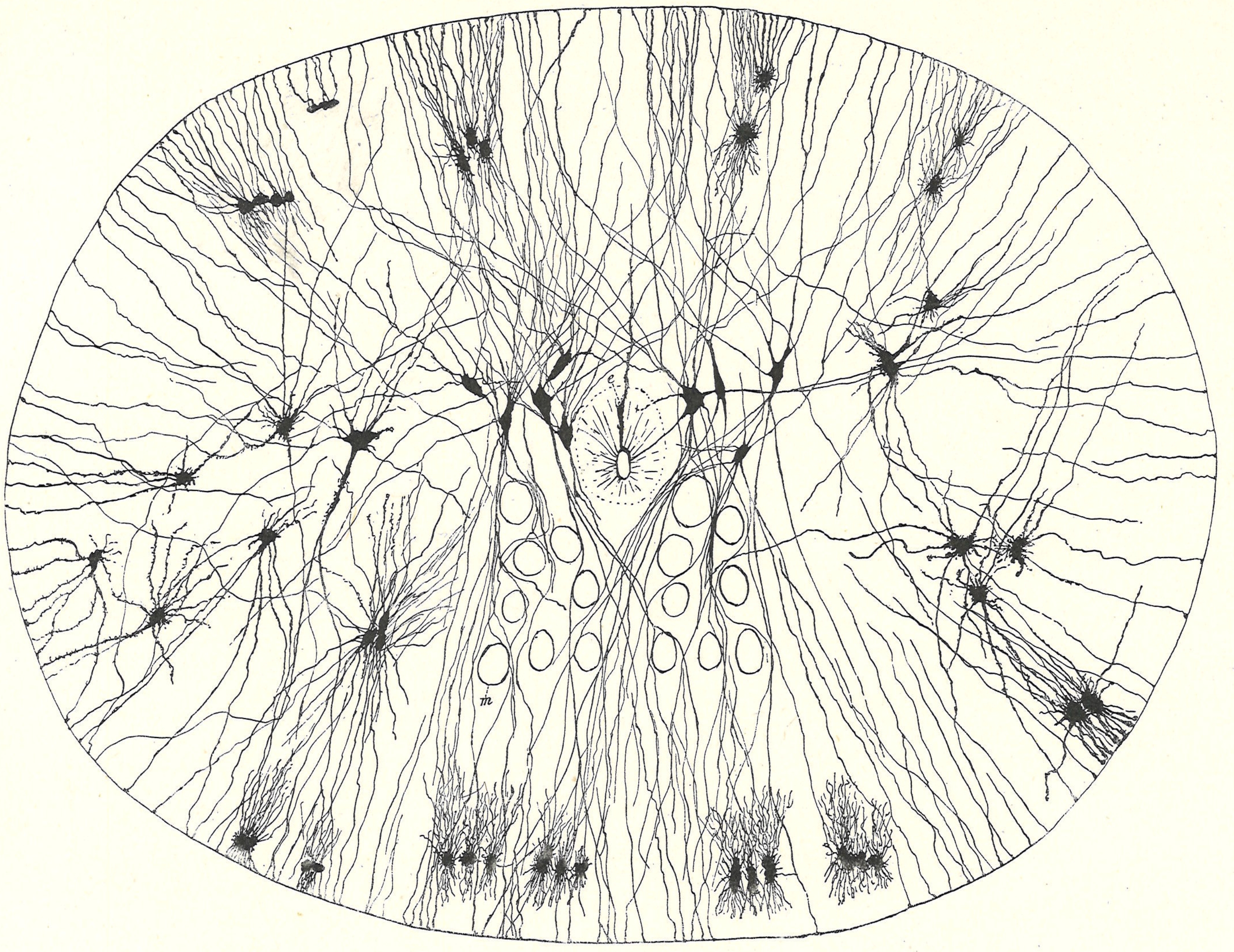

Fig. 2.

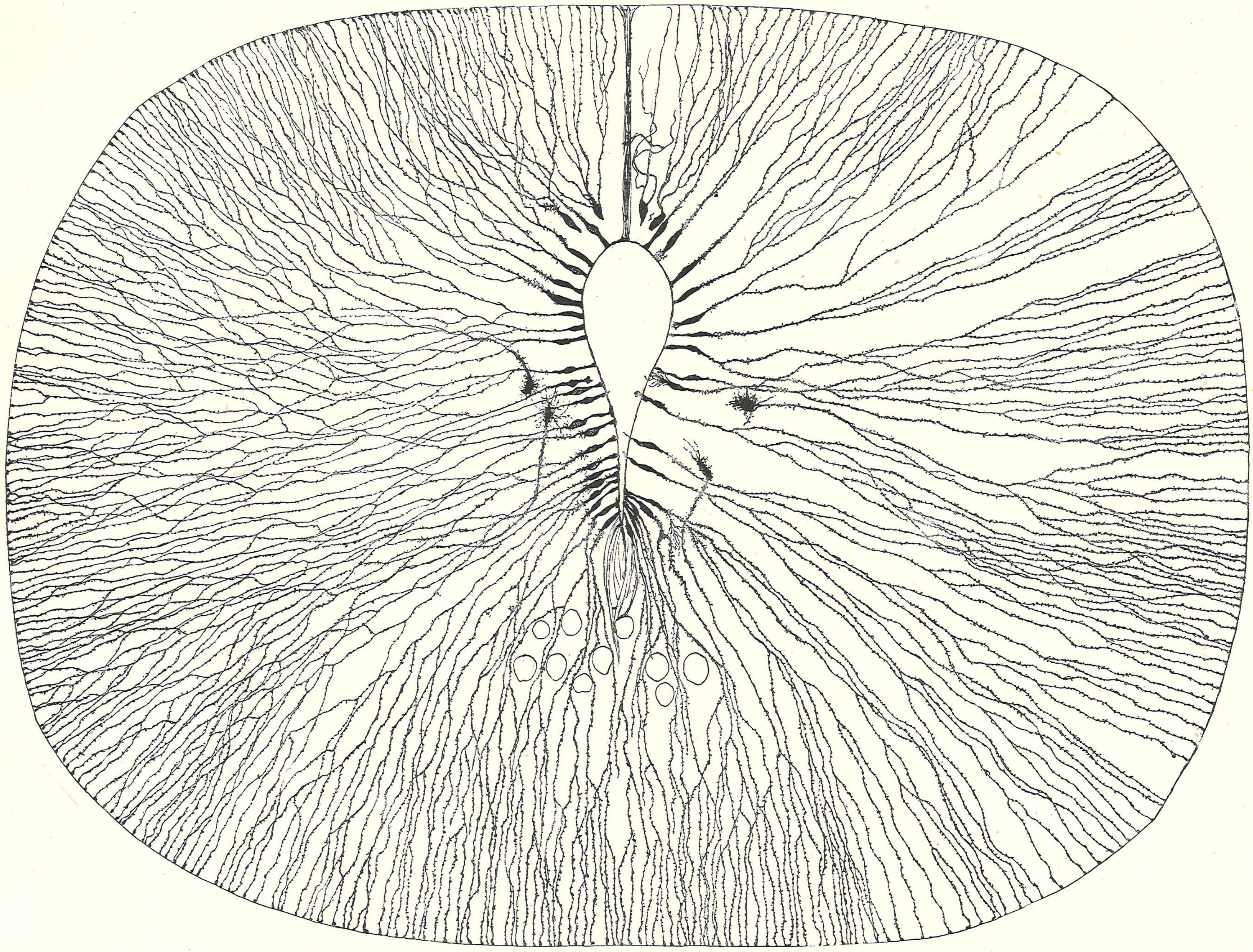

Gez. v. Gustaf Retzius. Typogr. Central-Tryckeriet. Fototypi v. J. Cederquist.

Tafel VII.

Ependym und Neuroglia

im Rückenmark und Gehirn von Petromyzon.

Fig. 1. Antero-laterale Partie eines Querschnitts vom vordersten Rückenmarksende eines jungen *Petromyzon*, mit dem von »Spinnenzellen» umgebenen Centralkanal und seitlichen dendritischen Neurogliazellen.

Fig. 2 und 3. Partien von der Ventrikelwand des Grosshirns eines 15 Cm. langen Petromyzon mit Ependymzellen.

Fig. 4. Querschnitt des Gehirns eines 16 Cm. langen Petromyzon; — *e*, Ependymzellen. Mehrere Ganglienzellen verschiedener Grösse sind auch wiedergegeben.

Die Figuren sind nach Golgi'schen Präparaten abgebildet. Fig. 1—3 sind bei Vér. Obj. 6 und Ocul. 3 (eingeschob. Tubus), Fig. 4 bei Vér. Obj. 6 und Ocul. 1 (eingeschob. Tubus) gezeichnet.

Fig. 1.

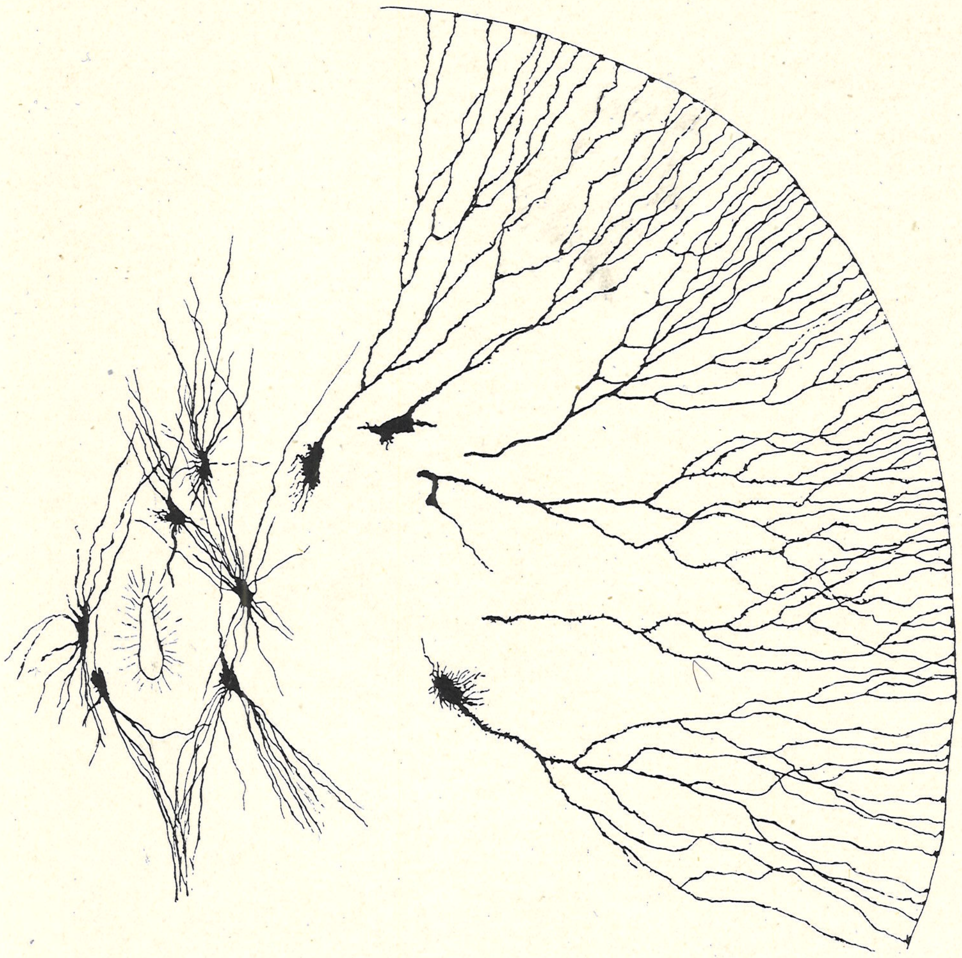

Fig. 2.

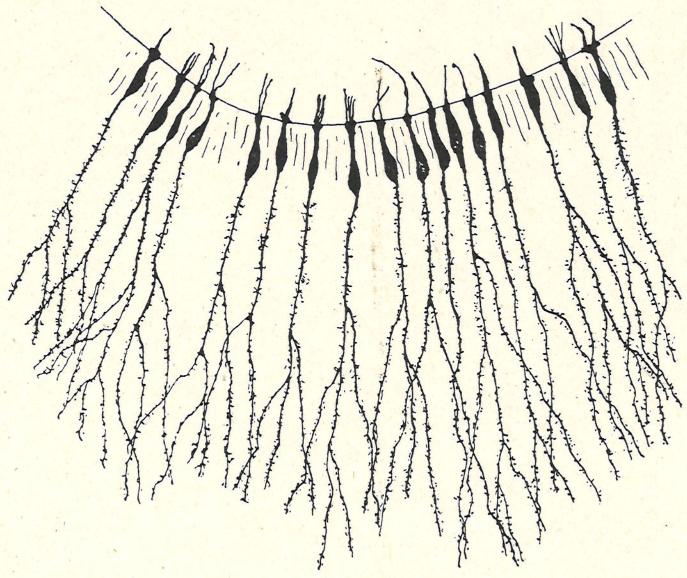

Fig. 3.

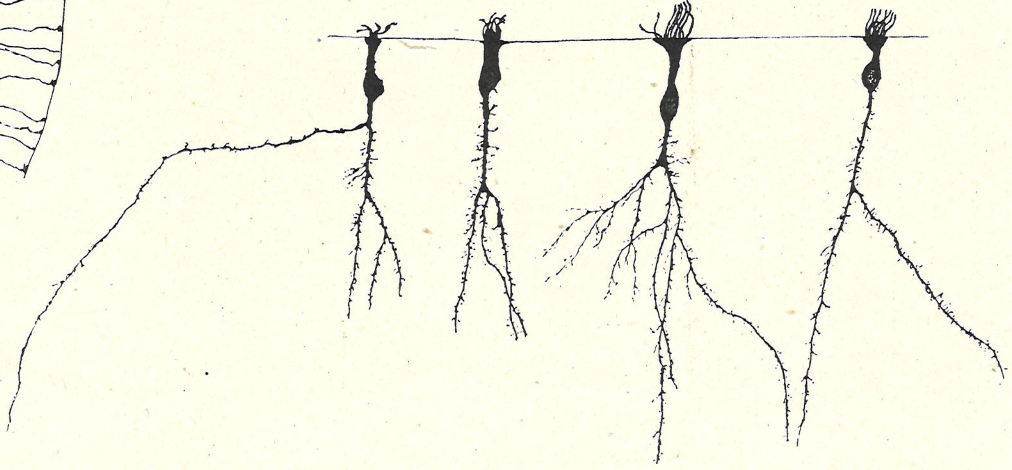

Fig. 4.

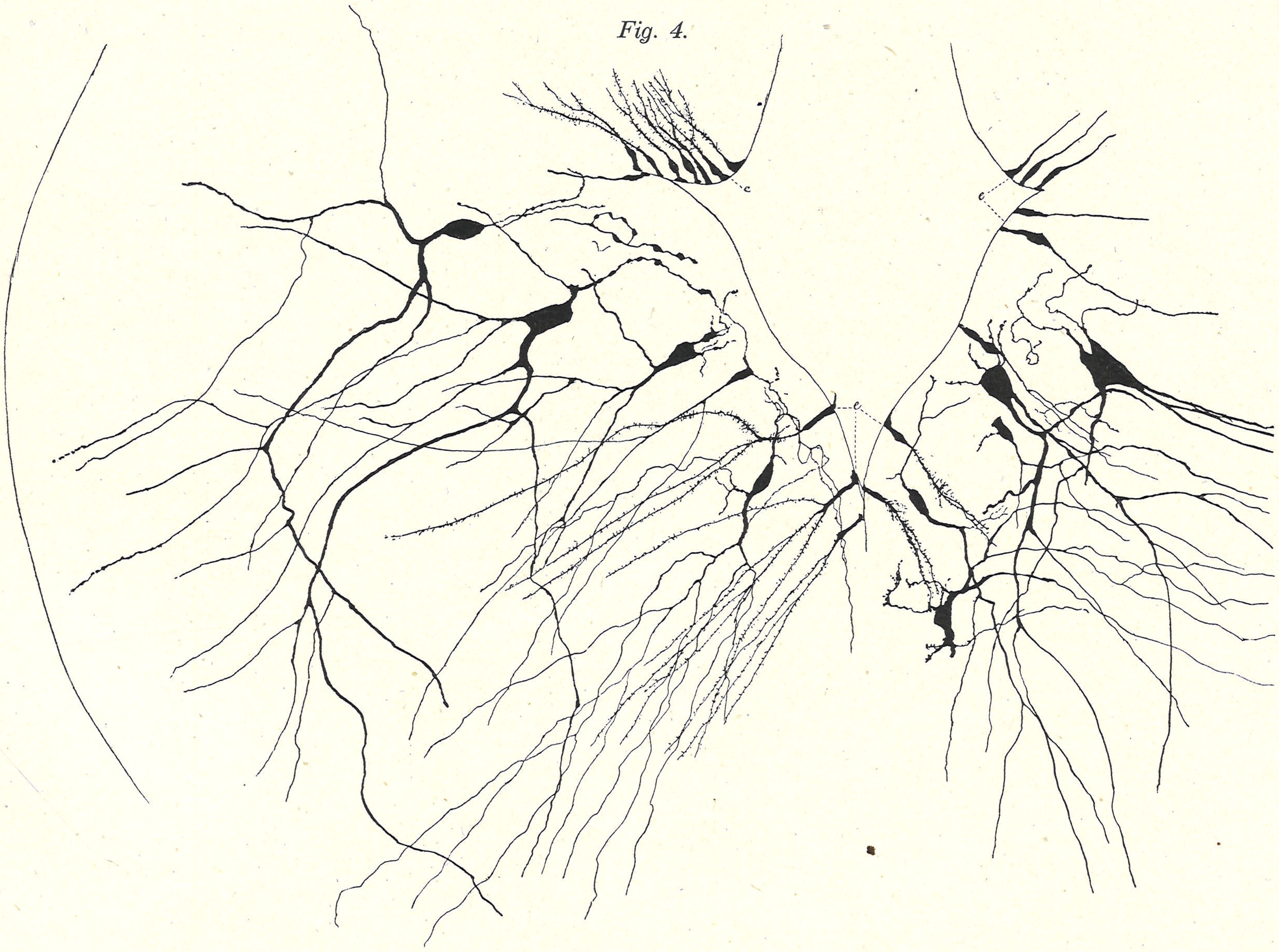

Gez. v. Gustaf Retzius. Typogr. Central-Tryckeriet. Fototypi v. J. Cederquist.

Tafel VIII.

Ependym und Neuroglia

der Knochenfische und Vögel.

Fig. 1—3. Querschnitte des Rückenmarks von jungen, 16 mm. langen *Lachsen.* Fig. 2 und 3 aus dem mittleren, Fig. 1 aus dem hinteren Theil des Markes. *Ependymzellen:* — *gz* Ganglienzellen.

Fig. 4 *a* **und 4** *b.* Die zwei lateralen Partien eines Querschnittes vom Grosshirn eines jungen *Lachses.* Ependym- und Neurogliazellen.

Fig. 5—8. Querschnitte des Rückenmarks und Gehirns von *Hühner*embryonen; — *Fig. 5.* Querschnitt des lumbalen Rückenmarks von einem 8-tägigen Hühnerembryo; Ependymzellen; — *Fig. 6.* Partie vom vorderen Ende des Rückenmarks eines 14 Tage alten Hühnerembryos; radiäre Neurogliazellen; — *Fig. 7.* Septum posticum in der Uebergangspartie vom Rückenmark in die Med. oblongata eines 8-tägigen Hühnerembryos; — *Fig. 8.* Partie eines Querschnitts vom Grosshirn eines 17-tägigen Hühnerembryos.

Sämmtliche Figuren sind nach Golgi'schen Präparaten wiedergegeben. Fig. 1—4 sind bei Vér. Obj. 6 und Ocul. 3 (eingeschob. Tubus), Fig. 5—8 bei Vér. Obj. 6 und Ocul. 1 (eingeschob. Tubus) gezeichnet.

Fig. 2.

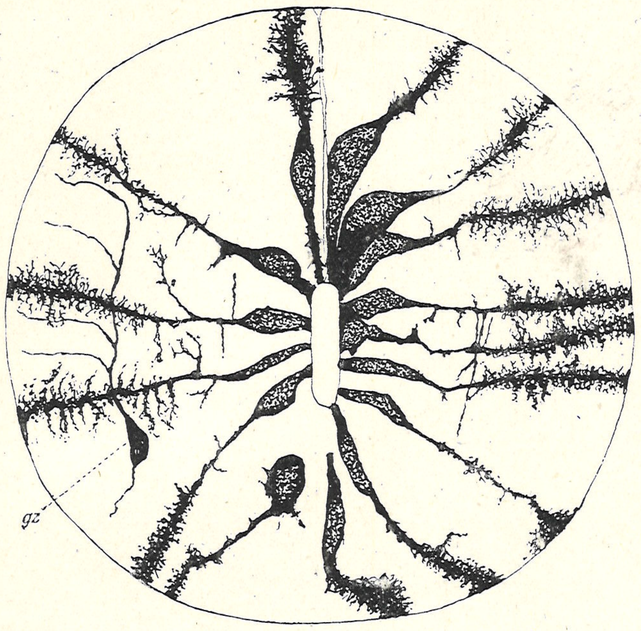

Fig. 3.

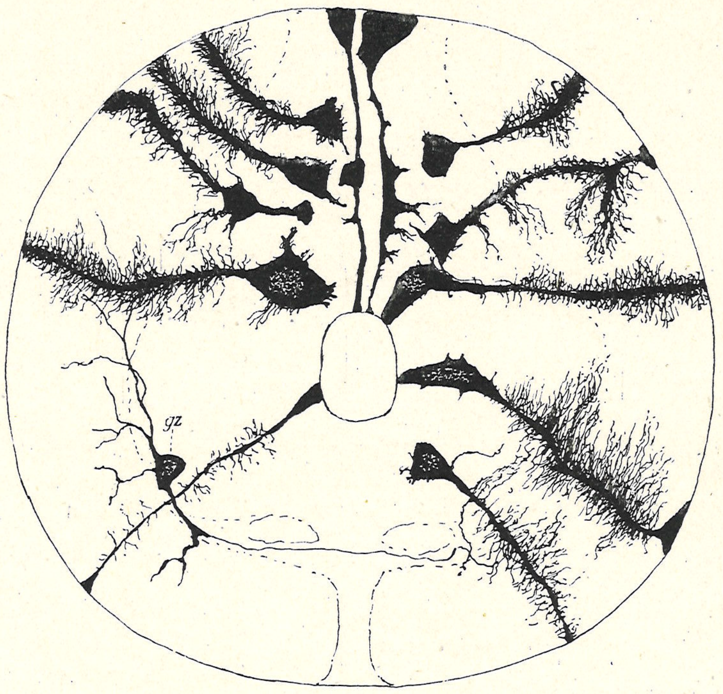

Fig. 1.

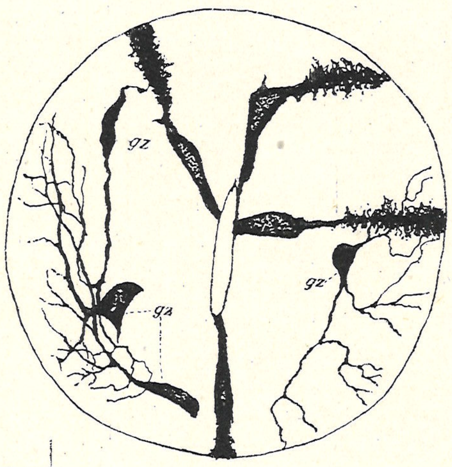

Fig. 4 a.

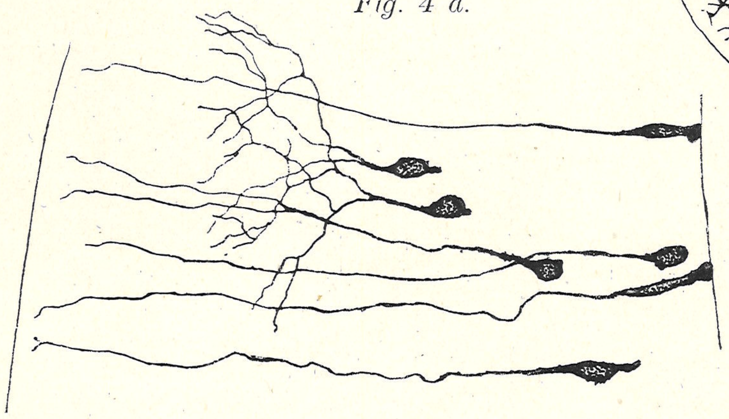

Fig. 4 b.

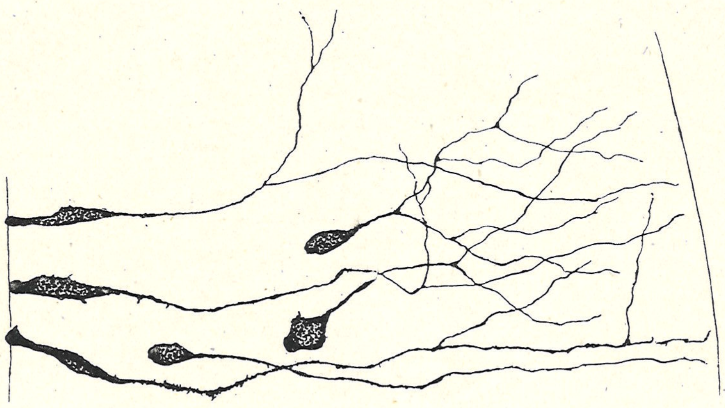

Fig. 7.

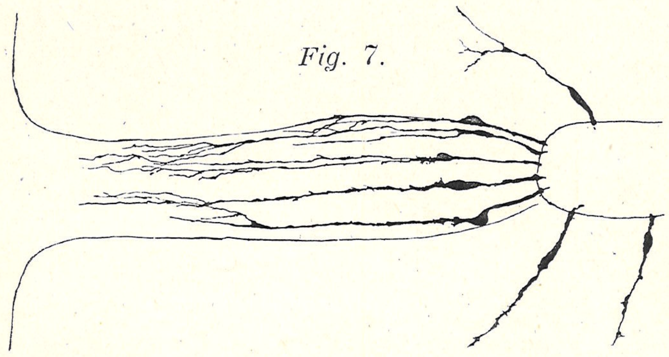

Fig. 8.

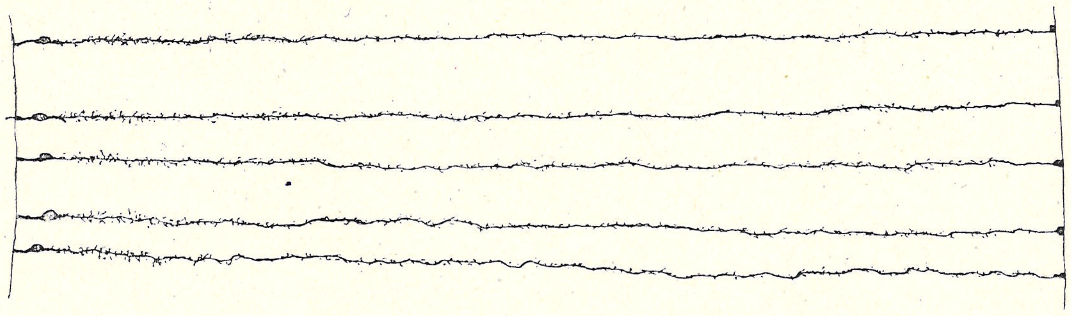

Fig. 5.

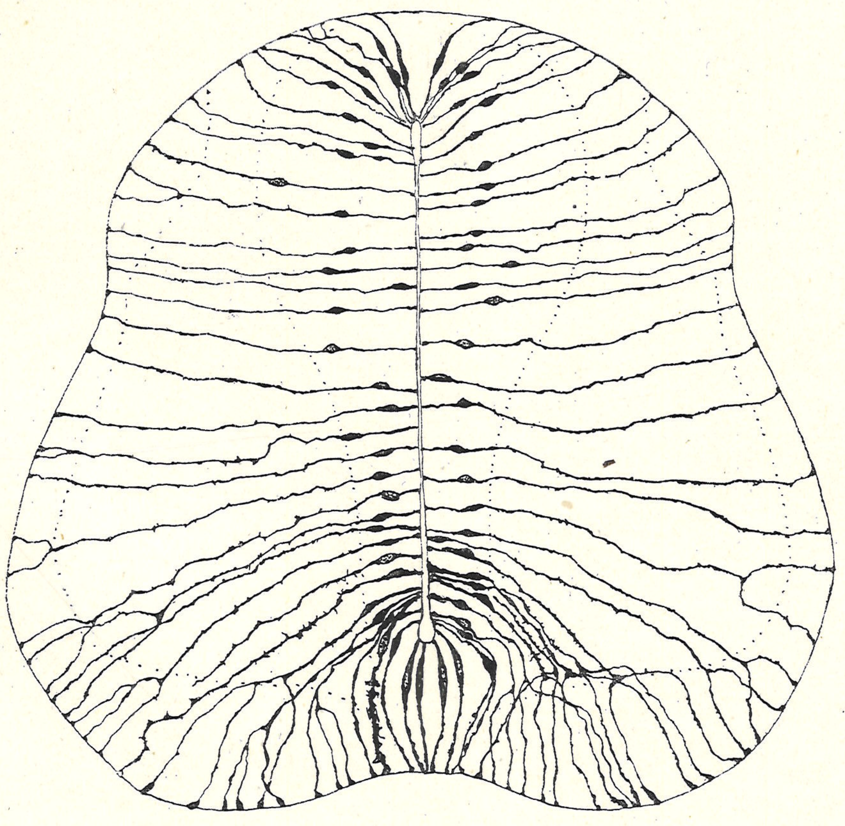

Fig. 6.

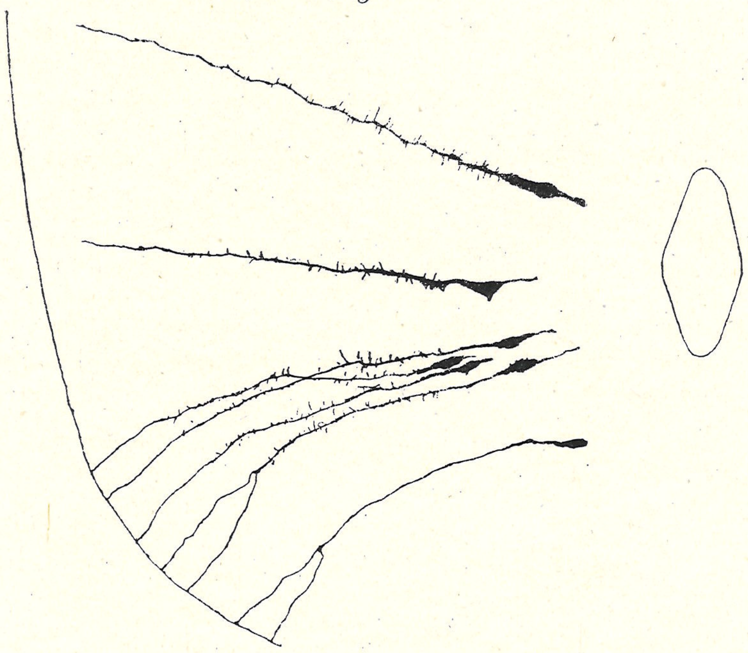

Gez. v. Gustaf Retzius. Typogr. Central-Tryckeriet. Fototypi v. J. Cederquist.

Tafel IX.

Ependym und Neuroglia

bei den Amphibien.

Fig. 1—3. Querschnitte vom Rückenmark und Gehirn der Larve von *Salamandra maculata;* — *Fig. 1.* Querschnitt des Rückenmarks, mit Ependym-, resp. Neurogliazellen; *gz* Ganglienzellen; — *Fig. 2.* Laterale Partie eines Querschnitts der Medulla oblongata; — *Fig. 3.* Partie eines Querschnitts vom Grosshirn; Ependymzellen.

Fig. 4—7. Querschnitte vom Rückenmark und Gehirn des jungen *Frosches* (R. temp.); — *Fig. 4.* Querschnitt des Rückenmarks; radiäre Neurogliazellen; — *Fig. 5.* Querschnitt der Medulla oblongata; Ependymzellen; — *Fig. 6.* Schnitt des Mittelhirns; *e,* Ependymzellen; — *Fig. 7.* Querschnitt des Grosshirns; *m,* Mittellinie, *gz,* Ganglienzellen; fünf Ependymzellen; *o* — Oberfläche des Gehirns.

Sämmtliche Figuren sind nach Golgi'schen Präparaten wiedergegeben.

Fig. 1—3, 4 und 7 sind bei Vér. Obj. 6 und Ocul. 1 (eingeschob. Tubus), Fig. 6 bei Vér. Obj. 6 und Ocul. 3, Fig. 5 bei Vér. Obj. 2 und Ocul. 3 (eingeschob. Tubus) gezeichnet.

Fig. 1.

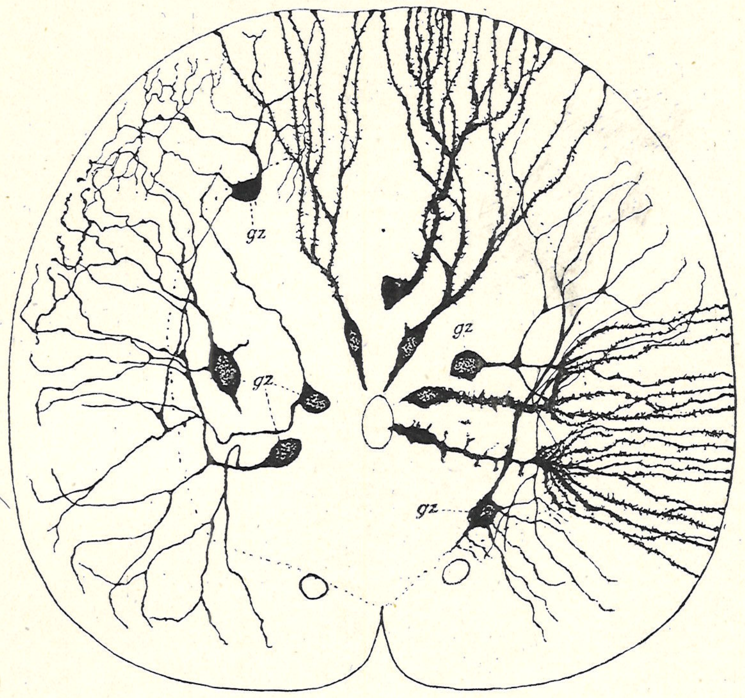

Fig. 2.

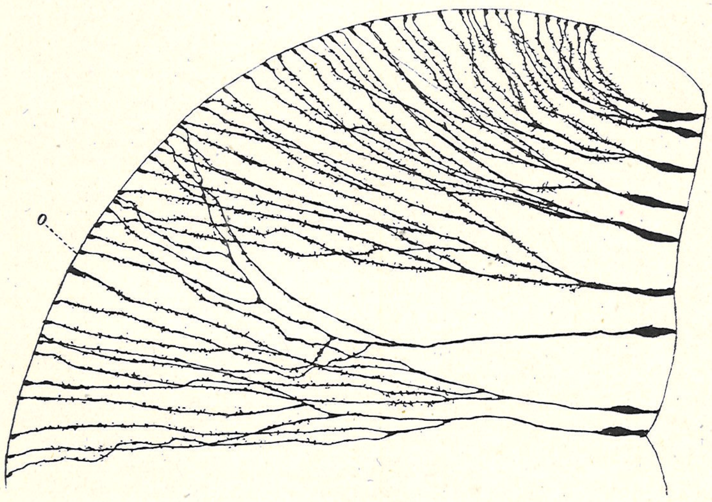

Fig. 3.

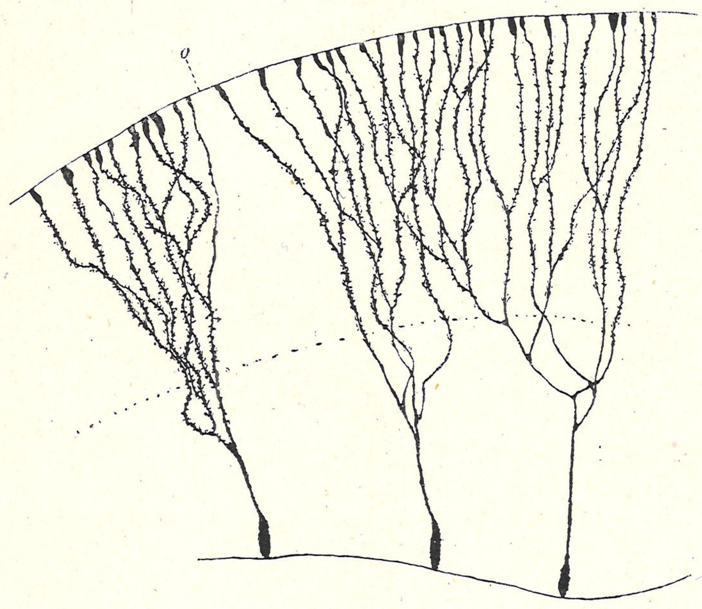

Fig. 7.

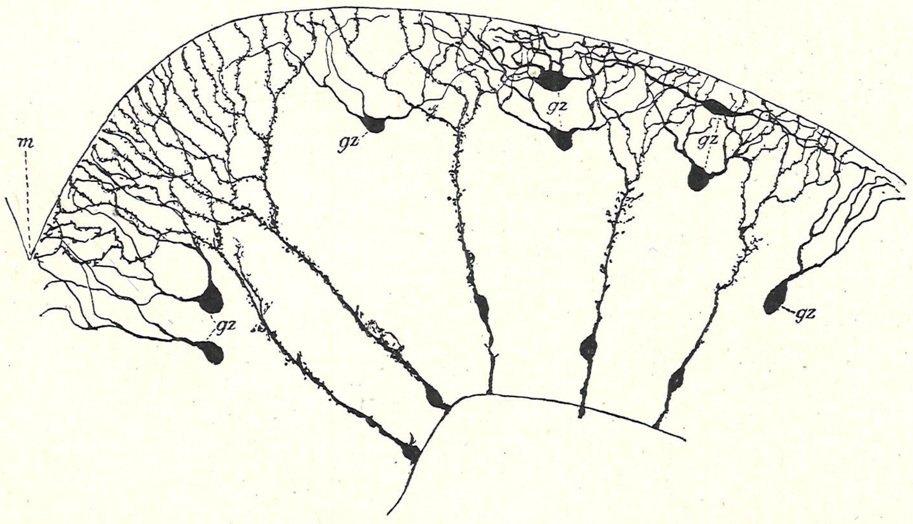

Fig. 6.

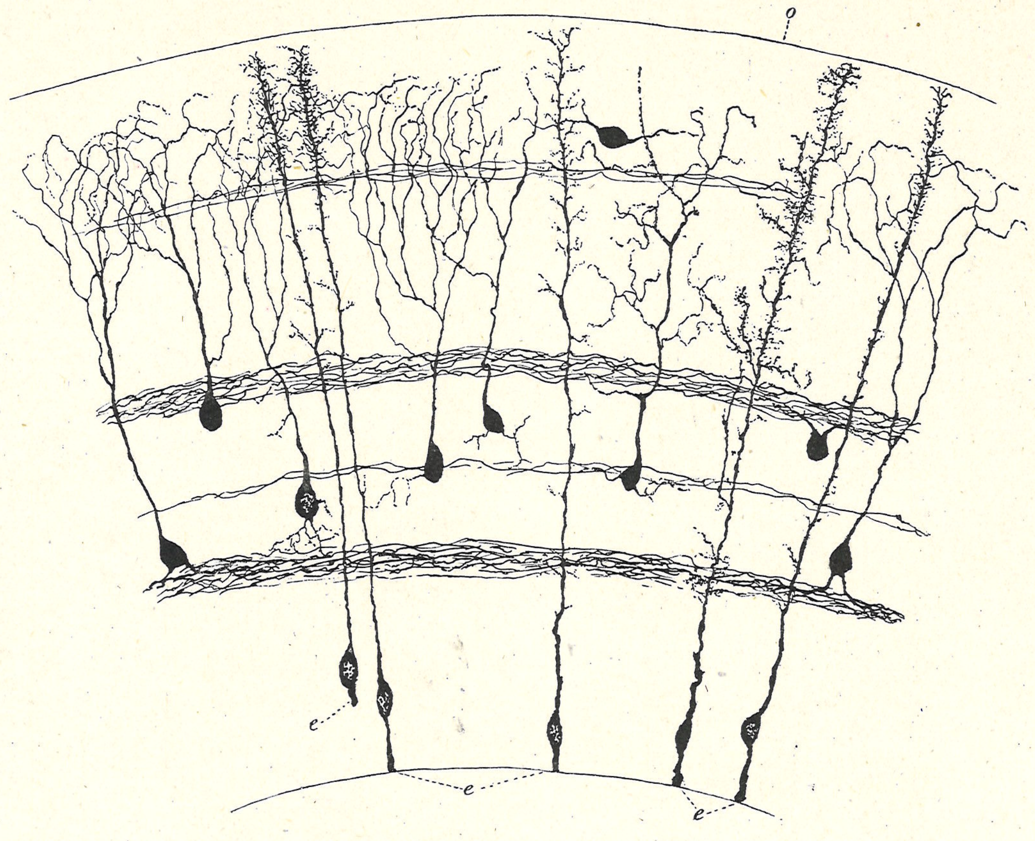

Fig. 4.

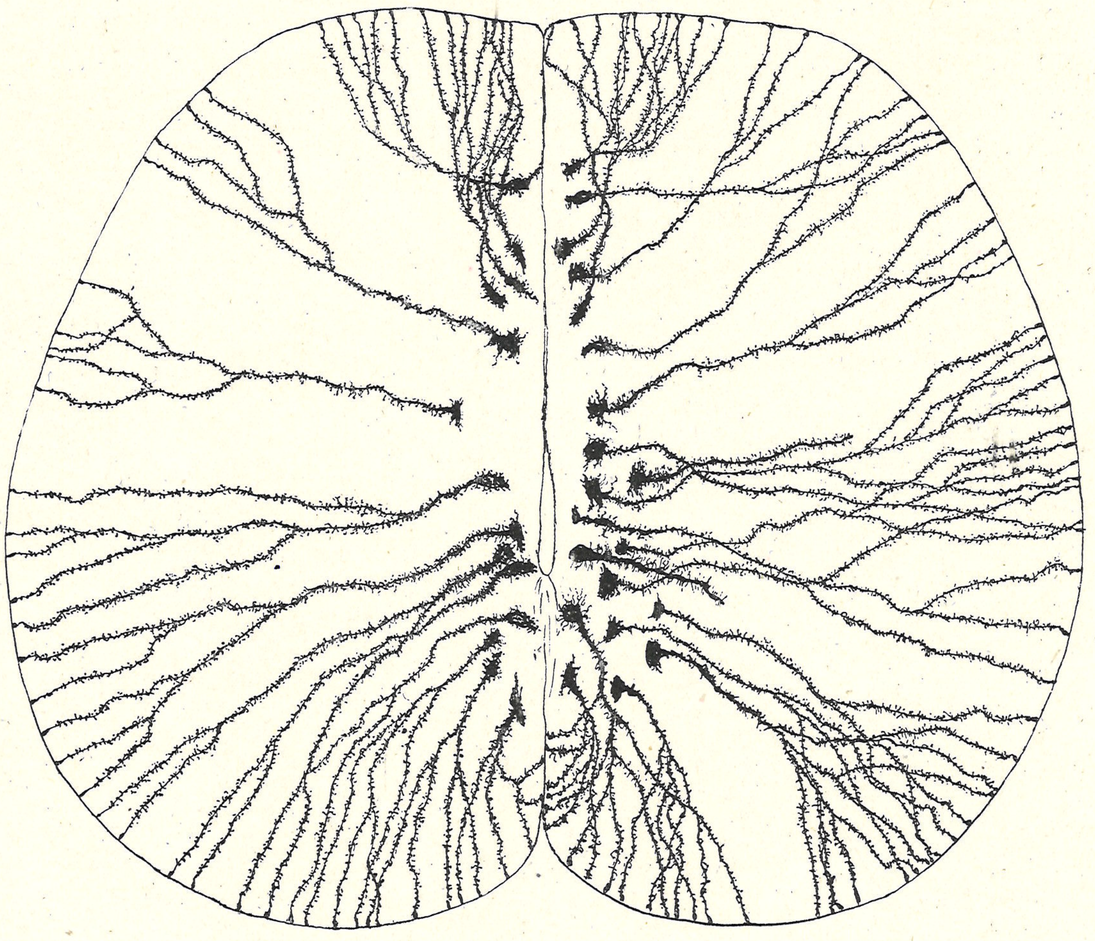

Fig. 5.

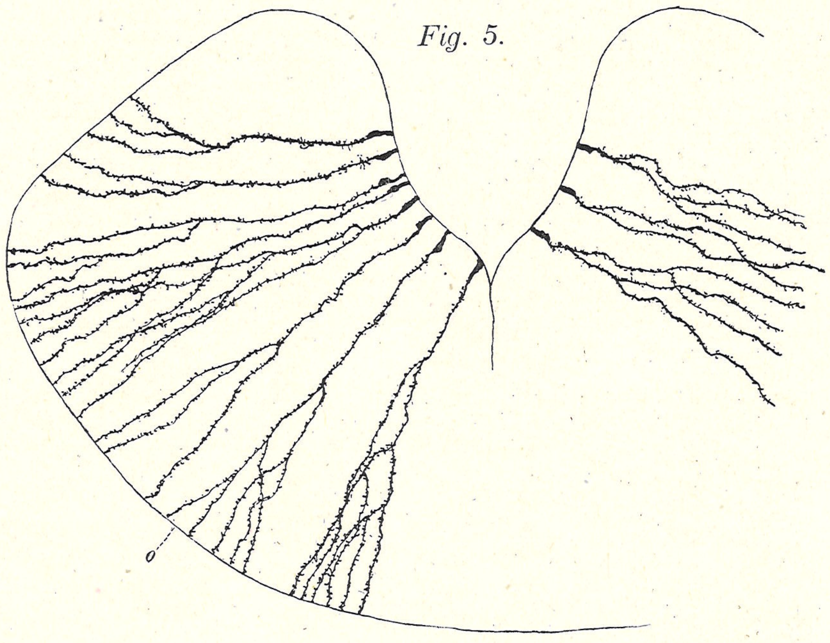

Gez. v. Gustaf Retzius. Typogr. Central-Tryckeriet. Fototypi v. J. Cederquist.

Tafel X.

Ependym und Neuroglia

bei den Säugethieren.

Fig. 1. Querschnitt vom Rückenmarke eines 3 Cm. langen *Katzen*embryos. Ependymzellen.

Fig. 2. Querschnitt vom Hinterende des Lendenmarks eines 12 Cm. langen *Hunde*embryos. Ependymzellen.

Fig. 3. Querschnitt vom Rückenmarke (Halsregion) eines 12 Cm. langen *Hunde*embryos. Ependym- und Neurogliazellen.

Fig. 4. Querschnitt vom Rückenmarke (Lendenregion) eines 12 Cm. langen *Hunde*embryos. Ependym- und Neurogliazellen.

Fig. 5. Partie eines Querschnitts vom Rückenmark (Lendenregion) eines 12 Cm. langen *Katzen*embryos. Anterolateralregion. Neurogliazellen der Strangsubstanz.

Sämmtliche Figuren sind nach Golgi'schen Präparaten wiedergegeben. Die Fig. 1 ist bei Vér. Obj. 2 und Ocul. 3 (eingeschob. Tubus), Fig. 2—4 bei Vér. Obj. 6 und Ocul. 1 (eingeschob. Tubus), Fig. 5 bei Vér. Obj. 6 und Ocul. 3 (eingeschob. Tubus) gezeichnet.

Fig. 2.

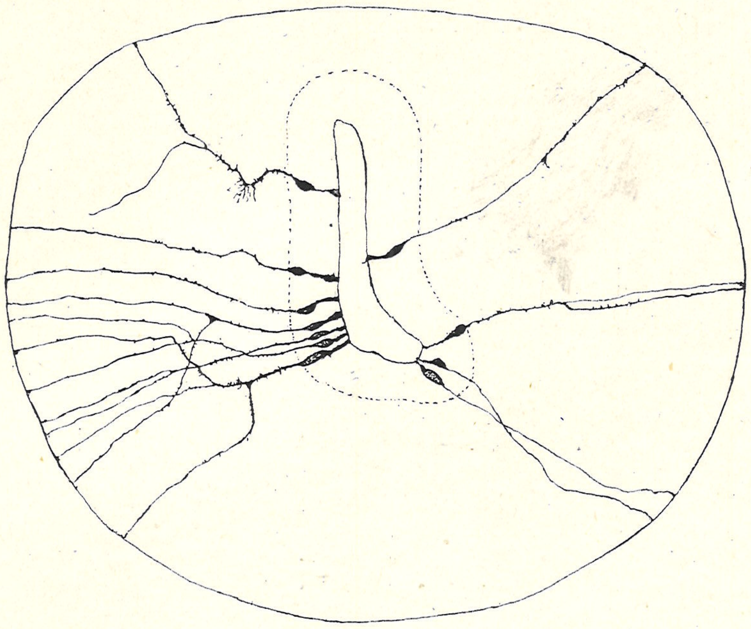

Fig. 3.

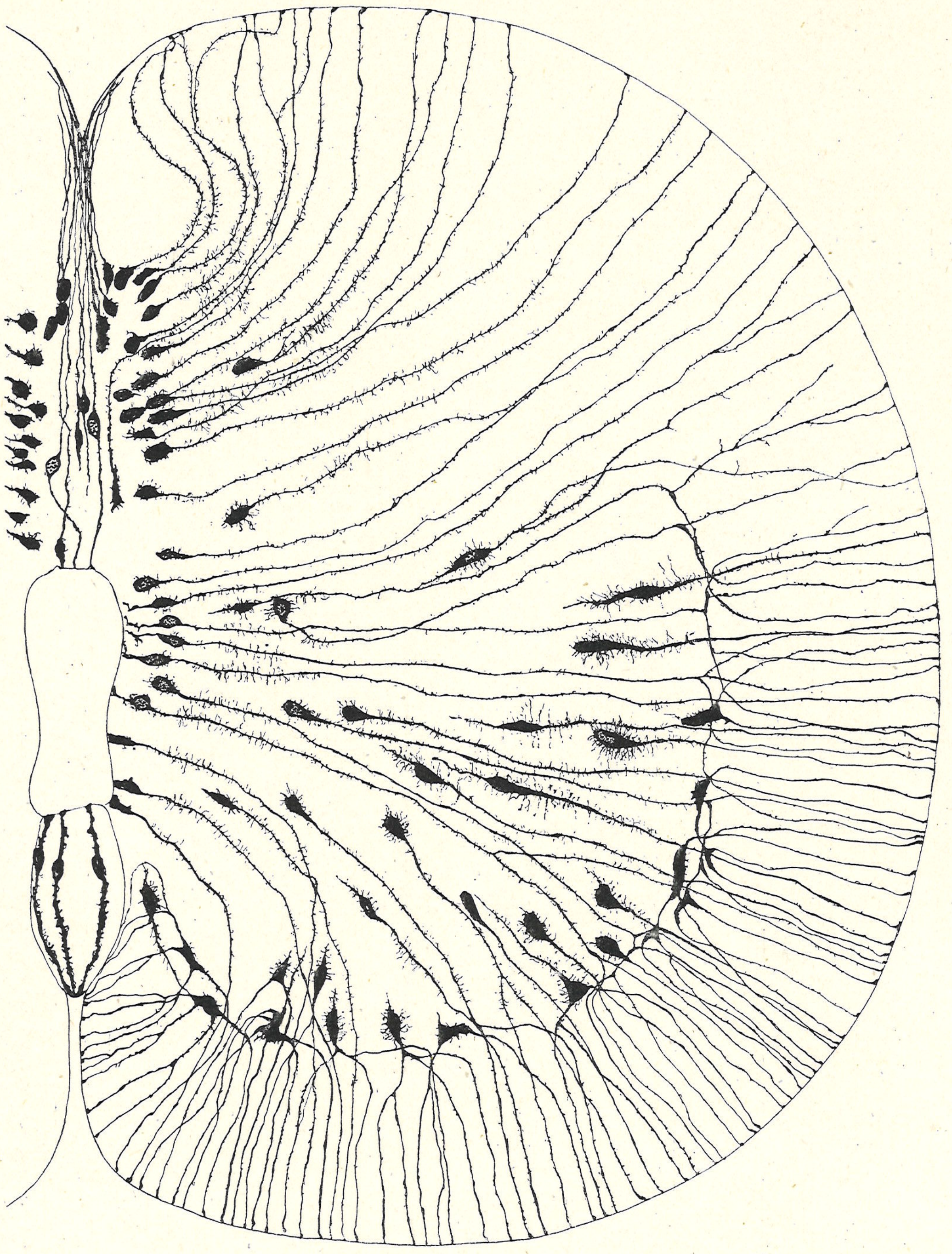

Fig. 1.

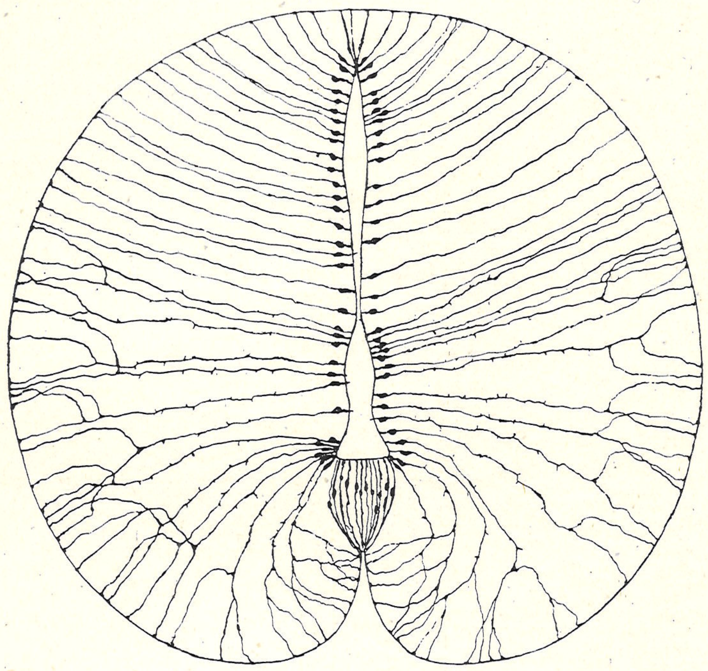

Fig. 4.

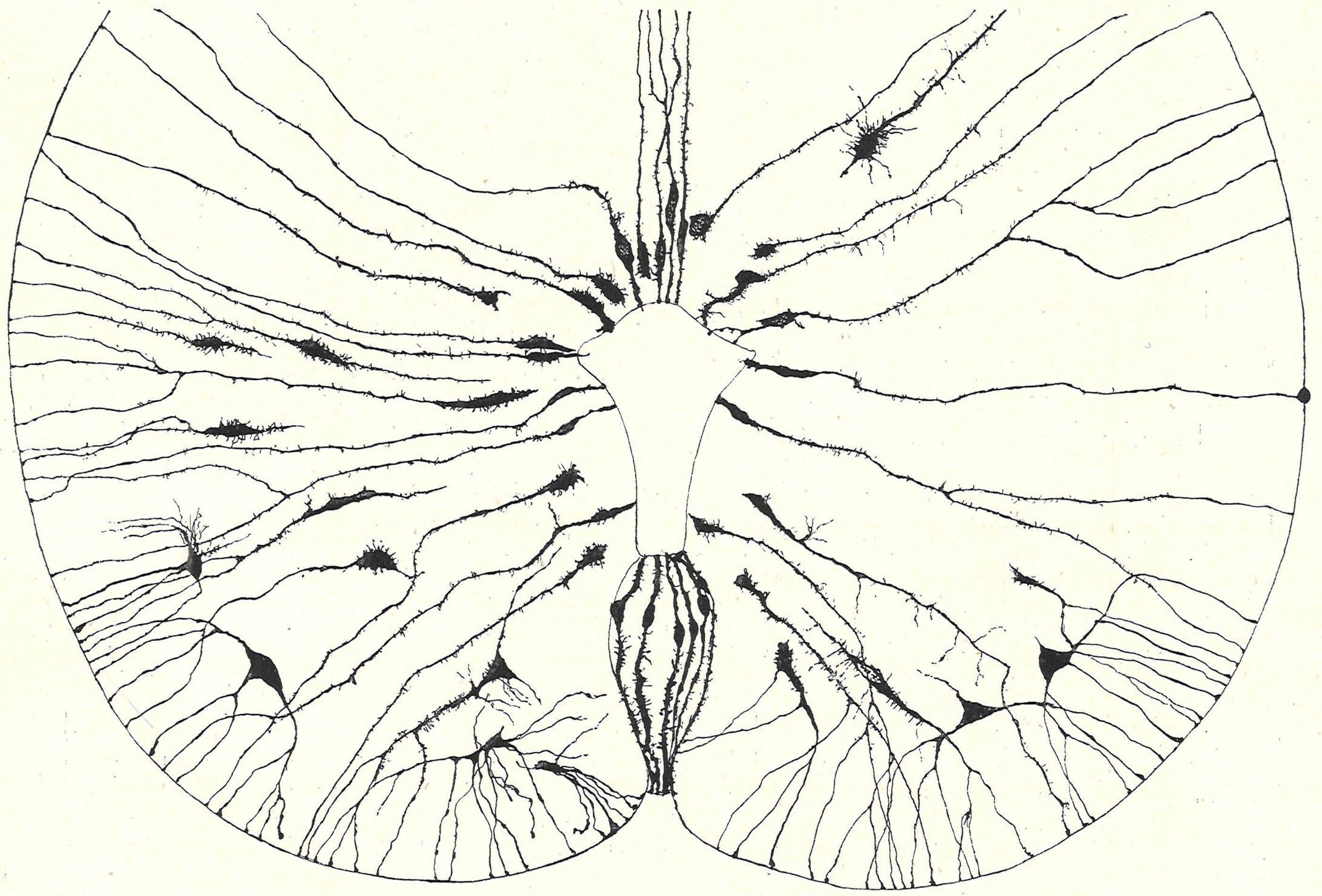

Fig. 5.

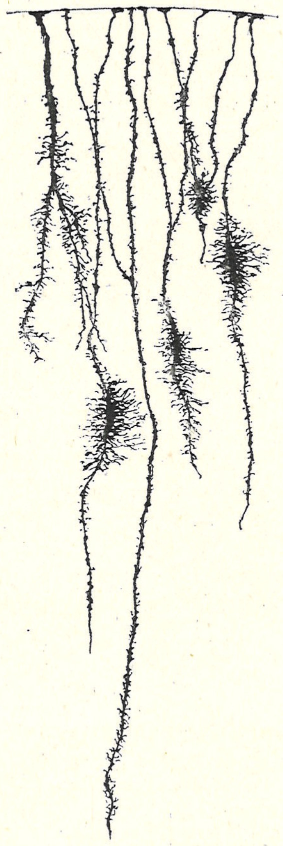

Gez. v. Gustaf Retzius. Typogr. Central-Tryckeriet. Fototypi v. J. Cederquist.

Tafel XI.

Ependym und Neuroglia

im menschlichen Rückenmark.

Fig. 1. Querschnitt vom Rückenmark (Lendenregion) eines 3 Cm. langen menschlichen Embryos. Ependym- und Neurogliazellen. Die punktirte Linie deutet die Grenze zwischen Strangsubstanz und grauer Substanz an. Einige von den Hintersträngen abgehende Collateralen sind wiedergegeben; *bl,* Blutgefäss.

Fig. 2. Septum posticum und dorsale Partie des Centralkanals vom Rückenmark eines 3 Cm. langen menschlichen Embryos. Ependymzellen.

Fig. 3. Querschnitt vom Rückenmark eines 15 Cm. langen menschlichen Embryos (Halsregion). Ependym- und Neurogliazellen.

Fig. 4. Ventraler Ependymkeil vom Rückenmarke (Halsregion) eines 15 Cm. langen menschlichen Embryos.

Sämmtliche Figuren sind nach Golgi'schen Präparaten wiedergegeben.

Die Fig. 1 und 2 sind bei Vér. Obj. 6 und Ocul. 1 (eingeschob. Tubus), die Fig. 3 bei Vér. Obj. 2 und Ocul. 3 (eingeschob. Tubus), die Fig. 4 bei Vér. Obj. 6 und Ocul. 3 (eingeschob. Tubus) gezeichnet.

Fig. 1.

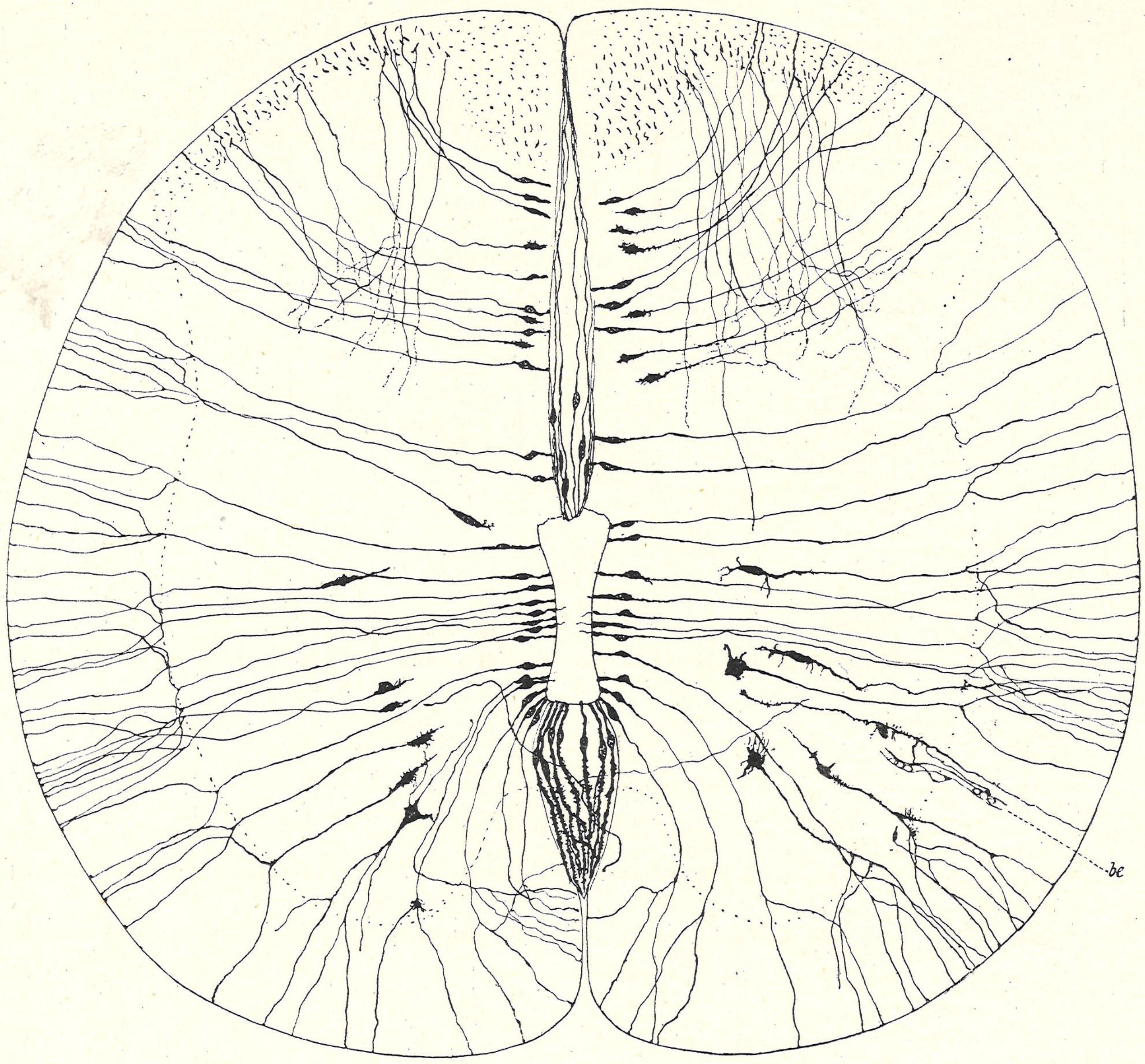

Fig. 2.

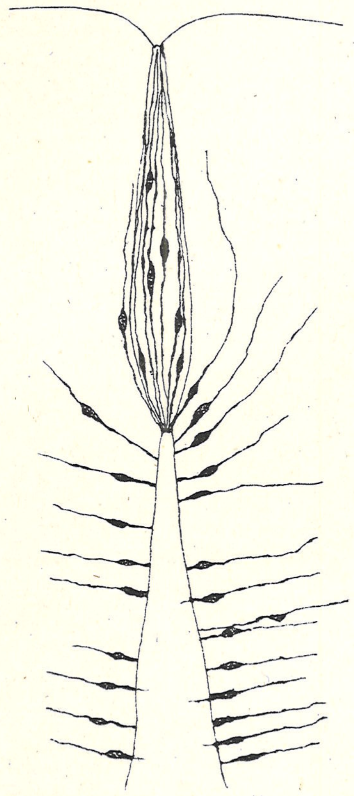

Fig. 3.

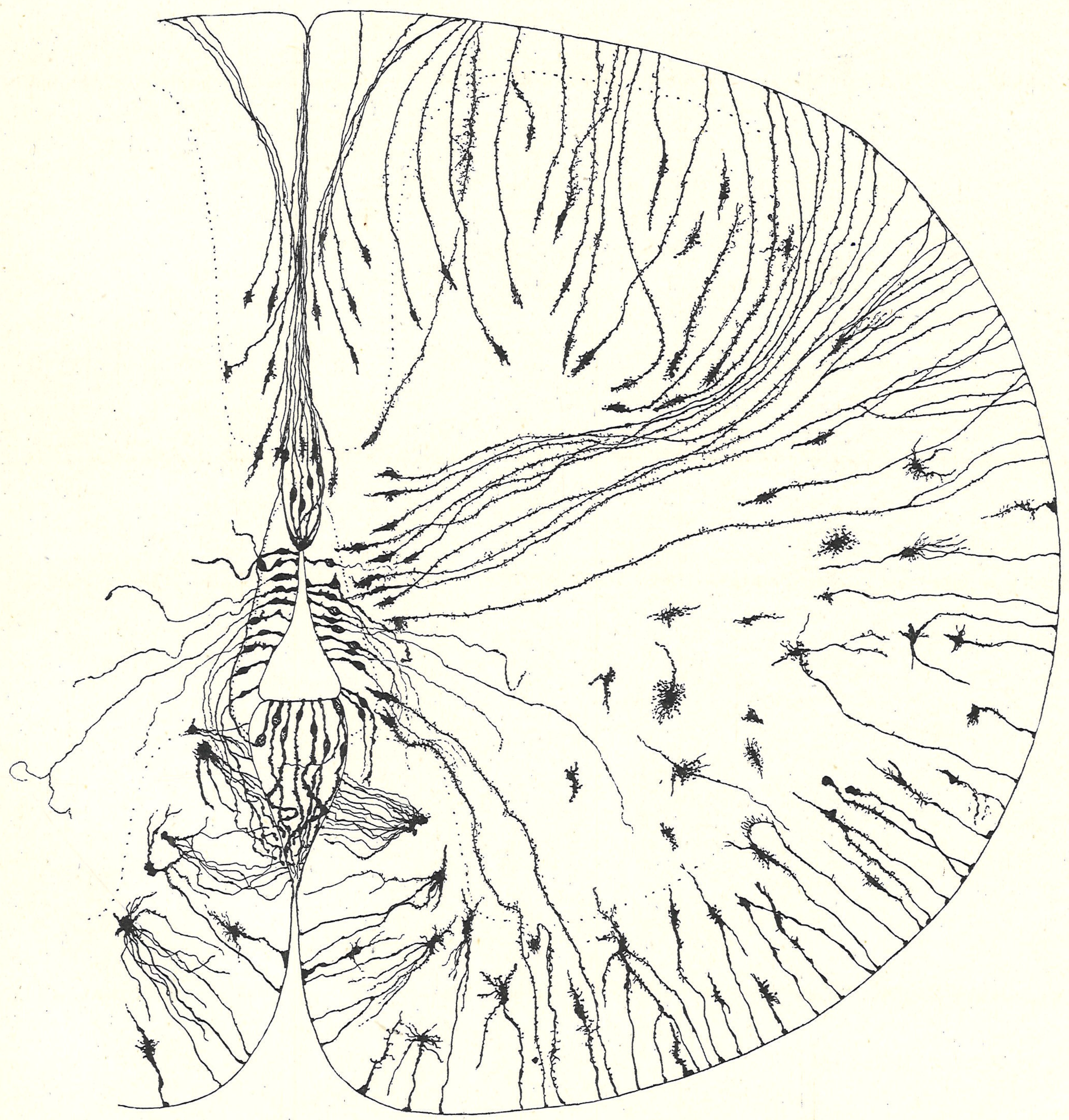

Fig. 4.

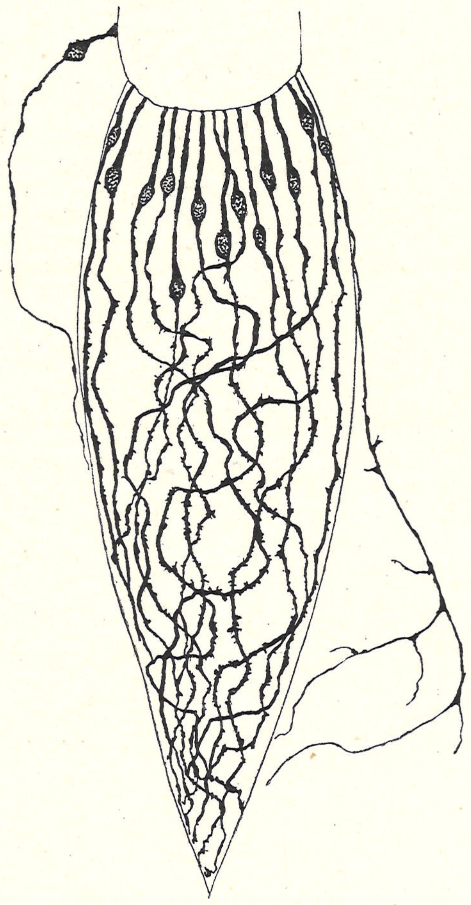

Gez. v. Gustaf Retzius. Typogr. Central-Tryckeriet. Fototypi v. J. Cederquist.

Tafel XII.

Ependym und Neuroglia

des menschlichen Rückenmarkes.

Fig. 1. Mittlere Partie vom cervicalen Rückenmarke eines 26 Cm. langen menschlichen Foetus.

ck — Centralkanal. *sp* — Septum posticum.

vF — vordere Fissur. *bl* — Blutgefässe.

Rings um den Centralkanal sieht man die *Ependymzellen* und nach aussen davon *Neurogliazellen* von verschiedenem Typus (Spinnenzellen, Mooszellen etc.).

Nach einem Golgi'schen Präparate bei Vér. Obj. 6 und Ocul. 3 (eingeschob. Tubus) gezeichnet.

Fig. 1.

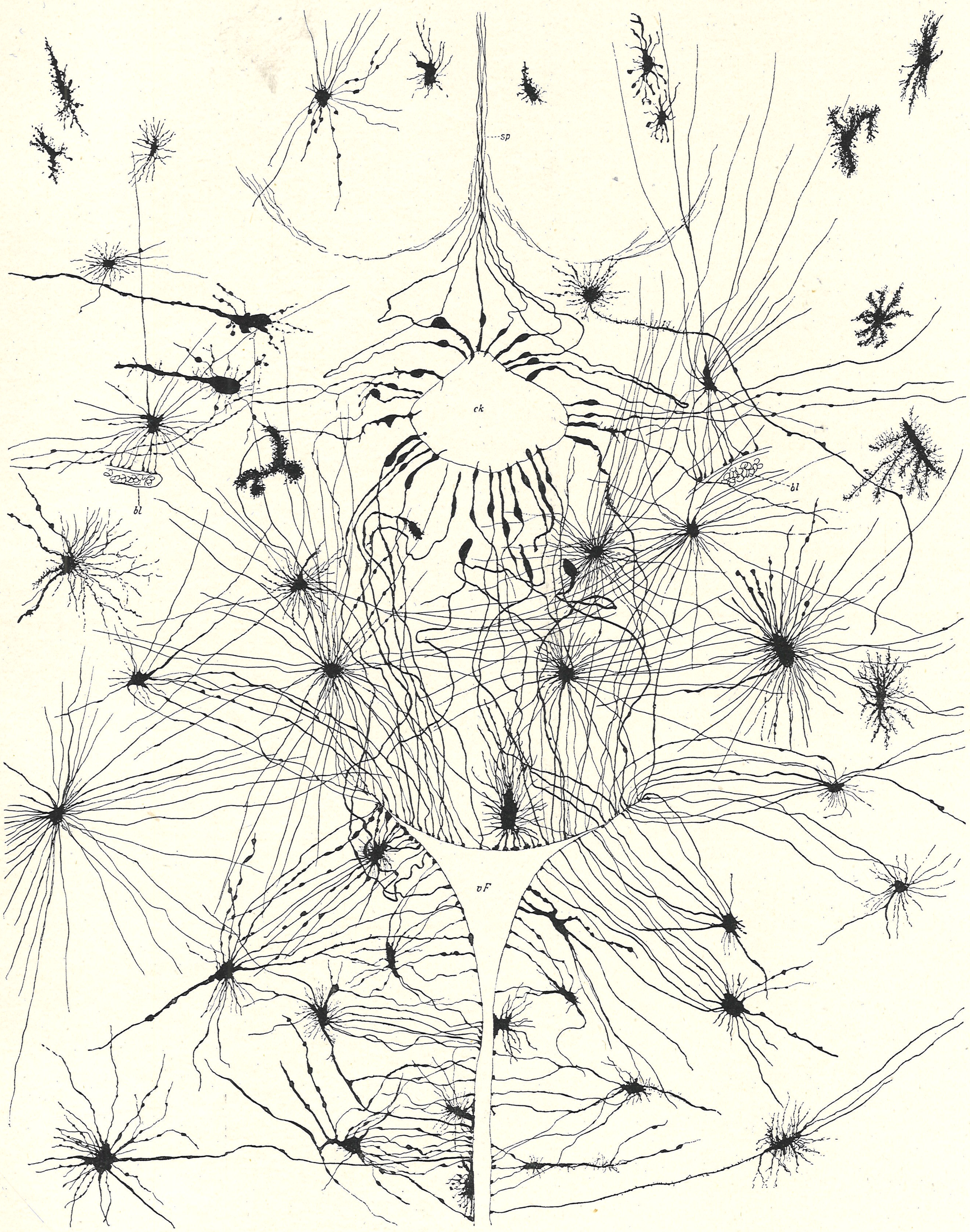

Gez. v. Gustaf Retzius. Typogr. Central-Tryckeriet. Fototypi v. J. Cederquist.

Tafel XIII.

Ependym und Neuroglia

des menschlichen Rückenmarkes.

Fig. 1. Partie vom Querschnitt des Halsmarkes eines 26 Cm. langen menschlichen Foetus. Antero-lateral region. *Neurogliazellen* verschiedener Gestalt.

o — Oberfläche des Markes.
p — Grenze der grauen Substanz.
w — Strangsubstanz (weisse Substanz).
gr — graue Substanz.

Fig. 2. Kleine Partie von einem Querschnitt des Rückenmarks eines 26 Cm. langen menschl. Foetus. Seitenstrang.

o — Oberfläche des Markes.

Fig. 3. Fünf Neurogliazellen aus der Substantia gelatinosa Rolandi. Von einem 26 Cm. langen menschl. Foetus.

Fig. 4. Partie eines verticalen Längsschnitts vom Vorderstrang eines 26 Cm. langen menschl. Foetus. Neurogliazellen verschiedener Gestalt.

o — Oberfläche des Markes.
w — Strangsubstanz.
gr — graue Substanz.

Die Figuren sind nach Golgi'schen Präparaten bei Vér. Obj. 6 und Ocul. 3 (eingeschob. Tubus) gezeichnet.

Fig. 1.

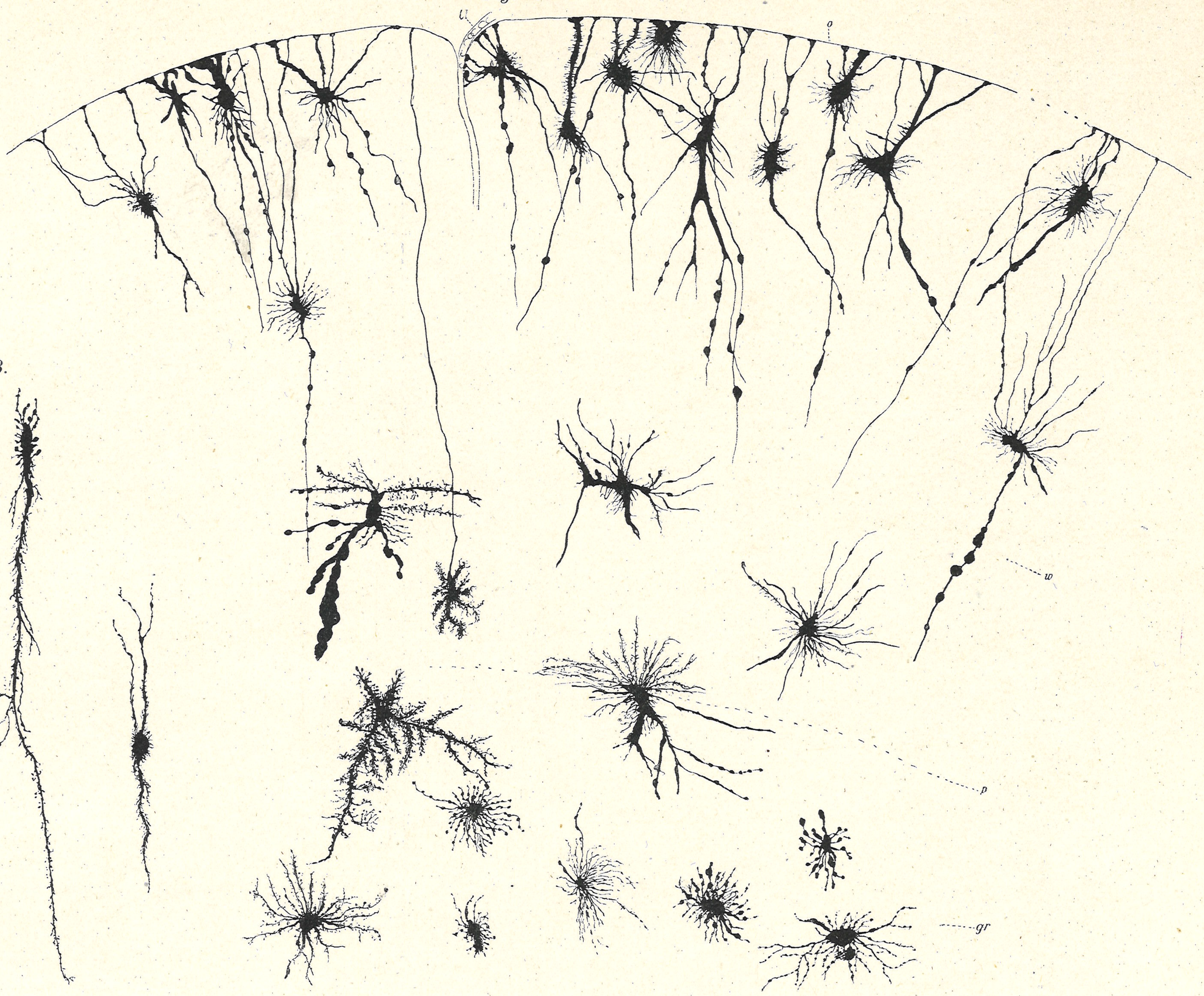

Fig. 3.

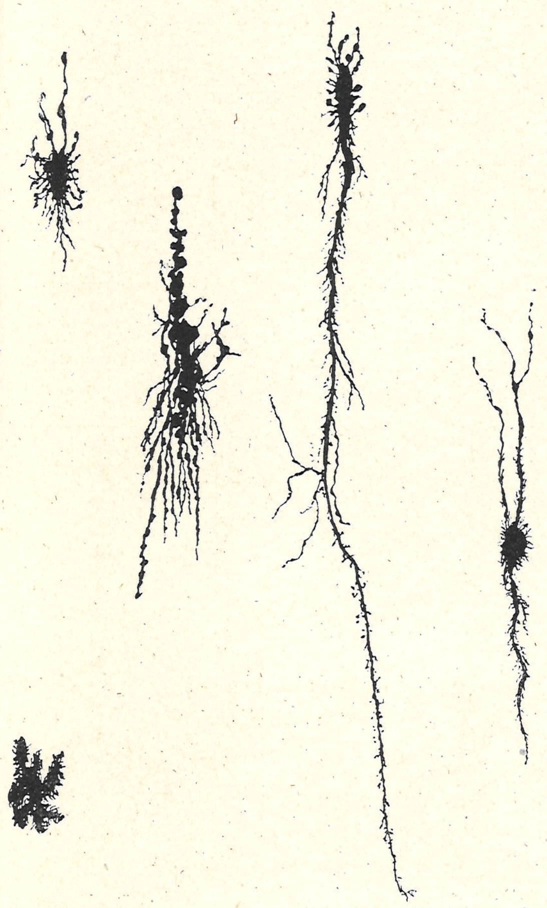

Fig. 4.

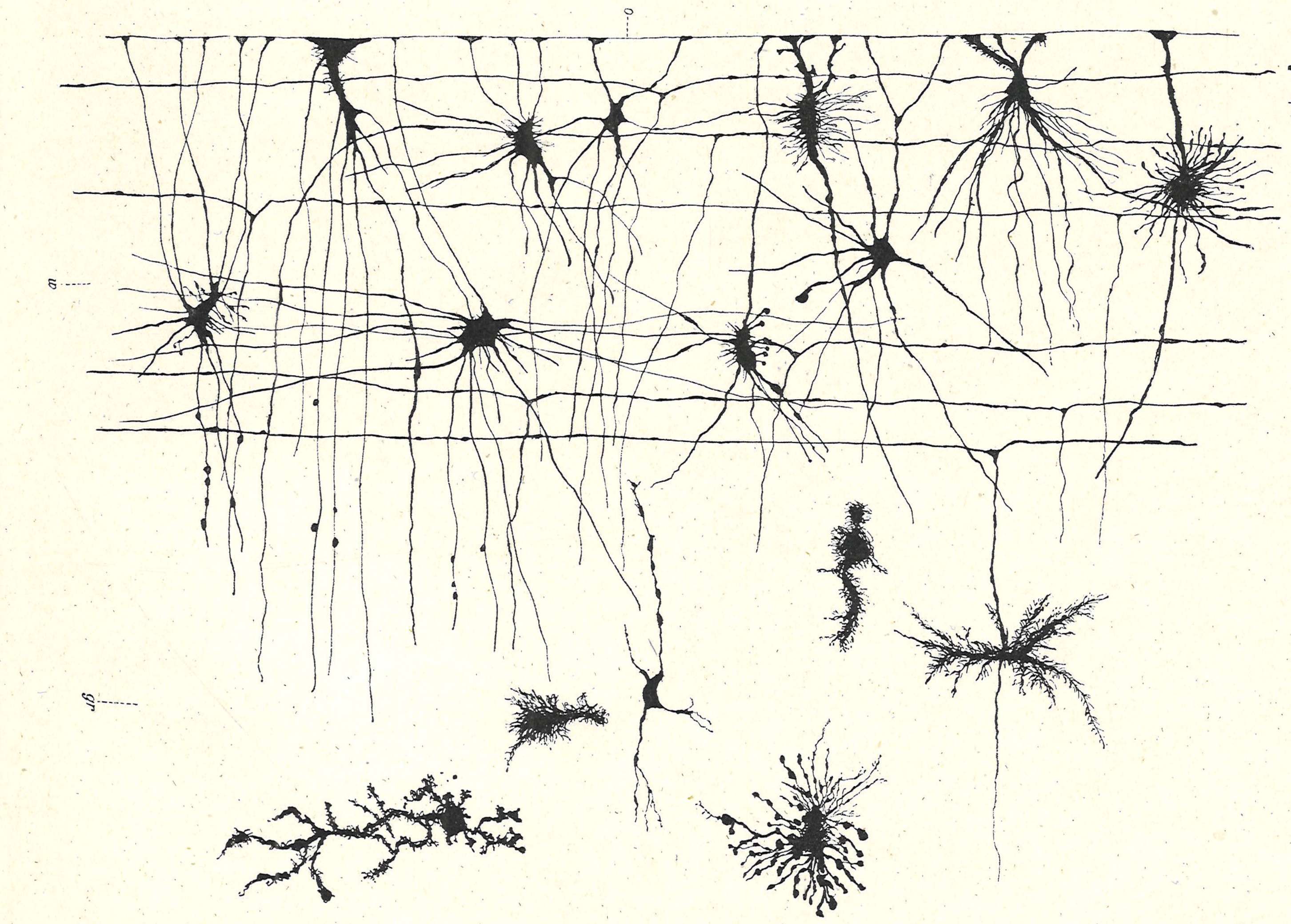

Fig. 2.

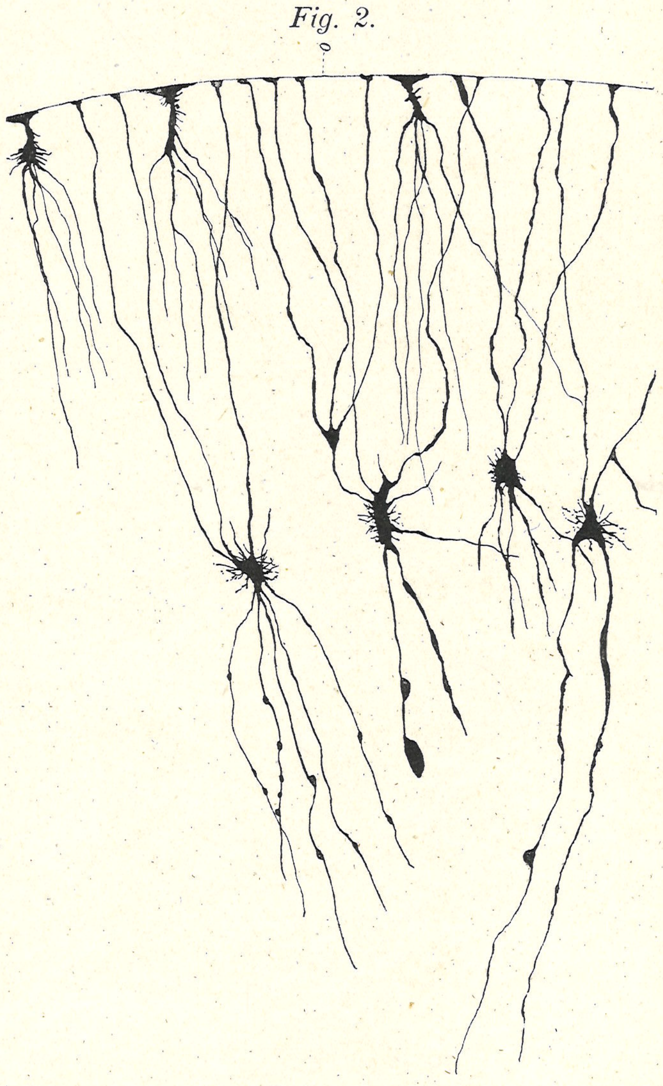

Gez. v. Gustaf Retzius. Typogr. Central-Tryckeriet Fototypi v. J. Cederquist.
