# Supplementary material for: The Concept of Neuroglia ‐ the State of the Art Circa 1900
Source: Glia. 2025 Feb 4;73(5):890–904. doi: 10.1002/glia.24678 (PMC11920685; doi:10.1002/glia.24678)
Supplement: Supplementary file 2 — Data S2. Translated text by Rezius, Volume V with single figures inserted into the text. [file GLIA-73-890-s007.pdf]

Biological Investigations

by

Prof. Gustaf Retzius

Studies on ependyma and neuroglia.

Tables V-XIII

1. Ependyma and neuroglia at cyclostomes. Tables V-VII
2. At bony fish- Table VIII, Figs. 1-4
3. At amphibia. Table IX
4. At birds. Table VIII, Figs. 5-8
5. At mammals. Table X
6. At humans. Tables XI-XIII

Studies on ependyma and neuroglia.

Tables V-XIII

When I published a preliminary report on the ependymal cells of the central organs[1] two years ago, it was my intention to address this topic in more depth later. At the anatomical congress in Munich in the same year, von Lenhossek[2] presented his investigations, *Zur Kenntnis der Neuroglia des menschlichen Rückenmarks* ("On the studies on neuroglia of the human spinal cord"), and this issue was so excellently presented that I could not add anything significant. I set my study aside at that time.

In the meantime, I often obtained images in my studies on the structure of the central organs, which provided novel insights into the ependyma and neuroglia. Since these insights are from animals, such as bony fish, which have been little studied, I have decided to publish the images I obtained on different occasions. These will help support the not yet generally accepted views and may fill some gaps. I therefore intend to provide not a summarizing account, but rather a series of additional contributions. Under these circumstances, there is no room for an extensive historical account; this has already been provided by others. I will therefore not touch the pre-Golgi era, but only provide important data from the later period of that issue. With respect to the pre-Golgi era, one can find profound accounts from Gierke[3], based on reports and views of previous scientists such as Deiters, Reil, Jastrowitz, Ranvier, and others.

With Golgi's epochal studies, a new era began providing new insights into neurohistology. He also advanced the field of ependyma and neuroglia, and due to his and Weigert's methods, we obtained tools to successfully advance this field. With respect to ependyma, it was known (Stieda, Reissner, and others) that at least in some vertebrates, the ependymal cells in the spinal cord extend thread-like processes radial to the surface, particularly to the front and rear end. But only with Golgi was this fact finally established and extended. In his extensive publication on the fine anatomy of the central nervous system[4], he reports only a preliminary description, which was based on profound investigations later confirmed by several others.

Numbers with an added # are comments from the translator and are summarized at the end of the translation.

[1] Gustaf Retzius, Zur Kenntnis der Ependymzellen der Centralorgane. Verhandl. d. Biolog. Vereins in Stockholm. Bd. 3, 1890-91 (1. März 1891)

[2] Mich. Von Lenhossek, Zur Kenntnis der Neuroglia des menschlichen Rückenmarks. Verhandl. d. Anatomischen Gesellschaft auf d. fünften Versamml. in München. 18.-19- Mai 1891.

[3] H. Gierke, Die Stützsubstanz des Centralnervensystems. Archiv f. mikrosk. Anatomie. Bd. 25, 1885.

[4] Camillo Golgi, Sulla fina anatomia degli organi centrali del sistema nervosa. Reggio Emilia, 1885. I have used the German translation provided by the author, which was published in Anatom. Anzeiger, Jahrg. V, No13 & 14, Juli 1890.

In chicken embryos at different stages (4th to 12th day of breeding), he found that the epithelium of the central canal played a direct and substantial role in the formation of the interstitial substance in each of its parts. This was true not only for the grey matter in its full extension but also for the white matter of the dorsal, lateral, and ventral strands, extending from the central canal to the outer limits of the spinal cord directly under the pia mater. The individual cylindrical epithelial cells of the central canal extend radially through the entire area of the spinal cord and reach the outermost periphery of this organ beneath the pia. Here, the thread-like processes of the individual cylindrical cells terminate; they sometimes form a conical swelling or a smaller extension, by which the processes attach either to the pia or to the vessel walls. Along their course, the more or less strong processes, which are extensions of the body of the individual epithelial cells, show arborizations. These are sometimes sparse but more often numerous and complex. The secondary branches sometimes reach the periphery of the cord where they terminate as mentioned above, sometimes they are lost in their course, and sometimes they attach to vessel walls.

Due to these delicate and dense extensions of fibers, a coherent tissue forms between the center and the periphery of the cord. Golgi further remarked, based on his results, that he assumed the interstitial stroma of the nerve centers belongs to tissues originating from the outer germinal layer or hornwort[1#]. He adds at the end of the corresponding chapter: “Based on this description it is evident that the epithelial part of the spinal cord belongs to those tissues which originate from the outer germinal layer and takes part in the generation of the tissue (of the neuroglia) embedded between the nervous elements. Based on several embryonic, chemical, and histologic facts,” he says, “I consider the cells of the neuroglia as being equivalent to the epithelia and I will present this in another publication as already stated.”

As far as I know, this publication has not yet appeared as promised by the author. The aforementioned preliminary report clearly outlines his statements and views. The researcher who examined the issue was Fridtjof Nansen,[1] and he studied Amphioxus[2#] and Myxine[3#]. In the first animals, he observed that the cylindrical epithelial cells, which surround the central canal, penetrate the white matter with their outer processes running partially in bundles and connect with their terminals at the ensheathment of the spinal cord. No neuroglial cells like in Myxine were found there. “We may thus consider,” says Nansen, “that these epithelial cells are the true neuroglial cells of Amphioxus and that these neuroglial cells are the first stage of these elements in vertebrates. In Myxine, two types of such elements are present, namely the epithelial cells which surround the central canal and which arise at the dorsal and ventral part of the cord to the surface. They also occur at the lateral parts while Nansen could not confirm their terminal ends there. Second

are the neuroglial cells, which are abundant in the grey matter, having a small cell body which is often equipped with multiple, less-branched processes. Those extend to the dorsal and ventral surface, sometimes to both, and terminate there. They can be nicely visualized with the Golgi method. The processes of these cells do not anastomose (fuse) among each other. At the lateral part of the canal are transition forms between these two cell types. “I think,” he says, “that we can conclude that the neuroglial cells are of ectodermal origin and that they originate from the epithelial cells which surround the central canal.”

After this issue was studied by the Italian researchers Falzacappa and Magini, it was further investigated by Ramón y Cajal and von Kölliker using the Golgi method. Cajal[2] supported the finding of Golgi in chicken embryos.

[1] Fridthof Nansen, The Structure and Combination of the Histological Elements of the Central Nervous System. Bergens Museums Aarsberetning for 1886. Bergen 1887.
[2] Ramon y Cajal, Sur l´origine et les ramifications des fibres nerveuses de la mole embryonaire. Anatom. Anzeiger, V. Jahrg. No 4. Febr. 1890.

He first described the distinct disposition of the ependymal cells in the anterior and posterior commissures, as well as in the lateral parts in more detail and provided good images of spinal cord cross sections of the chicken embryo at the 9th day of breeding; he mentioned small, thorny branches at the edges of the individual ependymal fibers and conic thickenings at the terminals below the pia mater. He even observed that the inner ends are extended by long and fine cilia inserting into the central canal. At the 8th day of breeding, says Cajal, one can observe certain structures between the ependymal elements which are shorter and do not reach the central canal; they stem from a dislocation and maybe proliferation of the other ependymal elements and are precursors of spider cells. The older the embryo becomes, the shorter and more branched appear the ependymal fibers which radially penetrate through the lateral parts of the spinal cord. The neuroglial and spider cells can be recognized in the spinal cord of chicken embryos from day 9th or 10th day on; they are first found in the ventral horn and soon after in the white matter of the strands and finally in the dorsal horn. The spider cells are essentially dislocated and transformed ependymal elements; from the 7th to 14th day, one finds all kind of transitions with respect to form and position. One can even observe in the cord of adult mammals neuroglial cells with a long central thread (ependymal process) which one can follow through the grey matter and which possess one or multiple peripheral, radial processes. In the cerebrum and in the cerebellum are neuroglial cells predominantly positioned in one orientation: the radial cells of the latter provide a nice example. A leucocytic origin of the neuroglial cells is not acceptable. These cells do not anastomose among each other, but are completely independent elements. – In some of his following studies, Cajal discusses occasionally the ependymal and neuroglial cells of other areas of the central organs such as those in cerebellum or the cerebral cortex.

In his work on the spinal cord, Kölliker[1] also discusses the issue of neuroglia. Early in development, the cord of young embryos harbors one type of glial cells, essentially the so-called epithelial cells of the central canal, which penetrate radially through the entire cord with their processes and terminate at the surface close to the pia with larger or smaller enlargements. The longer types of these elements, which all have only one nucleus close to the central canal, exhibit acute-angled branches and many side branches at their outer parts, creating the appearance of a net that does not actually exist.

The first appearance of these glial cells, studied by His and Vignal, can be easily recognized in young embryos and partly refers to cells of the medullary plate that grow into fiber cells with lateral branches. These original features of the glial cells are maintained for quite some time, as von Kölliker observed in a sheep embryo measuring 9 cm and a pig embryo measuring 10 cm.

In older embryos and after birth, elements ranging from the base of the ventral cleft to the dorsal midline remain; in contrast, other elements become increasingly faint, except for those around the central canal. Throughout the cord, numerous new glial cells with their characteristic star-shaped form appear, clearly developing from the undifferentiated cells of the cord anlage. As long as this organ remains undeveloped, they exist as roundish cells present in large numbers between the nervous elements in the white and grey matter, but they are no longer found later.

M. von Lenhossek[2] studied the development of the spinal cord of chicken embryos at the same time as Cajal. He described the development of the ependymal cells or radial fibers, which can already be stained by the third day. From the beginning, they are equipped with multiple threads and irregularities that branch off at right angles. They expand in later phases but are only present in sections of the inner grey matter. Divisions appear early in the outer region in the area of the white matter layer or close to it, appear first in the form of dichotomic separations and become slowly more complex.

[1] A. von Kölliker, Zur feinen Anatomie des centralen Nervensystems. Zweiter Beitrag: Das Rückenmark. Zeitschr. f. wissensch. Zoologie. Bd 51, 1890.
[2] Mich. Von Lenhossek. Zur ersten Entstehung der Nervenzellen und Nervenfasern bei dem Vogelembryo. Mittheil. aus dem anatom. Institut im Vesalianum zu Basel, 1890.

The central processes are always undivided. The cells at the floor plate are in the beginning significantly broadened, but shrink gradually until they are, at the 8th day, of the type of the other radial fibers. A complication of that simple support system appears at the 6th day due to the appearance of Deiters cells which appear first in the region of the central canal. They are nothing else than radial cells which have lost their central process, yet still project with their peripheral part to the surface of the cord. On the 12th day one finds multiple free neuroglial cells; they largely have a distinct, characteristic form and often do not reach the periphery with their outer processes. “This later sort of neuroglial cells is obviously not generated,” says von Lenhossek, “from a transformation and evasion of the primitive radial cells, but directly from mitosis in the germinal layer.”

Oyarzun [1] described highly branched ependymal cells in the ventral horn of amphibia (frog, newt[4#], salamander) after treatment with the Golgi method; I [2] provided a short illustration of the form and arrangement of these cells in brain and spinal cord of different vertebrates (pike, frog, rabbit, cat). In a report published at the same time [3] I also discussed the neuroglial cells of the outer cerebral and cerebellar cortex of mammals, and the latter cells were also recently studied by Martinotti [4]. I described, among other issues, the properties of ependymal cells in the spinal cord of the cat embryo, both anterior, posterior (in the frontal and rear wedge parts or ependymal wedge) as well as in the lateral parts. Based on my fragmented illustrations, I concluded, “that the arrangement of ependymal cells, recently reported by several researchers, seems to be a universal phenomenon. Not only in embryos but also in young animals or adult lower animals, such cells are largely present, extending from the ventricles or the central canal to the surface of the brain and spinal cord. However, they may be atrophied in some areas, making them difficult to detect. It is quite evident that these ependymal cells, which show a fixed habitus and often have lateral branches, form a support system."

Regarding the features of these ependymal cells, I supported Kölliker’s view. “These two types of cells are distinct. The origin and development of true neuroglial cells remain unknown.”

In his study on the spinal cord and cerebellum, Van Gehuchten[5] also discussed ependymal and neuroglial cells. Regarding the former, he supported Golgi’s view and provided a cross-sectional image of a rabbit close to birth with abundant ependyma. The neuroglial cells in the white and grey matter have multiple processes but do not always exhibit identical features.

Often one of the processes which is thicker, extends far out; this is commonly the case in the posterior part of the central canal, where the neuroglial cells in the medial septum extend to the surface of the spinal cord; many of the cells located close to the septum project against the previous perpendicular direction and send their processes up to the Substantia Rolandi. These cells are clearly ependymal cells, which somehow have lost their connection to the central canal. Van Gehuchten also described the Bergmann fiber cells of the cerebellum and provided images from them.

In the same year appeared the report by Lachi [6] on the development of the neuroglia. In that respect, he distinguished two periods. In the first, which lasts to the 8th or 9th day of breeding,

[1] A. Oyarzun, Über den feineren Bau des Vorderhirns der Amphibien. Arch. f. mikrosk. Anatomie. Bd. 35, 1890.

[2] Gustav Retzius, Zur Kenntnis der Ependymzellen der Centralorgane. Verhandl. d. Biolog. Vereins in Stockholm Bd. 3 (1890-1891); 15 März 1891.

[3] Gustav Retzius, Ueber den Bau der Oberflächenschicht der Grosshirnrinde beim Menschen und bei den Säugethieren, ebenda (same reference).

[4] Carlo Martinotti, Beitrag zum Studium der Hirnrinde und dem Centralursprung der Nerven. Internat. Monatsschrift f. Anat. und Phys. Bd. 7, 1890.

[5] A. Van Gehuchten, La structure des centres nerveux. La Moelle epiniere et le cervelet. La Cellule, t. 7, dep. 1. 20 avril 1891.

[6] Pilade Lachi, Contributo alla istogenesi della Nevroglia nel midillo spinale del pollo. Atti della Societa toscana di scienze naturali, resid. In Pisa. Memorie. Vol. 11, 1891.

the neuroglia is represented exclusively by spongioblasts of ectodermal origin. In the second period, from the 8th or 9th day to the first days after birth, there appear elements of mesodermal origin, first in the white and then also in the grey matter; these mesenchymal elements, proliferate multiple times by indirect division and extend processes after hatching, which are characteristic for neuroglial cells. From 21th day on, there are other elements of vascular origin, may it be endothelial cells or leucocytes.

In the spring of that year, von Lenhossek[1] presented his aforementioned lecture at the anatomical congress in Munich on the neuroglia of the human spinal cord, which was later published in the proceedings of the anatomical society. It is not possible to provide a complete account of this content-rich and precise illustration; I will limit myself to highlighting the most important statements.

Von Lenhossek studied human embryos, focusing primarily on a 14 cm-long embryo, which he successfully stained and used as the basis for his description. The support system of the cord consists of an exquisite fibrous structure: a rich system of long, thin fibers projecting from the inner parts of the cord to the periphery. Attached to these fibers are a large number of small lateral branches and appendages, forming a compact scaffold with only simple contact points. These fibers originate from distinct cells, residing partially as ependymal cells at the central canal and partially as Deiters' cells or neuroglial cells, arranged in specific patterns in the grey matter and less frequently in the white matter.

At this developmental stage, the distribution of these elements, consisting of longitudinal spindle-shaped or elliptic cell bodies with multiple fine, short fibers, varies across different regions. In the ventral horn, glial cells are least numerous, while in the dorsal horn their number increases steadily. Within the white matter, their number is lower than in the grey matter. All cells are unquestionably of the same origin; they stem from the ectoderm, undergoing mitosis in the innermost layer of the medullary tube and reaching their final destination through successive migration.

“I completely miss,” says von Lenhossek, “any other glial elements in the human embryo, even in advanced stages, besides those just described. It may be those related to blood vessels or those with characteristics of connective tissue cells. Thus, the support tissue of the human spinal cord appears to be exclusively a formation of the outer germinal sheet, at least until late stages of development.” The radial, so-called “pial septa” are peripheral processes assembled into larger bundles originating from deeper neuroglial cells. In the mature spinal cord, this pattern remains as a basic structure, albeit with extensive complications. However, the peripheral process gradually loses its importance; the

originally elongated, spindle-shaped cells increasingly acquire a spider-like character. While it is basically the same cell type, v. Lenhossek defines in the interest of clarity, three categories: 1) ependymal cells, 2) cells of the grey and 3) cells of the white matter. The ependymal cells have commonly a spindle-shaped cell body with a free, central cuticular plate and from its center protrudes one bristle which is intensively black labelled, long, stiff, out-standing and generally hook-like bent as observed in a 23 cm long embryo (in the 14 cm long embryo v. Lenhossek did not find it); this bristle cannot be considered as cilia. The description of this fine process which projects always to the periphery needs special attention depending on the region. In the region of the frontal commissure, the ependymal cells show a meridian-like, barrel-formed arrangement by converging their peripheral ends towards the frontal longitudinal fissure; the middle ones are sagittal; they are always undivided and commonly run in wave-form and are usually stronger than the other fibers;

[1] Mich. von Lenhossek, Zur Kenntnis der Neuroglia des menschlichen Rückenmarkes. Verhandl. d. Anatom. Gesellschaft auf der fünften Versamml. in München, 18-20 Mai 1891.

commonly they are smooth and only at its beginning equipped with delicate site branches. At the transition between the frontal and lateral wall of the central canal a sparse cell type is found which is distinct from other ependymal cells since its also bow-formed median oriented processes show multiple divisions; the 6-8 delicate, diverging terminal branches show a triangular thickened end at the frontal fissure facing part of the frontal strand. Such a branch pattern can also be found in the chicken embryo (4-5 day). The arrangement of the lateral ependymal cells is very typical in that they extend radially; the majority forms a massive bow with a concave shape oriented towards the front in the ventral half and towards the back in the dorsal half, while in the middle, they are almost straight. Their course is slightly wavy, possibly due to fixation, and a fork-like division is almost always found in the area of the white matter; the ends exhibit terminal knots. Between the lateral and posterior regions, the so-called septum posticum-forming fibers create a significant intermediate space that lacks ependymal fibers in the area of the dorsal horn and dorsal strands.

Von Lenhossek describes the peculiar reduction of the dorsal part of the central canal, which becomes similar to a sagittal cleft and disappears due to the fusion of its spaces. As a result, the cells located posteriorly at the midline move forward; many of these cells lose their ependymal character and transform into Deiters' cells. They retain their original position, remaining perpendicular to the sagittal median plane, and now form the early ependymal fibers of the dorsal horns and dorsal strands. The posterior ependymal cells, relatively thick, always undivided, and somewhat ragged, show a strong medial position and a densely bundled arrangement.

The septum posticum, according to the authors[5#], is not a continuation of the pia mater but is ependymal and belongs to the spinal cord substance. The cord is unquestionably undivided and shows only a weak groove. In context, the number of ependymal cells relative to other elements of the support system remains insignificant.

With respect to the glial cells of the grey mater, they a numerous in the area of the substantia gelat. centralis; they have a clumsy, roundish form with a fairly rich set of fibers, which shows a concentric arrangement at the central canal and smooth bordered constitution. Many of the neuroglial cells of the grey matter are equipped with a central process besides the peripheral one; the former is generally short, sometimes longer and extends towards the central canal without reaching it. In the peripheral parts of the grey matter, namely in the area between ventral and dorsal horn, there are spindle-shaped elements, perpendicular to the radial extension, and both of their ends convert into processes which in a bow-form fashion extend into the direction of the periphery, but do reach only one. Von Lenhossek describes

precisely the peculiar course of the neuroglial cells of the dorsal horns and in particular of the substantia gelat. Rollandi; their features are mainly based on the composition of numerous neuroglial cells and fibers, in particular due to their coat-like character. The neuroglial cells of the white matter are quite sparse in this period, but show quite some diversity with respect to forms; he divides them into four types. Type1, the simplest, is found in the dorsal strands where all these cells are found; the cell is elongated, radial oriented, lacks mostly a central process and is equipped only with one peripheral; this process is always undivided, smooth and a little bit in wave-form and extends outward; the examples found in the ventral and dorsal strands of that cell type are equipped with numerous, delicate secondary branches. Type 2 shows 2-5 or more peripheral processes which extend already separately from the cell respective from their peripheral pole and in a bushy, divergent arrangement; they are fairly strong, generally smooth, but also often diverse, running in waves and undergo further divisions; a short central process is not rare. Type 3 consists of spindle-shaped cells, and both of their ends extend into one process; those processes extend in a bow-form fashion to the periphery and divide further. Type 4 is corresponding to the region of the ventral strands oriented towards the frontal fissure; they are characterized by dividing processes extending towards the periphery with multiple, bushy branching. – All these cell types experience a transition during further development, by reducing their spindle-shaped body into a more regular spider-shaped form and the course of their processes becomes straighter. The neuroglial cells become more and more a common type; the number of branches remains diverse.

Ramón y Cajal[1] described the ependyma and neuroglia in the spinal cord and brain of reptiles (*Lacerta*[6#]), and in the brain of amphibians (*Rana*[7#]) and birds. Pedro Ramón[2] also provided illustrations of these elements in the reptile brain, and Cl. Sala[3] illustrated them in the spinal cord of amphibians (*Rana*). I[4] confirmed Nansen's reports on the neuroglial cells in the spinal cord of *Myxine*.

Then, M. von Lenhossek[5] studied the neuroglial cells in the spinal cord of *Pristiurus* embryos[8#]. He did not obtain good images of ependymal cells, but the other neuroglial cells were stained, particularly in the ventral cord section, in a coffee-brown color. The small, angled cells have only one main process; all processes, which be 5 to 6 or even more, extend to the surface of the cord. They have a highly divergent course, such that the extension of a single cell can envelop almost half of the cord. The processes are rough and stiff in composition, ending at the surface with a shank-like thickening. They are similar to the types of cells in Cyclostomes.

Finally, von Lenhossek provided a comprehensive overview of the neuroglia of the spinal cord in his description of the fine structure of the nervous system,[6] published last year. This illustration generally relates to his previous reports but contains novel aspects, such as the relationships of *Petromyzon*[9#] and *Raja*[10#].

As I now move on to my own results, I can state my opinion regarding the neuroglial concept. My views are generally consistent with those of Golgi, Nansen, Ramón y Cajal, and von Lenhossek. Therefore, I do not need to provide a further detailed description after this historical introduction. I will therefore limit myself to a brief description of my results and primarily provide an explanation of my corresponding figures.

1.Ependyma and Neuroglia at the Cyclostomes

Table V-VII.

Leaving the conditions in *Amphioxus* aside, since I do not have anything to add to the reports by Nansen, Rohde, and von Lenhossek, I will start with the Cyclostomes. Regarding *Myxine*, my ongoing studies on the ependyma and

neuroglia of the spinal cord have further supported Nansen's results. In the brains of these animals, I successfully obtained staining (see below).

I will not discuss the Myxine, but will go to the conditions of the Petromyzon. Already in my presentation of the nervous system of the Myxine (Biol. Unters. II, 1891) I have mentioned that the preparations from the spinal cord of the Petromyzon obtained with the Golgi method are very similar to those of Myxine, "as well as with respect to the ganglion cells and their processes as well as with the neuroglial cells." It was my intention to publish simultaneously an image of these preparations, yet the prepared picture (the below shown Fig. 1 of table V) was misplaced. Since then, Lenhossek published last year in his

[1] S. Ramon y Cajal, Pequenas Contribuciones al Conecimiento del sisterna nervioso. Trab. d. Laborat. histol. de la facultad de medicina de Barcelona. Aug. 1891.
[2] Pedro Ramon, El encefalo de los Reptiles. Trab. d. Laborat. histologia de la facultad de medicina de Zaragoza. Sept. 1891.
[3] Cl. Sala, Estruetura de la medulla espinal de los batracios. Trab. d. Laborat. histologia de la facultad de medicina de Barcelona. Febr. 1892.
[4] Gustav Retzius, Zur Kenntnis des Nervensystems von Myxine glutinosa. Biol. Unters. N. F. II, 2. 1892.
[5] M. von Lenhossek, Beobachtungen an den Spinalganglien und dem Rückenmark von Pristiurusembryonen. Anat. Anzeiger, 7. Jahrg., 1892.
[6] M. von Lenhossek, Der feinere Bau des Nervensystems im Lichte neuster Forschungen. Fortschritte der Medizin, 1892.

mentioned report in „Fortschritte der Medizin“ a good image of the spinal cord of Petromyzon. “The ependymal fibers,” he says in his description, “appear smooth, delicate and are sparse; in contrast, we find multiple glial cells, which are not evenly distributed over the entire cross-section, but rather restricted with their cell body to the stripe-type grey matter. They are characterized by their extensive arborization; these are not secondary, but with few exceptions primary processes reaching to the periphery. Each cell extends a bush of processes at the dorsal and ventral area of the cord, the ones positioned at the side also to the lateral edge of the cord (in contrast to the images by Nansen and Retzius, where they are illustrated with a unilateral extension). The medial branches of the cells at both sides of the midline cross often before and behind the central canal.” His description completely matches with my findings in Petromyzon. With respect to the data provided by Nansen and me for the Myxine spinal cord, it is not correct. Both Nansen (Pl. XI, Fig. 103) and so on, as well as me (Fig. I, S. 51, Biol. Unt. II) have also depicted neuroglial cells with processes projecting not only unilateral towards the periphery of the cord, but also towards the dorsal and ventral area and reach the surface, just like depicted by von Lenhossek for Petromyzon. In the text, Nansen further says: “The same neuroglial cells often send, at the same time, processes to the dorsal side as well as to the ventral side of the spinal cord.” In my text I say with respect to these cells: “Occasionally, fine fibers extend to the opposite direction, as Nansen has shown, and occasionally they reach to the surface like a bundle.” Indeed, there is a difference in that respect between Myxine and Pertomyzon; in Myxine, only a fraction of the neuroglial cells sends processes to both areas, namely to the dorsal and the ventral; a large fraction sends only processes to one side. In Petromyzon, in contrast, the latter is only a rare event: the neuroglial cells send reliably bilateral processes which reach the dorsal and ventral surface.

From Petromyzon fluviatilis, I studied partially larvae (Ammocoetes) of different size (5-20 cm long samples) as well as adult individuals. In the tail region I obtained good staining, with the rapid Golgi method, of neuroglial cell and ganglion cells of the cord. In particular, I obtained good preparations of larval forms from different stages and recently converted individuals. Here I found also a good staining of the elements in the brain. On the properties of the ganglion cells and the nerve fibers I will not focus on, since they are not in the center of this report. In the figures I have depicted a number of ganglion cells, to compare their distinct type with the neuroglial cells. With respect to the structural features of ependyma and neuroglia, I did not find remarkable differences between the investigated larval stages and adult individuals; therefore, I will not provide a particular description.

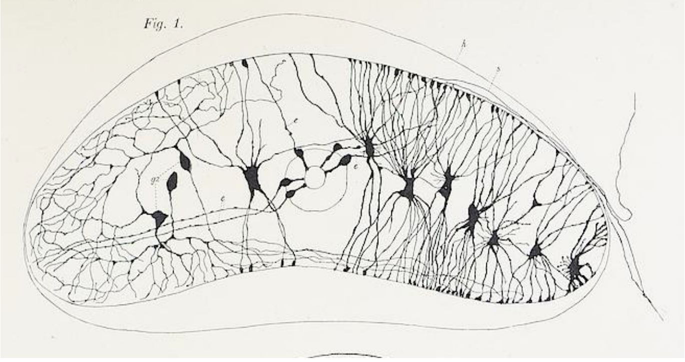

Fig.1 from Table V. Cross-section from the posterior part of the spinal cord of a 4 cm long Petromyzon. At the right from the central canal are seven neuroglial cells and on the left of it is a cell illustrated in a stained mode. Around the central canal are five ependymal cells present; - gz, three ganglion cells with their branched processes; - s, sensible nerve roots; - h; contour of the outer sheath.

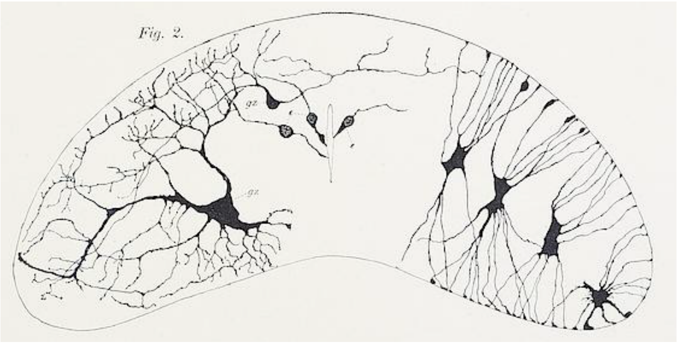

Fig. 2 from Table V. Cross-section from the posterior part of the spinal cord of an adult Petromyzon. On the right are four neuroglial cells, at the central canal three ependymal cells illustrated; gz, two ganglion cells, one small and a large with their branched processes are shown in the left half of the cord.

As emphasized by von Lenhossek, the cell bodies of the neuroglial cells are located in the flattened, dorsally convex, ventrally concave spinal cord of *Petromyzon*, within the inner small band corresponding to the grey matter of the cord. The cell bodies (Table V, Fig. 1-4) are positioned at varying heights, are generally small, and exhibit diverse but typically irregular polygonal shapes.

They give rise to a bundle of processes extending in both directions, dorsal and ventral. These processes are generally stiff, relatively short, and smooth, though they may occasionally appear jagged or varicose. Sometimes they originate individually and directly from the cell body, while at other times they branch off from a thicker extension of the cell body. These processes spread apart to varying degrees and run either stiff or slightly wavy, mostly undivided but occasionally dichotomously branched, toward the dorsal and ventral surfaces of the cord. There, they terminate in knob-like or conical thickenings of variable size.

When several closely apposed neuroglial cells are stained, their processes intersect at acute angles (Fig. 1, 4), creating in cross-sections a peculiar knit-like structure that extends from a node point (cell body) connecting the dorsal and ventral surfaces of the cord. The extension of the processes is not flat and does not occur in a single plane; instead, they extend in various directions, best observed in flat mounts of the cord stained with the Golgi method.

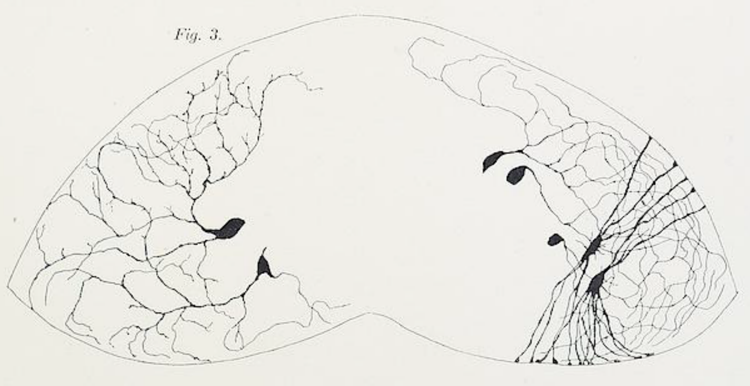

Fig. 3 from Table V. Cross-section from the posterior part of the spinal cord of a 40 cm long Petromyzon. Two neuroglial cells and five ganglion cells are displayed here.

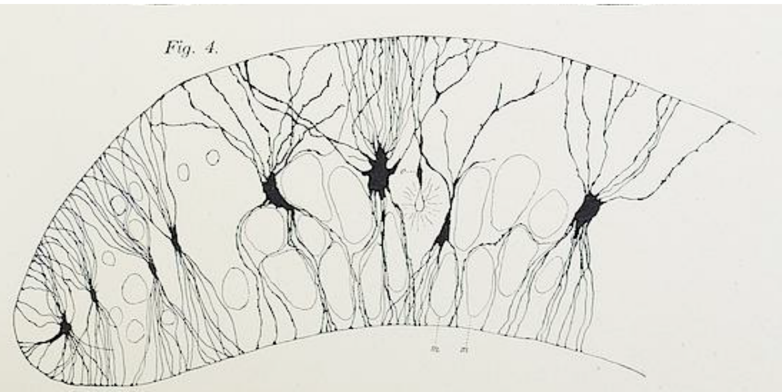

Fig. 4 from Table V. Cross-section from the anterior part of the spinal cord, not far behind the head, of a 14 cm long Ammocoetes. Eight glial cells and an ependymal cell (e) are displayed. A number cross-sectioned Müller fibers of different diameters are shown as contours (m); the processes of the neuroglial cells nuzzle between them.

The form of these glial cells is both characteristic and typical; it is quite variable, as shown in the figures, in terms of the size and shape of the cell body, as well as the number and course of the processes. In the frontal part of the cord, where the thick Müller fibers are present (Table V, Fig. 4), the processes bend into different, partially labyrinthine paths and extend between the fibers to the ventral surface. At the lateral edge of the cord, the processes are short and spread apart, radiating outward like a parasol. They not only arrange in two bundles but also originate laterally from the cell body, reaching both the rounded edge of the cord and the ventral and dorsal surfaces (Table V, Fig. 1, 2, 4).

The ependymal cells of the spinal cord of *Petromyzon* are distinct from neuroglial cells. They show a true embryonic appearance, are not particularly prominent, and stain only sporadically with the Golgi method. Most frequently labeled are the ependymal cells located dorsal to the central canal; their cell bodies and the initial parts of their peripheral processes are most prominently stained. In other cases, ependymal cells projecting laterally become apparent. In such preparations (Table V, Fig. 1, 2, 4c), it becomes evident that the ependymal cells are arranged around the central canal with small, nuclear-labeled cell bodies, as reported by Nansen for *Myxine* and by von Lenhossek for *Petromyzon*. They each send a fine, mainly unbranched, slightly knotted process to the periphery of the cord in a slightly winding course. In general, it is difficult to trace the process to the surface, though success is occasionally achieved. I could not find ciliary attachments projecting into the central canal at the ends of the ependymal cells in the cord.

I have never observed transition or intermediate forms of ependymal and neuroglial cells, not even in *Myxine*. Thus, I do not understand the evidence Nansen presented for the generation of neuroglial cells in the cord from ependymal cells. This relationship appears more evident in higher animals. I do not exclude the possibility that a more detailed investigation into the embryonic development of the spinal cord might provide evidence for such a relationship in Cyclostomes.

To my surprise, I found a distinctly different type of support structure in the medulla oblongata and brain of *Petromyzon*, which I will describe below. I attempted to identify transition forms between the medulla spinalis and medulla oblongata and transitions of the elements. In Fig. 1 of Table VI, I provide a cross-section of the frontal end of the spinal cord. The entire cord exhibits the types described above, except in the most frontal part, where the band-like structure of the cord transitions into a flattened cylindrical structure, and the neuroglial cells exhibit modified features. As shown in the figure, the cell bodies are no longer confined to a single band but are located partially near the central canal and partially

farther outward, even close to the surface. The neuroglial cells in these regions, or on both sides of the central canal, preserve their features characteristic of the cord. However, in response to the altered form of the cord, their processes are much longer to reach the dorsal and ventral surfaces. Farther outward, one finds elements with distinct forms that send their processes only to one side of the cord. Sometimes the cell bodies are close to the surface, while at other times, they are located farther away.

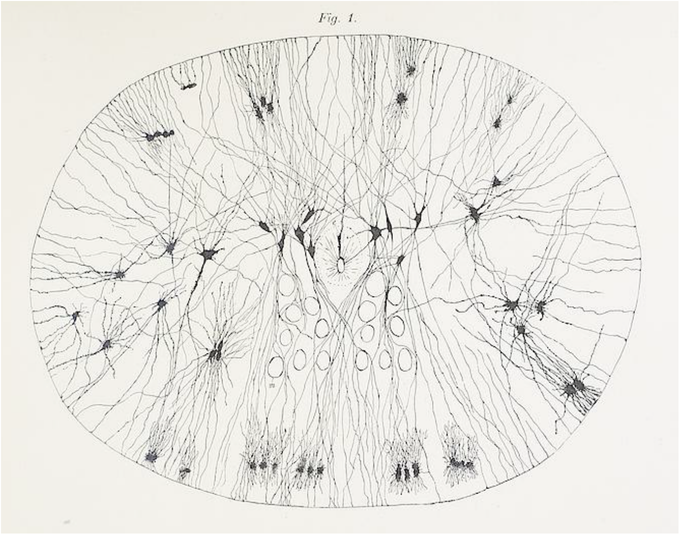

Fig. 1 from Table VI. Cross-section from the anterior part of the spinal cord of a Petromyzon. Different forms of neuroglial cells; - e, an ependymal cell; - - cross sections of Müller fibers.

In general, these neuroglial cells are more reminiscent to the corresponding elements in the white matter of higher animals. With respect to the ependymal cells, they are rarely found; in Fig. 1 of Table VI there is only one such cell shown, it shows the same type as in the other regions of the cord.

A bit more frontal from this area of the cord, at the most frontal end, the described types of glial cells are less abundant; one finds those only in the vicinity of the central canal (Fig. 1, Table VII); here they are arranged concentrically around it; I could not follow the processes to the surface. Yet there appears a different type of support element which is characterized by cell bodies at different distances away from the central canal; these are spindle-shaped, equipped with multiple short branches; a peripheral process originates from them projecting radially and which divides dichotomic; this results in the generation of a dendritic bundle of fine, knotty-jagged and wave like passing fibers, which extend in a conic arrangement to the periphery of the cord

and reach its surface where they terminate with one knot. The true ependymal cells, I did not succeed to stain.

When I studied the medulla oblongata closer, I found, as shown in Fig. 2 of Table VI, only the above-described support cells in this entire part of the brain. Most of them were true ependymal cells; their small, spindle shaped cell body containing a nucleus was found in close vicinity to the central canal; they send a central process to the canal and occasionally even a cilia elongation into the cavity; the other process is oriented radially outward reaching the surface of the cord after multiple dichotomic branching in a wave-form course and terminates in a knob-form. All these peripheral branches are equipped with dense spikes or knots. Between these true ependymal cells are occasionally elements present with a cell body thicker and spindle-shaped, equipped with mossy branches and not associated with the central canal, but rather positioned more outward. These cells fully correspond to those elements illustrated in Fig. 1 Table VII and I show them in Fig. 2 Table VI. In the medulla oblongata I found neuroglial elements characteristic for the spinal cord of cyclostome.

This was also observed in other parts of the brain of *Petromyzon*. Everywhere, I found ependymal elements with peripheral processes branching dichotomously at acute angles and running radially outward. Although I could not trace the branches to the periphery, their overall arrangement suggests such a course. In Fig. 4 of Table VII (c, e), I have depicted some of these cells alongside ganglion cells of varying sizes to illustrate the differences and similarities between the two elements. In Fig. 2 and 3 of Table VII, I depict two areas of the brain where the central parts of the ependymal cells are visible.

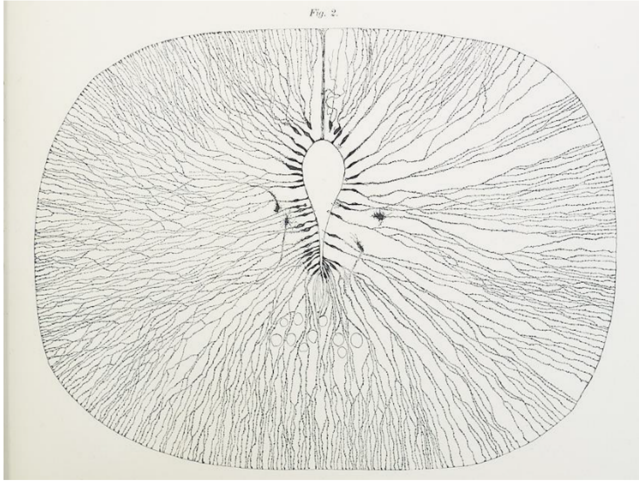

Fig. 2. From Table VI. Cross-section of the medulla oblongata of Petromyzon. Ependymal cell and neuroglial cells.

The peripheral processes with their numerous delicate side branches, and the predominantly bottle-shaped cell body with a central cuticular plate from which ciliary appendages extend, are clearly recognizable. In some cells, only one long cilium is observed; in others, two are present; in still others, three or more are visible, and some cells exhibit a true cilium arrangement. Whether these cilia actually flicker during life is unknown to me. The presence of one or multiple

ciliated cells is of interest, as it relates to ciliated ependymal cells in the central canal of the spinal cord in other vertebrates.

With this report it is evident that there are quite different support tissues in Cyclostomes; the characteristic neuroglial cells in the spinal cord are replaced by types of ependymal cells in the medulla oblongata and in the brain, which is common in reptiles and amphibia. In the brain and medulla oblongata of Petromyzn, I have indeed not found any neuroglial elements which are typical for the spinal cord.

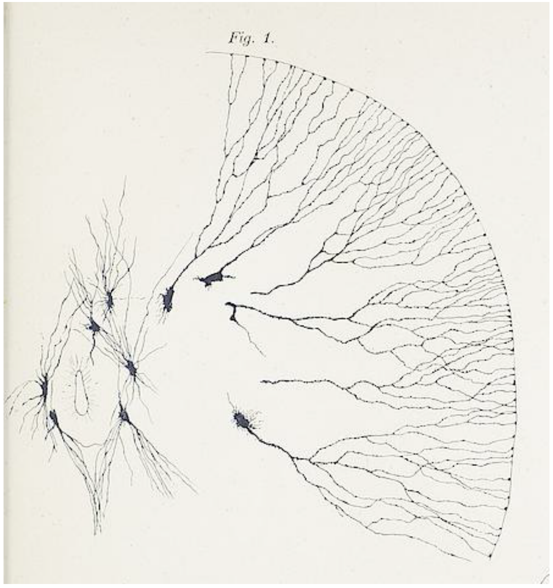

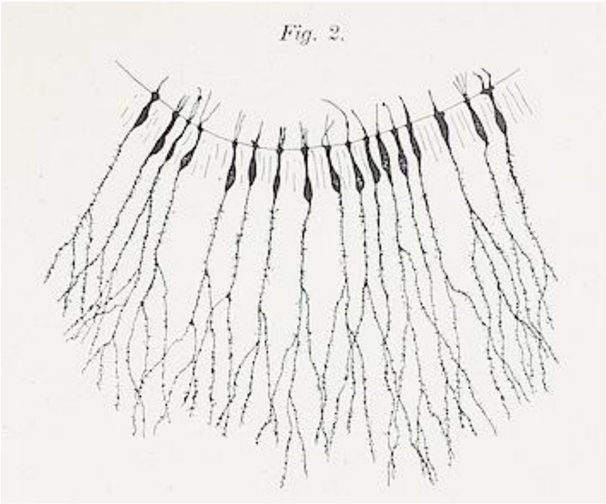

Fig. 1. From Table VII. Antero-lateral part of a cross-section from the most anterior spinal cord part of a young Petromyzon, with the central canal surrounded by spider cells and lateral dendritic neuroglial cells.
Fig. 2 From Table VII. Sections from the ventricular wall of the cerebrum of a 15 cm long Petromyzon with ependymal cells.

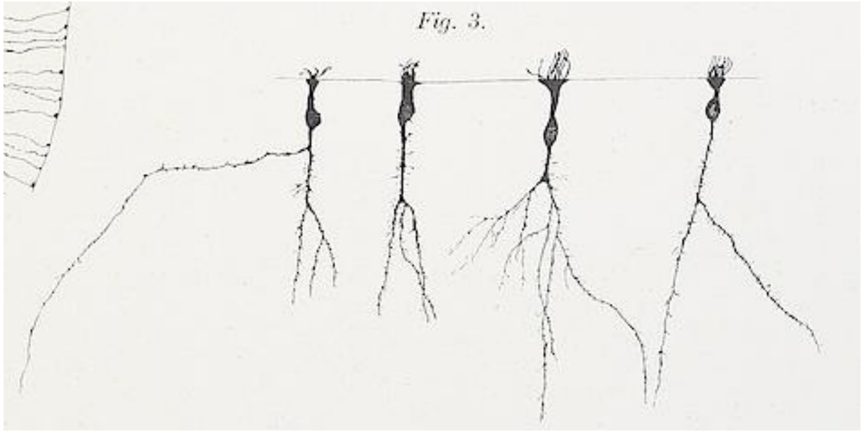

Fig. 3. From Table VII. Sections from the ventricular wall of the cerebrum of a 15 cm long Petromyzon with ependymal cells.

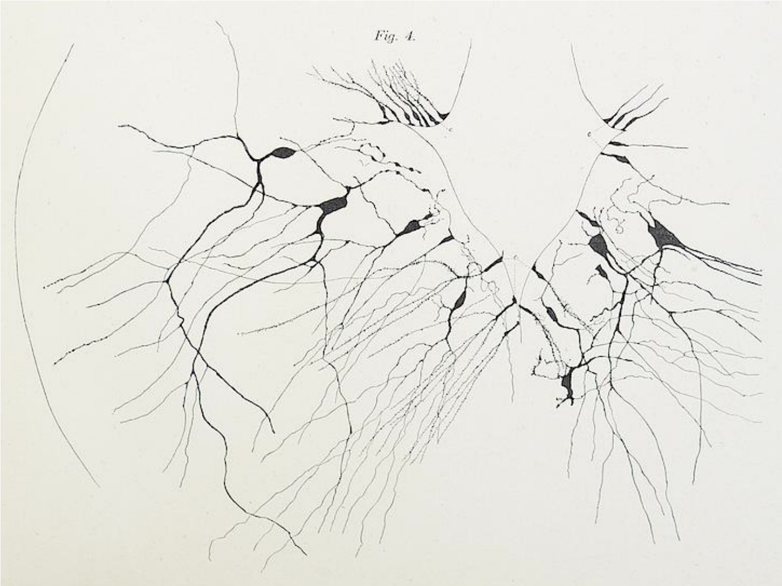

Fig. 4. From Table VII. Cross-section of the brain of a 16 cm long Petromyzon; s, ependymal cells, several ganglion cells of different size are displayed.

2. At the Bony Fish

Table VIII

In the teleostean, the peripheral continuation of the ependymal cells were observed using the earlier methods (Stieda, Mauthner); using the Golgi method, I have observed cells in the medulla oblongata and in ganglia of the cerebrum of pike which radially run from the central cavity to the periphery.

In recent time, this animal class has not been studied with the Golgi method. It was long my intention to fill this gap and even include the cartilaginous fish into these investigations. Experiments which I performed on adult individuals and almost carried out

fetuses of Acanthias [11#] several years ago using the chrome silver method did not provide conclusive results and I did not have access to smaller embryos. Last year, as mentioned above, von Lenhossek published his intriguing results on embryos of *Pristiurus*[12#], establishing the corresponding relationship of cartilaginous fish. I, therefore, focus on bony fish. Since studying embryonic conditions is of primary importance, I obtained a number of eggs and newly hatched specimens of the teleostean *Salmo salar*[13#] from a fish farm in Elfkarleby, Sweden. I kept the fish alive long enough to study them at various stages. Initially, I was unsuccessful in staining them with the Golgi method. After reducing the impregnation period of the chromic osmium mix to one day and applying a double treatment as described by Cajal, I achieved several successful stains of the ependyma and the nervous elements of the spinal cord and brain. Regarding the latter, I will focus in this volume[14#] on the relationship of the support tissue.

I studied a large number of salmons ranging from 16 mm in length (still in the egg) to 40 mm (one month after hatching). The relationships were very similar across all specimens, so I provide only an overview description. In Fig. 1–3 of Table VIII, I provide three cross-sections illustrating the common forms. Within a single slice, only a few ependymal cells are often stained, allowing them to be easily traced in their entirety. When a larger number are stained, as occasionally occurs, distinguishing them from one another becomes more difficult.

In the posterior part of the cord, the features are most primitive; here, the ependymal cells retain the character of true, small cylindrical cells radially arranged from the central canal to the periphery, with no or only a few side branches. Slightly more anteriorly, the type of cells changes, with their peripheral processes developing fine, short, lateral branches. The central end contains the elongated, spindle-shaped nucleus and represents the cell body, which is usually pointed toward the cavity of the central canal. Fig. 1 of Table VIII depicts such a cross-section, showing four ependymal cells alongside four ganglion cells for comparison.

The type of ependymal cell shown in Fig. 1 is predominantly found throughout the spinal cord and represents its common form. From this type, all other forms can be derived, as they are merely variations of it. Fig. 2 and 3 of Table VIII illustrate the common forms and several variations. Most of these cells have a cell body radially positioned against the central canal. The nucleus, which is oval, small spindle-shaped, or sometimes rod-shaped, varies in size, and the size of the cell body varies accordingly. Its form is primarily determined by the nucleus. Occasionally, the body containing the nucleus is positioned adjacent to the canal, exhibiting a triangular shape (Fig. 2 of Table VIII).

The cell body is at its surface smooth or jagged and uneven. The only peripheral process originates from its outer end; it is of variable thickness, but in general quite thick; it extends straight or slightly wavy radially to the surface of the cord. Right after its origin from the cell body it has some rectangular stronger branches which extend to a variable extend; towards the outer region the features of the branches changes in that they become fine, knotty or even mossy and are densely packed. This results in a peculiar appearance of the peripheral processes which is illustrated in the attached figures in several variations. The number of the lateral branches varies considerably; in some cases, it can be quite low (on the lower left in Fig. 3 of Table VIII), in others the branches occur in large quantities, even beard-shaped as shown in some ependymal cells in the same figure. As I mentioned above, the peripheral process is usually undivided except for some fine branches; it may occur that he can have stronger lateral branches,

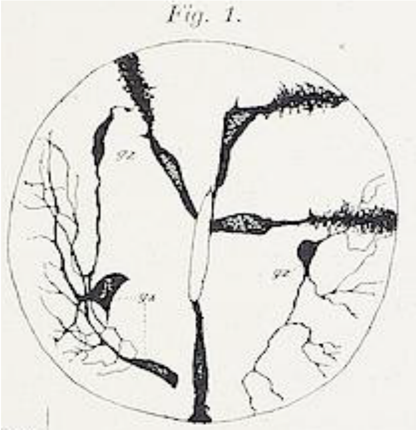

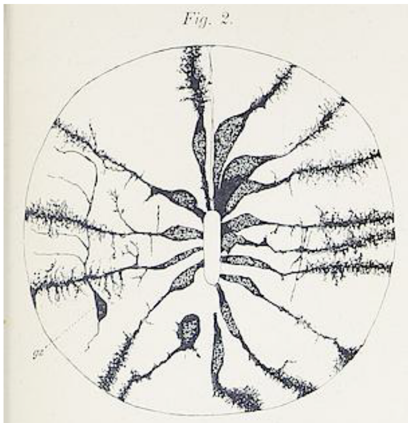

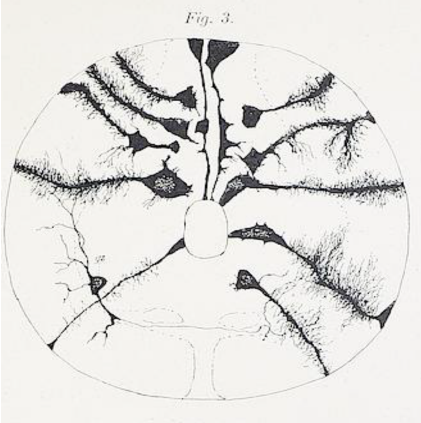

Fig. 1-3 from Table VIII. Cross-sections of the spinal cord of young, 16 cm long salmons. Fig. 2 and 3 from the middle, Fig. 1 from the posterior part of the cord. Ependymal cells; - gz ganglion cells.

and in single cases it can divide into 2-3 main branches, which run separately to the periphery. In the table below, which shows a nerve cell, a divided ependymal cell is also depicted. In the medial section, toward the front and rear, different relationships are observed. The ependymal cells often exhibit a bent form; the cell body is sagittal, and the peripheral process bends outward at a right angle (Fig. 1, 2 of Table VIII). In the posterior midline, the arrangement of the ependymal cells becomes even more peculiar. Many cell bodies do not reach the central canal but are instead positioned more or less upright against the septum posticum. It is evident that the former posterior cleft of the central canal has closed here, as in other cases; the ependymal cells and support cells, which have transformed into true neuroglial cells, remain oriented toward the septum. In the midline of the septum, long, slim, sparsely branched cells are found (Fig. 3 of Table VIII and below in Table XIX, over the nerve cells of the cord), and their nucleus is unusually positioned at the outer periphery. Most likely, their central process originates from behind in the septum and, after the closure of the canal's cleft, extends toward the front.

As shown in the figures, the support cells of the spinal cord described here form a highly contrasting, bushy cellular element with a characteristic shape distinct from ganglion cells. Internal hairy attachments or cilia with intrude into the canal, I have not observed in the young salmons. In older animals of Salmo, Gobius[15#], Gastrerosteus[16#], Anguilla[17#] I found ependymal cells of the same type; only their peripheral process showed a stronger, repetitive, even bushy ramification. I have not found other elements in this support system,. In the medulla oblongata and in the brain, the type of ependymal cell changes in that they become much smaller and slimmer and have much less branches; here and there divides the peripheral process on the way to its surface, and this occurs repetitively dichotomic.

In Fig. 4 a, b of Table VIII I have illustrated a part of a cross section of the cerebrum; the true ependymal cells can be easily recognized; in contrast it is difficult to recognize which of the cells can be considered as ependymal cell or ganglion cell if the cell body does not reach the brain ventricle.

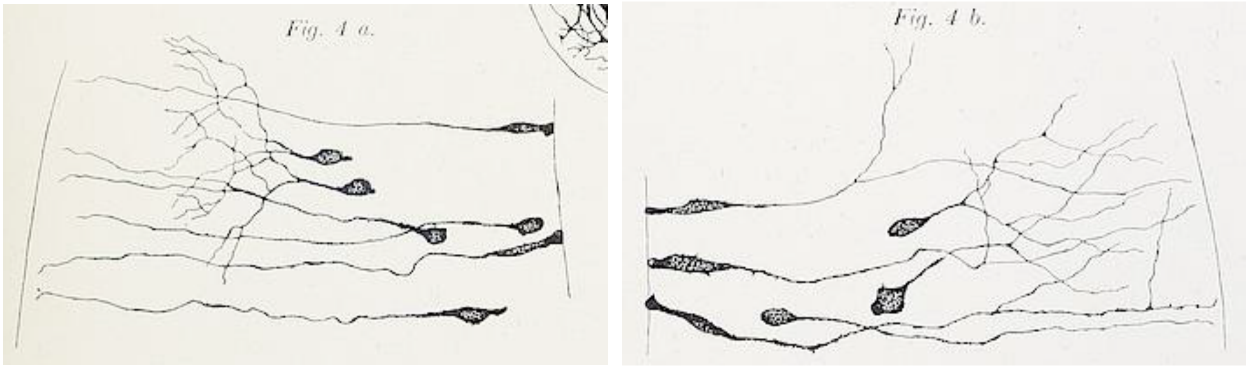

Fig 4a and 4b from Table VIII. The two lateral parts of a cross-section of the cerebrum of a young salmon. Ependymal cells and neuroglial cells.

3. At the Amphibia

Table IX.

Ependyma and neuroglia in amphibia had been several times the object of investigations. Already Reissner observed processes of ependymal cells extending outward. Using the Golgi method, Oyarzun, me, Ramon y Cajal and Cl. Sala have studied that topic.

This time, I have mainly studied larvae of Salamandra maculate[18#] and Rana temporaria[19#] and young individuals of these animals. My studies are so well confirming previous ones that I feel that it is not necessary to describe it. I refer therefore to the figures (Table IX). Fig. 1 shows a cross-section of the spinal cord from 2.5 cm long larvae of a salamander; the ependymal - respective neuroglial cells (that are those cells which have their cell bodies at the outside of the central canal) show the known type with a fascicle-shaped peripheral process which often branches already in the grey matter; the branches show a radial outward course; as a comparison I depicted some ganglion cells. Fig. 2 shows the lateral part of the medulla oblongata of 2.5 cm long larvae of a salamander; the type of ependymal cell is the same as in the spinal cord, only that the conic shaped branching occurs earlier. In Fig. 3 depicts a part of the cerebral cortex; the ependymal cells show the shape as shown by Oyarzun, me and Ramon y Cajal in amphibia.

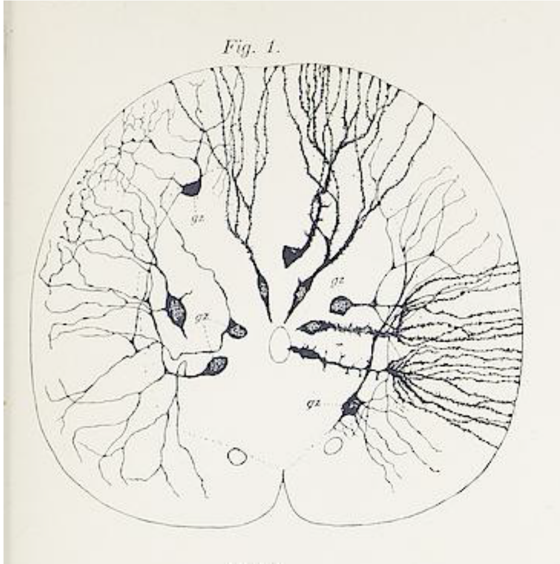

Fig. 1-3. From Table IX. Cross-sections from the spinal cord and brain of larvae from Salamandra maculate; - Fig. 1. Cross-section of the spinal cord with ependyma-, resp. neuroglial cells; gz - ganglion cells.

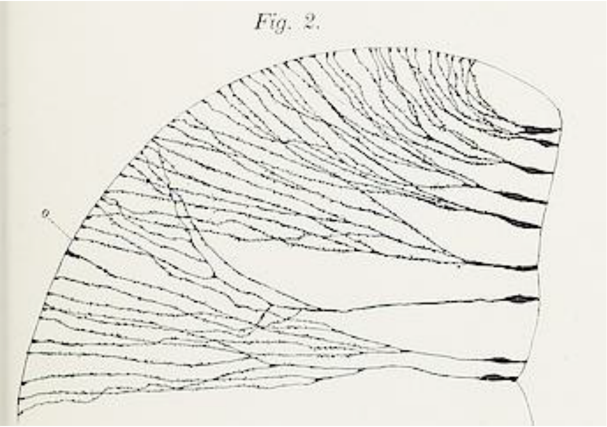

Fig. 2. From Table IX. Lateral section from a cross-section of the medulla oblongata.

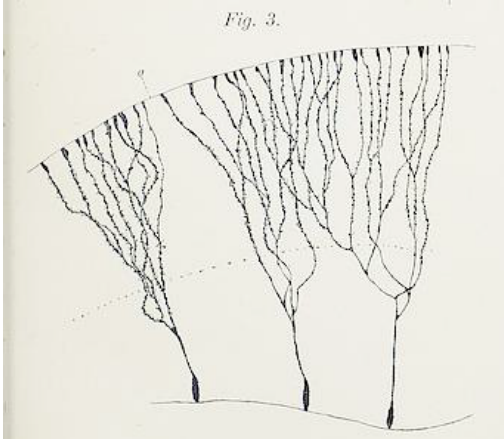

Fig. 3. From Table IX. Part from a cross-section of the cerebrum; ependymal cells;
o – surface of the brain.

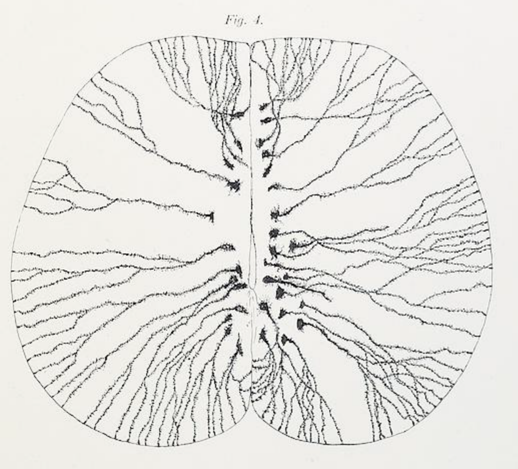

Fig. 4-7 From Table IX. Cross-sections from spinal cord and brain of a young frog (R. temp.);
Fig. 4. Cross section of spinal cord; radial neuroglial cells.

Fig. 4 depicts the cross-section of the spinal cord from a young, fully metamorphosed *Rana*. The arrangement and properties of the cells are consistent with those observed in salamander larvae, as well as the detailed figure provided by C. Sala from the spinal cord of an adult frog. The properties of the neuroglial cells in the frontal fissure and the septum posticum are noteworthy.

Fig. 5 illustrates the form of the ependymal cells in a cross-section of the medulla oblongata, where the same cell type is present. In Fig. 7, the upper-medial part of a cross-section of the hemisphere of the cerebral cortex (basal forebrain) of a young frog is shown. The type of ependymal cell is the same, except that branching occurs slightly farther outward. For comparison, several ganglion cells are included in this figure.

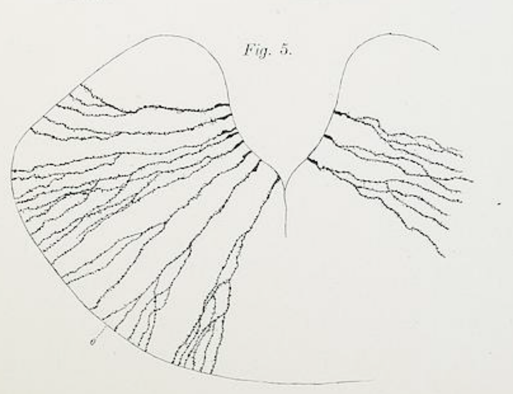

Fig. 5. Cross-section of the medulla oblongata; ependymal cells.

Fig. 6 illustrates a part of the midbrain of a young frog. Here, the ependymal cells are of a different type, extending a long peripheral process almost straight outward without dichotomous division, while sending numerous small, mossy branches in all directions, particularly at the outer end. Several ganglion cells in different arrangements are also included in this figure.

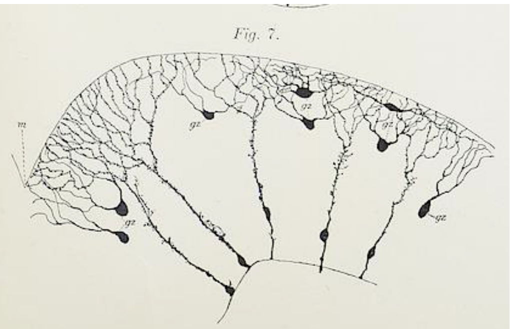

Fig. 7. Cross section of the cerebrum; m, midline, gz, ganglion cells; five ependymal cells.

In general, there is the same type of ependymal, respective neuroglial cell in amphibia in spinal cord, medulla oblongata and frontal brain; the peripheral process is equipped towards the outer end with conic-formed multiple branching: it is the same type which is found in the medulla oblongata and in the brain of Petromyzon. Other neuroglial cells beyond the ones described above, I have not found in amphibia.

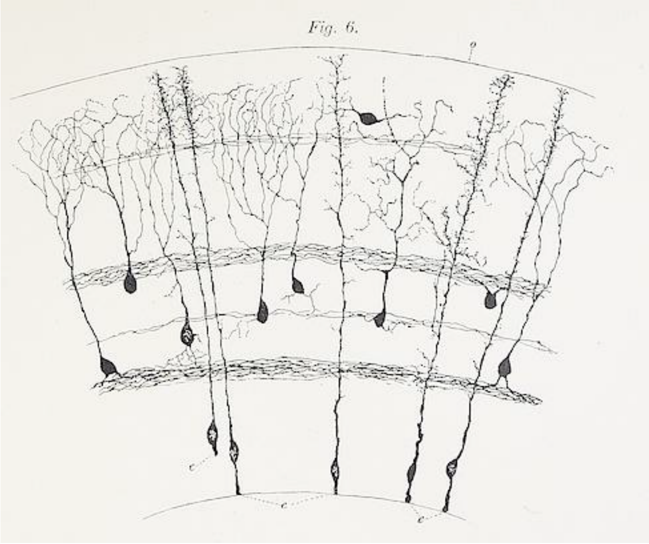

Fig. 6. Section of the mesencephalon; e, ependymal cells.

4. At Birds

For the study of the ependyma and the neuroglia of reptiles, I did not have proper material; by the excellent work of Ramon y Cajal and Pedro Ramon one knows essentially all important issues. With respect to the birds, there is conclusive evidence provided in particular by the work of Golgi, Ramon y Cajal and v. Lenhossek and it is not worthwhile to go into more details, in particular since my findings match those of the mentioned researchers.

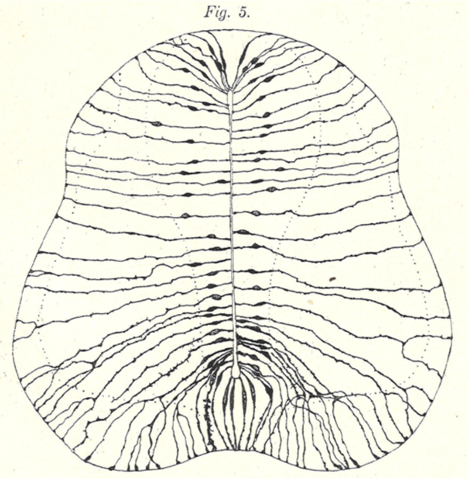

Fig. 5 – 8 from Table VIII. Cross-sections of the spinal cord and brain of a chicken embryo;
Fig. 5 cross-section of the lumbar spinal cord of an 8.day old chicken embryo, ependymal cells.

Out of my many preparations from the spinal cord of chicken embryos, I provide only one picture (Fig. 5 of Table VIII); it shows the cross-section of an eight-day old embryo. One may wonder that the cord at that stage is in such a primitive condition, being only little further developed as of a 4-day old embryo as shown by v. Lenhossek. This may be explained due to the fact that the distal part of the of the cord develops later as the other parts, as I have found, and I will deal with that in more detail in one of the following chapters. As shown in Fig. 5, the central canal represents a long, sagittal cleft and the anterior and posterior ependymal wedge is very short and primitive. The support cells extend all from the central canal to the surface; only those positioned at the frontal and antero-lateral circumference are branched at

their outer ends; none of the true neuroglial cells are visible; the white matter forms a thin coat layer (indicated by a dotted line in the figure) which is lacking in the front and at the end. In older stages of the cord the neuroglial cells become present as described by Golgi, Cajal and v. Lenhossek and also by Lachi. In Fig. 6 of Table VIII, I have depicted some of those cells (from a 14-day old embryo) in the known developmental stage; the cell bodies are outside the central canal, but at a distance, sending a peripheral process to the surface like true ependymal cells. As comparison, I have depicted some ependymal cells from a 17-day old embryo which show no branching of the peripheral process. I present these fragments only as examples.

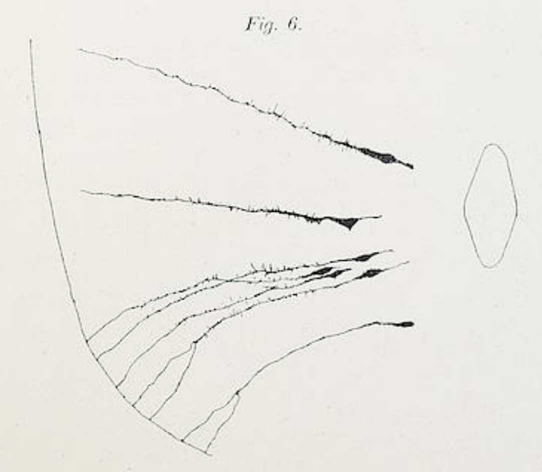

Fig. 6. Section from the frontal end of the spinal cord of a 14-day old chicken embryo; radial neuroglial cells.

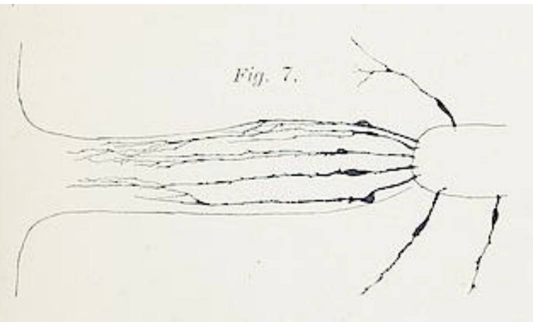

Fig. 7. Septum posticum at the transition section from the spinal cord into the med. oblongata of an 8-day old chicken embryo.

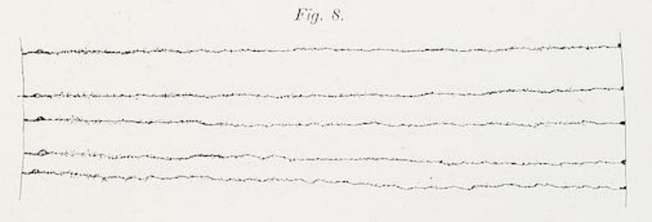

Fig. 8. Section from a cross-section of the cerebrum of a 17-day old chicken embryo.

5. At Mammals

Table X.

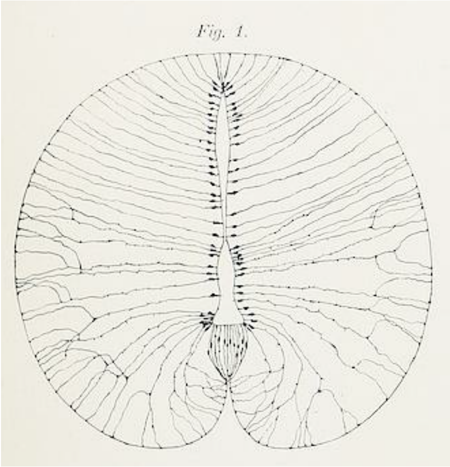

Fig. 1 from Table X. Cross-section from the spinal cord of a 3 cm long cat embryo. Ependymal cells.

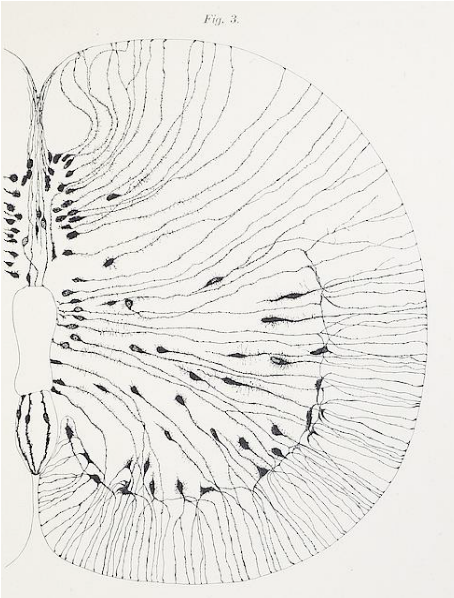

Fig. 3 *from Table X.* Cross-section from the spinal cord (neck region) of a 12 cm long dog embryo. Ependymal and neuroglial cells.

I have studied in this relationship in mouse, rat, cat, dog and human, and in particular the embryonic and young spinal cord and the cerebrum.

In the spinal cord of the studied mammals, the ependyma and the neuroglia are more or less very similar and it is indeed not necessary to present a special illustration for each of these animals.

The earliest stage where I accomplished a good staining of the support system in the spinal cord of mammals was in a 27[20#] mm long cat embryo. Already two years ago, I have provided a figure and a description of that stage that is very close to it (from a 30[20#] mm long embryo) and add to the figures at Table X a figures of this (Fig. 1). One can see the long, slit-shaped cross section of the central canal, the anterior and posterior ependymal wedge and the ependymal cells penetrating radially through the entire lateral fields; their peripheral processes branch at the frontal and antero-lateral regions at the outer edge to terminate at the surface by knotty thickenings. The hook-shaped bending of the outer ends can be found in different preparations as described by me earlier, seems however not a constant formation; it may perhaps be due to the shrinking of the surface due to fixation. There cannot yet any neuroglial cell be detected; no doubt there are some radial neuroglial cells present in the neck part of the cord. From the following stages, I have received the best images from dog embryos. In Fig. 3, I provide a half of a cross-section from the neck region of a 12 cm long dog embryo. Th image resembles to a certain extent to the one recently provided by von Lenhossek from a 14 cm long human embryo (Fortschritte d. Medizin, 1892, Table I). One can see, among other features, the anterior and posterior ependymal wedge, while the latter has protruded far ventrally, while at the same time the dorsal slit of the central canal has disappeared.

The remaining area of the slice is partially occupied by true ependymal cells, which span from the central canal to the surface, and partially by other cells whose cell bodies are positioned at varying distances from the central canal. These cells also send a peripheral process, similar to the ependymal cells, to the surface, where it terminates in a similar manner.

In the anterolateral region, the processes usually divide as soon as they enter the longitudinal strands or shortly before. The branches often bend parallel to the surface for a distance before projecting radially outward again, thus generating a tangentially running stripe at the surface along the border of the strand coat, which encompasses only the anterolateral region. Within this stripe, there are additionally oddly shaped cells whose elongated bodies are generally positioned parallel to the longitudinal axis of the cord surface. From both ends of these cell bodies originates a single process, which curves outward in an arc, generally dividing dichotomously before extending through the strand coat to the surface, where it terminates in a knob-like form.

The cell bodies of all these cells are generally equipped with short, fine processes, which give them a jagged and mossy appearance. Comparing all these cells reveals that they are very similar. They clearly represent a modification of the same cell type, reflecting different stages of development, and are essentially identical.

If the term "ependymal cell" is applied, as is customary, to all cells with a nucleus-containing cell body at the central canal that send a peripheral process to the surface, then the term "neuroglia" can confidently be applied to all other support cells, as done by Cajal, von Lenhossek, von Kölliker, and others. It should be emphasized that these two terms do not refer to fundamentally different cell types but rather to modifications of the same support elements. At this developmental stage, numerous transitional forms between these two cell types are present, leaving no doubt, upon close inspection, of their shared origin.

Among the radial neuroglial cells, there are individuals here and there with cell bodies positioned almost at the central canal, displaying a striking similarity to ependymal cells. Among the outer neuroglial cells, whose cell bodies are located at the inner border of the strand region, many project shorter or longer processes toward the interior. No cell bodies with nuclei are present in the strand region itself.

As von Lenhossek emphasized, the glial cells near the frontal fissure bend their processes toward it; these cells, in particular, are highly branched. In the posterior region of the cross-section, deviating features are observed, as described in detail by von Lenhossek. No true ependymal cells are present, only radial neuroglial cells with oval or spindle-shaped cell bodies equipped with fine, mossy processes. These cells are positioned perpendicular or slightly inclined to the sagittal midline, the *septum posticum autorum*, at variable distances from it. Toward the outside, they project their peripheral processes, which curve in a bow-like shape toward the back and always reach the surface, terminating unbranched in a knob-like form. The most posterior cells are even S-shaped, as extensively and detailedly described by von Lenhossek for humans.

As a result of the obliteration of the posterior cleft of the central canal, the original true ependymal cells disappear, leaving only the now radial neuroglial cells. Their arrangement, perpendicular to the septum, indicates their origin. Moreover, von Lenhossek demonstrated that the so-called septum is not of pial nature but belongs to the cord substance proper. It consists of ependymal cells, forming the posterior ependymal wedge. These cells move ventrally with the fusion of the dorsal cleft, contributing significantly to its closure and obliteration. This explanation is clearly correct and can be easily confirmed in mammalian and human embryos.

Regarding the cells of the septum, von Lenhossek’s description for humans also applies to mammals. At the dorsal end of the central canal, the cell ends are densely packed from both sides. Behind this point, they bend outward on both sides, while only those at the midline remain positioned sagittally. Farther laterally, the cells bend inward, and at the dorsal part of the septum, they form a narrow, compact bundle extending toward the shallow dorsal fissure.

The ependymal cells at the anterior ependymal wedge are short but fairly thick and generally unbranched, with jagged edges and barrel-like bends at the lateral parts, while remaining straight in the center. At the bottom of the ventral fissure, they usually terminate with very small knobs.

Support elements of a different type as just described, I have not found at this stage in the spinal cord of the dog.

In Fig. 4 of Table X, I have provided an illustration of a large part of a cross-section from the lumbar region of the same 14 cm long dog embryo. In general, there are concordant relations present so that I can abstain from a detailed description. Further below in the lumbar region was the arrangement and properties much more primitive (Fig. 2 of Table X) by being only ependymal cells present; no neuroglial cells were generated; the dorsal cleft was not fused.

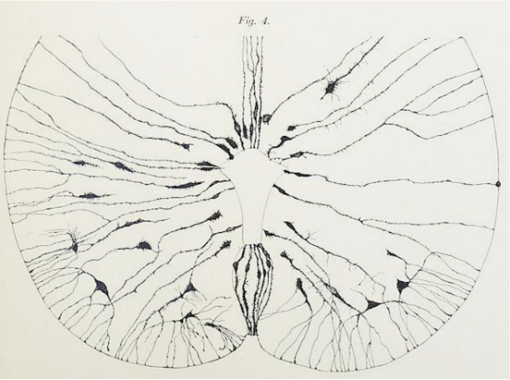

Fig. 4 *from Table X*. Cross-section from the spinal cord (lumbar region) of a 12 cm long dog embryo. Ependymal and neuroglial cells.

It is not my intention to further follow the development of the support elements of the spinal cord of mammals, since its presentation by too many details would be too much broadened and a lot of Figures would be necessary. The data presented here is sufficient to emphasize the most important issues in correspondence with von Lenhossek´s presentation. I will come back to this topic when describing this relationship in human.

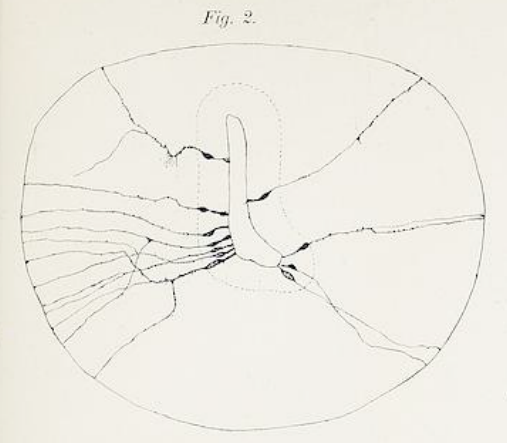

Fig. 2. from Table X. Cross-section from the posterior end of the lumbar cord a 12 cm long dog embryo. Ependymal cells.

With respect to the ependyma and the neuroglia of the brain, I have already earlier (Verhandl. d. Biol. Vereins in Stockholm, Bd 3, 1891) occasionally touched this issue and depicted the major forms of both cell types in the cerebral cortex of the young dog and also the Bergmann fiber cells of the cerebellar cortex of human and others; in the report quoted above, I have added to the figures some typical ependymal and neuroglial cells. These cell types have been long before described and illustrated by other researchers

such as Golgi, Martinotti, Cajal, von Kölliker, Van Gehuchten and others. A detailed description of their entire developmental history would be still of particular interest; it is yet now not my intention to deal with that issue.

6. At Human

Table XI - XIII

i

From my younger human embryos, I have only received once such fresh material that it could be stained with the Golgi method. It was from a 3 cm long embryo and in its spinal cord ependyma and neuroglia and also ganglion cells and collaterals were nicely stained here and there. The closure of the dorsal cleft of the central canal was differently far progressed in the different regions of the cord. In Fig. 1 of Table XII, I have depicted a stage, where this closure has already far progressed; in Fig. 2 of Table XI, is another stage shown where the cleft projects further dorsal and the so-called septum is shorter. In the Fig. 1, ependymal cells are distributed over the entire field which are positioned perpendicular against the cavity of the central canal, respective also to the fused dorsal cleft and from there they project their outer processes radially to the periphery; at the ventral parts they bend slightly ventrally as von Lenhossek has emphasized and as it is also the case for other mammals (dog etc.); only in the border region between the dorsal and ventral parts the ependymal fibers project straight lateral. The cell bodies of the ependymal cells are generally small and are larger only in the region where the nucleus is present; from the inner free end one can observe only here and there a hairy, often bend attachment projecting into the central canal; I mention this because von Lenhossek has such hairs only found in much later stages; the peripheral process runs in a wave-form outwards; it is still smooth and only little knobby or jagged. In the antero-lateral region it divides at the border of the strands dichotomic and the two branches can divide further; they extend from each other in a more or less large angle and bend outward to terminate at the surface with knob-like or conic thickenings. The processes of the ependymal cells positioned at both sides of the ventral ependymal wedge bend medial against the ventral fissure and divide extensively as von Lenhossek has emphasized. The ependymal cells located on both sides of the dorsal cleft of the central canal reach the cavity with their central ends when the cleft is open (Fig. 2 of Table XI). After its closure, only a few cells reach it (Fig. 1 of the same Table).

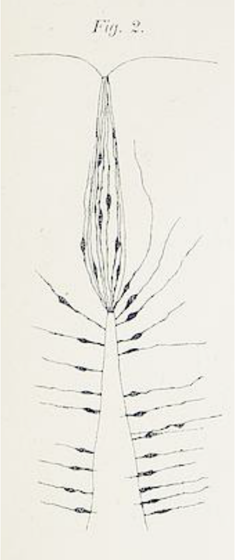

Fig. 2 from Table XI. Septum posticum and dorsal part of the central canal from spinal cord of a 3 cm long human embryo. Ependymal cells.

Fig. 1.

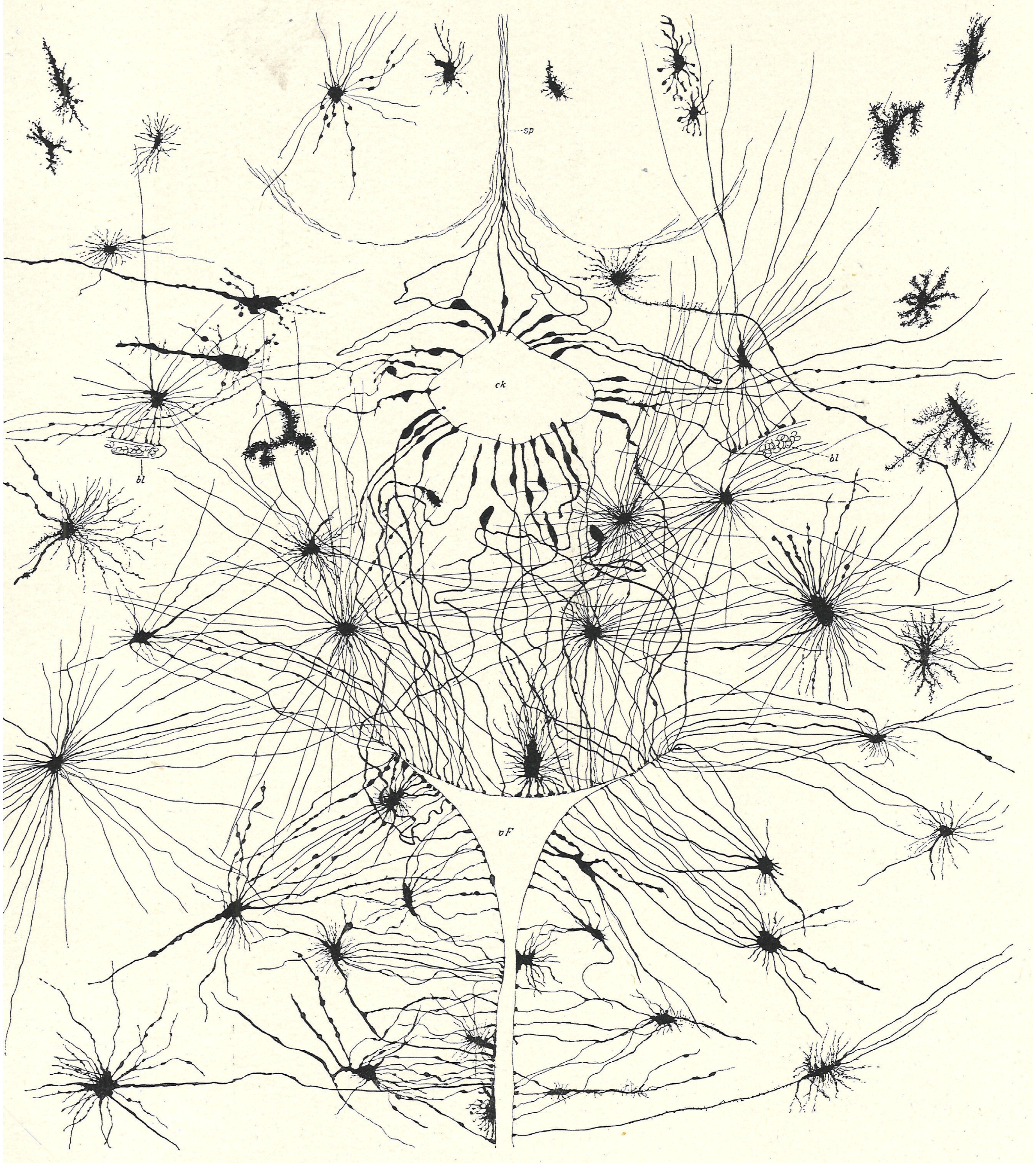

Fig. 1 from Table XII. Middle section from the cervical spinal cord of a 25 cm long human fetus.
Ck – central canal
sp – septum posticum.
vF – frontal fissure
bl – blood vessels
Around the central canal one can see ependymal cells and outside from it, neuroglial cells of different types (spider cells, mossy cells etc.).

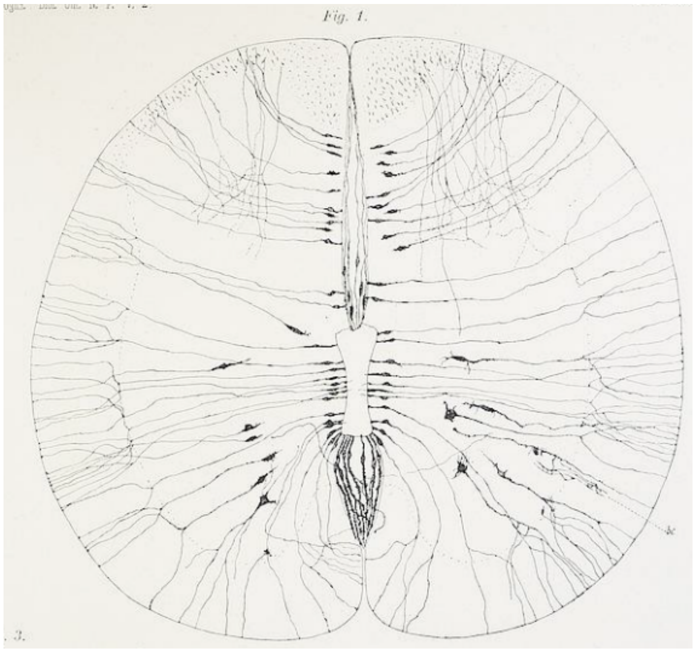

Fig. 1 from Table XI. Cross-section of the spinal cord (lumbar region) of a 3 cm long human embryo. Ependymal and neuroglial cells. The dotted line indicates the border between white matter and grey matter. Some collaterals from the dorsal roots are displayed; bl, blood vessel.

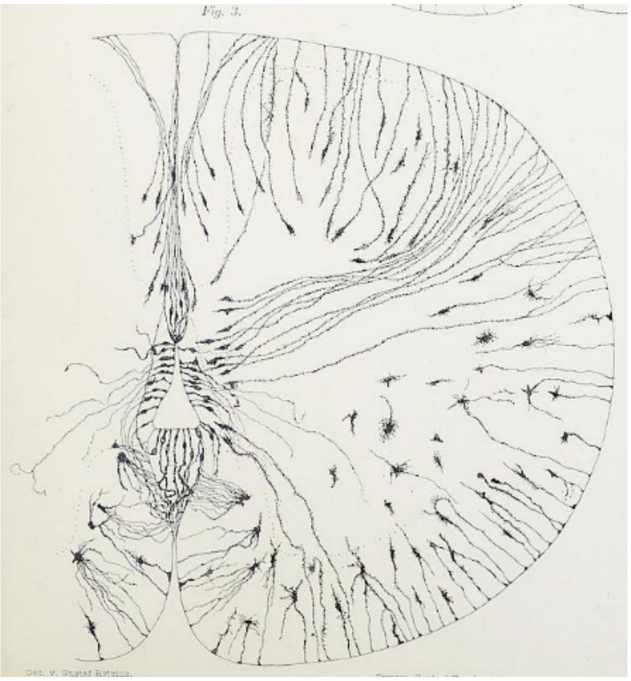

Fig. 3 from Table XI. Cross section from the spinal cord of a 15 cm long human embryo (neck region). Ependymal and neuroglial cells.

Cells are now present that can be considered neuroglial cells, with spindle-shaped, mossy cell bodies and generally unbranched processes on both sides of the septum, which they do not reach. The processes of these cells are intersected by the collaterals of the dorsal roots (Fig. 1 of Table XI). Neuroglial cells of this type are also present in the frontal part of the spinal cord. I have not encountered neuroglial cells of a different type at this stage. No cell bodies were found in the strand region (of the white matter). The two ependymal wedges have features as described above in dog and previously in detail by v. Lenhossek. With respect to the dorsal wedge is a comparison between Fig. 1 and Fig. 2 of Table XI quite insight-full while the latter figure shows an earlier, the figure 1, a later stage. The ventral wedge shows typical features; I will only mention that within its single fibers are dividing. From the following stages of development, I have chosen the spinal cord of a 15 cm long human embryo. This stage (Fig. 3 of Table XI) shows a higher developmental stage as illustrated by von Lenhossek on a 14cm long embryo. In particular, it should be emphasized that the entire lateral ependyma is behind in its development and the neuroglia is well developed. The cell bodies of the ependymal cells have mainly retained their features; however, their peripheral processes show an altered appearance; they sometimes coarsely granulated, sometimes extremely fine and no longer regularly radial, but have a bay-like course and soon disappear; the cells close to the ventral ependymal edge can be followed

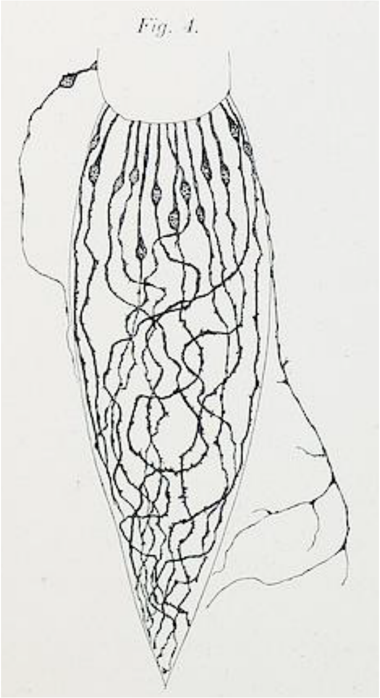

Fig. 4 from Table XI. Ventral ependymal wedge from spinal cord (neck region) of a 15 cm long human embryo.

to the ventral fissure despite of their fine caliber; the ones positioned more lateral project into different directions and often turn medial whereafter they appear to disappear; I could never follow these fibers up to the strand substance, but they ended usually close to the central canal. The two ependymal wedges show the properties described by von Lenhossek and others; in the ventral wedge, the peripheral thick and fine, ridged processes often bend in several big bows, as shown clearly at higher magnification in Fig. 4 of Table XI. The medial bending of the ependymal cell central ends at the dorsal wedge, as described by von Lenhossek, is clearly indicated in Fig. 3, as is the subsequent enlargement and then the brush-like constriction of the posterior parts of the wedge.

Long radial neuroglial cells are frequently present anterior and particularly posterior and show the features and arrangement as described above in the dog embryo and the human embryo as described by von Lenhossek. Their course before, behind and within the dorsal horn, respective in the Substantia gelatinosa Rolandi is from the mentioned researcher so well described, that I only will refer to his presentation.

With respect to the other neuroglial cells, one finds as mentioned above, a quite advanced development in the antero-lateral region both in the grey as well as in the white matter. The cells next to the ventral fissure already possess their later properties; the cell bodies are irregular and show only exceptionally a central process, while several peripheral processes project to the surface where they end in thickenings. In the remaining antero-lateral region there are also numerous neuroglial cells in the white matter (strand substance) with different forms; most of them are simple, little branched; in Fig. 3 of Table XI, I have displayed a number of the common forms and thus I refer to this Figure. The neuroglial cells of the grey matter are commonly fairly small and equipped with mossy and ragged branches; here and there one finds the still existing peripheral process, sometimes even a short central.

Of the following stages, I selected the spinal cord of a 26 cm long human embryo, since I obtained a nice staining of the neuroglial elements. On table XII and XIII, I have provided illustrations of regions in cross- and longitudinal sections of the spinal cord. I think that it is not necessary to provide a detailed description of these relationship, since I can confirm all the essential issues which von Lenhossek has provided. Yet I would like to emphasize some points, in which

my views diverge from his. With respect to the ependymal cells, it is my opinion as already in the 15 cm long embryo that they are in large parts atrophied. In ventral and dorsal direction, they are much maintained; a ventral wedge figure can no longer be found; star-shaped Deiters or spider cells are present here; nerve fibers or ganglion cell processes are widely protruded through the frontal commissure and the ependymal cells are to a certain extend pushed apart; yet they can be largely followed up to the fissure; most of them get here in labyrinthine courses; their central emerging hair process can often nicely be seen.

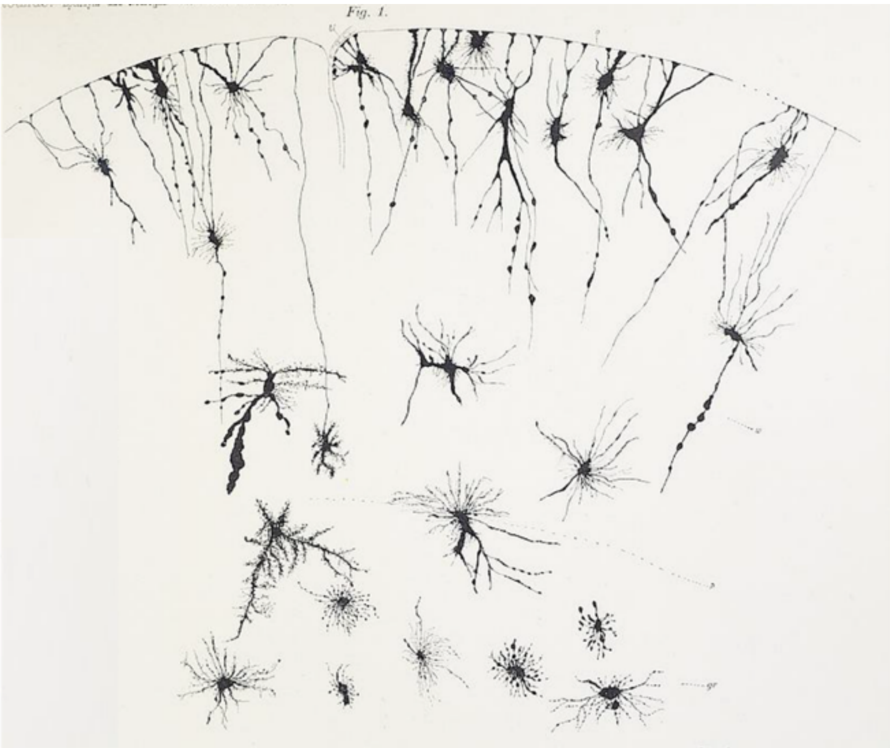

Fig. 1 from Table XIII. Region from a cross-section of the neck cord of a 25 cm long human fetus. Antero-lateral region. Neuroglial cells of different forms.
o – surface of the cord
p – border of the grey substance
w - white matter
gr grey matter

The ependymal cells of the dorsal ependymal wedge often bend their peripheral processes strongly in a lateral direction before entering the slim septum and projecting toward the back in an almost straight or slightly wavy course. The lateral ependymal cells are difficult to stain; their peripheral processes are usually fine, varied, and irregular in their course. They often bend and disappear from view. Von Lenhossek expressed the opinion that these fibers do not disappear. He assumed that what remains at this advanced stage would persist later. I cannot prove that the ependymal fibers degenerate and disappear completely, but they not only become sparse but also more irregular in their course, often bending medially before becoming invisible. However, all researchers agree that these fibers no longer play an important role and should be considered embryonic rudiments.

At this stage, the neuroglial cells have reached a high degree of development. In the vicinity of the central canal (Table XII, Fig. 1) the Deiters or spider cells are present in nice formation; their processes can be followed over large distances. In the grey matter (Table XIII, Figure1).

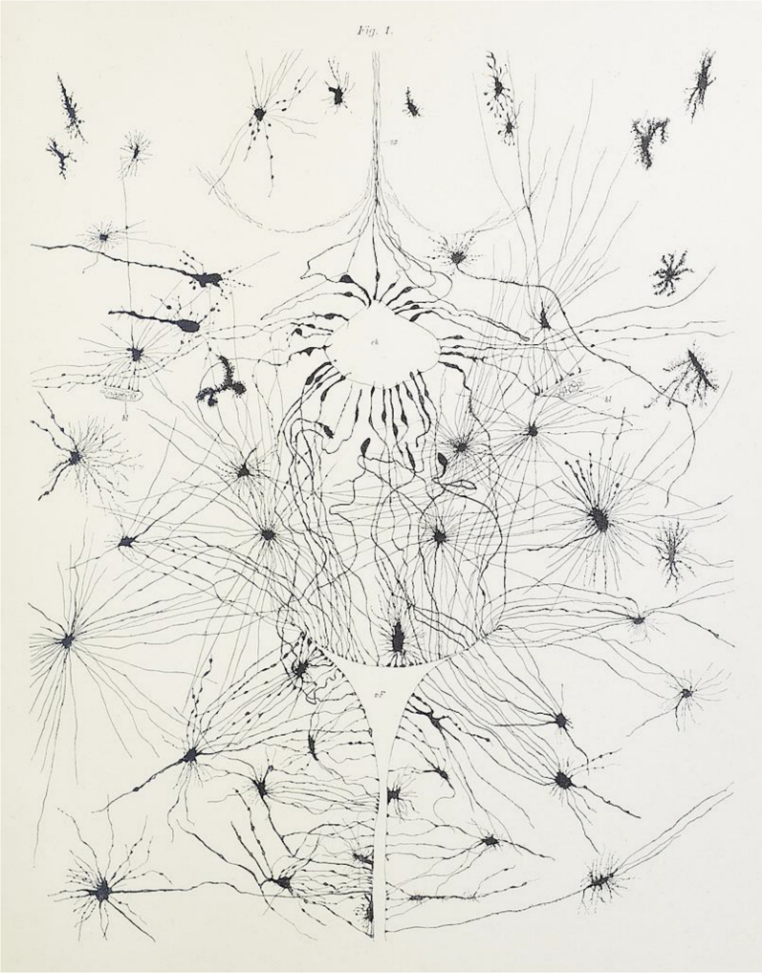

Fig. 1 from Table XII. Middle section from the cervical spinal cord of a 25 cm long human fetus.
Ck – central canal
sp – septum posticum.
vF – frontal fissure
bl – blood vessels
Around the central canal one can see ependymal cells and outside from it, neuroglial cells of different types (spider cells, mossy cells etc.).

A large number of scattered cells with diverse forms can be observed; they commonly have many fine processes, giving the cells a mossy appearance. The processes are often varied, sometimes equipped with thick, clumsy knots or numerous side processes. Occasionally, the processes can be seen attached to closely apposed blood vessels, as previously described by Golgi (Table XII, Fig. 1 at bl, showing spider cells behaving in this manner).

At this stage, I often fail to observe a peripheral process projecting to the surface in these neuroglial cells of the grey substance. The cells in the *Substantia gelatinosa Rolandi* are commonly elongated and mossy, as described by von Lenhossek. Additionally, many short and thick cells exhibit variable forms (Table XIII, Fig. 3). The neuroglial cells of the white matter (Table XIII, Fig. 1w and Fig. 2) also exhibit various forms, as described by von Lenhossek. Providing a general description is nearly impossible, so I refer to the attached figures.

In general, the cell body is small, irregularly angled, and equipped with dense, fine processes extending in all directions. Specifically, one or more thick, elongated, and stiff processes project centrally, while one, two, or more thick, stiff

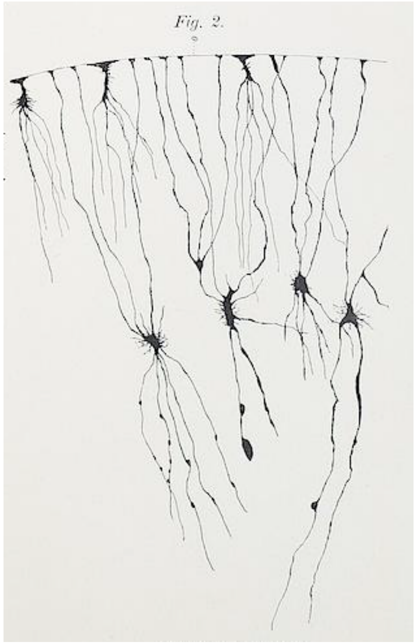

Fig. 2. From Table XIII. Fig. 2 Small area from a cross-section of the spinal cord of a 26 cm long fetus. Lateral strand
o – surface of the cord

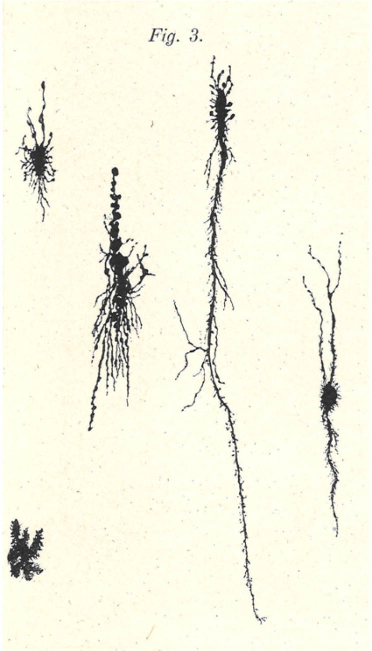

Fig. 3. From Table XIII. Five neuroglial cells from the Substantia gelatinosa Rolandi. From a 26 cm long human fetus.

processes extend to the periphery. These processes are either unbranched or generally branched, reaching the surface and terminating in a smaller or larger conical thickening.

The cell bodies are positioned at varying depths from the surface; many are located close to it or even directly at it, resulting in strangely shaped but highly variable forms, as shown in the figures. In general, the four types described by von Lenhossek can be identified, though many cells cannot be easily classified into these categories. Their variation is clearly extensive, as evident in the figures and as already noted by von Lenhossek.

From the above description it is obvious that I consider the support elements of the central nervous system of vertebrates as a particular sort of cellular elements in concordance with Golgi, Cajal and Lenhossek. Indeed, His has shown, many years ago, based on his profound and extensive investigations, that the support elements develop from a distinct form of embryonic cells, the spongioblasts.

The first developing support cells, the ependymal cells, play only a transitory role, in particular in higher vertebrates, they later step back or remain at a rudimentary state. In the meantime, the neuroglial cells develop from the same type of germ cell; first, they acquire the peculiar, long radial form of the ependymal cells, but soon they transform into the different forms of the neuroglial cells proper; in the different regions of the grey and white matter they obtain a distinct, highly variable typus.

Figure Legends

Table V

Ependyma and Neuroglia

in the spial cord of Petromyzon

Fig.1. Cross-section from the posterior part of the spinal cord of a 4cm long Petromyzon. At the right from the central canal are seven neuroglial cells and on the left of it is a cell illustrated in a stained mode. Around the central canal are five ependymal cells present; - gz, three ganglion cells with their branched processes; - s, sensible nerve roots; - h; contour of the outer sheath.

Fig. 2. Cross-section from the posterior part of the spinal cord of an adult long Petromyzon. On the right are four neuroglial cells, at the central canal three ependymal cells illustrated; gz, two ganglion cells, one small and a large with their branched processes are shown in the left half of the cord.

Fig. 3. Cross-section from the posterior part of the spinal cord of a 40 cm long Petromyzon. Two neuroglial cells and five ganglion cells are displayed here.

Fig. 4 Cross-section from the anterior part of the spinal cord, not far behind the head, of a 14cm long Ammocoetes. Eight glial cells are an ependymal cell (e) are displayed. A number cross-sectioned Müller fibers of different diameters are shown as contours (m); the processes of the neuroglial cells nuzzle between them.

All figures are drawn after Golgi preparations by using magnification by objective 6 and ocular 3 (inserted tube).

Table VI.

Ependyma and neuroglia

of the med. Spinalis and oblongata of the Petromyzon

Fig. 1 Cross-section from the anterior part of the spinal cord of a Petromyzon. Different forms of neuroglial cells; - e, an ependymal cell; - - cross sections of Müller fibers.

Fig. 2. Cross-section of the medulla oblongata of Petromyzon. Ependymal cell and neuroglial cells.

Both figures are drawn after Golgi preparations by using magnification by objective 6 and ocular 1 (inserted tube).

Table VII.

Ependyma and Neuroglia

In the spinal cord and brain of Petromyzon.

Fig. 1. Antero-lateral part of a cross-section from the most anterior spinal cord part of a young Petromyzon, with the central canal surrounded by spider cells and lateral dendritic neuroglial cells.

Fig. 2 and 3. Sections from the ventricular wall of the cerebrum of a 15 cm long Petromyzon with ependymal cells.

Fig. 4. Cross-section of the brain of a 16 cm long Petromyzom; s, ependymal cells, several ganglion cells of different size are displayed.

Both figures are drawn after Golgi preparations. Fig. 1-3 by using magnification by objective 6 and ocular 3 (inserted tube), Fig. 4 by using magnification by objective 6 and ocular 1 (inserted tube).

Table VIII.

Ependyma and Neuroglia

Of bony fish and birds

Fig. 1-3. Cross-sections of the spinal cord of young, 16 cm long salmons. Fig. 2 and 3 from the middle, Fig. 1 from the posterior part of the cord. Ependymal cells; - gz ganglion cells.

Fig 4a and 4b. The two lateral parts of a cross-section of the cerebrum of a young salmon. Ependymal cells and neuroglial cells.

Fig. 5 – 8. Cross-sections of the spinal cord and brain of a chicken embryo; - Fig. 5 cross-section of the lumbar spinal cord of an 8.day old chicken embryo, ependymal cells; - Fig. 6. Section from the frontal end of the spinal cord of a 14-day old chicken embryo; radial neuroglial cells; Fig. 7. Septum posticum at the transition section from the spinal cord into the med. Oblongata of an 8-day old chicken embryo; - Fig. 8. Section from a cross-section of the cerebrum of a 17-day old chicken embryo.

Both figures are drawn after Golgi preparations. Fig. 1-4 by using magnification by objective 6 and ocular 3 (inserted tube), Fig. 5-8 by using magnification by objective 6 and ocular 1 (inserted tube).

Table IX.

Ependyma and Neuroglia

at amphibia

Fig. 1-3. Cross-sections from the spinal cord and brain of larvae from Salamandra maculate; - Fig. 1. Cross-section of the spinal cord with ependyma-, resp. neuroglial cells; gz ganglion cells; - Fig. 2. Lateral section from a cross-section of the medulla oblongata; - Fig. 3. Part from a cross-section of the cerebrum; ependymal cells.

Fig. 4-7. Cross-sections from spinal cord and brain of a young frog (R. Temp.); - Fig. 4. Cross section of spinal cord; radial neuroglial cells; - Fig. 5. Cross-section of the medulla oblongata; ependymal cells; - Fig. 6. Section of the

mesencephalon; e, ependymal cells; - Fig. 7. Cross section of the cerebrum; m, midline, gz, ganglion cells; five ependymal cells; o – surface of the brain.

All figures are drawn after Golgi preparations. Fig. 1-3, 4 and 7 by using magnification by objective 6 and ocular 1 (inserted tube), Fig. 6 by using magnification by objective 6 and ocular 3. Fig. 5 by using magnification by objective 2 and ocular 3 (inserted tube).

Table X.

Ependyma and Neuroglia

at mammals.

Fig. 1. Cross-section from the spinal cord of a 3 cm long cat embryo. Ependymal cells.

Fig. 2. Cross-section from the posterior end of the lumbar cord a 12 cm long dog embryo. Ependymal cells.

Fig. 3. Cross-section from the spinal cord (neck region) of a 12 cm long dog embryo. Ependymal and neuroglial cells.

Fig. 4. Cross-section from the spinal cord (lumbar region) of a 12 cm long dog embryo. Ependymal and neuroglial cells.

Fig. 5. Region from a cross-section from the spinal cord (lumbar region) of a 12 cm long cat embryo. Antero-lateral region Neuroglial cells of the white matter.

All figures are drawn after Golgi preparations. Fig. 1 by using magnification by objective 2 and ocular 3 (inserted tube), Fig. 2-4 by using magnification by objective 6 and ocular 1 (inserted tube). Fig. 5 by using magnification by objective 6 and ocular 3 (inserted tube).

Table XI.

Ependyma and Neuroglia

at the human spinal cord

Fig. 1. Cross-section of the spinal cord (lumbar region) of a 3cm long human embryo. Ependymal and neuroglial cells. The dotted line indicates the border between white matter and grey matter. Some collaterals from the dorsal roots are displayed; bl, blood vessel.

Fig. 2. Septum posticum and dorsal part of the central canal from spinal cord of a 3cm long human embryo. Ependymal cells.

Fig. 3. Cross section from the spinal cord of a 15 cm long human embryo (neck region). Ependymal and neuroglial cells.

Fig. 4. Ventral ependymal wedge from spinal cord (neck region) of a 15 cm long human embryo.

All figures are drawn after Golgi preparations. Fig. 1 and 2 by using magnification by objective 6 and ocular 1 (inserted tube), Fig. 3 by using magnification by objective 2 and ocular 3 (inserted tube), Fig. 4 by using magnification by objective 6 and ocular 3 (inserted tube).

Table XII.

Ependyma and Neuroglia

at the human spinal cord

Fig. 1 Middle section from the cervical spinal cord of a 25 cm long human fetus.

Ck – central canal	sp – septum posticum.
vF – frontal fissure	bl – blood vessels

Around the central canal one can see ependymal cells and outside from it, neuroglial cells of different types (spider cells, mossy cells etc.).

Drawn after Golgi preparations by using magnification by objective 6 and ocular 3 (inserted tube).

Table XIII.

Ependyma and Neuroglia

at the human spinal cord

Fig. 1 Region from a cross-section of the neck cord of a 25 cm long human fetus. Antero-lateral region. Neuroglial cells of different forms.

o – surface of the cord p – border of the grey substance

w - white matter gr grey matter

Fig. 2 Small area from a cross-section of the spinal cord of a 26 cm long fetus. Lateral strand

o – surface of the cord

Fig. 3. Five neuroglial cells from the Substantia gelatinosa Rolandi. From a 26 cm long human fetus-

Fig. 4. Region from a vertical, longitudinal section of the ventral root of a 26 cm long fetus. Neuroglial cells in different variants.

o – surface of the cord

w – white matter

gr – grey matter

Drawn after Golgi preparations by using magnification by objective 6 and ocular 3 (inserted tube).

<u>Comments:</u>

1# Hornblatt (German expression)

2# Lancelet

3# Hagfish

4# Triton probably from Triturus and refers to newt

5# Referring only to Lenhossek

6# Genus lacerta belongs to the lizards

7# Frog

8# Genus of shark

9# Sea lamprey

10# Genus of skate

11# The spiny dogfish (Squalus **acanthias**), spurdog, mud shark, or piked dogfish is one of the best known species of the Squalidae (dogfish) family of sharks

12# Genus of shark

13# Salmon

14# Chapter 3 in this volume has the topic “On the nervous elements in the spinal cord of bony fish” and is not translated.

15# Gobius is a genus of fish in the family Gobiidae

16# Genus of stickleback

17# European eel

18# Fire salamander

19# Grass frog or common frog

20# this is probably a typo, it should be 2.7 cm and 3 cm as in the legend correctly stated
