# Supplementary material for: The Concept of Neuroglia ‐ the State of the Art Circa 1900
Source: Glia. 2025 Feb 4;73(5):890–904. doi: 10.1002/glia.24678 (PMC11920685; doi:10.1002/glia.24678)

# INHALT.

Seite

**1. Die Neuroglia des Gehirns beim Menschen und bei Säugethieren** ... 1

I. *Die Neuroglia der Hemisphären und Ganglien des Grosshirns.* Taf. I—X ... 6

A. Die Neuroglia der Grosshirnrinde des Menschen. Taf. I—V ... 6

B. Die Neuroglia der Grosshirnrinde des Hundes, der Katze und des Kaninchens. Taf. VI—VIII 12

C. Die Neuroglia der Grosshirnganglien, der Medulla oblongata und der Insula Reilii des Menschen. Taf. IX ... 14

D. Die Neuroglia der Grosshirnganglien, des Gyrus Hippocampi und des Fornix der Katze. Taf. X, Fig. 1—6 ... 15

II. *Die Neuroglia des Kleinhirns.* Taf. XI ... 16

A. Die Neuroglia des Kleinhirns des Menschen. Taf. XI ... 16

B. Die Neuroglia des Kleinhirns der Katze. Taf. XI, Fig. 6—8, 10—12 ... 18

III. *Die Neuroglia der Neuro-Hypophyse der Säugethiere.* Taf. XII ... 21

IV. *Die Neuroglia des Nervus opticus und der Retina des Menschen und der Säugethiere.* Taf. XIII 24

**2. Weitere Beiträge zur Kenntniss der Cajal'schen Zellen der Grosshirnrinde des Menschen.** Taf. XIV—XIX ... 29

**3. Ganglion ciliare.** Taf. XX ... 37

**4. Die embryonale Entwicklung der Rückenmarkselemente bei den Ophidiern.** Taf. XXI und XXII 41

**5. Die Endigungsweise des Gehörnerven bei den Reptilien.** Taf. XXIII, Fig. 1—7 ... 46

**6. Die Riechzellen der Ophidier in der Riechschleimhaut und im Jacobson'schen Organ.** Taf. XXIII, Fig. 8 ... 48

**7. Zur Entwicklung der Zellen des Ganglion spirale acustici und zur Endigungsweise des Gehörnerven bei den Säugethieren.** Taf. XXIV und XXV, Fig. 1—4 ... 52

**8. Kürzere Mittheilungen.** Taf. XXV, Fig. 5—11; Taf. XXVI und XXVII ... 58

I. *Zur Kenntniss des Ependyms im menschlichen Rückenmark.* Taf. XXVI, Fig. 1—4 ... 58

II. *Zur Frage von den freien Nervenendigungen in den Spinalganglien.* Taf. XXVI, Fig. 5 ... 59

III. *Ueber die Endigungsweise der Nerven an den Haaren des Menschen.* Taf. XXV, Fig. 5—9 ... 61

IV. *Einige Beiträge zur Kenntniss der intraepithelialen Endigungsweise der Nervenfasern.* Taf. XXVII 62

V. *Zur Kenntniss der Endigungsweise der Nerven in den Zähnen der Säugethiere.* Taf. XXVI, Fig. 8 und 9 ... 64

VI. *Die Pacinischen Körperchen in Golgi'scher Färbung.* Taf. XXVI, Fig. 6 ... 65

VII. *Verzweigte quergestreifte Muskelfasern.* Taf. XXV, Fig. 10 und 11 ... 65

**9. Ueber den Bau des Glaskörpers und der Zonula Zinnii in dem Auge des Menschen und einiger Thiere.** Taf. XXVIII—XXXII ... 67

# 1.

# Die Neuroglia des Gehirns beim Menschen und bei Säugethieren.

Bei meinen während einer Reihe von Jahren vermittelst der Golgi'schen Chromosmium-Silber-Methode fortgesetzten Untersuchungen über den Bau der nervösen Centralorgane hatte ich oft Gelegenheit, schöne Färbungen der Ependym- und Neurogliazellen bei verschiedenen Thieren und beim Menschen zu bekommen. Im J. 1891 gab ich eine kurze Mittheilung[1] darüber heraus und im vorigen Jahre veröffentlichte ich eine ausführlichere, durch eine Reihe von Abbildungen erläuterte Abhandlung über das Ependym und die Neuroglia des Rückenmarks der verschiedenen Wirbelthierclassen (auch des Menschen).[2]

In der vorliegenden Abhandlung beabsichtige ich eine Fortsetzung der Darstellung des fraglichen Gegenstandes mitzutheilen. Um aber diese Darstellung nicht zu weitläufig zu machen, werde ich mich diesmal auf die eigentliche Neuroglia des Gehirns des Menschen und einiger anderer Säugethiere beschränken. Die Ependymzellen und ihre Fortsätze sollen deshalb hier nur ganz beiläufig besprochen werden. Vor Allem werde ich die Neuroglia des menschlichen Gehirns behandeln und die der Säugethiere nur mehr des Vergleiches wegen besprechen.

Da ich hier unten ausschliesslich Ergebnisse von Untersuchungen mittelst der Golgi'schen Methode mittheile, so werde ich die Gesammtgeschichte der »Neurogliafrage» nicht eingehender besprechen, sondern nur die Arbeiten und Angaben derjenigen Forscher anführen, welche mit derselben Methode gearbeitet haben. Ich werde daher gar nicht auf die alten Controversen über die molekulare oder netzförmig-fibrilläre, die epitheloide oder bindegewebige Natur der Neuroglia eingehen, worüber schon mehrere geschichtliche Darstellungen und Besprechungen vorliegen. Hier soll in dieser Beziehung v. A. auf die Zusammenstellungen von Golgi[3] und Gierke[4] hingewiesen werden. In den ausführlichen und auf sehr eingehende Untersuchungen gestützten Neurogliastudien des letzteren Forschers sind auch die Angaben und Ansichten von Deiters, dem wir die eigentliche Entdeckung der typischen Gestalt der Neurogliazellen verdanken, von Jastrowitz, Boll und Ranvier besprochen und ausserdem manche neue Befunde mitgetheilt worden. Durch Weigert's treffliche Methoden wurde in der That unsere Kenntniss von der Verbreitung und dem Reichthum, sowie auch von den Formen der fraglichen Elemente in hohem Grade befördert. Golgi hat aber schon vor vielen Jahren eine Methode erfunden, welche, was gerade die Formen der Neurogliazellen betrifft, noch anschaulichere Bilder giebt, und der berühmte italienische Forscher hat selbst durch seine Arbeiten den wichtigsten Grund zu unseren neueren Errungenschaften auf diesem Gebiete wie auch auf demjenigen der ganzen modernen Nervenhistologie gelegt. Es ist deshalb ein sehr erfreuliches Ereigniss, dass seine betreffenden gesammelten Abhandlungen, welche zuerst theilweise in wenig zugänglichen italienischen medicinischen Zeitschriften veröffentlicht wurden, nunmehr in einer monumentalen deutschen Auflage vorliegen.[5]

[1] Gustaf Retzius, Ueber den Bau der Oberflächenschicht der Grosshirnrinde beim Menschen und bei den Säugethieren. Verhandl. d. Biolog. Vereins in Stockholm. Bd 3, März 1891.

[2] Gustaf Retzius, Studien über Ependym und Neuroglia. Biolog. Untersuchungen von Gustaf Retzius. N. F. Bd V, 2, 1893.

[3] Camillo Golgi, Contribuzione alla fina anatomia degli organi centrali del sistema nervoso, Rivista clinica di Bologna. Ser. 2 A. 1°, fasc. 11, novembre 1871 und A. 2°, fasc. 12, dic. 1871.

[4] Hans Gierke, Die Stützsubstanz des Centralnervensystems, Archiv f. mikrosk. Anatomie, Bd 25, 1885, und Bd 26, 1886.

[5] Camillo Golgi, Untersuchungen über den feineren Bau des centralen und peripherischen Nervensystems. Aus dem Italienischen übersetzt von Dr. R. Teutscher. Verlag von Gustav Fischer. Jena, 1894.

Was die Neuroglia betrifft, hat offenbar zunächst nach DEITERS GOLGI durch eingehende und selbstständige Untersuchungen die richtige Auffassung vom Typus der Neurogliazellen gewonnen und dargestellt. Dies betrifft auch besonders die Neuroglia des Gehirns, indem er sowohl in der grauen wie in der weissen Substanz, in der Rinde wie im Marke, diese Zellen als mit zahlreichen, feinen und langen Fortsätzen versehene Gebilde beschrieben und abgebildet hat; vom kleinen Zellenkörper strahlen diese Fortsätze in den verschiedenen Richtungen aus. Auch in der äussersten Rindenschicht des menschlichen Grosshirns hat er diese eigenthümlichen Zellen massenhaft gefunden und in seinen Tafeln abgebildet; in der Kleinhirnrinde hat er ebenfalls die Zellen der Bergmann'schen Fasern entdeckt und in der Körnerschicht wie auch im Marke die strahligen Neurogliazellen dargestellt.

In seiner grossen, zusammenfassenden Arbeit vom Jahre 1885[1] äussert GOLGI, nachdem er eingehend hervorgehoben hat, dass man durch Maceration des Gewebes in schwacher Bichromatlösung die strahlenförmigen Neurogliazellen der Grosshirnrinde und der weissen Substanz in isolirtem Zustande deutlich zum Vorschein bringen kann, jedoch zur klaren Darlegung der kleinsten Besonderheiten der Form, der Beziehungen, der Vertheilung u. s. w. der Bindegewebselemente (Neurogliazellen) zu der von ihm erfundenen combinirten Anwendung des Bichromats und des salpetersauren Silbers greifen muss. An den so erhaltenen Präparaten der grauen und der weissen Substanz »bemerkt man auf den ersten Blick:

1) Die Existenz einer beständigen, nur aus Neurogliaelementen bestehenden Schicht an der Oberfläche der Windungen. Diese Schicht ist sehr dick in der Höhe der Windung, dagegen dünn an den Seitenflächen zwischen zwei Windungen, wo sie bisweilen aus einer einzigen Zellenreihe besteht. Die Fortsätze dieser Zellen verlaufen zum Theil horizontal und bilden ein complicirtes Netz, welches die Oberfläche begrenzt, zum Theil dringen sie senkrecht in das Rindengewebe ein und bringen ein System von strahligen Fasern hervor, welches an das viel deutlichere und regelmässigere der Kleinhirnwindungen erinnert;

2) dass die letzten nach der Peripherie gerichteten Zertheilungen der Protoplasmafortsätze der pyramidalen Nervenzellen sich in dieser Schicht verlieren, wo sie sich mit den Neurogliazellen in Verbindung setzen;

3) dass die Neurogliazellen zahlreiche Verbindungen mit den Gefässwänden durch zum Theil sehr breite, zum Theil fadenförmige Fortsätze eingehen. Auch hier sieht man oft die Blutgefässe grosse Strecken weit durch zusammenhängende Reihen von Neurogliazellen umgeben, welche den Gefässwänden unmittelbar anliegen und von da aus ihre Fortsätze nach allen Richtungen aussenden; viele von diesen setzen sich an andere Gefässe an.»

Einige andere italienische Forscher, die grösstentheils Schüler von GOLGI waren und seine Methode anwendeten, haben dann in verschiedenen, den Bau der Centralorgane behandelnden Arbeiten auch die Neuroglia-Frage besprochen. So hat MONDINO[2] die strahlenförmigen Neurogliazellen aus mehreren Regionen des Gehirns (z. B. Vallecula Sylvii, Capsula externa, Insula Reilii) dargestellt. MAGINI[3] beschrieb an den langen Fortsätzen der strahlenförmigen Neurogliazellen der foetalen Gehirnrinde zahlreiche perlenschnurförmige Varicositäten. MARTINOTTI[4] gab dann eine Abbildung von Neurogliazellen der Grosshirnrinde, die einige interessante Formen darbieten. In der unter der Pia liegenden Schicht, sagt er, »finden sich Zellen vor, welche an die des Kleinhirnes erinnern, die platt an der freien Oberfläche befestigt und mit langen fadenförmigen und selten zerteilten Ausläufern versehen zum Teil in die graue Gehirnsubstanz auf $^2/_3$ ihrer Dicke eindringen». Unter dieser befinden sich andere von sehr verschiedener Form. Beim Hunde sah er solche, welche sehr starke, meist nicht getheilte Ausläufer nach der Peripherie hin senden, die bis unter die Pia gehen und an ihrem Ende eine dreieckige Ausbreitung bilden, welche sich zwischen die Ausläufer der tellerförmig gruppirten Zellen einschiebt; von denselben Zellen gehen in verschiedenen Richtungen viel dünnere Ausläufer aus, von denen einige fast die ganze Rinde nach abwärts hin durchlaufen. Beim Menschen begegnet man leicht solchen Zellen, und sie wurden bereits von GOLGI beschrieben. »Sie sind strahlenförmig, ihre Ausläufer, welche sich gegen die Peripherie hin richten, sind viel stärker als andere und endigen in einer Ausbreitung, die an ihrem äussersten Ende dreieckig ist. Diese Neurogliazellen, die von bemerkenswerther Stärke sind, bilden also für das darunter befindliche Gewirr markhaltiger Fasern eine kleine

[1] CAMILLO GOLGI, Sulla fina anatomia degli organi centrali del sistema nervoso, Reggio Emilia, 1885. S. auch die oben angeführte Ausgabe von GOLGI's Arbeiten in deutscher Sprache, 1894.

[2] CASIMIRO MONDINO, Ricerche macro e microscopiche sui centri nervosi, Torino 1887.

[3] GIUSEPPE MAGINI, Neuroglia e cellule nervose cerebrali nei feti. Atti d. 12. Congresso della Assoc. med. ital. in Pavia, sett. 1887. Vol. 1, Pavia 1888.

[4] CARLO MARTINOTTI, Beitrag zum Studium der Hirnrinde und dem Centralursprung der Nerven. Internat. Monatschr. für Anatomie und Physiologie. Bd 7, 1890.

Schutzschicht, die je nach der Oertlichkeit und nach dem Tiere mehr oder weniger dick ist; während die Zellen, welche mit den Fasern selbst vermischt sind, weniger stark werden und eher als Stütze dienen.»

Dann hat Ramón y Cajal in mehreren seiner für die Nervenhistologie bahnbrechenden Arbeiten auch die Neuroglia des Gehirns besprochen und abgebildet, obwohl er im Ganzen derselben seine Aufmerksamkeit weniger gewidmet hat als den eigentlichen nervösen Elementen. In der Kleinhirnrinde der jungen Katze hat er[1] Bergmann'sche Faserzellen und eine Neurogliazelle abgebildet und zugleich auch eine eingehende Darstellung der Elemente der von Vignal zuerst beschriebenen »äusseren Körnerschicht» gegeben. In seiner grösseren Abhandlung über den Bau der Grosshirnrinde[2] bespricht Cajal u. A. die Neuroglia-Frage ausführlicher. Er ist in Betreff der Entstehung der Neurogliazellen geneigt, der Ansicht zu huldigen, dass wenigstens ein Theil der Neurogliaelemente nach der Peripherie ausgewanderte Epithelzellen darstellen. Eine solche Emigration lässt sich in ihren verschiedenen Stufen in der foetalen Hirnrinde der Ratte und der Maus wahrnehmen, wo der Uebergang der Ependymzellen zu kürzeren, dickstämmigeren und mit vielen Fortsätzen versehenen Neurogliazellen darlegbar ist. Was aber die sog. Spinnenzellen betrifft, die keine radiale Anordnung zeigen und im Gehirn der Erwachsenen in Verbindung mit Blutgefässen vorhanden sind, meint Cajal, dass sie nicht vom Epithelium hervorgehen, sondern von den Endothelzellen der Blutgefässe oder von abgeplatteten Bindegewebszellen. Er ist zweifelhaft, ob solche Spinnenzellen auch von gewissen Elementen der Pia mater herrühren können. Auf seinen Tafeln theilt er einige Abbildungen mit, die die besprochenen Entwicklungsstadien wiedergeben.

In meiner vorläufigen Mittheilung beschrieb ich[3] die in der Grosshirnrinde des Hundes, des Kaninchens und der Ratte vorkommenden Neurogliazellen und lieferte einige Abbildungen von denselben. »Viele derselben liegen mit dem kernhaltigen Zellenkörper gerade in der Oberflächenebene und breiten sich dort abgeplattet aus, senden aber gewöhnlich wurzelartig verzweigte Ausläufer nach abwärts in die Rindensubstanz hinein. Nicht selten zieht ein starker Hauptast gerade oder geschlängelt in dieser Richtung hin, um nach reichlicher Verzweigung verschmälert aufzuhören. Andere Zellen liegen mit dem kernführenden Körper in die Rindensubstanz eingesenkt; manche derselben schicken aber, wie Martinotti beim Hunde gesehen hat, starke Ausläufer nach der Oberfläche hin, wo sie dicht unter der Pia mit einer dickeren platten Ausbreitung enden. Andere aber und v. A. die tiefer liegenden haben das von Golgi so reichlich abgebildete sternförmige Aussehen, haben aber verästelte Ausläufer.» In derselben Mittheilung beschrieb ich auch die Zellen der Bergmann'schen Fasern in der Kleinhirnrinde von 7- und 8-monatlichen menschlichen Foetus.

Etwa zur gleichen Zeit schilderte Van Gehuchten[4] aus der Kleinhirnrinde der jungen Katze Bergmann'sche Faserzellen sowie auch sternförmige Neurogliazellen mit vielen Fortsätzen, und zwar aus der Körnerschicht und der Marksubstanz (Hund). In seinen im vorigen Jahre erschienenen Vorlesungen über das Nervensystem des Menschen giebt Van Gehuchten[5] eine Darstellung der Neuroglia; sie besteht nach ihm aus den Ependymzellen und den Deiters'schen oder Spinnenzellen; diese letzteren kommen in der weissen und grauen Substanz überall vor. Van Gehuchten betont, dass ihre langen Fortsätze frei und ohne Anastomosen endigen; er schliesst sich der Ansicht an, dass dieselben modificirte Ependymzellen darstellen. Zwei solche Spinnenzellen aus der weissen Grosshirnsubstanz des Menschen sind in seinem Werke abgebildet.

Da ich hier nicht die Neuroglia des Rückenmarks zu berücksichtigen habe, so ist hier nicht der Platz, auf die betreffenden Arbeiten von M. von Lenhossék und *mir* und auch nicht auf diejenigen von Lachi und einiger anderer jüngerer italienischer Forscher einzugehen. Dagegen hat von Kölliker in der neuen Auflage seines grossen Handbuchs[6] eine umfassende Darstellung von der ganzen Neuroglia-Frage gegeben, welche, obwohl sie eigentlich die Verhältnisse des Rückenmarks behandelt, doch in mehrerer Beziehung auch die des Gehirns berührt. Nachdem er die Entwicklung der Neuroglia, resp. des Ependyms (v. A. im Rückenmark) dargestellt hat, geht er zu der Beschreibung der Neuroglia im ausgebildeten Marke über und bespricht dabei auch die des

[1] S. Ramón y Cajal, A propos de certains éléments bipolaires du cervelet, avec quelques détails nouveaux sur l'evolution des fibres cérébelleuses. Internat. Monatsch. für Anatomie und Physiologie, Bd 7, 1890.

[2] S. Ramón y Cajal, Sur la structure de l'écorce cérébrale de quelques mammifères. La Cellule t. 7, 1891.

[3] Gustaf Retzius, Über den Bau der Oberflächenschicht der Grosshirnrinde beim Menschen und bei einigen Säugethieren. Verhandl. d. Biolog. Vereins in Stockholm. Bd 3, März 1891.

[4] A. Van Gehuchten, La structure des centres nerveux. La moelle épinière et le cervelet. La Cellule. T. 7, 1, 1891.

[5] A. Van Gehuchten, Le système nerveux de l'homme. Leçons professées à l'Université de Louvain. 1893.

[6] A. von Kölliker, Handbuch der Gewebelehre des Menschen. 6. Aufl. Bd II, 1, 1893.

Gehirns. VON KÖLLIKER schliesst sich entschieden der Ansicht an, dass Ependym- und Sternzellen (Deiters'sche oder Golgi'sche Zellen) aus derselben Art von Embryonalzellen (Ektodermzellen) der Centralorgane hervorgehen und nicht zum Theil aus später eingewanderten Mesodermzellen rekrutirt werden. Was die letztere Art der Neurogliazellen, die sternförmigen (Spinnenzellen), betrifft, die v. KÖLLIKER die eigentlichen Golgi'schen Zellen nennt, so sind sie nach ihm in allen Theilen der grauen und weissen Substanz des Markes und auch des Gehirns in reichlichster Menge vorhanden und bilden mit ihren zahlreichen Ausläufern ein Gerüst, welches zwischen allen nervösen Elementen hindurchzieht und für viele derselben, vor Allem aber für die Nervenzellen, die stärkeren Nervenfasern und die Gefässe besondere Hüllen und Scheiden darstellt. Auch bilden diese Zellen besondere Umhüllungen grösserer Abschnitte, wie z. B. des gesammten Rückenmarks und vieler Theile des Gehirns, »die Gliahüllen». Was die Formen dieser Elemente betrifft, unterscheidet v. KÖLLIKER zwei Varietäten, die *Kurzstrahler* und die *Langstrahler;* die ersteren sind mit kürzeren, stark verästelten, die letzteren mit sehr langen, wenig getheilten Fortsätzen versehen. Die Kurzstrahler finden sich, wenn auch nicht ausschliesslich, so doch vor Allem in der grauen, die Langstrahler in der weissen Substanz; die Gestalt des Zellenkörpers ist bei den Kurzstrahlern im Allgemeinen in allen Richtungen sternförmig, bei den Langstrahlern mehr verlängert und auch meist stark abgeplattet, was auch bei den Kurzstrahlern vorkommt; die Gestalt der Zellen zeigt aber dieselben Wechselungen wie bei den multipolaren Ganglienzellen; die Zahl der Fortsätze ist selten unter fünf, häufig 10—15—20, und oft sind sie so zahlreich, dass man ihre Zahl nicht bestimmen kann; sie anastomosiren nie, wie GOLGI auch zuerst nachwies. Die Lehre von RANVIER (und WEIGERT), dass die Fasern (Fortsätze) nicht direkt den Zellen angehören, sondern gewissermassen selbständig sind, bespricht v. KÖLLIKER eingehend, indem er die fragliche Anschauung zu erklären versucht. Er theilt einige Abbildungen von Neurogliazellen des Gehirns mit, nämlich an Blutgefässe sich ansetzende Kurzstrahler aus den Hemisphären des Kaninchens und Langstrahler aus der weissen Substanz der Hirnrinde und des Cerebellum des Menschen. Ausserdem giebt er eine Beschreibung und Abbildung von den Zellen des Cerebellum (der Katze), ebenso von den Bergmann'schen Fasern.

Von den in der Fascia dentata vorkommenden Formen der Neurogliazellen gab RAMÓN Y CAJAL[1] eine Darstellung; er fand theils die schon von L. SALA beschriebenen Sternzellen in dem peripherischen Rande der Molekularschicht und dann noch eigenthümliche spindelförmige (verschieden gestaltete) Zellen in der Körner- und Molekularschicht.

Im vorigen Jahre veröffentlichte LLOYD ANDRIEZEN[2] Mittheilungen über ein mit der Golgi'schen Methode dargelegtes perivasculäres System von Neurogliazellen im menschlichen Gehirn, welches er auch bei mehreren anderen Säugethieren nachgewiesen hatte. Die von diesen Zellen ausgehenden Fasern bilden, sagt er, ein verdichtetes Netzwerk an zwei Oertlichkeiten, erstens an der Oberfläche des Gehirns und zweitens um die Blutgefässe desselben. Die Zellen sind zweierlei Art. Die erste besteht aus *protoplasmatischen* Elementen mesoblastischen Ursprungs und von activer lymphatischer Function, von sternförmiger oder dendritischer Natur, sowie von moosartigem und körnigem Aussehen; diese Zellen finden sich besonders in der grauen Substanz und bestehen theils aus grossen, mit dicken, an den Adventitialscheiden befestigten protoplasmatischen Fortsätzen versehenen, theils aus kleinen, die grösseren Ganglienzellen der Rinde unmittelbar umgebenden Zellen (den perizellulären Elementen). Die zweite Art stellt *faserige* Elemente (Fibre elements) epiblastischen Ursprungs, von stützender und passiver Function, dar; diese bilden sowohl ein diffuses Netzwerk wie auch die Verdichtungssysteme, nämlich 1. ein diffuses Netzwerk überall in der Rinde und der Marksubstanz, 2. die oben erwähnten Verdichtungssysteme an der Oberfläche und um die Blutgefässe herum. Das faserige Oberflächensystem stellt ein Filzwerk tangentialer Fasern dar, welche hauptsächlich von den »geschwänzten» Zellen herrühren; von diesen Zellen strömen Fortsätze oder Fasern tief in die Rinde hinab, sogar bis zu ihrer halben Dicke, bis zur Mitte der Pyramidenzellenschicht. Im perivasculären Fasersystem sind wenigstens drei Subvarietäten zu unterscheiden, und zwar erstens den Gefässen der Länge nach angeordnete Zellen, deren Faserfortsätze längs den Gefässen weit hin verfolgt werden können, die aber mehr oder weniger zahlreiche Fortsätze auch nach der Seite hin in die Grundsubstanz hineinschicken; diese Zellen liegen in mässiger Entfernung von einander und bilden ein loses Maschenwerk um die Gefässwand herum. Mit diesen Zellen vermischt findet man eine zweite Art von länglichen

[1] S. RAMÓN Y CAJAL, Estructura del asta de Amon y fascia dentata. Trabajos leidos ante la Sociedad española de historia natural, 1893.
[2] W. LLOYD ANDRIEZEN, On a system of fibre-cells surrounding the blood-vessels of the Brain of Man and Mammals, and its Physiological Significance. Intern. Monatschr. f. Anat. u. Physiol. Bd 10, 1893. — British Medical Journal, Juli 1893.

Zellen, die quer um die Gefässe herumliegen und mit den erst erwähnten Zellen zusammen das perivasculäre Netzwerk bilden, das in der Grundsubstanz unmittelbar um die Gefässe herumliegt. Drittens giebt es noch die gewöhnlichen sternförmigen, in der Grundsubstanz zerstreuten Zellen, welche hier und da auch Fasern zu dem perivasculären Netzwerk abgeben.

Dann erschien noch in demselben Jahre ein Aufsatz über die Neuroglia der menschlichen Hirnrinde von GREPPIN.[1] Er unterschied in derselben *zwei* Arten nicht nervöser Zellen: 1) Grosse, moos- oder buschförmige Zellen, deren zahlreiche Fortsätze sich von ihrem Ursprung an häufig theilen, unter sich und mit den Fortsätzen der benachbarten gleichartigen Gebilde zahlreiche Anastomosen eingehen und so einem dichten Netzwerk den Ursprung geben; der Zellkörper ist sehr unregelmässig, bald stark eckig, bald mehr länglich, nur selten rundlich gestaltet. 2) Zellige Elemente mit nur kleinem, meistens rundlichem oder ovalem Zellkörper, von dem radienförmig gerade, sich nicht oder nur selten theilende, lange Fortsätze abgehen, die in keiner Weise mit einander anastomosiren. GREPPIN hält es für annehmbar, dass die anastomosirenden Zellen den ursprünglichen Spongioblasten von HIS entsprechen, während die Sternzellen als reine bindegewebige Elemente aufzufassen seien.

In einer kurzen Mittheilung beschrieb AZOULAY[2] die Formen der Neurogliazellen des Cornu Ammonis, ebenso die des Rückenmarks und des Kleinhirns bei Kindern vom Alter von mehr wie einem Monate.

In einer in diesem Jahre erschienenen Mittheilung hat BERKLEY[3] eine Beschreibung der Neuroglia- und Ependymzellen der Infundibularregion und der Wände des dritten Ventrikels beim erwachsenen Hunde veröffentlicht. In derselben zeigt er, dass in diesen Regionen die Ependymzellen noch im *erwachsenen* Zustande nicht nur vorhanden sind, sondern sogar in *guter* Ausbildung vorkommen und von der Ventrikularwand bis zur Aussenfläche des Gehirns reichen. Er beschreibt die verschiedenen Zellentypen sowohl des eigentlichen Ependyms als der Neuroglia und theilt gute Abbildungen der Elemente mit. In den Wänden des Infundibulum unterscheidet er hinsichtlich des Ependyms (der ependymalen Neuroglia) eine Hauptform und mehrere Subvarietäten von verschiedenem Charakter des Stammtheiles und der Verästelungen; ausserdem kommen hier auch grosse sternförmige Neurogliazellen mit vielen radiirenden Faserfortsätzen vor. In den Wänden des dritten Ventrikels hat er eine schöne Reihe von verschiedenen Neurogliazellformen gefunden, nämlich erstens zwei Arten von Ependymzellen, und zwar lange, fadenförmige, variköse (beim Uebergang von der Infundibularhöhle zum dritten Ventrikel) und mit zahlreichen, nach den Seiten hin ausstrahlenden Zweigen versehene Ependymzellen (in der Ventrikularwand); ferner verschiedene Arten von stern- und moosförmigen Neurogliazellen. Alle diese Neurogliazellformen, nur die embryonalen Typen ausgenommen, stehen in genauem Verhältniss zu den naheliegenden Blutgefässen, und dies mittelst Anheftung ihrer knötchenförmigen Endigungen an die hyaline Scheide der Gefässe.

In dieser geschichtlichen Uebersicht habe ich absichtlich solche Regionen des Centralorgans nicht besprochen, welche zwar mit ihm genetisch zusammenhängen, aber von seinen typischen Verhältnissen mehr abweichen, wie Hypophysis cerebri, Nervi optici, Retina; ich werde aber unten anhangsweise einige dieser Organtheile behandeln.

Dagegen ist mir während des Druckes dieser Arbeit noch eine Mittheilung in die Hände gekommen, nämlich eine neue Beschreibung der Neurogliazellen der Kleinhirnrinde von VAN GEHUCHTEN;[4] er giebt darin mehrere Abbildungen und eine eingehendere Schilderung der Zellen der Bergmann'schen Fasern beim Menschen und zeigt dabei auch, wie sich diese Zellen in der Tiefe der Furchen verhalten.

Endlich soll hier hervorgehoben werden, dass CLAUDIO SALA[5] eine übersichtliche Darstellung der Neurogliafrage veröffentlicht hat, in welcher er die Verhältnisse bei den verschiedenen Wirbelthierclassen bespricht.

[1] L. GREPPIN, Ueber die Neuroglia der menschlichen Hirnrinde. Anatom. Anzeiger, Bd 9, N:o 3, Nov. 1893.

[2] M. L. AZOULAY, Note sur les aspects des cellules névrogliques dans les organes nerveux centraux de l'enfant. Comptes rend. hebd. d. s. de la Soc. de Biol., 1894, N:o 9.

[3] HENRY J. BERKLEY, The Neuroglia Cells of the Walls of the middle Ventricle in the adult Dog. Anatom. Anzeiger, Bd 9, N:o 24 und 25, Aug. 1894.

[4] A. VAN GEHUCHTEN, La Neuroglie dans le cervelet de l'homme. Bibliographie anatomique, Année 2, N:o 4, 1894.

[5] CLAUDIO SALA Y PONS, La Neuroglia de los Vertebrados. Barcelona 1894.

# I. Die Neuroglia der Hemisphären und Ganglien des Grosshirns.

(Taf. I—X.)

## A. Die Neuroglia der Grosshirnrinde des Menschen. (Taf. I—V.)

Ich gehe jetzt zur Darstellung meiner eigenen Befunde über und werde v. A. die Verhältnisse beim Menschen behandeln. Ich habe bei den vorliegenden Untersuchungen gesucht, die foetale Entwicklung der Neurogliazellen des Gehirns zu verfolgen. Da es mir nicht gelungen ist, mittelst der Golgi'schen Methode die Neurogliazellen in den ersten vier Monaten darzustellen, so kann ich die früheren embryonalen Stadien resp. ihre Ausbildung aus Ependymzellen nicht schildern, und ich gehe deshalb diesmal auf die Abstammungsfrage nicht ein. Es ist v. A. meine Aufgabe, hier die Formentwicklung der Neurogliazellen des Gehirns in der späteren Hälfte des Foetallebens darzustellen und dann noch ihren Uebergang in die Formen des erwachsenen Zustandes zu beschreiben. Was die eigentlichen Ependymzellen betrifft, welche von der Innenfläche der Ventrikelwände zu der Hirnoberfläche reichen, so haben sie einen so bestimmten, fast überall wiederkehrenden Typus und behalten denselben während des ganzen Foetallebens, dass ich sie diesmal nicht zu schildern beabsichtige. Ich gebe nur des Vergleiches wegen einige Abbildungen ihrer äusseren Enden. In Fig. 4 der Taf. I ist eine Gruppe solcher Gebilde vom 19.5 Cm. langen menschlichen Foetus (Frontallappen) dargestellt. In Fig. 1 derselben Tafel sind zwei derartige Ependymzellenenden (vom $6^1/_2$ Mon. alten Foetus) und in Fig. 1 der Taf. XV der 2. Abhandl. ist links eine Gruppe aus dem Gehirn eines 28 Cm. langen Foetus abgebildet. Die Zellen zeigen alle die von mir und Anderen schon früher (bei anderen Säugern) beschriebenen Eigenschaften. Die langen, schmalen, hin und wieder etwas knotigen und ein wenig geknickten, im Ganzen aber unter einander parallelen Fasern, welche mehr oder weniger senkrecht gegen die Oberfläche hin aufsteigen, theilen sich gleich vor dem Eintritt in die äusserste (sog. molekuläre) Rindenschicht dichotomisch, wobei gewöhnlich an der Theilungsstelle ein Knötchen vorhanden ist; die beiden Theiläste steigen unter meist spitzem Winkel nach der Oberfläche hin und biegen bald wieder in eine beinahe senkrechte Richtung um; sie theilen sich dann in der Regel noch ein oder mehrere mal dichotomisch, wonach die etwas feiner werdenden Aeste sich in gleicher Weise verhalten; alle Aeste steigen nun als ein Büschel feiner knotig-variköser Fasern nach der Oberfläche hin, um dort mit einem verschieden starken Knötchen zu endigen, das entweder rundlich-oval, oder konisch ist. Diese Ependymfaserenden lassen sich während des ganzen Foetallebens nachweisen, sie färben sich beim Menschen jedoch schwerer in den späteren Monaten; nach der Geburt habe ich sie nicht darlegen können. Zusammen mit den aufsteigenden Aesten der Cajal'schen Zellen bilden sie ein für die Hirnrinde sehr charakteristisches dichtes Gitter- oder Palissadenwerk.

In diesem Gitterwerk der äussersten Rindenschicht liegt nun — ausser den Zellenkörpern der Cajal'schen Zellen — eine überaus grosse Anzahl von Neurogliaelementen von verschiedenartiger Gestalt. Ich brauche fortwährend für alle diese Stützelemente der Centralorgane, welche nicht den echten ursprünglichen ependymalen Charakter haben, die Benennung »Neuroglia».

Die frühesten Stadien dieser Neurogliazellen, die ich mittelst der Golgi'schen Methode in der Grosshirnrinde nachweisen konnte, kamen bei einem 28 Cm. langen Foetus vor. Ich habe in der nächsten Abhandlung (N:o 2) dieses Bandes (in der Fig. 1 der Taf. XV) mehrere solche Zellen zusammen mit Cajal'schen Zellen und Pyramidenzellen abgebildet. Einige derselben dokumentiren sich als Neurogliazellen durch ihre Gestalt, durch den kleinen Zellkörper und den Charakter der Fortsätze; andere sind in Betreff der letzteren noch wenig entwickelt, aber gerade deshalb um so interessanter, weil sie noch sehr frühen Stadien entsprechen; bei diesen letzteren sieht man von dem kleinen Zellkörper nur einen oder einige wenige Fortsätze entspringen, die mit keinen oder doch nur sparsamen stacheligen Aesten versehen sind. In Form und Anordnung imitiren sie hier und da die Cajal'schen Zellen, so dass man sie in diesem Stadium nur durch die Grösse unterscheiden oder sogar zuweilen zweifelhaft sein kann, welche Art von Zellen vorliegt.

Dann habe ich bei Foetus in der Mitte des *siebenten* Monates sehr schöne und reichliche Färbungen der Neurogliazellen bekommen. In den Fig. 1—3 der Taf. I sind die in diesem Stadium gewöhnlichsten Formen derselben wiedergegeben, nämlich in der Fig. 1 an einem Vertikal- und in den Fig. 2 und 3 an Tangentialschnitten der Grosshirnrinde. In der Fig. 1 sind ausserdem rechts eine Cajal'sche Zelle und in der Mitte und links zwei äussere Enden von Ependymzellen-Fortsätzen abgebildet. Alle die übrigen Gebilde sind Neurogliazellen von verschiedener Form. An der eigentlichen Oberfläche traf ich hier noch keine gefärbten Zellkörper, wohl aber viele Zellen, die, mit ihrem Körper dicht unter der Oberfläche belegen, Fortsätze nach derselben schickten und dort mit knotenförmigen Enden endigten; diese Knoten, welche wie die übrigen Theile der Zellen scharf schwarzbraun gefärbt werden, sind von verschiedener, zuweilen sehr bedeutender Grösse, so dass sie umfangreicher wie der Zellkörper sein können (Fig. 1 der Taf. I). Der Zellkörper liegt in verschiedener Tiefe unter der Oberfläche; er ist im Ganzen klein, von rundlich-ovaler oder länglich ausgezogener Gestalt und mit eckigen Aussprüngen, so auch mit einer verschieden grossen Anzahl feiner, kurzer, moosig erscheinender Fortsätze versehen, die nach verschiedenen Seiten hin ausstrahlen. Ausserdem entspringen vom Zellkörper stärkere Fortsätze in wechselnder Anzahl, so dass bald nur ein einziger, bald zwei, drei, vier oder mehrere vorkommen, welche zuweilen direkt nach der Oberfläche ziehen, öfter aber zuerst nach der Seite hin verlaufen, um dann umbiegend und oft sich einmal oder wiederholt theilend, die Oberfläche zu erreichen, wo sie mit den oben besprochenen Knötchen endigen; nach unten (innen) schicken die Zellkörper auch oft einen stärkeren Fortsatz ab, welcher getheilt oder ungetheilt, gewöhnlich aber unter Abgabe von kleinen moosigen Aesten verschieden weit hinabragt und verfeinert endigt. Der Zellkörper umschliesst gewöhnlich eng den Kern, der in den Golgi'schen Präparaten nur schwach hervortritt. In Folge der wechselnden Gestalt dieser Zellen lohnt es sich kaum, die verschiedenen foetalen Typen derselben aufzustellen und zu beschreiben.

Nach unten (innen) von dieser Zellenregion finden sich eine Menge von Neurogliazellen, welche, obwohl unter sich sehr verschiedenartig, einen von demjenigen der oben beschriebenen abweichenden Typus zeigen. In der Fig. 1 der Taf. I sind einige der gewöhnlichsten dieser Zellen wiedergegeben. Ein Theil derselben ist »sternförmig» im alten Sinne, d. h. vom rundlich-ovalen Zellkörper gehen nach verschiedenen Seiten hin mehrere Fortsätze aus, die nach aussen radiiren, dabei aber sich wiederholt dichotomisch theilen und nach kürzerem oder längerem Verlaufe frei endigen; diese Fortsätze sind oft, besonders die nach oben hin ziehenden, mit Knötchen und Zacken besetzt und schicken kleine Aeste aus. Der Gestalt und Vertheilung der Fortsätze nach variiren auch diese Zellen, wie die Fig. 1 zeigt, recht sehr; bald gehen die Fortsätze nach allen Richtungen hin, bald überwiegend nach einer Seite ab. Die letztere Varietät bildet einen Uebergang zu einer anderen, sehr eigenthümlichen Form, welche dieser foetalen Periode charakteristisch ist. Wie die Fig. 1 der Taf. I (links) zeigt, schickt der kleine, ovale Zellkörper bei einer Anzahl von Neurogliazellen einen oder zwei stärkere Fortsätze nach unten hin in die Pyramidenzellenschicht; diese Fortsätze ziehen, hier und da dichotomisch verzweigt, sammt ihren stärkeren Aesten mehr oder weniger vertikal eine weite Strecke in die erwähnte Schicht hinab, um dort zuletzt unter feiner Verästelung zu endigen; während dieses Verlaufes schicken diese Fortsätze eine Anzahl von feinen, varikösen, verästelten, oft sogar moosigen Aestchen nach den Seiten hin. Neben diesen Zellen kommen, besonders etwas tiefer hinab, andere Zellen vor, welche solche Fortsätze nicht nur nach unten hin, sondern auch nach oben hin abgeben (Fig. 1 d. Taf. I).

Auf eine ausführlichere Beschreibung der fraglichen Neurogliazellen einzugehen, finde ich nicht nöthig. Es kommt zwar eine grosse Zahl von Varianten vor; dieselben ohne noch mehr Abbildungen zu beschreiben, lohnt sich aber kaum. Indessen habe ich zwei Partien von Tangentialschnitten beigefügt; die eine (Fig. 2 der Taf. I) ist ein Oberflächenbild, wo man von oben (aussen) her die Verzweigung und Anordnung der Fortsätze der oberflächlicheren Zellen sieht; in der zweiten (Fig. 3 der Taf. I), die nicht diese Zellen, sondern die etwas tiefer liegenden darstellt, sieht man ebenfalls die Anordnung und Verästelung der fraglichen Zellen und ausserdem noch die Befestigung einiger ihrer Fortsätze an den Blutgefäss-Scheiden, wie dieselbe schon längst von Golgi nachgewiesen und von mehreren Forschern bestätigt worden ist; ich erinnere in dieser Hinsicht besonders an v. Köllikers Darstellung und seine Fig. 415 (d. Handb. d. Gewebelehre Bd II, 1, 6. Aufl.), welche die entsprechenden Verhältnisse aus den Hemisphären des Kaninchen wiedergiebt. Nie sieht man aber direkte Verbindungen unter den Neurogliazellen selbst, *nie Anastomosirung* derselben.

Gegen das Ende des intrauterinen Lebens verändern sich allmählig die Typen der Neurogliazellen, jedoch ohne noch den vollständigen Charakter des ausgebildeten Zustandes zu erreichen. In den Fig. 4 und 5 der Taf. II

sind aus einer Reihe von Präparaten aus dem Stirnlappen eines noch nicht ausgetragenen (45 Cm. langen) menschlichen Foetus die gewöhnlichsten und prägnantesten Formen zusammengestellt. Hier sind echte Oberflächenelemente der Gehirnrinde sichtbar, d. h. solche, deren Zellkörper mit ihrer abgeplatteten oder etwas gewölbten oberen Fläche in dem Niveau der Rinde liegen, die Innenseite der Pia mater berührend oder sogar hier und da an ihr befestigt; zuweilen sieht man diese, von oben betrachtet, rundlich-polygonalen und verschieden grossen Zellkörper etwas hügelartig aus dem oberen Rande der Schnitte emporragen, oder sie zeigen sich sogar mit einem abgeplatteten, gefussten Knopfe versehen. Von diesen in dem Niveau der Gehirnoberfläche belegenen Zellkörpern gehen Fortsätze nach den Seiten hin und bilden, nach allen Richtungen hin radiirend, ein Oberflächennetz von Neurogliafasern, indem sie tangential durch die äusserste Rindenzone verlaufen und sich verschiedentlich durch einander weben. Vom unteren Umfang des Zellkörpers, welcher bald kurz und abgerundet, bald mehr oder weniger dreieckig nach unten hin hinabragt, gehen auch theils seitliche, theils nach unten (innen) hin ziehende Fortsätze aus, welche bald nur kurz sind, bald weit hinab durch die »Molekularschicht» und die Pyramidenzellenschicht verlaufen; diese letzteren Fortsätze sind fadenförmig, gestreckt, unverzweigt und hier und da mit Knötchen versehen (Fig. 5 der Taf. II).

Unter diesen Zellkörpern, deren Vorläufer in den früheren Perioden des Foetallebens ich bisjetzt nicht darzulegen vermochte, findet man die schon aus dem 7. Monate beschriebenen Elemente wieder, deren Körper ein wenig unter der Oberfläche der Rinde liegt, aber eine Anzahl von gewöhnlich stärkeren Fortsätzen mehr oder weniger divergirend nach der Oberfläche hin schickt, wo sie knotenförmig endigen (Fig. 4 und 5 der Taf. II). Darunter finden sich theils »sternförmige» Zellen mit vielen, nach allen Richtungen ausstrahlenden Fortsätzen, theils Zellen mit langen, ziemlich tangential verlaufenden, dicken, mit moosartigen Aesten versehenen Fortsätzen (Fig. 4 der Taf. II), welche sich zuweilen nach oben umbiegen, um an der Oberfläche zu endigen, zuweilen (Fig. 5 der Taf. II) auch nach unten hin ziehen. Dann habe ich noch die vertikal ausgestreckte Zellenart zu berücksichtigen, welche ich oben von 7-monatlichen Foetus beschrieben habe. In der That findet man die Zellen in diesem späteren Stadium wieder, obwohl sie dann eine gewisse Veränderung erfahren haben. Sie zeigen nämlich jetzt in der Regel sparsamere Fortsätze; diese sind aber, wie der Zellkörper selbst, sehr moosig, d. h. mit zahlreichen, feinen, knotig-varikösen Aesten versehen (Fig. 4 und 5 der Taf. II); die in Fig. 4 abgebildete Zelle schickt sogar zwei Fortsätze bis an die Oberfläche hinan, was seltener vorkommt.

Unter diesen Zellen trifft man nun eine Menge von Elementen, die dem »sternförmigen» Typus zugezählt werden müssen, obwohl sie recht grosse Wechselungen und auch Uebergänge zu den anderen Formen zeigen können, indem von ihnen ein oder mehrere längere Fortsätze entspringen (Fig. 4 und 5 der Taf. II). Die Fortsätze dieser Zellen sind zuweilen mit grösseren oder kleineren tropfenförmigen Verdickungen (»Varikositäten») besetzt (Fig. 4 und 5). Hier und da trifft man Neurogliazellen, welche einige Fortsätze zu einer Gefässwand schicken, an der sie sich mit konischem Fusse befestigen (Fig. 5 der Taf. II, rechts) oder lange Fortsätze den Gefässwänden parallel entsenden und dieselben damit gewissermassen umstricken, wie Golgi und später Andriezen genauer beschrieben haben.

Ich habe die Neuroglia des Gehirns auch bei einer Anzahl von *neugeborenen* Kindern untersucht und die Verhältnisse den eben geschilderten vom 45 Cm. langen Foetus so ähnlich gefunden, dass es hinreichend ist, auf die beigegebenen Figuren hinzuweisen. In Fig. 1 der Taf. II ist ein Vertikalschnitt, in Fig. 2 eine Zelle aus der Marksubstanz und in Fig. 3 ein Oberflächenbild eines 48 Cm. langen ausgetragenen Foetus dargestellt. Die in der Fig. 2 abgebildete Sternzelle aus der Marksubstanz zeigt, wie schon die unterste Zelle der Fig. 1, die schon eingetroffene Differenzirung der für die weisse Substanz charakteristischen Neurogliazellen. In der Fig. 3 sind vier Oberflächenzellen von oben her gesehen. *Anastomosen* unter den Gliazellen sind *nie* sichtbar.

In den *ersten Monaten nach der Geburt* behalten zwar die meisten Neurogliazellen der Grosshirnrinde ihren oben beschriebenen Charakter bei, nehmen aber mehr und mehr den Typus des ausgebildeten Stadiums an; dies ist vor Allem betreffs der oberflächlicheren Zellen der Fall. Die Fig. 1, 2 und 3 der Taf. III sind vom *zweimonatlichen*, die Fig. 4 und 5 ders. Tafel vom *dreimonatlichen* Kinde. In den Fig. 1 und 4 erkennt man die an der Oberfläche liegenden, hier und da knopfförmig emporragenden Zellkörper mit ihren tangential verlaufenden und ihren nach unten ziehenden sehr langen gestreckten Faserfortsätzen; ferner die verschieden weit unter der Oberfläche belegenen Zellkörper, die eine wechselnde Anzahl von divergirenden Fortsätzen nach der Oberfläche hin senden; dann weiter unten die eigenthümlichen, langen, mehr oder weniger vertikal gestellten, moosigen

Zellen und schliesslich die Sternzellen, welche in der grauen Substanz eine verästelte und moosige, gewundene, in der weissen (Fig. 2) eine wenig verästelte, feinfaserige, gestreckte Beschaffenheit der Fortsätze darbieten. Von der Oberfläche der Rinde her gesehen (Fig. 3 und 5 der Taf. III) zeigen die in derselben belegenen Zellkörper eine scharf contourirte, eckig-rundliche oder ovale Gestalt, und von ihnen entspringen nach verschiedenen Seiten hin lange, feine, faserige, gestreckt oder wenig geknickte, hier und da mit Knötchen besetzte Fortsätze, die sich kreuzen und den oben erwähnten Neurogliafilz der Rindenoberfläche bilden; ausserdem bemerkt man noch die von den Zellkörpern nach unten ausgehenden feinen, kurzen Fortsätze.

Schon am Ende des *ersten* Jahres und noch mehr in den zunächst folgenden Jahren gewinnen die Neurogliazellen der Grosshirnrinde ihre fertigen, ausgebildeten Formen. In der Fig. 1 der Taf. IV und in der Fig. 2 derselben Tafel habe ich zwei Partien von Vertikalschnitten als Beispiele mitgetheilt; jene rührt vom Stirnlappen eines *1-jährigen*, diese vom Stirnlappen eines *$5^1/_2$-jährigen Kindes* her. Die Neurogliazellen lassen sich nunmehr auf etwa *vier* Typen zurückführen, nämlich auf die mit dem Zellkörper die Oberfläche berührenden Zellen, deren lange fadige Fortsätze theils tangential in der Rindenoberfläche verlaufen, theils mehr oder weniger vertikal durch die äusseren Rindenschichten (die »Molekular»- und Pyramidenzellenschicht) ziehen, ferner auf die etwas unter der Oberfläche belegenen Zellkörper, welche Fortsätze bis zur Oberfläche der Rinde schicken, und endlich auf zwei Arten von Sternzellen, von denen die einen mit reichlicher verästelten, moosigen Fortsätzen versehen sind und der grauen Substanz angehören, die anderen lange feine, fadige, gestreckte und fast nur am Ursprung verzweigte Fortsätze besitzen. Für die Darstellung der Neuroglia wäre es gewiss bequem, ja sogar nothwendig, für die verschiedenen Typen besondere Benennungen zu haben. Zwar giebt es zwischen diesen Typen auch gewisse Uebergangsformen, welche ihre Zusammengehörigkeit beweisen; die meisten Elemente haben jedoch einen bestimmten Charakter und lassen sich ohne Zwang zu dem einen oder dem anderen Typus hinführen. Was nun die Benennungen betrifft, so hat von Kölliker die Neurogliazellen in *Kurz-* und *Langstrahler* getheilt, und diese Namen entsprechen in mancher Hinsicht den Verhältnissen, indem sie einen wichtigen Charakter der fraglichen Elemente berücksichtigen. Man könnte sie, auf Grund einer anderen Eigenschaft, auch *Krausstrahler* und *Schlichtstrahler* nennen, weil Kurzstrahler zugleich in Folge der moosigen, gewundenen und verästelten Beschaffenheit der Fortsätze ein gekraustes, die Langstrahler dagegen in Folge des geraden oder gestreckten Verlaufes ein schlichtes oder schlichthaariges Aussehen darbieten. Diese Benennungen reichen indessen nicht hin, um die verschiedenen Typen der Neurogliazellen zu charakterisiren, sondern sie sind eher als eine Classifikation der Typen zu betrachten, bei welcher noch Unterabtheilungen wünschenswerth sind. In Folge der vielen Formwechselungen ist es jedoch schwer, ganz zutreffende Benennungen zu finden.

Von Interesse ist es nun, die Neurogliazellen der Grosshirnrinde mit denen des Rückenmarks zu vergleichen, wie sie M. von Lenhossék[1] und *ich*[2] beschrieben haben. In der That lassen sich diejenigen des Gehirns auf die des Rückenmarks zurückführen. Es ist eine auffallende Uebereinstimmung der Typen vorhanden. Die Kurz- oder Krausstrahler der grauen Rückenmarksubstanz sind denen der grauen Substanz der Hirnrinde ähnlich; die des Rückenmarkes bieten zwar vielleicht mehr von der »Sternform» abweichende Varianten dar, im Ganzen sind sie jedoch von einem und demselben Typus. Im Inneren des Rückenmarks kommen dann auch langstrahlige »Sternzellen», und zwar ventral vom Centralkanal, in der vorderen weissen Commissur vor; diese für die Marksubstanz charakteristische Art von Zellen kommt nun überall in der weissen Substanz des Gehirns vor. Aber auch die oberflächlicheren Neurogliazellen des Rückenmarks, die die Stränge durchlaufen und entweder mit den Zellkörpern oder mit von ihnen ausgehenden Fortsätzen die Oberfläche erreichen, sind den entsprechenden Elementen der Grosshirnrinde vergleichbar. Beide schicken vom inneren Ende des Zellkörpers ein Büschel langer schlichter Fortsätze nach innen hin, obwohl diese Zellen im Gehirn als etwas regelmässiger geformt erscheinen; im Rückenmark giebt es auch, wie im Gehirn, Zellen, deren Körper etwas unter der Oberfläche liegen und eine Anzahl von Fortsätzen nach derselben hin schicken. Wenn man berücksichtigt, dass es auch in der äussersten Schicht der Gehirnrinde eine weisse Marklage giebt, so ist die Uebereinstimmung auffallend; in der Gehirnrinde senden aber die langstrahligen Zellen ihre Fortsätze tief in die grauen Schichten hinab.

Die an der Oberfläche der Hirnrinde befindlichen Zellkörper, welche ein Büschel langer, gestreckter Fadenfortsätze in die unterliegenden Schichten senden, hat offenbar Lloyd Andriezen gesehen; er giebt von ihnen eine

[1] M. von Lenhossék, Zur Kenntniss der Neuroglia des menschlichen Rückenmarks. Verh. d. Anatom. Gesellsch. auf d. fünften Versamml. in München, 1891.
[2] Gustaf Retzius, Studien über Ependym und Neuroglia, Biolog. Unters. v. G. Retzius. N. F., Bd V, 2, 1893.

ziemlich zutreffende Abbildung (Fig. 7 seiner oben angeführten Abhandlung) und nennt sie »caudate glia cells». Diese Benennung, »geschwänzte Zellen» oder Schwanzzellen, trifft recht gut zu, obwohl sie nicht die anderen Eigenschaften der Zellen, die Belegenheit des Zellkörpers an oder neben der Oberfläche und das Vorhandensein der von ihnen ausgehenden tangentialen Fortsätze, welche das Filzwerk von Gliafasern an der Hirnoberfläche bilden, berücksichtigt.

Wie ich oben hervorgehoben habe, ist es für die Beschreibung der Neuroglia- oder, kürzer ausgedrückt, Gliazellen (Gliäcyten) wichtig, Benennungen der verschiedenen Typen derselben festzustellen. Da ich stets ungern neue Namen einführe, sobald brauchbare schon von anderen Forschern vorgeschlagen sind, so werde ich im Anschluss an die von von Kölliker und Lloyd Andriezen angewendeten nur das mir nöthig Erscheinende hinzufügen, um die wesentlichen Typen bezeichnen zu können. Ich unterscheide also die *Sternstrahler* (*asteroide Gliäcyten*) und die *Schwanzstrahler* (*ureide Gliäcyten*),[1] je nachdem die Fortsätze nach *allen* Richtungen hin ausstrahlen oder vorwiegend nach *einer* Richtung hin verlaufen. Die ersteren entsprechen mithin den *Deiters'schen* oder *Golgi'schen Neurogliazellen* (den *Spinnenzellen, Sternzellen* der Autoren). Die letzteren sind *»the caudate glia cells»* Lloyd Andriezen's. Bei den Sternstrahlern unterscheide ich dann nach von Kölliker *Kurzsternstrahler* und *Langsternstrahler* (*macroasteroide* und *brachyasteroide Gliäcyten*). Bei den Schwanzstrahlern in gleicher Weise *Langschwanzstrahler* und *Kurzschwanzstrahler* (*macroureide* und *brachyureide Gliäcyten*). In diese Gruppen können nun die meisten Gliäcyten eingeordnet werden. Es giebt aber noch einige abweichende Formen, die nicht ohne Zwang unter diesen Bezeichnungen aufgeführt werden können. Erstens giebt es den schon oben mehrfach beschriebenen vierten Typus von Gliäcyten in der Grosshirnrinde, die nämlich, welche ihren Zellkörper etwas unter der Oberfläche belegen haben und von ihm aus eine Anzahl Fortsätze an dieselbe schicken, die dort mit Knötchen oder Füsschen endigen; diese Zellen sind in der Regel »sternförmig», Sternstrahler, aber die nach der Oberfläche gehenden Fortsätze sind länger und dort mit Endfüsschen versehen. Solche Zellen kommen auch in der Nähe von Blutgefässen vor, wo sie mit ihren konisch angeschwollenen Füsschen anheften. Ich werde sie *Fuss-Sternstrahler* (*podasteroide Gliäcyten*) nennen. Daneben kommen Gliazellen vor, welche nicht nur nach einer, sondern nach zwei Richtungen hin ungefähr ebenso stark entwickelte lange »Strahlschwänze» aussenden und in ihrer Gesammtform gewissermassen einer Sanduhr ähneln. Ich finde es aber richtiger, keinen neuen Gesichtspunkt einzuführen, sondern sie (nach den »caudate glia cells») als *Doppelschwanzstrahler* (biureide Gliäcyten) aufzuführen. Endlich giebt es Gliäcyten, welche *nur* flächenförmig ausgebreitet sind; solche Elemente kommen hier und da an der Oberfläche der Hirnrinde vor, und zu dieser Kategorie gehören auch die von Lloyd Andriezen beschriebenen, scheidenförmig um die Blutgefässe angeordneten Elemente; sie breiten sich also theils mehr in einer planen, theils mehr in einer gekrümmten Fläche aus. Ich werde sie als *Flächenstrahler* (plakoide Gliäcyten) bezeichnen; dieselben sind also entweder mehr plan, oder mehr tubulär.

Dagegen finde ich es jetzt nicht nöthig, die übrigen abweichenden Formen mit besonderen Namen zu belegen, vor Allem nicht die oben beschriebenen embryonalen und foetalen Typen, ebenso wie ich auch die verschiedenen Formen der Ependymzellen, resp. Fasern und ihre Uebergangsformen zu Gliäcyten nicht durch besondere Bezeichnungen differenziren will, obwohl es in der Zukunft wahrscheinlich gethan werden wird.

In Zusammenhang mit dieser Namenerörterung kann ich nicht umhin, auch in Betreff der Benennung der verschiedenen Schichten der Grosshirnrinde einige Worte zu äussern. Die äusserste Schicht wird seit Alters her als die erste, die zellenarme oder die Molekularschicht (die feinkörnige Schicht) bezeichnet. Keine dieser Bezeichnungen ist nunmehr passend. Meiner Ansicht nach ist hier der einzige gute Eintheilungsgrund die Art der in den verschiedenen Schichten befindlichen Nervenzellen, welche doch für eine jede das am meisten Bestimmende ist, wie man dies auch schon seit lange für die Pyramidenzellenschichten allgemein angenommen hat. Für die äusserste Schicht sind nun gerade die nervösen Zellen derselben, die Cajal'schen, das am meisten Charakteristische. Diese Zellen finden sich mit ihren Fortsätzen in verschiedenen Niveaus von der Oberfläche bis zur Kleinpyramidenzellenschicht und treten sogar, nach Allem was man bis jetzt kennt, nicht in diese Schicht hinein; die Cajal'schen Zellen mit ihren Fortsätzen haben also nur die äusserste Schicht ein, durchspinnen sie aber, vor Allem beim Menschen, in allen Richtungen. Ich finde es also sehr angemessen, diese Schicht nach den genannten Zellen

[1] In gewisser Hinsicht ähnelt ihre Form oft Kometenschwänzen, und vielleicht wird Jemand, um die astronomischen Beziehungen zu den Sternen aufrecht zu halten vorschlagen, die fraglichen Gebilde »Kometenzellen» zu nennen!

artige Disposition der Ependymzellen in der vorderen und hinteren Commissur sowie in den Seitenpartien des Rückenmarkes genauer und lieferte eine gute Abbildung des Rückenmark-Querschnittes des Hühnerembryos v. 9. Bebrütungstage; er erwähnt an den Rändern der einzelnen Ependymfäden kleine stachelige Aeste und eine zuweilen vorkommende Verzweigung des peripherischen Endes derselben, sowie konische Verdickungen dieser Enden unter der Pia mater, und er hatte sogar gesehen, dass das innere Ende sich zuweilen in ein in den Centralkanal einschiessendes, feines und langes Haar verlängert. Vom 8. Bebrütungstage an, sagt Cajal, bemerkt man zwischen den Ependymelementen stets gewisse Gebilde, die kürzer sind und den Centralkanal nicht erreichen; sie rühren von einer Dislocation und vielleicht von einer Proliferation der anderen Ependymelemente her und stellen die Vorstufen der Spinnenzellen dar. Je älter der Embryo ist, um so kürzer und verzweigter erscheinen die die Seitenpartien des Rückenmarks radiär durchziehenden Ependymfäden. Die Neuroglia- oder Spinnenzellen sind im Rückenmarke der Hühnerembryonen vom 9. oder 10. Tage an erkennbar; sie treten zuerst im Vorderhorn auf, bald danach findet man sie in der weissen Substanz der Stränge und zuletzt in den Hinterhörnern. Die Spinnenzellen sind nichts Anderes als dislocirte und sehr umgewandelte Ependymelemente; vom 7. bis 14. Tage trifft man unter ihnen alle Uebergänge sowohl in Betreff der Form wie der Lage. Man bemerkt sogar im Marke erwachsener Säugethiere Neurogliazellen, an denen man einen centralen Faden (Ependymfortsatz) weit durch die graue Substanz hindurch verfolgen kann und die noch einen oder mehrere peripherisch radiirende Fortsätze besitzen. Im Grosshirn und Kleinhirn sind orientirte Neurogliazellen nicht selten: die Radialzellen der Molekularschicht des letzteren stellen ein gutes Beispiel davon dar. Die leucocytische Herkunft der Neurogliazellen ist nicht annehmbar. Diese Zellen anastomosiren übrigens nicht mit einander, sondern sie sind vollständig selbstständige Elemente. — In einigen seiner folgenden Arbeiten bespricht Cajal ausserdem gelegentlich die Ependym- und Neurogliazellen anderer Partien der Centralorgane, z. B. die der Kleinhirn- und Grosshirnrinde.

In seiner Arbeit über das Rückenmark bespricht auch von Kölliker[1] die Neurogliafrage. Das Mark junger Embryonen besitzt, sagt er, anfänglich nur einerlei Gliazellen, die nichts Anderes sind, als die sogenannten Epithelzellen des Centralkanals, die mit ihren Ausläufern radienartig das ganze Mark durchziehen und an der Oberfläche desselben dicht an der Pia mit grösseren oder kleineren Verbreiterungen enden. Hierbei zeigen die längeren dieser Elemente, die alle nur *einen* Kern dicht am Centralkanale besitzen, in ihren äusseren Theilen spitzwinklige Verästelungen und viele Seitenästchen, so dass der Anschein eines Netzes erzeugt wird, ohne dass ein solches wirklich vorhanden wäre. Das erste Auftreten dieser Gliazellen, das besonders His und auch Vignal verfolgt haben, ist bei jungen Embryonen leicht zu sehen und führt auf einen Theil der Zellen der Medullarplatte zurück, die zu Faserzellen auswachsen und zugleich Seitenausläufer treiben. Das ursprüngliche Verhalten der Gliazellen erhält sich längere Zeit, und v. Kölliker fand es noch bei einem Schafembryo von 9 Cm. Länge und einem Schweineembryo von 10 Cm. Länge. Bei älteren Embryonen und nach der Geburt erhalten sich diejenigen dieser Elemente vollständig, die nach dem Grunde der ventralen Spalte des Markes und nach der dorsalen Mittellinie gehen; dagegen werden die anderen, bis auf die um den Centralkanal gelegenen Theile, nach und nach undeutlich, und es treten in allen Theilen des Markes eine Menge neuer Gliazellen, die bekannten sternförmigen Elemente auf, die sich unzweifelhaft allmählich aus den indifferenten Zellen der Markanlage entwickeln, welche, so lange dieses Organ nicht ausgebildet ist, in Form rundlicher Zellen in grosser Menge in der weissen und grauen Substanz zwischen den nervösen Elementen vorhanden sind und später sich nicht mehr nachweisen lassen.

M. von Lenhossék[2] stellte etwa gleichzeitig mit Cajal Untersuchungen über die Entwicklung des Rückenmarks des Hühnerembryos an. Er beschrieb die Entwicklung der Ependymzellen oder »Radiärfasern», die sich schon am dritten Tage imprägniren lassen; dieselben sind von Anfang an mit zahlreichen, ganz minimalen, unter rechtem Winkel abgehenden Fädchen und Unregelmässigkeiten besetzt, die in einer späteren Phase grössere Entfaltung gewinnen, indess nur an deren innerem, der grauen Substanz angehörendem Abschnitt vorhanden sind. Theilungen treten früh auf, und zwar erfolgen sie aussen, im Bereich der weissen Belegschicht, oder nahe derselben, und erscheinen zuerst in Form einfacher dichotomischer Spaltungen, um sich allmählig complicirter zu gestalten. Die

[1] A. von Kölliker, Zur feineren Anatomie des centralen Nervensystems. Zweiter Beitrag: Das Rückenmark. Zeitschr. f. wissensch. Zoologie. Bd 51, 1890.

[2] Mich. von Lenhossék, Zur ersten Entstehung der Nervenzellen und Nervenfasern bei dem Vogelembryo. Mittheil. aus dem anatom. Institut im Vesalianum zu Basel, 1890.

## B. Die Neuroglia der Grosshirnrinde des Hundes, der Katze und des Kaninchens.

(Taf. VI—VIII.)

Des Vergleiches mit den Verhältnissen beim Menschen wegen habe ich auch in dieser Hinsicht bei mehreren Säugethieren Untersuchungen angestellt, vor Allem bei dem Hund, der Katze, dem Kaninchen, der Ratte und der Maus, aber auch bei dem Ochsen, dem Schaf und dem Schwein. Hier theile ich indessen nur meine Befunde bei dem Hunde, der Katze und dem Kaninchen mit. Eine ausführliche Beschreibung finde ich nicht nöthig, da die beigegebenen Figuren die Verhältnisse besser veranschaulichen als die wörtliche Schilderung.

Beim *Hunde* (Taf. VI) beschränke ich mich auf eine Darstellung der Gliäcyten aus dem 1. und 2. Monate nach der Geburt und im erwachsenen (alten) Zustande. Schon bei zwei früheren Gelegenheiten habe ich die wichtigeren Typen beschrieben und abgebildet. Von der hier unten auf der Taf. VI mitgetheilten Figuren stellt die Fig. 1 die Rindenpartie eines 2-monatlichen und die Fig. 3 die eines 1-monatlichen Hundes dar. Die Fig. 2 giebt drei Gliazellen aus der Marksubstanz des 2-monatlichen Hundes wieder. In diesen Figuren erkennt man ohne Schwierigkeit die oben beim Menschen dargelegten Typen, obwohl beim Hunde gewisse Differenzen scharf hervortreten. Also haben wir hier im Inneren die *Sternstrahler,* nämlich die Kurzstrahler in der grauen Substanz (Fig. 1) und die Langstrahler (Fig. 2), obwohl hier die Benennungen Kraus- und Schlichtstrahler vielleicht passender wären. Die Kurzstrahler haben nämlich ein ausgeprägt krauses oder sogar moosiges Aussehen, während die Langstrahler der weissen Substanz meistens schlichte, gerade, feine Fortsätze zeigen, die oft keine bedeutende Länge haben. Von den die Oberfläche erreichenden Gliäcyten sind Fusssternstrahler beim Hunde besonders schön ausgebildet; die Fig. 1 und 3 stellen mehrere Beispiele von ihnen dar; man sieht hier von dem mit zahlreichen feinen Aesten versehenen Zellkörper, der im Ganzen ein moosiges Aussehen darbietet, die nach der Oberfläche ziehenden stärkeren Fortsätze ausgehen und, oft mit feinen, verzweigten Aestchen besetzt, an der Oberfläche selbst mit Knöpfchen oder konischen Füssen endigen; ausserdem sendet der Zellkörper auch oft einen Fortsatz oder einen moosigen Zopf nach unten hin ab. Die Schwanzstrahler sind auch vorhanden, obwohl sie eine etwas abweichende Gestalt zeigen, indem manche der an der Oberfläche belegenen breiten, knopfförmigen Zellkörper eine Anzahl von stärkeren, aber nicht besonders langen, oft moosig verästelten Fortsätzen entsenden, während andere nur einen Büschel kurzer moosiger Aeste abgeben. Eigenthümliche Formen mit seitlich gelegenen, an der Oberfläche endenden Fortsätzen kommen auch vor (Fig. 3).

Bei der Betrachtung der von der Pia vorsichtig befreiten Hirnoberfläche von aussen (oben) her erkennt man hier und da die gefärbten, an der Oberfläche belegenen Zellkörper der Schwanzstrahler und sieht von ihnen oft Fortsätze nach den Seiten hin ausstrahlen (Fig. 4, *a*, links); die Zellkörper sind rundlich-oval, zuweilen etwas eckig. Hier und da trifft man nun Partien der Oberfläche, an welchen nicht nur einzelne Zellkörper oder Gruppen von ihnen braunschwarz gefärbt sind, sondern auch zwischen denselben eine epithelioide Mosaikzeichnung (Fig. 4, *b*, rechts), deren rundlich-polygonale Felder verschieden gross sind. Ich kann nach genauer Betrachtung einer Anzahl solcher Präparate diese Mosaikzeichnung nicht anders deuten, als dass die Felder den Zellkörpern und den Fortsatzenden, Füssen, der an der Oberfläche befindlichen Zellkörper entsprechen. Es ist nicht unmöglich, dass es gerade dieses Mosaik gewesen ist, welches zuweilen von Forschern für eine epitheliale (endotheliale) Membran genommen wurde. Wenn man bedenkt, wie die Struktur hier gestaltet werden muss, kann es kaum etwas anders sein als eine Mosaikanordnung, indem ja eine dichte Masse von rundlich polygonalen Zellkörpern und zwischen ihnen befindlichen Füssen der Fusssternstrahler gerade die Oberfläche bildet. Bei starker Färbung mittelst der Golgi'schen Methode werden so viele dieser Endplatten geschwärzt, dass man das Mosaik nicht zu dechiffriren vermag, bei der schwächeren Färbung aber, wo die meisten Endplatten nur in ihren Contouren hervortreten (Fig. 4, *b*), liegt das Bild klar und deutlich vor. An Vertikalschnitten sieht man oft Gruppen von stark gefärbten Zellen (Fig. 3, rechts), wo die Grenzen der einzelnen Zellen nur schwer demonstrirbar sind. Man kann aber leicht fassen, dass sie zusammen ein Mosaik an der Oberfläche bilden müssen. An solchen Vertikalschnitten bemerkt man bei festsitzender Pia hier und da auch, dass diese Zellenkörperflächen der Pia dicht anliegen; zuweilen ragen sie aber, wie beim Menschen, knopfförmig über die Oberfläche empor, indem sie mit ihrer Endfläche einem Blutgefäss der Pia anhaften (Fig. 3, links).

Wenn wir dann nachsehen, wie sich die Verhältnisse im ausgebildeten Hundegehirn gestalten, so finden wir, wie die Fig. 5 und 6 der Tafel VI darthun, dass sie denen des jüngeren Stadiums sehr ähnlich sind. Die Schwanzstrahler sind aber etwas weiter entwickelt, zeigen nicht selten bedeutend breite, obere Zellkörperplatten, haben oft etwas längere und schlichtere Fortsätze als früher und sind weniger »moosig». Ausserdem trifft man hier an der Oberfläche tangentiale Fortsätze der Schwanzstrahler und auch besondere Flächenstrahler, obwohl diese tangentialen Faserausbreitungen nicht die starke Ausbildung zeigen wie im menschlichen Gehirn. Die Sternstrahler sind im ausgebildeten Hundegehirn denjenigen der jüngeren Altersperiode sehr ähnlich gestaltet.

Die in den Figuren abgebildeten Gliäcyten können zwar einen allgemeinen Begriff von den im Hundegehirn vorkommenden Formen darbieten; sie repräsentiren aber nicht alle Formen, denn es giebt eine Menge von Varianten, vor Allem in der Oberflächenschicht.

Auf derselben Tafel habe ich noch eine Figur (Fig. 7) mitgetheilt, die die äusseren Enden der Ependymzellen von einem neugeborenen, oder richtiger 5 Tage alten, Hunde vorstellt. Ich habe früher (Biol. Unt., N. F. B. V., Taf. I, Fig. 1) solche Zellen von einem 14 Cm. langen Hundefoetus veröffentlicht. Ich meinte, es könne von Interesse sein, zu zeigen, dass diese Fasern noch nach der Geburt des Hundes darstellbar sind und wie sie sich dann verhalten. Wie man sieht, haben sie hier denselben Typus, sind vielleicht nur etwas stärker entwickelt und ragen mit ihren knopfförmigen Enden bis an die Oberfläche hinan; zwischen ihnen sind drei Gliäcyten abgebildet, welche sämmtlich eigenthümliche Formen darbieten.

Bei der *Katze* (Taf. VII) findet man im ausgebildeten Stadium (Fig. 4—6) denjenigen des Hundegehirns sehr ähnliche Verhältnisse. Die Gliäcyten sind relativ gross und kräftig entwickelt, und die Elemente der Oberflächenschichten bieten im Allgemeinen ein ausgeprägt »mooisges» Aussehen dar. Uebrigens erkennt man hier ungefähr dieselben Formen wie im Hundegehirn, so dass ich auf eine eingehendere Beschreibung verzichte und auf die Abbildungen hinweise. Hier bemerkt man den scharfen Unterschied zwischen den Kurzsternstrahlern der grauen Substanz (Fig. 4) und den Langsternstrahlern der weissen Substanz (Fig. 5), ferner die verschiedenen Formen der Schwanzsternstrahler (Fig. 4) und die Fusssternstrahler, die jedoch in der Fig. 4 nur schwach repräsentirt sind. Von der Oberfläche betrachtet bieten die Zellenkörper der Schwanzstrahler (Fig. 6, 7, 8) mit ihren Fortsätzen ähnliche Gestalten dar; nur trifft man hier oft eigenthümliche, tropfförmige Vorsprünge, Unregelmässigkeiten und Einschnürungen der Endplatten (Fig. 7). Solche Schwanzstrahler sind auch an grösseren, in die Hirnsubstanz hineindringenden Blutgefässen vorhanden (Fig. 9), wo die Zelle von der Seite her gesehen wird.

In jüngeren Stadien, in den ersten beiden Wochen nach der Geburt, bei der 15 Tage (Fig. 3), der 13 Tage (Fig. 2) und der 8 Tage alten Katze (Fig. 1) findet man viel schwächer ausgebildete Gliäcyten. Zwar erkennt man die Haupttypen; manche der fraglichen Elemente zeigen aber noch gewissermassen einen embryonalen Habitus; dies ist besonders bei dem 8 Tage alten Thiere der Fall, aber noch bei dem 15 Tage alten sind Formen vorhanden, die später verschwinden oder modificirt werden. In der Fig. 2 habe ich auch die äusseren Enden zweier Ependymzellenfasern wiedergegeben, weil es von Interesse ist zu sehen, dass, wie beim Hunde, diese Elemente noch so lange nach der Geburt in ihrer foetalen Gestalt nachweisbar sind.

Beim *Kaninchen* (Taf. VIII) ähneln die Gliazellen der Grosshirnrinde, dem Hauptcharakter nach, ebenfalls denjenigen des Hunde- und Katzengehirns. Die »moosige» Beschaffenheit derselben tritt aber noch prägnanter hervor. Auch hier erkennt man die beim Menschen vorkommenden Typen, obwohl in ziemlich modifizirter Gestalt. Die Sternstrahler behalten aber auch hier ihr typisches Aussehen; von ihnen sind indessen in den Figuren (Fig. 1, 4, 7) nur Kurzsternstrahler abgebildet. Die Fusssternstrahler sind in der Hirnrinde des Kaninchens stark vertreten (Fig. 1, 4) und bieten manche interessante Varianten dar; bald ist nur ein solcher Fussfortsatz vorhanden; bald sind mehrere da, und zwar von sehr verschiedener Länge, je nachdem der Zellkörper mehr oder weniger tief unter der Oberfläche liegt. Der Zellkörper dieser Zellen ist oft mit einer Unzahl nach allen Richtungen ausstrahlender, feiner, moosiger Fortsätze versehen, so dass das Ganze wie ein schwarzer moosiger Ballen aussieht, in dessen Bau das Auge nicht einzudringen vermag (Fig. 1). Die mit ihrem Zellkörper an der Oberfläche liegenden Schwanzstrahler sind oft sehr breit, die Fortsätze des Schwanzes sind aber nicht besonders lang und in der Regel mit vielen kleinen Aestchen besetzt. Es giebt aber auch Schwanzstrahler, deren Zellkörperplatte klein ist, so dass das Oberflächenmosaik dieser Platten und der zwischen ihnen liegenden Füsse der Fusssternstrahler-

fortsätze grosse und kleine Felder in bunter Mischung enthält. Tangentiale Fortsätze der Schwanzstrahler sind sehr sparsam vorhanden (Fig. 7).

In jüngeren Stadien, beim neugeborenen Thier und in der ersten Woche nach der Geburt präsentiren die Gliäcyten theilweise noch foetale Charaktere. Dies ist besonders der Fall in den ersten beiden Tagen nach der Geburt, wie die Fig. 3 von einem $1^1/_2$ Tage alten Jungen darthut, wo Gliäcyten vorliegen, welche denen des Menschen aus der späteren Hälfte der Foetalperiode ähnlich sind. Schon bei dem 4 Tage alten Jungen sind die Elemente in mehr ausgebildetem Zustande vorhanden. Beim 14 Tage alten Kaninchen (Fig. 1) sind sie, wie oben beschrieben wurde, schon ziemlich fertig, sowie sie später beim alten Thier (Fig. 4 und 7) vorliegen. In den Fig. 2 und 3 sind des Vergleiches wegen auch Cajal'sche Zellen wiedergegeben.

## C. **Die Neuroglia der Grosshirnganglien, der Medulla oblongata und der Insula Reilii des Menschen** (Taf. IX).

Im Anschluss an die Darstellung der Neuroglia der Rinde der Grosshirnhemisphären wäre es von besonderem Interesse, alle die in ihrem Bau abweichenden Theile des Gehirns zu untersuchen. Es liegt indessen nicht in dem Plane dieser Arbeit, eine so umfassende Untersuchung diesmal auszuführen, sondern nur eine übersichtliche Darstellung der vorhandenen Gliaelemente zu geben. Deswegen werde ich mich darauf beschränken, die in den grösseren Grosshirnganglien vorkommenden Typen nachzuweisen und kurz ausserdem diejenigen der Hirnrinde in der Insula Reilii zu besprechen.

Ich beginne mit den *Corpora quadrigemina*. In der Fig. 1 der Taf. IX habe ich einen vertikalen Frontalschnitt des Corpus quadrigeminum anterius eines 42 Cm. langen Menschenfoetus dargestellt. Man trifft hier die oben aus der Hämisphärenrinde beschriebenen Typen wieder. Unten in der Figur sind Sternstrahler abgebildet, und zwar Kurzsternstrahler. An der Oberfläche des Hirntheils sieht man die Zellkörper von Schwanzstrahlern, die theils an der Oberfläche selbst, theils etwas unter ihr liegen und eine Anzahl kurzer, trichterförmig divergirender Fortsätze an die Oberfläche schicken, welche sich theilweise in der Oberflächenschicht ausbreiten und sich durch einander weben. Diese Schwanzstrahler senden nach unten (innen) bald kürzere, bald längere, ja sogar sehr lange Büschel von Fortsätzen aus; hier sind also sowohl Kurzschwanzstrahler wie Langschwanzstrahler vorhanden. Etwas tiefer unter der Oberfläche kommen ferner Schwanzstrahler vor, die mit ihren oberen Fortsätzen die Oberfläche nicht erreichen. Uebergangsformen zwischen den Schwanzstrahlern und den Sternstrahlern sind auch vertreten. Die nach der Oberfläche strebenden Fortsätze sind in der Regel mit Knoten besetzt.

In den *Corpora quadrigemina posteriora* sind dieselben Typen der Gliäcyten vorhanden.

Bei der Untersuchung der Rindenschichten der *Thalami optici* trifft man nun auch die beschriebenen Typen der Gliaelemente. In der Fig. 2 habe ich einen vertikalen Frontalschnitt des Putamen wiedergegeben. Sternstrahler sind im Inneren des Theiles vorhanden, und Schwanzstrahler liegen mit ihren Körpern an oder dicht unter der Oberfläche, ihre langen Schwänze tief nach innen (unten) sendend. Hier bemerkt man noch (rechts in der Figur) einen Fusssternstrahler, welcher die äusseren Fortsätze bis an die Oberfläche, zugleich aber auch einen Schwanzbüschel nach innen schickt, also eine Uebergangsform zwischen Fusssternstrahlern und Schwanzstrahlern bildet.

Aus den *Corpora striata* habe ich bis jetzt keine guten Präparate erhalten, und ich theile deshalb nur des Vergleiches wegen eine kleine Partie (Fig. 3) eines Vertikalschnittes aus dem Gehirn eines alten (65-jährigen) Mannes mit, welche einige der in meinen fraglichen Präparaten vorkommenden Gliazellen darbietet. Die Sternstrahler im Inneren sind schön ausgebildet; die der Oberfläche näher belegenen Elemente mit ihren steif ausstrahlenden Fortsätzen sind eigenthümlich und bedürfen eine weitere Untersuchung.

Ferner habe ich auch des Vergleiches wegen auf derselben Tafel (IX) eine Rindenpartie eines Querschnitts der *Medulla oblongata* vom menschlichen Foetus im 7. Monate wiedergegeben, um die in diesem Gehirntheil vorkommenden wichtigeren Typen der Gliazellen zu zeigen. Wie von vornherein anzunehmen war, stimmen diese Elemente vor Allem mit denen des Rückenmarks überein, wie sie von M. von Lenhossék und *mir* früher geschildert worden sind. In den grauen Partien im Inneren sind Kurzsternstrahler, in den weissen aber Langsternstrahler vor-

handen, und an der Oberfläche oder dicht unter ihr befinden sich die Zellkörper von zahlreichen Schwanzstrahlern, welche kurze Fortsätze nach oder an der Oberfläche aussenden und die langen Schwanzbüschel centralwärts abgeben. Bei stärkerer Färbung dieser Schwanzstrahler bekommt man ein so dichtes Gewirr von Zellkörpern und Fasern, dass sie nur schwer dechiffrirbar sind, wie oben in der Figur ersichtlich ist. Obwohl also die Gliazellen der Rinde und des Inneren der Medulla oblongata denjenigen des Rückenmarks sehr ähnlich sind, so lassen sich jedoch, wie man leicht findet, auch Vergleichspunkte mit denjenigen der Hirnganglien und sogar der Hemisphärenrinde nachweisen.

In den Querschnitten von der Medulla oblongata der menschlichen Foetus bekommt man oft gute Färbung der Elemente der *Oliven*. Da es mir von Interesse zu sein schien, auch die Gliazellen dieses Gehirntheils zu demonstriren, theile ich, auf der Taf. X eine Abbildung desselben obwohl bei schwächerer Vergrösserung, mit (Fig. 7). Wie aus dieser Figur hervorgeht, sind fast überall nur Langstrahler (Schlichtstrahler) vorhanden; jedoch bemerkt man unter denselben auch einzelne Kurzstrahler (Krausstrahler).

Von den übrigen Hirntheilen des menschlichen Gehirns werde ich hier kurz nur die Rinde der *Insula Reilii* berühren, und zwar nur in der Absicht zu zeigen, dass hier echte Rindenverhältnisse auch in Betreff der Gliaelemente vorhanden sind. Ich habe eine Abbildung (Fig. 5 der Taf. IX) der foetalen Insula gewählt, um die sich entwickelnden Gliazellformen auch in dieser Region darzulegen. Beim Vergleich mit den Abbildungen aus der übrigen Hemisphärenrinde findet man leicht die Uebereinstimmung der Gliazellformen; hier ist ausserdem auch das Ansetzen eines Gliazellfortsatzes mittelst eines grossen Knöpfchens an ein Blutgefäss (*bg*) sichtbar und dann noch eine Anzahl tangentialer Fasern mit vertikal aufsteigenden Aesten vorhanden; keine ihnen angehörigen Cajal'schen Zellen waren aber im Sehfelde nachzuweisen.

## D. Die Neuroglia der Grosshirnganglien, des Gyrus Hippocampi und des Fornix der Katze (Taf. X, Fig. 1—6).

Da es für die Kenntniss der Verhältnisse beim Menschen von Interesse ist, die entsprechenden Verhältnisse bei einem anderen Säuger zu betrachten, habe ich die Grosshirnganglien der Katze in den Kreis dieser Untersuchungen gezogen, und ich theile nun auf der Tafel X einige Abbildungen von vertikalen Frontalschnitten dieser Ganglien mit. Fig. 1 stellt die obere Partie eines *Corpus quadrigeminum anterius,* Fig. 2 die eines *Corpus quadrigeminum posterius,* Fig. 3 die eines *Corpus geniculatum* zweiwöchentlicher Katzen dar. In den Fig. 1 und 2 erkennt man die fast überall vorhandenen Haupttypen der Gliazellen, und zwar im Inneren die Kurzstrahler, in der Nähe der Oberfläche dagegen die Schwanzstrahler, die sowohl kurze wie lange Schwanzbüschel und theilweise auch eine tangentiale Ausbreitung der oberen Fortsätze zeigen. Ausserdem sind auch verschiedenartig gestaltete Fusssternstrahler vorhanden, von denen einige (besonders in Fig. 1) ausserordentlich lange äussere Fussfortsätze zeigen.

In der die Gliaelemente des Corpus geniculatum darstellenden Fig. 3 sind sowohl Kurzsternstrahler und Fusssternstrahler, wie auch Schwanzstrahler vorhanden, diese letzteren haben aber nur kurze Schwanzbüschel und zeigen nicht den gewöhnlichen, charakteristischen Typus.

Zu diesen Figuren aus den Ganglien füge ich, um diesen wichtigen Gehirntheil nicht ganz zu übergehen, noch ein paar Abbildungen aus dem Gyrus Hippocampi der Katze mit. In Betreff derselben und der Fascia dentata hat Ramón y Cajal schon eine sehr schöne Darstellung der Gliaelemente gegeben, so dass ich auf eine nähere Beschreibung nicht eingehen will, um so weniger, da er, besonders aus der Fascia dentata, weit bessere Abbildungen mitgetheilt hat. Was ich aber hier zu berücksichtigen wünsche, ist das Gliagewebe der Rinde des unteren Umfangs des Gyrus Hippocampi, von welcher die Fig. 4 einen Vertikalschnitt wiedergiebt. Man erkennt hier auch die, obwohl theilweise eigenthümlich gestalteten, Kurzsternstrahler und die Langschwanzstrahler in verschiedenen Varianten, auch solche, welche tief unter der Oberfläche liegen und lange Fortsätze nach ihr schicken, die sie nur zum Theil erreichen. Endlich füge ich in der Fig. 6 noch die Partie eines Querschnitts vom Fornix bei, in der seine etwas eigenthümlich gestalteten Gliazellen dargestellt sind. Gewissermassen lassen sich auch diese Elemente zu den genannten Haupttypen hinführen, obwohl sie verkümmert erscheinen.

## II. Die Neuroglia des Kleinhirns.

(Taf. XI.)

### A. Die Neuroglia des Kleinhirns des Menschen.

In der obigen geschichtlichen, die Neuroglia des Grosshirns betreffenden Darstellung, welche die Angaben der neueren Forscher, die mit der Golgi'schen Methode gearbeitet haben, enthält, habe ich auch diejenigen über die Neuroglia des Kleinhirns angeführt. Die von Bergmann entdeckten und nach ihm benannten Fasern der Kleinhirnrinde waren zwar längst von anderen Forschern constatirt und auch von *mir* und Key (1875) eingehend beschrieben worden. Ebenso hatte man in der Marksubstanz des Kleinhirns lange das Vorhandensein von Neurogliazellen gekannt. Die genauere Kenntniss der Gestaltung dieser Zellen und der eigentlichen Bedeutung der Bergmann'schen Fasern datirt aber von der Einführung der Golgi'schen Chromkali-Silbermethode her. Golgi wies in seiner grossen Arbeit (Sulla fina anatomia etc. 1885) den büschelförmigen Ursprung der fraglichen Fasern von Zellkörpern nach, die an der inneren Grenze der sog. Molekularschicht liegen, und legte zugleich die sternförmige Gestalt der Gliazellen der Marksubstanz dar (s. seine erwähnte Arbeit, Taf. XII, sowie die Taf. 16 der deutschen Sammelausgabe, 1894). Seitdem haben mehrere Forscher dasselbe Thema behandelt, unter denen besonders Ramón y Cajal, Van Gehuchten und von Kölliker genannt werden sollen. Selbst habe ich auch zwei mal (in dem Jahre 1891 beim Menschen und 1892 bei der Katze) das Verhalten der Bergmann'schen Faserzellen dargestellt. Und neulich, in diesem Jahre, hat Van Gehuchten eine eingehendere Darstellung der fraglichen Zellen des menschlichen Kleinhirns geliefert.

Obwohl wir also schon die wichtigeren Data in Betreff der Neurogliaelemente des Kleinhirns kennen gelernt haben, werde ich doch die Ergebnisse meiner schon im vorigen Jahre gemachten, hierauf bezüglichen Untersuchungen kurz mittheilen und einige meiner Abbildungen beifügen, theils weil bei einer übersichtlichen Darstellung der Neurogliaelemente des Gehirns diejenigen des Kleinhirns nicht unberücksichtigt bleiben dürfen, theils auch deshalb, weil ich einige bisher nicht hinreichend beachteten Entwicklungsstadien dieser Elemente (im frühen Stadium und im ausgebildeten Zustande) beim Menschen studirt habe, und dann auch weil die zwischen ihren äusseren Enden befindlichen mystischen Körner immer noch neue Besprechungen erfordern.

### A. Die Kleinhirn-Neuroglia beim Menschen (Taf. XI).

In der letzteren Hälfte der Foetalperiode lassen sich die Gliaelemente der Kleinhirnrinde färben. Die Bergmann'schen Faserzellen zeigen dann ein weniger charakteristisches Aussehen als später. Im 7. und 8. Monate des Foetallebens präsentiren sich nämlich die Zellkörper als rundlich-ovale, in verschiedener Höhe belegene Klumpen, von denen nur hier und da ein unterer Fortsatz ausgeht; vom äusseren Umfang entspringen ein oder zwei Fortsätze, welche unter spitzem Winkel divergiren und, sich wiederholt dichotomisch theilend, nach aussen hin ziehen. Es entsteht in dieser Weise ein bald schmälerer und wenig verzweigter, konisch gestalteter, bald reichlicher verästelter, weiter, ausgebreiteter Büschel von Fasern, die perlschnurförmig und fein sind und, allmählig unter einander paralleler geworden, nach der Oberfläche ziehen, wo sie mit konisch verdickten Füsschen endigen. Die Zellen haben zwar schon im 8. Monate (Fig. 1 der Taf. XI) den eigentlichen Typus bekommen; sie sind aber weniger regelmässig gestaltet als später, indem die Fasern noch nicht den schönen, geraden, unter einander parallelen Verlauf bekommen haben; der Zellkörper ist ferner noch mit gar keinen oder nur mit wenigen von den seitlichen und inneren Fortsätzen versehen, welche ihm später ein eigenthümliches Aussehen verleihen. Viele Zellenkörper liegen recht tief in die Kornzellenlage hinabgesenkt und durchlaufen dann die Schicht der Purkinje'schen Zellen (kürzer: die Purkinje'sche Schicht), die noch nicht dicke sog. Molekularschicht und die verhältnissmässig starke Schicht der

äusseren Körner. Wie in der Rinde des Grosshirns, ist auch hier nunmehr, nachdem wir eine viel genauere Kenntniss von dem Bau der Kleinhirnrinde gewonnen haben, die Bezeichnung »Molekularschicht» keineswegs zutreffend. Ich finde es deshalb viel richtiger, diese Schicht nach den in ihr eingeschlossenen charakteristischen Nervenzellen, den *Korbzellen,* zu benennen und sie als *die Schicht der Korbzellen* oder die *Korbzellenschicht* zu bezeichnen.[1] Die nach aussen davon belegene, embryonale Körnerschicht, welche zuerst von VIGNAL beschrieben wurde, nenne ich, um Verwechselungen mit der inneren, aus echten Nervenzellen bestehenden Kornzellenschicht zu vermeiden, die *Vignal'sche Zellenschicht* oder, kürzer, die *Vignal'sche Schicht.* Im Gegensatz zu den Autoren, welche bei der Bezeichnung anatomischer Elemente Personennamen ausschliessen wollen, finde ich solche Namen zuweilen sehr anwendbar und sogar nützlich, vor Allem dann, wenn eine andere zutreffende Bezeichnung sich schwer finden lässt; die Forschernamen enthalten nichts für den Bau und die Function Prejudicirendes und veralten deshalb auch nicht oder werden gar unrichtig (wie z. B. »die Molekularschicht» der Hirnrinde); ausserdem verbinden sie mit sich ein Stück der Geschichte unserer Wissenschaft, in der Regel den Namen des Entdeckers. Solche Bezeichnungen sollten aber auch deshalb verhältnissmässig sparsam angewandt werden.

Was nun die Bergmann'schen Faserzellen betrifft, so findet man sie schon in einem späteren foetalen Stadium verändert. Bei einem 45 Cm. langen menschlichen Foetus (Fig. 2 der Taf. XI) sieht man die noch etwas verschieden tief in der Kornzellenschicht belegenen, nunmehr eckig gewordenen Zellkörper mit mehreren oft, langen, eigenthümlich gestalteten Fortsätzen versehen, die vom unteren (inneren) Umfang des Körpers entspringen und nach unten und nach den Seiten hin verlaufen. Diese »inneren» Fortsätze, welche schon früher von CAJAL, *mir* und VAN GEHUCHTEN abgebildet worden sind, haben einen sehr sonderbaren Charakter, indem sie oft sehr grob und mit grossen, rundlichen, ovalen oder eckigen Verdickungen besetzt sind; besonders oft enden sie mit einem solchen grossen Knöpfchen, das sogar stärker als der Zellkörper selbst sein kann. Die Fig. 2 und Fig. 3 (von einem neugeborenen, 48 Cm. l. Foetus) der Taf. XI enthalten mehrere Varianten dieser inneren Fortsätze, welche sämmtlich in der Kornzellenschicht liegen und zuweilen weit in dieselbe hinab reichen. Ferner entspringen hier und da von den Zellkörpern feinere Fortsätze, die recht zahlreich sein können (Fig. 2, rechts, Fig. 3, links). Was die äusseren Fortsätze und die Bergmann'schen Fasern betrifft, so haben die letzteren ihre perlschnurartige Beschaffenheit etwas eingebüsst und sind auch im Ganzen einander mehr parallel angeordnet.

Aus den folgenden Entwicklungsstadien werde ich dasjenige des 3-monatlichen Kindes und das des Erwachsenen wählen. Beim *3-monatlichen Kinde* (Fig. 4 der Taf. XI) sind nun wieder die inneren Fortsätze reducirt worden; dieses ist zwar nicht vollständig geschehen, doch haben sie einen geringeren Umfang. Die Zellkörper sind aber fortwährend eckig und mit kürzeren Vorsprüngen besetzt; sie liegen in der Umgebung, oft etwas nach unten (innen) von den Purkinje'schen Zellen. Nach oben (aussen) hin schicken sie bald einen, bald zwei oder sogar drei Fortsätze aus, welche in die Korbzellenschicht emporsteigen und sich schnell und wiederholt dichotomisch verästeln; die ersten, gröberen Zweige biegen sich, etwa nach Art der Purkinje'schen Zellen, nach den Seiten hin und von ihnen entspringen dann die mehr oder weniger zahlreichen Bergmann'schen Fasern als feine, gleich dicke, nur hier und da mit kleinen Knötchen oder körnig erscheinenden Aestchen besetzte Fäden, die, unter einander immer mehr parallel geworden, fast gerade oder nur ganz wenig geschlängelt senkrecht zur Oberfläche emporsteigen, wo sie, nachdem sie durch die noch vorhandene, aber dünner gewordene Vignalsche Schicht gelaufen sind, mit je einem recht bedeutenden, rundlich-konischen Füsschen oder Knötchen endigen.

Im *ausgebildeten Stadium* (Fig. 5 der Taf. XI, von einem 33-jährigen Manne) erkennt man noch nach innen von der Korbzellenschicht, in der Umgebung und besonders nach unten von den Purkinje'schen Zellen, die eckigen, knotigen, sogar gelappten Zellkörper der fraglichen Gliäcyten, welche fortwährend einzelne gröbere und feinere Fortsätze nach unten und nach den Seiten hin schicken. Die nach oben in die Korbzellenschicht emporsteigenden Fortsätze behalten ihren früheren Habitus, und ihre nach oben (aussen) gehenden Aeste, die Bergmann'schen Fasern, verlaufen noch mehr gerade und parallel, um an der Oberfläche mit je einem konischen Knötchen zu endigen. Die Vignal'sche Zellenschicht ist längst vollständig verschwunden, aber die Korbzellenschicht ist im erwachsenen Stadium sehr viel dicker geworden, wie die bei derselben Vergrösserung gezeichneten Figuren 2, 4 und 5 zeigen. In Folge dessen sind auch die Bergmann'schen Fasern ebenso viel in die Länge gewachsen.

[1] Diese Schicht könnte auch nach den Longitudinal- oder Tangentialfasern (oder auch nach den Dendriten der Purkinjezellen), benannt werden, welche sie zu so bedeutendem Theile zusammensetzen. Ich finde es aber konsequenter, sie nach ihren Nervenzellen zu bezeichnen.

Sie bilden, ungefähr wie die äusseren Enden der Ependymzellen der Grosshirnrinde (und die vertikalen Aeste der Cajal'schen Zellen), ein reichliches parallelfaseriges Pallisaden- oder Gitterwerk, welches der Kleinhirnrinde wohl als ein wichtiges Stützwerk dient.

Zu welchem Typus der Gliäcyten soll man nun diese Zellen führen? Der Gestalt nach ähneln sie offenbar am meisten den *Schwanzstrahlern,* obwohl sie, im Gegensatz zu denjenigen der Grosshirnrinde, den Körper nach unten (innen), den Fadenschwanz nach oben (aussen) hin wenden.

Ausser diesen Schwanzstrahlern giebt es im Kleinhirn nur *Sternstrahler,* und zwar sowohl in der Kornzellenschicht wie in der Marksubstanz. Die meisten dieser von Golgi, Cajal, Van Gehuchten und von Kölliker abgebildeten Zellen sind Langsternstrahler von der Art, wie sie die Fig. 4 und 5 zeigen; diese Elemente besitzen oft sehr lange, gestreckte und schlichte Fortsätze, welche sogar, wie ich finde, an den Purkinjezellen vorbei, weit in die Korbzellenschicht hinaus verlaufen (Fig. 4 und 5 der Taf. XI). Ausserdem giebt es aber noch andere Sternstrahler, deren Fortsätze ein mehr knotig-gekraustes Aussehen darbieten und kürzer sind (Fig. 5); diese gehören eigentlich der Kornzellenschicht an und sind als Kurzsternstrahler zu bezeichnen. In jüngeren Stadien (Fig. 3 der Taf. XI, vom neugeborenen Kinde) sind die Fortsätze derselben weniger zahlreich, zuweilen perlschnurartig und haben im ganzen einen mehr embryonalen Charakter.

## B. **Die Neuroglia des Kleinhirns der Katze** (Taf. XI, Fig. 6, 7, 8, 10, 11, 12).

Von anderen Säugern habe ich in Betreff dieser Neuroglia vor Allem Katze, Hund, Kaninchen und Maus untersucht. Zur folgenden Darstellung wähle ich besonders das erst genannte Thier aus. Bei noch nicht ausgetragenen *Katzenembryonen* (Fig. 6) zeigen die Bergmann'schen Faserzellen einen moosig verästelten, in der Kornzellenschicht belegenen Zellkörper mit einigen etwas längeren Fortsätzen nach unten und nach den Seiten sowie einen oder zwei nach oben ziehenden, stärkeren, ebenfalls moosigen Fortsätze, welche unter meist sparsamer dichotomischer Verästelung nach oben durch die Purkinjeschicht und die noch verhältnissmässig dünne Korbzellenschicht ziehen, um dann in fortwährend gestreckter Richtung nach oben hin durch die noch dicke Vignal'sche Schicht zu verlaufen und mit konischen Füssen an der Oberfläche zu endigen. In der Korbzellenschicht bieten sie noch kleine seitliche Aestchen und Zacken dar, aber in der Vignal'schen sind solche nur spärlich vorhanden. Bei der *5-tägigen* Katze (Fig. 8) ist die moosige Beschaffenheit der noch verschieden weit hinab in der Kornzellenschicht belegenen, oft mit reichlich verästelten Aesten versehenen Zellkörper sehr ausgeprägt; das Verhalten der nach oben ziehenden Fortsätze ist beinahe unverändert. Von der Neuroglia der *3-tägigen* Katze gebe ich eine Abbildung (Fig. 7), wo zwei solche Zellen vorhanden sind. Dann theile ich von der *14 Tage* und (Fig. 9), ebenso von der *22 Tage* alten Katze (Fig. 11) Abbildungen mit. Aus denselben geht hervor, dass die Zellkörper ihr moosiges Aussehen theilweise einbüssen, obwohl sie fortwährend eckig und gezackt erscheinen; ferner dass die Bergmann'schen Faserfortsätze sich im Verhältniss zu der Verdickung der Korbzellenschicht verlängern und mehr parallel und gestreckt verlaufen, auch in dieser Schicht kleine Seitenzacken bekommen, während dagegen in der immer dünneren Vignal'schen Schicht die Seitenzacken nur sparsam sind. Diese so eben beschriebene Beschaffenheit bieten die fraglichen Zellen auch im erwachsenen Zustande dar, nachdem unter allmähligem Verschwinden der Vignal'schen Schicht die Korbzellenschicht ihre endliche Dicke erhalten hat.

Was die *Sternstrahler* betrifft, so erkennt man an den in der Kornzellenschicht belegenen Zellen in den Entwicklungsstadien vor und bald nach der Geburt einen Anklang an die oben beschriebenen Schwanzstrahler, indem von dem moosigen, senkrecht verlängerten Zellkörper sehr oft ein stärkerer Fortsatz nach oben hin zieht, der jedoch nach kürzerem oder längerem Verlaufe endigt, ohne die Korbzellenschicht erreicht zu haben (Fig. 8). In etwas späteren Stadien (Fig. 9, 11) bekommen die Zellen mehr das gewöhnliche, gekrauste Aussehen der Kurzsternstrahler. Die in der Marksubstanz belegenen Gliäcyten (Fig. 12) sind dagegen als Langsternstrahler zu betrachten.

Es ist zwar nicht meine Absicht, diesmal auf andere Gebilde der Kleinhirnrinde als die echten Gliäcyten einzugehen. Ich kann jedoch nicht umhin, auch die zelligen Elemente der zuerst von Vignal beschriebenen und bald nachher von Schwalbe besprochenen sog. äusseren Körner zu berühren, welche beim Foetus und noch eine

Zeit nach der Geburt constant vorhanden sind. In späterer Zeit hat sie vor Allen RAMÓN Y CAJAL[1] eingehender dargestellt und unter ihnen eine äussere (obere), aus mehr vertikal gestellten länglichen Zellen bestehende, und eine innere (untere), aus horizontal (tangential) liegenden bipolaren Zellen gebildete Schicht beschrieben. Ausserdem hat er in der »molekulären» Schicht vertikale bipolare Zellen beschrieben und abgebildet, die nach der erwähnten inneren Schicht der horizontalen bipolaren Körner hin einen Fortsatz schicken, welcher nervös zu sein scheint und in der fraglichen Schicht sich an eine longitudinale Faser ansetzt; Uebergangsformen zwischen diesen den inneren Körnern ähnelnden Zellen und den horizontalen bipolaren Zellen wurden auch gesehen, aber im Ganzen zu selten, um diese Zellen genetisch mit einander verbinden zu können. VON KÖLLIKER[2] hob in Betreff dieser aufsteigenden Elemente hervor, dass man sie beim Erwachsenen nicht gefunden hat, »und da auch die eigentlichen Körner bei jungen Geschöpfen schon vollkommen ausgebildet sind, ist auch nicht daran zu denken, dass sie Entwicklungsstadien derselben seien». In der französischen Auflage seiner neuen übersichtlichen Arbeit hat nun RAMÓN Y CAJAL[3] sich bestimmt dafür ausgesprochen, dass die Kornzellen der Kleinhirnrinde (die Zellen der inneren Körnerschicht) in der That durch diese aus den in der unteren Partie der Vignal'schen Schicht belegenen, bipolar ausgezogenen Zellen rekrutirt werden, indem sich diese nach unten (innen) hin verlängern und hinabsteigen. Nun hat neulich auch LUGARO[4] die Frage wieder aufgenommen und weitere Conklusionen gezogen. »Die äussere Körnerschicht», sagt er, »ist aus zwei Lagen zusammengesetzt, welche gut morphologisch unterschiedene, jedoch durch Uebergangsformen verbundene Elemente enthalten. Die äussere Lage besteht aus kugeligen oder birnförmigen Elementen, mit einem gegen die Oberfläche gerichteten Fortsatz. Sie zeigen manchmal andere kleine, ohne Regel gerichtete Fortsätze. Wegen dieser Kennzeichen sind sie als epithelioide embryonale Elemente anzusehen. Die Elemente der tiefen Lage sind bipolare, mit einem ovalen Körper, der sich in zwei Polen in zwei mehr oder weniger lange, parallel mit der Oberfläche und mit dem Gang der Windungen gerichtete Ausläufer fortsetzt. Diese Fortsätze sind um so dünner und ausgedehnter, je tiefer das Element gelegen ist, ihre Enden zeigen eine Anschwellung, den sogenannten Vermehrungskegel.» »An der Grenze zwischen der oberflächlichen und der tiefen Lage finden sich einige Bildungen, welche die stufenweise Umbildung der epithelioiden in die bipolaren Elemente durch Entstehung zweier Fortsätze zeigen. Diese Umbildung geht fortschreitend von innen nach aussen vor sich.» Die an der inneren Grenze der tiefen Lage befintlichen bipolaren Elemente haben den Körper gegen die Tiefe gerichtet und senden in die Molekularschicht einen Fortsatz. Uebergangsformen zu den inneren Körnern sind vorhanden. Aus dem Ausgeführten, sagt LUGARO, »kann man schliessen, dass die Körner durch fortschreitende Verwandlung der epithelioiden in horizontale Elemente, dieser in vertikalen, und endlich dieser letzteren in Körner entstehen, welche Verwandlung von der Wanderung des Zellkörpers von der Oberfläche nach der Tiefe begleitet wird».

Ich habe diese Angaben so ausführlich wiedergegeben, weil sie so bestimmt behaupten, dass das, was CAJAL zuerst angedeutet hat, sicher vor sich geht, und weil die Bedeutung der mystischen Vignal'schen Zellen dadurch erledigt sein soll. Die Erklärung enthält ja in der That viel Bestechendes. Ich bin aber dennoch zweifelhaft und schliesse mich von KÖLLIKER's Aeusserung an. Ich habe nämlich diesen Zellen seit mehreren Jahren bei jeder Untersuchung der Kleinhirnrinde meine Aufmerksamkeit gewidmet. Oft bekam ich mit der Golgi'schen Methode Färbung derselben, sowohl beim Menschen wie beim Kaninchen und der Katze, und ich bildete dann ihre verschiedenen Formen ab. Von diesen Abbildungen kann ich hier leider, wegen Mangel an Platz auf den Tafeln, nur wenige mittheilen. Beim Foetus fand ich nur (Fig. 6 der Taf. XI) in der noch relativ sehr dicken Zellenlage die verschiedensten Zellenformen, nämlich rundliche, ovale, cylindrische, unipolare, bipolare und multipolare in bunter Unordnung zwischen einander gestreut. Es lohnt sich hier kaum ohne hinreichende Figuren, eine genauere Beschreibung dieser Zellen zu geben. Nur so viel soll erwähnt werden, dass unter ihnen oft Zellen mit einem oder zwei, verschieden langen, Fortsätzen vorkommen, welche gerade oder schief gegen die Oberfläche gerichtet sind; zwischen ihnen liegen aber Elemente von sehr wechselnder Form und Richtung; die allermeisten haben einen nur schwachen Protoplasmamantel um den ovalen oder rundlichen Kern, doch ist das Protoplasma gewöhnlich an einer Seite der Zelle etwas reichlicher vertreten; in der That ähneln sie lymphoiden Zellen oder gewissen

[1] S. RAMÓN Y CAJAL, A propos de certains éléments bipolaires du cervelet avec quelques détails nouveaux sur l'évolution des fibres cerebelleuses. Intern. Monatschr. f. Anat. u. Phys., Bd 7, 1890.

[2] A. VON KÖLLIKER, Handbuch der Gewebelehre des Menschen, 6. Aufl., II Bd, 1, 1893.

[3] S. RAMÓN Y CAJAL, Les nouvelles idées sur la structure du système nerveux chez l'homme et chez les vertébrés. Paris 1894.

[4] ERNST LUGARO, Ueber die Histogenese der Körner der Kleinhirnrinde. Anatom. Anzeiger, Bd 9, N:o 23, Aug. 1894.

Epithelformationen. Beim Foetus sah ich aber durch die ganze Zellenlage hindurch nur die beschriebene Art von Zellen und keine Eintheilung in zwei Schichten.

In den ersten Wochen nach der Geburt gelang es mir nun hier und da am Kleinhirn der jungen Katzen die zuerst von Cajal und dann noch von Lugaro nachgewiesene untere (innere) Schicht von Zellen zu finden, und gerade in der von ihnen beschriebenen Anordnung und Gestalt. In der Fig. 9 (bei *t*) der Taf. XI sind in einem Vertikalschnitt und in Fig. 10 ders. Tafel in einem Tangentialschnitt der Kleinhirnrinde einer 14 Tage alten Katze zahlreiche solche bipolare Zellen in ihrer natürlichen Anordnung wiedergegeben. Es lässt sich in der That nicht bestreiten, dass diese Elemente embryonalen, anwachsenden Nervenzellen — mit den feinen Axencylinder- und den dickeren Protoplasmafortsätzen versehen — recht sehr ähneln. Wirkliche Uebergangsformen zwischen den Zellen der äusseren und der inneren Schicht, wie sie Cajal und Lugaro beschrieben haben, sah ich aber bisjetzt nicht; ja ich konnte diese innere Schicht bipolarer Zellen sogar nur hier und da und nicht überall in der Rinde nachweisen. Und endlich muss ich gestehen, dass es mir bisjetzt nicht gelungen ist, die von Cajal und dann von Lugaro beschriebenen, durch die Korbzellenschicht (Molekularschicht) senkrecht hinabsteigenden bipolaren Zellen zur Ansicht zu bekommen, obwohl ich Massen von Präparaten durchmustert habe. Entweder scheinen diese Zellen äusserst schwer färbbar zu sein, oder auch kommen sie selten oder nur an gewissen Orten vor. Ich will natürlicherweise nicht das Vorkommen dieser merklichen Zellenart bestreiten, da sie von einem so scharf beobachtenden Forscher wie Cajal beschrieben und von Lugaro, der in Mondino's Laboratorium arbeitete, bestätigt worden sind. Cajal hat selbst zuerst die Seltenheit der Uebergangsformen zu den inneren Kornzellen betont. In der That wäre es sehr eigenthümlich, wenn so spät im Leben, noch Monate nach der Geburt, Nervenzellen in so embryonaler Entwicklung begriffen sein sollten, um so viel mehr, als, wie von Kölliker betont, die inneren Kornzellen sonst schon früh angelegt und in ihrer Organisation weit vorgeschritten sind. In dieser Hinsicht lässt sich aber von der anderen Seite hervorheben, dass die Korbzellenschicht (Molekularschicht), welche grossentheils aus den Longitudinalfasern der inneren Kornzellen besteht, in foetalem Zustande und noch lange nach der Geburt dünn ist und allmählig wächst, was gerade für ein Hinzufügen neuer Zellfortsätze sprechen kann. Ich versuchte ferner herauszufinden, ob nicht vielleicht die Zellen der Korbzellenschicht durch ein Hineinrücken der Zellen der Vignal'schen Zellenschicht vermehrt werden, konnte hierfür aber keine Stütze finden.

Nach dem Standpunkt, den diese prinzipiell interessante Frage gegenwärtig einnimmt, muss ich sie also meinerseits bis auf Weiteres offen lassen, doch bin ich betreffs derselben eher skeptisch gestimmt.

Bevor ich den Gegenstand verlasse, habe ich aber eine für die Beurtheilung der Frage nicht unwichtige Beobachtung zu erwähnen, die ich im vorigen Winter gemacht habe. Da mir die embryonale Beschaffenheit der Vignal'schen Zellenlage stets auffiel, so suchte ich mittelst der gewöhnlichen Methoden nach Zellentheilungen in derselben. In der That gelang es mir sogleich, *Zellentheilungen*, Mitosen, nicht nur in der foetalen Rinde, sondern auch einige Zeit nach der Geburt aufzufinden. Sie kommen noch bei der *zweiwöchentlichen* Katze zahlreich vor, und zwar nicht nur nach der Oberfläche hin, sondern besonders in der Mitte der Schicht. In späteren Stadien scheinen mir Zeichen von Auflösung des Chromatins in den Kernen mancher Zellen vorzukommen. Jedenfalls dürften hierüber weitere Untersuchungen vorzunehmen sein.

Noch ein Befund soll hier kurz erwähnt werden. In der Vignal'schen Schicht habe ich sowohl bei jungen Katzen wie Kaninchen eine Art tangential verlaufender, variköser, aber nicht besonders feiner Fasern, die als Nervenfasern imponiren, mittelst der Golgi'schen Methode gefärbt bekommen. Es gelang mir, dieselben zuweilen auf weite Strecken an Querschnitten der Gyruli zu verfolgen. Sie theilen sich nicht, ziehen meistens einzeln in der Mitte der Schicht hervor, konnten aber nicht bis zu ihrem Ursprung und Ende verfolgt werden. Einmal sah ich sie, wie die Fig. 11 der Taf. XI wiedergiebt, in die Korbzellenschicht hinabtauchen. Was diese Fasern eigentlich sind, blieb mir trotz allen Suchens dunkel.

## III. Die Neuroglia der Neuro-Hypophyse der Säugethiere.

(Taf. XII.)

Es ist keineswegs meine Absicht, hier eine Darstellung vom Gesammtbau oder gar von der Entwicklung des merkwürdigen rudimentären Organs zu geben, das wir gewöhnt sind, die Hypophyse zu nennen; erstens gehört ja der sog. epitheliale (vordere) Theil desselben nicht dem Gehirn an, und zweitens würde eine eingehendere Schilderung des hinteren, mit dem Centralorgan direkt zusammenhängenden Hypophysentheiles (des Hinterlappens der Hypophyse, des *Processus seu Lobus infundibuli* oder der *Neuro-Hypophyse,* wie sie auch bezeichnet werden kann) eine ganz besondere und ausführliche Untersuchung erfordern. Dieselbe würde eine Reihe von Thierarten sowohl in Betreff des fertigen Baues als der Histogenese behandeln, was ausser dem Rahmen dieser Abhandlung liegt. Das, was ich hier beabsichtige, ist nur die Grundelemente der Neurohypophyse der Säugethiere zu besprechen, um vor Allem die Verbreitung und die Formen des Gliagewebes mittelst der Golgi'schen Methode zu eruiren.

Wenn man die grösseren Lehrbücher und die übrige die Hypophyse behandelnde Litteratur durchmustert, bemerkt man bald, dass der Bau ihres neuralen Theils im Ganzen sehr wenig bekannt ist. Die Beschreibungen sind im Allgemeinen kurz, schwebend und in mancher Beziehung unrichtig. Dies ist auch ganz natürlich, weil die bisher angewandten Methoden kaum genauere Ergebnisse liefern konnten. Erst die neueren Methoden sind geeignet, eine gute Einsicht in den Bau dieses Organes zu geben.

In seinem Lehrbuch der Neurologie sagt also Schwalbe,[1] nachdem er die bei den höheren Wirbelthieren, besonders bei den Säugethieren und dem Menschen stattgefundene, bedeutende Rückbildung des ursprünglich hohlen Organes erwähnt hat: »Seine Höhle schwindet für gewöhnlich, erhält sich nur noch in dem dünnen verbindenden Stiel, dem Infundibulum; aus dem grossen Lobus infundibuli der niederen Wirbelthiere ist nun ein relativ kleiner solider Processus infundibuli geworden, der sogenannte hintere Lappen der Hypophysis. Zu gleicher Zeit sind seine nervösen Elemente durch reichliches Hineinwuchern von Blutgefässen und Bindegewebe zur Atrophie gebracht, so dass der Processus infundibuli der Erwachsenen aus fibrillärem Bindegewebe besteht, welches an runden und spindelförmigen Zellen reich ist, zum Theil auch verästelte Zellen enthält. Die Faserbündel kreuzen sich dabei in den verschiedensten Richtungen, wie in dem Gewebe eines Spindelsarkoms (W. Müller). Unter den runden und spindelförmigen Zellen finden sich grössere, die in ihrem Protoplasma gelbe Pigmentkörner führen.»

Nach Toldt[2] besteht der *Trichterlappen* (Processus infundibuli) »aus fibrillärem, stark vascularisirtem Bindegewebe mit zahlreichen spindelförmigen oder selbst vielstrahligen Zellen, von denen einzelne gelb pigmentirt erscheinen und dadurch Ganglienzellen gleichen.»

Schäfer[3] äussert sich in ähnlicher Weise: »Die Höhle ist obliterirt, und jede nervöse Struktur ist durch das Einwachsen von Blutgefässen und Bindegewebe in das nunmehr solide Organ verdunkelt worden. Das Bindegewebe bildet geflechtartige Bündel, zwischen welchen zahlreiche spindelförmige und verzweigte Zellen sowohl wie einige wenige grössere Zellenkörper vorkommen, die in ihrem Protoplasma Pigmentkörner enthalten.»

In den übrigen neueren, grösseren Lehrbüchern habe ich ungefähr dieselben Angaben gefunden. Ich werde deshalb nur noch ein solches Werk anführen. »Die Hypophysis cerebri», sagt Rauber,[4] »besteht in ihrem kleinen hinteren, cerebralen Lappen aus spärlichen Nervenfasern, vielen Zellen, welche mit bipolaren oder multipolaren Nervenzellen eine gewisse Aehnlichkeit haben, aus Bindegewebe und vielen Blutgefässen.»

Unter den neueren Forschern, welche sich mit dem Bau der Hypophysis der höheren Thiere beschäftigt haben, ist vor Allem Lothringer[5] hervorzuheben. Er untersuchte das Organ sowohl beim Menschen wie bei verschiedenen

[1] G. Schwalbe, Lehrbuch der Neurologie, 2. Bd, 2. Abth. von Hoffmann's Lehrb. d. Anat. d. Menschen, 1881.
[2] C. Toldt, Lehrbuch der Gewebelehre, 3. Aufl., 1888.
[3] E. A. Schäfer, Quain's Elements of Anatomy, Vol. 3, p. 1; 10. Edition, 1893.
[4] A. Rauber, Lehrbuch der Anatomie des Menschen, 4. Aufl., II, 2, I, 1894.
[5] S. Lothringer, Untersuchungen an der Hypophyse einiger Säugethiere und des Menschen. Archiv f. Mikrosk. Anatomie, Bd 28, 1886.

Säugethieren (Hund, Katze, Pferd, Schwein, Kaninchen). Er beschreibt am eingehendsten die Verhältnisse beim Hunde, bespricht aber weniger den Hirntheil (Processus infundibuli oder die Neuro-Hypophyse) als den Epitheltheil. Ersterer ist in eine schalenförmige Vertiefung des letzteren eingesenkt, und jeder besitzt eine Höhle; wenn man den Hirntheil von dem Epitheltheil löst, so folgt mit jenem ein ihm innig anhängender Epithelsaum des letzteren, indem die Trennung in der spaltförmigen Höhle vor sich geht. Der Epithelsaum besteht aus einer mehrere Zellreihen hohen Epithelschicht, welche nur in der Nähe des Umschlagrandes eine grössere Mächtigkeit erreicht und zahlreiche kleine Cystenräume enthält. »Ueber das Gewebe des Hirntheiles haben wir», sagt Lothringer, »speciellere Untersuchungen nicht angestellt.» Die angewendete Behandlungsmethode war für ein Studium der complicirten Verhältnisse nicht günstig. »Der Hauptsache nach besteht dies Gewebe aus sich spitzwinklig durchflechtenden Faserzügen. Diese Stellen, von schmalen bindegewebigen Septen, die von der Oberfläche aus eindringen, durchzogen, bilden eine Grundlage, in deren Zwischenräumen lockere Gewebsmassen enthalten sind. Die zuerst genannten Faserzüge bieten da, wo sie in grösserer Masse auftreten, wie Schwalbe und W. Müller anführen, ein Bild, das sich füglich mit dem Spindelzellensarkom vergleichen lässt. Lang gestreckte Kerne erinnern an glatte Muskelfasern, doch sind auch zahlreiche Rundzellen und polygonale oder sternförmige Zellen in die Faserzüge eingelagert. Die lockeren, in den Zwischenräumen enthaltenen Gewebetheile bestehen aus sternförmig verästelten, durch Ausläufer untereinander verbundenen Zellen, deren Anordnung auf's Evidenteste dem Gliagewebe an Nervenzellen armer Hirntheile entspricht. Die Abgrenzung beider Formelemente — des aus Faserzügen und Glianestern gemischten Gewebes — ist keine scharfe.» An den der Weigert'schen Hämatoxylinbehandlung unterzogenen Präparaten erscheinen die Glianester als granulirte Ausfüllungsmasse der Zwischenräume zwischen den Faserbalken. Nur an sehr dünnen Präparaten lassen sich die Kerne der Gliazellen leicht erkennen. Der Uebergang des Tuber cinereum in den Trichterlappen findet sich in der Höhe des Umschlagtheiles; die graue Substanz des Tuber schiebt sich hier als sich abwärts verjüngender Saum zellenarmen Hirnrindengewebes zwischen den Epitheltheil der Hypophyse und das eigenartige Gewebe des Trichterlappens ein. Feinste, senkrecht zur Oberfläche gestellte Fasern bilden die Grundlage dieses Gewebes. In das mit dem letzteren zusammenhängende Gliagerüste sind lang gestreckte pyramidenförmige Ganglienzellen spärlich eingelagert. Die Blutgefässe des Trichterlappens bildet ein Netz mit grossen Zwischenräumen; die Randschlingen dieser Gefässe reichen bis unmittelbar unter den Epithelsaum, indem sie Bogen bilden, welche ohne jedes Zwischengewebe an die Zellen des Saumes grenzen.

Aus dem soeben Angeführten geht hervor, dass Lothringer, wie ich unten nachzuweisen suchen werde, doch in manchem eine richtige Auffassung vom Bau des Trichterlappens gewonnen hatte, obwohl er die eigentliche Natur des Gliagewebes und der Septa zu jener Zeit nicht einsehen konnte. Dieses wird am besten durch Behandlung des Organs nach der Golgi'schen Methode möglich. Schon vor mehreren Jahren habe ich dies gethan und eine schöne Färbung der Gewebstheile bekommen. Da, meines Wissens, noch kein anderer Forscher die Frage behandelt hat, werde ich hier meine Befunde kurz beschreiben.

Ich untersuchte die Hypophyse des *Menschen*, der *Katze*, des *Hundes* und des *Kaninchens*. Von diesen habe ich besonders *Katze* und *Hund* gut dazu geeignet gefunden, eine nähere Einsicht in den Bau des Organs zu geben. An Sagittal- und Frontalschnitten der Hypophyse älterer Katzenfoetus erkennt man, nach der Behandlung mit der Golgi'schen Methode, dass die Beschreibung Lothringer's in Betreff der gröberen Anordnungen im Ganzen zutreffend ist. Bei den genannten Thieren senkt sich der als rundlich erweiterter Fortsatz des tubulären Tuber cinereum leicht erkennbare »Lobus infundibuli» in eine schalenförmige Vertiefung der »epithelialen» Hypophyse ein (Fig. 1 der Taf. XII). Im Inneren des Tubustrichters sieht man einen schmalen Kanal, den Fortsatz des dritten Ventrikels, und im Inneren des Trichterlappens ist eine Höhle, die Trichterlappen-Höhle, sichtbar. Am unteren Umfang des Lappens erkennt man den von Lothringer genauer beschriebenen Epithelsaum, welcher mit scharfer Grenze an dem Gewebe des Lappens ansitzt und innig mit ihm zusammenhängt, nach aussen hin aber an die Spalte der epithelialen Hypophyse stösst. Oben am Trichterhals sieht man den Umschlagsrand dieses Epithelsaums in das Gewebe der epithelialen Hypophyse.

Schon bei schwacher Vergrösserung (Fig. 1) erkennt man nun in dem genannten Epithelsaume eine Menge durch die Golgi'sche Methode gefärbter, länglicher Zellen, welche zum grössten Theil ganz schmal, sogar fadenartig sind und durch den ganzen Saum reichen, aber nicht, wie Lothringer sagt, »aus einer mehrerer Zellreihen hohen Epithelschicht» bestehen. In der Fig. 4, *e* (von einem 2 Monate alten Hunde) und in Fig. 3, *ä* (von einem 8

Tage alten Kaninchen) der Taf. XII habe ich bei stärkerer Vergrösserung einige dieser in der That Epithelzellen ähnelnde Zellen abgebildet. Wie man aus diesen Figuren sieht, reichen jedoch nicht alle Zellen durch die ganze Schicht; es giebt auch eigenthümlich gestaltete, verzweigte Formen, welche zwischen den fadenförmigen eingebettet liegen und nur die eine Fläche des Saumes oder auch gar keine erreichen; die Zellenkerne liegen besonders oft in der Nähe des äusseren Endes; das innere Ende der fadenförmigen Zellen verbreitert sich oft zu einem dreieckigen Fusse, der gegen das eigentliche Gewebe des Trichterlappens stösst, und zwar in der angegebenen scharfen Grenzlinie oder Fläche, unter welcher die von Lothringer beschriebenen Blutgefässmaschen (Fig. 3 und 4, *bg*) dicht anliegen. An der äusseren Oberfläche des Epithelsaumes bekommt man oft ein schönes Mosaik von kleinen polygonalen Feldern, die äusseren Endflächen der Zellen (Fig. 2 *a*).

In dem Gewebe des Trichterlappens bemerkt man an den Golgischen Präparaten schon bei schwacher Vergrösserung eine grosse Menge von gefärbten Fasern und verästelten Zellen. An Frontalschnitten (Fig. 2) erkennt man eine grosse Masse solcher Fasern, welche von oben, d. h. vom Trichterhals, radiirend in den Lappen ausstrahlen. Sie imponiren als Nervenfasern, und es ist in der That schwer, eine derartige Ansicht auszuschliessen, obwohl sie hier und da etwas grob erscheinen und Gliafasern ähneln. Es giebt ja auch sehr lange Fortsätze von Gliazellen. Viele dieser Fasern sind auch letzterer Art, indem sie sich direkt als solche Fortsätze nachweisen lassen. In den inneren Partien des Lappens finden sich auch, wie Lothringer betont, grössere Blutgefässe, welche nach aussen hin ausstrahlen und das Gewebe, vor Allem dessen äusseren Theile bis zum Epithelsaum, mit einem reichlichen Maschenwerke schmälerer Gefässschlingen durchspinnen. Zwischen diesen Gefässschlingen streichen die zahlreichen Fasern hin, theilen sich hier und da dichotomisch, besonders in den peripheren Theilen, und endigen frei und verästelt. In diesem zwischen den Gefässen befindlichen dichten Gewebe erkennt man nun auch eine Menge durch das Chromsilber gefärbter verzweigter Zellen, die sich überall durch ihre charakteristischen Formen als Gliazellen erkennen lassen. In den Fig. 3, 4, 5, 6 und 7 habe ich solche Zellen theils aus der Peripherie des Lappens, theils aus dem Inneren desselben abgebildet. Fig. 4 stammt vom jungen Kaninchen, die übrigen vom 2—12-monatlichen Hunde her. Diese Gliazellen gehören zwar nicht den oben geschilderten Haupttypen an; ein Theil von ihnen ist zwar als Sternstrahler zu bezeichnen; andere tragen nur einzelne, zuweilen einseitig entspringende Fortsätze, die mit Zacken und moosförmig verästelten Vorsprüngen versehen sind. Die Betrachtung der Abbildungen giebt von ihrer Beschaffenheit gewiss eine bessere Auffassung als eine eingehendere Beschreibung, weshalb ich auf dieselben hinweisen kann. Die Gliaelemente bilden ein dichtes Flechtwerk; an stärker gefärbten Partien lassen sich die einzelnen Zellen deshalb nicht genauer studiren. Oft bekommt man aber auch Stellen, wo nur einzelne Zellen gefärbt sind, wie in den hier abgebildeten Partien, und dann kann man ihre Anordnung und Verzweigung leicht verfolgen. Eine eigentliche Anordnung giebt es aber nicht, sondern sie bilden ein Geflecht von Elementen, die in verschiedenster Weise unter einander gewebt sind; nur in der nächsten Umgebung der Blutgefässe bemerkt man hier und da eine tubuläre oder im Ganzen eine flächenartige Ausbreitung der Zellenfortsätze (Fig. 4), obwohl nicht so typisch wie die der von Lloyd Andriezen geschilderten, die Blutgefässe der Grosshirnrinde umstrickenden Gliazellen. Nie sah ich ein Anastomosiren der Gliazellen unter einander.

Echte Langsternstrahler habe ich aber auch im Trichterlappen oft wahrgenommen, und zwar im Halstheile nahe vor dem Uebergang zum Trichter. Fig. 8 stellt drei solche Zellen beim *17-jährigen* Manne dar. Im eigentlichen Trichterhalse, welcher diesen Uebergang zum Trichter vermittelt, findet man nun, dass die Gliazellen sich mehr oder weniger senkrecht zur Oberfläche anordnen. Lothringer scheint hier einen solchen fadenartigen Bau gesehen zu haben. Offenbar ist seine Beobachtung eben durch die Anordnung der Gliazellen zu erklären. Diese Gliazellen sind nun in der That als Ependymzellen zu bezeichnen, denn sie reichen von der inneren Fläche, der Kanalfläche, in deren Nähe die Zellkörper liegen, bis an die Oberfläche hinan; während dieses Verlaufes verzweigen sie sich zuweilen wiederholt dichotomisch; manche sind aber auch unverzweigt oder nur etwas gezackt. Sie entsprechen offenbar den neulich von Berkley (Anat. Anz., Aug. 1894) beschriebenen und abgebildeten Ependymzellen der Trichterregion.

In dem *Trichterlappen* der Hypophyse, der *Neurohypophyse* (Processus seu Lobus infundibuli), ist also *eine grosse Masse von Gliaelementen* vorhanden. LOTHRINGER hat dieses Gewebe dort auch schon (1886) erkannt und erwähnt. Wegen Mangel an guten Methoden konnte er aber die Formen dieser Elemente nicht nachweisen; zu jener Zeit war auch die Auffassung von der Beschaffenheit des Gliagewebes noch sehr schwebend; man kannte damals noch sehr wenig von seiner wirklichen Natur, und die betreffenden Ansichten der hervorragendsten Histologen gingen weit auseinander. LOTHRINGER spricht deshalb nur von Glianestern »als granulirte Ausfüllungsmasse der Zwischenräume zwischen den Faserbalken»; Kerne konnte er jedoch in ihr wahrnehmen. Durch die Golgi'sche Methode ist es aber gelungen, die typischen, reichlich verzweigten Gliazellen und Fasern in bunter Anordnung und von wechselnder Gestalt in dem Gewebe des Trichterlappens massenhaft zu demonstriren. Ausserdem ist eine Menge von langen Fasern vorhanden, von denen gewiss ein Theil als Gliafasern aufzufassen ist; ein anderer Theil besteht aber vielleicht aus Nervenfasern; jedenfalls will ich eine solche Annahme bis auf Weiteres nicht bestreiten. Weiter sind zahlreiche Blutgefässe mit ihren Adventialscheiden da. Anderes Bindegewebe konnte ich aber nicht nachweisen. Alles das, was von den Verfassern (auch LOTHRINGER) als solches Gewebe beschrieben wurde, gehört meiner Ansicht nach dem Gliagewebe (und den fraglichen Nervenfasern) an. Echte Nervenzellen, Ganglienzellen, konnte ich im Trichterlappen durch die Golgi'sche Methode nie nachweisen. Zwar sind negative Ergebnisse an sich nicht entscheidend; da ich aber eine Menge Färbeversuche gemacht habe, so bin ich zu der Ueberzeugung gekommen, dass solche Zellen dort in der Regel nicht vorhanden sind.

---

## IV. Die Neuroglia des Nervus opticus und der Retina des Menschen und der Säugethiere.

(Taf. XIII.)

Die Neuroglia des Opticus und der Retina, welche schon durch mehrere neue treffliche Arbeiten gut erkannt worden ist, habe ich hier zur Behandlung nur deshalb aufgenommen, weil diese typisch gestalteten Neurogliaformen in einer übersichtlichen Darstellung der Neuroglia nicht fehlen dürfen, sowie auch, weil ich diese Formen schon seit mehreren Jahren (1887) mittelst der Golgi'schen Methode dargestellt und von ihnen eine Reihe von Abbildungen gemacht habe. Ich kann aber unter Hinweis auf die Taf. XIII, wo einige dieser Abbildungen wiedergegeben sind, die Beschreibung der fraglichen Neurogliaformen kurz fassen.

Was die *Gliazellen des Opticus* betrifft, so waren ja die interstitiellen Zellen dieses Nerven schon lange bekannt. Vor Allem hatten *ich* und KEY in unserer grossen Monographie über das Nervensystem und das Bindegewebe [1] diese zwischen den Nervenbündeln und in dem Inneren derselben massenhaft vorhandenen, mit vielen feinen und langen Fortsätzen versehenen Zellen genau beschrieben und in schönen Abbildungen (Taf. XXXIV des genannten Werkes) dargestellt. Als ich nachher (im Jahre 1887) diese Gliazellen mittelst der Golgi'schen Methode in gefärbtem Zustande erhielt, fand ich es deshalb kaum nöthig, eine Mittheilung davon zu geben. Im folgenden Jahre erschien nun eine kurze Beschreibung der Gliazellen der peripherischen Nervenwurzeln von PETRONE [2], in welcher er sagt, dass er nicht nur im Opticus, sondern auch in mehreren anderen, sogar in echt peripherischen Nerven, in den intracraniellen Wurzeln, wirkliche Gliazellen gefunden habe, nämlich, ausser in dem Opticus und Olfactorius auch im Trigeminus, Acusticus, Facialis, Glossopharyngeus und dazu noch in einigen Wurzeln von Spinalnerven. Ich muss gestehen, dass ich hierüber etwas erstaunte, denn unter diesen Nerven sind doch nur zwei von centraler Natur; alle die übrigen sind echt peripherisch und enthalten mit Schwannschen Scheiden versehene Nervenfasern. Die Mittheilung PETRONE's erschien mir deshalb etwas abenteurlich, und gemachte Controllversuche gaben in Betreff der peripherischen Nervenwurzeln auch nur negative Resultate; ich bekam nur gefärbte echte Bindegewebszellen. Nun ist jedoch zu bemerken, dass die Bezeichnung der Figuren in PETRONE's Mittheilung so beschaffen ist, dass man nicht sicher zu verstehen vermag, welche von seinen Figuren sich auf die verschiedenen peripherischen Nerven beziehen.

[1] AXEL KEY und GUSTAF RETZIUS, Studien in der Anatomie des Nervensystems und des Bindegewebes. Bd I, 1875.
[2] LOUIS M. PETRONE, Sur la structure des nerfs cérébro-rachidiens. Intern. Monatschr. f. Anat. u. Physiol. Bd 5, 1888.

Im Jahre 1892 beschrieb dann Kallius[1] beim Menschen und bei verschiedenen Säugethieren die mittelst der Golgi'schen Methode gefärbten Neurogliazellen im Opticus, ebenso im Trigeminus, Acusticus und Vagus, und zwar nur in den Abschnitten kurz nach ihrem Austritt aus dem Gehirn.

Bald danach theilte Michel[2] mit, dass auch er mit dieser Methode die langstrahligen Neurogliazellen des Sehnerven in zahlreicher Menge dargestellt hatte.

Ungefähr zu gleicher Zeit gab Dogiel[3] eine eingehendere Beschreibung der Neuroglia der Retina des Menschen, v. A. d. Müller'schen Fasern, aber auch der Sternstrahler; diese letzteren finden sich, sagt er, ausschliesslich in der Nervenfaserschicht, und zwar in der Nähe der Papilla n. optici, an der Stelle, wo die Dicke der Schicht bedeutend ist, weiterhin in der ganzen Papilla und an der ganzen Ausdehnung des N. opticus. Diese Zellen mit ziemlich lang und oft bündelweise vom mehr oder weniger abgeplatteten Zellkörper ausstrahlenden Zellen sind in der Retina und der Papilla n. optici von geringerer Grösse als jene Zellen, welche zwischen den Faserbündeln des Sehnerven eingebettet sind; ihrerseits sind wiederum die an der Peripherie des Nerven belegenen Zellen etwas grösser als alle übrigen Zellen.

In einer späteren Arbeit über die Netzhaut bespricht Kallius[4] noch einmal die Neurogliazellen des Sehnerven und der inneren Retinaschichten (der Nervenfaser- und der inneren Ganglienzellenschicht).

Die Gliazellen der Retina waren, ebenso wie die Gliazellen des Opticus, schon lange vor der Einführung der Golgi'schen Methode bekannt, wie die Arbeiten Schwalbe's und mehrerer anderer Forscher darthun. Mittelst der genannten Methode hatte sie ja auch Ramón y Cajal in schöner Weise dargestellt und beschrieben. Ich verweise v. A. auf seine letzte grosse Arbeit über die Retina der Wirbelthiere.[5]

Das, was mich bei der Untersuchung der Neuroglia des Nervus opticus interessirte, war die Verfolgung des Ueberganges der ependymartigen Zellen in Sternstrahler. Ich hoffte von Anfang an, wenigstens in frühen Stadien der Entwicklung einen solchen für die Auffassung von der Herkunft der eigentlichen Gliazellen wichtigen Uebergang gerade hier in schöner Weise darthun zu können. Dies gelang mir aber bisher nicht in gewünschtem Grade, obwohl die Untersuchungen noch fortgesetzt werden sollen. Das, was ich im embryonalen Nervus opticus fand, bestand zwar aus früheren Stadien von Gliazellen, doch waren es eigentlich nur Sternstrahler. In der Fig. 1 der Taf. XIII ist eine peripherische Partie des Sehnerven von einem 28 Cm. langen menschlichen Foetus und in der Fig. 4 von einem 14 Cm. l. Katzenfoetus abgebildet. Die Zellen haben noch immer nur wenige Fortsätze, die einen gewundenen Verlauf und oft eine knotige Beschaffenheit darbieten. Aber schon beim 45 Cm. l. menschlichen Foetus (Fig. 2) ist der embryonale Typus mehr oder weniger in den späteren verwandelt; die Fortsätze sind zahlreicher, steifer, gestreckter geworden. Bei dem 5 Tage alten Kätzchen (Fig. 5 und 6) sind noch embryonale zusammen mit ausgebildeten Formen vorhanden. Bei 14 Tage alten Kätzchen ist die Ausbildung der Sternstrahler schon weit gekommen, wie die Fig. 7 darthut. Beim Hunde scheinen diese Zellen oft noch eine viel längere Zeit sparsamer gestrahlt zu bleiben.

Was das Verhalten der Sternstrahler zu den Nervenfaserbündeln betrifft, so war dies schon seit vielen Jahren genau bekannt. In dem oben angeführten Werke von *mir* und Key (1875) ist die Anordnung und Verbreitung dieser Zellen im Opticus so eingehend beschrieben und abgebildet, dass ich auf eine neue Darstellung derselben verzichten kann. Wir zeigten, dass die Hauptmasse der Zellen in den von dem blutgefässführenden bindegewebigen Korbwerk durchzogenen Lymphspalten zwischen den Nervenfaserbündeln angesammelt ist und dass sie ihre zahlreichen feinen Fortsätze nach allen Richtungen hin senden, wodurch sie ein reiches Flechtwerk bilden, und dies nicht nur in den erwähnten Lymphspalten und rings um die Nervenfasernbündel, - die Zellen und ihre Fortsätze sind im Gegentheil, wie aus unseren Abbildungen hervorgeht, auch im Inneren der Nervenfaserbündel reichlich vertreten. Vermittelst der Golgi'schen Methode lassen sich nun diese Verhältnisse sehr schön bestätigen. Nicht nur das Umspinnen der Bündel, sondern auch das Eindringen der Zellen und Zellenfortsätze in das Innere derselben kann man auf Querschnitten des Opticus leicht bestätigen (Fig. 3 der Taf. XIII, vom neugeborenen Kinde); die Zellenfortsätze durchlaufen in verschiedenster Weise die Nervenfaserbündel. Was nun die Formen der Glia-

[1] E. Kallius, Ueber Neurogliazellen in peripherischen Nerven. Nachrichten von d. k. Gesellsch. d. Wiss. etc. in Göttingen 1892.
[2] Michel, Sitz. Ber. d. Würzb. med. naturwiss. Gesellsch. 14 Jan. 1893.
[3] A. S. Dogiel, Neuroglia der Retina des Menschen. Arch. f. mikrosk. Anat. Bd 41, 1893.
[4] E. Kallius, Untersuchungen über die Netzhaut der Säugetiere. Anatom. Hefte, herausgeg. von Merkel und Bonnet, 1894.
[5] S. Ramón y Cajal, La rétine des vertébrés, la Cellule t. 9, 1, 1893 (Dép. 1892).

zellen betrifft, so sind verschiedene Varianten vorhanden; theils sind echte, regelmässige Sternstrahler da, theils gehen, wie DOGIEL hervorhebt, die Fortsätze vom Zellenkörper bündelweise ab, so dass die Zellen mit einem oder mehreren Schwänzen versehen zu sein scheinen. Dies ist besonders an der Oberfläche des Sehnerven der Fall; hier (Fig. 2) sind oft breite, etwas abgeplattete Zellenkörper, ungefähr wie an der Hirnoberfläche, dicht an der Pialscheide belegen, ihre Fortsätze in einem breiten Büschel in den Nerven hineinsendend; diese Zellen sind also als Schwanzstrahler zu bezeichnen. Andere Zellen, welche sich etwas unter der Oberfläche befinden, schicken Fortsätze nach der Pialscheide ab, die mit kleinen Knöpfchen oder Füsschen an ihr endigen (Fig. 1, 2, 5, 8), also einer Art von »Fusssternstrahlern» entsprechen.

In der *Lamina cribrosa* des Sehnerven verändert sich, wie auch *ich* und KEY (1875) eingehender beschrieben haben, die Beschaffenheit der Gliazellen. Die Fortsätze werden spärlicher und kürzer, so dass die Elemente im Ganzen kleiner erscheinen (Fig. 9, vom neugeborenen Hunde) und ordnen sich zugleich mehr quer über den Nerven an. Beim Uebergang zur Papilla n. optici verlängern sich die peripherischen Gliazellen wieder und schicken lange Fortsätze nach der Oberfläche hin, wo dieselben knopfförmig endigen; sie bieten also eine, obwohl unregelmässige Palissadenanordnung dar (Fig. 10). Hier stossen diese Enden der Gliazellen an die sich in den inneren spitzen Opticuswinkel eindringende Retina an (Fig. 10), jedoch ohne einen Uebergang zu den Müller'schen Fasern darzubieten.

Was übrigens die Form und Anordnung der eigentlichen Gliazellen der Retina betrifft, so sind dieselben von mehreren Forschern und in der neueren Zeit auch von CAJAL, DOGIEL und KALLIUS eingehend beschrieben worden, so dass ich sie hier nicht weiter zu berühren brauche.

Hinsichtlich der Müller'schen Fasern, welche offenbar den ependymalen Typus während des ganzen Lebens behalten, kann ich nicht umhin, einige Worte zu äussern. Durch die Golgi'sche und die Ehrlich'sche Methode ist es uns nunmehr vergönnt, über Manches in der Retinastructur, worüber wir früher unsicher, zweifelhaft oder ganz unwissend waren, zu einer klaren Auffassung zu kommen. Das wird wohl einem Jeden klar, der, wie ich, seit mehreren Jahrzehnten mit den alten Methoden an der Retina gearbeitet und die beiden neuen eingehender geprüft hat. Dies betrifft auch das Verhalten der Müller'schen Fasern zu den retikulirten Schichten. Früher konnte ich, wie mehrere andere Retinaforscher, mich nicht von der Verästelung dieser Fasern während des Verlaufs durch die innere retikulirte Schicht überzeugen. Durch die Golgi'sche Methode kam ich aber seit 1888 darüber ins Klare. Die Müller'schen Fasern senden nicht nur in der inneren, sondern oft auch in der äusseren retikulirten Schicht, wie v. A. RAMÓN Y CAJAL bewiesen hat, eine Menge feiner Aeste nach den Seiten hin ab, ungefähr in derselben Weise wie viele Ependymzellen des Gehirns und Rückenmarks. In der Fig. 15 (von der Katze), Fig. 16 (vom Kaninchen) und Fig. 17 (vom Menschen) habe ich einige solche Fasern abgebildet, um nicht fortwährend als Anhänger der alten Lehre von dem Nichtverzweigtsein dieser Fasern zu gelten.

Was die Entwicklung und die verschiedene Gestaltung der Müller'schen Fasern in den verschiedenen Wirbelthierklassen betrifft, so hat RAMÓN Y CAJAL durch seine herrlichen Untersuchungen über die Retina und v. A. in seiner letzten grossen Monographie schon alles Wesentliche darüber mitgetheilt. Ich werde deshalb, obwohl ich seit mehreren Jahren Erfahrungen über den Bau der Retina der verschiedenen Wirbelthierklassen gesammelt habe und eine Arbeit darüber vorbereitete, nicht näher auf den fraglichen Gegenstand eingehen, sondern in Betreff der Müller'schen Fasern nur auf der Taf. XIII (Fig. 11, 12, 13, 14, 17) einige Entwicklungsformen derselben mittheilen. In der Fig. 11 sieht man die Gebilde in ganz embryonalem Zustande; in den Fig. 12, 13 und 14 sind sie noch fadenförmiger geworden und fangen an, sich am inneren Ende zu verästeln. In der Fig. 17 bei $r$ (links) sind sie noch unverästelt, aber bei $r^1$ (rechts), weiter nach hinten in der Retina, haben sie in der inneren retikulirten Schicht feine Aeste und in der inneren Körnerschicht auch zackige Flügelausbreitungen bekommen; dagegen sind ihre äusseren Enden noch unverästelt und ohne Flügel. Was die hier, am äusseren Ende der Fasern, vorhandenen, zwischen die Stäbcheninnenglieder hervorschiessenden, feinen Aeste betrifft, so erkennt man sie in guten Golgi'schen Präparaten stets in schöner Weise; in der Regel sind sie gerade und parallel und reichen, bis zu den Aussengliedern; oft fand ich sie aber auch etwas gewunden und von körnigem, perlschnurartigem Aussehen (Fig. 15); zuweilen waren unter ihnen dickere keulenförmige Gebilde gefärbt (Fig. 16).

Auf der Taf. XIII habe ich, um die Orientirung der Fasern zu verdeutlichen, zusammen mit den Müller'schen Fasern einige andere Retinaelemente aus meinen Präparaten abgebildet. Es sind dies v. A. die von CAJAL

als amakrine Zellen bezeichneten Elemente (Spongioblasten); ich habe besonders die Verhältnisse der sich entwickelnden menschlichen Retina wiedergegeben. Die Fig. 17 der Taf. XIII stellt ein solches Bild dar, wo ich die verschiedenen Elemente zusammengeführt habe. Auf eine Beschreibung kann ich verzichten, da die Figur Alles deutlich zeigt. Nur darauf will ich hinweisen, dass die früher sogenannten inneren Körner in der inneren retikulirten Schicht beim 28 Cm. langen menschlichen Foetus noch keine eigentliche Verästelung ihres inneren Fortsatzes erhalten haben, sondern dass dieser Fortsatz in verschiedener Höhe der Schicht noch mit einem gekörnten *Knötchen* (einer »Wachsthumskeule») endigt und in diesem Stadium der inneren Stäbchenfaser, wie diese stets verbleibt, sehr ähnelt.

Bei dieser Gelegenheit ist es noch meine Pflicht anzugeben, dass ich in Betreff der Structur der retikulirten Schichten seit lange keine *netz*förmige Beschaffenheit derselben mehr annehme. Im Anfang der 80-iger Jahre, wie auch früher im Anfang der 70-iger Jahre, als ich meine Ansichten darüber veröffentlichte, waren die Untersuchungsmethoden nicht geeignet, diese äusserst schwierige Frage zu lösen, wie man auch zu jener Zeit in Betreff der Neuroglia der Centralorgane gar nicht ins Reine kommen konnte. Ich untersuchte damals vorwiegend mit der Ueberosmiumsäure und bekam deshalb bald eine körnige, bald eine netzförmige Structur. Ich hatte mich an solchen Präparaten längst (1870) überzeugt, dass die inneren Fortsätze der inneren Körnerzellen mehr oder weniger tief (bis auf $^3/_4$ der Dicke) in die innere retikulirte Schicht hinabtauchen, ohne dabei verzweigt zu werden; dann entzogen sie sich der weiteren Beobachtung. Ich bestritt deshalb die Lehre von Max Schultze, dass sie sich in dieser Schicht sofort verästeln und in ein diffuses Netzwerk übergehen. Die Golgi'sche, wie auch die Ehrlich'sche Methode haben aber längst dargelegt, dass wir gewissermassen *beide* etwas Recht hatten. Die meisten Fasern dringen ja mehr oder weniger tief *unverästelt* in die Schicht hinab, um sich dann reichlich zu verzweigen. Wie Kallius, glaube ich übrigens auch mehr an eine retikulirte, filzige als an eine *netz*förmige »Grundsubstanz» von der Art, wie sie mir früher die Osmiumpräparate zeigten. Was aber diese »Grundsubstanz» anbetrifft, so sind wir noch nicht über sie und ihre Structur vollständig ins Klare gekommen.

---

Zum Abschluss dieser übersichtlichen Abhandlung über die Neuroglia des Gehirns und einiger ihm mehr oder weniger angehörigen Organe, will ich nur noch betonen, dass ich mich *nie von anastomotischen Verbindungen der Gliazellen und ihrer Fortsätze,* und zwar weder bei den einzelnen Elementen selbst, noch unter den verschiedenen Zellenindividuen, überzeugen konnte. Ueberall, wo klare und sichere Bilder vorlagen, sah ich sie von einander getrennt.

Was dann die so viel besprochene *»Grundsubstanz» der Neuroglia* betrifft, so bin ich immer mehr zu der Auffassung gekommen, dass es keine eigentliche solche giebt. Die Gliazellen mit ihren Fortsätzen bilden die filzig geflochtene Stützsubstanz, in deren Maschen die Nervenzellen mit ihren Fortsätzen aufgehängt liegen; von bindegewebiger Substanz hat man nur die Blutgefässe mit ihren Scheiden, die zuweilen, wie im Rückenmark, abgeplattet und septenähnlich auftreten können. In den Maschen des Gliagewebes giebt es ausserdem nur Gewebssaft. Die homogene, structurlose Grundsubstanz, welche einige Forscher, wie Gierke, angenommen haben, ist nicht histologisch nachweisbar.

*»Gliahüllen»* lassen sich kaum unterscheiden. Zwar bildet die Glia (und das Ependym) an der äusseren und inneren Oberfläche den verdichteten Abschluss gegen die umgebenden Partien (Pia und die Cerebrospinalflüssigkeit der Ventrikel); diese Glia und das Ependym sind aber keine abgegrenzten Hüllen, sondern hängen überall mit dem übrigen unterliegenden Gliagewebe innig zusammen.

Mit der obigen Darstellung der Neuroglia habe ich, wie mehrfach betont ist, nur beabsichtigt, eine übersichtliche Schilderung dieses Gewebes zu geben. Um eine eingehendere Kenntniss dieser merkwürdigen, zuerst von Rudolf Virchow als *Neuroglia* bezeichneten und seitdem in mancher verschiedenen Weise aufgefassten und beschriebenen »Substanz» zu gewinnen, sind noch Untersuchungen in weitem Massstabe erforderlich, und zwar muss jede besondere Partie der Centralorgane bei verschiedenen Wirbelthieren und in verschiedenen Stadien der Entwicklung einzeln durchgearbeitet werden.

Nur ein paar Punkte werde ich diesmal noch berühren. Aus der obigen Darstellung geht es hervor, dass die Gliazellen des Gehirns während ihrer Entwicklung theilweise recht bedeutenden Formveränderungen unterworfen sind. Ihre Substanz muss deshalb wenigstens während des Foetallebens nicht starr, sondern gewissermassen beweglich, umformbar sein, obwohl sie später, v. A. was die Fortsätze betrifft, grossentheils erstarrt. Dies Steiferwerden der Gliaelemente, welches offenbar dazu dient, den echt nervösen Elementen ein geeigneteres Stützwerk darzustellen, schliesst jedoch nicht die Möglichkeit aus, dass den Gliazellen auch *andere Functionen* zukommen können, indem ja im Allgemeinen um den Kern herum etwas »lebensfähiges» Protoplasma vorhanden ist; v. A. aber können solche, z. B. sich auf die Ernährung des Organs, d. h. auf die Zusammensetzung des Gewebssaftes, sich beziehende Einflüsse der Gliaelemente während der Entwicklung — des Foetallebens — vorkommen. Ich habe hier nur auf diese Möglichkeit hinweisen wollen, jedenfalls ohne darauf zu urgiren, da noch keine Beweise vorliegen.

Was die *relative Menge* des Gliagewebes in den verschiedenen Regionen und Schichten des Gehirns betrifft, so ist es recht schwierig darüber etwas Sicheres auszusprechen. Da das Gliagewebe nicht nur eine Stützsubstanz der nervösen Elemente, sondern auch eine »Ausfüllungsmasse» zwischen diesen darstellt, so geht daraus hervor, dass es an den Stellen reichlicher vorhanden ist, wo die nervösen Elemente sparsamer sind und umgekehrt. Deshalb ist es auch an den Oberflächen der Centralorgane reichlicher vertreten als im Inneren, v. A. in den Schichten, wo die Nervenzellen angehäuft liegen. In den Pyramidenzellenschichten, aber auch in der Marksubstanz, ist das Gliagewebe in Folge dessen *relativ* spärlich; doch ist es auch an diesen Orten reichlicher als man zuweilen zu glauben scheint. Die Golgi'sche Methode kann bekanntlich in dieser Hinsicht nicht immer richtige Aufschlüsse geben; doch trifft man auch oft in Golgi'schen Präparaten Partien, z. B. von den Pyramidenzellenschichten, welche ein so dichtes Gewirr von Sternstrahlern enthalten, dass man sie mit den Blicken nicht durchdringen vermag. Die Nervenzellen liegen in eine moosige Masse so eingelagert, dass man sie kaum wahrnehmen kann.

Was endlich die Frage von dem Verhältniss der eigentlichen Neuroglia zu dem *Ependym* betrifft, so ist es nicht meine Absicht, diesmal auf diese Frage einzugehen. Dass auch im Gehirn, wie im Rückenmark, innige Verwandtschafts-Beziehungen zwischen diesen Gewebsarten vorhanden sind, geht aus manchen Umständen hervor. Dagegen scheint mir gerade im Gehirn eine histologische Differenzirung früher einzutreten als im Rückenmark.

Von besonderem Interesse ist das hier oben dargelegte Verhalten, dass die Ependymzellen, beim Menschen noch in den späteren Perioden des Foetallebens und bei Thieren noch nach der Geburt, bis an die Oberfläche der Hirnrinde noch in ihrer charakteristischen Gestalt nachweisbar sind.

## Tafel I.

# Die Neuroglia der Grosshirnrinde des Menschen.

**Fig. 1.** Vertikalschnitt der Grosshirnrinde eines $6^1/_2$ *Monat alten menschlichen Foetus.* Verschiedene Typen von Neurogliazellen in ihrer natürlichen Lage in der Rinde; — *c,* eine Cajal'sche Zelle; — *e,* Ependymzellenenden.

**Fig. 2.** Tangentialschnitt der Rindenoberfläche desselben *Foetus.* Neurogliazellen.

**Fig. 3.** Partie aus der Rinde desselben *Foetus* (etwa 2.5 Mm.) unter der Oberfläche. Ein Blutgefäss mit sich an ihn ansetzenden Neurogliazellen.

**Fig. 4.** Vertikalschnitt der Grosshirnrinde (Frontallappen) eines *19.5 Cm. langen menschlichen Embryos.* Aeussere Enden der Ependymzellen.

---

Die Figuren dieser Tafel sind nach Golgi'schen Präparaten bei Vér. Obj. 6 und Ocul. 3 (eingeschob. Tubus) gezeichnet.

*Fig. 1.*

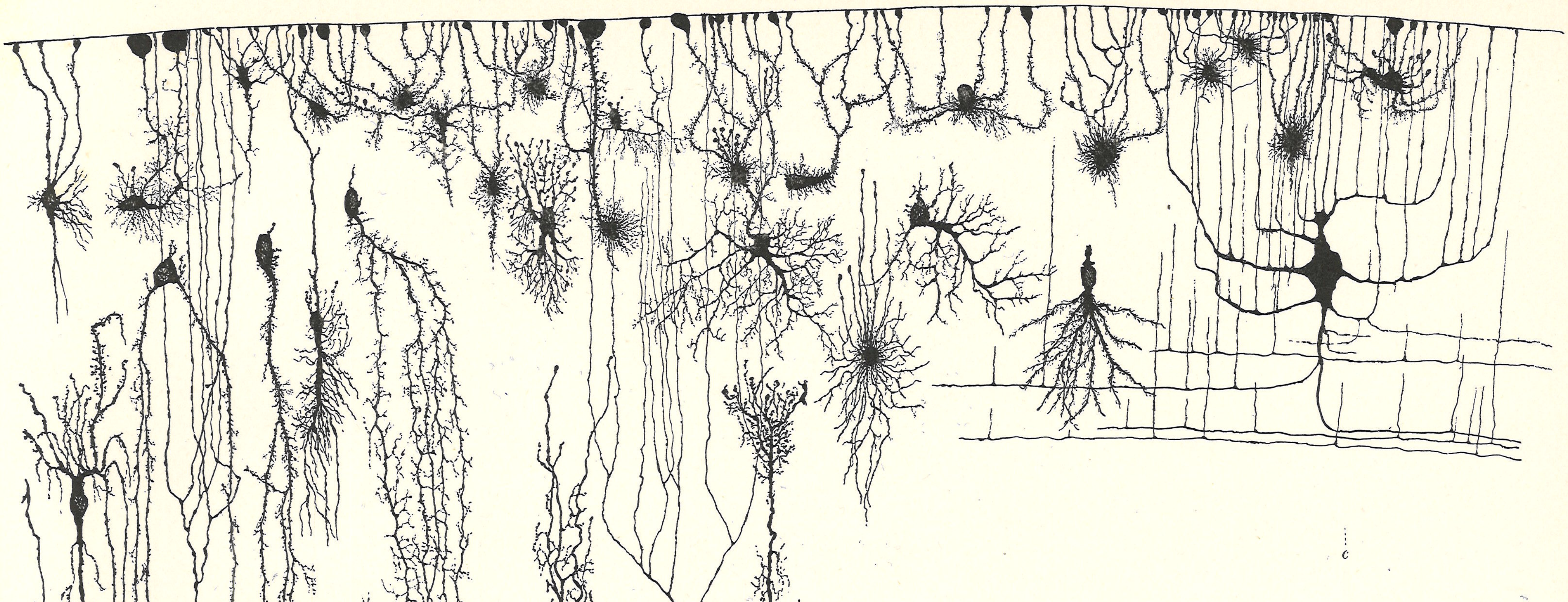

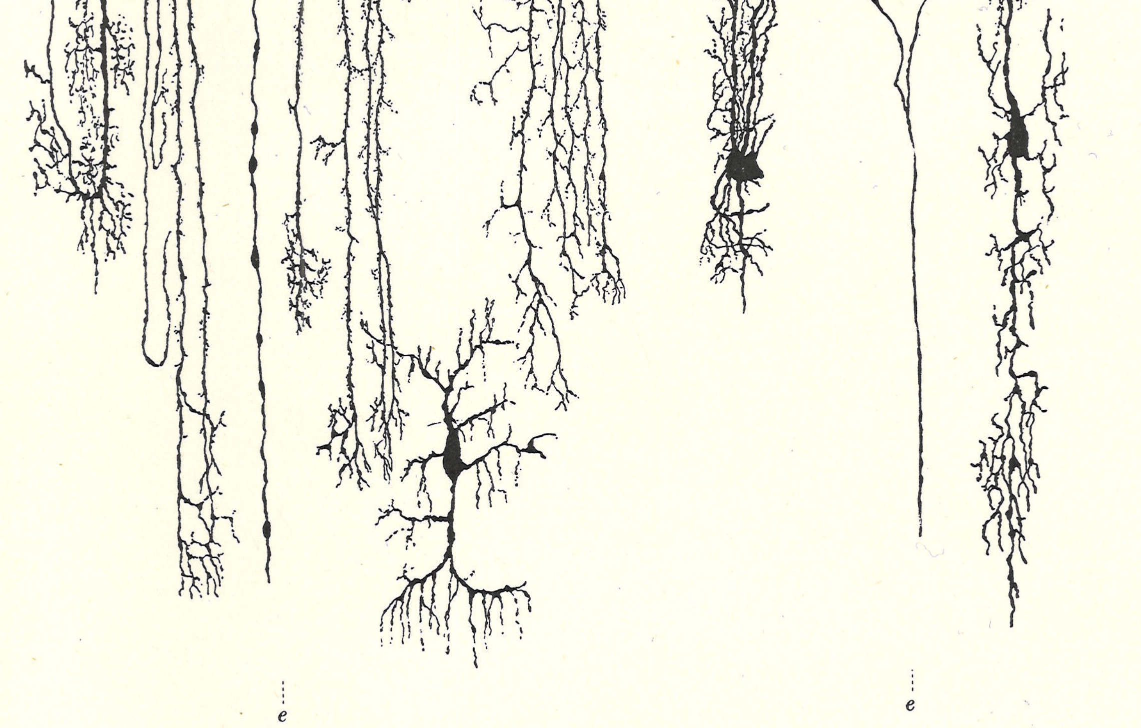


*Fig. 2.*

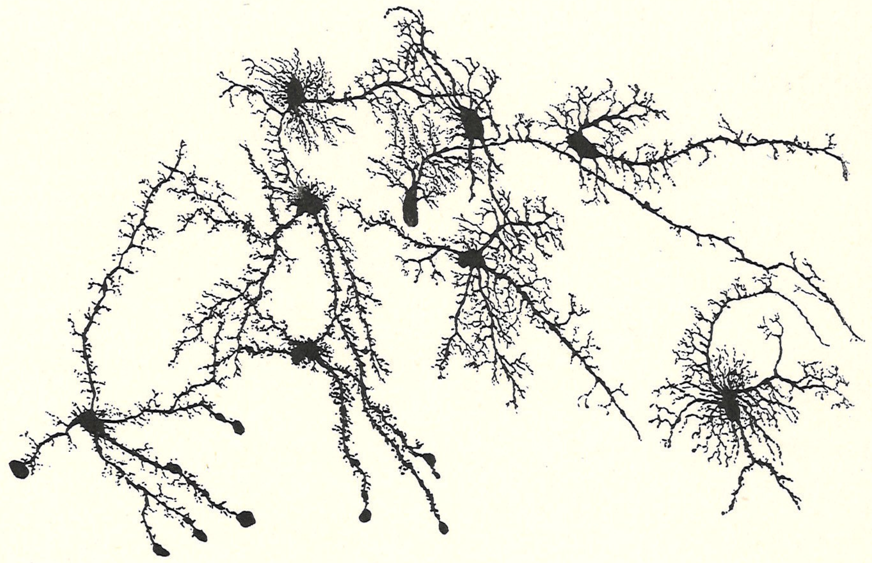

*Fig. 4.*

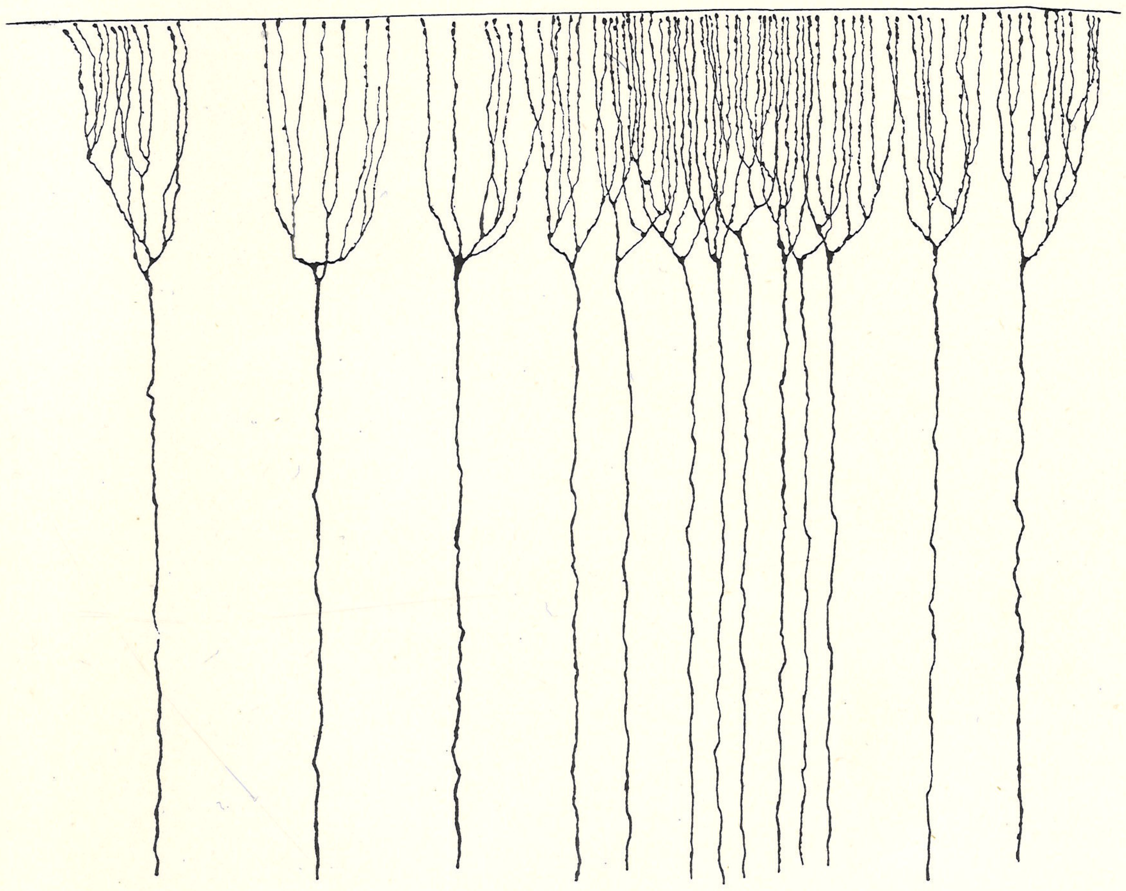

*Fig. 3.*

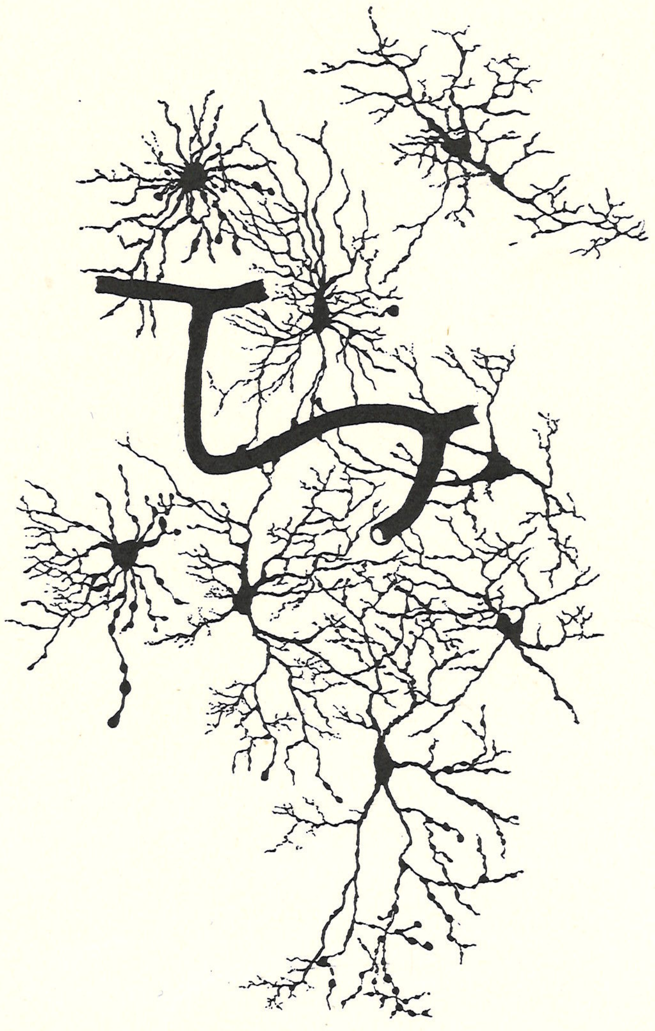

Gez. v. Gustaf Retzius. Typogr. Central-Tryckeriet. Fototypi v. J. Cederquist.

## Tafel II.

# Die Neuroglia der Grosshirnrinde des Menschen.

**Fig. 1.** Vertikalschnitt der Rinde einer Windung des Temporallappens von einem ausgetragenen *48 Cm. langen (weiblichen) menschlichen Foetus.* Neurogliazellen verschiedener Gestalt, die meisten von foetalen Typen.

**Fig. 2.** Ein Sternstrahler aus der inneren weissen Substanz des Präparates, welches in der Fig. 1 wiedergegeben ist.

**Fig. 3.** Tangentialschnitt der Rindenoberfläche einer Windung des Temporallappens von einem ausgetragenen *48 Cm. langen menschlichen Foetus.* Vier Zellenkörper von Schwanzsternstrahlern mit Fortsätzen von foetalem Typus.

**Fig. 4 und 5.** Vertikalschnitte der Rinde von Windungen des Frontallappens von einem *45 Cm. langen menschlichen Foetus.* Neurogliazellen sind in ihren verschiedenen foetalen Typen dargestellt. In der Fig. 5 sind ausserdem zwei Kleinpyramidenzellen und einige tangentiale Fasern (wahrscheinlich Fortsätze Cajal'scher Zellen), ebenso (unten rechts) ein von einer Gliazelle umstricktes Blutgefäss wiedergegeben.

---

Sämmtliche Figuren der Tafel sind nach Golgi'schen Präparaten bei Vér. Obj. 6 und Ocul. 3 (eingeschob. Tubus) gezeichnet.

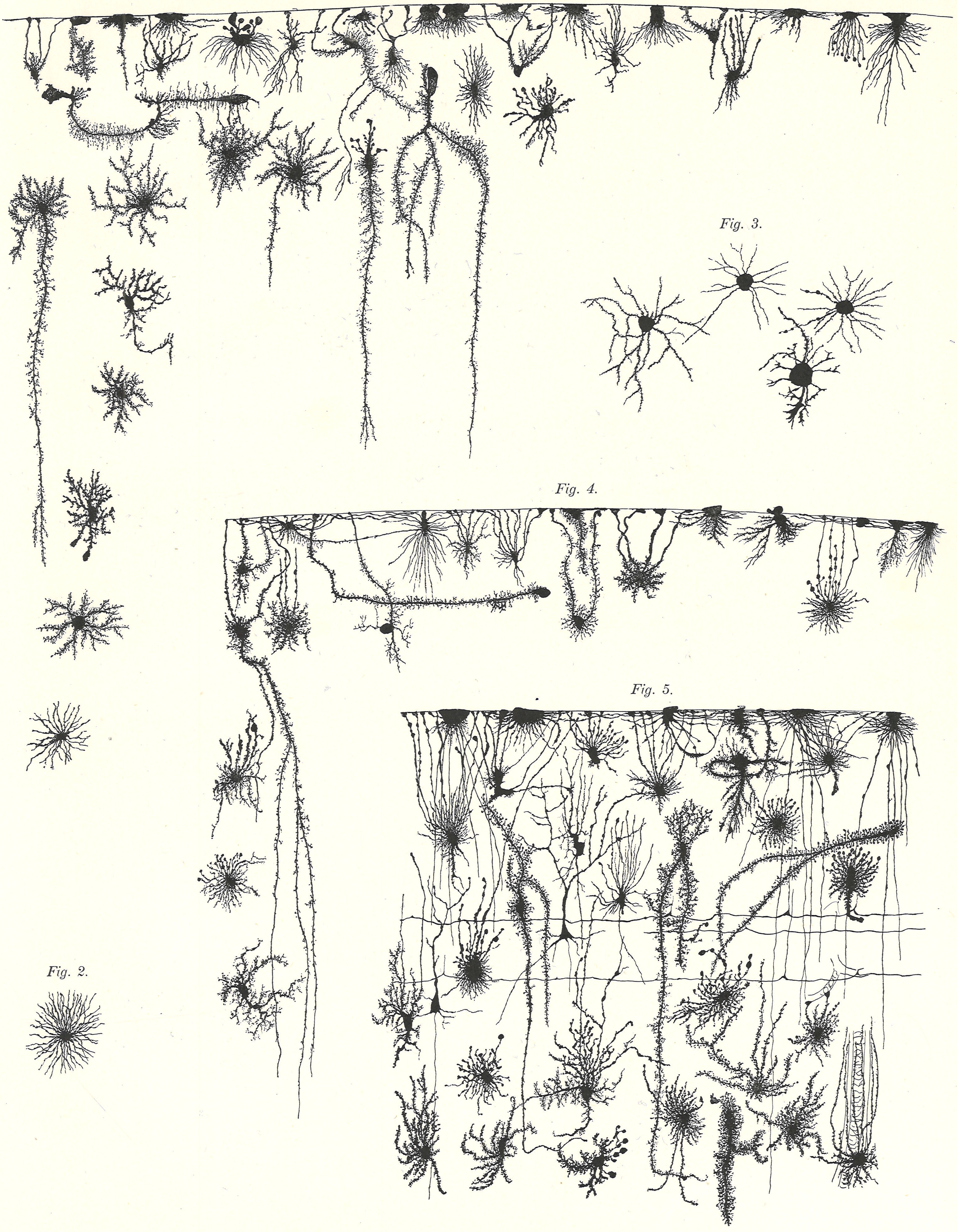

*Fig. 1.* *Fig. 2.* *Fig. 3.* *Fig. 4.* *Fig. 5.*

Gez. v. Gustaf Retzius. Typogr. Central-Tryckeriet. Fototypi v. J. Cederquist.

# Tafel III.

## Die Neuroglia der Grosshirnrinde des Menschen.

**Fig. 1.** Vertikalschnitt einer Windung (G. front. med.) der Grosshirnrinde eines *2 Monate alten Kindes.* Neurogliazellen von verschiedenen Typen in ihrer natürlichen Lage in der Rinde. In der Mitte dringt ein Blutgefäss von der Oberfläche hinab. Einige Tangentialfasern sind weiter nach unten sichtbar.

**Fig. 2.** Drei Sternstrahler aus einer tieferen Partie desselben Vertikalschnittes.

**Fig. 3.** Tangentialschnitt der Oberfläche der Rinde (Gyr. temp. inf.) eines *2 Monate alten Kindes.* Neurogliazellen von der Oberfläche her betrachtet.

**Fig. 4.** Vertikalschnitt der Rinde einer Frontallappen-Windung eines *3 Monate alten Kindes.* Neurogliazellen von verschiedenen Typen in ihrer Lage in der Rinde.

**Fig. 5.** Vier Neurogliazellen der Rindenoberfläche in tangentialer Ausbreitung (Tangentialschnitt der Rindenoberfläche).

---

Die Figuren dieser Tafel sind nach Golgi'schen Präparaten bei Vér. Obj. 6 und Ocul. 3 (eingeschob. Tubus) gezeichnet.

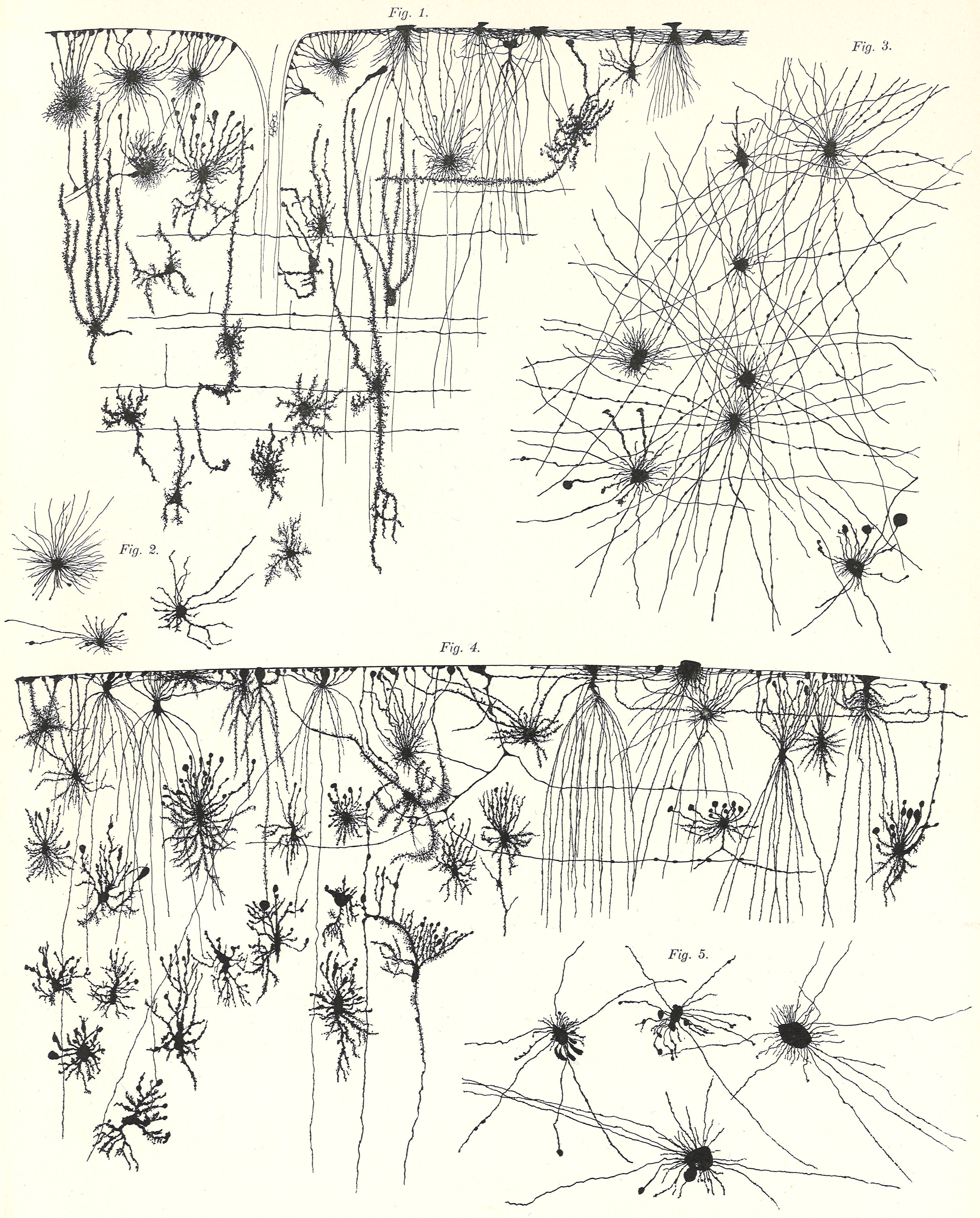

Fig. 1. Fig. 2. Fig. 3. Fig. 4. Fig. 5.

Gez. v. Gustaf Retzius. Typogr. Central-Tryckeriet. Fototypi v. J. Cederquist.

## Tafel IV.

# Die Neurogliazellen der Grosshirnrinde des Menschen.

**Fig. 1.** Vertikalschnitt der Rinde einer Windung des Frontallappens von einem *1-jährigen Kinde.* Schwanzstrahler, Sternstrahler und Fusssternstrahler.

**Fig. 2.** Vertikalschnitt der Rinde einer Windung des Frontallappens von einem $5^1/_2$ *Jahr alten Kinde.* Neurogliazellen von verschiedenen Typen.

**Fig. 3.** Vertikalschnitt der Rinde einer Windung des Occipitallappens (Gyr. occ. med.) von einem *17-jährigen Manne.* Neurogliazellen von verschiedenen Typen und eine Pyramidenzelle.

**Fig. 4.** Vertikalschnitt einer Windung des Parietallappens (Gyr. centr. post.) von einem *33-jährigen Manne.* Neurogliazellen von verschiedenen Typen.

**Fig. 5.** Tangentialschnitt der Rindenoberfläche (Gyr. centr. post.) eines *42-jährigen Weibes.* Flächen- und Schwanzstrahler sowie tiefer belegene Sternstrahler.

---

Die Figuren dieser Tafel sind nach Golgi'schen Präparaten bei Vér. Obj. 6 und Ocul. 3 (eingeschob. Tubus) gezeichnet.

*Fig. 2.*

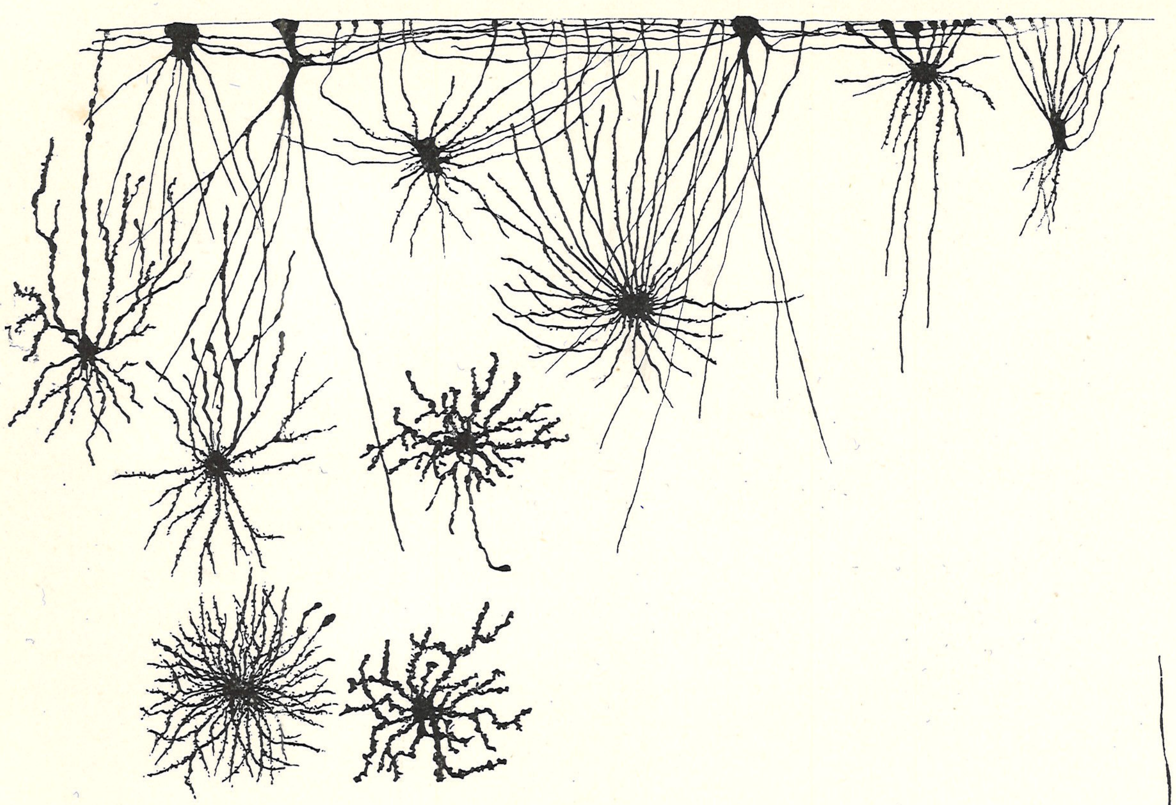

*Fig. 5.*

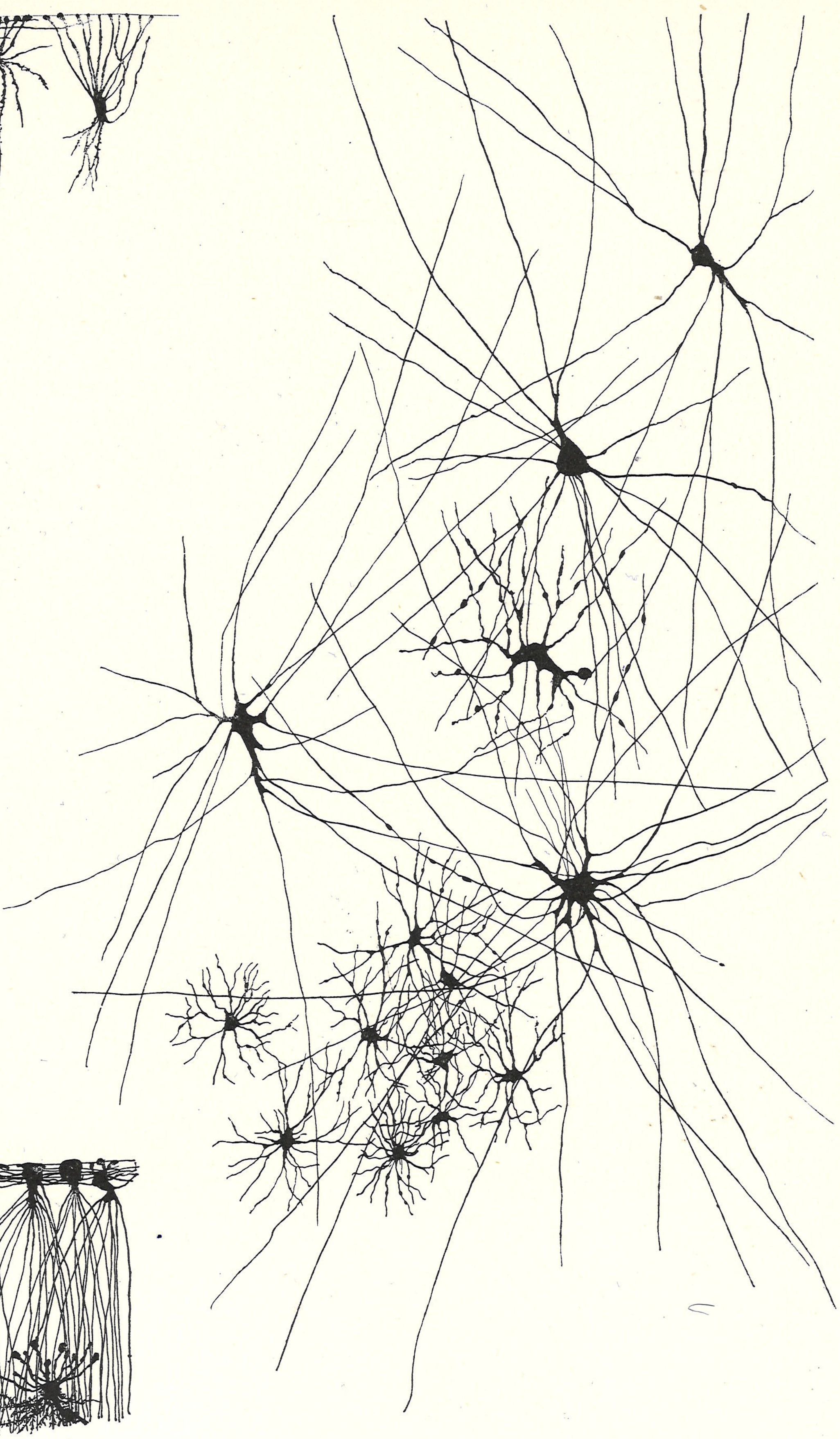

*Fig. 3.*

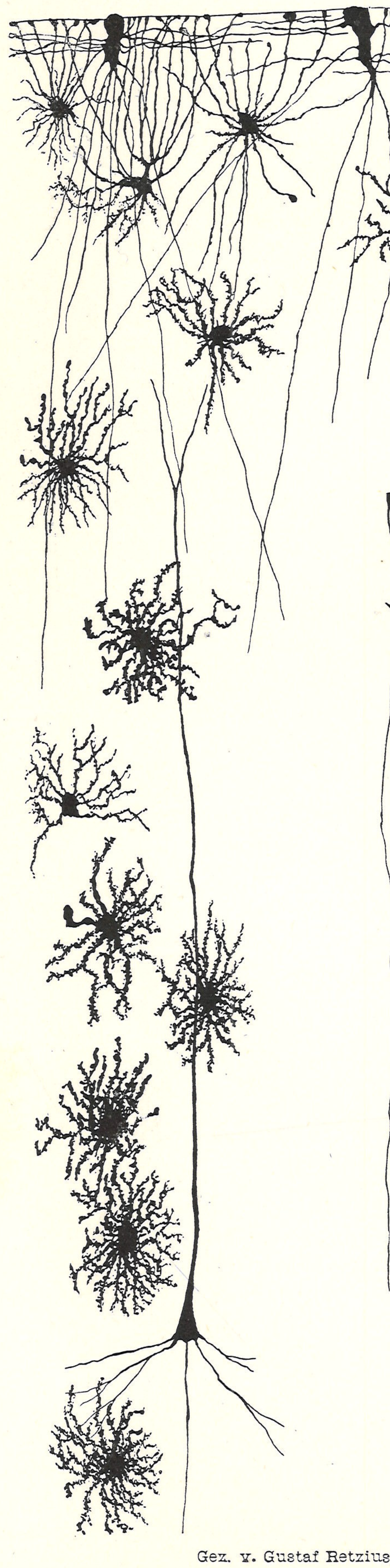

*Fig. 1.*

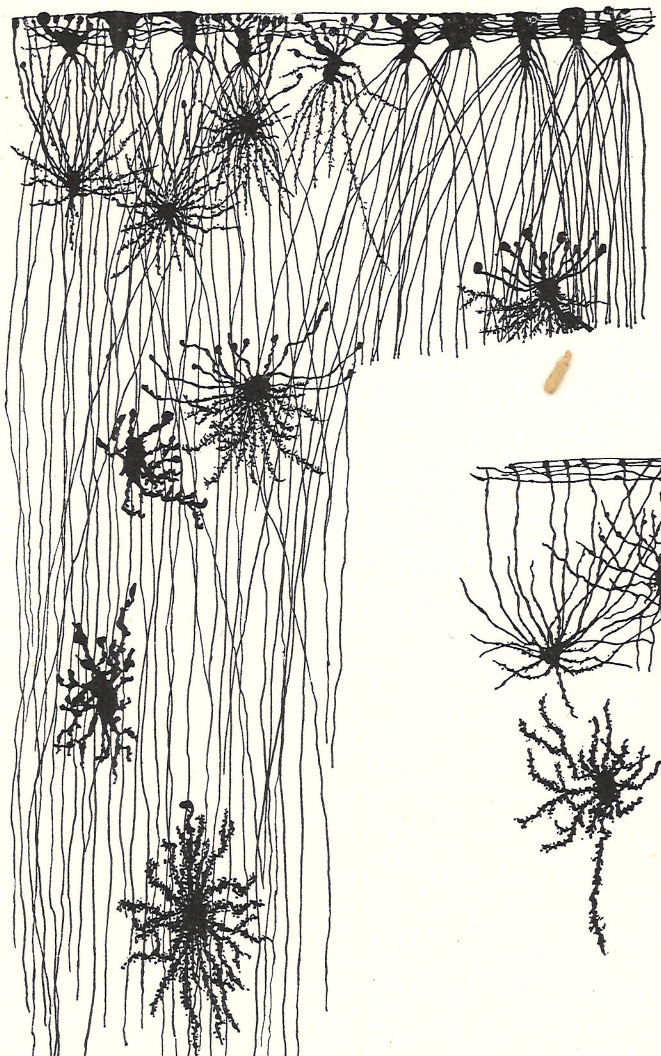

*Fig. 4.*

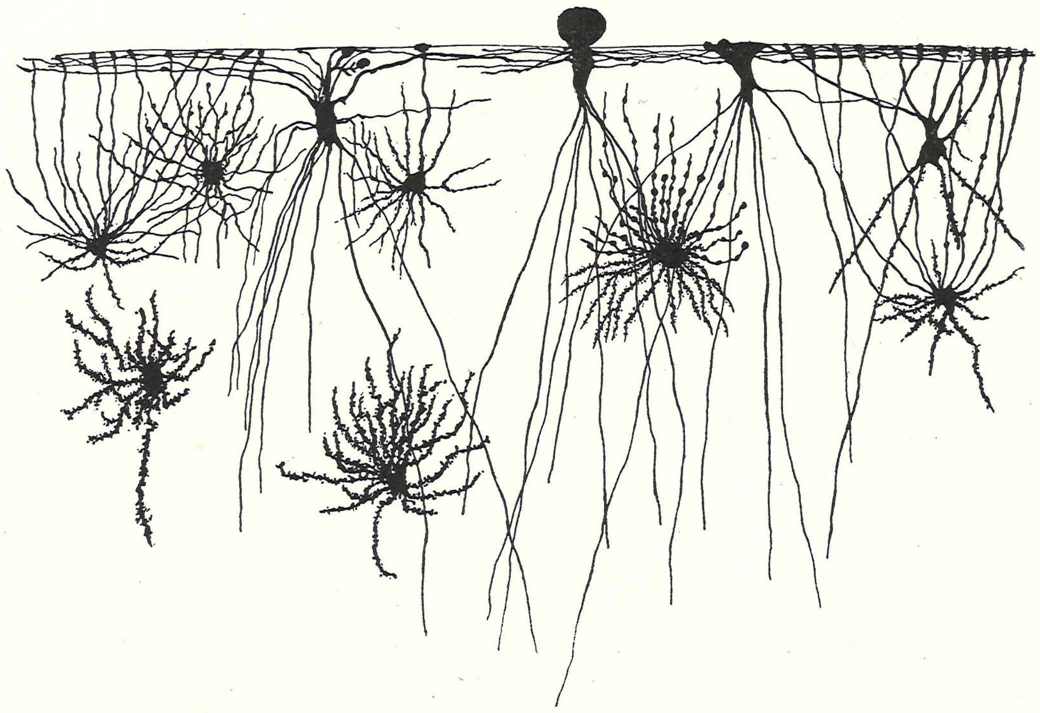

Gez. v. Gustaf Retzius. Typogr. Central-Tryckeriet. Fototypi v. J. Cederquist.

## Tafel V.

# Die Neuroglia der Grosshirnrinde des Menschen.

**Fig 1.** Vertikalschnitt eines Gyrus des Frontallappens, von einem *42-jährigen* Weibe.
**Fig. 2—5.** Vertikalschnitte aus der parietalen Region, von einem *70-jährigen* Manne.

---

In allen Figuren der Tafel ist die Oberfläche der Gehirnrinde nach der oberen Seite der Tafel gerichtet. Man sieht deshalb am oberen Rande der Figuren die Neurogliazellen, welche die Oberfläche erreichen und sich an ihr ausbreiten; unter diesen Zellen sieht man die tiefer belegenen Neurogliazellen in ihren verschiedenen Typen. In den Fig. 1 und 2 sind Blutgefässe vorhanden, an denen Neurogliazellen befestigt sind. In der Fig. 5 findet sich (oben links) eine gefärbte kleine Ganglienzelle. Sonst sind alle die abgebildeten Elemente Neurogliazellen.

Alle Figuren der Tafel sind nach Präparaten, nach der Golgi'schen Methode behandelt, wiedergegeben und bei Vér. Obj. 6 und Ocul. 3 (eingeschob. Tubus) gezeichnet.

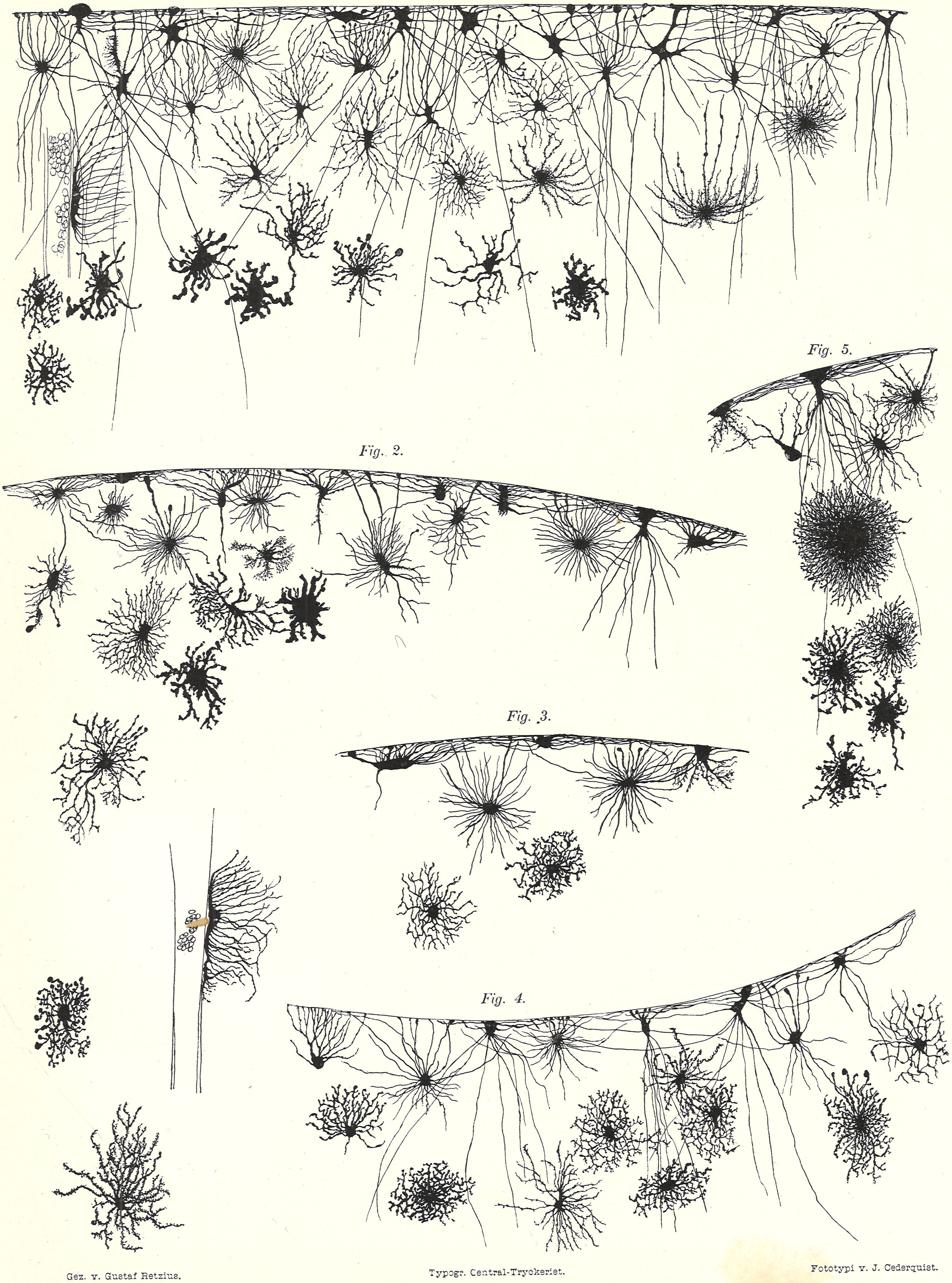

Fig. 1.

Fig. 2.

Fig. 3.

Fig. 4.

Fig. 5.

Gez. v. Gustaf Retzius.

Typogr. Central-Tryckeriet.

Fototypi v. J. Cederquist.

## Tafel VI.

# Die Neuroglia der Grosshirnhemisphären des Hundes.

**Fig. 1.** Vertikalschnitt eines Gyrus vom Vertex des Grosshirns eines *2 Monate alten Hundes.*

**Fig. 2.** Drei Langsternstrahler aus der Marksubstanz desselben Präparates, dessen Oberflächenpartie in der Fig. 1 wiedergegeben ist (v. *2 Monate alten Hunde).*

**Fig. 3.** Vertikalschnitt eines Gyrus vom Vertex des Grosshirns eines *1 Monat alten Hundes;* an dem Oberflächenrande sind zwei Blutgefässe der Pia mit anhaftenden Zellkörpern der Schwanzstrahler abgebildet.

**Fig. 4.** Partien von Tangentialschnitten der Oberfläche der Hemisphärenrinde eines *2 Monate alten Hundes.* — *a,* zwei an der Oberfläche belegene Gliazellkörper mit ihren Fortsätzen. — *b,* Partie der Oberfläche mit der Mosaikzeichnung, welche den Contouren der Zellkörper der an der Oberfläche befindlichen Gliazellen entspricht; zwei Zellkörper sind ganz gefärbt, von denen der eine mit gefärbten Fortsätzen versehen ist.

**Fig. 5 und 6.** Vertikalschnitte von Gyri vom Vertex des Grosshirns eines *erwachsenen (alten) Hundes.*

**Fig. 7.** Vertikalschnitt eines Gyrus vom Vertex des Grosshirns eines *5 Tage alten Hundes.* Ausser drei unter der Oberfläche befindlichen Gliazellen sind in der Figur die äusseren Enden von sechs Ependymzellen wiedergegeben.

---

In den Fig. 1, 3, 5, 6, 7 ist der Rand der vertikal durchgeschnittenen Hirnoberfläche nach der oberen Seite der Tafel gerichtet.

Alle Figuren der Tafel sind nach Präparaten abgebildet, welche nach der Golgi'schen Methode behandelt waren, und bei Vér. Obj. 6 und Ocul. 3 (eingeschob. Tubus) gezeichnet.

*Fig. 1.*

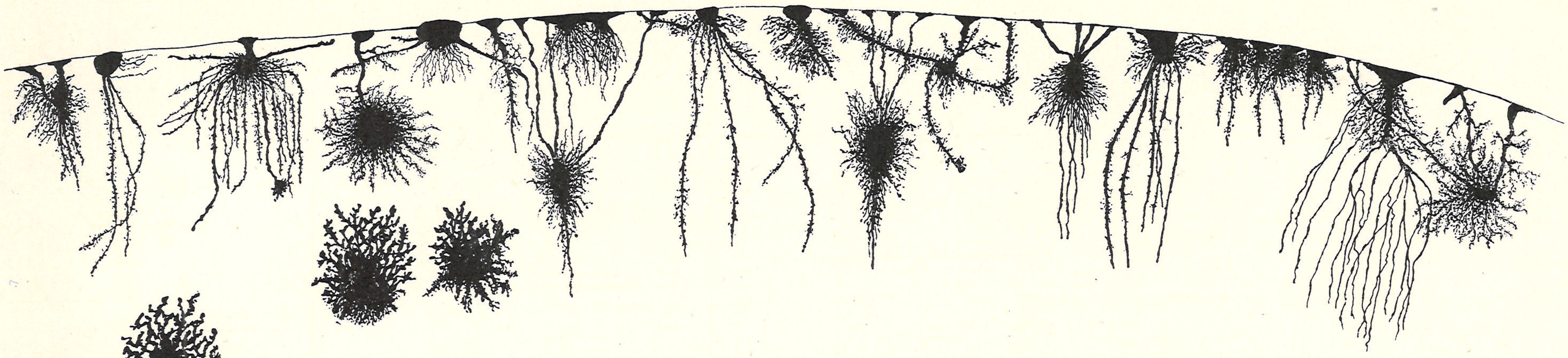

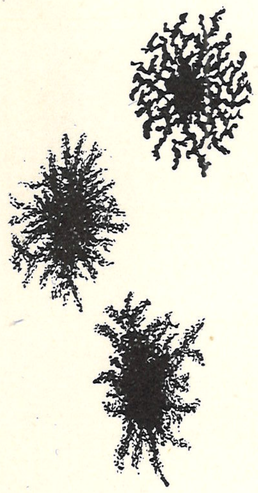

*Fig. 3.*

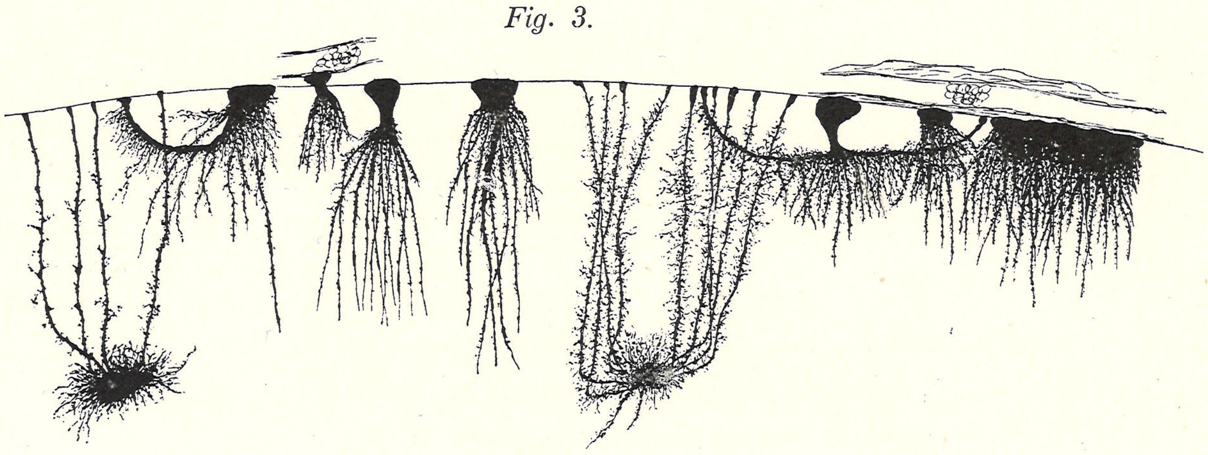

*Fig. 7.*

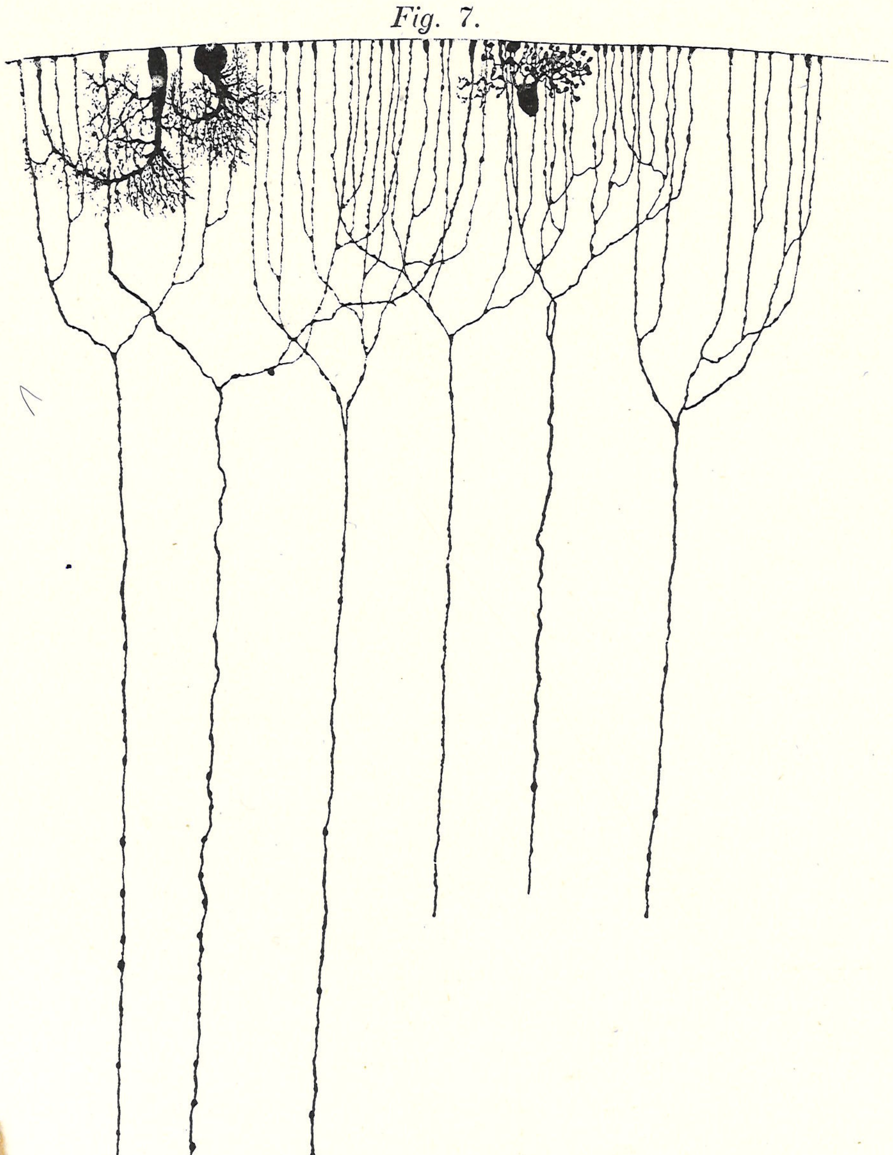

*Fig. 2.*

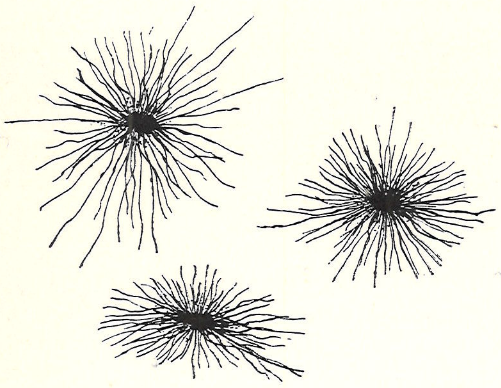

*Fig. 5.*

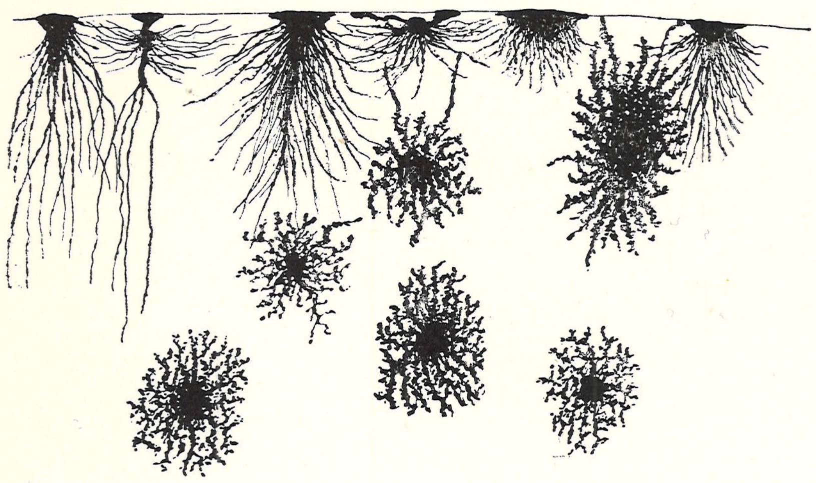

*Fig. 4.*

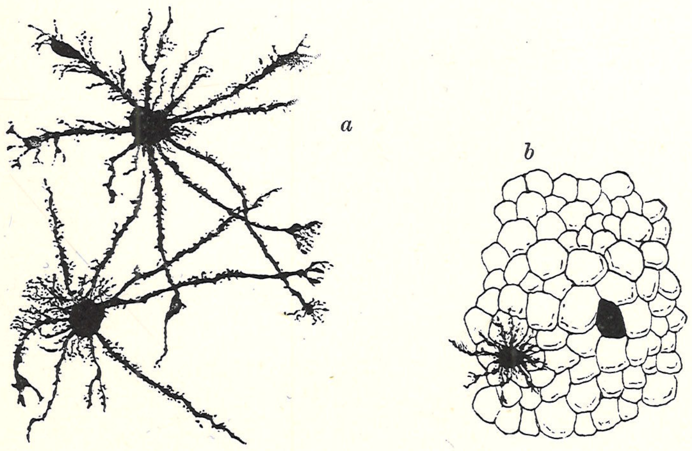


*Fig. 6.*

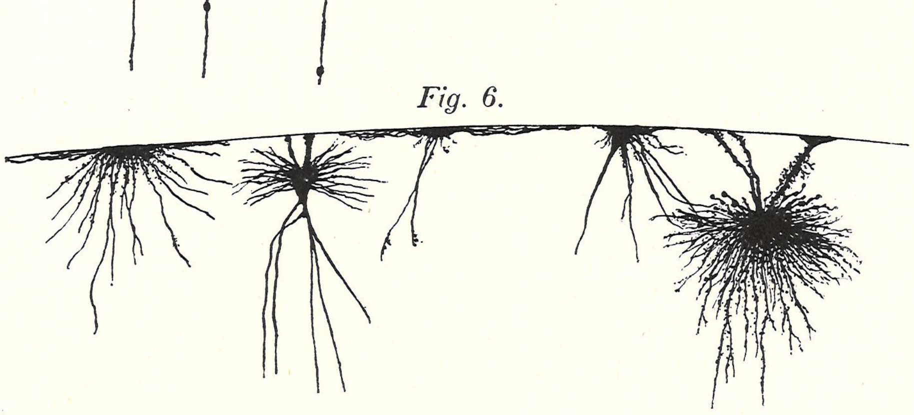

Gez. v. Gustaf Retzius. Typogr. Central-Tryckeriet. Fototypi v. J. Cederquist.

## Tafel VII.

# Die Neuroglia der Grosshirnhemisphären der Katze.

**Fig. 1.** Vertikalschnitt vom Vertex des Gehirns einer *8 Tage alten Katze;* die Gliazellen sind noch vom foetalen Typus.

**Fig. 2.** Vertikalschnitt vom Vertex des Gehirns einer *13 Tage alten Katze.* Neben den Gliazellen sind die äusseren Enden von zwei Ependymzellen wiedergegeben.

**Fig. 3.** Vertikalschnitt vom Vertex des Gehirns einer *15 Tage alten Katze.*

**Fig. 4.** Vertikalschnitt vom Vertex des Gehirns einer *erwachsenen (alten) Katze.*

**Fig. 5.** Drei Langsternstrahler aus der weissen Substanz unter der Rinde des Vertex des Gehirns einer *erwachsenen (alten) Katze.*

**Fig. 6—8.** Tangentialschnitte der Hirnoberfläche am Vertex. Von einer *erwachsenen (alten) Katze.* An der Oberfläche belegene Gliazellkörper mit Fortsätzen.

**Fig. 9.** Eine an einer Blutgefässscheide anhaftende Gliazelle, aus der Grosshirnrinde einer *erwachsenen (alten) Katze.*

---

In den Fig. 1—4 ist der Oberflächenrand nach der oberen Seite der Tafel gerichtet.

Sämmtliche Figuren der Tafel sind nach Präparaten wiedergegeben, die nach der Golgi'schen Methode behandelt waren, und bei Vér. Obj. 6 und. Ocul. 3 (eingeschob. Tubus) abgebildet.

*Fig. 1.*

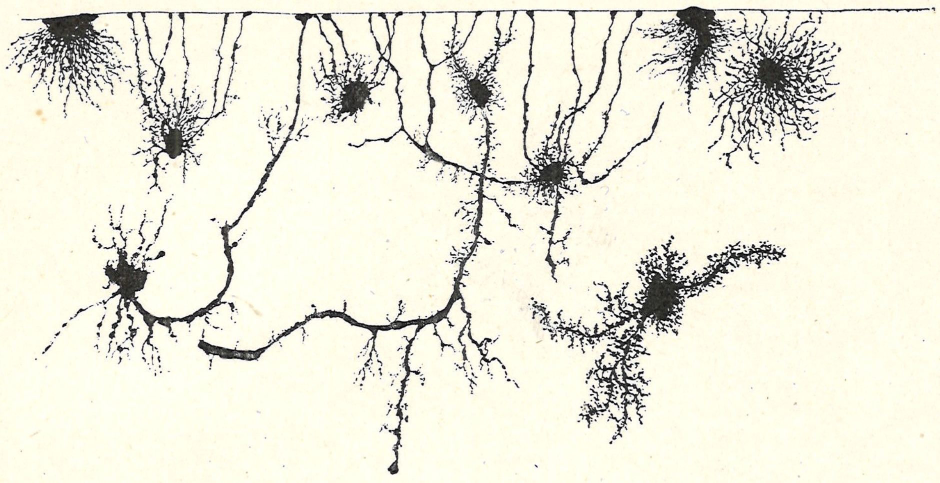

*Fig. 3.*

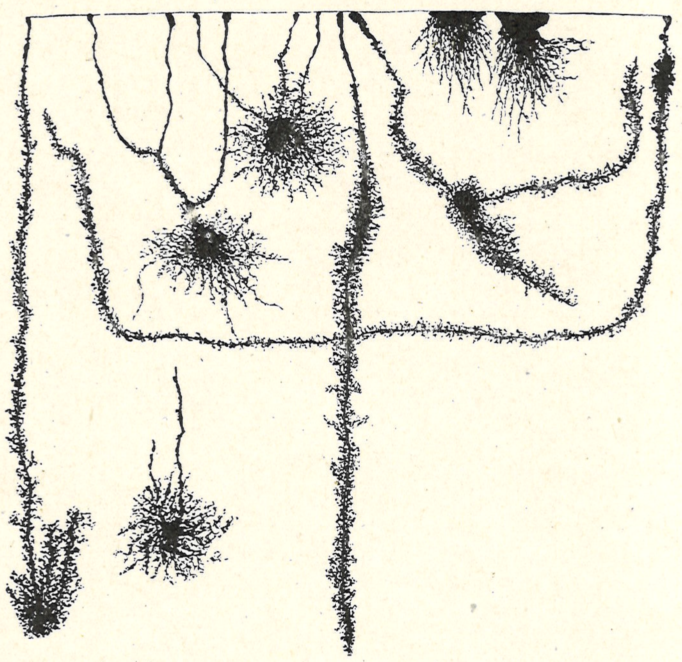

*Fig. 4.*

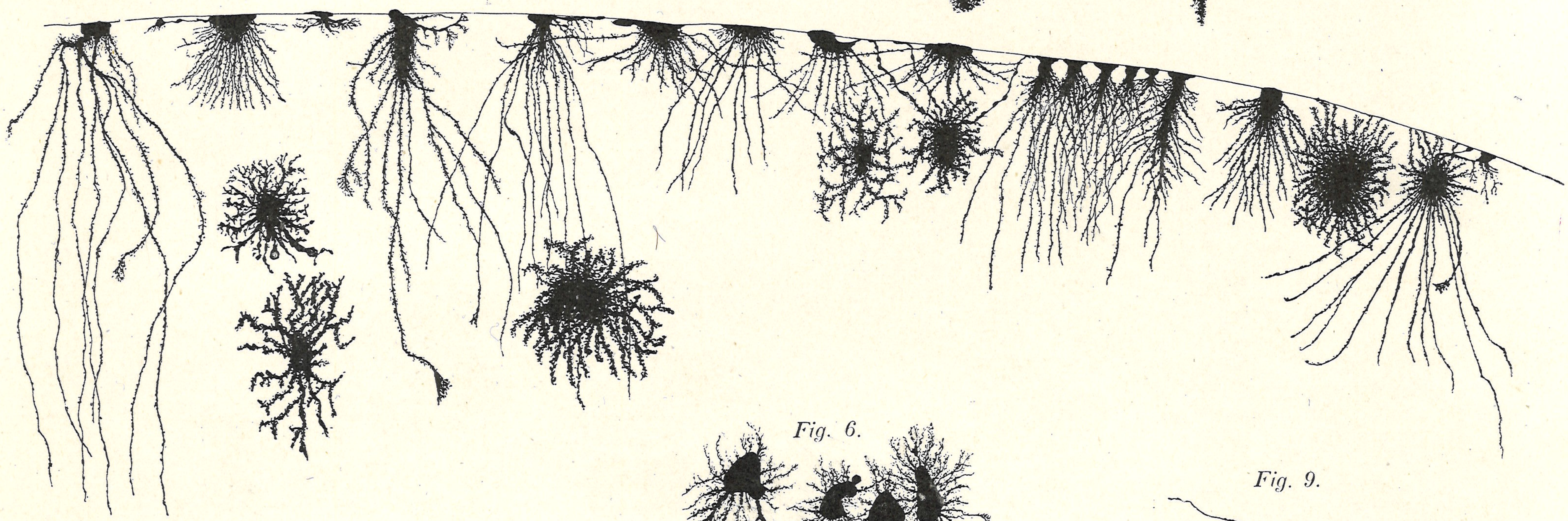

*Fig. 6.*

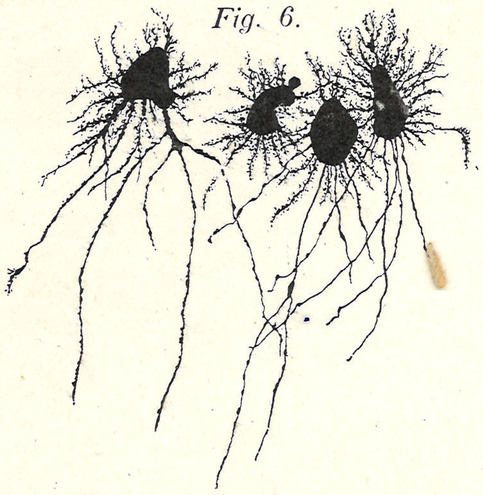

*Fig. 9.*

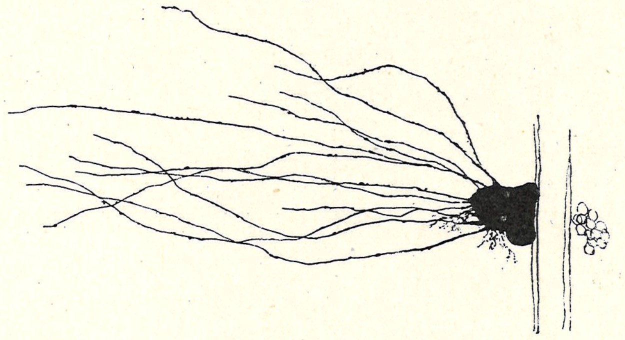

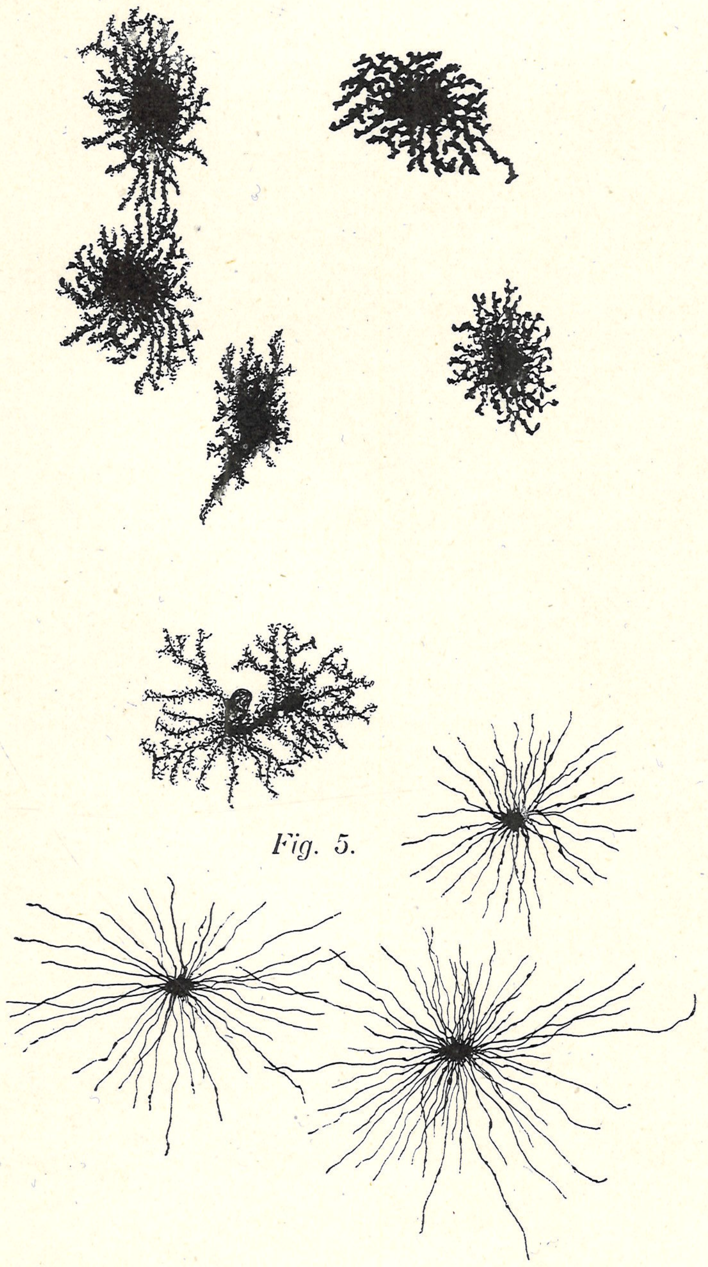

*Fig. 5.*

*Fig. 7.*

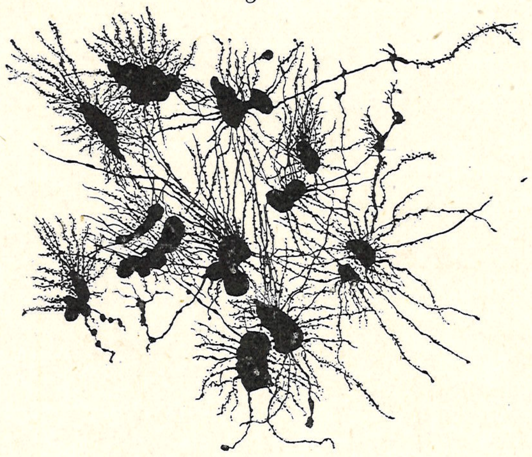

*Fig. 2.*

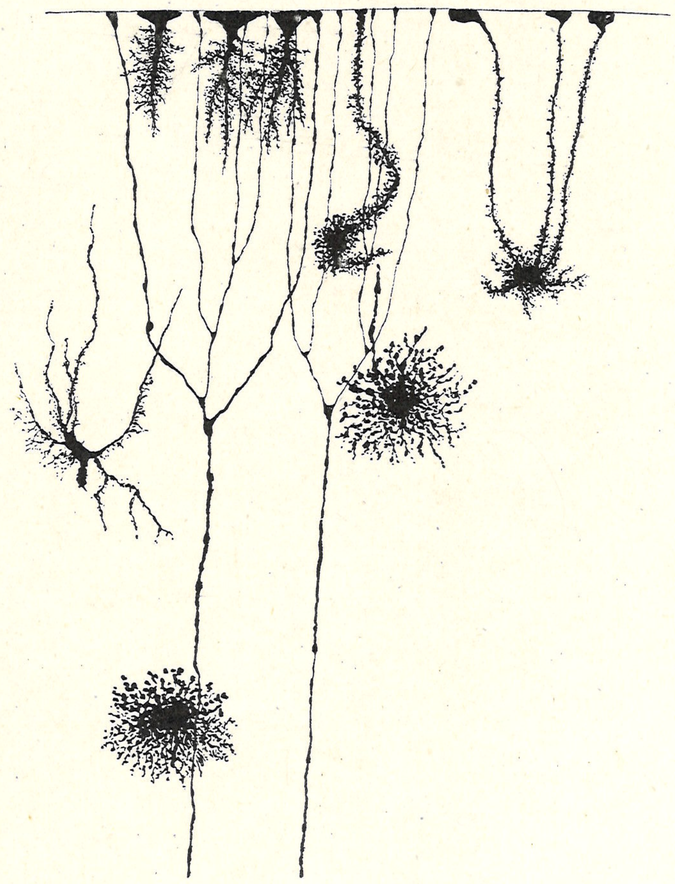

*Fig. 8.*

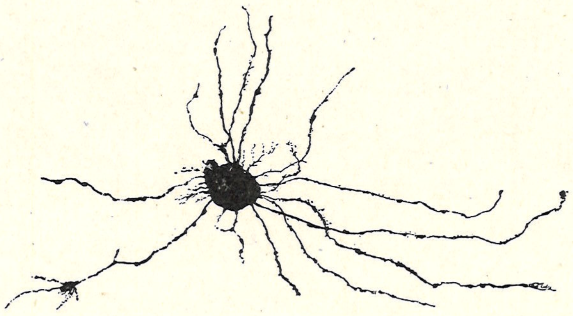

Gez. v. Gustaf Retzius. Typogr. Central-Tryckeriet. Fototypi v. J. Cederquist.

## Tafel VIII.

## Die Neuroglia der Grosshirnhemisphären des Kaninchens.

**Fig. 1.** Vertikalschnitt vom Vertex des Grosshirns eines *14 Tage alten Kaninchens.*

**Fig. 2.** Vertikalschnitt vom Vertex des Grosshirns eines *4 Tage alten Kaninchens.* Eine Cajal'sche Zelle ist von Gliazellen umgeben abgebildet.

**Fig. 3.** Vertikalschnitt vom vorderen Ende des Grosshirns eines *$1\frac{1}{2}$ Tage alten Kaninchens.* Rechts von der Gliazellengruppe ist eine Cajal'sche Zelle wiedergegeben.

**Fig. 4.** Vertikalschnitt vom Vertex des Grosshirns eines *erwachsenen (alten) Kaninchens.*

**Fig. 5.** Vertikalschnitt vom Vertex des Grosshirns eines *neugeborenen Kaninchens.* Unter den vier Gliazellen ist eine Ganglienzelle wiedergegeben.

**Fig. 6.** Vertikalschnitt der Rindenpartie des Grosshirns (Vertex) eines *5 Wochen alten Kaninchens.* Sechs Schwanzstrahler mit den an der Oberfläche befindlichen Zellkörpern.

**Fig. 7.** Vertikalschnitt vom Vertex des Grosshirns einer *erwachsenen (alten) Katze.*

**Fig. 8.** Vertikalschnitt aus den *Corpora quadrigemina* eines *8 Tage alten Kaninchens,* um des Vergleiches wegen die Form der Neurogliazellen dieser Hirnregion beim Kaninchen zu zeigen.

---

Sämmtliche Figuren der Tafel sind mit der Rindenkante nach der oberen Seite der Tafel gerichtet und bei Vér. Obj. 6 und Ocul. 3 (eingeschob. Tubus) wiedergegeben.

*Fig. 1.*

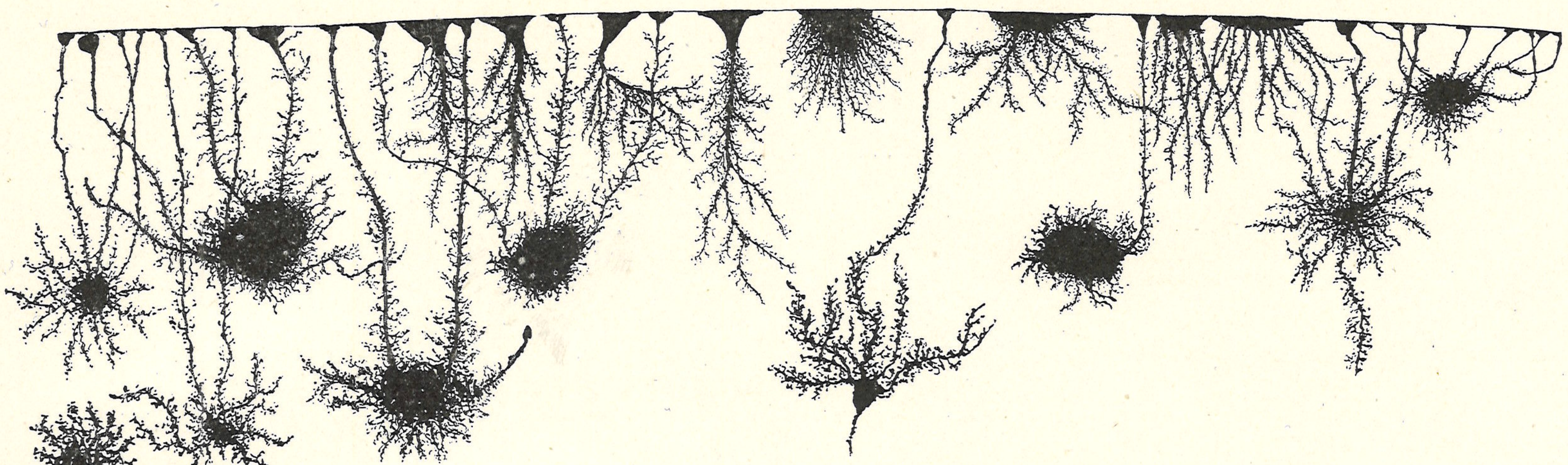

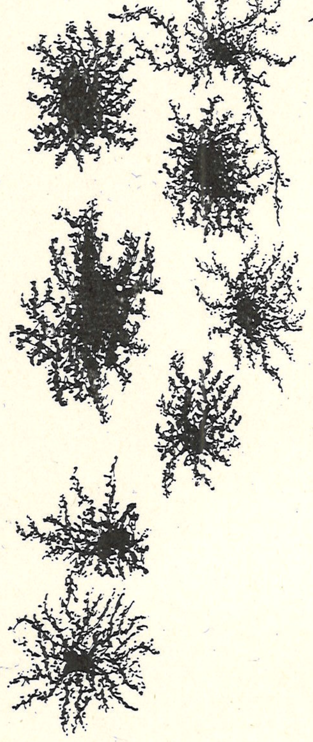

*Fig. 2.*

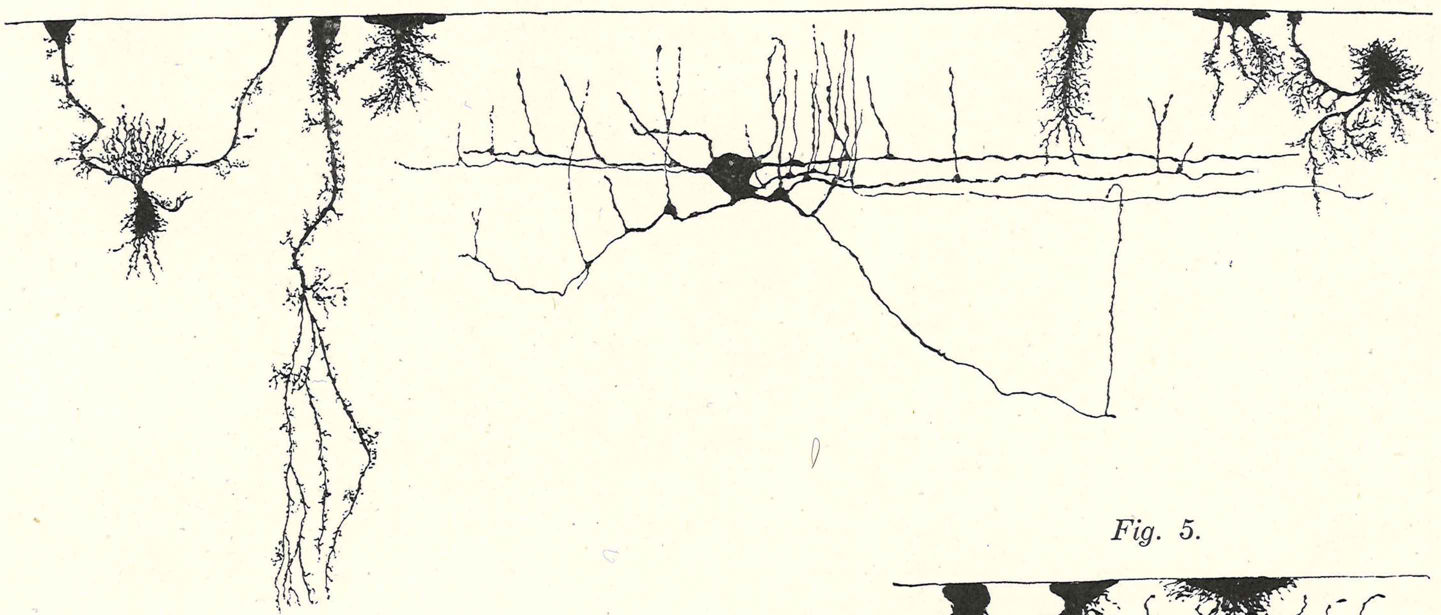

*Fig. 5.*

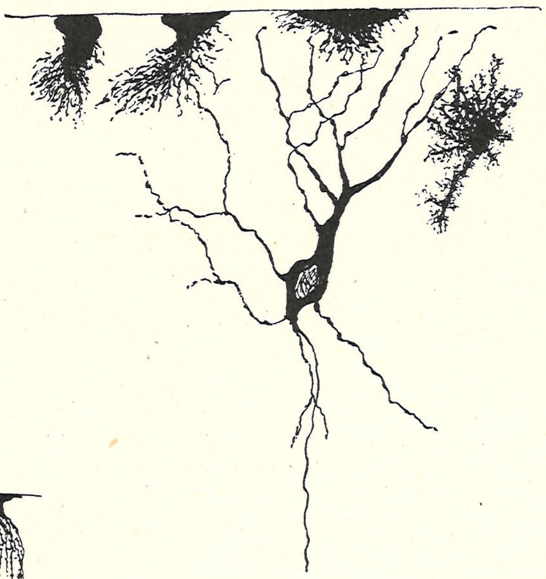

*Fig. 3.*

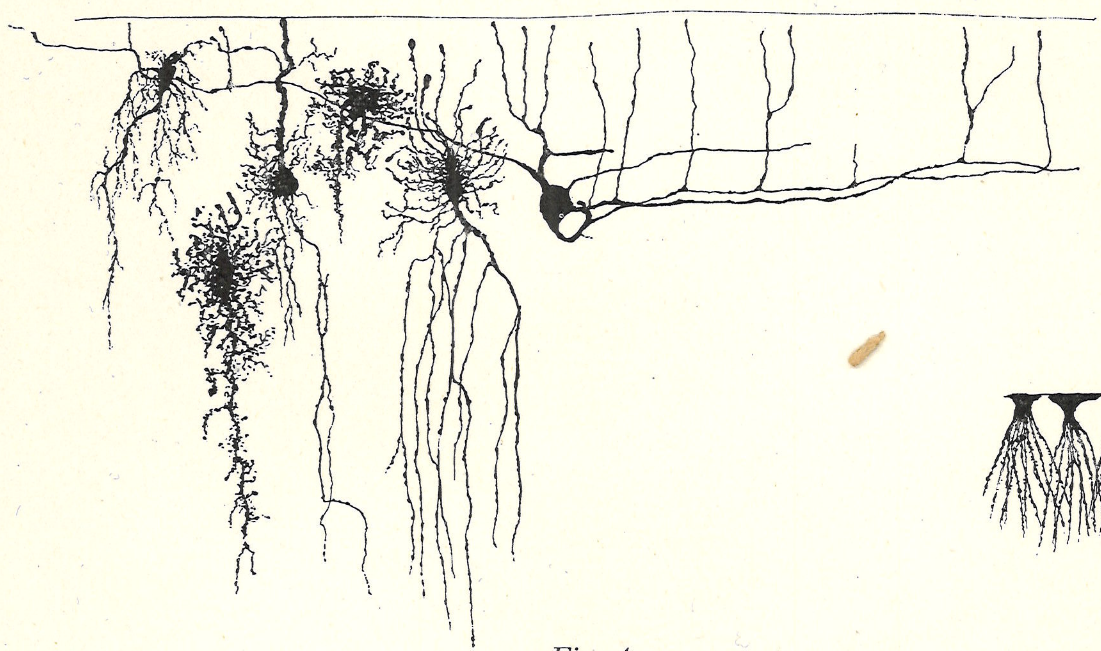

*Fig. 6.*

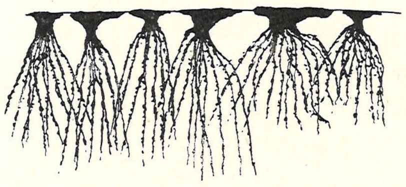

*Fig. 4.*

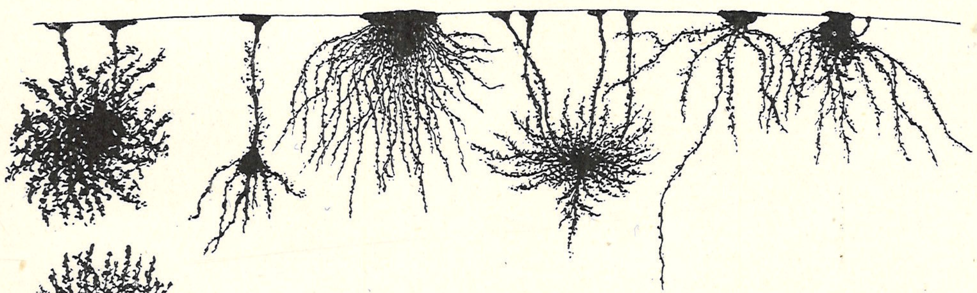

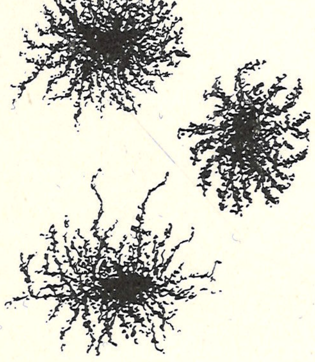

*Fig. 7.*

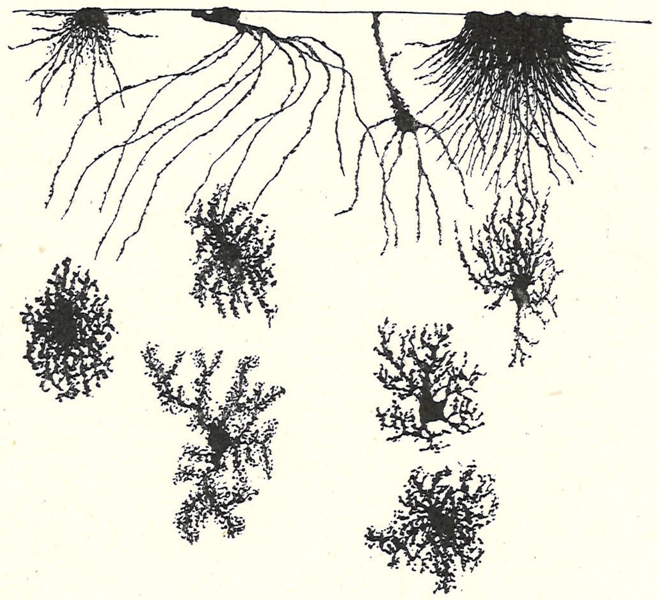

*Fig. 8.*

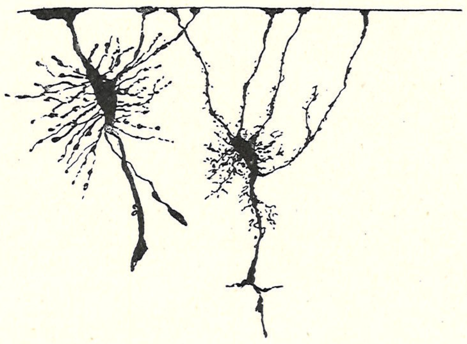

Gez. v. Gustaf Retzius. Typogr. Central-Tryckeriet. Fototypi v. J. Cederquist.

## Tafel IX.

# Die Neuroglia der Grosshirnganglien, der Insula Reilii und der Medulla oblongata des Menschen.

**Fig. 1.** Frontaler Vertikalschnitt der Rindenpartie des *Corpus quadrigeminum anterius* vom *42 Cm. langen menschlichen Foetus.*

**Fig. 2.** Frontaler Vertikalschnitt der Rindenpartie des *Putamen thalami optici* vom *42 Cm. langen menschlichen Foetus.*

**Fig. 3.** Vertikalschnitt der Oberflächenpartie vom *Corpus striatum eines 65-jährigen Mannes.*

**Fig. 4.** Vertikaler Querschnitt der lateralen Oberflächenpartie der *Medulla oblongata* eines $6^1/_2$ *Monate alten menschlichen Foetus.*

**Fig. 5.** Vertikalschnitt der *Insula Reilii* eines 39 Cm. langen menschlichen Foetus. — *bg,* ein Blutgefäss mit dem Ansatze eines Gliazellenfortsatzes.

---

Sämmtliche Figuren der Tafel sind nach Golgi'schen Präparaten bei Vér. Obj. 6 und Ocul. 3 (eingeschob. Tubus) gezeichnet.

*Fig. 1.*

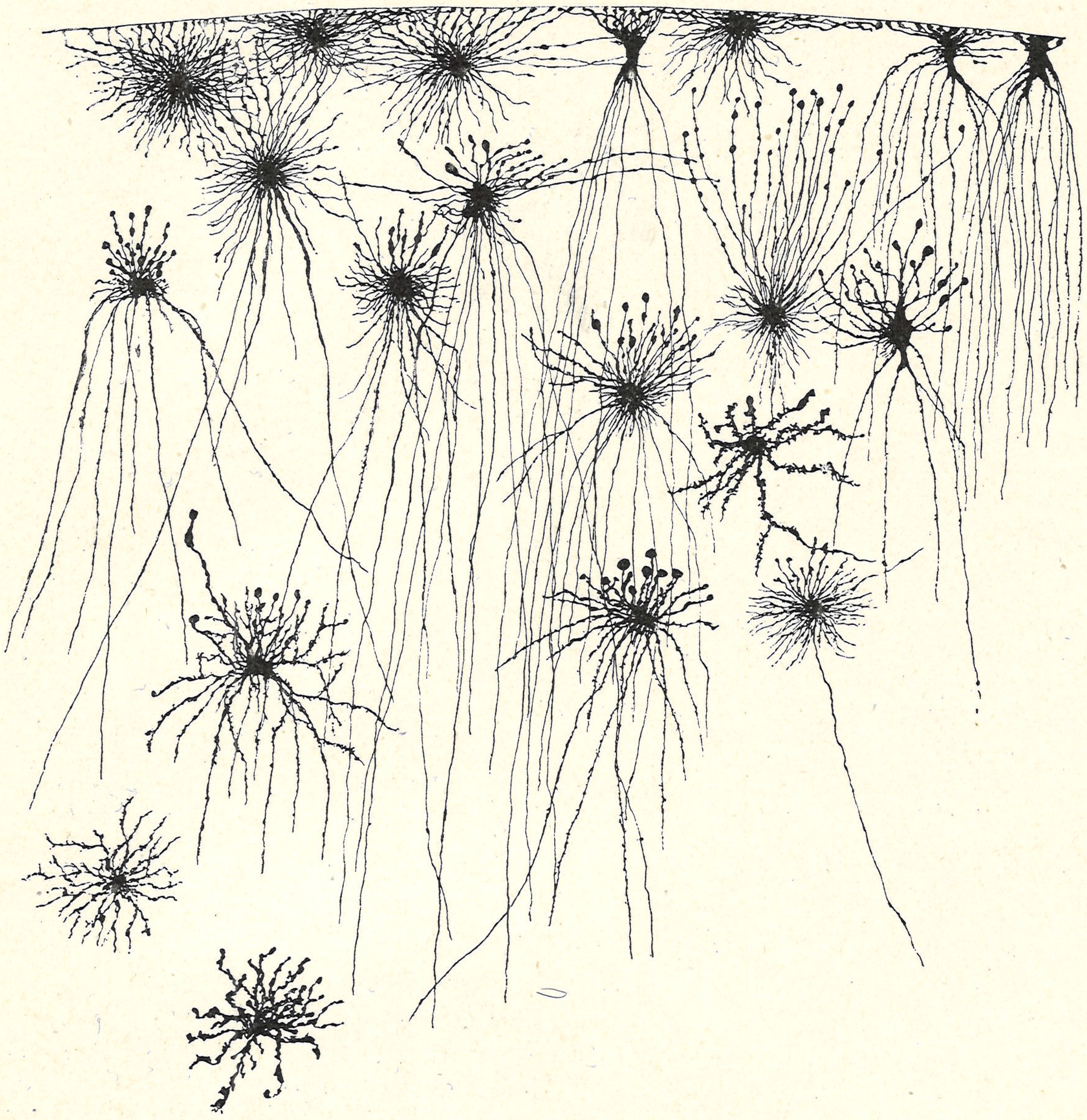

*Fig. 3.*

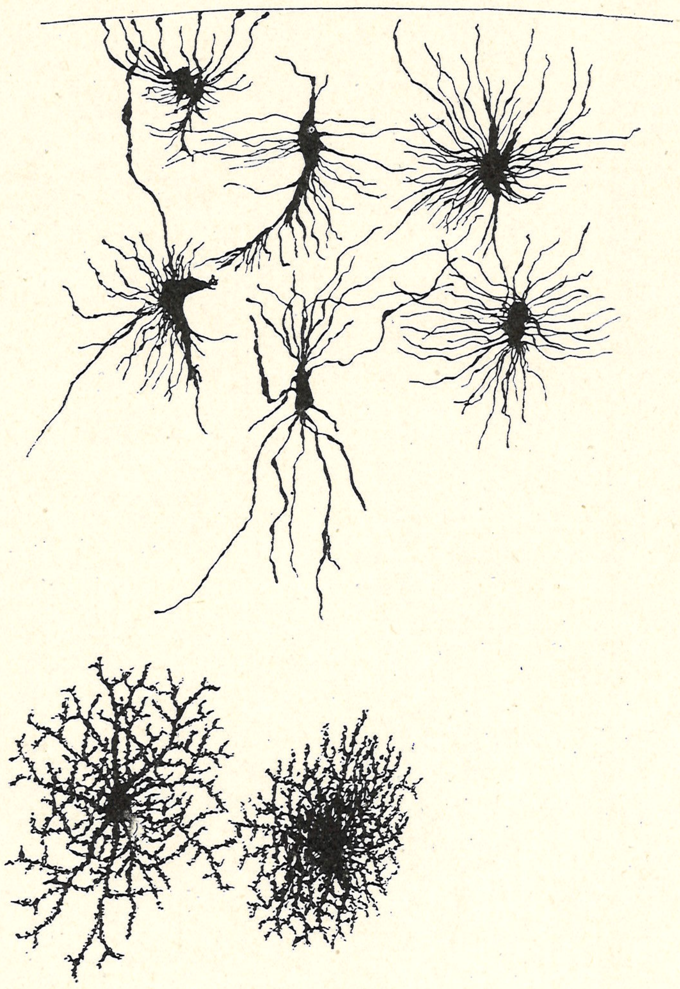

*Fig. 2.*

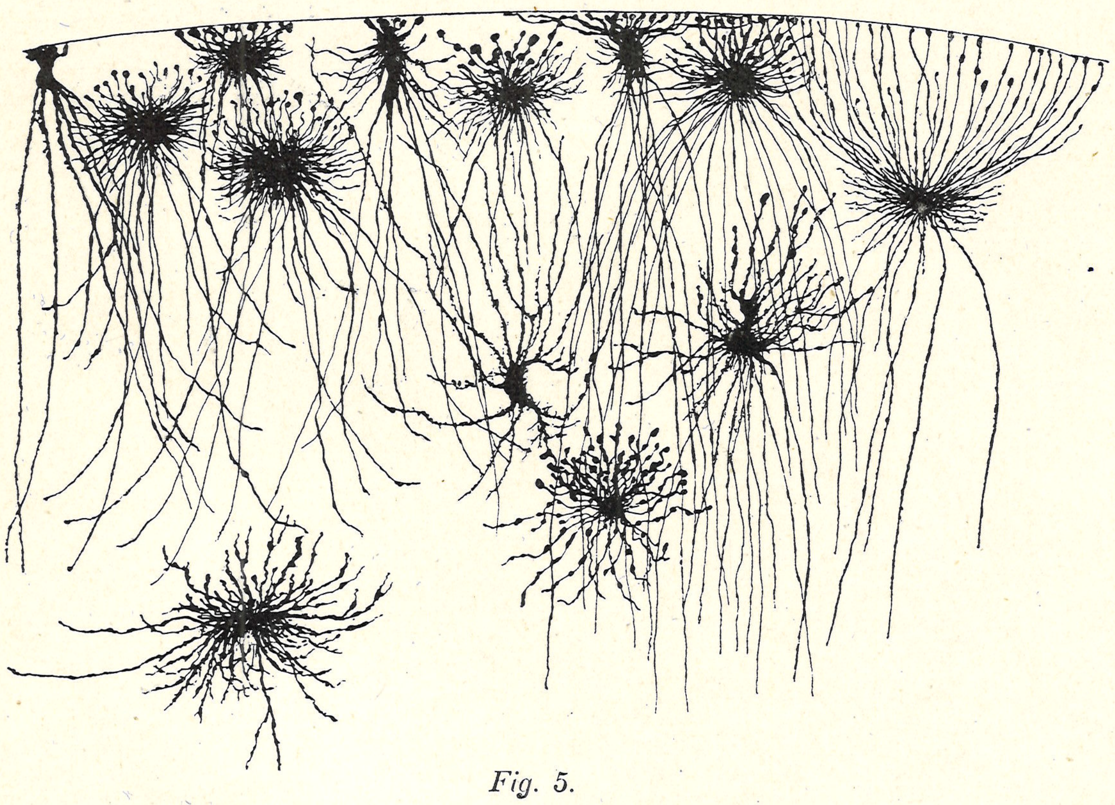

*Fig. 4.*

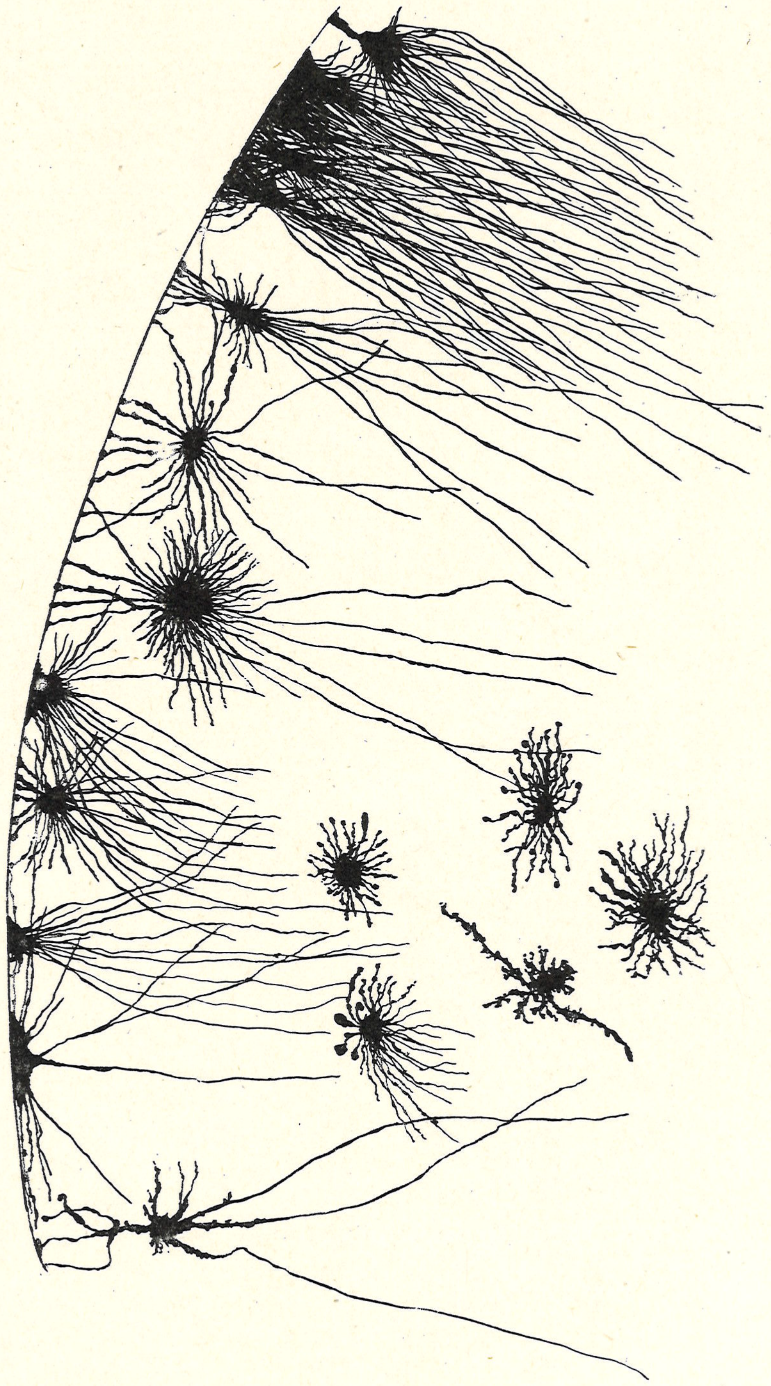

*Fig. 5.*

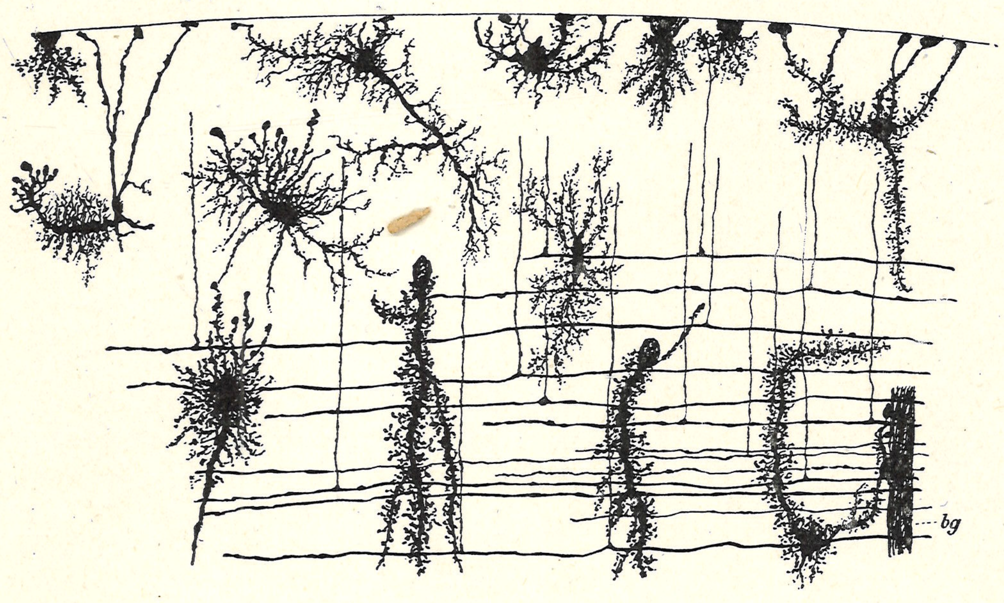


Gez. v. Gustaf Retzius. Typogr. Central-Tryckeriet. Fototypi v. J. Cederquist.

# Tafel X.

## Die Neuroglia der Grosshirnganglien und des Gyrus Hippocampi der Katze, sowie des Oliven des Menschen.

**Fig. 1.** Frontaler Vertikalschnitt des *Corpus quadrigeminum anterius* der *14 Tage alten Katze.*

**Fig. 2.** Frontaler Vertikalschnitt des *Corpus quadrigeminum posterius* der *14 Tage alten Katze.*

**Fig. 3.** Frontaler Vertikalschnitt des *Corpus geniculatum* der *14 Tage alten Katze.*

**Fig. 4.** Vertikalschnitt aus dem unteren Umfang des *Gyrus hippocampi* einer *14 Tage alten Katze.*

**Fig. 5.** Vertikalschnitt aus dem Inneren des *Gyrus hippocampi* einer *14 Tage alten Katze.*

**Fig. 6.** Vertikalschnitt des *Fornix* einer *14 Tage alten Katze.*

**Fig. 7.** Vertikaler Querschnitt der *Olive* eines *8 Monate alten menschlichen Foetus.* Wegen Mangel an Platz auf der Taf. IX wurde diese Figur hier wiedergegeben.

---

Sämmtliche Figuren der Tafel sind nach Golgi'schen Präparaten abgebildet. Die Fig. 1—6 sind bei Vér. Obj. 6 und Ocul. 3 (eingeschob. Tubus), die Fig. 7 bei Vér. Obj. 2 und Ocul. 3 (eingeschob. Tubus) gezeichnet.

*Fig. 1.*

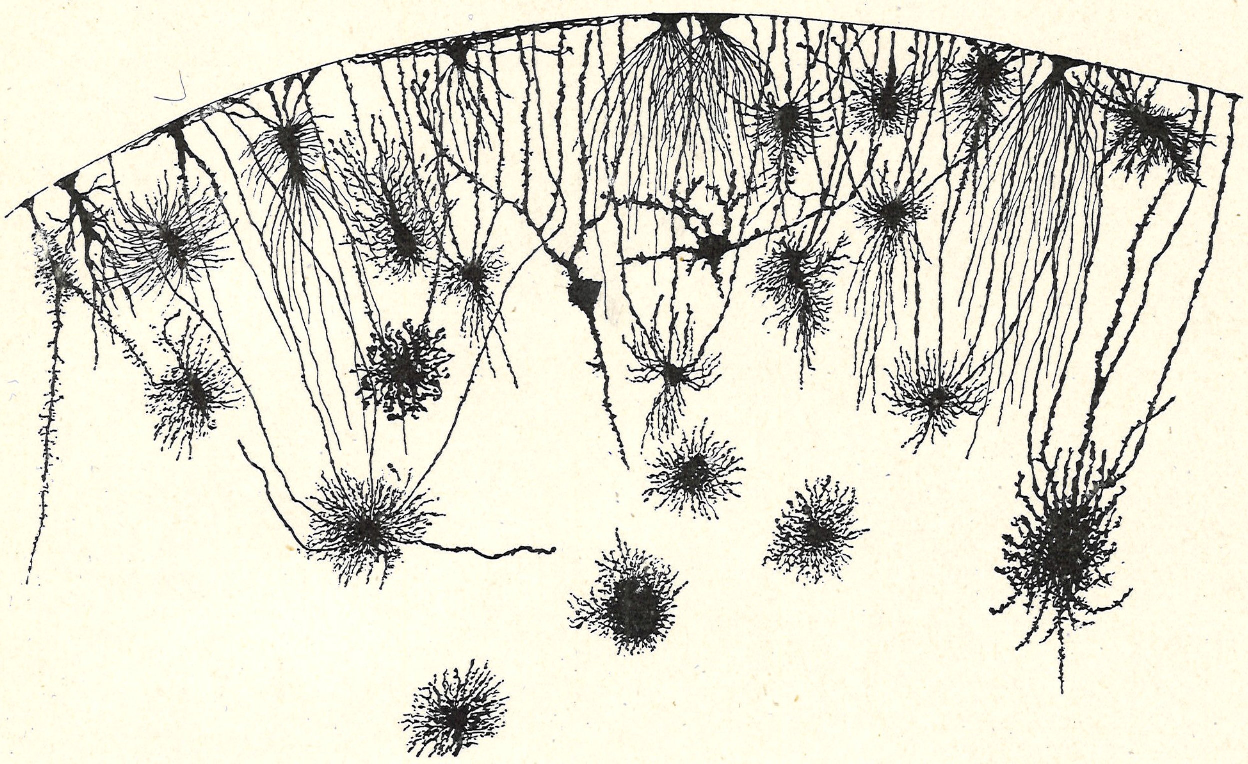

*Fig. 2.*

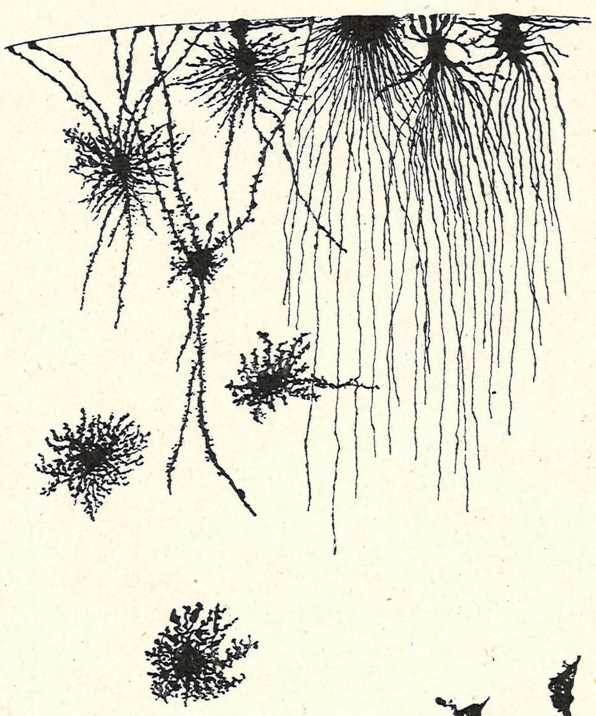

*Fig. 3.*

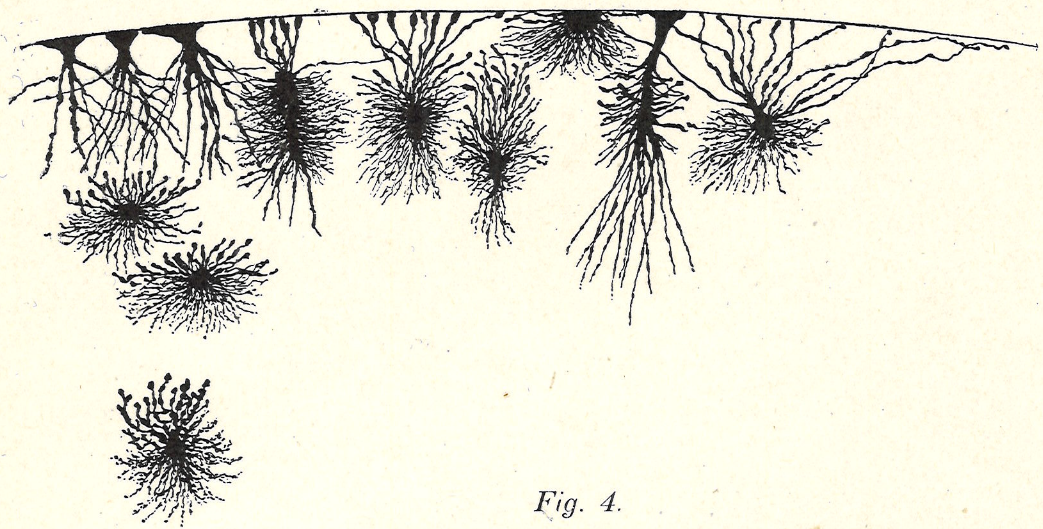

*Fig. 5.*

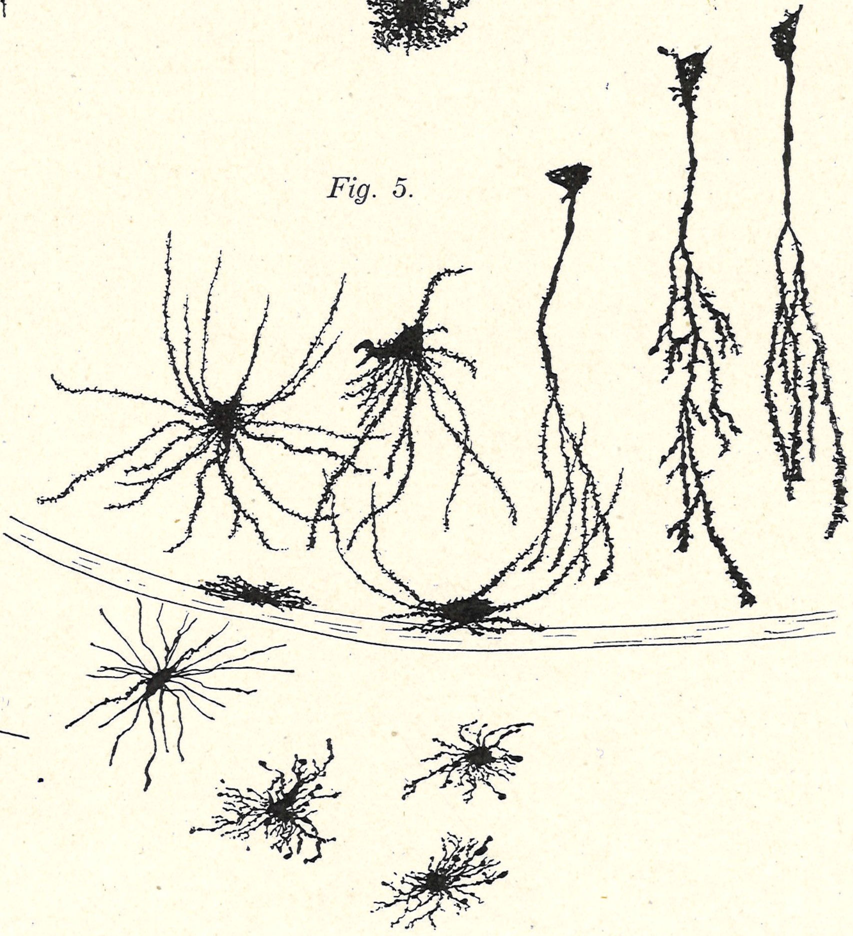

*Fig. 4.*

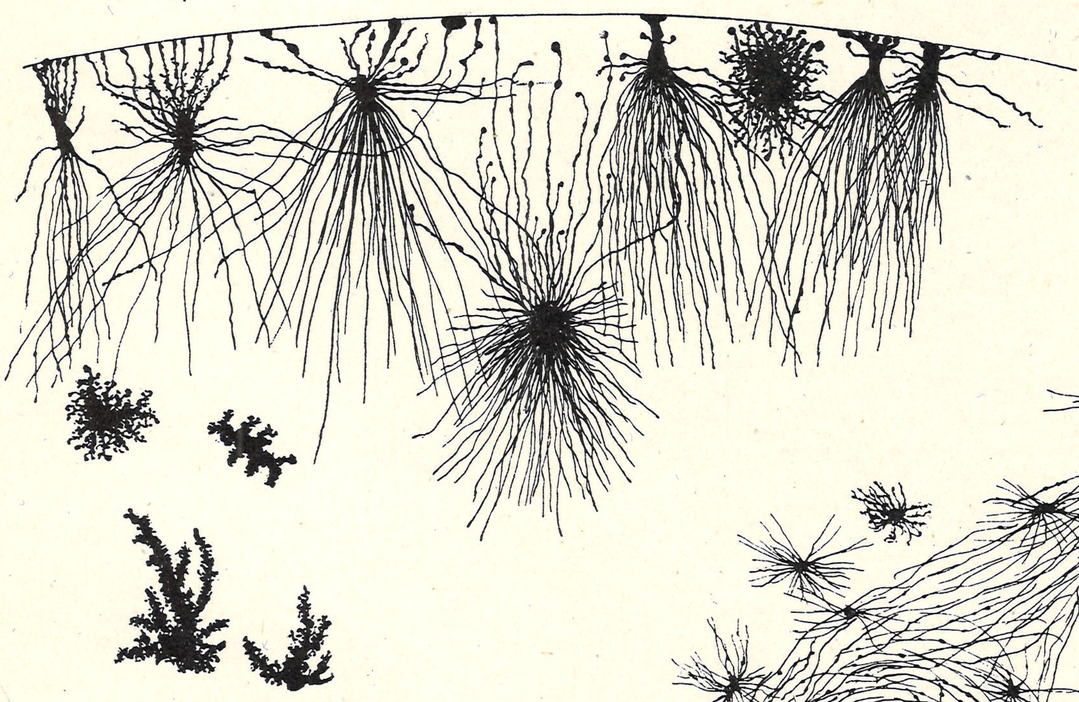

*Fig. 7.*

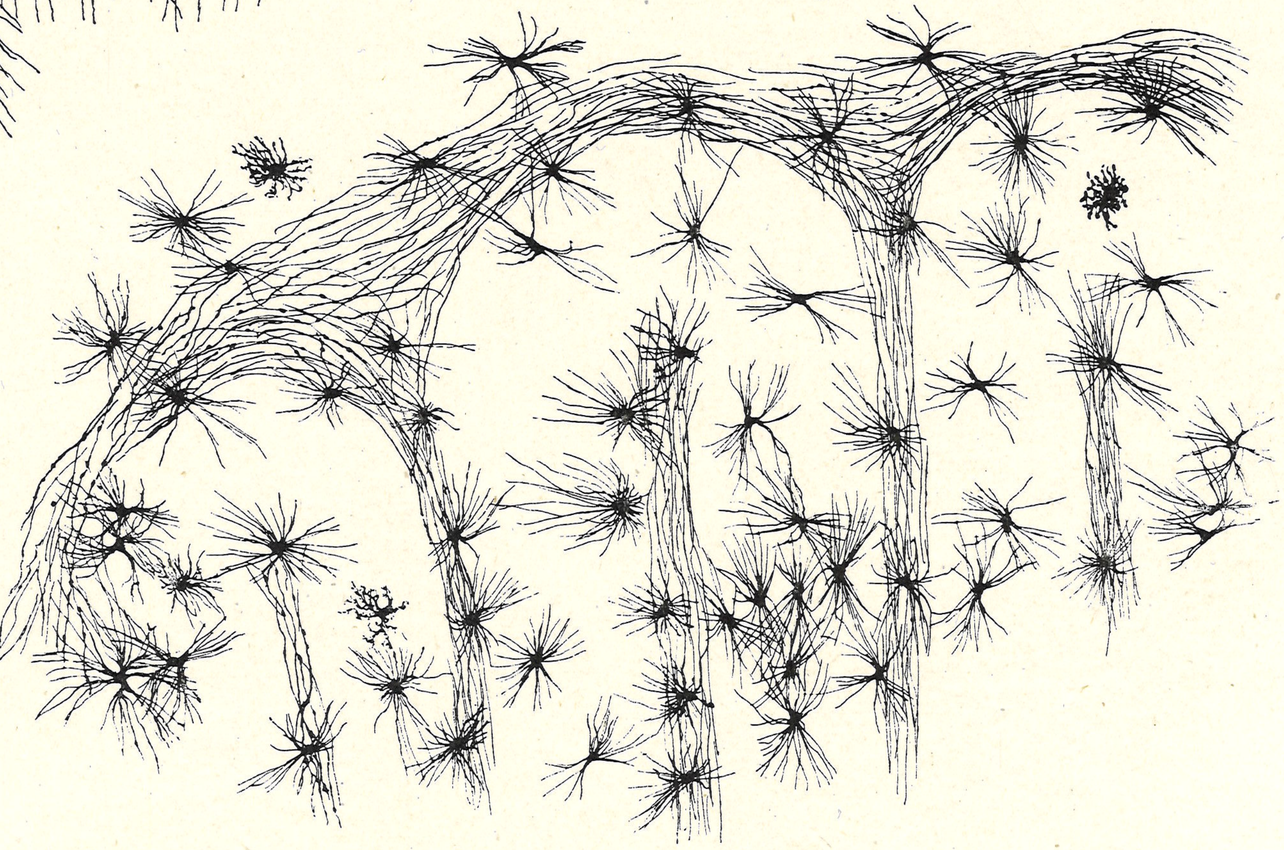

*Fig. 6.*

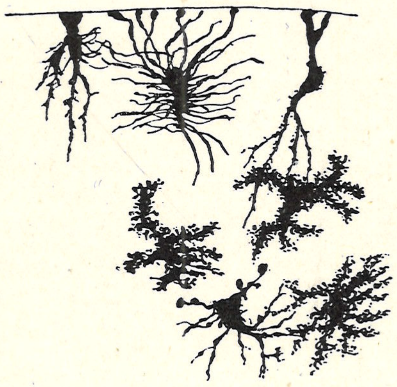

Gez. v. Gustaf Retzius. Typogr. Central-Tryckeriet. Fototypi v. J. Cederquist.

## Tafel XI.

# Die Neuroglia der Kleinhirnrinde des Menschen und der Säugethiere.

**Fig. 1.** Vertikalschnitt der Kleinhirnrinde eines *37 Cm. langen menschlichen Foetus,* mit fünf Bergmann'schen Faserzellen; *ä,* Vignal'sche Zellenschicht.

**Fig. 2.** Vertikalschicht der Kleinhirnrinde eines *45 Cm. langen menschlichen Foetus,* mit sieben Bergmann'schen Faserzellen; — *ä,* Vignal'sche Zellenschicht; — *m,* Korbzellenschicht.

**Fig. 3.** Vertikalschnitt der Kleinhirnrinde eines *48 Cm. langen menschlichen Foetus,* mit sechs Bergmann'schen Faserzellen und zwei Sternstrahlern in frühem Entwicklungsstadium; — *ä,* Vignal'sche Schicht; — *m,* Korbzellenschicht.

**Fig. 4.** Vertikalschnitt der Kleinhirnrinde eines *3 Monate alten Kindes,* mit zwei Bergmann'schen Faserzellen und drei Langsternstrahlern; — *ä,* Vignal'sche Schicht; — *m,* Korbzellenschicht.

**Fig. 5.** Vertikalschnitt der Kleinhirnrinde eines *33-jährigen Mannes,* mit drei Bergmann'schen Faserzellen, zwei Kurzsternstrahlern und zwei Langsternstrahlern.

**Fig. 6.** Vertikalschnitt der Kleinhirnrinde eines noch nicht ausgetragenen *Katzenfoetus,* mit zwei Bergmann'schen Faserzellen und verschiedenen Zellen der Vignal'schen Schicht; nach unten von der letzteren findet sich eine noch unentwickelte Korbzelle und ein Fortsatz einer anderen Zelle derselben Art.

**Fig. 7.** Vertikalschnitt der Kleinhirnrinde einer *3 Tage alten Katze,* mit zwei Bergmann'schen Faserzellen; — *ä,* Vignal'sche Schicht; — *m,* Korbzellenschicht.

**Fig. 8.** Vertikalschnitt der Kleinhirnrinde einer *$5^1/_2$ Tage alten Katze,* mit sechs Bergmann'schen Faserzellen und zwei Kurzsternstrahlern; — *ä,* Vignal'sche Zellenschicht; — *m,* Korbzellenschicht.

**Fig. 9.** Vertikalschnitt der Kleinhirnrinde einer *14 Tage alten Katze,* mit vier Bergmann'schen Faserzellen, fünf Kurzsternstrahlern und einer Zelle von schwer bestimmbarer Bedeutung; — *ä,* Vignal'sche Zellenschicht; — *t,* die tangentialen bipolaren Zellen dieser Schicht; — *m,* Korbzellenschicht mit einer Korbzelle (*k*); — *f,* Markstrahlung.

**Fig. 10.** Partie eines Tangentialschnittes der Kleinhirnrinde einer *14 Tage alten Katze,* mit einer Anzahl tangentialer bipolarer Elemente (untere Abtheilung der Vignal'schen Schicht).

**Fig. 11.** Vertikalschnitt der Kleinhirnrinde einer *22 Tage alten Katze,* mit drei Bergmann'schen Faserzellen und einem Kurzsternstrahler; — *ä,* Vignal'sche Zellenschicht mit eigenthümlichen, tangential verlaufenden Fasern; — *m,* Korbzellenschicht.

**Fig. 12.** Ein Langsternstrahler aus der Markstrahlung der Kleinhirnrinde einer *22 Tage alten Katze.*

---

Sämmtliche Figuren der Tafel sind nach Golgi'schen Präparaten bei Vér. Obj. 6 und Ocul. 3 (eingeschob. Tubus) gezeichnet.

*Fig. 1.*

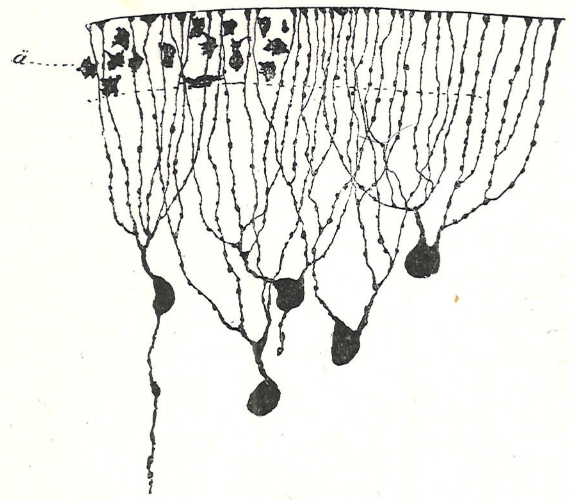

*Fig. 2.*

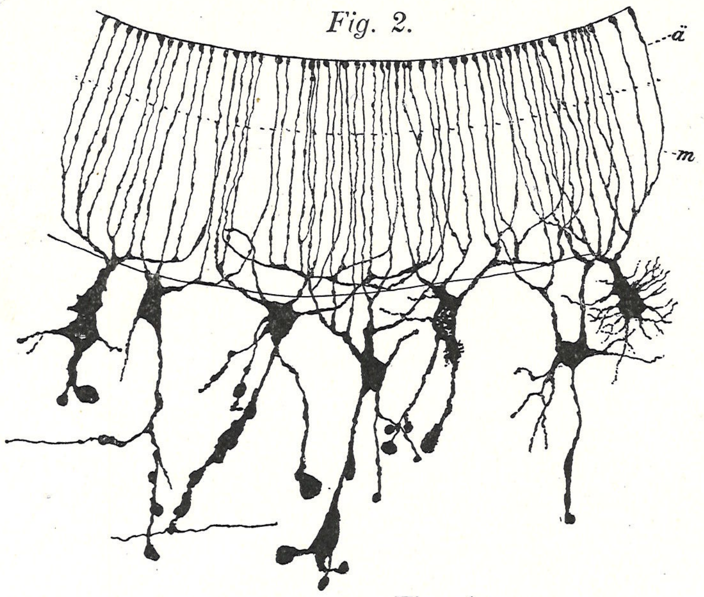

*Fig. 5.*

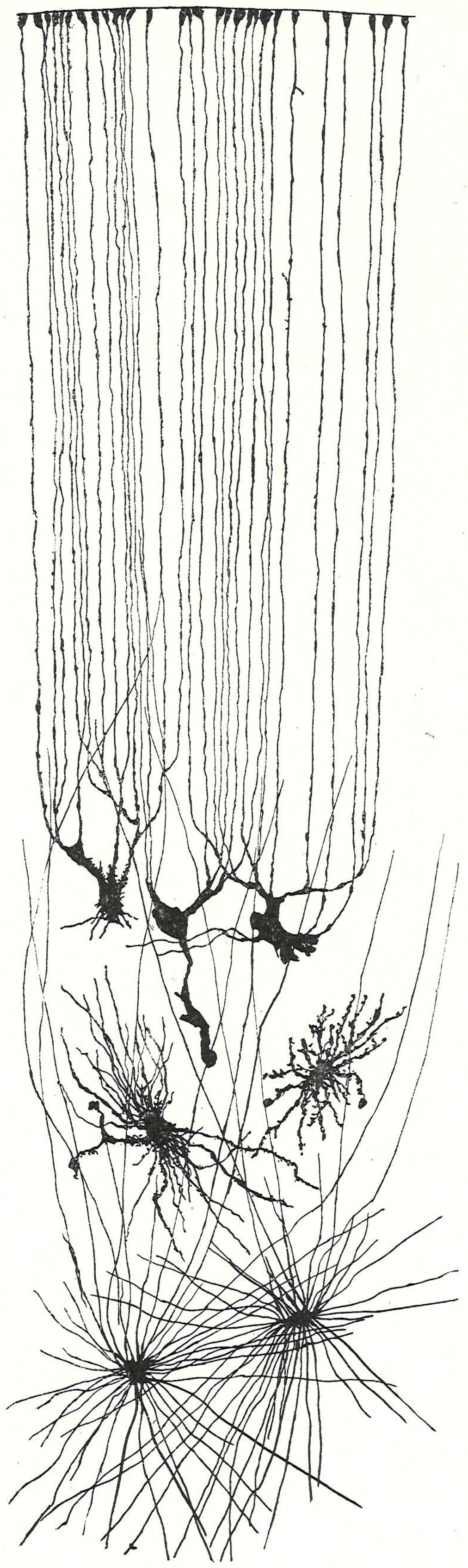

*Fig. 3.*

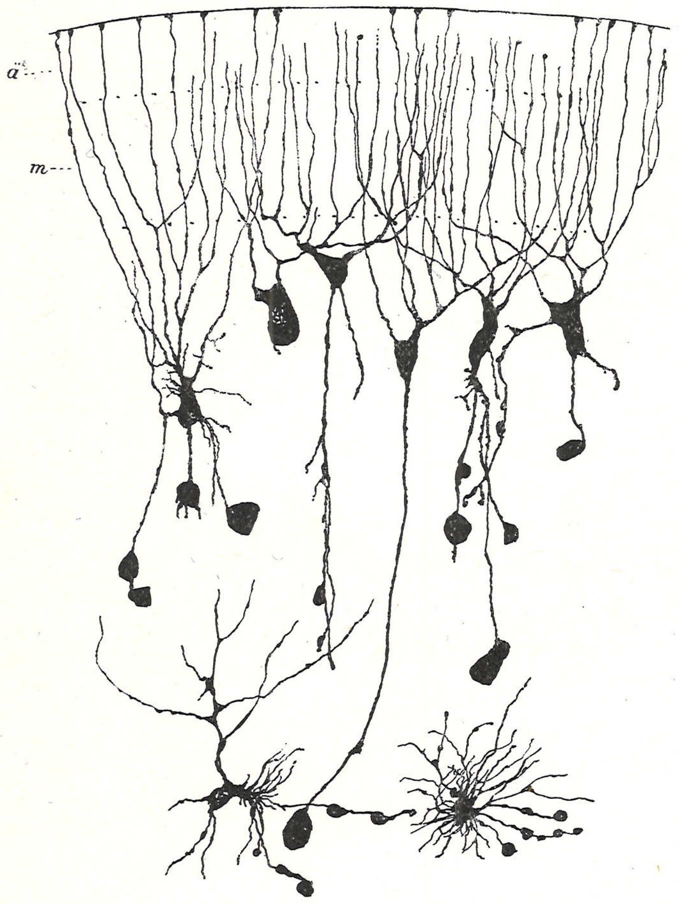

*Fig. 4.*

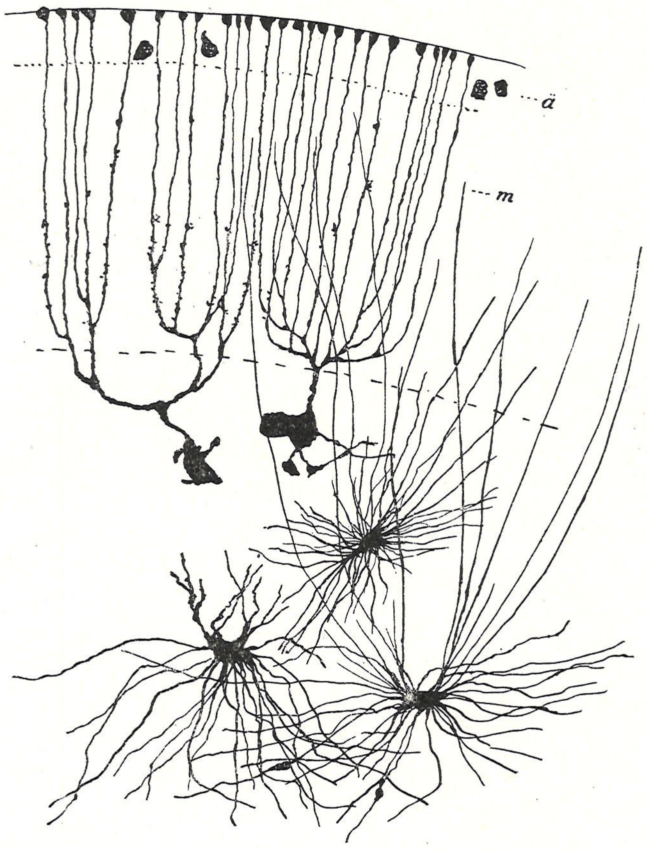

*Fig. 9.*

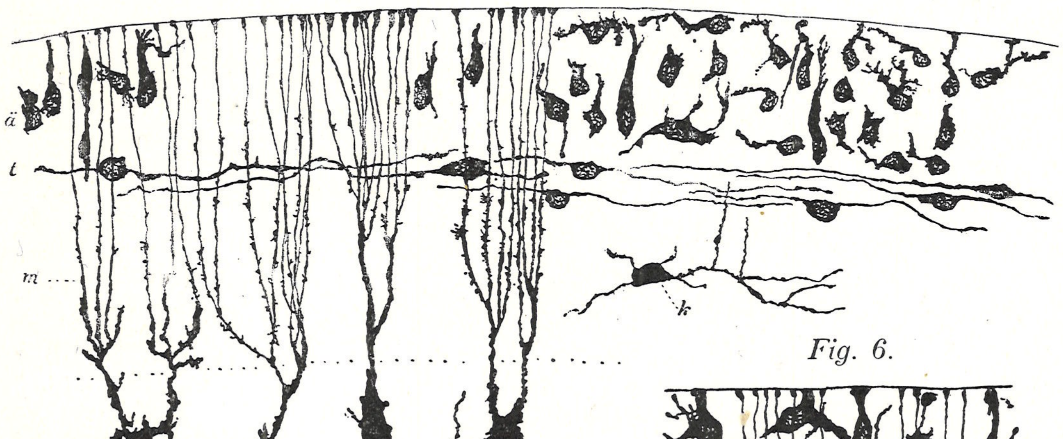

*Fig. 7.*

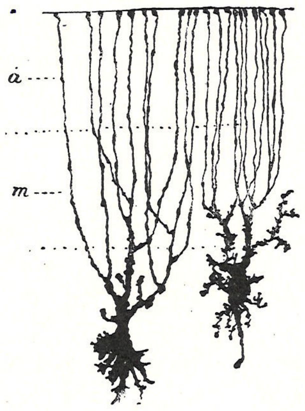

*Fig. 6.*

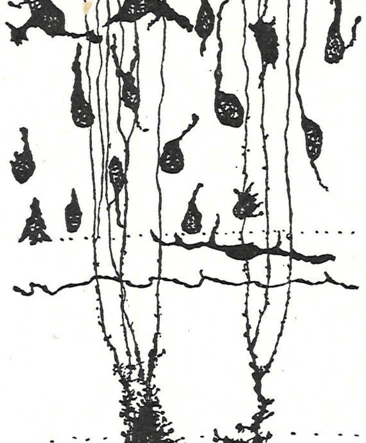

*Fig. 11.*

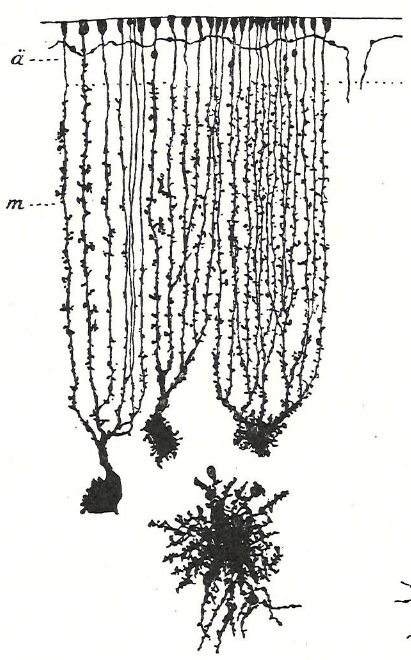

*Fig. 8.*

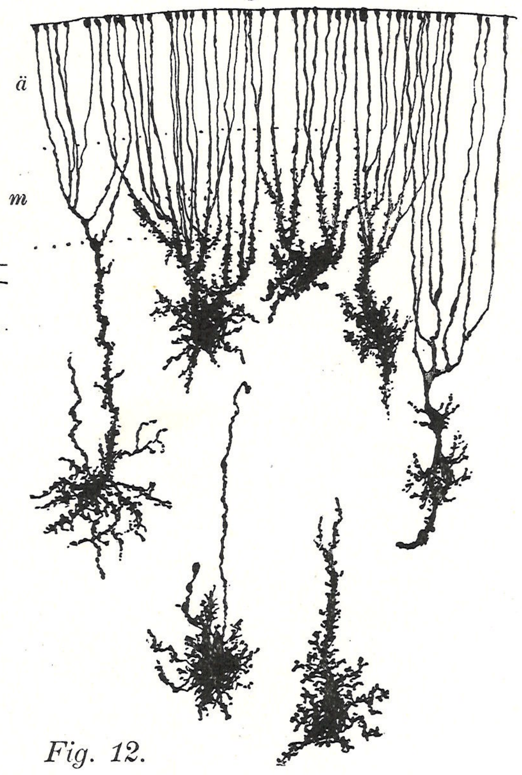

*Fig. 10.*

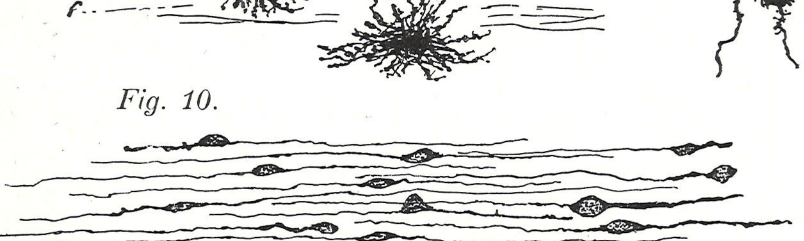

*Fig. 12.*

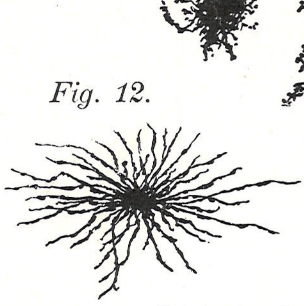

Gez. v. Gustaf Retzius. Typogr. Central-Tryckeriet. Fototypi v. J. Cederquist.

## Tafel XII.

# Die Neuroglia der Neuro-Hypophyse der Säugethiere.

**Fig. 1.** Sagittalschnitt der Neuro-Hypophyse eines *14 Cm. langen Katzenfoetus; — t,* dritter Ventrikel, welcher in den Trichterkanal übergeht; — *nh,* Höhle der Neuro-Hypophyse; — *ep,* Epithelsaum; — *eh,* Höhle der epithelialen Hypophyse; — *e,* epitheliale Hypophyse.

**Fig. 2.** Frontalschnitt der Neuro-Hypophyse eines *jungen Hundes; — t,* der vom dritten Ventrikel sich in die Neuro-Hypophyse hinabsenkende Trichterkanal; — *i,* Ventrikalwand mit Ependym und Neuroglia; nach unten hin knickt sich die Wand beiderseits nach aussen hin ein und bildet eine Falte; — *ep,* Epithelsaum; — *eh,* die nach aussen davon befindliche eng spaltenförmige Höhle der epithelialen Hypophyse (*e*).

**Fig. 2 a.** Mosaikzeichnung des Epithelsaums, von aussen gesehen (junger *Hund*).

**Fig. 3.** Randpartie der Neuro-Hypophyse (Vertikalschnitt) eines *8 Tage alten Kaninchens; — ä,* Epithelsaum mit gefärbten Zellen; — *bg,* Blutgefäss, welches dicht unter dem Epithelsaum verläuft. Verschiedene Neurogliazellen dargestellt.

**Fig. 4.** Randpartie der Neuro-Hypophyse (Vertikalschnitt) eines *2 Monate alten Hundes; — e,* Epithelsaum; — *bg,* Blutgefässe im Längsschnitt und Querschnitt. Neurogliazellen von verschiedener Form wiedergegeben.

**Fig. 5.** Eine Gruppe von Neurogliazellen aus dem Inneren der Neuro-Hypophyse eines *2 Monate alten Hundes.*

**Fig. 6.** Eine Gruppe von Neurogliazellen aus dem Inneren der Neuro-Hypophyse des *1-jährigen Hundes; — bg,* Querschnitt eines Blutgefässes.

**Fig. 7.** Neurogliazellen und Fasern aus dem Inneren der Neuro-Hypophyse eines *1-jährigen Hundes.*

**Fig. 8.** Drei Neurogliazellen aus dem Trichterhalse in der Nähe der Neuro-Hypophyse eines *17-jährigen Mannes.*

**Fig. 9.** Zwei Neurogliazellen und mehrere Fasern aus der Neuro-Hypophyse (in der Nähe des Halstheiles) eines *2 Monate alten Kindes; — bg,* Blutgefäss, an welches sich mehrere Fortsätze der einen Zelle ansetzen.

---

Sämmtliche Figuren sind nach Golgi'schen Präparaten wiedergegeben. Die Fig. 1 ist bei Vér. Obj. 2 und Ocul. 1 (eingeschob. Tubus), die Fig. 2 bei Vér. Obj. 2 (halb abgeschraubt) und Ocul. 1 (eingeschob. Tubus), die übrigen Figuren sind bei Vér. Obj. 6 und Ocul. 3 (eingeschob. Tubus) gezeichnet.

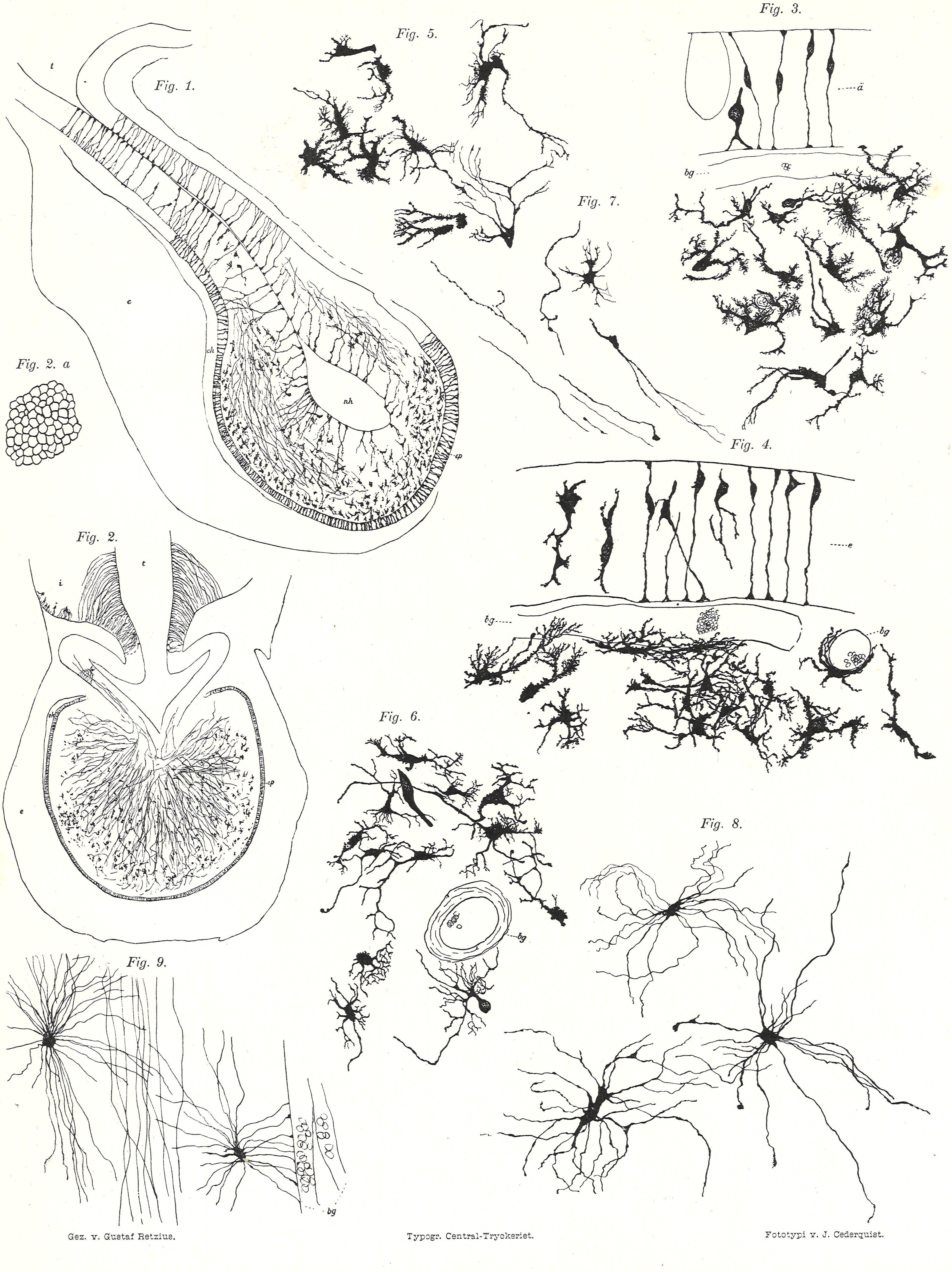


Gez. v. Gustaf Retzius. Typogr. Central-Tryckeriet. Fototypi v. J. Cederquist.

## Tafel XIII.

# Die Neuroglia des Sehnerven und der Netzhaut.

**Fig. 1.** Randpartie eines Sehnerven von einem *28 Cm. langen menschlichen Embryo;* — *o,* Pialscheide des Sehnerven.

**Fig. 2.** Randpartie mit der Pialscheide (*o*) des Sehnerven von einem *45 Cm. langen menschlichen Foetus;* — *bg,* Balken mit quergeschnittenen Blutgefässen.

**Fig. 3.** Partie vom Inneren eines Querschnitts des Sehnerven mit quergeschnittenen Nervenfaserbündeln (*nb*), Blutgefässen (*bg*) und drei Neurogliazellen. Von einem *48 Cm. langen menschlichen Foetus.*

**Fig. 4.** Randpartie mit der Pialscheide (*o*) von einem Querschnitte des Sehnerven eines *14 Cm. langen Katzenfoetus.*

**Fig. 5.** Randpartie mit der Pialscheide (*o*) von einem Querschnitte des Sehnerven einer *5 Tage alten Katze.*

**Fig. 6.** Randpartie mit der Pialscheide (*o*) von einem Längsschnitt des Sehnerven einer *5 Tage alten Katze.*

**Fig. 7.** Randpartie mit der Pialscheide (*o*) von einem Querschnitt des Sehnerven einer *14 Tage alten Katze.*

**Fig. 8.** Randpartie mit der Pialscheide (*o*) von einem Querschnitt des Sehnerven eines *1 Monat alten Hundes.*

**Fig. 9.** Randpartie mit der fibrösen Begrenzung (*o*) von einem Querschnitt des Sehnerven in der Gegend der Lamina cribrosa eines *neugeborenen Hundes.*

**Fig. 10.** Randpartie des Sehnerveneintritts in das Auge, im Längsschnitt. Von einem *28 Cm. langen menschlichen Embryo;* eine Anzahl gestreckter, gegen den Retinawinkel (*re*) senkrecht gerichteter Neurogliazellen sind dargestellt.

**Fig. 11.** Vertikalschnitt der Retina eines *$4^1/_2$ Tage alten Hühnerembryos.* Die innere Fläche (Limitans interna) ist nach der unteren Seite der Tafel gerichtet.

**Fig. 12—14.** Müller'sche Stützfasern in embryonalem Stadium. — *Fig. 12,* von dem *neugeborenen Mus decumanus;* — *Fig. 13,* von einem *4 Tage alten Mus musculus,* aus der Gegend der Ora serrata; — *Fig. 14* von einem *4 Tage alten Mus musculus,* aus der Umgebung der Papilla optici; *sp,* eine amacrine Zelle der inneren Körnerschicht.

**Fig. 15.** Partie eines Vertikalschnitts der Retina einer *1 Monat alten Katze.* — *r*, $r^1$, Müller'sche Stützfasern; — *m,* innere reticulirte und $m^1$ äussere reticulirte Schicht. Es sind auch eine Ganglienzelle, amacrine Zellen und Stäbchenelemente dargestellt.

**Fig. 16.** Partie eines Vertikalschnitts der Retina eines *6 Wochen alten Kaninchens.* Zwei Müller'sche Fasern und drei amacrine Zellen sind wiedergegeben. — *st,* Stäbchenschicht; — $m^1$, äussere reticulirte Schicht.

**Fig. 17.** Vertikalschnitt der Retina eines *28 Cm. langen menschlichen Embryos.* — *r,* Müller'sche Fasern aus der vorderen, peripherischen Zone; — $r^1$, Müllersche Fasern aus der hinteren Zone; — *k,* innere Körnerschicht; — *sp,* amacrine Zellen verschiedener Art. Es sind auch mehrere Stäbchen, Zapfen und Ganglienzellen wiedergegeben. Aus mehreren Präparaten zusammengestellt.

---

Sämmtliche Figuren der Tafel sind nach Golgi'schen Präparaten und bei Vér. Obj. 6 und Ocul. 3 (eingeschob. Tubus) gezeichnet. Nur die Fig. 17 ist bei ausgezogenem Tubus wiedergegeben.

1

*Fig. 1.*

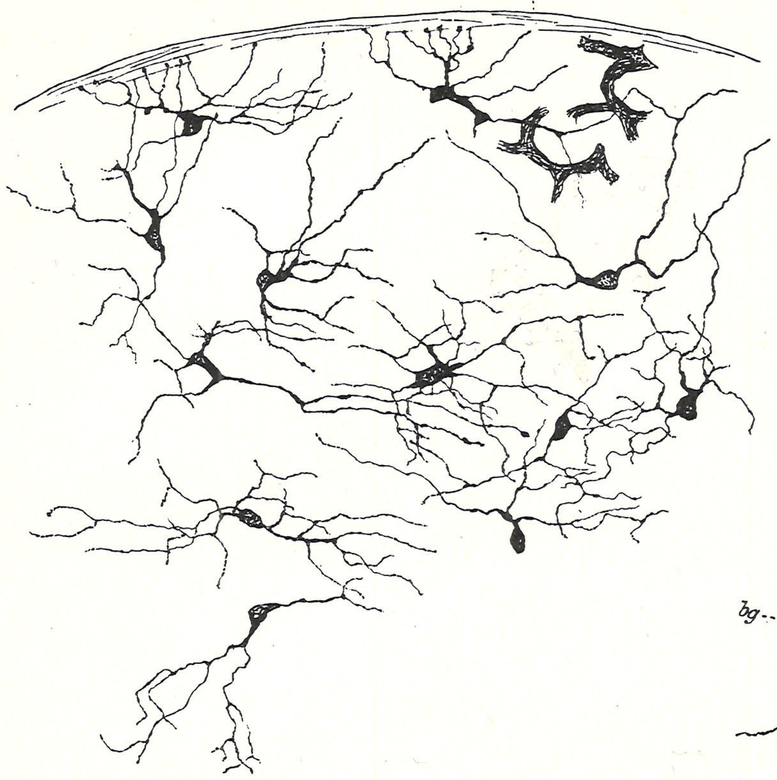


*Fig. 2.*

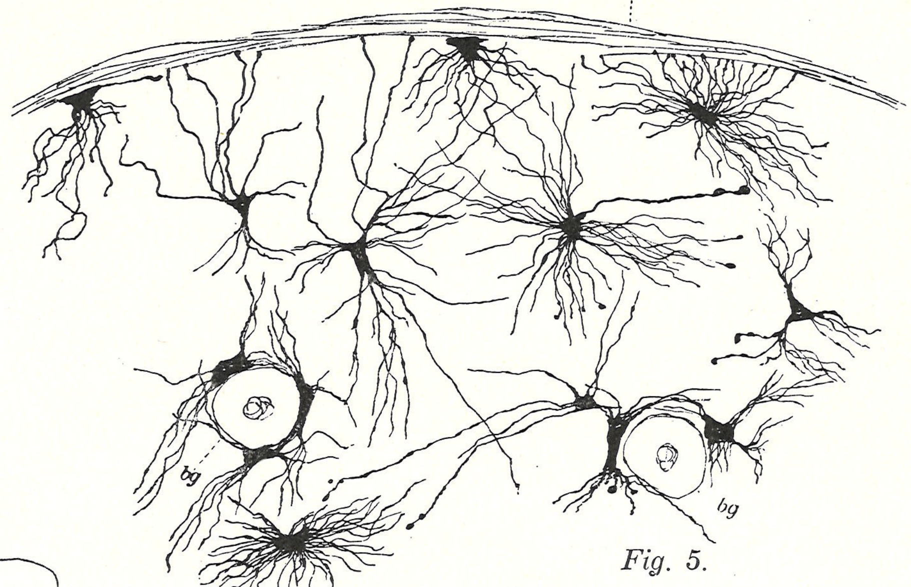


*Fig. 3.*

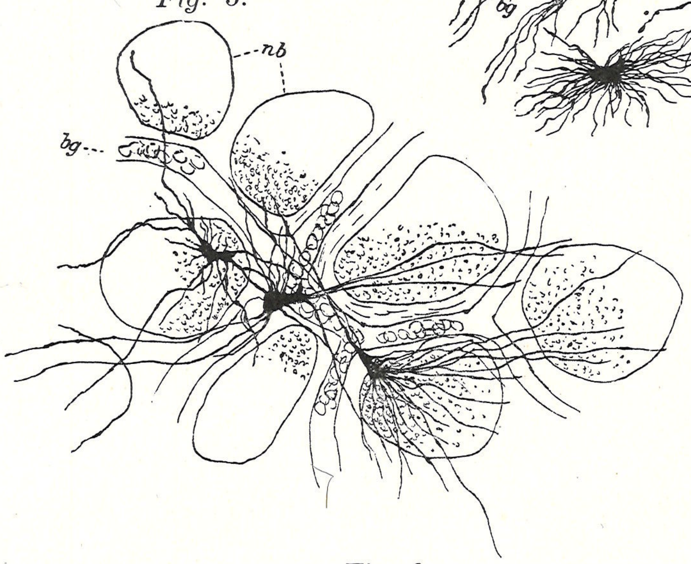


*Fig. 5.*

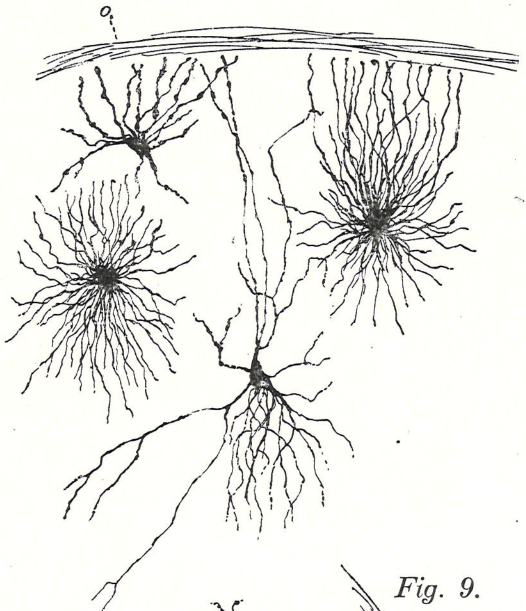


*Fig. 4.*

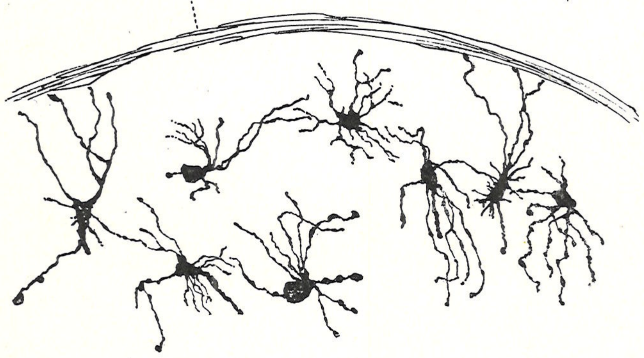


*Fig. 6.*

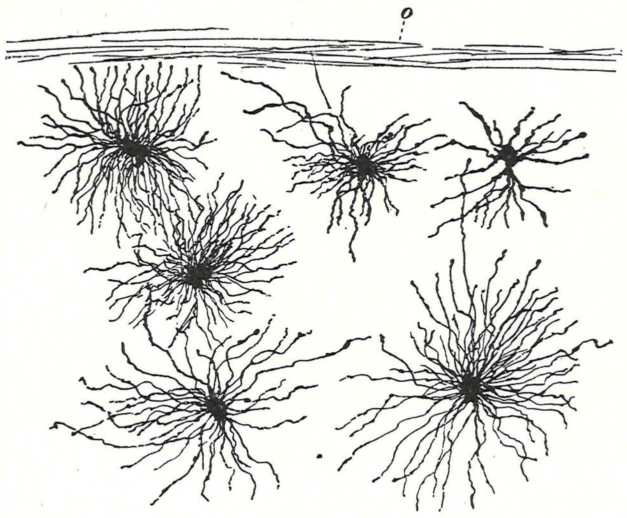


*Fig. 9.*

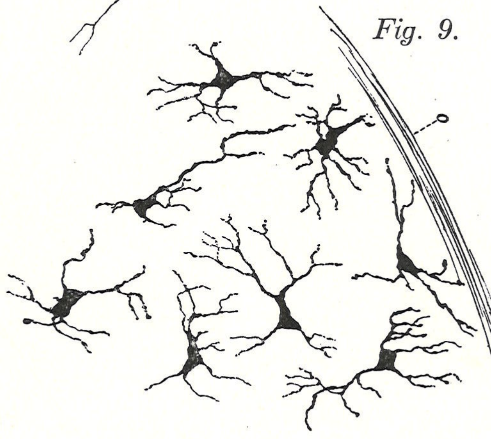


*Fig. 7.*

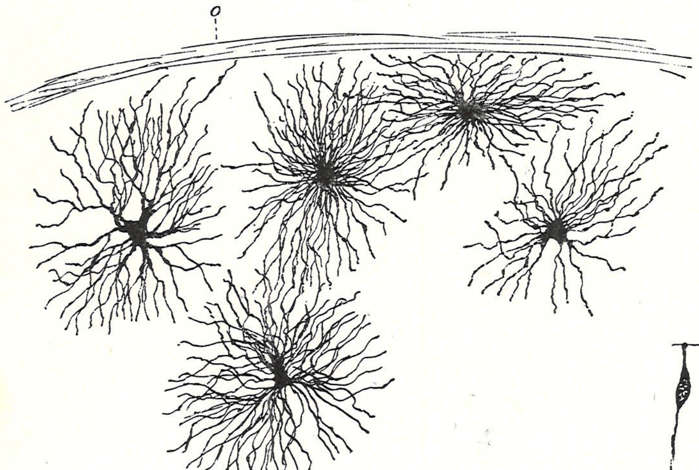


*Fig. 11.*

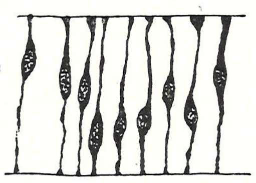

*Fig. 16.*

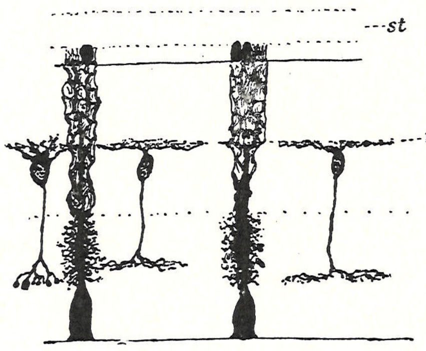


*Fig. 10.*

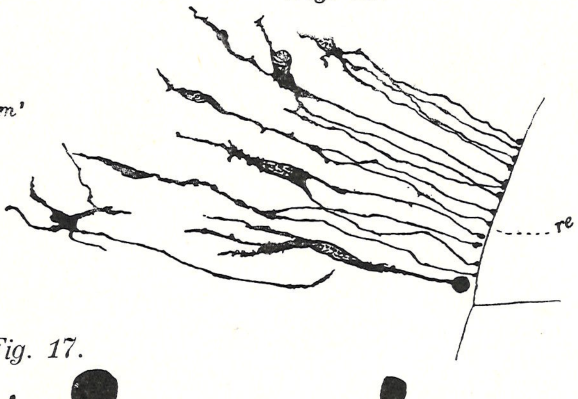


*Fig. 8.*

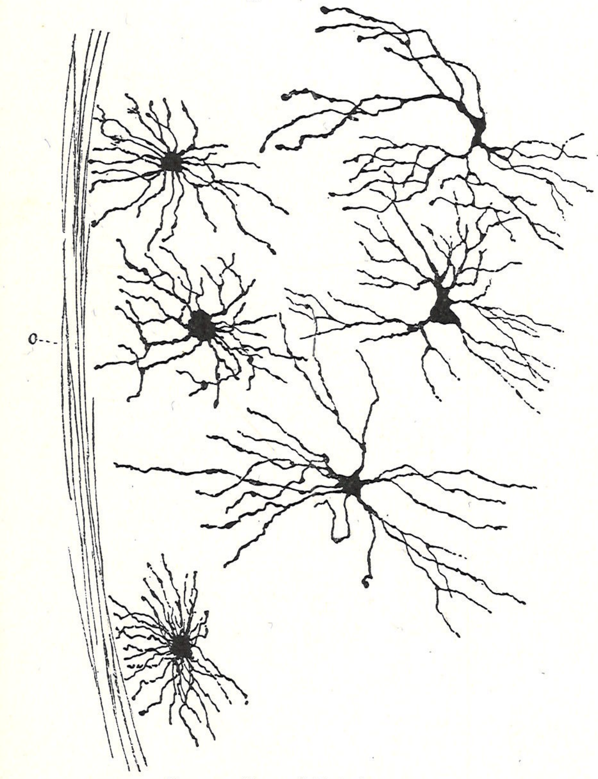


*Fig. 14.*

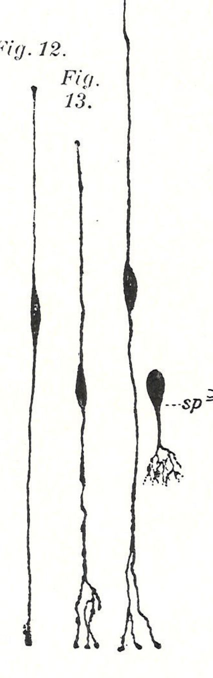


*Fig. 15.*

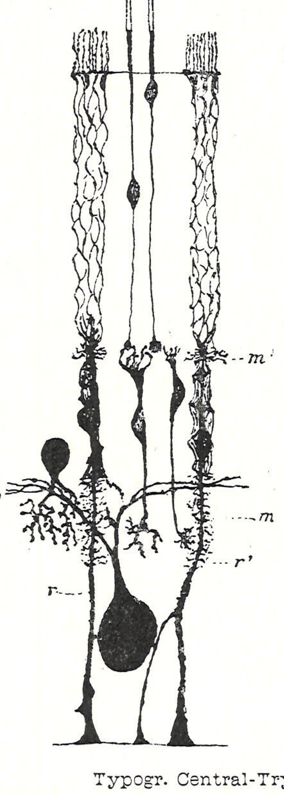


*Fig. 17.*

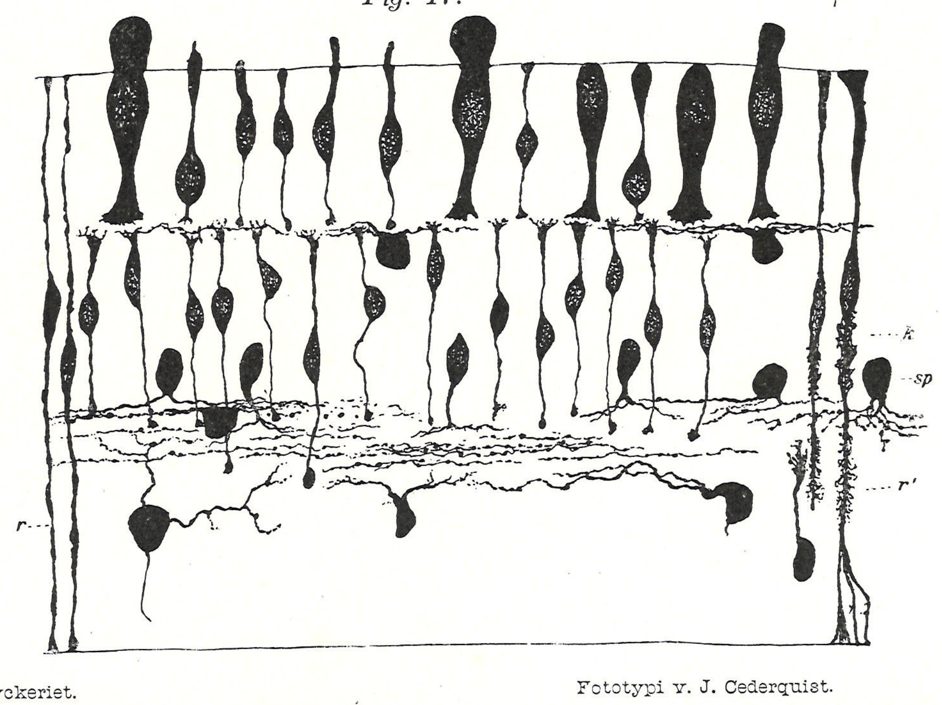

Supplement: Supplementary file 3 — Data S3. Scanned original publication by Retzius, Volume VI. [file GLIA-73-890-s004.pdf]
