# Supplementary material for: The Concept of Neuroglia ‐ the State of the Art Circa 1900
Source: Glia. 2025 Feb 4;73(5):890–904. doi: 10.1002/glia.24678 (PMC11920685; doi:10.1002/glia.24678)
Supplement: Supplementary file 4 — Data S4. Translated text by Rezius, Volume VI with single figures inserted into the text. [file GLIA-73-890-s009.pdf]

Biological Investigations

by

Prof. Gustaf Retzius

Neuroglia of the brain in humans and mammals

Tables I- XIII

Contents

1. The neuroglia of the human and mammalian brain

I. The neuroglia of the hemispheres and the ganglia of the cerebral cortex Tables I – X

A. The neuroglia of the cerebral human cortex Tables I – V

B. The neuroglia of the cerebral cortex of dog, cat and rabbit Tables VI – VIII

C. The neuroglia of the human brain ganglia, medulla oblongata and insula Reilii Table IX

D. The neuroglia of the cat brain ganglia, gyrus hippocampi and fornix Tables X, Figs. 1-6.

II. The neuroglia of the cerebellum Table XI

A. The neuroglia of the human cerebellum Table XI

B. The neuroglia of the cat cerebellum Table XI, Figs. 6-8, 10-12

III. The neuroglia of the mammalian posterior pituitary Table XII

IV. The neuroglia of nervus opticus and retina of human and mammals Table XIII

The neuroglia of the human and mammalian brain

During my continuous investigations on the structure of the central nervous organs using the Golgi chromium-osmium-silver method, I often had the opportunity to obtain clear stainings of ependymal and glial cells of different animals and humans. In the year 1891, I published a short note[1], and last year, I published a more extensive paper with a series of figures on the ependyma and the neuroglia of the spinal cord of different classes of vertebrates (including humans).[2] In the present publication, I intend to continue the presentation of the respective issue. In order not to be too excessive, I will restrict myself to the actual neuroglia of humans and some mammals. The ependymal cells and their processes will only be mentioned in passing. In particular, I will focus on the human neuroglia in the brain, using the mammalian neuroglia only as a comparison. Since I will focus on results based on the Golgi method, I will not present the entire history of neuroglial research but will only mention the work and results of those researchers who have used the same method. I will not discuss the old controversy regarding the molecular, net-type fibrillary, epithelioid, or connective tissue nature of the neuroglia, as this has already been reported in several historical accounts and discussions. In this respect, I will point in particular to the summaries by Golgi[3] and Gierke.[4]

In the intensive and very detailed studies on neuroglia by the latter researcher, he has reported on the statements and views of Deiters, to whom we owe the actual discovery of the typical form of neuroglial cells, and of Jastrowitz, Boll, and Ranvier, who provided additional results. By Weigert's important methods, our knowledge of the distribution, variety, and form of these elements has been significantly expanded. Many years ago, Golgi invented a method that produces more concrete images of the forms of neuroglial cells, and this famous Italian researcher laid the foundation for our knowledge of this subject and, even beyond, for the modern histology of the nervous system. It is therefore a pleasant event that his corresponding collective reports, which had been published in less accessible Italian medical journals, are now available in a monumental German edition.[5]

[1] Gustaf Retzius, Ueber den Bau der Oberflächenschicht der Grosshirnrinde beim Menschen und bei Säugetieren. Verhandl. D. Biolog. Vereins in Stockholm. Bd 3, 1891.

[2] Gustaf Retzius, Studien über Ependym und Neuroglia. Biolog. Untersuchungen von Gustav Retzius. N. F. Bd V, 2, 1883.

[3] Camillo Golgi, Contribuzione alla fina anatomia degli organi centrali des Sistema nervosa, Rivista clinica di Bologna. Ser. 2 A, 1, fasc. 11, novembre 1871 and A., 2, fasc. 12, dic. 1871.

[4] Hans Gierke, Die Stützsubstanz des Centralnervensystems, Archiv f. mikrosk. Anatomie, Bd 25, 1885 and Bd 26, 1896.

[5] Camillo Golgi, Untersuchungen über den feineren Bau des centralen und peripherischen Nervensystems. Aus dem Italienischen übersetzt von Dr. R. Teutscher. Verlag von Gustav Fischer. Jena, 1894.

With respect to neuroglia, after Deiters, it was Golgi who obtained and described the true concept of neuroglial cells due to his profound investigations. This is particularly true for the neuroglia of the brain, as he described and illustrated these cells as formations with numerous, delicate, and long processes in the grey and white matter, in the cortex and the spinal cord. From a small cell body, these processes extend in different directions. Additionally, in the outer cortex of the human cerebrum, he found masses of these cells and illustrated them in his tables. In the cortex of the cerebellum, he discovered the cells of the Bergmann fibers and illustrated the radial neuroglial cells in the granule cell layer and in the cord.

In his extensive summarizing publication from 1885[1], Golgi states that one can, after maceration of the tissue in bichromium solution, recognize the radial neuroglial cells of the cerebral cortex and white matter in isolation. Yet, for a clear demonstration of the peculiarities in form, relationship, distribution, and other features of the connective tissue elements (neuroglial cells), one must apply a combined treatment of bichromium with silver nitrate. In such preparations of the grey and white matter, one recognizes at first glance:

1. The existence of a consistent layer of neuroglial elements at the surface of the gyrus. This layer is thick at the top of the gyrus and thin on the lateral surface between two gyri, where it sometimes consists of only one cell layer. The processes of these cells run partially horizontal, forming a complex net that delimits the surface; they also partially penetrate vertically into the cortical tissue, generating a system of radial fibers reminiscent of the more obvious and regular structure in the cerebellar gyrus.
2. That the protoplasmic processes of pyramidal nerve cells branching into the periphery are lost in this layer where they come into contact with neuroglial cells.
3. That the neuroglial cells form multiple connections with the vessel walls through partially broad, partially thread-like processes. One often observes that blood vessels are surrounded over large distances by a continuous row of glial cells; these cells are closely attached to the vessel walls and project their processes in all directions, many of which connect to other vessels.

Some other Italian researchers, most of whom were students of Golgi using his methods, have discussed the neuroglia in several publications covering the structure of the central organs. Moreno[2] illustrated the radial neuroglial cells in several regions of the brain (e.g., vallecula sylvii, capsula externa, insula Reilii). Magin[3] described multiple pearl-string-shaped varicosities along the long processes of the radial neuroglial cells in the fetal cerebral cortex. Martinotti[4] provided an illustration of the neuroglial cells of the cerebral cortex, featuring some interesting forms. In the layer beneath the pia, he noted cells reminiscent of those in the cerebellum, which are flattened against the free surface and penetrate

with long, thread-like processes, rarely branched, into the grey matter, sometimes up to two-thirds of its thickness. Beyond that, he observed others of highly diverse forms. In dogs, he described cells sending strong, unbranched processes to the periphery, terminating under the pia with triangular enlargements; these insert between the processes of the dish-shaped cells. From the same cells, much thinner processes protrude in various directions, some penetrating the entire cortex below. In humans, these cells are frequently observed and were previously described by Golgi. They are radial, and their processes, directed towards the periphery, are stronger than others, ending in extensions triangular at their terminal ends. These neuroglial cells, which are of considerable strength, form a small protective layer for the crisscross of myelinated fibers below,

[1] Camillo Golgi, Sulla fina anatomia degli organi centrali del Sistema nervosa, Reggio Emilia, 1885, see also the above mentioned edition of Golgi´s reports in German language, 1894.

[2] Casimiro Mondino, Richerche macro e microscopiche sui centri nervosa, Torino 1887.

[3] Guiseppe Magini, Neuroglia e cellule nervosa cerebrali nei feti. Att d 12. Congresso della Assoc. med. Ital. in Pavia, sett. 1887, Vol1, Pavia 1888.

[4] Carlo Martinotti, Beitrag zum Studium der Hirnrinde und dem Centralursprung der Nerven. Inernat. Monatschr. für Anatomie und Physiologie, Bd 7, 1890.

This layer is more or less thick depending on location and on the type of animal; the cells which are mixed with the fibers are less strong and serve rather as a support.

Then Ramón y Cajal discussed and depicted the neuroglia of the brain in his groundbreaking studies on nervous system histology, although he did pay more attention to themes concerning the proper nervous elements. He depicted Bergmann fiber cells and a neuroglial cell in the cerebellum of a young cat[1] and provided an extensive presentation of the elements in the granule layer, which was first described by Vignal. In his more extensive treatise on the structure of the cerebral cortex, he addresses the question of neuroglia in greater depth.[2] With respect to the genesis of glial cells, he supports the view that at least part of the glial elements originate from epithelial cells that have migrated to the periphery. Such a migration can be observed in several stages in the fetal cerebral cortex of rats and mice, where the transition of ependymal cells into shorter, bulkier neuroglial cells with many processes can be documented. Regarding so-called spider cells, which do not show a radial arrangement and are present in the adult brain in connection with blood vessels, Cajal expresses the opinion that they do not originate from the epithelium but from endothelial cells of the blood vessels or flattened connective tissue. He doubts whether these spider cells originate from certain elements of the pia mater. In his tables, he provides images that depict the developmental stages under discussion.

In my preliminary report, I[2] described the neuroglial cells in the cerebral cortex of dogs, rabbits, and rats and provided images. Many of these cells are positioned, with their nucleus-containing cell body, at the surface level and extend from there, flattening out. They usually send root-like branched processes downwards into the cortical substance. Often, a strong main branch extends straight or winds in this direction and terminates in a narrow, highly branched structure. Other cells are embedded, with their nucleus-containing bodies, into the cortical substrate. As Martinotti observed in dogs, some send strong processes to the surface, where they end at the pia with a flat, thick extension. Others, particularly the lower-positioned ones, have the star-shaped appearance extensively shown by Golgi, but with branched processes. In the same report, I also described the Bergmann fiber cells in the cerebellum of a 7- and 8-month-old human fetus.

At the same time, Van Gehuchten[4] reported Bergmann fiber cells in the cerebellum of a young cat and star-shaped neuroglial cells with many processes in the granule cell layer and cord substance (dog). In his lecture on the human nervous system, published last year, Van Gehuchten[5] provides an account of neuroglia. According to him, it consists of ependymal cells and the spider cells of Deiters; the latter occur throughout the white and grey matter. Van Gehuchten emphasizes that their long processes end freely and without anastomoses. He supports the view that they represent

modified ependymal cells. Two such spider cells from the human white matter of cerebellar cortex are depicted in his work.

Since I do not focus on the neuroglia of the spinal cord, I will not describe the corresponding publications of M. von Lenhossek and myself, nor those by Lachi and some other younger Italian scientists. In the new edition of his large handbook[6], von Kölliker provides a comprehensive presentation of the entire neuroglia topic, and while he primarily addresses relations in the spinal cord, he also discusses conditions in the brain. After describing the development of neuroglia, especially the ependyma (focused on the spinal cord), he continues with a description of the mature cord and later addresses the brain.

[1] S. Ramon y Cajal. A propos de certaius elements bipolaires du cervelet, avec quelques details sur l´evolution des fibres cerebelleuses. Internat. Monatschr. für Anatomie und Physiologie, Bd7, 1890.

[2] S. Ramon y Cajal, Sur la structure de l´ecorce cerebrale de quelque mammiferes. La Cellule t. 7, 1891.

[3] Gustaf Retzius, Über den Bau der Oberfächenschicht der Grosshirnrinde beim Menschen und bei einigen Säugetieren. Verhandl. d. Biololg. Vereins in Stockholm. Bd 3, März 1891.

[4] A. Van Geruchten, La structure des centres nerveux. La moelle epiniere et le cervelet. La Cellule. T. 7, 1, 1891.

[5] A. Van Geruchten, Le systeme nerveuxde l´homme, Lecous professes a l´Universite de Louvain, 1893

[6] A. Von Kölliker, Handbuch der Gewebelehre des Menschen. 6. Aufl. Bd II, 1, 1893.

Von Kölliker strongly supports the view that the ependymal and star cells (Deiters or Golgi cells) originate from the same type of embryonic cells (cells of the ectoderm) and not from migrating mesodermal cells. Regarding the latter form of neuroglial cells, the star-shaped spider cells, which Kölliker describes as the proper Golgi cells, are represented in all parts of the grey and white matter of the cord and brain in a quite abundant manner. They form a scaffold with their multiple processes, crossing between all nervous elements and forming sheaths and wrappers for the nerve cells, larger nerve fibers, and vessels. These cells also form special wrappings for larger sections, such as the entire spinal cord or many parts of the brain, namely the glial cover. With respect to the form of these elements, von Kölliker distinguishes two variants: the Kurzstrahler and the Langstrahler. The former have shorter, strongly branched processes, while the latter have very long, sparsely divided processes. The Kurzstrahler are predominantly, but not exclusively, in the grey matter, while the Langstrahler are in the white matter. The cell body of the Kurzstrahler is commonly star-shaped, extending in all directions, whereas the Langstrahler are more elongated and often strongly flattened. Flattened shapes can also occur in the Kurzstrahler. The form of these cells shows the same diversity as that found in multipolar ganglion cells. The number of processes is rarely below five, often ranges from 10 to 15 or even 20, and is sometimes so numerous that it cannot be determined. They never anastomose, as Golgi first reported.

The doctrine by Ranvier (and Weigert) that the fibers (processes) do not directly belong to the cells, but are in a way independent, is discussed by von Kölliker in detail, and he explains the questioned assumption. He provides images of glial cells from the brain, specifically Kurzstrahler attached to the blood vessels in the hemispheres of rabbits and Langstrahler from the white substance of the human cerebral cortex and cerebellum. Moreover, he provides descriptions and images of cells from the cat cerebellum and the Bergmann fibers.

Ramón y Cajal[1] provided an illustration of the neuroglial cells of the fascia dentata. He found star cells in the peripheral border of the molecular layer, already described by L. Sala, and in addition, peculiar spindle-type (differently shaped) cells in the granule and molecular layers.

Last year, Lloyd Andriezen[2] published a report on a perivascular system of neuroglial cells in the human brain, obtained using the Golgi method, which he also verified in several other mammals. He states that the fibers extending from these cells form a dense network in two locations: first, at the surface of the brain, and second, around the blood vessels. The cells are of two types. The first consists of protoplasmic elements of mesodermal origin with active lymphatic function. These cells, which are star-shaped or dendritic in nature, as well as mossy or granular in appearance, reside in the grey matter. They are partially large cells with thick protoplasmic processes attached to the adventive sheath and partially small cells that enwrap the ganglion cells of the cortex. The second type represents fibrous elements of

epiblastic origin with supportive and passive functions. They form both a diffuse network and a compaction system, namely:

1. A diffuse network present throughout the cortex and spinal cord.
2. The above-mentioned compaction system at the surface and around the blood vessels.

The fibrous surface system represents a meshwork of tangential fibers stemming from the "tail-type" cells. From these cells processes or fibers extend that penetrate deep into the cortex, reaching as far as half its thickness, up to the pyramidal cell layer. In the perivascular fiber system, three variants can be distinguished. Firstly, cells are arranged along the length of the vessels with processes that can be followed along the vessels and also extend more or less numerous peripherally into the basic substance. These cells are positioned at a moderate distance from each other, forming a loose meshwork around the vessels. Mixed with these cells, one finds a second type of longitudinal cells

[1] S. Ramon y Cajal, Estructura del asta de Amon y fascia dentata. Trabajos leidos ante la Sociedad espanola de historia natural, 1893.

[2] W. Lloyd Andriezen, On a system of fiber-cells surrounding the blood vessels of the Brain of Man and Mammals, and its Physiological Significance. Intern. Monatsschr. f. Anat. u. Physiol. Bd10, 1893 – British Medical Journal, July 1893.

which are placed perpendicular to the vessels and together with the first mentioned cells form a perivascular network, that is in the basic substance around the vessels. Thirdly, there are the ordinary star-shaped cells dispersed in the basic substance, which occasionally also extend fibers to the perivascular network.

In the same year, a paper by Greppin[1] on the neuroglia of the human cerebral cortex appeared. Here, he distinguished two non-nervous cells:

1. Large mossy or bushy-type cells with multiple processes, which divide from their origin and among themselves. These processes anatomize with neighboring formations, generating a dense network. The cell body is irregular, sometimes angular, sometimes elongated, and rarely roundish.
2. Cellular elements with small, usually roundish or oval cell bodies, from which long radial-type processes originate. These processes rarely divide and never anastomose. Greppin assumes that the anastomosing cells correspond to the original spongioblasts described by His, while the star cells should be considered pure connective tissue elements.

In a short report, Azoulay[2] describes the forms of neuroglial cells in the Cornu Ammonis, the spinal cord, and the cerebellum in children a little over one month of age.

In a report published this year, Berkley[2] described the neuroglia and ependymal cells of the infundibular region and the walls of the third ventricle in adult dogs. He showed that in this region, ependymal cells are present in the adult stage, in a differentiated form, and extend from the ventricular wall to the outer surface of the brain. He describes the different cell types of the ependyma proper, as well as the neuroglia, and provides excellent images of these elements. In the walls of the infundibulum, he distinguishes, with respect to the ependyma (the ependymal neuroglia), one main form and several sub-varieties with distinct features of the trunk and branching. Moreover, large, star-shaped neuroglial cells with radial fibrous processes occur. In the walls of the third ventricle, he found a well-defined series of neuroglial cell forms, including two types of ependymal cells: long, thread-shaped, varicose cells (at the transition of the infundibular cave to the third ventricle) and ependymal cells with laterally extending branches (in the ventricular wall). Furthermore, there are different types of star- and mossy-type neuroglial cells. All these neuroglial cell forms, except for the embryonic types, are related to adjacent blood vessels through the attachment of their nodule-type endings to the hyaline sheath of the vessels.

In this historical overview, I have intentionally not discussed regions of the central organ that are genetically related but distinct from its typical structure, such as the hypophysis cerebri, nervi optici, and retina. I will address these tissues as a supplement below.

During the printing of this publication, I received a report with a new description of the neuroglial cells of the cerebellum by Van Gehuchten[4]. In it, he provides several images and a detailed description of neuroglial cells in the human cerebellar cortex, showing the features of these cells deep within the gyri.

Finally, it should be highlighted that Claudio Sala[5] provided an overview on neuroglia, discussing its relationship across different classes of vertebrates.

[1] L. Greppin, Ueber die Neuroglia der menschlichen Rinde. Anatom. Anzeiger, Bd9, No 3, Nov. 1893.
[2] L. Azoulay, Note sur le aspects des cellules nevroglique dans les organs nerveux centraux de l´enfant, Comptes rend. Hebd. D. s. de la Soc. De Biol., 1894, No 9.
[3] Henry J. Berkley, The Neuroglia Cells of the Walls of the middle Ventricle in the adult Dog. Anatom. Anzeiger, Bd 9, No 24 and 25, Aug. 1894.
[4] A. Van Gehuchten, La Neuroglie dans le cervelet de l´homme. Bibiolgraphie anatomique, Année 2, No 4, 1894.
[5] Claudio Sala y Pons, La Neuroglia de los Vertebrados. Barcelona 1894.

I. The Neuroglia of the hemispheres and the Ganglia of the Cerebrum.

(Tables I – X)

A. The Neuroglia of the human cerebral cortex. (Tables I – V)

I now present my findings and will specifically discuss the conditions in humans. In the present study, I have analyzed the fetal development of neuroglial cells in the brain. Since I did not succeed in visualizing the neuroglial cells within the first four months using the Golgi technique, I cannot report on the early embryonic stages or their formation from ependymal cells. Therefore, I will not discuss their lineage origin. My primary objective is to illustrate the development of the different forms of neuroglial cells and their transition into the forms observed in adults.

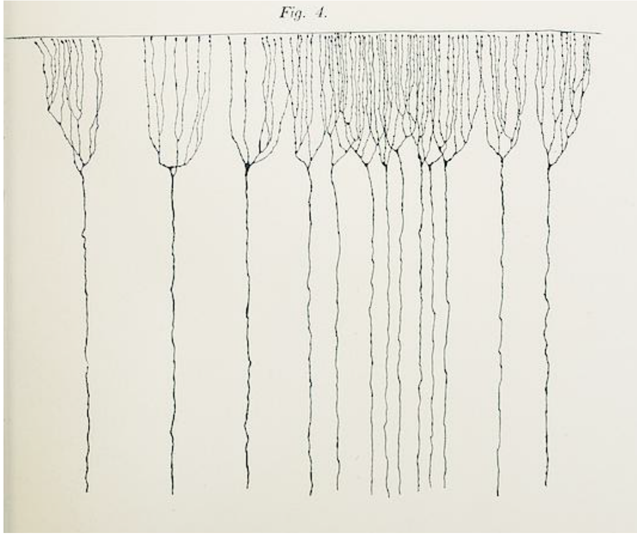

Fig. 4 from Table I. Vertical section of the cerebral cortex (frontal lobe) of a 19.5 cm long human embryo. Outer ends of the ependymal cells.

Regarding the ependymal cells proper, which span from the inner surface of the ventricular walls to the surface of the brain, they retain their repetitive structure throughout fetal development. I will not describe them in detail but will provide some images of their outer endings for comparison. In Fig. 4, Table I, a group of these formations from a human fetus measuring 19.5 cm in length is shown (frontal lobe). In Fig. 1 of the same table, two ependymal cell terminals are depicted (from a 6 ½-month-old fetus), while Fig. 1, Table XV of the second report, shows on the left a formation from the brain of a fetus measuring 28 cm in length. The cells display all the features described by me and others in previous studies, including those observed in other mammals.

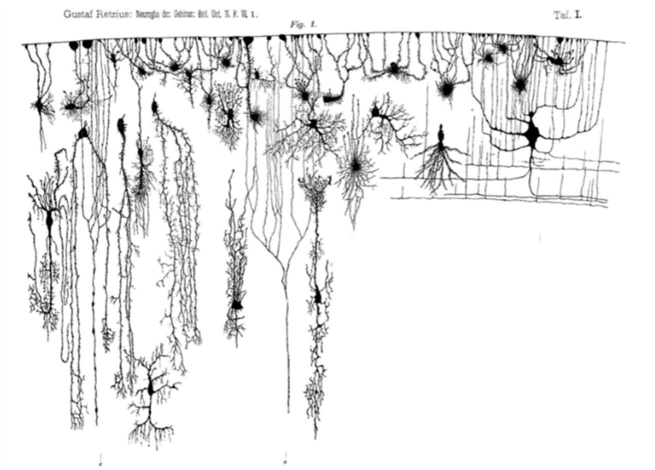

Fig. 1 from Table I. Vertical section of the cerebral cortex of a 6 1/2 old human fetus. Different types of neuroglial cells in their natural position in the cortex; - c, a Cajal cell, - e, terminals of ependymal cells.

The fibers are long, occasionally knobby, slightly bent, but run parallel to one another. They ascend more or less

vertically towards the surface and divide at the entry to the outer (so-called molecular) cortical layer dichotomously, with a knob at the point of division. The two divided branches ascend at a sharp angle towards the surface and then turn into an almost vertical direction. They typically divide one or more times dichotomously, with the delicate branches displaying similar features. All these branches ascend as a fascicle of capillary, knobby-varicose fibers towards the surface, where they terminate with a variably strong knot, which is either roundish-oval or conical. These terminals of the ependymal fibers can be observed throughout the fetal period, although they are difficult to stain during the later fetal stages. After birth, I could no longer detect them. Together with the ascending branches of the Cajal cells, they generate a characteristic grid or palisade formation.

In this grid formation of the outer cortical layer reside, aside from the cell bodies of the Cajal cells, a large number of glial elements of diverse shapes. In the following discussion, I will use the term neuroglia for all these support elements of the central organs that do not exhibit the true, original ependymal characteristics. The earliest stages of these neuroglial cells that I could demonstrate in the cerebral cortex using the Golgi technique were found in a 28 cm long fetus. In the next article (No. 2) of this volume, I have depicted (in Fig. 1 of Table XV) several of these cells together with Cajal cells and pyramidal cells.

Some of these cells can be identified as neuroglial cells based on their morphology, specifically the small cell body and the features of their processes. Others, by comparison, are less developed and therefore even more interesting, as they correspond to an earlier developmental stage. In these cells, one observes only one or a few processes originating from the small cell body, with few or no thorn-like branches. In form and arrangement, they are similar to Cajal cells, being at this stage distinct only in size. At times, it is not obvious which type of cell is being observed.

I have obtained abundant staining of neuroglial cells from a fetus in the middle of the seventh month. In Figs. 1–3 of Table I, some of the forms observed at this stage are illustrated, specifically in Fig. 1 in a vertical slice and in Figs. 2 and 3 in tangential slices of the cerebral cortex. In Fig. 1, in addition to a Cajal cell, one can see on the right, in the center, and on the left, two outer ends of processes of an ependymal cell. All other structures represent glial cells with distinct morphologies.

At the surface proper, I did not find labeled cell bodies, but I observed many cells situated just below the surface. These cells project processes to the surface, terminating with knot-like endings. These knots, which are strongly black-brown in color, vary in size—sometimes considerably—and may be larger than the cell body itself (Fig. 1 of Table I). The cell bodies are located at variable depths below the surface; they are generally small and roundish-oval or longitudinally elongated in form, often with angular extensions. They possess a variable number of delicate, short, mossy-type processes that extend in different directions.

Additionally, stronger processes originate from the cell body, varying in number—sometimes only one, in other cases two, three, four, or more. These processes sometimes extend directly to the surface but more frequently extend laterally first, subsequently bending and dividing once or multiple times before finally terminating at the surface. There, they end in the described knots. In the opposite direction, into the interior, another process extends from the cell body. This process may remain undivided or branch, typically featuring small, mossy branches that reach variable depths and terminate in a refined structure. The cell body tightly encloses the nucleus, which is only weakly visible in Golgi preparations. Due to the highly variable morphologies of these cells, it is not worthwhile to describe or categorize the different fetal cells in detail.

Fig. 2.

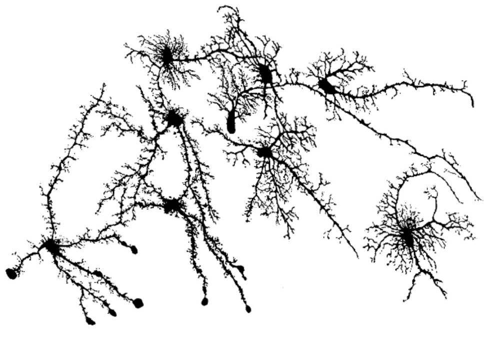

Fig. 2 from Table I. Tangential section of the cortical surface of the same fetus (a 6 1/2 month old human fetus), neuroglial cells.

Towards the interior from this cell region, one finds many glial cells which are distinct from the type described above despite showing diversity. In Fig. 1 of Table I, the commonly found cells are shown. Some of them are star-shaped, in the classic sense; from a roundish-oval cell body extend many processes into several directions; towards the outer region they are radial, are dividing repetitive in a dichotomic fashion and end freely after a

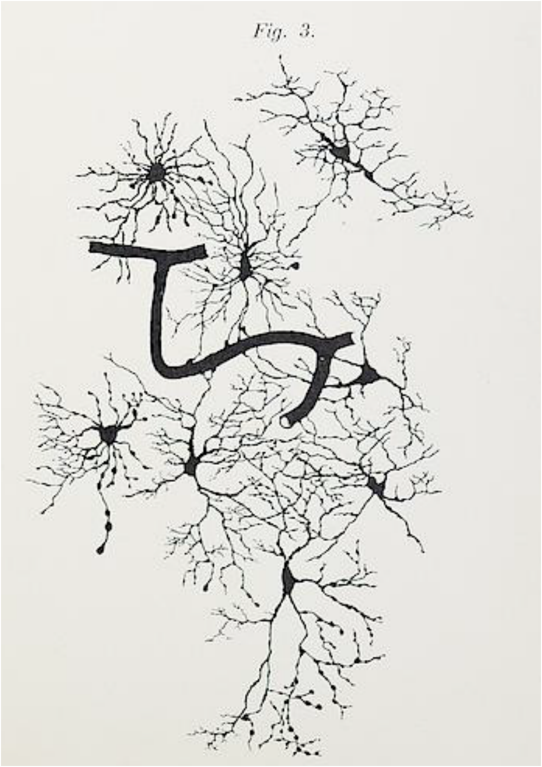

Fig. 3 from Table I. Section from the cortex of the same fetus (about 2.5 Mm.) below the surface. A blood vessel with attached neuroglial cells.

longer or shorter course; these processes are often equipped with small knots or spikes, sending out small branches, in particular those extending to the surface.

Based on the morphology and distribution of their processes, these cells vary considerably as shown in Fig. 1; sometimes their processes extend in all directions, sometimes mainly into one direction. The latter variation represents a transition to another unusual form which is characteristic for the fetal period. As shown in Fig. 1 of Table I (left), a number of these neuroglial cells with small, oval cell body sends one or two stronger processes to the interior towards the pyramidal cell layer; these processes extend vertically over a long distance into the mentioned layer, occasionally dividing in a dichotomic fashion and terminate with a delicate branching pattern; during their course the processes extend laterally a number of delicate, varicose, branched, often mossy small branches into all directions, Besides of those cells there are others in deeper layers which extend processes not only to deeper layers, but also towards the surface (Fig. 1 of Table I).

I do not find it worthwhile to provide a more extensive description of these neuroglial cells. There are a large number of variants, and describing them without additional images does not justify the effort. I have included images from two tangential slices. One (Fig. 2 of Table I) is a surface image that shows the branching pattern and arrangement of the processes of the superficial cells from an external perspective. The second (Fig. 3 of Table I) illustrates the cells in deeper layers, where one can observe the arrangement and branching patterns of these cells, as well as the attachment of some of their processes to the sheath of the blood vessels, as previously reported by Golgi and other researchers. In this context, I would like to draw attention to von Kölliker's illustration in Fig. 415 (d. Handb. d. Gewebelehre, Bd II, I, 6th edition), which depicts similar conditions in the hemispheres of the rabbit.

Direct connections among neuroglial cells or anastomoses are never observed. At the end of the intrauterine phase, the types of neuroglial cells undergo gradual changes, although they do not yet exhibit the characteristics of the adult stage.

Figs. 4 and 5 of Table II shows a series of preparations from the frontal lobe of a not delivered (45 cm long) human fetus summarizing the typical and succinct forms. True surface elements of the cerebral cortex are visible. With their cell body and its flattened or slightly convex surface they touch the interior side of the pia mater or even sometimes attach to it; seen from above one can recognize roundish-polygonal cell bodies of variable size in a hill-like fashion towering at the upper surface of the slice; sometimes they are equipped with a flattened, feet-like knot. These cell bodies, positioned at the level of the brain surface, extend processes radial into all directions and form a surface net of neuroglial fibers which extend tangential through the outer cortical layer and intermingle among each other. From the lower surface of the cell bodies, which is sometimes short and roundish, sometimes of triangular form pointing towards the interior, there extend processes, partially lateral, partially towards the interior; some are short, others extend deeply through the molecular and pyramidal cell layer; these

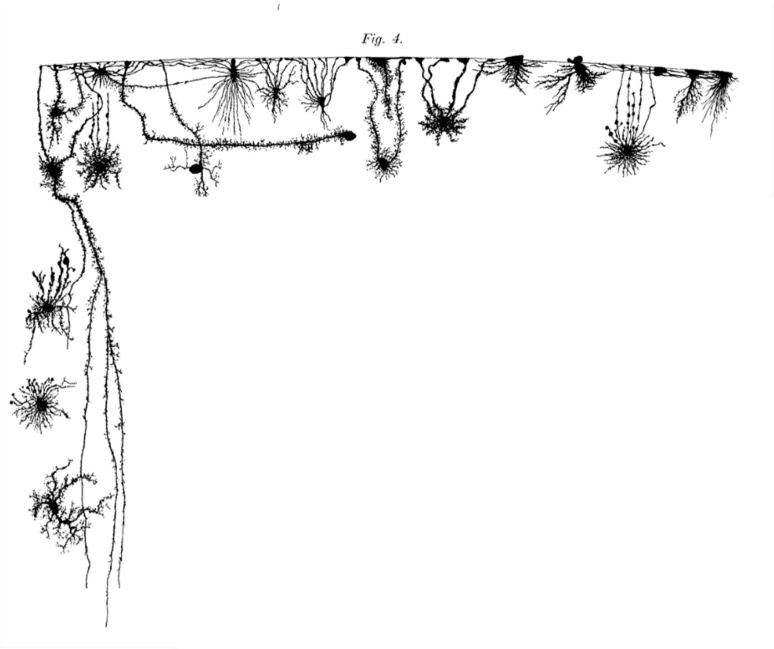

Fig. 4 from Table II. Vertical sections of the cortex of a gyrus of the frontal lobe from a 45 cm long human fetus. Neuroglial cells of different types are shown.

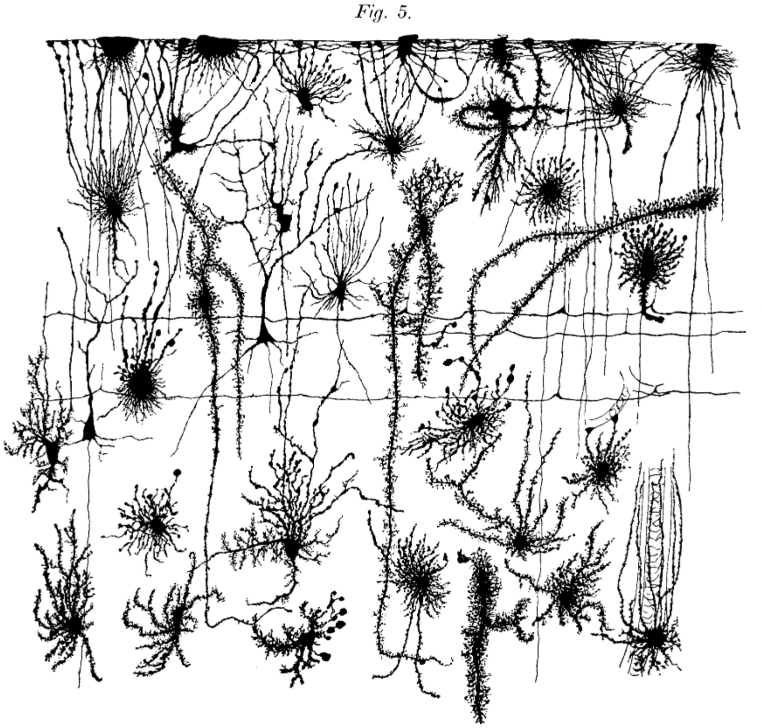

Fig. 5 from'Table II. are, in addition, two small pyramidal cells and some tangential fibers (probably processes of Cajal cells) shown and (on the bottom on the right) a blood vessel surrounded by a glial cell.

latter processes are thread-like elongated, unbranched and contain knots (Fig. 5 of Table II).

Below those cell bodies—whose precursors from earlier stages of fetal development I have not yet been able to identify—one can find the elements described in the seventh month. Their cell bodies are located slightly below the surface of the cortex, and they extend a number of typically stronger processes that diverge more or less towards the surface, where they terminate as knot-shaped structures (Figs. 4 and 5 of Table II). Below these are either star-shaped cells with many processes extending in all directions or cells with long, tangentially oriented processes, which are thick and possess mossy-type branches (Fig. 4 of Table II). These processes sometimes bend upward to terminate at the surface (Fig. 5 of Table II) or extend into deeper layers.

Next, I must consider the vertically extended cell types that I described above in the seven-month-old fetus. Indeed, such cells are still present at these later stages, although they have undergone some transition. They now exhibit fewer processes, and both the processes and the cell body itself are mossy in nature, meaning they possess multiple, delicate, knobby-varicose branches (Figs. 4 and 5 of Table II). The cell depicted in Fig. 4 even extends two processes towards the surface, which is a rare occurrence.

Among these cells, one encounters many elements that can be attributed to the star-shaped type, although there is a large variety and transitional forms leading to other shapes. These cells typically give rise to one or several processes (Figs. 4 and 5 of Table II). The processes of these cells sometimes contain larger or smaller drop-shaped enlargements (varicosities) (Figs. 4 and 5). Here and there, neuroglial cells are found projecting processes to a vessel wall, where they attach with a conical foot (Fig. 5 of Table II, right) or extend a long process parallel to the vessel wall, enwrapping it, as described in more detail by Golgi and Andriezen.

I have studied the neuroglia of the brain in a number of newborn children and found similar conditions to those described in the 45 cm long fetus. It is sufficient to refer to these figures. Fig. 1 of Table II shows a vertical slice, Fig. 2 illustrates a cell from the cord tissue, and Fig. 3 presents a surface image from a 48 cm long fetus. The star-shaped cell in Fig. 2 from the cord tissue, like the lowest cell in Fig. 1, already shows the differentiation characteristic of neuroglial cells in the white matter. Fig. 3 shows four superficial cells from above. Anastomoses between glial cells are never observed.

Gustaf Retzius: Neuroglia des Gehirns: Biol. Unt. N. F. VI. 1. Taf. II.

Fig. 1.

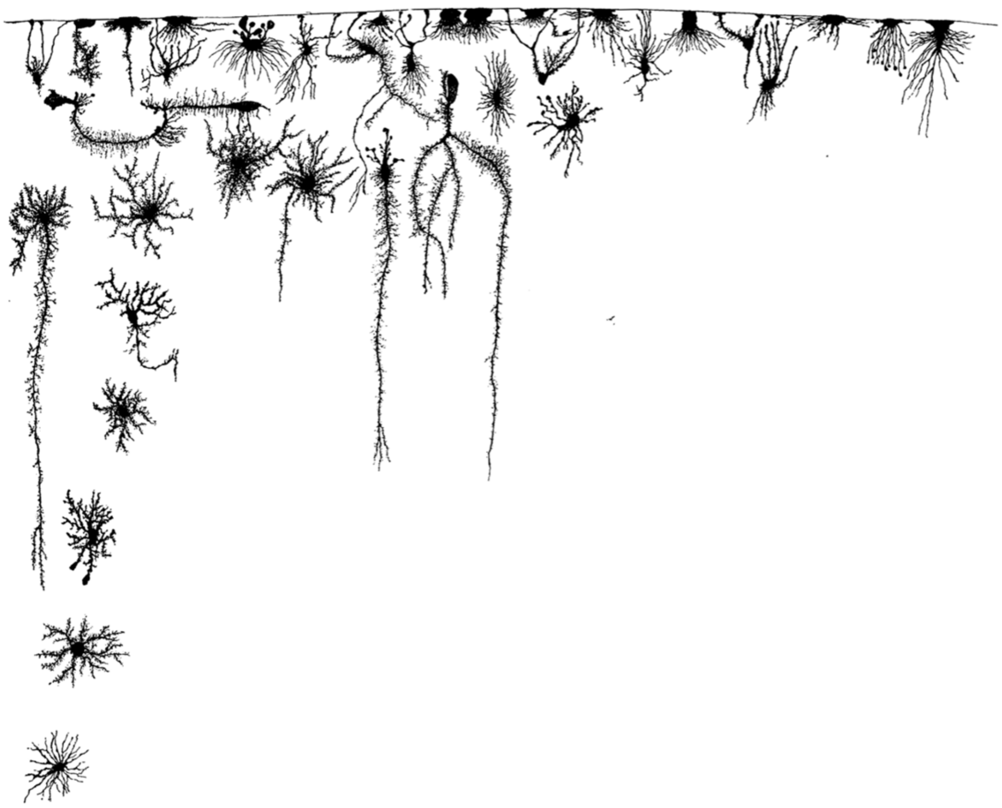

Fig. 1 from Table II. Vertical section in the cortex of a gyrus of the temporal lobe from a delivered 48 cm long (female) human fetus. Neuroglial cells of different forms, most of them of the fetal type.

Fig. 2.

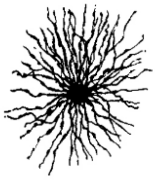

Fig. 2 from Table II. A Sternstrahler from the inner white substance of the preparation shown in Fig. 1.

Fig. 3.

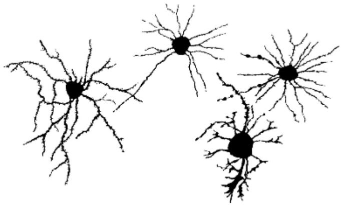

Fig. 3 from Table II. Tangential slice of the cortical surface of a gyrus of the temporal lobe of a delivered 48 cm long human fetus. Four cell bodies of Schwanzsternstrahler with processes of the fetal type.

In the first month after birth the majority of the neuroglial cells retain the features as described above, yet they more and more acquire the type of the differentiated state; this is particularly evident for the superficial cells. Figs. 1, 2 and 3 of Table III are from a two-month-old, Figs. 4 and 5 of the same Table from a three-month-old child. In Fig. 1 and 4, one can recognize cell bodies at the surface which partially protrude in knob-like fashion. Their processes are tangential oriented and are very long, are stretched and running downward; furthermore one finds cell bodies way below the surface, which project a variable number of diverging processes towards the surface; further below the peculiar, long, more or less vertically oriented cells

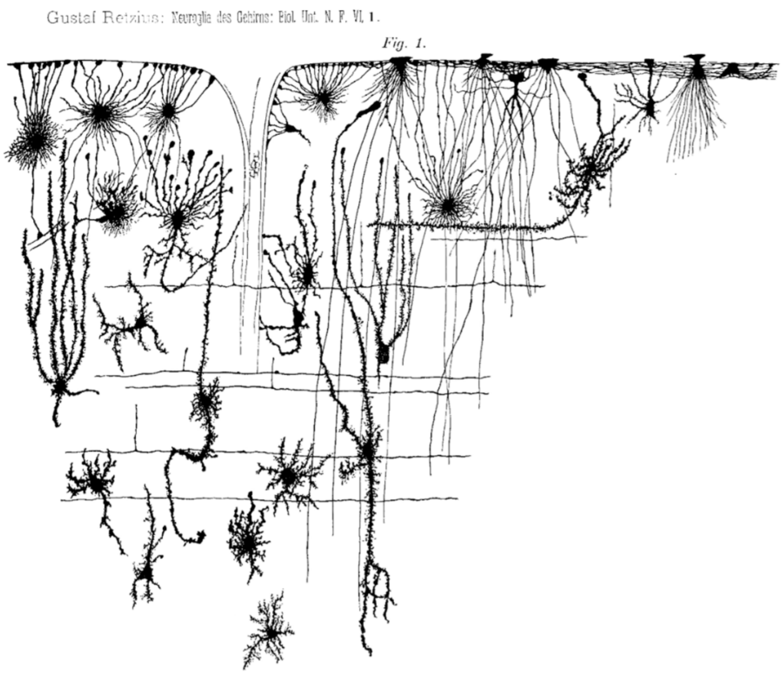

Fig. 1 from Table III. Vertical section of a gyrus (G. front. Med.) of the cerebral cortex of a 2-month-old child. Neuroglial cells of different types in their natural position in the cortex. In the center, a blood vessel inserts from the surface. Some tangential fibers are visible further below.

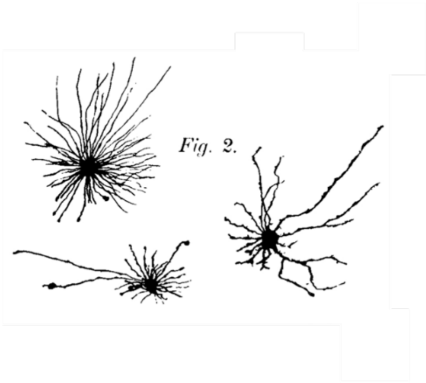

Fig. 2 from Table III. Three Sternstrahler from the deeper region of the same vertical slice.

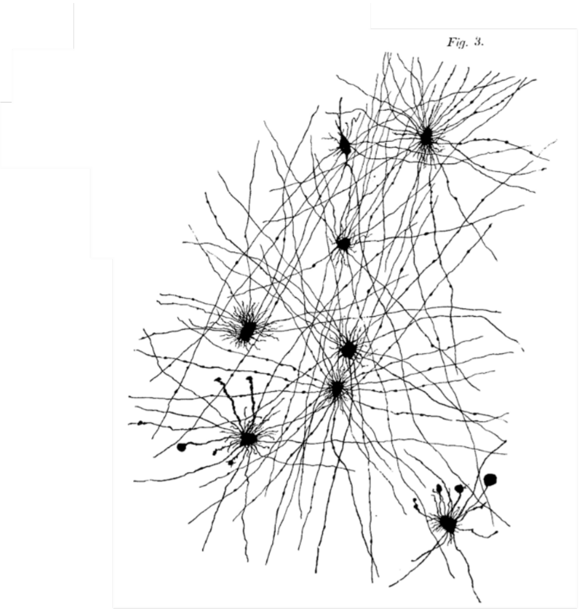

Fig. 3 from Table III. Tangential section of the surface of the cortex (Gyr. Temp. inf.) from a 2-month-old child. Neuroglial cells

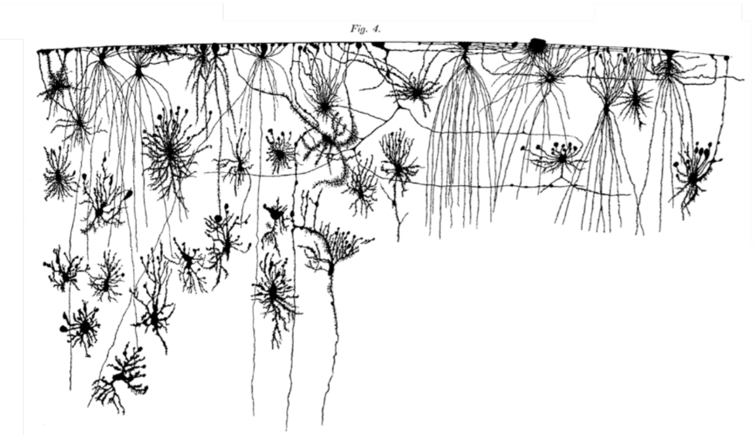

Fig. 4 from Table III. Vertical section of a gyrus of the frontal lobe in the cortex from a 3-month-old child. Neuroglial cells of different types in their position within the cortex.

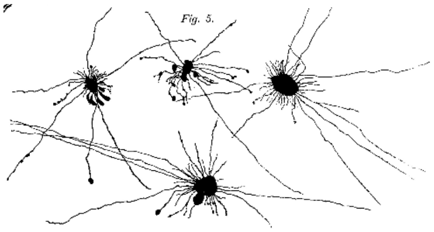

Fig. 5 from Table III. Four neuroglial cells of the cortical surface in tangential extension (tangential slice of the cortical surface).

and finally, the star-shaped cells. Their processes in the grey matter are branched, mossy and winding, in the white matter (Fig. 2) they are less branched, delicate fibered and elongated. Viewed from the surface of the cortex (Fig. 3 and 5 of Table III), the cells in that layer have a distinct, angular-roundish or oval shape; from these cells processes originate into different directions which are long, delicate, fibrous, elongated and only slightly bend and contain knots; they cross-over and generate the neuroglia felt of the cortical surface as described above; moreover, one can recognize the delicate short processes projecting down from the cell body.

By the end of the first year and continuing into subsequent years, the neuroglial cells of the cerebral cortex acquire their final, mature form. Fig. 1 of Table IV and Fig. 2 of the same table provide examples of vertical sections: the first is from the frontal lobe of a one-year-old, and the second is from the frontal lobe of a 5½-year-old child. The neuroglial cells can be subdivided into four types: cells with cell bodies touching the surface and long, fibrous processes running either tangentially to the surface or more or less vertically through the outer cortical layers (molecular and pyramidal cell layers); cells with cell bodies located below the surface that send processes to the cortical surface; and two types of star cells—one equipped with highly branched mossy processes in the grey matter, and the other with long, thread-like, elongated processes that are branched only at their origin.

For a systematic presentation of the neuroglia, it would be appropriate, and perhaps necessary, to establish specific names for the different types. There are transitional forms between these types, indicating their interrelationship. However, most of these elements have sufficiently defined characteristics to allow a categorization. Regarding nomenclature, von Kölliker divided the neuroglial cells into Kurzstrahler (short projectors) and Langstrahler (long projectors), with these names corresponding to the structural features of the elements. Additional names based on other characteristics could include Krausstrahler (frizzy projectors), for cells with mossy, winding, and branched processes, and Schlichtstrahler (simple projectors), for cells with straight and elongated processes. While these terms provide a broad classification, they are insufficient to fully characterize the different types of neuroglial cells, and further sub-classifications would be desirable. Due to the variability in form, finding precise names remains a challenge.

It is also relevant to compare the neuroglial cells of the cerebral cortex with those of the spinal cord, as described by von Lenhossek[1] and myself. Indeed, the neuroglial cells in the brain correspond closely to those in the spinal cord, with clear conformity between the types. The Kurzstrahler or Krausstrahler of the spinal cord grey matter are similar to those in the grey matter of the cerebral cortex. The cells in the spinal cord deviate slightly more from the star shape but are fundamentally of the same type. In the center of the spinal cord, there are long-projection "star cells" located ventral to the central canal in the frontal white commissure. These characteristic cell types of the cord substance are present

throughout the white matter. Similarly, the superficial neuroglial cells of the spinal cord, which pass through the strands and reach the surface with their cell body or processes, are comparable to those in the cerebral cortex. From the inner end of their cell body, both types send a bundle of long, simple processes inward. The cells in the cerebral cortex, however, are more regularly formed. In both the spinal cord and the brain, cells positioned below the surface send processes to the surface. Considering that the outer layer of the cortex also contains a white matter region, the similarity becomes even more evident. In the cerebral cortex, the long-projection cells send processes deep into the grey matter. Lloyd Andriezen has obviously observed cell bodies at the surface of the cerebral cortex which send bundles of long projecting processes into the layers below;

[1] M. v. Lenhossek, Zur Kentniss der Neuroglia des menschlichen Rückenmarkes. Verh. D. Anatom. Gesellsch. Auf d. fünften Versamml. In München, 1891.
[2] Gustav Retzius. Studien über Ependym und Neuroglia. Biolog. Unters. v. G.Retzius. N. F. Bd V, 2, 1893.

he provides a fairly correct image (Fig. 7 of above-mentioned publication) and calls them ´caudate glial cells´. This description caudate cells or tailed cells is quite appropriate while it does not consider the other properties of these cells, namely the position of the cell body at the surface, or the presence of tangential processes which form the felt of glial fibers.

As emphasized above, it is important for the description of neuroglia, or more succinctly named glial cells (Gliäcyten), to define different types. Since I hesitate to introduce new names if appropriate ones have already been proposed by other scientists, I will, in addition to those proposed by von Kölliker and Andriezen, only add names to describe the essential types. I distinguish Sternstrahler (star projectors) and Schwanzstrahler (tail projectors) (ureide Gliäcyten)[1], depending on whether their processes project in all directions or predominantly in one direction. The first group corresponds to the neuroglial cells of Golgi and Deiters (the star cells or spider cells of these authors). The latter are the caudate glial cells of Lloyd Andriezen.

Among the Sternstrahler (star projectors), I further distinguish, following von Kölliker, Kurzsternstrahler (short star projectors) and Langsternstrahler (long star projectors) (macroureide and brachyureide Gliäcyten). Most glial cells can be categorized into these groups. However, there are some deviating forms that cannot be included in this classification. First, there is the fourth type of glial cell of the cerebral cortex, as already described above. These cells have their cell body located below the surface and send a number of processes to the surface, where they terminate with knots and feet. These cells are commonly "star-shaped" Sternstrahler (star projectors), and their processes projecting to the surface are longer and equipped with endfeet. Such cells also occur in the vicinity of blood vessels, to which they attach with conic, swollen feet. I will call them Fuss-Sternstrahler (foot-star projectors) (podasteroide Gliäcyten).

Additionally, there are glial cells that send processes not in one direction but in two equally well-developed and long directions, resembling a sandglass in form. These cells can be considered a subtype of the caudate glial cells and will be termed Doppelschwanzstrahler (double tail projectors) (biureide Gliäcyten).

Finally, there are glial cells that are spread in a laminar fashion. Such elements occur sporadically at the surface of the cerebral cortex, and this category includes the sheath-like oriented elements around blood vessels, as described by Lloyd Andriezen. These cells extend partially in a flat plane and partially in a curved plane. I will name them Flächenstrahler (plane projectors) (plakoide Gliäcyten). These cells can either be more planar or more tubular in structure.

In contrast, I do not find it necessary to name the other deviating forms, particularly those of the embryonal and fetal types, nor the different types of ependymal cells, respective fibers, and their transitional forms to Gliäcyten, although this may happen in the future.

In the context of this name finding, I would also like to comment on the terminology of the different layers of the cerebral cortex. The outermost layer is commonly termed as the first, with low cell density or the molecular layer (the fine-granular layer). None of these terms fit. According to my opinion the only relevant classification is determined by the nerve cells which reside in this layer, which is the most important and determining as it has been long considered for pyramidal cell layer. For this outer layer, the characteristic nervous cell is the Cajal cell. These cells reside with their processes in different levels from the surface to the small pyramidal cell layer, but do not enter it as far as we know; these Cajal cells reside with their processes only in this first layers and penetrate it in all directions, in particular in human. I find it therefore appropriate to term this layer after these cells;

[1] to a certain extent their form reminds on a comet tail, and maybe someone in accordance with the astronomic terminology of stars may propose to term ´comet cells´ for these formations!

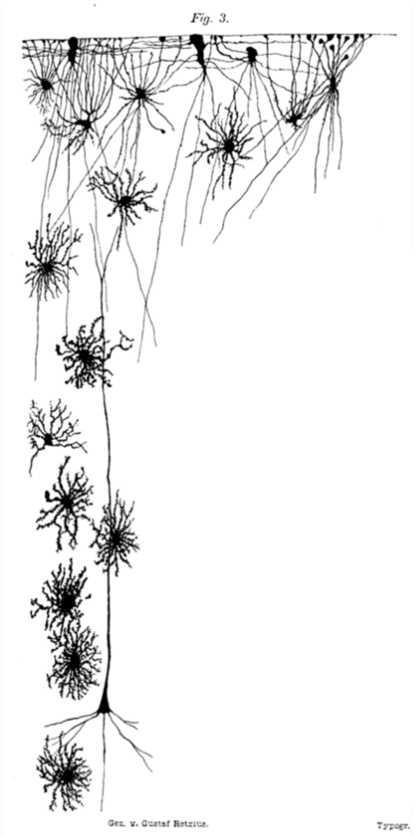

Fig. 3 from Table IV. Vertical section of one cortical gyrus of the occipital lobe (Gyr. occ. med.) of a 17-year-old man. Neuroglial cells of different types and a pyramidal cell.

and to make this term as incisive as possible, one could consider them as the layer of the Cajal cells or shorter the Cajal cell layer, quite in accordance with the layers below, the layer of the small pyramidal cells or the small pyramidal cell layer (small pyramidal layer) or the layer of the large pyramidal cells, the large pyramidal cell layer (large pyramidal layer). For the fourth layer above the white matter is best the terminology of Cajal: the layer of the polymorph cells or the polymorph cell layer.

After his excursion on the field of nomenclature, I will return to the illustration of neuroglia in the cerebral cortex. The last stage which I have described above are from a 1-year-old and 5 1/2-year-old child. In an 8-year-old I did not see any differences and I therefore provide no illustrations. From a 17-year-old man, I provide Fig. 3 of Table IV; one can recognize the form of glial cells as in the child. At a 33-year-old man (Fig. 4 of Table IV) it is the same. At a Langschwanzstrahler (long tail projector) there is a colossal, balloon-shaped protrusion protruding into the brain surface; such protrusion, usually to a smaller extent occur occasionally and are attached to the pia at regions where the pia is attached to the cerebral cortex.

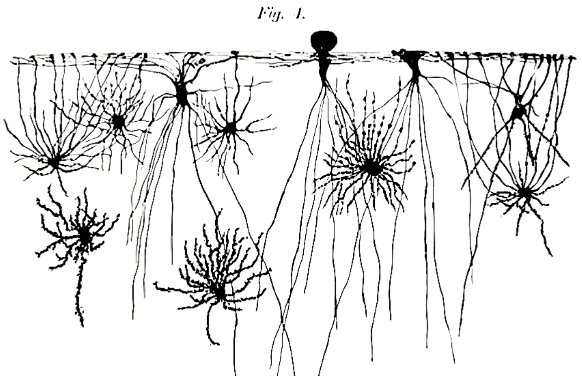

Fig. 4 from Table IV. Vertical section of one cortical gyrus of the parietal lobe (Gyr. centr. post.) from a 32-year-old man. Neuroglial cells of different types.

Then I have studied the neuroglia in different human brains of medium age. Fig. 1 of Table V shows a part of a vertical slice from the frontal lobe from a 42-year-old woman and Fig. 5 from Table IV a tangential slice (from the gyr. Centr.post.). On can recognize (in the last figure) the main types, Sternstrahler (star projector) and Schwanzstrahler (tail projector) in different variants; striking are in particular the thick, coarse-armed Sternstrahler, which we have not found

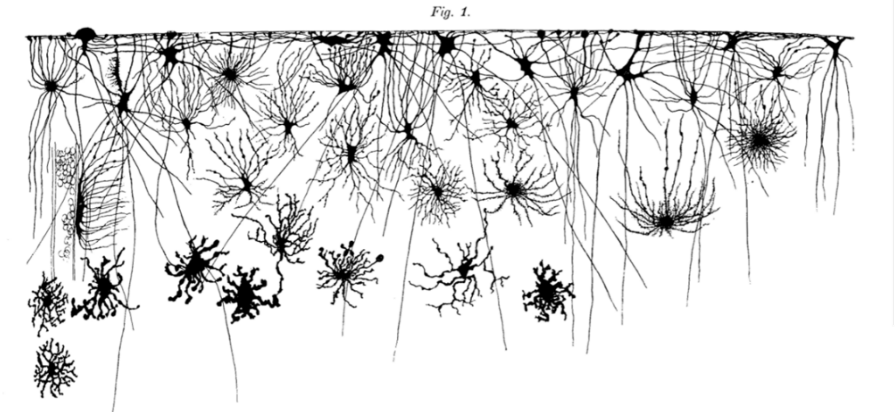

Fig. 1 from Table V. Vertical section of a gyrus of the frontal lobe from a 42-year-old female.

there are several Fusssternstrahler (foot star projector) which send their processes to the surface. At the latter, one can recognize a true Flächenstrahler (plane projector) without tail. Finally, one finds at the blood vessels (left) a sometimes-occurring unusual form, which has not been illustrated by Lloyd Andrizien; the cell body is attached to the vessel sheath, but the processes do not surround it, but project in contrast to the brain substance. The tangential image, seen from the surface, shows four Schwanzstrahler (tail projector), from which part of the plane processes can be recognized, but not the tails. In between is a cell which corresponds to the category of the Fussstrahler (foot projector), but not very typical and finally a group of Sternstrahler (star projector) in deeper layers.

I have studied the neuroglia of older individuals and found corresponding conditions. This is why I show

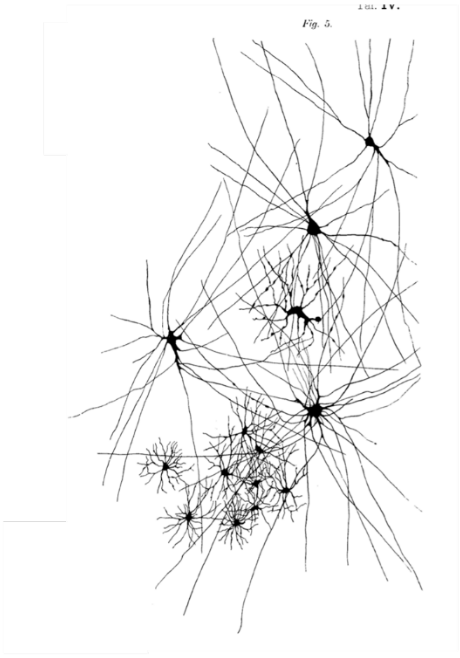

Fig. 5 from Table IV. Tangential section of the cortical surface (Gyr. centr. post.) of a 42-year-old female. Flächen- and Schwanzstrahler and Sternstrahler at deeper layers.

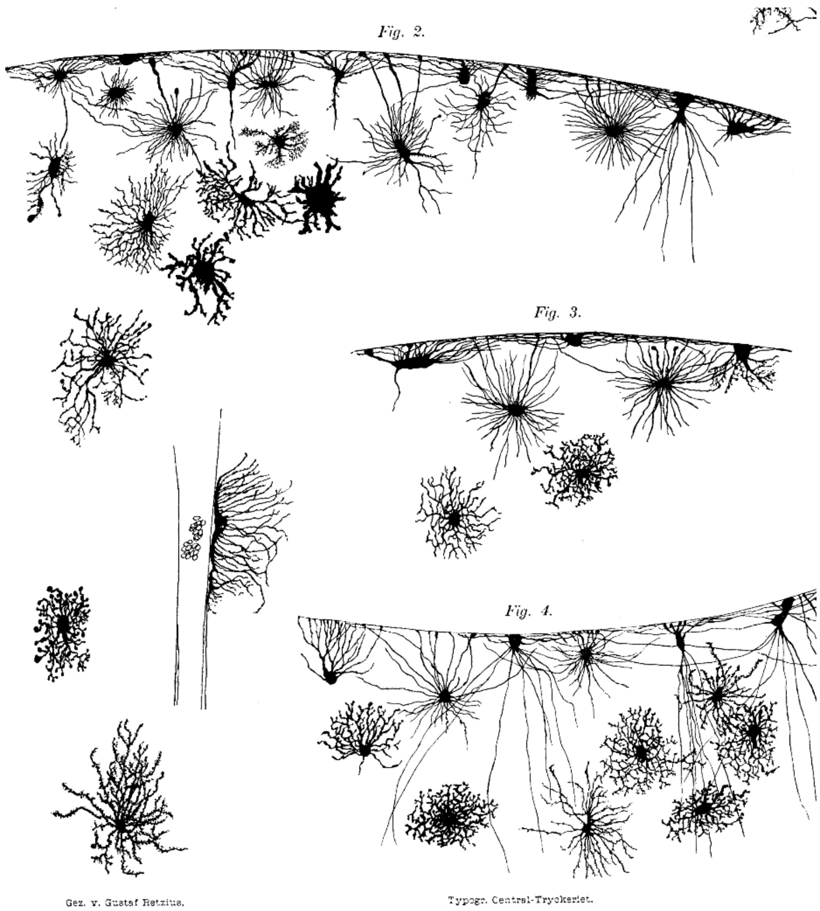

Fig. 2 – 4 from Table V. Vertical section from the parietal region from a 70-year-old man.

man. Figs. 2-5 of Table V show parts of a vertical slice from the parietal region of the cerebral cortex. In all these tissues, one can recognize the above described, different types of Gliäcytes. Among the Sternstrahler (star projector) there are numerous coarse-armed cells (Fig. 2 and Fig. 5). Moreover, there are several Flächenstrahler (plane projectors) visible (Fig. 2 and 3). Delicate projecting cells are still numerously present and they are sometimes so abundantly branched that they could appear, if not studied in detail, as a black clump (Fig. 5). In Fig. 2, I have depicted such a cell, like in Fig. 1 of Table IV from a 42-year-old woman; a cell whose cell body attaches along the vessel sheath while sending the processes to the brain substance. What is obvious in studies of neuroglia of the cerebral cortex of elderly individuals is the stiffening of the processes of the elements at the surface; they appear more stretched, coarser and more equipped with less branches. Whether the processes become rarer, I cannot decide; in these preparations it appears to be the case, but this can also be due to the Golgi method which may stain less processes in this old tissue.

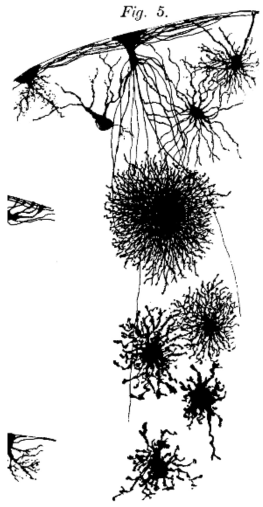

Fig. 5 from Table V, Vertical section from the parietal region from a 70-year-old man.

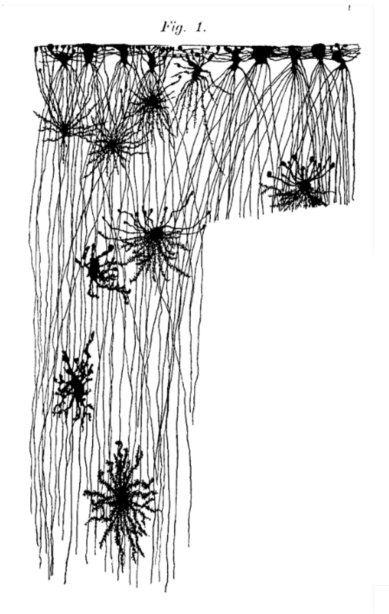

Fig. 1 from Table IV. Vertical section of one cortical gyrus of the frontal lobe from a 1-year-old child.

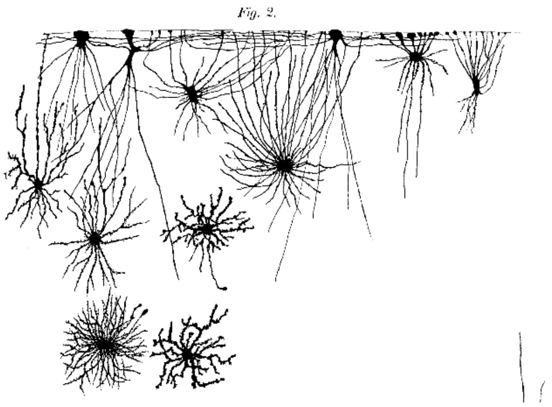

Fig. 2 from Table IV. Vertical section of one cortical gyrus of the frontal lobe from a 5 ½-year-old child. Neuroglial cells of different types.

B. The neuroglia of the cerebral cortex of dog, cat and rabbit

(Tables VI – VIII)

To compare the conditions in human, I have studied several mammals, in particular dog, cat, rabbit, rat and mouse, but also bovine, sheep and pig. Here I will only report on my results in dog, cat and rabbit. I do not feel that an extensive description is necessary, since the attached figures show the relations better than a verbal description.

In dog (Table VI), I restrict my illustration of Gliäcytes to the first and second month after birth and to the adult (old) stage. Already at two earlier opportunities, I have described and illustrated the most important types. Among the lower figures on Table VI, Fig. 1 shows the cortical area of a 2-month-old, Fig. 3 of a 1-month-old dog. Fig. 2 shows three glial cells in the white matter of a 2-month-old dog. In these figures one can recognize easily the types as described in human, while there are some differences quite obvious. Thus, we have in the interior the Sternstrahler, namely the Kurzstrahler in the grey matter (Fig. 1) and the Langstrahler (Fig. 2), despite of the term Krausstrahler or Schlichtstrahler would be more appropriate. The Kurzstrahler have a distinct fizzy or mossy appearance, while the Langstrahler of the white matter have simple, straight and delicate processes which are not very long. Among the surface located cells, the Fusssternstrahler are particularly well represented; Fig. 1 and 3 shows examples and one can recognize the cell bodies with multiple, delicate branches, which results in a mossy appearance; stronger processes project to the surface and are often equipped with delicate branches and they terminate at the surface with knots or conic feet; in addition, the cell body projects a

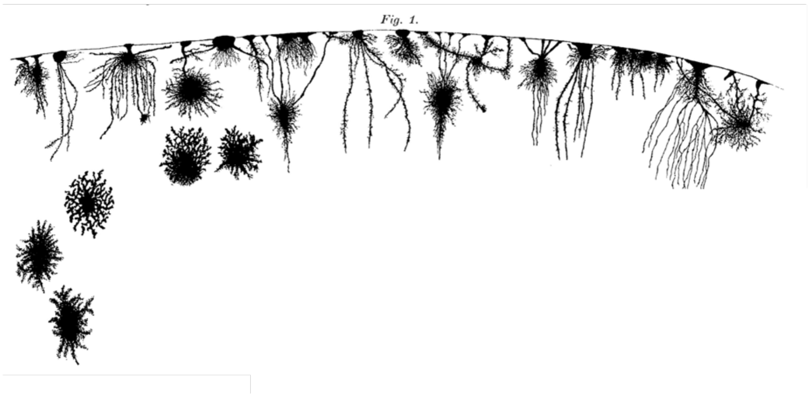

Fig. 1 from Table VI. Vertical section of a gyrus from the vertex of the cerebrum of a 2-month-old dog.

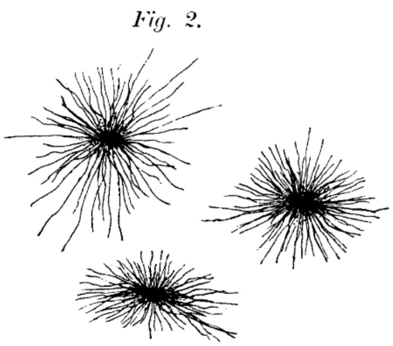

Fig. 2 from Table VI. Three Langsternstrahler from the white matter of the same preparation; its surface is shown in Fig. 1 (from a 2-month-old dog).

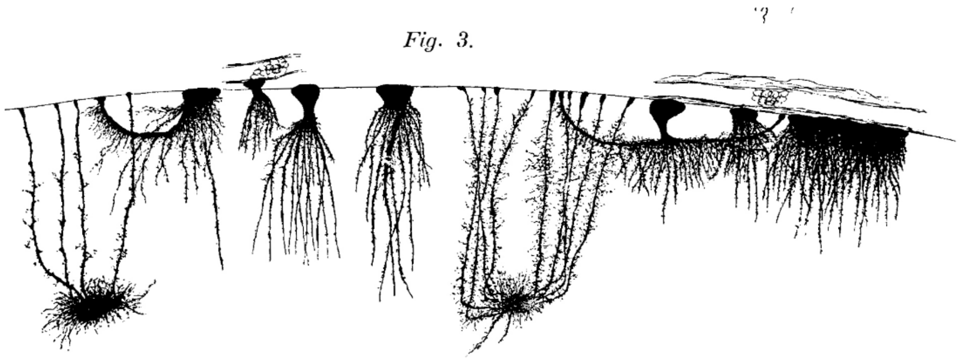

Fig. 3 from Table VI. Vertical section of a gyrus from the vertex of the cerebrum of a 1-month-old dog; at the surface rim are two blood vessels of the pia depicted with attached cell bodies of Schwanzsternstrahler.

process or a mossy plait towards the interior. The Schwanzstrahler are also present, while they show a slightly variant form; the broad, knob-shaped cell bodies close to the surface project a number of stronger, but not too long, mossy, branched processes, some are only a bundle of shorter, mossy branches. There also exist peculiar forms with lateral processes terminating at the surface (Fig. 3).

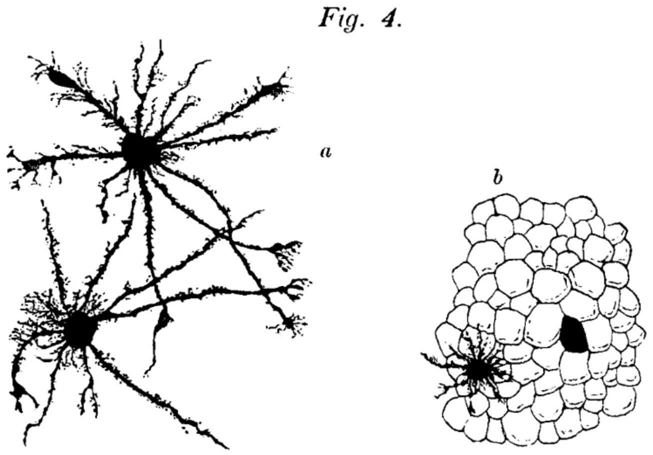

Fig. 4 from Table VI. Parts of tangential sections of the surface of the cortical hemisphere from a 2-month-old dog. – a, two glial cell bodies with their processes positioned at the surface – b, part of the surface with mosaic pattern, corresponding to the contour of the cell bodies of glial cells at the surface; two cell bodies are entirely attained, one with stained processes.

While inspecting the brain surface freed of pia from above, one can recognize here and there the stained cell bodies of the Schwanzstrahler located at the surface with processes extending into all directions (Fig. 4a, left); the cell bodies are roundish-oval, sometimes angular. At occasions, one observes areas on the surface, where not only single or groups cell bodies are brown-black labelled, but in between an epithelioid mosaic delineation (Fig. 4 b, right) with roundish-polygonal fields of variable size. After inspecting a number of preparations with this mosaic patter, I conclude that these fields correspond to the cell bodies and their process terminals, the feet of the surface located cell bodies. It is not impossible that this mosaic was considered by some researchers as an epithelial (endothelial) membrane. Considering as the structure is, it can be nothing else as a mosaic arrangement in which a dense mass of polygonal cell bodies intermingles with feet of the Fusssternstrahler at the surface. With strong Golgi staining there are so many endplates labelled black that one cannot resolve the mosaic, with less intense staining where most endplates appear

as contours (Fig. 4 b), the image is clearer and more obvious. In vertical slices one often observes groups of dark labelled cells (Fig. 3, right) where the borders of the cells are difficult to discern. One can easily imagine that it can form a mosaic at the surface. At those vertical sections with attached pia, one can observe that the surface of the cell bodies is closely attached to the pia.; sometimes they protrude, like in humans, above the surface in a knob-like fashion and attach with their terminal area to a blood vessel of the pia (Fig. 3, left).

When we inspect the conditions in the mature dog brain, we find that it is very similar to the earlier stages, as shown in Figs. 5 and 6 of Table VI. The Schwanzstrahler are slightly more developed, often displaying broader, upper cell body plates and possessing longer, simpler, and less mossy processes compared to the earlier stages.

The Gliäcytes depicted here represent the general forms found in the dog's brain, but not all forms are included; there are a number of variants, particularly in the surface layer.

On the same table, namely VI, I have provided a figure (Fig. 7) that shows the outer edges of the ependymal cells of a newborn, or more accurately, a 5-day-old dog. I have previously (Biol. Unt., N. F. B. V., Table I, Fig. 1) published similar cells in a 14 cm-long fetal dog.

I believe it may be of interest to demonstrate that these fibers can still be observed after birth and to describe their function. As shown, they are of the same type, possibly slightly more developed, and their knob-shaped ends reach the surface. Among them, three Gliäcytes are depicted, displaying peculiar forms.

Fig. 5.

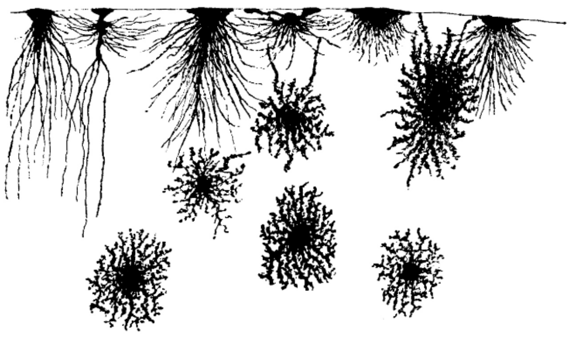

Fig. 5 from Table VI. Vertical section of gyri from the vertex of the cerebrum of an adult (old) dog.

Fig. 6.

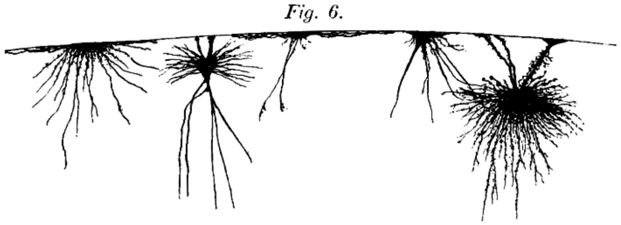

Fig. 6 from Table VI. Vertical section of gyri from the vertex of the cerebrum of an adult (old) dog.

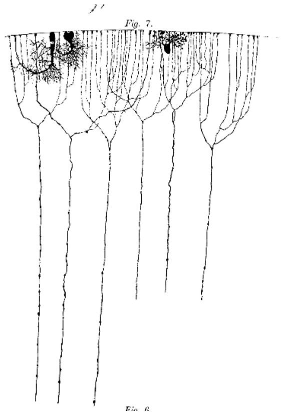

Fig. 7 from Table VI. Vertical section of a gyrus from the vertex of the cerebrum of a 5-day-old dog. Besides the three glial cells present below the surface, there are the outer edges of six ependymal cells displayed in the figure.

In cat (Table VII) one finds similar conditions in the adult (Fig. 4 - 6) as in the dog brain. The Gliäcytes are fairly big and strongly developed, the elements of the surface layer generally have a mossy appearance. One can recognize similar forms as in the dog brain and I will not provide a detailed description and refer to the figures. One observes the strong difference between the Kurzsternstrahler of the grey matter (Fig. 4) and the Langsternstrahler of the white matter (Fig. 5), furthermore the different forms of Schwanzsternstrahler (Fig. 4) and the Fusssternstrahler which are weakly represented in Fig. 4.

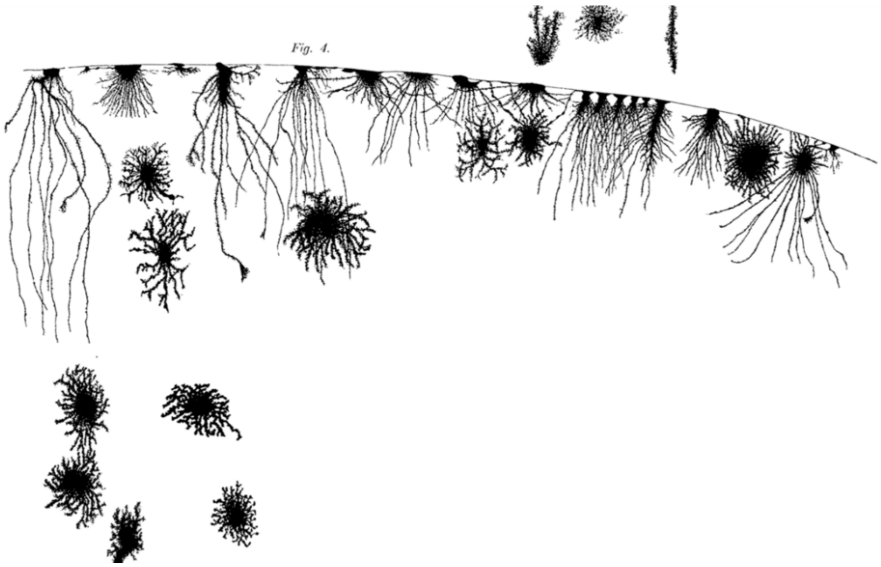

Fig. 4 from Table VII. Vertical section from the vertex of the brain from an adult (old) cat.

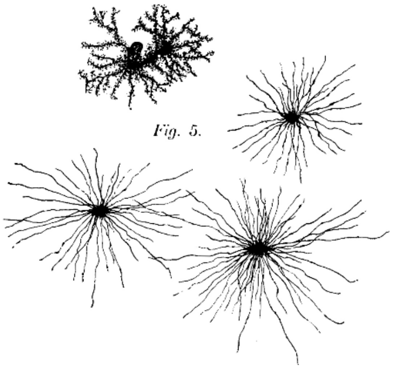

Fig. 5 from Table VII. Three Langsternstrahler from the white matter below the cortex at the brain vertex of an adult (old) cat.

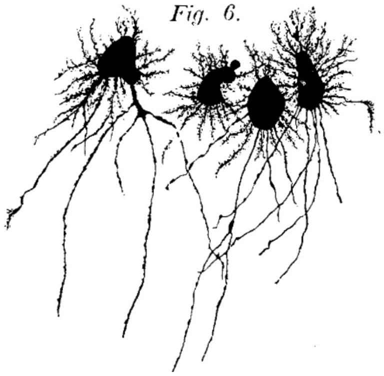

Fig. 6 Table VII. Tangential section of the brain surface at the vertex. From an adult (old) cat. Glial cell bodies with processes positioned at the surface.

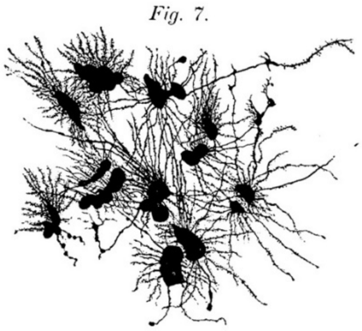

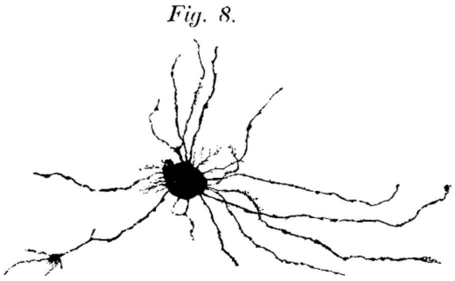

Fig. 7 and 8 Table VII. Tangential section of the brain surface at the vertex. From an adult (old) cat. Glial cell bodies with processes positioned at the surface.

Fig. 9.

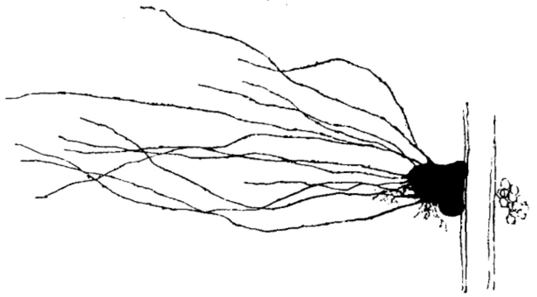

Fig. 9. A glial cell attached to a blood vessel sheath from the cerebral cortex of an adult (old) cat.

Viewed from the surface, the cell bodies of the Schwanzstrahler (Fig. 6, 7, 8) represent similar forms with their processes; one finds peculiar drop-type protrusions, irregularities and constrictions of the end plates (Fig. 7). Such Schwanzstrahler are also present at larger blood vessel which penetrate into the brain tissue (Fig. 9, where the cell is viewed from the side).

At earlier stages, in the first two weeks after birth, at the 15-day-old (Fig. 3), 13-day-old (Fig. 2) and 8-day-old cat (Fig. 1) one finds less differentiated Gliäcytes. One can recognize the main types: some of these elements show embryonic features; this is particularly the case in the 8-day-old animal, but still in the 15-day-old there are forms present which disappear later or are modified. In Fig. 2, I have depicted the outer ends of two ependymal cell fibers, since it is of interest that these elements can be found long after birth in their embryonic form, like in dogs.

Fig. 1.

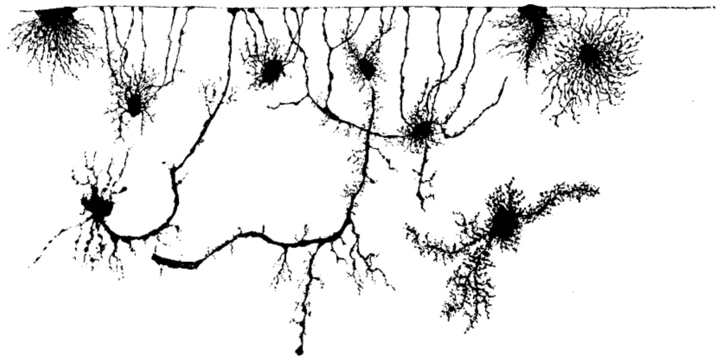

Fig. 1 from Table VII. Vertical section from the vertex of the brain from an 8-day-old cat; the glial cells are of the fetal type.

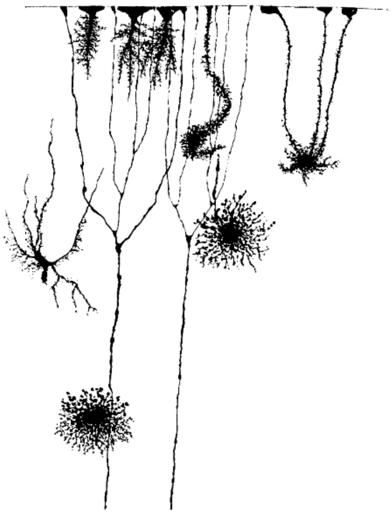

Fig. 2 from Table VII. Vertical section from the vertex of the brain from a 13-day old cat. Besides the glial cells there are the outer edges of two ependymal cells displayed.

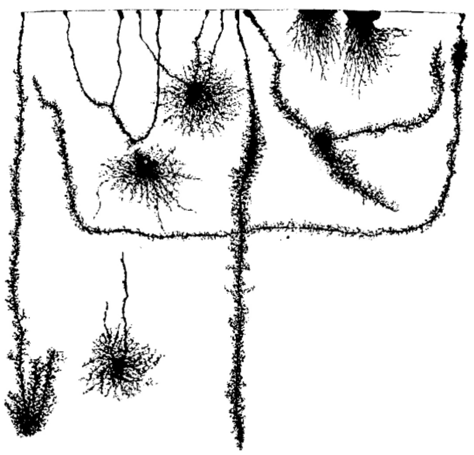

Fig. 3 from Table VII. Vertical section from the vertex of the brain from a 15-day-old cat.

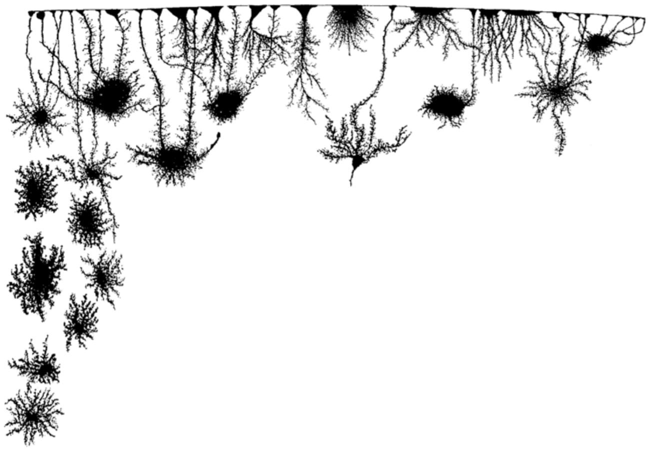

Fig. 1 from Table VIII. Vertical section at the vertex of the cerebrum of a 14-day old rabbit.

In rabbit (Table VIII) the glial cells of the cerebral cortex are generally also similar to those in dog and cat brain. The mossy features are even more pronounced. Also, here one can recognize the types defined in humans, albeit in modified shape. The Sternstrahler maintain their typical appearance; among them only Kurtsternstrahler are depicted (Fig. 1, 4, 7). The Fusssternstrahler are quite abundant in the cortex (Fig. 1, 4) and show interesting variants; sometimes there is only one foot process present; sometimes there are several of different length, depending whether the cell body is more or less deep under the surface. The cell bodies of these cells are often

equipped with a high number of fine, mossy processes extending in all directions, so that the whole looks like a black, mossy cluster which cannot be penetrated by eye (Fig. 1). The Schwanzstrahler which are positioned with their cell

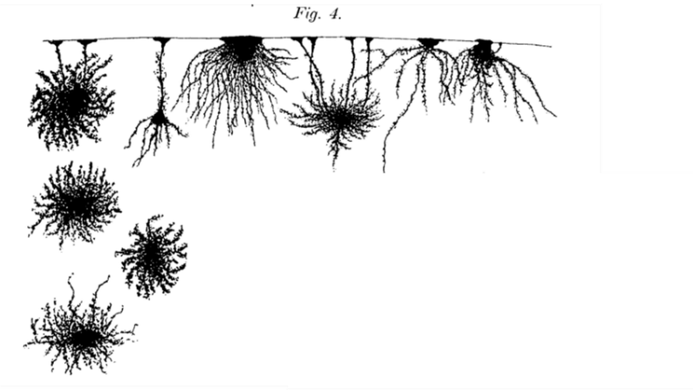

Fig. 4 from Table VIII. Vertical section at the vertex of the cerebrum of an adult (old) rabbit.

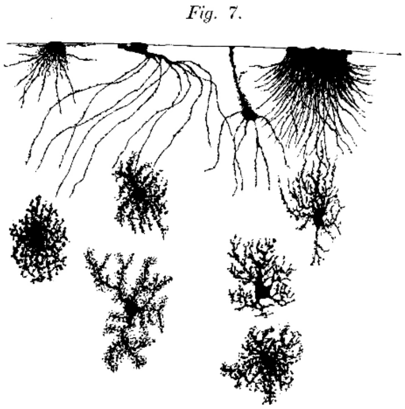

Fig. 7 from Table VIIII. Vertical section from vertex of the cerebrum of an adult (old) cat.

body at the surface, are often quite broad, the processes of the tail are not very long and generally equipped with many little branches. Here are also Schwanzstrahler with a small cell body plate, so that the surface mosaic of these plates together with the intermediate placed feet of the Fussstrahler processes generates a

varied mix of small and large fields. Tangential processes of the Schwanzstrahler are rare (Fig. 7).

At younger stages, at the newborn animal and in the first week after birth, the Gliäcytes exhibit partially still a fetal character. This is particularly the case in the first two days after birth, as shown in Fig. 3 at a 1½-day-old male. Here are Gläcytes which are similar to humans in the later half of the fetal period. At a 14-day-old rabbit (Fig. 1) they are, as described above, fairly mature, as found later at an old animal (Fig. 4 and 7). In Fig. 2 and 3, Cajal cells are shown for comparison.

Fig. 2.

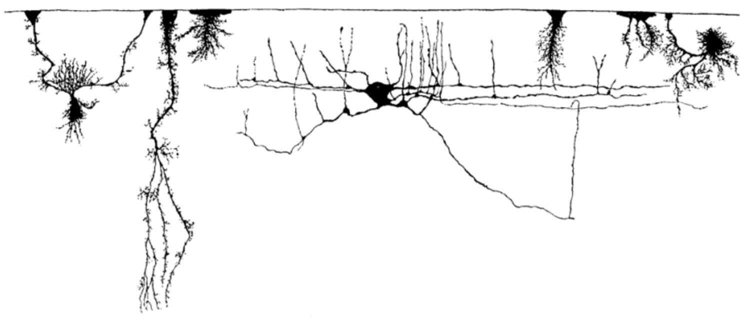

Fig. 2 from Table VIII. Vertical section at the vertex of the cerebrum of a 4-day old rabbit. A Cajal cell is depicted surrounded by glial cells.

Fig. 3.

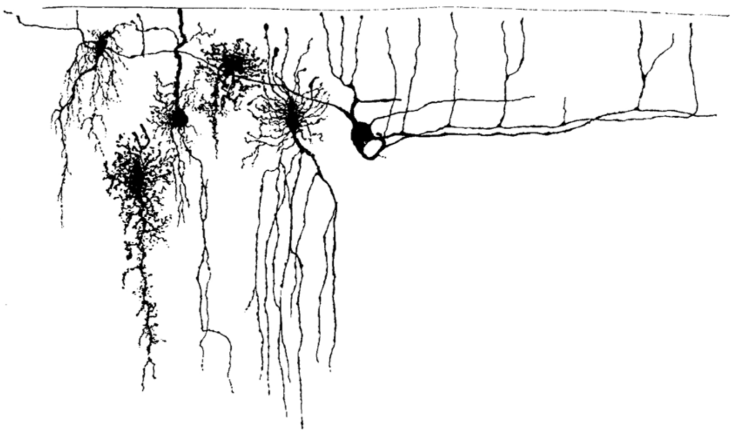

Fig. 3 from Table VIII. Vertical section at the frontal end of the cerebrum of a 1 ½ day old rabbit. To the right of the group of glial cells is a Cajal cell shown.

C. The neuroglia of the cerebral ganglia, the medulla oblongata and the insula Reilii of human

(Table IX)

Subsequent to the presentation of neuroglia in the cerebral cortex it is of particular interest to study the parts of the brain which deviate in their structure. It is not the intention of this work to provide a comprehensive study, but rather give an overview of the represented glial elements. I will therefore restrict myself to report on the types present in the larger ganglia of the cerebrum and further to describe those on the insula Reilii of the cortex.

Fig. 1.

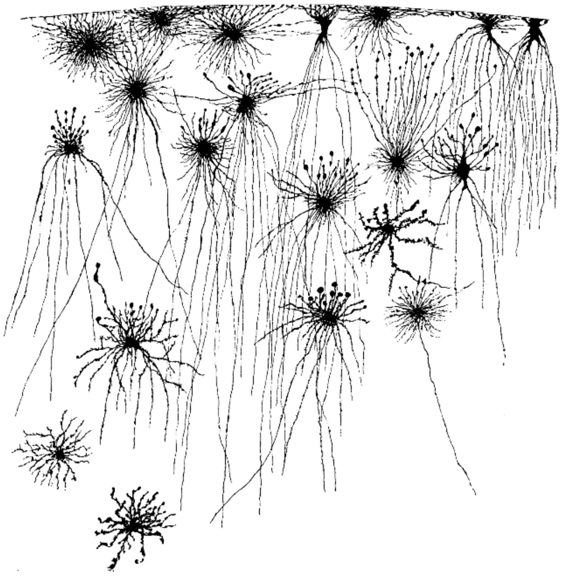

Fig.1 from Table IX. Frontal vertical section of the cortical part of the Corpus quadrigeminum anterius from a 42 cm long human fetus.

I start with the Corpora quadrigemina. In Fig. 1 Table IX I have illustrated a vertical frontal section of the Corpora quadrigeminus anterius of a 12 cm long human fetus. One finds the types described above in the cortical hemisphere. At the lower part of the figure are Sternstrahler depicted, in particular Kurzsternstrahler. At the surface of this part of the brain one can observe the cell bodies of Schwanzstrahler which are located close to the surface, sometimes slightly below sending a number of short cone-type, diverging processes to the surface, which partially extend at the surface layer and intermingle. These Schwanzstrahler send sometimes shorter, sometimes longer, even very long bundles of processes downward (to the interior); here are both Kurzschwanzstrahler and Langschwanzstrahler present. Slightly deeper below the surface are Schwanzstrahler which do not reach the surface with their processes. Transition forms between Schwanzstrahler and Sternstrahler are also present. The processes extending to the surface are commonly equipped with knots.

In the Corpora quadrigemina posteria are the same types of Gliäcytes present.

In the examination of the cortical layers of the Thalami optici one finds the above-described types of glial elements. In Fig. 2, I have provided a vertical frontal section of the putamen. Sternstrahler are present in the inner part and Schwanzstrahler are with their cell body at or close to the surface, sending their long tails deep into the interior. Here

one can observe (on the right in the Figure) a Fusssternstrahler, which sends the outer processes up to the surface and at the same time a tail bundle to the interior, thus a transition between a Fusssternstrahler and a Schwanzstrahler.

Fig. 2.

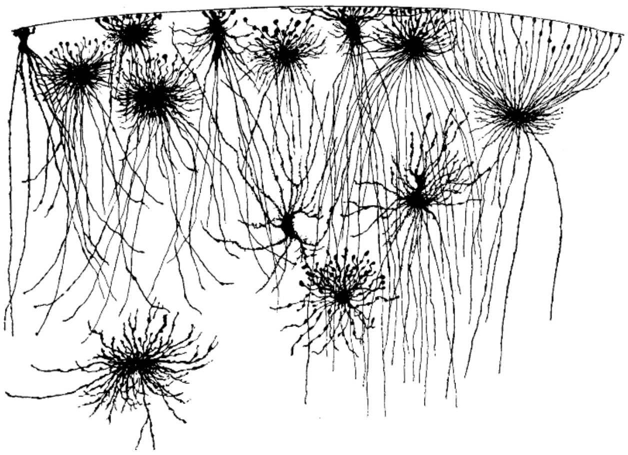

Fig.2 from Table IX. Frontal vertical section of the cortical part of the Putamen thalami optici from a 42 cm long human fetus.

From the Corpora striata I have not obtained good preparations and I provide as comparison only a small section (Fig. 3) of a vertical section from the brain of an old (65-year) man which provides some of the glial cells in this preparation. The Sternstrahler in the interior are well developed; the elements at the surface with their stiffly projecting processes are peculiar and require further investigation.

Moreover, I have provided, for comparison, on the same Table (IX) a cortical cross-section of the medulla oblongata of a human fetus in the 7th month to illustrate the important types of glial cells in this brain region. As actually assumed, these elements are in particular similar to those in the spinal cord, as reported previously by me and M. von Lenhossek. In the grey areas are Kurzsternstrahler, in the white Langsternstrahler presen

Fig. 3.

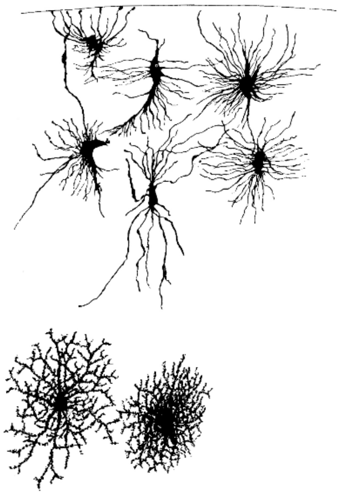

Fig. 3 from Table IX. Vertical section of the surface area from Corpus striatum of a 65-year-old man.

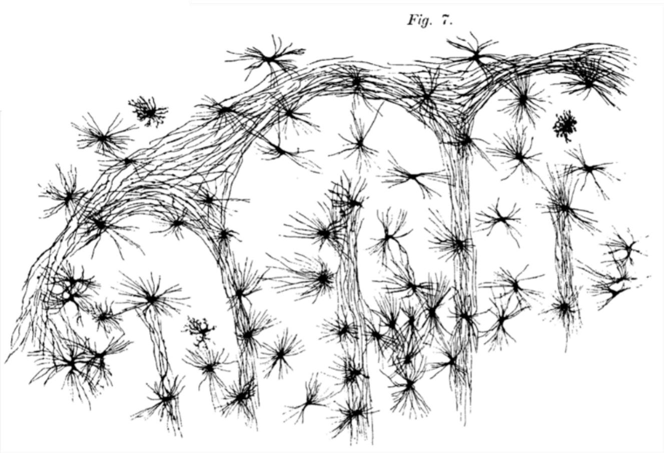

Fig. 7 from Table X. Vertical cross section of the olive from an 8 months old human fetus. Due to lack of space in Table IX, this Figure is placed here.

and at the surface or slightly below are the cell bodies of numerous Schwanzstrahler which send short processes to the surface and long tail bundles to the center. With strong staining, one finds a dense network of cell bodies and fibers, which is difficult to differentiate as evident in the upper part of the figure. Despite off the glial cells of the cortex and the center of the medulla oblongata are similar to those of the spinal cord, there are also similarities to those in the brain ganglia or the cerebral hemispheres.

In cross-sections of the medulla oblongata of a human fetus one obtains often good staining of the olives. Since it seems to me of interest to demonstrate also the glial cells of this part of the brain, I provide in Table X an image from there at lower magnification (Fig. 7). As shown in this figure, there are almost only Langstrahler (Schlichtstrahler) present; yet one can observe below also single Kurzstrahler (Krausstrahler).

From the other parts of the human brain, I will briefly mention only the cortex of the insula Reilii with the intention to demonstrate that there are genuine cortical conditions with respect to the glial elements. I have selected a figure (Fig. 5, Table IX) of the fetal insula to illustrate the developing glial cell forms in this region. In comparison with illustrations from the other cortical hemispheres, one can easily find matches of the glial cell forms; moreover, here one can recognize an attachment of a glial cell process to a blood vessel with a large knot and a number of tangential processes with vertical ascending branches; no Cajal cells could be recognized in this field of view.

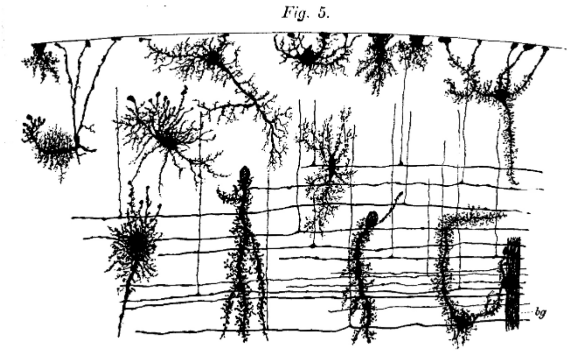

Fig. 5 from Table IX. Vertical section of the Insula Reilii of a 39 cm long human fetus – bg, a blood vessel with an attachment of a glial cell process.

D. The neuroglia of the brain ganglia, of the gyrus hippocampi and the fornix of the cat (Table X, Figs. 1 - 6).

Since it is of interest for the conditions of humans to compare the relationships of other mammals, I have included investigations on the cerebral ganglia of the cat and I provide illustrations of vertical frontal sections of these ganglia in Table X. Fig. 1 shows the upper part of the Corpus quadrigeminus anterius, Fig. 2 of the corpus quadrigeminus posterius, Fig. 3 of the corpus geniculatum of two-week old cats. In Fig. 1 and 2, one can recognize almost everywhere the main

Fig. 1.

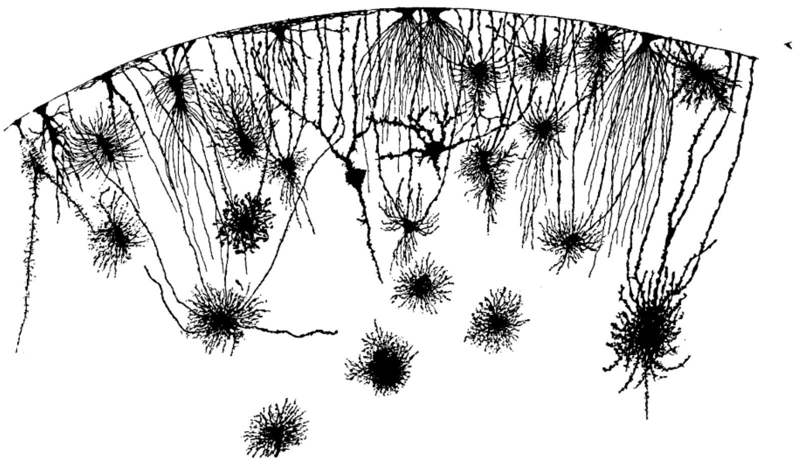

Fig. 1 from Table X. Frontal vertical section of the Corpus quadrigeminum anterius of a 14-day old cat.

Fig. 2.

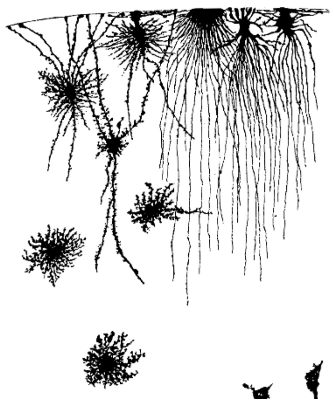

Fig. 2 from Table X. Frontal vertical section of the Corpus quadrigeminum posterius of a 14-day old cat.

Fig. 3.

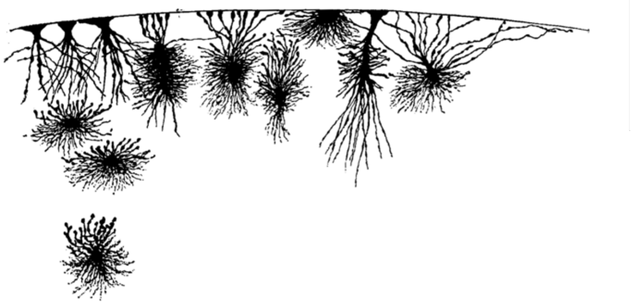

Fig. 3 from Table X. Frontal vertical section of the Corpus geniculatum of a 14-day old cat.

types of glial cells, namely in the interior the Kurzstrahler, close to the surface, on the other hand, the Schwanzstrahler which show short as well as long tail bundles and partially also tangential extensions of the upper processes. Moreover, there are differently formed Fusssternstrahler present, some showing particularly long (in particular in Fig. 1) foot processes.

Fig. 3 illustrates the glial elements of the corpus geniculatum, including Kurzsternstrahler, Fusssternstrahler, and Schwanzstrahler. The latter have only short tail bundles and do not exhibit their usual, characteristic type. To complement these figures from the ganglia, I include illustrations of the gyrus hippocampi of the cat to ensure important areas of the brain are not omitted. Regarding the gyrus hippocampi and the fascia dentata, Ramon y Cajal has already provided excellent illustrations of the glial elements, so I will not focus on these, as his depictions are more comprehensive.

What I wish to highlight, however, is the glial tissue in the lower part of the gyrus hippocampi, which I illustrate in Fig. 4 as part of a vertical section. In this figure, one can observe the partially peculiarly formed Kurzsternstrahler and Langschwanzstrahler in various forms, including those located deep below the surface that project long processes, some of which only partially reach the surface. Finally, I include Fig. 6, which presents a cross-section of the fornix, illustrating glial cells with unusual formations. To some extent, these elements can be associated with the main types mentioned earlier, although they appear somewhat atrophied.

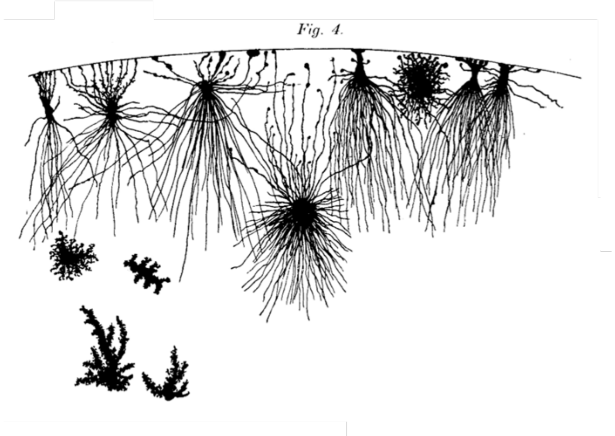

Fig. 4 from Table X. Vertical section from the lower area of the Gyrus hippocampi of a 14-day old cat.

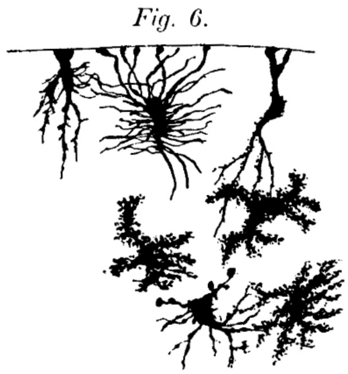

Fig. 6 from Table X. Vertical section of the Fornix of a 14-day old cat.

II. The neuroglia of the cerebellum

(Table XI)

A. The neuroglia of the human cerebellum

In the historical presentation above on the neuroglia of the cerebrum, which includes the findings of recent researchers working with the Golgi method, I have also incorporated information about the neuroglia of the cerebellum. The fibers discovered by Bergmann, and subsequently named after him, were verified by other researchers as well as by me and Key, who provided an intensive description in 1875. Additionally, the presence of glial cells in the white matter of the cerebellum was recognized early on.

The advancement of precise knowledge regarding the morphology of these cells and the significance of the Bergmann fibers coincides with the introduction of the Golgi chrome-potassium-silver method. Golgi confirmed the bundle-like origin of the fibers from cell bodies positioned at the inner border of the molecular layer and also described the star-shaped glial cells of the white matter (see his referenced publication, Table XII, as well as Table 16 of the German summary edition, 1894). Since then, several researchers, including Ramon y Cajal, Van Gehuchten, and von Kölliker, have contributed to this area of study. I have illustrated the properties of the Bergmann fiber cells twice: first in humans in 1891 and later in cats in 1892. Most recently, this year, Van Gehuchten provided an extensive illustration of these cells in the human cerebellum.

Although we now possess the most relevant data regarding the neuroglial elements of the cerebellum, I will briefly add my results, obtained during the previous year, along with some illustrations. I do this partly because a comprehensive overview of the neuroglial elements of the brain must include the cerebellum, and partly because I have studied the developmental stages of these elements (in both early and fully developed stages) in humans. Additionally, the enigmatic granules at their outer ends still require further description.

A. The neuroglia of the human cerebellum (Table XI)

In the last half of the fetal period, the glial elements of the cerebellar cortex can be labelled. The Bergmann fiber cells show a less characteristic feature as later. In the 7th and 8th month of the fetal live the cell bodies are roundish-oval, as

Fig. 1.

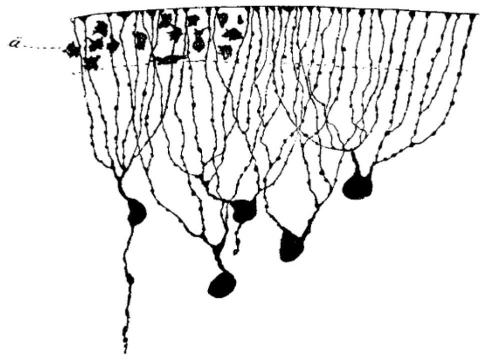

Fig. 1 from Table XI. Vertical section of the cerebellar cortex of a 37 cm long human fetus, with five Bergmann fiber cells; - ä. Vignal cell layer.

clumps positioned at different levels which show a lower extending process; from the outer part there originate one or two processes which diverge in a pointed angle and divide dichotomic multiple times extending to the outer surface. In this way there are fibers generated which are partially slim and less branched, conic-formed, partially highly branched, or partially wider, extended bundles of fibers; the latter are like string of pearl-shaped and delicate, becoming parallel when extending to the surface where they end with conic enlarged feet. The cells obtain already in the 8th month (Fig. 1 of Table XI) that typical type; they are less uniformly shaped than later, as the fibers do not yet have the nice, straight, parallel course; the cell bodies have either no or few lateral and inner processes, which determine their later unusual appearance. Many cell bodies are deeply inserted into the granule cell layers and run through the the Purkinje cell layer which does not contain the thick, so-called molecular layer and the relatively strong layer of the outer granules.

As in the cerebral cortex, the term "molecular layer" is no longer appropriate now that we have gained more detailed knowledge of the structure of the cerebellar cortex. I find it more suitable to refer to this layer as the "layer of basket cells" or "basket cell layer" due to the presence of the characteristic nerve cells known as basket cells[1]. The outer layer, which consists of embryonic granule cells first described by Vignal, could be termed the "Vignal cell layer" or simply the "Vignal layer," to avoid confusion with the internal granule cell layer that contains true nerve cells.

In contrast to authors who oppose the use of personal names, I find such names practical and beneficial, particularly when alternative terms are difficult to establish. The names of researchers do not carry implications regarding structure or function and, therefore, do not become obsolete or misleading (unlike, for instance, the term "molecular layer" in the cerebral cortex). Moreover, these terms are tied to the history of our science, often honoring the discoverer. Such terms, however, should be used sparingly.

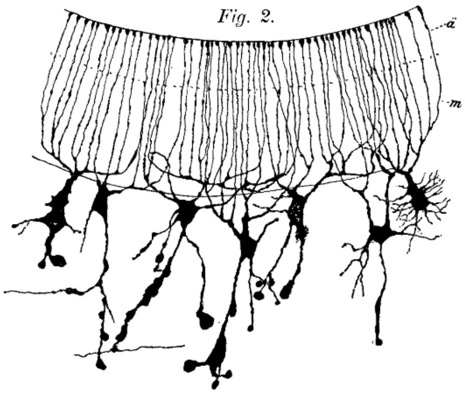

Fig. 2 from Table XI. Vertical section of the cerebellar cortex of a 45 cm long human fetus, with seven Bergmann fiber cells; - ä. Vignal cell layer; - m granule cell layer.

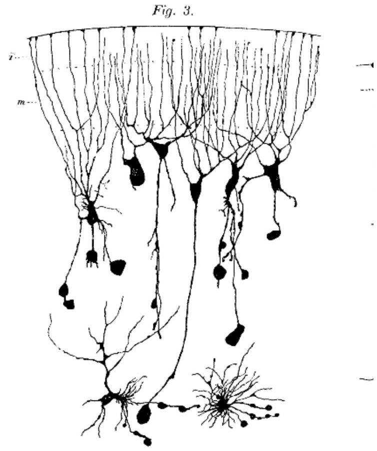

Fig. 3 from Table XI. Vertical section of the cerebellar cortex of a 48 cm long human fetus, with six Bergmann fiber cells and two Sternstrahler in early stage of development; - ä. Vignal cell layer; - m, basket cell layer.

With respect to the Bergmann fiber cells, they change in the later fetal stage. At a 45 cm long fetus (Fig. 2 Table XI) one can observe the cell bodies which have become angled and being at differently deep positions in the granule cell layer; they have several, often long, strangely formed processes which originate from the lower (inner) section of the cell body and extend into deeper layers or laterally. These ´inner´ processes which have been depicted by Cajal, me and Van Gehuchten, have a strange character being often coarse and often occupied with round, oval or angled enlargements; they often end in a large knot which can even be larger than the cell body proper. Fig. 2 and Fig. 3 on Table XI (from a new-born 48 cm long fetus) contain several variants of these inner processes, which all are positioned in the granule cell layer and extend deeply into it. Here and there originate delicate processes from the cell body which can be quite

numerous (Fig. 2, right, Fig. 3, left). With respect to the outer processes and the Bergmann fibers, they have less of a string of pearl property and are more arranged in parallel.

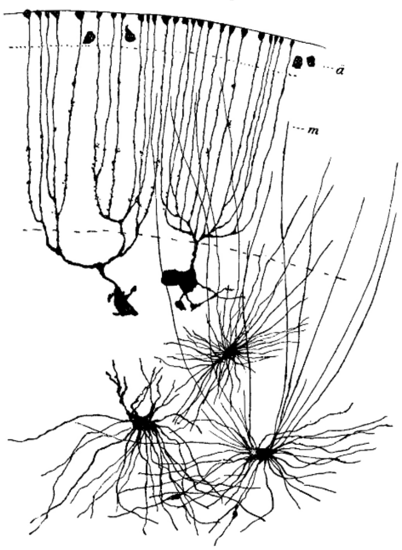

Fig. 4 from Table XI. Vertical section of the cerebellar cortex of a 2-month-old child, with two Bergmann fiber cells and three Langsternstrahler; - ä. Vignal cell layer; - m, basket cell layer

From the subsequent developmental stages, I will select one from a 3-month-old child and of an adult; at the 3-month-old child (Fig. 4 of Table XI), the inner processes are reduced; this is not complete, but they have less of an extend. The cell body are continuously angled and equipped with shorter protrusions; they are in the surround, often below (to the interior) of the Purkinje cells. To the upper (outer) region they send one, sometimes two or even three processes, which ascend to the basket cell layer and rapidly branch, multiply and dichotomic; the first, coarse processes bend to the side similar as the Purkinje cells, and there the more or less multiple Bergmann fibers originate as fine, equally thick threads only occasionally equipped with small knots or granular appearing branches; They extend vertical to the surface in a parallel fashion, less wiggled where they end with a quite substantial roundish, conic foot or knot after passing the still existing, but thinner Vignal layer.

In the adult stage (Fig. 5 of Table XI from a 33-year old man) one can recognize the angled, knobbly, even lobed cell bodies of these Gliäcytes, internal of the basket cell layer and below the Purkinje cells which continuously send single, coarse und more delicate processes to the interior and the sides. The processes ascending into the basket cell layer retain their previous properties and their branches extending upward (to the outer), namely the Bergmann fibers are straighter and more parallel and end at the surface with a conic knot. The Vignal cell layer has long disappeared, but the granule cell layer has become much thicker in the adult, as Figures 2, 4 and 5 show at the same magnification. As a result, the Bergmann fibers have grown in length.$

Fig. 5.

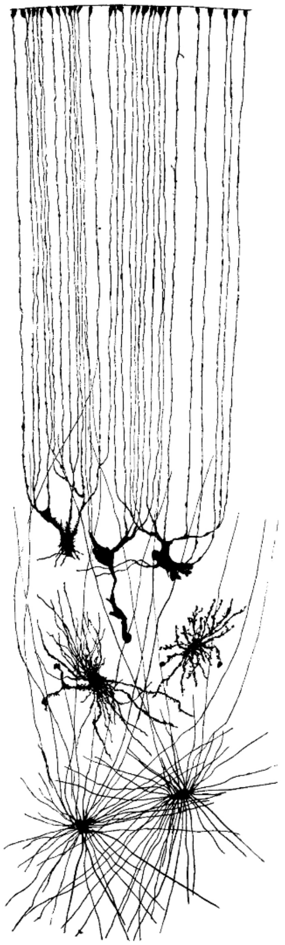

Fig. 5 from Table XI. Vertical section of the cerebellar cortex of a 33-year-old man, with three Bergmann fiber cells, two Kurzsternstrahler and two Langsternstrahler.

[1]This layer could also be named based on the longitudinal and tangential fibers (or after the dendrites of the Purkinje cells which comprise the major parts. I find it, however, more consistent to name them after the nerve cells.

They form similar to the outer ends of the ependymal cells of the cerebral cortex (and the vertical branches of the Cajal cells) a rich, parallel fibered palisade of grid formation, which serves as an important support structure of the cerebellar cortex.

To which type do these Gliäcytes correspond? According to their shape they correspond best to the Schwanzstrahler, despite off their body is oriented below (to the interior) and their fiber tail upward (to the outer) in contrast to the corresponding of the cerebral cortex.

Besides these Schwanzstrahler, there are only Sternstrahler in the cerebellum, both in the granule cell layer as well as in the white matter. The majority of the cells, depicted by Golgi, Cajal, Von Gehuchten and von Kölliker, are Langsternstrahler of the type as shown in Fig. 4 and Fig. 5; these elements often have long, stretched and simple processes which extend into the basket cell layer passing the Purkinje cells, as I found (Fig. 4 and Fig. 5 of Table XI). Moreover, there are other Sternstrahler with shorter processes of a knot-curly appearance (Fig. 5); these belong to the granule cell layer and should be named Kurzsternstrahler. In earlier stages (Fig. 3 of Table XI from a new-born child) their processes are less numerous, partially like a string of pearls and have a more embryonic character.

B. The neuroglia of the cat cerebellum (Table XI, Figs. 6, 7, 8, 10, 11, 12)

Among other mammals, I have predominantly studied neuroglia in cats, dogs, rabbits, and mice. For the following description, I have selected the first-mentioned animal. In unborn cat embryos (Fig. 6), the Bergmann fiber cells exhibit a mossy, branched cell body positioned within the granule cell layer, with some longer processes projecting downward and laterally, and one or two projecting upward. The upward-extending processes are stronger, mossy in appearance, and display sparse dichotomic branching. These processes pass through the Purkinje cell layer, the relatively thin basket cell layer, and the still-thick Vignal layer in a stretched formation, terminating with conic feet at the surface. In the granule cell layer, the fibers exhibit small lateral branches and spikes, which become rarer in the Vignal layer.

In a 5-day-old cat (Fig. 8), the mossy appearance of the cell bodies is quite pronounced, and they are positioned deep within the granule cell layer, often with highly branched processes. The upward-extending processes do not differ significantly in features. I provide an image of the neuroglia of a 3-day-old cat (Fig. 7) in which two of these cells are visible. Additionally, I include images of a 14-day-old cat (Fig. 9) and a 22-day-old cat (Fig. 11). It can be observed that the cell bodies partially lose their mossy appearance, appearing angled and jagged. Moreover, the Bergmann fiber

processes elongate in parallel with the thickening of the basket cell layer, extending more parallel and stretched. They develop small side spikes within this layer, although such spikes are rare in the Vignal layer, which becomes thinner. These described features persist into adulthood after the basket cell layer has reached its final thickness and the Vignal layer has disappeared.

Regarding the Sternstrahler, one can observe, among the cells in the granule cell layer both before and soon after birth, a relationship to the Schwanzstrahler described above. This is due to the fact that they often exhibit a strong upward-extending process, which terminates after a shorter or longer course without reaching the basket cell layer (Fig. 8). At a later stage (Figs. 9, 11), the cells acquire the typical curly appearance of Kurzsternstrahler. The Gliäcytes in the white matter (Fig. 12) can be more accurately classified as Langsternstrahler.

It is not my intention to focus on formations of the cerebellar cortex other than the true Gliäcytes. However, I must briefly mention the cellular elements first described by Vignal and later by Schwalbe—the so-called outer granules—which are present during fetal development and for a certain period after birth.

Fig. 6.

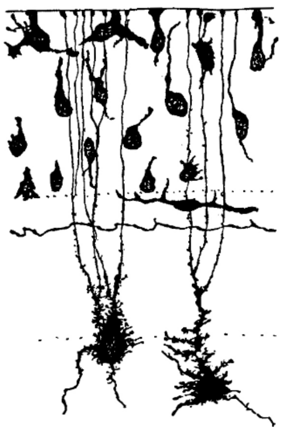

Fig.6 from Table XI. Vertical section of the cerebellar cortex of a not yet delivered cat fetus with two Bergmann fiber cells; - ä. Vignal cell layer; - m, basket cell layer.

Fig. 7.

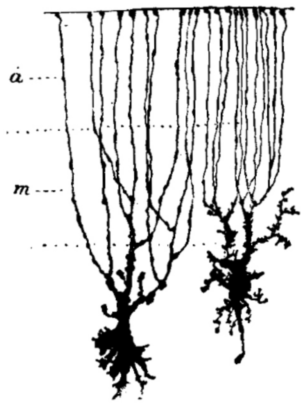

Fig.7 from Table XI. Vertical section of the cerebellar cortex of a 3 day old cat, with two Bergmann fiber cells; - ä. Vignal cell layer; - m, basket cell layer.

Fig. 8.

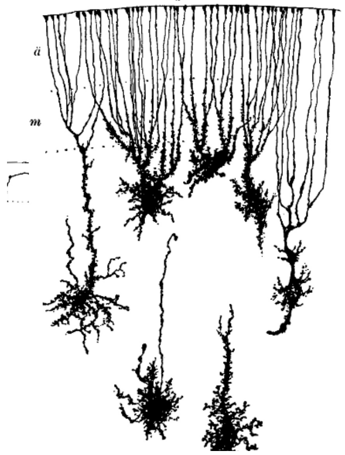

Fig. 8 from Table XI. Vertical section of the cerebellar cortex of a 5 ½ day old cat, with six Bergmann fiber cells and two Kurzsternstrahler; - ä. Vignal cell layer; - m, basket cell layer.

Fig. 9.

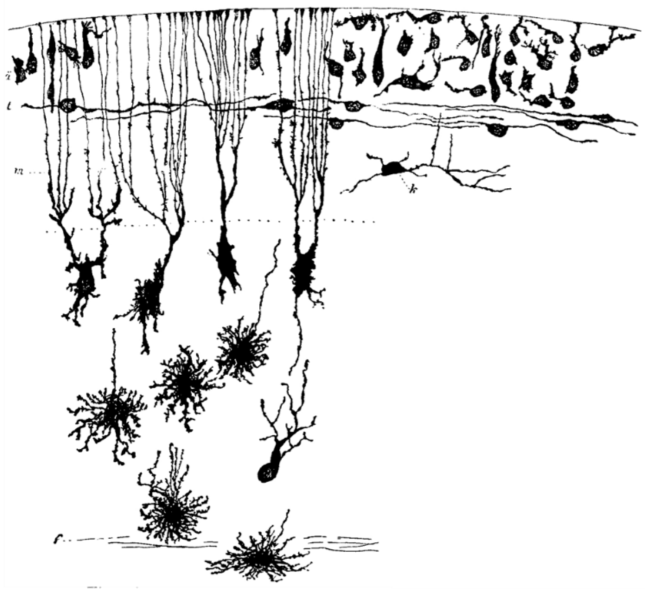

Fig. 9 from Table XI. Vertical section of the cerebellar cortex of a 14-day old cat, with four Bergmann fiber cells and five Kurzsternstrahler and a cell with unclear identification; - ä. Vignal cell layer; - t, tangential, bipolar cells of this layer; - m, basket cell layer with a basket cell (k); - f, white matter.

Fig. 10.

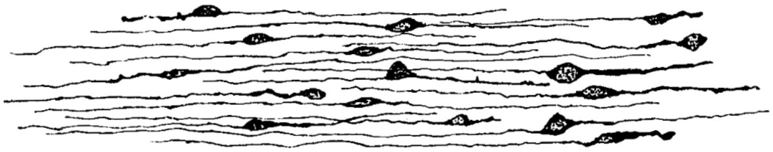

Fig. 10 from Table XI. Part of a tangential section of the cerebellar cortex of an 11-day old cat with a number of tangential bipolar elements (lower part of the Vignal layer).

Fig. 11.

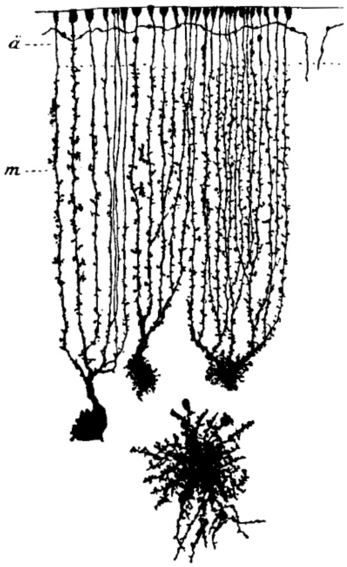

Fig. 11 from Table XI. Vertical section of the cerebellar cortex of a 22-day old cat, with three Bergmann fiber cells and one Kurzsternstrahler; - ä. Vignal cell layer with strange, tangential running fibers; - m, basket cell layer.

Fig. 12.

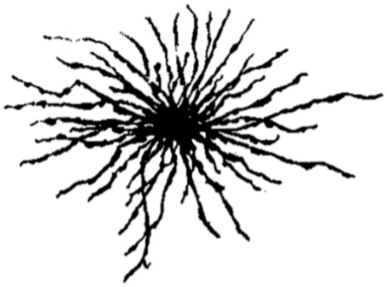

Fig. 12 from Table XI. One Langsternstrahler from the white matter of the cerebellar cortex of a 22-day old cat.

At later times, they were illustrated in detail by Ramon y Cajal[1], who described an outer (upper) layer consisting of vertically oriented, elongated cells, and an inner (lower) layer containing horizontally (tangentially) positioned bipolar cells. Furthermore, he described and illustrated vertical bipolar cells in the molecular layer that extend a process to the inner layer of bipolar granule cells, which appear to be neural and attach to a longitudinal fiber in that layer. Transitional forms between cells resembling inner granule cells and horizontal bipolar cells were also observed, but these were too rare to establish a genetic connection between them.

Von Kölliker[2] emphasized that these ascending elements are not found in adults and that, since proper granule cells are already present in young creatures, they cannot represent developmental stages. In the French edition of his comprehensive publication, Ramon y Cajal[3] highlighted that the granule cells of the cerebellar cortex (those in the internal granule cell layer) originate from bipolar elongated cells in the lower part of the Vignal layer, which extend downward and descend. Recently, Lugaro[4] also addressed this issue and provided further insights.

The outer granule cell layer is composed of two sub-layers containing morphologically distinct elements that exhibit transitional forms. The outer sub-layer consists of roundish, pear-shaped elements with a process directed toward the surface. These elements sometimes display small, irregular processes, giving them an epithelioid, embryonic appearance. The deeper sub-layer contains bipolar cells with oval bodies that extend two relatively long processes running parallel to the surface and following its contours. These processes are thinner and elongated according to the depth of the cells, and their ends feature a swelling, referred to as the augmentation cone. At the boundary between the superficial and deeper layers, transitional formations can be observed where the epithelioid elements develop two processes, marking their transition into bipolar cells. This transition progresses from the inner to the outer layer. The bipolar elements at the inner border of the deeper layer project their bodies inward and extend a process into the molecular layer. Transitional forms of these inner granule elements are present.

According to Lugaro, it can be concluded that granule cells form through the continuous transition of epithelioid into horizontal elements, a process accompanied by the migration of cell bodies from the surface to deeper layers.

I have described this extensively because, as Cajal first suggested, the explanation surrounding the Vignal cells is now clearer and contains compelling arguments. However, I have reservations and align with Kölliker's observations. Over the past few years, I have focused on these cells during my studies of the cerebellar cortex. Using the Golgi method, I often obtained staining of these cells in humans, rabbits, and cats. I illustrate some of their various forms but can only provide a limited number due to space constraints.

In a fetus, I found multiple cell forms within the still relatively thick layer (Fig. 6 of Table XI), including roundish, oval, cylindrical, unipolar, bipolar, and multipolar cells, arranged in an unorganized mix. It should be noted that there are cells with one or two variably long processes oriented straight or inclined toward the surface. Among these are elements with diverse forms and orientations. Most cells possess a thin protoplasm surrounding an oval or round nucleus, with more prominent protoplasm at the sides of the cells. Indeed, they resemble lymphoid cells or certain epithelioid formations.

[1] S. Ramon y Cajal, A propos de certains elements bipolaires du cervelet avec quelques details noveaux sur l´evolution des fibres cereblleuses. Inter. Monatsschr. F. Anat. U. Phys., Bd 7, 1890.
[2] A. von Kölliker, Handbuch der Gewebelehre des Menschen. 6. Auf., II Bd, 1, 1893.
[3] S. Ramon y Cajal, Les novelles idees sur la structure du systeme nerveux chez l´homme et les vertebres. Paris 1894.
[4] Ernst Lugaro, Über die Histogenese der Körner der Kleinhirnrinde. Anatom. Anzeiger, Bd 9, No 23, Aug. 1894.

In a fetus, I observed only the various types of cells as described earlier, but no separation into two distinct layers. During the first week after birth, I occasionally succeeded in identifying the lower (inner) layer of cells, particularly in the form and arrangement first described by Cajal and later by Lugaro. In Fig. 9 (at t) of Table XI, multiple bipolar cells are displayed in a vertical slice, and in Fig. 10, a horizontal slice of the cerebellar cortex in a 14-day-old cat. It cannot be denied that these elements bear a strong resemblance to embryonic, maturing nerve cells with delicate axons and thicker protoplasmic processes. However, I did not observe true transitional forms between the cells of the outer and inner layers, as described by Cajal and Lugaro. The inner layer of bipolar cells was observed only occasionally and not throughout the cortex. Furthermore, I must admit that I did not succeed in identifying the vertically descending bipolar cells described by Cajal and Lugaro, despite examining numerous preparations. Either these cells are particularly difficult to stain, or they are rare and localized to specific regions. I do not deny the existence of this particular cell type, as it has been described by such meticulous researchers as Cajal and Lugaro, who worked in Mondino's laboratory. Cajal himself emphasized the rarity of transitional forms to the inner granule cells. Indeed, it would be peculiar for nerve cells to remain in an embryonic stage so late in life, even months after birth, especially as von Kölliker noted that the internal granule cells are already present and well-developed in their organization. On the other hand, it is worth noting that the basket cell layer (molecular layer), which primarily consists of longitudinal fibers from the inner granule cells, is very thin during the fetal stage and shortly after birth, growing only gradually. This could indicate the addition of new cell processes. I attempted to determine whether the cells of the basket cell layer are augmented by contributions from the Vignal cell layer, but I found no evidence to support this hypothesis. Regarding this interesting question, it remains unresolved, and I remain skeptical. Before concluding on this matter, I should mention an important observation I made last winter that is relevant to this question. Given the embryonic nature of the Vignal layer, I sought evidence of cell division using conventional methods. Indeed, I observed mitotic cell divisions not only in the fetal cortex but also for some time after birth. These divisions were still numerous in a two-week-old cat, occurring not only at the surface but also in the middle of the layer. At later stages, I observed signs of chromatin dissolution in the nuclei of some cells. This observation requires further investigation. One additional finding warrants brief mention. In the Vignal layer of young cats and rabbits, I labeled various tangentially running fibers using the Golgi method. These fibers, though not particularly delicate, resemble nerve fibers. I succeeded in tracing them over large distances in cross-sections of the gyri. They do not branch but extend singularly into the middle of the layer. However, I could not trace them from their origin to their endpoint. On one occasion, as shown in Fig. 11 of Table XI, I observed these fibers diving into the basket cell layer. The nature and function of these fibers remain unclear to me.

III. The Neuroglia of the pituitary gland of mammals

(Table XII)

It is not my intention to provide a complete illustration or even a detailed account of the development of this peculiar and rudimentary organ commonly referred to as the pituitary gland. Firstly, the epithelial (frontal) part does not belong to the brain, and secondly, an exhaustive description of the posterior part connected to the central organ (the posterior lobe of the pituitary, also referred to as the *Processus seu Lobus infundibuli*) would require a specialized and extensive investigation. Such an endeavor would necessitate studying a range of animal species, encompassing both the adult structure and its histogenesis, which is beyond the scope of this publication. Here, I intend to discuss only the fundamental elements of the mammalian pituitary gland, particularly the distribution and forms of the glial tissue, as revealed by the Golgi method.

Upon studying the pituitary in larger textbooks or other literature, one quickly recognizes that little is known about the neural component of this organ. The descriptions are often brief, ambiguous, and, in some respects, incorrect. This is understandable, as the methods previously applied did not yield precise results. Only the more modern techniques provide the accuracy required for a deeper understanding of the structure of this organ.

In his textbook on neurology, Schwalbe[1] notes, after discussing the significant regression of this originally hollow organ in higher vertebrates, particularly in mammals and humans: “Its cavity usually disappears, leaving only the thin connecting shaft, the infundibulum. From the large *lobus infundibuli* of lower vertebrates, only a relatively small solid *processus infundibuli*, the so-called posterior lobe of the pituitary, remains. At the same time, its nervous elements undergo atrophy due to the numerous infiltrations of blood vessels and epithelial tissue. Thus, the *processus infundibuli* of the adult consists of fibrillary connective tissue, rich in round and spindle-shaped cells, and partially containing ramified cells. The fiber bundles cross in various directions, resembling the tissue of a spindle sarcoma (W. Müller).”

Below the round and spindle-shaped cells are larger ones which contain yellow pigment granules in their protoplasm. According to Toldt[2], the processus infundibuli consists of fibrillary, strongly vascularized connective tissue with multiple spindle-shaped or multi-projecting cells; some appear yellow pigmented and remind on ganglion cells.

Schäfer[3] states similarly: the cave is clogged, and any nervous structure is obscured by the invasion of blood vessels and connective tissue into the now solid organ. The connective tissue forms netting bundles in which multiple spindle-shaped and branched cells occur, some with larger cell bodies which contain pigment granules in their protoplasm.

In the other, newer and larger textbooks I have found similar statements. I will therefore only refer to one book. Rauber[4] states that the Hypophysis cerebri consists in its smaller, posterior, cerebral lobe out of sparse nerve fibers, many cells which are similar to bipolar or multipolar nerve cells and out of connective tissue and blood vessels.

Among the recent researchers dealing with the structure of the pituitary, predominantly Lothringer[5] should be highlighted. He studied this organ in humans and in different mammals (dog, cat, horse, pig, rabbit).

[1] G. Schwalbe, Lehrbuch der Neurologie, 2. Bd, 2. Abth. Von Hoffmann´s Lehrb. D. Anat. D. Menschen, 1881.
[2] C. Toldt, Lehrbuch der Gewebelehre, 3. Aufl., 1888.
[3] E. A. Schäfer, Quain´s Elements of Anatomy, Vol. 3, P. 1; 10. Edition, 1893.
[4] A. Rauber, Lehrbuch der Anatomie des Menschen, 4. Aufl., II, 2, I, 1894.
[5] S. Lothringer, Untersuchungen an der Hypophyse einiger Säugetiere und des Menschen. Archiv f. Mikrosk. Anatomie, Bd 28, 1886.

In most detail, Lothringer describes the relationship in the dog, focusing less on the brain part (processus infundibuli or the posterior pituitary) than the epithelial part. The former is inserted into a cup-shaped depression of the latter, and each contains a cavity; when the brain part is disconnected from the epithelial part, a closely attached epithelial seam follows the latter, resulting in a gap-like cavity upon separation. This epithelial seam consists of an epithelial layer made up of multiple cell rows, which is thicker at the rim and contains several small cystic spaces.

"On the tissue of the brain part, we have not conducted special studies," states Lothringer. The applied method was not suitable for studying these complex relationships. This tissue mainly consists of acute-angled penetrating fiber strands. The locations of narrow connective tissue septa, inserted from the surface, form the basis for loose tissue masses found in these intermediate spaces. When the first-mentioned fiber strands appear in larger masses, they resemble the appearance of a spindle cell sarcoma, as described by Schwalbe and W. Müller. Long, stretched nuclei resemble smooth muscle fibers, while multiple round cells and polygonal or star-shaped cells are embedded in the fiber strands. The loose tissue in the intermediate spaces consists of star-shaped, branched cells connected by processes, which most closely resemble the glial tissue found in brain areas with sparse nerve cells. The separation between the two elements, fiber strands and mixed tissue of glial nests, is not distinct. Preparations treated with Weigert's hematoxylin staining show the glial nests as granulated filling masses in the intermediate spaces between the fiber strands. Only in thin preparations can the nuclei of the glial cells be easily identified. The transition of the tuber cinereum into the cone (posterior) lobe occurs at the level of the envelope part. The grey matter of the tuber inserts as a descending, narrowing seam of cell-poor cortical tissue between the epithelial part of the pituitary and the unusual tissue of the cone (posterior) lobe. Delicate, vertically oriented fibers form the foundation of this tissue. The glial framework associated with the latter contains a few elongated pyramidal ganglion cells. The blood vessels of the cone (posterior) lobe form a network with large intermediate spaces. The terminal loops of these vessels extend below the epithelial seam, forming loops that connect directly to the cells of the seam without any intervening tissue. Based on the description above, it is evident that Lothringer made a correct assumption regarding the formation of the cone (posterior) lobe, as I will elaborate below. However, he did not recognize the true nature of the glial tissue and the septa at his time. This could only be fully appreciated after treatment of this organ with the Golgi method. I performed such an experiment several years ago and obtained excellent staining of these tissue parts. Since, to the best of my knowledge, no other researcher has addressed this question, I will describe my results below.

I studied the pituitary of human, cat, dog and rabbit. Among those, in particular cat and dog was well suited to obtain closer insight into the structure of this organ. One can recognize in sagittal and frontal sections of the pituitary of older

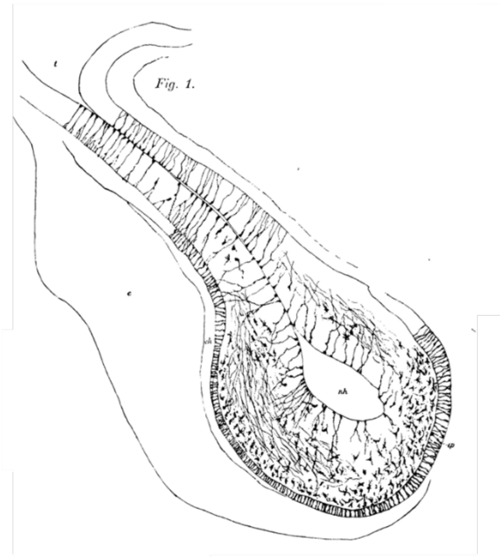

Fig. 1 from Table XII. Sagittal section of the pituitary of a 14 cm long cat fetus; - t, third ventricle which transits into the cone canal; - nh, cavity of the neuro pituitary; - ep, epithelial seam; - eh, cavity of the epithelial pituitary; - e, epithelial pituitary.

cat fetuses, using the Golgi method, that in general the description of Lothringer is correct. At the animals mentioned, lobus infundibuli as a roundish enlarged process of the tuber cinereum inserts into cup-formed depression of the epithelial pituitary (Fig. 1 of Table XII). In the interior of the tubular cone one can observe a small canal, an extension of the third ventricle and in the interior of the cone (posterior) lobe is a cavity visible, the cone lobe-cavity. At the lower envelope of the lobe one can recognize the epithelial seam as described by Lothinger; it is attached with a sharp border to the tissue of the lobe and closely connected and it contacts at the outside the cleft of the epithelial pituitary. On the top of the cone (posterior) neck, one can recognize the rim of this epithelial seam in the tissue of the epithelial pituitary.

Even at low magnification (Fig. 1) one can recognize a lot of elongated cells stained with the Golgi technique in the mentioned epithelial seam; they are mainly quite small, even thread-like and span through the entire seam, they do not form an epithelial layer out of multiple rows of cells, as Lothringer stated. In Fig. 4, e (from a 2-month-old dog) and in Fig. 3 (from an 8 day old rabbit) of Table XII, I have illustrated, at higher magnification, these cells resembling epithelial cells.

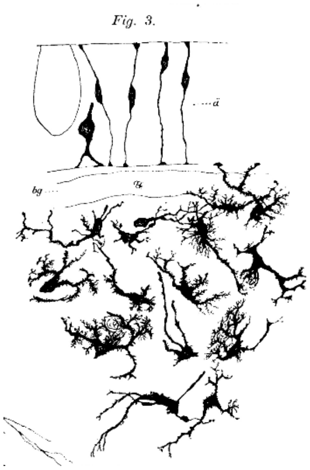

Fig. 3 from Table XII. Border part of the neuro pituitary (vertical section) of an 8-day old rabbit; - c, epithelial seam with stained cells; bg, blood vessel which runs close to the epithelial seam. Different neuroglial cells are shown.

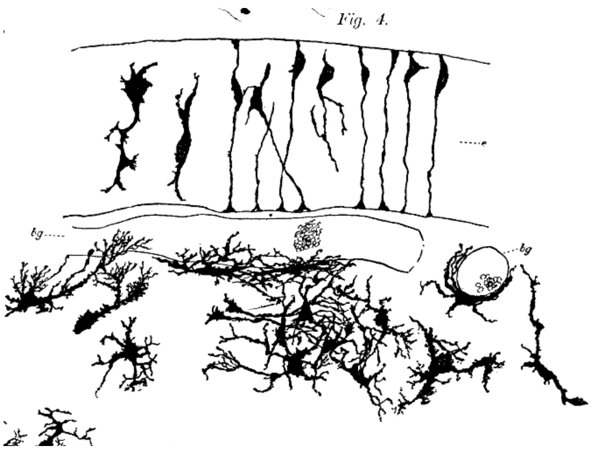

Fig. 4 from Table XII. Border part of the neuro pituitary (vertical section) from a 2-month-old dog; - c, epithelial seam; - bg, blood vessel in longitudinal and cross section. Neuroglial cells are shown in different forms.

As one can see in these figures, not all cell span through the entire layer; there are some peculiar shaped, branched forms which are embedded between the thread-shaped and only reach one layer of the seam or not at all; the nuclei are often close to the outer end; the inner end of thread-shaped cells enlarges often to a triangular foot which contacts the tissue of the cone (posterior) lobe, in fact at the sharp border line or area at which the blood vessel mesh are closely attached as described by Lothringer (Fig. 3 and 4, bg). At the outer surface of the epithelial seam one can often see a nice mosaic of small polygonal fields formed by the outer terminal areas of the cells (Fig. 2 a).

Fig. 2. a

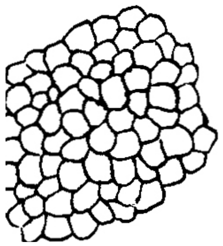

Fig. 2a from Table XII. Mosaic pattern of the epithelial seam, seen from outside (young dog).

In the tissue of the cone (posterior) lobe one can recognize in Golgi preparations even at low magnification a large number of stained fibers and branched cells. In frontal sections (Fig. 2) one can recognize a large number of such fibers which project from above, namely from the cone neck radially into the lobe. They look like nerve fibers, and it is indeed difficult to exclude such a view, while they also appear coarse similar to glial fibers. There are also very long processes of glial cells. Many of these fibers are of that type and can be established as processes (of glial cells). As stated by Lothringer, in the inners parts of the lobe there are larger blood vessels which project outward and penetrate the tissue, in particular the outer parts of the epithelial seam by an abundant meshwork of slim vessel loops.

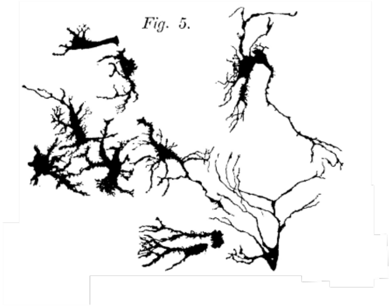

Fig. 5 from Table XII. A group of neuroglial cells from the interior of the neuro pituitary of a 2-month-old dog.

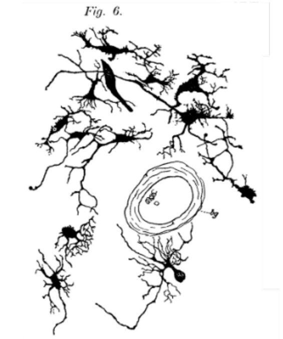

Fig. 6 from Table XII. A group of neuroglial cells from the interior of the neuro pituitary of a 1-year-old dog; - bg, cross section of a blood vessel.

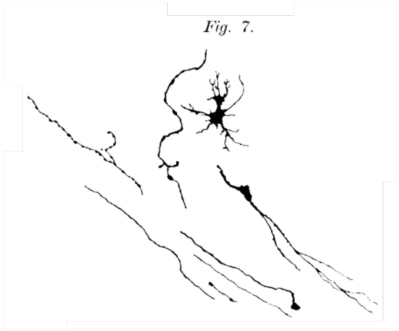

Fig. 7 from Table XII. Neuroglial cells and fibers from the interior of the neuro pituitary of a 1-year-old dog.

Between these vessel loops stretch the numerous fibers which divide here and there dichotomic, in particular in the peripheral parts and end free and branched. In this dense tissue one can recognize a number of branched cells stained with chrome silver which can be recognized as glial cells due to their characteristic form. In Figs. 3, 4, 5, 6 and 7,

I have illustrated these cells, partially at the periphery of the lobe and partially from the interior. Fig. 4 is from a young rabbit, and the others are from 2 to 12-month-old dogs. These glial cells do not belong to the major types described above. Some can be considered Sternstrahler, while others have only single processes on one side, which are equipped with spikes and mossy protrusions. The figures provide a better impression than an extensive description, and I refer to those.

The glial elements form a dense network. In more strongly stained regions, they are difficult to study. Occasionally, one finds regions where only single cells are stained, as in the illustrated parts, and one can easily trace the arrangements and branching. There is no particular arrangement, but they form a network of elements intermingled in different ways. Only in close proximity to blood vessels does one occasionally observe a tubular or laminar extension of processes (Fig. 4), though not as typical as the glial cells wrapping blood vessels in the cerebral cortex, as described by Lloyd Andriezen. I have never observed glial cells anastomosing among each other.

I have often observed Langsternstrahler in the cone (posterior) lobe, particularly in the neck region close to the transition to the cone. Fig. 8 illustrates three such cells from a 17-year-old man. In the actual cone neck, which forms the transition to the cone, glial cells are arranged perpendicular to the surface. Lothringer seemed to have observed such a thread-like formation. His observation can be explained by the arrangement of the glial cells. These glial cells can indeed be considered epithelial cells since they extend from the inner surface, the canal area where the cell bodies are located, to the surface. During this course, they sometimes divide dichotomously multiple times. Some, however, remain unbranched and slightly spiky. These cells correspond to the ependymal cells of the cone (posterior) region recently described and illustrated by Berkley (Anat. Anz., Aug. 1894).

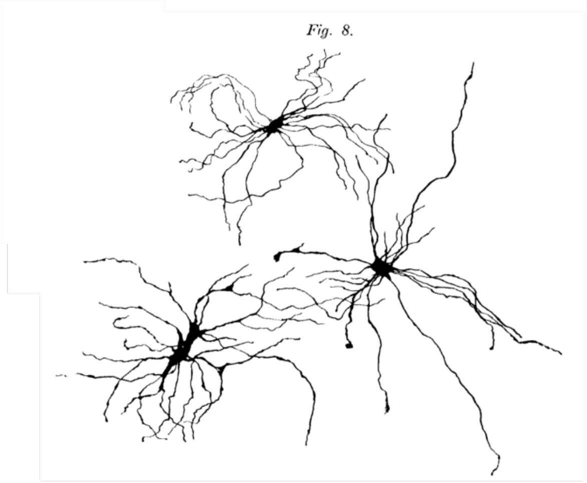

Fig. 8 from Table XII. Three neuroglial cells from the cone neck in the vicinity of the neuro pituitary of a 17-year-old man.

In the posterior lobe of the pituitary, the neurohypophysis (Processus seu Lobus infundibuli) there is also a large mass of glial cells present. Lothringer has recognized this tissue (1886) and mentioned it. Due to a lack of good methods, he could not recognize the forms of these elements; at this time, the concept of glial tissue was still vague; one did not know much of its true nature and the opinions of the eminent histologists diverged. Lothringer just mentioned glial nests as granulated filling material of the intermediate spaces of the fiber tracts; nuclei he could recognize within. Due to the Golgi method, it was possible to demonstrate the presence of the typical, highly ramified glial cells and fibers in mixed arrangement and varying morphology in the tissue of the posterior lobe. Moreover, there are many fibers present and a certain portion can be considered as glial fibers; another portion may perhaps represent nerve fibers; I will currently not dispute this assumption. Furthermore, there are many blood vessels with their aventitial sheath present. Other connective tissue I could not establish. All the tissue described by these authors (including Lothringer) is glial tissue (and potentially nerve fibers) according to my opinion. True nerve cells, ganglion cells in the posterior lobe I could not verify with the Golgi method. Negative results are not conclusive; since I made many staining experiments, I am convinced that such cells are normally not present.

IV. The Neuroglia of the nervus opticus and the retina of humans and mammals

(Table XIII)

The neuroglia of the optic nerve (opticus) and the retina have been well recognized in several excellent studies, and I have included them here, as these typical neuroglial forms should not be omitted in an overview presentation of neuroglia. Moreover, I have illustrated these forms over several years (1887) using the Golgi method and provided a number of figures. Referring to Table XIII, where I provide these illustrations, I will only briefly describe the neuroglial forms.

With respect to the glial cells of the optic nerve, these interstitial cells in this nerve were long known. Primarily, Key and I described these cells in detail in our extensive monograph on the nervous system and connective tissue[1]. These cells are massively present between the nerve bundles and within the interior, with many fine and long processes. We provided detailed illustrations (Table XXXIV of the mentioned publication). When I later (in the year 1887) obtained stainings of these cells with the Golgi method, I found it unnecessary to provide another report.

In the following years, only a short description of the glial cells of the peripheral nerve roots was published by Petrone[2]. He claims to have found glial cells not only in the optic nerve but also in many other, even true peripheral nerves, including the intracranial roots. Except in the optic and olfactory nerves, he found them in the trigeminal, acoustic, facial, glossopharyngeal, and spinal nerve roots. I must admit, I was surprised, since among these nerves, only two are of central nature. All the others are truly peripheral and contain nerve fibers with Schwann sheaths. This report by Petrone seems somewhat dubious, and the control experiments in the peripheral nerve roots only yielded negative results. I obtained only staining of true connective tissue. It should be noted that the figures in Petrone's report are presented in such a way that it is not clear which figures refer to the different peripheral nerves.

1 Axel Key and Gustav Retzius, Studien in der Anatomie des Nervensystems und des Bindegewebes. Bd. I. 1875.
2 Louis Petrone, Sur la structure des nerfs cerebro-rachidiens. Intern. Monatsschr. F. Anat. Physiol. Bd 5. 1888

In 1892, Kallius[1] described neuroglial cells in the optic nerve (opticus), stained with the Golgi method, as well as in the trigeminal, acoustic, and vagus nerves, particularly in sections at the exit from the brain. Shortly after, Michel[2] reported that he also illustrated long-projecting neuroglial cells in the optic nerve in large numbers.

Around the same time, Dogiel[3] provided a detailed description of the neuroglia in the retina of humans, particularly the Müller fibers, but also of the Sternstrahler. The latter, he states, are only found in the nerve fiber layer, close to the Papilla n. optici, in the region where the thickness of the layer is considerable. Furthermore, they are found in the entire papilla and in the extension of the entire optic nerve. These cells, with fairly long projections, often in bundles, from a more or less flattened cell body, are smaller in the retina and in the papilla n. optici compared to those cells embedded in the fiber bundles of the optic nerve. Cells located at the periphery of the nerve are larger than the other cells.

In a later report on the retina, Kallius[4] again describes the neuroglial cells of the optic nerve and the internal retinal layers (the nerve fiber and the inner ganglion cell layer).The glial cells of the retina, as well as the glial cells of the optic nerve, were known long before the introduction of the Golgi method, as evidenced by the work of Schwalbe and several other researchers. With the mentioned method, Ramon y Cajal described and presented them beautifully. I refer to his last major study on the retina of vertebrates[5].

What I was interested in the investigations on the neuroglia of the opticus was to analyze the transition of the ependymal cells into Sternstrahler. I hoped in the beginning to resolve the origin of glial cells, by finding transitions in early stages of development. I did not succeed as I had expected, despite of these studies will still be continued. What I found in the embryonic Nervus opticus consisted of early stages of glial cells, actually only Sternstrahler. Fig. 1 of Table XIII shows the peripheral part of the optic nerve from a 25 cm long human fetus and Fig. 4 from a 14 cm long cat fetus. The cells have always only few processes which show a curved course and a knobby appearance.

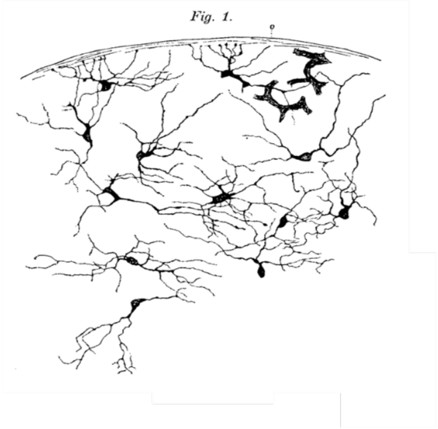

Fig. 1 from Table XIII. Border region of an optic nerve from a 28 cm long human embryo; - o, pial sheath of the optic nerve.

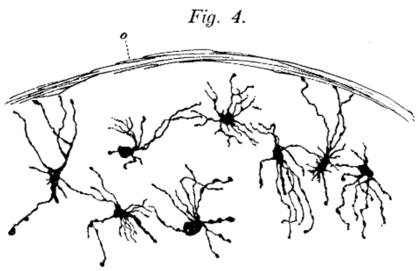

Fig. 4 from Table XIII. Border region of the pial sheath (o) from a cross section of optic nerve from a 11 cm long cat fetus.

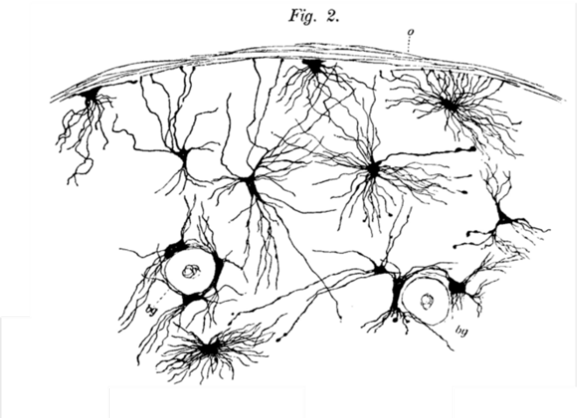

Fig. 2 from Table XIII. Border region with pial sheath (o) of the optic nerve from a 45 cm, long human fetus; - bg, white matter with cross-cut blood vessel.

Already at a 45 cm long human fetus (Fig. 2) has the embryonic type already more or less transformed into the later form; the processes are more numerous, stiffer and elongated. At a 5-day old cat (Fig. 5 and 6) are embryonic forms present together with developed forms. At a 14-day old cat, the development of the Sternstrahler has far proceeded as shown in Fig. 7. At the dog, the cells remain over a longer time less mature.

The relationship of the Sternstrahler to the bundles of nerve fibers was known for many years. In the above-mentioned publication by me and Key (1875), the arrangement and distribution was thoroughly described and illustrated, and I will set aside a new presentation. We showed that the majority of the cells accumulate in the lymphatic clefts between the nerve fiber bundles and these clefts are infiltrated by a blood vessel containing, connective tissue meshwork; the cells project their fine processes into all

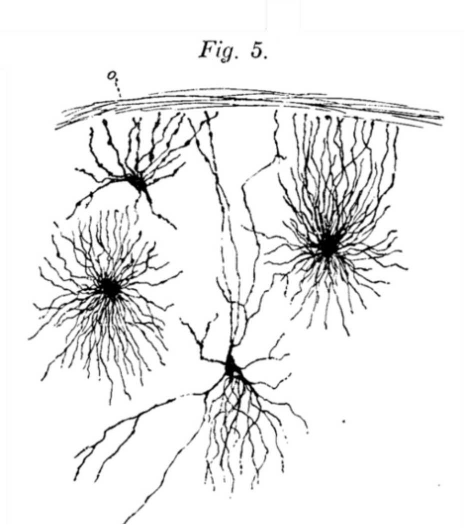

Fig. 5 from Table XIII. Border region of the pial sheath (o) from a cross section of optic nerve from 5-day old cat.

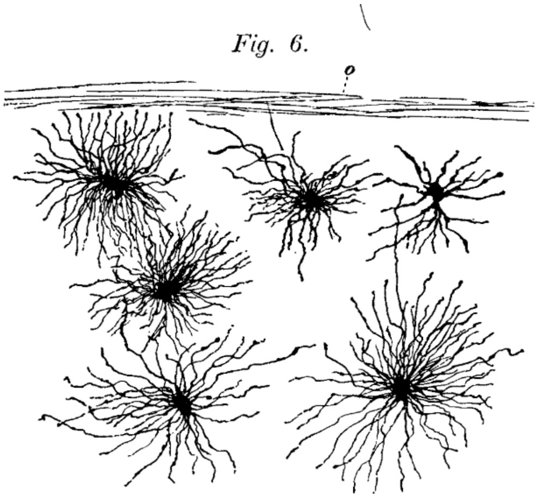

Fig. 6 from Table XIII. Border region of the pial sheath (o) from a longitudinal section of optic nerve from 5-day old cat.

directions forming a dense meshwork not only in the mentioned lymphatic clefts, but also around the bundles of nerve fibers; the cells and their processes are also abundantly present in the interior of the bundles of nerve fibers as shown in our illustrations. These relationships can now be confirmed with the Golgi technique. Not only the wrapping of the

bundles, but also the infiltration of the cells and their processes into the interior can be confirmed in cross-sections of the optic nerve (Fig. 3 of Table XIII, from a new-born child); the cell processes penetrate the bundles of nerve fibers in a different manner. With respect to the forms of glial cells, there are different variants present;

Fig. 3.

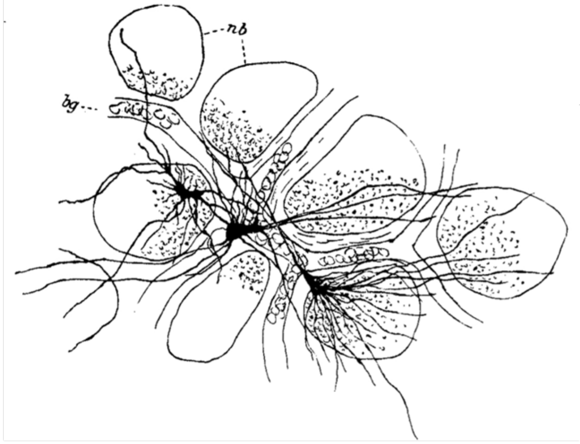

Fig. 3 from Table XIII. Region from the interior of a cross-section of the optic nerve with cross-cut nerve fiber bundles (nb), blood vessels (bg) and three neuroglial cells. From a 48 cm long human fetus.

Fig. 7.

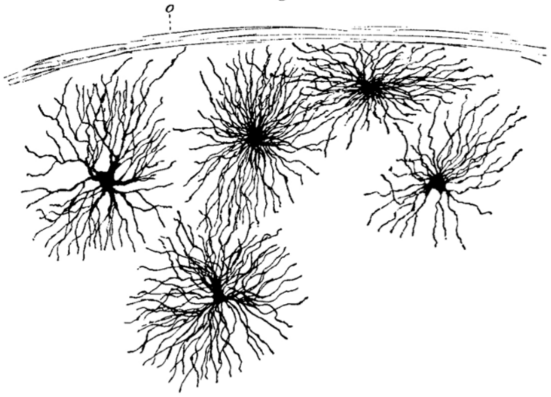

Fig. 7 from Table XIII. Border region of the pial sheath (o) from a cross section of optic nerve from 14-day-old cat.

[1] E. Kalius, Ueber Neurogliazellen in peripherischen Nerven, Nachrichten von d. k. Gesellsch. d. Wiss. etc. In Göttingen 1892.
[2] Michel, Sitz, Ber. D. Würzb. Med. naturwiss. Gesellsch. 14. Jan, 1893.
3 A. S. Dogiel, Neuroglia der Retina des Menschen. Arch. F. mikrosk. Anat. Bd 41, 1893.
[4] E. Kalius, Untersuchungen zur Netzhaut der Säugetiere. Anatom. Hefte, herausgeg. Von Merkel Und Bonnet, 1894.
[5] S. Ramon y Cajal, La retine des vertebres, la Cellule t. 9, 1, 1893 (Dep. 1892).

partly there are true, regular Sternstrahler present, partly the processes extend from the cell body in bundles so that the cells are equipped with one or more tails as emphasized by Dogiel. This is in particular present at the surface of the optic nerve; here (Fig. 2) are often broad, flattened cell bodies placed close to the pial sheath, similar as at the brain surface, and they extend their processes in broad bundles into the nerve; these cells can be named Schwanzstrahler. Other cells, which are further below the surface, send their processes to the pial sheath ending with small knobs or feet there (Fig. 1, 2, 5, 8) corresponding to a type of Fusssternstrahler.

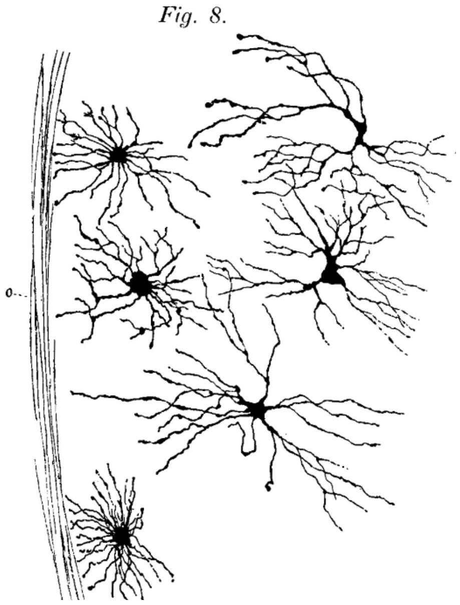

Fig. 8. From Table XIII. Border region of the pial sheath (o) from a cross section of optic nerve from 1-month-old dog.

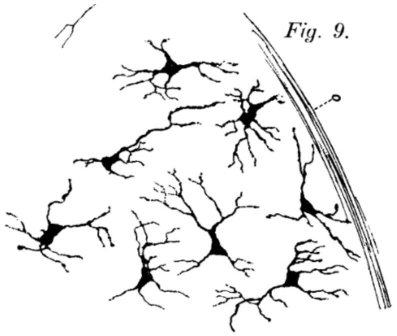

Fig. 9 from Table XIII. Border region with fibrous limitation (o) from a cross-section of an optic nerve in the area of the Lamina cribrosa of a new-born dog.

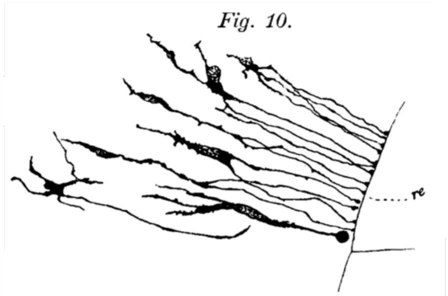

Fig. 10 from Table XIII. Border region of the entry of the optic nerve into the eye, in longitudinal section. From a 28 cm long human embryo: a number of stretched neuroglial cells are displayed which are perpendicular oriented to the retinal wall (re).

In the Lamina cibrosa[4#] of the optic nerve the properties of the glial cells change as extensively described by me and Key (1875). The processes are less frequent and shorter, so that these elements appear smaller (Fig. 9 from a new-born dog) and are oriented perpendicular to the nerve. At the transition to the Papilla n. optici, the peripheral glial cells are elongated again and send long processes to the surface where they terminate in a knob-type fashion; the represent a unregular palisade formation (Fig. 10). The terminals of these glial cells touch the retina which is inserting at the angle of the optical nerve (Fig. 10), yet not providing a transition of the Müller fibers.

With respect to the form and arrangement of the glial cells in the retina, they have been extensively described in recent times by several researchers, including Cajal, Dogiel, and Kallius, and I will not discuss them further.

Regarding the Müller fibers, which maintain their ependymal type throughout life, I must add a few words. Thanks to the Golgi and Ehrlich methods, it is now possible to resolve issues related to the retinal structure that were previously uncertain, dubious, or even unknown. This has become clear to everyone, including myself, as I had worked on the retina for decades using the old methods and have now tested the new ones. This pertains to the relationship between the Müller fibers and the reticular layers. In earlier days, I could not, like many other retinal researchers, convince myself of the branching of these fibers in the inner reticular layer. Thanks to the Golgi method, this has become clear to me since 1888. The Müller fibers do not only project multiple fine branches into the inner reticular layer, but also into the outer reticular layer, as shown by Ramon y Cajal, similar to ependymal cells in the brain and spinal cord. In Fig. 15 (from a cat), Fig. 16 (from a rabbit), and Fig. 17 (from a human), I have depicted those fibers to no longer support the old doctrine that these fibers are unbranched.

Regarding the development and formation of the Müller fibers in the different classes of vertebrates, Ramon y Cajal has provided all the essential details in his excellent investigations on the retina, particularly in his last extensive monograph. Despite my experience over several years on the formation of the retina in different classes of vertebrates and having prepared a publication on the subject, I will not provide further details. However, with respect to the Müller fibers, I will provide some developmental forms in Table XIII (Figs. 11, 12, 13, 14, 17). In Fig. 11, one can see the structure in the embryonic state; in Figs. 12, 13, and 14, they have become more thread-like and begin to branch at the inner end. In Fig. 17 at r (left), they are unbranched, but at r1 (right), further behind in the retina, they have fine branches in the inner reticular layer and even jagged wing-like enlargements in the inner granule cell layer. In contrast, at the outer edges, they are unbranched and without wings. With respect to the fine branches at the outer end of the fibers, which protrude between the inner rod parts, one can recognize them nicely in Golgi preparations. In general, they are straight and parallel, extending to the outer parts. Often, I found them slightly bent, with a granular, rope- or pearl-like appearance (Fig. 15); occasionally, thicker, club-like formations were stained (Fig. 16).

On Table XIII I have depicted some other retinal elements together with the Müller fibers to illustrate their orientation.

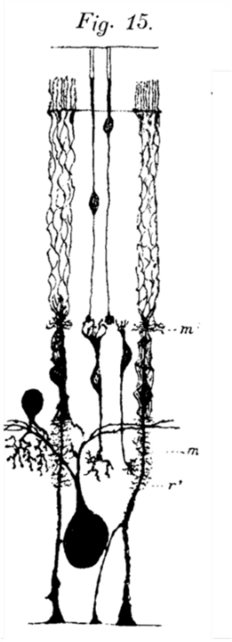

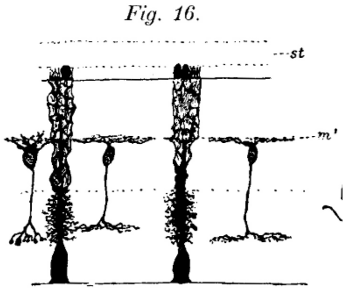

Fig. 16 from Table XIII. Region of a vertical section of the retina of a 6-week-old rabbit. Two Müller fibers and three amacrine cells are shown. – st, rod layer; - m^1, inner reticular layer.

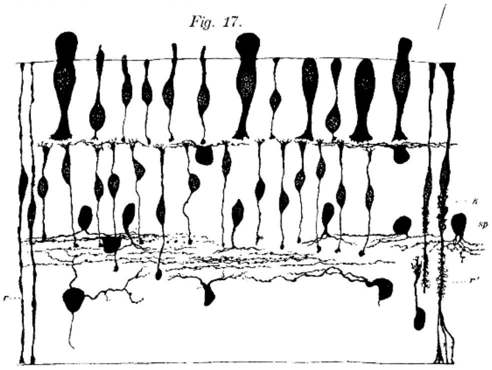

Fig. 17 from Table XIII. Vertical section of the retina from a 28 cm long human embryo. – r, Müller fibers from the frontal peripheral zone; r^1, Müller fibers from the posterior

Fig. 15 from Table XIII. Region from a vertical section of the retina of a 1-month-old cat, - r, r^1, Müller support fibers, - m, inner reticular and m^1 outer reticular layer. There is also one ganglion cell, amacrine cells and rod elements illustrated.

Fig. 11.

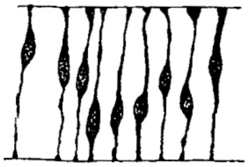

Fig. 11 from Table XIII. Vertical section of the retina of a 4 ½ day old chicken embryo. The inner surface (Limitans interna) is oriented to the lower side of the Table.

Fig. 12. *Fig. 13.* *Fig. 14.*

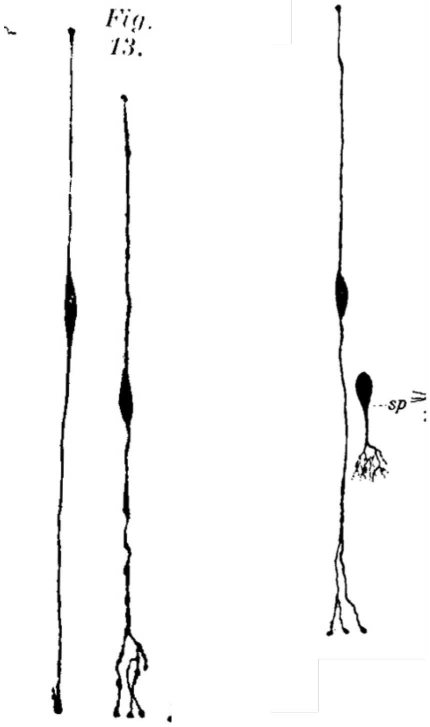

Fig. 12 – 14 from Table XIII. Müller support fibers in the embryonic stage. – Fig. 12 from a new-born Mus decunanus[5#]; Fig. 13 from a 4-day old Mus musculus, at the region of the Ora serrata; Fig. 14 from a 4-day-old Mus musculus, at the region of the papilla optici; sp, an amacrine cell of the inner granule cell layer.

These are mainly the elements named by Cajal as amacrine cells (Spongioblasts); I have particularly presented the relationship of the developing human retina. Fig. 17 of Table XIII presents such an image, where I have brought together several elements. I can abstain from description since the figure clearly shows it all. I would like to point out that the formerly so-called inner granules in the inner reticular layer in a 28 cm long embryo do not show branching of their inner process, but the process terminated at different levels of the layer with a granular knot (a growth club) and it reminds in this state at an internal rod fiber.

At this opportunity I have to mention that with respect to the reticular layers, I no longer consider it to be of net-type arrangement. In the early 80th or even at the beginning of the 70th, when I first published my observations, the methods of investigation were not appropriate to resolve this difficult question as one could not resolve issues of neuroglia in the central organs. I made my investigations predominantly with osmium acid and this resulted in a granular and net-type structure. In those preparations (1870), I was convinced that the inner processes of the inner granule cells insert more or less deep (up to a ¾ of the thickness) into the inner reticular layer without branching; then I could not observe them further. I refuted the doctrine by Max Schultze that they are branching in this layer and transit into a dense network. Golgi and Ehrlich have shown, that we were both partially right. Most of the fibers insert more or less unbranched into the layer and subsequently branch extensively. Like Kallius, I support the view of a reticulate, felt-type rather than a net-type basic substance of the type as I had seen it in my earlier osmium preparations. With respect to this basic substance, we have not yet resolved its structure so far.

At the end of this overview publication on the neuroglia of the brain and some of their associated organs, I would like to emphasize that I have never been convinced of anastomotic connections of glial cells and their processes, neither among the individual elements nor between different individuals. Wherever I obtained clear and convincing images, I saw them separated from each other.

Regarding the much-discussed basic substance of the neuroglia, I have reached the conclusion that it does not exist. The glial cells and their processes form the felt-like supportive substance, and in their mesh, the nerve cells with their processes are embedded. The only connective tissue substance present is in the blood vessels and their sheath, which can occur, as in the spinal cord, flattened and of a septal type. The mesh of glial tissue contains only tissue fluid. A homogeneous, structureless basic substance, as assumed by some researchers like Gierke, cannot be substantiated histologically.

Glial covers are difficult to establish. The glia (and the ependyma) forms a condensed termination at the outer and inner surface against the adjacent parts (pia and cerebrospinal fluid of the ventricles). However, this glia and the ependyma are not a delimited cover but are closely associated with the underlying glial tissue.

With the above description of the neuroglia, I have intended, as mentioned several times, to provide an overview of this tissue. To obtain in-depth knowledge of this peculiar substance, named by Rudolf Virchow as neuroglia and described from different aspects, extensive further investigations are required. In particular, each part of the central organ in different vertebrate species and in different stages of development needs to be individually studied.

I would like to touch upon a few points. From the description above, it is evident that the glial cells of the brain partially undergo a substantial change in morphology during development. Their substance must therefore not be rigid during the fetal period, but rather flexible and transformable, despite the fact that they are later, particularly with respect to the processes, rather stable. This stabilization of the glial elements serves to provide a proper support system for the nervous elements. It does not exclude the possibility that glial cells may have other functions since there is living protoplasm around their nuclei. In particular, there could be influences of glial cells related to the nutrition of the organ, the composition of the extracellular fluid, and the development of the fetal phase. I would like to mention these possibilities, despite the fact that there is not yet proof.

With respect to the relative amount of glial tissue in the different regions and layers of the brain, it is difficult to make conclusions. Since the glial tissue is not only a support substance for the nervous elements but also a filling mass between them, it is evident that it is more abundant where the nervous elements are sparse and vice versa. It is therefore more abundant at the surface of the central organ compared to the interior, particularly in areas where the nerve cells accumulate. In the pyramidal cell layer, but also in the cord, the glial tissue is relatively sparse; however, it is actually more abundant than generally believed. The Golgi method does not provide true conclusions in that respect, but often one finds in Golgi preparations areas, e.g., in the pyramidal cell layer, containing a dense network of Sternstrahler which cannot be penetrated by the eye. The nerve cells are embedded in this mossy mass, so that they can hardly be recognized.

With respect to the relationship between the true neuroglia and the ependyma, it is not my intention to address that issue. That there are close relationships between these types of tissues in the brain and spinal cord is evident from several instances. To me, there seems to be an earlier histologic differentiation in the brain compared to the spinal cord.

Of particular interest is the feature that in humans, the ependymal cells can be detected in the late period of fetal life and in animals even after birth at the surface of the cortex in their characteristic form.

Comments

1#. Kurzstrahler and the Langstrahler: Short beamer or long beamer

2#. anastomose: fuse

3#. Cone is the term describing the posterior pituitary, also the neuro pituitary

4#. The lamina cribrosa is a mesh like structure forming the exit for the optic nerve from the retina. It fills a whole in the sclera.

5# Old term for the brown rat (Rattus norvegicus), also known as the common rat, street rat, sewer rat, wharf rat, Hanover rat, **Norway rat** and **Norwegian rat**.

Figure Legends

Table I.

The neuroglia of the human cerebral cortex.

Fig. 1. Vertical section of the cerebral cortex of a 6 1/2 old human fetus. Different types of neuroglial cells in their natural position in the cortex; - c, a Cajal cell, - e, terminals of ependymal cells.

Fig. 2 Tangential section of the cortical surface of the same fetus, neuroglial cells.

Fig. 3 Section from the cortex of the same fetus (about 2.5 Mm.) below the surface. A blood vessel with attached neuroglial cells.

Fig. 4 Vertical section of the cerebral cortex (frontal lobe) of a 19.5 cm long human embryo. Outer ends of the ependymal cells.

The figures are drawn after Golgi preparations at magnification objective 6, ocular 3 (inserted tubus).

Table II

The neuroglia of the human cerebral cortex.

Fig. 1. Vertical section in the cortex of a gyrus of the temporal lobe from a delivered 48 cm long (female) human fetus. Neuroglial cells of different forms, most of them of the fetal type.

Fig. 2. A Sternstrahler from the inner white substance of the preparation shown in Fig. 1.

Fig. 3. Tangential slice of the cortical surface of a gyrus of the temporal lobe of a delivered 48 cm long human fetus. Four cell bodies of Schwanzsternstrahler with processes of the fetal type.

Fig. 4 and 5. Vertical sections of the cortex of a gyrus of the frontal lobe from a 45 cm long human fetus. Neuroglial cells of different fetal types are shown. In Fig. 5 are, in addition, two small pyramidal cells and some tangential fibers (probably processes of Cajal cells) shown and (on the bottom on the right) a blood vessel surrounded by a glial cell.

All figures are drawn after Golgi preparations at magnification objective 6 , ocular 3 (inserted tubus).

Table III.

The neuroglia of the human cerebral cortex.

Fig. 1. Vertical section of a gyrus (G. front. Med.) of the cerebral cortex of a 2-month-old child. Neuroglial cells of different types in their natural position in the cortex. In the center, a blood vessel inserts from the surface. Some tangential fibers are visible further below.

Fig. 2. Three Sternstrahler from the deeper region of the same vertical slice.

Fig. 3. Tangential section of the surface of the cortex (Gyr. Temp. inf.) from a 2-month-old child. Neuroglial cells seen from the surface.

Fig. 4 Vertical section of a gyrus of the frontal lobe in the cortex from a 3-month-old child. Neuroglial cells of different types in their position within the cortex.

Fig. 5. Four neuroglial cells of the cortical surface in tangential extension (tangential slice of the cortical surface).

The figures of this Table are drawn after Golgi preparations at magnification objective 6, ocular 3 (inserted tubus).

Table IV.

The neuroglial cells of the human cerebral cortex.

Fig. 1 Vertical section of one cortical gyrus of the frontal lobe from a 1-year-old child. Schwanzstrahler, Sternstrahler and Fusssternstrahler.

Fig. 2 Vertical section of one cortical gyrus of the frontal lobe from a 5 ½-year-old child. Neuroglial cells of different types.

Fig. 3 Vertical section of one cortical gyrus of the occipital lobe (Gyr. occ. med.) of a 17-year-old man. Neuroglial cells of different types and a pyramidal cell.

Fig. 4 Vertical section of one cortical gyrus of the parietal lobe Gyr. centr. post.) from a 32-year-old man. Neuroglial cells of different types.

Fig. 5. Tangential section of the cortical surface (Gyr. centr. post.) of a 42-year-old female. Flächen- and Schwanzstrahler and Sternstrahler at deeper layers.

The figures of this Table are drawn after Golgi preparations at magnification objective 6, ocular 3 (inserted tubus).

Table V.

The neuroglia of the human cerebral cortex.

Fig. 1 Vertical section of a gyrus of the frontal lobe from a 42-year-old female.

Fig. 2 – 5. Vertical section from the parietal region from a 70-year-old man.

In all figures the surface of the cerebral cortex is directed to the upper side of the Table. Therefore, one can see the neuroglial cells at the upper rim of the figure which reach the surface and extend there; below these cells one can see the neuroglial cells of different types in deeper layers. In Fig. 1 and 2 are blood vessels present, at which glial cells are attached. In Fig. 5 there is (upper left) a stained small ganglion cell. Apart from that all elements are neuroglial cells.

The figures of this Table are drawn after Golgi preparations at magnification objective 6 , ocular 3 (inserted tubus).

Table VI.

The neuroglia of the cerebral hemispheres of the dog.

Fig. 1. Vertical section of a gyrus from the vertex of the cerebrum of a 2-month-old dog.

Fig. 2. Three Langsternstrahler from the white matter of the same preparation; its surface is shown in Fig. 1 (from a 2-month-old dog).

Fig. 3 Vertical section of a gyrus from the vertex of the cerebrum of a 1-month-old dog; at the surface rim are two blood vessels of the pia depicted with attached cell bodies of Schwanzsternstrahler.

Fig. 4. Parts of tangential sections of the surface of the cortical hemisphere from a 2-month-old dog. – a, two glial cell bodies with their processes positioned at the surface – b, part of the surface with mosaic pattern, corresponding to the contour of the cell bodies of glial cells at the surface; two cell bodies are entirely attained, one with stained processes.

Fig. 5 and 6. Vertical section of gyri from the vertex of the cerebrum of an adult (old) dog.

Fig. 7. Vertical section of a gyrus from the vertex of the cerebrum of a 5-day-old dog. Besides the three glial cells present below the surface, there are the outer edges of six ependymal cells displayed in the figure.

In fig. 1, 3, 5, 6, 7 is the edge of the vertical sectioned brain surface directed to the upper end of the Table.
All figures of this Table are drawn after Golgi preparations at magnification objective 6, ocular 3 (inserted tubus).

Table VII.

The neuroglia of the cerebral hemispheres of the cat.

Fig. 1 Vertical section from the vertex of the brain from a 8 day old cat; the glial cells are of the fetal type.

Fig. 2. Vertical section from the vertex of the brain from a 13-day old cat. Besides the glial cells there are the outer edges of two ependymal cells displayed.

Fig. 3. Vertical section from the vertex of the brain from a 15-day old cat.

Fig. 4. Vertical section from the vertex of the brain from an adult (old) cat.

Fig. 5. Three Langsternstrahler from the white matter below the cortex at the brain vertex of an adult (old) cat.

Fig. 6 – 8. Tangential section of the brain surface at the vertex. From an adult (old) cat. Glial cell bodies with processes positioned at the surface.

Fig. 9. A glial cell attached to a blood vessel sheath from the cerebral cortex of an adult (old) cat.

In Fig. 1-4, the surface rim is oriented to the upper side of the Table.
All figures of this Table are drawn after Golgi preparations at magnification objective 6, ocular 3 (inserted tubus).

Table VIII.

The neuroglia of the cerebral hemispheres of the rabbit.

Fig. 1. Vertical section at the vertex of the cerebrum of a 14-day old rabbit.

Fig. 2. Vertical section at the vertex of the cerebrum of a 4-day old rabbit. A Cajal cell is depicted surrounded by glial cells.

Fig. 3. Vertical section at the frontal end of the cerebrum of a 1 ½ day old rabbit. To the right of the group of glial cells is a Cajal cell shown.

Fig. 4. Vertical section at the vertex of the cerebrum of an adult (old) rabbit.

Fig. 5. Vertical section at the vertex of the cerebrum of a new-born rabbit. Below the four glial cells is a ganglion cell depicted.

Fig. 6. Vertical section of the cortical region of the cerebrum (vertex) of a 5-week-old rabbit. Six Schwanzstrahler with cell bodies at the surface.

Fig. 7. Vertical section from vertex of the cerebrum of an adult (old) cat.

Fig. 8 Vertical section of the Corpora quadrigemina of a 8 day old rabbit, to show the comparison with respect to the from of glial cells in this region in the rabbit.

All Figures of this Table are with the cortical rim oriented to the upper side of the Table and
at magnification objective 6 , ocular 3 (inserted tubus).

Table IX.

The neuroglia of the brain ganglia, the insula Reilii and the medulla oblongata of human.

Fig. 1. Frontal vertical section of the cortical part of the Corpus quadrigeminum anterius from a 42 cm long human fetus.

Fig. 2 Frontal vertical section of the cortical part of the Putamen thalami optici from a 42 cm long human fetus.

Fig. 3. Vertical section of the surface area from Corpus striatum of a 65-year-old man.

Fig. 4. Vertical cross section of the lateral surface area of the medulla oblongata of a 6 ½ old human fetus.

Fig. 5. Vertical section of the Insula Reilii of a 39 C. long human fetus – bg, a blood vessel with an attachment of a glial cell process.

All figures of this Table are drawn after Golgi preparations at magnification objective 6, ocular 3 (inserted tubus).

Table X.

The neuroglia of the cerebral ganglia and the gyrus hippocampi of the cat, as well as the olive of human.

Fig. 1. Frontal vertical section of the Corpus quadrigeminum anterius of a 14-day old cat.

Fig. 2 Frontal vertical section of the Corpus quadrigeminum posterius of a 14 day old cat.

Fig. 3. Frontal vertical section of the Corpus geniculatum of a 14-day old cat.

Fig. 4 Vertical section from the lower area of the Gyrus hippocampi of a 14-day old cat.

Fig. 5. Vertical section from the interor of the Gyrus hippocampi of a 14-day old cat.

Fig. 6 Vertical section of the Fornix of a 14-day old cat.

Fig. 7. Vertical cross section of the olive from a 8 months old human fetus. Due to lack of space in Table IX, this Figure is placed here.

All figures of this Table are drawn after Golgi preparations. Fig. 1 – 6 are drawn at magnification objective 6, ocular 3 (inserted tubus), Fig. 7 at magnification objective 2, ocular 3 (inserted tubus).

Table XI.

The neuroglia of the cortex of the cerebellum of human and mammals.

Fig. 1. Vertical section of the cerebellar cortex of a 37 cm long human fetus, with five Bergmann fiber cells; - ä. Vignal cell layer.

Fig. 2. Vertical section of the cerebellar cortex of a 45 cm long human fetus, with seven Bergmann fiber cells; - ä. Vignal cell layer; - m granule cell layer.

Fig. 3. Vertical section of the cerebellar cortex of a 48 cm long human fetus, with six Bergmann fiber cells and two Sternstrahler in early stage of development; - ä. Vignal cell layer; - m, basket cell layer.

Fig. 4. Vertical section of the cerebellar cortex of a 2-month-old child, with two Bergmann fiber cells and three Langsternstrahler; - ä. Vignal cell layer; - m, basket cell layer

Fig. 5. Vertical section of the cerebellar cortex of a 33-year-old man, with three Bergmann fiber cells, two Kurzsternstrahler and two Langsternstrahler.

Fig. 6. Vertical section of the cerebellar cortex of a not yet delivered cat fetus with two Bergmann fiber cells; - ä. Vignal cell layer; - m, basket cell layer.

Fig. 7. Vertical section of the cerebellar cortex of a 3 day old cat, with two Bergmann fiber cells; - ä. Vignal cell layer; - m, basket cell layer.

Fig. 8. Vertical section of the cerebellar cortex of a 5 ½ day old cat, with six Bergmann fiber cells and two Kurzsternstrahler; - ä. Vignal cell layer; - m, basket cell layer.

Fig. 9. Vertical section of the cerebellar cortex of a 14-day old cat, with four Bergmann fiber cells and five Kurzsternstrahler and a cell with unclear identification; - ä. Vignal cell layer; - t, tangential, bipolar cellsof this kayer; - m, basket cell layer with a basket cell (k); - f, white matter.

Fig. 10. Part of a tangential section of the cerebellar cortex of an 11-day old cat with a number of tangential bipolar elements (lower part of the Vignal layer).

Fig. 11. Vertical section of the cerebellar cortex of a 22-day old cat, with three Bergmann fiber cells and one Kurzsternstrahler; - ä. Vignal cell layer with strange, tangential running fibers; - m, basket cell layer.

Fig. 12. One Langsternstrahler from the white matter of the cerebellar cortex of a 22 day old cat.

All figures of this Table are drawn after Golgi preparations at magnification objective 6 , ocular 3 (inserted tubus).

Table XII.

The neuroglia of the pituitary gland of mammals

Fig. 1 Sagittal section of the pituitary of a 14 cm long cat fetus; - t, third ventricle which transits into the cone canal; - nh, cavity of the neuro pituitary; - ep, epithelial seam; - eh, cavity of the epithelial pituitary; - e, epithelial pituitary.

Fig. 2. Frontal section of the neuro pituitary of a young dog; t, cone canal which descends from the third ventricle; - ventricle wall with ependyma and neuroglia; towards the bottom the wall bends outward on both sides and forms a wrinkle; - ep, epithelial seam; - eh, the outward placed narrow gap-like cavity of the epithelial pituitary (e).

Fig. 2a. Mosaic pattern of the epithelial seam, seen from outside (young dog).

Fig. 3. Border part of the neuro pituitary (vertical section) of a 8 day old rabbit; - c, epithelial seam with stained cells; bg, blood vessel which runs close to the epithelial seam. Different neuroglial cells are shown.

Fig. 4. Border part of the neuro pituitary (vertical section) from a 2-month-old dog; - c, epithelial seam; - bg, blood vessel in longitudinal and cross section. Neuroglial cells are shown in different forms.

Fig. 5 A group of neuroglial cells from the interior of the neuro pituitary of a 2-month-old dog.

Fig. 6. A group of neuroglial cells from the interior of the neuro pituitary of a 1-year-old dog; - bg, cross section of a blood vessel.

Fig. 7. Neuroglial cells and fibers from the interior of the neuro pituitary of a 1-year-old dog.

Fig. 8 Three neuroglial cells from the cone neck in the vicinity of the neuro pituitary of a 17-year-old man.

Fig. 9. Two neuroglial cells and multiple fibers from the neuro pituitary (close to the neck part) of a 2-month-old child; - bg, blood vessel to which several processes of the one cell are attached.

All figures of this Table are drawn after Golgi preparations Fig. 1 is at magnification objective 2, ocular 1 (inserted tubus); Fig. 2 is at magnification objective 2 (half screwed out), ocular 1 (inserted tubus); the other figures at magnification objective 6, ocular 3 (inserted tubus).

Table XIII.

The neuroglia of the optic nerve and the retina.

Fig. 1 Border region of an optic nerve from a 28 cm long human embryo; - o, pial sheath of the optic nerve.

Fig. 2. Border region with pial sheath (o) of the optic nerve from a 45 cm, long human fetus; - bg, white matter with cross-cut blood vessel.

Fig. 3. Region from the interior of a cross-section of the optic nerve with cross-cut nerve fiber bundles (nb), blood vessels (bg) and three neuroglial cells. From a 48 cm long human fetus.

Fig. 4. Border region of the pial sheath (o) from a cross section of optic nerve from a 11 cm long cat fetus.

Fig. 5. Border region of the pial sheath (o) from a cross section of optic nerve from 5-day old cat.

Fig. 6. Border region of the pial sheath (o) from a longitudinal section of optic nerve from 5-day old cat.

Fig. 7. Border region of the pial sheath (o) from a cross section of optic nerve from 14-day old cat.

Fig. 8. Border region of the pial sheath (o) from a cross section of optic nerve from 1 month old dog.

Fig. 9. Border region with fibrous limitation (o) from a cross-section of an optic nerve in the area of the Lamina cribrosa of a new-born dog.

Fig. 10. Border region of the entry of the optic nerve into the eye, in longitudinal section. From a 28 cm long human embryo: a number of stretched neuroglial cells are displayed which are perpendicular oriented to the retinal wall (re).

Fig. 11. Vertical section of the retina of a 4 ½ day old chicken embryo. The inner surface (Limitans interna) is oriented to the lower side of the Table.

Fig. 12 – 14. Müller support fibers in the embryonic stage. – Fig. 12 from a new-born Mus decunanus[5#;] Fig. 13 from a 4 day old Mus musculus, at the region of the Ora serrata; Fig. 14 from a 4 day old Mus musculus, at the region of the papilla optici; sp, an amacrine cell of the inner granule cell layer.

Fig. 15. Region from a vertical section of the retina of a 1 month old cat, - r, r^1, Müller support fibers, - m, inner reticular and m^1 outer reticular layer. There is also one ganglion cell, amacrine cells and rod elements illustrated.

Fig. 16. Region of a vertical section of the retina of a 6-week-old rabbit. Two Müller fibers and three amacrine cells are shown. – st, rod layer; - m^1, inner reticular layer.

Fig. 17. Vertical section of the retina from a 28 cm long human embryo. – r, Müller fibers from the frontal peripheral zone; r^1, Müller fibers from the posterior zone; k, internal granule layer; - sp, amacrine cells of different type. There are several rod, cones and ganglion cells shown. Composed from different preparations.

All figures of this Table are drawn after Golgi preparations at magnification objective 6, ocular 3 (inserted tubus). Only Fig. 17 is shown with extended tubus.
