# Supplementary material for: The Concept of Neuroglia ‐ the State of the Art Circa 1900
Source: Glia. 2025 Feb 4;73(5):890–904. doi: 10.1002/glia.24678 (PMC11920685; doi:10.1002/glia.24678)
Supplement: Supplementary file 5 — Data S5. Scanned original publication by Lenhossek. [file GLIA-73-890-s006.pdf]

VI. Die Stützzellen des Rückenmarkes.

Nicht in geringerem Masse, als hinsichtlich der nervösen Elemente, hat die Golgi'sche Methode bezüglich der Stützzellen der Centralorgane fördernd auf den Gang unserer Erkenntnis und auch durch den genaueren Nachweis der Herkunft und der Entwickelungsweise dieser Elemente umgestaltend auf die herrschenden Vorstellungen eingewirkt.

War nun auch hier durch die Silbermethode manches interessante Ergebnis gewonnen, so litten doch diese Angaben, namentlich soweit es sich um das Rückenmark handelt, noch vor kurzem alle an dem Übelstande, dass sie sich nur auf die Centralorgane nicht völlig entwickelter Geschöpfe bezogen. In der I. Auflage dieses kleinen Werkes bemerkte ich, dass von allen Teilen der Centralorgane bisher das Rückenmark auf seine Stützelemente am genauesten untersucht sei, musste aber hinzusetzen, dass „auch in Betreff der Rückenmarksneuroglia ein nicht unwesentliches Hindernis eines abschliessenden Verständnisses in dem Umstande liegt, dass namentlich von den höheren Vertebraten und dem Menschen bisher nur junge Exemplare und Embryonen zur Untersuchung verwendet wurden, daher immer noch der Einwand erhoben werden kann, ob sich nicht in einem oder anderem die Verhältnisse später noch ändern.“ Ich bezeichnete es als die nächste Aufgabe der Forschung, die Darstellung der Stützzellen auch am entwickelten Marke mit der Golgi'schen Methode zu versuchen.

Seitdem ist nun die in diesen Worten als Desiderat bezeichnete Aufgabe in vollem Masse erfüllt worden durch die meisterhafte Darstellung, die v. Kölliker in seinem Lehrbuche[1]) vom Aufbau

[1]) A. v. Kölliker, Handbuch der Gewebelehre des Menschen, 6. Aufl., Bd. II, Leipzig 1893, p. 136.

des centralen Stützsystems gab, und in der auch vielfach Golgibilder aus dem entwickelten Marke berücksichtigt sind. Kölliker's Darstellung hat viel Licht in die Frage nach der Beschaffenheit der „Neuroglia“ gebracht, vor allem dadurch, dass sie gegenüber der in der letzten Zeit gar zu sehr vorherrschenden einseitig histiogenetischen Betrachtungsweise des Stützsystems das Bild der fertigen Anordnung wieder in den Vordergrund rückte und mit den entwickelungsgeschichtlichen Daten zu einem erschöpfenden, einheitlichen Bilde verknüpfte.

Auch ich selbst habe mich mittlerweile jener Aufgabe unterzogen und zwar mit befriedigendem Ergebnis. Freilich war es wieder nicht das Rückenmark völlig erwachsener Menschen, worauf ich meine Untersuchungen erstreckt hatte, denn hier war in der That nicht viel Aussicht auf Erfolg, sondern das von neugeborenen Kindern und solchen bis zum ersten Lebensjahre, indes darf man wohl annehmen, dass sich über diese vorgeschrittene Periode hinaus, wo doch schon das Rückenmark durch die vollkommene Markhaltigkeit der weissen Substanz seinen reifen Zustand dokumentiert, wesentliche Veränderungen nicht mehr einstellen.

Meine Versuche ergaben nun, dass das Golgi'sche Verfahren zur Darstellung der elementaren Zusammensetzung der Neuroglia, auch in ihrem fertigen Zustande, allen bisher zu diesem Zwecke angewandten Methoden bei weitem überlegen ist, freilich nur soweit es sich darum handelt, die Zellen in ihren äusseren Umrissen zur Ansicht zu bringen, denn die innere Struktur wird damit natürlich nicht erkannt. Die Bilder, die die Chromsilbermethode von den Spinnenzellen giebt, sind, wie dies auch v. Kölliker (a. a. O. p. 147) betont, in der That unübertrefflich schön. Dabei lassen sich diese so lehrreichen Präparate spielend leicht erzielen: man bringe 2—3 mm hohe Säulchen aus dem Rückenmarke eines Kindes auf drei Tage in die Golgi'sche Mischung, dann auf zwei Tage in die Silberlösung und kann mit grosser Wahrscheinlichkeit auf Erfolg rechnen. In keinem einzigen Falle versagte mir die Methode, stets erschienen mehr oder weniger Stützzellen imprägniert. Es kommen diese Zellen umso deutlicher zur Ansicht, als die Nervenfasern um diese Zeit schon wegen ihrer Markscheiden der raschen Imprägnation widerstehen und auch die Nervenzellen sich nur spärlich und dann auch stets ohne ein längeres Stück ihres Axons schwärzen. So wird das Bild der „Glia“ durch keine anderen Dinge verhüllt.

An den Bildern nun, die man erhält, und die ich namentlich

auf der Tafel I möglichst treu wiederzugeben bestrebt war, treten uns in vollendeter Klarheit und grosser Zahl die altbekannten Formelemente des medullaren Stützgerüstes, die Gliazellen (Deiters-sche, Golgi'sche Zellen) entgegen, und zwar wesentlich in der Form, wie wir sie aus vielen bisherigen auch vorgolgischen Schilderungen, vor allem aus den ersten genauen Beschreibungen von Deiters und Golgi (1871), dann aus denen von Boll, Kölliker, Jastrowitz, Gierke u. a. kennen; so liefern denn unsere neuen Erfahrungen in der Hauptsache eine Bestätigung früherer Anschauungen. Es handelt sich in der That um reichverzweigte, sternförmige, äusserst zierliche, zarte Gebilde, die in grosser Zahl über die graue und weisse Substanz verstreut sind. Diese Zellen bilden nun für sich allein das Stützgerüst des Rückenmarkes, sie bilden nicht den Hauptbestandteil, sondern die einzigen Elemente dessen, was man bisher „Neuroglia" genannt hat. Es giebt eigentlich gar keine „Neuroglia" im Sinne eines besonderen Gewebes, sondern nur eine besondere reichverzweigte, sternförmige Zellengattung im Gewirr des Gehirns und Rückenmarkes, deren reiche Ausstrahlungen in der grauen Substanz mit den anderen Elementen, den Nervenzellen, in kompliziertester Weise durcheinander geschlungen sind, in der weissen Substanz sich zwischen den Nervenfasern und ihren Bündeln derartig hindurchwinden, dass dadurch für die Fasern ein Stützgerüst zu stande kommt. Desgleichen giebt es keine selbständigen „Gliafasern", sondern nur Ausläufer von Gliazellen, gleichwie die Dendriten nicht als besondere Bildungen, sondern nur als die Fortsätze der Nervenzellen aufzufassen sind.

Diese Grundvorstellung, die wohl zuerst von Kölliker im Jahre 1862[1]) angedeutet, im wesentlichen aber in einer 1871 erschienenen vortrefflichen Arbeit Golgis[2]) zuerst in entschiedener und erschöpfender Weise ihren Ausdruck gefunden hat, bildet den Kernpunkt eines richtigen Verständnisses dessen, was man das Stützsystem der Centralorgane nennt. Das Festhalten dieser Anschauung wird nun gewiss vielen nicht leicht fallen, indem die herrschenden Vorstellungen, die sich namentlich im pathologischen Gebiete sehr breit gemacht haben, in dieser Richtung hindernd und störend wirken müssen. Denn noch immer wird vielfach die

1) A. v. Kölliker, Handb. d. Gewebelehre, 4. Aufl., 1862, p. 304.

2) C. Golgi, Contribuzione alla fina Anatomia degli organi centrali del sistema nervoso. Rivista clinica di Bologna, 1871—1872. Siehe Sammelwerk p. 25 ff.

Auffassung des „Stützgewebes" im wesentlichen von der Vorstellung beherrscht, als würde es sich dabei um eine Art selbständigen Stromas, um eine Grundsubstanz, um eine besondere, in sich zusammenhängende Gewebsgattung handeln, um etwas, worin die Nervenzellen und Fasern wie in eine fremde Zwischenmasse, ein differentes Medium, eingebettet sind, und das zugleich eine Art Kitt für sie darstellt. Die Hauptrolle spielt in dieser Auffassung eine ungeformte Kittsubstanz, die „Glia", die das eigentliche Bindemittel für die nervösen Elemente abgiebt; zu dieser mörtelartigen Zwischenmasse kommen nun als zu ihr gehörige, aber sekundäre Elemente besondere Zellen, „Gliazellen" und faserige Bildungen, „Gliafasern".

Wenn sich im Laufe der Jahrzehnte eine solche Auffassung allmählich eingebürgert hat, so ist das in der That nicht zu verwundern, musste doch die Unfähigkeit der meisten Methoden, die hier in Betracht kommenden Gewebselemente so wie sie wirklich sind, zur Ansicht zu bringen, unfehlbar zu einer solchen Vorstellung führen. Denn was zeigen uns etwa ungefärbte Schnitte des Rückenmarkes oder auch fast alle die gewöhnlichen Färbungen? Wir sehen die ungefärbten oder gefärbten Nervenzellen oder richtiger nur ihre Fragmente, denn die Ausläufer bleiben doch zumeist unsichtbar, eingebettet in ein bald homogenes, bald mehr oder weniger körnig oder faserig aussehendes Substrat, wie etwa die Knorpelzellen in ihre Intercellularsubstanz; in den weissen Strängen erscheinen die Nervenfaserbündelchen wie von einem zusammenhängenden, mit Kernen besetzten derben Balkenwerk getragen. Aus diesem, auf der Unvollkommenheit der Methoden beruhenden Bilde musste unvermeidlich die Vorstellung einer kitt- oder stromaartigen Grundsubstanz entspringen, und diese Vorstellung nun fand wieder ihren Ausdruck in der Bezeichnung jenes scheinbaren Grundgewebes als Neuroglia, d. h. als „Nervenkitt". Ich befürchte, dass wir die Vorstellung eines Mörtels nicht losbekommen werden, so lange wir an diesem Ausdrucke festhalten. Der Gedanke, dass wir es nicht mit einer besonderen Gewebsart, sondern nur mit einer besonderen Gattung von Zellen in dem centralen Zellen- und Faserfilz zu thun haben, würde meiner Überzeugung nach die kräftigste Förderung dadurch erfahren, dass wir die bisherigen einschlägigen Bezeichnungen fallen liessen und ganz frische, mit hergebrachten Trugvorstellungen nicht assoziierte heranzögen. Ich würde z. B. vorschlagen, alle bloss der Stützfunktion dienenden Zellen der Centralorgane Stütz-

zellen oder Zwischenzellen im allgemeinen, Spongiocyten[1]), ihre bei höheren Vertebraten verbreitetste Form immer nur als Spinnenzellen oder „Astrocyten“ zu bezeichnen und sich des Kollektivnamens all dieser Elemente „Neuroglia“ bloss cum grano salis zu bedienen, so lange wenigstens die richtigen Vorstellungen nicht gehörig gefestigt sind. Da dies Buch aber für weitere Kreise von Fachgenossen bestimmt ist und es vor allem auf ein allgemeines Verständnis abgesehen hat, will ich, zwar etwas inkonse-

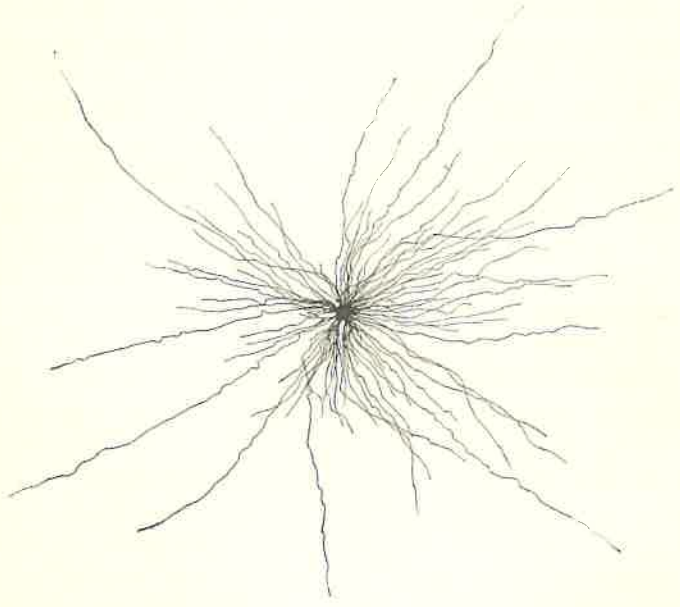

Fig. 20.
Spinnenzelle aus dem Rückenmarke eines 3/4jährigen Kindes.

quent, die hier befürworteten Namen abwechselnd mit den alten anwenden.

„Astrocyten“, sternförmige Zellen, sind die kleinen Elemente, die das Stützsystem des Rückenmarkes bilden, in der That (Fig. 20), kein anderer Vergleich giebt ihre Eigenart so treffend wie-

[1]) Dieser neue griechische Terminus technicus war schon niedergeschrieben, als ich durch die Freundlichkeit des Autors eine kleine Notiz erhielt (P. A. Fish, The Terminology of the Nerve Cell. Journal of Comparative Neurology, Vol. IV, 1894, p. 171), worin der gleiche Name für „Gliazelle“ vorgeschlagen wird. Ein merkwürdiges Zusammentreffen ist ferner, dass Fish auch für die Nervenzelle, gleich mir, Neurocyt empfiehlt, ein Wort, das aber in dieser Benützung schon älteren Datums ist. Den Nervenfortsatz nennt Fish Neurit.

der wie dieser. Zwar ist auch der von Jastrowitz[1]) eingeführte Namen „Spinnenzellen“ — ein Ausdruck, der sehr beliebt geworden ist — geeignet, eine richtige Vorstellung von ihrem Habitus zu vermitteln, doch ist Gierkes Vorwurf nicht unberechtigt, dass noch Niemand Spinnen mit so vielen Beinen gesehen hat, wie diese Zellen Fortsätze haben. Immerhin muss ich gestehen, dass mir dieser Name von den deutschen Bezeichnungen der Astrocyten noch der glücklichste zu sein scheint. „Pinselzellen“, wie sie Boll genannt hat, würde höchstens nur für eine bestimmte Form dieser Elemente passen, für diejenigen, bei denen die Fortsätze nur von der einen Seite der Zelle entspringen, für die überwiegende Mehrzahl ist der Ausdruck unpassend. Die Astrocyten sind beim Menschen zahlreicher und auch im Verhältnis zum Querschnitt des Rückenmarkes beträchtlich kleiner als bei den übrigen Säugern und Vertebraten, überhaupt kleine Elemente mit schmalem Protoplasmasaum um den Kern herum. Färbt man einen Rückenmarksquerschnitt mit Hämatoxylin, Nigrosin, Methylenblau u. s. w., so weist jedes Stückchen des Querschnittes zahlreiche scheinbar „freie“, mit allen diesen Farbstoffen sich stark tingierende Kerne auf. Diese Kerne nun, soweit sie nicht etwa denen von Nervenzellen oder Leukocyten entsprechen, gehören zu den Astrocyten. Der minimale Zellkörper und noch mehr die strahlige Verästelung haben die Eigenart, dass sie sich bei den meisten Färbungen gar nicht in distinkter Weise zu erkennen geben. Die relativ besten Bilder geben noch von den gewöhnlichen Tinktionen Karmin und Pikrokarmin.

Um so klarer tritt das ganze kleine Gebilde hervor an den Chromsilberbildern. Ein dichter Rasen von Ästen entströmt nach allen Richtungen hin dem unscheinbaren, oft gleichsam kaum mehr als einen Knotenpunkt der strahlenförmigen Verästelung darstellenden Zellleib. Die Äste haben etwas merkwürdig Straffes, Steifes an sich, und das ist es, was den Gliazellen ihr charakteristisches Gepräge aufdrückt. Dadurch unterscheiden sie sich hauptsächlich von den Nervenzellen; die Unterscheidung der beiden Zellarten bereitet im Rückenmarke dem geübten Auge in keinem Falle Schwierigkeiten. Die Nervenzellen haben mit ihren baumförmig verästelten, sich bei jeder Zelle anders verhaltenden Dendriten etwas Individuelles, etwas Freies an sich, die Gliazellen

[1]) Jastrowitz, Studien über die Encephalitis und Myelitis des ersten Kindesalters. Archiv f. Psychiatrie, Bd. III, 1871, p. 162.

etwas Schablonmässiges, Steifes. Hier herrscht nicht jene Mannigfaltigkeit wie bei jenen. Dieser verschiedene Habitus ist ja nur ein Spiegelbild der zwischen den beiden Zellengattungen bestehenden funktionellen Verschiedenheit: die Nervenzellen stellen gleichsam automatisch wirkende, mit einem reichen inneren Eigenleben ausgestattete, in ihrem Protoplasma die wunderbare Mannigfaltigkeit der Nervenvorgänge tragende Apparate dar, während die Astrocyten Elemente sind, die ohne innere Bewegung, die ihnen zukommende Aufgabe, sei nun diese bloss die Stützfunktion oder wie Pedro Ramón[1]), R. y Cajal[2]) und Cl. Sala[3]) meinen, die Bildung von Isolatoren, bloss durch ihre stumme Gegenwart, durch ihre physikalischen Eigenschaften, wie Fremdkörper, die in das von grossartigen inneren Regungen erzitternde Nervengewebe eingeschaltet sind, erfüllen. Ausserdem liegt schon ein unterscheidendes Merkmal, beim Menschen wenigstens, in den geringen Dimensionen des Zellkörpers, überhaupt in der Kleinheit der ganzen Zelle mit samt ihrer ganzen Verästelung im Vergleich zu den Neurocyten.

Man kann im Rückenmarke mit v. Kölliker (Lehrbuch p. 145) im allgemeinen zwei Astrocytenformen unterscheiden: Langstrahler und Kurzstrahler. Diese beiden Gattungen sind aber numerisch sehr verschieden vertreten; die Kurzstrahler bilden die Minderheit und kommen nur im Bereich der grauen Substanz vor. Es ist daher gerechtfertigt, der allgemeinen Schilderung der Spinnenzellen die überall verbreiteten, zahlreicheren Langstrahler zu Grunde zu legen.

Die Äste entspringen nun an diesen selten ganz gleichmässig verteilt ringsum vom Umfange des Zellkörpers; gewöhnlich heben sich in der Verästelung einzelne dichtere Büschel, wie „Strahlenbündel“ hervor. Zwischen den Ursprüngen der Äste und namentlich der stärkeren Stämmchen zeigt der Kontur des Zellkörpers bogenförmige Einziehungen. Die Äste sind im allgemeinen fein, namentlich mit den Dendriten verglichen, die meisten bei den typischen Langstrahlern von gleicher Stärke und was sehr bezeichnend ist, von Anfang bis Ende gleich dick. Einzelne Fasern zeichnen sich allerdings manchmal durch auffallende Breite aus.

---

[1]) P. Ramón, El encéfalo de los reptiles, Barcelona 1891, p. 10.

[2]) S. R. y Cajal, Significación fisiologica de las expansiones protoplasmáticas y nerviosas de las células de la sustancia gris. Rivista de ciencias médicas de Barcelona, 1891, Nr. 22 und 23.

[3]) Cl. Sala y Pons, La neuroglia de los vertebrados. Barcelona 1894, p. 39.

Im allgemeinen erscheinen sie an Golgi'schen Präparaten etwas dicker als an anderweitigen, was man vielleicht auf eine Umhüllung der Fäserchen mit der Chromsilbermasse zurückführen darf. Varikositäten kommen nicht vor, die Fasern zeigen das Verhalten von Nervenfasern und können mit solchen in der That, wenn sonst keine entscheidenden Anhaltspunkte vorliegen, verwechselt werden. In Wirklichkeit besitzen sie wahrscheinlich einen gestreckteren, geradlinigeren Verlauf; die kleinen zackigen Biegungen, die sie vielfach an Golgi'schen Präparaten, aber auch an anderen, zeigen, sind zum Teile wenigstens offenbar Schrumpfungsergebnisse, was schon daraus wahrscheinlich wird, dass sie an manchen Präparaten stärker ausgeprägt sind, an anderen fast ganz fehlen. Im allgemeinen kann man die Äste der Langstrahler, namentlich im Vergleich zu den Dendriten, als ungeteilt kennzeichnen, indessen kommt es öfters vor, dass ein Fortsatz sich spitzwinklig in zwei Äste gabelt, doch erfolgt dies immer in der Nähe der Zelle und wiederholt sich kaum. Deiters hat bekanntlich die Fortsätze der Gliazellen als reichverästelt geschildert, während Golgi (1871) ihnen jede Verzweigung und Teilung absprach. Die Wahrheit liegt zwischen diesen beiden Extremen, indessen doch viel näher zu Golgi's Darstellung.

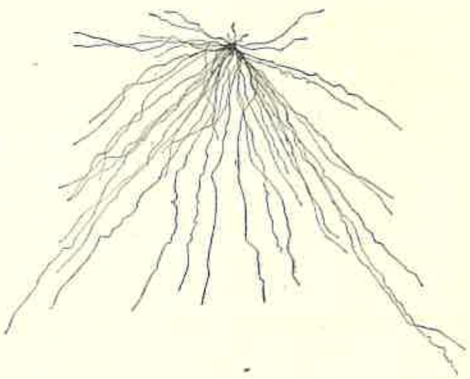

Fig. 21.

Spinnenzelle aus dem Rückenmarke des $^{3}/_{4}$ jährigen Kindes, mit einseitiger Entwickelung der Ausläufer.

Die Fortsätze der Astrocyten sind von verschiedener Länge, manche durchdringen, oft ganz isoliert, weite Gebiete des Markes, andere endigen schon ganz in der Nähe ihres Ursprunges, dazwischen findet man alle Übergänge. Am dichtesten stehen sie natürlich unmittelbar am Zellkörper; hier drängen sie sich häufig so stark zusammen, dass sie die Konturen des Zellkörpers ganz verdecken.

Wenn oben gesagt wurde, dass die Ausläufer der Astrocyten nach allen Richtungen hin ausgehen, so gilt das nur für die Mehrzahl der Zellen. An allen Stellen des Querschnittes findet man Astrocyten, bei denen die Äste eine einseitige

Entwickelung zeigen (Fig. 21), indem sie sich büschelartig nur auf die eine Seite beschränken, oder wie zwei Pinsel den beiden Polen einer spindelförmigen Zelle aufsitzen. Dies ist eine individuelle Verschiedenheit, indem wie gesagt, derartige Zellen überall auftauchen können, aber sie ist gleichzeitig eine lokale Besonderheit, da, wie wir hören werden, für gewisse Stellen, namentlich für die Randgebiete der grauen Substanz, das überwiegende Vorkommen derartiger Formen bezeichnend ist.

Alle Ausläufer der Astrocyten, die längeren wie die ganz kurzen, endigen mit freien Spitzen. Diese Thatsache hat wohl Deiters zuerst erkannt, Boll und namentlich Golgi haben sie aber genauer begründet. Bei Ranvier und Gierke trat wieder ein Rückschritt ein, indem sie eine anastomotische Verbindung der benachbarten Gliazellen untereinander annahmen. Dies stellt sich an der Hand der Golgi-Bilder sehr klar als ein Irrtum heraus. Die Astrocyten sind vollkommen unabhängige Gebilde, sie stellen ebenso wie die Nervenzellen, Einheiten für sich dar. Das Gewirr, das aus ihren Ästen hervorgeht und das namentlich in der grauen Substanz einen ansehnlichen Teil des Nervengewebes bildet, ist ebenso wie das Neuropilema ein Gefilze, ein Astropilema oder Spongiopilema und kein Netz.

Ist für die Darstellung der einzelnen Gliaelemente die Golgi'sche Methode weitaus das ausgiebigste Verfahren, so giebt sie über die Art und Weise, wie die Spinnenzellen und ihre Fortsätze, die „Gliafasern" quantitativ über den Rückenmarksquerschnitt verteilt sind, über ihr Kollektivverhalten, bei ihrer Eigenart, dass sie immer nur einzelnen Exemplaren einer auf dem Schnitt in grosser Zahl vertretenen Zellsorte ihre Gunst zuwendet, keinen sicheren Aufschluss. Gewisse Anhaltspunkte liefert auch das Chromsilberverfahren nach dieser Seite hin, denn man hat es bald heraus, dass da, wo mehr Gliazellen beisammenliegen, durchgehends auch mehr zur Imprägnation gelangen. Es besteht unzweifelhaft ein gewisser Parallelismus zwischen der Menge der imprägnierten Zellen und der Anzahl der an der betreffenden Stelle faktisch vorhandenen. Indessen hat ein solcher Schluss immer etwas Unsicheres an sich; in dieser Beziehung leistet die Golgi'sche Methode nicht das Gewünschte.

Hier sind andere Methoden am Platze; es sind in der letzten Zeit zwei Verfahren erfunden worden, die in dieser Beziehung Vorzügliches, ja vielleicht Unübertreffliches leisten. Die erste

stammt von Weigert; sie ist leider noch nicht so ausführlich veröffentlicht, dass man sie nachmachen könnte[1]). Die andere Methode, die mit der Weigert'schen offenbar nahe verwandt ist und ungefähr auch die gleichen Bilder, nur weniger klare, liefert, ist zwar in ihrer Anwendung zur Darstellung der Stützzellen des Nervensystems von Beneke[2]) eingeführt worden, stellt aber auch nichts anderes, als eine geringe Modifikation einer anderweitigen Weigert'schen Methode dar, nämlich der sog. Weigert'schen Fibrinfärbung. Die ursprüngliche Weigert'sche Fibrinfärbung besteht bekanntlich in folgendem[3]): Die in Alkohol gehärteten Objekte werden in feine Schnitte zerlegt. Färbung der Schnitte 5—15 Minuten lang in konzentrierter Anilinwasser-Gentianaviolett-Lösung. Die Schnitte werden in 0,6 % Kochsalzlösung abgespült und auf dem Objektträger mit Fliesspapier abgetrocknet. Wiederholtes Auftröpfeln von Jodjodkaliumlösung (1 : 2 : 100) auf den Objektträger, darauf wieder Abtrocknen mit Fliesspapier. Nun folgt die Entfärbung mit Anilinöl-Xylol (2 : 1), Entfernung des Anilin-Xylols durch reines Xylol. Die Schnitte werden in Kanadabalsam aufgehoben. Bei diesem ursprünglichen Verfahren wird bei längerer Einwirkung des Anilinxylols alles, bis auf das Fibrin, und bis auf etwa vorhandene Organismen, entfärbt. Beneke hat es nun zur Darstellung der Gliazellen nur insofern modifiziert, als er das Mischungsverhältnis der beiden zur Entfärbung dienenden Stoffe etwas abgeändert hat, indem er mehr Xylol nimmt (Xylol 3: Anilinöl 2), wodurch er die entfärbende Kraft des Gemisches soweit abschwächt, dass der Farbstoff nunmehr von vielen Gewebsbestandteilen, wie Bindegewebsfasern, elastischen Fasern, Muskeln, Epithelien (Kromayer) und auch Gliazellen festgehalten wird.

Der Freundlichkeit des Herrn Prof. Weigert verdanke ich mehrere nach seiner Gliamethode gefärbte Präparate aus dem menschlichen Rückenmarke. Die Methode giebt in der That äusserst instruktive und zierliche Bilder, und es ist im Interesse namentlich

---

1) Weigert giebt in seinem Neurogliaaufsatze (Anat. Anz. 1890, p. 550) nur soviel an, dass die Präparate mit Metallsalzen, die eine organische Säure enthalten, gebeizt werden; sowohl in Betreff der Metalle als in Bezug auf die Säure führen verschiedene Stoffe zum Ziele. Gefärbt werden die Schnitte mit Methylviolett; sie werden dann mit Jod behandelt und mit Anilinölxylol ausgewaschen.

2) Beneke, Über eine Modifikation des Weigert'schen Fibrinverfahrens. Anat. Anz., Jahrg. VIII, 1893, Suppl. p. 165.

3) Siehe Friedländer-Eberth, Mikroskopische Technik, 5. Auflage, Berlin 1894.

der pathologischen Untersuchung des Nervensystems der Wunsch dringend zu betonen, dass sie von ihrem Urheber bald zur allgemeinen Kenntnis gebracht werde, mag sie auch noch nicht zu jenem subtilen Grade der Vollendung vervollkommnet sein, in der Weigert eine Conditio sine qua non erblickt für eine Methode, die aus seinen Händen als zur Publizität reif hervorgehen soll. Die Gliafasern nehmen eine dunkelblaue Färbung an, ihr dichtes, feines Gespinne durchzieht den Schnitt, und schon bei schwacher Vergrösserung, ja teilweise schon mit freiem Auge betrachtet, fallen die Stellen, wo sich eine grössere Ansammlung von Gliaelementen findet, als dunklere Flecke auf. Die Nervenzellen samt ihren Ausläufern, sowie auch die Zellkörper der Astrocyten bleiben total ungefärbt; das weiche Protoplasma ebenso wie die Substanz des Achsencylinders widersteht überhaupt der Färbung. Ist aber die Methode im Rückenmark eine exquisit selektive, so ist sie es nicht in weiterem Sinne. Es färben sich damit ausserhalb des Rückenmarkes vielfach Bindegewebs- und elastische Fasern. Dies ist auch an den mir von Weigert überlassenen Präparaten, an denen teilweise auch die Rückenmarkshäute mit geschnitten waren, ohne weiteres ersichtlich. Wenn sich aber so verschiedenartige, nur in ihrer Form, ihrer physikalischen Beschaffenheit übereinstimmende Elemente färben, so wird uns das Wesen jener eklektischen Eigenschaft klar: der Erfolg der Färbung ist bei der Weigert-schen Methode an einen bestimmten Dichtigkeitszustand, an eine bestimmte Form der Elemente geknüpft, indem nur dichtere, zu gleichmässigen, zarten Fasern konzentrierte Teile den Farbstoff zu fesseln vermögen. Giebt doch auch Beneke an, dass seine, der Weigert'schen offenbar nahestehende Methode alle fibrillären Gebilde, wie fibrilläres Bindegewebe, elastisches Gewebe, Knochenfibrillen, Sharpey'sche Fasern und auch Gliafasern zu färben im stande sei, während „das Zellprotoplasma sich überhaupt nicht färbt". Es handelt sich also keineswegs um eine Gliareaktion.

In dieser Hinsicht also ist die Färbung als eine unvollkommene zu bezeichnen. Sie ist nicht geeignet, eine Zelle ganz darzustellen, sondern sie wird nur faserartige Gewebsbestandteile, die gewisse physikalische Bedingungen erfüllen, zur Ansicht bringen können, und zwar isoliert, als selbständige Gebilde, ob sie thatsächlich mit Zellen zusammenhängen oder nicht. Der Aufgabe, eine Gliazelle komplet zu färben, ist sie nicht gewachsen, sie färbt nur einen Teil davon, nur ihre fibrillären Anhänge, die „Gliafasern". Dies ist aber eine gefährliche Eigenschaft, denn sie kann leicht zu der irrtümlichen Vorstellung führen, als wären diese „Gliafasern" etwas selbständiges, wie etwa die Fasern des fibrillären Bindegewebes, als wären dann die „Gliazellen" nur als sekundäre, davon unabhängige Gebilde in dieses Fasergewirr eingeschlossen. Ein solcher Schluss ist wirklich auch neuerdings im Anschluss an eine frühere Angabe Ranvier's aus diesen Bildern gezogen worden. Eine andere Gefahr besteht darin, dass diese Bilder die Vorstellung, als ob es sich um eine Art von Bindegewebe handelte, wieder beleben könnten. Man muss diese Bilder allemal in der Phantasie in dem Sinne ergänzen, dass man die gefärbten Fasern auf ungefärbt gebliebene Zellen, aus denen sie radiär hervorspriessen, zurückführt. Um diese Zellen selbst sichtbar zu machen, dazu bedarf es anderweitiger Verfahren, der Schnittfärbungen, Isolierungen und vor allem der Golgi'schen Methode. Wenn alle anderen Methoden, Karminfärbungen, die Golgische Färbung, besonders aber die Isolierung dieser Elemente, wie sie schon im Jahre 1863 von Kölliker, dann später in vollendeterer Weise von Deiters, Boll und anderen verwertet worden ist, in übereinstimmender Weise strahlenförmige Zellen, die Fasern von einem centralen Zellkörper als seine Äste ausgehend zeigen, so wird, glaube ich, diese eine Methode nicht geeignet sein, die bestehende Auffassung umzustürzen, umsoweniger, als ja die Erscheinung, dass man bei ihr lauter isolierte Fasern sieht und nicht mit Zellen verbundene, leicht aus der oben erwähnten besonderen Eigenart der Methode erklärt werden kann. Wenn Weigert als Stütze der Ranvier'schen Ansicht die Beobachtung geltend macht, dass man an den blauen Gliapräparaten die „Gliafasern" nicht regelmässig wie Radien von einem ungefärbten oder schwach als Zelle gefärbten Mittelpunkt ausstrahlen, sondern sie vielfach über die Zellkörper hinwegziehen sieht, so ist demgegenüber einerseits zu betonen, dass jene Schnitte viel zu fein sind, um das Ausströmen der Gliafasern von Zellen zu demonstrieren, sie zeigen nur Fragmente der Fortsätze, andererseits aber, dass die Astrocytenfortsätze so dicht durcheinandergeschlungen sind, dass jeder Astrocyt nicht nur von seiner eigenen Verästelung umgeben, sondern auch von den Ausläufern anderer Astrocyten umflochten erscheinen muss.

Die Geschichte der „Neuroglia" reicht bis ins Jahr 1811 zurück. Keuffel [1]), dessen Arbeit in diesem Jahre erschien, hatte das Rückenmark

1) G. G. Th. Keuffel, Über das Rückenmark. Reil's Archiv, Bd. 10, 1811, p. 123.

mit Kali causticum behandelt und die erweichte Marksubstanz durch eine Art Auspinselung entfernt. Hierdurch gelang es ihm, die Gliabalken, die die weisse Substanz durchsetzen, in Form eines Maschenwerkes darzustellen. Keuffel nannte die Substanz dieses Netzwerkes, das sich auch in die graue Substanz zu erstrecken schien und das er auf Fortsetzungen der Pia mater zurückführte, „verdichteten Zellstoff“ oder „Neurilem des Rückenmarkes“.

Die erste eigentlich histologische Angabe findet sich erst bei Arnold 1844[1]. Arnold kennt schon eine „körnige Grundmasse“, in die die Nervenzellen eingebettet sind.

Viel ausführlicher ging im Jahre 1853 auf diese Substanz Virchow[2]) ein und vindicierte ihr eine allgemeine Verbreitung in den Centralorganen des Nervensystems. Virchow sah sie ungefähr ebenso wie Arnold: als weiche, amorphe oder körnige Zwischenmasse; ausserdem beschreibt er in dieser Masse eingestreut dazugehörige runde oder linsenförmige Zellen. Es ist dies wohl die erste, wenn auch noch unvollkommene Angabe über Gliazellen, und man darf daher Virchow als den eigenlichen Entdecker dieser Elemente bezeichnen. Nach Virchow's Ansicht handelt es sich in der von ihm später[3]) als Neuroglia bezeichneten Substanz um ein der „Bindesubstanz im Grossen“ zugehöriges Gewebe, während R. Wagner[4]), der sie ungefähr ebenso beschreibt, wie Virchow, es nicht für unmöglich hält, dass sie nervös ist und zur Bildung der Nervenzellen diene, eine Ansicht, die später namentlich von Henle und Merkel[5]) energisch unterstützt und näher ausgeführt wurde.

Bei Bidder (1857)[6]) kompliziert sich das Bild des Stützgewebes. Es besteht nunmehr nicht nur aus einer reichlichen Menge einer formlosen Masse, die in frischem Zustande als gallertig durchscheinend, nach Chromsäureeinwirkung als körnig geschildert wird, sondern in diese eingeschlossen noch aus längs- und querverlaufenden Fasern sowie auch aus vielen Bindegewebskörperchen, die teils fortsatzlos, teils mit 2—3 Ausläufern versehen und mit einander anastomosierend geschildert werden. Die Balken der weissen Substanz fasst Bidder als Fortsetzungen der Pia mater auf. Bidder schreibt der „Bindesubstanz“ offenbar einen viel zu grossen Anteil an der Bildung des Rückenmarkes zu. Das Hauptverdienst der Bidder'schen Arbeit möchte ich darin erblicken, dass in dem bis dahin als amorph oder körnig geschilderten „Rückenmarksstroma“ die Gegenwart von Fibrillen betont wird und dass die Zellen hier zuerst teilweise wenigstens als sternförmig, mit Ausläufern versehen erscheinen, wobei allerdings zu bemerken ist, dass in der grauen Substanz Kölliker schon 1855 solche Zellen beschrieben hat.

1) Fr. Arnold, Handbuch der Anatomie. Bd. I, Freiburg i. Br., 1844, p. 260.

2) R. Virchow, Über eine im Gehirn und Rückenmarke gefundene Substanz mit der chemischen Reaktion der Cellulose. Archiv f. pathol. Anat. u. Physiol., Bd. VI, 1853, p. 136.

3) R. Virchow. Gesammelte Abhandlungen, Frankfurt, 1856, p. 890.

4) R. Wagner, Neurologische Bemerkungen. Göttinger Nachr., 1854, Nr. 3, p. 28

5) J. Henle u. Fr. Merkel, Über die sog. Bindesubstanz der Centralorgane des Nervensystems. Zeitschr. f. ration. Medizin. Bd. 34, 1868, p. 48.

6) F. Bidder und C. Kupffer, Untersuchungen über die Textur des Rückenmarkes, Leipzig, 1857.

Wir müssen nun mehrere weniger bedeutsame Angaben über unsern Gegenstand überspringen, um wieder zu einem wichtigen Moment in der Geschichte der „Neuroglia“ zu gelangen. Es war gewiss ein grosser Fortschritt, als Kölliker[1]) im Jahre 1863 die heute in der Hauptsache als richtig erkannte Ansicht vertrat, dass das Stützgewebe des Rückenmarkes aus nichts anderem als aus einem Komplex sternförmig verästelter Zellen bestehe, die sich mit einander in solcher Weise verbinden, dass sie ein Netzwerk für die nervösen Elemente darstellen. Kölliker nahm damals gleich Bidder, Anastomosen zwischen den Zellfortsätzen an, also ein wirkliches Netz.

Deiters[2]) gebührt aber unstreitig das Verdienst, die Elemente dieser Stützsubstanz, die Astrocyten, zuerst auf dem Wege der Isolation in annähernd richtiger Form dargestellt zu haben. Freilich giebt Kölliker schon im Jahre 1855 Abbildungen solcher isolierten Zellen, die ersten Abbildungen, die darüber vorliegen, doch sind an den Kölliker'schen Figuren die Fortsätze noch etwas zu spärlich ausgefallen. Aber auch die Deiters'sche Zeichnung ist nicht vollkommen; die Äste sind auch hier nicht in gehöriger Zahl dargestellt, auch sind sie viel zu gewunden, viel zu stark verzweigt und viel zu zart; die Zelle giebt den Typus des gewöhnlichen langstrahligen Astrocyten nicht getreu wieder, höchstens kommt sie dem Habitus der Kurzstrahler nahe. Auch die im Text gegebene Darstellung der Stützsubstanz lässt viel zu wünschen übrig. Deiters kehrt p. 38 wieder zu der „porösen, körnig aussehenden Grundmasse“ zurück, er giebt in ihr die Gegenwart von zahlreichen „freien Kernen“ zu und nimmt freie Fasern, ursprünglich als Zellenausläufer angelegt, dann aber von diesen sich emanzipierend, an. Im Innern nun dieses Schwammgewebes liegen die Zellengebilde des Stützsystems als verästelte Bindegewebskörper, die mit einander nicht anastomosieren. Schliesslich lässt Deiters von der Pia mater her zahlreiche Balken in die weisse und teilweise sogar bis in die graue Substanz hinein eindringen, eine Anschauung, an der wir heute auch nicht mehr festhalten können.

Erst sieben Jahre nach der Veröffentlichung des Deiters'schen posthumen Werkes sollte eine Darstellung der Stützzellen des Rückenmarkes erscheinen, die wir heute, im Besitze der seitherigen Erfahrungen und namentlich derjenigen, die die Golgi'sche Methode vermittelt hat, in den wesentlichsten Punkten als zutreffend bezeichnen können. Das Jahr 1871, in dem Golgi's Arbeit „Beitrag zur feineren Anatomie der Centralorgane des Nervensystems“[3]) erschien, eine Abhandlung, die ausschliesslich dem Stützgewebe der Centralorgane gewidmet ist, bezeichnet daher in der That einen Markstein in der Entwickelung unserer einschlägigen Kenntnisse. Es sei vorweg bemerkt, dass Golgi's Darstellung noch in die Zeit vor der Erfindung der von ihm später eingeführten Chromsilbermethode fällt. Sie beruht hauptsächlich auf dem Studium von Zupfpräparaten und Schnitten, ungefärbt oder mit Karmin

1) A. Kölliker, Handbuch der Gewebelehre, 4. Aufl., 1863, p. 304—306, 5. Aufl., 1867, p. 266.

2) O. Deiters, Untersuchungen über Gehirn und Rückenmark, 1865, p. 44.

3) C. Golgi, Contribuzione alla fina anatomia degli organi centrali del sistema nervoso. Rivista clinica di Bologna, 1871. — S. Sammelwerk, p. 25 ff.

behandelt. Golgi's Arbeit ist leider nicht so bekannt geworden, als sie es verdient; Verf. selbst muss gestehen, dass er sie erst jetzt, nachdem sie in deutscher Übersetzung erschienen ist, in extenso kennen lernte. Golgi führt mit grosser Genauigkeit bei der Hirnrinde, der Kleinhirnrinde und auch dem Rückenmarke die von Kölliker angeregte Auffassung durch, dass das „interstitielle Stroma" lediglich aus verzweigten Zellen besteht. Die Schilderung dieser Elemente sowie auch ihre bildliche Wiedergabe ist so vortrefflich, dass, wenn man eine persönliche Bezeichnung zur Benennung der Astrocyten heranziehen will, sie nicht anders als Golgi'sche Zellen heissen können. v. Kölliker führt sie auch mit Recht unter diesem Namen an; indessen hat der berühmte italienische Forscher schon so vielerlei andere Zellen entdeckt, die teilweise schon nach ihm benannt werden, dass es meiner Ansicht nach angezeigt ist, um Verwechselungen vorzubeugen, von jenem persönlichen Namen abzusehen. Golgi schildert jene Elemente als kleine Zellen, von denen aber zahlreiche starre, glänzende, dünne Fasern ausstrahlen. Diese Fasern sind unverästelt und bilden keine Anastomosen miteinander. An den interstitiellen Zellen der weissen Substanz sieht man eine so ungeheuere Menge von solchen Fortsätzen, dass man aus ihrem bündelartigen Zusammentreten allein die „Septa" der weissen Substanz erklären kann, ohne auf Einsenkungen der Pia rekurrieren zu müssen. In der grauen Substanz schildert Golgi die Fortsätze im allgemeinen als weicher und zarter als in der weissen, namentlich sind sie sehr zart an den Zellen, die mitten zwischen den Nervenzellengruppen liegen. Vielleicht kann man diese Angabe schon als einen Hinweis auf die weiter unten zu besprechenden „Kurzstrahler" auffassen. — Freilich wird ein Wort nach unseren heutigen Kenntnissen in der gesamten Golgischen Darstellung durch ein anderes ersetzt werden müssen. Golgi bezeichnet die Spinnenzellen konsequent als „Bindegewebszellen", ihren Komplex, die „Glia", als Bindesubstanz, wenn er auch bemerkt, dass dieses Gewebe dem gewöhnlichen Bindegewebe nicht ohne weiteres gleichgestellt werden könne. Heute weiss man aber, dass man es hier nicht mit mesodermalen Bindegewebszellen, sondern mit ektodermalen Elementen zu thun hat, wenn sie auch in ihrer Form noch so sehr das Verhalten von sternförmigen Bindegewebszellen aufweisen. Es handelt sich eben um eine „Konvergenzerscheinung".

Die äusserst umfangreiche, mühevolle Arbeit Gierke's [1]), die ausführlichste Publikation über unsern Gegenstand in den letzten zwanzig Jahren, bedeutet, so genau sie auch in mancher Hinsicht ist, doch im ganzen keinen wesentlichen Fortschritt gegenüber Golgi's Darstellung, ja in vielen Beziehungen kann sie sogar von einem retrograden Charakter nicht frei gesprochen werden. Dies lehrt vor Allem ein Blick auf die Abbildungen der Gierke'schen Arbeit. Solche Stützzellen, wie sie in Fig. 1a und 2 abgebildet sind, giebt es im Rückenmarke fürwahr nicht. An diesen Figuren sieht man die Fortsätze der Spinnenzellen unter wellenförmigem Verlauf sich reichlich baumförmig verästeln und, allmählich zarter werdend, sich schliesslich, namentlich in Fig. 1a, in viele unmessbar feine, netzförmig verbundene Reiserchen auflösen. Auch die „Kurzstrahler" sehen nicht so aus. Die Figuren illustrieren natürlich nur die gleichlautende Beschreibung des Textes. Alle Stützzellen sollen mit einander durch Anastomosen zusammenhängen, eine Annahme, die

[1]) H. Gierke, Die Stützsubstanz des Centralnervensystems. Archiv f. mikrosk. Anat. Bd. 25, 1885. p. 441 und Bd. 26, 1886, p. 129.

man ja schon seit Deiters', Golgi's und Boll's [1]) Mitteilungen für abgethan halten konnte. Gliazellen mit drei, ja sogar noch mehr Kernen, werden als sehr häufig angegeben. (?) Dass alle Stützzellen, die Körper wie die Fortsätze, ja sogar die Kerne, bei völlig ausgewachsenen Menschen und Tieren verhornt sein sollen, ist eine Behauptung, für die Gierke die histochemischen und tinktoriellen Beweise schuldig bleibt. Der grösse Irrtum aber, der unserer Überzeugung nach den Gierke'schen Vorstellungen über die Zusammensetzung der Stützsubstanz anhaftet, ist der, dass er den Schwerpunkt bei der „Glia" auf eine weitverbreitete, die Hauptmasse dieses Gewebes ausmachende ungeformte Grundsubstanz legt, bei der er sogar die Entstehung verfolgt zu haben angiebt, indem er sie aus einer allmählichen Umwandlung der Zellkörper ableitet.

Gleichwohl ist Gierke's Arbeit in vielen Einzelheiten als höchst verdienstvoll zu bezeichnen; ihr Hauptverdienst besteht vor allem darin, dass das gesamte Stützgerüst des Centralnervensystems mit Entschiedenheit als ektodermal, von jeder bindegewebigen Beimischung frei hingestellt wird.

Im Jahre 1890 hat Weigert im X. Internationalen Medizinischen Kongress zu Berlin einen Vortrag gehalten, der bald darauf als kurzer Aufsatz im Anatomischen Anzeiger erschien [2]), worin er auf Grund seiner neuen, oben besprochenen Gliafärbung zuerst eine verlässliche Darstellung der quantitativen Verteilung der „Gliafasern", d. h. der Spinnenzellenfortsätze, über die verschiedenen Teile des Rückenmarksquerschnittes gab. Die Weigertschen Mitteilungen stellen eine dankenswerte Bereicherung unserer Kenntnisse dar, mit der Beschränkung freilich auf die genannte topographische Frage, denn mit der Auffassung, die sich Weigert von den Beziehungen der Gliafasern zu den Gliazellen gebildet hat und die sich namentlich an die Ranvier's [3]) anschliesst, können wir uns nicht einverstanden erklären.

Mit Gierke und Weigert schliesst die „vorgolgische Periode" in der Entwickelung unseres Wissens über den Bau des cerebro-spinalen Stützsystems ab. Eine rege Thätigkeit wird nun mit Hilfe der Chromsilbermethode entfaltet, die bald zu wichtigen Resultaten führt. Diese Resultate lassen sich in zwei Punkte zusammenfassen:

1. Durch die unvergleichlich klaren Golgi-Bilder der fertigen Astrocyten werden einige früheren Darstellungen, vor allem die Golgi's aus dem Jahre 1871 und teilweise die von Boll bestätigt und vollkommen sichergestellt.

2. Auf die Histiogenese der Gliazellen fällt ein neues Licht, ihre Herkunft aus dem Ektoderm erscheint gesichert und alle Einzelheiten ihrer Entwickelung werden aufgeklärt. Hier wollen wir einstweilen abbrechen, um erst weiter unten den Faden unserer geschichtlichen Darstellung wieder aufzunehmen.

---

[1]) F. Boll, Die Histologie und Histiogenese der nervösen Centralorgane. Archiv f. Psychiatrie u. Nervenkrankh. Bd. IV, 1874, p. 1.

[2]) C. Weigert, Bemerkungen über das Neurogliagerüst des menschlichen Centralnervensystems. Anat. Anz. Jahrg. V, 1890, p. 543.

[3]) L. Ranvier, De la névroglie. Comptes rendus de l'Acad. des Sc. Tome 94, 1882, p. 1536. Ferner: Archives de physiologie normale et pathologique, 1883, p. 177. — Technisches Lehrbuch der Histologie. Übersetzt von Nicati und v. Wyss. Leipzig, 1888, p. 972.

Die Astrocyten zeigen je nach den einzelnen Provinzen des Querschnittes gewisse lokale Verschiedenheiten, die sich teils auf Grösse und Verästelungsweise der einzelnen Elemente beziehen, teils darauf, wie dicht gedrängt sie liegen und wie sie angeordnet sind.

Wir wollen hier zunächst von den Ependymzellen, die ja eigentlich auch in das Gebiet des Stützsystems gehören, absehen, da es zweckmässiger scheint, sie im Anschluss an die Histogenese des Stützsystems zu besprechen.

Die stärkste Anhäufung von Spinnenzellen findet sich im mittleren, kommissuralen Abschnitt der grauen Substanz, im Gebiet der sog. Substantia gelatinosa centralis und auch etwas weiter seitlich. Dies geht Hand in Hand damit, dass hier Nervenelemente, sowohl Zellen wie Fasern, ganz fehlen; das Gebiet, das entwickelungsgeschichtlich allen Nervenzellen des Markes zur Bildungsstätte dient, entbehrt selbst im reifen Zustande völlig solcher. In die Lücke treten die Stützelemente ein. An Weigert'schen Markscheidenfärbungen präsentiert sich diese Zone namentlich in ihrem centralen Teil als sattgelb gefärbtes, von keiner Faser durchsetztes Feld. An Kernfärbungen, wie z. B. Thionin, Magentarot u. s. w., erscheint der Centralkanal, vorausgesetzt, dass der Schnitt nicht zu dünn ausgefallen ist, umlagert von gedrängt stehenden Kernen, deren Masse sich unter allmählicher Auflockerung auch seitlich, bis zur Einmündung der Kommissur in die Säulen, erstreckt. Diese vielen Kerne entsprechen den Kernen der hier massenhaft angesammelten Spinnenzellen. Aber nicht nur durch ihre Zahl, auch durch ihre Grösse zeichnen sich die Astrocyten dieses Abschnittes aus, und zwar bezieht sich diese Grösse weniger auf den Zellkörper, der zwar auch etwas ansehnlicher ist als anderwärts, als auf den Reichtum ihrer Verästelung, auf ihre langen, kräftigen, steifen und stets ungeteilten Fortsätze. Was aber an diesen Zellen das auffälligste ist, das ist die Anordnung ihrer Fortsätze. Die medialen davon schlagen nämlich eine konzentrische Richtung um den Centralkanal herum ein und verfilzen sich in dessen Umkreis zu einem dichten, regelmässigen Faserkranz. Dieser cirkuläre Filz ist es, der der erwähnten Substanz, nebst ihrer Armut an Nervenelementen, ihr charakteristisches Gepräge verleiht; hierdurch nimmt die Substanz bei der neuen Weigert'schen Gliafärbung jenen dunkelblauen Ton an, der sie, wie auch Weigert betont, schon bei schwacher Vergrösserung aus dem sonst heller gefärbten Querschnitt hervortreten

lässt. Die konzentrische Faserung in der Umgebung des Centralkanals ist übrigens schon mehreren früheren Forschern aufgefallen, so hat Stilling[1]) schon im Jahre 1842 an dieser Stelle als „Ringkommissur" eine Schicht von „Cirkulärfasern der feinsten Art" beschrieben.

An der Bildung des cirkulären Filzes beteiligen sich aber nicht nur die Zellen, die unmittelbar unter dem Ependym des Centralkanales liegen, sondern auch solche, die etwas mehr abseits davon ihren Sitz haben. Diese senden dann dichte Büschel an den Centralkanal heran, deren Fasern in der Nähe des Ependyms angekommen nach zwei Seiten hin schön auseinanderweichen und in die konzentrische Richtung einlenken.

Betrachten wir den Faserring an Präparaten, wo er vollkommen imprägniert ist, so erkennen wir, dass er seine volle Dichtigkeit nicht gleich an dem Ependym, nicht knapp unter den basalen Enden der Ependymzellen besitzt. Unmittelbar darunter liegt nämlich zuerst ein zwar auch faserreicher, aber doch nicht so dichter, hellerer Streifen, und dann erst folgt der dichte Faserkranz. In der Richtung gegen die Seitenteile hin nimmt das Fasergewirr an Dichtigkeit allmählich ab. Es ist noch zu bemerken, dass die konzentrische, in der Querebene des Rückenmarkes gelegene Richtung nicht die einzige ist in dem periependymalen Fasergefilze. Die Astrocyten sind auch hier, wie überall anderswo, nach allen Seiten hin verästelt, sie entsenden, die meisten wenigstens, ihre Fortsätze nicht nur in der Querebene, sondern auch nach der Längsachse des Rückenmarkes; dies erkennt man ohne weiteres an Längsschnitten. So sind auch in dem periependymalen Stützgewebe vielfach andere Faserrichtungen vertreten. Man findet Längsfasern u. s. w., nur herrscht die cirkuläre weitaus vor. — Angesichts der im Vorstehenden dargelegten Struktur des centralen Gebietes wäre es meiner Ansicht nach hoch an der Zeit, den ihm anhaftenden alten Stilling'schen Namen „Substantia gelatinosa centralis" durch einen passenderen zu ersetzen, da diese Region doch nichts Gelatinöses an sich hat.

Die Stützzellen, die zwischen dem Centralkanal und dem Grunde der vorderen Fissur liegen, senden oft einige dichte Büschel quer durch die vordere Kommissur hindurch und bilden so vor dem Centralkanal eine „Spinnenzellenkommissur"; auch hinter dem Centralkanal findet sich, wenn auch weniger ausgesprochen, eine ähnliche

[1]) B. Stilling und Wallach, Untersuchungen über die Textur des Rückenmarkes. Leipzig 1842, p. 23.

Anordnung vor. Wesentlich kompliziert wird noch das Stützgerüst im Bereich der vorderen Kommissur durch sagittal gestellte, bipolare Astrocyten, deren Äste in ein vorderes und hinteres Büschel zusammengefasst sind, wovon ersteres zur vorderen Fissur geht, letzteres von der vorderen Seite her in die cirkuläre Faserung einmündet. Die Spinnenzellen, die in der Nähe der Hinterstränge im Bereich der sogenannten hinteren grauen Kommissur sitzen, lassen ihre Ausläufer in grosser Zahl in die vorderste Spitze der Hinterstränge einströmen; die medialen davon kreuzen sich im Septum posterius. Dieses Septum besteht, wie wir noch sehen werden, aus einem Bündel von Ependymzellen, daneben aber wird es sehr wesentlich verstärkt durch Spinnenzellen, die sich in ihm in seiner ganzen Länge eingestreut finden, sowie auch durch die trichterförmig in das Septum einmündenden medialen Fortsatzbüschel der Spinnenzellen, die hinter dem Centralkanal, zu beiden Seiten neben dem vordersten Teil des Septums angehäuft sind.

In der eigentlichen grauen Substanz, d. h. den Hörnern kommen zwei Spinnenzellenformen vor: die typischen Astrocyten, wie sie oben geschildert wurden, und eine zweite, schon erwähnte Form, die unlängst von Kölliker genauer beschrieben wurde. Kölliker nennt die hier in Rede stehenden Stützzellen „Kurzstrahler" im Gegensatz zu den typischen „Langstrahlern" und charakterisiert damit in treffender Weise ihre auffallendste Eigenschaft. Denn was sie hauptsächlich auszeichnet, das ist die Kürze ihrer Ausläufer; sie verhalten sich zu den Langstrahlern wie Zwerge zu normalen Individuen. Aber die Äste sind nicht nur sehr kurz, sondern auch sehr zart; sie erscheinen weniger strahlenförmig als vielmehr in der Form eines die Zelle umgebenden dichten Rasens, eines echten Buschwerkes. Dabei sind sie zu Varikositäten geneigt, wodurch die ganze Zelle mit ihrer Verästelung manchmal einen merkwürdig körnigen Habitus erhält, der allerdings sehr oft noch durch eine unvollkommene Imprägnation gesteigert wird. Denn diese Zellen schwärzen sich, wie auch Kölliker bemerkt, selten so rein und tadellos, wie die Langstrahler. Ein weiteres Charakteristikum besteht hier noch darin, dass die Ästchen oft verzweigt sind und dass sie sich gegen ihre Spitzen hin allmählich verdünnen. Die Zellen bilden einen ganz eigenartigen, von den gewöhnlichen Astrocyten abweichenden Typus, daher sie von diesen schon bei schwacher Vergrösserung leicht unterscheidbar sind. Häufig imprägnieren sie sich nicht in dem satten schwarzen Ton, wie die anderen Astrocyten, sondern mehr hellbraun; derart imprägnierte Kurzstrahler geben aber dann die besten Bilder, indem die Aste ganz fein, von den sonst so häufigen körnigen Niederschlägen ganz oder relativ frei zur Ansicht gelangen.

Ich habe diese Form nicht gleich bei der allgemeinen Schilderung der Astrocyten beschrieben, um nicht etwa die Vorstellung zu erwecken, als handelte es sich um eine überall gleich stark vertretene, gleichberechtigte Zellengattung. Dies scheint nach meinen Erfahrungen doch nicht der Fall zu sein, ich glaube, dass die Kurzstrahler im Rückenmarke mehr nur eine lokale Abart darstellen, indem sie nur in den grauen Hörnern vorkommen, aber auch hier nicht die einzigen Elemente bilden, denn es kommen daneben noch wenigstens in gleicher Zahl auch typische Langstrahler vor. In der grauen Substanz sind sie nicht auf eine bestimmte Gegend beschränkt, sondern können sowohl im Vorderhorn, im Mittelgebiet der grauen Substanz, wie auch im Hinterhorn auftauchen, am häufigsten treten sie aber doch, wie mir scheint, im Vorderhorn, und zwar speziell im Gebiet der hier gelegenen starken Zellenanhäufungen, mitten zwischen den motorischen Zellen in die Erscheinung. Der mittlere, Kommissuralteil der grauen Substanz entbehrt solcher Elemente vollständig, die sagittale Linie, die den medialen Rand der Vorderhörner mit dem der Hinterhörner verbindet, bildet die Schranke für ihr Auftreten. Ebenso fehlen sie vollkommen in der weissen Substanz.

Die Langstrahler, die sich in der grauen Substanz finden, kommen in allen Variationen vor, mit rundlichem oder mehr länglichem Zellkörper, mit allseitig ausstrahlenden oder nur einseitig oder bei spindelförmigem Zellkörper von den beiden Polen ausgehenden Fortsätzen. Im Randgebiet der grauen Substanz, an der Grenze gegen die weisse findet man sehr reichlich vertreten diejenige Form, bei der die Ausläufer fächerförmig nur von der einen Seite ausströmen, wobei sie dann immer von der der grauen Substanz zugekehrten Seite entspringen. Man kann diese Zellen als für die Randpartieen geradezu typisch bezeichnen; man trifft sie im ganzen Umkreis des Vorderhorns und im Hinterhorn besonders an dessen medialer Grenzlinie, von der grauen Kommissur bis zur Rolando'schen Substanz, an. Namentlich beherbergen die kegelförmigen Zacken, mit denen die graue Substanz in die weissen Stränge hineinragt, solche Elemente, wobei die Spitze der Zacke den Zellkörper enthält, der Kegel selbst die fächerförmige Verästelung. Aber auch in den Gebieten zwischen den Zacken fehlen derartige Elemente nicht. Da diese Zellen, von denen Tafel I

und die Figur 21, S. 183 eine Vorstellung vermittelt, besonders reich verästelt sind, erscheint die Anordnung der Spinnenzellen und namentlich der „Gliafasern" gegen die Randzonen der grauen Hörner zunehmend dichter, was sich auch an Weigert'schen Gliapräparaten ausprägt, indem die Vorderhörner in ihrem ganzen Umkreise und die Hinterhörner an ihrer medialen Begrenzung gegen den Rand hin eine stärkere Schattierung zeigen. Man kann geradezu sagen, dass die graue Substanz gegen die weisse hier gleichsam durch einen aus dicht verfilzten Astrocyten bestehenden Wall umgeben ist.

Aber auch eine andere Spinnenzellenform kommt hauptsächlich der Grenzlinie der beiden Substanzen, namentlich derjenigen zwischen Hinterhorn und Hinterstrang zu: bipolare Astrocyten, bei denen die beiden Fortsatzbüschel tangential an der Grenzlinie hinlaufen. Alle diese quantitativen und qualitativen lokalen Differenzen der „Glia" erklären sich meiner Meinung nach aus dem wechselseitigen Verhalten der Nervenelemente und der Stützzellen, im Sinne eines kompensatorischen Verhältnisses.

Es giebt kein schwierigeres Problem in der Histologie des Rückenmarkes als die Frage nach der Zusammensetzung der sogenannten Substantia gelatinosa Rolandi. Es ist dies eine alte Streitfrage der Rückenmarksanatomie. Nervöses Ganglion oder Neurogliaanhäufung, das sind die Alternativen, um die sich die Anschauungen der Forscher in der früheren Periode gruppieren. Meynert[1]) und W. Krause[2]) haben sie als nervös aufgefasst, ja ihren sich in das obere Cervikalmark und darüber erstreckenden Teil geradezu als „unteren sensibeln Trigeminuskern" bezeichnet. Bechterew[3]) hat dagegen in ihr konsequent eine Anhäufung von centraler Stützsubstanz ohne Beimischung nervöser Elemente erblickt, eine Auffassung, der auch ich mich in einer früheren vorgolgischen Arbeit[4]) anschliessen zu sollen glaubte. Ohne eine bestimmte Äusserung über den allgemeinen Charakter der Rolando'schen Substanz, haben ihr schon früher mehrere Forscher, von denen ich Stilling, Clarke, Kölliker, Schwalbe, Gierke und namentlich H. Virchow[1]) hervorheben möchte, Nervenzellen zugeschrieben. Diese Angaben fanden ihre Bestätigung durch die Erfahrungen, die die Golgi'sche Methode geliefert hat. Golgi, R. y Cajal, v. Kölliker, Van Gehuchten, Lenhossék haben an der Hand des Chromsilberverfahrens nachgewiesen, dass sich in der Substanz in der That eine Anzahl charakteristischer Nervenzellen eingelagert finden; sie sollen an einer späteren Stelle ausführlich zur Sprache kommen. Dieser Nachweis involviert aber noch keine Entscheidung in der Frage nach der Natur der Substanz; das Bezeichnende für sie kann ja noch immer darin liegen, dass sie neben den Nervenzellen besonders viel Gliaelemente oder sonst was anderes enthält.

1) Th. Meynert, Vom Gehirne der Säugetiere. Stricker's Handbuch der Lehre von den Geweben, Wien 1870, Bd. II, p. 777.

2) W. Krause, Handbuch der menschlichen Anatomie, Bd. I, Allgemeine Anatomie, Hannover 1876, p. 389 und 420.

3) W. Bechterew, Über einen besonderen Bestandteil der Seitenstränge des Rückenmarkes. Archiv f. Anat. u. Physiol. Anat. Abt., 1886, p. 4.

4) M. v. Lenhossék, Untersuchungen über die Entwickelung der Markscheiden und den Faserverlauf im Rückenmarke der Maus. Archiv f. mikrosk. Anat., Bd. 33, 1889, p. 78.

Die wichtigste Angabe über die Natur der Rolando'schen Substanz aus neuerer Zeit findet sich in dem schon oben citierten kurzen aber inhaltsreichen Aufsatze von Weigert (a. a. O. p. 548). Weigert wies mit Hilfe seiner Gliafärbung nach, dass die Rolando'sche Substanz diejenige Partie des Rückenmarkes ist, in der die Gliafasern am spärlichsten sind. Sie lässt sich daher mit der sog. „Substantia gelatinosa centralis" auf keinen Fall in eine Reihe stellen. Denn diese besteht durch und durch aus einer verfilzten Gliafasermasse. Über die Natur der Rolando'schen Substanz äussert sich Weigert nicht; sie wird nach ihm ausser von einigen Nervenzellen und spärlichen Gliafasern hauptsächlich von einer „physiologisch unbestimmten reichlichen übrigen Substanz" gebildet.

Die Bilder, die ich mit der Golgi'schen Methode von der Rolando'schen Substanz an dem Rückenmark von Kindern aus dem ersten Lebensjahr erhalten habe, lassen mich dieser Darstellung vollkommen beistimmen. Auch an den Golgi-Bildern fällt ihre Armut an Astrocyten auf. Wäre sie, wie etwa die centrale Substanz, ein dichtes Konvolut von langstrahligen Spinnenzellen, so müssten sich der p. 184 dargelegten Erfahrung gemäss auch hier massenhaft diese reichverzweigten Elemente schwärzen. Dies ist nun nicht der Fall, und ich muss mich daher, auf Grund meiner neuesten Erfahrungen, vollkommen an Weigerts Ausspruch anschliessen, dass die Rolando'sche Formation keineswegs eine

1) H. Virchow, Über Zellen in der Substantia gelatinosa Rolando. Referiert im Neurol. Centralbl., 1887, p. 263.

Gliaanhäufung darstellt. Ich werde noch Gelegenheit haben, auf diese Formation zurückzukommen, und vielleicht wird es mir dann gelingen, die Frage nach ihrer Zusammensetzung, wenn auch nicht ganz bestimmt zu beantworten, so doch um einen Schritt der Lösung näher zu bringen.

Jedenfalls entbehrt aber die Rolando'sche Substanz der gewöhnlichen „Glia" nicht vollkommen, denn es färben sich immerhin gelegentlich Spinnenzellen in ihr, aber nicht mehr, ja eher etwas weniger als in andern Gebieten der grauen Substanz.

Diese Elemente gehören dann bald in die Kategorie der Langstrahler, bald in die der typischen Kurzstrahler. Die Langstrahler, denen man hier begegnet, unterscheiden sich von den anderwärts vorhandenen Zellen dieser Art dadurch, dass ihre Fortsätze etwas derber und nicht so üppig sind wie anderswo, auch lassen die Zellen ihre Ausläufer nicht gleichmässig nach allen Richtungen hin, sondern mehr nur sagittal ausstrahlen, namentlich ist konstant ein stärkeres vorderes sagittales Fortsatzbüschel, das aus ganz parallelen und ziemlich langen Fasern besteht, während die seitlichen und hinteren Fortsätze in der Regel viel unansehnlicher sind.

Zwischen dem hinteren Umfange der Rolando'schen Substanz und dem Rande des Rückenmarkes befindet sich ein schmales, faserarmes, helles Gebiet, das gleichsam eine Brücke zwischen dem Seitenstrang und dem Hinterstrang darstellt; dies ist die „Randzone" von Lissauer. Sie umfasst die Eintrittsstelle der hinteren Wurzeln und die zu beiden Seiten davon befindliche Partie; ihre Form und Breite ist je nach den einzelnen Rückenmarksgebieten verschieden. Diese ganze Randzone nun, die eigentlich schon zur weissen Substanz gehört, zeichnet sich durch eine überaus reichliche Ansammlung von Astrocyten aus; dicht gedrängt stehen hier diese Elemente, wobei sie noch durch ihre ausserordentlich reichliche und sehr feine Verästelung auffallen.

Von hier aus erstreckt sich noch die Glia, d. h. der Komplex der ektodermalen Spinnenzellen zapfenförmig oder in Form mehrerer Bündel, die sich auf Querschnitten wie Plaques ausnehmen (Hoche), eine Strecke weit in das bindegewebige Perineurium der hinteren Wurzeln hinein, wie das von Staderini[1]) für die Wurzeln der Hirnnerven und neuerdings von

[1]) Staderini, Contributo allo studio del tessuto interstiziale di alcuni nervi cranieasi dell' uomo. Monitore zoolog. italiano, Anno I, 1890, p. 232.

Weigert (a. a. O, p. 547), Hoche[1]), Edinger[2]) und J. Schaffer[3]) für die sensibeln Wurzeln der Rückenmarksnerven sehr ausführlich beschrieben wurde. Hoche wies die gleichen Verhältnisse auch in den vorderen Wurzeln nach.

Auch jener schmale, noch zur grauen Substanz gehörige halbmondförmige Saum, der die eigentliche Rolando'sche Substanz von der hinteren Seite umfasst und als „Marginalzone der Rolando'schen Substanz" bezeichnet wird, begreift zahlreiche, hauptsächlich tangential angeordnete Spinnenzellen in sich.

Die Astrocyten der weissen Substanz gehören alle in die Kategorie der typischen Langstrahler. Von mehreren Seiten wird hervorgehoben, dass sie kräftiger, grösser, mit derberen Fortsätzen versehen seien als die der grauen Substanz. Damit stimmen meine Erfahrungen nicht überein; ich finde fast das Gegenteil, die meisten Astrocyten der Stränge erscheinen an meinen Präparaten eher kleiner, zarter verästelt als die Langstrahler der grauen Substanz, vor allem als die des centralen Gebietes, ihre Äste kürzer, einfacher. Was mir zunächst am meisten auffällt, ist, dass die meisten sich doch im ganzen relativ wenig von den Astrocyten der grauen Substanz unterscheiden; es ist dies deshalb zu verwundern, weil sie hier in ihrer Gesamtheit doch etwas ganz anderes zu stande bringen, als dort. In der grauen Substanz handelt es sich um eine diffuse Durchflechtung von Stützzellen und Nervenelementen, die komplizierten unregelmässigen Verästelungen der Nervenzellen, ihre zackigen Zellkörper werden von den Astrocytenfortsätzen korbartig umsponnen; in der weissen Substanz tritt uns ein ganz anderes Verhalten entgegen, hier handelt es sich mehr um Bildung eines regelmässigen, die Längsfasern umkreisenden Maschenwerkes, worin durch reichlicheres Zusammentreten der Astrocyten und ihrer Fortsätze einzelne derbere Züge, sog. „Gliasepta" hervortreten. Man sollte nun erwarten, dass dieses so gründlich verschiedene Gesamtverhalten auch mit etwas anderen Formverhältnissen der einzelnen Gliaelemente einhergeht, namentlich dass die

[1]) A. Hoche, Beitrag zur Kenntnis des anatomischen Verhaltens der menschlichen Rückenmarkswurzeln etc. Habilitationsschrift, Heidelberg 1891.

[2]) L. Edinger, Vorlesungen über den Bau der nervösen Centralorgane, 4. Aufl., 1893, p. 16.

[3]) J. Schaffer, Die oberflächliche Gliahülle und das Stützgerüst des weissen Rückenmarksmantels. Anat. Anz., Bd. IX, 1894, p. 262. — Derselbe: Beiträge zur Kenntnis des Stützgerüstes im menschlichen Rückenmarke. Arch. f. mikr. Anat., Bd. 40, 1894, p. 54 ff.

Fortsätze vielleicht noch steifer sind oder andere Besonderheiten zeigen; dies ist nun, wie gesagt, nicht der Fall, zwischen den Astrocyten der weissen Substanz und den Langstrahlern der grauen bestehen keine sehr auffälligen Unterschiede.

Die Astrocyten, die inmitten der Stränge liegen, zeigen in der Regel eine mehr oder weniger gleichmässige Ausbreitung ihrer Äste nach allen Seiten hin. Je mehr wir aber unseren Blick gegen die Peripherie des Rückenmarkes hin wenden, desto ausgesprochener tritt uns an den Spinnenzellen eine charakteristische Eigenart entgegen. Sie besteht darin, dass nun die peripheriewärts gerichtete Verästelung mehr und mehr mächtiger wird, die innere an Dichtigkeit abnimmt. Die Zelle gravitiert nun nach der Oberfläche hin, ihr Schwerpunkt wird in die peripherische Verästelung verlegt. Den regelmässigsten Habitus zeigen die Zellen, die im Bereich des eingerollten, der vorderen Fissur zugekehrten „fissuralen" Abschnittes der Vorderstränge liegen; aber auch an anderen Stellen findet man stellenweise recht zierliche Exemplare. Von der kleinen Zelle geht ein dichter Pinsel von Ästen armleuchterartig, nach der Peripherie hin divergierend, aus, wobei die Äste einen mässig welligen, sich stellenweise überkreuzenden Verlauf zeigen und breit auseinanderfahrend die Peripherie erreichen, wo sie mit kleinen Knötchen endigen. Es ist an dieser Stelle darauf hinzuweisen, dass überhaupt alle Spinnenzellenfortsätze, die die Oberfläche des Markes erreichen, dort kleine rundliche oder fussförmige Anschwellungen bilden; der Komplex dieser Knötchen stellt die eigentliche Oberfläche des Organs dar. Von der inneren Seite der der Oberfläche nahe gelegenen Zellen entspringt ein zweites, ebenfalls divergierendes Fortsatzbüschel, das aber in der Regel nur aus spärlichen Fasern besteht, doch sind diese Fasern stark und oft von aussergewöhnlicher Länge; sie dringen nicht selten in der Bahn der sogen. Gliasepta von ihrem oberflächlichen Ursprunge her durch die ganze weisse Substanz hindurch tief in das Gebiet der grauen hinein.

Auch die Zellen, die in tieferen Schichten der weissen Stränge ihren Sitz haben, lassen von ihren peripherischen Fortsätzen einige bis zur Oberfläche hinaustreten, wobei diese breit, wie gespreizt auseinanderweichen. In der Verästelung aller Spinnenzellen der weissen Substanz herrscht überhaupt diejenige Richtung vor, die durch die Anordnung der gröberen „Gliasepta" vorgeschrieben ist.

Diese Gliasepta stellen nichts anderes dar, als die zu gröberen Bündeln zusammengefassten peripherischen Ausläufer der

in der weissen und teilweise auch in den peripherischen Lagen der grauen Substanz befindlichen, kettenartig zu Längsreihen angeordneten Astrocyten. Ob sich an der Bildung dieser Züge beim erwachsenen Menschen auch noch etwa die äusseren Fortsetzungen der Ependymzellen beteiligen, ist sehr unwahrscheinlich, wie das weiter unten näher besprochen werden soll. Jedenfalls aber bilden embryonal die Ependymfasern den massgebenden Faktor für die spätere Anordnung und Richtung der Gliasepta, denn bevor noch eigentliche Spinnenzellen in die Erscheinung getreten sind, zeigen die mit ihren Fortsätzen bis zur Oberfläche vordringenden Ependymzellen schon eine bestimmte typische Richtung, in der die Grundzüge der späteren Anordnung der Gliabalken unverkennbar sind. Wenn sich später die Golgi'schen Zellen anlegen, so halten sie sich ganz genau an diesen Grundplan, zuerst in den Verlauf ihres embryonalen „Hauptausläufers" (siehe weiter unten), und später, wenn dieser schwindet, in ihrer strahligen sekundären Ausbreitung im Bereich der weissen Substanz.

Diese so typische und regelmässige Anordnung der Gliabalken ist natürlich schon vielen früheren Forschern aufgefallen, und es liegen darüber schon aus der vergangenen Periode sehr ausführliche Darstellungen vor (so z. B. bei Frommann). Ein Irrtum, dem man früher ganz allgemein huldigte, war die Ansicht, dass es sich hier um Einstrahlungen der Pia mater handle; noch in den Schilderungen von Schwalbe[1]), Vignal[2]) (p. 391), Obersteiner[3]) u. a. spielen diese „Pialsepta" eine grosse Rolle. Und doch ist keine Thatsache sicherer, als die, dass wir es hier ausschliesslich mit Bündeln von ektodermalen Stützzellen zu thun haben. Auch das sogen. Septum posterius gehört in diese Kategorie mit dem Unterschied freilich, dass an dessen Bildung daneben noch den hinteren Ependymzellen des Centralkanales, die an dieser Stelle auch bei erwachsenen Geschöpfen ihre Fortsätze bis zur Oberfläche entsenden, ein wesentlicher Anteil zufällt. Von der Pia mater ist in diesem hinteren medianen Streifen jedenfalls nichts enthalten, von bindegewebigen Teilen überhaupt nur Blutgefässe und ihre Adventitia.

1) G. Schwalbe, Lehrbuch der Neurologie. Erlangen 1881, p. 303.

2) W. Vignal, Sur le développement des éléments de la moëlle des mammifères. Archives de Physiologie normale et pathologique, Tome 1884, p. 230.

3) H. Obersteiner, Anleitung beim Studium des Baues der nervösen Centralorgane. 2. Aufl., Wien 1892.

Auch die Zusammensetzung der die oberflächlichste Schichte des Rückenmarkes bildenden „Gliahülle" (Gierke a. a. O. p. 510) oder Gliarinde tritt an den Golgibildern mit einer Deutlichkeit zu Tage, die nichts zu wünschen übrig lässt (Fig. 22). Betrachtet man die mit der Weigert'schen Kupferhämatoxylinfärbung hergestellten Querschnitte des Rückenmarkes, aus welcher Gegend immer, so erkennt man, dass die dunkelblauen Faserbündel der Stränge nirgends direkt bis an die Oberfläche heranreichen, sondern überall noch von einem hellgelben, faserlosen Saum gegen die Oberfläche hin bedeckt werden. Diese oberflächliche Gliaschichte, für die ich den Namen „Peridym" vorschlagen möchte, hat schon in den vergangenen Jahrzehnten die Aufmerksamkeit mehrerer Forscher gefesselt. Schon Bidder erwähnt ihrer kurz (a. a. O. p. 35), Frommann[1]) hat ihr eine ausführliche Darstellung zu Teil werden lassen und auch ihren Aufbau wenigstens so weit erkannt, dass er sié aus verfilzten Fasern mit eingestreuten Zellen bestehen liess. Kölliker (Handbuch 5. Aufl. 1867, p. 268) ging einen Schritt weiter; wir finden bei ihm schon die richtige, aber noch etwas zu allgemein gehaltene Angabe, dass die Rindenschicht aus einem dichtesten, zartesten Gewirr von „Bindesubstanzzellen" bestehe. Golgi hat aber erst im Jahre 1871 ihre Zusammensetzung ganz zutreffend klargelegt, indem er nachwies, dass sie sich teils aus eigenen verästelten, tangentialen Gliazellen, teils aus den gegen die Oberfläche hinlaufenden Fortsätzen etwas tiefer gelegener Gliazellen zusammensetzt. Von neueren Forschern hat sich namentlich Schaffer (a. a. O.) sehr eingehend mit dieser Schichte befasst.

Nach Durchsicht meiner Weigert'schen Serien finde ich, dass das Peridym des Rückenmarkes nicht in allen Höhen gleich dick ist, sondern im Bereich der Intumescenzen, namentlich der Lendenanschwellung, eine besonders starke Entwickelung zeigt und namentlich auch von letzterer gegen den Conus terminalis hin eine allmähliche Zunahme an Breite erkennen lässt. Am beträchtlichsten ist es, wie schon Frommann beschrieben hat, an den Eintrittsstellen der vorderen und hinteren Wurzeln. Schaffer hat kürzlich mit Recht darauf hingewiesen, dass die Gliahülle bei verschiedenen Individuen oft einen ungleichen Entwickelungsgrad zeigt. Die innere Grenze des Peridyms erscheint stets zackig, infolge der vielen kegelförmigen Einmündungen der Gliasepta. An Nigrosinfärbungen ebenso wie auch bei der

[1]) C. Frommann, Untersuchungen über die normale und pathologische Anatomie des Rückenmarkes. Jena 1864, p. 28.

neuen Weigert'schen Gliafärbung stellt es sich als dunkler Saum dar. Die mit der letzteren hergestellten Präparate zeigen in dieser Schicht einen dichten Filz teils tangentialer, teils longitudinaler, teils auch radiärer Fasern.

Es hätte keinen Zweck, auf die mehr oder weniger mangelhaften Bilder, die noch andere Methoden über diese Substanzlage ergeben, einzugehen, es scheint zweckmässiger, gleich die schlechthin abschliessenden Bilder ins Auge zu fassen, die die Golgi'sche Methode bei Neugeborenen und Kindern davon gewährt (siehe Fig. 22). Das Peridym besteht nicht, wie das manche meinen, aus einem Geflecht isolierter Fasern, sondern aus lauter stark verästelten Zellen, aus Astrocyten. Man findet vor allem als Hauptbestandteil ganz tangential gelegene flächenhaft ausgebreitete Astrocyten, deren Fortsätze parallel mit der Oberfläche nach allen Seiten hin auseinanderstrahlen und die cirkuläre und longitudinale Faserung der Schichte bedingen. Selten liegt der Zellkörper ganz auf der Oberfläche, in der Regel hat er in den mittleren oder tieferen Schichten des Peridyms seine Lage, so dass er noch gegen die Oberfläche hin ganz minimale, gleich mit einem Knötchen endigende Ästchen abgeben kann. Auch von den tangen-

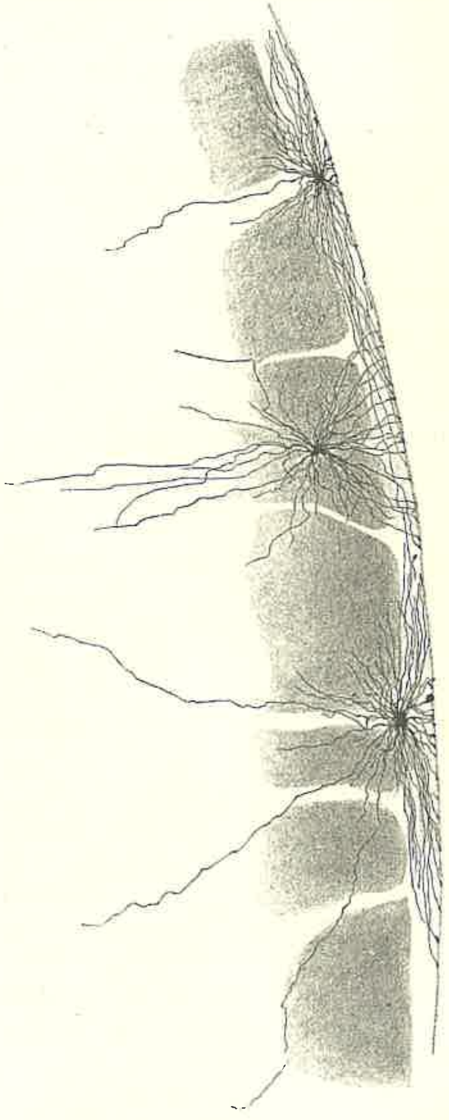

Fig. 22.
Oberflächliche Spinnenzellen („Peridym") aus dem Rückenmarke eines 3/4 jährigen Kindes.

tialen Fasern biegen die meisten, oft nach langem Verlauf, schief gegen die Oberfläche hin um und laufen da in eine Endverdickung aus. Gewöhnlich besitzen diese oberflächlichen Astrocyten neben ihren tangentialen Ästen auch noch ein inneres, rückläufiges Büschel, dessen spärliche Fasern in der Bahn der Gliasepta in die weisse Substanz eintauchen, um darin manchmal ziemlich weit einzudringen.

Zweitens kommen Astrocyten in Betracht, die nicht direkt in der Breite des Peridyms, sondern entweder unmittelbar darunter oder auch etwas tiefer, aber im ganzen doch noch in der Nähe der Oberfläche innerhalb der weissen Substanz liegen und die einen Teil ihrer äusseren Fortsätze im Peridym in die Tangentialrichtung umlenken lassen.

Einen sehr wesentlichen Anteil an der Bildung des Peridyms nehmen schliesslich noch die buschigen starren, bis zur Oberfläche vordringenden radiären Ausläufer von tiefer gelegenen Spinnenzellen; sie sind es, die die radiäre Faserung dieser Schicht bedingen.

Alle diese Fasergebilde, mögen es nun radiäre Fortsätze tiefer befindlicher Zellen oder tangentiale Ausläufer der oberflächlichen peridymalen Astrocyten sein, finden, wie schon erwähnt, mit kleinen Verdickungen ihr Ende. Diese Knötchen treten auf der freien Oberfläche des Markes mosaikartig zu einer offenbar lückenlosen, kompleten, äusserst feinen Grenzmembran zusammen, einer Art Cuticula (Membrana limitans meningea, His), die gegen die Pia mater hin das ektodermale Rückenmark vollkommen abschliesst. Die Knötchen sind an den einzelnen Fasern nicht immer von gleicher Dicke, was ich mir daraus erkläre, dass die Ausstrahlung der auf der Oberfläche ausmündenden Radiärfasern vielleicht stellenweise von verschiedener Dichtigkeit ist, so dass das Entstehen von Lücken in der Grenzmembran bloss durch stärkere Verdickung einzelner Faserenden vermieden werden kann. Gierke hat diese Grenzmembran fälschlich zur Pia gerechnet und sie als „Endothelmembran" beschrieben.

Aber diese aus den Endknötchen zusammengesetzte Grenzhaut, diese Cuticula medullae spinalis, ist äusserst zart. Sie erscheint an gut konservierten Präparaten als ein sehr feiner homogener Saum, unter dem gleich die tangentiale Faserung des Peridyms beginnt. Manchmal freilich tritt uns ein anderes Bild entgegen. Zwischen jenem Saume und der tangentialen Faserung der Gliarinde kann ein spaltförmiger Zwischenraum zur Ansicht kommen. Ich halte diese Erscheinung für ein Kunstprodukt und erkläre mir ihr Zustandekommen auf folgende Weise. Es scheint, dass jene Cuticula an die innerste Lage der Pia mater in äusserst fester Weise angelötet ist. Wodurch dieser Zusammenhalt bewerkstelligt wird, ist freilich nicht klar, denn ein organischer, durch Gewebsteile vermittelter Zusammenhang, etwa in Form von Piafortsätzen, die in das Rückenmark eintreten, besteht ganz sicher nicht. Es muss hier meiner Ansicht nach eher eine amorphe Kittlage im Spiele sein, jedenfalls ist aber der Zusammenhang ziemlich fest, denn bei den Schrumpfungsvorgängen, die sich durch die Einwirkung gewisser Härtungsflüssigkeiten an dem Rückenmarke und speziell an dem Peridym einstellen, zeigt sich manchmal die merkwürdige Erscheinung, dass die geschilderte Cuticula einen innigeren Zusammenhang hat mit der Pia als mit der Faserung des Peridyms. Denn die Spalte, die als Produkt der Schrumpfung zu stande kommt, liegt nicht zwischen Pia und Grenzhäutchen, sondern zwischen dem Grenzhäutchen und dem Peridym. Die Spalte ist nach dem Grade der Schrumpfung von verschiedener Breite und erscheint leitersprossenartig durchsetzt von den herausgezerrten Endstücken der radiären Fortsätze der an der Oberfläche und in deren Nähe gelegenen Spinnenzellen. Insbesondere scheint die von Kultschitzky eingeführte Härtungsmethode die Entstehung eines solchen künstlichen Zwischenraumes zu befördern; an den vielen sonstigen Rückenmarksschnitten, die sich in meinem Besitz befinden, vermisse ich ihn vollständig, während er an einem mir vorliegenden Präparate, das nach jenem Härtungsverfahren behandelt ist, stellenweise als weite Lücke erscheint. Ich kann also in diesem Raume unmöglich etwas Natürliches, Präformiertes erblicken und bin daher nicht in der Lage, der Ansicht von Gierke und Schaffer (a. a. O. Arch. f. mikr. Anat. p. 48), die ihm die Bedeutung eines Lymphraumes beilegen, zustimmen zu können.

Die Bildung derartiger Grenzhäutchen beschränkt sich aber nicht nur auf die Oberfläche des Rückenmarkes. Eine ganz ähnliche Terminalmembran kommt auch als Wandschichte den Kanälen und Kanälchen zu, in die die das Innere des Rückenmarkes durchdringenden Blutgefässstämmchen eingebettet sind. Betrachtet man das Verhältnis der Spinnenzellen zu den Blutgefässen an Golgi-Präparaten, so findet man, dass sie sich zu den Wandungen der Kanäle, in denen die stärkeren Gefässe liegen, ungefähr in derselben Weise verhalten, wie zur Oberfläche des Markes.

Stellt sich ein Blutgefäss den Spinnenzellen in den Weg, so weichen die Ausläufer der letzteren ihm nicht aus, sondern endigen knapp vor dem Gefäss mit einer ähnlichen Verbreiterung, wie sie die Gliafasern auf der Oberfläche des Rückenmarkes aufweisen. Da nun die Astrocytenausläufer von allen Seiten auf das Blutgefäss eindringen, so entsteht rings um dieses herum eine ausserordentlich zarte röhrenförmige Cuticularmembran, durch die sich die Rückenmarkssubstanz gegen das Blutgefäss gleichsam abschliesst, ein förmlicher Kanal, in dem das Gefäss, von seiner schwachen Adventitia umgeben, als selbständiger, mit der Rückenmarksstruktur nicht zusammenhängender Fremdkörper eingebettet ist.

An sehr vielen gewöhnlichen Färbepräparaten des Rückenmarkes und auch des Gehirns findet man Stellen, wo das Gefäss seinen Kanal nicht ganz auszufüllen scheint, indem zwischen ihm und der Wandschicht ein schmaler heller leerer Raum in die Erscheinung tritt. Es bleibt einstweilen ganz der subjektiven Meinung überlassen, ob diese Räume, die übrigens ohne Frage infolge der Schrumpfung der Blutgefässe stets in wesentlich gesteigerter Form erscheinen, wirklich perivaskulären Lymphräumen entsprechen, wie sie His[1]) und Obersteiner[2]) seiner Zeit gedeutet haben. Einstweilen kann dieser Annahme freilich nicht viel Wahrscheinlichkeit zugesprochen werden, da einerseits der Nachweis eines Zusammenhanges dieser Räume mit äusseren Lymphgefässen am Rückenmarke bisher noch nicht gelungen ist (auch His vermochte diesen Zusammenhang nicht nachzuweisen), andererseits aber jener epicerebrale resp. epimedulläre Raum, d. h. der eigentlich gar nicht existierende Spalt zwischen der Oberfläche der Centralorgane und der „Intima pia“, dessen innere Fortsetzungen diese Spalten darstellen würden, nach den herrschenden Anschauungen, die hauptsächlich auf den berühmten Untersuchungen von Key und Retzius[3]) basieren, nicht als Lymphraum gilt.

Aber unsere Kenntnisse von dem Bau und der Natur der „Glia“ würden im höchsten Grade fragmentarisch bleiben, wenn wir nicht

[1]) W. His, Über ein perivaskuläres Kanalsystem in den nervösen Centralorganen und über dessen Beziehungen zum Lymphsystem. Zeitschr. f. wiss. Zoologie, Bd. XV, 1865, p. 127.

[2]) H. Obersteiner, Über einige Lymphräume im Gehirne. Sitzungsberichte der kais. Akad. d. Wissensch. zu Wien, Bd. 61, Abt. I, 1870, p. 57.

[3]) A. Key und G. Retzius, Studien in der Anatomie des Nervensystems und des Bindegewebes. I. Hälfte, Stockholm 1875, p. 41.

auch ihre Entwickelung ins Auge fassten. Diese ist es erst, die ein richtiges Verständnis der Stützzellen ermöglicht. Die Betrachtung der fertigen Astrocyten giebt über die Herkunft, den Charakter dieser Elemente keinen sicheren Aufschluss. Betrachtet man die reichlich verästelten Gliazellen, wie sie sich etwa an Golgischen Präparaten darstellen, so scheint es auf den ersten Blick noch am nächsten zu liegen, sie gleichzustellen den sternförmigen Zellen des Bindegewebes. Ist schon durch den Habitus der einzelnen Elemente eine solche Betrachtungsweise nahegelegt, so muss die Anschauung, dass man es mit einer Gattung von Bindegewebe zu thun habe, noch gefördert werden durch die Art und Weise, wie sich die Astrocyten in ihrer Gesamtheit verhalten, wie sie sich zu einem Stützgerüst verbinden, namentlich im Bereich der weissen Substanz. Hierzu kommt noch, dass bei den meisten Färbungen die der Oberfläche des Rückenmarkes anliegende Piallamelle sich ebenso färbt, wie die Rindenschichte des Rückenmarkes, das Peridym, so dass die beiden in der Regel in enger Berührung stehenden, ja geradezu verklebten Teile gleichsam zu einem einheitlichen, durch keine Grenzlinie unterbrochenen Lager zusammenzufliessen scheinen, woraus die Vorstellung entspringen kann, als wären die in das Peridym einmündenden Gliasepta der weissen Substanz Fortsetzungen, „Einstrahlungen“ der Pia mater.

Kein Wunder also, dass Jahrzehnte hindurch sowohl die Spinnenzellen wie auch überhaupt alles, was im Rückenmarke und im weiteren Umfange im gesamten Centralnervensystem zwischen den Nervenzellen und Nervenfasern liegt, schlechthin als „Bindegewebe“ galt, die Zellen unbedenklich als „Bindegewebszellen“ figurierten. War diese Auffassung gleich von Anfang an, zu einer Zeit, da man noch in der Zwischensubstanz der Centralorgane eine weiche, körnige Masse erblickte, die vorherrschende, so musste sie in dem Masse noch tiefer Wurzel fassen, als in der Stützsubstanz ein fibrillärer Bau aufgedeckt wurde. Denn so lange die Glia als eine granuläre Masse galt, musste man sich doch sagen, dass diese Substanz, mag sie auch in die Bindegewebsgruppe gehören, von dem Bilde des gewöhnlichen Bindegewebes, wie es uns anderweitig entgegentritt, doch etwas abweiche (siehe z. B. Virchow's Cellularpathologie, 3. Auflage 1862, p. 257); sowie sich aber die Glia als ein Faserretikulum erwies, schien der Anschluss an das faserige Bindegewebe nach allen Richtungen hin gesichert.

Es ist noch nicht lange her, dass die bindegewebige Natur der Glia geradezu als selbstverständlich galt. Nicht nur dass Golgi z. B. in seiner oben erwähnten, so wichtigen Arbeit aus dem Jahre 1871 konsequent von Bindegewebszellen spricht, eine Anschauung, die er freilich schon im Jahre 1885 aufgegeben hat, nicht nur dass noch bei Gerlach (1870), bei Boll (1874) diese Auffassung vorherrscht, ja noch in der Zusammenstellung von Obersteiner vom Jahre 1892 (II. Auflage) erscheint das Stützgewebe als Bindesubstanz, und man kann sogar in Arbeiten aus der allerjüngsten Zeit Stellen finden, wo die Spinnenzellen den „Zellen des retikulären Bindegewebes“ gleichgestellt werden (s. z. B. Neurol. Centralblatt, 1893, p. 803 u. s. w.). Den Höhepunkt hat diese Lehre bei Gerlach erreicht, der die gesamte Glia aus elastischen Fasern bestehen liess.

Die neueren Forschungen haben hier nun gründlich Wandel geschafft. Die „Neuroglia“ ist ektodermal, dies ist das Hauptergebnis und das sicherste Ergebnis, zu dem diese Forschungen geführt haben. Die Stützzellen gehen aus derselben Anlage hervor, wie die Nervenzellen; damit soll nicht gesagt werden, dass sie auch in physiologischem Sinne als „nervös“ zu gelten haben. Nur die Anlage ist gemeinsam, in funktioneller Hinsicht tritt bald eine Differenzierung ein in Zellen, die durch Entsendung einer Nervenfaser zu den nervösen Vorgängen in Beziehung treten, in Neurocyten, und in solche, die sich zur Bildung eines Stützgerüstes für die Nervenelemente vereinigen und daneben noch, nach der Auffassung von P. Ramón, R. y Cajal und Cl. Sala, die Bedeutung von Isolatoren haben, in Stützzellen, Spongiocyten. Diese funktionelle Differenzierung lässt sich durchaus in eine Reihe stellen mit derjenigen, die in einem einfachen Sinnesepithel, z. B. der Riechschleimhaut vor sich geht, wenn sich dessen Elemente in Sinneszellen und indifferente Epithelzellen trennen. Freilich zeigen die Stützelemente der Sinnesepithelien überall recht einfache Formverhältnisse, während hier die der Stützfunktion dienenden Zellen in merkwürdig komplizierter Weise umgewandelt sind.

Wenn wir der historischen Entwickelung dieses so fundamentalen Nachweises nachgehen, so kommen wir auf Angaben zurück, die nicht auf der direkten Beobachtung der Entwickelungsweise der „Zwischensubstanz“ beruhen, sondern nur den Eindruck wiedergeben, den die „Glia“ in ihrem fertigen Zustande auf den Beschauer machte. So ist z. B. die alte Angabe

R. Wagner's[1]) aufzufassen, dass die „feinkörnige“ Zwischensubstanz „nervös“ sei, womit wohl gemeint ist, dass sie wie die nervösen Elemente, dem Ektoderm entstamme.

Es liegt aber auf der Hand, dass wenn über die Frage nach der Herkunft der Stützelemente eine bestimmte Antwort erzielt werden soll, dies bloss durch die direkte Beobachtung ihrer Histiogenese möglich ist. Daher das Bestreben der Histologen, die sich mit der „Glia“ abgaben, ihre Untersuchungen auch nach dieser Seite hin auszudehnen. Hier sind namentlich Boll (a. a. O.), Vignal[2]) und Gierke (a. a. O.) zu nennen. Die Resultate, zu denen die drei Forscher gelangten, stimmen in der Hauptsache überein. Alle drei fassen die wesentlichen Elemente der Glia, die Spinnenzellen, nicht als bindegewebige Eindringlinge auf, sondern als Elemente, die an Ort und Stelle aus ursprünglichen Bildungszellen der Anlage des Centralnervensystems entstehen. So sagt Boll (p. 14), dass „die bindegewebigen Elemente von vornherein an Ort und Stelle vorhanden sind und einen integrierenden Teil der Embryonalanlage bilden und nicht erst durch von der Pia mater aus eindringende Fortsätze zwischen die nervösen Teile eingeschoben werden.“ Es muss aber angesichts dieser Stellungnahme als eine Inkonsequenz erscheinen, wenn Boll die Astrocyten trotzdem immer als „Bindegewebszellen“, die Glia als „Bindegewebe des Centralnervensystems“ bezeichnet. Auch Vignal kam zu diesem Ergebnis. Indem er die anfänglich ganz kernlose weisse Substanz des Rückenmarkes bei menschlichen Embryonen sich später allmählich mit Kernen, d. h. mit Gliazellen bevölkern sah und dabei alle Hinweise darauf, dass diese Zellen von aussen her eindringen, vermisste, sprach er die Vermutung aus, dass sie aus dem Bereich der grauen Substanz in die weisse hinauswandern, eine Deutung, die natürlich auch die Annahme einer ektodermalen Herkunft in sich schliesst.

Am entschiedensten ist aber ohne Frage für die ektodermale Natur des Stützgewebes Gierke eingetreten. Freilich ist dabei zu bemerken, dass die positiven embryologischen Beobachtungen dieses Forschers über die Vignal's nicht wesentlich hinausgehen. Auch Gierke ist wohl zu seinem so energisch vertretenen Standpunkte mehr durch Betrachtung des fertigen Gewebes, als durch eine erfolgreiche Beobachtung des werdenden gekommen.

Eine sehr mächtige Stütze gewannen die von diesen Forschern vertretenen Anschauungen durch die embryologischen Untersuchungen von His[3]), die sich von den schon erwähnten Untersuchungen dadurch unterscheiden, dass sie von viel früheren Stadien ausgehen. Aus den His'schen Forschungen ging die Herkunft wenigstens eines Teiles der Neuroglia aus den Zellen der Medullarplatte mit Bestimmtheit hervor. Für die Deiters'schen Zellen allerdings hatte His die Möglichkeit eines bindegewebigen Ursprunges offen gelassen. Er unterschied an der Neuroglia als Hauptbestandteil ein faseriges Netzwerk, das Myelospongium, das ausschliesslich aus den verzweigten äusseren

1) R. Wagner, Neurologische Bemerkungen. Göttinger Nachrichten, 1854, p. 28.

2) W. Vignal, Sur le développement des éléments de la moëlle des mammifères. Archives de Physiol. normale et pathol. T. 1884, p. 230.

3) W. His, Histogenese u. Zusammenhang der Nervenelemente. Archiv f. Anat. u. Physiol. Anat. Abt. 1890, p. 103.

Fortsätzen der Ependymzellen hervorgehe, und darein eingelagert die Deiters'schen Pinselzellen, in denen er sekundär hinzugekommene, in das Markgerüst als fremde Elemente eingetretene Bindegewebszellen vermutete.

Hier ist noch nachzutragen, dass auch Götte[1]) schon früher auf Grund seiner embryologischen Untersuchungen alles, was im Rückenmark ist, auch die Stützzellen, aus dem Ektoderm ableitete.

In eine ganz neue Phase trat die Angelegenheit, als die Golgi'sche Methode zur Untersuchung dieser Frage herangezogen wurde. Es kann dies in der That als ein Wendepunkt in der Neurogliafrage betrachtet werden, denn haben auch die früheren Methoden nach dieser Seite hin manche wichtige Anhaltspunkte geliefert, so war ihre Leistungsfähigkeit doch immerhin sehr beschränkt, sowohl was die einwandfreie Sicherheit der Resultate betrifft, wie auch in dem Sinne, dass sie nur die Hauptpunkte, nicht aber alle Einzelheiten der hier in Frage kommenden Vorgänge zu ermitteln gestatten. Erst durch die Golgi'sche Methode erhielten wir über die Histiogenese der Stützzellen ganz positive klare Bilder, Bilder, die in allen Einzelheiten erschöpfende Anschauungen gewähren.

Die erste einschlägige Beobachtung rührt von Golgi her. Golgi führte im Jahre 1885[2]) an der Hand der Chromsilbermethode den bestimmten Nachweis, dass sich in der Embryonalperiode die basalen Enden sämtlicher Ependymzellen, d. h. der sog. Epithelzellen des Centralkanales als radiäre Fasern bis zur äussersten Oberfläche des Markes erstrecken[3]). Diese wichtige Angabe bildete den Ausgangspunkt der weiteren Entwickelung unserer einschlägigen Kenntnisse, ebenso wie jenes Stadium selbst den Ausgangspunkt darstellt für die Bildung der Neuroglia. Golgi selbst hat die weiteren Entwickelungsvorgänge nicht verfolgt.

Fritjof Nansen[4]) gelang es im Jahre 1886 zum erstenmal, die gesamte Neuroglia des Rückenmarkes bei einem Wirbeltiere mit der Golgischen Methode klar darzustellen. Seine Untersuchungen beziehen sich auf Myxine. Die Astrocyten zeigen bei diesem Tiere ein sehr charakteristisches Aussehen, ein Verhalten, das sie ohne weiteres als ektodermale, den Nervenzellen gleiche Elemente kennzeichnet. Auch sind bei diesem Tiere die von Golgi beim Hühnerembryo nachgewiesenen langen Ependymfasern zeitlebens, auch im ausgebildeten Zustande vorhanden. Die Verhältnisse liegen hier also

---

1) A. Götte, Entwickelungsgeschichte der Unke. Leipzig, 1875, p. 275.

2) C. Golgi, Sulla fina anatomia degli organi centrali del sistema nervoso. Milano, 1885/86, p. 178.

3) Genauer genommen hat vor Golgi schon Hensen das radiäre System auf Grund anderer Methoden beschrieben. Hensen giebt für das Kaninchen an, dass selbst zu einer Zeit, wo schon graue Substanz sich dem noch sehr breiten Epithel des Centralkanales auflagert, von den Zellen dieses Epithels Fortsätze in Form von Radiärfasern durch das ganze Mark ausgehen und sich mit ein wenig verbreiterter Basis an die „Membrana prima" inserieren (Zeitschrift für Anatomie und Entwickelungsgeschichte, Band I, 1876, p. 372.)

4) Fr. Nansen, Structur and Combination of the Histological Elements of the Central Nervous System. Bergen's Museums Aarsberetning for 1886. Bergen 1887, p. 160.

viel durchsichtiger, als bei höheren Wirbeltieren, und obgleich Nansen die Entwickelungsvorgänge der Stützelemente nicht direkt untersucht hat, so konnte er doch aus deren Betrachtung im entwickelten Marke durch einen Schluss a posteriori den wichtigen Satz ableiten, dass „auch die Neurogliazellen einen ektodermalen Ursprung haben und sich aus den Epithelzellen des Centralkanales entwickeln".

Ramón y Cajal[1]) hat erst den Vorgang der Entwickelung der Astrocyten bei dem Hühnchen an der Hand der Silberbilder durch fortlaufende Untersuchung verschiedener Stadien klargelegt; auch bezüglich der Säuger vermochte er in seiner Arbeit schon einige einschlägige Bemerkungen beizufügen. Das Ergebnis, wozu Cajal kam, stimmt mit Nansen's Auffassung, von dessen Arbeit übrigens Cajal keine Kenntnis hatte, überein. Cajal schildert zunächst das Verhalten der radiären Ependymzellen im Hühnchenrückenmarke sehr genau, wobei er Golgi's Angaben namentlich durch die Beschreibung des merkwürdigen Verhaltens dieser Zellen ventral und dorsal vom Centralkanal ergänzt. Vom 8. Tage an gewahrt man einige Ependymzellen, deren Zellkörper nicht mehr knapp am Centralkanal, sondern weiter auswärts liegen. Diese Elemente bilden die Vorläufer der Spinnenzellen. Letztere treten in ihrer charakteristischen Form am 9.—10. Tage in die Erscheinung und lassen sich alle auf dislozierte und stark umgewandelte Ependymzellen zurückführen.

An Cajal schlossen sich bald eine Reihe anderer Forscher, wie v. Kölliker[2]), Van Gehuchten[3]), Lenhossék[4]), Retzius[5]) und Cl. Sala[6]) an, durch deren Bemühungen nicht nur die von dem spanischen Histologen mitgeteilten Befunde in den wesentlichsten Punkten Bestätigung erfuhren, sondern sich auch zahlreiche weitere Einzelheiten ergaben. Ich selbst darf das Verdienst für mich in Anspruch nehmen, zuerst bei dem menschlichen Embryo die einschlägigen Verhältnisse ausführlich beleuchtet zu haben. Wir können hier von einer Inhaltsangabe all' dieser Arbeiten absehen, da die Thatsachen, die darin niedergelegt sind, im folgenden ausführlich zur Sprache kommen sollen.

---

1) S. R. y Cajal, Sur l'origine et les ramifications des fibres nerveuses de la moëlle embryonnaire. Anat. Anz. Jahrg. V, 1890, p. 115. — Derselbe: Nuevos observaciones sobre la estructura de la médula espinal de los mamíferos. Barcelona, 1890.

2) A. Kölliker, Zur feineren Anatomie des centralen Nervensystems. Zweiter Beitrag: Das Rückenmark. Zeitsch. für wissensch. Zoologie. Bd. LI, 1890, p. 31.

3) A. Van Gehuchten, La Structure des centres nerveux. La moëlle épinière et le cervelet. La Cellule, T. VII, 1891, p. 104.

4) M. von Lenhossék, Zur Kenntniss der Neuroglia des menschlichen Rückenmarkes. Verhandl. der Anat. Gesellsch., 5. Versamml. 1891. (Anat. Anz. p. 193.) — Vergl. ausserdem die Darstellung des Stützgewebes in der 1. Auflage dieses Buches, p. 45—62.

5) G. Retzius, Ependym und Neuroglia. Biolog. Untersuch., Neue Folge V, 1893, p. 9.

6) Cl. Sala y Pons, La Neuroglia de los Vertebrados. Barcelona 1894.

Den Ausgangspunkt der Entwickelung bildet das Stadium, wo das eben zur Abschnürung gelangte Medullarrohr aus einer einzigen Schichte (Hensen) säulenförmiger Zellen besteht. Zwar täuschen die in verschiedenen Höhen an den Zellen angebrachten Kerne an Färbepräparaten eine Mehrschichtigkeit vor, doch ist dies ein Trugbild, indem alle Zellen mit ihren beiden oft unscheinbaren Enden den Centralkanal und die äussere Grenzfläche des Medullarrohres erreichen.

Diese ersten Zellen des Centralnervensystems gehören in ihrer späteren Verwendung in die Kategorie der Stützzellen, und so erscheint das Stützsystem der Centralorgane ontogenetisch älter als die nervösen Elemente. Mit der Zunahme des Markes an Umfang verlängern sich diese Zellen beträchtlich, ja sie verdünnen sich allmählich zu zarten Fasern, die nun das Mark, vom Centralkanal ausgehend, als Ependymfasern in radiärer Anordnung durchsetzen. Der innere Teil, derjenige der dem Centralkanal näher liegt, bewahrt mehr den Charakter eines eigentlichen Zellkörpers; er umfasst einen anfangs noch in verschiedener Entfernung vom Centralkanal gelegenen, später sich mehr und mehr an ihn herandrängenden Kern. Dieser Teil ist die „Ependymzelle", der äussere die „Ependymfaser". Das ganze System dieser radiären Fasern stellt nun das Ependymgerüst oder das Ependymium des Markes dar.

Golgi war, wie schon oben erwähnt, der erste, der die radiäre Anordnung der ersten Stützelemente bei dem Hühnchen nachwies und zeigte, dass auf einer frühen Stufe die ganze „Glia" durch diese Ependymzellen allein gebildet wird. Golgi's Befunde wurden seitdem sowohl für das Hühnchen, wie für andere Wirbeltiere von einer Reihe von Forschern bestätigt, so von Fritjof Nansen (Amphioxus, Myxine), Burckhardt[1] (Amphibien), Falzacappa[2] (Hühnchen), R. y Cajal (Hühnchen, Säuger, Reptilien), Lachi[3] (Hühnchen), v. Kölliker[4]) (Säuger), Lenhossék (Mensch, Säuger, Hühnchen,

[1]) R. Burckhardt, Histologische Untersuchungen am Rückenmarke der Tritonen. Archiv f. mikrosk. Anat., Bd. XXXIV, 1889, p. 142.

[2]) E. Falzacappa, Ricerche istologiche sul midollo spinale. Rendiconto della R. Accademia dei Lincei, Vol. V. 1889, p. 696.

[3]) P. Lachi, Contributo alla istogenesi della nevroglia nel midollo del pollo. Memoria della Soc. Toscana di Scienca natur., Vol. 11, Pisa 1890.

[4]) A. Kölliker, Zur feineren Anatomie des centralen Nervensystems. Zweiter Beitrag: Das Rückenmark. Zeitschr. f. wissensch. Zool., Bd. 51, 1890, p. 32. — Derselbe: Handbuch der Gewebelehre, 6. Aufl., Bd. II, 1893, p. 136.

Selachier, Petromyzon), Van Gehuchten[1]) (Hühnchen, Säuger), Retzius[2]) (sämtliche Wirbeltierklassen), Lawdowsky[3]) (Frosch), Cl. Sala[4]) (sämtliche Wirbeltierklassen, besonders Batrachier). Bei allen Wirbeltieren erscheint in den Anfangsstadien der Entwickelung das Mark von einem regelmässig angeordneten, schönen Ependymgerüst durchzogen, überall bildet dieses den Vorläufer der späteren Stützvorrichtungen.

Es scheint uns nun geboten, die weitere Entwickelung der Stützelemente nicht in der Weise darzustellen, dass wir gleich auch das Verhalten der anderweitigen Stützzellen in verschiedenen Phasen betrachten, sondern so, dass wir zunächst das Schicksal dieser ursprünglichsten Elemente des Markes, der Ependymzellen, im Zusammenhange weiter verfolgen und schliesslich auch noch ihr definitives Verhalten im entwickelten Rückenmarke ins Auge fassen, ein Punkt, den wir in der vorhergehenden Schilderung der Stützzellen absichtlich unberücksichtigt liessen. Wir wollen dabei die Beziehungen der Ependymzellen zu den sich bildenden Astrocyten ausser Acht lassen, und immer nur einfach das gegebene Bild analysieren, das das Ependymium in bestimmten Phasen darbietet, ohne Rücksicht darauf, ob es dieselben Elemente sind, die aus einer Phase in die andere hinübergenommen werden, oder ob sich eine beständige Neubildung abspielt.

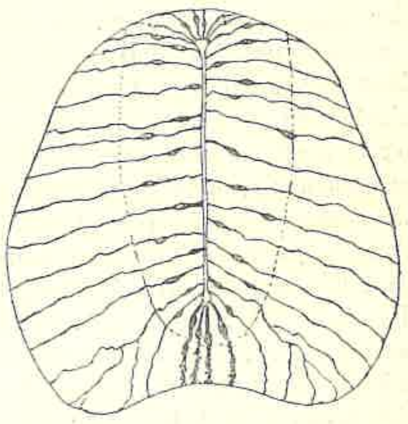

Fig. 23.
Querschnitt des Medullarrohres eines 4tägigen Hühnerembryos mit imprägnierten Radiärzellen.

Die geschilderte frühe Phase giebt Fig. 23 wieder, die die

[1]) A. Van Gehuchten, La Structure des centres nerveux. La moëlle épinière et le cervelet. La Cellule, T. VII, 1891, p. 104. — Derselbe: Le système nerveux de l'homme. Lierre 1893, p. 224.

[2]) G. Retzius, Zur Kenntnis der Ependymzellen der Centralorgane. Verhandl. des Biol. Vereins in Stockholm, 1891. — Derselbe: Ependym und Neuroglia. In Biol. Unters. N. F. V, 1893, p. 9.

[3]) M. Lawdowsky, Vom Aufbau des Rückenmarkes. Archiv f. mikrosk. Anatomie, Bd. 38, 1891, p. 264.

[4]) Cl. Sala y Pons, Estructura de la médula espinal de los batracios. Barcelona 1892. — Derselbe: La Neuroglia de los Vertebrados. Barcelona 1894.

Ependymzellen aus dem Medullarrohr eines 4tägigen Hühnerembryos darstellt. Von dem noch spaltförmigen Centralkanal her durchziehen die Ependymfasern seitlich in fast paralleler, oben und unten in radiär divergierender Anordnung das Mark. Das Zusammendrängen ihrer kernhaltigen Teile in den inneren Substanzlagen des Medullarrohres veranlasst hier die Bildung einer breiten kernreichen Schichte, der „Innenschichte" von His, der „Ependymkernzone", wie ich sie nennen möchte. Sie entspricht im allgemeinen dem späteren Epithel des Centralkanales, das sich aus dem Zustande dieser breiten Zone durch allmähliches Heranrücken der Kerne bis hart an den Centralkanal ableiten lässt. Alle Ependymfasern endigen am Rande des Markes mit kleinen dreieckigen Verbreiterungen. Die Ependymfasern der „Bodenplatte" (His), d. h. der späteren vorderen Kommissur zeigen ein merkwürdig rauhes Aussehen, sie sind plump und mit kleinen Stacheln versehen; auch lässt sich an ihnen schon auf dieser frühen Stufe eine allerdings einstweilen erst schwach angedeutete tonnenförmige Anordnung nachweisen, die sich auch an den seitlich davon gelegenen Ependymfasern ausprägt. Diese Anordnung, die, wie wir sogleich sehen werden, erst später recht eigentlich zur Geltung kommt, ist eine Folge der schon in dieser Phase beginnenden Einrollung der Vorderstränge, derjenigen Erscheinung, die allmählich zur Bildung der vorderen Fissur führt. Bemerkenswert ist noch, dass die Ependymzellen, die in der ventralen Abteilung des Markes rechts und links neben der Bodenplatte liegen, sich schon jetzt dadurch vor den anderen, stets ungeteilten Fasern auszeichnen, dass ihr Fortsatz sich während seines peripherischen Verlaufes in 3—4 oder mehr Äste auflöst. In der Regel ist es auf diesem Stadium jederseits nur eine einzige Zelle, die diese Eigenart erkennen lässt. Diese schon so früh verästelte Zellengattung lässt sich durch die nächsten Phasen hindurch verfolgen und setzt uns dadurch in den Stand, zu bestimmen, welche Umlagerungen in dem sich entwickelnden Marke vor sich gehen; es stellt sich in dieser Beziehung heraus, dass die Gegend, in der sie sich verästeln, das Gebiet also, das ursprünglich die ganze Breite der ventralen Fläche des Markes bildet, später ganz in die Bildung der ventralen Fissur aufgenommen wird; die ganze spätere Stirnseite des Markes rückt also von der Seite her heran.

Etwas spätere Stadien vergegenwärtigen die Figuren 24 und 28. Zwei Punkte sind es, worin gegenüber dem früheren Verhalten

an den Ependymzellen eine Änderung eingetreten ist. An dem nach aussen ziehenden Fortsatz bemerkt man, namentlich im Bereich der grauen Substanz, kleine Unregelmässigkeiten, Varikositäten, rauhe Fädchen; auffallender aber ist der Umstand, dass fast alle Ependymfasern in ihren äusseren Teilen, von der Stelle an, wo sie die weisse Substanz betreten, namentlich beim 10tägigen

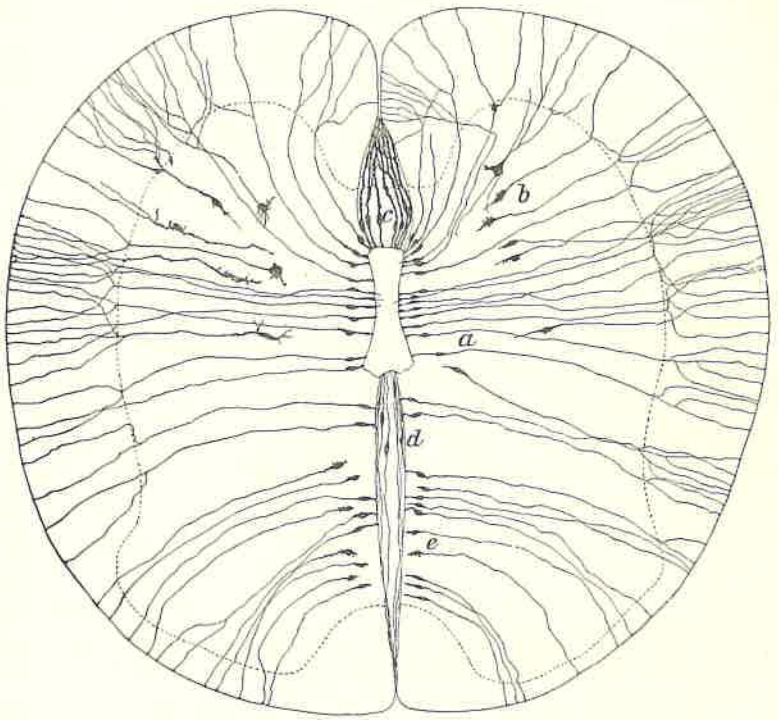

Fig. 24.

Rückenmark eines 3 cm langen menschlichen Embryos, mit imprägnierten Ependymzellen und Astroblasten, nach Retzius. a Ependymzellen, b Astroblast, c vorderer Ependymkeil, d Septum posterius, e Astroblasten, die zum Septum posterius orientiert sind.

Hühnchen, vielfach einer Teilung in mehrere Äste unterliegen, ja manchmal förmliche Büschel bilden, deren Zweige breit auseinanderstrahlend alle zur Peripherie ziehen, um dort mit den charakteristischen Endknötchen auszulaufen.

Aber die Rauhigkeit sowohl, wie die Teilung des Endstückes der Ependymfasern ist keine allgemeine Erscheinung, sie kommt

z. B. dem menschlichen Rückenmarke wenigstens in dem Masse, wie dem des Hühnchens, nicht zu. Dies erhellt namentlich aus der Figur auf der Tafel II, die das Stützsystem des Rückenmarkes von einem 14 cm langen menschlichen Embryo darstellt. Uns interessiert an dieser Zeichnung speziell nur die linke Hälfte, in der die Ependymzellen isoliert zur Ansicht gebracht sind. Wir haben es hier mit einem etwas späteren Stadium zu thun; jenes Anfangsstadium, da das ganze Stützsystem nur von Ependymzellen gebildet wird, ist beim menschlichen Embryo mit der Golgi'schen Methode bisher noch nicht untersucht worden. Denn schon beim 3 cm langen Embryo, dessen Rückenmark, nach einer Zeichnung von Retzius, in der Fig. 24 wiedergegeben ist, sind neben den Ependymzellen auch die Vorläufer der späteren Gliazellen vorhanden.

Ich möchte nun den Leser bitten, das Verhalten der Ependymzellen auf Tafel II, sowie an den beiden Figuren 24 und 29 etwas genauer ins Auge zu fassen. Die schlanken, spindelförmigen Zellkörper der „Ependymzellen" schliessen sich am Centralkanal zur Bildung des bekannten zierlichen Epithelkranzes aneinander und gehen am basalen Pol in je einen zarten, glatten, nervenfaserartigen Fortsatz über, der in einer bestimmten Anordnung, unter sanften Schlängelungen, radiär gegen die Oberfläche hinstrebt, um an ihr mit einer kleinen, kegel- oder keulenförmigen Verdickung zu endigen. Im äussersten, der weissen Substanz angehörenden Abschnitt gabeln sich diese Fasern in der Regel spitzwinklig in 2—3 Äste; eine reichere Verästelung kommt nur denjenigen Ependymfasern zu, deren Zellkörper gerade an der Grenze zwischen vorderer und seitlicher Abteilung des Centralkanals ihren Sitz haben; die Fortsätze dieser besonders verästelten Ependymzellensorte breiten sich am medialen, der vorderen Fissur zugekehrten Teil der Vorderstränge aus. — Die Zahl der Ependymfasern ist eine beschränkte, namentlich ziehen sie seitlich in weiteren Abständen von einander.

In der Gegend der vorderen Fissur erscheinen die Ependymfasern auch auf diesem Stadium derber als die seitlichen und lassen die schon bei dem einige Tage alten Hühnchen angedeutete meridianartige Anordnung noch viel ausgesprochener, in viel zierlicherer Form erkennen. Ramón y Cajal hat diese Anordnung beim Hühnchen und bei Säugern zuerst beschrieben, Retzius[1]) sie durch den Namen „vorderes Keilstück" gekennzeichnet;

[1]) G. Retzius, Zur Kenntnis der Ependymzellen der Centralorgane. Verhandl. d. Biol. Vereins in Stockholm, 1891.

v. Kölliker nennt diese Bildung „ventrales Ependymseptum". Man vermag die Entstehung dieser Figur an Entwickelungsstadien (beim Hühnchen) von Schritt zu Schritt zu verfolgen; sie hängt, wie schon gesagt, mit der Bildung der vorderen Fissur zusammen.

Hinten in der Mittellinie bilden die Ependymfasern auch eine ähnliche, aber viel unansehnlichere Tonnenfigur, das „hintere Keilstück" von Retzius und treten dann zu einem sagittalen Bündel, dem hinteren Ependymstrang oder Septum posterius zusammen. Dieses Septum läuft, aus ganz parallelen Fasern bestehend, in gerader Richtung nach hinten, um die Oberfläche im Bereich des schwachen Sulcus posterior zu erreichen, wobei die Fasern in ihrem allerletzten Stück eine schwache Divergenz zeigen und an ihrem Endpunkte alle die gewöhnlichen Verbreiterungen erkennen lassen. Es ist also gleich hier darauf hinzuweisen, dass auf diesem Stadium eine „hintere Fissur" nicht existiert.

An der seitlichen Ausbreitung der Ependymfasern fällt eine beträchtliche Lücke auf: das ganze Gebiet der Hinterhörner und auch der Hinterstränge ermangelt — abgesehen vom Septum posterius — der Ependymfasern. Auch diese Erscheinung findet in der Entwickelungsgeschichte ihre Erklärung. Ursprünglich stellt der Centralkanal, wie auf Fig. 23, eine lange sagittale Spalte dar, die ventral und dorsal bloss durch eine schmale Boden- und Deckplatte abgeschlossen wird. Dieser spaltförmige Hohlraum schwindet nun nach und nach bis auf den ventralsten Abschnitt; der ganze dorsale Teil gelangt zur Obliteration. Was geschieht nun aber mit den vielen Ependymzellen, die diesen ansehnlichen dorsalen Abschnitt begrenzten; werden sie allmählich nach vorn gezogen und in den Epithelkranz des später so viel kleineren rundlichen Centralkanals aufgenommen? Dies ist nicht der Fall. Wie ich dies zuerst mit Hilfe der Golgi'schen Methode genau nachgewiesen zu haben glaube[1]), bleiben sie alle in ungefähr derselben Gegend, in der sie sich ursprünglich befanden, sitzen, nur rücken ihre Zellkörper unter Verlust ihres Härchens etwas nach aussen, wodurch sie aus Ependymzellen zu Deiters'schen Zellen, angehenden Spinnenzellen degradiert werden. Da nun das aber gerade diejenigen Ependymzellen sind, die ursprünglich Hinterhorn und Hinterstrang durchsetzten, so bleiben diese Abschnitte bei der

[1]) M. v. Lenhossék, Zur Kenntnis der Neuroglia des menschlichen Rückenmarkes. Verhandl. d. Anat. Gesellsch., 5. Versamml., 1891, Anatom. Anz. p. 93.

späteren Verteilung der Ependymfasern auf dem Umriss des Markes unberücksichtigt. Aus dieser Darstellung geht also von selbst hervor, dass der ursprünglich in dorso-ventraler Richtung sehr ausgedehnte Centralkanal nicht durch gleichmässige Reduktion seine spätere Form und Grösse gewinnt, sondern dass es sich um eine richtige Verlötung der Wandschichten seines dorsalen Abschnittes handelt. Ähnliche Anschauungen wurden schon früher auf Grund embryologischer Untersuchungen von Waldeyer[1]), Balfour[2]), His[3]), Barnes[4]), Corning[5]) ausgesprochen. Zu demselben Ergebnis gelangen einige neuere einschlägige Arbeiten, wie die von Wilson[6]) und Prenant[7]).

Noch wäre bezüglich der am Centralkanale stehenden Zellkörper (s. Fig. 25) zu erwähnen, dass jede Zelle zu innerst einen verdickten Cuticularsaum (Membrana limitans interna) und von dessen Mitte hervorragend ein Härchen trägt. Dieses „Härchen“ ist schon sehr frühzeitig vorhanden. Retzius hat es schon bei dem 3 cm langen menschlichen Embryo nachweisen können; an den Golgi-Bildern präsentiert es sich als ein stets in der Einzahl vorhandenes, an seinem Ende manchmal umgekrümmtes, oft sehr langes Stäbchen, das mehr den Eindruck einer starren Cuticularbildung, als den eines „Flimmerhaares“ macht. In der I. Auflage dieses Werkes habe ich mich denn auch auf Grund dieses Verhaltens gegen die Deutung dieser Bildungen als „Flimmerhaare“ ausgesprochen. Indessen hat seitdem v. Kölliker (Handbuch d. Gewebelehre p. 143) soviel Beweise für die Flimmerung dieser Elemente beigebracht, dass ich an meinem Standpunkt nicht mehr

1) W. Waldeyer, Über die Entwickelung des Centralkanals im Rückenmark. Archiv f. path. Anat., 1876, Bd. LXVIII, p. 20.

2) F. M. Balfour, Handbuch der vergleichenden Embryologie. Übersetzt von C. Vetter. Jena 1881, II. Bd.

3) W. His, Zur Geschichte des menschlichen Rückenmarkes und der Nervenwurzeln. Abh. d. math.-phys. Klasse d. Kgl. Sächs. Ges. d. Wiss., Bd. XIII, 1886, p. 479.

4) Barnes, On the Development of the posterior fissure of the Spinal cord and the Reduction of the Central Canal in the Pig. Proc. Amer. Acad. arts and sc. 1884.

5) H. K. Corning, Über die Entwickelung der Substantia gelatinosa Rolandi beim Kaninchen. Archiv f. mikrosk. Anatomie, Bd. 31, 1888, p. 594.

6) J. T. Wilson, On the Closure of the central canal of the spinal cord in the foetal lamb. Transact. intern. med. Congress Sydney 1892.

7) A. Prenant, Critériums histologiques pour la détermination de la partie persistante du canal épendymaire primitif. Internat. Monatsschrift f. Anat. u. Physiol., Bd. XI, 1894, p. 1.

festhalten zu können glaube, um so weniger, als ich mir in der That sagen muss, dass es sich in dem starken „Stäbchen“, das die Golgi-Präparate darbieten, vielleicht doch nur um den Komplex feinerer, durch die Chromsilbermethode zu einer scheinbar einheitlichen Bildung verlöteter Härchen handelt.

Aus der Tafel II wird man erkennen, dass den Ependymzellen schon in dieser Phase im ganzen ein bescheidener Anteil am Stützgerüst zukommt. Doch gewinnen sie

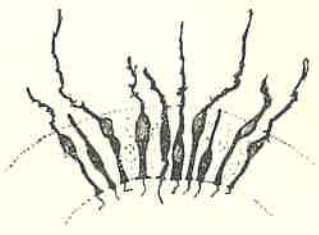

Fig. 25.

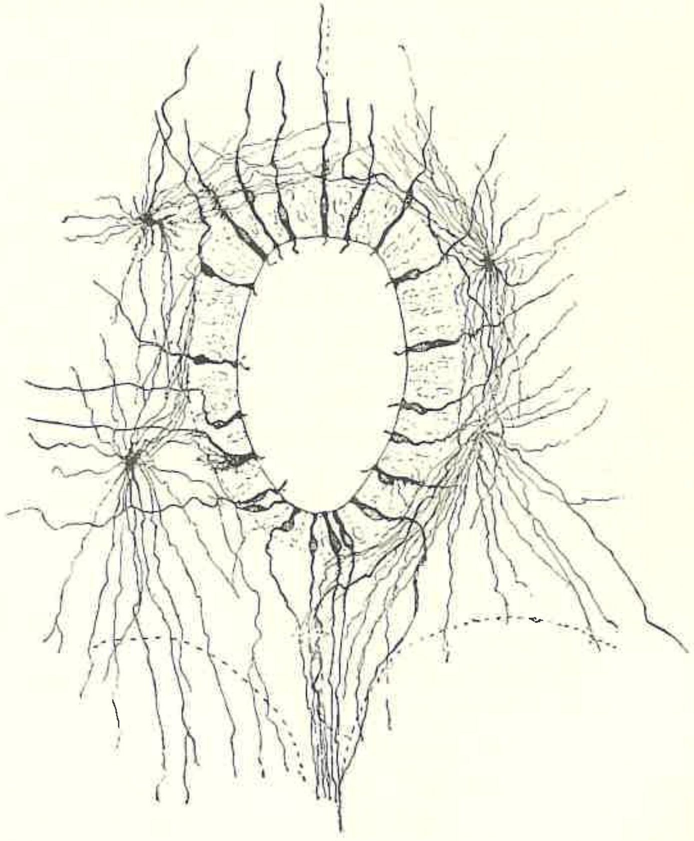

Fig. 26.

Fig. 25 u. 26. Aus dem Rückenmark eines 23 cm langen menschlichen Embryos. Ependymzellen und Substantia gelatinosa centralis.

an Bedeutung und Interesse einerseits durch den Umstand, dass die Richtung ihrer Ausläufer bestimmend ist für die Anordnung der übrigen späteren Stützzellen — sie bilden noch immer gleichsam das Skelett des gesamten Gliagerüstes — dann aber noch mehr dadurch, dass sie onto- wie phylogenetisch den ältesten Bestandteil des Stützgerüstes, die direkten Abkömmlinge der die Medullarplatten bildenden Ektodermzellen oder vielmehr diese selbst in modifizierter Form darstellen. Ihr Komplex bildet das „Urgerüst" des Markes, zu dem sich die übrige Neuroglia als neuerer Erwerb hinzugesellt.

Es fragt sich nun, was mit dem Ependymgerüst weiterhin geschieht, vor allem wie es sich im Rückenmarke des entwickelten Menschen verhält? Besteht es in der Form, wie es uns in Tafel II entgegentritt, nur in der Fötalperiode, oder wird es auch in die definitive Einrichtung unverändert übernommen? Es erhebt sich namentlich die Frage, ob auch späterhin alle Ependymfasern bis zur freien Oberfläche ausstrahlen. Bei Amphibien und Fischen, einschliesslich der Cyclostomen und des Amphioxus, ist dies bestimmt auch im ausgebildeten Zustande der Fall. Für die höheren Vertebraten hingegen ist es nur bezüglich des „vorderen Keilstückes" und des Septum posterius allgemein zugegeben, für die seitlichen Ependymfasern hat R. y Cajal bei mehreren Anlässen die Ansicht ausgesprochen, der sich in der Folge auch Retzius, Cl. Sala und v. Kölliker angeschlossen haben, dass sie im Laufe der Entwickelung einer Atrophie unterliegen, so dass sie dann schon in der Nähe des Centralkanales ein freies, verzweigtes Ende finden.

In der I. Auflage glaubte ich mich gegen diese Anschauung aussprechen zu sollen, indem ich die Vermutung äusserte, dass die Annahme einer solchen Atrophie auf dem Misslingen der Färbung der Ependymzellen auf späteren Stadien beruhen könne. Ich hielt es für wahrscheinlich, dass sich die Ependymfasern auch im ausgebildeten Zustande als äusserst dünne, spärliche Fasern bis an den Rand des Rückenmarkes hinschlängeln.

Auf Grund meiner seitdem fortgeführten Untersuchungen bin ich nun von dieser Anschauung vollkommen zurückgekommen und gebe nun auch das Eintreten jener Reduktionsvorgänge, wie sie jene Forscher schildern, zu.

Ich lege der nachfolgenden Darstellung den Zustand des Ependymiums beim $^3/_4$jährigen Kinde zu Grunde (Taf. I), wobei ich von

der Auffassung ausgehe, dass das in dieser Phase bestehende Verhalten bereits in der Hauptsache als ein definitives zu betrachten sei.

Die Reihe der den Centralkanal begrenzenden Epithelzellen zeigt gegenüber dem früheren Verhalten keinen wesentlichen Unterschied; der Kern liegt innerhalb des Zellkörpers bald ganz am Centralkanale, bald etwas entfernter davon, wodurch geringe Verschiedenheiten in der Form der Zellen hervorgerufen werden. Im Kerne fallen bei geeigneter Färbung mehrere auffallend stark tingierte Kernkörperchen auf; die schon oben erwähnte Cuticularplatte an der Innenfläche der Zellen ist noch vorhanden und färbt sich mit der neuen Weigert'schen Gliafärbung intensiv blau, und zwar merkwürdigerweise nicht als zusammenhängender Streifen, sondern, wie das Weigert nachwies, in Form mehrerer nebeneinander liegender Punkte. Auch Prenant[1]) hat neuerdings diese Punkte bei Schafembryonen wahrgenommen, doch sah er an jeder Zelle nur zwei davon, je einen an den beiden seitlichen Endpunkten der Cuticularplatte.

Die vorderen Ependymzellen laufen in derbe faserige Fortsätze aus; diese erreichen auch im reifen Zustande den Boden der vorderen Fissur und die benachbarten untersten Teile ihrer Seitenwandung. Die meridianartige Anordnung ist freilich jetzt schon so ziemlich verwischt, die Fasern bilden keine regelmässigen Bogen mehr wie früher, sondern lassen oft unregelmässige Schlängelungen erkennen, sie bilden ein förmliches Gewirr miteinander, wodurch die ganze Figur ihre frühere so typische Form einbüsst. Dabei erscheinen die Fasern ziemlich derb, aber glatt und ungeteilt.

Auch die hinteren Ependymzellen bewahren insofern ihr primitives Verhalten, als sie auch jetzt noch unter Bildung des Septum posterius bis zur oberflächlichen hinteren Medianfurche heranziehen; dies kommt an meinen Präparaten ganz deutlich zur Ansicht. Bevor sie sich aber zu diesem Septum vereinigen, zeigen sie im Bereich der sogen. hinteren grauen Kommissur eine breit aufgelockerte Anordnung, die an den früheren „hinteren Ependymkeil" erinnert, daraus hervorgegangen ist, sich aber davon durch den unregelmässig durcheinander geschlungenen Verlauf der Ependymfasern unterscheidet. Namentlich bilden die seitlichen Fasern oft

1) A. Prenant, Critériums histologiques pour la détermination de la partie persistante du canal épendymaire primitif. Internat. Monatsschr. f. Anat. u. Physiol., Bd. XI, 1894. p. 5.

sehr weit ausgezogene, unregelmässige Schlingen. Im Septum selbst aber weisen die Ependymfasern einen parallelen Verlauf auf.

Das Septum posterius ist also wesentlich eine Bildung des Ependyms, wozu noch zahlreiche eingelagerte Spinnenzellen kommen. Der Streifen galt bis vor kurzem als eine Einsenkung der Pia mater und man hat daher beharrlich von einer „hinteren Fissur“ gesprochen, die allerdings von jenem Fortsatz vollkommen ausgefüllt werde. Diese Darstellung erweist sich nun als unhaltbar; das Rückenmark ist hinten an den meisten Stellen unzweifelhaft ungespalten; es weist wohl einen Sulcus, aber keine Fissura posterior auf, indem das Septum posterius, das, nebenbei gesagt, oft gar nicht stärker erscheint als die übrigen Gliasepta der weissen Substanz, eine eigene Bildung des Rückenmarkes darstellt und von der Pia mater nichts enthält[1]). Dass eine eigentliche Diskontinuität der Rückenmarksubstanz durch das Septum posterius nicht veranlasst wird, ergiebt sich auf das evidenteste daraus, dass man innerhalb des Septum posterius selbst richtige Spinnenzellen antrifft, die ihre Verästelung in beide Rückenmarkshälften hineinragen lassen, und dass die benachbarten, im Hinterstrang befindlichen Astrocyten ihre Fortsätze vielfach ungeniert quer über das Septum hinwegschicken. Indessen soll nicht geleugnet werden, dass man doch, namentlich am Rückenmarke Erwachsener, an manchen Stellen die Andeutung einer von dem hinteren Sulcus ausgehenden, aber nie tief eingreifenden Spaltbildung wahrnimmt. Man findet sie namentlich, wie ich in Übereinstimmung mit einer älteren Angabe von Arnold[2]) und mit der soeben erschienenen Darstellung Schaffer's (a. a. O. Arch. f. mikr. Anat. 1894, p. 43) an meinen Präparaten sehe, im Lumbalteil ausgeprägt, sie schneidet aber nie tiefer ein als bis zu einem Drittel des Septum posterius. Diese Spaltbildung ist eine sekundäre Erscheinung, sie ist, wie ich glaube, überall an den Eintritt von Blutgefässen in der hinteren Mittellinie geknüpft, und wenn man auch auf dem Querschnitte kein Blutgefäss findet, so erklärt sich das wohl daraus, dass sich die Spalte in der Längsrichtung noch etwas über die Eintrittsstelle des Gefässes ausdehnt. Jedenfalls ist sie als eine

[1]) Zu ähnlichem Ergebnis gelangte unlängst A. Robinson in einer fleissigen embryologischen Arbeit (On the development of the posterior colums etc. Studies in Anatomy from the Anat. Departm. of the Owens College. Vol. I., Manchester 1891, p. 98).

[2]) Fr. Arnold, Bemerkungen über den Bau des Hirns und Rückenmarks. Zürich 1838, p. 3.

sekundäre Auseinanderspaltung innerer Elemente des Rückenmarkes aufzufassen und lässt sich mit der vorderen Fissur nicht vergleichen, denn diese kommt durch Wachstumsdifferenzen der ventralen Teile, als eine grubenförmige Vertiefung der Oberfläche zwischen gewucherten Partien zu stande.

Was die seitlichen Ependymzellen betrifft, so ist zunächst hervorzuheben, dass ihr Gebiet innerhalb des Epithelkranzes im ausgebildeten Rückenmarke sehr beschränkt ist, indem die Ependymzellen, deren Fortsätze sich zur vorderen und hinteren Kommissur hinneigen, sich also an der Bildung der beiden Ependymkeile beteiligen, von vorn und hinten ziemlich weit auf die Seitenwand des Centralkanals übergreifen; nur ein kleines Stück des Epithels bleibt für die seitlichen Ependymzellen übrig. Offenbar ist hier über das in Tafel II dargestellte Stadium hinaus noch ein reichlicher Austritt von seitlichen Ependymzellen, eine Umwandlung solcher zu Spinnenzellen vor sich gegangen. Die Zellen, die noch im Epithel sitzen geblieben sind, setzen sich zwar auch im ausgebildeten Marke, an ihrem basalen Pole in eine Faser fort, die das Gebiet der sogen. Substantia gelat. centralis radiär durchsetzt; diese Fasern sind aber hier von Anfang an sehr dünn, viel dünner, als die Ependymfasern der vorderen und hinteren Gegend und erreichen stets noch im Bereiche des kommissuralen Teiles der grauen Substanz ihr Ende, indem sie hier in der Regel in zwei bis drei Zweige zerfallen, die nach einem sehr unregelmässigen Verlauf bald frei auslaufen. Man darf also aus diesem Bilde auf einen Schwund des langen peripherischen Fortsatzes schliessen. Diese Atrophie beginnt ungefähr, wenn der menschliche Embryo 35 cm lang ist; noch bei dem 30 cm langen sah ich die Fasern bis zur Pia mater hinausziehen. Indessen scheinen hier individuelle Verschiedenheiten zu herrschen, denn Retzius hat schon bei 15 cm Länge eine Atrophie nachweisen können. Nach dieser Phase gelingt es dann seltener, einzelne Fasern soweit hinaus zu verfolgen, auch zeigen sie dann mehr und mehr durch ihre Schlängelungen und ihre sehr variköse Beschaffenheit Hinweise auf einen beginnenden körnigen Zerfall, bis sich allmählich das geschilderte reduzierte Bild einstellt. Indessen kann ich nicht umhin zu bemerken, dass man hier auch eine andere Deutung heranziehen könnte, bei der eine „Atrophie“ nicht in Betracht käme, die Auffassung nämlich, dass die kurzen Ependymzellen mit den früheren, langen nicht identisch sind, dass sie eine neue, schon ohne die langen Ausläufer sich anlegende

Zellengeneration darstellen, indem die früheren langen Ependymzellen alle aus dem Epithel hinausgewandert sind und sich zu Gliazellen umgeformt haben.

Es soll hier gleich darauf hingewiesen werden, dass diese Verkümmerung des Ependymgerüstes nur eine den höheren Formen zukommende Erscheinung ist, bei niederen Gattungen der Vertebraten spielt das Ependymium zeitlebens eine hervorragende Rolle. Auch möchte ich noch besonders betonen, dass sich die vorstehende Darstellung durchaus nur auf das Rückenmark bezieht. In manchen anderen Teilen des Centralnervensystems bleiben nämlich die Ependymzellen und Fasern auch nach vollendetem Wachstum in ihrer embryonalen Form erhalten, auch bei höheren Vertebraten. Hierauf näher einzugehen, liegt nicht im Plane dieser Arbeit[1]).

Dass der Centralkanal samt seinem Ependym bei dem Menschen keine funktionelle Bedeutung besitzt und mehr nur ein entwickelungsgeschichtliches Überbleibsel darstellt, ergiebt sich aus der bekannten Thatsache, dass sich am Centralkanal später sehr oft regressive Veränderungen einstellen, wie vor allem die Obliteration. Freilich ist in solchen Fällen stets die Frage zu erwägen, ob es sich nicht um ein Kunstprodukt handelt, um ein Erzeugnis der mechanischen Manipulationen bei der Herausnahme des Rückenmarkes? Wissen wir doch aus der sehr sorgfältigen experimentellen Arbeit Van Gieson's[2]), zu welch eingreifenden Veränderungen in der ganzen inneren Anordnung des Rückenmarkes scheinbar unbedeutende äussere Eingriffe führen können. Wenn durch ein unvorsichtiges Manipulieren bei der Herausnahme des Rückenmarkes so weitgehende Veränderungen, wie eine scheinbare Verdoppelung des Markes, „Heterotopieen" grauer Substanz u. s. w. zu stande kommen können, wie sehr muss derartigen Alterationen der kleine Centralkanal ausgesetzt sein.

Gehen wir nun über zur Entwickelung der eigentlichen „Gliazellen", unserer Astrocyten. Meine eigenen Erfahrungen über diesen Punkt, speziell was die ersten Entwickelungsphasen

[1]) Wer sich über das Verhalten der Stützelemente in anderen Provinzen des Centralnervensystems orientieren will, findet die gesuchten Angaben teils einzeln zerstreut in den betreffenden Spezialarbeiten, teils aber ausführlich zusammengestellt in der allerdings nicht einem Jeden leicht zugänglichen verdienstvollen Dissertation von Cl. Sala y Pons: La Neuroglia de los Vertebrados. Barcelona 1894.

[2]) Ira van Gieson, A study of the Artefacts of the Nervous System. New York medical Journal, 1892.

betrifft, beziehen sich hauptsächlich auf Hühnerembryonen, doch ersehe ich aus den in meinem Besitze befindlichen Präparaten von sehr jungen Säugerembryonen, dass die Sache auch hier im wesentlichen in der gleichen Weise vor sich geht.

Die Bildung der Astrocyten kündigt sich so viel ich sehe beim Hühnchen am 8. Tage an (Fig. 27); bis dahin sind nur Ependymzellen vorhanden. In dieser Phase nun gewahrt man, zu allererst im Bereich des Vorderhorns, einige Elemente, die an die Epen-

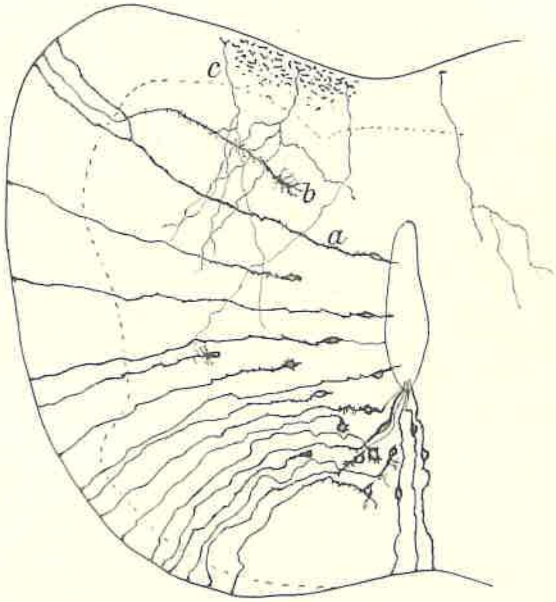

Fig. 27.

Rückenmark des 8tägigen Hühnchens. Entwickelung der Stützzellen. a Ependymzellen, b schon herausgerückte Ependymzelle (Astroblast), c Kollateralen des Vorderstranges.

dymzellen den frappantesten Anschluss zeigen, mit ihren faserförmigen Fortsetzungen wie diese die Peripherie des Markes erreichen, wo sie mit der bekannten dreieckigen Verbreiterung endigen, sich aber von den eigentlichen Ependymzellen dadurch unterscheiden, dass ihr Zellkörper nicht mehr am Centralkanal, sondern weiter auswärts liegt, mit dem Ependym höchstens durch einen sehr feinen Faden zusammenhängt, in der Regel aber mit ihm gar keine Verbindung mehr hat. Der Zellkörper erscheint spindelförmig, in der Richtung der peripherischen Faser verlängert,

in der ersten Phase vollkommen glatt. Anfangs findet man solche Zellen nur in den inneren Teilen der grauen Substanz und sehr spärlich, aber schon an den nächstfolgenden Tagen (Fig. 28) erscheinen sie etwas zahlreicher und dringen schon bis an den Rand der grauen Substanz hinaus.

Die hier geschilderte Erscheinung schliesst das wichtigste bei der Frage nach der Entstehungsweise

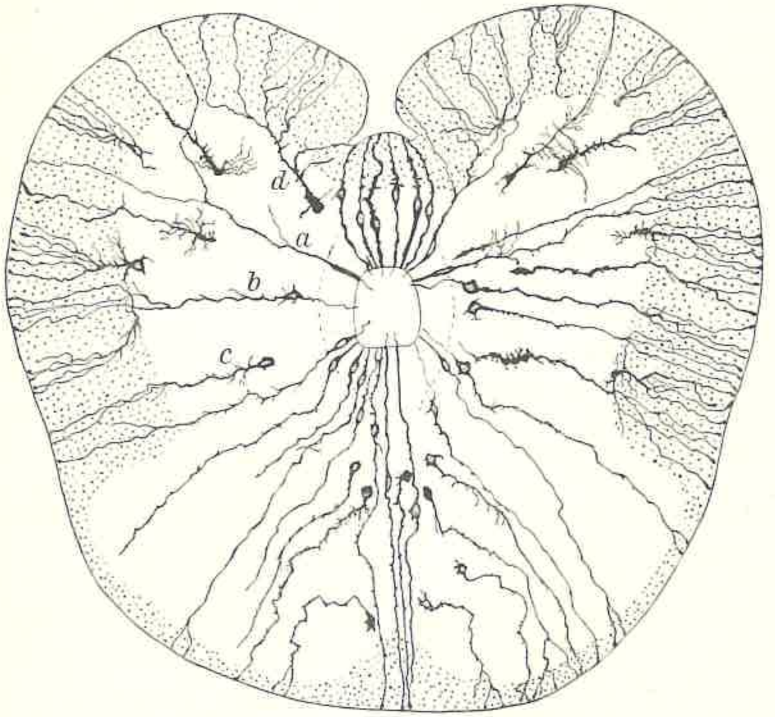

Fig. 28.
Entwickelung der Stützzellen im Rückenmarke des 10 tägigen Hühnchens. a Ependymzelle, b herausgewanderte Ependymzelle, die mit dem Centralkanal noch durch einen feinen Faden zusammenhängt, c Vorläufer der späteren Spinnenzellen.

der Gliazellen in Betracht kommende Faktum in sich, sie bildet den Schlüssel für das Verständnis dieses Problems. Es ist nicht schwer, durch Vergleichung aller Übergangsformen den bestimmten Nachweis zu führen, dass diese Elemente aus einem Zustande hervorgegangen sind, da sie genau, wie die Ependymzellen, am Centralkanale lagen, ja sogar, wie diese, mit einem Härchen versehen waren; sie entwickeln sich aus diesem Zustande

in der Weise, dass ihr Zellkörper allmählich aus dem Epithelverbande in centrifugaler Richtung herauswandert. Der an den Centralkanal befestigte Teil des Zellkörpers verdünnt sich anfangs dabei, er erhält sich noch eine Zeit lang samt dem „Stäbchen“ als äusserst zartes Fäserchen, schwindet aber bald, indem er offenbar an den Zellkörper herangezogen und zu seiner Vergrösserung aufgebraucht wird. Handelt es sich dabei um einen spontanen Bewegungsvorgang der Zelle oder um das Ergebnis eines durch den peripherischen Fortsatz ausgeübten Zuges? Wer könnte dies bestimmt entscheiden.

Die hier geschilderten Elemente sind die Vorläufer eines Teiles wenigstens der späteren Spinnenzellen, der Astrocyten (Astroblasten). Diese gehen also aus der gleichen Anlage wie die Nervenzellen hervor, nämlich aus den um den Centralkanal als Keimschichte aufgereihten Mitosen. Auch in der weiteren Entwickelung ist die Analogie mit Neuroblasten überraschend: wie bei jenen die Bildung des Nervenfortsatzes, so leitet sich hier schon im Momente der Entstehung die des peripherischen Ausläufers ein; wie jene, gelangen sie dann durch allmähliches Herausrücken vom Centralkanal her in äussere Gebiete des Markes, zuerst die graue, dann auch die weisse Substanz bevölkernd. Wahrscheinlich stellen die vielen Ependymzellen, die man bei Embryonen findet, nicht definitive Bildungen, sondern stets nur vorübergehende Entwickelungsstufen späterer Spinnenzellen dar, nur die letzte Ependymgeneration und die Ependymzellen an der Bodenplatte und der Deckplatte bleiben als Elemente des Ependyms auch im vollentwickelten Marke erhalten. Allem Anscheine nach spielt sich im Laufe der Embryonalentwickelung oder wenigstens in deren ersten Stadien ein kontinuierlicher Neubildungs- und Verschiebungsvorgang von Ependymzellen ab. Aus einem Teil der Mitosen in der innersten Markschichte entwickeln sich nicht Nervenzellen, sondern Elemente, die zur Peripherie des Rückenmarkes einen derben Fortsatz entsenden und in ihrem inneren Teil anfangs ganz den Habitus einer Epithelzelle darbieten. Dies ist das Ependymzellenstadium der Astrocyten. Darauf folgt dann die geschilderte Verkürzung des Faserteiles der Zelle, wodurch der Zellkörper nach aussen hin gezogen wird.

Wie gesagt, beginnt der Vorgang des Herauswanderns im Vorderhorn oder ist wenigstens hier zuerst nachweisbar. Sehr bald indes tauchen auch in den seitlichen Gebieten des Medullarrohres eben so wie auch im Hinterhorn ausgewanderte Ependym-

zellen auf. Noch beim 8tägigen Hühnchen (Fig. 27) beschränken sich diese Elemente auf die graue Substanz, aber schon am 10. Tage haben nicht nur viele davon den Rand der weissen erreicht, sondern man findet schon einige, die in diese letztere eingedrungen sind, doch ist deren Zahl noch sehr gering. Erst allmählich wandern sie mehr und mehr, durch zunehmende Verkürzung ihres peripherischen Fortsatzes, in die peripherischen Zonen des Rückenmarkes, in den um diese Zeit noch relativ schmalen Saum weisser Substanz hinaus. Es ist, als ob an der Grenze der grauen Substanz ein Hindernis für die hinausströmenden Astroblasten bestände, das sie erst nach einiger Anstrengung, nach einigem Verweilen an dieser Stelle zu überwinden im stande sind. Dem Gesagten entsprechen auch die Beobachtungen, die man an gewöhnlich gefärbten Serien junger Embryonen sowohl vom Vogel als von Säugern und vom Menschen machen kann. So findet man z. B. bei dem zweimonatlichen Embryo die graue Substanz dicht erfüllt von Kernen, die zu einem guten Teile den Kernen der herauswandernden Astroblasten entsprechen, während die weisse Substanz um diese Zeit noch fast ganz kernlos erscheint. Erst in späteren Stadien treten auch darin Gliakerne in die Erscheinung, durch Herausrücken solcher von der grauen Substanz her, in welch letzterer sich jetzt die Anordnung der Kerne mehr und mehr aufgelockert gestaltet. Schon Vignal hat diese Beobachtungen registriert und sie auch in dem hier dargelegten Sinne gedeutet; genauere Angaben finden sich hierüber in v. Kölliker's Gewebelehre (6. Aufl. 1893 p. 133).

Wahrscheinlich sind die zuerst entstehenden Astroblastengenerationen alle für die weisse Substanz bestimmt und nur die späteren bleiben in der grauen. Hiefür können wir einen sehr bestimmten Beweis in der in Fig. 24 reproduzierten Retzius'schen Zeichnung erblicken, die den Zustand der Stützzellen bei dem 3 cm langen Embryo vergegenwärtigt. Vergleichen wir sie mit unserer Tafel II, die einem späteren Stadium — 14 cm Länge — entnommen ist. Man erkennt, dass in der Retzius'schen Zeichnung bei den meisten Astroblasten der Fortsatz im Bereich der weissen Substanz verästelt ist, während an meiner Figur nur die der weissen Substanz angehörigen Elemente dieses Verhalten erkennen lassen. Allem Anscheine nach sind auch alle Ependymzellen, die diese Figur enthält, Vorläufer von Astrocyten der weissen Substanz, worauf die reichliche Teilung ihres Ausläufers hinweist. Höchst wahrscheinlich ist von den späteren Glia-Elementen der grauen Substanz in dieser Periode noch nicht viel angelegt.

Während ihres Hinauswanderns stellt sich an den Stützzellen noch eine Erscheinung ein, die mit dem Vorgang der Dendritenbildung an den Neuroblasten die frappanteste Analogie aufweist: an dem anfangs glatten Zellkörper treten nämlich sehr frühzeitig kleine Spitzen, Ästchen in die Erscheinung, die ebenso wie die Dendriten, aus dem Zellprotoplasma in Form von Hervorragungen entstehen. Dies sind die „sekundären Ästchen" der Astroblasten.

Die Fädchen erstrecken sich bei den meisten Zellen auch auf ein kürzeres oder längeres Stück des Fortsatzes.

Auf dem geschilderten Übergangsstadium verharrt das Stützsystem des Markes durch eine lange Periode hindurch; beide Abbildungen, Fig. 24 und Tafel II, obgleich weit auseinander liegenden Entwickelungsstufen des Embryos entnommen, stellen diesen Zustand dar. Gleichwohl ergiebt eine genauere Vergleichung der beiden Figuren nicht unwesentliche Unterschiede, vor allem die Thatsache, dass bei dem 3 cm langen Embryo die herausgewanderten Astroblasten noch spärlich sind und sich auf die graue Substanz beschränken, während sie bei dem 14 cm langen nicht nur an Zahl beträchtlich zugenommen haben, sondern auch eine mehr gleichmässige Verteilung über den Querschnitt des Rückenmarkes erkennen lassen.

Unterwerfen wir noch die Figur auf Tafel II (14 cm langer Embryo) einer etwas genaueren Analyse speziell mit Rücksicht auf die in der rechten Hälfte dargestellten Stützzellen.

Wir sehen den Querschnitt des Markes von einem dichten System kräftiger, radiärer Fasern säulenartig durchsetzt, die von inneren Teilen in einer bestimmten, durch die Ependymfasern vorgezeichneten Richtung nach der Oberfläche hin ausstrahlen und das Bild des Stützgerüstes vollkommen beherrschen. Sie gehen von den noch immer länglichen und noch immer erst mit ganz kurzen Ästchen versehenen Zellkörpern aus.

Das Verhalten ist etwas anderes in der grauen und in der weissen Substanz. In der grauen setzt sich jede Zelle überall nur in einen einzigen peripherischen Ausläufer fort, der sich erst in den äussersten Gebieten des Markes, nahe an dem Rand, in eine Anzahl — in der Regel 3—4 — divergierende Äste zerfasert, die alle die Pia mater erreichen, um an sie mit den schon von den Ependymfasern her bekannten kleinen Terminalklümpchen heran-

zutreten. Soweit er in seinem Lauf der grauen Substanz angehört, erscheint der Fortsatz im Gegensatz zu den glatten Ependymfasern überall mit zahlreichen minimalen Fäserchen und Anhängseln besetzt, während er im Bereich der weissen Substanz eher von glatter, gleichmässiger Beschaffenheit ist.

Höchst charakteristisch ist in diesem Stadium die Anordnung der Neurogliafaserung im Gebiet der hinteren grauen Kommissur und zu beiden Seiten davon. Bei der Unmöglichkeit, die etwas umständlichen Verhältnisse mit einigen Worten anschaulich zu schildern, möchte ich die Blicke des Lesers auf Tafel II lenken. Die Zellkörper erscheinen hier nicht, wie bei den übrigen Stützelementen, radiär zum Centralkanal orientiert, sondern stehen mitsamt dem Anfangsteil ihres peripherischen Ausläufers senkrecht oder schief zum Septum posterius. Die aus ihnen entspringenden Ausläufer reihen sich, anstatt einfach radiär zur Oberfläche zu laufen, in S-förmig geschwungenen Kurven aneinander, ungefähr parallel der Grenze zwischen Hinterstrang und grauer Substanz. Der quere Anfangsteil lenkt unter sanfter Biegung oder oft auch stärker accentuierter Knickung in den weiteren, sagittalen Abschnitt über, der mit schwacher, nach innen gewendeter Konvexität Hinterhorn und Hinterstrang durchsetzt, um stets ungeteilt die Oberfläche zu erreichen. Dabei ziehen die Fasern nicht genau parallel miteinander, sondern beschreiben, je mehr lateral gelegen, desto ausgeprägtere Kurven, wodurch ihre vorderen Abteilungen eine konvergierende, ihre hinteren eine divergierende Anordnung gewinnen. Die Stelle, wo sie am engsten zusammentreten, entspricht derjenigen Gegend des medialen Hinterhornabschnittes, die die „Einstrahlungsbündel" der hinteren Wurzel als Eintrittspforte in die graue Substanz benützen. Auch die weiter aussen im Hinterhorn befindlichen Stützzellen zeigen die Tendenz, sich mit dem Zellkörper und dem ersten Abschnitt des Fortsatzes quer zur Mittellinie zu richten und so einen Bogen zu bilden. Man darf sagen, dass während in den vorderen und mittleren Teilen des Querschnittes die Anordnung der Stützzellen vom Centralkanal als dem Mittelpunkt ihrer radiären Ausbreitung beherrscht wird, für die Lagerung der dahinter befindlichen Gliaelemente der massgebende Faktor im Septum post., d. h. in dem obliterierten Abschnitt des Centralkanales gegeben ist. Ich bemerke schliesslich, dass wir die erste Reproduktion der geschilderten Anordnung v. Kölliker verdanken. Die Fig. 28 seiner Rückenmarksarbeit (Rückenmark des Schafes) lässt sie ganz deutlich erkennen. Ausführlich habe ich sie zuerst in meiner Neurogliaarbeit[1]) beschrieben, Retzius hat unlängst meine Angaben bestätigt.

Diese Anordnung hängt, wie schon oben angedeutet, mit den Obliterationsverhältnissen des Centralkanales zusammen. Die merkwürdige Stellung der Zellkörper lässt sich daraus erklären, dass diese Zellen ursprünglich die Ependymzellen des dorsalen, obliterierenden Teiles des Centralkanales gebildet haben und bei ihrer Herauswanderung und Umgestaltung zu Stützzellen ihre ursprüngliche Orientierung eine Zeit lang noch beibehalten.

Die Astroblasten, die um diese Zeit in der Rolando'schen Substanz liegen, zeichnen sich durch eine sehr auffallende Besonderheit aus. Ich finde sie, ebenso wie schon früher Cajal bei der neugeborenen Katze, mit einem so reichen Buschwerk ausserordentlich zarter Fasern versehen, wie es sonst an keiner anderen Stelle des embryonalen Rückenmarkes vorkommt. Ja sogar die Fasern, die aus weiter vorne gelegenen Astroblasten der Hinterhörner entspringend, die Rolando'sche Substanz bloss zum Durchtritt benutzen, weisen — soweit sie der Substanz angehören — diesen dichten pelzigen Überzug auf.

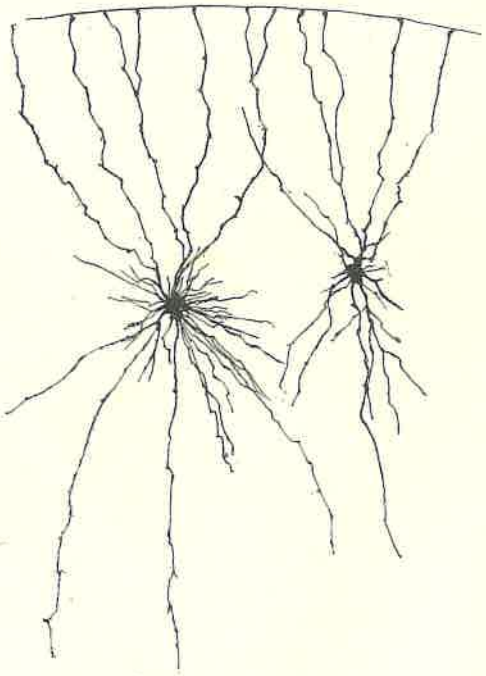

Fig. 29.
Stützzellen aus der weissen Substanz des Rückenmarkes eines 30 cm langen Embryos.

Die vielen Spinnenzellen, aus denen sich bei dem Embryo die Neuroglia der weissen Substanz zusammensetzt, schliessen sich zum kleineren Teile an die der grauen Substanz eigentümliche Zellform an, der Mehrzahl nach aber besitzen sie ihren eigenen, sehr charakteristischen Habitus (Fig. 29).

---

1) M. v. Lenhossék, Zur Kenntnis der Neuroglia des menschlichen Rückenmarkes. Verhandl. d. anat. Gesellsch., V. Vers. München 1891, p. 193.

Ihr Hauptmerkmal besteht darin, dass sie statt eines einzigen, mehrere peripherische, bis an die Pia mater herantretende Fortsätze, oft ein ganzes Büschel solcher aufweisen. Der Verlauf dieser ist kein gerader, gestreckter, sie ziehen nicht einfach pinselförmig an den Rand, sondern weichen breit, armleuchterartig auseinander. Die grösste Anzahl solcher Ausläufer lassen die Gliazellen im Bereich des eingerollten, der vorderen Fissur zugekehrten Abschnittes der Vorderstränge erkennen. Hier zeigen also die Stützzellen schon frühzeitig ein Verhalten, das ihrem späteren Typus sehr nahekommt. Es herrscht übrigens eine grosse Mannigfaltigkeit der Formen; der Zellkörper erscheint um diese Zeit noch zumeist in die Länge gezogen, aber oft quergestellt, bogenförmig gekrümmt, gleichsam den Scheitel des durch die peripherische Verästelung gebildeten Bogens darstellend.

Das im Vorstehenden geschilderte radiäre Stützsystem bildet bei dem Menschen und den höheren Säugern im wesentlichen nur eine embryonale Erscheinung. Noch bei dem 18 cm langen Embryo finde ich diesen Typus ausgeprägt, aber von diesem Zeitpunkte an leitet sich mehr und mehr eine eingreifende Metamorphose ein; allmählich weicht der radiäre Typus der späteren Anordnung. Schon an Präparaten vom 23 cm langen Embryo zeigen die Stützzellen der grauen Substanz ein etwas anderes Gepräge. Die in der früheren Epoche so rudimentären kleinen sekundären Ästchen sind nun zu enormer Entwickelung gelangt, sowohl was ihre Zahl als auch was ihre Länge betrifft, die Zellen erscheinen nun mehr und mehr unter dem Bilde typischer Spinnenzellen, wenn auch noch nicht mit so komplizierter Verästelung, wie später. Nur da und dort gewahrt man noch in der grauen Substanz Zellen, die wie früher einem langen radiären Fortsatze zum Ausgangspunkte dienen, bei den meisten tritt dieser nun ganz zurück, und die allseitig vom Zellkörper ausstrahlenden sekundären Fädchen gewinnen die Oberhand, wobei der Zellkörper seine frühere längliche Form gegen eine gleichmässig sternförmige eintauscht. Allmählich werden die radiären Stützzellen durch die einen anderen Typus aufweisenden Spinnenzellen ersetzt, die nun in grosser Zahl die graue und teilweise auch die weisse Substanz bevölkern. Nur in der letzteren behalten viele, aber auch nicht alle Stützzellen ihren primitiven Habitus bis zu einem gewissen Grade bei, indem sie nach wie vor eine Anzahl von Ästen an die Peripherie des Rückenmarkes in radiärer Anordnung herantreten lassen.

Es fragt sich nun, wie sind diese neuen Zellen entstanden? Ein grosser Teil der Spinnenzellen lässt sich ohne Frage zurückführen auf die embryonalen radiären Stützzellen. Man darf annehmen, dass die Radiärzellen sich zu Spinnenzellen in der Weise umgestalten, dass sich ihre schon in den ersten Phasen in Form von feinen Fädchen angelegten sekundären Äste nun stärker entwickeln, ihr früher so mächtiger peripherischer Fortsatz hingegen einer Art Atrophie unterliegt, wofür an manchen Präparaten in der That direkte Anhaltspunkte vorzuliegen scheinen. Jedenfalls ist dies der ursprüngliche, typische Bildungsvorgang der Spinnenzellen. Die Astrocyten, die sich nach dieser Art entwickeln, gehen also durch drei Entwickelungsstadien hindurch: zuerst durch das Stadium der Ependymzelle, dann vermöge der excentrischen Dislokation des Zellkörpers durch das des radiären Astroblasten, aus diesem Stadium geht erst schliesslich durch Atrophie des Ausläufers die Spinnenzelle hervor.

Gilt aber dieser Entwickelungsmodus ausnahmslos für alle Spinnenzellen? Teils durch direkte Beobachtungen, teils durch verschiedene Erwägungen bin ich zur Überzeugung gekommen, dass man wenigstens beim Menschen diese Entstehungsweise nicht für alle Astrocyten in Anspruch nehmen darf. Dazu ist nicht genug Kontinuität zwischen dem ersten, dem radiären Stadium, und dem zweiten, dem Spinnenzellenzustand vorhanden, man müsste einen allmählichen Übergang, überall Erscheinungen einer allmählichen Reduktion des peripherischen Fortsatzes, eine zunehmende Umbildung zu Spinnenzellen nachweisen können. Dem ist aber nicht so. Ziemlich unvermittelt tauchen, wenn der Embryo ungefähr 20 cm lang ist, die Spinnenzellen, schon in ihrer charakteristischen Form, zuerst wohl in der Substantia gelatinosa centralis, dann aber auch weiter aussen auf, und bei vielen fehlt jeder Hinweis darauf, dass sie sich aus den Radiärzellen entwickelt haben. Dann ist die Zahl der späteren Spinnenzellen im menschlichen Rückenmarke auch viel zu gross, als dass man sie alle auf frühere Radiärzellen, die eine viel beschränktere Zahl aufweisen, zurückführen könnte. Ich möchte daher die Ansicht aufstellen, dass bei vielen Spinnenzellen, namentlich denjenigen der grauen Substanz, jener umständliche Entwickelungsmodus caenogenetisch durch einen wesentlich abgekürzten, einen einfacheren ersetzt wird, indem sie nicht durch jenes radiär-faserige Stadium hindurchgehen, sondern — entweder in den inneren Lagen des Markes oder

auch weiter auswärts gleich an jener Stelle, wo sie später sitzen[1]) — aus Keimzellen als kleine, anfangs fortsatzlose, sich aber bald mit einem Rasen von allseitig auswachsenden Fortsätzen umgebende typische Astrocyten entstehen, bei denen jener starke peripherische, ohnedies dem Untergang geweihte Hauptfortsatz gar nicht mehr zur Entwickelung kommt. Diese Entstehungsweise würde mehr der Auffassung entsprechen, die sich frühere Forscher, wie Boll, Vignal, Gierke, und von den neueren Kölliker von der Bildungsweise der Spinnenzellen gebildet haben, welch letzterer diese Elemente auf „indifferente Zellen der Markanlage“ zurückführt.

Auf alle Fälle aber, ob nun die Astrocyten so oder so entstehen, ein wesentlicher Unterschied zwischen ihnen besteht, wenn sie fertig sind, in ihrer äusseren Erscheinung nicht, und was die Hauptsache ist: alle haben dieselbe Herkunft. Denn beide Arten, die aus den Radiärzellen hervorgehenden wie die direkt entstehenden, sind ektodermale Elemente, beide sind Abkömmlinge von Bildungszellen, die von vornherein dem Marke selbst angehören und in der letzten Analyse auf Stammzellen zurückzuführen sind, die als Bestandteile der Medullarplatten dem äusseren Keimblatt entstammen. Man kann in dem direkten Bildungsmodus nur eine Abkürzung des indirekten erblicken.

In den letzten Monaten der intrauterinen Periode verändert sich der Habitus der Astrocyten nur mehr sehr wenig. Das Wesentlichste ist, dass sie gegenüber der Gesamtzunahme des Markes in ihrer Grössenentwickelung zurückbleiben, daher sie relativ immer kleiner und kleiner erscheinen. Wahrscheinlich nehmen sie noch bis in die letzten Phasen an Zahl zu, wofür man aber erst dann bestimmtere Anhaltspunkte gewinnen wird, wenn das Mark einmal in seinen letzten Entwickelungsstufen auf die Gegenwart von Mitosen gründlich untersucht sein wird. Das Breitenwachstum des Rückenmarkes lässt sich allerdings auch aus anderen Momenten, wie die Substanzzunahme der Nervenzellen und vor allem das Auftreten der Markscheiden erklären. — Eine zweite Erscheinung, die sich noch an den Astrocyten in den letzten Monaten nachweisen lässt, ist die allmählich reichere Entfaltung ihrer Fortsätze.

[1]) Um diese Alternative zu entscheiden, müsste das Rückenmark von höheren Säugern in den späteren Stadien seiner Entwickelung auf den eventuellen Gehalt an Mitosen und auf die Lagerung derselben untersucht werden, eine Arbeit, die noch aussteht.

Die Herkunft aus dem Ektoderm gilt also meiner Überzeugung nach ausnahmslos für alle Elemente des Stützsystems, für alle Astrocyten. Ich finde mich hierin im Widerspruch mit einer früheren, vielleicht auch heute schon nicht mehr festgehaltenen Angabe R. y Cajals, wonach zu der ektodermalen Anlage der Neuroglia in vorgeschritteneren Stadien der Entwickelung noch eine wenn auch geringe mesodermale Zuthat hinzutrete in Form von Gliazellen, die von etwas abweichendem Typus und vielleicht von bindegewebiger Natur seien, sei es dass sie ausgewanderten Leukocyten, sei es dass sie aus den Blutgefässen abgelösten Endothelzellen entsprächen. Ich habe für eine solche Auffassung auch nicht die geringsten Anhaltspunkte gewonnen. Da alle Langstrahler im wesentlichen von gleichartiger Beschaffenheit sind, und für einen Teil davon die Entstehung aus den ektodermalen Ependymzellen Schritt für Schritt zu verfolgen ist, so ist kein Grund anzunehmen, dass die anderen von so prinzipiell verschiedener Natur seien. Man könnte dabei höchstens noch an die in ihrem Habitus allerdings etwas abweichenden Kurzstrahler denken, indessen sprechen einerseits die nicht gerade seltenen, auch von Kölliker betonten Übergangsformen zu den Langstrahlern gegen eine verschiedene Herkunft dieser Elemente, andererseits noch überzeugender aber der Umstand, dass es mir in mehreren Fällen gelang, in frühen Phasen die Andeutung eines peripherischen Fortsatzes an Zellen dieser Art nachzuweisen (Fig. 30), worin man ein entscheidendes Merkmal für ihre ektodermale Entstehung erblicken kann.

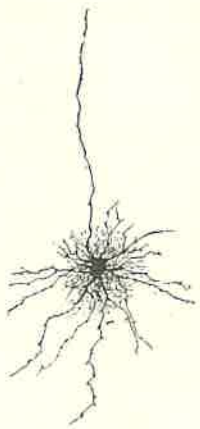

Fig. 30.
Kurzstrahler mit noch vorhandenem peripherischen Ausläufer, aus dem Rückenmarke eines 30 cm langen Embryos.

Wenn nun auch alle Stützzellen als Bildungen des Ektoderms, als ureigene Elemente des Medullarrohres zugegeben werden, so bleibt immer noch die Frage zu diskutieren, ob nicht in einer anderen Form wahres Bindegewebe — abgesehen von den Blutgefässen — in den Aufbau des Rückenmarkes eintritt. Es ist hier namentlich an die vielen Angaben von den von der Peripherie her eindringenden Pialfortsätzen zu erinnern.

Schon im vorhergehenden wurde mehrmals der Überzeugung Ausdruck gegeben, dass diese Darstellungen alle auf Täuschung

beruhen. Die „Pialsepta“ sind nichts anderes als zu gröberen Bündeln zusammengefasste Komplexe von Spinnenzellen und ihren Fortsätzen; das Peridym, d. h. die oberflächliche Randschichte des Markes besteht nicht aus Pialgewebe, sondern aus einem Gewirr von tangentialen Spinnenzellen. Die scharfe Abgrenzung dieses Peridyms von der Pia mater kommt bei keiner Behandlungsweise schöner zur Ansicht als bei der von Schaffer eingeführten Methode, die in einer nach besonderen Vorschriften vorgenommenen Doppelfärbung in Hämatoxylin und Eosin besteht. Alles, was zu der „Neuroglia“ gehört, also auch das Peridym, nimmt an solchen Präparaten die prächtig rote Eosinfärbung an, während die bindegewebige Pia mater braun erscheint, von dem Peridym überall scharf getrennt. Auch die Blutgefässe grenzen sich samt ihrer Adventitia von der medullaren Randschichte durch die gleiche Tinktionsdifferenz sehr bestimmt ab. Ist ein Blutgefäss auf dem Schnitt gerade tangential getroffen, so dass nur die Adventitia, nicht aber das Lumen auf dem Präparate erscheint, so kann es den Anschein haben, als läge eine „Pialeinsenkung“ vor. Serienschnitte, wie sie Schaffer untersucht hat, klären hier den Irrtum auf. Aber Schaffer behauptet, in sehr seltenen Fällen doch einige ganz kleine in die Rückenmarkssubstanz einbiegende Bindegewebszüge wahrgenommen zu haben, bei denen sich ein dazugehöriges Blutgefäss nicht nachweisen liess. Jedenfalls aber kann dieses Verhalten, wenn es wirklich zutrifft, als ein äusserst seltenes Vorkommnis ausser Acht gelassen werden und könnte etwa durch die Annahme seine Erklärung finden, dass es sich um die Reste der Adventitia von Blutgefässen handelt, die einer regressiven Veränderung anheimgefallen sind.

Ich habe, um in dieser in vieler Beziehung, namentlich für die Pathologie wichtigen Frage noch weitere Sicherheit zu erhalten, auch jene Modifikation der Chromsilbermethode nicht unversucht gelassen, durch die Oppel[1]) das feine Bindegewebsgerüst der Leber, der Milz und der Lymphdrüsen in so gelungener Weise darzustellen vermochte, habe aber auch damit vollkommen negative Resultate erhalten. Nirgends hat sich in der Rückenmarkssubstanz, ausser an den Blutgefässen, Bindegewebe gefärbt, und wenn dies auch nur ein negatives, den Einwand des technischen Misslingens der Färbung nicht ausschliessendes Ergebnis ist, so darf es doch auch wohl neben anderen Beweisen mit in die Wagschale fallen.

[1]) A. Oppel, Eine Methode zur Darstellung feinerer Strukturverhältnisse der Leber. Anat. Anz., Jahrg. V, 1890, p. 143.

Es wäre noch ein Wort zu sagen über die Adventitia der das Rückenmark durchspinnenden Blutgefässe. An den Kapillaren ist eine solche gar nicht vorhanden, aber auch an den stärkeren Stämmchen spielt sie eine sehr untergeordnete Rolle. Was aber das wichtigste ist: sie besitzt keinen Zusammenhang mit der eigentlichen Rückenmarkssubstanz, mit der „Neuroglia“, diese schliesst sich gegen die Blutgefässe vollkommen ab.

Ich möchte also mit aller Entschiedenheit gleich v. Kölliker gegen jeden Versuch einer dualistischen Auffassung der Neuroglia Stellung nehmen. Wir dürfen meiner Überzeugung nach nicht mehr zögern, die Frage, ob im Rückenmark überhaupt Bindegewebe enthalten sei, auf Grund der Golgi'schen und anderer Bilder bestimmt zu verneinen, natürlich abgesehen von den das Mark durchziehenden Blutgefässen. Die früher so vielfach geäusserten Angaben vom Eindringen von Bindegewebsbalken von der Pia mater her beruhen, wie das schon Boll erkannt hatte, auf einer Verwechselung mit Gliafasern. Alle Fasergebilde in der Stützsubstanz des Markes stellen Fortsätze von Astrocyten dar; das Rückenmark erscheint uns als durch und durch ektodermales Organ, das auch seine inneren Stützvorrichtungen aus eigenen Mitteln zu bestreiten in der Lage ist und nur das zu seiner Ernährung dienende Kanalsystem, samt Inhalt natürlich, einer fremden Hilfe entlehnt.

Wenn sich in dieser Hinsicht ein begründetes Urteil abgeben lässt, so bereitet eine andere Frage ungleich mehr Schwierigkeiten: diejenige nämlich nach der Existenz oder Nichtexistenz jener „Grundsubstanz“, die bald als körnige oder netzförmige, bald als homogene Masse in den Darstellungen aller früheren Forscher, namentlich in denen Boll's und Gierke's, eine so hervorragende Rolle spielt. Positive Angaben in dieser Richtung können wir kaum zugeben, enthüllen uns doch unsere neuen Methoden auch an Stellen, wo man früher nur eine strukturlose Masse sah, einen wunderbaren Reichtum an Fasergebilden und mahnen uns daher zum grössten Skepticismus in der Aufnahme solcher Angaben. Als Postulat kann die Hypothese einer Grundsubstanz wohl auch nicht gelten, denn angesichts des grossartigen, teils aus den Verästelungen der Nervenelemente, teils aus den Ausbreitungen der Astrocyten hervorgehenden Filzes, der uns an Golgi'schen Präparaten entgegentritt, könnte man sich schliesslich den Aufbau des Markes auch ohne den Notbehelf einer eigentlichen Verbindungsmasse, bloss aus der Verfilzung der Fasern, wie etwa einen Ballen

festverfilzter Haare, vorstellen, unter Herbeiziehung etwa einer die vielleicht vorhandenen minimalen Zwischenräume durchtränkenden serösen Flüssigkeit.

Werfen wir nach dieser gedrängten Schilderung noch einen Blick auf die Beschaffenheit der Stützzellen im Rückenmarke anderer Säuger und Vertebraten, soweit sie bisher untersucht sind. Drei wichtige Sätze lassen sich aus den auf diesem Gebiete gewonnenen Erfahrungen ableiten: 1. Dass das Stützsystem des Markes überall aus Zellen besteht, aus Ependymzellen und mehr oder weniger verzweigten Stützzellen (Gliazellen) und dass diese Elemente alle gleich den Nervenzellen ektodermaler Abkunft sind, im Rückenmarke selbst entstehen. 2. Dass die Anordnung der Stützzellen bei den verschiedenen Tieren zwar gewisse Differenzen erkennen lässt, solange man bei dem Vergleiche nur den Zustand des vollkommen entwickelten Markes bei höheren und niederen Wirbeltieren berücksichtigt, dass aber diese Unterschiede in sehr natürlicher Weise ihre Erklärung finden, sowie man zurückgeht auf die Entwickelungsvorgänge des Markes. Denn es ergiebt sich die hochinteressante Thatsache, dass der einfachere Typus, den das Stützsystem bei niederen Vertebraten aufweist, denselben Zuständen entspricht, die von den höheren und höchsten Formen als vorübergehende, embryonale Stadien durchlaufen werden. So schliesst also auch die Anordnung der Stützzellen im Marke überzeugende Beweise in sich einerseits für die Gemeinsamkeit der Organisation der Wirbeltiere, andererseits für den Satz, dass phylogenetische Entwickelungszustände in der individuellen Entwickelung höherer Formen vielfach ihre Wiederholung finden.

Hochinteressant ist das Verhalten des Stützsystems im Rückenmark des Amphioxus. Wie zuerst Nansen[1]) und Rohde[2]) nachgewiesen haben und wie ich es kürzlich mit der Golgi'schen Methode, wenn auch in fragmentarischer Weise, bestätigen konnte, wird das gesamte Stützgerüst durch die Ependymfasern, die sich radiär vom Centralkanal gegen die Oberfläche ausbreiten, dargestellt. Ich finde die Ependymfasern ziemlich derb und ungeteilt. Bei dem Mangel von eigentlichen Gliazellen sehen wir also bei Amphioxus den Zustand, der bei den Vertebraten die allerfrüheste Phase der Entwickelung darstellt, als dauernde Einrichtung realisiert. Auch v. Kölliker hat unlängst von den Stützzellen des Amphioxusrückenmarkes eine ähnliche Darstellung gegeben.

Im Cyklostomenrückenmark wurden die Stützzellen zuerst von Nansen, dann von Retzius[3]) mit der Golgi'schen Methode dargestellt, und zwar bezogen sich die Schilderungen und Abbildungen beider Forscher auf Myxine. In der I. Auflage dieses Werkes vermochte ich diesen Darstellungen ein Bild der Neuroglia aus dem Rückenmark des Neunauges (s. die beistehende Figur 31) beizufügen.

1) Fr. Nansen, Structur and Combination of the Histological Elements of the Central Nervous System. Bergen's Museums Aarsberetning for 1886 Bergen 1887, p. 160.

2) E. Rohde, Histologische Untersuchungen über das Nervensystem von Amphioxus lanceolatus. Schneider's Zoolog. Beitr., Bd. 2, H. 2, Breslau 1888.

3) G. Retzius, Zur Kenntnis des centralen Nervensystems von Myxine glutinosa. Biolog. Untersuchungen, N. F. II, Stockholm 1891, p. 51.

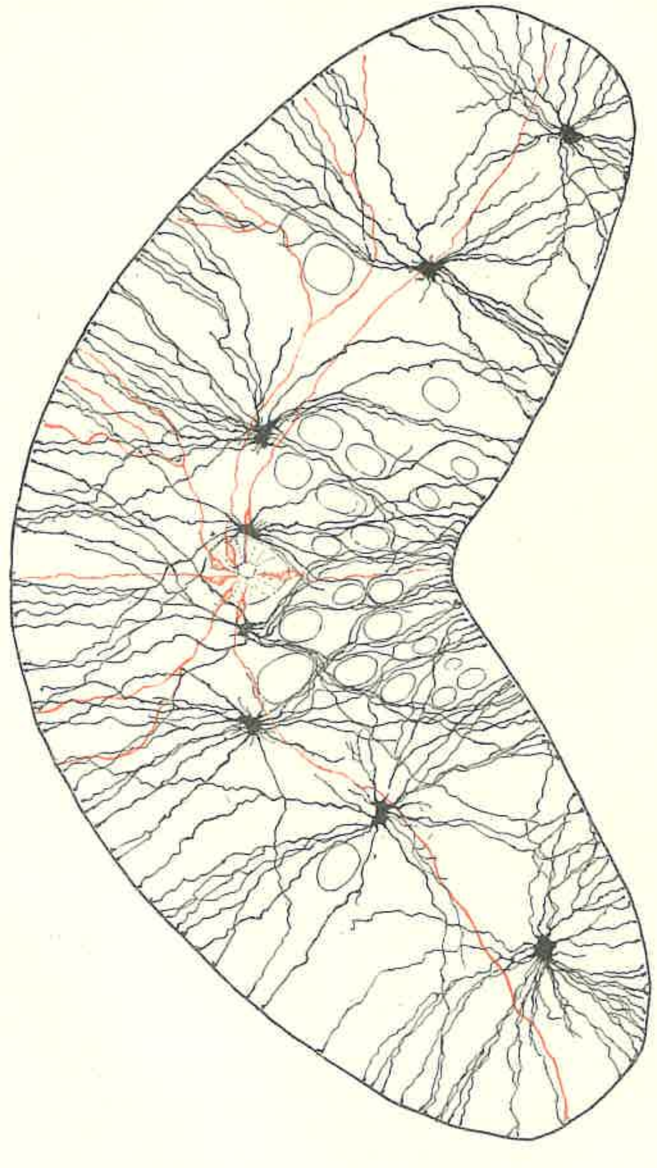

Fig. 31.

Rückenmark des Petromyzon mit imprägnierten Stützzellen. Ependymzellen rot, Astrocyten schwarz.

Die Ependymzellen erscheinen glatt, zart und sind sehr spärlich; dagegen finden wir zahlreiche Astrocyten, die sich aber nicht gleichmässig über den ganzen Querschnitt verteilen, sondern sich mit ihrem Zellkörper hauptsächlich auf die einem Streifen ähnliche graue Substanz beschränken. Sie zeichnen sich durch enorme Verästelung aus. Jede Zelle lässt sowohl an die ventrale wie an die dorsale Fläche des Markes ein Buschwerk von Zweigen herantreten, die am seitlichsten gelegenen auch an die laterale Kante des Markes. Die meisten Äste erreichen die Peripherie und endigen da mit kleinen Knötchen. Die medialen Äste der zu beiden Seiten der Mittellinie befindlichen Zellen kreuzen sich vielfach vor und hinter dem Centralkanal. Diese Beschreibung wurde unlängst von Retzius[1]) konstatiert. Bei Myxine scheint nach Nansen und Retzius insofern ein kleiner Unterschied zu bestehen, als die

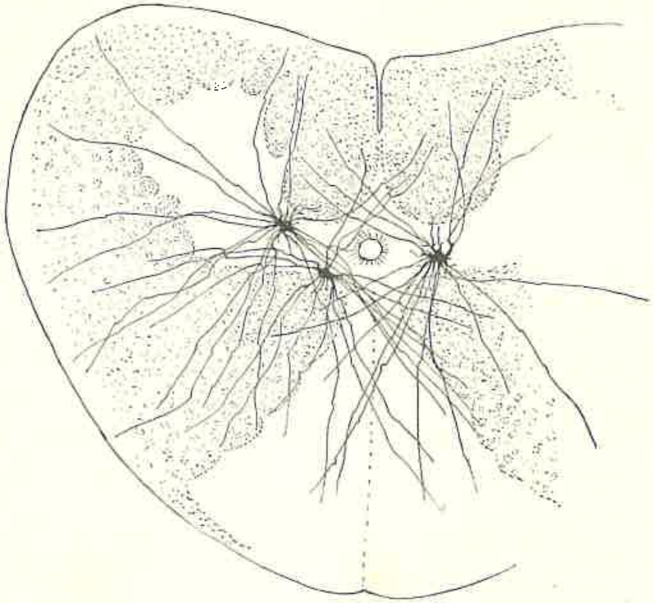

Fig. 32.
Stützzellen aus dem Rückenmarke eines 16 cm langen Acanthiasembryos.

Astrocyten ihre Äste zuweilen nicht nach den beiden Seiten des Markes, sondern nur nach der einen, sei es die dorsale oder die ventrale, hinstrahlen lassen; nur einigen kommt eine bilaterale Verästelung, wie bei Petromyzon, zu.

Von Selachiern ist mir die Darstellung der Glia mit der Golgi'schen Methode bei Acanthias, Scyllium[2]) und bei Raja[3]) gelungen. Die vorstehende Abbildung (Fig. 32) zeigt einige imprägnierte Astrocyten im Rückenmarke eines

[1]) G. Retzius, Studien über Ependym und Neuroglia. Biolog. Untersuchungen, N. F., V, 1893, p. 16.

[2]) M. v. Lenhossék, Beobachtungen an den Spinalganglien und dem Rückenmarke von Pristiurusembryonen. Anat. Anz., Jahrg. VII, 1892, p. 536.

[3]) M. v. Lenhossék, Zur Kenntnis des Rückenmarkes der Rochen. In: Beitr. z. Histolog. d. Nervensystems u. d. Sinnesorgane. Wiesbaden 1894, p. 60.

16 cm langen Acanthiasembryos. Sie zeigen mit dem Verhalten der analogen Elemente im Rückenmark von Petromyzon eine grosse Ähnlichkeit. Auch hier scheint die sehr merkwürdig geformte graue Substanz die Hauptträgerin der Zellkörper der Astrocyten zu sein, auch hier zeichnen sich diese Elemente durch ihre langen, starren, allseitig ausstrahlenden, sich vor und hinter dem Centralkanal schön regelmässig kreuzenden Äste aus, doch scheinen sie mir nicht ganz bis zur Oberfläche hinauszudringen, sondern schon in deren Nähe, schon unterhalb des hier sehr mächtig entwickelten oberflächlichen Dendritengeflechtes ihr Ende zu finden. Die Ependymzellen vermochte ich bei Acanthias nicht darzustellen, dagegen gelang mir ihre Imprägnation bei Raja, wo sie sich als zarte, ungeteilte, spärliche, bis zur Oberfläche hinausziehende Fasern ergaben. Die eigentlichen Gliazellen präsentierten sich bei Raja unter einer Form, die von der bei Acanthias und auch Scyllium verwirklichten gänzlich abwich und genau dem Verhalten dieser Zellen bei Amphibien entsprach: sie stellten sich genau wie die „Astroblasten" der Säuger und Vögel dar, als längliche, mit einem feinen Flaum versehene, sich nach aussen hin in einen einzigen derben peripherischen Ausläufer fortsetzende Zellen.

Über die Stützzellen im Rückenmarke der Knochenfische sind wir noch sehr wenig unterrichtet. Die einzigen hierhergehörigen auf der Golgischen Methode beruhenden Angaben finden sich in dem so wichtigen Neurogliaaufsatze von Retzius, (Biolog. Unters. V., p. 18), doch scheinen sich auch diese weniger auf das völlig entwickelte als vielmehr auf das noch in der Entwickelung begriffene Rückenmark zu beziehen. Merkwürdig plump und pelzig erscheinen die Ependymzellen, die spärlichen Gliazellen, die an den Retziusschen Bildern zu sehen sind, stellen auch keine eigentlichen Astrocyten dar, sondern nur etwas herausgerückte Ependymzellen. Sollten diese Bilder wirklich den fertigen Zustand des Teleostierrückenmarkes wiedergeben, was ich bezweifeln möchte, so hätte man es hier mit einem Typus zu thun, der tief unter dem bei den Selachiern, ja sogar den Cyclostomen realisierten stünde.

Die Neuroglia des Batrachierrückenmarkes wurde schon früher von Lawdowsky[1]) und dann unlängst von Cl. Sala, einem Schüler Cajals[2]) sowie auch von Retzius in genauester Weise beschrieben. Ich vermag die Schilderung Sala's, die sich auf das entwickelte Rückenmark bezieht, auf Grund eigener Präparate zu bestätigen. Auffallend ist vor allem die grobe und pelzige Beschaffenheit der Ependymfasern, sowie der Umstand, dass sie sich sehr stark verästeln. So verhalten sie sich bei jugendlichen Formen, es scheint aber, dass sich später an den seitlichen Ependymfasern ähnliche atrophische Erscheinungen einstellen, wie bei höheren Vertebraten. Die Stützzellen, die Sala und Retzius darstellen konnten, ebenso wie auch die, die ich an meinen Präparaten sehe, sind auch hier keine eigentlichen Spinnenzellen, sondern nur herausgerückte Ependymzellen. Der Zellkörper erscheint an ihnen sehr plump, sie sind nur mit spärlichen sekundären Ästen, dafür aber mit einem sehr kräftigen peripherischen Fortsatz ausgestattet, der — und darin liegt das Charakteristische für die Stützzellen des Amphibienrückenmarkes — schon innerhalb der grauen Substanz in einen reichlichen Pinsel derber Äste zerfällt, die die

[1]) M. Lawdowsky, Vom Aufbau des Rückenmarkes. Arch. f. mikrosk. Anatomie, Bd. 38, 1891, p. 264.

[2]) Cl. Sala y Pons, Estructura de la Médula espinal de los Batracios. Barcelona 1892.

metamorphosierte Elemente sich noch weiterhin vermehren. Diese Erwägungen führen mich, freilich ohne jeden positiven histologischen Anhaltspunkt, dazu, die Hypothese anzuregen, ob nicht etwa neben den vollausgebildeten Spinnenzellen in den Centralorganen noch agenetische, zeitlebens auf dem ursprünglichen glatten Zustande verharrende Gliazellen, gewissermassen nicht ganz aufgebrauchte Reste aus der ersten Entwickelung, vorhanden sind und ob es nicht vielleicht diese Elemente sind, die durch irgend welche uns bisher noch unbekannte Reize in einen Zustand der Wucherung geraten und so zur Entstehung jener Gliome führen können? Diese Hypothese wird sich freilich ebenso schwer beweisen, wie widerlegen lassen, denn noch besitzen wir keine Methode, die ausnahmslos alle Stützzellen eines Schnittes mit all ihren Fortsätzen so sicher zur Ansicht zu bringen im stande wäre, dass man sich bestimmt darauf verlassen könnte, dass die fortsatzlos erscheinenden Stützzellen eines Schnittes auch in Wirklichkeit fortsatzlos sind.

Die hier aufgestellte Hypothese hat jedenfalls den Vorzug, dass sie sich auf die schönste Weise in Einklang bringen lässt mit jener von den meisten Pathologen vertretenen Lehre, die die erste Ursache aller Gliageschwülste in gewisse Entwickelungsstörungen verlegt, die Anlage dazu in Hemmungserscheinungen der ersten Bildungsvorgänge sucht. Die Hypothese würde demnach folgendermassen lauten: Greift an irgend einer Stelle des Rückenmarkes im Laufe der Entwickelung ein Hemmnis ein, so werden an jener Stelle die Stützzellen, die schon an Ort und Stelle liegen, nicht zu ihrer normalen Ausgestaltung gelangen können, sondern sie bleiben auf einer embryonalen, rudimentären Stufe stehen, womit aber dann auch die Fähigkeit einer späteren krankhaften, ins Unbegrenzte gehenden Vermehrung verbunden ist.

Wir wissen nach den Untersuchungen von Hoffmann (a. a. O.), Oppenheim[1]), Raymond[2]) u. a., dass der Lieblingssitz dieser primären Gliawucherungen die dorsale Partie des Rückenmarkes und vor allem die hintere Mittellinie ist, jenes Gebiet also, das beim jungen Embryo durch die dorsale Abteilung des spaltförmig sich nach hinten erstreckenden, später in jenem Abschnitte zum Verschluss gelangenden Centralkanales in Anspruch genommen wird. Diese Thatsache findet auf dem Boden der dargelegten Hypothese leicht ihre Erklärung, denn da an dieser Stelle infolge der Obliteration des Centralkanales besonders komplizierte Vorgänge bei der Entwickelung der Stützzellen vor sich gehen, so muss hier auch leichter eine Störung des normalen Entwickelungsganges Platz greifen können.

1) H. Oppenheim, Über atypische Formen der Gliosis spinalis. Archiv f. Psychiatrie, Bd. XXV, 1893, p. 1.

2) Raymond, Contribution à l'étude des tumeurs nevrogliques de la moëlle épinière. Archives de Neurologie, Vol. XXVI, 1893, p. 97.

Aber nicht alle Gliome sind auch richtige Astrome, d. h. nicht alle bestehen aus Spinnenzellen. Es giebt zellenreichere, weiche Formen, die sich aus kleinen fortsatzlosen Elementen aufbauen. Man hat derartige Formen oft als Gliosarkome bezeichnet, ein Terminus, gegen den wir von histogenetischem Standpunkte aus entschieden Stellung nehmen müssen. Denn da die gesamte „Glia", wie wir sahen, ein Produkt des Ektoderms ist, unter einem Sarkom aber stets eine bindegewebige Geschwulst verstanden wird, so kann ein „Sarkom" unmöglich zugleich ein Gliom sein. Nehmen dergleichen Geschwülste also von der Glia ihren Ausgang, was auch am wahrscheinlichsten ist, so bleiben sie Gliome, wie ihre Zellen auch beschaffen sein mögen; gehen sie aber hervor, was ja auch möglich ist, aus einem bindegewebigen Mutterboden, sei es die Adventitia der medullaren Blutgefässe, seien es die Hirnhäute, so haben wir es mit reinen Sarkomen zu thun. Es wäre ja allerdings denkbar, dass beides nebeneinander einhergehe, Geschwulstbildung aus der Glia und aus dem Bindegewebe, dass ein gegenseitiges Durchwachsen zweier Gewebsarten vorliege: solchen Mischgeschwülsten könnte allerdings unbedenklich der Namen Gliosarkom beigelegt werden. Indes ist eine solche kombinierte Form bisher histologisch nicht nachgewiesen und die bisherige Anwendung des Wortes Gliosarkom ist auch nicht in diesem Sinne gemeint. Höchst wahrscheinlich gehen auch die sogenannten Gliosarkome aus dem Boden der Glia hervor, nur werden die neugebildeten Elemente nicht zu Astrocyten, sondern bleiben teilweise oder alle, wie ihre Mutterzellen, fortsatzlose Keimzellen.

Es wird nun aber gewiss nicht in allen Fällen leicht sein, die hier geschilderten spontanen Gliawucherungen scharf abzugrenzen gegen jene Formen von Gliavermehrung, die nicht primäre Zustände, sondern Folge- und Begleiterscheinungen von Erkrankungen und Degenerationsvorgängen der nervösen Elemente des Markes darstellen. Es ist merkwürdig, wie ausserordentlich empfindlich und reaktionsfähig nach dieser Seite hin die Spinnenzellen sind[1]). Be-

1) Siehe hierüber den soeben erschienenen Aufsatz Nissl's: Mitteilungen über Karyokinese im centralen Nervensystem. Allg. Zeitschr. f. Psychiatrie, 1894, sowie auch: Über eine neue Untersuchungsmethode der Centralorgane,

steht wo immer im Rückenmarke eine Atrophie, ein degenerativer Ausfall von Nervensubstanz, sei es an Nervenzellen, sei es an Nervenfasern, so wird man progressive Veränderungen an den Stützzellen nie vermissen, wodurch jene regressiven Vorgänge gleichsam kompensiert werden. Diese progressiven Erscheinungen bestehen wohl nur zum geringeren Teile in einer eigentlichen Vermehrung der Gliaelemente [1]) (wobei wieder an die oben erwähnten rudimentär verbliebenen Stützzellen zu denken wäre), die Hauptrolle spielt dabei wohl die Zunahme des Zellkörpers an Volumen und vor allem die Verdickung der Gliafasern, d. h. der Fortsätze der Astrocyten. Sie erscheinen nun derber, kräftiger sei es durch eine Art hydropischer Quellung, sei es durch eine wirkliche Assimilation neuer Stoffe und treten dann an den Schnitten bei mikroskopischer Betrachtung auch bei mangelhaften Färbungen stärker hervor. Ihr Komplex setzt sich als eine verdichtete, dunkle, sklerosierte Partie vom übrigen Querschnitte ab. Ich vermute, dass in vielen Fällen, wo eine primäre Gliawucherung angenommen wurde, nur eine sekundäre, durch den Zerfall von Nervensubstanz veranlasste kompensatorische Spinnenzellenverdickung im Spiele war. Merkwürdig ist dabei, dass sich dieser Vorgang an den Astrocyten bei einer lokalen Erkrankung der Nervenelemente nicht auf die betreffende Stelle beschränkt, sondern im weiteren Umfange diffus auch in das gesunde Nervengewebe hinausgreift; es scheint als ob der die

speziell zur Feststellung der Lokalisation der Nervenzellen. Centralblatt f. Nervenheilkunde und Psychiatrie, Bd. XVII, 1894, p. 337.

[1]) Dass bei pathologischen Prozessen doch auch richtige Teilungen der Stützzellen vorkommen, und dass sie auf karyokinetischem Wege vor sich gehen, hat unlängst Nissl in dem oben citierten Aufsatz in der Hirnrinde von Paralytikern, Dementen und Alkoholisten nachgewiesen. Merkwürdig ist dabei, dass sich die in Betracht kommenden Mitosen mit den gewöhnlichen Methoden schwer nachweisen lassen, dass man zu ihrer Darstellung eines besonderen, von Weigert angegebenen Verfahrens bedarf. Dieses besteht in Folgendem: Härtung in Alkohol, Einbettung in Celloidin oder Aufkleben ohne Einbettung in Celloidin mit Gummi. Die Schnitte kommen vorerst auf eine halbe Stunde in Tinctura ferri acetici Rademacheri, dann nach Abspülung in Wasser in Weigert's Hämatoxylin (Hämatoxylin 1, Alkohol 10, Wasser 90), wo sie ½ Stunde bleiben. Oberflächliche Abspülung, rasche Differenzierung in 1 HCl : 100 Alkohol 70%. Hierauf Wasser, Entwässerung, Aufhellung, Einschluss in Balsam. Vor Nissl haben allerdings schon Fürstner und Knoblauch (Über Faserschwund in der grauen Substanz und über Kernteilungsvorgänge im Rückenmarke unter pathologischen Verhältnissen. Arch. f. Psychiatrie, Bd. XXII, 1891, p. 135) gezeigt, dass nach künstlichen Verletzungen des Rückenmarkes die Gliazellen Erscheinungen mitotischer Vermehrung aufweisen.

Wucherung verursachende Reiz radiär von dem primären Erkrankungsherde ausginge. Am schönsten lassen sich diese progressiven Vorgänge des Stützgewebes an dem oberflächlichen Gliasaume des Rückenmarkes, dem Peridym nachweisen. Fast bei allen chronischen Erkrankungen des Rückenmarkes zeigt dieses Peridym eine mehr oder weniger auffallende Verdickung namentlich im Bereich der Seitenstränge, die soweit gehen kann, dass das Rückenmark wie von einem breiten, geschlossenen, aus verfilzten Astrocyten bestehenden Ring umgeben erscheint. Gewiss mehr als einmal ist diese Peridymverdickung als „Randdegeneration" aufgefasst worden. Die Veranlassung zur Verbreiterung der Gliarinde kann sowohl in degenerativen Vorgängen an den Nervenfasern der Stränge wie auch in atrophischen Zuständen der Nervenzellen der grauen Substanz gegeben sein. Ich hatte unlängst Gelegenheit, eine reichhaltige Sammlung pathologischer Rückenmarkspräparate auf diesen Punkt hin durchzuprüfen und habe die Peridymverdickung nur bei akuten Rückenmarksaffektionen wie Myelitis vermisst. Sehr schön ausgeprägt fand ich sie unter anderem in einem Falle, wo alle Stränge normal erschienen und nur in den Zellen der Vorderhörner sich eine offenbar chronisch entstandene Pigmentatrophie nachweisen liess. Bei allen Strangdegenerationen, bei der multiplen Sklerose, bei gliomatösen Herden scheint das Peridym progressive Veränderungen zu erfahren, so auch bei jener Form der Degeneration der Hinterstränge, die das anatomische Substrat der Tabes darstellt und ich glaube, dass die „peripherische Sklerose", die öfters als Kombinationserscheinung bei Tabes beschrieben und abgebildet wurde, (s. z. B. die in der Pathologischen Anatomie E. Ziegler's, 6. Auflage, II. Bd., Fig. 156, p. 289 reproduzierte Figur Westphal's), nichts anderes darstellt als eine durch die Erkrankung der Hinterstränge veranlasste kompensatorische Peridymzunahme.
