# Supplementary material for: The Concept of Neuroglia ‐ the State of the Art Circa 1900
Source: Glia. 2025 Feb 4;73(5):890–904. doi: 10.1002/glia.24678 (PMC11920685; doi:10.1002/glia.24678)
Supplement: Supplementary file 6 — Data S6. Translated text by Lenhossek. [file GLIA-73-890-s003.pdf]

THE FINE STRUCTURE OF THE NERVOUS SYSTEM

IN THE LIGHT OF RECENT RESEARCH

A GENERAL CONSIDERATION OF THE STRUCTURAL PRINCIPLES OF THE NERVOUS SYSTEM ALONG WITH A REPRESENTATION OF THE FINE STRUCTURE OF SPINAL CORD

from

MICHAEL v. LENHOSSÉK

IN WÜRZBURG

THE SECOND COMPLETELY REVISED EDITION

WITH 6 PLATES AND 60 FIGURES IN THE TEXT

BERLIN NW.6

FISCHER'S MEDICINE BOOKSHOP H. KORNFELD

1895

176

Chapter VI

The support cells of the spinal cord

The Golgi method has significantly advanced the recognition of support cells in the spinal cord, including their origin and development, in a manner similar to its contributions to the understanding of nerve cells. While the silver method yielded novel and interesting findings, it was limited to results obtained from immature specimens of the nervous system. In the first edition of my book, I noted that the spinal cord was best studied, though with the limitation that these studies were restricted to embryos, immature humans, and immature higher vertebrates. It should always be considered that these relationships change in mature specimens.

In the meantime, excellent illustrations from the central support system were provided in the textbook by von Kölliker[1].

[1] A. v. Kölliker, Handbuch der Gewebelehre des Menschen. 6. Auflage, Bd. II, Leipzig 1893, p. 136). This refers also to Golgi´s images from the developing cord.

177

Kölliker's illustrations have provided important insights into the features of *Neuroglia*, shifting the focus from the previously considered histogenetic view to the adult system. By doing so, he integrated the developmental perspective into a mature support system, resulting in a more coherent understanding. I have also conducted my own studies with satisfactory results. These studies were not based on the spinal cords of adult humans, as such efforts were unlikely to be successful. Instead, they focused on specimens from newborn or one-year-old children. It can be assumed that, at this developmental stage, when the spinal cord exhibits myelination of the white matter, the tissue can be considered fairly mature and unlikely to undergo significant changes.

My experiments revealed that the Golgi method is superior to all previous ones for identifying the fundamental composition of Neuroglia also in the adult stage. This is limited to recognizing the shape of the cells, but does not reveal

the internal structure. The images of the spider cells as revealed with the chrome/silver method are simply beautiful as also mentioned by Kölliker (last reference, p147). The staining can be easily obtained if one incubated a 2- 3 mm long piece of spinal cord from a child for three days into the Golgi solution, then for 2 days into the silver solution and one will obtain a successful staining. It always worked in my hands and there were always support cells labelled. These cells are more obviously visible since the nerve fibers due to their myelin sheath and nerve cells are not labelled as rapidly and were not as numerously visible. They also lacked the labelling of the axon. This makes the Glia well visible and is not obstructed by other things.

178

The images I present in Table I (see at the end of the text) depict, with high precision and in large numbers, the elements of this medullar supportive structure—the glial cells (Deiters cells, Golgi's cells)—as known from pre-Golgi publications, but especially from the first precise descriptions by Deiters and Golgi (1871), and later by Boll, Kölliker, Jastrowitz, Gierke, and others. Our new observations support all previous findings. These observations describe highly ramified, star-shaped, delicate structures widely distributed in the grey and white matter**. These cells form the support structure of the spinal cord.** While not the main structure, they are the only elements described as "Neuroglia." "Neuroglia" is not a separate tissue but consists of highly ramified, star-shaped cells embedded in the brain and spinal cord tissue. They intermingle with nerve cells in the grey matter and with nerve fibers in the white matter, thereby forming a support system. Moreover, there are no separate glial fibers; instead, there are only extensions from glial cells, similar to how dendrites are extensions of nerve cells. This foundational assumption, first proposed by Kölliker in 1862 [1)] and further supported by Golgi's work in 1871 [2)], forms the basis of what is considered the support system of the central nervous system. This concept may not be universally accepted, as observations from pathological conditions appear to contradict it.

[1)] v. Kölliker, Handbuch der Gewebelehre des Menschen. 4. Auflage, 1862, p. 304),

[2)] C. Golgi, Contribuzione alla fina Anatomia degli organi centrali del sistemia nervosa, Rivista clinica die Bologna, 1871-1872, p 25ff.

It is still assumed that this supportive tissue is an independent element, a basic coherent substance in which nerve cells and fibers are embedded, serving as a putty for them. It is considered an unstructured putty-like substance, the "Glia," which serves as a foreign embedding material—a distinct medium for embedding the nervous elements. This putty-like embedding material is thought to play the primary role and contains secondary elements, including specialized cells called "glial cells" and fiber elements referred to as "glial fibers." Over the last decades, this concept has been established, since the methods of that time could not illustrate the tissue elements as they really are and therefore has led to this concept. What do show us unstained spinal cord slices or the labeling with the ordinary stains? We see the unstained or partially stained nervous cells, or even only their fragments while the processes remain invisible embedded into a homogeneous or granular substrate, such as cartilage cells in the intercellular substance; in white matter regions, the nerve bundles look like being caried by a structure with embedded nuclei. Based on these observations based on these insufficient methods the concept of a putty based basic substance emerged and this is reflected in the term **Neuroglia**, nerve putty. I am afraid that the view will be stuck as an imagination of a cement rather than a special type of cells within this central tissue if we continue to use that term. The vision that we are not considering a special tissue, but a special class of cells requires a different definition,

180

I therefore propose to name all supportive cells of the nervous system in general as **supportive cells or intermediate cells**, Spongiocytes [1)], and the common form in higher vertebrates as **spider cells or "astrocytes"** and use the term neuroglia only cum grano salis[1#] as long as the concept is not finally approved. Since this book is for a broader audience, I will use the new terminology, a bit inconsistently, together with the old one.

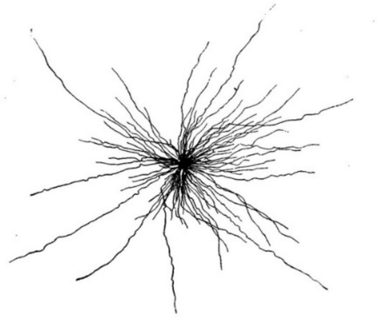

Figure 20. *Spider cell from the spinal cord of a three-month-old child.*

Astrocytes, star-shaped cells, are, indeed, the small elements of the supportive system of the spinal cord (Fig. 20), no other comparison described their feature as accurate than this.

1) After I wrote down this Terminus technicus, I kindly received a note from the author (P. A. Fish, The Terminology of the Nerve cell Journal of Comparative Neurology, Vol. IV, 1894, p. 171) in which the same name was proposed for glial cell. A strange coincidence is that Fish named the nerve cell, like me Neurocyte. This term has, however, been previously coined. The nervous process Fish termed neurite.

The term ´spider cells ´introduced by Jastrowitz [1)] has become fashionable, and describes their features. However, Gierkes argument should be considered that nobody has seen spiders with so many legs as these cells have processes. I must confess that this name for the astrocytes is, among the German terms, the better one. ´Brush cells´ [#2] as Boll named them would only fit for those cells which have processes originating only at one side of the cell, but for most of them it is not appropriate. In humans, astrocytes are more numerous and smaller relative to the diameter of the spinal cord compared to other mammals or vertebrates. They are generally small elements with a thin rim of protoplasm surrounding the nucleus. Labeling serial sections of the spinal cord with hematoxylin, nigrosine, or methylene blue results in strong nuclear staining with all these dyes. These nuclei, if not derived from nerve cells or leukocytes, belong to astrocytes.

These small cell bodies, and especially their radial ramifications, are difficult to label with dyes. The best images are obtained using carmine or picrocarmine stains. Much clearer images are produced by chrome/silver staining. A dense array of processes originates from the barely visible cell body. These processes are strangely stiff and this is characteristic for glial cells. This distinguishes them from neurons and this difference is obvious for a trained eye. The nerve cell dendrites are unique with their tree-like shape which seems to be more individual, more freely while the glial cells

[1)] Jastrowitz, Studien über die Encephalitis und Myelitis des ersten Kindesalters. Archiv f. Psychiatrie, Bd. III, 1871, p. 162

are more patterned, stiff. They are not as heterogeneous. These different features mirror the functional difference between these two cell types: the nerve cells represent the functional elements for the multiple nervous features embedded in their protoplasm, while the astrocytes represent elements without internal movement fulfilling supportive function or the formation as isolators as suggested by **Pedro Ramon** [1]**, R. y Cajal** [2] **and CL. Sala** [3]. They are kind of foreign objects due to their silent presence and their physical properties in the dynamic and active nervous tissue. At least in human, they are distinct by their tiny cell body and their processes as compared to the neurocytes [#3]. In the spinal cord, one can distinguish two forms of astrocytes according to **v. Kölliker** (textbook page 145), **Langstrahler** and **Kurzstrahler**. They are not equally abundant. Th*e* Kurzstrahler *are a minority and are found only in the grey matter. It is therefore reasonable to consider the* Langstrahler as the predominant spider cells.

The processes are seldom evenly distributed around the soma. Ramifications often appear as dense bundles, with visible projections within these bundles. The soma is indented between the origins of the processes, particularly the larger ones. The processes are commonly thin, similar to dendrites, and notably, their diameter remains consistent from beginning to end. Some processes, however, are remarkably large.

1) P. Ramon, El encefalo de los reptiles. Barcelona, 1891, p. 10
2) S. R. y Cajall, Significacion fisiologica de las expansions protoplasmaticas y nerviosas de la celulas de la sustancia gris. Rivista de ciencias medicas de Barcelona, 1891, Nr. 22 and 23.
3) Cl. Salay Pons, La neuroglia de los vertebrados. Barcelona 1894, p. 39.

The processes labelled with the Golgi stains appear slightly thicker which could be due to the wrapping with the chrome/silver mass. There are no varicosities and the processes are similar to nerve fibers and can be easily mixed up. In reality, they are characterized by a more linear course. The small spike-like bends, which are found in Golgi stains could be shrinkage artefacts, since they are more often observed in some preparations while they are absent in others. In general, the processes of the Langstrahler are unbranched in contrast to the dendrites. It is frequently observed that they branch in s small angle, mainly close to the soma, but it usually happens only once. Deiters described the processes of glial cells as highly branched, while Golgi (1871) described them as unbranched. The truth is probably between the two extremes, but closer to Golgi´s description.

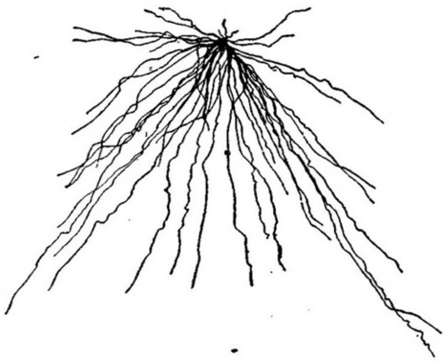

Figure 21*. Spider cell from the spinal cord of a 9-month-old child, with unipolar development of processes*

The processes of the astrocytes vary in length; some penetrate quite in isolation large areas of tissue, others terminate close to their origin. They are most dense close to the soma. They are often so dense that they cover the soma. While it was mentioned above that the processes of astrocytes extend **in all directions**, so is this only true for the majority of cells. In all regions of the cross section one can find astrocytes with processes extending into **one direction** (Fig. 21)

or extend like two brushes on the opposing poles of the cells. This is an individual difference and can occur anywhere, but is also a local peculiarity. As shown below, this is often found at the rim of the grey matter.

All processes of the astrocytes, the long and short ones, **end in free tips**. This has been first recognized by **Deiters**, Boll and Golgi have described them more precisely. Ranvier and Gierke made a step backward, since they assumed

that the processes of neighboring cells are fused. This is a clear error as shown by the Golgi images. The astrocytes are completely independent elements and are like neurons, units by themselves. The meshwork formed by their processes and which forms quite a substantial volume in grey matter is rather like the neuropilema, a **felt, an astropilema or spongopilema** and not a network.

The Golgi method is optimal for the characterization of individual glial elements, it does, however, not provide a **quantitative** characterization of their distribution in the spinal cord section, since it selectively labels only a few of the most abundant cell types. It can be observed that in regions with a higher density of glial cells, more are being labelled. There seems to be a correlation between the density of the cells and the number of labelled cells. There, however, remains uncertainty and the Golgi method seems not optimal.

Here other methods come into play. Two procedures have been developed which yield excellent results.

185

One was developed by Weigert; however, it has not been published in sufficient detail to be reproducible[1]. The other method is closely related and produces similar but less clear images. It was introduced by Beneke[2] and is a slight modification of Weigert's fibrin staining method. The original Weigert fibrin method is as follows[3]: Tissue hardened in alcohol is sliced. The slices are stained for 5–15 minutes in a concentrated aniline-water-Gentiana-violet solution. The slices are rinsed in a 0.6% NaCl solution and dried on a glass slide using filter paper. Repeatedly apply drops of iodide-potassium solution (1:2:100) to the object, followed by drying with filter paper. This is followed by destaining with aniline-oil-xylol (2:1) and the removal of aniline-xylol using pure xylol. The slices are stored in Canada balsam. Using this original recipe, everything except fibrin and any organisms present will be destained. Beneke modified the method by altering the mixture of the destaining substances, using more xylol (xylol 3: aniline-oil 2). This adjustment reduces the destaining power, resulting in residual staining of connective tissue, elastic fibers, muscle components, epithelia (Kromayer), and glial cells. Thanks to the courtesy of Prof. Weigert, I received several specimens of human spinal cord stained with his method. The methods indeed results in instructive delicate images and it is in the interest

1) Weigert reports in his glia article (Anat. Anz. 1890, p 550) only that much, namely that the preparations are stained with metal salts containing an organic acid; with respect to the metal as well as the acid, several yield results. Stained are the slices with methyl-violett, they were treated with iodide and washed with aniline-xylol

2) Beneke, Über eine Modifikation des Weigert´schen Fibrinverfahrens. Anat. Anz. Jahrg. VIII, 1893, Suppl. p165.

3) see Friedländer-Eberth. Mikroskopische Technik. 5. Auflage, Berlin 1894

186

for pathologic studies that it will be published in detail by its inventor. Although the method was not yet perfect, which Weigert actually considered as *conditio sine qua non* [4#] for its final publication. The glial fibers are labeled dark blue, and their dense, fine meshwork occupies the slice. At low magnification, or even with the naked eye, areas with accumulations of glial elements appear as dark spots. The nerve cells, including their processes, as well as the cell bodies of astrocytes, remain unstained. The soft protoplasm and the substance of the axons are devoid of stain.

While the method is very selective in the spinal cord, this is not true outside it. Outside the spinal cord, it also labels connective and elastic fibers. This was evident in the samples provided to me by Weigert, which included the outer spinal cord layers. Considering that different elements with similar forms and physical properties are stained, it becomes clear that this labeling depends on specific density and structure. Only dense, continuous fibers retain the stain. Also, Beneke confirms that his modified Weigert method labels all fibrillary structures, such as fibrillary connective tissue, elastic tissue, bone fibrils, Sharpey ´s fibers[#5] and also glia fibers, while the cellular protoplasm is not stained. It is therefore not a glial labelling. In this context, the staining should be considered as uncomplete. It is not useful to label cells. It labels only fibrous structures which fulfill defined physical requirements and actually as isolated continuous structures, independent whether they are connected by cells or not. **It does not serve to completely stain glial cells, it only stains part of it, the fibrous components, the glial fibers.** This is a dangerous

187

property, as it can easily lead to erroneous concepts, such as the idea that glial fibers represent an entity similar to connective tissue fibers. This could imply that glial cells are merely secondary structures embedded within the fiber network. Such a conclusion was recently drawn, partly based on a report by Ranvier. Another concern is that these images may promote the view that glial tissue is merely connective tissue. It must be understood that these images should be replaced by representations of the unstained cells that give rise to the stained processes. Visualizing the cells requires alternative techniques, such as the Golgi method.

Other methods, such as carmine staining and the Golgi method, have demonstrated ramified cells with fibers extending from a central cell body, as described in 1863 by Kölliker and later confirmed and expanded upon by Deiters and Boll. Therefore, Weigert's method is not suitable for dismissing the concept of the uniformity of the glial cell body and its processes.

Weigert argues based on Ranviers opinion that the glial fibers do not radially originate from an unlabeled or weakly labelled cell and that they even run over cell bodies. One can argue against that that the fibers are much too thin to clearly see that and that the processes of astrocytes are so dense that processes from different cells overlap.
The history of the Neuroglia dates back to the year 1811. Keuffel´s [1)] report appeared in that year and he treated the spinal cord

[1)] G. G. Th. Keuffel, Über das Rückenmark. Reil´s Archiv, Bd. 10, 1811, p. 123

188

with Kali causticum and removed the weakened cord substance by a kind of brushing. This allowed him to visualize the glial bars which penetrate the white matter in a form of a mesh (network). Keuffel named the substance of that network which extended into the grey matter and which he considered as an extension of the pia mater ´condensed cell material´ or ´neurilem of the spinal cord´.
The first histological report is by Arnold in 1844 [1)]. Arnold recognized a granulated basis material in which nerve cells are embedded.
Virchow [2)] studied that substance in more detail in 1853 and assumed a wide-spread distribution in the central nervous system. Similar as Arnold, he viewed it as a soft, amorph, granular intermediate substance; but he described embedded round, lense-shaped cells[#6]. This is obviously the first, but yet uncomplete report [3)] on these cells and one may consider that Virchow was the first who recognized these cells. According to Virchow this substance, later named by him as ´Neuroglia´ represents a tissue belonging to the ´connective substance at large´. R. Wagner [4)] did not exclude that these are nervous elements and speculated that it may serve to generate neurons, a hypothesis which was later strongly supported by Henle and Merkel [5)].

With Bidder (1857) [6] the issue becomes more complex. He considers it not as a formless mass which is transparent under normal conditions, and which becomes granular after treatment with chrome acid. He rather assumes that it contains longitudinal and perpendicular fibers, connective tissue bodies without or with 2 to 3 processes which are fused. The connection of the white matter he considers as a continuum of the pia mater. Bidder considered a much too high contribution of the connective substance to the formation of the spinal cord. His advance was the recognition of the fibers and star-shaped cells with processes. As a note, it should be added that Kölliker described such cells already in 1855 in grey matter.

[1] Fr. Arnold, Handbuch der Anatomie. Bd, 1, Freiburg i. Br. 1844, p. 260
[2] R. Virchow, Über eine im Gehirn und Rückenmark gefundene Substanz mit der chemischen Reaktion der Cellulose. Archiv f. pathol. Anat. u. Physiol. Bd. VI, 1853, p. 136.
[3] R. Virchow, Gesammelte Abhandlungen. Frankfurt, 1856, p. 890.
[4] R. Wagner. Neurologische Bemerkungen. Göttinger Nachr. 1854, Nr. 3, p. 28.
[5] J. Henle and Fr. Merkel, Über die sog. Bindesubstanz der Centralorgane des Nervensystems. Zeitschr. F. ration. Medizin. Bd. 34, 1868, p. 48.
[6] F. Bidder and C. Kupffer, Untersuchungen über die Textur des Rückenmarks, Leipzig, 1857.

We will jump over some less important issues to come to an important moment in the history of ´Neuroglia´. It was a major advance when Kölliker [1] recognized in 1863 that the connective tissue of the spinal cord is composed by nothing else as of a complex of star-shaped, intermingled cells, which are connected among each other to form a network for the nervous elements. Kölliker, as Bidder, considered that their processes are inter-connected and form a true net.

Deiters [2] provided the first correct image of the elements of the connective substance, the astrocytes. While Kölliker had depicted isolated cells already in 1855, he did not recognize the extensive processes. But also, the Deiters images are not yet perfect, the processes are less numerous, they are too curved, too much arborized and delicate and do not reflect the typical Langstrahler astrocyte rather a Kurzstrahler. The description in the text is also ambiguous. Deiters refers on page 38 to the "porous, granular basic material." He claims the presence of free nuclei and suggests the

existence of independent fibers originating from cells but becoming isolated. Within this spongy tissue are cellular elements described as ramified connective tissue bodies, which are not fused. Finally, Deiters reports connections from the pia mater intruding into the white and even grey matter, an assumption that we can no longer support.

Only seven years after Deiters's posthumous publication, an image of a support cell in the spinal cord was published that resembles the image we recognize today. The year 1871, when Golgi's report on the fine anatomy of the nervous system[3] appeared, marked a milestone. Notably, Golgi's image was created before the invention of the chrom/silver stain. It was primarily based on teased tissue and carmine staining.

1) A. Kölliker, Handbuch der Gewebelehre des Menschen. 4. Auflage, 1863, p. 304-306), 5. Aufl. 1867, p. 266
2) O. Deiters, Untersuchungen über Gehirn und Rückenmark, 1865, p. 44.
3) C. Golgi, Contribuzione alla fina anatomia degli organi centrali del sistema nervoso, Rivista clinica di Bologna, 1871, p. 25ff.

Golgi's study is not as well-known as it deserves to be. I must confess that I only became aware of it recently, after it appeared in a German translation. Golgi accurately describes the “interstitial stroma” as being exclusively composed of ramified cells in the cortex, cerebellum, and spinal cord. The description and illustration of these cells are so remarkable that astrocytes could be referred to as Golgi cells. Von Kölliker actually used that term.

Since this renowned Italian scientist already has many cells named after him, I personally would not name it after him to avoid confusion. Golgi describes these elements as small cells from which multiple stable, fine fibers originate. These processes are neither arborized nor do they fuse (anastomose). In the interstitial cells of the white matter, the abundance of these processes suggests that their bundling explains the septa of the white matter, without requiring reference to the insertions of the pia mater.

In the grey matter, Golgi describes the processes as more delicate compared to those in the white matter, and even more so when embedded between groups of neurons. Perhaps the description of the Kurzstrahler in the following text can already be referenced.A term used by Golgi should, however, be replaced. Golgi names the spider cells always as connective tissue cells and their entire complex, the “glia”, as connective tissue. He realized, however, that it is distinct from ordinary connective tissue. Today we know that these cells are not mesodermal, but ectodermal elements, even if they appear from their morphology as star-shaped connective cells. It can be considered as a “convergence appearance”.

The highly complex work by Gierke [1)] on this subject over the last 20 years has not advanced the field beyond Golgi´s images, it can even be considered as a step back. This is particularly evident when looking at the images of Gierkes work. Such supportive cells which are depicted in Fig. 1a and 2 do not exist in the spinal cord. In these Figures, the processes of the spider cells are arborizing in wave form, become smaller with distance and become finally, as seen in Fig. 1a almost dissolved in a netlike mesh. Also, the Kurzstrahler do not look like pictured. The figures correspond to a similar description in the text. All supportive cells are assumed to be interconnected by anastosomen, an assumption, which

[1)] H. Gierke, Die Stützzellen des Centralnervensystems. Archiv f. mikrosk. Anat. Bd. 25, 1885, p. 441 and Bd. 26, 1886, p 129.

was already refuted after Deiters, Golgi´s and Boll´s [1)] reports. Glial cells with three, and even more nuclei were frequently reported (?)[#7]. Gierke´s claim that all supportive cells, the soma as well as the processes and even the nuclei in adult humans and animals should be keratinized has not been substantiated by chemical or staining experiments. The greatest error on Gierkes assumption on the composition of the supportive substance is the following: he assumes that the major mass of ´Glia` is an amorph, wide spread base substance, which develops from a gradual transformation of cell bodies.

Nevertheless, Gierkes work contains also some very relevant details. His main merit is his statement that the supportive element of the central nervous system is of ectodermal origin with no contribution of connective tissue.

In 1890 Weigert gave a talk at the X. International Medical Congress in Berlin which was followed by a short report in the Anatomischer Anzeiger [2)] with the following contribution: based on his staining method he reliably describes the quantitative distribution of the glial fibers, i.e. the processes of spider cells, in the different regions of the spinal cord cross section. This is an important contribution on the topographic distribution, while we do not support his and Ranvier´s concept [3)] on the relation between glial fibers and cells.

With Gierke and Weigert ends the pre-Golgi period on the study of the cerebro-spinal support system. The research with the Golgi-technique leads to novel findings which can be summarized in two points:

1. The clear images of Golgi-stained adult astrocytes finally confirm previous reports by Golgi from 1871 and by Boll.
2. The histogenesis of glial cells as of ectodermal origin is now confirmed and all details are uncovered. Here we will interrupt our historic introduction for a moment and continue later.

[1)] F. Boll, Die Histologie und Histogenese der nervösen Centralorgane. Archiv f. Psychiatrie u. Nervenkrankh. Bd. IV, 1874, p. 1.
[2)] C. Weigert, Bemerkungen über das Neurogliagerüst des menschlichen Centralnervensystems. Anat. Anz. Jahrg. V, 1890, p. 543.
[3)] L. Ranvier, De la névroglie. Comptes rendus de l´Acad. des Sc. Tome 94, 1882, p.1536. In addition: Archives de physiologie normale et pathologique, 1883, p.177 – Technisches Lehrbuch der Histologie. Translated by Nicati and v. Wyss. Leipzig, 1888, p. 972.

192

Astrocytes differ depending on the **region** and show **different local features** within the cross-sections with respect to size, ramification, density and distribution.

We will now not report on ependymal cells, which belong to the supportive system, but report about them later when describing the development of the supportive system.

The highest accumulation of spider cells, one finds close to the **middle commissural area** of the grey matter, in the area of the substantia gelatinosa centralis and further lateral of it. This coincides with the fact that nervous elements, both cells as well as processes are missing; this area which serves as an origin of nerve cells of the cord during development, is completely missing nerve elements in the mature state. This gap is filled with support elements. The Weigert myelin sheath stain shows this region as well-stained in yellow but not penetrated by a fiber. Nuclear stains with Thionin or Magenta Red show that the central canal, if not cut too thin, is surrounded by densely packed nuclei that extend less densely toward the entry zone of the commissure into the columns. These numerous nuclei correspond to those of the abundantly accumulated spider cells.

Not only is the number of astrocytes remarkable, but their size is as well. In this region, the cell body is relatively normal, only slightly larger than in other regions, but its multiple processes are long, thick, stiff, and always unbranched. The most remarkable feature is the arrangement of the processes. The medial processes are concentric around the central canal and intermingle to form a dense meshwork. This circular felt, characterized by its lack of nerve elements, is distinctive for that region. This region is labeled in dark blue by the Weigert glia stain, which is visible even at lower magnifications.

193

This concentric fibers in the region of the central canal have been recognized even earlier. Stilling [1)] reported in 1842 a layer of circular fibers forming a circular commissure.

To the formation of the circular felt not only contribute cells close under the ependyma, but also those, which are a bit apart. Those send tight bundles of processes to the central canal. These fibers diverge as soon as they reach the ependymal surface, diverge towards two sides, and turn into the concentric direction.

Observing the fiber ring in fully impregnated preparations, it is evident that it is neither tightly adjacent to the ependyma nor slightly below the basal terminals of the ependymal cells. Just below is a fiber-rich band, but not yet as dense as the lighter layer, which is followed by the dense fiber rim. Toward the lateral parts, the density of the fibers decreases. It should be noted that concentric orientation is not the only arrangement in the peri-ependymal fiber felt. The astrocytes are also, as elsewhere, ramified in all directions. Most project both vertically and horizontally within the plane of the spinal cord, which can be easily observed in longitudinal sections. The peri-ependymal tissue also exhibits different fiber directions. Longitudinal fibers and others are present, but circular fibers are the most abundant. Based on the structures described above, it is, in my opinion, time to replace the term *Substantia gelatinosa centralis*, as coined by Stilling, with a more appropriate one, as this region is not gelatinous.

The supportive cells, which are located between the central canal and the frontal fissure, often send dense bundles across the frontal commissure and thus form in front of the central canal a ´spider cell commissure´; also, behind the central canal one finds, less pronounced, such similar structure.

[1)] Stilling and Wallach, Untersuchungen über die Textur des Rückenmarks. Leipzig 1842, p. 23.

194

The support structure in the frontal commissure is more complex. There are sagittally oriented, bipolar astrocytes, and their processes are confined to a frontal and a rear bundle. The frontal bundle projects to the frontal fissure, while the rear enters the circular fibers. The spider cells located near the dorsal roots, in the area known as the dorsal grey commissure, project their processes to the tip of the dorsal roots. The medial processes cross in the septum posterius. This septum, as we will see later, consists of a bundle of ependymal cells. It is reinforced by spider cells dispersed throughout its entire length. Additionally, bundles of spider cell processes enter the septum, located behind the central canal on both sides of its frontal part.

In the grey matter, respective in the horns, there are two types of spider cells: the typical astrocytes as described above and a second form described by Kölliker. He describes them as Kurzstrahler in contrast to the typical Langstrahler and thereby well describes their specific feature. They are characterized by short processes; they are related to the Langstrahler like dwarfs to normal people. But the processes are not only short, but also very feathery; they appear

less in a radial form, but rather like a surrounding dense lawn or a bush surrounding the cells. They contain varicosities, which gives the cell with its branches an image of a granular meshwork, a feature often exaggerated by imperfect staining. As also Kölliker noted, these cells are less dark stained when compared to Langstrahler. They have another peculiar feature, which is distinct from astrocytes, namely that the processes are ramified and become thinner towards their ends. This can be easily distinguished, even at low magnification. They are often not stained dark black as other astrocytes, but rather light brown.

195

Such labelled Kurzstrahler yield the best images, since their delicate processes are well visible, free of granular depositions.

I have not described this special form in the general introduction to astrocytes to avoid creating the expectation that they are an abundantly distributed cell type. I believe that the Kurzstrahler in the spinal cord are a local subtype present only in the horns, but they are not the only cells there. At least as many Langstrahler are present. In the grey matter, they are not restricted to a specific region and can occur in the ventral and dorsal horns, as well as in the region between them. However, they seem to be more abundant in the ventral horn, particularly between the motor neurons. They are not present in the middle commissure, so the sagittal line connecting the dorsal and ventral horns forms a boundary. Similarly, they are completely absent in the white matter. The Langstrahler in the grey matter occur in several variants: some have a more rounded or elongated cell body, with processes extending in all directions or oriented in a single direction, while spindle-shaped cells have processes extending from the two poles of the cell body. At the border between grey and white matter, many cells project from the side directed toward the grey matter in a fan-like fashion. These cells appear typical at these borders and are found throughout the dorsal horn and in the ventral horn, particularly at the medial border, from the grey commissure to the Rolando substance. Cone-shaped protrusions extend from the grey into the white matter, with the cell body located at the tip of such a spike, while the cone contains the fan-like branching. However, these cells are also found between the cones.

These cells are shown in Table I (see at the end of the text), and Fig. 21, page 183 which illustrates that they are highly ramified. This gives the impression that the arrangement of the spider cells and their processes at the border of the horns is much denser. This is also shown in the Weigert´s glia stain showing a dense labelling at the border of the ventral horn and at the medial border of the dorsal horn. One can get the impression that the grey matter is circumvented (or shielded) by a wall of felted astrocytes against white matter.

But also another type of spider cell is found at the border between dorsal horn and root: these are bipolar astrocytes which extend processes tangentially to the border line. All these qualitative and quantitative differences are explained, according to my opinion, by the interaction of nerve elements and support cells, in the sense of a compensatory relationship. The most difficult problem in the histology of the spinal cord is the composition of the so-called Substantia gelatinosa Rolandi. This is an old dispute in the anatomy of the spinal cord. A nervous ganglion or an accumulation of neuroglia, these are the alternatives which are disputed by researchers. Meynert [1] and Krause [2] considered them as nervous describing their upper part as the ´the lower sensory trigeminal nucleus´. Bechterew [3] in contrast, viewed it as an accumulation of central support substance without nervous elements, a concept which I also supported in the pre-Golgi period [4]. Without a definite statement about the general

[1] Th. Meynerth, Vom Gehirn der Säugetiere. Stricker´s Handbuch der Lehre von den Geweben. Wien 1870, Bd. II, p. 777.
[2] W. Krause, Handbuch der menschlichen Anatomie, Bd. I, Allgemeine Anatomie, Hannover 1876, p. 389 and 420.
[3] W. Bechterew, Über einen besonderen Bestandteil der Seitenstränge des Rückenmarks. Arch. f. Anat. und Physiol. Anat. Abt., 1886, p. 4.
[4] M. v. Lenhossek, Untersuchungen über die Entwicklung der Markscheiden und den Faserverlauf im Rückenmark der Maus. Arch f. mikrosk. Anat., Bd. 33, 1889, p. 78.

character of the Rolando substance, several researchers have noted its nervous component such as Stilling, Clarke, Kölliker, Schwalbe, Gierke and in particular H. Virchow [1]. This view was supported by the application of the Golgi stain. Golgi, Ramón y Cajal, von Kölliker, Van Gehuchten, and Lenhossek demonstrated through silver/chrome staining that nerve cells are embedded in this substance; I will return to this in more detail later. However, this finding does not determine the nature of the substance; it is still possible that, in addition to nerve cells, it also contains glial elements or other components. The most important finding regarding the nature of the Rolando substance comes from the previously cited work by Weigert (a.a.O., p. 548).

Based on his glial stain he demonstrated that the Rolando substance is the region in the spinal cord with the lowest density of glial fibers. It is therefore clearly distinct from the “Substantia gelatinosa centralis” which consists throughout of a felt-like mass of glial fibers. Weigert did not speculate on the nature of the Rolando substance; he considered that it is formed by a “physiologically undefined alternative substance” containing some nerve cells and only few glial fibers. The images which I obtained from the Rolando substance of the spinal cord of children up to one year old using the Golgi method support this view. There were only few astrocytes. Distinct from the central substance, which contains a high density of long projecting spider cells, there is not much labelling of ramified elements. I therefore support Weigert´s view that the Rolando formation does not represent a glial accumulation.

[1] H. Virchow, Über Zellen in der Substantia gelatinosa Rolando. Reported in Neurol. Centralbl., 1887, p. 263.

198

In the future I will have the opportunity to come back to that formation and maybe I will succeed to answer the question of its composition, maybe not completely but getting a step closer.

In any case, the Rolando substance is not completely void of normal glia since spider cells are stained occasionally but less frequent than in other areas of grey matter. These elements belong either to the category of Langstrahler or the typical Kurzstrahler. The Langstrahler found here are distinct from similar cells found elsewhere. Their processes are thicker and less frequent, projecting unevenly in all directions but primarily oriented in the sagittal direction. This frontal, sagittally oriented bundle of processes consists of parallel, very long fibers, while the lateral and rear processes are generally much smaller. Between the rear perimeter of the Rolando substance and the edge of the spinal cord is a small light area containing few fibers, which serves like a bridge between the ventral and dorsal root. It is called the **´border zone of Lissauer´** [#8]. It comprises the entry zone of the dorsal root and the area on both sides. Its form and width vary among the different areas of the spinal cord. This zone which actually belongs to the white matter is characterized by a strong **accumulation of astrocytes**. They are densely packed and characterized by numerous and very fine ramifications. From here extends the glia, or in other words the complex of ectodermal spider cells, cone-shaped or in the form of several bundles, into the connective tissue-like perineurium of the dorsal root. This has been described by Staderini [1] for the nerve roots and recently in a comprehensive manner by

[1] Staderini, Contributo allo studio del tessuto interstiziale di alcuni nervi craniensi dell' uomo. Monitore zoolog. italiano, Anno I, 1890, p. 232.

Weigert (loc. cit., p. 547), Hocher [1] Edinger [2], and J. Schaffer [3] for the sensory roots of the spinal cord nerves. Hocher showed a similar relationship also at the ventral roots.

Also, the small crescent-shaped rim which belongs to grey matter and which embraces the Rolando substance from its back side and is termed the ´**marginal zone of the Rolando substance**´, contains numerous spider cells which are tangentially orientated.

The astrocytes of the **white matter** belong all into the category of **typical Langstrahler**. It is generally assumed that they are more robust, larger and contain thicker processes than those in grey matter. This is actually not my experience; I find almost the opposite. Most astrocytes in the nerve bundles of my preparations are smaller and more delicately ramified than the Langstrahler in the grey matter. Especially those in the central area where they have shorter and more simple processes. What was most obvious to me is that they are not much different than the astrocytes in grey matter. This is surprising because they serve another purpose as in grey matter. In grey matter the nerve cells and the support cells are intermingled. The complex irregular processes of the nerve cells and their spiky cell body are enwrapped by astrocyte processes like a basket. In white matter they have a very different behavior. Here the astrocytes and their processes form a kind of regular meshwork circumventing the longitudinal fibers, which contains so called Gliasepta. One would expect that this significantly different behavior also results in different structures of these glial elements, namely that the processes are stiffer or have other peculiarities.

[1] A. Hoche, Beitrag zur Kenntnis des anatomischen Verhaltens der menschlichen Rückenmarkswurzeln etc. Habilitationsschrift, Heidelberg 1891.
[2] L. Edinger, Vorlesungen über den Bau der nervösen Centralorgane, 4. Aufl., 1893, p. 16.
[3] J. Schaffer, Die oberflächliche Gliahülle und das Stützgerüst des weißen Rückenmarkmantels. Anat. Anz., Bd. IX, 1894, p. 262. – Derselbe: Beiträge zur Kenntnis des Stützgerüstes im menschlichen Rückenmarke. Arch. f. mikr. Anat., Bd. 40, 1894, p. 54 ff.

This is not the case. There are no obvious differences between astrocytes in white matter and the Langstrahler in grey matter. The astrocytes which are in the middle of the nerve bundles extend their processes uniformly into all directions. However, the more we are focusing on the periphery of the spinal cord, the more we find spider cells with a characteristic property. The ramification towards the periphery becomes more numerous and stronger while the opposite is the case

for the orientation towards the center. The difference relies in the fact that the cells project more to the surface and the peripheral ramification is more pronounced. The most regular features show those cells which are at the fissure of the ventral cord. One finds these slender cells, however, also at other places. A dense brush of processes emanates from these cells like a chandelier diverging towards the periphery. The processes show a wave form course and they can cross over each other and show a widely branching at the periphery where they end with little bulbs. It should be noted here that all processes of the spider cells which reach the surface of the cord form **small roundish or foot formed swellings**. The complex of these nodules forms the true surface of the organ. A second diverging fiber bundle, consisting of only a few fibers, originates from cells located near the surface. These fibers are strong and often extend over long distances. They often intrude into the so-called glia septa, and from their surface origin, they can extend through the entire white matter, even deep into the grey matter.

Additionally, cells located in the deeper layers of the white matter have processes that reach the surface. These processes can be widely spread. The ramifications of the spider cells in the white matter are oriented in the same direction as the glia septa. The **glia septa** are basically composed of bundles of peripheral processes of astrocytes in the white and peripheral grey matter where they are arranged in chain-like longitudinal rows.

201

It is **quite unlikely** that peripheral processes of the ependymal cells are part of these chains in the adult human, see below. Anyway, in the embryonic stage the ependymal fibers are the major factor for the later orientation and direction of the Gliasepta. Before the actual spider cells appear, the ependymal cells with their processes reaching up to the surface show a typical orientation similar to the later arrangement of the glial bars. When the Golgi cells later appear, they follow this basic plan, first along their embryonic main process (see below) and later when this disappears, maintain their secondary extension in the white matter.

This typical and regular arrangement of the glial bars has been already noted by researchers before and this has also been illustrated in detail before (for instance by Frommann). A previous misinterpretation was the idea that this was an insertion from the Pia mater. Still in the description by Schwalbe [1)], Vignal [2)], Obersteiner [3)] etc. play these pial septa an important role. But it is certain now that these are exclusively bundles of ectodermal support cells. Also, the Septum posterius belongs to this category; with the difference, however, that the posterior ependymal cells of the central canal,

which send in the adult their processes to the surfaces, also participated in its formation. Anyway, the Pia mater does not contribute to this dorsal medial band, from the connective tissue it contains anything except blood vessels and their Adventitia[9#].

[1)] G. Schwalbe, Lehrbuch der Neurologie. Erlangen 1881, p. 303.
[2)] W. Vignal, Sur le développement des éléments de la moëlle des mammifères. Archives de Physiologie normale et pathologique, Tome 1884. p. 230.
[3)] H. Obersteiner, Anleitung beim Studium des Baues der nervösen Centralorgane. 2. Aufl., Wien 1892.

The composition of the **glial cover** (Gierke loc. cit., p. 510) or **glial rim,** which forms the outer layer of the spinal cord, can also be very well visualized with the Golgi stain, (Figure 22). If one studies any cross-sections of the spinal cord labelled with the Weigert copper haematoxylin stain, so it is evident that the dark blue fiber bundles of the roots do not reach the surface. Instead, they are completely covered towards the surface by a **light yellow fiberless seam**. This surface glial layer for which I propose the name peridym, was already noted by several researchers over the past decades. Already Bidder mentioned it shortly (loc. cit., p. 35). Frommann [1)] has described it in more detail and has recognized that it is composed of felt-like fibers with interspersed cells. Kölliker (Handbuch 5. Aufl. 1867, p. 268) went a step further. We find a correct notion in his work that this rim layer is composed of a dense meshwork of most delicate ‘connecting tissue cells’. But only Golgi correctly described in the year 1871 that it is partially composed of the ramified tangential glial cells and from processes running toward the surface originating from glial cells in deeper areas. Recently Schaffer described that layer in more detail (loc. cit.,).

After studying the sections I received from Weigert, I found that the peridym of the spinal cord varies in thickness. In the area of the intumescences [10#], namely at the swellings of the loins [11#], it is strongly developed and increases in size toward the conus terminalis. As Frommann already described, it is broadest at the entry zones of the ventral and dorsal horns. Schaffer correctly reported that the glial cover differs in its developmental state among individuals. The inner border of the peridym is always spiky due to the numerous intrusions of the Gliasepta. After staining with nigrosine or Weigert’s glial stain, it appears as a dark seam.

[1)] C. Frommann, Untersuchungen über die normale und pathologische Anatomie des Rückenmarkes. Jena 1864, p. 28.

203

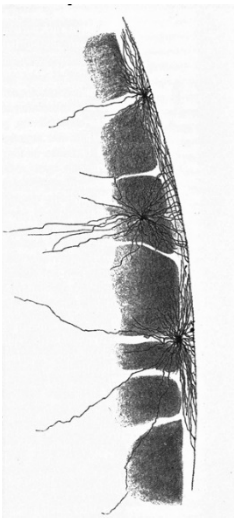

***Figure 22**. Spider cells at the surface ("peridym") from a spinal cord of a 9 month old child.*

The slides with the Weigert glial stain show, in this layer, a thick felt and fibers partially oriented in tangential and longitudinal direction or even radially.

It makes no sense to describe the more or less imperfect images obtained with other staining methods but to rather study the images enabled by the Golgi method on newborns and children (see Figure 22). The peridym is not composed, as some researchers thought, out of a felt of isolated fibers, but of **highly ramified cells, namely astrocytes**. The main component are astrocytes positioned tangentially and stretched out. Their processes are running parallel to the surface into all directions and they form the circular and longitudinal patterning of this layer. Rarely is the cell body right at the surface, but usually it is located in the middle or in lower layers of the peridym. These cells have very small processes towards the surface which terminate with a bulb.

204

The tangential fibers often turn toward the surface after a long course and end with thickenings (end bulbs). In addition to their tangential processes, these surface astrocytes have an inner, backward-directed bundle composed of only a few fibers, which projects into the Glia septa in the white matter and penetrates it deeply. There are also astrocytes located not within the peridym but slightly below it or even deeper. They are still close to the surface within the white matter, and part of their outer processes change direction within the peridym to a tangential orientation.

A significant part of the peridym formation comes from bushy stiff processes of spider cells, running radially towards the surface. These cells are positioned in deeper layers and are responsible for the radial fiber pattern of this layer.

All these fibers, both the radial processes from deeper layer cells or the tangential processes of superficially located peridym astrocytes, have thickenings (bulbs) at the end. These bulbs form a mosaic at the surface and actually form a thin and complete **border membrane**, kind of a cuticula (Membrana limitans meningea, His) which covers the ectodermal spinal cord (*and isolates it*) completely against the Pia mater. The bulbs of the different fibers are not always of similar thickness. I assume this avoids gaps in the border membrane, which would occur if the bulbs were of similar

size since these projections of the radial fibers sometimes differ in tightness. Gierke wrongly considered this membrane as part of the Pia, by describing it as ‘endothelial membrane’.
This border membrane composed of the endbulbs is very delicate and is termed Cuticula medullae spinalis. On well conserved preparations it appears as fine homogeneous seam and just below are the tangential fibers of the peridym. Sometimes, however, we see another picture. Between this seam and the tangential fibers of the glia rim appears a cleft-like intermediate space.

205

I believe this is an artifact, and I think it happens as follows: It seems that this cuticula is soldered to the inner layer of the Pia mater. How this attachment is accomplished is not clear, as an organic adhesion mediated by tissue specializations, such as pia processes entering the spinal cord, does not exist. In my opinion, a more amorphous glue must be present. This attachment is quite strong, as when shrinking occurs due to hardening solutions, the aforementioned cuticula appears more strongly attached to the *Pia mater* than to the fibers of the peridym. The resulting gap observed during shrinking is not between the *Pia mater* and the border sheath but between the border sheath and the peridym. This gap can vary depending on the shrinking process; it appears ladder-like and is interstratified with the ripped-out end bulbs of the radial processes, which originate from surface spider cells or those in their vicinity. Specifically, the hardening method introduced by Kultschitzky promotes the formation of such artificial gaps. In many of my spinal cord slices, I did not observe this gap formation, whereas in another preparation treated with the aforementioned hardening method, I clearly observed it. I am therefore of the opinion that this is not a natural phenomenon, and I cannot support the claim by Gierke and Schaffer (*loc. cit., Arch. f. mikr. Anat.*, p. 48) that it is a lymphatic space.

Formation of such border sheath is not restricted to the surface of the spinal cord. A very similar border sheath or terminal membrane is also present at the wall of the canals in which the blood vessels are imbedded which are found in the center of the spinal cord. If one analyses, in Golgi preparations, the relationship of the spider cells to the blood vessels, one finds that the former have a similar relationship to the walls of the canals containing larger vessels as to the surface of the cord.

If a blood vessel obstructs a spider cell, its processes do not circumvent the vessel but terminate at it with a thickening, similar to what is observed on the surface of the spinal cord. Since the processes of astrocytes converge from all directions toward the blood vessel, they form a delicate, tube-like cuticular membrane that separates the spinal cord substance from the blood vessel. A canal is thus formed, within which the blood vessel, surrounded by weak adventitia, is embedded in the spinal cord structure as a distinct foreign body.

In many stained preparations of the spinal cord and the brain one can find areas where the vessel does not completely fill its canal so that there is a narrow, light and empty space between the canal and the vessel. It remains speculative whether these spaces, which certainly become more pronounced by the shrinking process of the blood vessels during hardening, really represent perivascular lymphatic spaces as suggested by His [1)] and Obersteiner [2)]. At present this remains unlikely since first, there is still no proof for a connection of these spaces with the outer lymphatic vessels in the spinal cord (also His could not show this connection). Second, this epicerebral resp. epimedullar space, i.e. the non-existing cleft between the surface of the central organs and the 'Intima pia', the inner continuation of which would represent these clefts, are not to be considered as a lymphatic space according to the renowned studies by Key and Retzius [3)].

Our knowledge on the structure and nature of glia would remain highly fragmentary

[1)] W. His, Über ein perivaskuläres Kanalsystem in den nervösen Centralorganen und über dessen Beziehungen zum Lymphsystem. Zeitschr. f. wiss. Zoologie, Bd. XV, 1865, p. 127.

[2)] H. Obersteiner, Über einige Lymphräume im Gehirne. Sitzungsberichte der kais. Akad. d. Wissensch. zu Wien, Bd. 61, Abt. I, 1870, p. 57.

[3)] A. Key und G. Retzius, Studien in der Anatomie des Nervensystems und des Bindgewebes. I. Hälfte, Stockholm 1875, p.41.

if we would not consider their **development**. Only this will result in the final understanding of the supportive cells. The study of adult astrocytes will not give reliable information on their origin and the character of these elements. If one studies the highly ramified glial cells as revealed in Golgi preparations, so it seems to be in the first view quite obvious to relate them to the star shaped cells of the connective tissue. The morphology of these elements would support that view and furthermore the observation that the astrocytes form a supportive structure, in particular in the area of the

white matter. An additional observation is that most stainings also label the pial lamella at the surface of the spinal cord as well as the cortical layer of the spinal cord, the peridym, so these two structures are in very close contact, basically glued together and form a continuous layer which is apparently not separated by any border line. This could lead to the assumption that the glia septa of the white matter terminating into the peridym are a continuation, an invasion of the pia mater.

It is unsurprising that, for decades, spider cells and everything else in the spinal cord and the entire central nervous system that lies between the nerve cells and nerve fibers were considered connective tissue, and the cells were regarded as connective tissue cells. This opinion originated in the early days, when the intermediate substance of the central organs was regarded as a soft, granular matter. This view was further supported by the observation that the supportive substance has a fibrillary structure. Thus, as long as glia was regarded as a granular mass, it was stated that, although it belongs to connective tissue, it is distinct from the normal connective tissue found in other regions (see, e.g., Virchow, Cellularpathologie, 3rd ed., 1862, p. 257). Once glia was considered a fiber reticulum, the assumption that it was fibrous connective tissue appeared to be established.

208

It was not until recently that the connective tissue nature of glia was considered to be common knowledge. Golgi for instance was strictly talking about connective tissue cells in his important work from 1871, a view which he gave up already in 1885. This view was also brought forward by Gerlach (1870) and Boll (1874) and even still in the summary by Obersteiner in 1892 (II. ed.) considering the supportive tissue as a connective substance. Even in most recent reports one can find text passages where spider cells are considered as' cells of the reticular connective tissue' (e.g. Neurol. Centralblatt, 1893, p. 803 etc.). This view culminated with Gerlach who considered that the entire glia consisted of elastic fibers.

The recent research has changed that picture fundamentally. The **neuroglia is now considered ectodermal** and this is the main result and a reliable result, which has come from recent research. The supportive cells origin from the same Anlage as the nerve cells. This does not mean that they are considered nervous in the physiological sense. Only the origin is common while with regard to the functional aspects there is a differentiation into neurocytes, i.e. cells that are characterized by outgrowth of a nerve and related to nervous functions, and those cells that form the supportive

structure for the nerve elements. These support cells, spongiocytes, also serve as isolators according to P. Ramón, R. y. Cajal and Cl. Sala. This functional differentiation is similar as in sensory epithelium, for instance in the olfactory mucosa where the elements develop into sensory neurons and epithelial cells. Of course, the support elements of the sensory epithelia are characterized by simple morphologies, while in the nervous system the cells with the support function are highly complex.

When we study the historical development of this fundamental recognition, we find statements that are not based on the direct observation of the developmental processes of the 'intermediate substance' but rather on the impression that the glia in its final stage made on the observer.

209

In that sense the report by R. Wagner [1)] can be interpreted who described the fine granular intermediate substance as nervous which probably means that it originates like the nervous elements from the ectoderm.

If we want to resolve the question of the origin of the supportive elements, it is mandatory to directly study the histogenesis of glia. This is the reason why histologists studying glia extended their analysis to development, and in this context Boll (a. a. O.), Vignal [2)] and Gierke (a. a. O.) should be mentioned in particular. These three researchers came to the same main conclusion. All three considered the elements of glia, the spider cells, not as connective tissue intruders but as elements that originate locally from precursor cells in the *Anlage* of the central nervous system. Boll (p. 14) reported that connective tissue elements are present locally from the beginning, forming an integral part of the embryonic tissue. They are not later inserted into the nervous parts via intruding processes originating from the *Pia mater*. Based on that statement, it seems inconsistent that Boll still referred to astrocytes as connective tissue cells and glia as the connective tissue of the central nervous system. Vignal reached the same conclusion. He observed that the initially nucleus-free white matter of the spinal cord in human embryos becomes filled with nuclei, i.e., glial cells, during development, and he found no evidence that these cells intruded from outside. Instead, he suggested that they migrate from the grey into the white matter, an interpretation that, of course, supports their ectodermal origin.

Gierke was the strongest proponent of the ectodermal origin of the supportive tissue. However, it should be noted that his embryological observations do not significantly advance Vignal's findings. Gierke's strong standpoint is primarily based on the inspection of adult tissue rather than developmental observations.

The view of the above scientists was strongly supported by the embryologic investigations of His [3], which are distinct from the previous studies in that he analyzed very early stages. The research by His clearly demonstrates that at least part of the neuroglia originates from cells of the medullar plate. Although, for the origin of the Deiters cells, His left the possibility open that they are originating from connective tissue. He distinguished two elements of neuroglia, the main element being a fibrous network called Myelospongium, which is originating from the ramified outer processes of the ependymal cells

[1] R. Wagner, Neurologische Bemerkungen. Göttinger Nachrichten, 1854, p. 28.
[2] W. Vignal, Sur le dévelopment des éléments de la moëlle des mammiféres. Archives de Physiol. normale et pathol. T. 1884, p 230.
[3] W. His, Histogenese u. Zusammenhang der Nervenelemente. Archiv. f. anat. u. physiol. Anat. Abt. 1890, p. 103.

210

and therein embedded the Deiters brush cells [12#], which he considered to be secondary elements invading the cord structure as foreign, connective tissue cells.

It should also be mentioned that Götte[1], based on his embryological studies, had earlier considered all components in the spinal cord, including the supportive cells, to be derived from the ectoderm. This research entered a new phase when the Golgi method was applied to address this question. This marked a significant turning point in the study of neuroglial cells. While previous methods yielded several important observations, these techniques were quite limited, both in the reliability of their results and in their ability to reveal main points without detailing these processes. Only the Golgi method provided a clear and comprehensive view of the histogenesis of supportive cells, capturing all details.

Golgi made the first observations. Using the chrome-silver method, Golgi demonstrated in 1885 [2] that during embryonic development, the basal ends of all ependymal cells, i.e., the so-called epithelial cells of the central canal, extend as radial fibers toward the outer surface of the cord [3]. This important finding marks the starting point for the further development of our knowledge and the origin of neuroglia. Golgi himself did not investigate the developmental processes further.

In 1886, Fritjof Nansen [4] was the first who succeeded to depict the entire neuroglia of the spinal cord of a vertebrate using the Golgi method. His studies were done in muxines [13#]. The astrocytes of these animals have very characteristic

features, which easily identifies them as ectodermal elements similar to nerve cells. In these animals, the long ependymal fibers observed by Golgi in the chicken embryo exists throughout life, even in the adult.

[1] A. Götte, Entwicklungsgeschichte der Unke. Leipzig, 1875, p. 275.
[2] C. Golgi, Sulla fina anatomia degli organi centrali del sistema nervoso. Milano, 1885/86, p. 178.
[3] Actually, before Golgi, Hensen has already described the radial system based on other methods. Hensen reports for the rabbit that even at a time point when the gray matter lays on top of the fairly large epithelium of the central canal, one can find cells in that epithelium which have processes that extend through the entire cord in the form of radial fibers and insert with a slightly enlarged basis into the „Membrana prima[14#]". (Zeitschrift für Anatomie und Entwicklungsgeschichte, Band I, 1876, p. 372.)
[4] Fr. Nansen, Structur and Combination of the Histological Elements of the Central Nervous System. Bergen's Museum Aarsberetning for 1886. Bergen 1887, p. 160.

211

Hence, the conditions are here are much more obvious as compared to the higher vertebrates, and despite that Nansen did not study the developmental processes of the support elements directly, the really important conclusion he could derive: neuroglial cells are of ectodermal origin and develop from epithelial cells of the central canal.

Ramón y Cajal [1] has studied the development of astrocytes in chicken based on his silver impregnation analyzing several (*developmental*) states. He also commented in his studies on the situation in mammals. His results support Nansen´s view, despite of the fact that Cajal did not know Nansens ' work. First, Cajal carefully reports the properties of the radial ependymal cells in the chicken embryo, complementing Golgi's observations by describing the unusual properties of these cells located ventral and dorsal of the central canal. From day 8 on, one can observe ependymal cells with cell bodies no longer close to the central canal, but located more outward. These elements are the precursors of spider cells. The latter appear in their characteristic form between day 9 and 10 and can be all traced back to dislocated and strongly transformed ependymal cells. Cajal's observations were supported by several other researchers such as von v. Kölliker [2], Van Gehuchten [3], Lenhossek [4], Retzius [5] and Cl. Sala [6] who not only confirmed the major points of the Spanish histologist but also added further details. I myself was the first to analyze these conditions in the human embryo. Here, we will not give all the details of these studies because they will be subsequently discussed.

[1] S.R. y Cajal, Sur l'origine et les remifications des fibres nerveuses de la moëlle embryonnaire. Anat. Anz. Jahrg. V, 1890, p. 115. – Derselbe: Nuevos observaciones sobre la estructura de la médula espinal de los mamiferos. Barcelona, 1890.
[2] A. Kölliker, Zur feineren Anatomie des centralen Nervensystems. Zweiter Beitrag: Das Rückenmark. Zeitsch. für wissensch. Zoologie. Bd. LI, 1890, p. 31.
[3] A. Van Gehuchten, La Structure des centres nerveux. La moëlle épinière et le cervelet. La Cellule, T. VII, 1891, p. 104.
[4] M. von Lenhossék, Zur Kenntnis der Neuroglia des menschlichen Rückenmarkes. Verhandl. der Anat. Gesellsch., 5. Versamml. 1891 (Anat. Anz., p. 193.) – Vergl. ausserdem die Darstellung des Stützgewebe in der 1. Auflage dieses Buches, p. 45 – 62.
[5] G. Retzius, Epidym und Neuroglia. Biolog. Untersch., Neue Folge V, 1893, p. 9.
[6] Cl. Sala y Pons, La Neuroglia de los Vertebrados. Barcelona 1894.

The beginning of development is the stage where the medullar tube has just contracted and is composed of a single layer of column-like cells (Hensen). The nuclei of the cells appear to be at different levels, creating the illusion of a multilayered structure. However, this is an illusion, as all cells terminate with their two often inconspicuous ends at the central canal and the border layer of the medullar tube. These initial cells of the central nervous system belong to the category of support cells. Therefore, it appears that the support system of the central nervous system is ontogenetically older than the nervous elements. As the cord increases in size, these cells elongate considerably.

They are thinning out into fine fibers which penetrate the cord in a radial fashion as ependymal fibers. The inner part, which is closer to the central canal has more a character of a cell body, incorporating a nucleus which is in early development at different distances from the central canal [15#] and later in development gets closer to the central canal. This part is the ependymal cell and the outer part the ependymal fiber. The entire system of these radial fibers represents the ependymal framework or the ependymium of the cord.

Golgi who was the first who reported on the radial arrangement of these first order supportive elements in the chicken, and demonstrated that, at this early stage, the entire glia is formed only by the ependymal cells. Golgis's observations were since supported by other studies for the chicken but also for other vertebrates by a number of scientists, e.g. by Fritjof Nansen (Amphioxus, Myxine), Burckhardt [1)] (Amphibia), Falzacappa [2)] (chicken), R. y. Cajal (chicken, mammals, reptiles), Lachi [3)] (chicken), v. Kölliker [4)] (mammals), Lenhossék (humans, chicken, mammals, Selachier [16#], Petromyzon [17#]),

[1)] R. Burckhardt, Histologische Untersuchungen am Rückenmarke der Tritonen. Archiv f. mikrosk. Anat., Bd. XXXIV, 1889, p. 142.
[2)] E. Falzacappa, Ricerche istologiche sul midollo spinale. Rendiconto della R. Accademia dei Lincei, Vol. V. 1889, p. 696.
[3)] P. Lachi, Contributo alla istogenesi della nevroglia nel midollo del pollo. Memoria della Soc. Toscana di Scienca natur., Vol. 11, Pisa 1890.
[4)] A. Kölliker, Zur feineren Anatomie des centralen Nervensystems. Zweiter Beitrag: Das Rückenmark. Zeitschr. f. wissensch. Zool., Bd. 51, 1890, p. 32. - Derselbe: Handbuch der Gewebelehre, 6. Aufl., Bd. II, 1893, p. 136.

Van Gehuchten [1)] (chicken, mammals), Retzius [2)] (all classes of vertebrates), Lawdowsky [3)] (frog), Cl. Sala [4)] (all classes of vertebrates, in particular batrachians [22#]). In the beginning of the development, the cord is penetrated by the regularly arranged, beautiful ependymal structure in all vertebrates. This is the precursor of the later support structure.

It seems the best strategy is not to focus on the subsequent development of the supportive structure by including the other support cells, but rather follow the development of these first elements of the cord, the ependymal cells, and eventually follow their fate in the developing spinal cord, an issue which we have not focussed on before. Thereby we will not study relationships between the ependymal cells and the newly forming astrocytes but rather analyse how the ependyma develops in different phases, independent whether they are the same elements which pass on to the next phase or whether it is a newly formed element. This early phase is illustrated in figure 23 which shows the ependymal cells of the medullar tube of a 4-day-old chicken embryo.

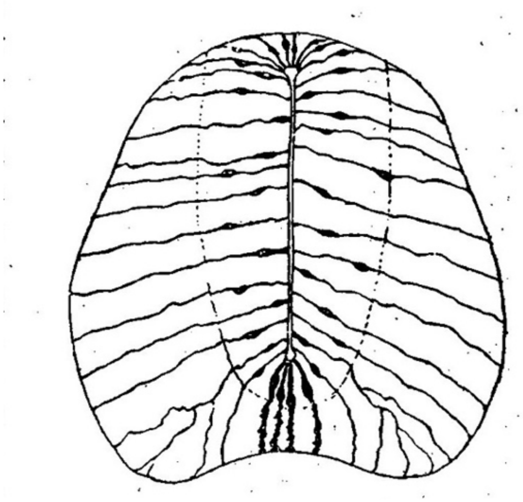

Figure 23*. Cross section of a medullar tube of a 4-day-old chicken embryo with labelled radial cells.*

1) A. Van Gehuchten, La Structure des centres nerveux. La moëlle épinière et le cervelet. La Cellule, T. VII, 1891, p. 104. – Derselbe: Le système nerveux de l'homme. Lierre 1893, p. 224.
2) G. Retzius, Zur Kenntnis der Ependymzellen der Centralorgane. Verhandl. des Biol. Vereins in Stockholm, 1891. – Derselbe: Ependym und Neuroglia. In Biol. Unters. N. F. V, 1893, p. 9.
3) M. Lawdowsky, Vom Aufbau des Rückenmarkes. Archiv f. mikrosk. Anatomie, Bd. 38, 1891, p. 264.
4) Cl. Sala y Pons, Estructura de la médula espinal de los batracios. Barcelona 1892. – Derselbe: La Neuroglia de los Vertebrados. Barcelona 1894.

214

From the still split-shaped central canal, ependymal fibers run through the cord, laterally in an almost parallel arrangement and radially diverging at the top and bottom. The accumulation of their nucleus-containing parts in the inner region of the medullar tube results in the formation of a broad, nucleus-rich layer, called the inner layer by His, or the ependymal nuclear zone, as I would name it. In general, it corresponds to the later epithelium of the central canal, which develops from the broad layer through a successive shifting of the nuclei toward the central canal. All fibers of the ependymal cells terminate at the edge of the cord in small triangular enlargements. Ependymal fibers of the floor plate (His), or the later frontal commissure, have an unusually rough appearance. They are plump and feature small spines. One can already recognize at this early stage their **barrel-type arrangement**. This arrangement is, however, not yet fully developed and also obvious in the laterally positioned ependymal fibers. This arrangement, which will become much more evident later, results from the curling of the ventral roots which finally leads to the formation of the frontal fissure. The ependymal cells which are positioned left and right of the floor plate in the ventral area of the cord, are distinct from the unbranched fibers in that their processes branch into three to four fibers. Generally, it is usually only a single cell where this feature can be recognized at this stage. This early highly branched class of cells can be followed during the subsequent stages and enables us to understand, which rearrangements occur in the developing cord. The area in which they ramify, which originally forms the entire width of the ventral area of the cord, will be later completely included in the formation of the ventral fissure. Accordingly, the entire frontal area of the cord approaches from the sides.

Later stages are represented by figures 24 and 28. There are two features, which change in comparison to the properties of the earlier ependymal cells.

215

On the outward directed process one notes, in particular in the area of the grey matter, small irregularities, varicosities and rough threads. Even more obvious is that almost all ependymal fibers in their outer parts, in particular in the area where they enter the white matter, are branched into multiple parts, particularly in the 10-day old chicken. They form even bundles with branches that are broadly arranged and they are oriented towards the periphery where they terminate with characteristic endbulbs/nodules.

However, this rough appearance including the branching of the terminal part of the ependymal fibers is not a common feature. For example, it is not found to the same extent in the human spinal cord.

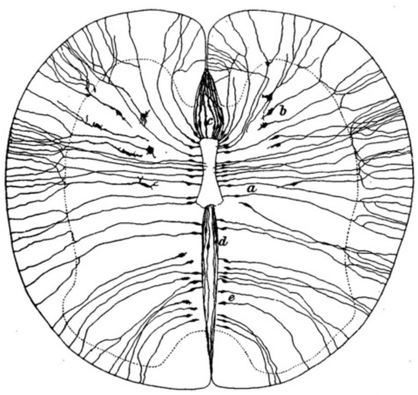

Figure 24. *Spinal cord of a 3-cm-long human embryo with labelled ependymal cells and astroblasts according to Retzius. a. ependymal cells, b. astroblast, c. frontal ependymal wedge, d. Septum posterius, E. astroblasts oriented towards the Septum posterius.*

216

This is illustrated in the figure in Table II (see at the end of the text), which shows the support system of the spinal cord in a 14-centimeter-long human embryo. Particularly interesting in this figure is the left part, where the ependymal cells are shown separately. This represents a later stage. The initial phase, in which the support system is exclusively formed by ependymal cells, has not yet been studied in the human embryo using the Golgi method. In a 3-cm-long embryo, as depicted in Retzius's drawing in Figure 24, both ependymal cells and the precursors of later glial cells are already present. I encourage readers to study the properties of the ependymal cells in Table II and Figures 24 and 29 in more detail.

The slim, spindle-shaped cell bodies of the ependymal cells consolidate at the central canal, forming the well-known delicate epithelial crest. At the basal pole, they transform into a delicate, smooth, nerve fiber-like process that projects radially toward the surface in a gentle, wave-like arrangement. It terminates in small, cone- or club-shaped thickenings.

In the outer region belonging to white matter, these fibers usually furcate into two to three branches in a sharp angle. A more extensive branching is only found in those ependymal fibers, which have their cell body at the border between the frontal and lateral part of the central canal. The processes of those highly branched ependymal cells are found in

the medial frontal roots, opposed to the frontal fissure. The number of the ependymal cells is restricted and they project at a wider distance from each other.

In the area of the frontal fissure the ependymal fibres appear rougher than the lateral ones and their meridian arrangement in a few-day-old chicken can be recognized as a very delicate form. Ramon y Cajal described this arrangement in chicken and in mammals first. Retzius [1)] named it “anterior wedge part”.

[1)] G. Retzius, Zur Kenntnis der Ependymzellen der Centralorgane. Verhandl. d. Biol. Vereins in Stockholm, 1891.

217

v. Kölliker described this formation as “ventral ependymal septum”. One can study the formation of this arrangement in several developmental stages (in chicken). It is related to the formation of the frontal fissure.

In the posterior region of the middle line, the ependymal fibers form a similar but a shabbier barrel-like formation, called “anterior wedge part” by Retzius. They form a sagittal bundle, the posterior ependymal bundle or Septum posterius. This Septum formed by parallel fibers extends in straight direction towards posterior to reach the surface in the area of the weak Sulcus posterior. At the most terminal part, the fibers show weak diversions and at the endings they all show the common enlargements. It should also be mentioned here that in this developmental stage a “posterior fissure” does not exist.

A gap can be recognized in the lateral extension of the ependymal fibers. The entire area of the dorsal horns and dorsal roots lacks ependymal fibers, except for the *Septum posterius*. This phenomenon can be explained by the developmental process. The central canal is originally a long sagittal cleft, as shown in Figure 23, which is terminated ventrally and dorsally by a small floor and ceiling plate. This cleft-shaped space disappears over time, except for the ventral part. The entire dorsal region disappears. What happens to the many ependymal cells that occupy the considerable dorsal region? Are they pulled forward and integrated into the epithelial crest of the later, much smaller central canal? This does not occur. As I demonstrated with the Golgi technique [1)] all remain in roughly the same region where they originally were, but their cell bodies move outward, lose their cilia, and transform from ependymal cells into Deiters cells, the future spider cells. Since these are the epithelial cells that originally penetrated the dorsal horn and dorsal root, they are not recognized at later stages in the cord.

[1)] M. v. Lenhossek, Zur Kenntnis der Neuroglia des menschlichen Rückenmarkes. Verhandl. d. anat. Gesellsch, 5. Versamml., 1891, Anatom. Anz. p. 93.

It is therefore evident that the central canal, which is originally fairly large in the dorsal-ventral direction, does reach its later size and form **not by an even reduction** but rather by a **fusion of the walls of the dorsal sections**. A similar view was put forward earlier based on investigations on embryos by Waldeyer [1)], Balfour [2)], His [3)], Barnes [4)], Corning [5)]. A similar result is also based on more recent publications, such as by Wilson [6)] and Prenant [7)].

Regarding the cell bodies lining the central canal (see Figure 25), it should be noted that each of these cells has an internal, enlarged cuticular seam (*membrana limitans interna*), and a small hair extends from its center. This hair is present early in development, as described by Retzius in the 3-cm-long human embryo. Based on Golgi staining, it appears as a single, very straight, often long, small rod, giving the impression of a stiff cuticular formation rather than a cilium. In the first edition of this book, I opposed the interpretation of it as a cilium. However, new evidence from v. Kölliker (*Handbuch der Gewebelehre*, p. 143) supports its identification as a cilium, and I no longer insist on my original viewpoint.

[1)] W. Waldeyer, Über die Entwicklung des Centralkanals im Rückenmark. Archiv f. path. Anat., 1876, Bd. LXVIII, p. 20.
[2)] F. M. Balfour, Handbuch der vergleichenden Embryologie. Übersetzt von C. Vetter. Jena 1881, II. Bd.
[3)] W. His, Zur Geschichte des menschlichen Rückenmarkes und der Nervenwurzeln. Abh. d. math.-phys. Klasse d. Kgl. Sächs. Ges. d. Wiss., Bd. XIII, 1886, p. 479.
[4)] Barnes, On the Development of the posterior fissure of the Spinal cord and the Reduction of the Central Canal in the Pig. Proc. Amer. Acad. arts and sc. 1884.
[5)] H. K. Corning, Über die Entwicklung der Substantia galatinosa Rolandi beim Kaninchen. Archiv f. mikrosk. Anatomie, Bd. 31, 1888, p. 594.
[6)] J. T. Wilson, On the Closure of the central canal of the spinal cord in the foetal lamb. Transact. Intern. med. Congress Sydney 1892.
[7)] A. Prenant, Critériums histologiques pour la détermination de la partie persistante du canal épendymaire primitif. Internat. Monatsschrift f. Anatn. u. Physiol., Bd. XI, 1894, p. 1.

I must indeed admit that this strong rod as it is shown in Golgi preparations may consist of a complex of fine hairs which are apparently fused due to the chrome/silver method.

On Table II (see at the end of the text) one can recognize that already at this stage the ependymal cells modestly contribute to the support structure.

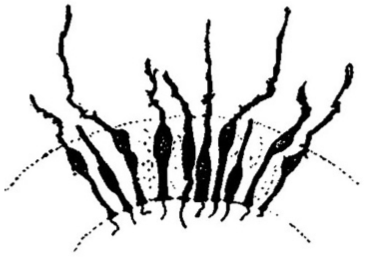

Fig. 25.

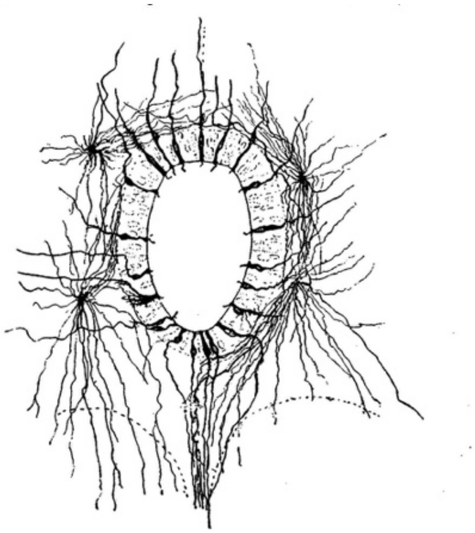

Figures 25 and 26*. From the spinal cord of a 23-cm-long embryo: ependymal cells and substantia gelatinosa centralis.*

220

They increase in importance and attention due to the fact that the **direction of their processes determines the arrangement of the support cells** in the later phase (they form the skeleton of the entire glial structure). Even more important – they form the oldest part of the support structure based on onto- and phylogenetic evidence and they are the direct descendants of the ectodermal cells which form the medullar plate. They may even represent these cells in a modified form. Their complex represents the original structure of the cord to which the other neuroglia is added at a later stage.

It is an interesting question how this ependymal structure further develops, in particular in the spinal cord of the evolved human. Does it exist in the form which is illustrated on Table II (see at the end of the text) only in the embryonic phase or will it be later taken over without changes? In particular the question arises whether all later ependymal fibers project to the surface. This is indeed the case for amphibia and fish including cyclostome [17#] and the amphioxus [18#]. For the higher vertebrates, however that can be only confirmed for the frontal wedge and the septum posterius. For the lateral ependymal fibers, R. Y. Cajal expressed the opinion that they degenerate during development, resulting in a free, branched terminal close to the central canal. This has been also concluded by Retzius, Cl. Sala and v. Kölliker.

In the first edition I was opposing this view and I speculated that this degeneration is only due to an insufficient staining of the ependymal cells in these later stages. I considered it likely that the ependymal fibers in the adult have very thin sparse fibers reaching to the edge of the spinal cord.

Based on my newer studies **I have revised my view and I now support these reductions processes as reported by the previously cited researchers**. I base my following description on the properties of the ependymium on studies of a 9-month-old child and I assume that at this phase it already represents the final stage.

221

The properties of the epithelial cells at the border of the central canal are not different from those in earlier stages. Within the cell body, the nucleus is positioned close to the central canal or, occasionally, slightly farther from it, resulting in variable forms of these cells. Within the nuclei, strongly stained nucleoli can be recognized when properly stained. The aforementioned cuticular plate in the inner region of the cells is still present and is intensely labeled blue with the new Weigert glia stain. Interestingly, the staining does not show continuous bands but instead reveals several adjacent spots, as reported by Weigert. Recently, Prenant [1)] also recognized these spots in the sheep embryo, although he observed only two per cell, located at the lateral endpoints of the cuticular plate.

The **anterior ependymal cells** terminate in rough fibrous processes. Also **in the adult stage they end at the floor of the frontal fissure** and at the neighbouring lower parts of its side walls. The meridian arrangement is at this stage quite blurry and the fibers do not form regular curves as in earlier stages, but appear quite as an entanglement. As a result, the whole arrangement loses its original, typical form. The fibers appear rough but even and unbranched.

The **posterior ependymal cells** also remain in their **primitive state,** in that they still form the septum posterius while projecting to the posterior median groove. This is quite obvious in my preparations. Before they are fused to this septum, they show a broad loosened arrangement at the posterior grey commissure, reminiscent of the earlier posterior ependymal wedge. It may have originated from the latter, but it is distinct from it due to the irregular course of the ependymal fibers. These lateral fibers form long irregular loops.

[1)] A. Prenant, Critériums histologiques pour la détermination de la partie persistante du canal épendymaire primitif. Internat. Monatsschr. f. Anat. u. Physiol., Bd. XI, 1894, p. 5.

In the septum itself, however, the ependymal fibers run parallel.

Thus, the septum **posterius essentially originates from the ependyum,** including additionally numerous embedded spider cells. This band was considered until recently as a sinking (insertion) of the pia mater and one has considered it as the "posterior fissure", which is completely filled by this extension. This view is no longer supportable; **in the lumbar part, the spinal cord is clearly unsplit in most regions**. It shows a sulcus but no fissura posterior. The septum posterius, which is not larger as the other glia septa of the white matter, represents a genuine formation of the spinal cord and does not contain anything from the pia mater [1)]. The fact that the *septum posterius* does not create a discontinuity in the spinal cord substance is evident from the presence of real spider cells within the *septum posterius*, which project into both halves of the spinal cord. Moreover, astrocytes located in the neighboring posterior funiculus project across the septum. However, it should not be denied that, particularly in the spinal cord of adults, a shallow slit formation originating from the posterior sulcus can be observed. It is observed in the lumbar region, as I noted in my preparations, consistent with older reports from Arnold [2)] and a recent report by Schaffer (*loc. cit.*, *Arch. f. mikr. Anat.* 1894, p. 43). However, it never extends deeper than a third of the *septum posterius*. This slit formation is a secondary phenomenon related to the entry of blood vessels at the posterior midline. Even if such vessels are not observed in cross sections, this could be explained by the slit extending longitudinally beyond the zone of vessel entry.

[1)] A similar result was obtained recently by A. Robinson in a diligent embryologic study (On the development of the posterior colums etc. Studies in Anatomy from the Anat. Departm. of the Owens College. Vol. I., Manchester 1891, p. 98).
[2)] Fr. Arnold, Bemerkungen über den Bau des Hirns und Rückenmarks. Zürich 1838, p. 3.

It must be considered as a secondary splitting up of inner elements of the spinal cord and cannot be compared to the frontal fissure since this is generated through growth differences of the ventral parts, as a groove-like insertion of the surface between sprouted parts.

With respect to the **lateral ependymal cells**, it should be emphasized that their area within the epithelial crest in the adult spinal cord is quite limited. Their processes are oriented towards the anterior and posterior commissure and they contribute to the formation of both ependymal wedges. They extend, from front and back, far into the lateral wall of the

central canal. Only a small part of the epithelium remains for the lateral ependymal cells. Obviously, beyond the developmental stage as illustrated in table II (see at the end of the text), a considerable number of lateral ependymal cells move out and convert into spider cells. Those cells remaining in the epithelium in the adult cord extend a fiber from their basal pole, which penetrates the area of the so-called substantia gelatinosa centralis in a radial fashion. These fibers are very thin, much thinner than ependymal fibers of the frontal and posterior area and terminate usually in the commissural part of the grey matter by splitting into two or three branches which end freely after an irregular pathway. One can conclude that this **long peripheral process disappears**. This atrophy starts (developmentally) when a human embryo is 35 cm long, while I have observed that in a 30 cm long embryo, the fibers still extend to the Pia mater. There seems however to be variability in different individuals since Retzius observed the atrophy already in a 15 cm long embryo. After this phase one can rarely follow the course of single fibers, due to their couverture and their very varicous properties indicating a beginning of a granular disintegration until the reduced picture described above gradually emerges. I have to note, however, that one can come to another conclusion, namely that the atrophy does not occur but that the short **ependymal cells are not identical to the long earlier forms, but are a novel cell type** without long processes, which migrate out of the epithelium and convert into glial cells.

224

It should be mentioned that the degeneration of the ependymal structure **is only found in higher vertebrates** while in lower vertebrates the ependyma plays lifelong an important role. I should also emphasize that the above statements refer **only to the spinal cord**. In some other parts of the central nervous system the ependymal cells and their fibers remain also in the adult in the previously embryonic form, also in higher vertebrates. It is not the focus of this work to go into more the details [1]. The central canal, including its ependyma, has no functional significance in humans and is a developmental relic, as evidenced by regressive changes in the central canal, particularly obliteration. In such cases, it must always be considered that an artifact may result from mechanical manipulation during the dissection of the spinal cord. Careful experimental studies by Van Gieson [2] have shown that even minor external manipulations can lead to substantial changes in the internal arrangement of the spinal cord. Considering that careless manipulation during spinal cord dissection can lead to substantial changes, such as apparent duplication of the cord or heterotopies of grey matter, it is likely that the central canal may also undergo changes.

Let us **focus now on the development of the glial cells proper**, namely our astrocytes. My own experiences on that issue, in particular with regard to the first developmental phases, are mainly related to chicken embryos, but I can also recognize based on preparations, which I have of very early mammalian embryos, that these processes are very similar there.

1) If one is interested to learn more about the support elements in other areas of the central nervous system, one can partly find this information quite scattered in different specialized publications, or very well summarized in the commendable dissertation by Cl. Sala y Pons: La Neuroglia de los Vertebrados. Barcelona 1894. Unfortunately, this study is not easily accessible.

2) Ira van Gieson, A study of the Artefacts of the Nervous System. New York medical Journal, 1892.

225

The formation of astrocytes begins in chickens on day 8, as I have observed (Fig. 27). Until that stage, only ependymal cells are present. At this stage, beginning in the ventral horn, some elements resembling ependymal cells project toward the periphery of the cord with their fiber-like processes and terminate in the well-known triangular enlargement. They are distinct from the ependymal cells in that their cell-body is **no longer located at the central canal** but is more outward positioned and connected with the ependyma at most by a very thin process. However, often there is no connection to the ependyma. The cell-body appears spindle-shaped, elongated into the direction of the peripheral fiber and in this first phase completely smooth.

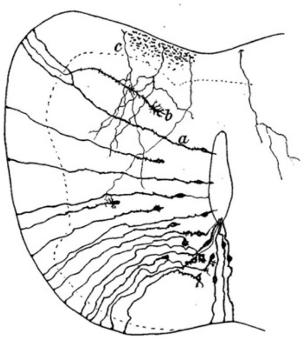

Figure 27. *Spinal cord of an 8-day-old chicken. Development of the support cells. a) ependymal cells; b) already detached ependymal cells (astroblast); c) collaterals of the ventral roots.*

226$

In the beginning one finds these cells only in the inner part of the grey matter and only quite sparse, but already in the following days (Fig. 28) they appear more numerous and extend to the edge of the grey matter.

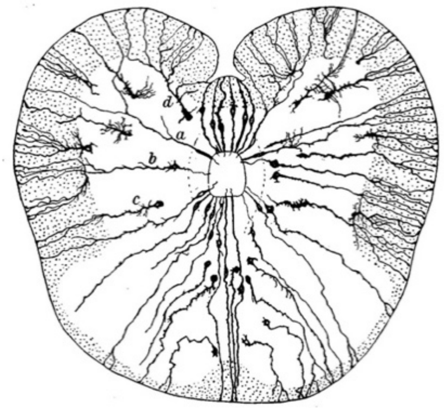

Figure 28. *Development of the support cells of the 10 day old chicken. a) ependymal cell, b) emigrated ependymal cell, which is still connected to the central canal by a thin thread, c) precursor of a later spider cell.*

The processes described here are the essential facts for the generation of the glial cells and are the key for understanding this problem. By comparing all the transitional forms, it is easy to conclude that these elements arise from the same state since they are positioned, like the ependymal cells, at the central canal. They also have a little hair like those. They develop from that state in the way that their cell body migrates out of the epithelial layer in centrifugal direction.

227

Their cell body is in the beginning attached to the central canal, is thinning at this end and subsequently only attached with a very delicate fiber. The latter disappears soon and is obviously incorporated into the cell body and is used for its enlargement. The question is whether this is represents a spontaneous movement of the cell or whether it is a result of the pull exerted by the peripheral process. This is difficult to decide.

The elements described here are the precursors of at least some of the later spider cells, namely the astrocytes (**astroblasts**). They originate from the same Anlage as the nerve cells, namely from the mitotic cells strung together as a germ layer around the central canal. In subsequent development, the analogy with neuroblasts is surprising: like the formation of processes in neurons, one observes the generation of the peripheral process in astrocytes. Like neurons, they gradually move away from the central canal to the outer areas of the cord, populating first the grey matter and then the white matter. It is likely that the numerous ependymal cells found in embryos represent not final formations but transient developmental stages of spider cells. Only the last generation of ependymal cells at the floor and ceiling plates remain as elements of the ependyma in the fully developed cord. It is evident that, during embryonic development, particularly in the early stages, there is continuous formation and movement of ependymal cells. From some mitotic cells of the inner cord layer, not only are nerve cells generated, but also elements that send rough processes to the periphery of the spinal cord. These cells exhibit features of epithelial cells.

This is the **ependymal stage of the astrocytes**. This is followed by the already described shortening of the cell fiber by which the cell body is moved outward.

As already stated, this migration starts in the ventral horn or at least can be seen here first. Soon later migrated ependymal cells appear also in the lateral area of the medulla tube as well as in the dorsal horn.

228

In the 8-day-old chicken (Figure 27), these elements are still restricted to the grey matter, but by the 10th day, many have reached the rim of the white matter, and some have even penetrated into it, although their number is still low. Gradually, they migrate further, with an increasing shortening of their peripheral processes, into the peripheral zones of the cord, reaching the seam of the white matter, which at that time is still very narrow.The border of the grey matter appears to act as an obstacle for migrating astroblasts, requiring effort and periods of rest to pass. This finding is supported by observations from normal staining series of young embryos from birds, mammals, and humans. For instance, in a 2-month-old human embryo, the grey matter is densely filled with nuclei that largely correspond to those of emigrating astroblasts, while the white matter remains devoid of nuclei at that time.

Only in later stages glial nuclei are present (in the white matter) which seem to be moving away from the grey matter in which the arrangements of nuclei become sparser. Already Vignal made this observation and has interpreted it in that sense. More precise statements one can find in v. Kölliker's “Gewebelehre” (6. Aufl. 1893, p. 133).

It is most likely that the first generation of astroblasts is destined for the white matter while the later ones remain in the grey matter. This can be based on the finding in Figure 24 produced from Retzius' illustrations, which describes the condition of the support cells at a 3-cm-long embryo. Compare it with our Table II, which shows a later stage, i.e. 14 cm length. One can recognize in the drawing by Retzius that most astroblasts have a branched process in the white matter while in my illustration only those in the white matter display this feature. It seems obvious that also all ependymal cells shown in this figure, are precursors of astrocytes of the white matter, indicated by the branches of its process. It is likely that in this period glial elements of grey matter are not yet generated.

229

During the emigration of these support cells, another feature similar to the formation of dendrites in neuroblasts can be observed: small tips or twigs, like dendrites, appear fairly early on the originally smooth cell body as protrusions from the cell protoplasm. These protrusions are the secondary twigs of the astroblasts. In most cells, these threads[19] also occur along shorter or longer parts of the process.

The support system of the cord remains in this transitional stage for some time. Both illustrations (Figure 24 and Table II) depict this condition, despite being from distinct stages of the embryo. A detailed comparison of these figures reveals significant differences. In the 3-cm-long embryo, the emigrated astroblasts are sparse and restricted to the grey matter, whereas in the 14-centimeter-long embryo, their numbers have increased considerably, and they are more evenly distributed across the cross-section of the spinal cord.

Let us examine the figure in Table II (14-centimeter-long embryo) in greater detail, particularly regarding the support cells shown on the right half.

We see that the cross section of the cord is interstratified with a dense system of strong, radial fibers in a columnal fashion, which project from the inner parts into the direction determined by ependymal fibers, namely towards the surface, and dominate the image of the support system. They originate from the still elongated cell bodies, which still are equipped with very short twigs.

These features are distinct in the grey and white matter. In the grey matter each cell continues with only one peripheral process which diverges only towards the outer region of the cord into generally three to four branches which all reach the pia mater in the form of the terminal bulbs already known from ependymal fibres.

230

As long as this process is in the grey mater, it is distinct from the smooth ependymal fibers by the presence of multiple tiny fibrils and appendages while in the white matter it is smooth and more even. Very characteristic at this stage is the arrangement of neuroglia fibers **in the area of the posterior grey commissure and on both sides of it**. Since it is quite difficult to illustrate these complex conditions in words, I would rather ask the reader to focus on Table II. The cell bodies are not oriented radially to the central canal like the other support elements, but are, at the beginning of their peripheral process, located rather vertically or oblique with respect to the Septum posterius. The processes originating from them are not arranged radially to the surface but instead run in curves and an S-shape, approximately parallel to the border between the dorsal root and grey matter. The transverse first part is directed, with gentle bending or stronger buckling, into the next sagittal section. It penetrates the dorsal horn and dorsal root in an inverted convex form to reach the surface in an undivided manner. The fibers are not completely parallel but are curved, with this curvature being more pronounced in lateral positions, where the frontal fibers acquire a converging and the posterior fibers a diverging arrangement. The position where they are closest corresponds to the region of the medial dorsal horn, where the entry bundles of the dorsal root enter the grey matter.

Additionally, the outer support cells of the dorsal horn tend to arrange their cell bodies and initial processes transversely to the midline, forming an arch. In the frontal and medial parts of the cross-section, the arrangement of support cells appears to be determined by the central canal as the midpoint of their radial extension, whereas for the posterior glial elements, the determining factor is the *Septum posterior*, i.e., the obliterated part of the central canal.

I would like to note that v. Kölliker was the first to describe this arrangement. Figure 28 of his spinal cord study (spinal cord of the sheep) illustrates it clearly.

I have extensively reported this in my study on glia [1] and Retzius has recently supported my findings.

This arrangement is related, as mentioned above, to the obliteration conditions of the central canal. The unusual position of the cell bodies can be explained by the fact that these cells originally formed the ependymal cells of the dorsal, obliterable part of the central canal, and during their emigration and conversion to support cells, they retained their original orientation for quite some time. The astroblasts present at this stage in the Rolando substance are characterized by a remarkable feature. Similar to earlier observations by Cajal in the newborn cat, I found that they are characterized by a large bush-like arrangement of delicate fibers, which cannot be observed in other areas of the embryonic spinal cord. Even fibers originating from the most posterior astroblasts of the dorsal horns, which merely pass through the Rolando substance, exhibit this dense, furry arrangement.

The numerous spider cells that compose the neuroglia in the white matter of the embryo only occasionally exhibit the features of the cell forms in the grey matter, while the majority display their own distinctive characteristics (Figure 29).

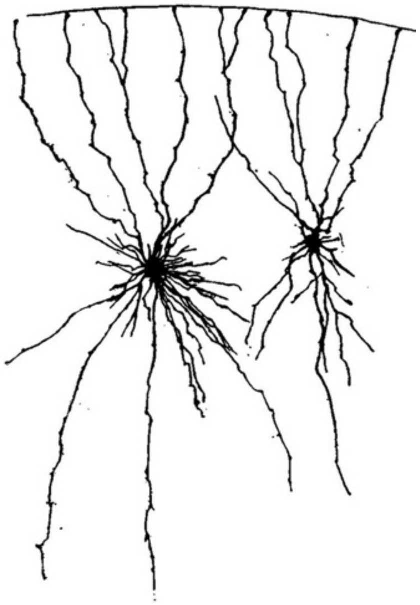

Figure 29*. Support cells of the white matter of the spinal cord of the 30-centimeter-long embryo.*

[1] M. v. Lenhossék, Zur Kenntnis der Neuroglia des menschlichen Rückenmarks. Verhandl. d. anat. Gesellsch., V. Vers. München 1891, p. 193.

232

Their major characteristic is that they have not one single process but several peripheral ones which extend to the pia mater and even sometimes in bundles. The course of these processes is not straight and stretched and they do not extend in a brush like fashion to the rim, but diverge widely in a chandelier-like fashion. Most of these processes are from glial cells in the region of the rolled-in ventral roots facing the frontal fissure. Here the support cells show already features which are similar to the later stages. There is, by the way, a large variety of shapes. The cell body is at this stage in most cases still elongated but often in a transverse orientation, bent in a bow form, representing the peak of the bow formed by the peripheral ramification.

This **radial support system** as described above i**s only an embryonic appearance in humans and in higher mammals**. I observed this type of arrangement in an 18-cm-long embryo; however, from this point onward, a substantial metamorphosis begins, with the radial type gradually being replaced by the later arrangement. In preparations from a 23-cm-long embryo, the support cells of the grey matter already exhibit a different imprint. The rudimentary secondary twigs from the earlier period now develop significantly in both number and length. The cells increasingly resemble typical spider cells, although they have not yet developed the highly complex ramifications seen later. Only occasionally are cells found in the grey matter with a long radial process, as seen in earlier development. For most cells, this process has disappeared, and the secondary filaments protruding from the cell body have become dominant, transforming the cell body from its earlier elongated form into a star-like shape. The radial support cells are gradually replaced by spider cells, representing another type, which are now numerous in the grey matter and partially present in the white matter. In the white matter, many (though not all) support cells retain their simple structure to some degree, still possessing processes that project radially to the periphery of the spinal cord.

233

One can now ask the question how these new cells originate.

A large part of the spider cells can be, without question, ascribed to the embryonic radial support cells. One can assume that the radial cells convert to spider cells by further developing the fine, secondary filaments generated in the first phases while its original large peripheral process undergoes atrophy, which could be observed in some

preparations. This seems to be the original typical generation of these spider cells. Hence, the astrocytes, which develop in this fashion, undergo three developmental stages. First, as a stage of the ependymal cell, then by eccentric dislocation of the cell body the stage of radial astroblasts and from this stage they develop finally into spider cells by the atrophy of the process.

Does this developmental sequence refer to all spider cells? Partially by direct observation, partially by different considerations, I came to the conclusion that this **developmental sequence does not apply for all astrocytes**, at least in human. There is insufficient continuity between the first, radial stage, and the second, spider cell stage. One might expect to observe a gradual transition, with a reduction of the peripheral process and an increasing transformation into spider cells. However, this is not the case. Spider cells appear almost instantly in embryos approximately 20 cm in length, already displaying their characteristic form. They first appear in the *substantia gelatinosa centralis* and later in more peripheral regions, with many showing no indication of having developed from radial cells. Additionally, the number of spider cells in the human spinal cord is far too large to have descended from the earlier radial cells, which are much less frequent.

I would like to propose that for many of the **spider cells, in particular those in the grey matter, the complicated developmental mode has been cainogenetically** [19#] **replaced by a much shorter, simpler** in which they do not pass the radial fiber stage. In contrast, they develop in the inner layers of the cord or in the more peripheral regions where they are later found - from germ cells as small, in the beginning process-less cells, transforming into typical

astrocytes surrounded by many processes [1)]. They do not develop the strong peripheral process, which is doomed anyway. This mode of generation of the spider cells corresponds more to the view of earlier researchers like Boll, Vignal, Gierke and recently Kölliker while the latter deduces these elements from undifferentiated cells of the cord Anlage.

No matter whether the astrocytes develop in the one or the other mode, **there is no difference between them in the outer appearance** and what the major point is: **all are from the same descendants**. Both forms, those developing from the radial cells and those directly originating, are ectodermal elements. Both are descendants of germ cells which

are belonging to the cord and can be traced back to germ cells which are part of the medullar plate, the outer blastodermic layer. The direct formation is only a shortcut of the indirect.

In the **last months of the intrauterine period**, the features of the astrocytes change only little. The essential point is that, compared to the expansion of the cord, they lag in size, which causes them to appear progressively smaller. They most likely increase in number during this final phase, but this can only be confirmed through studies of mitosis in the cord during its last developmental stages. However, the increase in the spinal cord's diameter can also be attributed to other factors, such as the growth of nerve cell substance and the development of the myelin sheath. Another observation regarding astrocytes during the final months of development is the continuous expansion of their processes.

[1)] To decide between these alternatives, one had to study the spinal cord of higher mammals at the later stages of their development with respect to mitosis and their positions. The research is still lacking.

235

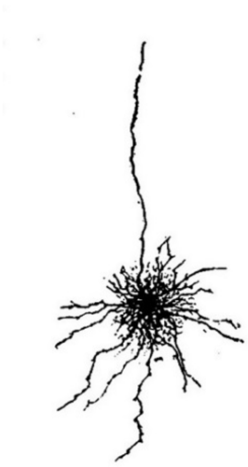

***Figure 30**. Kurzstrahler with still existing peripheral process, from the spinal cord of a 30 cm long embryo.*

According to my opinion all elements of the support system, in particular all astrocytes, have their origin from the ectoderm. I am in contradiction with earlier, maybe today no longer supported reports by R. y Cajal who considered that in addition to the ectodermal Anlage of the neuroglia at later stages a small portion of mesodermal components are added in the form of glial cells which are from a different type and, maybe, of connective tissue nature, and it may be that they are emigrating leucocytes or endothelial cells detached from the blood vessels. I have not found any evidence to support this view. Since all Langstrahler are generally of the same phenotype and since one can follow their development from the ectodermal ependymal cells step by step in some cases, there is no reason to believe that the others are of a fundamentally different nature. One could at most think of Kurzstrahler which have a distinct habitus, but against that (that they are of different origin) speaks the finding also from Kölliker that there are many transitional forms relative to the Langstrahler. Even more convincing is my frequent observation that these cells have early in development a peripheral process (figure 30) which supports their origin from the ectoderm.

Considering that all supportive cells originate from the ectoderm, that they are the fundamental elements of the medullar tube, so there is still an open question whether other forms of true connective tissue enter the spinal cord, except for the blood vessels. I would remind in this context on the many reports about pial processes invading from the periphery. As already alluded above, one has an impression that all these descriptions are based on illusion.

236

The “pial septa” are nothing more than a complex of spider cells and their processes, arranged as rough bundles. The peridym, i.e., the surface rim layer of the cord, is not composed of pial tissue but consists of an entanglement of tangential spider cells. The strong separation of the peridym from the pia mater is most clearly observed using the method introduced by Schaffer, which involves double-labeling with hematoxylin and eosin according to a defined recipe. All neuroglial elements, including the peridym, are well labeled by the red eosin stain in these preparations, whereas the pia mater, composed of connective tissue, appears brownish and is distinctly separated from the peridym. Additionally, the blood vessels, including their adventitia, are clearly distinct from the medulla rim zone due to the differential staining. When a blood vessel is cut tangentially, such that only the adventitia and not the lumen is visible in the preparation, it may give the impression of a pial insertion. Serial sections, as studied by Schaffer, correct this misinterpretation. However, Schaffer notes that, in very rare cases, he has observed small connective tissue insertions that were not associated with blood vessels. Nonetheless, this condition can be disregarded, if it is indeed true, as it is an extremely rare event and need not be considered. It may be explained as remnants of the adventitia of blood vessels undergoing regressive degeneration.

Since this is in many aspects, particularly for pathology, an important issue, and to get more certainty, I have used the modification of the chrome silver method by which Oppel [1] stained the delicate connective tissue of liver, spleen and lymphatic glands and I obtained only negative results. Nowhere in the spinal cord, except for the blood vessels, I found connective tissue labelling, even though this is a negative and maybe due to technical problems not exclusive result, so it is just an additional support.

[1] A. Oppel, Eine Methode zur Darstellung feinerer Strukturverhältnisse der Leber. Anat. Anz., Jahrg. V, 1890, p. 143.

237

I would like to add a word about the adventitia of the blood vessels penetrating the spinal cord. It is not present at the capillaries and also at the stronger trunks it plays a minor role. The most important is: there is no connection with the genuine spinal cord substance, with the neuroglia, since this is completely separated from the blood vessels.

With all decidedness I would like to **take position** alike von Kölliker **against a dualistic concept of neuroglia**. In my conviction, based on the images by Golgi and others, we should not hesitate to **negate the question** whether there is a connective tissue in the spinal cord, of course apart from the blood vessels penetrating the cord. The earlier reports about the penetration of connective tissue from the pia mater are based on a mix-up with glial fibers as Boll has recognized earlier. All processes in the support substance of the cord are processes of astrocytes; the spinal cord is a genuine ectodermal organ which provides also an intrinsic, internal support system and which only relies on the canal system including its content of course, providing nutrition as help from outside.

While we have a clear opinion on the above, another, more concerning question arises: the existence or non-existence of a “basic substance,” described by earlier researchers such as Boll and Gierke as a granular, net-shaped, or even homogeneous mass. We can no longer support this, as our new methods demonstrate that regions previously described as containing a structureless mass now reveal a richly organized arrangement of fibers. Therefore, one must be very skeptical of these earlier reports. The hypothesis of a basic substance cannot be regarded as a valid postulate, given the extensive branching of the nervous elements and the felting formed by astrocytes, as demonstrated in Golgi preparations. We can explain the composition of the cord without invoking a connecting mass, relying instead on the felting of fibers, similar to felted hair, and, at most, a fluid filling tiny potential spaces.

238

After this condensed report I would like to take a look at the arrangement of support cells in the spinal cord of other mammals and vertebrates as studied so far. Three important sentences can be deduced from the insights obtained in this area:

1. The support system of the cord consists of cells, of ependymal cells and more or less branched support cells (glial cells), and these elements are all of ectodermal origin similar to nerve cells and generated in the spinal cord itself.
2. The arrangement of these support cells shows differences in different animals as long as one only looks at the fully developed cord in higher and lower vertebrates. But these differences have natural explanations if one goes back into the developmental processes of the cord. There is a highly interesting fact that the simpler type of support system of lower vertebrates shows similar conditions as that of higher and highest forms as they pass transient embryonal stages. Thus, the arrangement of the support cells in the cord also convincingly shows that there is a common organisation of the vertebrates and that phylogenetic developmental stages in the individual development of higher forms are repetitions.

The characteristics of the support system in the spinal cord of amphioxus [18#] are highly interesting. As first reported by Nansen[1)] and Rohde [2)], and partially confirmed by my observations using the Golgi method, the entire support system is composed of ependymal fibers that extend radially from the central canal to the surface. I observed that the ependymal fibers are rough and undivided. Due to the absence of proper glial cells, amphioxus represents a stage akin to the earliest developmental phase observed in vertebrates, where this condition is permanent. Furthermore, v. Kölliker provided a similar description of the support cells in the spinal cord of *amphioxus*.

In the spinal cord of the cyclostomes [17#], the support cells were first described by Nansen, subsequently by Retzius [3)] using the Golgi method and the reports and figures of both researchers are based on myxini [13#]. In the first edition of this work, I contributed an image of neuroglia form the spinal cord of the lamprey (see enclosed attached figure 31).

[1)] Fr. Nansen, Structure and Combination of the Histiological Elements of the Central Nervous System. Bergen's Museums Aarsberetning for 1886 Bergen 1887, p. 160.
[2)] E. Rohde, Histologische Untersuchungen über das Nervensystem vom Amphioxus lanceolatus. Schneider's Zoolog. Beitg., Bd. 2., H. 2, Breslau 1888.
[03)] G. Retzius, Zur Kenntnis des centralen Nervensystems von Myxine glutinosa. Biolog. Untersuchungen, N. F. II, Stockholm 1891, p. 51.

239

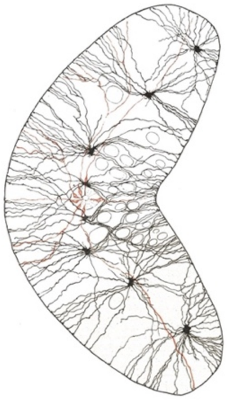

Figure 31*. Spinal cord of Petromyzon with impregnated support cells. Ependymal cells red, astrocytes black.*

240

The ependymal cells appear smooth, delicate, and very sparse. In contrast, there are many astrocytes; however, these are not evenly distributed across the entire cross-section, as their cell bodies are primarily located in the band-like grey matter. They are characterized by extensive branching. Each cell extends into the ventral and dorsal areas of the cord, forming a meshwork of branches, with the most lateral ones reaching the edge of the cord. Most branches extend to the periphery, terminating in small bulbs. The medial branches of cells positioned on either side of the midline cross either in front of or behind the central canal. This description was recently provided by Retzius [1)]. In Myxine, there appears to be a slight difference. According to Nansen and Retzius, the astrocytes extend their processes to only one side of the cord, either dorsal or lateral, with only a few exhibiting bilateral branching as seen in Petromyzon.

Among the Selachier [16#] I succeeded to depict glia with the Golgi method in Acanthias [20#], Scyllia [21# 2)] and in Raja [22# 3)].

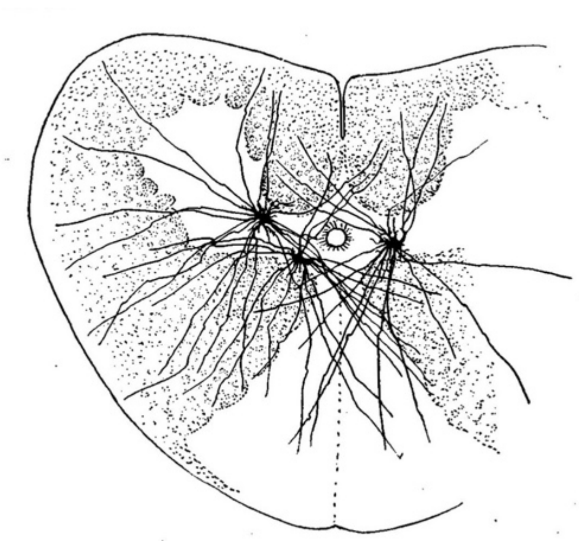

***Figure 32**. shows some stained astrocytes in the spinal cord of a 16-cm-long embryo of acanthias.*

1) G. Retzius, Studien über Ependym und Neuroglia. Biolog. Untersuchungen, N. F., V, 1893, p. 16.

2) M. v. Lenhossék, Beobachtungen an den Spinalganglien und dem Rückenmarke von Pristiurusembryonen. Anat. Anz., Jahrg. VII, 1892, p. 536.

3) M. v. Lenhossék, Zur Kenntnis des Rückenmarkes der Rochen. In: Beitr. z. Histolog. d. Nervensystems u. d. Sinnesorgane. Wiesbaden 1894, p. 60.

241

They show a high similarity with the analogue elements of the spinal cord of Petromyzon. Also here is the strangely formed grey matter the main region of the cell bodies of the astrocytes; also here are the elements characterized by their long, rigid branches which extend in all directions and cross before and behind the central canal. However, they seem not to penetrate to the surface, but close by as they terminate beneath the powerfully developed superficial meshwork of dendrites. I could not visualize the ependymal cells in Acanthias but I could stain them in Raja where I found them as delicate, unbranched, sparse fibers reaching up to the surface. The glial cells proper in Raja present themselves in a form, which is completely distinct from Acanthias and also Scyllium, but rather similar to the cells in amphibia: they are similar to the “astroblasts” of the mammals and birds, as longitudinal cells provided with a fine fluff extending outwards into a single rough peripheral process.

We know little about the support cells of the spinal cord of bonefish. The only statement based on Golgi staining is found in the important neuroglial report by Retzius (Biolog. Unters. V., p. 18), yet also these are less related to the fully developed by rather to the still developing spinal cord. Strangely plump and furry appear the ependymal cells, the few glial cells which can be seen in the images from Retzius and they do not represent the astrocytes but rather look like pressed out ependymal cells. Should these images really represent the final stage of the spinal cord of Teleosts[28#], what I doubt, would it be of a type which is deep below Selachier or even the cyclostomes.

The neuroglia of the spinal cord of the Batrachia [22#] has been earlier described by von Lawdowsky [1)] and recently by Cl. Sala [2)], a student of Cajal, and also by Retzius in a detailed fashion. I can support the description by Sala of the developed spinal cord based on my own preparations. Remarkable are the rough and furry bodies of the ependymal fibers including the fact that they are highly branched. These features are found in the juvenile forms, it seems however that later the lateral ependymal fibers show a similar atropic appearance like in higher vertebrates. The support cells, which could be demonstrated by Sala and Retzius including those, which I could see in my preparations, are not true spider cells but rather pressed out ependymal cells. The cell bodies appear very plump and are equipped with only few secondary branches, yet with a very strong peripheral process which – and this is characteristic for the support cells of the spinal cord of the amphibia - branches in the grey matter in a wide brush of rough processes. The latter penetrate the white matter in a radial fashion, to end at the outer surface, each with a cone-like bulb, which results that the white matter appear stained in a stripe-like manner.

1) M. Lawdowsky, Vom Aufbau des Rückenmarks. Arch. f. mikrosk. Anatomie. Bd. 38, 1891, p. 264.
2) Cl. Sala y Pons, Estructura de la Médula espinal de los Batracios. Barcelona 1892.

However, I would like to consider that it is probable in accordance with Kölliker [1] that beside those cells, there are also real astrocytes as described by Lawdowsky, which did not label in my preparations as well as in Sala's. Because I assume in concordance with Kölliker that one can observe nuclei of glia in the white matter preparations.

With respect to reptiles there is a precise description by R. y Cajal [2]. Despite of the fact that only adult animals were investigated, it was obvious that the support cells of the spinal cord are still in a strange embryonic stage. The ependymal cells seem not to undergo any atrophy; they send their process also in the adult stage to the pia mater. They were obviously no proper astrocytes but only cells, which have one or more radial processes terminating to the surface and thus represent emigrated ependymal cells, similar to astroblasts of mammals. In the white matter the ependymal fibers decompose, similar as the radial processes of the glial cells, into fascicules ("penacho periferico") of diverging branches projecting to the surface.

The development of support cells in the spinal cord of birds has been well studied by several groups. However, the adult stage of glia has not been well studied using the Golgi method, making it difficult to determine whether they represent a more advanced stage compared to the spinal cord of reptiles or if they remain of the embryonic type. In particular, it remains unclear whether typical spider cells, like those in mammals, are present. In some preparations of 15-day-old chickens, I may have observed real astrocytes, but the images are not entirely convincing.

Regarding mammals, I can confine myself to the observation that similar relations exist as I have reported for humans, both in terms of development and in adulthood. This is at least evident in my preparations from rodents, carnivores, and oxen. It should, however, be emphasized that the smaller the animal and its spinal cord, the more robust but also sparser the spider cells appear in relation to the cross-section, with their processes being rougher. Nevertheless, I cannot support Gierke's view, particularly regarding the cortex, that glia are better developed at the expense of nerve elements in mammals compared to humans, as the astrocytes gain in size and the extent of their projections in mammals is offset by their reduced numbers.

1) A. v. Kölliker, Handb. d. Gewebelehre, 6. Aufl., Bd. II, 1893, p. 189.

2) S. R. y Cajal, Pequeñas comunicaciones al conocimiento del sistema nervioso. La médula espinal de los reptiles. Barcelona 1891, p. 48.

In conclusion, I must add some general remarks about the role these support cells play in the pathology of the spinal cord. There is likely no disease of the spinal cord that does not affect the "glia." Not only are they affected secondarily by alterations in the nervous elements, but they can also be the primary source of pathological changes. The latter are always associated with a marked increase in the support system. If this manifests as tumors characterized by uncontrolled growth, a glioma results. If this proliferation remains within certain boundaries, while simultaneously causing early disintegration of the newly formed support tissue (syringomyelia), it results in a form recently described by Hofmann [1)] as gliomatosis or primary gliosis, distinct from true glioma.

The glioma, as first described by Golgi [2)] and Simon [3)], consist of similar highly branched spider cells as we know them from astrocytes of the normal support system and thus one can describe these tumors also as astroms. That normal astrocytes and glioma are fairly similar cellular elements can be deduced by their very similar morphological appearance. However, another question is whether the glioma really originate from the existing, typical astrocytes, i.e. from the fully developed spider cells. It is difficult to imagine that such highly differentiated elements like the astrocytes, with a protoplasm, which is reduced to a minimum, and with the largest part of their cell bodies appearing converted into fibrous elements, should retain the ability to divide. What should happen during that process with the processes? To my knowledge, histology does not show any example that such strangely metamorphosed elements can still divide.

[1)] J. Hoffmann, Zur Lehre von der Syringomyelie. Deutsche Zeitschr. f. Nervenheilkunde, Bd. 8, 1893, p. 1.
[2)] C. Golgi, Sui gliomi del cervello. Rivista sperim. di Freniatria, 1872. S. Sammelwerk, p. 56, "Über die Gliome des Gehirns".
[3)] Th. Simon, Das Spinnenzellen- und Pinselzellengliom. Archiv für pathol. Anat. u. Physiol. 1874.

This consideration, without having any positive histological finding, leads me to the hypothesis that beside the fully differentiated spider cells in the central nervous system, there are additionally **agenetic glial cells, which throughout life remain in their original plain condition**, basically not used rests from early development. Might they perhaps be the elements, which get into a proliferation status by so far unknown stimuli and lead to the formation of the glioma? This hypothesis is of course very difficult to substantiate or to rebut, since still we have no method that reliably stains all support cells of a slice with all its processes so that one could state that the process-less appearing support cells within a slice are really process-less.

In any case, this hypothesis aligns with the view of most pathologists, who consider the origin of all glioma tumors as developmental disturbances and assume that gliomas arise from the inhibition of early developmental steps. Accordingly, the hypothesis is as follows: If any obstacle occurs during the development of the spinal cord, the support cells at that location fail to acquire their normal morphology, remaining in their embryonic rudimentary stage and retaining the capacity for disease-related unlimited proliferation.

Investigations by Hoffmann (a. a. O.), Oppenheim [1)], Reymond [2)], and others indicate that the preferred location of these primary gliomas is the dorsal part of the spinal cord, particularly the posterior midline. This region, in the young embryo, represents the dorsal part of the central canal, which initially extends backward as a slit and later closes. This fact can be easily explained

245

since at this location due to the obliteration of the central canal, there are highly complex events occurring during the development of the support cells and it can be assumed that a disturbance of the normal developmental process could fairly easily take place.

However, not all gliomas are true astrocytomas, i.e., not all are composed of spider cells. Some gliomas consist of soft, small, process-less elements. These forms have been described as gliosarcoma, a term against which we must take a firm stand from a histogenetic perspective. Since all “glia” we have observed originate from the ectoderm, and sarcomas arise from connective tissue tumors, it is evident that a “sarcoma” cannot simultaneously be a glioma. Thus, if such tumors originate from glial cells, as is most likely, they must be considered gliomas regardless of the appearance of the cells. If, instead, they arise from a connective tissue source, such as the adventitia of the medullar blood vessels or the meninges, they should be classified as true sarcomas. It is conceivable that both events occur in parallel—tumor formation from glia and connective tissue—which then intermingle, forming mixed tumors that could be termed gliosarcomas. However, such combined forms have not yet been histologically verified, and the term gliosarcoma was not originally intended to describe this phenomenon. Gliosarcomas most likely originate from the glial floor, where newly formed elements fail to transform into astrocytes and instead remain entirely or partially as process-less germ cells.

Certainly, it may not be easy in all cases to clearly demarcate the above-described spontaneous glial tumors from other forms of glial proliferation, which are not primary but a response to diseases and degeneration of the nervous elements of the cord. It is strange how strongly sensitive in this regard the responses of these spider cells are [1)].

[1)] See the recently published paper by Nissl: Mitteilungen über Karyokinese im centralen Nervensystem. Allg. Zeitschr. f. Psychiatrie 1894, as well as: Über eine neue Untersuchungsmethode der Centralorgane, speziell zur Feststellung der Lokalisation der Nervenzellen. Centralblatt f. Nervenheilkunde und Psychiatrie, Bd. XVII, 1894, p. 337.246

246

Wherever atrophy occurs in the spinal cord, such as degenerative depletion of nervous tissue in the nerve cells or fibers, progressive changes in the support cells compensate for these regressive events. These progressive events are only partially due to the proliferation of glial elements [1)] (considering the rudimentary support cells mentioned above). The primary contribution is an increase in the volume of cell bodies and, particularly, the thickening of glial fibers, i.e., the processes of astrocytes. They now appear rough and dark, possibly due to hydropic [23#] swelling or the assimilation of new substances. They are more visible in slices under microscopic inspection, even with inadequate staining. Their structure is distinct from the rest of the cross-section, characterized by a condensed, dark, and sclerotic appearance. I assume that in many cases where primary glial proliferation was presumed, it was actually a secondary event—a compensatory thickening of spider cells caused by the disintegration of nervous tissue. Remarkably, the response of astrocytes to localized disease in nervous elements is not confined to the affected area but extends diffusely into healthy nervous tissue. It appears that the trigger for this response radiates outward from the primary disease site.

1) Nissl could recently demonstrate that pathologic processes can also be accompanied by a true division of the support elements as described in the above-mentioned publication on the cortex of paralytics, demented and alcoholics. It is curious that the divisions (mitosis) can hardly be established by conventional methods, but the detection requires a special procedure described by Weigert. This consists of the following: Fixation in alcohol, embedding in celloidin or attachment to rubber without embedding in celloidin. The slices will be first incubated for half an hour in Tincture ferri acetici Rademacherie, then after washing with water into Weigert´s

hematoxyline (hematoxyline 1, alcohol 10, water 90) where they remain for ½ hour. Superficial washing and rapid differentiation in 1 HCl: 100 70% alcohol. Subsequently water, dehydration, brightening, embedding in balsam. Prior to Nissl, Fürstner and Knoblauch (Über Faserschwund in der grauen Substanz und über Kernteilungsvorgänge im Rückenmarke unter pathologischen Verhältnissen. Arch. f. Psychiatrie, Bd. XXII, 1891, p. 135) have already demonstrated that after experimental lesioning of the spinal cord, glial cells undergo processes of mitotic amplification.

247

These progressive changes in the support tissue are most visible at the superficial margin of the spinal cord glia, known as the peridym. In almost all chronic spinal cord diseases, the peridym appears moderately to significantly enlarged, particularly in the lateral cord region, sometimes to the extent that the spinal cord seems encased by a wide, closed rim of felted astrocytes. This thickening of the peridym is often described as rim degeneration. This enlargement of the glial rim can result from degenerative processes affecting the nerve fibers of the lateral cord or from atrophic conditions of the nerve cells in the grey matter. I recently analyzed a comprehensive collection of pathological spinal cord preparations and found that peridym thickening was absent only in cases of acute spinal cord conditions such as myelitis. Notably, I observed a pronounced case where all lateral bands appeared normal, but there was obvious chronic pigment atrophy in the cells of the anterior horns. In all spinal cord degenerations, whether in cases of multiple sclerosis or glioma cores, the peridym undergoes progressive changes. This also occurs in the degeneration of the dorsal columns, the anatomical substrate of tabes. I believe that this “peripheral sclerosis,” often described as a feature of tabes (illustrated, for example, in *Pathologische Anatomie* by E. Ziegler, 6th edition, vol. II, Fig. 156, p. 289, reproduced by Westphal), is simply a compensatory increase in the peridym due to the degeneration of the dorsal columns.

TABLES

Table 1. *Supporting cells (astrocytes) from the spinal cord of a year-old child, stained using the Golgi method.*

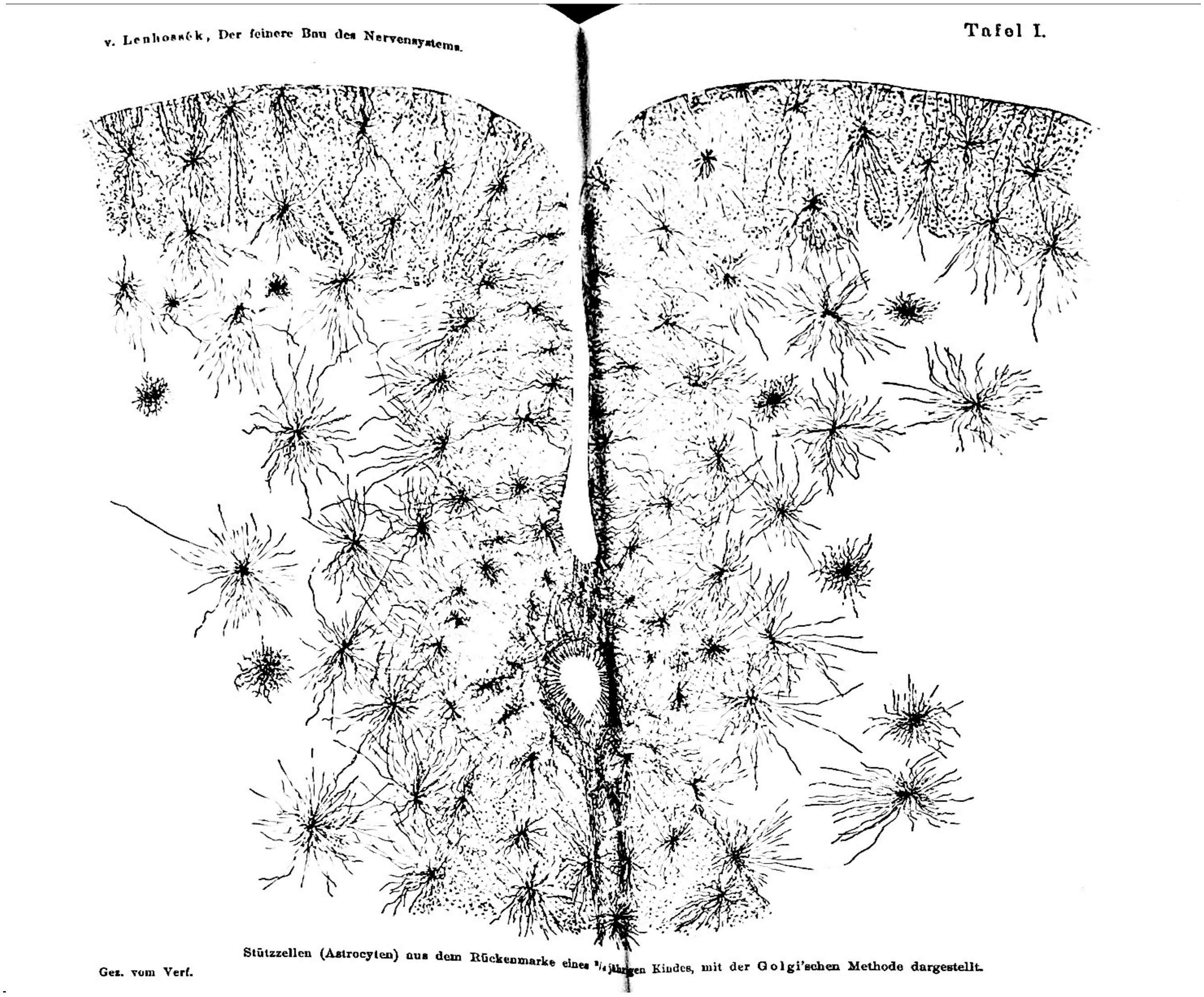

Table 2. *Spinal cord of a 14 cm long human embryo stained with the Golgi method, with labelled supporting cells. Left: Ependymal framework, right: precursors of spider cells (astroblasts).*

v. Lenhossék, Der feinere Bau des Nervensystems.

Tafel II.

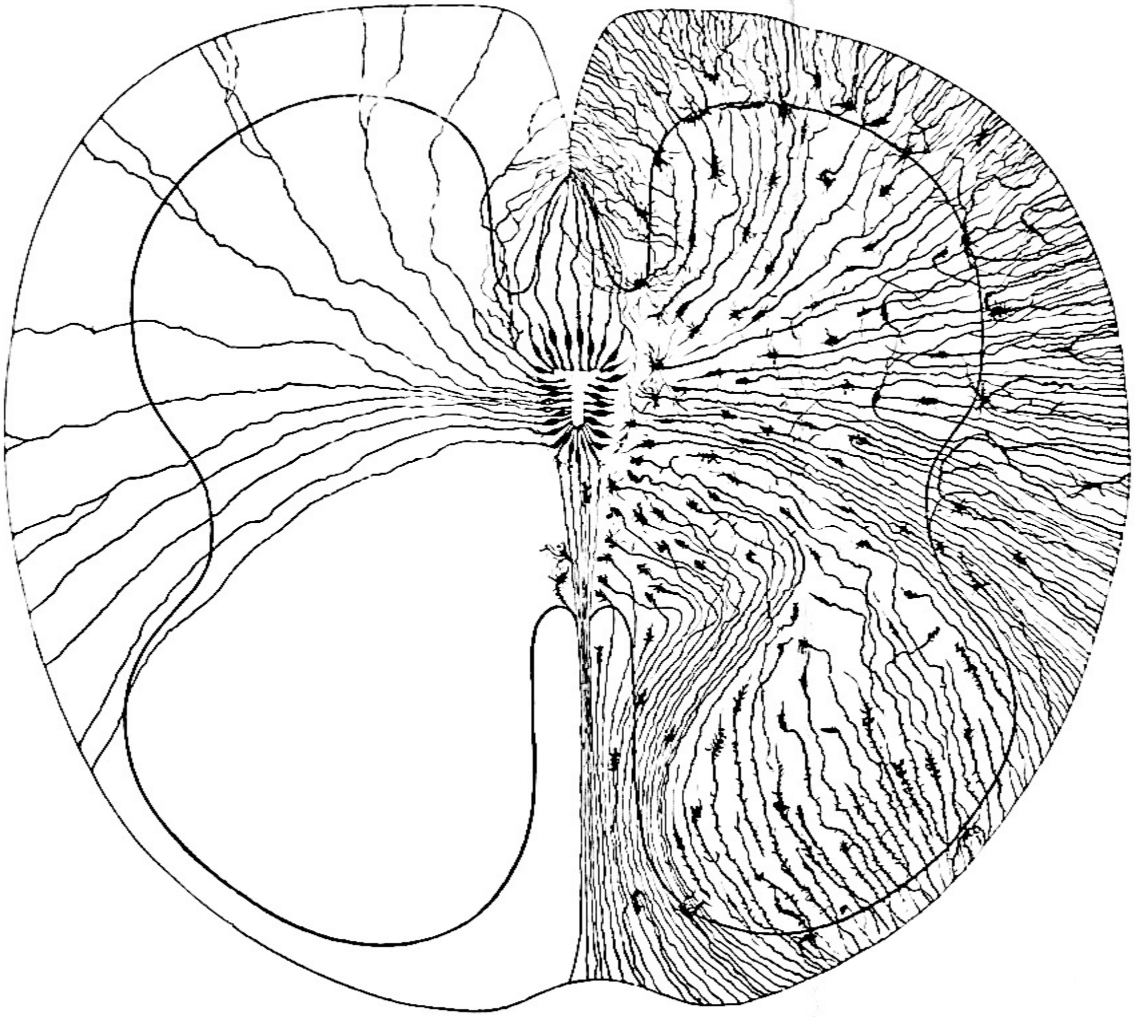

Rückenmark eines 14 cm langen menschlichen Embryos, nach Golgi behandelt, mit imprägnierten Stützzellen
Links Ependymgerüst, rechts Vorläufer der Spinnenzellen (Astroblasten).

Gez. vom Verf.

Comments

[1#] With a grain of salt
suggests to view something, specifically claims that may be misleading or unverified, with skepticism

[2#] Original German term: Pinselzellen

[3#] Here he used the term neurocytes for neurons

[4#] Latin: a necessary condition

[5#] Sharpey's fibres (bone fibres, or perforating fibres) are a matrix of connective tissue consisting of bundles of strong predominantly type I collagen fibres connecting periosteum to bone.

[6#] The German word ´Linse´can either mean lense or lentil

[7#] The ? is in the original text

[8#] The posterolateral tract (fasciculus of Lissauer, Lissauer's tract, tract of Lissauer, dorsolateral fasciculus, dorsolateral tract, zone of Lissauer) is a small strand situated in relation to the tip of the posterior column close to the entrance of the posterior nerve roots.

[9#] The adventitia is the outer layer of fibrous connective tissue surrounding an organ.
The outer layer of connective tissue that surrounds an artery, or vein – the tunica externa, is also called the tunica adventitia.

[10#] An intumescent is a substance that swells as a result of heat exposure, leading to an increase in volume and decrease in density. Intumescence refers to the process of swelling

[11#] Lendenanschwellung (Swelling of loins). The loins, or lumbus, are the sides between the lower ribs and pelvis, and the lower part of the back.

[12#] Original German term: Deiters´sche Pinselzellen

[13#] Myxine is a genus of hagfish of the class Myxini (also known as Hyperotreti) and order Myxiniformes are eel-shaped, slime-producing marine fish (occasionally called slime eels)

[14#] Latin term meaning Primary membrane

15# We today know that these cell bodies moves up and down with each division. Not much of a surprise that you find them at different locations at a given time point

16# Modern sharks are classified within the clade Selachimorpha (or Selachii)

17# Cyclostomata like the sea lamprey (Petromyzon marinus) is a parasitic lamprey native to the Northern Hemisphere. It is sometimes referred to as the "vampire fish".

18# Amphioxus is a previous name for the genus Branchiostoma, common name lancelet

19# Caenogenesis describes the development of structures and organs in an immature form of an animal that are adaptations to its way of life and are not retained in the adult form

20# The spiny dogfish (Squalus acanthias), spurdog, mud shark, or piked dogfish is one of the best known species of the Squalidae (dogfish) family of sharks, which is part of the Squaliformes order

21# Old name for the zebra shark (Stegostoma tigrinum) It is a species of carpet shark and the sole member of the family Stegostomatidae.

22# The Batrachia are a clade of amphibians that includes frogs and salamanders

23# Hydropic: characterized by swelling and taking up of fluid
