# Supplementary material for: The Concept of Neuroglia ‐ the State of the Art Circa 1900
Source: Glia. 2025 Feb 4;73(5):890–904. doi: 10.1002/glia.24678 (PMC11920685; doi:10.1002/glia.24678)
Supplement: Supplementary file 7 — Data S7. Scanned original publication by Weigert. [file GLIA-73-890-s008.pdf]

Festschrift

zum

fünfzigjährigen Jubiläum des ärztlichen Vereins

zu

Frankfurt a. M.

3. November 1895.

Beiträge

zur

Kenntnis der normalen menschlichen Neuroglia

von

Carl Weigert.

Mit XIII Tafeln.

Festschrift

zum

fünfzigjährigen Jubiläum des ärztlichen Vereins

zu

Frankfurt a. M.

3. November 1895.

Erscheint gleichzeitig als Heft II von Band XIX der „Abhandlungen, herausgegeben von der Senckenbergischen naturforschenden Gesellschaft.“ Commissionsverlag von Moritz Diesterweg.

Vorrede.

> Det gik mig som En, der vandrer i en Labyrinth. Gang for Gang havde jeg fundet, og jeg stod nu lige ved det Sted, hvor den endte. Men her fandtes en Muur, hvis Dör ingen Nögle formaatte at aabne, og som jeg med mine Kräfter var for svag til at spränge. Hvergang jeg tog et nyt Udgangspunkt, kom jeg dog tilbage til den samme uoverstigelige Muur. Indenfor den laa Skatten, men jeg syntes ikke at väre den, der skulde häve den.
>
> Vilhelm Bergsöe: Fra den gamle Fabrik.
> Anden Deel S. 82.

Die folgende Arbeit beruht auf Resultaten, die durch eine neue Methode gewonnen worden sind. Um die Methode auch nur auf den jetzigen Standpunkt zu bringen, dazu habe ich vom 5. Dezember 1888 bis heute gearbeitet, und zwar habe ich alle meine von Amtsgeschäften freie Zeit so gut wie ausschliefslich dieser Methode gewidmet.

Es war noch in den ersten Jahren dieser Arbeitsperiode, da besuchte ich einen als Lehrer, Forscher und Mensch gleich hochstehenden Gelehrten und erzählte ihm, dafs ich mit meiner Arbeit gar nicht zu Ende kommen könnte. Im Laufe des Gesprächs sagte der verehrte Gelehrte ungefähr folgendes zu mir: „Haben Sie wohl einmal darüber nachgedacht, warum wir Theoretiker eigentlich wissenschaftlich arbeiten? Gewinn haben wir ja nicht davon; denn für die gröfsten Entdeckungen auf unsern Gebieten wird kein Pfennig bezahlt, und dafs das wissenschaftliche Arbeiten für die Carrière nichts nützt, haben Sie ja selbst erfahren. Also warum arbeiten wir? Wegen des Ruhms? Der Ruhm ist fadenscheiniger und vergänglicher, als Spinnengewebe, und man könnte in unserer schnelllebigen Zeit das Wort des

Baccalaureus im Faust getrost dahin abändern, dafs man sagte: Bist du auch kurze Zeit berühmt gewesen, bald weifs kein Mensch mehr was von dir zu sagen. Warum arbeiten wir nun aber doch? Einfach deshalb, weil uns das wissenschaftliche Forschen eine hohe und reine Freude bereitet."

Wenn dies Motiv mein langes Arbeiten veranlafst hätte, dann hätte ich jetzt fast sieben recht glückliche Jahre hinter mir, — aber leider war es ganz anders. Wenn ich die allererste Zeit abrechne, in der ich ein neuentdecktes fruchtbares Gebiet vor mir zu sehen glaubte, und in der ich meinte, nur die Hand ausstrecken zu brauchen, um dies Gebiet zu besitzen, — wenn ich diese kurze Spanne Zeit abrechne, so war das Arbeiten an der neuen Methode gerade das Gegenteil von Vergnügen und von Freude. Es war eine Kette von immer neuen Hoffnungen und immer neuen Enttäuschungen, eine Kette von immerwährenden quälenden Geduldsproben. Mufste ich doch zu Zeiten Wochen lang, immer aber viele Tage lang warten, ehe ich wissen konnte, ob ein neuer Versuch geglückt wäre, oder nicht.

Wie einem in solcher Zeit zu Mute ist, das hat ein dänischer Naturforscher, der zugleich ein ganz hervorragender Poet ist, ausgezeichnet geschildert. Ich meine Vilhelm Bergsöe. Dieser erzählt in seinem Romane „Fra den gamle Fabrik" (Aus der alten Fabrik) die Leiden eines Chemikers, der einer Entdeckung auf der Spur ist, aber über das „nästen", das „beinahe", nicht herauskommt. Für meine hochgeschätzten skandinavischen Freunde habe ich einen hierauf bezüglichen Passus in der Ursprache an die Spitze dieser Vorrede gestellt, hier mag dessen deutsche Übersetzung folgen:

> „Es ging mir", sagt Olsen, der Chemiker in jenem Roman, „wie einem, der in einem Labyrinthe wandelt. Gang für Gang hatte ich gefunden, und ich stand nun an der Stelle, wo es zu Ende war. Aber hier befand sich eine Mauer, deren Thür kein Schlüssel zu öffnen vermochte, und die zu sprengen meine Kräfte zu schwach waren. Jedesmal, wenn ich wieder einen neuen Ausgangspunkt nahm, kam ich doch zu derselben unübersteiglichen Mauer zurück. Innerhalb derselben lag der Schatz, aber ich schien nicht der zu sein, der ihn heben sollte."

Ich habe mir in dem dänischen Citate, wie in der Übersetzung, erlaubt, die Praesentia der Verba des Originals in die Praeterita zu verwandeln. Ob ich ein Recht dazu habe, das müssen die entscheiden, die die neue Methode versuchen werden.

Warum habe ich aber dann doch weiter gearbeitet, wenn das Arbeiten an der Methode so unerquicklich war? Warum habe ich dem Rate meiner Freunde nicht gefolgt und etwas „lohnenderes" vorgenommen? Nun, ich konnte einfach nicht loskommen. Die Arbeit hatte noch etwas besonders tückisches an sich. Immer stand ich zwar vor dem abscheulichen „beinahe", aber immer glaubte ich, der nächste Versuch müsse gelingen, — wieder ganz so, wie es Bergsöe bei seinem Chemiker (Bd. II S. 146) schildert. Nur Tage oder Wochen schien es, und die lange Arbeit ist belohnt! Aber aus Tagen und Wochen wurden Monate, aus Monaten viele Jahre, die Zeit verging, ohne dafs ich es merkte, bis schliefslich ein einigermafsen annehmbarer Erfolg doch noch erreicht war.

Tantae molis erat, aber nicht, Romanam condere gentem, sondern eine simple histologische Methode zu finden. Habe ich da nicht Öl und Zeit verschwendet, habe ich da nicht meine Arbeitskräfte vergeudet, oder um mit dem Chemiker bei Bergsöe zu reden, „die Goldkörner des Lebens wie Sand verstreut," („Livets Guldkorn jeg spredte som Sand")?

Das wird sich zeigen. Eine neue Methode ist eben ein Schlüssel, um die Thür in der unübersteiglichen Mauer zu öffnen, die die wissenschaftlichen Schätze umschliefst. Der Schlüssel, den ich der wissenschaftlichen Welt übergebe, schliefst zwar nicht ganz leicht, er mufs erst noch sorgfältig abgefeilt werden, aber er schliefst doch, und jedermann kann sich daran machen, die Schätze zu verwerten, von denen ich in diesem Buche nur einige Proben darbringe. Wenn dann (von meinem bescheidenen Anteil abgesehen) recht viel von jenen Schätzen durch diejenigen gehoben wird, welche sich der neuen Methode bedienen werden, dann bin ich vollständig befriedigt, dann sage ich getrost: Oleum et tempus non perdidi.

Freilich weifs ich sehr wohl, dafs es Leute giebt, die die Erfindung einer neuen Methode als eine minderwertige wissenschaftliche Leistung betrachten, und die die Erfinder selbst, so zu sagen, über die Achsel ansehen. Schaut man aber genauer zu, so nehmen diese selben Leute die Methoden der von ihnen so gering geschätzten Erfinder mit dem allergröfsten Eifer zu Hilfe, um ihre eignen wissenschaftlichen Bauten so recht handwerksmäfsig ausführen zu können. Mancher Maurergeselle mag ja auch den Architekten, nach dessen Plänen er arbeitet, deshalb gering achten, weil dieser die Ziegeln nicht selbst übereinander schichtet. Es mufs eben auch solche Käuze geben!

Mit der neuen Methode veröffentliche ich auch eine Reihe von Beobachtungen. Für eine fast siebenjährige Arbeit werden diese manchem vielleicht etwas mager, jedenfalls aber sehr lückenhaft erscheinen. Ich bitte aber zu bedenken, dafs ich bis in die letzte Zeit immer noch mit den Unvollkommenheiten der Methode zu kämpfen hatte, und so lange das der Fall ist, ist der Geist nicht frei genug für eine intensive Thatsachenforschung. So recht konnte ich mich erst seit kurzem der Ernte hingeben, für die ich vor so langer Zeit die Saat ausgeworfen hatte. Unter diesen Umständen wäre es vielleicht besser gewesen, wenn ich das „nonum prematur in annum" buchstäblich befolgt hätte, aber das ging nicht an. Ich hatte mich dazu verpflichtet, diese Arbeit als Jubiläumsschrift für den ärztlichen Verein zu Frankfurt a. M. am 3. November 1895 gedruckt vorzulegen, und da war denn ein weiteres Hinausschieben der Veröffentlichung nicht mehr möglich. So mögen denn die Leser das unfertige und unvollkommene in diesem Buche entschuldigen.

Die Verpflichtung, die ich übernommen hatte, war eine etwas voreilige, aber der Wunsch in dieser Schrift den Frankfurter Kollegen ein Zeichen meiner Dankbarkeit zu überreichen, liefs mich die Schwierigkeiten, die meiner noch harrten, übersehen. Es sind jetzt zehn und ein halbes Jahr her, dafs mir durch die Berufung an das Senckenbergische medi-

zinische Institut nicht nur eine Zufluchtsstätte gewährt, sondern ein geradezu beneidenswertes Feld der Wirksamkeit eröffnet wurde. In dieser ganzen Zeit haben mir die hiesigen Kollegen so viel liebenswürdige Freundlichkeit erwiesen, habe ich durch den Verkehr mit ihnen lernend und lehrend so viel geistige Anregung gehabt, dafs man den Wunsch, zu der Feier des Jubelfestes ihres Vereins etwas beizutragen, wohl verstehen wird. Möge der Geist der Kollegialität und des ernsten wissenschaftlichen Strebens, der vor 50 Jahren eine Anzahl Ärzte zu einem engeren Aneinanderschliefsen zusammen geführt hat, dem ärztlichen Vereine immer treu bleiben, möge er bis in die fernste Zukunft blühen und gedeihen!

Frankfurt am Main.

Dr. Senckenbergisches pathologisch-anatomisches Institut.

Der Verfasser.

Inhaltsverzeichnis.

		Seite
1. Abschnitt:	Historische Übersicht	1
2. Abschnitt:	Die Neurogliafasern in ihrem Verhältnis zu den Zellen	30
3. Abschnitt:	Über die Neuroglianatur der durch die neue Methode gefärbten Fasern	43
4. Abschnitt:	Verhältnis der Neurogliafasern zu etwaigen andern Neurogliasubstanzen und zum Bindegewebe. Chemisches	54
5. Abschnitt:	Besprechung der histogenetischen Stellung der Neuroglia	58
6. Abschnitt:	Anderweitige histologische Eigenschaften der Neurogliafasern	67
7. Abschnitt:	Allgemeine Topographie der Neurogliafasern	72
8. Abschnitt:	Spezielle Topographie der Neurogliafasern	81
	1) Rückenmark	81
	2) Medulla oblongata	98
	3) Pons	103
	4) Pedunculus cerebri	103
	5) Vierhügel	104
	6) Zirbeldrüse	105
	7) Kleinhirn	106
	8) Grosshirn	108
	9) Gyrus hippocampi. Cornu Ammonis	110
	10) Balken und Fornix	114
	11) Opticus und Chiasma	116
	12) Corpora mamillaria	117
	13) Sehhügel	118
	14) Streifenhügel und Kapseln	120
9. Abschnitt:	Die physiologische Bedeutung der Neuroglia	121
10. Abschnitt:	Methode	128
Figurenerklärung		146

Beiträge
zur
Kenntnis der normalen menschlichen Neuroglia.

Von
Prof. Dr. C. Weigert.

---

1. Abschnitt:
Historische Übersicht.

Es giebt eine ganze Menge von Leuten, welche meinen, dafs man in den Naturwissenschaften noch „garnichts“ weifs. In der That sind ja der ungelösten Fragen noch sehr viele, und noch viel mehr Fragen sind noch garnicht aufgeworfen; denn es ist eine Eigentümlichkeit der naturwissenschaftlichen Forschung, dafs sich an die Beantwortung jeder Frage die Aufstellung neuer, vorher ungeahnter Fragen anschliefst, dafs jedes „darum“ gar viele „warum?“ gebiert, die erst wieder ihr „darum“ erfordern, und dafs dies in unendlicher Kette weiter geht. Die Kette ist in der That unendlich, im kleinen und im grofsen, im Raume und in der Zeit, und wenn wir bedenken, dafs wir nur über endliches verfügen, so verstehen wir, warum ein Faust darüber verzweifelt, dafs er die Kräfte der Natur rings um sich her nicht enthüllen kann. Diesem unendlichen gegenüber, was wir wissen müfsten, ist das endliche, was wir wissen können, unter allen Umständen gleich null, und von diesem Gesichtspunkte aus haben jene Leute, die da glauben, in den Naturwissenschaften wisse man noch „garnichts,“ ja ohne Frage recht. Aber es giebt noch einen anderen Gesichtspunkt, als den dieser Leute, die unmögliches verlangen, und als den des Faust, der unmögliches erstrebt, den Gesichtspunkt nämlich, von dem aus man das, was wir jetzt wissen, nicht mit dem vergleicht, was wir wissen müfsten, sondern mit dem, was man früher gewufst hat. Diesem „nichts“ gegenüber ist das, was wir jetzt wissen, sehr grofs, und darum sollte Goethe

nicht so sehr über die Leute spotten, die ein grofses Ergetzen darin finden, sich in den Geist der Zeiten zu versetzen, um sich dann darüber zu freuen, „dafs wir's zuletzt so herrlich weit gebracht". Wir können uns als Naturforscher in der That diese Freude gönnen, — denn trotz derselben werden wir ja immer vor Überhebung geschützt, wenn wir uns daran erinnern, wie viel noch zu forschen ist, selbst wenn wir nicht das unmögliche, unendliche verlangen.

Diese Freude können wir uns auch mit Bezug auf das Centralnervensystem gönnen, so sehr wir gerade da durch die Fülle der noch zu lösenden Fragen zur Bescheidenheit gemahnt werden. Wir brauchen gar nicht in die Zeiten des Hippocrates, des Rhazes oder anderer ganz alter Namen zurückzugehen, noch im Anfang dieses Jahrhunderts waren die Vorstellungen über den feinern Bau des Hirns und Rückenmarks, über die Funktionen ihrer verschiedenen Teile noch ungemein mangelhafte. Die alten Fragen, ob das Hirn aus dem Rückenmarke käme oder umgekehrt, ob das Rückenmark ein Nerv wäre oder nicht, wurden noch eifrig diskutiert, und gerade die letzterwähnte Frage gab die Veranlassung zu jener berühmten Untersuchung, welche pflichtgemäfs an die Spitze jeder geschichtlichen Erörterung über die Neuroglia gestellt wird, zu der von Keuffel „Über das Rückenmark".[1]

Freilich glaubte Keuffel nicht, dafs das Rückenmark als ganzes ein Nerv wäre. Man kannte ja damals schon die graue Substanz, die in den peripherischen Nerven nicht existiert, und man sprach von einem „hydrogenen und oxygenen Gegensatz" im Centralnervensystem,[2] wobei dem hydrogenen die graue, dem oxygenen die weifse Substanz entsprach, aber die letztere war doch in ihrem Aussehen den Nerven so ähnlich, dafs Keuffel nachsah, ob denn nicht in dieser Substanz auch jener Bestandteil ein Analogon hätte, den sein Lehrer Reil in den peripherischen Nerven gefunden hatte, nämlich das Neurilemm.

Keuffel war freilich nicht der erste, der am Rückenmark „dieselbe strangförmige Struktur beobachtete, welche Reil an den Nervenbündeln entdeckt hatte", sondern Villars in Strassburg, wie Keuffel selbst berichtet. Villars hat auch zum ersten Male „kleine Scheibchen" aus dem Rückenmark geschnitten, während man vorher nur die üblichen groben Präparationsmethoden auch zum Studium des Rückenmarks benutzte. Aber die blofse Anfertigung von Schnitten genügte nicht, um Klarheit über die etwaige Anwesenheit eines „Neurilemms" zu schaffen, so dafs Villars nicht recht vorwärts gekommen zu sein scheint, und Keuffel wandte daher auch chemische Agentien bei seinen Forschungen an. Er benutzte schon Sublimatlösungen und verdünnte Salpetersäure zur Härtung des Rückenmarks, aber

---

[1] Reils und Authenrieds Archiv. Band X, S. 161 ff.

[2] Reils und Authenrieds Archiv. Band IX, S. 485.

gerade zum Nachweis eines neurilemmähnlichen Bestandteiles verwandte er eine andere Methode. Er that kleine Stückchen von Rückenmark auf eine Woche oder länger in Kalilauge (1/2—1 Drachme auf eine Unze Wassers, d. h. 2—4 Gramm Kali causticum auf 30 Gramm Wasser). Dann machte er feine Schnitte von den Stückchen, brachte sie in Wasser, pinselte sie aus und untersuchte sie teils mit blofsem Auge, teils mit einem „sehr scharfen“ Mikroscope.

Freilich entspricht das, was er gesehen hat, nicht dem, was wir jetzt „Neuroglia“ nennen, sondern das, was er vor sich hatte, war wohl das Gefäfsnetz des Rückenmarks; denn wie schon Henle und Merkel angegeben haben, verschwindet bei der von Keuffel benutzten Methode die echte Neuroglia, während die Gefäfse und das eigentliche Bindegewebe erhalten bleiben. Man kann sich davon leicht überzeugen, wenn man Gefrierschnitte vom Rückenmark mit der obigen Kalilauge behandelt und dann in viel Wasser bringt. Keuffel giebt denn auch in der That an, dafs die Fasern aus kleinen Kugeln zusammengesetzt gewesen seien, die bei den geringen Vergröfserungen, welche damals den Forschern zu Gebote standen, wohl nur die roten Blutkörperchen in den Gefäfsen gewesen sein können. Auch aus seinen Zeichnungen geht hervor, dafs er die eigentliche Neuroglia nicht vor sich hatte, denn gerade die Stelle der dichtesten Anhäufung derselben, die Umgebung des Centralkanals, erscheint in seinen Zeichnungen ganz hell.

Wenn man daher Keuffel als Entdecker der Neuroglia hinstellt, so geschieht das durchaus mit Unrecht, aber es war doch schon ein grofser Fortschritt, dafs er über die Lagerung der Nervenfasern in den von dem „Neurilemm“, dem „verdichteten Zellstoff“ umschlossenen Räumen eine Vorstellung bekam. Er verglich die weifse Substanz mit einem spanischen Rohre, bei dem die längsgestellten Höhlen von Nervenfasern ausgefüllt waren. Wenn man bedenkt, dafs noch 14 Jahre später Rolando (Sulla struttura del midollo spinale, Torino 1824) der Meinung war, die weifse Substanz bestände „aus einer gefalteten Markhaut, deren umgeschlagene Ränder abwechselnd im Centrum und in der Peripherie lägen“, so wird man wohl zugeben müssen, dafs Keuffel seiner Zeit weit vorausgeeilt war. Zur Erkenntnis des wahren Sachverhalts waren damals, abgesehen von allem andern, die Mikroscope noch zu mangelhaft.

Friedrich Arnold,[1] aus dessen Buche das Citat über Rolando entnommen ist, schlofs sich den Ausführungen von Keuffel an, ohne wesentlich neue Thatsachen zu finden, und so ist denn seit der Arbeit Keuffels bis zum Auftreten des nächsten selbständigen Forschers eine Pause von 36 Jahren. Erst 1846 kam Virchow mit neuen Beobachtungen,

[1] Bemerkungen über den Bau des Hirns und Rückenmarks. Zürich 1839.

welche die Anwesenheit einer spezifischen nicht nervösen Substanz im Centralnervensystem wirklich nachwiesen. **Jetzt erst war die Neuroglia entdeckt.**

Virchow ging beim Nachweis desjenigen Gewebes, welches er später (1853) „Neuroglia“ nannte, nicht vom Rückenmark, sondern vom Ependym der Hirnventrikel aus. Schon 1846[1] erwähnt er unterhalb der Epithelzellen der Ventrikel „eine ganz strukturlose Membran, die häufig aus ziemlich regelmäfsigen, parallel neben einander liegenden sehr feinen und blassen Fibrillen (Faltungen?) zusammengestellt erscheint“. Zuweilen sah er in dieser Membran nach Essigsäurezusatz Kerne, meist aber fehlten sie. Durch „Reizung“ des Ependym kämen die bekannten perlartigen Granulationen auf demselben zustande, die er den Pacchionischen Granulationen, den knötenförmigen Verdickungen der serösen Häute als „ähnliche Bildungen“ an die Seite stellt. Das Ependym sei also eine selbständige Bildung und nicht, worüber man sich damals stritt, eine Fortsetzung der Pia mater oder der Arachnoidea oder beider.

Vier Jahre nachher glaubt Virchow sogar diese Ependymmembran mit dem Scalpell isolieren zu können[2] und auch später[3] behauptet er noch Henle gegenüber, dafs die Existenz dieser Haut schon makroscopisch nicht zweifelhaft sein kann — Annahmen, die sich natürlich sehr bald als nicht mehr haltbar erwiesen.

Im folgenden Jahre giebt er denn auch selbst schon an, dafs das Ependym sich ohne bestimmte Grenze zwischen die nervösen Elemente des Centralnervensystems einschliefslich der höheren Sinnesnerven fortsetzt, dafs überall hier eine „weiche, der Bindesubstanz zugehörige Grundmasse“ die Nervenelemente durchsetzt und zusammenhält, so dafs das Ependym nur der an der Oberfläche frei hervortretende Teil dieser Bindemasse ist.

Zwei Jahre später[5] erwähnt er zum ersten Male eine pathologische Wucherung der Bindesubstanz des Centralnervensystems bei einem Falle von Tabes. In diesen gewucherten Massen sah er nach Härtung in Chromsäure an Stelle der sonst feinkörnigen Substanz ganz dicht gelagerte vielfach verfilzte äufserst feine aber derbe Fibrillen zum Vorschein kommen. Virchow legt jedoch auf die Fibrillen als notwendige Bestandteile der Neuroglia kein Gewicht, so dafs er sogar in der Cellularpathologie (2. Auflage 1859, S. 252 ff.) noch besonders

---

[1] Über das granulierte Ansehen der Wandungen der Gehirnventrikel. Zeitschrift für Psychiatrie, 1846. Ges. Abh. S. 885 ff.

[2] Virchows Archiv. Band 3, S. 246.

[3] Virchows Archiv. Band 5, S. 592.

[4] Virchows Archiv. Band 6, S. 138.

[5] Virchows Archiv. Band 8, S. 540.

erwähnt, dafs allerdings an manchen Stellen die „Neuroglia“ wie Bindegewebe aussieht, an anderen Stellen aber „eine sehr weiche Beschaffenheit besitzt, so dafs es überaus schwierig ist, eine Beschreibung von ihrem Aussehen zu geben“. Auch die Zellen schildert er als nur hier und da sternförmige oder spindlige wie im echten Bindegewebe, sonst aber als sehr weiche und zerbrechliche rundliche Gebilde.

Virchow war sich schon ganz klar darüber, dafs diese Bindesubstanz von dem gewöhnlichen Bindegewebe zu unterscheiden wäre und aus diesem Grunde hat er ihr ja eben auch einen besonderen Namen „Nervenkitt“ gegeben. Dieser Name sollte besonders auf das mehr homogene Wesen dieser Substanz hinweisen, im Gegensatz zu der typisch faserigen Beschaffenheit des gewöhnlichen Bindegewebes. Es ist auch bemerkenswert, dafs Virchow schon beobachtet hatte, wie leicht die Neuroglia kadaverösen Veränderungen ausgesetzt ist, und ferner, dafs er das Fehlen des typischen Nervenkitts in den peripherischen Nerven konstatiert hat.

Er erwähnt auch schon, „dafs die Gefäfse innerhalb der Neuroglia verlaufen, welche daher von der Nervenmasse fast überall noch durch ein leichtes Zwischenlager getrennt sind und nicht im unmittelbaren Kontakt mit derselben sich befinden“ (Cellularpathologie, 3. Aufl., S. 255), er hebt die Zugehörigkeit des Centralkanals zum „centralen Ependymfaden“ hervor, — mit einem Worte, es ist erstaunlich, was er damals alles schon richtig erkannt hatte, wenn ihm auch die typisch faserige Beschaffenheit der Neuroglia nur in den krankhaften Wucherungen deutlich zur Erkenntnis gekommen ist. Trotz alledem aber wird man nicht umhin können, Deiters[1] Recht zu geben, welcher sagt, dafs bei allen diesen ersten Arbeiten über die Neuroglia es sich „mehr um eine geistreiche Divination, als um eine durch stringente Beweise gestützte Behauptung“ gehandelt habe. Einen „stringenten Beweis“ dafür, dafs die Grundmasse des Ependyms die Natur einer Bindesubstanz habe, oder dafs die Zellen, die er als Neurogliazellen anspricht, nicht nervöser Natur seien, hat Virchow nicht gebracht, ja einen solchen Beweis konnte in der damaligen Zeit überhaupt kein Mensch bringen, dazu waren die Methoden und die Kenntnisse noch zu mangelhaft. Wenn man auch schon die grofsen Nervenzellen kannte und grobe markhaltige Fasern nachzuweisen vermochte, so wufste man doch weder die kleinen Nervenzellen, noch die feineren markhaltigen oder gar die vielen marklosen Nervenfibrillen im Centralnervensystem zu erkennen. Daher konnte denn auch Henle mit demselben Rechte, soweit es sich um „stringente Beweise“ handelt, behaupten, dafs die Epithelzellen in den Hirnventrikeln nicht auf einer

[1] Untersuchungen über Hirn und Rückenmark des Menschen und der Säugetiere. Braunschweig 1865.

Bindesubstanz, sondern direkt auf Nervengewebe aufsäfsen. Virchow war aber Henle in „geistreicher", oder sagen wir lieber „genialer" Divination in diesem Punkte über.

Bei der Unvollkommenheit der damaligen Methoden ist es erklärlich, dafs in der nächsten Zeit keine rechten Fortschritte in Bezug auf die Neuroglia gemacht wurden. Zwar bemühten sich Bidder und Kupffer wenigstens (in dem richtigen Bewufstsein, dafs dies durchaus nötig wäre), Kennzeichen aufzufinden, durch welche man das, was man für Bindegewebe halten sollte, auch in der That vom Nervengewebe unterscheiden könnte — Kennzeichen, nach denen zu suchen Virchow noch gar nicht für nötig gefunden hatte, — aber die technischen Hilfsmittel waren zur Entscheidung dieser Frage noch nicht genügend. Bidder und Kupffer[1] nahmen zunächst an, dafs man die Neurogliazellen von den Nervenzellen dadurch unterscheiden könnte, dafs sich die letzteren in Chromsäure gelb bis rötlich färbten, während die bindegewebigen Zellen ungefärbt blieben — ein Unterschied, der schon damals als nicht stichhaltig erkannt wurde (z. B. von Kölliker). Die bindegewebige Intercellularsubstanz ferner suchten sie dadurch als solche zu erkennen, dafs sie einen Zusammenhang ihrer Fasern mit anderen sicher nicht nervösen Elementen nachwiesen.

Für Bindegewebsfasern hielten sie von dieser Überlegung ausgehend einmal die von Hanover (1844) entdeckten fadenförmigen Fortsätze der Epithelzellen des Centralkanals, die Hanover noch als Nervenfasern angesprochen hatte. Diese Fasern hängen mit anderen zusammen, die von eckigen in Chromsäure ungefärbten Zellen ausgehen, deren Ausläufer auch untereinander kommunizieren, so dafs Bilder entstehen, „welche an die anastomosierenden Fortsätze der Knochenkörperchen in dünnen Schliffen erinnern" (S. 45). (Ein Zusammenhang von Epithel- und Bindegewebszellen galt damals für gar nicht so merkwürdig. Auch an den Zottenepithelien des Darms z. B. glaubten andere Forscher, dasselbe statuieren zu können.)

Als zweite Art des Zusammenhangs von Neuroglia mit sicher nicht nervösen Teilen betrachteten sie den Übergang von Fasern der Pia mater ins Centralnervensystem. Solche Fasern treten nach ihnen einmal an der ganzen freien Oberfläche, sodann aber durch den Piafortsatz der hinteren und vorderen Spalte ins Rückenmark. Diese letzteren Fasern gehen ohne bestimmte Grenze in die graue Substanz über und von dieser namentlich durch die Processus reticulares in die weifse (S. 48). Die graue Substanz erscheint ihnen daher mit Ausnahme der Nervenzellen ganz aus Bindegewebe zu bestehen. Das Bindegewebe wird von ihnen teils als formlose, hyaline oder gekörnte Masse beschrieben, teils lassen sie in ihm spiralige und elastische Fasern, wie im gewöhnlichen Bindegewebe, verlaufen (S. 93).

[1] Untersuchungen über die Textur des Rückenmarks und die Entwicklung seiner Formelemente. 1857.

Wie man sieht, war das Bestreben dieser Autoren, Klarheit in die Unterschiede der bindegewebigen und nervösen Elemente des Rückenmarks zu bringen, sehr lobenswert, aber bei der mangelhaften Technik wurden sie zu Irrtümern geführt: Eine Fortsetzung der Piafasern in die Neurogliafasern existiert ja garnicht und in der grauen Substanz sind aufser den „Nervenzellen“, d. h. den damals bekannten Leibern derselben, noch grofse Massen nervösen Gewebes vorhanden.

Diese graue Substanz war überhaupt in der Neurogliafrage die Crux autorum bis in die neueste Zeit herein und sie veranlafste höchst unfruchtbare Streitigkeiten, einmal über die Natur der kleineren in ihr enthaltenen Zellen, sodann aber auch über die „moleculare“, schwammige Zwischenmasse. Es kam zur Verwirrung dieser Angelegenheit noch hinzu, dafs man die Rindenschicht des Rückenmarks auch zur „grauen“ Substanz rechnete, ja dafs man die Zwischenmasse zwischen den Nervenfasern der weifsen Substanz der grauen an die Seite stellte. Als daher Max Schultze in den molecularen Retinaschichten etc. ein Netzwerk analog dem der Lymphdrüsen (auch mit eingelagerten Kernen) entdeckt haben wollte, das für die graue „moleculare“ Masse (selbst des Gehirns) typisch sein sollte, so konnte es sich ereignen, dafs man diese Auffassung der Struktur auf alle Neurogliamassen, auch die der weifsen Substanz übertrug. Namentlich führte Kölliker[1] diese Anschauung konsequent durch. Er gab an, dafs sowohl in den weifsen, als auch, und zwar ganz besonders, in den grauen Massen ein dichtes Netzwerk mit eingelagerten Kernen vorhanden sei. Die Kerne entsprechen, ähnlich wie in den Lymphdrüsen, Zellen mit zahlreichen verästelten Ausläufern. Besonders eng ist das Netzwerk in der grauen Substanz des Grofshirns. Das Reticulum hängt sowohl, wie dies ja auch Bidder und Kupffer für ihre Zwischensubstanz angenommen hatten, mit den Ausläufern der Ependymzellen, als mit dem Bindegewebe der Pia mater zusammen. Besonders kernreich ist es in der Körnerschicht des Kleinhirns und der des Ammonshorns. Kölliker spricht sich auch entschieden dafür aus, dafs dieses Reticulum, wenn es auch mit der Pia in Beziehung tritt, doch kein gewöhnliches Bindegewebe sei, und dafs überhaupt, mit Ausnahme der Adventitia der gröfseren Gefäfse etc., kein gewöhnliches Bindegewebe im Innern des Centralnervensystems vorkommt, Auch er hebt, wie schon Virchow, die Beziehungen des Netzwerks zu den Gefäfsen hervor, bei denen, wenn diese nicht gerade sehr grofs sind, die Adventitia nur aus diesem Netzwerk besteht „und nur selten auch fibrilläres Bindgewebe enthält“.

So richtige Ansichten auch in dieser Köllikerschen Darstellung enthalten sind, so

---

[1] Gewebelehre des Menschen, citiert nach der vierten Auflage (1863), S. 303 ff.

hat er augenscheinlich bei der von ihm angewendeten Methode die eigentliche Structur der Neuroglia nicht gesehen und sicherlich auch allerlei künstliche Netzwerke besonders in der molecularen Masse mit Neuroglia verwechselt. Das folgt nicht etwa daraus, dafs er die Neuroglia ein Reticulum bilden, d. h. aus anastomosierenden Fäden bestehen läfst, denn wenn er die richtige Structur gesehen hätte, so wäre die Annahme einer Anastomosierung der Fäden etwas sehr nebensächliches gewesen, aber seine Abbildungen beweisen deutlich, dafs er, wie gesagt, garnicht die Neuroglia in ihrer Reinheit vor sich gehabt hat. Fig. 166 und 167 sind Bilder, wie sie nicht in der weifsen Substanz vorkommen, denn die Zwischenräume zwischen den Nervenfasern sind mit einer ganz diffusen Masse erfüllt. Fig. 168 ist ebenfalls eine Abbildung, wie sie nie für die Neurogliastruktur gegeben werden könnte, sondern wohl die irgend eines Kunstproduktes. Als letzteres sind jedenfalls auch die Netzwerke aufzufassen, die er in den grauen Substanzen wahrnahm, denn gerade an den Orten, wo er die Reticula besonders eng und besonders reichlich fand (Grofshirnrinde, Körnerschicht des Kleinhirns) ist die Neuroglia aufserordentlich spärlich.

Überhaupt hatte damals (in Deutschland wenigstens) noch keiner die richtige Neurogliastruktur gesehen, ja Stilling leugnete überhaupt die Anwesenheit einer „bindegewebigen“ Substanz im Centralnervensystem.

Hingegen hatte schon 1859 in England J. L. Clarke[1] wenigstens annähernd das richtige im Rückenmarke wahrgenommen. Jedenfalls ist dies für die Rindenschicht dieses Organs zuzugeben, die er ganz richtig als ein Lager in einander verwebter hauptsächlich der Oberfläche paralleler Fasern beschreibt (S. 441). Die Fasern läfst er auch in die weifse Substanz abbiegen, welche sie durchsetzen, um sich einem ähnlichen Netzwerk in der grauen Substanz anzuschliefsen. Die Bindegewebszellen haben nach ihm verschieden geformte Kerne und in deren Umgebung ist teils eine körnige Substanz vorhanden, teils sind die Kerne direkt an die Bindegewebefasern angelegt (S. 442): „bei Erwachsenen sind die Zellleiber verschwunden und es bleiben nur die Kerne zurück“.

Aber Clarke war sich klar genug darüber, dafs die Zeit noch nicht gekommen war, um zwischen nervösen und bindegewebigen Elementen scharf zu unterscheiden und so schliefst er denn seine Betrachtung (S. 442) mit den Worten: „These observations render it apparently impossible, to print out the exact distinction between the connectif and the nerve tissue, and might suggest the question, whether there is any actual and essential difference

[1] Philosophical transactions. 1859. S. 437 ff.

between them or whether the connectif tissue of the cord be intermediate in its nature passing on the one hand into nerve-tissue and on the other into the pia mater.“ [1] —

Richtige Bilder der Neuroglia des Rückenmarks, wenigstens in der weiſsen Substanz und um den Centralkanal herum, hat dann ein Forscher gesehen, mit dem (und mit Clarke) eine neue Epoche in der Geschichte der Neuroglia beginnt, nämlich Frommann[2], dessen Arbeiten in fast allen geschichtlichen Darstellungen ganz en bagatelle behandelt werden. Der Grund dafür liegt wohl darin, daſs es geradezu eine Qual ist, sich durch die entsetzlich weitschweifigen Schilderungen der minimalsten Details, durch die ungemein unklaren langausgedehnten Erörterungen des Autors, — man muſs wohl sagen — hindurchzuwürgen, so daſs es wohl nur wenige fertig gebracht haben, überhaupt die Arbeiten Frommanns zu lesen oder gar dabei die Spreu vom Weizen zu sondern. Hat man das aber einmal gethan, so findet man, daſs dieser augenscheinlich vortreffliche Beobachter, der freilich nur das Rückenmark bearbeitet hat, eigentlich alles gesehen hat, was man mit der so unsicheren Carminmethode sehen kann. Seine Beschreibungen und Abbildungen der Neuroglia in der weiſsen Substanz und um den Centralkanal herum sind geradezu für die damalige Zeit musterhaft.

Für die Frage, um die es sich hier handelt, ist es zunächst gleichgültig, ob er die Fasern für hohl oder solid, verästelt oder nicht verästelt, für anastomosierend oder nicht anastomosierend, für selbständige Gebilde oder für Zellausläufer hält: die richtigen Fasern hat er jedenfalls gesehen und zwar (höchstens mit Ausnahme von Clarke) zuerst gesehen, und in möglichster Vollständigkeit vor sich gehabt.

Bei der von Frommann benutzten Carminmethode erscheinen die Fasern ja als Zellfortsätze und Frommann spricht sich ganz klar in Bezug hierauf aus. Er sagt (S. 45 f., Teil I): „Daſs die Ausläufer der Zellen sich in die Fasern fortsetzen, ist direkt nicht nachzuweisen; man kann zwar einzelne derselben ungeteilt und mit nicht abnehmender Stärke über gröſsere Strecken verfolgen, indessen über ihre weiteren Schicksale läſst sich nichts ermitteln. Da aber beide ein gleiches Aussehen besitzen, ein gleiches Verhalten gegen Carmin zeigen, indem die stärkeren sich färben und zwischen den feineren und gröberen Fasern dieselben Gröſsendifferenzen bestehen, wie zwischen den Ausläufern und ihren Ver-

---

[1] Auf einige interessante und richtige Beobachtungen Clarkes über das Epithel des Centralkanals kommen wir in der speziellen Topographie zu sprechen.

[2] Untersuchungen über die normale und pathologische Anatomie des Rückenmarks. Teil I, Jena 1864. Teil II, Jena 1877.

ästelungen, so glaube ich, dafs die Fasern alle aus den Ausläufern der Zellen hervorgegangen und wie diese hohl sind, und dafs somit die ganze Bindesubstanz der weifsen Substanz aus einem zusammenhängenden Netzwerk von Kanälchen von wechselnder Gröfse besteht, für welche die zahlreich eingeschalteten Zellen Sammel- und Mittelpunkte bilden."

Noch schärfer führt es der Teil II, S. 9 aus.

Wenn wir die noch wenig klaren Auseinandersetzungen Clarkes abrechnen, so ist also Frommann der erste gewesen, der die richtigen Neurogliafasern, nicht Kunstprodukte, wie Kölliker, als Zellausläufer betrachtete.

Die Unabhängigkeit der Neuroglia von der Pia mater, selbst an den Piafortsätzen, urgiert er ganz sachgemäfs.

Auch die Gegend um den Centralkanal beschreibt er nicht nur so richtig, wie es damals möglich war, sondern er ist auch der erste gewesen, der die Einstrahlung der Neurogliafasern zwischen die Zellen des Centralkanals schildert.

Er ist fernerhin der erste gewesen, welcher den kadaverösen Zerfall der Neurogliafasern in Körnchen durchaus klar beobachtet hat (I, S. 49).

Freilich in Bezug auf die graue Substanz ist er nicht glücklich gewesen. Er klagt auch selbst darüber, dafs man in dieser die feinen Axencylinder von den Fasern der Bindesubstanz nicht unterscheiden könne. Das schmälert sein grofses Verdienst, zum ersten Male viel richtiges gesehen zu haben, aber nicht, denn das ist die Schuld seiner unvollkommenen Methodik. Wir werden bei unserer Beschreibung der Neuroglia Frommanns Ergebnisse noch öfters zum Vergleich heranziehen. —

Ein weiterer wesentlicher Fortschritt in der Lehre von der Neuroglia wurde nun durch die berühmten Untersuchungen von Deiters gemacht, die an ihrem Werte selbst dadurch nichts verlieren, dafs sie nur im Fragment (nach dem Tode des Verfassers) herausgegeben werden konnten.

Auch Deiters[1] ging, wie Bidder und Kupffer, zunächst an die Beantwortung der so wichtigen Vorfrage, was man denn im Centralnervensystem als nervöse Bestandteile und was man als nicht nervöse Zwischen- oder Bindemasse betrachten solle.

Er sagte sich mit Recht, dafs man bei der Beurteilung dieser Verhältnisse nicht von einem schematischen Bindegewebsbegriff ausgehen müsse. „Wer z. B.," schreibt er, „im Bindegewebe unter allen Umständen eine faserige Masse sieht, zwischen deren Fasern aus-

[1] Untersuchungen über Gehirn und Rückenmark des Menschen und der Säugetiere. Braunschweig 1865

gebildete sternförmige Zellkörper liegen sollen, der wird einer doppelten Gefahr ausgesetzt sein, entweder die ausgebreitete Anwesenheit von Bindegewebe überhaupt in Frage zu stellen, oder dasselbe in seinem Charakter überall wiederfinden zu wollen, z. B. jede sternförmige Ganglienzelle leicht zu einer Bindegewebezelle zu stempeln.“ (S. 28) Man wird vielmehr, meint er ganz richtig, nicht verlangen können, daſs die im nervösen Centralorgan vorkommenden Stützsubstanzen gleich dem gewöhnlichen (wie wir jetzt sagen, collagenen) Bindegewebe beschaffen seien, sondern wird sich unter Umständen damit begnügen können, daſs man nachweist, gewisse Bestandteile könnten nicht nervös sein, sondern müſsten als eine Zwischensubstanz angesehen werden, die ihrerseits aber von gewöhnlichem Bindegewebe verschieden sein könnte.

Zunächst freilich nimmt er wie Bidder und Kupffer an, daſs auch echtes Bindegewebe in die Centralorgane eintreten könne, d. h. solches, welches sicher mit der Pia mater zusammenhängt. Diese Art Zwischensubstanz kommt nach ihm nicht überall vor, sondern nur an bestimmten Stellen. Hier ist sie den Müllerschen Fasern in der Retina zu vergleichen. „In gröſster Ausdehnung und in zweifellosester Form kommt sie da vor, wo die weiſse Substanz die äuſsere Peripherie bildet, also am Rückenmark. Hier zieht ein den Nervenfasern fremdes Gewebe bekanntlich in dichten Massen durch die Bündel derselben und schlieſst zuletzt fast jede Nervenprimitivfaser mehr oder weniger ab.“ (S. 36.) Auch in die graue Substanz läſst er Fasern der Pia eintreten, einmal wie Bidder und Kupffer, im Rückenmark, wo die Piafortsätze der vorderen nnd hinteren Fissur in die graue Substanz ausstrahlen sollen, sodann aber an der Oberfläche des Kleinhirns. Hier finden sich jene radiären Fasern, die auch Bergmann in Greifswald entdeckt hat, und die daher „Bergmannsche Fasern“ genannt werden.[1] Es muss aber bemerkt werden, daſs der Bergmannsche Aufsatz zwar schon erschienen war, als die Deiterssche Arbeit herauskam, daſs aber Deiters keine Kenntnis davon haben konnte und daher als Mitentdecker dieser Fasern zu betrachten ist, die er nur fälschlich für Fortsätze der Pia ansieht. Endlich rechnet Deiters in diese Kategorie noch die mehrfach erwähnten Fortsätze der Epithelzellen des Centralkanals und des Ependyms.

In Beziehung zu dem bis jetzt erwähnten steht die Arbeit von Frommann, die

---

[1] Deiters hat augenscheinlich die richtigen Bilder vor sich gehabt, ob aber Bergmann wirklich die wahren „Bergmannschen Fasern“ gesehen hat, ist mir noch zweifelhaft. Vgl. „Kleinhirn“ in unserem Abschnitt über spezielle Topographie der Neuroglia. Bergmanns Arbeit steht in der Zeitschrift für rationelle Medizin, Neue Folge, Band 8, S. 360.

Deiters ebenfalls noch nicht kennen konnte, wesentlich höher, als die des letzteren. Hat doch Frommann die Unabhängigkeit der Neurogliafasern in der weifsen Substanz des Rückenmarks von den Fasern der Pia mater ganz richtig erkannt, und hat er doch bereits die Ansicht ausgesprochen, dafs auch diese Fasern mit den sogenannten Zellausläufern identisch sind, was Deiters ganz entgangen ist.

In Bezug auf die mit dem Bindegewebe der Pia zusammenhängenden Fasern glaubte also Deiters jeden Zweifel ausgeschlossen, — wie wir jetzt wissen, irrtümlicher Weise. Schwerer schien ihm die Frage nach der Beurteilung anderer etwaiger Zwischensubstanzen, doch wufste er sich auch hierbei zu helfen. Alle diejenigen modifizierten Protoplasmamassen, die sich von den Zellen emanzipiert hatten, und nicht mehr als zu ihnen gehörig betrachtet werden konnten, mufsten nach der Lehre von Max Schultze als Zwischensubstanzen angesehen werden. An und für sich ist die Ansicht von Max Schultze durchaus zutreffend, aber die Anwendung auf den vorliegenden Fall war verfrüht, — auch hier waren die Methoden nicht ausreichend, um vor Irrtümern zu schützen. In solche Irrtümer ist denn auch Deiters verfallen, indem er als eine zweite Form der Zwischensubstanz jene früher und später so viel besprochene „schwammig-poröse“ (moleculäre) Substanz (S. 39) anführt. Sie soll in der grauen Substanz die „Hauptmasse“ darstellen, in welcher die Nervenzellen und vereinzelte Nervenfasern eingebettet liegen, aber auch in der weissen soll sie vorkommen. Die Masse könne ja garnicht nervöser Natur sein, meint Deiters, denn von einer Leitungsisolation könne hier nicht die Rede sein, sie sei vielmehr nach der obenerwähnten Definition, welche Max Schultze gegeben hat, als Zwischensubstanz zu betrachten, da sie im ausgebildeten Zustande von den Zellleibern ganz unabhängig ist. An ihrer Erzeugung können sich freilich sowohl Nervenzellen als die gleich zu erwähnenden freien Kerne beteiligen, so dafs sie vom rein entwicklungsgeschichtlichen Standpunkte aus etwas neutrales, zwischen Nerven- und Bindegewebe stehendes darstelle. Indem sie sich aber allmählich von beiden Zellarten emanzipiere, stelle sie schliefslich eine echte Intercellularsubstanz, ein eigenartiges Bindesubstrat, dar.

Wir wissen jetzt, dank der Resultate der Golgischen Methode, dafs diese Annahme ganz irrig war. Die „schwammig-poröse Masse“ ist eben garnicht schwammig-porös, sie ist garnicht von den Zellen emanzipiert, sondern stellt ein ungeheures Gewirr von Zelldendriten und Axencylindern dar, in dem isolierte Leitungen sehr wohl möglich sind.

Als drittes bindegewebiges Element (aufser den echten „Bindegewebs“-Fasern und der porösen Grundmasse) betrachtete nun Deiters auch noch Zellen. Auch in dieser Frage

hielt er sich an die Lehren von Max Schultze, und er benutzte auch die von diesem erfundene Isolierungsmethode. Diese besteht bekanntlich darin, dafs man Stückchen des Centralnervensystems in dünnen Lösungen von Chrompräparaten gleichzeitig etwas härtet und maceriert.

Max Schultze hatte damals seine mit Recht so berühmten Arbeiten über die Zelle schon publiziert und hatte in Bezug auf das Bindegewebe festgestellt, dafs hier die Zellen einen rudimentären, d. h. protoplasmaarmen Charakter hätten. Das können wir auch heutzutage für das gewöhnliche und zwar wohlgemerkt normale, pathologisch nicht veränderte Bindegewebe zugeben, aber Deiters ging nun noch einen Schritt weiter. Er nahm nicht nur an, dafs im Bindegewebe die Zellen protoplasmaarm wären, sondern meinte nun auch, dafs alle Zellen, die er für protoplasmaarm hielt, bindegewebig wären. Er nannte diese Zellen, die wenig oder anscheinend gar kein Protoplasma, d. h. keinen „ausgesprochenen Zellcharakter" hatten: „Zellaequivalente", und wo er solche fand, hielt er sie für bindegewebige Zellen, zumal er konstatiert zu haben glaubte (S. 48), dafs alle Zellen im Centralnervensystem, bei welchen eine Zusammengehörigkeit mit nervösen Elementen bestimmt nachzuweisen war, ein entwickelteres, mehr solides Protoplasma hätten. Von diesem Grundsatze ausgehend, verfiel er wieder in den Irrtum, die sogenannten „Körner" im Kleinhirn und im Ammonshorn, sowie alle übrigen damals so genannten „freien Kerne" des Centralnervensystems für bindegewebig zu erklären. Andererseits hat er aber doch eine Art von Zellen richtig als „bindegewebig" erkannt, das sind diejenigen Gebilde, die wir jetzt noch Deitersssche Zellen nennen.

Deiters schildert sie als Zellaequivalente, bei denen um den Kern herum nur ein sparsames Protoplasma (d. h. echtes gekörntes Protoplasma) vorhanden ist, das sich in lange mehr oder weniger veränderte glatte Fortsätze auszieht und dadurch je nach Umständen den Anschein faseriger Bildungen erzeugt (S. 38). Die Fortsätze haben von Anfang an ein festes, wenn auch zartes Aussehen, einen ganz scharfen, glatten Contour und einen beträchtlichen Glanz. Sie strahlen in grofser Masse nach allen Seiten aus und verästeln sich auf das mannigfaltigste unter immer gabelförmiger Spaltung (S. 45). Er fand diese Zellaequivalente sowohl in der grauen wie in der weifsen Substanz, und das meiste, was man von anscheinenden Fasern im Centralnervensystem sieht (mit Ausnahme der oben erwähnten Einstrahlungen), fafst er als solche „Zellausläufer" auf. Besonders reichlich (irrtümlicherweise) fand er sie auch in der Substantia gelatinosa Rolando.

Der Schreiber dieses kann ja nicht zugeben, dafs jene sonderbaren strahligen Gebilde,

die Deitersschen Zellen, von ihrem Entdecker ganz richtig gedeutet wurden, aber trotzdem mufs er konstatieren, dafs mit der Entdeckung jener „Zellen“ ein grofser Fortschritt gemacht war. Denn, wie man sie auch auffafst, sie sind einigermafsen charakteristisch geformte Elemente und durch ihren Nachweis war, wenn man die nötige Vorsicht dabei nicht aufser Acht liefs, die Möglichkeit gegeben, wenigstens die Anwesenheit der Neuroglia auch an solchen Orten festzustellen, wo die Verhältnisse nicht gar so einfach lagen, wie das z. B. in der weifsen Substanz des Rückenmarks der Fall ist. Über die wahre topographische Verteilung konnte man sich freilich an Zerzupfungspräparaten kein Urteil bilden. Ob ihr Nachweis allein, selbst mit besseren Methoden und an Schnittpräparaten hierfür genügt, wird sich erst später besprechen lassen. —

Wir wollen hier auch gleich die Ansichten von Henle anfügen. In der Arbeit mit Merkel[1] wird die Darstellung so durch eine heute zum Teil schwer kontrollierbare Polemik durchsetzt, dafs die Meinungen der Autoren nicht recht klar zu Tage treten. Aus dieser Arbeit werden wir aber später einige wichtige chemische Notizen entnehmen. Wir halten uns hier an die Darstellung, die Henle in der ersten Auflage seines berühmten Handbuchs der systematischen Anatomie, Abschnitt Nervenlehre,[2] giebt.

Henle unterscheidet als Zwischensubstanz zunächst eine diffuse feinkörnige Masse. Diese bildet die äufsere Schicht der Rinde des Grofshirns und Kleinhirns, so wie eine dünne Rindenschicht des Rückenmarks, umgiebt in geringer Mächtigkeit den Centralkanal und stellt den peripherischen Teil der hinteren grauen Säulen des Rückenmarks dar (Substantia gelatinosa Rolando). Sie erscheint nirgends ganz rein, namentlich enthält sie aufser Nervenzellen auch lymphkörperchenähnliche „Körner“. Am reinsten ist sie in der Substantia gelatinosa Rolando. Bindegewebefasern sind auch vorhanden, aber schwer von nackten Axencylindern zu unterscheiden, da sie sich wie diese in Kalilauge lösen. Einen überwiegenden Teil bilden diese Bindegewebefasern in den äufsersten Lagen der Hirn- und Rückenmarksrinde. Sie gehören aber einer anderen Varietät an, als z. B. das Bindegewebe der Pia mater, mit der sie nur in Berührung stehen. Diese Varietät ist die verfilzte, deren steife Fibrillen in mannigfaltigsten Richtungen von kleinen multipolaren Zellen ausgehen.

Henle hat also augenscheinlich die richtigen Fasern nur an wenigen Orten gesehen,

---

[1] Über die sogenannte Bindesubstanz der Centralorgane des Nervensystems. Zeitschrift für rationelle Medizin. 3. Reihe, Band 34.

[2] Braunschweig 1871.

an vielen Stellen, wie um den Centralkanal herum etc., sind ihm die Fasern als eine diffuse feinkörnige Masse erschienen, wie sie an diesen Stellen garnicht vorhanden ist. —

Der nächste Forscher, der die Angaben von Deiters im wesentlichen bestätigte und dessen ja nur fragmentarisch aufgefundene Mitteilungen erweiterte, ist Golgi.[1]

In Bezug auf die thatsächlichen Verhältnisse der Zellen, weicht er nur in einigen, nicht gerade wesentlichen Punkten von Deiters ab. Er hat mehr Fortsätze als dieser an den Zellen konstatiert, hat statt der vielen von Deiters angenommenen Teilungen nur sehr spärliche und auch diese nur in geringer Entfernung vom Abgangspunkte beobachtet (S. 8), er bestreitet die Anastomosen, die übrigens auch schon Frommann zweifelhaft erschienen, etc.

In der Hauptsache aber, daſs solche mit langen Fortsätzen versehene isolierbare Zellen charakteristisch für die Neuroglia sind, stimmt er mit Deiters überein. Er erwähnt freilich Deiters nur in einer Anmerkung (S. 31), wo er von nicht genau präcisierten Abweichungen seiner Ansicht spricht.

In anderer Hinsicht hat er aber mehr gesehen, als Deiters. Vor allem ist es ihm gelungen, auch an Schnittpräparaten die charakteristischen „Deitersschen Zellen“ wahrzunehmen, nicht bloſs an Isolationspräparaten. Er betrachtet diese Gebilde, die Deiters als „Zellaequivalente“ auffaſste, als richtige Zellen, ähnlich wie Frommann, der aber noch nicht so typisch „verzweigte“ Bilder vor Augen hatte.

Er hat ferner die Beziehungen dieser „Zellen“ zu den Gefäſsen genauer studiert. Zwar wuſste man schon seit Virchow, daſs die Gefäſse einen Neurogliamantel haben, Golgi zeigte dies aber in sehr charakteristischer Weise und hat namentlich auch bemerkt, daſs entfernter liegende Zellen ihre „Fortsätze“ an die Gefäſse heranschicken. Ferner hebt Golgi sehr richtig hervor, daſs bei älteren Leuten die Neuroglia der Hirnrinde viel stärker ausgebildet ist, als bei jüngeren.

Doch genügten die von ihm damals angewandten Methoden noch nicht, um Irrtümer über die Verteilung der Neuroglia auszuschlieſsen. Selbst seine Abbildungen der weiſsen Substanz des Rückenmarks bleiben, was die Fasern („Zellausläufer“) anbetrifft, doch sehr hinter denen von Frommann zurück. Unzureichend ist auch seine Schilderung der grauen

---

[1] Beitrag zur feineren Anatomie des Centralnervensystems. Bologna 1871, citiert aus den „Untersuchungen über den feineren Bau des centralen und peripherischen Nervensystems. Jena 1894, S. 1 ff. Wir werden der Kürze wegen diese „Untersuchungen“ im folgenden immer unter dem Titel „Gesammelte Abhandlungen“ citieren.

Substanz des Rückenmarks, in der er die Substantia gelatinosa Rolando fast ausschliefslich aus Neuroglia bestehen läfst (S. 34).

An der Grofshirnrinde hat er ganz richtig und zwar als erster gesehen, dafs von den mehr tangentialen Neurogliafasern der oberflächlichen Schicht eine Reihe mehr senkrechter Fasern herabsteigt. Er hatte auch an Osmiumpräparaten ganz richtig erkannt, dafs an der Oberfläche sehr viele, in der Tiefe immer weniger Neurogliazellen vorhanden seien (S. 7), nichtsdestoweniger spricht er schon auf der folgenden Seite sub 2 den Satz aus, „dafs Zellen von gleicher Beschaffenheit in beträchtlicher Zahl über alle Schichten[1] der Hirnrinde zerstreut sind, wo sie ein zusammenhängendes Stützgewebe bilden“. Noch schärfer betont er dies (S. 9 f.) bei Beschreibung von Schnitten, die er nach einer von ihm modifizierten Bichromatbehandlung bekommen hat. Er sagt: „. . . an den Rändern der Schnitte und an deren dünnsten Stellen zeigt sich das interstitielle Stroma auch in den tiefsten Schichten der Hirnrinde als deutlich gefasert, nicht netzförmig im Sinne Schultzes und Köllikers. Damit will ich jedoch nicht das gleichzeitige Vorhandensein einer amorphen, feinkörnigen Intercellularsubstanz in allen Präparaten, die ich beschreiben werde, leugnen, denn ich habe immer Spuren davon gefunden, auch müfste ein Teil derselben bei den Präparaten entfernt worden sein. Aber es scheint mir zweifellos, dafs die sogenannte feinkörnige, oder netzförmige, oder schwammige, oder punktförmig moleculare, amorphe oder gelatinöse Substanz diese verschiedenen Benennungen in Folge von Veränderungen in der Leiche oder durch die Präparationsmethode erhalten hat. . . . Dies alles scheint mir dafür zu sprechen, dafs das interstitielle Stroma der Hirnrinde zum gröfsten Teile aus Bindegewebszellen und ihren Fortsätzen besteht.“

Die Bindegewebszellenfortsätze sollen dann ebenso wie die feinsten protoplasmatischen Fortsätze der Nervenzellen zerfallen können und die Zerfallsprodukte beider die moleculare Substanz erzeugen.

Auch bei ihm spukt also noch das Gespenst von der interstitiellen Natur, wenigstens eines grofsen Teils der „schwammigen“ Substanz in der Grofshirnrinde, ein Gespenst, das gerade durch die von Golgi später erfundene Methode verscheucht worden ist, obgleich Golgi selbst noch 1885 daran festhielt, dafs auch in den tiefsten Schichten des Grofshirns die Verhältnisse so liegen, wie er sie 1871 geschildert hat.[2]

Auch Golgis Angaben über die Molecularschicht des Kleinhirns waren z. T.

[1] d. h. nicht blofs über die oberflächliche, von denen er sub 1 gesprochen hat.

[2] Gesammelte Abhandlungen. S. 162.

irrtümlich, wenn er auch natürlich die sogenannten Bergmannschen Fasern bestätigt hat, und sie richtig (im Gegensatz zu Bergmann) ebenso beschreibt, wie Deiters etc. Er glaubt aber auch für die Molecularschicht des Kleinhirns, dafs sich hier ein zusammenhängendes Stroma findet, welches aus an Fortsätzen reichen Bindegewebszellen besteht. Alle Kerne, welche in der Molecularschicht zerstreut sind, gehören, wie er glaubt, Bindegewebszellen an (S. 17).

Auch die Körnerschicht des Kleinhirns läfst er, wie alle andern Teile des Centralnervensystems ein zusammenhängendes Stroma, bestehend aus Bindegewebszellen mit zahlreichen langen Fortsätzen, haben, welche sich nie oder selten verzweigen, ja er glaubt sogar, dafs die Körner selbst bindegewebige Elemente seien, welche zu den echten mit Ausläufern versehenen Bindegewebszellen häufige Übergänge zeigten. (S. 21.)

Einige der Irrtümer, die das Kleinhirn betreffen, sind später von Golgi selbst unter Anwendung seiner neuen Methode berichtigt worden, die Schilderung des reichen Neurogliagerüsts in der Körnerschicht etc. hält er aber auch 1885 aufrecht. [1]

Am einflufsreichsten oder, wie wir sagen müssen, am verhängnisvollsten waren aber die Ansichten Golgis über das Verhältnis der Fasern zu den Zellen. Zwar hatte schon Frommann ähnliche Meinungen ausgesprochen, aber diese wurden sehr wenig beachtet (auch Golgi erwähnt Frommann nur ganz nebenbei), die Angaben von Deiters waren zu unbestimmt, weil er vorsichtiger Weise nicht von Zellen, sondern von „Zellaequivalenten" sprach, und so war es denn gerade Golgi, durch dessen hier erwähnte und vor allem durch dessen spätere Arbeiten sich die Ansicht mehr und mehr Geltung verschafft hat, dafs die Deitersschen Zellen mit samt ihren Ausläufern echte Zellen seien, und dafs das ganze Neurogliagerüst nichts als das Ausläufergeflecht dieser Zellen darstellte, dafs abgesonderte Fasern überhaupt nicht vorkämen. Wie sehr hierbei gerade die gewaltige Autorität Golgis in den Vordergrund getreten ist, das geht auch daraus hervor, dafs man in neuster Zeit sogar so weit gegangen ist, die sonst „Deitersschе Zellen" genannten Gebilde als „Golgische Zellen" zu bezeichnen. —

Eine ähnliche Beschreibung der Deitersschen Zellen wie Golgi, giebt übrigens (und zwar unabhängig von dem letztern) Jastrowitz, [2] der aber auch Deiters nicht erwähnt.

---

[1] Gesammelte Abhandlungen. S. 167.

[2] Über Encephalitis und Myelitis im ersten Kindesalter. Archiv für Psychiatrie (Band 2, S. 389 ff. und Band 3, S. 162 ff.)

Von Jastrowitz rührt der Name „Spinnenzellen" zur Bezeichnung der Deitersschen Zellen her, doch nimmt er neben diesen noch quadratische und rechteckige, in Reihen liegende Zellen als zur Neuroglia gehörig an. Solche sollen sich nach ihm in der weifsen Substanz des Gehirns finden (er hat nur das Gehirn bearbeitet), und er glaubt, dass diese Zellen rudimentäre Spinnenzellen darstellen. Die „moleculäre" Substanz der Grofshirnrinde hält er nicht für Neuroglia, sondern glaubt, dafs sie „dem nervösen Gewebe viel näher steht, als dem Bindegewebe". Er trennt sie daher auch ganz richtig von der Belegschicht des Rückenmarks. Sehr merkwürdig und nach unserer jetzigen Auffassung ausserordentlich paradox ist seine Schilderung der Beziehung des Ependyms zur Neuroglia. Der betreffende Passus sei hier wörtlich angeführt:

„Je weiter gegen die Ventrikelhöhle, desto gehäufter werden diese Zellen (sc. die Spinnenzellen) angetroffen, sie folgen dichtgedrängt auf einander, indem die Fortsätze meist rückwärts und seitlich ausweichen und schliefslich setzen sie, eins bei eins an einander liegend, das Ependym-Epithel zusammen. Hierbei erleiden sie nur insofern eine Modifikation, als am freien Ende die Fortsätze wegfallen und durch einen doppelt contourirten, oft ziemlich breiten und meist ungefärbten Saum ersetzt werden. Die spärlichen seitlichen und die hinteren in der Richtung gegen die dritte Schicht (sc. des Balkens) ziehenden Ausläufer sind namentlich sehr zart und brechen leicht ab, von den letzteren zeichnet sich jedoch einer durch seine Stärke aus, und an ihm, dem oft einzig erhaltenen, hängt die kelchähnliche (cylindrische) Epithelzelle wie an einem Stiele." . . . . .

„Wir sehen demnach hier bis in alle Einzelheiten die Identität der Gliazellen mit dem sogenannten Epithel der Ventrikel, dessen gleichfalls bindegewebige Natur somit zweifellos erscheint. Es wird daher mit vollem Recht als ein Epithelium spurium, s. Endothel bezeichnet."

Jastrowitz deutet demnach die Beziehungen des Ependymepithels zur Neuroglia gerade umgekehrt, wie das jetzt üblich ist. Während man jetzt wegen der engen, namentlich entwickelungsgeschichtlichen Beziehung der Neuroglia zum Ependymepithel die Neuroglia als etwas epitheliales ansieht, sieht Jastrowitz im Gegenteil das Ependym für etwas bindegewebiges, für ein Endothel an: eine Frage, die bis dahin niemals aufgeworfen war, da der Gegensatz zwischen Epithel und Bindegewebe früher garnicht so klar in das Bewufstsein der Histologen eingedrungen war. —

Die jetzt zu erwähnende Arbeit von Boll[1] ist schon mit Berücksichtigung nicht nur der Deitersschen, sondern auch der Golgischen Veröffentlichungen geschrieben. Boll spricht sich noch entschiedener wie Deiters für die „differenzierte Natur“ der Fasern aus. Er sagt beim Vergleich der Deitersschen Zellen mit denen des (embryonalen) Bindegewebes (S. 8):

„Hier wie dort ist die Zelle, der histiologische Centralteil, nichts anderes als ein Centrum für eine grofse Menge differenzierter Fasern, die nach allen, nach zwei oder nach einer Seite hin ausstrahlen. Hier wie dort liegt in dem Centrum dieser Zelle ein Kern, umgeben von einer gröfseren oder — wie in den weitaus meisten Fällen — geringeren Menge körniger Substanz. Hier wie dort mufs sich die Untersuchung bescheiden, ob in dieser Menge körniger Granulationen, die das Centrum dieses Faserconvoluts einnehmend den Kern umgiebt, lebendiges, leistungsfähiges Protoplasma oder amorphe Eiweifssubstanz zu sehen ist.“

Wie wir später sehen werden, ist diese Auffassung schon ein wesentlicher Fortschritt gegen Golgi, der die ganzen Gebilde als richtige Zellen ansah und noch in späteren Arbeiten Deiters deshalb tadelt, weil er den vorsichtigen Ausdruck „Zellaequivalente“ für seine Gebilde gebraucht hat. Den entscheidenden Schritt in dieser Frage that freilich, wie wir sehen werden, erst Ranvier.

Boll setzt aber übrigens mit Recht trotz dieser Ähnlichkeiten aus entwicklungsgeschichtlichen (und chemischen) Gründen die Neuroglia in einen Gegensatz zum gewöhnlichen Bindegewebe.

Auch Boll nimmt, wie Jastrowitz, an, dafs neben den hier zum ersten Male als „Deitersche“ bezeichneten Zellen reihenförmig angeordnete rechteckige vorkämen. Zwischen beiden Arten von Neurogliazellen findet er „Übergänge“. — Seine Schilderung der weifsen Substanzen ist unzureichend. Er läfst in der weifsen Hirnsubstanz 50—60, in der des Rückenmarks 5—6 Nervenfasern gemeinschaftlich in einer Neurogliaumhüllung liegen, er glaubt auch nicht sicher, dafs die queren Fasern in der weifsen Substanz des Rückenmarks wirklich Neurogliafasern sind etc. Von seinem Standpunkte aus hatte er mit seiner Vorsicht ganz recht, denn er fürchtete Verwechslungen mit freien Axencylindern, die ja in der That (als Collateralen) hier vorkommen.

---

[1] Die Histiologie und Histiogenese der nervösen Centralorgane. Archiv für Psychiatrie etc. Bd. 4. 1874. S. 1ff.

Seine Beschreibung der Neuroglia in der Grofshirnrinde ist richtiger, als die Golgische. Er hebt ganz richtig hervor, dafs nur an der Oberfläche eine grofse Menge Deitersche Zellen vorkommen, in der Tiefe aber sind sie nach ihm um vieles seltner und erscheinen meist nur in Begleitung der Gefäfse. Er kennt also nicht das „zusammenhängende“ Neurogliageflecht in den tiefen Hirnrindenschichten, das Golgi annahm. Auch über die Körnerschicht des Kleinhirns urteilt er richtiger als letzterer und sagt darüber das einzige, was damals zu sagen möglich war, nämlich, dafs man über die Natur der „Körner“ nichts wisse. —

Als letzte Arbeit in dieser Gruppe muss die von Gierke[1] erwähnt werden. Von dieser Arbeit könnten wir eigentlich in unserer historischen Übersicht ganz absehen, denn irgend etwas wesentlich neues, was richtig wäre, findet sich in ihr nicht. Im Gegenteil sie enthält neben den wenigen richtigen Angaben, die noch dazu sämtlich schon bekannte Dinge betreffen, fast lauter ganz falsche Behauptungen, so dafs es geradezu unbegreiflich ist, dass diese Arbeit von den hervorragendsten Autoren immer mit besonders lobenden Zusätzen „gründlich“, „vortrefflich“ etc. bedacht zu werden pflegt. Es gehört in der That zu den Ironien der geschichtlichen Darstellungen, dafs die Arbeit von Frommann stets nur so nebenbei erwähnt wird, und die von Gierke als etwas ausgezeichnetes immer wieder hervorgehoben wird. Hier sei nur einiges aus seiner Arbeit mitgeteilt.

Die Deitersschen Zellen schildert Gierke ähnlich wie Golgi, Jastrowitz und Boll mit dem kleinen Unterschied, dafs er die „Zellfortsätze“ verzweigt sein lässt, und mit der Abweichung, dafs er sie für „verhornt“ hält (nach Kühne und Ewald). Neben diesen Zellen, deren Körper und deren Kerne nach ihm im Alter atrophiren können, nimmt er noch eine „Grundsubstanz“ als Bestandteil der Neuroglia an, die aber nicht, wie bei den älteren Forschern als körnig, sondern als glashell geschildert wird. Diese glashelle Grundsubstanz bildet nach ihm die Grundlage der grauen Substanz. Eine besonders grosse quantitative Entwicklung besitzt sie in den äussern Hüllen des Centralnervensystems, in der Grosshirnrinde und in der Substantia gelatinosa centralis. In der weissen Substanz ist sie sparsam (S. 459) — alles ganz willkürliche, unbegründete Behauptungen. Die „Grundsubstanz“ besitzt nach Gierke eine nicht ganz unbedeutende Elasticität (S. 464), aber nur im frischen Zustande. Einige Stunden schon nach dem Tode wird sie weicher und dadurch

[1] Die Stützsubstanz des Centralnervensystems. Archiv für mikroscopische Anatomie. Bd. 25. S. 441 ff.

wird nach ihm die Erweichung des Centralnervensystems bedingt — sonst nimmt man ja an, dafs diese cadaveröse Erweichung in der Erweichung des Myelins ihren Grund hat.

Die Stützsubstanz im allgemeinen (d. h. „Grundsubstanz" und Neuroglia) ist nach Gierke so verbreitet, dass sie überall im Centralnervensystem vorkommt und „kein noch so kleines Fleckchen zu finden ist, was derselben entbehrt" — auch das ist eine unbewiesene Behauptung. Einigermafsen, wenn auch nicht ganz richtig ist seine Schilderung der weifsen Substanz des Rückenmarkes, doch enthält sie nichts, was nicht Frommann schon besser geschildert hätte. Die Schilderung der grauen Substanz hat dieselben Fehler, wie die der früheren Autoren. Ganz unklar und schief dargestellt sind die Verhältnisse an der Medulla oblongata, bei der er kein Wort von den so auffallenden Verhältnissen an den Oliven sagt; nur die Ependymschicht schildert er besser als seine Vorgänger.

Was nun gar das Hirn anbelangt, so sind da alle Beschreibungen, so weit sie neu sind, ganz irrig, am Kleinhirn so falsch, dass man selbst aus der Abbildung (Fig. 21) garnicht herausbekommt, was er eigentlich gesehen hat. Auch in der Grofshirnrinde hat er die richtige Neuroglia garnicht gesehen. Was er als solche abbildet (Fig. 19 a), ist die zu einem Maschenwerk geschrumpfte „Molecularsubstanz". Das geht nicht nur aus seiner eignen Abbildung hervor, sondern auch daraus, dafs er sich auf eine ähnliche von Stricker als auf eine „sehr zutreffende" beruft. —

Diese letzterwähnte Abbildung ist zwei Arbeiten beigegeben, einmal der von Stricker und Unger „Untersuchungen über den Bau der Grosshirnrinde" [1] und sodann noch einmal der von Unger allein (Histologische Untersuchungen der traumatischen Hirnentzündung). In Betreff dieser Arbeiten genügt es wohl, die Schlufssätze der Arbeit von Stricker und Unger zu citieren (S. 156):

I. Die Ganglienzellen und ihre Axencylinderfortsätze (!) tragen Ausläufer, welche continuirlich in ein Netzwerk von Bindesubstanz übergehen.

II. Es giebt Übergangsformen von den Zellen der Bindesubstanz zu den Ganglienzellen.

Wer an diesen Sätzen noch nicht genug hat, mag die genannten zwei Arbeiten sowie die 32. Vorlesung in Strickers „Vorlesungen über allgemeine und experimentelle Pathologie" selbst nachlesen. —

---

[1] Wiener Sitzungsberichte. Band 80. 1879.

Die von Boll bereits ausgesprochenen Ideen bekamen nun aber eine viel bessere thatsächliche Grundlage in der wichtigen, geradezu epochemachenden Arbeit von Ranvier. [1] Das, was dieser Autor mitteilte, war viel wichtiger, als die Fragen nach der etwas mehr oder weniger reichlichen Zahl der Ausläufer, nach deren Verzweigung oder Nichtverzweigung etc.

Für die Aufklärung der wahren Natur der Deitersschen Zellen war Ranvier so zu sagen praedestiniert, da er nach seinen Arbeiten über das gewöhnliche Bindegewebe fast notgedrungen ein ähnliches Verhältnis der Zellen und Fasern auch im Stützgewebe des Centralnervensystems annehmen mufste. Er begnügte sich aber nicht mit einer blofsen Annahme, sondern brachte den thatsächlichen Nachweis dafür, dafs die sogenannte Deitersche Zelle ein Kunstprodukt ist, bei welchem die von der Zelle unabhängigen, aber von ihr wie von einem Centrum ausstrahlenden Fasern nur anscheinend vom Protoplasma ausgehen, in Wirklichkeit aber an dasselbe nur angelehnt sind.

Auch hier wieder war es eine besondere Methode und, wie wir gleich hinzusetzen wollen, eine besonders günstig wirkende Carminlösung, der er seine Erfolge verdankte.

Diese Methode bestand darin, dass er Rückenmarkstückchen auf 24 Stunden in Drittelalkohol brachte, dann zerteilte und die Bröckel in einem Reagenzgläschen mit destilliertem Wasser schüttelte, mit Picrocarmin färbte und dann absetzen liefs. Den Bodensatz nahm er mit einer Pipette auf und brachte ihn in ein neues Reagenzglas mit sehr verdünnter Überosmiumsäure. Wenn sich die Massen dann zu Boden gesetzt hatten, nahm er sie wieder heraus und untersuchte sie mikroskopisch. Auf diese Weise hatte er zuerst eine Dissociation und Färbung und dann eine definitive Fixierung der dissociierten Elemente erlangt.

An Praeparaten aus ausgebildeten Rückenmarken, die auf diese Weise hergestellt waren, fand er nun, dafs die „Zellfortsätze“ keine wirklichen Verlängerungen des Protoplasmaleibes seien, wie seit Frommann alle Autoren glaubten (aufser Boll), sondern von diesem differenzierte, wirkliche Fasern darstellten, welche den Zellleib durchsetzen, oder an ihn angelehnt sind. Sie strahlen von dem Zellleibe als Mittelpunkt nach allen Seiten (ungeteilt) aus, aber dieser Zellleib selbst setzt sich nicht einfach in sie fort, sondern stellt einen chemisch und morphologisch abgesetzten Körper dar.

Das gilt aber wohlgemerkt nur für die Neurogliazellen des fertigen Rückenmarks. Im embryonalen Zustande sind die Zellen wirklich sternförmig, und die Fortsätze

---

[1] 1) De la névroglie. Comptes rendus. 5. Juni 1892. 2) De la névroglie. Archives de physiologie normale et pathologique. 15. Februar 1883. Im Texte ist die letztere Arbeit zu Citaten benutzt, die erste war nur eine vorläufige Mitteilung.

sind einfache Verlängerungen des Zellleibes. Die Differenzierung der Fasern von letzterem erfolgt erst später, ganz wie beim gewöhnlichen Bindegewebe. —

So war denn eine ganz neue Auffassung des Neurogliagerüstes gegeben. Dieses besteht nach Ranvier also nicht aus Zellen allein, sondern aus Zellen und aus Fasern. Er weist auch ganz richtig darauf hin, dafs die bisherigen Resultate zu der Täuschung führen mussten, dass Zellen und Fasern eins seien, weil in Praeparaten aus Müllerscher Flüssigkeit die Refractionsindices der Fasern und des Zellleibes so ähnlich sind, dafs eine Abtrennung der erstern von dem letztern nicht möglich war.

Freilich war diese Zerzupfungsmethode nicht ausreichend, um über die Topographie der Neuroglia ins Klare zu kommen, ja sie hat sogar Ranvier an andern Stellen des Centralnervensystems im Stich gelassen, so dafs er die ganz irrige Meinung ausspricht, die Neurogliafasern des Gehirns von Erwachsenen schienen nicht aus dem embryonalen Stadium, d. h. dem der undifferenzierten Zellfortsätze herauszukommen. (S. 182.)

Die Ansicht von Ranvier hat sich absolut keiner Anerkennung zu erfreuen gehabt. Vollkommen für seine Auffassung ausgesprochen hat sich, abgesehen von einigen Ranvier nahe stehenden Gelehrten, eigentlich nur der Schreiber dieser Arbeit. Das Verdienst Ranviers wird in seinem ganzen Werthe erst später hervortreten.

Eine besondere Stellung in der Neurogliafrage nimmt, oder nahm wenigstens früher Schwalbe[1] ein, dessen Arbeit wir hier anschliefsen wollen. Er unterscheidet (S. 393) einen mesodermalen und ectodermalen Bestandteil der Stützsubstanz im Centralnervensystem. Als mesodermalen Bestandteil betrachtet er aufser hier und da vorhandenen elastischen (oder diesen nahe stehenden) Fasern vor allem die Neurogliazellen, die er den Wanderzellen an die Seite setzt. Sie haben nach ihm keine Ausläufer, aber auch keine Beziehung zur gliösen Intercellularsubstanz, so dafs seine Ansicht sowohl von der von Frommann, Deiters, Golgi etc. vertretenen, als von der Ranvierschen durchaus abweicht. Das, was er als Intercellularsubstanz bezeichnet, ist für ihn ectodermatischen Ursprungs, ebenso wie die Epithelzellen des Centralkanals. Sie ist in zweierlei Abarten vorhanden. Einmal als Nervenkitt (echte Neuroglia). Dieser ist eine durchaus homogene, weiche Substanz und enthält im natürlichen Zustande keinerlei Fasern. Die von anderen Autoren beobachteten Fasern sind Kunstprodukte, die durch cadaveröse Gerinnung oder

---

[1] 1) Handbuch der Augenheilkunde von Gräfe und Sämisch I. S. 342. Leipzig 1874. 2) Lehrbuch der Neurologie. Erlangen 1881. S. 393 ff.

durch coagulierende Agentien, z. B. durch Alcohol, hervorgebracht werden. Diese Substanz ist durchaus einer epithelialen Kittsubstanz zu vergleichen. Sie bräunt sich auch wie diese mit Silbernitrat. Als fernere ectodermatische Stützsubstanz ist eine in der That aus sehr feinen, eng verwebten Fäden bestehende, daher eine Granulierung vortäuschende Substanz anzusehen, die er auch als „granulierte Substanz“ bezeichnet. Sie findet sich in besonderen Schichten, an verschiedenen Stellen des Rückenmarks, an der Oberfläche des Grofs- und Kleinhirns und in der Retina. Diese Substanz ist als Hornspongiosa aufzufassen, entsprechend den Angaben von Ewald und Kühne.

Es braucht kaum hervorgehoben zu werden, und wird sich im speziellen Teile noch weiter erweisen, dafs diese Ansichten nicht aufrecht zu halten sind. Schwalbe dürfte wohl selbst auch jetzt von ihnen zurückgekommen sein. Immerhin ist es bemerkenswert, dafs er gleich Ranvier, die Neurogliazellen als solche fortsatzlos sein läfst. Die auch von Ranvier dargestellten Fäden aber hat er nicht zu Gesichte bekommen.

Von neuern Schriftstellern, die mit andern Methoden, als den bisherigen (abgesehen von der Golgischen) gearbeitet haben, sei zunächst Luigi Maria Petrone erwähnt. Er ist der erste gewesen, welcher Säurefuchsin und Picrinsäure zur Neurogliafärbung benutzt hat, eine Färbung, die dann später (1889) von van Gieson[1] weiter ausgebildet wurde. Die Methode des letzteren ist dann von Kultschitzky[2] ganz wenig modifiziert worden. Petrone[3] hat auch mit der Golgischen Imprägnation und mit Carmin-

[1] Laboratory notes of technical methodes for the nervous system. New-York medical Journ. 1889.

[2] Über eine Färbungsmethode der Neuroglia. Anatomischer Anzeiger. 8. Bd. 1893.

[3] Gazzetta degli Ospidali 1886—1886, Gazzetta Lombarda 1886—1887. (Vorläufige Mitteilungen, mir nicht zugänglich). Sulla struttura della nevroglia dei centri nervosi cerebro-spinali, Gazzetta degli Ospidali 1888. Diese leztere Arbeit trägt die Überschrift: Dal Senckenbergschen Pathologischen Institut von Frankfurt a. M., Prof. Weigert, und ist aus Breslau datiert, wo sich Petrone damals aufhielt. Man könnte daraus schliefsen, dafs ich irgend ein Verdienst bei dieser Arbeit hätte, zumal Petronc am Schlusse bemerkt, dafs er die Structuren der Medulla oblongata, des Isthmus des Gehirns und aller Hirnteile für sich in Anspruch nehme, so dafs mancher glauben könnte, ich hätte wenigstens an den Resultaten, die er vom Rückenmark etc. schildert, Anteil. Aber auch das ist nicht richtig. Ich bin an der Arbeit nicht nur unbeteiligt, sondern habe auch die betreffenden Präparate garnicht gesehen. Ja, ich mufs sogar ausdrücklich hervorheben, dafs ich von dem Petroneschen Aufsatz erst vor ganz kurzem Kenntnis genommen habe, sonst hätte ich in meinem Artikel „Technik“ der Merkel-Bonnetschen „Ergebnisse“ gewiss ihm die Priorität in Betreff der Säurefuchsin-Pikrinsäure-Färbung gewahrt. Die Gründe für diese höchst sonderbar erscheinenden Dinge sind recht trauriger Art gewesen, entziehen sich aber der Öffentlichkeit.

Pikrinsäure gearbeitet. Für die eigentlichen Färbungen benutzte er Präparate, die ebenso vorbereitet waren, als wenn sie zu meiner Kupfer-Haematoxylinmethode benutzt werden sollten.

Petrone unterscheidet zwei Arten von Neurogliazellen, die eigentlichen Deitersschen Zellen und die „Lamellen“, platte rechteckige Zellen ohne Ausläufer, die besonders an den Kreuzungsstellen der Nervenfasern vorkommen. Für erstere nimmt er gegen Ranvier Partei, und glaubt, was ganz irrtümlich ist, dafs Ranvier durch platte Zellen, die mit Neurogliafasern zufällig in Verbindung standen, getäuscht worden sei. Für die weifse Substanz bestreitet er die Anastomosierung der Neurogliafasern, hingegen glaubt er, dafs in der grauen das „Schultze-Köllikersche Netz“ vorkäme. Freilich ist er sich klar darüber, dafs für die graue Substanz seine Methode, die ja durchaus nicht electiv färbt, unzureichend sei „wegen der Unmöglichkeit, in der wir uns infolge der gegenwärtigen Beobachtungsmittel befinden, die Neuroglia von den andern, sie umgebenden Substanzen zu unterscheiden.“

In der That ist auch für ihn die Substantia gelatinosa Rolando reicher an Neurogliazellen, als die übrige graue Substanz, was ganz irrig ist, am Klein- und Grofshirn findet er an der Rinde in der oberflächlichsten Schicht keine eigentliche Neuroglia, wohl aber „Lamellen“ und Bindegewebe, das von der Pia mater herabsteigt. Die dichte Anhäufung der Neuroglia am Ependym ist ihm entgangen etc.

Hingegen hat er merkwürdiger Weise etwas gesehen, was vor meiner Veröffentlichung 1890 niemand anders gesehen hatte, nämlich die so dichte Neurogliamasse in den Oliven, und ich bedauere, dass ich 1890 noch nicht seine Arbeit kannte (vgl. die Anmerkung S. 24), sonst hätte ich das damals schon konstatiert.

Die weifsen Substanzen hat er möglicherweise ziemlich richtig geschildert, doch ist das nicht sicher, zumal da gar keine Abbildungen beigegeben sind.

Im Jahre 1890 habe ich selbst [1] dann eine vorläufige Mitteilung über die Resultate meiner neuen Färbung gegeben, die sich damals noch im Stadium des „beinah fertig“ befand, in einem Stadium, aus dem sie absolut nicht herauszubringen war. Ich konnte aber doch schon einiges von Thatsachen mitteilen. Einmal konnte ich mich durchaus der oben besprochenen Ansicht von Ranvier anschliefsen, dafs die Neurogliafasern keine „Zellfortsätze“ sind. Als ganz neu müssen sodann die Mitteilungen über die topographische Verteilung

[1] Bemerkungen über das Neurogliagerüst des menschlichen Centralnervensystems. Anatomischer Anzeiger 1890, S. 543 ff.; und: Zur pathologischen Histologie des Neurogliafasergerüsts. Centralblatt für allgemeine Pathologie und pathologische Anatomie 1890, S. 729 ff.

in der grauen Substanz des Rückenmarks bezeichnet werden, namentlich die Thatsache der geringen Neurogliamenge in der Substantia gelatinosa Rolando. Auch die Körnungen am freien Rande der Epithelien waren bis dahin unbekannt. Ebenso (bis auf die, mir noch nicht bekannte Notiz bei Petrone) der aufserordentliche Reichtum der Oliven an Neurogliafasern. Ich schilderte kurz die Verhältnisse der Rindenschichten, der Substantia grisea centralis, des obliterierenden Centralkanals im Rückenmark, hob ebenfalls, wie schon frühere Autoren hervor, dass die einstrahlenden „Piafortsätze" kein Bindegewebe, sondern Neuroglia wären, und brachte zu den bisher bekannten Unterschieden des Bindegewebes und der Neuroglia noch einen neuen tinctoriellen hinzu. Auch die „Körbe" um die Purkinjeschen Zellen und um die Vorderhorn-Zellen, sowie die Verhältnisse am Opticus skizzierte ich etc.

Lavdowsky,[1] dessen Arbeit im Jahre darauf erschien, hat eine grosse Anzahl Methoden (auch die Golgische) benutzt, Methoden, die hauptsächlich auf der Anwendung „saurer" Anilinfarben beruhen. An Schnittpräparaten ist er augenscheinlich nicht glücklich gewesen, denn die topographischen Verhältnisse kommen in den Abbildungen nur lückenhaft heraus. Er hält die Neurogliafasern für hohl, und ist der Meinung, dafs sie echte Zellausläufer (gegen Ranviers Auffassung) sind. In der grauen Substanz bildet die Neuroglia ein richtiges „Netz", in der weifsen nicht, so dass er hier eine ähnliche Anschauung wie Petrone vertritt. Die Unterschiede der topographischen Ausbreitung der Neuroglia in den verschiedenen Teilen der grauen Substanz sind ihm entgangen. Auch er klagt (S. 289) darüber, dafs man die Neurogliafasern da, wo sie mit Fortsätzen der Nervenzellen und Nervenfasern untermischt sind, nicht von diesen unterscheiden kann.

Die hier noch zu erwähnende Arbeit von Popoff,[2] der mit der durch Kultschitzky modifizierten allbekannten van Giesonschen Methode gearbeitet zu haben scheint, ist mir nur aus dem sehr kurzen Referat der Revue neurologique, Band 1, 1893, S. 557, bekannt. Er nimmt corpuscules ramifiés et non ramifiés, in der Neuroglia an. Die Ramificationen teilen sich nicht und anastomosieren nicht, sie sind „divisions protoplasmatiques ordinaires des cellules" (also abweichend von Ranviers Ansicht) und nicht hohl, wie Lavdowsky meint. Ausserdem kommen auch freie Fasern vor. In der grauen Substanz sind die

---

[1] Vom Aufbau des Rückenmarks. Archiv für mikroscopische Anatomie. Bd. 38 (1891). S. 263 ff.

[2] De la névroglie et de sa distribution dans les régions du bulbe et de la protubérance chez l'homme adulte. Arch. de psych., de neurologie et de médecine légale. 1893. 11. Bd., p. 1.

Maschen der Neuroglia zwischen den nervösen Elementen weiter als in der weissen, (was nicht so allgemein richtig ist), doch variiert die Dichte der Neuroglia. Am dichtesten ist sie in der Olive (Bestätigung meiner Angabe) der „gelatinösen" Substanz, im Hypoglossus-Vagus- und Facialis-Kern, geringer im Kern des Acusticus, des Abducens und den zerstreuten grauen Massen im Pons, im Trigeminuskern etc. Einige der Bemerkungen dieses Referats kommen später noch zur Erwähnung.

Schlussbemerkungen.

Hiermit wollen wir unsere historische Übersicht schliessen. Sie macht durchaus keinen Anspruch auf Vollständigkeit. Einmal sind mir gewifs eine Anzahl Veröffentlichungen entgangen, andere konnte ich nicht nachsehen, noch andere waren schon gar zu „historisch" geworden, wie die von Jacubowitsch u. a. Wir haben ferner alles weggelassen, was sich auf die chemischen und entwicklungsgeschichtlichen Verhältnisse bezieht, da diese Arbeiten, so weit nötig, später an geeigneterer Stelle besprochen werden sollen.

Auch die mit Hilfe der Golgischen Methode gewonnenen Resultate werden besser im Verein mit unsern eignen Untersuchungsergebnissen später im einzelnen besprochen, aber einige allgemeine Betrachtungen über das Verhältnis dieser Methode zur Neurogliaforschung wollen wir als Schlussbemerkung hier anschliefsen.

Die Erfolge der Golgischen Methode gerade in ihrer Anwendung auf die Neuroglia sind ungemein überschätzt worden. In Wirklichkeit sind sie auch nicht im entferntesten mit den immensen Fortschritten zu vergleichen, die wir derselben Methode in Bezug auf die nervösen Elemente verdanken. Was die letzteren anbelangt, so ist die Golgische Methode im wahren Sinne epochemachend gewesen, aber wenn manche Gelehrte auch in der Geschichte der Neuroglia eine neue Epoche seit Anwendung der Golgischen Imprägnation datieren, der gegenüber die Zeit vorher wie eine praehistorische Periode erscheinen soll, — so ist das ungemein übertrieben.

Von wirklichen Erfolgen hat die Golgische Methode nur solche auf dem Gebiete der Entwicklungsgeschichte aufzuweisen. Für die Lehre von der Anordnung der Neuroglia im ausgebildeten Körper hingegen sind die Resultate äufserst dürftige, ja vielfach geradezu falsche gewesen, und die weitgehende Überschätzung dieser Resultate ist nur dadurch zu erklären, dafs man sich der Grenzen, welche diese, wie jede Methode hat, nicht bewufst war. Erst ganz neuerdings fangen die Mängel der Methode an, hier und da erkannt zu werden,

so von Lenhossék, Greeff und Retzius, aber die Bedeutung der Silberbilder wird immer noch wesentlich überschätzt.

Die Gründe dafür, warum mit der Golgischen Methode für die wichtigste Frage, die Topographie der Neuroglia, nur dürftige Resultate zu erlangen waren, liegen auf der Hand. Vor allem konnte sie der Hauptanforderung, die man für die Lehre von einer Stützsubstanz stellen mufs, nicht entsprechen: sie konnte das Gerüst nicht im Zusammenhange, d. h. vollständig, darstellen. Dieser für die Ergründung einer Stützsubstanz fundamentale Fehler kommt bei den nervösen Elementen, bei denen es wesentlich auf die Beziehung der einzelnen Elemente zu einander ankommt, nicht nur nicht in Betracht, sondern er hört hier auch auf, ein Fehler zu sein und wird ein Vorteil, da man bei einer vollständigen Darstellung des Nervengewebes sich garnicht mehr in dem Gewirr desselben „auskennen“ würde. Bei einer Stützsubstanz aber mufs man eine wenigstens stellenweise Vollständigkeit der Elemente durch eine brauchbare Methode erreichen können. Das kann aber die Golgische Methode nicht leisten. Abgesehen davon, dafs sie immer nur unvollkommen, hier und da einen Bestandteil der Neuroglia imprägniert, sind die imprägnierten Bestandteile nur die Zellen und die unmittelbar von ihnen ausstrahlenden Fasern („Fortsätze der Zellen“). Alle von den Zellen getrennten Fasern sind garnicht mehr als Neurogliaelemente zu diagnostizieren.

Auf einem einigermafsen vollständig gefärbten Präparat kann man sich aber davon überzeugen, dafs dadurch die Mehrzahl der Neurogliafasern sich der Kenntnis entzieht, selbst wenn man die grofse Dicke, welche nach Golgi imprägnierte Schnitte haben dürfen, in denen also möglichst viele Fasern bis zu den Zellen verfolgt werden können, in Betracht zieht.

Die Golgische Methode hat aber noch einen andern Nachteil für die Forschung gehabt. Sie stellt, wie erwähnt, nur die Zellen und die ihnen anliegenden Fasern dar. Ganz abgesehen nun davon, dafs bei der entstehenden Silhouette die chemisch-physikalischen Unterschiede der Fasern von den Zellen verschwinden, und so Trugbilder von Zellen mit „Fortsätzen“ entstehen, die uns später ausführlicher beschäftigen werden, so wurde durch die Einseitigkeit der Methode die Aufmerksamkeit ganz von den Fasern („Zellfortsätzen“) abgelenkt und auf die „Zellen“ konzentriert. Es hat nun sicherlich auch ein Interesse, die Formen der (Schein-) Zellen der Neuroglia nach der Golgischen Methode zu studieren, aber für die Funktion wesentlicher sind doch auch hier, wie beim Knochen, bei den elastischen und Bindewebsmassen, die gerüstbildenden Elemente, die Neuroglia-

fasern, („Zellfortsätze“ nach den meisten Autoren), ihre Massenhaftigkeit, ihr Verlauf und die Form ihrer Verflechtungen, und für diese hatte man unter Anwendung der Golgischen Methode kaum noch Interesse, oder höchstens ein Interesse, das sich ganz gleichgiltigen Fragen fast allein zuwandte, und die eigentlich wichtige Topographie, wenn auch nicht vollkommen ignorierte, so doch sehr vernachlässigte. —

Unter diesen Umständen musste es sehr erwünscht sein, eine Methode zu finden, welche gerade die Topographie der Neuroglia zu ergründen ermöglichte. Eine solche Methode musste gar viele Anforderungen erfüllen, wenn sie ihren Zweck nicht verfehlen sollte. Sie musste das Stützgerüst deutlich und isoliert, d. h. ohne Färbung der nervösen Elemente, vor allem ohne eine solche der Axencylinder, tingieren. Sie mufste das Gerüst vollständig darstellen und sollte eigentlich an richtig behandelten Praeparaten nie versagen.

Das war eine schwierige Aufgabe, die lange, lange Jahre unausgesetzter Arbeit erforderte, und die vielleicht noch nicht ganz erfüllt ist. Ob die von uns benutzte neue Methode gegenüber den früheren Vorteile bietet, die die lange Arbeit lohnen, das mögen die Leser nach Kenntnisnahme der folgenden Abschnitte entscheiden. Hier seien vorerst die Mängel der Methode gleich von vornherein erwähnt.

Die Methode ist unfähig, die Entwickelungsgeschichte der Neuroglia weit zurückzuverfolgen. Die Methode stellt ferner, abgesehen von den Kernen der Neurogliazellen, nur die, wie wir sehen werden, in besonderer Weise differenzierten Fasern dar. Wenn daher, was a priori durchaus nicht bestritten werden kann, Zwischensubstanzen im Centralnervensystem existieren, welche solcher differenzierter Fasern entbehren, so entgehen diese bei Anwendung der Methode vollkommen der Kenntnisnahme.

Aber so sehr diese Mängel für den Embryologen und den normalen Histologen von Bedeutung sein mögen, für den pathologischen Anatomen kommen sie kaum in Betracht. Die Methode ist aber gerade für die pathologische Anatomie gesucht worden. Ehe sie jedoch für diese zur Anwendung kommen konnte, mufste erst nachgeforscht werden, wie sich denn die normale Topographie der Neuroglia mit der neuen Methode darstellte. Das war eigentlich nur eine Vorarbeit, ein Nebenzweck der Arbeit, aber der Verfasser will es gern gestehen, dafs ihm die Verfolgung dieses Nebenzweckes von ganz besonderem Interesse gewesen ist.

2. Abschnitt:

Die Neurogliafasern in ihrem Verhältnis zu den Zellen.

Färbt man Präparate nach der neuen, am Schlusse dieser Abhandlung mitgeteilten Methode, so sieht man eine grofse Menge blau gefärbter Fasern. Aufser diesen Fasern sind (eventuell die roten Blutkörperchen in den Gefäfsen und) die Kerne aller Zellen gefärbt.

Von den Zellleibern sind die der gröfseren Ganglienzellen gelb gefärbt, und man erkennt an ihnen sehr schön die von Nissl so genau studierten Zeichnungen, die sich in dunklerer, mehr bräunlicher Färbung in dem übrigen, helleren Protoplasma deutlich abheben (Taf. II, Fig. 1a). Auch die gröberen Zellausläufer und Axencylinder sind gelblich gefärbt, die feineren sind unsichtbar. Ebenso sind die Zellleiber der kleinen Ganglienzellen nur schwach gelblich oder garnicht tingiert; die Leiber derjenigen Zellen, die man als Neurogliazellen auffafst, sind ebenfalls ungefärbt, also unsichtbar.

Uns interessiert vorläufig nur das Verhältnis jener blauen Fasern zu den gleichfalls blau gefärbten Kernen; andere untergeordnetere histologische Eigentümlichkeiten der ersteren werden wir in einem besonderen Kapitel besprechen. Unter den Kernen sind solche, die man nach den geltenden Auffassungen nur als Kerne von Gliazellen auffassen kann, weil sie an Stellen liegen, wo Ganglienzellen, so viel man weifs, nicht vorkommen, z. B. in der weifsen Substanz des Rückenmarks. Diese Kerne präsentieren sich in zweierlei Haupttypen: gröfsere bläschenförmige Kerne mit körnig aussehendem Chromatin und kleinere, in denen das Chromatin eine homogene dunkele Masse darstellt.

So verschieden diese beiden Kernarten auch aussehen, so giebt es doch Fälle, in denen man nicht weifs, zu welcher der beiden Unterabteilungen man ein bestimmtes Kernexemplar rechnen soll, so dafs man, wenn man Lust hat, „Übergänge“ zwischen beiden Kernformen statuieren kann.

Von diesen beiden Kernformen sind es nun viele der helleren, bläschenförmigen, punktierten Gebilde, welche zu den Fasern in charakteristischer räumlicher Beziehung stehen.

Nur ausnahmsweise, vielleicht auch gar nicht, thun dies die kleineren Kerne mit dunklerer Färbung.

Die Fasern gehen nämlich vielfach bis dicht an den (hellen) Kern heran oder sind von ihm nur durch einen kleinen Zwischenraum getrennt, den man durch (ungefärbtes, daher unsichtbares [1]) Protoplasma sich ausgefüllt zu denken hat. Sie gehen dabei teils neben dem Kern vorbei nach der anderen Seite in ziemlich gerader Linie gleichmäfsig fort, teils biegen sie am Kern mit mehr oder weniger scharfem Bogen ab, um ebenfalls jenseits des Kerns weiter zu verlaufen (vgl. Taf. I, Fig. 1 A—E). Ein Teil der Fasern, der im Schnittpräparat oberhalb oder unterhalb des Kernes verläuft (nicht wie die bisher erwähnten seitlich von diesem), (z. B. Taf. I, Fig. 1 A, D, E) verhält sich im übrigen ebenso, nur mufs man natürlich, um die scharfe Absetzung zwischen Faser und Kern zu bemerken, die Schraube des Mikroskops spielen lassen. Wieder andere Fasern kann man nur bis in die Nähe des Kerns verfolgen (vgl. Taf. I, Fig. 1 B), wo sie scharf enden, ohne sich über den Kern hinaus fortzusetzen; doch sind diese seltener als die, die sich jenseits des Kerns weiter ins Gewebe verfolgen lassen. Ob diese nur bis in die Gegend des Kerns reichenden Fasern wirklich hier enden, oder ob man es nur mit solchen zu thun hat, deren (abgebogene) Fortsetzung durch die Schnittführung unterbrochen wurde, mufs dahingestellt bleiben.

Sehr charakteristische Bilder entstehen nun dann, wenn, wie sehr häufig, die Fasern in ganzen Büscheln um den Kern gelagert sind, so dass eine spinnen-, pinsel- oder sternförmige Figur entsteht, in deren Mitte der Kern mit seinem zu supponierenden, unsichtbaren Protoplasma liegt (vgl. Tafel I, Fig. 1 [2]). Uebergänge der Fasern in dies unsichtbare Protoplasma sind nicht zu bemerken. Sie müssten sich in der Weise geltend machen, dafs die Fasern allmählich in der Nähe des Kerns blasser würden und sich dann in dessen Umgebung verlören. Das ist aber niemals der Fall.

Es gehört sehr wenig Phantasie dazu, um in diesen Kerncentren mit den strahlenförmig an sie angelagerten Fasern jene Gebilde wieder zu erkennen, die man als Deiterssche Zellen, Neurogliazellen, Spinnenzellen, Pinselzellen, Astrocyten, Gliaecyten etc. beschrieben hat. Ganz besonders macht sich dieser Eindruck dann geltend, wenn der Zwischenraum

---

[1] Das Protoplasma ist durch andere Methoden, z. B. durch neutrales Karmin, in unseren Präparaten sichtbar zu machen: es fehlt also nicht etwa.

[2] Ähnliche Bilder finden sich vielfach andeutungsweise in unseren Abbildungen. Da diese letzteren sonst aber möglichst ohne Anwendung der Schraube gezeichnet sind, so ist die Spinnenform etc. nicht so deutlich, wie in diesen mit Schraubendrehung gezeichneten Figuren.

zwischen den Faserbüscheln und dem Kern verschwindend klein ist, so dass man schon genauer zusehen muss, um die scharfe Absetzung der Fasern wahrzunehmen. Stellt man unter solchen Verhältnissen die Mikroskoplinse nicht scharf ein, so glaubt man ohne weiteres einen „Astrocyten" vor sich zu haben. Auch an Photographien solcher Präparate, wenn sie nicht aufserordentlich scharf ausfallen, sehen die Kerne mit ihren angelegten Fasern genau wie Deiterssche Zellen aus. —

An vielen andern Stellen tritt jedoch die Beziehung der Fasern zu den Kernen nicht in so charakteristischer Form auf. Teils liegen die Kerne in einem solchen Gewirr von Fasern, dafs man über eine Gruppierung der letzteren nicht ins klare kommen kann, teils tritt eine nachweisbare Beziehung von Fasern zu Kernen auch an solchen Stellen nicht hervor, an denen das Gewirr gar nicht so grofs ist. In letzterm Falle kann man sich doch aber manchmal noch überzeugen, dafs auch hier verlarvte „Astrocytenbilder" vorliegen, z. B. durch Änderung der Schnittrichtung, indem die Ausstrahlung der Fasern in einer andern Ebene, als man gerade vor sich hat, erfolgt. So sieht man im Rückenmark, wie schon Golgi[1] erwähnt, diese Bilder auf Vertikalschnitten reichlicher, als auf Horizontalschnitten

Endlich gelingt es auch, diese „Astrocytenbilder" noch manchmal herauszubekommen, wenn man die Leiber der Neurogliazellen z. B. durch neutrales Karmin färbt — eine Doppelfärbung, die freilich für die feineren Fasern nicht günstig ist. Dieses Mittel hilft dann, wenn der Raum zwischen Kern und Fasern zu gross ist, um die Beziehungen beider hervortreten zu lassen, d. h. wenn der Zellleib, der ohne Doppelfärbung unsichtbar bleibt, zu umfangreich ist.

Aber trotz alledem kann man wohl sagen, dafs sehr viele Kerne zwischen den Fasern (namentlich vielleicht sämmtliche kleine, dunkelgefärbte) sich in keiner Weise als Centra von Strahlensystemen erkennen lassen. Dafs umgekehrt bei weitem nicht alle Fasern sich bis zu Kerncentren verfolgen lassen, ist bei der grofsen Länge derselben und bei dem Umstande, dafs sie nicht in ihrer ganzen Ausdehnung in einer Schnittebene liegen können, nicht zu verwundern, denn die Berührungsstelle mit den Kernen ist doch immer nur ein ganz kleiner Abschnitt ihres Verlaufs.

Trotzdem so viele Kerne ohne charakteristische Beziehung zu den Fasern sind, trotzdem die meisten Fasern keine Beziehung zu den Kernen erkennen lassen, wird man doch nicht umhin können, alle die nach unserer Methode gefärbten Fasern für identisch

---

[1] Gesammelte Abhandlungen. S. 158.

mit den Gebilden zu halten, die man seit Frommann für Ausläufer der Neurogliazellen hält.[1]

Für diejenigen Fasern, welche strahlen-, spinnen- oder pinselförmig um Kerne gruppiert sind, ist die Ähnlichkeit des Gesammtbildes mit den typischen Astrocyten, wie wir schon erwähnten, eine so in die Augen springende, dafs an der Identität der „Ausläufer“ und der „Fasern“ nicht gezweifelt werden kann. Namentlich, wenn man sich den kleinen Zwischenraum zwischen Kern und Fasern ausgefüllt denkt, so gleicht das Bild ganz einer durch Isolation gewonnenen Deiters schen Zelle oder einer Golgischen Silhouette.

Für die übrigen Fasern, die man nicht zu einem Kerne in strahlenförmige Anlagerung treten sieht, wird man aber schon von den Überlegungen ausgehend, die Frommann vor mehr als dreifsig Jahren angestellt hat, (vergl. die historische Uebersicht), das Urteil dahin abgeben, dafs sie mit den eben erwähnten, in so charakteristischer Weise um die Kerne gruppierten Fasern identisch sind. Sie sind diesen in ihrem ganzen Aussehen, in ihrer Färbbarkeit etc. so ähnlich, dafs sie schwerlich verschiedener Art sein können. Aber für die Gleichheit der freien Fasern mit den Astrocytenfasern spricht auch noch ein anderer Umstand.

Die neue Methode weist nämlich überall da, wo nach den alten Methoden „Ausläufer von Neurogliazellen“ in bestimmter Anordnung dargestellt wurden, die „Fasern“, wenn auch reichlicher, so doch in derselben Anordnung nach. Das gilt zunächst für die Rindenschicht, die weisse Substanz und die Umgebung des Centralkanals im Rückenmark. Es sei ferner an die Bergmannschen Fasern im Kleinhirn, an die oberflächliche Rindenschicht im Grofshirn und an den Optikus erinnert. Auch die Golgische Methode, die freilich überall nur Bruchstücke des reichen „Zellausläufergeflechts“ zu Tage fördert, läfst an der Gleichheit der Anordnung nicht zweifeln, über die Reichlichkeit des Geflechtes freilich gestattet sie kein sicheres Urteil.

Wir können demnach, wenn wir aus dem Vorstehenden das Facit ziehen, eins wohl mit Sicherheit sagen:

Die von uns dargestellten Fasern sind kein Novum, kein bisher unbekanntes Strukturelement, sondern sie sind identisch mit

---

[1] Ob man wirklich ein Recht hat, die Zellen und ihre Ausläufer, also unsere „Fasern“, für Neurogliazellen zu halten, das werden wir später ausführlich erörtern. Vorläufig bezeichnen wir diese Zellen nur den geltenden Anschauungen folgend als Neurogliazellen.

dem, was man bisher als Ausläufer der Deitersschen Zellen beschrieben hat.

Wir hätten sonach von den bei unserer Methode gefärbten Elementen zunächst die Fasern mit Gebilden identifiziert, die durch die bisherigen Untersuchungsmethoden längst bekannt waren. Von den Kernen aber, oder was dasselbe besagt, von den Zellen, denen diese Kerne ja zugehören müssen, entsprechen nur diejenigen sicher den Kernen und Zellleibern der „Deitersschen Zellen", welche in typischer Weise mit strahlenförmig angeordneten Fasern in Beziehung stehen. Wir haben aber gesehen, dafs (auch abgesehen von den als Kerne von Ganglienzellen zu diagnostizierenden) eine grosse Menge von Kernen zwischen unseren Fasern darinliegt, in deren Umgebung die Fasern ganz regellos verlaufen; und doch müssen wir nach den geltenden Anschauungen diese Kerne an vielen Stellen sicher für Neurogliakerne halten, denn sie liegen, wie schon erwähnt, zum grofsen Teil an Orten, wo nach den bisherigen Erfahrungen Nervenzellen nicht vorkommen.

Wie soll man solche Neurogliakerne resp. -Zellen auffassen?

Hierbei sind zwei Möglichkeiten vorhanden. Die eine wäre die, dafs beim ausgebildeten Menschen eben viele der Neurogliazellen ihren Charakter als Astrocyten verloren haben. Eine ähnliche Auffassung findet sich schon bei Jastrowitz und Boll, in neuerer Zeit z. B. bei Petrone und Popoff. Auch Kölliker sagt ausdrücklich[1]: „Ferner mufs ich sagen, dafs solche freie Zellkörper doch zu häufig und mit zu bestimmten Formen sich finden, als dafs man sie nur für zufällig abgelöste Bestandteile der Golgischen Zellen" (d. h. der Astrocyten) „halten könnte."

Die meisten anderen Forscher freilich, die mit der Golgischen Methode gearbeitet haben, glauben nicht an diese „fortsatzlosen" Zellen, — aber die Golgische Methode macht diese eben nicht kenntlich, und so entgehen sie der Beobachtung.

Wenn wir diese Möglichkeit zugeben, so müfsten wir sogar sehr reichliche Neurogliazellen als nicht zum Typus der Astrocyten gehörig betrachten, genau wie dies Jastrowitz und namentlich Boll schon geschildert haben.

Es wäre aber noch eine zweite Möglichkeit vorhanden, nämlich die, dafs viele dieser Zellen Astrocyten im alten Sinne des Wortes wären, d. h. dafs sie nicht mit

---

[1] Handbuch der Gewebelehre des Menschen. 6. Auflage. Leipzig 1893. 2. Band, S. 150.

differenzierten, an sie nur angelehnten Fasern im Verhältnis eines Strahlencentrums stünden, sondern dafs sie nichtfaserige, d. h. protoplasmatische Ausläufer besässen. Solche protoplasmatische, also echte Ausläufer sind aber, wie wir sehen werden, durch unsere Methode nicht sichtbar zu machen, wir können daher über ihre An- oder Abwesenheit kein Urteil abgeben und müssen die Entscheidung über diese zweite Möglichkeit offen lassen.

Wir haben soeben einen Gegensatz zwischen „Astrocyten im alten Sinne“ und unseren astrocytenähnlichen Gruppierungen der Fasern um die Kerne statuiert und haben im ersteren Falle von echten, d. h. protoplasmatischen Ausläufern, im letzteren von differenzierten, nur angelehnten Fasern gesprochen.

Wie wir in der historischen Übersicht gesehen haben, haben so ziemlich alle Autoren die Anschauung, dafs ein solcher Gegensatz garnicht existiert, sondern dafs auch im ausgebildeten Körper, von dem hier allein die Rede ist (abgesehen von den fortsatzlosen Zellen), nur Astrocyten mit echten Ausläufern vorkommen. Nur Ranvier hat (freilich blofs für das Rückenmark) die Ansicht ausgesprochen, dafs solche Neurogliazellen mit echten Fortsätzen zwar im Embryo vorkommen, dafs aber sonst die „Astrocyten“ Gebilde mit nur angelehnten, differenzierten Fasern darstellen.[1]

Wer hat nun Recht? Ranvier (für das Rückenmark) und der Schreiber dieses (für das ganze Centralnervensystem) auf der einen, oder alle anderen Autoren seit Frommann auf der anderen Seite?[2]

Gegen Ranviers Lehre sind mancherlei Einwände erhoben worden, namentlich bestritt Golgi[3] sogar die Thatsächlichkeit von Ranviers Befunden.

---

[1] Boll ist zwar in gewisser Beziehung ähnlicher Ansicht wie Ranvier gewesen, aber war sich doch nicht so klar, wie dieser, über den prinzipiellen Gegensatz der Fasern und Zellausläufer. In seinem Aufsatze nennt er die Fasern doch immer „Zellfortsätze“.

[2] Wegen Lloyd Andriezen vgl. S. 38, Anm. Es mufs ganz besonders darauf hingewiesen werden, dafs sowohl bei Ranvier, als bei uns, der Kern dieser im Centrum von strahlig angelehnten Fasern liegenden Zellen ausgezeichnet sichtbar ist. Dieser Hinweis ist deshalb notwendig, weil vor kurzem Paladino gesagt hat (Bolletino della R. Academia medica di Roma, 1891. Fasc. II, S. 8 des Sep.-Abdr.), Ranvier und ich hätten alternde Zellen vor uns gehabt, bei denen der Kern fehlte. Diese Meinung von Paladino ist um so merkwürdiger, als ja ein Blick auf die Ranvierschen Zeichnungen, in denen die Kerne grofs und deutlich abgebildet sind, die Irrtümlichkeit einer solchen Annahme aufs klarste beweist.

[3] Über die feinere Anatomie des Centralnervensystems (1885) in den gesammelten Abhandlungen. Jena 1894. S. 157.

Golgi giebt an, dafs er genau nach Ranviers Vorschrift sich Präparate hergestellt und doch niemals etwas anderes an den „Astrocyten“ gefunden habe, als dieselben Zellausläufer, die er auch in seinen eigenen, nach anderen Methoden hergestellten Präparaten gesehen hatte.

Durch unsere Methode ist aber ganz sicher nachzuweisen, dafs Ranvier doch richtig gesehen hat, so dafs diesen positiven Resultaten gegenüber das negative, das Golgi erhalten hat, nicht in Betracht zu kommen brauchte. Aber bei einem so hervorragenden Forscher mufs man sich doch wohl fragen, warum es ihm wohl nicht geglückt sein mag, die doch sicher richtigen Bilder von Ranvier zu Gesichte zu bekommen? Jedenfalls mufs Golgi bei seiner Nachprüfung irgend etwas anderes gemacht haben, als Ranvier. Da sonst eine Abweichung kaum möglich war, so darf man wohl die Vermutung aussprechen, dafs die Verschiedenheit im Golgischen und im Ranvierschen Verfahren in der Picrocarminfärbung zu suchen sein dürfte. „Picrocarmin“ und „Picrocarmin“ ist eben etwas ganz verschiedenes. Wenn man von der Kernfärbung absieht, die man mit einiger Sicherheit erreichen kann, so färbt das eine Picrocarmin so, das andere anders, je nach dem Präparate, das man gerade besitzt, und das ist der Grund, warum dieser Farbstoff jetzt schon so ziemlich aufser Gebrauch gesetzt ist, zumal man ja auch für die Kernfärbungen viel bessere andere Carmine hat. Wahrscheinlich war nun das von Ranvier benutzte Carmin so abgestimmt, dafs es die Fasern, aber nicht, oder wenig, die Zellleiber färbte, während Golgis Picrocarmin beide in gleichem Tone tingierte und daher ununterscheidbar machte.

Auch Ranviers Methode war übrigens eine noch unzureichende. Das geht daraus hervor, dafs er behauptet, im Grofshirn wären die Deitersschen „Zellen“ von anderer Beschaffenheit, wie im Rückenmark. Hier wären keine abgesetzten Fasern vorhanden, sondern nur Protoplasmaausläufer der Zellen, ganz wie sie die früheren Autoren für sämtliche Deiterssche Zellen angenommen hatten, und wie er es selbst für die embryonalen Gebilde festgestellt hat. Kölliker hatte daher vollkommen Recht, wenn er diese Angabe von Ranvier gegen dessen Auffassung der entsprechenden Zellen im Rückenmark verwertete, denn es ist gar kein Grund vorhanden, warum im Gehirn die Deitersschen Zellen auch beim Erwachsenen „embryonal“ geblieben sein sollten, während sie im Rückenmark einen anderen Charakter bekommen hätten. Nun, dieser Einwand von Kölliker fällt jetzt einfach deshalb fort, weil auch im Grofshirn genau solche dem Zellleib nur angelehnte differenzierte Fasern nachzuweisen sind, wie im Rückenmark und wie überhaupt im ganzen Centralnervensystem des ausgebildeten Körpers. —

Man begnügte sich aber nicht damit, die Thatsächlichkeit von Ranviers Befunden zu bestreiten, sondern versuchte nach den herrschenden Anschauungen die anscheinenden Irrtümer von Ranvier zu erklären. So hat namentlich Golgi[1] und ihm folgend Kölliker[2] darauf hingewiesen, dafs das, was Ranvier für Fortsetzungen der Fasern im Innern und am Rande des Protoplasmaleibes angesehen hatte, einfach Faltungen waren, welche Fasern nur vortäuschten. Durch unsere Methode springt das Irrtümliche dieser Meinung sofort in die Augen. Es wäre geradezu wunderbar, wenn diese Faltungen so überwiegend häufig in den Verbindungslinien der präsumptiven Zellausläufer nicht nur, sondern auch in der Richtung, die der jeweiligen Krümmung dieser Ausläufer entspricht, verlaufen sollten, so dafs das Bild einer einheitlichen aus den zwei Ausläufern und der „Falte“ gebildeten Faser entsteht, und die beiden Ausläufer einerseits, die Falte andererseits nicht gesondert erscheinen. Ferner gelingt es niemals durch wirkliche Faltenbildungen, die zufällig da sind oder künstlich erzeugt werden, die entsprechende bei uns dunkel gefärbte Faser vorzutäuschen. Wenn ferner die Fasern senkrecht zur Schnittfläche an der Zelle hinlaufen, so erscheinen sie als Punkte, — und ein Punkt kann doch keine Falte sein.

Aber es wäre immerhin noch möglich, dafs durch Ranvier und unsere Methode zwar abgesetzte Fäden statt der Ausläufer dargestellt würden, dafs aber diese Darstellung auf irgend ein Kunstprodukt hinausliefe. Da die Ranviersche Methode noch eine sehr unsichere war, die ihren Erfinder für das Grofshirn, andere hervorragende Forscher, wie Golgi, überhaupt im Stiche liefs, so lag diese Vermutung gewifs nahe, und man kann es den Untersuchern nicht übel nehmen, wenn sie trotz der Veröffentlichungen von Ranvier an ihren altgewohnten Auffassungen festhielten. Aber unsere Methode mag sonst gar manches zu wünschen übrig lassen, in der uns hier beschäftigenden Frage ist sie ganz sicher, und da müssen wir denn sagen, nicht die Bilder von Ranvier, sondern die nach den alten Methoden erhaltenen Bilder waren Trugbilder.

---

[1] Gesammelte Abhandlungen. S 158.

[2] Handbuch der Gewebelehre. 6. Auflage. 2. Bd. S. 149 f.

Man begnügte sich aber nicht damit, die Thatsächlichkeit von Ranviers Befunden zu bestreiten, sondern versuchte nach den herrschenden Anschauungen die anscheinenden Irrtümer von Ranvier zu erklären. So hat namentlich Golgi[1] und ihm folgend Kölliker[2] darauf hingewiesen, dafs das, was Ranvier für Fortsetzungen der Fasern im Innern und am Rande des Protoplasmaleibes angesehen hatte, einfach Faltungen waren, welche Fasern nur vortäuschten. Durch unsere Methode springt das Irrtümliche dieser Meinung sofort in die Augen. Es wäre geradezu wunderbar, wenn diese Faltungen so überwiegend häufig in den Verbindungslinien der präsumptiven Zellausläufer nicht nur, sondern auch in der Richtung, die der jeweiligen Krümmung dieser Ausläufer entspricht, verlaufen sollten, so dafs das Bild einer einheitlichen aus den zwei Ausläufern und der „Falte" gebildeten Faser entsteht, und die beiden Ausläufer einerseits, die Falte andererseits nicht gesondert erscheinen. Ferner gelingt es niemals durch wirkliche Faltenbildungen, die zufällig da sind oder künstlich erzeugt werden, die entsprechende bei uns dunkel gefärbte Faser vorzutäuschen. Wenn ferner die Fasern senkrecht zur Schnittfläche an der Zelle hinlaufen, so erscheinen sie als Punkte, — und ein Punkt kann doch keine Falte sein.

Aber es wäre immerhin noch möglich, dafs durch Ranvier und unsere Methode zwar abgesetzte Fäden statt der Ausläufer dargestellt würden, dafs aber diese Darstellung auf irgend ein Kunstprodukt hinausliefe. Da die Ranviersche Methode noch eine sehr unsichere war, die ihren Erfinder für das Grofshirn, andere hervorragende Forscher, wie Golgi, überhaupt im Stiche liefs, so lag diese Vermutung gewifs nahe, und man kann es den Untersuchern nicht übel nehmen, wenn sie trotz der Veröffentlichungen von Ranvier an ihren altgewohnten Auffassungen festhielten. Aber unsere Methode mag sonst gar manches zu wünschen übrig lassen, in der uns hier beschäftigenden Frage ist sie ganz sicher, und da müssen wir denn sagen, nicht die Bilder von Ranvier, sondern die nach den alten Methoden erhaltenen Bilder waren Trugbilder.

---

[1] Gesammelte Abhandlungen. S 158.

[2] Handbuch der Gewebelehre. 6. Auflage. 2. Bd. S. 149 f.

Die nach den alten Methoden und nach der Golgischen[1] erhaltenen Bilder bedeuten nämlich nur, dafs bei diesen die Fasern und Zellleiber wegen ihrer gleichen Lichtbrechung (Ranvier) oder gleichen Färbbarkeit nicht differenziert werden, so dafs beide in chemischer (und morphologischer) Beziehung eins zu sein scheinen. Dafs dies aber in der That nur Schein ist, das beweisen eben unsere Präparate, welche ganz sicher zeigen, dafs Fasern und Zellleib im chemischen Sinne von einander durchaus verschieden sind. Das ist aber der Kernpunkt der ganzen Frage, und die Wichtigkeit derselben mag es entschuldigen, wenn wir hier etwas genauer auf die in Betracht kommenden Verhältnisse eingehen.

Die Sachlage ist hier genau dieselbe, wie bei allen chemischen und physikalischen Reaktionen. Zwei Körper, sagen wir z. B. Kalium- und Natriumverbindungen mögen noch so viele Reaktionen gemeinschaftlich haben, eine oder mehrere Reaktionen, die bei beiden verschieden ausfallen, entscheiden trotzdem auf das bestimmteste, dafs beide Körper von einander verschieden sind. Diese Reaktionen brauchen garnicht im eigentlichen Sinne chemisch zu sein. In der organischen Chemie unterscheidet man zwei Stoffe, die sonst wer weifs wie viele gemeinschaftliche chemische Eigenschaften haben, schon durch die Verschiedenheit des Siedepunktes oder durch die verschiedene Einwirkung auf das polarisierte Licht.

Nun sind die mikroskopischen Färbungen auch Reactionen, die wir immer als chemische bezeichnen, obgleich sie möglicherweise physikalische, wenigstens unter Umständen, sind. Aber für uns Histologen ist diese Unterscheidung bedeutungslos. An diesen Grenzgebieten verwischen sich ja die Gegensätze chemisch und physikalisch, und aufserdem würde ja auch die physikalische Reaktion immer von der Stofflichkeit der zu färbenden Gewebsteile abhängen, — und um die handelt es sich ja nur. Sind die Färbungen, die Imprägnationen etc. Reaktionen, so folgt aus der gleichen Färbung zweier Gewebsteile die Gleichheit der Stofflichkeit beider nur sehr bedingt, gerade wie bei chemischen und physi-

---

[1] Mit der Golgischen Methode hat nur ein einziger Forscher den unsern entsprechende Bilder, wenigstens unter besonders günstigen Umständen erhalten. Das ist Lloyd Andriezen (The Neuroglia elements of the human Brain. British medical Journal 1893, 29. Juli). Er sagt S. 4 des Sep.-Abdr.: „With a wide angle of light perfectly focussed and free from chromatic aberration and with equally good lenses the best preparations will show a very small quantity of protoplasm in the cell body, which, however, is mainly constituted of the meeting and intercrossing fibres. Many of these neuroglia fibres pass right through the cell body.“ Wer Golgibilder kennt, wird allen Respekt vor einem Forscher haben, der diese feine Beobachtung machen konnte.

kalischen Reaktionen im engeren Sinne. Zwei Strukturelemente, die eine oder mehrere Farbenreaktionen gemeinsam haben, können eben doch chemisch (oder physikalisch s. o.) verschieden sein, und man muss sie als verschieden auffassen, wenn irgend eine andere Färbung oder dergl. an den beiden Strukturelementen verschieden ausfällt (vorausgesetzt, dafs die Färbungen sichere und konstante sind). So färben sich in unseren Präparaten Kerne und Neurogliafasern gleich, nichtsdestoweniger wird es keinem Menschen einfallen, in den Neurogliafasern Kernchromatin zu vermuten, denn irgend eine andere Kernfärbung läfst die Neurogliafasern ungefärbt.

So färben sich aber auch die Fasern der Neuroglia und die Zellleiber gleich, wenn man Carmin, Nigrosin oder die Golgische Imprägnation benutzt. Beide sind aber trotzdem als chemisch (s. o.) verschieden zu betrachten, wenn auch nur eine einzige andere Methode sie als verschieden darstellt. Hier sind es aber sogar zwei Methoden, die Ranviersche und die unserige, die diese Verschiedenheit aufdecken. Bei der unserigen ist nicht blofs eine Abstufung von hell und dunkel vorhanden, sondern man sieht den Zellleib überhaupt nicht und kann ihn nur in der Umgebung des Kerns supponieren und das um so eher, als man ihn in denselben Präparaten mit anderen Methoden auch färben kann. Wenn daher Lenhossék[1] an meiner Methode tadelt, dafs man den Zellleib nicht sieht, so ist dies Moment für die vorliegende wichtige Frage nicht nur nichts Nachteiliges, sondern im Gegenteil aufserordentlich vorteilhaft, denn gerade das absolute Ausbleiben der Färbung (in Präparaten aus normalen Organen) läfst die chemische Verschiedenheit von Zellleib und Faser erst recht scharf hervortreten.

Wir wollen uns aber die Methoden, durch welche Zellleib und Faser gleich erscheinen, etwas genauer auf ihre Leistungsfähigkeit in dieser Frage betrachten.

Ueber die Unzuverlässigkeit des Lichtbrechungsvermögens[2] bei der Beurteilung feinerer Strukturverhältnisse braucht man heutzutage nichts mehr zu sagen, hingegen mufs in Bezug auf die hier in Betracht kommenden Tinktionen doch ausdrücklich darauf hingewiesen werden, dafs alle die Färbungen, welche einen Zusammenhang der Fasern mit dem Zellleib vortäuschen, nicht blofs mit Rücksicht auf Protoplasma und Fasern, sondern ganz im allgemeinen aufserordentlich wenig

---

[1] Der feinere Bau des Nervensystems. 2. Auflage. Berlin 1895. S. 186 f.

[2] Es sei aber wieder daran erinnert, dafs Boll trotz der Schwierigkeit der Unterscheidung die Differenzierung der Fasern vom Protoplasma schon bemerkt hat. (Vgl. die historische Übersicht)

electiv sind. Neutrales Carmin, Nigrosin etc. färben ja im Centralnervensystem eigentlich alles mit Ausnahme der Markscheiden. Es sind freilich geringfügige Unterschiede in der Intensität der Färbung in sofern vorhanden, als die gröberen Axencylinder dunkler tingiert erscheinen und dergl. Doch sind das keine prinzipiellen Färbungsauslesen.

Noch weniger elektiv, als das heutzutage recht geringschätzig behandelte Carmin etc., ist nun aber die Golgische Imprägnation. Hier finden sich nicht einmal konstante Unterschiede in der Intensität der Färbung, sondern alles kann wenigstens gleichmäfsig dunkel oder hell imprägniert sein. Alle Elemente des Centralnervensystems mit Ausnahme der Markscheiden werden ja von der Golgi-Methode imprägniert: Nervenzellen mit ihren Dendriten und Axencylinderfortsätzen, Neurogliazellen und -Fasern, Ependymzellen, ja sogar Gefäfse, freilich je nach der Laune der Tinktion jeder Bestandteil bald einzeln, bald in den verschiedensten, ganz unberechenbaren Kombinationen mit einem oder mehreren der anderen. Unelektiver, wenn man das Wort gebrauchen darf, kann schliefslich eine Methode kaum noch sein. Aber gegenüber dem Carmin etc. hat diese Methode noch einen sehr grofsen Nachteil. Bei den Färbungen im engern Sinne ist doch das eigentliche Strukturbild der Zellen noch so weit erkennbar, als es überhaupt durch Unterschiede der Lichtbrechung und geringfügiger Differenzen in der Färbungsintensität erkennbar sein kann. Bei der Golgi-Methode fallen aber die Strukturbilder wegen der Undurchsichtigkeit der Silberverbindung ganz oder so gut wie ganz fort, das gesamte imprägnierte System einer Zelle erscheint einfach als Silhouette. Selbst der Kern ist nur hier und da als hellerer Fleck angedeutet, ja sogar die Gefäfse erscheinen oft nicht als hohle Röhren, sondern als solide Stränge.[1]

Was würde man aber sagen, wenn jemand auf den Resultaten der Golgi-Methode fufsend einem grofsen Teile der Zellen im Centralnervensystem die Kerne absprechen wollte? Jederman würde eine solche Behauptung energisch zurückweisen, denn man kann ja durch andere Methoden mit Leichtigkeit in allen Zellen den Kern sichtbar machen. Was aber den Kernen recht ist, ist den Neurogliafasern billig. Wenn auch die hier in Betracht kommenden neuen Methoden nicht so einfache sind,

---

[1] Ähnlich ist das auch bei den Gallencapillaren etc. Während diese bei unserer Färbung als hohle Röhren mit einer verblüffend deutlichen Membran erscheinen, stellt sie die Golgifärbung als solide Bälkchen dar. Ich konnte schon Anfang 1889, also ehe noch die anderen Färbungen der Gallencapillaren bekannt gegeben waren, Herrn Geheimrat Heidenhain in Breslau die nach meiner Methode gefärbten Präparate übersenden.

wie die Kernfärbungen, so sind sie doch eben so sichere, und mit Hilfe derselben gelingt es aufs klarste, Fasern und Protoplasma zu differenzieren.

Wer sich also nicht genau desselben Fehlers schuldig machen will, wie einer, der die Kerne in den meisten Zellen des Centralnervensystems leugnet, der muſs notgedrungen auch die gesonderte Existenz der Neurogliafasern gegenüber den Zellleibern zugeben. In keinem Falle ist aber gegenüber der Auffassung von Ranvier und von mir die Berufung auf die Resultate der Golgi-Methode irgendwie noch statthaft, so hervorragende Forscher auch bis jetzt noch immer mit dem Einwande kommen, daſs Golgi-Bilder gegen unsere doch geradezu mit den Händen zu greifenden Befunde sprächen, um so weniger, als selbst mit der Golgischen Methode, freilich nur unter besonders glücklichen Umständen, unsere Ansicht bestätigt wurde (Lloyd Andriezen, vgl. oben S. 38 Anmerkung[1]).

Wir können nach alledem mit der gröſsten Sicherheit folgende Sätze aufstellen:

1. Die Neurogliafasern, die man bisher als Fortsätze der Deitersschen Zellen aufgefaſst hat, sind nicht mit dem Protoplasma chemisch identische Gebilde, sondern sind von diesem stofflich durchaus verschieden.

2. Die chemische Verschiedenheit tritt nicht etwa allmählich in mehr oder weniger weiter Entfernung vom Zellleib an den „Fortsätzen" auf, sondern die Differenzierung besteht von Anfang an, schon in unmittelbarer Nähe des Zellkerns.

3. Die meisten der sogenannten Fortsätze der Zellen sind überhaupt schon aus dem Grunde keine Fortsätze, weil

---

[1] Ganz unverständlich ist mir eine Bemerkung Golgis geblieben, der als Grund gegen die Ranviersche Auffassung anführt, daſs die „Zellausläufer" sich in inniger, komplizierter Weise mit den Gefäſswänden in Verbindung setzen (Ges. Abh. S. 158). Warum sollten „Fasern" sich nicht in ebenso inniger und komplizierter Weise an die Gefäſse ansetzen resp. zu ihnen hinstreben? Sehen wir doch ähnliches an den elastischen Fasern gerade gegenüber den Gefäſsen, aber auch gegenüber anderen Gewebsteilen. Im Übrigen ist der Ansatz der Neuroglia an die Gefäſse garnicht etwas so spezifisches, sondern nur die Teilerscheinung eines allgemeinen topographischen Gesetzes, das wir später kennen lernen werden.

bei ihnen je zwei anscheinende Ausläufer einen an der Zelle vorbeilaufenden gemeinschaftlichen Faden bilden. Dieser wird durch den Zellleib in keiner Weise unterbrochen, wie das doch bei „Ausläufern“ der Fall sein müſste, die ja jeder einzeln von dem Zellleibe ihren Ursprung nehmen würden. Mit einem Worte: **Es handelt sich hier garnicht um Fortsätze oder Ausläufer von Zellen, sondern um Fasern, die vom Protoplasma vollkommen differenziert sind.**

Wenn daher Frommann, später Golgi und letzterem folgend so ziemlich alle neueren Autoren gesagt haben, daſs die Neuroglia nur aus Zellen und deren Fortsätzen besteht, so trifft dies beim Menschen nur für die Embryonalzeit zu. Im ausgebildeten normalen Zustande besteht die Neuroglia aus Zellen und auſserdem aus Fasern, von denen die letzteren in räumlicher Ausbreitung so kolossal überwiegen, daſs man sie als den wesentlicheren Bestandteil der Neuroglia ansehen muſs.

3. Abschnitt:

Über die Neuroglianatur der durch die neue Methode gefärbten Fasern.

Im vorigen Abschnitt haben wir nachzuweisen gesucht, dafs die von uns differenziell gefärbten Fasern dem entsprechen, was man bisher irrtümlicherweise als Ausläufer der Deitersschen Zellen angesehen hat. Demzufolge haben wir die Fasern in Übereinstimmung mit sämtlichen Autoren als „Neuroglia“-Fasern, zunächst vorläufig, bezeichnet. Wenn diese Bezeichnung eine definitive werden soll, dann müfste aber der Beweis, dafs man es hier mit Neuroglia, d. h. mit einer nichtnervösen Zwischensubstanz zu thun hat, entweder schon früher erbracht sein, oder jetzt erst geliefert werden.

Wir werden zunächst zeigen, dafs bisher wirkliche Beweise für die Neuroglianatur der Fasern resp. der zu ihnen gehörigen Zellen in der normalen Histologie nicht vorliegen.

Von denjenigen älteren Beweisen, die sich auf einen unmittelbaren Zusammenhang von gewissen Fasern des Centralnervensystems mit denen der Pia mater, d. h. mit echten Bindegewebsfasern stützten, können wir ohne weiteres absehen, denn die seitherigen Untersuchungen von Frommann an haben ergeben, dafs ein solcher Zusammenhang garnicht existiert. Wir können auch die Deitersschen Beweise nicht als stringente anerkennen, so hoch man auch seine Bestrebungen schätzen mufs, die ihn als fast einzigen veranlafsten, nach neuen Kriterien für die Bindesubstanznatur gewisser Bestandteile im Centralnervensystem zu suchen.

Über seine Annahme, dafs die „schwammig-poröse“ Masse in den grauen Substanzen Neuroglia wäre, weil sie einen von den Zellen emanzipierten Bestandteil darstellte, brauchen wir garnicht zu reden, denn wir wissen jetzt, dafs diese Masse weder von den Zellen emanzipiert, noch Neuroglia ist. — Die nach ihm benannten Gebilde ferner hielt er deshalb für Bindegewebszellen, weil sie keine typischen Protoplasmaleiber hätten und daher nicht eigentliche Zellen, sondern Zellaequivalente darstellten. Abgesehen davon, dafs diese

Ansicht keine Anerkennung gefunden hat, indem alle Autoren die Deitersschen Zellen auch wirklich für echte Zellen halten, geht die Unbrauchbarkeit dieses Beweises für unsere Frage schon daraus hervor, dafs Deiters von demselben Gesichtspunkte ausgehend, echt nervöse Gebilde für bindegewebige Elemente erklärt hat (die Körner im Klein- und Grofshirn).

Sieht man von pathologisch-anatomischen Gesichtspunkten ab, so bleibt nunmehr als Beweis für die Neuroglianatur gewisser Formbestandteile des Centralnervensystems nur der (schon von Virchow benutzte) Beweis per exclusionem übrig, den man z. T. unbewufst nach dieser oder jener Richtung hin gemacht hat, d. h. man betrachtete das als Neuroglia, was man aus irgend einem Grunde nicht für nervös ansehen konnte. Ein solcher Beweis kann unter günstigen Umständen durchaus genügend sein. Gerade aber beim Centralnervensystem sind der Fallstricke so viele, dafs man mit einem Schlusse per exclusionem aufserordentlich vorsichtig sein mufs.

So haben es die älteren Autoren für ganz selbstverständlich gehalten, dafs zwischen den markhaltigen Fasern in der weifsen Substanz des Rückenmarks keine nervösen Elemente vorhanden wären, und dafs demnach alles, was zwischen diesen Fasern läge, einer Bindesubstanz entsprechen müsse. Jetzt wissen wir, dafs dort massenhafte Collateralen von Axencylindern verlaufen, dafs also durchaus nicht alle Fasern, die man da sieht, notwendiger Weise Neuroglia sein müssen, wie die älteren Forscher ohne weiteres glaubten. Auch in der Umgebung des Centralkanals im Rückenmark enthält die von den älteren Autoren für ganz nervenfrei gehaltene hintere Commissur reichliche Nervenfasern. Die „moleculare Masse“ in den grauen Substanzen nun gar, die man auch für nicht nervös, also für etwas „bindegewebiges“ hielt, hat sich als so reich an nervösen Elementen erwiesen, dafs für eine „moleculare Masse“ bei den höheren Geschöpfen eigentlich kaum Platz zu sein scheint.

Nicht anders, wie bei Beurteilung der Fasern und granulierten Massen, ging man bei Beurteilung der Deitersschen Zellen vor, nachdem man darauf verzichtet hatte, die von dem Entdecker derselben vorgebrachten allgemeinen Gesichtspunkte zu verwerten. Man findet bei keinem der Autoren jener Zeit auch nur den Versuch gemacht, die nichtnervöse Natur der Deitersschen Zellen zu beweisen, für so selbstverständlich hielt man es, dafs sie ihrem ganzen Aussehen nach nicht nervös sein könnten. Dieser Schlufs per exclusionem gründete sich aber nur darauf, dafs sie nicht so aussahen, wie die damals allein bekannten grofsen Nervenzellen. Wie ungerechtfertigt ein solcher Schlufs war,

geht schon daraus hervor, dafs man von demselben Gesichtspunkte ausgehend wieder, wie schon Deiters, sicher nervöse Teile für Neuroglia erklärte. So hielt z. B. auch Golgi noch 1871 die Körner des Kleinhirns für nicht nervöse Gebilde.

Seit den aufserordentlichen Erfolgen der Golgischen Methode hat man freilich in ihrer wahren Natur auch solche Ganglienzellen erkannt, von deren Existenz man früher garnichts wufste, und man hat es fertig gebracht, diese Ganglienzellen mit allen ihren Ausläufern darzustellen. Aber gerade jetzt, nachdem man so vieles, was man früher der Neuroglia zurechnete, als nervös erkannt hat, gerade jetzt glaubt man erst recht, dafs die Deitersschen Zellen nach der Gestalt, die sie bei Chromsilberimprägnation zeigen, absolut nicht Ganglienzellen sein können, d. h. dafs sie per exclusionem der Neuroglia entsprechen müfsten.

Dieser Schlufs wäre zutreffend, wenn die Kriterien, nach denen man die nervöse Natur der Deitersschen Zelle blofs nach der Form ihrer Silhouette bei Anwendung der Golgischen Methode ausschliefsen zu können glaubt, wirklich sichere wären und keine Ausnahme gestatteten.

Da sowohl Ganglienzellen als Astrocyten bei der Chromsilbermethode aus einem Zellkörper und aus Ausläufern zu bestehen scheinen, so könnten die Momente, die für einen fundamentalen Unterschied beider Zellarten sprechen sollten, entweder an den Ausläufern oder am Zellkörper, oder an beiden gefunden werden.

1. Betrachten wir zuerst die Ausläufer der Deitersschen Zellen, so könnte einmal der Unterschied gegenüber denen der Ganglienzellen in einem wesentlichen Charakteristikum oder in der einfachen Form der Fortsätze zu finden sein.

a) Was den Charakter der Ausläufer betrifft, so wissen wir, dafs die Nervenzellen zweierlei wohlcharakterisierte Arten von Ausläufern haben sollten, Dendriten und Axencylinderfortsätze. Die Deitersschen Zellen zeigen eine solche Differenzierung ihrer Fortsätze nicht, und wenn in der That die Ganglienzellen diese beiden Arten Ausläufer stets scharf differenziert aufwiesen, so wäre hiermit ein fundamentaler Unterschied beider Zellarten schon aus der Form der Silhouette zu entnehmen. Aber dieser scharfe Unterschied zwischen Dendriten und Axencylinderfortsätzen findet sich wohl bei dem verbreitetsten Typus der Ganglienzellen, aber durchaus nicht bei allen.

α) Es giebt Nervenzellen, welche gar keine Dendriten besitzen, z. B. die Zellen der Spinalganglien [1] und solche in den peripherischen Geflechten des Sympathicus. Diese haben nur Axencylinderfortsätze.

β) Umgekehrt giebt es vielleicht Nervenzellen ohne Axencylinderfortsatz, die dann also nur Dendriten besäfsen, z. B. in der Körnerschicht des Bulbus olfactorius und in den peripherischen Sinnesapparaten. [2]

γ) Es giebt Nervenzellen, bei denen die gewiegtesten Kenner noch nicht einig darüber sind, wie man ihre Fortsätze deuten soll. Zu diesen gehören eventuell die sub β angeführten, dann aber auch z. B. die Ramón y Cajalschen Zellen der Grofshirnrinde, deren Fortsätze so unbestimmt charakterisiert sind, dafs Retzius diese Zellen zuerst [3] garnicht als Nervenzellen anerkennen wollte, und dafs er auch dann, als er ihre wahre Natur erkannt hatte, sagte: [4] „Unter den Fortsätzen der fraglichen Zellelemente ist es nun aber schwer, charakteristische Unterschiede aufzufinden; man trifft zwar dickere und dünnere Fortsätze an, doch zeigen dieselben keine Eigenschaften, durch die sich Axencylinder und Protoplasmafortsätze bestimmt unterscheiden.“ Die Unsicherheit der Beurteilung dieser Fortsätze geht auch daraus hervor, dafs von zwei andern Autoritäten, die eine, Ramón y Cajal, den betreffenden Zellen viele Axencylinderfortsätze, die andere, van Gehuchten, aber nur einen einzigen zuschreibt.

Mit einem Worte, wenn auch die grofse Mehrzahl der anerkannten Ganglienzellen zwei wohlcharakterisierte Arten von Fortsätzen besitzt, so giebt es doch Ausnahmen, welche beweisen, dafs echte Nervenzellen diese typischen

---

[1] Ramón y Cajal betrachtet zwar den einen Axencylinderfortsatz, den cellulipetalen, als Dendriten, doch kann sich das nur auf die funktionelle Natur desselben beziehen, denn im histologischen Sinne, auf den es uns hier allein ankommt, ist doch ein Fortsatz, der zum Axencylinder eines markhaltigen Nerven wird, unter allen Umständen ein Axencylinderfortsatz.

[2] Vgl. Kölliker, Gewebelehre, 6. Auflage, 2. Bd, S. 43. Bei diesen Zellen steht freilich die Deutung der Ausläufer noch nicht ganz fest. Man kann sie daher eventuell in die Kategorie γ rechnen. Vgl. Golgi, Gesammelte Abhandlungen, S. 51, und Ramón y Cajal, Notas preventivas sobre la retina y gran simpático. Barcelona 1891. S. 4 f.

[3] Über den Bau der Oberflächenschicht der Grofshirnrinde beim Menschen und bei den Säugetieren. Verhandlung des Biologischen Vereins in Stockholm. Bd. 1. 1891, 15. März.

[4] Biologische Untersuchungen. Neue Folge. V. Bd. No. 1 und 2. Stockholm 1893. S. 7.

Formen der Fortsätze nicht zu besitzen brauchen. Ist das aber einmal festgestellt, so liegt a priori nicht der geringste Grund dagegen vor, dafs die Deitersschen Zellen nicht eine weitere Ausnahme unter den Nervenzellen darstellen sollten. Man brauchte sich auch darüber garnicht aufzuregen, dafs die Fortsätze der Deitersschen Zellen so wenig typisch sind, dafs sie der eine sämtlich für (sehr zahlreiche) Axencylinderfortsätze, der andere vielleicht sämtlich für Dendriten, der dritte vielleicht für beides erklären würde, denn solche unbestimmten Ausläufer kommen ja auch bei echten Ganglienzellen vor.

b) Wenn aber auch in dem Charakter der Fortsätze (Axencylinderfortsatz und Dendriten) kein fundamentaler Unterschied zwischen beiden Zellarten zu finden ist, so wäre es doch möglich, dafs trotzdem die blofse Form der (unbestimmten) Ausläufer der Deitersschen Zellen genügte, um diese unter allen Umständen von den Nervenzellen abzutrennen. Da die Ausläufer der letzteren sich verzweigen, so wäre ein sicherer Unterschied dann vorhanden, wenn alles das, was an den Astrocyten bei der Golgischen Färbung als Fortsatz erscheint u. zw. an allen sogenannten Neurogliazellen absolut unverzweigt wäre.

Wäre aber auch nur die Möglichkeit einer Verzweigung der Ausläufer von Zellen zugegeben, die man der Neuroglia zurechnet, so würde das nicht mehr genügen, um eine unüberbrückbare Kluft zwischen Nervenzellen und Astrocyten zu statuieren. Nun sind aber alle Autoren[1] darüber einig, dafs bei Anwendung der Golgischen Methode die „Neurogliazellen“ spärlichere oder reichlichere Verzweigungen zeigen. Besonders reichliche werden an den

[1] Lenhossék scheint zu meinen, dafs Golgi noch immer an der Annahme einer fast absoluten Ungeteiltheit der Fortsätze festhalte. Dem ist aber nicht so. Anfangs (1871) hatte er zwar angenommen, dafs nur beim Beginne der Fortsätze solche Teilungen (und da auch nur selten) vorkommen. Später hat er aber seine Meinung etwas geändert. An Isolationspräparaten, die er zur Kontrolle von Ranvier anfertigte, hat er gefunden (Ges. Abhandl. S. 157 f.), dafs man ihre Teilungen in der Nähe ihres Ursprungs zwar häufiger sieht, „aber oft verzweigen sie sich auch in grofser Entfernung davon.“ Colella nun gar hat in neuerer Zeit aus Golgis Laboratorium eine Arbeit veröffentlicht, in der er an embryonalen Zellen sogar sehr reichliche Verzweigungen beschreibt (de nombreux ramuscules secondaires longs et courts naissent sur le trajet . . . . ils se terminent librement par une riche arborisation en patte d'oie). Archives ital. de Biologie. Bd. 20. S. 214.

„Kurzstrahlern“ beschrieben, die noch dazu als mit den Langstrahlern durch vielerlei „Übergänge“ verbunden geschildert werden [1], — und das genügt, um zu konstatieren, dafs das Vorhandensein der Verzweigungen oder das Fehlen derselben keinen prinzipiellen Unterschied zwischen Nervenzellen und Neurogliazellen ergiebt.

Auch die Art der Verzweigungen ist bei der grofsen Mannigfaltigkeit, mit der sich die Nervenzellen verzweigen, zur Unterscheidung nicht zu verwenden: man wird zwischen den Verzweigungen der „Neurogliazellen“ und denen der Nervenzellen stets Analogieen finden können.

2. Die Körper beider Zellarten zeigen keine prinzipiellen Differenzen. Bei den Neurogliazellen ist er ja im allgemeinen recht klein, aber wenn man z. B. die Doppelpyramiden am Lobus piriformis [2] betrachtet, so findet man hier gerade so kleine Zellkörper, und umgekehrt bildet z. B. van Gehuchten Neurogliazellen ab, die einen so mächtigen Protoplasmaleib besitzen, dafs sie jede Ganglienzelle darum beneiden könnte. [3]

3. Noch weniger sind gewisse secundäre Hilfsmittel geeignet, fundamentale Unterschiede zwischen Neurogliazellen und Nervenzellen aufzustellen. Manche geben an, dafs sich die ersteren bei der Golgischen Methode mehr rostbraun, statt schwarz, färben, dafs sie sich schneller als letztere imprägnieren und dergl. Aber man kann sich leicht überzeugen, dafs diese Unterschiede sehr schwankende sind. Auch Neurogliazellen färben sich oft schwarz, die Ganglienzellen auch rostbraun, und neben den anfangs hauptsächlich gefärbten Astrocyten finden sich doch immer einige Nervenzellen mitgefärbt. Damit ist also auch nichts anzufangen.

4. Das ausschlaggebende Moment aber, welches aufs deutlichste beweist, dafs absolute, d. h. sichere Differenzen zwischen den beiden Zellarten nicht bestehen, ist das, dafs die ersten Autoritäten unter Umständen zweifelhaft sein können, ob bestimmte Klassen von Zellen Neurogliazellen oder Nervenzellen

---

[1] Vgl. darüber Kölliker, Gewebelehre. 6. Auflage. 2. Band. S. 144 ff.

[2] Kölliker, Über den Fornix longus von Forel und die Riechstrahlungen im Gehirn des Kaninchens. Anatomische Gesellschaft. 1894. Figur 1.

[3] La mœlle épinière et le cervelet. La Cellule. Bd. 7. 1891. Fig. 38 und 42.

sind, und dafs sie sich in der Diagnose irren können. Wir haben das bereits von den Ramón y Cajalschen Zellen der Grofshirnrinde erwähnt; es sei auch noch an die Korbzellen in der Schicht der Purkinjeschen Zellen erinnert. Und so kam denn sogar Kölliker[1] bei Besprechung dieser Korbzellen zu der resignierten Bemerkung: „Da durch Silber nach Golgis Methode Neurogliaelemente und Nervenzellen sich färben, so ist eine Entscheidung nicht leicht und bleibt eine solche in erster Linie dem Takte und der Erfahrung des Einzelnen überlassen."

5. Auch die Histogenese läfst uns bei dem Suchen nach scharfen Unterschieden im Stich, denn beide Zellarten haben eben denselben Ursprung.

Nehmen wir alles zusammen, so können wir demnach sagen: Die Kriterien, nach welchen man die nervöse Natur der Deitersschen Zellen ausschliefsen zu können glaubt, sind keine sicheren, d. h. eine Ausnahme nicht gestattenden. Der Schlufs per exclusionem auf ihre Neuroglianatur kann daher richtig ausgefallen sein, aber wenn er richtig war, so war das nur „die Folge einer glücklichen Divination, aber nicht die einer stringenten Beweisführung," um einen Deitersschen Satz zu wiederholen.

Wir haben bisher die Möglichkeit, dafs jemand den Deitersschen Zellen eine nervöse Natur zuschreiben könnte, nur als „problema" behandelt. Es wird daher von Interesse sein, dafs in der That in neuerer Zeit eine Arbeit Colellas erschienen ist, die dieses Problema zur Wirklichkeit zu machen sucht, und zwar stammt die Arbeit aus keinem geringeren Laboratorium, als aus dem von Golgi. Da die Arbeit aus diesem Laboratorium kommt, verdient sie wohl Beachtung. Es sei ganz speziell darauf hingewiesen, dafs es sich in den nachfolgenden Sätzen Colellas[2] um die Natur der embryonalen Neuroglia handelt, nicht etwa um ihre Abstammung aus nervösem Material. Das beweisen die Anfangsworte deutlich genug. Colella sagt wörtlich:

„Leur" (d. h. der Deitersschen Zellen) „mode d'origine n'est pas un argument décisif pour juger de leur nature et le champ reste ouvert à de nouvelles recherches pour savoir, si les élements

[1] Das Kleinhirn. Zeitschrift für wissenschaftliche Zoologie. Bd. 49. 1890. S. 675.

[2] Sur l'histogenèse de la névroglie dans la moelle épinière. Archives ital. de biologie. Bd. 20. S. 212 ff.

de la névroglie de la moelle épinière sont de nature nerveuse, épithéliale ou connective. Pourtant les recherches de Magini sur les systèmes de filaments épithéliaux se colorant comme les fibres nerveuses à myéline et celles de Caporaso et Sgotto sur la propriété de l'épithélium du canal central chez les tritons et les larves des grénouilles tendrent à assigner à la névroglie embryonnaire une nature nerveuse.“

Welche nervöse Rolle die Neuroglia spielen soll, wird allerdings in dem sehr kurzen Aufsatze nicht gesagt; man müfste ihr wohl eine ganz besondere Aufgabe zuschreiben, vielleicht irgend eine sympathische. Aber man braucht sich darüber nicht den Kopf zu zerbrechen. Die „nouvelles recherches“, die Colella mit Recht verlangt, sind von uns angestellt, und von jetzt an kann nicht mehr der geringste Zweifel darüber existieren, dafs wenigstens die Neuroglia, die den sogenannten Fortsätzen der Deitersschen Zellen, in specie der „Langstrahler“ entspricht, eine echte Intercellularsubstanz ist, d. h. im morphologischen Sinne eine „nature connective“ besitzt.

Wenn wir uns nämlich auf den Standpunkt stellen, zu welchem die Ranvierschen und unsere Untersuchungen die Basis abgeben, so ist der Boden, den wir unter den Füfsen haben, doch nicht mehr ein so schwankender, wie das bisher der Fall war.

1. Durch unsere Untersuchungen ist zunächst auch ein Schlufs per exclusionem möglich: Unsere Färbung tingiert, wenn wir von den hier nicht in Betracht kommenden Zellkernen absehen, überhaupt nichts, was nach allen geltenden Anschauungen als nervös betrachtet werden kann, d. h. weder Ganglienzellen, noch ihre Protoplasmafortsätze, noch Axencylinder. Dieser Schlufs per exclusionem ist viel sicherer, als die bisherigen, denn er stützt sich nicht, wie diese, auf die immerhin schwankenden Formen, sondern auf eine chemische Reaktion, u. zw. auch wieder nicht von dem so unsicheren Gesichtspunkte einer Gleichheit der Reaktion, sondern von dem einer Verschiedenheit derselben aus. Wir haben ja S. 38f. gesehen (und auch die von Colella erwähnten Untersuchungen von Magini [1] dürften wieder zur Warnung dienen), dafs man eine gleiche histochemische Reaktion nur sehr bedingt als Kriterium benutzen

[1] Die Arbeit von Magini ist mir unbekannt. Das obige Urteil ist durch die Bemerkung bei Colella veranlafst.

kann. Um so sicherer ist aber ein Schlufs, der sich auf die Verschiedenheit der chemischen Reaktion stützt, die unter allen Umständen einer stofflichen Verschiedenheit entspricht. —

2. Aber wir brauchen uns mit diesem Schlusse per exclusionem nicht zu begnügen, wir haben noch andere, positiv beweisende Gründe, dafs wir es hier mit einer echten Intercellularsubstanz zu thun haben.

Was ist denn eine echte Intercellularsubstanz?

Wie wir in der historischen Übersicht gesehen haben, hat sich auch Deiters diese Frage vorgelegt. Seine Antwort gab er mit einer von Max Schultze herrührenden Definition. Nach dieser sind Intercellularsubstanzen „modifizierte Zellsubstanzen, die sich von den Zellleibern emanzipieren und dann nicht mehr als unmittelbar damit zusammengehörig betrachtet werden können.“

Man wird sich weiter erinnern, dafs Deiters auf dieser Definition fufsend die moleculare, von ihm schwammig-porös genannte Masse der grauen Substanzen für Intercellularsubstanz erklärt hat. Da nun diese seine Annahme sich seitdem als irrig herausgestellt hat, so mufs in der Schultzeschen Definition oder in der Deitersschen Verwendung derselben ein Fehler liegen, den wir natürlich vermeiden müssen.

In der That hat Deiters nachzuweisen unterlassen, dafs die schwammig-poröse Masse modifizierte Zellsubstanz ist. Er hat sich damit begnügt, dafs diese Massen mit den Zellleibern anscheinend nicht mehr unmittelbar zusammenhängen, — eine Ansicht, die der Wirklichkeit nicht entsprach und nur durch die damals übliche Methodik vorgetäuscht wurde. Wäre aber der Zusammenhang auch mit unseren jetzigen Methoden nicht nachweisbar, so folgte daraus noch lange nicht, dafs die schwammig-poröse Masse Intercellularsubstanz ist, denn zu dieser gehört auch der Nachweis einer Modifikation der „emanzipierten“ Zellsubstanz.

Wir können aber ferner auch die Schultzeschen Anforderungen an eine echte Intercellularsubstanz noch strenger formulieren. Wir brauchen uns nicht mit dem unbestimmten Worte „Modifikation“ zu begnügen, sondern können fordern, dafs die modifizierten Massen kein echtes Protoplasma mehr sind.

Sind nun bei unseren Fasern die beiden Erfordernisse Modifikation zu nichtprotoplasmatischen Substanzen und Emanzipation vom Zellleibe erfüllt? Diese Frage kann man wohl mit der gröfsten Bestimmtheit bejahen.

a) Die Modifikation der Zellsubstanz, aus der diese Fasern ja doch nach den embryologischen Untersuchungen herstammen,[1] ist ganz auffallend. Die Fasern reagieren auf die neue Farbe, das Protoplasma garnicht, Ja, nicht nur das Protoplasma der Deitersschen Zellen färbt sich durch unsere Tinktion nicht, sondern alle normalen Protoplasmen färben sich entweder nicht, oder sogar in der Kontrastfarbe. Hier ist demnach ein fundamentaler Gegensatz vorhanden, auf der einen Seite das Zellprotoplasma, auf der anderen Seite ein typischer Faden, an dem man gewifs nichts protoplasmatisches bemerken kann.

b) Dieses Moment allein würde aber nicht genügen. Das geht daraus hervor, dafs auch der Axencylinder modifiziertes Zellprotoplasma ist, das sich färberisch vom Protoplasma des Zellleibes, wenigstens graduell, unterscheiden läfst. Aber dieser Axencylinder ist nirgends vom Zellprotoplasma „emanzipiert", er geht sicher in dieses hinein, seine Eigenheiten ganz allmählich verlierend.

Zum Nachweis einer Intercellularsubstanz gehört eben noch die Emanzipation vom Zellleibe, und, wie wir gesehen haben, ist diese bei den in Rede stehenden Fasern, im Gegensatz z. B. zu den Axencylindern, eine ganz vollkommene. Die Fasern stehen nur in Contiguität mit dem Zellleibe, sie sind mit ihm nicht, wie gerade die Axencylinder, als Ausläufer verbunden, sondern die zu- und abführenden Teile des Fadens, wenn man diese Ausdrücke brauchen darf, sind miteinander so innig vereinigt, dafs sie eben etwas zusammenhängendes, eine gemeinschaftliche Fibrille darstellen, die glatt über die anliegende Zelle hinwegläuft.

Auf diese Weise tritt die Neuroglia endlich wieder durchaus in die Reihe der Bindesubstanzen, aber wohlgemerkt nur vom morphologischen Standpunkte aus.

Gerade wie bei den typischen mesodermatischen (mesenchymatischen, parablastischen) Bindegewebsarten ist diese eigenartige Bindesubstanz des Centralnervensystems aus Zellen und aus davon unabhängiger, hier sogar, wie beim collagenen Bindegewebe, faseriger Zwischensubstanz zusammengesetzt.

---

[1] Sollte jemand der Meinung sein, dafs die Fasern garnicht aus der Zelle, sondern von vornherein intercellular entstünden, so bedürfte es gar keines Nachweises einer Modifikation der Zellsubstanz, dann wären die Fasern selbstverständlich „Intercellularsubstanz". Aber soviel ich sehe, ist es weder für die Neuroglia, noch für das Bindegewebe möglich, einen solchen Standpunkt einzunehmen.

3. Ja noch mehr. Sie verhält sich nicht nur morphologisch absolut wie eine echte, wenn auch besonders geartete Bindesubstanz, sondern auch pathologischer Weise reagiert sie genau in derselben Weise, wie das typische Bindegewebe. Wie dieses immer da wuchert, wo das spezifische Parenchym zu Grunde geht, so wuchert auch die Neuroglia, wie allbekannt, und wie sich mit unserer Methode erst recht nachweisen läfst, immer dann, wenn das spezifische Gewebe seines Organs, d. h. das Nervengewebe, zu Grunde gegangen ist.

Fassen wir das Gesagte zusammen, so ergiebt sich folgendes:

Die von uns gefärbten Fasern sind als nicht nervöse Intercellularsubstanz aufzufassen,

1. weil bei unserer Färbung alles nervöse ungefärbt bleibt, die Fasern sich aber dunkelblau tingieren (Schlufs per exclusionem),
2. weil die Fasern eine modifizierte, nicht mehr protoplasmatische, und vom Zellleib emanzipierte Substanz besitzen,
3. weil sich die Fasern (und die dazu gehörigen Zellen) pathologischer Weise ganz wie eine Bindesubstanz verhalten, d. h. wuchern, wenn das spezifische, nervöse Gewebe zu Grunde geht.

4. Abschnitt:

Verhältnis der Neurogliafasern zu etwaigen anderen Neurogliasubstanzen und zum Bindegewebe. Chemisches.

Durch unsern Nachweis, dafs die Neurogliafasern als echte faserige Intercellularsubstanz zu betrachten sind, ist auch gleichzeitig der bisher noch ausstehende Beweis geliefert, dafs wenigstens die typischen Deitersschen Zellen nicht nervöse, sondern gliöse Zellen sind. Diese vermeintlichen Zellen sind ja nichts anderes, als wirkliche Zellen mit dicht anliegenden, von ihnen als von einem Centrum ausstrahlenden Neurogliafasern. Sie sind die Bruchstücke des Neurogliagerüstes, in denen die Fasern mit den Zellen in Contiguität getroffen werden.

Desgleichen gilt dieser Nachweis auch für diejenigen embryonalen Zellen, von denen wir wissen, dafs sie später jene fädige Intercellularsubstanz erzeugen, also für die typischen Langstrahler, so weit sie mit Sicherheit als solche zu erkennen sind. Ebenso würde dieser Beweis unter derselben Voraussetzung des sicheren Erkennens für diejenigen Langstrahler gelten, welche etwa auch im späteren Leben ihre embryonale Natur noch beibehalten haben, eine Möglichkeit, deren wir S. 34 f. gedacht haben. Auch bei diesen könnte man annehmen, dafs sie gelegentlich noch einmal in die Lage kämen, fädige Intercellularsubstanz entstehen zu lassen.

Aber damit ist auch alles erschöpft, wofür unsere Beweisführung in Betreff der Neuroglianatur gilt. Schon die sogenannten Kurzstrahler und alle anderen ähnlichen Formen gehören nicht zu den Gewebsbestandteilen, von denen man mit irgend welcher Sicherheit annehmen könnte, dafs sie eine vom Zellleib differenzierte Zwischensubstanz erzeugen. Ebenso wenig gilt das für diejenigen Gebilde, welche Ranvier und Lloyd Andriezen als „protoplasmatische Gliazellen der Grofshirnrinde“ beschrieben haben. Alle diese Zellen entziehen sich nicht nur dem Nachweis durch unsere Methode, sondern auch all den Kriterien, die wir zur stringenten Beweisführung für ihre Neuroglia-

natur als notwendig erkannt haben. Die Kurzstrahler etc. und die protoplasmatischen Zellen können Neurogliaelemente sein, sie können es auch nicht sein, es fehlt jeder Beweis nach der einen oder andern Richtung, und wir müssen daher eine Beurteilung derselben durchaus ablehnen.

Wir müssen uns ferner jedes Urteils über alle anderen Arten Zwischensubstanzen im Sinne der Autoren durchaus enthalten. Wir sehen ja in unseren Präparaten nichts von einer molekularen, netzförmigen oder glasigen „Grundsubstanz", nichts von der spongioblastischen Neuroglia im His schen Sinne, nichts von einer Hornspongiosa. Diese letztere hat, um dies besonders zu betonen, mit unseren „Neurogliafasern" nichts zu thun. Abgesehen davon, dafs die äufsere Erscheinung des Gerüstes dieser Hornspongiosa von unseren Bildern ganz abweicht, so hat sie auch schon aus dem Grunde mit unseren Neurogliafasern nichts gemein, weil sie sich auch innerhalb der Markscheiden (selbst der peripherischen Nerven) vorfindet, welche bei unserer Methode ganz leer erscheinen. Aus demselben Grunde hat unsere Neurogliafaserung nichts gemein mit dem von Paladino geschilderten „Nevroglio mielinico",[1] worüber wir ebenfalls jedes Urteil ablehnen.

Im Anschlufs an die Frage, ob die Neurogliafasern Hornsubstanz darstellen, sei auch noch die nach etwaigen anderen chemischen Beziehungen dieser Fasern hier gleich mit abgemacht.

Zunächst mufs konstatiert werden, dafs unsere Fasern mit denen des leimgebenden Bindegewebes chemisch absolut nicht übereinstimmen.

Schon Henle und Merkel haben solche chemische Unterschiede zwischen dem echten Bindegewebe und zwischen dem, was sie molekulare Massen nennen, aufgestellt.[2] Die molekularen Massen im Sinne von Henle und Merkel entsprechen aber so ziemlich dem, was wir jetzt als faserige Neuroglia auffassen.

Kochendes Wasser löst leimgebendes Bindegewebe, die „molekulare Masse" aber nicht, umgekehrt wird diese, aber nicht das Bindegewebe, durch successive Einwirkung von Kalilauge und Wasser zerstört. Auf eine weitere Differenz hat Boll aufmerksam gemacht.[3]

---

[1] Dei limiti precisi tra il nevroglio e gli elementi nervosi del midollo spinale. R. acad. di Roma. IX. Fasc. 2. 1893.

[2] Über die sogenannte Bindesubstanz der Centralorgane des Nervensystems. Zeitschrift für rationelle Medizin. 3. Reihe. Band 34 (1869). S. 59.

[3] Archiv für Psychiatrie. Bd. 4. S. 20.

Essigsäure macht die Fasern zwar etwas erblassen, läfst sie aber nicht zu unsichtbaren Massen verquellen, wie das bei Bindegewebsfasern der Fall ist. Auch bei unserer Färbung verhält sich Bindegewebe und Neuroglia verschieden.

Wir können ferner sagen, dafs die Gerlach'sche vielfach acceptierte Annahme, die Neurogliafasern wären elastische, durchaus irrig ist, so verführerisch für diese Auffassung das starrgeschwungene Aussehen der Neurogliafasern auch sein mag. Einmal färben sich elastische Fasern nach unserer Färbung absolut nicht, sodann aber kann man umgekehrt nachweisen, dafs sich unsere Fibrillen nicht mit den für elastische Fasern geeigneten Methoden tingieren, und endlich spricht die geringe Widerstandsfähigkeit der Neurogliafasern gegen postmortale Einflüsse und gegen Kalilauge ohne weiteres gegen die Identifizierung derselben mit elastischeu Fasern. —

Weiterhin mufs noch der Beziehung zum fädigen Fibrin gedacht werden. Arndt[1] sagt darüber:

„. . . . Allein, dafs alles Bindegewebe sei, was sich in dieser Weise“ (sc. wie es Jastrowitz beschreibt) „präsentiert, und das in Sonderheit die Kerne, welche im Marklager zwischen den Nervenfasern liegen, das wage ich auch heute noch zu bestreiten . . . . . . Die Balken und Fasern, in und an denen sich jene Kerne, unter denen sicherlich auch manches weifse Blutkörperchen ist, vorfinden, halte ich demnach für Gerinnsel von Lymphe, der sich nach dem Tode und während der Präparation eine nicht unerhebliche Menge von Mark, das sich aus den Scheiden ablöste, beigemischt hat.“

Bei unserer Methode färbt sich in der That auch Fibrin, wenn solches z. B. in den Gefäfsen vorhanden ist, mit. Wollte man aber aus dieser gleichen Reaktion einen Schlufs auf eine chemische Gleichheit machen, so würde man wieder in den von uns so oft gerügten Fehler verfallen. Dann müfste man auch die Membranen der Gallenkapillaren, die doppeltlichtbrechende Substanz der Muskeln, die Zellkerne etc. für Fibrin erklären. Man kann sich gerade tinctoriell von der Verschiedenheit der Neurogliafasern und des Fibrins überzeugen.

Macht man nämlich an einem gewöhnlichen Alkoholpräparat die richtige, von uns angegebene Fibrinfärbung, so färbt sich zwar das Fibrin, aber nicht die Neuroglia. Aber man braucht diese färberische Reaktion garnicht, denn dafs unsere Fasern resp. die

---

[1] Zur Histiologie des Gehirns. Archiv für Psychiatrie. Band III. S. 470 f.

sogenannten Deitersschen Zellen ein einfaches Gerinnungsprodukt sein könnten, ist schon aus dem Grunde absolut ausgeschlossen, weil die grofse Regelmäfsigkeit in der Beschaffenheit der Geflechte, die für jede bestimmte Stelle des Centralnervensystems feststeht, von vornherein einer Gerinnung im Sinne Arndts und auch, wie wir gleich hinzufügen wollen, im Sinne Schwalbes widerspricht. Gerinnungen vorher flüssiger Massen haben stets etwas wechselndes, zufälliges an sich, was sich mit jener Regelmäfsigkeit durchaus nicht verträgt.

Schwalbe war zu seiner Meinung durch Injektionsresultate gekommen. Injektionsmassen dringen nach ihm anstandslos zwischen die Nervenfasern ein, so dafs also nach seiner Meinung kein fester Kitt die Fasern vereinigen kann. Das ist gewifs ganz richtig, aber die Neurogliafasern sind eben gar kein fester Kitt, sondern isolierte Fasern, zwischen denen Injektionsmasse noch genug Platz hat, und zwischen die sie daher leicht eindringen kann. —

Auch unverändertes Protoplasma können die Neurogliafasern nicht sein, wie wir mehrfach hervorgehoben haben — aber positiv können wir über ihre Natur noch nichts aussagen. Von den negativen Resultaten ist das wichtigste, dafs sie von allen Fasern des gewöhnlichen Bindegewebes ganz verschieden sind.

---

5. Abschnitt:

Besprechung der histogenetischen Stellung der Neuroglia.

Wir haben gesehen, dafs die Neuroglia, soweit sie überhaupt in sicher nachweisbarer Gestalt auftritt, durchaus dem Typus des gewöhnlichen Bindegewebes entspricht, d. h. aus Fasern und aus Zellen besteht, die mit diesen Fasern im ausgebildeten Zustande sich nur in Contiguität befinden. Trotz dieser Übereinstimmung des Typus im Bau, weicht die Neuroglia aber morphologisch und chemisch so bedeutend von dem gewöhnlichen Bindegewebe ab, dafs sie schon dadurch eine ganz eigenartige Sonderstellung gewinnt. Diese Sonderstellung behält sie bei, ja zeigt sie noch deutlicher unter pathologischen Verhältnissen: niemals wird aus Neuroglia „Bindegewebe" oder umgekehrt.

Wohl kann es vorkommen, dafs Neurogliafasern ins Bindegewebe (die Pia mater) hineinwachsen, aber dann verwandelt sich nicht das Bindegewebe in Neuroglia, sondern die Fasern der letzteren stehen in unmittelbarem Zusammenhange mit denen im angrenzenden nervösen Gewebe, sie sind also nur über ihre natürliche Grenze hinaus gewachsen, das Bindegewebe verhält sich ihnen gegenüber aber ganz passiv.

Zu diesen chemischen, morphologischen und allgemein-biologischen Unterschieden zwischen Neuroglia und Bindegewebe kommt nun aber noch der histogenetische. Seit Vignal[1] die Ansicht, dafs die Neuroglia ectodermatischen Ursprungs sei, zuerst bestimmt ausgesprochen hat, hat sich dieselbe immer mehr und mehr die Anerkennung der Autoren errungen, und namentlich seitdem durch die Golgische Methode gerade die Untersuchung der embryonalen Verhältnisse aufserordentlich gefördert worden ist, sind fast alle Autoren zu Vignals Anschauungen übergetreten.

Man hat die Entwicklung der Neuroglia sowohl im phylogenetischen, als im ontogenetischen Sinne mit der Golgischen Methode erforscht und ist dabei ziemlich allgemein

[1] Archives de physiologie. 1884.

zu der Ansicht gekommen, dafs die Deitersschen Zellen nichts sind, als ausgewanderte Zellen der in epithelialer Form angeordneten Ectodermzellen der Medullarplatte.

Die unterste Stufe in dieser Entwickelungsreihe, die aber bis hoch in die Säugetierreihe hinein, andeutungsweise noch bis in den ausgebildeten, erwachsenen Zustand hin sich erhält, ist die, dafs die Epithelzellen des Centralkanals resp. die der Ventrikelwände lange Fortsätze peripheriewärts aussenden, die das ganze Gebiet des betreffenden nervösen Centralorgans durchsetzend bis an die Pia mater reichen. Kürzere Fortsätze der Epithelzellen im Centralnervensystem sind schon lange bekannt. Schon Hannover hat sie gesehen, aber ihre Deutung als Stützsubstanz, die sich namentlich auf den Nachweis der Verlängerung der Fortsätze bis zur Pia mater stützt, ist neueren Ursprungs. Für gewöhnlich wird diese Entdeckung Golgi zugeschrieben, doch macht schon Lenhossék[1] darauf aufmerksam, dafs bereits Hensen 1876 die Fortsätze der Epithelzellen bis zur Pia hin verfolgt hätte. Aber auch Hensen ist nicht der Entdecker dieser Thatsache, ist auch nicht derjenige, welcher zuerst aus ihr den Rückschlufs gemacht hat, dafs man es hier mit einer Stützsubstanz zu thun hat. Beide Verdienste kommen einzig und allein Mauthner zu, der schon 1861 (Wiener acad. Sitzungsber.) mit kurzen, aber absolut klaren Worten die Sachlage festgestellt hat. Um dem verstorbenen Gelehrten wieder zu seinem Rechte zu verhelfen, sei die betreffende Stelle hier wörtlich wiedergegeben:

„Die den Centralkanal auskleidenden Epithelzellen mit den von ihnen ausgehenden Fortsätzen, von welchen einzelne Forscher, wie Stilling, zu glauben geneigt sind, dafs sie nervöse Gebilde seien, sind unbedingt samt den Fortsätzen der Pia mater dem Stützgewebe des Rückenmarks beizuzählen. Ich war namentlich so glücklich, im obersten Teil des Hechtrückenmarks von den nach rückwärts gelegenen Epithelzellen des Centralkanals kolossale Fortsätze abgehen zu sehen, welche ohne mit irgend welchen anderen zelligen Elementen in Zusammenhang zu treten, bis an die Peripherie des Rückenmarks gelangten und in den Fasern der Pia mater untergingen."

Durch Anwendung der Golgischen Methode war es nun ein leichtes, die Existenz solcher bis zur Pia reichender Epithelfortsätze als etwas ganz regelmäfsiges in den früheren Stadien der Ontogenese und Phylogenese nachzuweisen, und die Reste der mit solchen Aus-

[1] Der feinere Bau des Nervensystems. 2. Auflage. 1895. S. 210.

läufern versehenen Epithelien selbst im ausgebildeten Säugetiere aufzufinden. Jetzt konnte man auch durch den Nachweis von Zellgebilden, die man als Übergangsformen zu den eigentlichen Deiters schen Zellen auffassen konnte, die Ansicht aufstellen, dafs ontogenetisch, wie phylogenetisch die letzteren Zellen sich aus den Epithelzellen der Medullarplatte, d. h. aus denen der Ventrikel und des Centralkanals entwickeln.

Den meisten Lesern dieser Arbeit werden die Thatsachen, um welche es sich hier handelt, bekannt sein. Für diejenigen, welche in dieser Frage aber nicht orientiert sind, wird es vielleicht wünschenswert sein, ein Referat über den Stand der Angelegenheit zu bekommen. Wir benutzen für ein solches die Arbeit von Sala y Pons: La Neuroglia de los Vertebrados (Madrid 1894). Wir wählen diese, einmal weil die Darstellung eine sehr gute ist, und dann, weil für manche Leser ein Bericht gerade über die Arbeit von Sala y Pons erwünscht sein dürfte, da dieselbe nur spanisch erschienen ist und daher nicht jedermann zugänglich sein dürfte. Der Bericht ist in Petitschrift gedruckt, so dafs ihn diejenigen, die mit den Fragen vertraut sind, überschlagen können.

Sala y Pons sagt, dafs die Nervenzellen, die ja vom Ectoderm abstammen, eigentlich den alten Familientraditionen folgen und wie ihre Brüder, die Epithelien, in unmittelbarer Beziehung mit einander hätten stehen müssen, oder höchstens durch eine spärliche Kittsubstanz hätten getrennt sein dürfen. Aber unter diesen Verhältnissen hätten sie ihre Bestimmung nicht erfüllen können, da dann jede isolierte Übertragung von nervösen Strömen unmöglich gewesen wäre. Die mesodermatischen Elemente zu Hilfe zu rufen, war unmöglich; durch diese konnten sie also die für sie so nötige Isolierung nicht bekommen, und so verwandelten sich denn von Anfang an, während ein Teil der Zellen aus der Anlage des Centralnervensystems zu dem höheren Range der Nervenzellen sich entwickelte, andere Zellen zu Neurogliazellen um. Diese opferten freilich ihren Ehrgeiz, wurden aber doch zu einem zwar bescheidenen, aber immerhin sehr nützlichen Gewebsbestandteil, ohne den das richtige Funktionieren der Nervenmaschine nicht möglich gewesen wäre. (S. 6.)

Sala y Pons giebt dann weiter eine Zusammenfassung der von ihm, Lenhossék, Ramón y Cayal etc. gewonnenen Resultate. Zunächst (S. 36) stellt er vom ontogenetischen Standpunkte aus fest, dafs die primitiven Zellen, welche sich als Stützsubstanz zwischen die nervösen Elemente einschieben, die epithelialen Zellen sind. Ihre Körper bilden einen Wall, der die inneren Höhlen der nervösen Centralorgane begrenzt (Ependym). Sie sind mit Wimpern versehen und schicken einen feinen Fortsatz nach aufsen hin, der das ganze Organ durchsetzt und „mit dem charakteristischen Conus“ unter der Pia mater ansetzt (S. 37). Nach einiger Zeit vollzieht sich bereits der Übergang dieser Körper, indem der radiale, peripherwärts laufende Fortsatz sich teilt und an bestimmten Stellen dornige Anhänge erhält. Durch die Verzweigungen

im peripherischen Teile des Fortsatzes wird der Ansatz an die Pia mater fester als vorher, und die Fortsätze ziehen nun gewissermafsen mit amöboiden Bewegungen den Zellkörper mehr nach aufsen, so dafs immer weniger Elemente die innere Oberfläche begrenzen, und diese daher bei fortschreitender Entwicklung kleiner wird, so zwar, dafs schliefslich beim Erwachsenen die Höhlen sehr reduziert sind.[1]

Je weiter die Entwicklung fortschreitet, desto mehr nähert sich der Zellkörper der äufseren Oberfläche, wobei er unregelmäfsiger und zottiger wird und nur die Fixierung an der Pia mater und die noch vorhandene radiäre Orientierung zeigt noch an, dafs man es mit einem Abkömmling der Epithelzellen zu thun hat. Ein Schritt weiter und die Verbindung mit der Pia hört auf, der Zellkörper liegt frei mitten in der nervösen Substanz, nach allen Richtungen seine Fortsätze aussendend, die zart und gebogen (flexuosos) sind und so den Charakter der wahren Spinnenzellen aufweisen. Es handelt sich also bei der Bildung der letzteren weder um eingewanderte Mesodermzellen, noch um indifferente Abkömmlinge des Ectoderms, sondern die Epithelien wandeln sich Schritt für Schritt in Spinnenzellen um.

(S. 38.) Diese ontogenetische Stufenfolge macht sich auch phylogenetisch geltend, ja auch unter den verschiedenen Abteilungen des Centralnervensystems einer und derselben Tierart haben diejenigen, welche eine ältere Abstammung haben, differenziertere Formen, als die, die auf einer kleineren phylogenetischen Ahnenreihe beruhen. So finden wir bei den Vögeln im Rückenmark und Kleinhirn richtige Spinnenzellen, im Grofshirn aber Übergangsformen. Bei Amphibien und Reptilien finden wir Übergangsformen im Rückenmark; in der Hirnrinde und im Lobus opticus aber als Stützsubstanz nur epitheliale Zellen. Ja, in denselben Organen finden wir Unterschiede. So bei den Fischen. Hier sind im eigentlichen Kleinhirn Neurogliazellen vorhanden, die denen der Säugetiere ähneln, in der Valvula cerebelli aber primitive Formen, durchaus entsprechend dem Umstande, dafs die Valvula cerebelli der Fische auch sonst einen mehr embryonalen Charakter besitzt.

Man kann nach Sala y Pons ferner zeigen, dafs beiderlei Formen, die epithelialen und die Deitersschen Zellen, sich in der Verrichtung derselben Funktion ersetzen können. So sind einzig und allein epitheliale Zellen als Stützsubstanz bei geringerer ontogenetischer oder phylogenetischer Entwicklung vorhanden (Rückenmark der Fische. Hirnrinde der Amphibien und Reptilien, nervöse Centralorgane der Säugetiere am Anfang der Entwicklung), während umgekehrt die Spinnenzellen bei höheren Entwicklungsstufen das Feld beherrschen (Rückenmark der Vögel und Säugetiere, Hirnrinde und Kleinhirn der letzteren), in den Zwischenstufen (Hirnrinde, Lobus opticus der Vögel) finden sich sowohl epitheliale Zellen, als solche Elemente, welche sich genügend der Spinnenzellenform nähern. Dem entspricht es auch, dafs mit der Zunahme der Dicke der Organe die epithelialen mit ihren Fortsätzen bis zur Peripherie reichenden Stützzellen mehr und mehr abnehmen. —

---

[1] Sala übersieht dabei, dafs die Verengerung nur eine relative ist. Absolut genommen ist ja die Oberfläche der Ventrikelhöhlen eines erwachsenen Menschen z. B. ungeheuer viel gröfser, als die eines menschlichen Embryo. Es findet also keine Verminderung, sondern eine erhebliche Vermehrung der Ependymzellen statt.

So weit die Salasche Darstellung. Ganz so einfach ist freilich die Sache nach Ansicht anderer Autoren nicht. Selbst diejenigen, welche durchaus auf dem Standpunkt stehen, dafs die Neuroglia ectodermatischen Ursprungs ist, weichen in mancher Hinsicht von den Meinungen ab, die bei Sala vorgetragen werden.

So ist z. B. schon Lenhossék,[1] dem wir sehr sorgfältige Arbeiten über die Embryologie der Neuroglia verdanken, nicht ganz mit Sala y Pons in Uebereinstimmung. Auch nach seiner Meinung entstehen zwar Astrocyten in der Weise, dafs die mit langen Fortsätzen versehenen Ependymzellen nach aufsen rücken, u. zw. Ependymzellen, die ganz denen entsprechen, welche bei ganz jungen Embryonen die alleinige Stützsubstanz darsellen, also Flimmern („ein Härchen") tragen und einen peripherischen, bis zur Pia reichenden radiären Fortsatz besitzen. Aber, und hierin liegt eine wesentliche Differenz gegenüber Sala y Pons, nur ein Teil der Deitersschen Zellen, wenigstens der höheren Säugetiere, entsteht auf diese Art, für einen anderen Teil kann man dies nicht nachweisen, sondern dieser entsteht in einer von den Ependymzellen nicht so direkt abhängigen Weise. „Ziemlich unvermittelt tauchen, wenn der Embryo (sc. der menschliche) ungefähr 20 Cm. lang ist, die Spinnenzellen schon in ihrer charakteristischen Form . . . auf, und bei vielen fehlt jeder Hinweis darauf, dafs sie sich aus den Radiärzellen entwickelt haben. Dann ist die Zahl der späteren Spinnenzellen im menschlichen Rückenmark auch viel zu grofs, als dafs man sie alle auf frühere Radiärzellen, die eine viel beschränktere Zahl aufweisen, zurückführen könnte." (S. 234.) Er meint daher, dafs diese Zellen durch einen caenogenetisch abgekürzten Entwickelungsmodus entstehen, indem sie nicht durch jenes radiär-faserige Stadium hindurchgehen, sondern aus Keimzellen entstehen, die anfangs fortsatzlos sind, sich aber bald mit allseitig sie umgebenden Fortsätzen versehen.

Noch weiter gehen Vignal und Kölliker. Sie lassen alle Neurogliazellen aus indifferenten Zellen entstehen, von denen einige Neuroblasten, andere Ependymfaserzellen, noch andere Spinnenzellen erzeugen.

Nach der Ansicht der bisher genannten Autoren, die gegenwärtig von den meisten, auch von Retzius, geteilt wird, gehen aber diese (auch die nach der Meinung einiger Forscher indifferenten) Anlagen der Spinnenzellen aus dem Ectoderm der Medullaranlage hervor, nicht aus mesoblastischen Einwanderern. Dieser Ansicht schliefst sich auch

---

[1] Der feinere Bau des Nervensystems. 2. Auflage. 1895.

Schrader an, dessen Auffassung der einschlägigen Verhältnisse uns weiter unten besonders beschäftigen wird.

Aber so verbreitet gegenwärtig diese Ansicht auch ist, ganz ohne Gegner ist sie nicht. Nicht nur, dafs einige, Lacchi und Valenti z. B., einen gemischten Ursprung der Deitersschen Zellen annehmen, d. h. sie teils aus dem Ectoderm entstehen, teils aus dem Mesoderm einwandern lassen, so hat vor allem kein geringerer, als His, eine absolut andere Auffassung der Entstehung der Spinnenzellen, wie Vignal, Kölliker, Ramón y Cajal, Lenhossék, Retzius etc. Auch er nimmt zwar an, dafs aus dem Ectoderm der Medullarplatte ein Teil der Zellen nicht zu Nervenzellen (Neuroblasten) wird, sondern eine Gerüstsubstanz erzeugt, aber gerade diese letzteren Zellen, die „Spongioblasten", haben mit den Deitersschen Zellen garnichts zu thun. Die Deitersschen Zellen sind vielmehr nach His sämtlich eingewanderte mesoblastische Gebilde, die also gar keine Beziehung zu der ectodermatischen Anlage des Centralnervensystems besitzen, d. h. diejenige Neuroglia, die wir in unseren Präparaten allein nachweisen können, ist echte Bindesubstanz auch im histogenetischen Sinne, wenn wir uns der Ansicht von His anschliefsen. — —

Wir haben in Kürze den gegenwärtigen Stand der Frage nach der histogenetischen Stellung der Neuroglia im vorstehenden besprochen, und wir müssen nun untersuchen, wie unsere eigenen Anschauungen mit den embryologischen Erfahrungen in Einklang zu bringen sind. Zunächst kann man wohl das eine sagen, dafs ein doppelter Ursprung der von uns dargestellten Neuroglia im höchsten Grade unwahrscheinlich ist.[1] Die Neurogliafasern in unseren Präparaten sind morphologisch und chemisch so einheitlich und so charakteristisch beschaffen, dafs man nicht glauben kann, ein Teil derselben entstamme dem Mesoderm, ein anderer dem Ectoderm, also zwei sehr verschiedenen Ursprungsstellen. Es bliebe also nur die Möglichkeit übrig, dafs unsere Neuroglia insgesamt entweder mesodermatischen oder ectodermatischen Ursprungs wäre.

Leider ist unsere Methode für embryologische Untersuchungen nicht geeignet, da ja in den früheren, hier allein in Betracht kommenden Entwickelungsstufen noch keine abgesetzten Fasern bestehen. Wir können daher nur theoretisch untersuchen, in welcher Weise unsere Resultate mit den von anderen Autoren gewonnenen Anschauungen in Einklang zu bringen sind.

---

[1] Wenn im folgenden von Neuroglia kurzweg gesprochen wird, so ist darunter nur die in unseren Präparaten in Form blauer Fasern hervortretende gemeint.

im höheren Alter von interstitiellen Fasern durchwachsen werden, so ist vor allem das histogone Verhalten der Medullarplatte ein ganz eigenartiges, paradoxes. Sie erzeugt ja aus ihren Epithelzellen die so reich verzweigten Nervenzellen, also (ganz abgesehen von deren physiologischem Charakter) in ihrer Form durchaus von anderen Epithelabkömmlingen abweichende Elemente. Die nervösen Zellen haben ferner in früher Embryonalperiode ein ebenfalls bei epithelialen Gebilden sonst ganz unerhörtes Wanderungsvermögen. Auch die Formen der embryonalen Neurogliazellen weichen ihrer zahlreichen langen Ausläufer wegen von allen sonstigen Epithelien ab. Unter diesen Verhältnissen kommt es schliefslich garnicht darauf an, ob zu den übrigen paradoxen Eigenschaften der Abkömmlinge der Medullarplatte noch eine weitere dazu kommt: die Erzeugung differenzierter Fasern, für die wir an den übrigen Epithelien nur eine ganz entfernte Analogie in der Erzeugung von Cuticularsubstanzen finden. Es ist aber mit Beziehung auf diese letzteren vielleicht doch nicht so ganz zufällig, dafs unsere Methode auch diese differenziert zu färben gestattet.

Man wird sich demnach vorzustellen haben, dafs die Natur auf zwei ganz verschiedenen Wegen denselben morphologischen und biologischen Effekt erreicht: sie erzeugt Bindegewebe als Stützsubstanz vom Mesoderm aus, Neuroglia als Bindesubstanz vom Ectoderm aus. Wenn man sich über die von anderen Epithelien so abweichende Form der Neurogliazellen in ihrem Embryonalzustande nicht gewundert hat, so mag man sich auch mit der Modifizierung und Emanzipierung der faserigen Bestandteile im ausgebildeten Körper abfinden. — —

Alle die Auseinandersetzungen in diesem Abschnitte sind nur, so zu sagen, vom grünen Tische aus gemacht. Das letzte Wort in dieser Angelegenheit haben die Embryologen zu sprechen. Aber, wie auch ihre Entscheidung ausfällt, um die Thatsache, dafs die Neuroglia morphologisch und biologisch sich wie eine Bindesubstanz verhält, kann man jetzt nicht mehr herumkommen.

6. Abschnitt:
Anderweitige histologische Eigenschaften der Neurogliafasern.

Wir hatten bis jetzt von histologischen Eigentümlichkeiten der, wie wir also jetzt bestimmt sagen können, „Neuroglia"-Fasern nur das Verhältnis dieser Fasern zu den Zellen besprochen. Wir mufsten dann die weitere Schilderung des mikroscopischen Verhaltens der Fasern aussetzen, weil zuerst einmal die Natur derselben aufgeklärt werden mufste. Nunmehr können wir die anderweitigen Eigenschaften dieser Gebilde besprechen, zumal wir jetzt auch in die Lage versetzt sind, die nötigen Vergleiche mit den Angaben früherer Autoren zu machen, Vergleiche, welche so lange nicht angestellt werden konnten, als nicht die Identität unserer Fasern mit dem, was bisher als „Ausläufer der Deiters-schen Zellen" etc. beschrieben wurde, definitiv festgestellt war.

Genau so, wie die bereits erörterten Verhältnisse der Neurogliafasern zu den Neurogliazellen, gelten auch die folgenden Eigenschaften der Fasern für das gesamte Centralnervensystem, für die grauen, wie die weifsen Massen, für Grofshirn, Kleinhirn, Rückenmark etc. Unterschiede sind nur in Bezug auf die Menge und Anordnung der Neuroglia vorhanden, aber diese Unterschiede sind grofs genug, um sehr wesentliche Differenzen an den verschiedenen Örtlichkeiten im Centralnervensystem statuieren zu können. Diese Differenzen werden uns in den der Topographie gewidmeten Abschnitten beschäftigen. Jetzt wollen wir die gemeinschaftlichen Eigentümlichkeiten der Fasern durchsprechen.

1. Die Fasern sind mehr oder weniger gerade (natürlich nicht im mathematischen Sinne), oder sie verlaufen in starr geschwungenen Biegungen. Niemals sind sie eng geschlängelt. Findet man sie doch in einem Präparate in engen vielfachen Krümmungen verlaufend, so kann man sicher sein, dafs die Präparate geschrumpft sind. Man kann sogar die Schlängelung der Fasern künstlich erzeugen, wenn

man z. B. Stücke aus dem Centralnervensystem einer energischen Oxalsäurebehandlung unterzieht. Kann man schon makroscopisch die Schrumpfung der Stücke erkennen, so kann man auch sicher sein, je nach dem Grade der Schrumpfung mehr oder weniger enge Schlängelungen der Fasern mikroscopisch wahrzunehmen.

2. Die Fasern sind durchaus solide, eine Höhlung ist an keiner zu entdecken. Auf dem Querschnitt erscheinen sie alle als blaue Punkte, nicht als Kreise, wie es der Fall sein müfste, wenn die Fasern hohl wären, so dafs wir uns in diesem Punkte der Ansicht von Frommann und aus neuerer Zeit der von Lavdowsky nicht anschliefsen können.

3. Die Fasern sind ganz glatt, ohne „körnige Beschaffenheit", ohne umschriebene Auftreibungen und Verdickungen. Doch gilt dies nur für frisch eingelegte und sorgfältig gehärtete Präparate. Hat man hingegen z. B. ein Rückenmark, das beim Durchschneiden im ungehärteten Zustande auf seiner Schnittfläche die weifse Substanz vorquellen läfst, das also schon die kadaveröse Quellung der Markmassen zeigt, so kann man ganz sicher sein, dafs man dann einen körnigen Zerfall der Fasern findet (oder dafs man die Fasern überhaupt nicht mehr färben kann, s. u.). Diesen kadaverösen Zerfall der Fasern hat Frommann zuerst beschrieben, Virchow hatte aber schon im allgemeinen bemerkt, dafs die „Neuroglia" durch postmortale Einflüsse zerstört wird.

Die kadaverösen Zerfallskörnchen sind anfangs klein, in der Richtung der Fasern liegend, bei stärkerer postmortaler Schädigung werden sie gröfser, die kleinen Tröpfchen fliefsen förmlich zusammen, und die so entstandenen gröfseren Tropfen liegen weiter auseinander und unregelmäfsig verteilt. Schliefslich scheinen sie sich aufzulösen, jedenfalls kann man an ganz schlechten Stücken keine Färbung mehr erzielen. Auch die Körnchen der früheren Zerfallsstadien färben sich schon schwerer, als die normalen Fasern.

Die varikösen Neurogliafasern (Zellausläufer), die manche Autoren (bei Anwendung der Golgischen Methode) abbilden resp. beschreiben, sind wohl auch nichts anderes als kadaverös bereits veränderte gewesen.

Wie der körnige Zerfall zu Stande kommt, ist fraglich. In meiner vorläufigen Mitteilung vom Jahre 1890 habe ich bereits darauf aufmerksam gemacht, dafs die kadaveröse Quellung des Myelins hierbei eine Rolle zu spielen scheint. Wenigstens sind, wie ich damals schon anführte, die weifsen Substanzen diejenigen, die den Zerfall zuerst zeigen. Es wäre ja auch nicht undenkbar, dafs die kadaverös erweichten Neurogliafasern durch die quellenden Markscheiden zersprengt würden. —

4. Ebensowenig wie Varicositäten zeigen die Neurogliafasern in unseren Präparaten irgend welche moosartigen oder sonst wie beschaffenen Ansätze. Ramón y Cajal beschreibt derartige Strukturen an embryonalen Neurogliazellen und bei niederen Tieren. Bei diesen ist es z. T. sogar so, dafs ein und derselbe Zellausläufer je nach den Schichten, die er passiert, glatt oder mit moosartigen Rauhigkeiten besetzt erscheint. Wenn man hier nicht etwa Kunstprodukte annehmen will, so wird man daher diese Ansätze als ein vorübergehendes phylogenetisches oder ontogenetisches Entwicklungsstadium der Zellausläufer ansehen können, das im ausgebildeten Centralnervensystem des Menschen keine Spuren zurückgelassen hat.

5. Endlich zeigen unsere Fasern niemals etwas von jenen konischen oder flaschenförmigen Erweiterungen, wie sie von Golgipräparaten so vielfach geschildert werden. Der Ansatz der „Zellausläufer“ an Gefäfsumgrenzungen, an freie Oberflächen überhaupt, soll nach diesen Schilderungen immer mit einer solchen Verbreiterung enden. An unseren Präparaten sind diese Ansätze in keiner Weise verdickt, die Faser ist bis zu ihrem Ende so schlank und gleichmäfsig, wie in ihrem früheren Verlaufe. Da nun unsere Färbung eine elektive ist, so sind die mit ihr gewonnenen Resultate jedenfalls die mafsgebenden. Man mufs demnach annehmen, dafs sich bei der Golgischen Methode irgend etwas mitfärbt, was nicht zur Faser gehört, resp. was eine andere chemische Beschaffenheit, wie diese, besitzt.

Was dieses „etwas“ ist, ist schwer zu sagen. Vielleicht handelt es sich um eine (bei unserer elektiven Färbung natürlich unsichtbare) Kittsubstanz. Es könnte aber auch sein, dafs sich der Silberniederschlag einfach zwischen die Oberfläche des Organs und die letzten (sehr oft schief umgebogenen) Enden der Fasern absetzt, so dafs also ein reines Kunstprodukt vorläge.

6. Die Fasern sind von verschiedener Dicke, von den allerfeinsten, nur bei guter Färbung sichtbar zu machenden bis zu 1,5 μ Dicke. Die ganz dicken Fasern kommen nur unter pathologischen Verhältnissen vor, namentlich bei der progressiven Paralyse in der Grofshirnrinde, doch sieht man etwas dünnere, aber immer noch recht dicke Fasern manchmal auch unter anscheinend normalen Verhältnissen beim Menschen, ganz besonders im Hinterhorn des Rückenmarks und den entsprechenden Stellen der Medulla oblongata. Diese Fasern strahlen auch von Centren aus, in denen Kerne liegen, so dafs man solche Gebilde, wenn man sie nach der alten Ausdrucksweise als „Zellen“ bezeichnen will, „Monstrezellen“ nennen kann, wie ich das in meiner vorläufigen Mitteilung vom Jahre 1890 gethan

habe. Diese sehr auffallenden Gebilde in anscheinend normalen Teilen scheinen bis dahin der Aufmerksamkeit ganz entgangen zu sein. Die dicken Fasern bei progressiver Paralyse hingegen sind schon mehrfach gesehen und abgebildet worden (natürlich als „Zellausläufer“).

Wir kommen jetzt zu zwei die Neurogliafasern betreffenden Fragen, die eine ganz nebensächliche Bedeutung haben, aber von den Histologen in der neueren Zeit als wer weifs wie wichtige Dinge behandelt wurden.

7. Das eine ist die Frage, ob die Neurogliafasern sich teilen, oder nicht. Diese Frage hätte ein gröfseres Interesse für sich zu fordern gehabt, wenn sie diagnostisch für den Unterschied gegenüber den Ausläufern von Ganglienzellen, also auch für den Unterschied der Ganglienzellen und Neurogliazellen selbst verwendet werden konnte (n. b. bei Betrachtung von Golgipräparaten). Wir haben aber im Abschnitt III gesehen, dafs die Angaben der Autoren eine solche diagnostische Verwertung der Teilungen nicht zulassen, so dafs in dieser Beziehung jedes Interesse an denselben fortfällt.

Man darf auch die Wichtigkeit dieser Frage nicht im entferntesten vergleichen mit der der gleichen Frage bei den Ganglienzellausläufern. Bei den Nervenelementen ist die Verzweigung der Zellausläufer von höchstem physiologischem Interesse, da dadurch die Möglichkeit ungeheuer vieler Verbindungen der Neurone gegeben wird, — ein Moment, das bei einer Intercellularsubstanz garnicht in Frage kommt.

So wollen wir denn auch nur kurz erwähnen, dafs wir an unseren Präparaten Teilungen der Fasern nicht bemerkt haben. Die Teilungen, welche man an Golgi-Präparaten beobachtet hat, können (so weit nicht embryonale Verhältnisse in Betracht kommen) vielleicht dadurch erklärt werden, dafs bei der Silberimprägnation zwei sehr nahe an einander liegende Faserteile zu einer gemeinschaftlichen Silhouette verschmelzen, etwa, wie es Ranvier annahm, durch Mitfärbung einer verkittenden Substanz, doch ist die Frage zu gleichgültig, um etwa eingehendere Untersuchungen darüber anzustellen.

8. Eine zweite ebenso untergeordnete Frage ist die, ob die Neurogliafasern mit einander anastomosieren oder nicht. Auch hier hat man die Wichtigkeit einer solchen Frage bei den nervösen Elementen ganz falscher Weise auf die bei den interstitiellen übertragen. Bei den nervösen Elementen ist der Nachweis des Fehlens von Anastomosen deshalb physiologisch vom höchsten Interesse, weil nur bei fehlenden Ana-

stomosen eine Isolierung der Neurone denkbar ist. Bei einer Zwischensubstanz ist das Fehlen oder Vorhandensein von Anastomosen aber etwas absolut gleichgültiges, — und doch sind die Histologen sogar so weit gegangen, Golgi ein besonderes Verdienst daraus zu machen, dafs er die Nichtanastomosierung der Fasern zuerst konstatiert hat, dafs er gezeigt hat, die Fasern bildeten ein „Geflecht" und kein „Netz"!!

Lesern, denen die Sprechweise der modernen Neurohistologen unbekannt ist, wird es auffallend erscheinen, dafs man die Worte „Netz" und „Geflecht" in einen Gegensatz bringt, da doch ein Netz auch ein Geflecht ist. Ein Drahtnetz ist doch auch ein Drahtgeflecht. Aber man nennt nun einmal ein „Netz" eine solche Durchflechtung von Fäden, Zellausläufern und dergl., bei denen diese an den Berührungsstellen mit einander verschmelzen, anastomosieren, „Geflecht" eine solche, bei der Anastomosen nicht vorhanden sind.

Bei der minimalen Wichtigkeit dieser Frage genügt es auch wieder, wenn wir erwähnen, dafs auch wir, soweit es das Fasergewirr gestattete, nichts von Anastomosen bemerkt haben.

Um Irrtümer zu vermeiden, sei aber darauf hingewiesen, dafs sowohl das Fehlen der Teilungen, wie das der Anastomosen, häufig nur durch Heben oder Senken des Tubus zu entschleiern war, und dafs daher in unseren Zeichnungen, in denen die Niveaudifferenzen nicht wiedergegeben werden konnten, Teilungen oder Anastomosen vorgetäuscht werden, die in Wirklichkeit nicht vorhanden waren.

7. Abschnitt:

Allgemeine Topographie der Neurogliafasern.

Die topographische Anordnung der Neurogliafasern ist eine sehr mannigfaltige, wenn auch für jede Stelle des Centralnervensystems durchaus charakteristische. In dieser Mannigfaltigkeit treten aber gewisse Gesetzmäfsigkeiten auf, die es uns ermöglichen, wenigstens einige allgemeine Regeln über die Verteilung der Neuroglia aufzustellen.

1. Geradezu als Gesetz, das keine wirkliche Ausnahme besitzt, kann zunächst der Satz aufgestellt werden, dafs unter dem Epithel der Ventrikel und des Centralkanals stets eine dicke Schicht sehr eng verwebter Neurogliafasern liegt, und dafs diese Geflechte die dichtesten sind, die im Centralnervensystem normaler Weise vorkommen (vgl. z. B. Taf. III, Fig. 2 und 3, Taf. X, Fig. 1, Taf. XI, Fig. 1 u. a.).

Eine scheinbare Ausnahme von dieser Regel stellt sich nur am Plexus chorioideus ein. Auch dieser ist ja mit Ventrikelepithel bekleidet, aber unter diesem Epithel findet sich eine ependymäre Neurogliamasse nur an denjenigen Stellen, an welchen der Plexus sich mit nervösen Massen verbindet (z. B. an der Fimbria). Von hier aus geht die Neuroglia noch eine Strecke weit in den Ansatz des Plexus chorioideus hinein. Alle übrigen Teile des Plexus chorioideus haben aber unter dem Epithel keine Neuroglia, sondern Bindegewebe; es ist aber auch in den tieferen Schichten im Plexus weiter keine Neuroglia nachzuweisen (mit Ausnahme eben der Ansatzstellen an nervöse Teile).

Die Dichtigkeit der ependymären Neurogliamassen ist ja bis zu einem gewissen Grade schon lange bekannt. Hat doch schon Virchow vor 50 Jahren diese Stellen besonders hervorgehoben.

Bei unserer Färbung tritt die Massenhaftigkeit der Neuroglia aber besonders deutlich hervor, da ja jede einzelne Faser distinct gefärbt erscheint. Das Geflecht ist so dicht, dafs man sich fragt, ob denn aufser der Lymphe (oder was sonst die Maschen ausfüllt) noch

etwas anderes Platz hat, und doch wissen wir, dafs z. B. in der hinteren Commissur des Rückenmarks massenhafte Nervenfasern eingebettet sind. Wenn man genauer zusieht, so bemerkt man aber doch, dafs noch Raum genug für die feinen Nervenfasern vorhanden ist. Der erste Eindruck, den man bei Betrachtung dieser dichten Neurogliamassen hat, ist vielmehr durch einen rein psychologischen Vorgang bedingt. Jede vollständige Färbung hat eben etwas aufdringliches an sich. Sie erweckt, wenn die gefärbten Elemente sehr dicht liegen, gar zu leicht die Idee, dafs diese ganz allein den Platz beherrschen.

---

2. Ein weiteres, aber doch nicht ganz ausnahmsloses Gesetz ist das, dafs die äufseren Oberflächen im Centralnervensystem ebenfalls eine Verdichtung der Neuroglia aufweisen, die aber im allgemeinen nicht so eng gewebt und so dick ist, wie die ependymären Anhäufungen (vgl. Taf. I, Fig. 2 und 3, Taf. VII, Fig. 4, Taf. IX, Fig. 1, Taf. X, Fig. 2, Taf. XI, Fig. 2).

Seit sehr langer Zeit bekannt ist dies Gesetz für das Rückenmark, dessen Rindenschicht schon längst als eine besonders dichte, wie man früher glaubte, von Nervenelementen ganz freie Neurogliaanhäufung betrachtet wurde. Genauer beschrieben haben sie zuerst Clarke und Frommann. Die Rindenschicht am Grofshirn hat Golgi zuerst geschildert, und mit unserer Färbung kann man sich leicht überzeugen, dafs so ziemlich alle Teile des Centralnervensystems solche verdichteten Rindenschichten aufweisen, aber doch mit einer, ebenfalls zuerst (1871) von Golgi erkannten Ausnahme: der Oberfläche des Kleinhirns, wie ich das auch 1890 hervorgehoben habe (Taf. IX, Fig. 5). Unter krankhaften Verhältnissen freilich ändert sich hier das Bild, und bei progressiver Paralyse z. B. findet sich an der Kleinhirnoberfläche oft eine typische dichte „Rindenschicht".

---

3. Diese beiden ersten Gesetze gelten aber nicht nur für die beim ausgebildeten menschlichen Centralnervensystem gegenwärtigen, sondern auch bis zu einem gewissen Grade für die früher vorhandenen, aber bei der fortschreitenden Entwickelung wieder verschwundenen inneren und äufseren Oberflächen. Wie die vom Kiel eines Schiffes gestörte Meeresoberfläche noch lange und weithin durch eine Furche den früheren Gang des Schiffes erkennen läfst, so lassen die verschwundenen inneren und äufseren Oberflächen nach ihrer Verwachsung als Spuren noch mehr oder weniger breite, mehr oder weniger lange, mehr oder weniger dichte Neurogliaanhäufungen zurück. Wir

werden derartige Neurogliaverdichtungen in der Darstellung der speziellen Topographie mehrfach (z. B. beim Ammonshorn) erwähnen müssen, und wollen sie, da man für diesen neuen Begriff doch ein neues Wort braucht, mit Rücksicht auf das obige Gleichnis als „Kielstreifen" bezeichnen: als „Streifen" deshalb, weil sie wohl meist streifenförmig sich darstellen. Vielleicht können diese Kielstreifen gelegentlich auch einmal zur Entscheidung entwickelungsgeschichtlicher Fragen verwendet werden. Diese Kielstreifen stehen an einem Ende immer noch mit einer inneren oder äufseren Oberfläche in Verbindung, das andere liegt in der Tiefe der betreffenden Teile des Centralnervensystems.

4. Aber nicht nur die äufseren Oberflächen und die subepithelialen Partieen weisen eine Verdichtung der Neuroglia auf, sondern auch andere Stellen, bei denen sich im Innern, d. h. in der Tiefe der nervösen Teile oberflächenartige Abgrenzungen finden. Solche Fälle treten z. B. ein:

a) wenn sich die Nervenfasern der weifsen Substanz in abgesetzte Bündel formieren. Unter diesen Umständen bildet sich an der Oberfläche der Bündel häufig eine verdichtete Randschicht aus, aber diese Verdichtungen sind nicht nur geringfügiger, als die eigentlichen Rindenschichten der freien Oberflächen, oder gar als die ependymären Massen, sondern sie treten überhaupt nur an gröberen Bündeln und auch da nicht immer deutlich erkennbar auf. Als Beispiele für diese Randverdickungen seien die an der Pyramidenkreuzung (Taf. V, Fig. 3) und an den Opticusbündeln (Taf. VII, Fig. 4a) erwähnt.

b) Ebenfalls geringfügig und durchaus nicht regelmäfsig sind die Neurogliaverdichtungen um grofse Ganglienzellen herum (z. B. Taf. II, Fig. 2 u. a.), die „Neurogliakörbe" um dieselben. Besonders häufig sind sie um die grofsen Zellen der Vorderhörner des Rückenmarks, sowie um die entsprechenden der Medulla oblongata und des Pons. Ganz regelmäfsig finden sie sich um die zerstreuten einzelliegenden Ganglienzellen in letzteren Organen. Hingegen vermifst man sie ganz oder findet sie nur in Form vereinzelter Fädchen angedeutet dann, wenn in der weiteren Umgebung der Ganglienzellen Neurogliafasern überhaupt sehr spärlich oder gar nicht vorhanden sind. So ist es z. B. in den tieferen Schichten der Grofshirnrinde. Im übrigen lassen sich bestimmte Regeln nicht aufstellen.

c) Sehr mächtig können aber die Neurogliamassen an den Grenzen der die Gefäfse bergenden Räume werden, sogar mächtiger, als an der äufseren Oberfläche.

Auch das ist schon lange bekannt und z. B. schon von Virchow hervorgehoben worden. Die besonders starken Verdickungen der Neuroglia sind freilich nur in der Umgebung gröfserer Gefäfse zu bemerken, an kleineren pflegen sie viel geringfügiger, aber immerhin doch meist angedeutet zu sein (vgl. Taf. IX, Fig. 2 und 3). Nur da, wo in der weiteren Umgebung der Gefäfse Neurogliafasern ganz oder fast ganz fehlen, habe ich auch um die Gefäfse herum eine Neurogliaanhäufung entweder ganz vermifst, oder nur durch feine spärliche Fäserchen angedeutet gefunden. So ist es wieder in der Tiefe der Grofshirnrinde.

Was die Richtung der Neurogliafasern, die die Gefäfse umscheiden, anbelangt, so ist dieselbe anscheinend eine der Gefäfsaxe überwiegend parallele (intrinsic fibres von Lloyd Andriezen[1]). Doch macht Lloyd Andriezen mit Recht darauf aufmerksam, dafs diese anscheinend parallele Richtung eigentlich eine spiralige ist. Man sieht daher nicht blofs auf reinen Längsschnitten, sondern auch auf Schiefschnitten die Neuroglia in der Umgebung der Gefäfse als Fasern und nicht als Pünktchen. Als Pünktchen müssen sie ja dann erscheinen, wenn der Schnitt die Fasern senkrecht zu ihrem Verlaufe trifft (vgl. Taf. IX, Fig. 3. Unten sind die Fasern als Pünktchen zu sehen). Ja die Spiralwindungen können so enge sein, dafs auch auf reinen Querschnitten durch die Gefäfse die Neuroglia in Form langer Fasern erscheint.

Aber der spiralige, der Axe mehr parallele, resp. der concentrische Verlauf der Neurogliafasern ist nicht der einzige, den sie in der Umrandung der Blutgefäfse zeigen. Es kommen vielmehr auch Fasern genug vor, die in radiärer Richtung, oft von weither, dem Gefäfse zustreben und sich dann schief umbiegend den übrigen Fasern beigesellen (extrinsic fibres von Lloyd Andriezen). Sehr charakteristische Bilder entstehen dann, wenn sich diese extrinsic fibres bis zu einem Kerncentrum hin verfolgen lassen. Solche Fasern wurden in nicht ganz zutreffender Weise zuerst von Roth[2] beschrieben. Er hatte Paraffinpräparate benutzt, für die die Technik damals noch nicht ausgebildet war, und bekam daher eigentümliche Schrumpfungen. Durch diese wurde die Täuschung veranlafst,

---

[1] On a system of fibre-cells surrounding the blood-vessels of the brain of man and mammals. Internationale Monatsschrift für Anatomie 1893.

[2] Virchows Archiv. Band 46 (1869), S. 243. Zur Frage der Bindesubstanz in der Grofshirnrinde.

dafs die radiären Fasern, ehe sie an das Gefäfs herantreten, einen leeren (Lymph-) Raum durchzögen. Golgi[1] hat dann diesen Irrtum berichtigt, und er war so der erste, der in sachgemäfserer Weise die extrinsic fibres (natürlich als „Zellausläufer") schilderte. In unseren Präparaten sind solche Fasern oft genug zu sehen (z. B. auch Taf. IX Fig. 2 oben). Sehr reichlich sind sie an etwas gröfseren Gefäfsen oft zu finden, und ich habe zur Illustration dieser Verhältnisse eine besondere Abbildung Taf. VIII, Fig. 2 von einer Gefäfsumgrenzung im Pedunculus cerebri gegeben. Nach rechts hin ist der Gefäfsraum, dessen Inhalt in der Zeichnung weggelassen worden ist. Von links her strahlen sehr reichliche extrinsic fibres, die zu Kerncentren zu verfolgen sind, an die Gliahülle des Gefäfses heran und verlieren sich in dieser. Es sei noch einmal darauf hingewiesen (vgl. S. 69), dafs in unseren Präparaten niemals die konischen Verdickungen der Ansatzstellen zu sehen sind, wie sie an Golgi-Präparaten als etwas ganz regelmäfsiges beschrieben werden.

Ganz besonders schön und ganz regelmäfsig sieht man diese radiär der Gefäfsumgebung zustrebenden Fasern bei progressiver Paralyse in der Grofshirnrinde, also an einer Stelle, an welcher sie normalerweise durch unsere Methode sonst nur selten zu finden sind. Bei progressiver Paralyse finden sich nämlich in der Grofshirnrinde sehr zahlreiche, neugebildete „Astrocyten", die teils von der gewöhnlichen Beschaffenheit sind, teils aber (und zwar sehr oft) sogenannte „Monstrezellen" darstellen (vgl. S. 69). Die von diesen ausstrahlenden, oft sehr dicken Fasern haben nun die ausgesprochene Tendenz, nach den Gefäfsen in mehr oder weniger senkrecht-radiärer Richtung hinzustreben und sich hier (immer ohne Conus) zu inserieren. —

Wie sich der Leser vielleicht erinnert, hatte Golgi (vgl. S. 41 Anm.) in dieser innigen und verwickelten Verbindung der Neuroglia mit den Gefäfsen etwas so merkwürdiges zu sehen geglaubt, dafs er diesen Befund gegen Ranviers Ansicht von der Fasernatur der „Zellfortsätze" verwerten zu können meinte. Wir haben l. c. bereits darauf hingewiesen, dafs die Verhältnisse der Neuroglia zu den Gefäfsen garnicht inniger und komplizierter sind, als die der elastischen Fasern z. B., und wir haben schon daraus entnommen, dafs der Einwand Golgis nicht berechtigt war. Dazu kommt aber, was wir l. c. nur erst andeuten konnten, dafs die ganze Art der Neurogliaverdichtung um die Gefäfse herum nichts ist, als eine Teilerscheinung der so verbreiteten „Rindenschichtbildungen".

---

[1] Gesammelte Abhandlungen. S. 6 f., Taf. I, Fig. 4.

Die Gefäfse sind ja für das Centralnervensystem etwas genau so fremdes, wie die eigentliche Pia mater, und so ist denn die Grenze des Nervengewebes gegen ein Gefäfs nichts anderes, als eine innere Oberfläche, die den äufseren Oberflächen des Hirns und Rückenmarks durchaus entspricht. — Wenn wir ferner bedenken, dafs die Neurogliafasern doch wohl eine Stützsubstanz darstellen, und dafs solche Stützsubstanzen an vielen Stellen nachweislich nach mechanischen Prinzipien angeordnet sind, so werden wir uns auch über den verwickelten Bau der Neurogliahüllen um die Gefäfse herum nicht wundern. Wir werden dies um so weniger thun, als die äufseren Rindenschichten oft ganz analoge Verhältnisse aufweisen, wenn diese auch der abweichenden mechanischen Anforderungen wegen nicht absolut mit denen an den inneren Rindenschichten, d. h. an den Gefäfsgrenzen, übereinstimmen.

Auch an den äufseren Begrenzungen haben wir eine eigentliche Rindenschicht, d. h. eine dichte Neurogliamasse zu constatieren, die den intrinsic fibres der Gefäfse entspricht, und von dieser ausstrahlende, resp. in sie eintretende, zur Oberfläche senkrechte, mehr zerstreute Fasern, die also den extrinsic fibres analog sein würden. Welche mechanische Bedeutung die Neurogliahülle gerade der Gefäfse hat, werden wir gegen den Schlufs dieser Abhandlung besprechen, wo wir uns überhaupt mit der physiologischen Rolle der Neuroglia zu beschäftigen haben werden.

---

5. Was die allgemein-topographischen Verhältnisse der weifsen Substanzen im Centralnervensystem anbelangt, so läfst sich als allgemeine Regel aufstellen, dafs so ziemlich jede markhaltige Nervenfaser in den weifsen Substanzen von der benachbarten durch Neurogliafasern getrennt ist (vgl. Taf. I, Fig. 2, Taf. VI, Fig. 1, Taf. VII, Fig. 3 und 4, Taf. IX, Fig. 2 und 3 u. a). So entsteht ein im ganzen weitmaschiges Geflecht in den Markmassen.

Doch gilt dies Gesetz nur für die eigentlichen weifsen Massen. Da, wo zwischen die einzelnen markhaltigen Fibrillen graue Substanz eingefügt ist, kann wohl (und zwar sehr reichliche) Neuroglia ebenfalls dazwischen geschoben sein, sie braucht aber nicht vorhanden zu sein. Letzteres ist z. B. an den so mächtigen radiären Einstrahlungen in den tiefen Schichten der Grofshirnrinde der Fall. Hier ist eben keine eigentlich weifse Substanz, sondern graue, vorhanden, und für diese können wir, wie sich sub 6 zeigen wird, allgemeine Regeln nicht aufstellen.

Auch in den weifsen Substanzen ist aber das Neurogliageflecht durchaus nicht uniform zu nennen.

Wenn auch im allgemeinen jede Nervenfibrille von der anderen durch Neuroglia abgegrenzt ist, so ist die Anzahl der Neurogliafasern zwischen je zwei Nervenfasern doch eine sehr verschiedene. In den inneren Teilen der Medulla oblongata, im Grofs- und Kleinhirn etc. sieht man zwischen je zwei Nervenfasern anscheinend oft nur eine einzelne Neurogliafaser oder doch sehr spärlich nebeneinanderliegende. In anderen Fällen, z. B. in den nach der Aufsenperipherie zu liegenden Teilen der Medulla oblongata oder des Rückenmarks (besonders in seinem oberen Teile) sind zwischen je zwei Nervenfibrillen ganze Bündel von Neurogliafasern eingefügt. Das gleiche gilt für die letztgenannten Stellen in der Nähe derjenigen grauen Massen, welche ihrerseits sehr reichliche Neurogliafasern aufweisen, z. B. in der Nähe der Vorderhörner.

Überhaupt ist die Lage der weifsen Stränge von gröfstem Einflufs auf die Reichlichkeit ihrer Neuroglia. Namentlich da, wo weifse Massen dicht unter dem Ventrikelepithel oder auch nur in der Nähe des Ependyms verlaufen, zeigen sich oft ganz aufserordentlich dichte Neurogliamassen.

Solche Fälle sind z. B. die Striae acusticae, die direkt vom Epithel bedeckt sind (vgl. Taf. VII, Fig. 2), die Fasern der vorderen Rückenmarks-Commissur, die Marksubstanz des Kleinhirns und Grofshirns, da wo sie an das Ependym anstöfst etc.

Ebenso sind die weifsen Massen, die an eine äufsere Oberfläche resp. an die unmittelbar an dieser liegende Rindenschicht angrenzen, reicher an Neuroglia, als die davon entfernteren. Daher sind auch die äufseren Teile der weifsen Substanz des Rückenmarks etc. reicher an Neuroglia, als die in der Tiefe liegenden Teile. — Kielstreifen verhalten sich in ihrem Einflusse wie die entsprechenden Oberflächen, von denen sie ausgehen. — Über die Bedeutung der Bündelbildung ist S. 74 gesprochen worden. —

Die Richtung der Fasern in den weifsen Massen ist niemals eine ganz einheitliche, fast stets aber überwiegt die eine in ganz auffallender Weise. Im Grofs- und Kleinhirn ist das die Richtung der Nervenfasern, im Rückenmark die dazu quere Richtung. Bemerkenswert ist ferner auch hier ein Einflufs der äufseren und inneren Rindenschichten. Liegen in deren Nähe weifse Massen, so treten in diese aus der Rindenschicht sehr häufig reichliche radiäre, d. h. zur Oberfläche, event. zum Verlauf der Nervenfasern, senkrechte Faserzüge ein (vgl. Taf. XI Fig. 2). An den inneren Rindenschichten, d. h. an den Ependymmassen, könnte man dabei an den Einflufs von Ependymfasern denken, die ja so verlaufen müfsten. Da sich aber dieselbe Erscheinung auch an den äufseren Rindenschichten findet, so kann man eine solche Annahme nicht machen, sondern mufs an irgend welche noch

unbekannte, andere Ursachen, wohl mechanischer Art, denken (wie bei den extrinsic fibres der Gefäfse).

6. Für die grauen Substanzen, natürlich abgesehen von den Ependymmassen, lassen sich allgemeine Regeln nicht aufstellen, und es spricht für die Unzulänglichkeit der bisherigen Untersuchungsmethoden, dafs man die vielen Verschiedenheiten nicht oder nur unvollkommen finden konnte. So ist es garnicht richtig, dafs, wie Popoff (vgl. oben S. 27) angiebt (wenn das Referat korrekt gemacht ist) in den grauen Substanzen die Maschen der Neuroglia allgemein weiter wären, als in den weifsen. Es ist auch keine allgemeine Regel darüber aufzustellen, dafs in den grauen Substanzen die Neuroglia reichlicher, oder dafs sie spärlicher wäre, als in den weifsen: in manchen ist sie viel reichlicher, in anderen viel spärlicher. Auch der von Sala y Pons gemachte Versuch, diese Verschiedenheiten der Neurogliamengen in den grauen Substanzen zu erklären, ist nicht als gelungen zu betrachten. Sala y. Pons glaubt nämlich, dafs diejenigen grauen Massen, in denen reichliche markhaltige Nervenfasern verlaufen, reicher an Neuroglia sind, als die, in denen das nicht der Fall ist. Das ist aber nicht richtig, wie sich in der speziellen Topographie erweisen wird. Die Körnerschicht des Kleinhirns, die tiefsten Schichten der Grofshirnrinde sind, um nur diese Beispiele anzuführen, doch gewifs reich an markhaltigen Nervenfasern und doch sehr arm an Neuroglia.

In der grofsen Mannigfaltigkeit der Neurogliageflechte der grauen Substanzen lassen sich höchstens gewisse Typen aufstellen, die aber unter einander sehr abweichend sind: so der Typus der Stillingschen Nervenkerne, der der Grofshirnrinde etc. Doch ist es wohl noch verfrüht, diese Typen genauer zu spezialisieren.

7. Zwischen Neurogliafasern und nervösen Gebilden läfst sich niemals auch nur der geringste Uebergang nachweisen. Von nervösen Elementen sind in unseren Präparaten die Ganglienzellenkörper und deren gröbere Protoplasmafortsätze sowie die dickeren markhaltigen Nervenfasern deutlich zu erkennen u. zw. in der Kontrastfarbe tingiert. An diesen erkennbaren nervösen Elementen schneidet die Neuroglia stets scharf ab. Die von Rohde bei niederen Tieren[1] konstatierten intracellulären Neurogliaelemente der Ganglienzellen fehlen vollkommen beim Menschen. Die Neurogliafasern treten wohl oft dicht an den Körper der Ganglienzellen heran, ja bilden an manchen

---

[1] Ganglienzelle und Neuroglia. Archiv für mikroscopische Anatomie. Bd. 42. S. 423 ff.

Stellen die bereits erwähnten dichteren Geflechte, aber in den Körper oder in einen sichtbaren Fortsatz der Zelle hinein tritt niemals auch nur eine einzige Neurogliafaser.[1]

Was die markhaltigen Fasern betrifft, so hat Paladino (und ihm schliefst sich Colella an) die Behauptung aufgestellt, dafs auch innerhalb der Markscheide ein Neurogliagerüst nachzuweisen wäre. Auch davon ist in unseren Präparaten nicht die Spur zu bemerken. Welche Bedeutung freilich die von Paladino gefundenen Gerüstsubstanzen haben, ist eine andere Frage. Neuroglia in unserem Sinne sind sie aber jedenfalls nicht. Möglicherweise handelt es sich dabei, wie Kölliker meint, um Kunstprodukte, doch liegt die Entscheidung dieser Frage aufserhalb unserer Aufgabe. —

Bekanntlich hat ferner Golgi eine besondere, anderweitige Beziehung der Neuroglia zu den Nervenzellen angenommen. Nach seiner Meinung sollen die Protoplasmafortsätze sich mit der Neuroglia in Verbindung setzen. Die feineren Ausläufer der Dendriten sind zwar an unseren Präparaten nicht als solche zu erkennen. Man sieht nur die gröberen, in der Kontrastfarbe tingierten, während die feineren als die vielbesprochene „moleculare Masse“ erscheinen. Irgend welche „Übergänge“ von diesen Gebilden zu Neurogliafasern sieht man nie, in allen grauen Massen, in denen überhaupt Neurogliafasern zu erkennen sind, sind diese absolut scharf an den Seiten und an den Enden gegen die Umgebung abgesetzt. Aus unseren Präparaten kann man also nur schliefsen, dafs Dendriten dicht neben der Neuroglia liegen können. Das hat gewifs noch niemand bestritten. Eine innigere Verbindung im Sinne von Golgi läfst sich an unseren mit der neuen Färbung erhaltenen Präparaten nicht erkennen. Ob auf andere Weise eine solche Verbindung nachzuweisen ist, müssen wir aber natürlich dahingestellt sein lassen.

---

[1] Um Irrtümer zu vermeiden, sei speziell darauf hingewiesen, dafs Bilder in unseren Zeichnungen, wie in Taf. IX, Fig. 4, nicht etwa gegen die obige Anschauung zu verwerten sind. Auch an solchen Bildern kann man sich durch Drehung der Schraube am Mikroscop sehr leicht überzeugen, dafs die Neurogliafasern den Zellen nur aufliegen, nicht in sie hineingehen. In der Zeichnung konnte ich das nicht wiedergeben.

8. Abschnitt:

Spezielle Topographie der Neurogliafasern.

Vorbemerkung.

Die folgende Schilderung der speziellen Topographie der Neurogliafasern ist nur eine Skizze. Es wird noch eines sehr, sehr langen Studiums bedürfen, um diese Skizze zu vervollständigen. Für den Verfasser war, wie der Leser aus der Vorrede entnommen haben wird, die Zeit zu kurz bemessen, um mehr als das folgende zu geben.

Die reichen Verflechtungen der Neurogliafasern gewähren alle einen geradezu ästhetischen Anblick, „che l'occhio contempla sempre con sommo incanto", wie Petrone sich ausdrückt, und es hatte etwas für sich, wenn der verstorbene Hermann v. Meyer, dem ich die Präparate öfters zeigte, zu sagen pflegte: „Das sind sehr gefährliche Präparate. Man verliebt sich in die schönen Figuren und vergifst dabei, sie zu studieren." So schlimm ist es nun nicht — man studiert die Präparate doch, aber sehr schwer ist es, eine gute Beschreibung der Geflechte zu geben. Abbildungen geben ja noch die beste Vorstellung der mannigfaltigen Faserverschlingungen, aber die meinigen liefern doch nur eine schwache Vorstellung von der Wirklichkeit. Ich bin ein sehr ungeübter Zeichner und konnte daher nur Bilder wiedergeben, wie ich sie mit möglichst geringer Schraubenbenutzung sah. Wir sind aber gewöhnt, mit Hilfe der Schraube mehrere hinter einander liegende Ebnen des Präparats geistig zu einem gemeinschaftlichen Bilde zusammenzufassen. So hat man denn fast überall bei Betrachtung der Schnitte unter dem Mikroskop den Eindruck, dafs die Fasern viel reichlicher, als in unseren Zeichnungen, vorhanden sind.

I. Rückenmark.

A. Rindenschicht.

Von Alters her bekannt ist die das Rückenmark aufsen in wechselnder Breite umgebende „Rindenschicht". Diese ist zwar nicht so ganz frei von Nerven, wie man früher geglaubt hat, aber sie besteht doch zum überwiegenden Teile aus Neurogliafasern. Die

beste Beschreibung dieser Schicht hat Frommann gegeben, und wenn wir in dieser die Abweichungen der allgemeinen Anschauungen von unseren Auffassungen nicht berücksichtigen, resp. in Gedanken berichtigen, so können wir seine Schilderung ohne weiteres folgen lassen. Frommann sagt (I, S. 28):

„Die Rindenschicht besteht aus einem dichten, engmaschigen Netzwerk von Fasern und verästelten Zellen und bildet für die ganze Oberfläche des Marks einen abwechselnd dicken Überzug. Der Durchmesser der Rindenschicht schwankt zwischen 0,01 und 0,06 mm., meist beträgt er, wie auch Goll angiebt, 0,02—0,03 mm. Am dichtesten ist er in der Nachbarschaft der hinteren und der stärkeren der vorderen Wurzeln, am Eingange in die hintere Fissur und häufig in der Nähe der Stellen, wo eine Einziehung der Oberfläche sich findet, und die Rindenschicht in ihrer ganzen Dicke sich in die weifse Substanz einsenkt.“ „Die Maschen sind häufig zwischen den Fasern so schmal, dafs sie den Durchmesser der letzteren kaum übertreffen. Eine überall wiederkehrende Anordnung derselben zu bestimmten von einander geschiedenen Lagen konnte ich nicht wahrnehmen und nur im allgemeinen an den stärkeren zwei Hauptrichtungen, eine longitudinale und eine quere, verfolgen. Die gleich gerichteten Fasern kreuzen sich teils unter spitzen Winkeln, teils laufen sie parallel, und die zwischen ihnen bleibenden Lücken werden ausgefüllt von einem Netzwerk äufserst zarter Fasern.“ „Die stärkeren Fasern sind 0,001—0,002—0,003 [1] mm. dick, hell glänzend, von scharfem Contour und durch Karmin blafs rot gefärbt, die schwächeren sind um die Hälfte, oder den dritten Teil schwächer und erscheinen durch Karmin nicht gefärbt.“

Wir haben dem nur hinzuzufügen, dafs in unseren Präparaten alle Fasern, die schwächeren, wie die stärkeren, dunkelblau gefärbt erscheinen, so dafs man die Richtung der Fasern, so weit es das Gewirr derselben zuläfst, viel besser verfolgen kann, als dies Frommann an seinen Karminpräparaten zu thun vermochte. Die Hauptmasse der Fasern pflegt meist mehr oder weniger schief tangential zu verlaufen, es kommen aber noch aufser vertikalen auch radiäre Fasern vielfach vor, und da, wo stärkere Fortsätze der Rindenschicht in die Tiefe der weifsen Substanz eindringen, bilden sie oft nach innen konvergierende Büschel, die sich in bald zu erwähnender Weise weiterhin wieder auflösen. (Taf. I, Fig. 2 u. 3.)

Gegen die Pia mater zu ist normaler Weise die Rindenschicht scharf abgesetzt, doch kommt es oft genug vor (Taf. I, Fig. 3), dafs Faserbüschel wie die Haare einer

---

[1] So dicke Fasern, wie Frommann angiebt, habe ich unter normalen Verhältnissen nie gesehen. Vgl. S. 69. — Anmerkung des Verfassers.

Bürste über die sonst glatte Oberfläche der Rindenschicht heraus ragen, wie dies auch Frommann bemerkt hat. — In die stärkeren Nervenwurzeln giebt die Rindenschicht dicke, meist ziemlich parallel mit den Nervenfasern verlaufende Bündel ab, die dieselben aber nur eine kurze Strecke weit begleiten, — auch das hat Frommann bereits gesehen (I, S. 30). (Vergl. auch meine Mitteilung 1890.)

In die Rindenschicht findet man auch Kerne eingestreut, um die herum man aber kaum jemals spinnenförmige Faseranlagerungen aus dem dichten Netze herausheben kann. Meist sind die Kerne auch von jener kleineren, mit dichten Chromatinmassen versehenen Art, die auch sonst astrocytenartige Faserbildungen um sich herum nicht aufzuweisen pflegen. Es ist ferner bemerkenswert, worauf schon Golgi (Ges. Abh., S. 159) hingewiesen hat, dafs in der Rindenschicht die Kerne im Vergleich zu dem dichten Fasergewirr recht spärlich sind, — ein Beweis, dafs es sehr verfehlt wäre, aus der Anzahl der Kerne, d. h. der Zellen, einen Schlufs auf die Menge der Neurogliafasern zu machen.

B. Weisse Substanz.

Mit der Rindenschicht hängen Fasern und Faserzüge zusammen, die von jener auszustrahlen scheinen. Sie bilden bald dickere Massen, gewissermafsen eine direkte Fortsetzung der gesammten Rindenschicht in mehr oder weniger verjüngter Form, bald sind es nur einzelne Fasern und Fasergruppen, welche in das Innere hineinstrahlen (Taf. I, Fig. 2 u. 3). Die dichteren Neurogliamassen, die von der Rindenschicht her in die Tiefe dringen, hat Frommann als „Stammfortsätze“ bezeichnet. Sie umscheiden die von der Pia her die Rindenschicht durchsetzenden und in die weifse Substanz, hauptsächlich in halbwegs radiärer Richtung eindringenden Gefäfse. Aufser den meist geringfügigen adventitiellen Bindegewebsmassen um die Gefäfse herum (nur neben dem Centralkanal sind oft die Adventitien der, hier vertikalen, Gefäfse auffallend mächtig) dringt kein Bindegewebe in die weifse Substanz ein, wie schon Frommann wufste, und wie es jetzt wohl allgemein anerkannt ist. Die Gefäfse teilen die weifse Substanz in sehr unvollkommen geschiedene gröbere Bündel, die in ihrer Form etwa Kreissektoren entsprechen.

Ein ganz besonders grofses und langes Gefäfs pflegt im Sulcus longitudinalis posterior in das Rückenmark einzustrahlen, und mit diesem Gefäfs eine bindegewebige Adventitia. Diese Einstrahlung erfolgt in eng aufeinander liegenden Etagen immer wieder, und so kann es denn kommen, dafs man auf vielen Querschnitten vom Sulcus longitudinalis

posterior bis an die hintere Commissur reichend einen „Piafortsatz“ (Gefäfs mit Adventitia) zu sehen bekommt. Man glaubt dann eine typische, natürlich von reichlicher Neuroglia begrenzte Fissur vor sich zu haben. An anderen Stellen aber wird diese Fissur gewissermafsen lückenhaft. Das Gefäfs und seine bindegewebige Adventitia fehlt auf dem Querschnitt an verschiedenen Stellen. In der Mittellinie pflegt aber auch dann eine mehr oder weniger verdichtete Neurogliaschicht vorhanden zu sein, welche die Hinterstränge bilateral symmetrisch teilt.

Frommann schildert die Verhältnisse des „Septum posterius“ folgendermafsen (I, S. 31): „Die Dicke des Septum schwankt zwischen 0,004 bis 0,024 mm. Im Hals- und Lendenteil ist es breiter, als im Rückenteil, wo es oft nur ein paar Fäserchen enthält.“ „Hier und da fehlt es, obschon selten, stellenweise ganz, und die beiden Hinterstränge gehen ununterbrochen in einander über. Mitunter spaltet es sich in zwei Septa, welche sich wieder vereinigen.“ „In seinem hintern Teile ist es in der Regel breiter, als nach der Commissur zu, und erst kurz vor dem Übergange in letztere gewinnt es wieder an Breite.“

In neuerer Zeit hat besonders Lenhossék[1] sich mit den Verhältnissen des fälschlich sogenannten Septum posterius beschäftigt. Mit seinen Angaben mufs ich mich, wie das vorstehende zeigt, durchaus einverstanden erklären. Lenhossék sagt weiterhin (S. 222): „Diese Spaltbildung ist eine sekundäre Erscheinung, sie ist, wie ich glaube, überall an den Eintritt von Blutgefäfsen in der hinteren Mittellinie geknüpft, und wenn man auch auf dem Querschnitt kein Blutgefäfs findet, so erklärt sich das wohl daraus, dafs sich die Spalte in der Längsrichtung noch etwas über die Eintrittsstelle des Gefäfses ausdehnt.“

Über die gefässfreie Gliaverdichtung in der Mittellinie der Hinterstränge kann man aber doch verschiedener Meinung sein. Es könnte einmal so sein, wie sich das Lenhossék zu denken scheint, d. h. die Gefäfseinstrahlungen könnten so dicht aufeinander etagenweise folgen, dafs die gliösen Hüllen der Gefäfse in vertikaler Richtung immer miteinander verschmölzen. Es könnte ferner sein, dafs die Hinterstränge als zwei grofse „Bündel“ zu betrachten wären, die dann analog anderen solchen strangförmig zusammengefafsten Massen eine Randschicht zwischen sich hätten (vgl. S. 74 suba).

[1] Auch Schaffer in Wien (Archiv für mikroscopische Anatomie, Band 44) hat über die Rinden schicht und die Stammfortsätze geschrieben, ohne aber etwas wesentlich neues an Thatsachen vorzubringen.

Es scheint mir aber am wahrscheinlichsten, dafs wir es hier mit einer „Kielstreifenbildung“ zu thun haben. Das Rückenmark stellt ja in der frühesten Embryonalperiode eine flächenhaft ausgebreitete Gewebsmasse dar, die sich dadurch zu einer Röhre schliefst, das die beiden Seitenteile dorsal (hinten) zusammenwachsen. Man könnte sich daher sehr wohl denken, dafs diese Nahtstelle in der Mittellinie des ausgebildeten Rückenmarks sich noch als Kielstreifen (S. 73 f.) kenntlich macht.

Abgesehen von den dichteren Neurogliamassen, welche die einstrahlenden Gefäfse begleiten, ist nun die weifse Substanz von einem lockeren Gerüst von Neuroglia durchsetzt, welche, dem allgemeinen topographischen Gesetze entsprechend, zwischen jede einzelne Nervenfaser und ihre Nachbarfaser eindringt. So sind denn alle einzelnen Nervenfasern durch Neurogliafasern von einander geschieden.

Was die Richtung dieser Fasern anbelangt, so hat man, wenn man die Fasern auf dem Querschnitt eines Rückenmarks betrachtet, zunächst ganz den Eindruck, als wenn, wenigstens in den Vorder- und Seitensträngen, fast nur ziemlich horizontal verlaufende Fasern als Gerüst vorhanden wären. Es sind aber auch vertikale resp. schiefe Fasern da, die nur, weil sie spärlicher sind und als Punkte resp. kurze Abschnitte erscheinen, nicht so ins Auge fallen. Auf Längsschnitten überzeugt man sich besser (Taf. I, Fig 3), dafs auch solche Fasern zugegen sind. In den Hintersträngen kommen, wenigstens bei älteren Leuten, auch auf dem Querschnitt die nicht horizontalen Fasern reichlicher und demnach deutlicher zu Gesichte. Charakteristisch ist es, dafs unter pathologischen Verhältnissen gerade die vertikalen Fasern ungemein überwiegen. Bei kleinen Kindern hingegen ist das Netz der Neurogliafasern in der weifsen Substanz ein ungemein regelmäfsiges radiäres System mit sehr wenig anders gerichteten Fasern. Das Bild erinnert dann ganz auffallend an das primäre Neurogliagerüst, welches die Ependymfasern im Embryo bilden.

Bei Erwachsenen hört diese Regelmäfsigkeit auf, d. h. zu den radiären Horizontalfasern gesellen sich hier viele in mehr oder weniger schiefer Richtung zu diesen verlaufende, aber ebenfalls ziemlich horizontale Fasern, ganz abgesehen von den schon erwähnten Vertikalfasern (vgl. Taf. I, Fig. 2). In den der Rindenschicht nahe gelegenen Teilen und denen in der Nähe der Vorderhörner sind die zwischen den Nervenfasern liegenden Neurogliamassen reichlicher, als in den dazwischen liegenden Partien (S. 78). —

Eine besondere Untersuchung verdient noch das Gebiet der Hinterstränge. Es ist nämlich auffallend, wie ungemein häufig bei Erwachsenen in diesen, besonders im Halsmark, (aber auch in den anderen Abteilungen des Rückenmarkes) nicht nur, wie wir oben hervorgehoben, Vertikalfasern überhaupt vorkommen, sondern stärkere, gruppenweise liegende Anhäufungen senkrechter Fasern sich finden, so dafs man degenerative Prozesse vor sich zu haben meint. Am reichlichsten pflegen diese Heerde dichter Neurogliamassen in den Gollschen Strängen zu sein. Bei kleinen Kindern fehlen sie. Ob das nun normale Verhältnisse bei Erwachsenen sind, ist mir nicht ganz sicher. Lichtheim hat zuerst darauf hingewiesen, dafs bei perniciöser Anämie Neurogliawucherungen in den Hintersträngen zu beobachten sind. Es wäre daher sehr leicht möglich, dafs auch bei anderen langdauernden Krankheiten, Phthisen, Nephritiden, Carcinosen etc. derartige „Neurogliawucherungen“ aufträten, die nur mit den Methoden, die Lichtheim noch brauchen mufste, nicht nachzuweisen waren. Es könnte aber auch sein, dafs die geringeren Grade dieser „Neurogliawucherungen“ etwas ganz normales wären, was nur bisher nicht zu konstatieren war. Leider war es mir nicht möglich, in der letzten Zeit Rückenmarke in genügender Frische von plötzlich gestorbenen Leuten zu bekommen, so dafs ich diese Frage noch offen lassen mufs.

Zum Schlufs sei noch darauf hingewiesen, dafs die vorderen Wurzeln, die ja als gesonderte Bündel eine Strecke weit in die weifse Substanz einstrahlen, diesem Bündelcharakter entsprechend eine, wenn auch zarte Randschicht besitzen (Taf. I, Fig, 4a).

Die Neurogliakerne in der eigentlichen weifsen Substanz sind z. T. grofse bläschenförmige Gebilde mit körnig erscheinendem Chromatin, z. T. die kleineren kompakten Kerne. „Astrocyten“ sieht man auf Längsschnitten mehr, als auf Querschnitten, aber nicht so reichlich, wie an anderen Stellen des Centralnervensystems.

C. Graue Substanz.

Während die Verhältnisse der Neuroglia in der weifsen Substanz so leicht zu erkennen sind, dafs sie eigentlich schon Frommann im ganzen richtig geschildert hat, liegt die Sache bei der grauen Substanz ganz anders. Mit Ausnahme der Gegend des Centralkanals und der Spitze des Hinterhorns, von denen ebenfalls Frommann eine ziemlich gute Schilderung gegeben hat, die von keiner neueren übertroffen wurde, sind die topographischen Verhältnisse in der grauen Substanz ganz mangelhaft, zum grofsen Teil geradezu falsch dargestellt worden. Auch in den Arbeiten, welche mit der Golgischen Methode gemacht

wurden, sind nicht nur die alten Irrtümer beibehalten, sondern auch diesen noch neue hinzugefügt worden. Nur Lenhossék hat in der neuen Auflage seines Lehrbuches die von mir schon 1890 mitgeteilten Anschauungen bestätigt.

Ganz allgemein, aber auch ganz fundamental ist der Irrtum, der durch die Golgische Methode hervorgerufen resp. bestätigt wurde, dafs die topographischen Verhältnisse der Neuroglia in der grauen Substanz ganz gleichmäfsige wären, und dafs in der grauen Substanz weniger Neuroglia vorhanden sei, als in der weifsen. Beides ist falsch. Wir müssen vielmehr die einzelnen Abschnitte der grauen Substanz gesondert betrachten, da in jedem einzelnen andere Neurogliageflechte vorliegen, und dabei wird es sich zeigen, dafs in den meisten Abteilungen die Neuroglia reichlicher ist, als in der weifsen Substanz. Gerade für die grauen Substanzen ist es aber sehr schwer, den eigenartigen Charakter der Neurogliafaserung in Worten zu schildern, und auch die von uns beigefügten Tafeln geben von der Reichlichkeit und Eleganz der Netze nur eine mangelhafte Vorstellung.

a) Vorderhorn.

Das Neurogliageflecht des Vorderhorns hat bei Neugeborenen ein viel regelmäfsigeres Gepräge, als bei Erwachsenen. Die Fasern verlaufen hauptsächlich horizontal und bilden fächerförmige Bündel, deren Spitzen in die Ausläufer der Vorderhörner hineinstrahlen, während der breite Teil des Fächers nach innen zu gekehrt ist. Das Bild wird noch dadurch besonders elegant, dafs sich die Basen der Pyramiden vielfach decken (Taf. II, Fig. 1). Bei Erwachsenen findet man noch Andeutungen dieses Verhaltens an den Spitzen der Ausläufer des Vorderhorns. Schon ganz in der Nähe derselben aber und im ganzen inneren Teile ist von solch regelmäfsigen Zügen nichts mehr zu sehen (Taf. II, Fig. 3). Vielmehr ist hier die ganze Substanz von reichlichen Fasernetzen durchzogen, welche in so verschiedener Richtung laufen, dafs Quer- und Längsschnitte des Vorderhorns kaum Unterschiede erkennen lassen, wenn man von den Eintrittsstellen der Wurzeln absieht. — Die hier verlaufenden Gefäfse entsprechen dem allgemein topographischen Gesetze und zeigen eine Verdichtung der Neuroglia, — ihrer geringeren Gröfse entsprechend aber nicht in solcher Mächtigkeit, wie die Gefäfse der „Stammfortsätze“. Auch über das Verhalten der grofsen motorischen Ganglienzellen ist bereits in der allgemein-topographischen Übersicht gesprochen worden. Die leichten Verdichtungen (Taf. II, Fig. 3) setzen sich auch auf die dickeren Fortsätze der Ganglienzellen in Form von Begleitfasern fort, deren Verlauf der Richtung

der Fortsätze im allgemeinen parallel ist. (Taf. II, Fig. 3 rechts. Hier sind die Begleitfasern senkrecht durchschnitten, daher als Punkte erscheinend.)

Die Masse der Neurogliafasern des Vorderhorns ist recht grofs, gröfser als in der eigentlichen weifsen Substanz (also abgesehen von der Rindenschicht und den Stammfortsätzen). Namentlich grofs ist sie an den vorderen und seitlichen Rändern, die man oft schon mit dem blofsen Auge als etwas dunklere, schleierartig aussehende Massen hervorgehoben findet. Andererseits ist die Dichtigkeit des Neurogliageflechts auch nicht entfernt mit der in der Substantia grisea centralis oder der an der Spitze des Hinterhorns zu vergleichen. Neurogliakerne findet man zwischen die Fasern eingestreut, teils mit, meist aber ohne Strahlenkranz von Fasern.

Die Fasern unterscheiden sich im übrigen in keiner Weise von denen der weifsen Substanz. Wenn daher in den mit der Golgischen Methode ausgeführten Arbeiten immer davon die Rede ist, dafs im Vorderhorn besonders viel „Kurzstrahler" wären, die sich von den Langstrahlern, d. h. den echten Deitersschen Zellen unterscheiden sollen, so finde ich in den Vorderhörnern absolut nichts, was auf die Anwesenheit anderweitiger Neurogliaelemente, als der typischen (Langstrahler-) Fasern hindeutete. Ja, wenn man die Reichlichkeit dieser Fasern einerseits, das sehr entwickelte nervöse Material der Vorderhörner andererseits in Betracht zieht, so begreift man nicht recht, wie hier noch ein zweites, bei unserer Methode nicht nachweisbares, Neurogliageflecht von andersgearteten „Ausläufern" Platz haben soll. Wir haben freilich oben (S. 73) gesehen, dafs dieses „Nichtplatzhaben" etwas sehr zweifelhaftes ist, aber hier liegt die Sache doch wesentlich anders.

Man mufs eben bedenken, dafs nach Angabe der Autoren diese „Kurzstrahler" an Zahl mindestens so reichlich sein sollen, wie die Langstrahler, und dafs die Zahl der Ausläufer an den ersteren aufserdem noch viel bedeutender sein soll, als an den letzteren.

---

b) Hinterhorn.

α. Die Spitze des Hinterhorns, die Lissauersche Randzone, ist in ihren Neurogliaverhältnissen von Frommann bereits beschrieben worden, doch klagt er gerade für diese Stelle mit Recht darüber, dafs die von ihm benutzte Carminfärbung sehr unsichere Resultate liefert, weil sie ja eine Unterscheidung der Neurogliafasern von den hier speziell sehr zahlreichen Axencylindern nicht gestattet. Bei der Golgischen Methode ist das erst recht der Fall. Die Lissauersche Zone ist bei unserer Färbung mit einem ungemein dichten Neurogliageflecht versehen, das freilich doch nicht so eng gewebt ist, wie das der

Substantia grisea centralis im Rückenmark. Die Fasern verlaufen teils in horizontalen Verflechtungen (Taf. II, Fig. 4b), teils vertikal, manchmal in letzterer Richtung überwiegend. —

β. Substantia spongiosa. Ziemlich scharf setzt sich die Lissauersche Randzone nach vorn zu gegen die Substantia spongiosa (Taf. II, Fig. 4a) ab. In dieser ist das Neurogliageflecht lange nicht so dicht. Dieses lockere Neurogliageflecht ist bald breiter, bald schmaler, bald länger, bald kürzer, manchmal nur angedeutet, wie das dem aufserordentlich wechselnden Verhalten der Substantia spongiosa nach Form und Ausdehnung entspricht.[1] Das gleiche gilt für die Faserrichtung. Wohl stets finden sich radiäre Bündel, aber diese brauchen nicht ausschliefslich vorhanden zu sein, sondern sie können Maschenräume mit andersgerichteten Fasern umschliefsen (Taf. II, Fig. 4a). Weiter nach vorn aber, nach der Substantia gelatinosa zu, treten gewöhnlich die radiären Fasern als Hauptmasse (neben spärlich anders verlaufenden) auf (Taf. II, Fig. 3). Diese setzen sich dann, öfters mit einer geringfügigen Verdichtung ziemlich scharf gegen die folgende Zone des Hinterhorns, die Substantia gelatinosa Rolando, ab.

γ. Substantia gelatinosa Rolando. Alle bis zum Jahre 1890 erfolgten Beschreibungen des Neurogliagerüsts in der Substantia gelatinosa Rolando kommen darin überein, dafs hier ein sehr reiches Neuroglianetz vorliege, ja die meisten Autoren behaupteten, dafs die genannte Substanz so ziemlich reine Neuroglia darstellte, wenn man von den durchziehenden wenigen Nervenfasern absah und von den Ganglienzellen, die sich hier vorfanden. Im Jahre 1890 habe ich zum ersten Male die Behauptung aufgestellt, dafs gerade umgekehrt, wie man bisher angenommen hat, die Substantia gelatinosa Rolando aufserordentlich arm an Neurogliafasern ist, so arm, dafs kein einziger Teil des Rückenmarks mit ihr in dieser Beziehung verglichen werden kann. Es ist mir eine ganz besondere Freude, dafs Lenhossék im Gegensatz freilich zu allen anderen, die mit der Golgischen Methode gearbeitet haben, in der zweiten Auflage seines Buches sich meiner Ansicht durchaus angeschlossen hat.

Freilich fehlen hier die Fasern nicht, sie sind nur spärlich. Die Fasern verlaufen hauptsächlich radiär, doch finden sich überall auch in anderer Richtung verlaufende Fäserchen. Zwischen den Fasern bleiben aber verhältnismäfsig grofse leere Stellen, die für diese Substanz ganz charakteristisch sind. Die radiären Fasern sind z. T. Fortsetzungen

---

[1] Lissauer, Beitrag zum Faserverlauf im Hinterhorn des menschlichen Rückenmarks und zum Verhalten desselben bei Tabes dorsalis. Arch. für Psych. 17. Bd. Heft 2. S. 12 des Sep.-Abdr.

der ebenso gerichteten Fasern aus der Substantia spongiosa, deren oben erwähnte ziemlich scharfe Absetzung gegen die Substantia gelatinosa Rolando in der Weise erfolgt, dafs die Fasern schnell spärlicher werden und schliefslich aus dem gröfsten Teile des Areals verschwinden. Weiter nach vorn zu geht die an Neuroglia so arme Zone ganz allmählich in eine viel dichter gewebte Neurogliamasse über, welche den Übergang zu den Clarkeschen Säulen resp. zum Vorderhorn und zur Substantia grisea centralis bildet.

δ. Die Clarkeschen Säulen (Taf. III, Fig. 1). Die Clarkeschen Säulen enthalten ein in verschiedenen Richtungen verlaufendes Maschenwerk von Neurogliafasern, die aber, wenigstens in den hinteren Teilen, etwas spärlicher sind, als in den Vorderhörnern, aber doch bei weitem reichlicher, als in der Rolandoschen Substanz, wie ich schon 1890 mitgeteilt habe.

c) Die Gegend des Centralkanals.

α. Substantia grisea centralis. Schon in der allgemein-topographischen Übersicht haben wir darauf hingewiesen, dafs die Umgebung des Centralkanals im Rückenmarke, wie alle ependymären Schichten, ungemein reich an Neuroglia ist. Der Reichtum an Neurogliafasern gerade dieser Gegend ist so kolossal, dafs an jedem nach unserer Methode gefärbten Querschnitte des Rückenmarks die Umgebung des Centralkanals als dunkelblauer Fleck schon für das blofse Auge kenntlich ist.

Dieser Reichtum an Neurogliafasern betrifft die ganze Umgebung des Centralkanals. Es besteht weder eine zwischen vordere und hintere Commissur eingeschobene, scharf abgesetzte „Ringcommissur“, wie die älteren Forscher annahmen, noch ist es allein die hintere Commissur (Taf. III, Fig. 3, vom Kinde), welche diesen Faserreichtum zeigt, wenn auch natürlich innerhalb der dichten Neurogliamassen in der vorderen Commissur die Räume für die groben markhaltigen Nervenfasern ausgespart sind (Taf. III, Fig. 2, vom Kinde). Aber zwischen diesen einzelnen Nervenfasern liegt ein ebenso dichtes Neuroglianetz, wie sonst auch um den Centralkanal, also ein Netz, das in seiner Dichtigkeit garnicht mit dem der sonstigen weifsen Substanzen zu vergleichen ist. An den Seiten geht die mächtige centrale Gliaanhäufung ganz allmählich in die weniger dichte der Vorderhörner über, so allmählich, dafs sich der gröfsere Faserreichtum noch weithin seitlich zu erkennen giebt. Nach hinten zu ist die Absetzung gegen die dorsalen Stränge im Gegensatz dazu eine recht scharfe.

Bei neugeborenen Kindern überwiegen in dieser Fasermasse horizontale sich schief durchkreuzende Fasern, doch sind sie nicht ausschliefslich vorhanden (Taf. III., Fig. 3).

Bei älteren Individuen treten immer mehr und mehr vertikale Fasern auf. Wenn auch immer noch anders gerichtete dazwischen zu sehen sind, so wird jedenfalls das Querschnittsbild immer mehr von den (punkt- und strichförmig erscheinenden) vertikalen Fasern beherrscht.

Dieser aufserordentliche, von mir bereits 1890 geschilderte Neuroglia-Reichtum der Substantia grisea centralis ist in neuester Zeit ebenso bestritten worden, wie meine Angaben über die verschiedene Reichlichkeit der Neuroglia in den verschiedenen Partieen der grauen Substanz überhaupt, und zwar von keinem geringeren, als von Kölliker, auf Grund seiner Erfahrungen an Golgipräparaten. Er sagt nämlich (Bd. 2, S. 153),[1] dafs die Menge der Neurogliazellen in allen Teilen der grauen Substanz ziemlich gleich sei, in der Substantia gelatinosa centralis ebenso gut, als in den ventralen und dorsalen Säulen und in der grauen Commissur, und fährt dann fort: „Ich betone das absichtlich, weil Weigert aus seinen neuen Färbungen der Gliafasern andere Schlüsse zieht. Er fand blau sich färbende Fasern in ungemeiner Menge in der Substantia gelatinosa centralis. . . . . Ich erkläre mir dieses Ergebnis daraus, dafs in der grauen Commissur nicht nur Fortsätze der Golgischen Zellen, sondern auch die sehr zahlreichen Ausläufer der Ependymzellen mitgefärbt werden."

Darauf habe ich folgendes zu erwidern: Bei meinen früheren und jetzigen Angaben handelt es sich einzig und allein um die Neurogliafasern. Über deren reichliche oder nicht reichliche Anwesenheit kann man aber nach der Golgischen Methode garnicht sicher urteilen. Nicht nur, dafs diese überhaupt nur die Zellen und die mit ihnen verbundenen Faserstümpfe, also nur einen kleinen Teil der Fasern überhaupt, zu diagnosticieren gestattet, färbt sie auch diese „Astrocyten" in so wechselnder Menge, je nach ihrer unberechenbaren Laune, dafs man aus einer geringen Menge der nachgewiesenen Astrocyten nicht auf eine geringe Menge der vorhandenen schliefsen kann. Ferner steht, wie wir schon oben nach einer Bemerkung von Golgi konstatiert haben, die Menge der Zellen durchaus nicht in einem konstanten geraden Verhältnis zur Menge der Fasern. Auch an unseren Präparaten kann man das erkennen. Man sieht in denselben zwar nur die Kerne der Zellen, aber da ja jeder Kern einer Zelle entspricht, so zeigt die Menge der Kerne die Menge der Zellen direkt an. Da kann man denn sehen, dafs die Menge der Fasern in gar keinem konstanten Verhältnis zur Menge der Zellen steht.

[1] Handbuch der Gewebelehre des Menschen. 6. Auflage. 1893.

Was ferner die Annahme Köllikers betrifft, dafs an dem Fasergewirr der Substantia grisea centralis (wohlgemerkt nicht blofs der grauen Commissur, wie Kölliker erwähnt) auch Ependymfasern Teil nehmen können, so ist dagegen von unserem Standpunkt aus a priori nicht das geringste einzuwenden. Man müfste da nur mehrere Voraussetzungen machen: einmal die, dafs die Ependymfasern selbst im spätesten Alter beim Menschen nicht verkümmern, wie öfters angenommen wird, denn gerade in der frühesten Kindheit sind um den Centralkanal lange nicht so viel Fasern da, wie im höheren und höchsten Alter.

Sodann müfste man voraussetzen, dafs wenigstens im höheren Alter des Menschen die Bildung der Neurogliafasern genau mit derselben Differenzierung und Emanzipation vom Zellleibe einhergeht, wie bei den eigentlichen Neurogliazellen, denn im höheren Alter sieht man die abgestofsenen Epithelzellen ganz frei zwischen den neugebildeten Neurogliafasern darin liegen, ohne organische Verbindung (vgl. sub cβ, S. 94 f.).

Endlich müfste man annehmen, dafs die Ependymfasern nicht nur in ihrer Entstehung, sondern auch als fertige Fasern in ihrem ganzen Verhalten, in ihrem Aussehen, ihrem Verlauf und ihrer Färbbarkeit ganz mit den echten Neurogliafasern übereinstimmen: mit einem Worte, man müfste annehmen, dafs Ependymfasern und Astrocytenfasern (Neurogliafasern) in jeder Beziehung identisch wären. An unserer Darstellung und Auffassung würde demnach selbst unter Annahme der Köllikerschen Vermutung auch nicht das allergeringste zu ändern sein.

Freilich gestattet unsere Methode nicht, die Beteiligung der Ependymfasern an der Bildung des Neurogliageflechts beim Menschen zu eruieren, wir müssen daher den positiven Nachweis einer Beteiligung der Epithelzellen an der Erzeugung von Neurogliafasern anderen Autoren überlassen, aber eins können wir sicher sagen: wenn die Epithelzellen an der Faserbildung um den Centralkanal einen Anteil haben, so erzeugen sie typische Neurogliafasern.

Die Unkenntnis der Gliaverdichtung um den Centralkanal herum hat in der pathologischen Anatomie des Rückenmarks grofse Verwirrung angerichtet. Sie hat zu der Fabel von der „erweichten centralen Gliose“ in der Lehre von der Syringomyelie geführt. Die Verwirrung wurde noch dadurch vergröfsert, dafs man „Gliose“, d. h. krank-

hafte Vermehrung der Neurogliafasern mit „Gliom“ verwechselte. Bei den Gliomen sind die Gliafasern nicht vermehrt, sondern die Gliazellen. Ja nicht nur das, sondern diese letzteren verlieren zum grofsen Teile die Fähigkeit, abgesetzte Fasern zu erzeugen und bleiben in ihrem ursprünglichen protoplasmatischen Zustande. Man darf sich daher nicht wundern, wenn man gerade in Gliomen echte Deiterssche Zellen findet, wie im Embryo. Das Verhältnis der Gliome zur Gliose ist also, wie das des Sarkoms zur entzündlichen Bindegewebswucherung, oder wie zum Fibrom. Bei der Lehre von der „erweichten centralen Gliose“ (fälschlich „erweichtes centrales Gliom“ genannt) soll es sich nun um eine Vermehrung von typischer faseriger Neuroglia mit Erweichung handeln. Aber die Neuroglia ist normalerweise um den Centralkanal sehr vermehrt, und der normalen Massenhaftigkeit gegenüber kann sie sogar (in manchen Fällen wenigstens) bei Syringomyelie resp. Hydromyelie vermindert sein. Umgekehrt findet man in der That garnicht selten wirklich krankhafte über die Grenzen des centralen Ependymfadens hinausgehende „Gliosen“ u. zw. bei der multiplen Sklerose. Diese erweichen aber nie, wie es überhaupt noch niemals nachgewiesen ist, dafs echte Gliosen erweichen — mit einem Worte die Auffassung der Syringomyelie als erweichte centrale Gliose hat nicht den Schatten einer Wahrscheinlichkeit für sich. Wieso es eventuell sekundär zu einer Vermehrung der Glia bei der Syringomyelie kommen kann, das habe ich an einem anderen Orte bereits kurz besprochen.[1]

β. Centralkanal. Bei jugendlichen Individuen liegt das Epithel glatt auf der dichten Neurogliamasse. Die Fortsätze der Epithelzellen in diese Masse hinein sind bei unserer Methode nicht zu erkennen.

Die Epithelien selbst liegen als gleichmäfsige, durch nichts unterbrochene Reihe mit ihren grofsen Kernen und ihrem bei unserer Methode gelblich gefärbten Protoplasma da. (Vgl. Taf. III, Fig. 2 und 3. Die Kerne sind in diesen Figuren nicht mitgezeichnet.) An ihrer Innenwand sieht man auf jeder Epithelzelle Gruppen kleiner, blau gefärbter Körnchen, die von mir zuerst gesehen und schon 1890 beschrieben wurden. Auch die Existenz dieser Körnchen ist nunmehr von Lenhossék bestätigt worden. Ich machte damals die Bemerkung, dafs es sich hier um cuticulare Abscheidungen handeln dürfte, dafs man es namentlich nicht

[1] Zur pathologischen Histologie des Neurogliafasergerüsts. Centralblatt für allg. Path. und path. Anat. 1890. S. 736 f.

mit deformierten Flimmerhaaren zu thun habe. Diese Vermutung kann ich jetzt auf das bestimmteste beweisen.

Bei einem Embryo von 15 cm Scheitel-Steifslänge fanden sich im dritten Ventrikel die Flimmerhaare wundervoll erhalten. Sie safsen auf jeder Zelle in mehrfacher Anzahl auf, und mehrere der Flimmerhaare waren immer zu einer Pyramide mit nach innen gekehrter Spitze verbunden (wie eine Gewehrpyramide aussehend), doch so, dafs man jedes einzelne Flimmerhaar genau von dem benachbarten abgrenzen konnte. Da die Existenz des Flimmerbesatzes am (embryonalen) Ependymepithel immer noch Zweifeln begegnet, so habe ich das Präparat abgezeichnet (Taf. IV, Fig. 1). Unter diesem Flimmerbesatz fanden sich nun die Körnchen gefärbt. Sonst war von Neurogliafärbung nicht viel zu sehen, im Rückenmark waren nur im peripherischen Teile radiäre Faserabschnitte tingiert.

An Neugeborenen habe ich keine deutlichen Flimmerhaare mehr wahrgenommen, bei älteren Kindern etc. natürlich erst recht nicht, aber sonst bleibt das Epithel zunächst noch einige Jahrzehnte ganz intakt, das Lumen des Centralkanals weit. Aber allmählich ändert sich das Bild, obgleich sich nicht genau angeben läfst, von welchem Alter ab, — die Veränderung mag wohl, wie so viele Alterserscheinungen, bald früher, bald später eintreten.

Als den geringsten Grad der Veränderungen, die im Fortschreiten des menschlichen Lebens am Centralkanal erfolgen, kann man den bezeichnen, dafs die Epithelzellen stellenweise etwas von einander weichen, und dafs in die so entstehenden Zwischenräume zwischen die einzelnen Epithelzellen vereinzelte Neurogliafasern meist von radiärer Richtung eingelagert sind. Bei höheren Graden der Veränderung lösen sich an einigen Stellen die Epithelzellen nicht nur von einander, sondern auch von ihrer Unterlage ab, und so werden denn breitere oder schmälere Räume von Epithel entblöfst. In diese Räume dringen nun förmliche Büschel ziemlich paralleler Neurogliafasern herein, die direkt mit denen der Substantia grisea centralis in Verbindung stehen. Die abgelösten Epithelzellen gehen aber nicht verloren, sondern liegen unregelmäfsig zerstreut in den Neurogliamassen darin. Einen solchen Fall hat schon Frommann abgebildet.

Bei weiterem Fortschreiten des Prozesses sind verschiedene Fälle möglich:

1. Aus den abgestofsenen Epithelien bilden sich ein oder mehrere unregelmäfsig durcheinander geworfene Haufen, die mit den gewucherten Neurogliamassen den nunmehr lumenlosen Centralkanal einnehmen. (Taf. IV, Fig. 4.)
2. Ein Teil der abgelösten Epithelzellen ist zur Bildung eines richtigen, am Innenrande punktierten, einfachen Lumenringes zusammengetreten. Bei oberflächlicher

Ansicht scheint ein solcher Centralkanal ganz normal zu sein (Taf. IV, Fig. 3 u. 5), aber das hier vorhandene Lumen ist wesentlich kleiner, als ein entsprechendes bei einem jugendlichen Individuum, ganz abgesehen davon, dafs sich hier stets die sub 4 zu erwähnenden anderweitigen Veränderungen finden.

3. Eine dritte Möglichkeit ist die, dafs nicht ein einfaches Lumen entsteht, sondern zwei oder mehrere entsprechend kleinere Lumina, die sämtlich von einem (am Innenrande punktierten) Epithelkranze umgeben sind (Taf. IV Fig. 2). Alle die kleinen Lumina sind durch mächtige Neurogliamassen geschieden.

Das sub 2 und 3 geschilderte Zusammenhalten der Epithelien zu lumenumkränzenden Reihen könnte einmal auf einem Zusammenbleiben derselben beruhen, d. h. die Epithelzellen könnten als zusammenhängende Fetzen abgestofsen werden, die sich nur mit ihren Enden zusammenzuschliefsen brauchten, um ein Lumen oder mehrere Lumina zu erzeugen. Der Vorgang könnte aber auch so gedeutet werden, dafs die Epithelien zwar einzeln abgestofsen werden, aber, wenn der nötige Platz dazu da ist, sich gewissermafsen biotactisch wieder aneinanderlegen, wie dies Roux für die künstlich getrennten ersten Embryonalzellen gezeigt hat.

4. Neben allen diesen Abarten der Epithelzusammenlagerung finden sich immer auch mehr vereinzelte Epithelzellen mitten in der gewucherten Neurogliamasse darin. Bald liegen sie ganz einzeln und sind bei unserer Methode nur dann einigermafsen sicher zu erkennen, wenn ihr Protoplasmaleib grofs ist und gelblich gefärbt erscheint (Taf. IV, Fig. 2, 3 u. 4). In manchen Fällen sieht man auch Reste der Punktierung (von der Fläche eventuell). Andere Male liegen sie in Häufchen oder in Reihen (Taf. IV, Fig. 2). Diese schliefsen sich öfters kielstreifenartig an die Enden der (centralen) gröfseren Haufen oder der (sekundären) Lumina an, liegen aber von ihnen durch Neurogliafaserzüge getrennt, oder werden wenigstens von solchen allseitig umsponnen und durchsetzt (Taf. IV, Fig. 3). In anderen Fällen liegen sie den gröfseren centralen Ansammlungen mehr parallel, so dafs gewissermafsen concentrische Epithellager gebildet werden.

Es mufs jedoch besonders darauf aufmerksam gemacht werden, dafs es, wenn nicht die oben erwähnten Kennzeichen vorliegen, bei unserer Färbung oft schwer ist, vereinzelt liegende, so zu sagen atrophische Epithelzellen von Gliazellen zu unterscheiden. (Vgl. Tafel IV, Fig. 3 u. 4.)

Nach alledem fassen wir den primären Vorgang bei der sogenannten Obliteration des Centralkanals als einen passiven auf, in einer Lockerung und späteren Abstofsung der Epithelien bestehend, nicht, wie Brissaud[1] als eine primäre Wucherung der letzteren. Für unsere Ansicht spricht schon das, dafs diese Veränderungen gerade im vorgeschritteneren Alter auftreten, wo die idioplastische Kraft der Zellen überhaupt abnimmt, jedenfalls nicht so zunimmt, dafs sie activ zu gröfseren Leistungen, d. h. zu Wucherungen geneigt sein sollten. Dafür spricht ferner der Umstand, dafs man schon in verhältnismäfsig normalen Centralkanälen das Hineinwachsen der Gliafasern in die Zwischenräume zwischen die, doch also auseinanderweichenden, Epithelzellen sieht. Weiter spricht dafür der Umstand, dafs mit dieser Veränderung stets eine Verkleinerung des Raumes verbunden ist, der dem Centralkanal zukommt, nicht eine Vergröfserung, wie es bei activer Wucherung sein müfste.

Dafs neben diesen passiven Vorgängen und in Folge derselben auch active Prozesse einhergehen, beruht auf dem von mir schon so oft seit mehr als zwanzig Jahren entwickelten biologischen Prinzip, dafs nach Aufhebung des Gewebswiderstandes (durch passive Momente) Wucherungsvorgänge eintreten. Diese Aufhebung des Gewebswiderstandes wird hier durch die Loslösung der Epithelien (und die Resorption der Spinalflüssigkeit?) bedingt. Die Wucherungsprozesse bestehen einmal sicher in einer Wucherung der Neurogliafasern über ihre sonst durch die Epithelien gebildete Schranke hinaus, möglicherweise auch in einer sekundären Wucherung der losgelösten, von ihrem gegenseitigen Gewebsdruck (und dem Druck der Spinalflüssigkeit?) befreiten Epithelzellen.

Ob sich an der Neubildung der Neurogliafasern nur die typischen Neurogliazellen oder auch die Epithelien des Centralkanals beteiligen, das müssen wir, nach dem, was wir S. 92 gesagt haben, als offene Frage behandeln.

Wenn auch das Einstrahlen der Neuroglia in den Raum des ursprünglichen Centralkanals erst von Frommann (und zwar bis jetzt von ihm ganz allein!) beobachtet wurde, so ist doch die Thatsache der „Obliteration“ des Centralkanals, wie man alle die Vorgänge zusammen genannt hat, längst bekannt. Speziell die Zersprengung der Epithelmasse auf der einen Seite und die Bildung mehrerer Lumina auf der anderen Seite hat

[1] Revue neurologique. Bd. 2. S. 545 ff.

schon Clarke 1859 sehr gut geschildert. Er sagt:[1] „In the human spinal cord the canal is often completely filled up, what would appear to be the débris of the epithelium; for nothing is to be seen but a confused heap of nuclei, which are here mostly large and round: but sometimes in the midst of this heap there remains a small opening or canal, which strange to say is still lined or surrounded at its margin by the usual regular layer of columnar cells, and what is still more curious I occasionally find particularly in the cervical region two such secondary canals, each lined in the ordinary way.“

Diese Schilderung der Vorgänge scheint ganz vergessen worden zu sein. Auch Brissaud, der im allgemeinen eine Bestätigung der Clarkeschen Angaben liefert, scheint sie nicht gekannt zu haben.

Die Golgische Methode dürfte auch zur Auffindung dieser schon den Alten bekannten, wenn auch erst von Frommann richtig dargestellten Thatsache insuffizient sein. Wenigstens schliefse ich das daraus, dafs ein so genauer Kenner der durch diese Methode zu erschliefsenden Thatsachen, wie Lenhossék, auch nur an die Möglichkeit denken konnte, dafs die Obliteration des Centralkanals durch Mifshandlung des Rückenmarks bei der Herausnahme zu Stande gekommen sein könnte, also in ähnlicher Weise, wie dies van Gieson in seiner berühmten Arbeit für so vieles andere nachgewiesen hat. Daran ist aber garnicht zu denken. Ganz abgesehen von dem aufserordentlich typischen der Neurogliawucherung, das mit einer zufälligen Verletzung bei der Herausnahme des Rückenmarks garnicht in Einklang zu bringen wäre, ganz abgesehen davon, dafs diese Veränderung sich in sonst ganz wohl erhaltenen, mit gröfster Behutsamkeit herausgenommenen Rückenmarken findet, wäre es doch gar zu wunderbar, wenn die mechanische Schädigung des Rückenmarkes niemals bei jugendlichen, immer aber bei alten Individuen eintreten sollte, während die übrigen von van Gieson geschilderten Kunstprodukte in allen möglichen Altersstufen zu Stande kommen.

Wir können also getrost die Obliteration des Centralkanals zu den natürlichen Altersveränderungen des menschlichen Körpers rechnen. Auch Brissaud protestiert dagegen, hier Kunstprodukte sehen zu wollen.

---

[1] Philosophical transactions. 1859. S. 455.

2. Medulla oblongata.

Wenn wir einen Querschnitt der Medulla oblongata, der nach der neuen Methode behandelt ist, makroscopisch betrachten, so finden wir an ihm folgendes. So lange der Centralkanal noch geschlossen ist, ist dessen Umgebung, ganz wie im Rückenmark, als dunkelblauer Fleck erkennbar. Mit dem Auftreten der Oliven aber kommt ein neues Element hinzu, das schon für das bloſse Auge dem Querschnitt der Medulla oblongata bei unserer Färbung ein sehr charakteristisches Gepräge verleiht. Die Oliven sind nämlich als dunkelblaue Flecke deutlich gegen die ganze übrige Umgebung abgehoben. Ferner sieht man an den höher gelegenen Partieen der Medulla oblongata, d. h. da, wo der Centralkanal dem Ventrikel Platz gemacht hat, den Saum des letzteren als dunkelblauen, an seinem unteren Ende verwaschenen Sreifen, und auſserdem einen dunkleren Strich, der der Raphe entspricht. Oben seitlich sind verwaschene bläuliche Zeichnungen zu bemerken.

Bei der mikroscopischen Betrachtung beginnen wir wieder mit der Rindenschicht.

A. Rindenschicht.

Sie ist ähnlich beschaffen, wie am Rückenmark, aber doch mit einer Ausnahme. Da nämlich, wo an der Oberfläche Nervenbündel parallel mit dieser, d. h. tangential, verlaufen, pflegt die Rindenschicht als besondere Lage nur wenig angedeutet zu sein.

Nichtsdestoweniger fehlt sie hier eigentlich nicht, sondern sie ist gewissermaſsen in das Innere der tangentialen Nervenmassen verlegt, die besonders in ihren oberflächlichen Zügen ein sehr reiches Neurogliageflecht aufweisen, in welchem namentlich auch radiäre Faserungen zu bemerken sind. (Taf. VI, Fig. 2. Fibrae arciformes externae.)

B. Weisse Substanz.

In der weiſsen Substanz ist, wie überall, jede Nervenfibrille von der anderen durch Neuroglia getrennt.

Beim Vergleich mit einem Rückenmarksquerschnitt fällt einem aber auf, daſs die Anordnung der Neurogliafasern eine viel kompliziertere ist. Das kommt daher, weil in der Medulla oblongata die Nervenfasern nicht mehr so gleichmäſsig in vertikaler Richtung verlaufen, wie das für die Hauptmasse der markhaltigen Fibrillen im Rückenmark gilt.

Die Nervenfasern der Medulla oblongata sind vielmehr zu vielfach durchflochtenen Bündeln angeordnet, und da in diesen Bündeln die Neurogliafasern hauptsächlich den Nervenfibrillen parallel verlaufen, so durchkreuzen sich auch die Richtungen der Neuroglia-

fasern. An den gröberen Bündeln sieht man hier und da auch verdichtete Randschichten. Ganz besonders gilt das für die sich kreuzenden Pyramidenbündel (Taf. V, Fig. 3). Es sei noch daran erinnert, dafs die Zusammenlagerung der Pyramidenfasern zu Bündeln schon im oberen Teile des Rückenmarks, vor der Kreuzung also, beginnt.

Dafs die der Peripherie benachbart gelegenen Teile der weifsen Substanz in der Medulla oblongata besonders reich an Neuroglia sind, wurde schon S. 78 konstatiert.

In der Gegend der Raphe lösen sich die Bündel mehr und mehr in einzelne Nervenfibrillen auf. Diese durchkreuzen sich und sind durch reichliche, verschieden gerichtete Neurogliafasern von einander getrennt (Taf. VI, Fig. 1). Schon durch diese zahlreichen sich durchflechtenden Neurogliafasern erscheint die Raphe dem blofsen Auge dunkler gefärbt, als die Umgebung. Dazu kommt aber noch etwas anderes.

Von der Ventrikelseite her sowohl, wie von der pialen Oberfläche dringen noch zwei stärkere Verdichtungen in die Raphe ein. Die vom Boden des Ventrikels herkommende Verdichtung der Neuroglia nimmt etwa das obere Drittel der Raphe für sich in Anspruch. Sie hängt mit dem Ependym nicht nur direkt zusammen, sondern stimmt mit diesem zunächst auch im Gefüge vollständig überein. Allmählich freilich wird dieses lockerer und lockerer und klingt gegen den Beginn des mittleren Drittels der Raphe ganz ab.

Die zweite (ventrale) Verdichtung nimmt ungefähr das untere Drittel der Raphe für sich in Anspruch. Sie hängt mit der äufseren Rindenschicht direkt zusammen, ist dieser entsprechend konstruiert und besitzt also nicht so dichte Massen, wie der dorsale (ventrikuläre) Verdichtungsstreifen. Auch sie verliert sich gegen das mittlere Drittel hin.

Beide eben erwähnten Verdichtungen des oberen und unteren Drittels der Raphe sind ohne Zweifel als „Kielstreifen“ (S. 74) zu betrachten.

C. Graue Massen.

Die Substantia gelatinosa Rolando und die Reste der Vorderhörner verhalten sich wie im Rückenmark. Die Kerne der zarten (Taf. VI, Fig. 4) und Keilstränge zeigen ein sehr unregelmäfsiges Maschenwerk, oft mit leichten Verdichtungen um die Ganglienzellen. Die Masse der Neuroglia ist geringer, als die der ventrikulären Kerne.

Bei den letzteren macht sich der Einflufs des Ependyms und des dorsalen Kielstreifens in sofern geltend, als deren mächtige Neurogliamassen sehr allmählich in der Tiefe der Nervenkerne abklingen. Aber auch die vom Ependym entfernteren Teile der ventrikulären (dorsalen) Kerne sind noch reich an Neurogliafasern (Taf. V, Fig. 4: Vom Ependym

entfernterer Teil des Hypoglossuskerns). Zwischen den Geflechten der verschiedenen dorsalen Nervenkerne scheinen Unterschiede in der Anordnung zu bestehen, doch bedürfen diese zum Verständnis ihres Wesens noch weiterer Studien.

Die Pyramidenkerne (Taf. VI, Fig. 2), der Nucleus ambiguus (Taf. VII, Fig. 1) sowie sonstige eingestreute Ganglienzellhaufen zeigen sehr unregelmäfsige Neurogliageflechte, die recht dicht gewebt sind, wenn auch lange nicht so dicht wie die in den Oliven. Die Ganglienzellen dieser Gruppen haben meist Körbe um ihre Körper und entlang ihrer gröberen Fortsätze (Taf. VII, Fig. 1). Ganz regelmäfsig finden sich diese Körbe um die in der Medulla oblongata zerstreuten einzelliegenden Ganglienzellen, wie wir schon S. 74 sub b erwähnten. —

Das dichteste Neurogliagefüge (immer mit Ausnahme des Ependyms) hat aber in der Medulla oblongata die Olive, wie schon der oben beschriebene makroskopische Anblick beweist. Die Olive gehört überhaupt zu denjenigen Teilen des Centralnervensystems, die ein hervorragend dichtes Neuroglianetz besitzen. Freilich eine so eximierte Stellung, wie Petrone der Olive in Bezug auf die Dichtigkeit ihres Neurogliageflechtes zuschreibt, hat diese nicht. Er glaubt, sie hätte das dichteste Netz im ganzen Centralnervensystem und ruft bewundernd aus: „Chi non lo vede, non lo crede!“ Er konnte eben mit seinen so wenig elektiven Methoden die dichteren ependymären Netze nicht entwirren, obgleich es immerhin auffallend ist, dafs er die Dichtigkeit des Neurogliageflechts in der Olive, die vorher allen entgangen war, doch entdeckt hat.

Die Golgische Methode hat sich auch hier wieder als so unzureichend für die Erkennung der topographischen Verhältnisse erwiesen, dafs die schon mit blofsem Auge erkennbare Dichtigkeit des Neurogliageflechts in den Oliven mit Hilfe dieser Methode weder vor meiner (und Petrones) Mitteilung erkannt wurde, noch nach dieser eine Bestätigung erfahren hat, obgleich die Medulla oblongata mehrfach auf ihre Neuroglia hin untersucht worden ist.

Die Neurogliaverdichtung macht sich vor allem auch in den weifsen (markhaltigen) Fasermassen geltend (Taf. VI, Fig. 3), die die Oliven umschlingen und durchziehn. Innerhalb der eigentlichen grauen Massen ist das Gefüge ein wenig lockerer, aber immer noch sehr dicht. Die Fasern durchkreuzen sich in den verschiedensten, aber hauptsächlich in der frontalen Ebene verlaufenden Richtungen, so dafs sie sehr kleine (0,002—0,005 mm im Durchmesser haltende) Maschenräume umschliefsen. Im allgemeinen sind die Fasern sehr fein, doch durchziehen auch gröbere das Feld. Selbst in diesem Gewirr kann man,

wenn man die Schraube spielen läfst, oft genug mit reichlichen Strahlen versehene „Astrocyten" wahrnehmen. In unserer Figur, die mit möglichst geringer Schraubenbenutzung gezeichnet ist, treten solche aber nicht deutlich hervor. Aufser den hellen, gröfseren, oft mit strahlig angelehnten Fasern versehenen Kernen giebt es aber auch viele dunklere, kleinere, die keine gruppierten Fasern um sich zeigen.

D. Ependym.

Das Ependym zeigt im allgemeinen die ihm gebührende Neurogliaverdichtung in reichem Mafse, doch sind hier gewisse Eigentümlichkeiten zu erwähnen. Einmal verlaufen dicht unter dem Epithel ja an einigen Stellen mächtige markhaltige Faserbündel, die Striae acusticae. Hier findet sich keine besondere gliöse Ependymschicht, das Epithel sitzt vielmehr direkt den markhaltigen Nervenfasern auf. Dafür sind diese (analog den Fibrae arciformes externae) von einer dichten Neurogliamasse durchsetzt, ganz anders wie sonst die weifsen Massen (Taf. VII, Fig. 2: rechts sind die Fasern längs getroffen, links schief). Die Faserrichtung ist hauptsächlich parallel dem Verlaufe der Nervenfasern, doch treten auch genug senkrecht dazu verlaufende ein. Bemerkenswert ist auch, dafs die Bündel Lücken frei lassen, d. h. auf kurze Strecken gewissermafsen überhängen. In diese Lücken setzt sich das Epithel fort, und so werden auf den Schnitten cystenähnliche Räume vorgetäuscht (Taf. VII, Fig. 2 a).

Eine fernere Eigentümlichkeit wird durch den Plexus chorioideus hervorgerufen. Nicht nur, dafs zu ihm kegelige Fortsätze vom Ependym her kommen, in welche die Neuroglia (vgl. oben S. 72) sich eine kleine Strecke weit fortsetzt, er liegt vielmehr an den Seitenteilen des vierten Ventrikels auch flach auf, so dafs der Ventrikelboden hier nicht mehr direkt von Epithel, sondern von Bindegewebe bedeckt ist. Ein eigentliches Hineinwachsen des Bindegewebes in die Substanz der Medulla oblongata, von dem Gierke spricht, habe ich nie bemerkt. —

Eine weitere Eigentümlichkeit wird hier (und überhaupt am Ependym) durch die von Virchow entdeckten „Ependymwucherungen" bedingt, die man wohl als noch an der Grenze des normalen stehend ansehen kann, insofern als Altersveränderungen noch an dieser Grenze stehen. Freilich kommen sie in ganz besonderer Mächtigkeit auch unter direkt pathologischen Verhältnissen vor. In letzter Zeit ist mir nur ein solcher Fall von „glasigen Körnchen" im Ependym frisch genug zur Sektion gekommen, der der folgenden Erörterung zu Grunde gelegt ist.

Während sich das Epithel in der Medulla oblongata, so lange der Centralkanal noch geschlossen ist, ganz wie das des Rückenmarks verhält und im Alter die Ablösungen, Durchwachsungen mit Neuroglia etc. zeigt, gerade wie dieses (Taf. IV, Fig. 5), so ändert sich das in dem offenen Ventrikel. Hier bleibt das Epithel im allgemeinen wohlerhalten (auch mit Körnchensaum versehen) in zusammenhängender Schicht liegen, nur hier und da weichen die Zellen etwas auseinander und lassen einen Neurogliafaden zwischen sich treten.

In dem erwähnten Falle von „Ependymwucherungen" zeigten aber die Excrescenzen, die buckelförmig über das Niveau der Ventrikeloberfläche hervorragten, auf der Kuppe des Buckels einen Epitheldefekt (vgl. Taf. V, Fig. 1). Erst an den unteren Teilen der Abhänge trat das Epithel wieder auf. Anfangs waren die Zellen etwas niedriger, sehr bald nahmen sie aber ihre gewöhnliche Gestalt an. Lagen zwei solcher Knötchen dicht neben einander, so verschmolzen die epithelentblöfsten oberen Teile, während die basalen Abhangsteile, die ja von Epithel bedeckt waren, das nicht zu thun vermochten. Dadurch wurden epithelumgrenzte Hohlräume abgeschnitten, die wie geschlossene Cysten erschienen, in Wirklichkeit aber vielleicht tunnelförmig waren (Taf. V, Fig. 2).

Sollte sich auch in anderen derartigen Fällen der ebenerwähnte Epithelverlust finden, so wäre die Pathogenese dieser Wucherungen eine sehr einfache. Man brauchte nur den Epithelverlust als das primäre anzusehen. Durch den Wegfall des Epithels wäre ja dann der Gewebswiderstand für die unterliegende Neurogliamasse beseitigt, und es würde die schlummernde, d. h. bisher in ihren natürlichen Schranken gehaltene idioplastische Kraft der Neurogliazellen wieder in thätige, im wahren Sinne des Wortes lebendige Kraft übergeführt, und so eine die physiologischen Grenzen überschreitende Neurogliawucherung hervorgerufen werden.

Prinzipiell würden diese Verhältnisse also den früher für den Centralkanal des Rückenmarks geschilderten durchaus ähnlich sein. Auch in letzterem findet eine Abstofsung des Epithels und eine durch sie bedingte Neurogliawucherung statt, aber bei aller Übereinstimmung im Prinzip finden sich doch Unterschiede zwischen unserem Falle von Ependymwucherungen und denjenigen Erscheinungen, welche zur Obliteration des Centralkanals führen.

Einmal sind die Ependymwucherungen durchaus nicht so regelmäfsige Erscheinungen, wie die analogen Prozesse am Centralkanal des Rückenmarks. Dann aber sind noch Unterschiede vorhanden, die aus der Verschiedenheit der Lokalitäten unschwer zu erklären sind.

In den Ventrikeln begrenzt ja das Epithel nicht einen sehr langen und sehr engen Hohlraum, sondern eine weite Höhle. In dem engen Centralkanal nun bleiben die abgestofsenen Epithelzellen liegen und werden nur von der Neuroglia durchwachsen. Fallen aber an der Umgrenzung des Ventrikels Zellen ab, so werden sie nicht durch die Engigkeit des Raumes an Ort und Stelle festgehalten, sondern sie fallen in den weiten Hohlraum und verschwinden in unbekannter Weise.

Wenn sich die in unserem Einzelfalle gefundenen Thatsachen regelmäfsig vorfinden sollten, so würden sie uns noch über etwas anderes aufklären.

Die Ependymwucherungen sehen bekanntlich oft nicht einfach grau aus, sondern sie haben ein tautropfenähnliches, durchscheinendes Aussehen. Ein solcher Fall lag hier vor, und dem entsprechend sehen wir denn, dafs im Gipfel der Wucherung (Taf. V, Fig. 1) die Neurogliafasern sehr sparsam sind im Gegensatz zu den Teilen in der Tiefe des Buckels und an seiner Basis. Diese „hyaline“ Umwandlung (das Wort „Hyalin“ aber nur im morphologischen, nicht im Sinne von Recklinghausens gebraucht) dürfte sich ähnlich erklären, wie die hyaline Umwandlung, die bei der Syringomyelie des Rückenmarks beobachtet[1] wird, nämlich durch den Druck des Liquor cerebrospinalis, dessen Wirkung nicht durch das schützende, dem Druck angepafste Epithel paralysiert wird. In ähnlicher Weise habe ich die „Hyalinbildung“ vor Jahren bereits für andere Fälle auf Druckwirkung zurückführen können.[2]

---

3. Pons.

Die weifsen Substanzen und die ventrikulären Kerne verhalten sich denen der Medulla oblongata entsprechend. Die überall sonst eingestreuten Nervenkerne sind sehr reich an sich mannigfach durchflechtenden Neurogliafasern. Ihr Typus ist der des Nucleus ambiguus (Taf. VII, Fig. 1). Die von Popoff angegebenen Abstufungen in der Dichtigkeit der Netze, welche die verschiedenen Nervenkerne durchziehen (S. 27), kann ich nicht bestätigen.

---

4. Pedunculus cerebri.

Von besonderen Bestandteilen sind hier zu erwähnen die Substantia nigra und der Nucleus ruber. Die erstere (Taf. VIII, Fig. 1) zeigt ein reiches Neurogliageflecht etwa von

---

[1] Vgl. Weigert, Centralblatt für allgemeine Pathologie und pathologische Anatomie. 1890. S. 737.
[2] Deutsche medizinische Wochenschrift. 1885. S. 814.

dem Charakter des Vorderhorngeflechts, aber doch eigenartig. Die Ganglienzellen sind darin oft mit zarten Körben versehen.

Ganz anders ist die Neuroglia des roten Kerns beschaffen. Sie stellt ein äufserst zierliches Geflecht dar, mit ungemein zahlreichen grofsen Astrocytenformen, die lange feine Fasern in das Gewebe absenden. Hier treten sie zwischen je zwei markhaltige Fasern als Zwischenmasse hinein, geben an die Ganglienzellen zarte Körbe ab und umscheiden natürlich auch die Gefäfse. Im ganzen hat das Geflecht den Typus der weifsen Substanz des Grofshirns, nur sind die Astrocytenformen viel zahlreicher und ausgebildeter. Auch die eingestreuten Ganglienzellen machen natürlich einen Unterschied aus.

---

5. Vierhügel.

Die Vierhügel haben ein reiches Neuroglianetz von einem geradezu ästhetischen Charakter. Schon für das blofse Auge tritt die Bläuung des Organs stärker hervor, als bei sonstigen so grofsen Abschnitten im Centralnervensystem, und die makroskopische Betrachtung giebt schon ein Bild von der Grundanlage des Neurogliageflechtes.

Betrachtet man einen Frontalschnitt mit blofsem Auge (Taf. XIII, Fig. 4) oder mit der Lupe, so sieht man in der Mittellinie einen dunkelblauen Verbindungsstreifen die Oberfläche mit dem Aquaeductus Sylvii verbinden. Oben ist dieser Verbindungsstreifen ca. 2 mm breit, nach unten zu verschmälert er sich etwas, wobei er an den oberen Rand des Aquaeductus Sylvii herantritt. An den Seitenrändern des letzteren sind die oberen Hälften für das blofse Auge nicht durch eine starke Bläuung der angrenzenden Teile ausgezeichnet, hingegen zeigt die untere Hälfte beiderseits einen sehr dunklen Ansatz. Der obere Rand dieses dunklen Ansatzes fällt etwas schief nach aufsen und unten ab und reicht beiderseits etwa einen Millimeter weit, um dann mit Bildung einer verhältnismäfsig scharfen Spitze zu enden. Von dieser Spitze ab gehen die äufsern Ränder des blauen medialen Feldes mit leichter lateralwärts gekehrter Convexität nach unten, und unterhalb des Aquaeductus Sylvii findet sich so ein einheitlicher im allgemeinen dunkelblauer Streif, der immer mehr sich verschmälernd die ganze Substanz der Vierhügel in eine rechte und linke Hälfte teilt. Wenn wir den Streifen nur im allgemeinen als dunkelblau bezeichneten, so geschah dies deshalb, weil unmittelbar am unteren Rande des Aquaeducts in dem hier schon gemeinsamen Streifen ein kleines, etwas helleres Feld zu sehen ist. Die centralen Teile

der beiden Vierhügel, d. h. die vom lateralen Rande und der Mittellinie entfernten, erscheinen dem blofsen Auge ein ganz klein wenig heller blau, als das übrige Areal.

Die mikroscopische Untersuchung bestätigt den kolossalen Neurogliareichtum der dunkelblauen Stellen. Nur sind diese Partieen bei mikroscopischer Betrachtung nicht so scharf begrenzt, wie man nach dem Anblicke mit dem blofsen Auge glauben könnte. Vielmehr löst sich das in der Mittellinie resp. am Rande des Aquaeductus Sylvii ungemein dichte Fasernetz ganz allmählich in die weitere Umgebung auf. Auch der obere Rand des Aquaeducts, der in seinen Seitenteilen sich für das blofse Auge nicht so dunkel ausnimmt, hat eine ependymäre, nur nicht so weit in die Tiefe reichende Verdickung, die zu schmal ist, um sich makroscopisch bemerkbar zu machen.

Die übrigen Partieen der Corpora quadrigemina zeigen ein im allgemeinen ziemlich gleichförmiges (Taf. VIII, Fig. 4), dichtmaschiges Neurogliageflecht ohne Vorherrschen einer bestimmten Richtung. Die Maschen sind unregelmäfsig dreieckig, viereckig, polyedrisch oder rundlich. Nur wo Bündel von Nervenfasern eingelagert sind, zeigen diese den ausgesprochenen Typus der weifsen Substanz mit den Nervenfasern mehr parallel gerichteten Zügen. Einzelne Nervenfasern machen sich nicht besonders störend geltend. Die zahlreichen Ganglienzellen haben Andeutungen einer Korbbildung in ihrer Umgebung. „Astrocyten“ sind sehr reichlich zu sehen. —

Der Oculomotoriuskern liegt der Mittellinie sehr nahe, und sein medialer Teil liegt in dem sehr dichten Neurogliageflecht des Mittelteils eingebettet, aber auch die lateralen Abschnitte sind noch ungemein reich an Neurogliafasern (Taf. VIII, Fig. 3). —

Die obere Fläche der Vierhügel, die nicht, wie der Aquaeductus Sylvii mit Epithel bekleidet ist, besitzt ebenfalls eine verdichtete Rindenschicht von 0,075 mm ungefährer Dicke, die sich nach innen zu ziemlich rasch in ein lockeres Geflecht auflöst. Die erwähnte Verbindung der Mitte der Oberfläche mit dem oberen Rande des Aquaeductus Sylvii ist als Kielstreifen aufzufassen, ebenso die nach unten gehende Verlängerung der ependymären dichten Neurogliaanhäufung.

---

6. Zirbeldrüse.

Die Zirbeldrüse besitzt in ihrem inneren unteren Abschnitt ein ganz ungemein mächtiges Neuroglialager. Es ist so mächtig, dafs es für das blofse Auge als grofser blauer Fleck erscheint (Taf. XIII, Fig. 5). Oberhalb dieses Flecks ist eine kleine Höhle.

Die mikroscopische Untersuchung zeigt diese Stelle aus einem dichten Geflecht kräftiger Neurogliafasern bestehend, so eigenartig, wie sonst nirgends im Centralnervensystem. Von dieser dichten Masse gehen ähnlich beschaffene dünnere, dichte Züge zwischen die Zell anhäufungen der Zirbeldrüse hinein (Taf. XIII, Fig. 3). Die Zellen selbst sind von einem reichlichen aber lockeren Geflechte von kräftigen Neurogliafasern durchzogen (Taf. XIII, Fig. 3).

---

7. Kleinhirn.

Das Kleinhirn entbehrt, wie wir schon S. 73 sub 2 erwähnten, einer dichteren Rindenschicht. In dieser Beziehung nimmt es ja im ganzen Centralnervensystem eine isolierte Stellung ein, gerade wie in der anderen, mit der ersten vielleicht zusammenhängenden Eigenschaft, dafs es der einzige Ort ist, an dem sich markhaltige Fasern nicht in gröfserer Menge (z. B. als Tangentialfasern) in der Nähe der Oberfläche befinden.

In der Molekularschicht sieht man nun in Abständen von etwa 0,01 mm, manchmal aber enger, manchmal weiter stehend, radiäre Fasern von der Oberfläche her in die Tiefe strahlen und sich in der Gegend der Purkinjeschen Zellen verlieren (Taf. IX, Fig. 5). Hier und da sind dieselben an der Oberfläche umgebogen und legen sich dann flach an diese an. Geschieht dies vielfach, so entsteht eine freilich nur aus einer Faserlage bestehende rudimentäre Rindenschicht. Vielleicht ist aber auch das schon eine Alterserscheinung.[1]

Das sind die altberühmten „Bergmannschen Fasern". Es ist mir aber mehr als zweifelhaft, ob gerade Bergmann die Fasern richtig gesehen hat. Er beschreibt sie nämlich[2] als „netzförmig" mit einander verbunden, während sie doch radiäre Fasern ohne Netz- (oder, wie man jetzt sagt, Geflechts-) Bildung darstellen. Ja, in einem späteren Aufsatze[3] protestiert er sogar ausdrücklich gegen Kupffer,[4] „der die nach innen dringenden

---

[1] Umgekehrt findet sich hier bei Embryonen, und sogar noch deutlich bei neugeborenen Kindern, eine mehrschichtige Lage von Zellen. Diese hat nach den allgemeinen Angaben zuerst Hess (De cerebelli textura. Dorpater Dissertation) 1858 beschrieben, doch ist mir dessen Schrift nicht zugänglich. Die Schicht ist in neuerer Zeit vielfach als „äufsere Körnerschicht", von Retzius als „Vignalsche Schicht" geschildert worden.

[2] Zeitschrift für rationelle Medizin. Neue Folge. Bd. 8.

[3] Dieselbe Zeitschrift. 3. Reihe. Bd. 11. S 264.

[4] In Stephanys Beiträgen zur Histologie der Rinde des grofsen Gehirns. Dorpat 1860. Mir nicht zugänglich.

Fasern vorwiegend gerade nach innen laufen und so mit den Radialfasern der Netzhaut mehr Ähnlichkeit darbieten läfst.“ Demnach kann Bergmann die Fasern garnicht rein gesehen haben, wenn er nicht überhaupt etwas anderes gesehen hat, als was wir jetzt mit seinem Namen belegen. Kupffer dürfte sie eher richtig wahrgenommen haben, doch hielt er sie, wie Bergmann angiebt, für Nervenfasern. Sicher hat sie aber Deiters ganz sachgemäfs beschrieben, u. zw. unabhängig von Bergmann u. a. (vgl. S. 11), so dafs er als der eigentliche Entdecker der Fasern anzusprechen ist.

Der von ziemlich allen Autoren gemachte Vergleich der Bergmannschen Fasern mit den Müllerschen Fasern der Retina scheint mir aber doch nicht zutreffend zu sein. Zunächst möchte ich, freilich mit aller Reserve, die Meinung aussprechen, dafs die Müllerschen Fasern chemisch nicht mit der Neuroglia übereinstimmen (meine Untersuehungen über die Retina sind noch nicht abgeschlossen), sodann aber sind die Müllerschen Fasern viel dicker und an beiden Enden büschelförmig gespalten, so dafs die ganze Ähnlichkeit sich eigentlich nur auf den radiären Verlauf beziehen kann.

Die Bergmannschen Fasern sind bei jugendlichen Individuen spärlicher, als bei älteren Leuten. Sehr reichlich werden sie stellenweise bei progressiver Paralyse, noch reichlicher bei multipler Sklerose. Über die krankhafte Bildung einer Rindenschicht dabei haben wir S. 73 sub 2 schon gesprochen.

Aufser den eigentlich radiären Fasern sieht man in der Molecularschicht in den oberflächlichen Teilen sehr spärliche, nach unten zu reichlichere, aber doch immer sehr zerstreute quere Fasern, besonders in der Nähe der Purkinjeschen Zellen (Taf. IX, Fig. 4). Um die Purkinjeschen Zellen selbst sind bei jugendlichen Menschen spärliche Fasern, bei alten Leuten reichlichere korbartige Faseranhäufungen zu finden (Taf. IX, Fig. 4 von einer alten Frau entnommen). Bei progressiver Paralyse und multipler Sklerose nehmen diese Fasern aufserordentlich zu. —

Sehr zweifelhaft ist es mir, ob alles das, was am Kleinhirn aus Golgipräparaten als Neuroglia beschrieben worden ist, auch wirklich dieser zugerechnet werden kann, doch ist der Vergleich der „Zellsilhouetten“ mit meinen Bildern nicht so leicht sicher auszuführen. —

In der Körnerschicht habe ich so gut wie gar keine Neurogliafasern unter normalen Verhältnissen, wohl aber reichlich bei progressiver Paralyse etc. gefunden. Selbst um die

Gefäfse herum war nur selten einmal eine zu entdecken, ganz im Gegensatz zu den Angaben von Golgi (vgl. S. 17). —

Hingegen zeigt die Marksubstanz, wie alle Autoren konstatieren, ein sehr schönes Neurogliageflecht, ganz dem Typus der weifsen Substanzen entsprechend: die Fasern hauptsächlich, aber nicht ausschliefslich, parallel den Nervenfasern verlaufend, mit reichlich eingelagerten schönen „Astrocyten". (Taf. IX Fig. 3.)

Da, wo das Kleinhirn die Decke des vierten Ventrikels bildet, ist es natürlich mit Ependym bedeckt. Der Einflufs desselben macht sich den allgemein-topographischen Regeln entsprechend auch in der angrenzenden Markmasse des Kleinhirns geltend. Sie ist hier von einem viel dichteren Neurogliageflecht durchsetzt, als an den vom Ependym entfernt liegenden Stellen.

---

8. Grosshirn.

Die Rindenschicht am Grofshirn ist von verschiedenen Autoren mit der des Kleinhirns zusammengestellt worden, was durchaus unzutreffend ist. Am Grofshirn ist vielmehr eine typische, dicht unter der Pia mater gelegene, aus eng verwebten Fasern bestehende echte Rindenschicht vorhanden (Taf. IX, Fig. 1 a), die am Kleinhirn fehlt. Ihre Dicke ist sehr wechselnd und dürfte zwischen 0,003 bis 0,03 variieren, je nach der Stelle des Grofshirns und je nach dem Alter des Individuums. Im höheren Alter wird sie, wie auch schon Golgi angegeben hat, dicker und ihre Fasern werden gröber. Die Richtung der Fasern in dieser eigentlichen Rindenschicht ist eine sehr wechselnde, im allgemeinen aber schief tangentiale.

Auch für die Erkenntnis dieser und der folgenden Schicht reicht die Golgische Methode nicht aus. Sie giebt nur sehr unvollkommene Bilder, wie die zahlreichen Abbildungen lehren, die sich in den Veröffentlichungen vorfinden. Namentlich versagt sie für das höhere Alter, wie das Retzius konstatiert hat.[1]

Von der dichteren, eigentlichen Rindenschicht strahlen dann lockere hauptsächlich (aber wieder nicht ausschliefslich) schief radiär gerichtete Fasermassen in die Tiefe. Zunächst sind sie, wenn auch diskret stehend, doch noch recht zahlreich (Taf. IX, Fig. 1 b), allmählich aber werden sie immer spärlicher und verlieren sich schliefslich ganz.

[1] Die Neuroglia des Gehirns beim Menschen und bei Säugetieren. Jena 1894. S. 11.

Diese zweite Schicht reicht auch an verschiedenen Stellen mehr oder weniger weit hinab, bei alten Leuten tiefer. Bis zur unteren Grenze der kleinen Pyramidenzellen lassen sie sich sehr oft verfolgen. Lloyd Andriezen[1] giebt an, dafs sie „bis in die Mitte der Pyramidenzellen reichen". Die in der Zone der radiären Neurogliafasern liegenden Gefäfse zeigen je nach ihrer Gröfse geringere oder stärkere Gliahüllen.

In den tieferen Schichten der Grofshirnrinde, auch in der der radiären markhaltigen Fasern habe ich Neuroglia nur in ganz zerstreuten Fäserchen gesehen, auf weite Strecken sogar ganz vermifst, so dafs ich das zusammenhängende Geflecht von Bindesubstanz, welches Golgi noch 1885 annimmt, absolut nicht bestätigen kann.

In der Marksubstanz hingegen ist wieder ein reiches Neurogliageflecht vorhanden vom Typus der weifsen Substanzen überhaupt, in specie sehr ähnlich dem entsprechenden im Kleinhirn. Nur sind die Fasern im Grofshirn etwas feiner und die Maschen etwas enger (vgl. Taf. IX, Fig. 2). —

Es sei noch einmal besonders darauf hingewiesen, dafs die von Golgi, Ranvier, Lloyd Andriezen und Retzius geschilderten, als Neurogliazellen angesprochenen protoplasmatischen Zellen mit der neuen Methode nicht wahrgenommen werden können. Für den Fall, dafs es wirklich zutreffen sollte, wie dies Lloyd Andriezen meint, dafs diese „protoplasmatic elements" mesoplastischen Ursprungs wären, im Gegensatz zu den epiplastischen „fibre-elements", so wäre damit ein so prinzipieller Gegensatz gegen die eigentlichen Astrocyten geschaffen, dafs schon aus diesem Grunde die „protoplasmatischen Elemente" von der eigentlichen Neuroglia abzutrennen wären. Die Berechtigung einer solchen Annahme können wir freilich weder anerkennen noch ablehnen.

Hingegen irrt Lloyd Andriezen ganz sicher, wenn er (British medical Journal 1893, 29. Juli) meint, dafs „the protoplasmatic glia elements are really the elements, which exhibit a morbid hypertrophy in pathological conditions (alcoholism, G. P.) and which may show further morbid activities, in the last stage of which their protoplasma will deposit numerous organised fibrillae, in the act of doing which the protoplasma proper is used up except a scanty remnant, which may persiste, ghost-like, to marke the position of what was once a protoplasmatic cell body." Gerade in patho-

---

[1] Internationale Monatsschrift für Anatomie. 1893. S 537.

logischen Fällen, ganz besonders bei G. P. (general paralysis), sieht man nicht nur ungeheure Mengen von typischen „Astrocyten“, also nicht von protoplasmatic cells, neu auftauchen, mit echten, nur sehr dicken Neurogliafasern, sondern es trifft gerade hier nicht zu, dafs das Protoplasma verbraucht wird, und nur ein „Gespenst des Zellleibs“ zurückbleibt. Gerade bei der progressiven Paralyse sind die Zellleiber ungewöhnlich grofs, man kann sogar dicke protoplasmatische Fortsätze sehen, an die die Fasern (freilich scharf von ihnen abgesetzt) sich eine Strecke weit anlehnen.

---

9. Gyrus hippocampi. Cornu Ammonis.

Das Ammonshorn ist ein so kompliziertes Organ, dafs man sich nicht wundern kann, wenn in ihm auch die Neurogliaverhältnisse sehr verwickelt sind. Entsprechend dem Umstande, dafs bei der Entstehung des Ammonshorns allerlei Einstülpungen und Umbiegungen stattgefunden haben, tritt hier auch die Kielstreifenbildung mehrfach hervor.

Zur Erleichterung des Verständnisses haben wir eine Zeichnung in Lupenvergröfserung auf Taf. XIII, Fig. 2 beigefügt (1 : $3^1/_2$). Auf dieser sind aber nur diejenigen Neurogliazüge angegeben, welche man bei dieser Vergröfserung nachweisen kann. Einige Details sind auf Taf. X, Fig. 1 bis 3 und Taf. XIII, Fig. 1 bei starker Vergröfserung abgezeichnet. Die folgende Beschreibung geht in der Richtung der Pfeile in Fig. 2, Taf. XIII.

A. Gyrus hippocampi.

Wir beginnen mit der Umbiegungsstelle des Gyrus hippocampi zum Ammonshorn (Taf. XIII, Fig. 2 a). Am Gyrus hippocampi liegen bekanntlich markhaltige Fasern frei an der Oberfläche. Diese entsprechen ja den gewöhnlichen Transversalfasern, sind aber mächtiger, als diese, und liegen nicht in graue Substanz eingebettet. Die weifse Schicht ist keine kontinuierliche, sondern besteht aus netzförmigen Zügen, in deren Maschen die Rindenoberfläche grau erscheint. (Substantia reticularis alba Arnoldi.)

Entsprechend dem Umstande, dafs hier markhaltige Züge frei an der Oberfläche liegen, finden wir die Neurogliaverhältnisse etwas abweichend von denen der übrigen Grofshirnrinde. Zwar liegt auch hier eine Rindenschicht von dicht verflochtenen Fasern in einer Dicke von etwa 0,02 mm oben auf, aber dann folgen nicht direkt die (hauptsächlich)

radiär verlaufenden, in die Rinde einstrahlenden Neurogliafasern, sondern es kommt zunächst eine ca. 0,2 mm breite Lage, die aus einem recht dichten Geflechte von Neurogliafasern besteht, welche nach allen Richtungen hin verlaufen. Innerhalb dieses Fasergeflechts sind „Astrocyten“ zu sehen. Die Fasern fassen Maschen ein, die grofs genug sind, um je eine markhaltige Faser in sich aufzunehmen. Erst aus dieser oben und unten einigermafsen scharf begrenzten Schicht entwickeln sich dann die in die Tiefe gehenden hauptsächlich radiär gestellten Fasern, die zum Typus der Neuroglia in der Grofshirnrinde gehören. Sie verlaufen auch mit immer abnehmender Dichtigkeit (ca. 0,4 mm weit) in die darunter liegende Hirnrinde, deren tiefste Schichten auch hier wieder die für diese Stellen charakteristische aufserordentliche Spärlichkeit der Neuroglia aufweisen. Abgesehen von der Abweichung, die durch das Auftreten der oberflächlich liegenden mächtigen Tangentialfaserzone und die dadurch veranlafste Einschiebung einer besonderen Neurogliaschicht gegeben ist, entspricht also die Rinde des Gyrus hippocampi durchaus den übrigen Rindenteilen. Ja, an denjenigen Stellen dieses Gyrus, die der freiliegenden Tangentialfasern entbehren, und die demnach schon dem blofsen Auge grau erscheinen, ist die Neuroglia genau so, wie an den übrigen Rindenteilen beschaffen.

Auf einem Schnitte durch den Gyrus hippocampi wechseln die beiden Arten der Neurogliaverteilung mehrfach ab. In der Nähe der Fissura hippocampi (Taf. XIII, Fig. 2 b), wo das eigentliche Ammonshorn beginnt, scheint aber stets die weifse oberflächliche Schicht und die ihr entsprechende Neurogliaanordnung vorhanden zu sein.

B. Fissura hippocampi.

Von der Fissura hippocampi aus erstreckt sich eine Fortsetzung resp. Verschmelzung der Oberflächen des Gyrus hippocampi und des Ammonshorns weit in die Tiefe. Wir werden uns daher nicht wundern, hier einen langen 0,15—0,25 m. m. breiten Kielstreifen der Neuroglia zu finden. Derselbe ist schon mit dem blofsen Auge oder der Lupe zu erkennen (Taf. XIII, Fig. 2 c). Er besteht aus einem dichten Geflecht von Fasern, die den hier verlaufenden markhaltigen Nervenfasern hauptsächlich parallel ziehen, aber doch so, dafs immer noch Nebenfasern in den beiden anderen Richtungen zu beobachten sind. Die Weite der Maschen variiert von 0,002 bis 0,006 mm. Da wir es hier mit den verschmolzenen Tangentialfasern des Gyrus hippocampi und der Ammonshornoberfläche zu thun haben, so finden wir auch, dem Rindentypus entsprechend, von dem Neurogliageflecht dieser Tangentialfasern nach beiden Seiten hin Neurogliafasern ausstrahlend, die zum Verlauf der

Nerven hauptsächlich schief oder senkrecht stehen (Radiärfasern), und die sich allmählich in der Tiefe der anliegenden Rindenschichten verlieren. Die den Tangentialfasern entsprechende und so weit wie diese ins Innere reichende Neuroglia ist hier von demselben dichten Gefüge, wie an der Oberfläche des Gyrus hippocampi, also anders, wie das sonst bei den Tangentialfasern der Grofshirnrinde der Fall ist. —

Die nun folgende rundliche Vorwölbung des Gyrus dentatus (Taf. XIII, Fig. 2 d) hat noch kein Ependym. Sie ist mit einer gewöhnlichen Oberflächenrindenschicht überzogen. —

C. Ammonshorn und Fimbria.

Jetzt kommen wir wieder an eine Einknickung, der unteren Grenze der sich hier ansetzenden Fimbria entsprechend (Taf. XIII, Fig. 2 e). An dieser Einknickung ist die Rindenschicht ungemein entwickelt (Taf. X, Fig. 3), und von ihr aus gehen mehrere für das blofse Auge oder die Lupe ganz gut kenntliche Züge aus (Taf. XIII, Fig. 2 f, f_{I} u. f_{II}). Diese Neurogliazüge sind je nach der Art, wie sie vom Schnitt getroffen werden, 0,2—0,5 mm breit und bestehen aus einem sehr zierlichen, dichten und verhältnismäfsig regelmäfsigen Netzwerk mit polyedrischen Maschen. Dies Netzwerk ist eins der dichtesten und dabei zartesten Geflechte von Neurogliafasern, die sich in weifsen Substanzen des Centralnervensystems finden. Es handelt sich in der That um weifse Substanzen, nämlich um diejenigen, denen die spezifischen Ammonshornzellen seitlich ansitzen. (Taf. X, Fig. 2, starke Vergröfserung.)

Einer dieser Züge verläuft nach der oberen Fläche hin, sich dieser parallel richtend (Taf. XIII, Fig. 2 f), einer in Absätzen parallel der lateralen und ventralen Grenze zwischen Ammonshorn und Gyrus hippocampi (Taf. XII, Fig. 2 f_{II}), und ein dritter zwischen beiden (f_{I}). Auf einer Seite oder auf beiden Seiten dieser Züge liegen die charakteristischen Zellen des Ammonshorns. In diese (Taf. X, Fig. 2, starke Vergröfserung) erstreckt sich ein lockeres Neuroglia-Fasergeflecht, dessen einzelne Fasern hauptsächlich senkrecht zum Verlaufe der Markfasern stehen, aber namentlich in der Nähe dieser letzteren auch durch Fasern anderer Richtungen vielfach durchflochten werden. Bei denjenigen Zellen des Ammonshorns, die in der Nähe der freien Oberfläche oder der tiefen Tangentialfasern liegen, vermischen sich diese, die Zellschicht durchquerenden Fasern mit anderen, die von der freien Oberfläche resp. den tiefen Tangentialfasern her radiär in die Tiefe gehen. Liegen die Ammonshornzellen aber entfernter von den genannten Stellen (wie bei f_{I}), so erreichen die radiären Fasern die spezifischen Zellen des Ammonshorns nicht, und da verlieren sich

denn die Neurogliafasern in die tiefen Rindenschichten, die auch hier ungemein faserarm sind. — —

Wir verfolgen nun die Oberfläche des Ammonshorns in der begonnenen Richtung weiter und kommen nun an die Fimbria. Diese ist sehr reich an Neurogliafasern, die die Nervenfasern durchflechten (Taf. X, Fig. 3, schwächere Vergröfserung; Taf. XIII, Fig. 1, starke Vergröfserung). Auf der lateralen Oberfläche ihres Anfangsteiles zeigt sie die dünnere Oberflächenrindenschicht (Taf. XIII, Fig. 1 a), auf der medialen Seite die dickere Ependymschicht (Taf. XIII, Fig. 1 b).

An die Fimbria setzt sich der Plexus chorioideus an, in den die Neuroglia nur eine kurze Strecke weit hineinzieht (Taf. XIII, Fig. 1 c).

Das Ependymepithel der Fimbria und auch weiterhin das des Ammonshorns ist stellenweise in Form von Höckern (Taf. XIII, Fig. 1 d), aber auch manchmal in der von schlanken Papillen abgehoben, — gewissermafsen die ersten Andeutungen einer Plexusbildung.

An derjenigen Stelle, an welcher die Fimbria zum Plexus chorioideus abbiegt, ist eine Einknickung vorhanden, und von dieser aus erstreckt sich ein Kielstreifen (XIII, 2 g) ins Innere.

Die nun folgende (ventriculäre) Fläche des Ammonshorns ist mit Epithel überkleidet, und zeigt eine dicke ependymäre Neurogliaanhäufung, [an die sich lockere, mehr radiär verlaufende Fasern anschliefsen (Taf. X, Fig. 1, starke Vergröfserung).

Gehen wir die ventriculäre Seite des Ammonshorns entlang, so kommen wir schliefslich an die Verbindungsstelle des Alveus mit der dorsalen Ventrikelwand (Taf. XIII, Fig. 2 h). Von hier aus erstreckt sich weithin ein Kielstreifen von Neuroglia (Taf. XIII, Fig. 2 i), der seine Entstehung aus der Verschmelzung zweier Oberflächen auch dadurch zu erkennen giebt, dafs in der Nähe des Ventrikels noch Epithel, zuerst in zusammenhängender Lage, dann in unterbrochenen Zügen zu finden ist, bis es schliefslich ganz verschwindet.

Auch dieser Kielstreifen ist mit blofsem Auge zu sehen. Er besteht aus einem dichten Neurogliageflecht und verdünnt sich nach der Tiefe immer mehr, um endlich zu verschwinden. Von seinen beiden Seiten strahlen hauptsächlich schiefe Neurogliafasern ab, deren spitze Winkel sich nach der Ventrikelseite hin öffnen.

Dann kommt, entsprechend der den Kielstreifen umgebenden weifsen Substanz, ein Geflecht von Neurogliafasern, wie es für die Marksubstanz des Grofshirns typisch ist.

---

10. Balken und Fornix.

Der Balken besitzt zwei mit Bezug auf die Neurogliaverhältnisse verschiedene Oberflächen, eine obere epithelfreie und eine untere, teils mit dem Fornix verschmolzene, teils mit Epithel bedeckte. Dieses Epithel gehört ja zur Auskleidung der Seitenventrikel.

Die obere Fläche zeigt eine 0,01—0,03 mm dicke verdichtete Rindenschicht (Taf. XI, Fig. 2 a). An diese schliefst sich nach der Tiefe (Taf. XI, Fig. 2 b) an denjenigen Stellen, wo längsverlaufende Nervenbündel der Oberfläche anliegen, ein Neurogliageflecht an, welches zwar lange nicht die Dichtigkeit einer Rindenschicht besitzt, aber doch eine engere Verflechtung von Fasern aufweist, als die mehr in der Tiefe liegende quer gerichtete Nervenfaserschicht. Die Hauptrichtung der Fasern ist die zur Oberfläche senkrechte, doch finden sich zwischen diesen Hauptfasern andere sie verbindende, die in den beiden zur Oberfläche parallelen Richtungen resp. schief verlaufen. Diese dichtere Schicht, die unterhalb der noch dichteren Rindenschicht liegt, ist etwa 1/3 mm dick und verliert sich nach unten in die Neurogliamassen der queren Markfaserschicht. Die letztere entfernt sich nicht von dem Typus der weifsen Hirnsubstanz, enthält also auch hauptsächlich den Nervenfibrillen parallel verlaufende Fasern mit den üblichen Nebenfasern in anderen Richtungen. (Taf. XI, Fig. 3. Man beachte, dafs diese Zeichnung des Platzes wegen um 90° gedreht ist. Man mufs also die Tafel so halten, dafs der rechte Rand nach unten kommt.)

Die erwähnte dichtere, hauptsächlich aus radiären Fasern zusammengesetzte Neuroglialage unterhalb der Rindenschicht fehlt aber an denjenigen Stellen, wo Ganglienzellenmassen an der Oberfläche des Balkens liegen, oder sie ist erst unterhalb derselben andeutungsweise vorhanden. Die Ganglienzellenmassen sind meines Wissens von Jastrowitz[1] entdeckt worden. Viel später sind sie dann wieder von Golgi[2] beschrieben worden, dem aber die Angaben von Jastrowitz entgangen waren. Auf dem Querschnitt erscheinen diese Ganglienzellenanhäufungen als kuppenförmige Vorsprünge. Bei Anwendung der Neurogliafärbung heben sich diese Stellen schon bei schwächerer Vergröfserung gegen ihre Umgebung ab.

Bei stärkerer Vergröfserung (Taf. XII, Fig. 1) findet man, dafs gleich unter der hier recht dünnen verdichteten Rindenschicht eine an Neurogliafasern arme Partie den Ganglien-

---

[1] Studien über Encephalitis und Myelitis des ersten Kindesalters. Schlufsartikel. Archiv für Psychiatrie. III. 1872. S. 167 f.

[2] Über die feinere Anatomie der Centralorgane des Nervensystems. 1885. Gesammelte Abhandlung. S. 135 ff. und Tafel 28.

zellenanhäufungen entspricht. Die Fasern zwischen den Ganglienzellen bilden ein lockeres Geflecht von hauptsächlich zur Oberfläche senkrechten Fasern. Sie reichen von der Rindenschicht bis zur unterliegenden Markfaserschicht und stehen mit beiden Neurogliageflechten in Verbindung.

Überall findet man im Balken „Astrocyten“. In der tiefen Markmasse liegen aber auch, wie überall, anscheinend quadratische Neurogliazellen, wie schon Jastrowitz angegeben hat, oft in kleinen Längsreihen. Doch strahlen von diesen Längsreihen öfters Fasern nach der Umgebung von den Seitenrändern der gesammten Zellreihe aus. —

Anders als die obere Fläche verhält sich die mit Epithel bekleidete resp. mit dem Fornix verschmolzene untere Partie des Balkens (Taf. XII, Fig. 1). Unter dem Epithel findet sich eine 0,1 bis 0,2 mm dicke, aus sehr eng verflochtenen Neurogliafasern bestehende Schicht, die sich nach oben (also nach der tiefen Nervenfaserlage hin) ohne scharfe Grenze in ein lockeres Geflecht auflöst, das seinerseits ungefähr 0,3 mm dick ist und sich ebenfalls allmählich in das noch lockerere Neuroglialager der tieferen Markmasse verliert. Die Fasern dieser oberhalb der dichten Ependymschicht direkt gelegenen Zone sind wieder in ihrer Hauptrichtung zur Oberfläche senkrecht gestellt und unterscheiden sich so von den Fasern der tieferen weifsen Massen, die ja in ihrer Hauptrichtung zur Oberfläche parallel verlaufen. Dadurch, dafs zwischen den zur Oberfläche senkrechten Fasern der zweiten Schicht quere Nebenfasern verlaufen, entsteht ein sehr zierliches Geflecht.

Das Epithel fehlt auch hier manchmal stellenweise. Dann wuchert die Neuroglia in flachen Wülsten über die freie Oberfläche hervor (Ependymwucherung), doch habe ich so grofse Wucherungen, wie im vierten Ventrikel, nicht gesehen, auch keine schon hyalin degenerierten. —

Die dichte Ependymschicht nimmt an Dicke zu, bis sie da, wo der Fornix sich mit dem Balken verbindet, etwa $1/2$ mm Stärke erreicht (Taf. XI, Fig. 4). Über diese Verbindungsgrenze hinaus, also zwischen Fornix und Balken setzt sie sich dann als dicker kurzer Kielstreifen fort, so dafs also an dieser Verbindungsstelle eine ungemein grofse Menge Neuroglia liegt. —

Der Fornix selbst zeigt an seiner lateralen (den Seitenventrikel begrenzenden) Seite ebenfalls Epithelbelag mit der dazu gehörigen verdichteten Ependymneuroglia. Seine Fasern sind durch eine für eine weifse Substanz recht reichliche Menge Neuroglia von einander getrennt und vielfach zu kleineren durch Randschichten geschiedenen Bündeln angeordnet

(Taf. XI, Fig. 4). Das ist namentlich auch an der medialen Fläche dicht auf der Kielstreifenbildung der Mittellinie zu sehen. —

Die mediale Fläche des Fornix (Ventriculus septi pellucidi) hat kein Epithel und nur eine dünne verdichtete Rindenschicht. Auch hier sind überall „Astrocyten“ zu finden.

II. Opticus und Chiasma.

Dafs im Opticus Neuroglia enthalten ist, wufste schon Virchow. Leber hat hier auch Deitersche Zellen nachgewiesen. Im Jahre 1890 habe ich dann das wesentliche, was mit der neuen Methode von der Neuroglia am Opticus erkannt werden konnte, bereits erwähnt. Seitdem sind verschiedene Mitteilungen nach Untersuchungen mit der Golgischen Methode erschienen, die aber nichts neues zu dem schon bekannten dazugebracht haben. Einer Beschreibung und Abbildung bei Ramón y Cajal[1] mufs jedoch aus dem Grunde gedacht werden, weil namentlich die letztere so vortrefflich ist, wie das die Golgische Methode überhaupt zu erreichen gestattet.

Ich hatte in der erwähnten vorläufigen Mitteilung schon angegeben, dafs der Opticus ein reiches Neurogliageflecht besitzt, das sich an der Oberfläche des ganzen Nerven stärker, an der Oberfläche der einzelnen Bündel schwach verdickt zeigt, mit einem Worte: seiner Neuroglia nach verhält sich der Opticus ganz wie eine in kleinere Bündel abgeteilte, zu einem Gesamtbündel vereinigte weifse Hirnsubstanz. Mehr zu sagen, ist nicht nötig. (Vgl. Taf. VII, Fig. 3 und 4.) —

Zu welchen Irrtümern aber auch hier wieder die Golgische Methode führen kann, wenn es sich um die Beurteilung der Topographie handelt, das geht aus einer Bemerkung Greeffs hervor.[2] Greeff erklärt es nämlich für eine Täuschung, dafs gerade unter der Oberfläche des Sehnerven die Neuroglia am dichtesten wäre. Die Täuschung könnte nur dann herbeigeführt werden, wenn man die Präparate nicht lange genug im Golgischen Gemische liefse, denn dann dringt dies nur in die äufseren Schichten ein. Läfst man sie länger darin, so findet man gerade umgekehrt am Rande nur wenige und schlecht gefärbte Zellen, in der Mitte aber ein dichtes Zell- und Fasergewirr. Dabei giebt Greeff

---

[1] Notas preventivas sobre la retina y gran simpático de los mamiferos. Barcelona 1891.

[2] Die Spinnenzellen — Neurogliazellen — im Sehnerv und der Retina. Archiv für Augenheilkunde. Band 29. S. 11 des Separatabdrucks.

selbst zu, dafs man mit der Golgischen Methode über die Dichtigkeit der Neuroglia nur schwer eine Vorstellung gewinnen kann (S. 10) — aber trotzdem er sich darüber klar war, verfiel er in den Irrtum, die verdichtete Rindenschicht am Opticus für eine Täuschung zu erklären. —

Auch im Chiasma bleiben die Verhältnisse durchaus dem Opticus analog, nur dafs eben hier wegen der Durchflechtungen der Bündel das Bild der Neurogliafasern verwickelter wird, da diese ja in jedem Bündel eine von den anliegenden, sich mit ihm kreuzenden Bündeln verschiedene Hauptrichtung aufweisen müssen.

Am Chiasma ist die laterale und vordere Rindenschicht etwa 0,04 mm dick, hinten aber wird das Chiasma von einer dickeren aus sehr dicht gewebten Fasern bestehenden Schicht überkleidet, die ca. $1/4$ mm stark und daher schon für das blofse Auge als dunkelblauer Streifen kenntlich ist. Diese grofse Dicke und Dichte der Schicht ist einigermafsen auffallend, da ja hier kein epithelbedecktes Ependym vorhanden ist, sondern der Oberfläche nur Bindegewebe aufliegt.

Dies Bindegewebe trennt das Chiasma bis auf den mittelsten Teil vom Trichter, welcher an der dem Chiasma zugekehrten Seite eine etwa ebenso dicke Rindenschicht besitzt, die sich nach der Tiefe zu in zerstreute Fasern auflöst. In der Medianlinie verschmelzen beide Hirnteile, indem das Bindegewebe fortfällt, und da sind denn beide Organe, ohne dafs noch freie Oberflächen vorliegen, durch eine dicke dichte Neurogliaschicht, einen Kielstreifen, von einander getrennt. In den seitlichen Teilen der dichten Neurogliamasse liegen grofse Ganglienzellen eingesprengt.

---

12. Corpora mamillaria.

Die äufsere Oberfläche der Corpora mamillaria zeigt auf ihrer lateralen Seite eine sehr dicke, dichtgewebte Rindenschicht (ca. 0,1 mm). Diese verschmälert sich beim Umbiegen in die mediale Oberfläche der Hügel bis auf 0,02 mm, in welcher ungefähren Dicke sie diese letztere bekleidet. Die dichten Massen lösen sich nach der Tiefe hin in lockrere Geflechte auf, die aber immer noch sehr reichliche Fasern enthalten. Von denen strahlen dann diskrete Fasern in die in der Substanz der Corpora mamillaria liegenden Ganglienzellen und Ganglienzellengruppen nach den verschiedensten Richtungen aus.

Die ependymäre Fläche zeigt wieder eine ungefähr 0,1 mm im Durchmesser haltende subepitheliale Neurogliaverdichtung, die in ihrer grofsen Dicke sehr stark gegen die viel dünnere, nur durch eine schmale Gewebsbrücke von ihr getrennte äufsere Rindenschicht an den medialen Flächen der beiden Hügel absticht. An die ependymären Verdichtungen schliefst sich wieder eine noch etwas dickere Lage locker gewebter, aber immer noch ziemlich reichlicher Fasern an, die nach der Tiefe sich allmählich mehr und mehr verlieren. In den gröfseren Ganglienhaufen des centralen Höhlengraus, die hier liegen, sind die Fasern aber wieder reichlicher, in verschiedener Richtung verlaufend.

Der Fornix zeigt auch in seinem Endteile die Neurogliaverhältnisse der weifsen Substanzen.

---

13. Sehhügel.

Das Studium der Neuroglia der grofsen Centralganglien hat mir grofse Schwierigkeiten gemacht, weil gerade hier die fixierenden und beizenden Flüssigkeiten sehr schwer in die Tiefe dringen. Die Schilderungen der Neurogliaverhältnisse, die in diesem und dem nächsten Abschnitte folgen, bedürfen daher ganz besonders noch ergänzender Studien.

Die Oberfläche des Sehhügels hat dreierlei verschiedene Charaktere:

1. Die Gegend des Plexus chorioideus. Der Plexus chorioideus scheint der Oberfläche des Sehhügels ganz locker aufzuliegen. Hat man aber ganz frische Gehirne, so überzeugt man sich, dafs die Verbindung doch keine gar so lockere ist, man sieht vielmehr von der Unterfläche des Plexus Gefäfschen in die Sehhügeloberfläche eindringen. An dieser Stelle hat nun der Sehhügel keinen Epithelüberzug, oder vielmehr zwischen das Epithel und das Nervengewebe ist echtes Bindegewebe, d. h. der Plexus, eingeschoben, der erst seinerseits auf seiner freien Fläche von Epithel bekleidet ist. Die Verhältnisse sind also ähnlich, wie in den lateralen Teilen des Ventrikelbodens an der Medulla oblongata.
2. Die zweite Abart der Oberflächenbeschaffenheit am Sehhügel ist durch die oberflächlich liegenden markhaltigen Nervenfasern bedingt.
3. Die dritte Art endlich ist die, wo (jenseits des Sulcus Monroi) die grauen Massen zu Tage liegen.

Schon durch diese drei verschieden beschaffenen Oberflächenregionen werden Unterschiede in den ependymären Neurogliamassen bedingt. In der ersterwähnten Gegend, der des Plexus chorioideus, ist eine auffallend dünne (0,01—0,02 mm dicke) verdichtete Oberflächenschicht vorhanden. Man könnte vielleicht annehmen, dafs die Einschiebung des bindegewebigen Plexus die Ursache für diese Dünnheit der eng gewebten Neuroglialage ist. Von der strahlen dann mehr lockere, aber doch noch faserreiche z. T. radiär gerichtete Neurogliamassen in die Tiefe (Taf. XII, Fig. 2a).

Aber auch an den anderen Stellen der Sehhügeloberfläche hat die ependymäre Schicht eine ungemein wechselnde Dicke von 0,025 bis 0,17 mm im Durchmesser schwankend. Für diese Schwankungen weifs ich keine Gründe anzugeben. Da wo etwas gröfsere Gefäfse in der oberflächlichen Schicht liegen, zeigt die ependymäre Verdichtung eine starke Massenzunahme.

Liegen weifse Faserzüge unmittelbar an der Oberfläche, so kann entweder eine abgesetzte Ependymschicht darüber liegen, oder es ist so wie bei den Striae acusticae der Medulla oblongata, d. h. das Epithel liegt direct auf den Nervenbündeln auf. Im letzteren Falle sind dann die Nervenfibrillen von sehr dichten Neurogliamassen durchsetzt, unter denen auch lange Radiärfasern auffallen, während die übrigen in den beiden anderen Richtungen verlaufen. In der dichten Schicht sind dann aber die Maschen für die markhaltigen Nervenfasern ausgespart. In dem Falle, dafs noch eine besondere Neurogliaverdichtung zwischen Epithel und Nervenbündel eingeschoben ist, zeigt das letztere immer noch reichliche Fasern, aber doch nicht so dichte Netze, als wenn das Epithel allein die Grenze gegen den Ventrikel bildet.

An den grauen Stellen der Sehhügeloberfläche ist die Ependymschicht zwar auch von wechselnder Dicke, aber sonst von gewöhnlicher Beschaffenheit (Taf. XII, Fig. 3a). —

Unterhalb der verdichteten Neurogliamassen an der Oberfläche des Sehhügels finden sich dann mehr lockere, aber doch faserreiche Neurogliamassen. Ist die auf diese zweite Zone folgende Schicht ärmer an Neuroglia, so zeigt sich, wie so häufig, die Tendenz der Fasern in radiärer Richtung zu verlaufen (Taf. XII, Fig. 2b), in anderen Fällen aber schliefst sich an die ependymäre oder an die dieser entsprechende interfibrilläre Neurogliamasse ein unregelmäfsiges Geflecht direkt an (Taf. XII, Fig. 3b), also ähnlich wie an den ventrikulären Stillingschen Nervenkernen.

Die Neurogliaverhältnisse in den tieferen Regionen des Thalamus opticus scheinen sehr verschieden zu sein, wie ja auch seine Ganglienzellen zu sehr mannigfachen Gruppen

zusammengestellt sind (Nissl). Gerade diese Verhältnisse bedürfen noch eines weiteren Studiums und setzen eine vorherige genaue Kenntnis der „Sehhügelkerne“ voraus. Ich gebe hier (Taf. XII, Fig. 4) nur die Abbildung einer besonders typischen Geflechtsbildung aus dem Pulvinar. Hier findet man grofse Astrocytenformen, von denen reichliche, aber locker liegende Fasern ausstrahlen, die auch leichte Verdichtungen um die Ganglienzellen erzeugen. Das Bild erinnert sehr an die Neuroglia des roten Kerns, nur schien die letztere mir reichlicher zu sein. Im Sehhügel tritt auch das Netz der Nervenfibrillen nicht deutlich hervor, wie das doch im roten Kern der Fall ist.

14. Streifenhügel und Kapseln.

Der Kopf des Streifenhügels ist mit einer Ependymschicht von sehr wechselnder Dicke bekleidet, die auch hier stellenweise auffallend, dünn ist, — also ganz ähnlich, wie am Sehhügel.

An diese Schicht schliefst sich dann die übliche mehr lockere Fasermasse an mit vielen radiären Fasern. Sonst aber zeigen die tieferen Schichten des Nucleus caudatus und Linsenkerns ganz abweichende Verhältnisse gegenüber dem Sehhügel. Die Neuroglia ist mit Ausnahme der Umgebung etwas gröfserer Gefäfse ungemein sparsam, so sparsam, dafs man wohl sagen kann, Streifenhügel und Linsenkern zeigen Neurogliaverhältnisse, wie sie dem Typus der Grofshirnrinde entsprechen.

Die Kapseln und die weifsen Züge im Corpus striatum etc. sind mit zarten, engen, dem Typus der weifsen Hirnsubstanz entsprechenden Neurogliagerüsten versehen.

9. Abschnitt:

Die physiologische Bedeutung der Neuroglia.

Es wäre sehr interessant, wenn man aus den geschilderten topographischen Verhältnissen der Neuroglia auch ein allgemeines physiologisches Prinzip herauserkennen könnte. Dafs die Neurogliafasern eine Zwischensubstanz darstellen, ist ja zweifellos. Eine Zwischensubstanz hat irgend welche passiven Funktionen. Welche hat nun die Neuroglia?

Das eine ist sicher, dafs die Neuroglia eine raumausfüllende Aufgabe hat. Das beweist vor allem die pathologische Histologie, denn überall da, wo durch Untergang von nervösem Material Platz frei wird, wuchert die Neuroglia und füllt mit ihren Fasern den frei gewordenen Raum aus. Ob dieser Untergang nur die Markscheide betrifft, wie das bei multipler Sklerose der Fall sein soll, oder die ganze Nervenfaser, wie bei der Tabes und bei den sekundären Degenerationen, ob ganze Nervenzellen zu Grunde gehen, wie bei Poliomyelitis anterior, oder Teile derselben, wie bei der progressiven Paralyse, ob das ganze Nervenmaterial (d. h. Zellen und Fasern) zerstört wird, wie bei ischämischen Nekrosen, — immer sehen wir dem Defekt entsprechende geringere oder gröfsere Mengen von Neurogliafasern den frei gewordenen Raum ausfüllen. Ganz besonders möchte ich betonen, dafs entgegen einer früher von mir geäufserten Ansicht, es sich mit der neuen Methode leicht erweisen liefs, dafs auch die festen Narben nach ischämischen Nekrosen nicht Bindegewebe enthalten, sondern aus dichtgewebten, kolossalen Neurogliamassen bestehen.

Aber neben dieser Funktion als Füllmaterial könnten der Neuroglia noch andere Aufgaben obliegen, und hierüber sind in der That schon mancherlei Hypothesen aufgestellt worden. Die bekannteste ist die von Golgi, der den Dendriten der Ganglienzellen eine nutritive Funktion zuschrieb. Diese sollten sie dadurch erfüllen, dafs sie sich mit den Ausläufern der Neurogliazellen in Beziehung setzten, d. h. diese letzteren sollten in irgend einer Weise mit der Ernährung der Ganglienzellen in Verbindung stehen. Wir haben schon

S. 80 ausgeführt, dafs unsere Präparate eine Beziehung zwischen Dendriten und Neurogliafasern nicht zu erkennen erlauben, während es aber immerhin möglich wäre, dafs jemand mit anderen Methoden eine solche Beziehung entdeckte. Hingegen können wir jetzt eins mit Bestimmtheit sagen: welches auch immer diese noch zu erweisenden Beziehungen sein mögen, die Meinung Golgis, dafs die Dendriten gerade deshalb als protoplasmatische Ernährungsfortsätze aufzufassen wären, weil sie sich mit den „Neurogliazellen" in Beziehung setzten, kann nicht richtig sein. Dieser von Golgi angeführte Grund konnte so lange als stichhaltig angesehen werden, als man die „Ausläufer" der Deitersschen Zellen für richtige protoplasmatische Zellfortsätze hielt. Jetzt aber können wir bestimmt sagen, dafs sie keine protoplasmatischen Fortsätze, ja dafs sie überhaupt keine Fortsätze der Zellen sind. Man mufs daher die Idee durchaus fallen lassen, dafs diese Fasern für den Chemismus der Neurogliazellen und erst recht, dafs sie, wenn auch indirekt, für den Chemismus der Ganglienzellen von Bedeutung sein könnten.

Auch die Annahme, dafs die Neurogliafasern wie capillarste (sit venia verbo) seröse Gefäfse auch nur die Ernährungssäfte leiten könnten, müssen wir zurückweisen. Diese Möglichkeit war so lange vorhanden, als man mit Frommann, Lavdowsky u. a. der Meinung sein konnte, dafs die Fasern hohl wären. Auch diese Meinung haben wir aber oben zurückgewiesen (S. 68 sub 2). —

Eine fernere Hypothese über die Bedeutung der Neuroglia ist die von P. Ramón, dem sich auch S. Ramón y Cajal und Sala y Pons angeschlossen haben. P. Ramón und die anderen genannten Forscher mit ihm glauben nämlich, dafs die Neuroglia ganz wesentlich die Aufgabe habe, zur Isolierung der nervösen Leitungen zu dienen, d. h. die Bildung schädlicher Nebenleitungen in den Nervenströmen (corrientes nerviosos) zu verhindern.

Die Gründe für diese Ansicht fafst Sala y Pons in folgender Weise zusammen: „Die Annahme, dafs die Neuroglia eine einfache Stützsubstanz sei, genügt nicht, um die Eigentümlichkeit zu erklären, dafs die Fasern („Zellfortsätze") an manchen Stellen dicht, an anderen weniger dicht sind, ja fast vollkommen fehlen. Sie erklärt auch die Thatsache nicht, dafs die „Zellfortsätze" beim Durchgang durch die eine Region glatt, beim Übergange in eine andere zottig sind, was doch als eine Vermehrung der Oberfläche angesehen werden mufs. Es ist vielmehr wahrscheinlich, dafs die Neuroglia einen anderen Zweck hat, nämlich den, dafs sie die unnützen und schädlichen Kontakte der Nervenelemente verhindern soll.

Daraus erklärt es sich, dafs sie in der weifsen Substanz überall so reichlich ist, während sie in der grauen Substanz da, wo keine Durchgangsfasern existieren, fehlt oder sehr vermindert auftritt. Auch die zottigen Anhänge der Fasern erklären sich so, dafs diese in denjenigen Zonen zu beobachten sind, wo die Kontakte verringert werden sollen, dafs sie aber da fehlen, wo eine derartige Aufgabe nicht zu erfüllen ist, d. h. da, wo die Enden und Collateralen der Axencylinder mit den Körpern und Dendriten der Ganglienzellen in Kontakt treten sollen, und ein Zusammenstofs der Nervenströme erfolgen mufs. Umgekehrt ist die Neuroglia dann in genügender Reichlichkeit vorhanden, wenn solche Übergänge der Nervenströme von einem Gebilde auf das andere verhindert werden sollen.“ [1]

Soweit Sala y Pons. Mit Bezug auf diese Hypothese von P. Ramón müssen wir aber sagen, dafs weder die Thatsachen, auf welche sie sich stützt, richtig sind, noch die theoretische Begründung zutreffend genannt werden kann. Schon die allererste Annahme, dafs die Neuroglia der weifsen Substanz sehr reichlich sei gegenüber der der grauen, stimmt absolut nicht. Wir haben im Gegenteil gesehen, dafs die reichlichsten Neurogliamassen gerade in gewissen grauen Substanzen zu finden sind. Es stimmt auch ferner gar nicht, dafs in den grauen Substanzen diejenigen Stellen arm an Neuroglia sind, wo zahlreiche Kontakte von Dendriten und Axencylindern statthaben. In den Oliven, den Vierhügeln, den Stillingschen Nervenkernen etc. sind doch gewifs reichliche derartige Kontakte zu konstatieren, — und doch ist an diesen Partieen ein ungemein reiches Neurogliagerüst vorhanden, viel reichlicher, als in den weifsen Substanzen. Endlich stimmt es nicht, was wir schon früher (S. 79 sub 6) erörtert haben, dafs diejenigen grauen Massen, welche viele durchgehende markhaltige Fasern enthalten, auch eine gröfsere Masse von Neuroglia besitzen sollten.

In alle diese thatsächlichen Irrtümer sind die berühmten spanischen Forscher nur deshalb geraten. weil sie sich für ihre Neurogliauntersuchungen nur der Golgischen Methode bedienen konnten, deren Unzuverläfsigkeit für die Beurteilung topographischer Verhältnisse wir jetzt wohl genügend kennen gelernt haben.

Aber nicht blofs die thatsächlichen Verhältnisse, auf welche sich die Hypothese von P. Ramón stützt, treffen nicht zu, auch gegen die theoretische Begründung läfst sich mancherlei einwenden. Gerade in den weifsen Substanzen erscheint eine Isolierung durch

[1] Sala y Pons, La Neuroglia de los Vertebrados. Madrid 1894. S. 40.

Neuroglia ganz überflüssig, denn hier sind ja die Axencylinder von dicken, isolierenden Markscheiden so wie so umgeben. Es bedürfte also einer weiteren isolierenden Schicht, wie sie die Neuroglia darstellen soll, durchaus nicht.

Viel eher müfste man umgekehrt erwarten, dafs in den grauen Substanzen gerade an denjenigen Stellen, wo die Enden der Axencylinder und Collateralen mit den Dendriten in Kontakt treten, eine isolierende Substanz erwünscht wäre.

Diese Kontakte dürfen doch auch die corrientes nerviosos nicht in regelloser Weise von einer Bahn auf die andere überleiten. Es darf nicht irgend ein beliebiger Nervenast mit einem beliebigen Dendriten, den er im Vorbeiziehen trifft, oder mit einem anderen Nervenast der Nachbarschaft in leitende Verbindung treten. Viel eher müfsten hier Vorrichtungen getroffen sein, die den Nervenströmen nur die ihnen vorgeschriebene Bahn zu beschreiten erlauben und die alle Nebenbahnen ausschalten, für welche doch bei der reichen Verzweigung und Durchflechtung der Dendriten und Axencylinder so sehr viel Gelegenheit gegeben ist. Gerade hier soll nun keine isolierende Schicht nötig sein, — und in der weifsen Substanz mit ihren viel einfacheren Bahnen sollen die dicken Markscheiden nicht genügen!

Wenn wir ferner bedenken, dafs gerade im Gegensatz zu den Markscheiden die Neurogliafasern nur Geflechte, aber nirgends festgeschlossene Massen darstellen, wie sie eine isolierende Schicht doch erforderte, so werden wir wohl nicht umhin können, die Hypothese von P. Ramón fallen zu lassen, — höchstens könnte man für diejenigen Stellen eine isolierende Wirksamkeit der Neuroglia annehmen, wo diese zusammenhängende, von nervösen Elementen freie, oder fast freie, Schichten bildet. Das würde z. B. an den äufseren und inneren Oberflächen denkbar sein.[1]

Um Mifsversändnisse zu vermeiden, sei aber doch noch speziell darauf hingewiesen, dafs zur Isolation der Dendriten und Axencylinder unter einander, von deren präsumtiven Notwendigkeit wir oben sprachen, nicht etwa eine wirkliche „Grundsubstanz“ oder eine, noch zu entdeckende, andere Neuroglia absolut nötig wäre. Vielleicht genügt es schon, dafs die feinen Reiserchen in Gewebsflüssigkeit baden, die ja in ähnlicher Weise zur Iso-

---

[1] Als Curiosum sei noch mitgeteilt, dafs Schleich den Schlaf auf eine „Reizung der Neuroglia“ zurückführt. (Schmerzlose Operationen Berlin 1894. S. 78 ff.) Eine gereizte Intercellularsubstanz ist jedenfalls etwas sehr merkwürdiges. Schleich giebt S. 89 eine Abbildung der Neuroglia in der Hirnrinde. Man sieht da ein reiches Gespinnst von Neurogliafasern um eine Ganglienzelle herum, aber dieses Gespinnst ist eben — ein Hirngespinnst.

lierung dienen könnte, wie das Öl in den Transformatoren hochgespannter Ströme der Technik, wobei man freilich voraussetzen mufs, dafs bei den so minimalen Stromspannungen, wie sie in den Dendriten etc. herrschen, die Gewebsflüssigkeit als Isolator genügt, — wer kann aber wissen, wie die Natur sich hilft? —

Wenn wir der Neuroglia eine raumausfüllende Aufgabe zuschreiben, so erscheint gerade das, was die spanischen Forscher zu ihrer Hypothese veranlafst hat, garnicht so wunderbar. Ihnen war die wechselnde Menge der Neuroglia im Inneren der Organe das merkwürdige und einer speziellen Erklärung bedürftige. Aber man kann sich sehr leicht vorstellen, dafs in den verschiedenen Regionen des Centralnervensystems die nervösen Elemente bald so dicht nebeneinander liegen, dafs für eine andere Substanz, die Neuroglia, kein Raum vorhanden ist, bald so locker, dafs Zwischenräume bleiben, die dann je nach deren Gröfse von mehr oder weniger reichlicher „Bindesubstanz“ ausgefüllt werden. So könnten in den tiefen Schichten der Grofshirnrinde die Durchflechtungen der Dendriten und Axencylinder so dichte sein, dafs hier keine Neuroglia Platz hätte, während umgekehrt in den Oliven die Dendriten und Nervenfasern lockerer gefügt wären, und daher Raum genug übrig bliebe, der dann von der Neuroglia ausgefüllt werden müfste. Also die wechselnde Menge der Neuroglia innerhalb der Organe könnte man sich ganz gut ohne Zuhilfenahme der doch nicht haltbaren Isolationshypothese erklären.

Sehr merkwürdig und einer weiteren Erklärung dringend bedürftig ist vielmehr etwas anderes, nämlich nicht die Menge im Inneren der nervösen Teile, sondern die ungemein wechselnde und dabei doch typische Anordnung der Neuroglia in den verschiedenen Teilen des Centralnervensystems, sowie die wechselnde Menge an den Oberflächen der Organe, wo die Neuroglia doch nicht als einfaches Füllmaterial angesehen werden kann. Hierbei sind es besonders die häufig wiederkehrenden Typen, die einem unwillkürlich die Idee aufdrängen, dafs die Raumausfüllung, die der Neuroglia unter physiologischen und pathologischen Verhältnissen obliegt, nicht in regelloser Weise vor sich geht. Es müssen auch hier irgend welche statischen Gesetze die verschiedenen Geflechtsformen beherrschen, in ähnlicher Weise, wie das für andere Bindesubstanzen längst nachgewiesen ist: für die Anordnung der normalen Knochenbälkchen durch Culmann, Hermann v. Meyer u. a,, für die pathologischen Knochenverhältnisse durch Julius Wolff, für die Fasern in der Delphinflosse, ja für die Verzweigungen der Blutgefäfse durch Wilhelm Roux etc.

Durch solche mechanischen oder statischen Gesetze müssen vor allem die dichten Geflechte an den inneren und äufseren Oberflächen bedingt sein. Für die Gefäfse speziell hat bereits Lloyd Andriezen darauf aufmerksam gemacht,[1] dafs hier die oft so starke Gliahülle die Aufgabe hat, die Hirnsubstanz gegen die „undue expansions“ der Gefäfse zu schützen. Die Hirngefäfse haben nur eine schwache Adventitia und sind überhaupt sehr dünnwandig, so dafs ihre eigene Wand keinen genügenden Widerstand für den schwankenden Blutdruck gewähren würde. Sie bedürfen daher dringend einer Unterstützung durch die Neurogliascheide. Lloyd Andriezen macht ferner darauf aufmerksam, dafs diese, wenn auch dichte, Schutzwehr einen maschigen Bau besitzt, so dafs die Saftströmungen in das Blut hinein und aus ihm heraus in keiner Weise gehindert werden.

Als eine ähnliche Schutzvorrichtung gegen irgend welche, noch unbekannte mechanische Einflüsse könnte man sich auch die anderen Neurogliaverdichtungen an den Oberflächen erklären, — denn dafs die Verdichtungen um die Gefäfse herum zu den Oberflächenverdichtungen zu rechnen sind, das haben wir ja S. 76 f. besprochen. Aber damit ist die Sache noch nicht abgethan.

Die typische, so oft wiederkehrende Anordnung bedarf zunächst der Erklärung. Wir haben ja S. 77 darauf aufmerksam gemacht, dafs sowohl die Verdichtung der Glia um die Gefäfse, als die an den äufseren Oberflächen auch in der Anordnung Ähnlichkeiten aufweist. Beide zeigen ein hauptsächlich aus mehr oder weniger transversalen Fasern geflochtenes, besonders dichtes Maschenwerk, und an dieses sich anschliefsend ein weniger dichtes, aber doch faserreiches Geflecht von vornehmlich radiären Faserzügen — das kann nicht zufällig sein. Auch der Wechsel in der Beschaffenheit dieser Oberflächenverdichtungen (im weitesten Sinne) mufs eine Bedeutung haben. Warum ist die Ependymschicht an den Centralganglien so verschieden dick? Warum besitzt die laterale Seite der Corpora candicantia eine so starke, die mediale eine so schwache Rindenschicht? Warum fehlt diese an der Oberfläche des Kleinhirns ganz etc. etc.? Warum fehlen die Radiärfasern an vielen grauen Substanzen unter der Ependymschicht, während sie an anderen grauen doch vorhanden sind und an den weifsen so regelmäfsig auftreten? Warum ist über markhaltigen Nervenfasern manchmal eine abgesetzte Ependym- oder äufsere Rindenschicht vorhanden, manchmal aber nur eine interfibrilläre? Was sind hier

---

[1] On a system of fibre-cells surrounding the blood-vessels of the brain of Man and Mammals. Internationale Monatsschrift für Anatomie und Physiologie. 1893. S. 539.

und an anderen Stellen für geheimnisvolle Ansprüche an Druck-, Zug- und Scheerfestigkeit gestellt, dafs solche typischen Trajectorien entstehen?

So liefsen sich der Fragen noch viele aufstellen, und noch mehr werden sich ergeben, wenn die Topographie der Neuroglia noch besser studiert sein wird.

Wir sehen eben wieder, entsprechend dem, was wir in den einleitenden Worten zu dieser Arbeit gesagt haben, dafs sich an die Beantwortung der Frage nach den topographischen Verhältnissen der Neurogliafasern, wie an die Beantwortung jeder naturwissenschaftlichen Frage die Aufstellung immer neuer, vorher ungeahnter Fragen anschliefst, dafs jedes „darum“ gar viele „warum?“ gebiert, — und das wird wohl auch hier in unendlicher Kette weitergehen. —

10. Abschnitt:
Methode.

In den Schlufsbemerkungen zu unserer historischen Übersicht haben wir schon der hauptsächlichsten Ansprüche Erwähnung gethan, die an eine Methode zur Färbung der Neuroglia zu stellen sind. Wir müssen aber hier etwas genauer auf dasjenige eingehen, was wir von einer brauchbaren Methode verlangen müssen.

1. Das erste Erfordernis ist das, dafs die Färbung eine elective ist, d. h. dafs sich nichts mitfärbt, was mit Neurogliafasern verwechselt werden kann, oder was das deutliche Hervortreten der Fasern hindert.

Es ist höchst interessant zu sehen, wie im Laufe der Zeit die Ansprüche in dieser Hinsicht allmählich gestiegen sind. Ich erinnere mich noch an mein bewunderndes Staunen, als ich in meinen ersten Studiensemestern im Berliner physiologischen Institut die, wenn ich mich recht erinnere, nach Gollschen Abbildungen gezeichneten Rückenmarkstafeln betrachtete. In diesen Bildern war alles rot gefärbt mit Ausnahme der Markscheiden, und doch wurde schon diese technische Leistung Golls für ein Meisterwerk gehalten. Ähnliche Bilder mufs auch Kölliker noch für die Figuren in der 4. Auflage seiner Gewebelehre vor Augen gehabt haben, denn auch da zeigen die Zwischenräume zwischen den markhaltigen Nervenfasern des Rückenmarks dieselben diffusen, undifferenzierten Massen, wie sie auf jenen Tafeln zu sehen waren.

Man war also damals schon zufrieden, wenn man die Markscheiden ungefärbt und alles andere in roten, womöglich verschieden abgestuften Tönen vor sich hatte.

Als zweites Entwicklungsstadium ist das anzusehen, dafs man die in der weifsen Substanz des Rückenmarks vorhandenen Neurogliafasern so mit Carmin färbte, dafs die in ihren Maschenräumen befindliche Substanz (Gewebsflüssigkeit?), welche in den Gollschen und Köllikerschen Präparaten noch mitgefärbt gewesen war, von der Färbung ausgeschlossen, oder doch sehr blafs tingiert wurde. Eine solche Färbung zu bekommen, war grofsenteils

Glücksache, denn auf die damals üblichen Carminlösungen war gar kein Verlafs, wie ich mich aus meiner eigenen Jugend erinnere. In dieser Weise immerhin schon distincter gefärbte Präparate mufs Frommann erhalten haben.

Jetzt konnte man etwas erkennen, was früher zu erkennen nicht möglich gewesen war, nämlich dafs die für Neuroglia angesprochenen Bestandteile einen faserigen Charakter hätten, und Frommann nannte sie daher auch stets „Fasern", obgleich er der Meinung war, dafs es eigentlich Zellausläufer wären.

Als man soweit war, konnte man wenigstens in der weifsen Rückenmarkssubstanz und an ähnlich günstig beschaffenen Stellen die Neurogliafaserung studieren. Wie wir jetzt wissen, und wie schon Boll vermutet hatte, sind aber auch diese Bilder selbst für die bestgeeignete Stelle, d. h. für die weifse Rückenmarkssubstanz, keine sicheren gewesen, da auch hier Axencylindercollateralen verlaufen, von deren Existenz man damals noch keine rechte Ahnung hatte, — und die Axencylinder werden auch bei dieser besseren Carminfärbung mittingiert.

In den weniger günstig beschaffenen Partieen nun gar, ganz besonders in den grauen Massen, war die Unsicherheit eine so grofse, dafs sie selbst bei sehr bescheidenen Ansprüchen unbequem wurde, und so klagen denn alle Autoren, von Clarke und Frommann bis auf Petrone und Lavdovsky, über die Unsicherheit in der Beurteilung dessen, was man bei Carmin- und ähnlichen Methoden zur Neuroglia rechnen soll. —

Jetzt müssen wir von einer Neurogliamethode verlangen, dafs sie weder die Markscheiden, noch die (präsumptive) Gewebsflüssigkeit, noch die Axencylinder, noch die Dendriten der Ganglienzellen färbt. Alle Methoden, bei denen eine Axencylinder- und Ganglienzellenfärbung nicht mit Sicherheit ausgeschlossen werden kann, sind ohne weiteres zu verwerfen.

Wie gefährlich Methoden, bei denen sich Axencylinder mitfärben, namentlich für den pathologischen Anatomen sind, das zeigt ein Beispiel aus neuester Zeit. Popoff[1] hat aus dem Flechsigschen Laboratorium eine vorläufige Mitteilung veröffentlicht, in der er über Resultate seiner Untersuchungen bei disseminierter Sklerose berichtet. Er wandte bei diesen Untersuchungen eine dreifache Färbung an, in der wieder das „patentsaure Rubin", richtig genannt: „Patent-Säurerubin" (alias Säurefuchsin) eine Rolle spielt. Hierbei behauptet

[1] Zur Histologie der disseminierten Sklerose. Neurologisches Centralblatt, 1894. S. 321.

er nun folgendes gefunden zu haben: „Ferner kann ich auf Grund meiner Untersuchungen nicht mit der allgemein herrschenden Meinung einverstanden sein, dafs es sich um Wucherungen des Bindegewebes handle. Meine mikroscopischen Präparate zeigen deutlich, dafs dasjenige, was die meisten Beobachter für zwischen den Nervenfasern liegende Bindegewebszüge hielten, nur Veränderungsprodukte der Nervenfasern selbst sind“ (S. 322). Namentlich soll es sich hier um gewucherte und veränderte Axencylinder handeln.

Von der Unrichtigkeit dieser so ungemein paradoxen Behauptung kann man sich — ganz abgesehen von den Resultaten der älteren Beobachtungen — durch unsere Färbung auf das schlagendste überzeugen. Schon bei ganz akut verlaufenden Fällen von multipler Sklerose, erst recht bei chronischen Formen, überzeugt man sich geradezu handgreiflich, dafs es sich hier in der That um ganz kolossale Wucherungen von „Bindegewebe“, soll heifsen von Neuroglia, handelt. Eine Methode, die Irrtümer ermöglicht, wie die sind, in die Popoff geraten ist, ist unter allen Umständen absolut unbrauchbar. —

Dafs auch die Ganglienzellen und ihre Protoplasmaausläufer ungefärbt bleiben müssen, ist ohne weiteres klar. Nicht nur, dafs die Dendriten eventuell auch einmal mit Neurogliafasern verwechselt werden können, so liegt vor allem bei den Methoden, welche die Nervenzellen in demselben, oder in einem ähnlichen Tone färben, wie die Neuroglia, der grofse Nachteil vor, dafs sich die feinen Neurogliafasern nicht genügend von dem reichen Geflechte der Dendriten abheben, und dafs sie daher der sicheren Kenntnisnahme entgehen. Wenn man das berücksichtigt, und wenn man bedenkt, dafs die Leiber der Deitersschen Zellen sich im allgemeinen sogar schwerer färben, als die Ganglienzellen, so wird man sagen können: alle die Methoden, welche die Leiber der Deitersschen Zellen in demselben Farbentone färben, wie die Neurogliafasern, d. h. alle Methoden, bei denen die Fasern als wirkliche Ausläufer der genannten Zellen erscheinen, sind für das topographische Studium der Neuroglia nicht zu verwerten. Wenig brauchbar sind auch diejenigen Methoden, welche nur einen leichten Unterschied in der Intensität der Farbe zwischen Zellleib und Faser ergeben. —

Im allgemeinen weniger wichtig ist es, dafs die anzuwendende Methode das Bindegewebe nicht mitfärbt. Einmal ist das doch auch eine nichtnervöse Substanz, eine Zwischenmasse, wie die Neuroglia, dann aber ist die Structur des Bindegewebes so verschieden von der Neurogliastructur, dafs Verwechslungen kaum zu befürchten sind.

Hatte doch schon der Entdecker der Neuroglia, Virchow, mit seinen primitiven Methoden den Unterschied zwischen Neuroglia und echtem Bindegewebe erkannt.

Unter Umständen kann es aber, namentlich für den pathologischen Anatomen, doch erwünscht sein, das Bindegewebe ungefärbt zu bekommen, und so soll man wenigstens die Möglichkeit haben, die collagenen Massen von der Färbung auszuschliefsen. Was die elastischen Fasern betrifft, so liegt nicht die geringste Schwierigkeit vor, ihre Färbung zu verhindern. Sie färben sich, im Gegensatz zum collagenen Gewebe, überhaupt nur mit ganz eigenartigen Methoden. —

In der Erfüllung aller bis jetzt besprochenen Forderungen genügt unsere neue Methode allen Ansprüchen.

---

2. Ein zweites wichtiges Erfordernis ist die Sicherheit der Methode, d. h. jedes regelrecht hergestellte Präparat sollte an jeder Stelle jede einzelne hier vorhandene Neurogliafaser zeigen. Diese Forderung ist für den normalen Anatomen weniger wichtig, als für den pathologischen. Wenn der normale Anatom an irgend einem Präparat auch nur eine einzige Stelle vollständig gefärbt bekommt, so kann er sich damit zufrieden geben. Dann weifs er eben, wie an dieser Stelle das Neurogliageflecht immer beschaffen ist. Der pathologische Anatom mufs anspruchsvollor sein aus Gründen, die ich früher einmal entwickelt habe.[1]

Im vollen Sinne des Wortes habe ich die hier besprochene Forderung trotz langjähriger Bemühung noch nicht erfüllt. Es passiert mir doch noch, dafs im Inneren der Stücke leere Flecke zum Vorschein kommen, wo Neurogliageflechte da sein müfsten, — aber ziemlich sicher ist die Methode doch.

Wie gering man aber auch seine Ansprüche an die Sicherheit einer Methode stellen mag, eins wird man unter allen Umständen verlangen können, nämlich das, dafs der Erfolg der Methode nicht auf der Schneide eines sehr kurzen Zeitabschnittes bei irgend einer der dabei vorkommenden Prozeduren steht. Wenn z. B. ein Forscher angiebt, dafs eine Sekunde mehr oder weniger über den Erfolg der Färbung entscheidet, so wird man eine solche Färbung verwerfen müssen.

---

[1] Merkel und Bonnets Ergebnisse der Anatomie und Entwicklungsgeschichte. 1894. 3 Bd. S. 19f.

3. Sehr wünschenswert ist es weiterhin, dafs bei einer Neurogliafärbung auch die anderen Elemente, wenigstens soweit es zur Orientierung nötig ist, erkennbar gemacht werden. Vor allem ist es zu erstreben, dafs man die Kerne sieht, absolut nötig ist das für pathologische Prozesse. Die Kerne können auch ohne jede Inconvenienz in demselben Farbentone gefärbt sein, wie die Neurogliafasern. Kein Mensch wird einen Kern mit einer Neurogliafaser verwechseln, und die Klarheit der Bilder wird durch die Anwesenheit der Kerne in keiner Weise beeinträchtigt; eher ist das Gegenteil der Fall. Diese Forderung war sehr leicht zu erfüllen.

Mehr Schwierigkeiten machte es, die nervösen Elemente wenigstens so weit sichtbar zu machen, dafs man in den Präparaten die Orientierung nicht verliert. Höhere Ansprüche zu stellen war nicht nötig, aber durchaus erforderlich war es, dafs die nervösen Elemente in einer Kontrastfarbe, also nicht in einem ähnlichen Farbentone, wie die Neurogliafasern, gefärbt waren, aus Gründen, die oben sub 1 entwickelt worden sind.

Die Schwierigkeit war deshalb eine so grofse, weil sämtliche von mir durchprobierte Farbstoffe nicht mit Sicherheit eine Schädigung der Neurogliafärbung vermeiden liefsen. Ich bin dann schliefslich auf einen anderen Stoff gekommen, der nicht nur die Neurogliafärbung nicht schädigt, sondern sogar die Intensität der Farbe erhöht. Man erhält ja dabei keine Bilder, wie sie etwa die Golgimethode für die Ganglienzellen liefert, aber man will ja auch keine Ganglienzellen studieren, sondern nur ihre Lage erkennen. Nebenbei stellte es sich heraus, dafs wenigstens die gröberen Nisslschen Körnungen sehr hübsch hervortraten. Das war schon mehr, als eigentlich nötig war, aber es war doch sehr gut mitzunehmen.

4. Eine grofse Unbequemlichkeit war es für mich eine lange Zeit, dafs die Fasern zwar gefärbt waren, aber so blafs, dafs sie mit schwacher Vergröfserung kaum als Fasern zu erkennen waren. Ich erstrebte daher eine gröfsere Prägnanz der Färbung, und für bescheidene Ansprüche ist diese auch erreicht. Man mufs nur nicht gleich verlangen, dafs die Fasern so schwarz erscheinen sollen, wie bei der Golgischen Methode, es genügt schon, wenn man bei schwachen Vergröfserungen einen guten Überblick über die Geflechte bekommt. Man kann sie ja dann immer noch mit starken Vergröfserungen im Détail studieren.

5. Ein von den pathologischen Anatomen und besonders von den Klinikern seit lange empfundener Mifsstand ist der, dafs die Präparate für die übliche Härtung in doppeltchrom-

sauren Salzen so ungeheuer lange Zeit brauchen. Es ist ja richtig, dafs bei histologischen Methoden das „tuto“ bei weitem dem „cito et jucunde“ vorangeht, aber alles hat seine Grenzen. Wenn man Monate lang warten soll, bis ein Präparat genügend gehärtet und gebeizt ist, so ist das eine Zumutung, die man nur dann ertragen kann, wenn auf keine andere Weise das „tuto“ zu erreichen ist. Schon vor langer Zeit habe ich versucht, diesem Mifsstand abzuhelfen. Zunächst zog ich die Erlickische Flüssigkeit aus ihrer absoluten Vergessenheit hervor, — aber sie dringt zu ungleichmäfsig ein, um brauchbare Resultate zu liefern. Dann versuchte ich es mit der Wärme, aber auch dabei vergingen noch Wochen, und man hatte es nicht in der Gewalt, die Präparate vor Brüchigkeit zu schützen. Wir werden sehen, dafs man jetzt schon in vier Tagen die Präparate zur Markscheidenfärbung ohne Anwendung von Wärme vorbereiten kann. Solche Präparate könnte man auch zur Neurogliafärbung benutzen, doch ist für diese eine andere nur wenig längere Zeit beanspruchende Härtung zu empfehlen.

6. Sehr viel Schwierigkeiten machte es mir auch eine lange Zeit, dafs die Härtungen und die weiteren Behandlungen die Stücke zum Schrumpfen brachten, brüchig werden liefsen, oder dergl.

Ich mufste ganze Prozeduren deshalb aufgeben und neue suchen, denn es ist durchaus erforderlich, dafs die mit den Präparaten vorzunehmenden Manipulationen diese nicht schädigen. Auch dieses Ziel ist zu meiner Zufriedenheit erreicht.

7. Wünschenswert, wenn auch nicht gerade absolut nötig, war es schliefslich auch, den Präparaten Dauerhaftigkeit zu verleihen. Meine ersten Präparate haben sich recht gut gehalten, sie sehen jetzt nach fünf, sechs Jahren noch sehr schön aus. Als ich dann aber dle Methode nach den anderen, wichtigeren Gesichtspunkten umarbeitete, hatte ich sehr unter dem Verblassen der Präparate zu leiden. Sie hielten sich kaum 8—14 Tage in gutem Zustande. Die jetzigen Präparate scheinen sich zu halten, aber eine Garantie für die lange Dauer der Haltbarkeit kann ich nicht übernehmen.

Wie bei allen empirisch gefundenen Methoden, so hat es auch bei der Neurogliamethode zunächst grofse Schwierigkeiten gemacht, hinter das Prinzip der Methode zu kommen, und doch war es zu deren Vervollkommnung durchaus nötig, über dieses Prinzip klar zu werden.

Noch im Jahre 1890 war ich auf falschen Wegen. Ich glaubte damals, wie ich es auch in meiner vorläufigen Mitteilung veröffentlicht habe, „dafs die Präparate mit Metallsalzen gebeizt werden müfsten, die eine organische Säure enthalten.“ Ich mühte mich noch lange nachher mit Versuchen ab, die passende organische Säure und das passende Metallsalz zu finden, bis ich denn endlich dahinter kam, dafs Metallsalz und organische Säure in einem ganz anderen Verhältnis, als in dem einer einfachen Verbindung, zu einander stehen müfsten. Das Metallsalz mufste in einer hochoxydierten Verbindung den Präparaten einverleibt werden, und die organischen Säuren, die ich mit Erfolg benutzt hatte, spielten nur die Rolle eines Reduktionsmittels.

Wenn wir dieses empirisch gefundene Prinzip uns theoretisch zurecht legen wollen, so kann das vielleicht in folgender Weise geschehen: Der Farbstoff haftet nur an der Neuroglia, wenn diese eine stark reduzierte Metallverbindung enthält. Eine solche stark reduzierte Metallverbindung läfst sich aber direkt nicht an die Neuroglia befestigen. An dieser haftet das Metall nur in hoch oxydiertem Zustande, oder eventuell, wie wir sehen werden, in einer Mischung höherer und höchster Oxydationsstufen. Man mufs daher, um jene Färbungsmöglichkeit zu erreichen, zunächst das Metall in höher oxydiertem Zustande der Neuroglia zuführen und dann erst die starke Reduktion vornehmen.

Das ist freilich nur eine Hypothese. Es wäre ja auch denkbar, dafs die Metallverbindung nur eine Veränderung der Neuroglia selbst bewirkte. Mir schien aber die erste Hypothese wahrscheinlicher, weil wir auch sonst aus der technischen Färberei wissen, dafs an sehr feinen Niederschlägen basische Anilinfarben besser haften (z. B. das Methylgrün an sehr fein verteiltem Schwefel). —

Nachdem ich über das Prinzip der Färbung ins klare gekommen war, variierte ich die Metallverbindungen und Reduktionen in der mannigfaltigsten Weise, in der Hoffnung, doch schliefslich eine sichere, elective Färbung zu erzielen. Über eine gewisse Grenze kam ich aber nicht heraus, bis ich endlich nach vielen Irrgängen fand, dafs der Fehler ganz wo anders lag: nämlich im allerersten Teile der Operationen, die mit den Präparaten aus dem Centralnervensystem vorgenommen werden müssen.

Ich wufste zwar schon von Anfang an (vgl. meine Mitteilung aus dem Jahre 1890), dafs man nur ganz frisches Material „von guter Consistenz“ benutzen dürfte, aber ich glaubte, dafs die üblichen Härtungsmethoden dieses Material auch ganz sicher fixierten, um so mehr, als ja für die Markscheidenfärbung diese sichere Fixierung nachgewiesen war. Als

nun gar das Formol aufkam, das die Präparate des Centralnervensystems so schnell fixierte, war mir der Gedanke ganz fern, dafs ein frisch eingelegtes, in der üblichen Weise zerschnittenes Hirn oder Rüchenmark nicht durchaus gut konserviert sein sollte. Aber schliefslich fand ich, dafs die Neuroglia in dieser Beziehung ungemein empfindlich war.

Wenn die Härtungsflüssigkeit nicht binnen 24 Stunden das Präparat vollkommen durchdrungen und fixiert hat, sind die inneren Teile für die Neurogliafärbung ungeeignet geworden, resp. überhaupt alle Teile, in die die Flüssigkeit nicht gleich eingedrungen ist.

Wir haben hier also einen Unterschied gegen die einfach kadaveröse Erweichung zu konstatieren. Bei dieser zerfällt die Neuroglia zwar in Körnchen, aber bleibt doch noch eine ganze Zeit färbbar, bei der Zersetzung innerhalb der Härtungsflüssigkeiten verliert sie aber von vornherein ihre Färbbarkeit. Es ist wohl anzunehmen, dafs das Wasser der Härtungsflüssigkeiten diesen Unterschied bedingt. Leider aber konnte man den Übelstand nicht dadurch vermeiden, dafs man absoluten Alkohol verwendete. Alkohol ist vielmehr für ungebeizte Präparate, was die Neuroglia anbelangt, sehr schädlich. Es nützt auch nichts, wenn man etwa im Alkohol Metallverbindungen auflöst. Man erhält immer höchst unsichere, oft ungemein mangelhafte Neurogliafärbungen. Wir haben uns schliefslich in einer sehr einfachen, aber freilich auch sehr unbequemen Weise geholfen (unten sub 1a). — —

Nach alledem zerfallen also die für die Neurogliafärbung nötigen prinzipiellen Mafsnahmen in 3—4 Teile: 1a. Fixierung der dem Centralnervensystem entnommenen Stücke, 1b. Beizung mit höher oxydierten Metallverbindungen. Diese beiden Akte können eventuell zu einem vereinigt werden. 2. Reduktion der Metallverbindung. 3. Färbung.

1. Fixierung und Beizung.

a) Diese beiden Prozeduren kann man, wie gesagt, getrennt oder vereint vornehmen. Man trennt sie, wenn man sich die Möglichkeit offen halten will, die Präparate auch nach anderen Methoden, als gerade nach unserer neuen zu behandeln, z. B. nach der Marchischen, der Golgischen, der Nisslschen oder der Markscheidenmethode. In diesem Falle fixiert man die Stücke mit Formol (1:10). Man hüte sich vor schwächeren Lösungen; diese fixieren nicht gut genug. Stärkere anzuwenden, hat aber auch keinen Zweck, sie leisten auch nicht mehr. Will man aber eine ordentliche Neurogliafärbung erzielen, so ist es durchaus nötig, das Material in möglichst kleine, nicht über einen halben Centimeter dicke Stücke zu zer-

schneiden und so in die Fixierungsflüssigkeit hineinzuthun. Dafs das Material ganz frisch, d. h. nicht kadaverös erweicht sein darf, ist selbstverständlich.

Gröfsere Stücke mögen durch das Formol schliefslich noch so hart geworden sein, für eine sichere Neurogliafärbung taugen sie nichts mehr.

Dieser Zwang, so kleine Stücke einzulegen, hat etwas sehr unangenehmes, ich habe aber vorläufig noch kein Mittel gefunden, um ihn zu umgehen.

Zur Härtung bedient man sich grofser flacher, mit Deckel versehener Schalen, z. B. solcher, wie sie in der Bacteriologie zur Aufbewahrung von Plattenkulturen verwendet werden. Auf den Boden legt man in üblicher Weise Fliefspapier. So vermeidet man am besten die Verkrümmungen der dünnen Stücke. Nach dem ersten Tage mufs man die Formollösung wechseln, später ist es nicht mehr nötig. Sind die Stücke hart geworden (etwa nach vier Tagen) und weitere Verkrümmungen nicht mehr zu befürchten, so kann man die Präparate in hohe, weniger platzraubende Gläser hineinthun. Sie halten sich dann Jahr und Tag noch färbungsfähig.

b) Die Beizung kann man an den mit Formol gehärteten Stücken gerade so gut vornehmen, wie an frischen. Ich habe das schon in meinem Artikel „Technik“ in den Merkel-Bonnetschen Ergebnissen der Anatomie und Entwicklungsgeschichte 1894 mitgeteilt. Man kann aber, wie erwähnt, auch Fixierung und Beizung verbinden. Diese Verbindung von Fixierung und Beizung ist eigentlich die seit langer Zeit für das Centralnervensystem gebräuchliche Methode. Alle Härtungen in Bichromat haben ja den Zweck, gleichzeitig eine Beizung vorzunehmen. Mit Chromaten gebeizte Stücke lassen auch eine Neurogliafärbung zu, wenn die Beizung und Härtung nicht etwa in der hier und da noch gebräuchlichen, ursprünglichen Müllerschen Flüssigkeit ($2^1/_2$ % Kaliumbichromat mit oder ohne 1 % Glaubersalz) stattgefunden hat. In so dünnen Lösungen geht die Färbbarkeit der Neuroglia ganz verloren. Hingegen zeigen Stücke, die in der (jetzt wohl meist benutzten) gesättigten (ca. fünfprozentigen) Lösung von doppeltchromsaurem Kalium gehärtet werden, wenn man die Stücke genügend klein eingelegt hat, bei passender Behandlung die Neuroglia sehr gut gefärbt, aber ich bin von der Chromhärtung doch ganz zurückgekommen, weil man da nie sicher ist, dafs

sich nicht auch Axencylinder mitfärben. Das ist ein so fundamentaler Fehler, dafs ich auf die Chromhärtung, wie auf so viele andere von mir aufgegebene Methoden, garnicht eingehen würde, wenn ich nicht bei meinen Versuchen etwas gefunden hätte, was für die Markscheidenfärbung von grofsem Nutzen ist.

Es ist mir nämlich gelungen, die Zeit, die zur gehörigen Härtung und Beizung der Präparate für die Markscheidenfärbung nötig ist, ganz wesentlich abzukürzen, und zwar auf 4—5 Tage.

Durch theoretische Überlegungen habe ich herausgefunden, dafs die Verbindung der Markscheiden mit dem Chromat, welche für die Bildung des Farblacks nötig ist, dann ungemein rasch vor sich geht, wenn man einer starken Bichromatlösung ein Chromoxydsalz in passender Menge zusetzt. Zu wenig darf man von letzterem nicht verwenden, weil sonst die Härtung und Beizung zu langsam erfolgt, zu viel deshalb nicht, weil dann die Flüssigkeit zu schwer eindringt, und weil die Präparate zu rasch brüchig werden.

Welches Bichromat man benutzt, ist gleichgültig, man kann Kalium, Natrium oder Ammonium bichromicum nehmen. Natrium bichromicum löst sich am leichtesten und ist am billigsten. Auch die Wahl des Chromoxydsalzes ist ziemlich frei, man kann essigsaures, oxalsaures Chromoxyd oder irgend ein anderes in der Technik gebräuchliches verwenden; aber am meisten möchte ich den sehr billigen, leicht in krystallisierter Form zu beschaffenden Chromalaun (schwefelsaures Chromoxydkalium) empfehlen.

Die Lösung besteht also aus: 5 % Kalium (Natrium oder Ammonium) bichromicum und 2 % Chromalaun in Wasser. Man löst durch Kochen. Sollten sich beim Erkalten Niederschläge bilden, so giefst oder filtriert man ab, denn sonst bilden diese Niederschläge einen feinen Schlamm um die Stücke, der das Eindringen der Flüssigkeit erschwert.

Auch in diese Mischung dürfen nicht zu dicke Stücke eingelegt werden, da sonst die Lösung nicht rasch genug durchdringt. Die Beizung und Härtung mufs vielmehr in 4—5 Tagen vollendet sein. Man kann die Stücke zwar auch bis 8 Tage in der Chromalaunbichromatlösung lassen, aber nicht länger, sonst werden sie brüchig. Dann werden sie mit Wasser ordentlich abgespült und in üblicher Weise mit Alkohol nachbehandelt.

Bei dieser Methode hat die Notwendigkeit, dünnere Stücke anzuwenden, nichts unbequemes. Man kann nämlich die Präparate in gröfseren Stücken vorher in Formol härten.

Aus so gehärteten Massen sind die dünneren Scheiben mit Leichtigkeit herauszuschneiden, ohne dafs man ein Verkrümmen derselben zu befürchten hat.

Man kann aber auch die Härtung direkt in jener Mischung vornehmen (natürlich an kleinen Stücken), nur thut man gut, dann der Lösung noch 10 % Formol zuzusetzen.

Für die Neurogliafärbung benutze ich aber solche in Bichromat gehärtete Stücke nicht mehr, sondern für diese findet eine andere Beizung statt, die ich vorläufig als die typische Neurogliabeize empfehlen möchte. Es ist eine Kupferbeize, bei der (wie bei der Kupferung zum Zwecke der Markscheidenfärbung) das neutrale essigsaure Kupferoxyd den Hauptbestandteil bildet. Es kam aber darauf an, eine Mischung herzustellen, die einmal die bei der gewöhnlichen wässerigen Lösung des genannten Kupfersalzes so störenden Niederschläge vermeidet, und die andererseits gut an der Neuroglia haftet. Man kann dieses Ziel auf verschiedene Weise erreichen.

Ich gebe hier nur eine Mischung an, die sich mir recht gut bewährt hat: sie besteht aus 5 % essigsaurem Kupferoxyd, 5 % gewöhnlicher Essigsäure und 2½ % Chromalaun in Wasser. Bei ihrer Bereitung müssen aber einige Vorsichtsmafsregeln befolgt werden. Würde man nämlich zu einer kalt bereiteten Chromalaunlösung Kupfer und Essigsäure zusetzen, oder umgekehrt, so würde man einen voluminösen grünlichen Niederschlag erhalten. Ganz anders ist es, wenn man das Chromalaun in Wasser kocht und nachher mit Kupfer und Essigsäure zusammen bringt: dann entsteht dieser Niederschlag nicht. Ich erkläre mir dies so, dafs die grüne Modifikation, welche das Chromalaun beim Kochen mit Wasser bildet, sich der essigsauren Kupferlösung gegenüber anders verhält, als die violette, die bei der Lösung auf kaltem Wege entsteht. Es ist aber wohlgemerkt nötig, dafs man die Chromalaunlösung richtig zum Kochen bringt, nicht etwa blofs erwärmt, denn nur so wird alles violette Salz in grünes übergeführt.

Man kocht daher erst das Chromalaun mit Wasser (in einem emaillierten Deckeltopfe). Wenn es im vollen Kochen ist, dreht man die Flamme aus, fügt hierauf zuerst die Essigsäure dazu, und dann das feingepulverte neutrale essigsaure Kupferoxyd. Man rührt nun fleifsig um, bis man mit dem Glasstabe fühlt, dafs das Kupfersalz sich bis auf einen kleinen Rest gelöst hat. Dann läfst man erkalten. Die Flüssigkeit bleibt immer klar.

Diese Lösung ist auch für die Markscheidenfärbung zu empfehlen, da sie an den chromierten Stücken keine Niederschläge macht, und

andererseits gegenüber der Seignettesalzlösung den Vorteil darbietet, dafs eine weitere Kupferung mit einfach-wässeriger Lösung des Kupfersalzes überflüssig ist.

In die essigsaure Kupferoxyd-Chromalaunlösung kommen die Stücke, wenn man sie vorher (mindestens 4 Tage) in Formol gehärtet hat, 4—5 Tage lang bei Brütofentemperatur, oder bei Zimmertemperatur wenigstens 8 Tage. Interessiert einen aber weiter keine andere Färbung, als die der Neuroglia, so ist es besser, die frischen, nicht über 1/2 cm dicken Stücke mit Umgehung des einfachen Formols direkt in jene Kupferchromalaunlösung zu bringen, der man aber dann 10% Formol zusetzen mufs. Den zweiten Tag wechselt man, später ist ein Wechseln hin und wieder vielleicht erwünscht, aber nicht nötig.

Zur Markscheidenfärbung eignen sich diese nicht gechromten Stücke ebensowenig, wie die mit blofsem essigsaurem Kupferoxyd behandelten, was ich im Gegensatz zu van Gieson bemerken möchte. Der Farbenüberschufs geht in der Differenzierungsflüssigkeit viel zu schnell und zu ungleichmäfsig aus den Schnitten heraus.

Auch die direkt in die Kupferchromalaun-Formol-Lösung eingelegten Stücke verweilen (und zwar bei Zimmertemperatur) mindestens 8 Tage in der Flüssigkeit. Längerer Aufenthalt schadet nichts, die Stücke werden nie brüchig.

Die zum Schneiden bestimmten Stücke werden mit Wasser abgespült, in gewöhnlicher Weise in Alkohol entwässert und mit Celloidin durchtränkt.

2. Reduktion. Die Reduktion der chromierten Präparate erfolgt für die Neurogliafärbung in anderer Weise, als die der gekupferten. Da aber die bei Chrompräparaten erzielten Neurogliafärbungen den Ansprüchen, die man stellen mufs, vorläufig nicht genügen, so verzichte ich darauf, auf die hierbei möglichen Reduktionsverfahren einzugehen. Aber für andere Zwecke mufs doch ein solches erwähnt werden.

Von vielen Seiten, namentlich von Seiten der Augenärzte, ist es nämlich als ein Mifsstand empfunden worden, dafs an Chrompräparaten die Färbung des Fibrins und der Mikroorganismen nach dem von mir angegebenen Verfahren nicht gelingt. Um dieses aber doch zu ermöglichen, ist es nur erforderlich, die Schnitte aus solchen Präparaten in reduzierende Flüssigkeiten zu bringen. Es genügt schon, wenn man die Schnitte einige Zeit, am besten einige Stunden, in 5%iger Oxalsäure liegen läfst. Dann gelingt die Fibrinfärbung etc. auch an Präparaten, die in Kaliumbichromat ge-

härtet sind. Für Neurogliafärbungen ist dies Verfahren ungenügend. Wir wollen daher jetzt die Reduktion der gekupferten Schnitte besprechen.

Die Reduktion der gekupferten Schnitte erfolgt sehr leicht, aber, wenn man die feineren Fasern einigermafsen sicher gefärbt haben und die Schnitte nicht brüchig werden lassen will, so verringert sich die Zahl der möglichen Reduktionsverfahren. Die für photographische Zwecke empfohlenen so mannigfaltigen Reduktionsmittel, die ich alle durchprobiert habe, sind z. B. ungeeignet. Das gilt ganz besonders für die in alcalischer Lösung anzuwendenden, da diese die Schnitte schädigen. Andere Reduktionsmittel sind wieder zu schwach, die Reduktion mufs vielmehr eine sehr energische sein. Als bestes Verfahren empfiehlt sich die in der Technik schon lange gebräuchliche, aber erst von Lustgarten in die Histologie eingeführte Reduktion durch Behandlung mit Kalium hypermanganicum und schwefliger Säure. Lustgarten hat diese Reduktion im Leipziger pathologischen Institute (selbständig) 1884 zuerst angewendet. Er brachte sie nach Wien, und hier ist sie dann von Pal (ganz wenig verändert) zu einer Modifikation meiner Markscheidenfärbung benutzt worden. Man kann die Lustgartensche Methode direkt verwenden. Besser aber wirkt noch eine kleine Modifikation derselben, bei der ein Stoff in Anwendung kommt, der als Contrastfarbe und als Verstärker von Nutzen ist.

Dieser Stoff ist unter dem Namen „Chromogen“ von den Höchster Farbwerken in die Technik eingeführt und mir, wie so vieles andere, in liebenswürdigster Weise zur Disposition gestellt worden, wofür ich hiermit meinen besten Dank ausspreche. In der Technik wird dieser Stoff, der selbst kein Farbstoff ist, zu Färbungszwecken benutzt, für uns aber leistet er in anderer Beziehung Dienste.

Chromogen ist eine Naphthalinverbindung, nämlich das saure Natronsalz der 3—6 Disulfosäure des 1—8 Dioxynaphthalins, also:

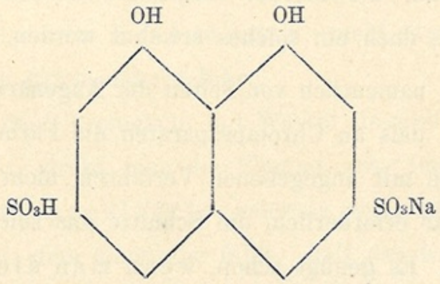

Die Lösung reagiert sauer und wirkt reduzierend, indem dabei die hydrochinonartige Verbindung in eine chinonartige übergeht. Die Reduktion von Seiten der einfach-wässrigen

Lösung ist aber nicht kräftig genug, um die feinsten Fasern färbbar zu machen, man mufs daher die Reduktionsfähigkeit derselben nach dem Prinzip der von Lustgarten in die Histologie eingeführten Methode wesentlich verstärken.

Zu diesem Zwecke löst man 5 % Chromogen und 5 % Ameisensäure (die von mir benutzte hatte ein spezifisches Gewicht von 1,20) in Wasser. Man filtriert sorgfältig. Vor dem Gebrauche setzt man zu 90 CC dieser Flüssigkeit 10 CC einer 10 %igen Lösung von dem in der Photographie gebräuchlichen Natriumsulfit (einfach schwefligsaurem Natron) hinzu.

Man bringt die Schnitte zunächst auf etwa 10 Minuten in eine ca. ⅓ prozentige Lösung von Kalium hypermanganicum, wäscht sie nach vorsichtigem Abgiefsen dieser Lösung durch Aufschütten von Wasser aus, giefst auch dieses Wasser ab und thut dann die besprochene Reduktionsflüssigkeit zu den Schnitten hinzu. Schon nach wenigen Minuten sind die vorher durch das übermangansaure Kalium gebräunten Schnitte entfärbt, aber man läfst sie doch zweckmäfsiger noch 2—4 Stunden in der Lösung.

Wenn man jetzt die Schnitte in der bald zu erwähnenden Weise färbt, so sind die Neurogliafasern blau, das Bindegewebe aber ist farblos. Unter Umständen ist es ja erwünscht, das Bindegewebe farblos zu bekommen, dann kann man jetzt die Vorbereitung für das Färben abschliefsen.

Für gewöhnlich kommt es aber auf eine Farblosigkeit des Bindegewebes nicht an (vgl. S. 130 f), und für diese Fälle thut man gut, der eigentlichen Reduktion noch eine Prozedur folgen zu lassen, bei der freilich das collagene Gewebe blau mit einem Stich ins Violette wird. Aber diese folgende Prozedur hat einmal den grofsen Vorteil, dafs durch sie die Neurogliafasern viel dunkler werden, und auch die feinsten deutlich hervortreten, und sodann den, dafs in der früher angedeuteten Weise (S. 132 sub 3) die Ganglienzellen, die Ependymzellen und die gröberen Axencylinder einen gelblichen Ton annehmen. Man lasse sich daher die kleine Mühe und den kleinen Zeitverlust nicht verdriefsen.

Diese weitere Operation besteht darin, dafs die Schnitte nach Abgiefsen der Reduktionsflüssigkeit und nach zweimaligem Aufgiefsen von Wasser in eine einfache (also nicht mit Säure versetzte) gesättigte wässerige Chromogenlösung kommen. Diese bereitet man sich durch Auflösen von 5 % Chromogen in Aqua destillata. Man filtriere sorgfältig.

In dieser Lösung bleiben die Schnitte über Nacht. Je länger man sie darin läfst, desto mehr werden die nervösen Elemente in der Kontrastfarbe tingiert. Dann giefst man

wieder zweimal Wasser auf, und nun sind die Schnitte färbbar. — Es kann aber oft vorkommen, dafs man die Färbung der Schnitte nicht bald vornehmen kann. Würde man die Schnitte lange in Wasser lassen, so würde ihre Färbbarkeit bald schwächer werden. Auch reiner (natürlich wegen des Celloidins verdünnter) Alkohol ist nicht sicher, wohl aber Alkohol mit Oxalsäurezusatz (90 CC 80%iger Alkohol mit 10 CC 5%iger Oxalsäurelösung). In dieser Alkoholmischung können die Schnitte tagelang liegen bleiben, ohne die Färbbarkeit zu verlieren, so dafs man die Färbung vornehmen kann, wenn man gerade dazu Zeit hat. Durch die Alkoholbehandlung scheinen die Schnitte nach der Färbung auch haltbarer zu werden.

3. Färbung. In der ersten Zeit glaubte ich die Sicherheit der Neurogliafärbung durch Modifikation der verschiedenen Prozeduren bei der (von mir von Anfang an verwendeten) Fibrinmethode erzwingen zu können. Es stellte sich aber heraus, dafs nur höchst geringfügige Modifikationen dieser Methode nötig sind, und dafs durch weitere Veränderungen eine Sicherheit in der Färbung nicht zu erzielen ist, dafs es vielmehr wesentlich auf die sub 1 und 2 besprochene Behandlung der Präparate ankommt.

Die Fibrinmethode kann ich wohl als allgemein bekannt voraussetzen. Ich gebe daher nur die kleinen Abweichungen von meinem ursprünglichen Verfahren an und füge noch einige Bemerkungen über Dinge hinzu, die nach meinen Erfahrungen nicht immer genügend beachtet werden.

Zur Fibrinfärbung nach meinem Verfahren sind drei Lösungen nötig: 1. eine Methylviolettlösung, 2. eine Jodjodkaliumlösung, 3. eine Anilinölxylolmischung. Die zweite dieser Lösungen ist dem Gramschen Verfahren zur Färbung von Microorganismen entlehnt, die dritte ist von mir erfunden.

Da aber nach dem ursprünglichen Gramschen Verfahren, d. h. bei Anwendung des Alkohols statt des Anilinölxylols, eine Fibrinfärbung nie erfolgt, so ist für das Fibrin durch die Einführung dieser Mischung eine neue Methode entstanden. Den Bacterien gegenüber gewinnt die Methylviolett-Jod-Methode durch das Anilinölxylol nur an Sicherheit. Für die Bacterien ist demnach die Fibrinmethode nur als Modifikation der Gramschen zu betrachten.

Für die Neurogliafärbung bleibt die Jodjodkaliumlösung unverändert (gesättigte Lösung von Jod in fünfprozentiger Jodkaliumlösung). Hingegen sind die anderen Flüssigkeiten ein wenig zu modifizieren. Statt der wässerigen Methylviolettlösung benutzt man eine (heifsgesättigte und nach dem Erkalten von dem Bodensatz abgegossene) alkoholische

Lösung (70—80 % Alkohol). Dieser Lösung setzt man auf je 100 CC 5 CC einer fünfprozentigen wässerigen Oxalsäurelösung zu. Dieser Zusatz ist zwar für die Färbung selbst nicht nötig, aber die Präparate scheinen sich bei Anwesenheit einer geringen Oxalsäuremenge besser zu halten. Anilinöl setzt man aber der alkoholischen Methylviolettlösung nicht zu.

Die Anilinölxylollösung ist nicht im Verhältnis von 2 Anilinöl zu 1 Xylol anzuwenden, wie bei der typischen Fibrinmethode, sondern von beiden Stoffen werden gleiche Raumteile miteinander gemischt.

Im übrigen ist das Verfahren bei der Neurogliafärbung ganz dem der Fibrinfärbung entsprechend. Die Schnitte dürfen also nicht gar zu dick sein, d. h. nicht dicker als 0,02 mm. Schnitte von dieser Dicke sind ja mit Leichtigkeit anzufertigen. Die Färbung erfolgt, wie alle folgenden Prozeduren, auf dem Objektträger. Man beachte dabei, dafs die Schnitte dem Glase faltenlos aufliegen müssen. Damit dies mühelos erreicht wird, ist es nötig, die Schnitte in eine grofse Schale mit Wasser zu bringen und sie dann mit einem Objektträger aufzufangen, den man vorher mit Alkohol abgerieben hat. An so gereinigten Objektträgern adhärieren die Schnitte im allgemeinen ohne Faltenbildung. Sollten sich doch Falten vorfinden, so tauche man den Objektträger auf der Kante stehend so in die Schale mit Wasser, dafs die Falte wagrecht steht. Dann gleicht die Falte sich von selbst aus.

Die Farbflüssigkeit wird auf den (abgetrockneten) Schnitt aufgeträufelt. Die Färbung erfolgt fast momentan. Es schadet nichts, es nützt aber auch nichts, wenn man die Lösung länger auf dem Schnitte stehen läfst.

Auch die Jodjodkaliumlösung wird auf den (gefärbten und abgetrockneten) Schnitt aufgeträufelt und gleich wieder abgegossen. Auch hier nützt eine längere Einwirkung des Jods nichts. Bei sehr langer Berührung mit der Jodlösung wird die Färbung eher schlechter, als besser.

Bei der Auswaschung mit Anilinölxylol geniere man sich nicht, recht gründlich zu verfahren. Erst nach viertel- oder halbstündiger Einwirkung dieser Lösung findet ein Abblassen der feineren Fasern statt.

Das Anilinölxylol mufs vor dem Einlegen der Schnitte in Balsam sehr sorgfältig mit reinem Xylol mehrmals abgewaschen werden, sonst halten sich die Präparate nicht. Die Neuroglia ist darin empfindlicher, wie das Fibrin, was mir erst sehr spät klar geworden ist.

Sehr merkwürdig ist es auch, dafs die Schnitte sich besser halten, wenn man sie nicht gleich ins Dunkle bringt, sondern erst 2—5 Tage im diffusen Tageslicht offen liegen läfst.

Zum Schlufs noch eine Bemerkung über das Abtrocknen der Schnitte mit Fliefspapier. Hierfür ist nicht jede Sorte Fliefspapier geeignet, vor allem taugen die Papiere nichts, die eine gekörnte Oberfläche haben. Wir wenden seit Jahren das Filtrierpapier No. 1116 der Firma Ferdinand Flinsch, Grofser Kornmarkt 12 in Frankfurt a. M., an.

Man beachte auch, dafs man die Fliefspapierbäusche auf dem Schnitt nicht verschiebt, sonst zerreifsen die Präparate. Man halte daher mit zwei Fingern der linken Hand den Papierbausch recht fest an dem (hier leeren) Teile des Objektträgers angedrückt.

Kurz zusammengefafst hätten wir also bei der neuen Methode, wenn es sich allein um die Neurogliafärbung handelt, folgende Prozeduren vorzunehmen:

1. Fixierung und Beizung in essigsaurer Kupferoxydchromalaunlösung mit Zusatz von Formol: 8 Tage.
2. Vorbereitung der Schnitte zum Schneiden (Celloidinmethode): 3 Tage.
3. Anfertigung der Schnitte.
4. Reduktion durch Kalium hypermanganicum und durch Chromogenlösung plus schwefliger Säure.
5. Verstärkung der Färbbarkeit für die Neuroglia und Kontrastfärbung der nervösen Elemente durch einfach-wässerige Chromogenlösung.
6. (Modifizierte) Fibrinmethode.

3—6 dauern zusammen einen Tag. Gesamtzeitraum 12 Tage.

Wie ich z. T. schon 1890 mitgeteilt habe, kann man eine im Prinzip ganz ähnliche Färbungsmethode noch für viele andere Gewebselemente benutzen: zur Darstellung der Gallencapillaren, der cuticularen Substanzen an den Nierenepithelien und sonstigen Epithelzellen, sur Färbung der Milzstructuren, der doppeltlichtbrechenden Substanz der quergestreiften (und glatten) Muskeln, oder (mit einer Modifikation) zur Darstellung der Zwischenscheiben in den Muskelfasern etc. Präparate mit derartigen Färbungen haben seit Jahren viele Kollegen bei mir gesehen.

Über alle diese Dinge behalte ich mir weitere Mitteilungen vor.

Den Abschlufs der neuen Methode, den ich jetzt erreicht habe, kann ich nur als einen vorläufigen ansehen. Wenn man die Unbequemlichkeit bei der Härtung mit in den Kauf nimmt, so sind zwar die von uns aufgestellten Forderungen teils vollständig, teils so erfüllt, dafs die Methode wenigstens brauchbar ist. Aber einer sehr wichtigen Forderung, der der Sicherheit ist im idealen Sinne noch nicht Genüge geleistet. Ehe aber die Methode nicht eine geradezu mathematische Sicherheit besitzt, ist sie nicht als vollendet zu bezeichnen.

Dazu kommt noch ein Fehler, der freilich für den pathologischen Anatomen und den „menschlichen Histologen“ nicht ins Gewicht fällt: **die Neurogliafärbung geht bisher nur am menschlichen Centralnervensystem gut anzuwenden. Für Tiere ist sie noch nicht zu empfehlen.** Kaninchengehirne wenigstens zeigen die Neuroglia immer nur andeutungsweise und nicht recht electiv gefärbt. Woran das liegt, weifs ich noch nicht. Hoffentlich gelingt es mir mit der Zeit, diesen und die anderen Mängel zu beseitigen.

Nachdem aber jetzt gezeigt ist, dafs eine elective und vollständige Färbung der Neuroglia wenigstens mit einiger Sicherheit zu erreichen ist, so werden vielleicht andere, frische Kräfte, die nicht durch einen langjährigen, engbegrenzten Gedankenkreis gehemmt sind, auf ganz neuen Wegen eine vollkommene Methode zu Stande bringen. Nach meinen bisherigen Erfahrungen werden aber auch manche versuchen, auf meinen eigenen Pfaden weiter zu wandeln und die von mir eröffneten Wege zu verbessern. Ich bin auch fest überzeugt, dafs, wie bei meinen früher veröffentlichten Methoden, so auch bei dieser für solche Leute, d. h. für die Herren Modifikanten, eine reiche Ernte zu erwarten ist.

Figurenerklärung.

Vorbemerkung.

Fast alle Figuren sind mit Hilfe des Abbeschen Zeichenapparates, ein grofser Teil mit dem neuen Modell (No. 44a) und auf dem Bernhardschen Zeichentisch gezeichnet. Die gröberen Formen, die nicht gar zu gedrängt stehenden Fasern, und alle Kerne wurden genau Strich für Strich nachgezogen. Bei dichteren Fasermassen war das nicht möglich, und bei diesen ist daher nur der Charakter des Geflechts so gut wie möglich wiedergegeben. Auch die ganz feinen Fasern erschienen im Zeichenapparate zu verschwommen, um sie direkt mit der Feder oder dem Bleistift verfolgen zu können.

Der neue Zeichentisch (Zeiss, No. 105a des Katalogs von 1895) ist bekanntlich verstellbar. Bei No. 90 der Skala liegt er mit dem Fufse des Mikroscops in gleicher Höhe, bei No. 45 mit dem Mikroscoptische, bei No. 0 steht er auf seiner höchstmöglichen Stelle über dem Tische.

Alle Zeichnungen, mit Ausnahme der Figuren 2, 4 und 5 auf Tafel XIII, sind mit Zeissschen Apochromaten gemacht und zwar von diesen wieder alle, mit Ausnahme von Figur 3 auf Tafel X, mit der Homogenimmersion 3 mm, 1,30 Apertur. Figur 3 auf Tafel X ist mit Apochr. 8 mm gezeichnet.

Die meisten Figuren sind ferner mit einer Stellung des Zeichentisches auf 0, einige mit der auf 45, Figur 1 Tafel I und Figur 3 Tafel X mit der auf 90 gemacht.

Am Schlufs der Tafel XIII finden sich die Zeichnungen eines Objectivmicrometers für alle die hier in Anwendung gekommenen Vergröfserungen und für die verschiedenen Stellungen des Zeichentisches abgebildet, (je 5 Hundertelmillimeter) nämlich:

Fig. 6:	Homogenimmersion,	Zeichentisch auf 90,	Vergröfserung	A.
„ 7:	„	„ „ 45,	„	B.
„ 8:	„	„ „ 0,	„	C.
„ 9:	Apochromat 8 mm,	„ „ 90,	„	D.

Die Vergröfserung A ist nur bei Figur 1 Tafel I in Anwendung gekommen, die Vergröfserung D nur bei Figur 3 Tafel X.

Die Ganglienzellen und die sicheren Ependymzellen sind, ihrem Aussehen in den Präparaten entsprechend, überall gelb gezeichnet, die Axencylinder nur in einigen Figuren teilweise als gelbe Punkte besonders markiert.

Tafel I.

Fig. 1. Astrocytenformen. Vergröſserung A.
„ 2. Querschnitt durch die weiſse Substanz des Rückenmarks. Seitenstrang, Randpartie mit Pia. Vergröſserung C.
„ 3. Schräger Vertikalschnitt durch die weiſse Substanz des Rückenmarks. Vergröſserung C. Rechts Pia, dann die Rindenschicht mit Büschelbildung nach der Pia hin. In der Mitte und links mehrere Stammfortsätze.
„ 4. Eintritt der vorderen Wurzel. Vergröſserung C. Vertikalschnitt. Oben vordere Wurzel. a Randschicht derselben, unten mehrere Gefäſse.
„ 5. Substantia gelatinosa Rolando. Vergröſserung C.

Tafel II.

Fig. 1. Randpartie aus dem Vorderhorn eines neugeborenen Kindes. Vergröſserung C. Bei a eine Ganglienzelle mit Nisslscher Körnung. Unten weiſse Substanz.
„ 2. Dasselbe vom Erwachsenen. Vergröſserung C. Unten sind ausnahmsweise die Axencylinder markiert.
„ 3. Vorderer Teil der Substantia spongiosa des Hinterhorns. Vergröſserung C. Oben die Grenze gegen die Substantia gelatinosa Rolando.
„ 4. Lissauersche Randzone (unten b) und Substantia spongiosa (a) des Hinterhorns. Vergröſserung C.

Tafel III.

Fig. 1. Clarkesche Säule. Vergröſserung B.
„ 2. Centralkanal mit vorderer Commissur vom Kinde. Vergröſserung B. (Axencylinder angedeutet.)
„ 3. Centralkanal mit hinterer Commissur vom Kinde. Vergröſserung B. Unten Vertikalfasern (a).

Auf dieser Tafel sind die Kerne nicht mitgezeichnet.

Tafel IV.

Fig. 1. Fötales Ependymepithel mit Flimmerhaaren. Darunter Randstreifen mit Punkten. Vergröſserung B.
„ 2. Centralkanal im höheren Alter. Vergröſserung C. a, b zwei neugebildete Lumina, c unregelmäſsiger Epithelhaufen.
„ 3. Desgleichen. Vergröſserung C. a einfaches neues Lumen.
„ 4. Desgleichen. Vergröſserung C. Unregelmäſsiger Epithelhaufen.
„ 5. Desgleichen aus dem unteren Teile der Medulla oblongata. Vergröſserung B. a neugebildetes Lumen, b unregelmäſsige Epithelmasse, von Neurogliafasern durchsetzt.

Tafel V.

Fig. 1. Ependymwucherung im 4. Ventrikel. Vergröſserung B.
„ 2. Cystenähnlicher Raum zwischen zwei benachbarten Ependymwucherungen. Vergröſserung B.
„ 3. Pyramidenkreuzung. Vergröſserung C. a verdichtete Randschichten.
„ 4. Hypoglossuskern, vom Ependym entfernterer Teil. Vergröſserung B.

Tafel VI.

Fig. 1. Raphe der Medulla oblongata mit Nachbarschaft. Vergröfserung C.
„ 2. Fibrae arciformes externae und Pyramidenkern. Vergröfserung C.
„ 3. Olive. Vergröfserung C. Unten Markschicht.
„ 4. Nucleus gracilis. Vergröfserung C.

Tafel VII.

Fig. 1. Nucleus ambiguus Vergröfserung C.
„ 2. Striae acusticae. Vergröfserung C. Bei a cystenähnliche Räume.
„ 3. Opticus, Längsschnitt. Vergröfserung C.
„ 4. Opticus, Querschnitt. Vergröfserung C. Oben die verdichtete äufsere Rindenschicht. a Randschicht eines Bündels.

Tafel VIII.

Fig. 1. Hirnschenkel. Substantia nigra. Vergröfserung C.
„ 2. Gliahülle um einen Gefäfsranm aus dem Hirnschenkel. Vergröfserung C. Rechts der Gefäfsraum. Das Gefäfs selbst ist nicht mitgezeichnet.
„ 3. Oculomotoriuskern. Vergröfserung C.
„ 4. Vierhügel. Abschnitt aus dem inneren Teile. Vergröfserung C.

Tafel IX.

Fig. 1. Grofshirnrinde. Schläfenlappen. Vergröfserung C.· a Rindenschicht. b Radiärfaserschicht.
„ 2. Weifse Substanz des Grofshirns. Vergröfserung C.
„ 3. Weifse Substanz des Kleinhirns. Vergröfserung C. In der Mitte ein Gefäfs. Am unteren Rande desselben quergetroffene Fasern.
„ 4. Purkinjesche Zellen von einer alten Frau. Vergröfserung B. Unten Beginn der Körnerschicht.
„ 5. Oberflächlicher Teil der Molecularschicht. Vergröfserung B. Pia mater unten.

Tafel X.

Fig. 1. Ependymäre Oberfläche des Ammonshorns. Vergröfserung C. Links Ependymschicht, rechts Radiärfaserschicht.
„ 2. Gyrus dentatus. Vergröfserung C. Unten piale Oberfläche. Diese Figur ist aus zwei Zeichnungen, dem oberen und dem unteren Teil der Abbildung entsprechend, zusammengesetzt. Die Zeichnungen pafsten vortrefflich aneinander. Ihre Grenze markiert sich in der Figur durch die Einschnürung der Mitte.
„ 3. Ansatz der Fimbria. Vergröfserung D.

Tafel XI.

Fig. 1. Ventrale (ependymäre) Oberfläche des Balkens. Vergröſserung C. Unten Ependym.
„ 2. Dorsale Fläche des Balkens. Vergröſserung C. a Rindenschicht. b dichte Markschicht.
„ 3. Tiefe Markschicht des Balkens. Vergröſserung C. Die Figur muſs um 90° gedreht werden, wenn ihre Lagerung der der anderen Figuren dieser Tafel entsprechen soll.
„ 4. Ansatz des Fornix an den Balken. Vergröſserung C. Fornix unten.

Tafel XII.

Fig. 1. Ganglienzellenhaufen an der dorsalen Balkenoberfläche. Vergröſserung C.
„ 2. Sehhügeloberfläche vom Plexus chorioideus bedeckt. Vergröſserung C. a Rindenschicht. b Radiärfaserschicht.
„ 3. Sehhügeloberfläche mit Epithel bekleidet. Vergröſserung C. a Ependymschicht. b Ganglienzellenschicht.
„ 4. Aus der Tiefe des Pulvinar. Vergröſserung C.

Tafel XIII.

Fig. 1. Fimbria mit Plexus chorioideus. Vergröſserung C. a Piale Oberfläche. b Ependym. c Plexus chorioideus. d Epithelhöcker.
„ 2. Ammonshorn. Vergröſserung $3^1/_2$ fach Erklärung im Text.
„ 3. Zirbeldrüse. Vergröſserung C.
„ 4. Vierhügel. Natürliche Gröſse.
„ 5. Sehhügel, hintere Commissur, Zirbeldrüse. Natürliche Gröſse.
„ 6—9. Maſsstäbe für die Figuren. Erklärung S. 146.

Berichtigungen.

Seite 9 Zeile 1 von unten lies 1867 statt 1877.

Seite 22 Zeile 3 von unten lies 1882 statt 1892.

Seite 24 Zeile 14 von unten lies 1885—1886 statt 1886—1886.

Seite 62 Zeile 9 von oben lies „darstellen“ statt „darsellen“.

Seite 63 Zeile 1 von oben, Seite 65 Zeile 3, 6 und 15 von oben lies „Schaper“ statt „Schrader“.

Seite 78 Zeile 5 von oben lies „spärliche“ statt „spärlich“.

Seite 89 Zeile 12 von oben lies „spärlicher“ statt „spärlich“.

Seite 89 Zeile 15 von oben ist hinter „Rolando“ einzuschalten: (Taf. I Fig. 5).

Druck von Aug. Weisbrod, Frankfurt a. M.

Fig. 2.

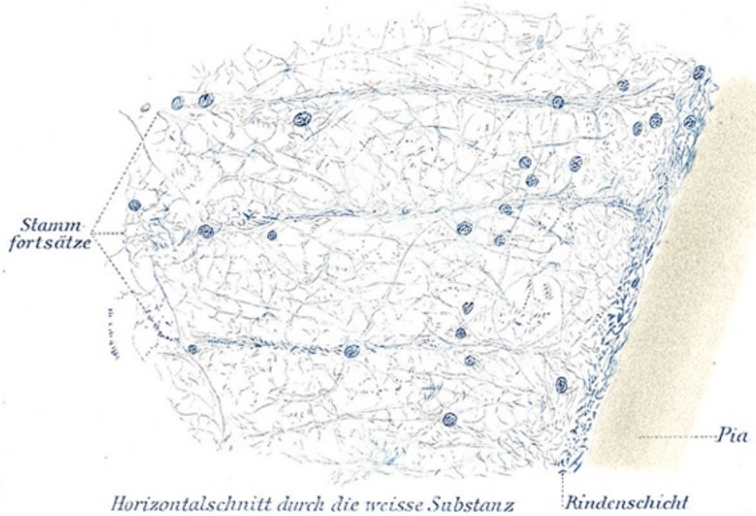

Horizontalschnitt durch die weisse Substanz

Fig. 5.

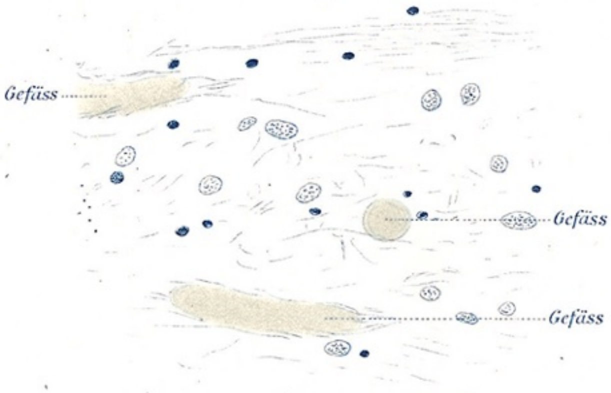

Hinterhorn I (Subst. gelat. Rolando)

Fig. 1.

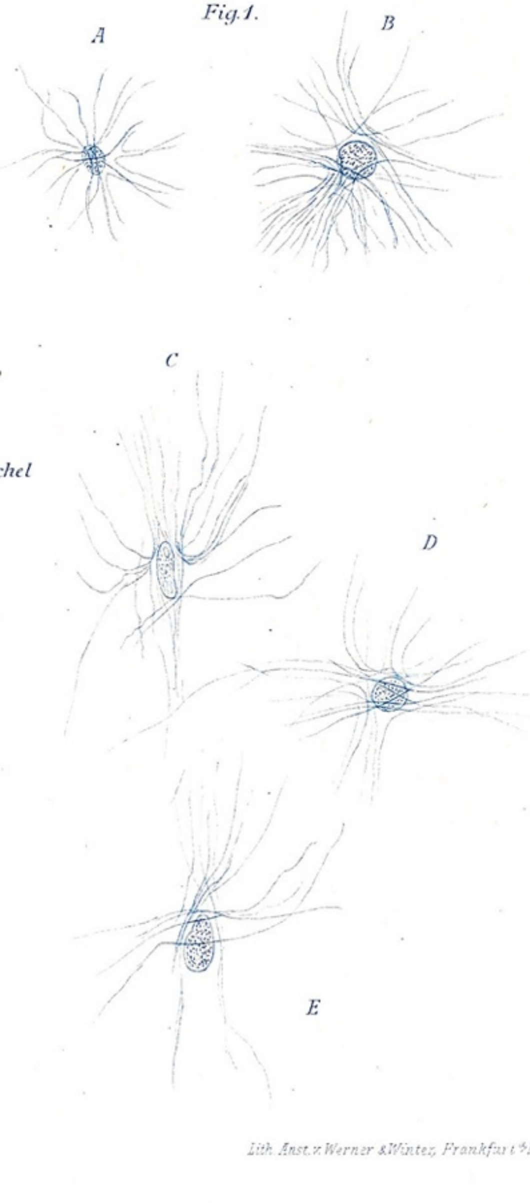

Fig. 3.

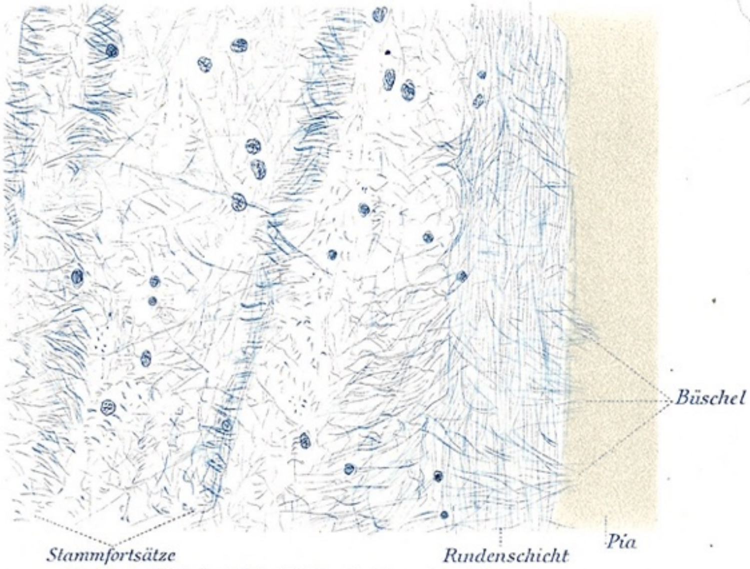

Vertical- (Schräg-) Schnitt durch die weisse Substanz

Fig. 4.

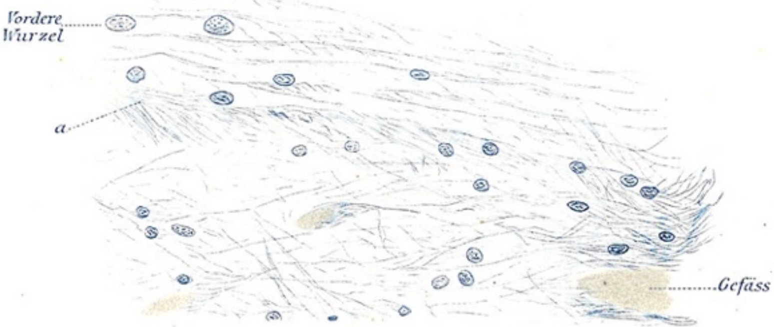

Verticalschnitt (vordere Wurzel.)

Weigert del. *Lith. Anst. v. Werner & Winter, Frankfurt a/M.*

Fig. 1. Sogenannte Astrocyten Fig. 2-5 Rückenmark

Fig. 1.

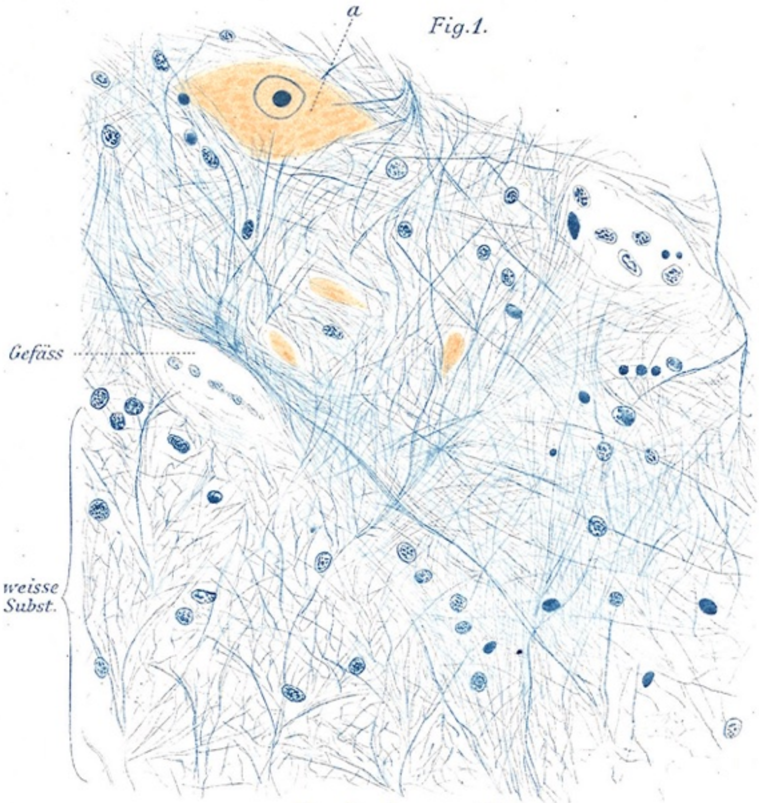

Vorderhorn (Kind)

Fig. 3.

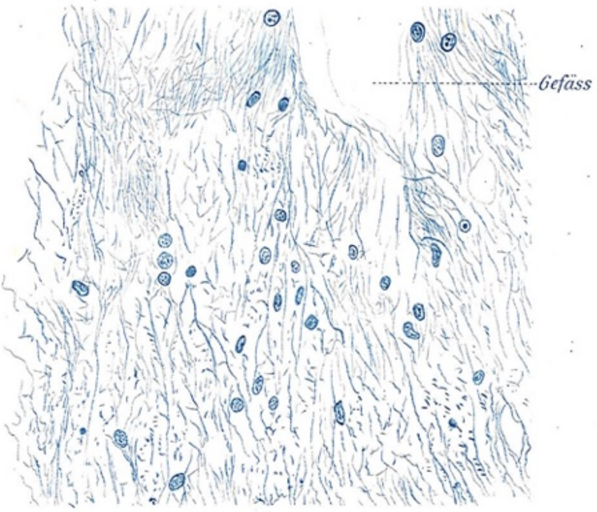

Hinterhorn II

Fig. 2.

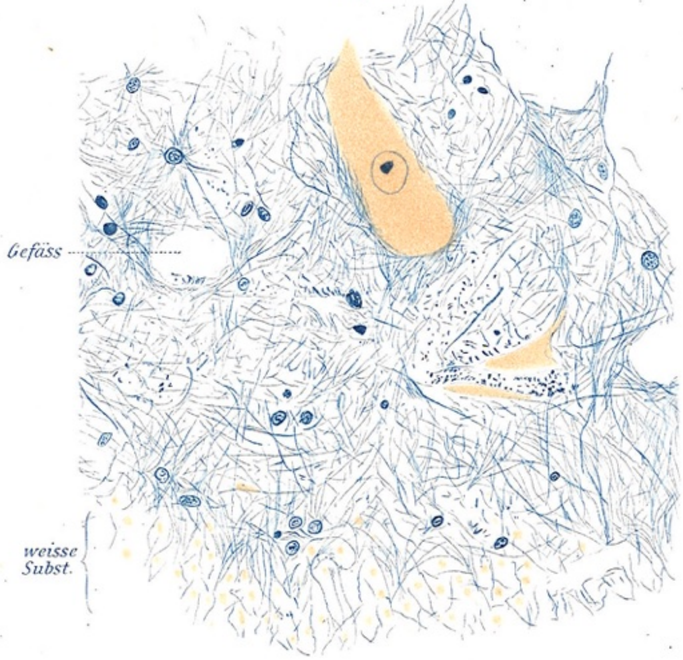

Vorderhorn (Erwachsner)

Fig. 4.

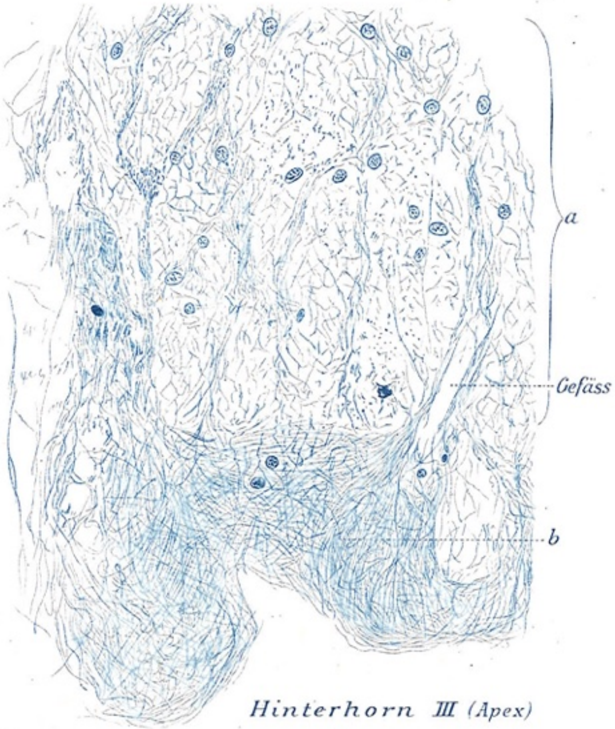

Hinterhorn III (Apex)

Weigert del. *Lith. Anst. v. Werner & Winter, Frankfurt a/M.*

Rückenmark

Fig. 1.

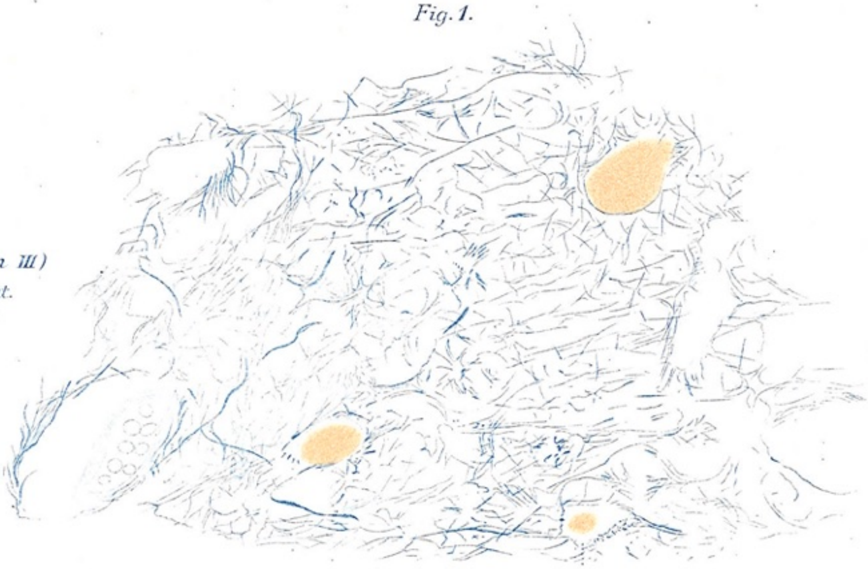

Fig. 2.

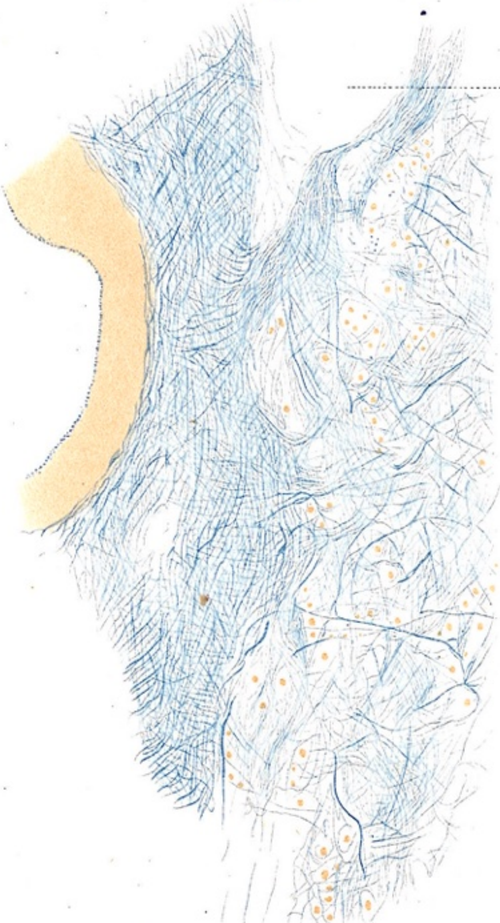

Fig. 3.

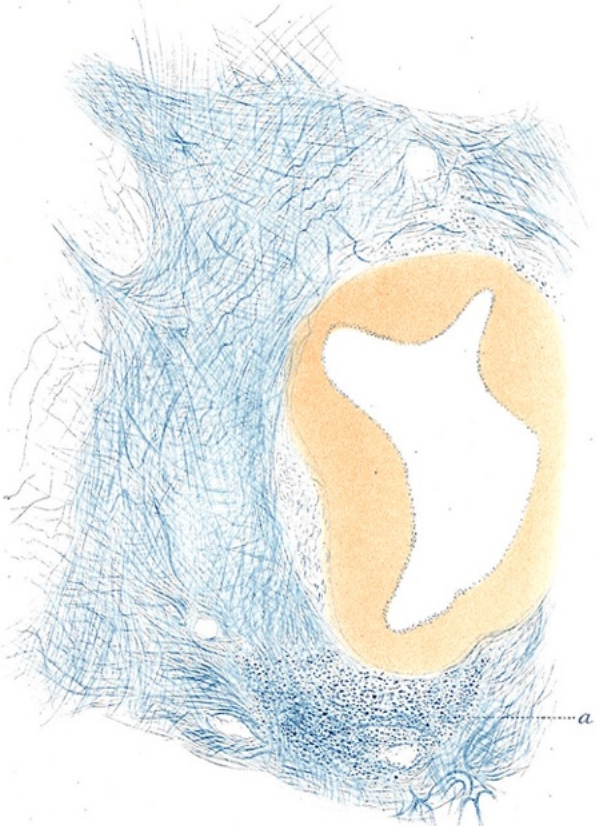

Weigert del.
Lith. Anst. v. Werner & Winter, Frankfurt a/M

Rückenmark

Fig. 1.

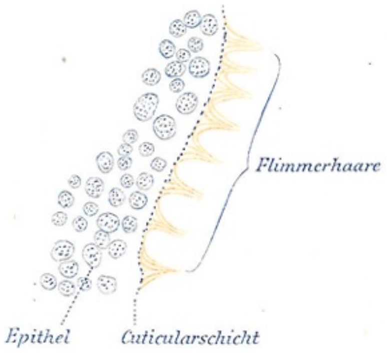

Fig. 3.

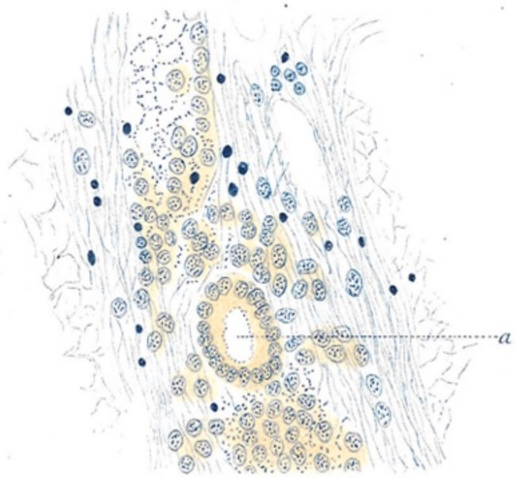

Fig. 2.

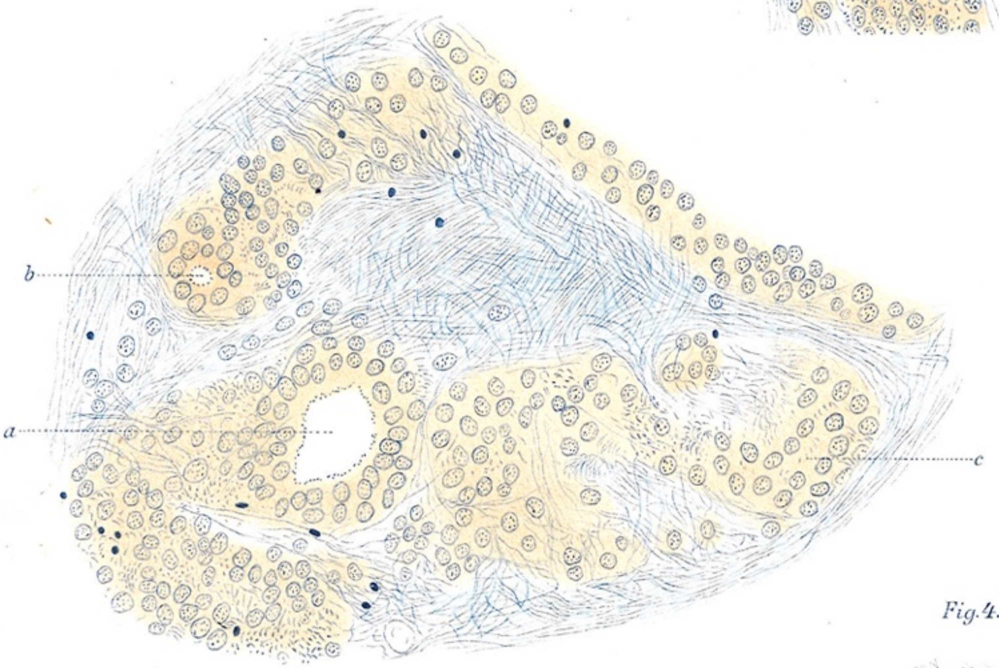

Fig. 4.

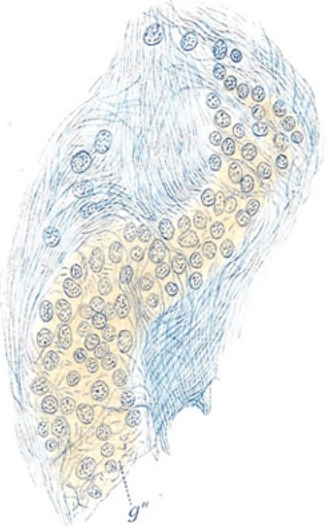

Fig. 5.

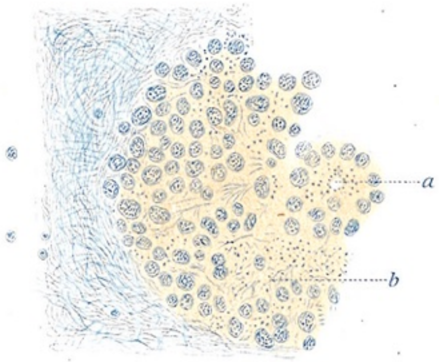

Weigert del.

Lith. Anst. v. Werner & Winter, Frankfurt a/M.

Ependym und Centralcanal

Fig. 4.

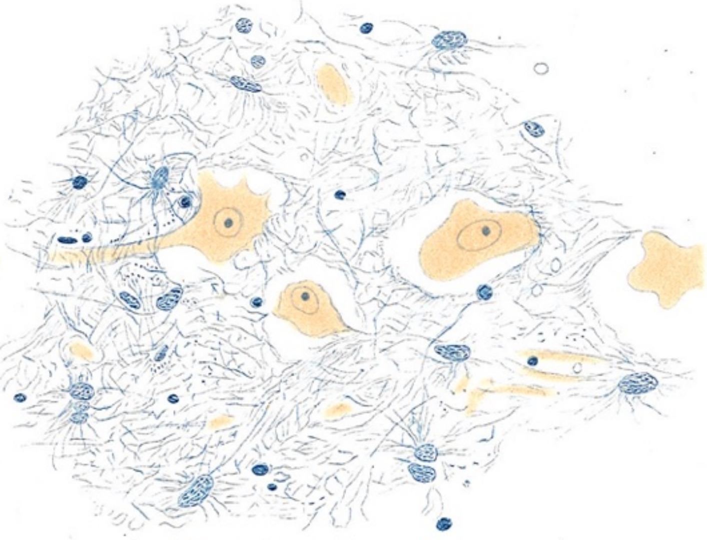

Hypoglossuskern

Fig. 1.

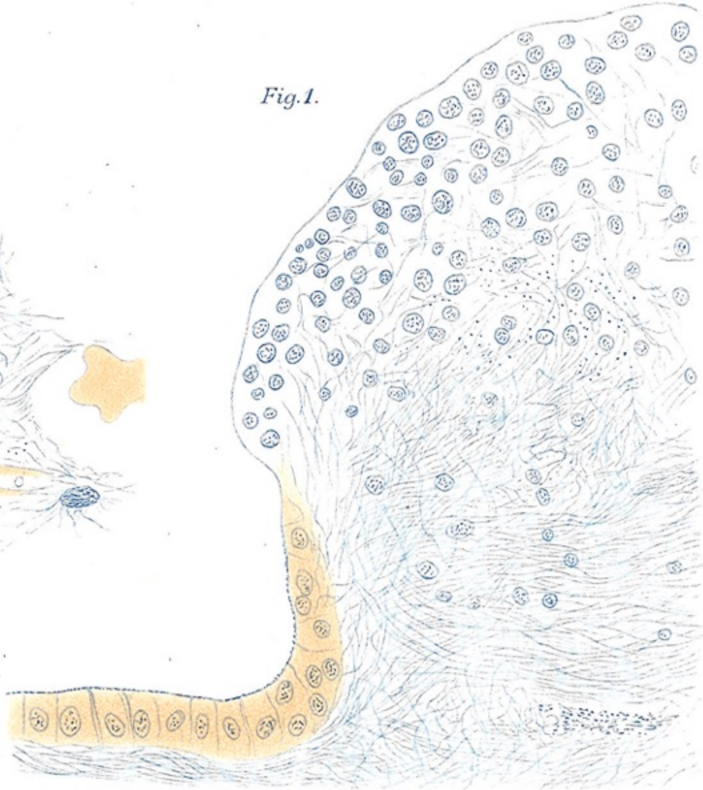

Ependymwucherung I

Fig. 3.

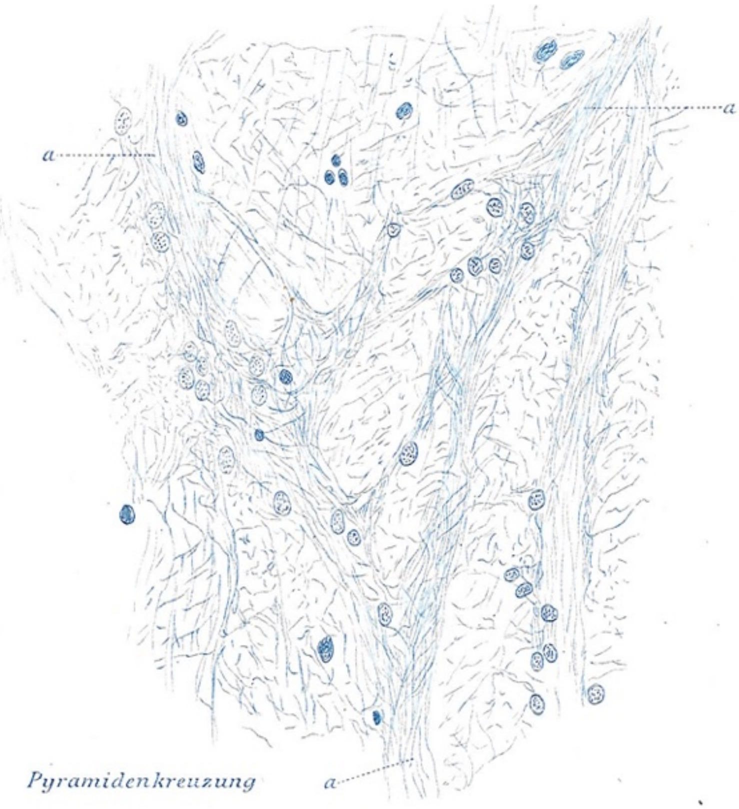

Pyramidenkreuzung

Fig. 2.

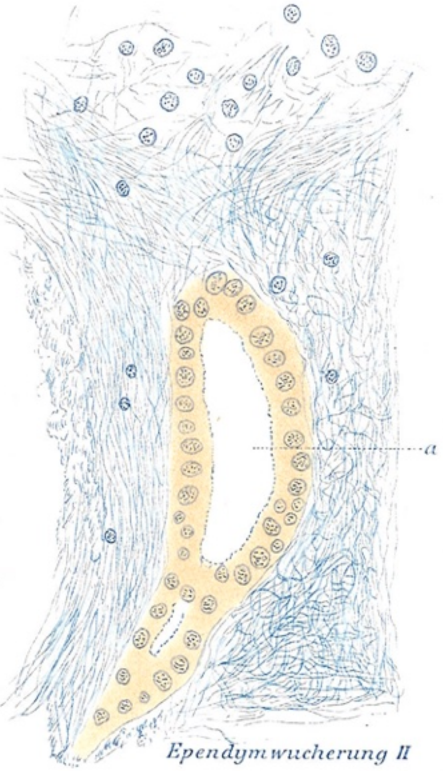

Ependymwucherung II

Weigert del.

Lith. Anst. v. Werner & Winter, Frankfurt a/M.

Medulla oblongata

Fig. 4.

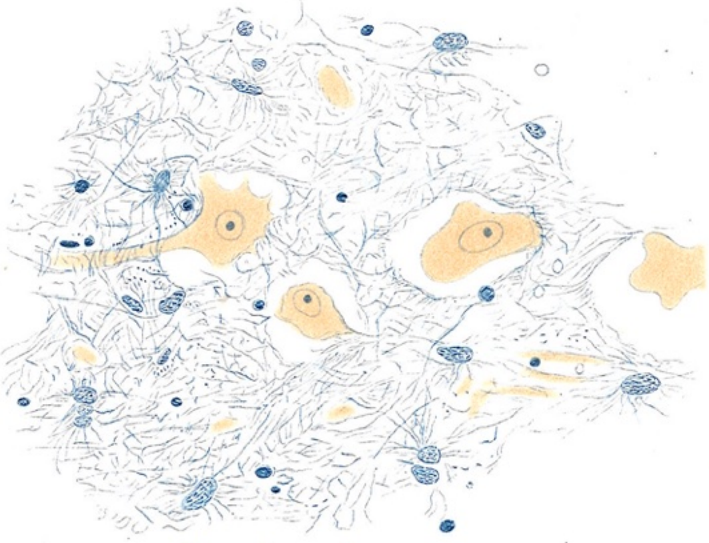

Hypoglossuskern

Fig. 1.

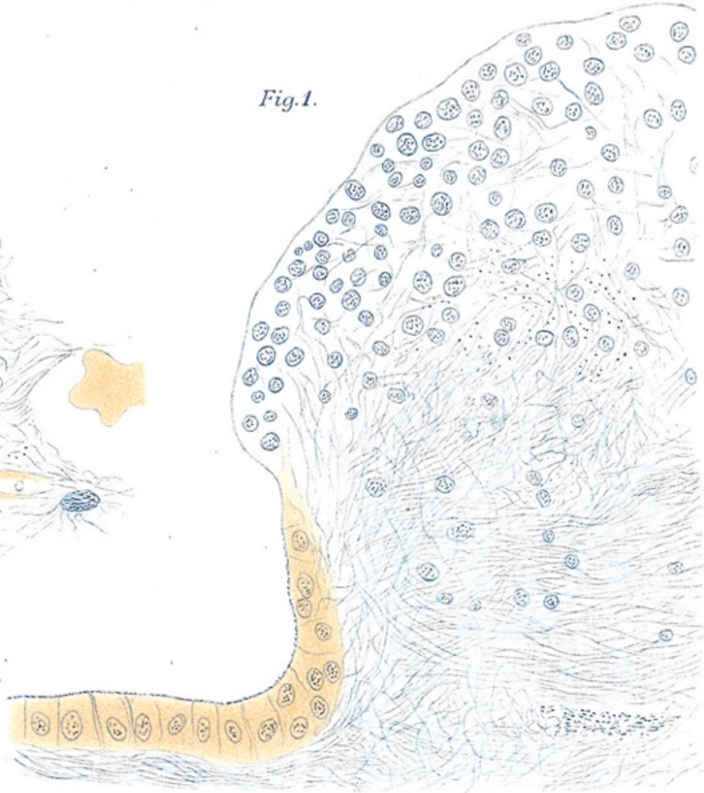

Ependymwucherung I

Fig. 3.

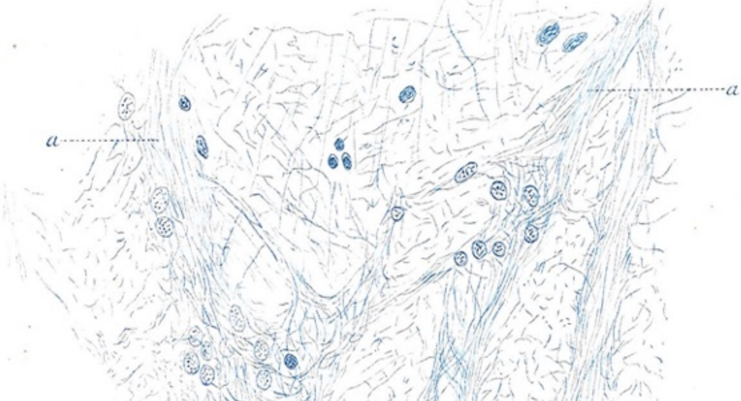

Pyramidenkreuzung

Fig. 2.

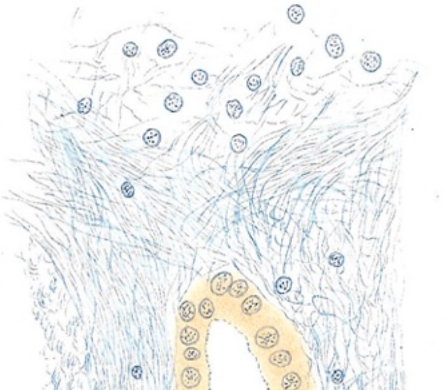

Ependymwucherung II

Weigert del. Lith. Anst. v. Werner & Winter, Frankfurt a M.

Medulla oblongata

Fig. 1.

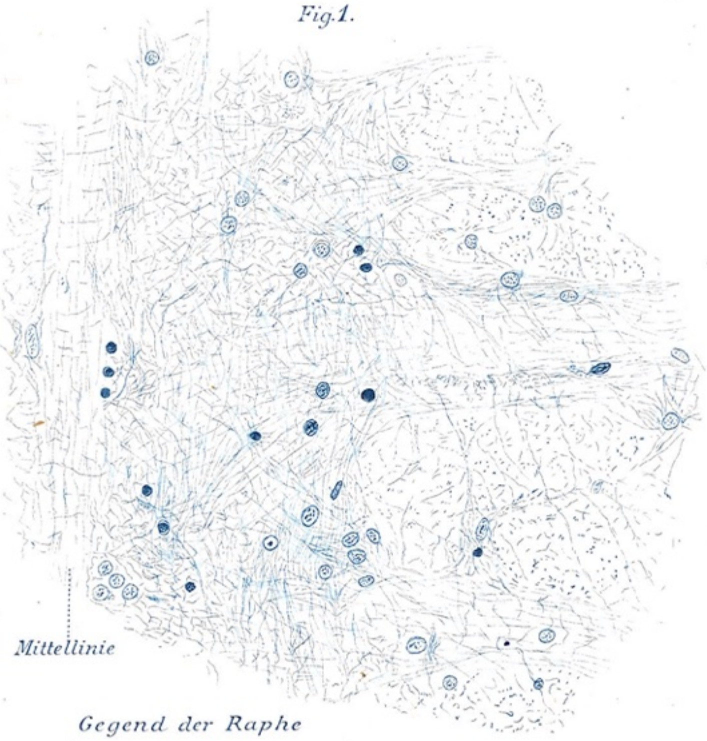

Gegend der Raphe

Fig. 2.

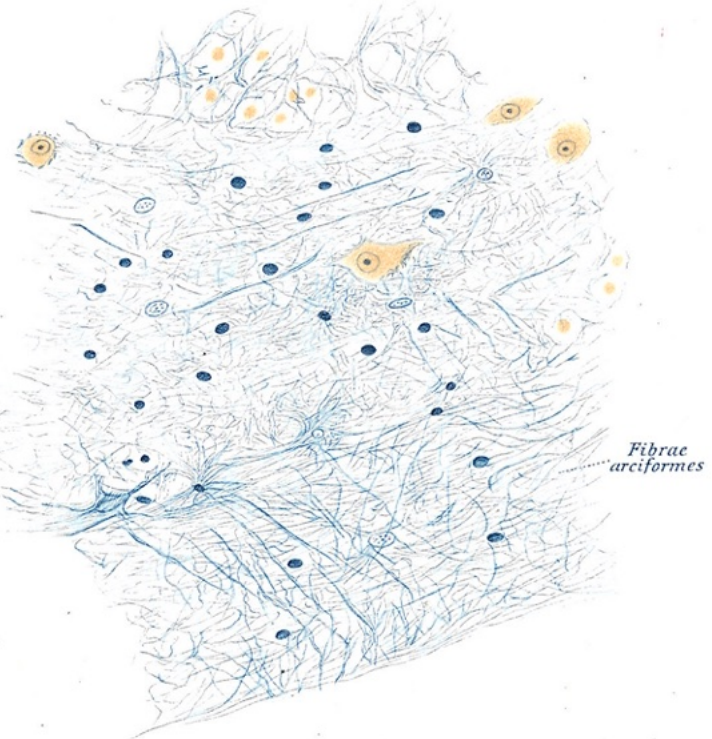

Pyramidenkern

Fig. 3.

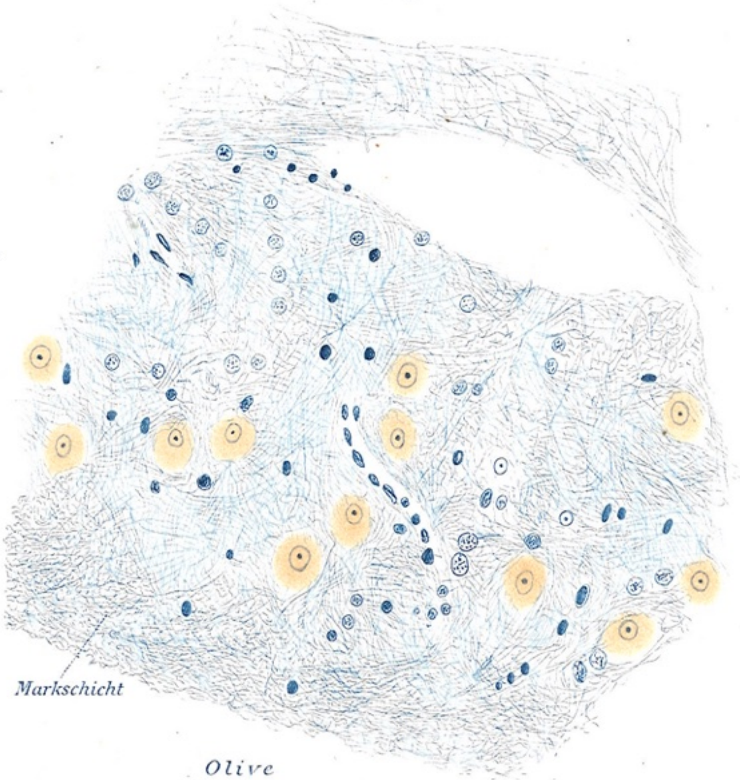

Olive

Fig. 4.

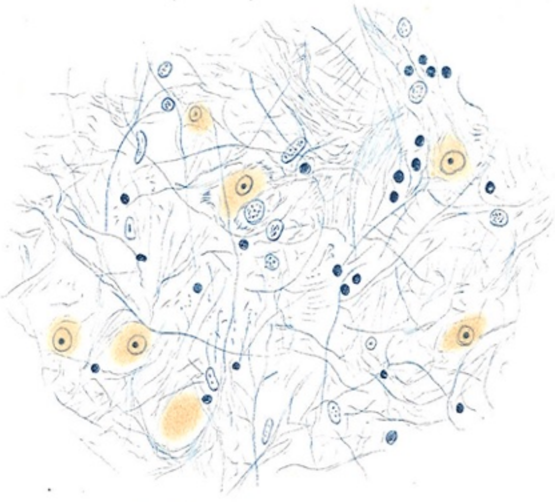

Nucleus gracilis

Weigert del.

Lith. Anst. v. Werner & Winter, Frankfurt a/M.

Medulla oblongata

Fig. 1.

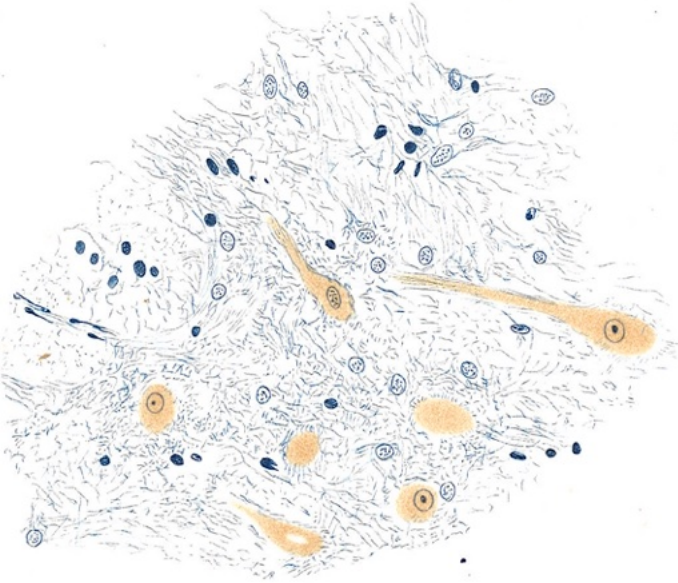

Nucleus ambiguus.

Fig. 2.

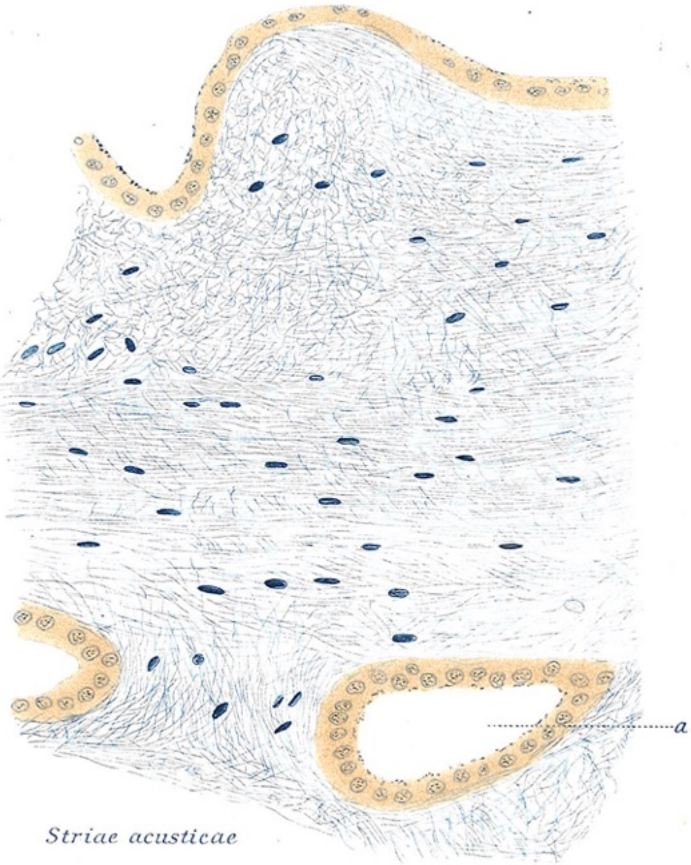

Striae acusticae

Fig. 3.

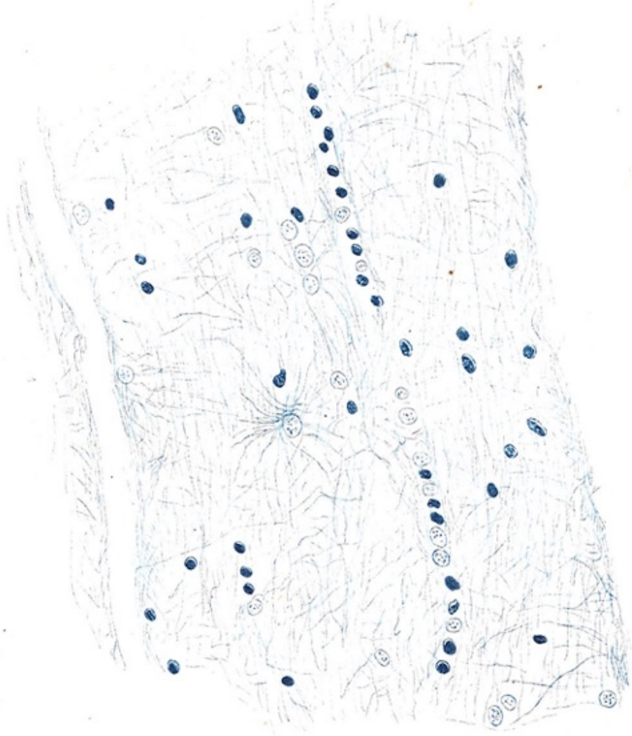

Längsschnitt

Fig. 4.

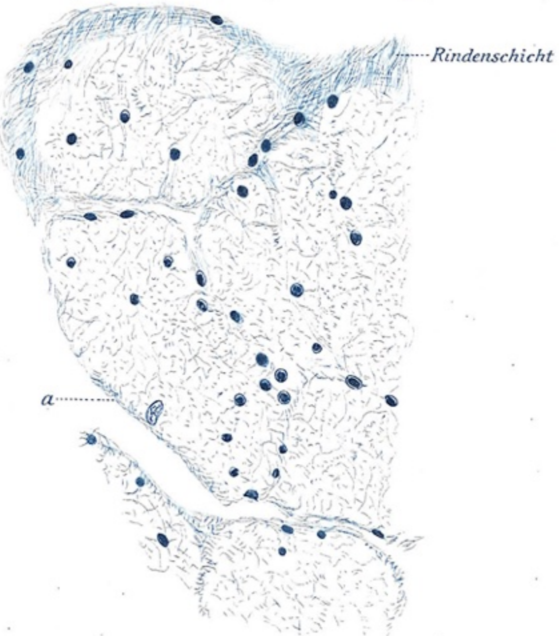

Querschnitt

Weigert del.

Lith. Anst. v. Werner & Winter, Frankfurt a/M.

1. 2. Medulla oblongata 3. 4. Opticus

Fig. 1.

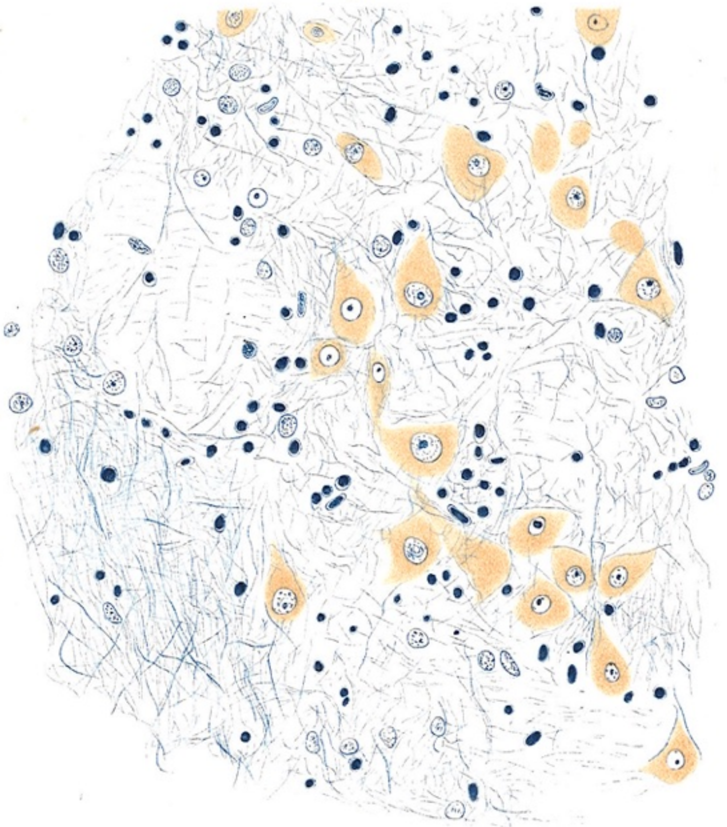

Substantia nigra

Fig. 4.

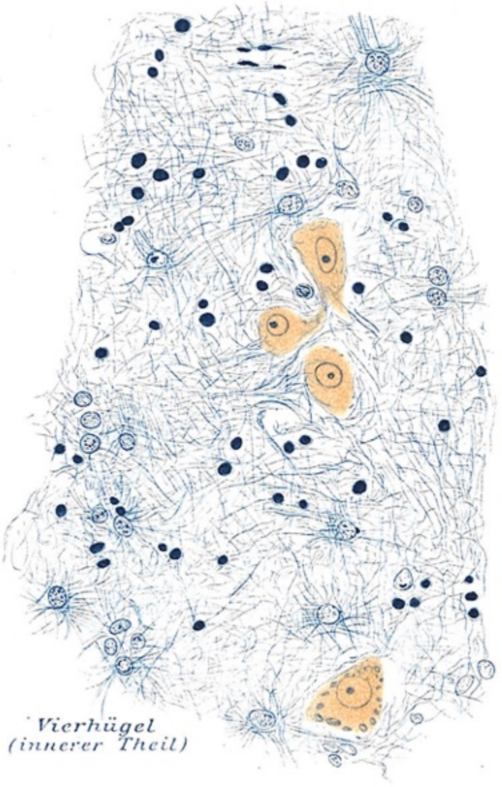

Vierhügel (innerer Theil)

Fig. 2.

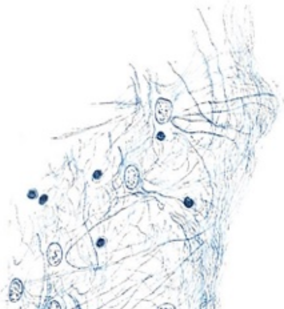

Gliahülle um einen Gefässraum

Fig. 3.

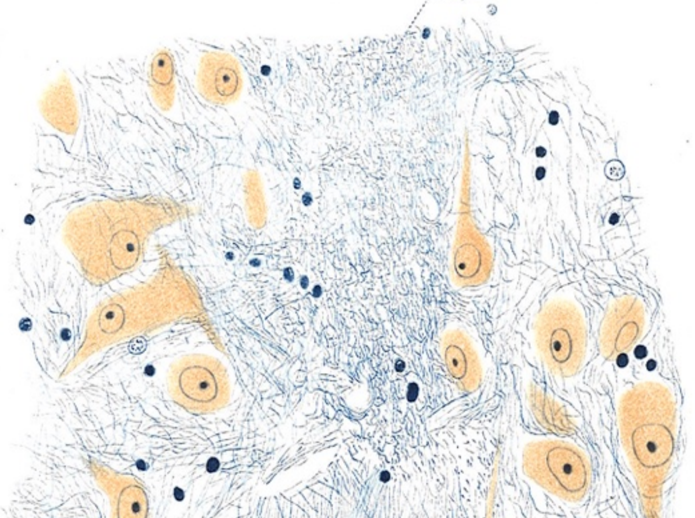

Oculomotoriuskern

Weigert del. Lith. Anst. v. Werner & Winter, Frankfurt a/M.

Fig. 1. 2 Hirnschenkel. Fig. 3, 4 Vierhügel.

Fig. 1.

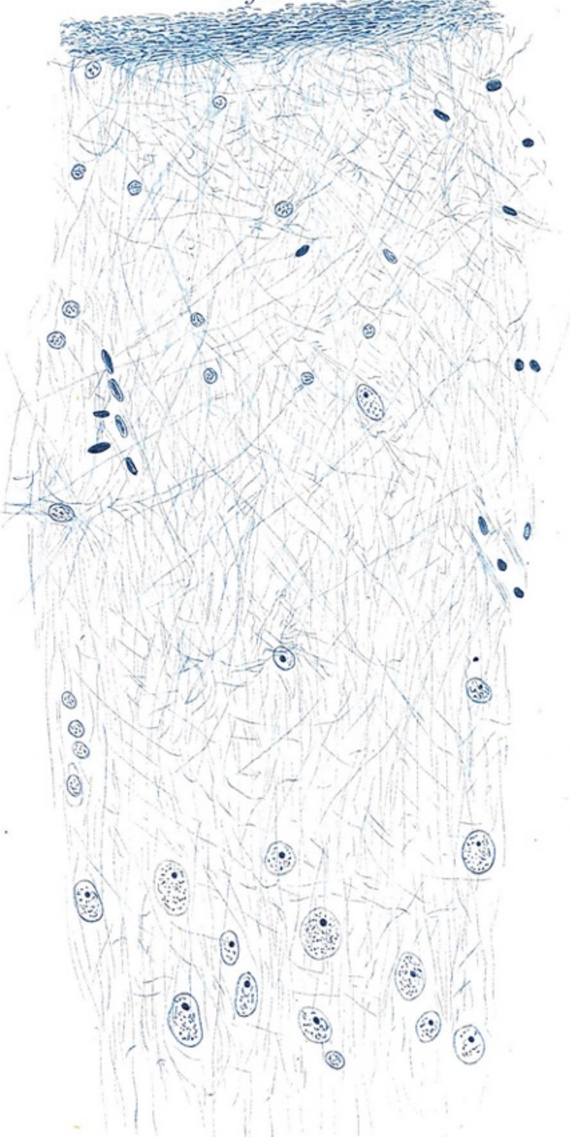

Rinde

Fig. 2.

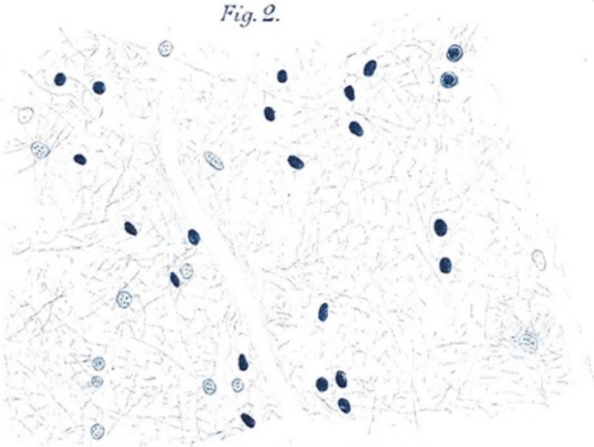

Marksubstanz

Fig. 3.

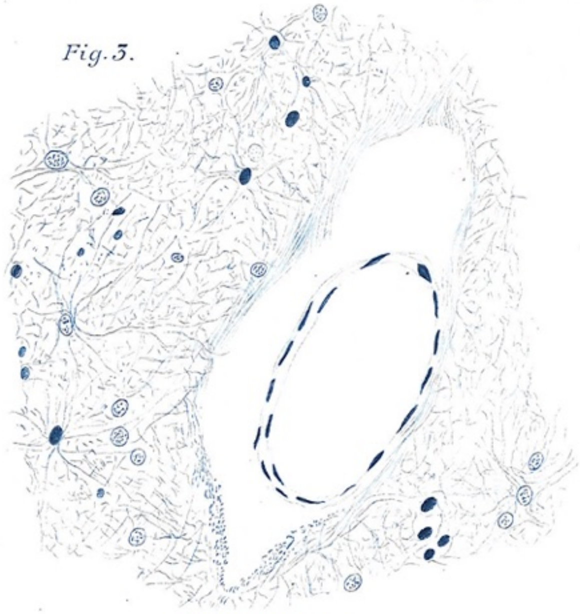

Marksubstanz

Fig. 4.

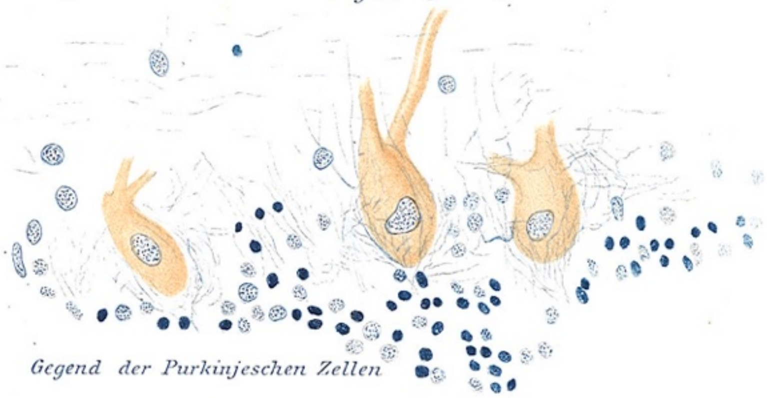

Gegend der Purkinjeschen Zellen

Fig. 5.

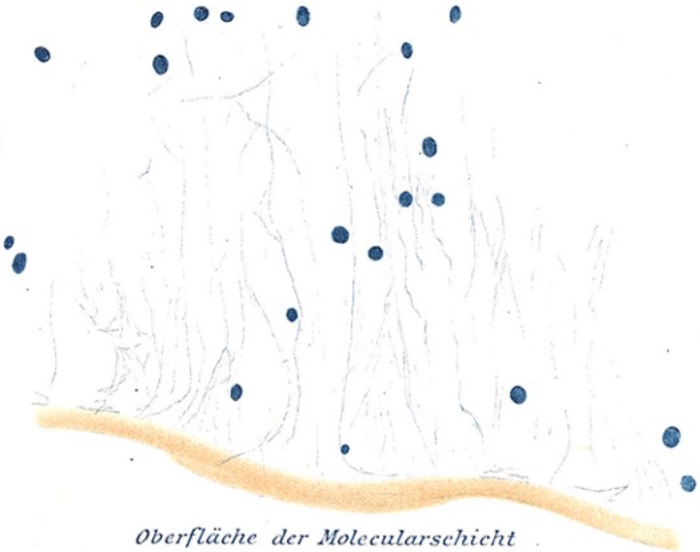

Oberfläche der Molecularschicht

Weigert del.

Lith. Anst. v. Werner & Winter, Frankfurt a/M

1-2. Grosshirn 3-5 Kleinhirn.

Fig. 2.

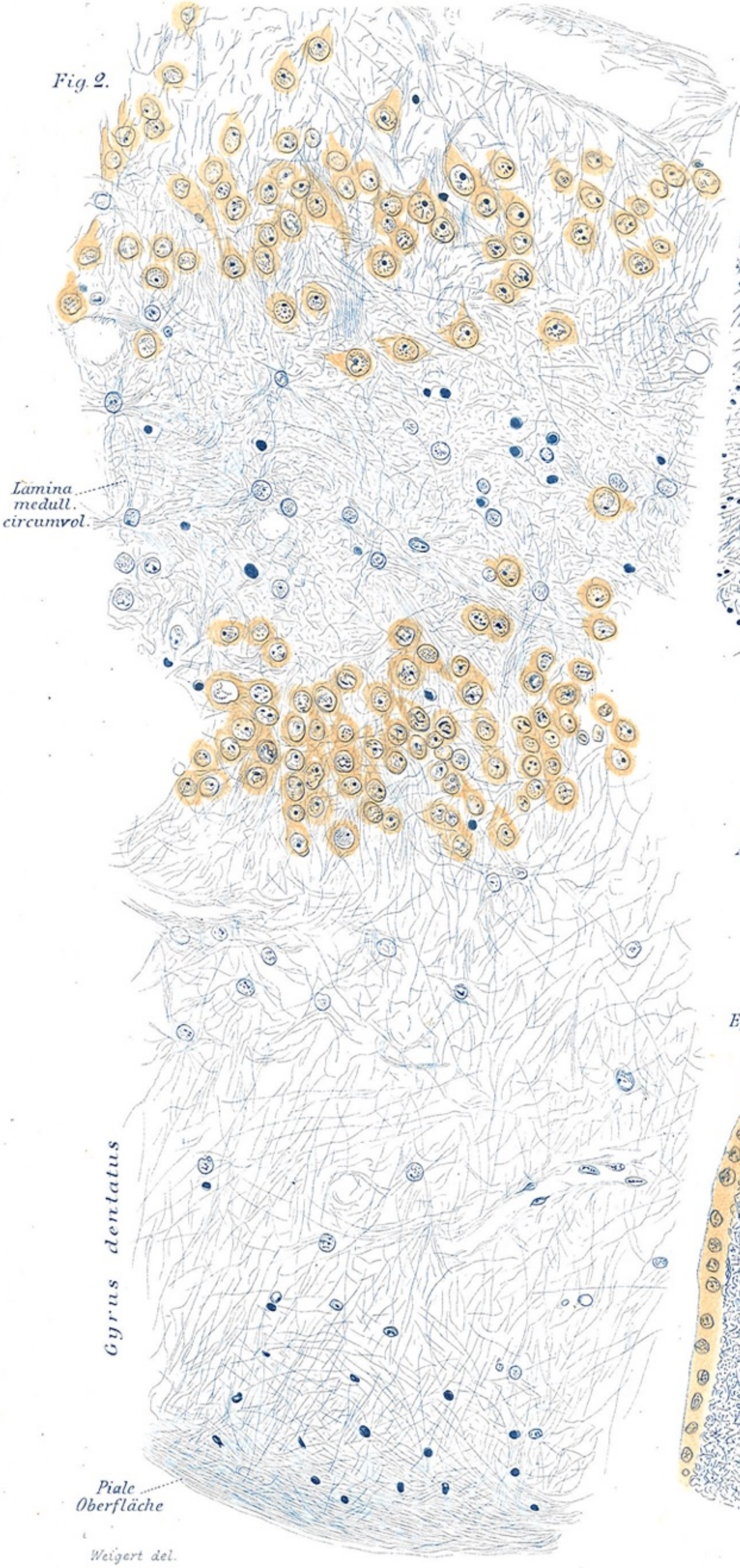

Fig. 3.

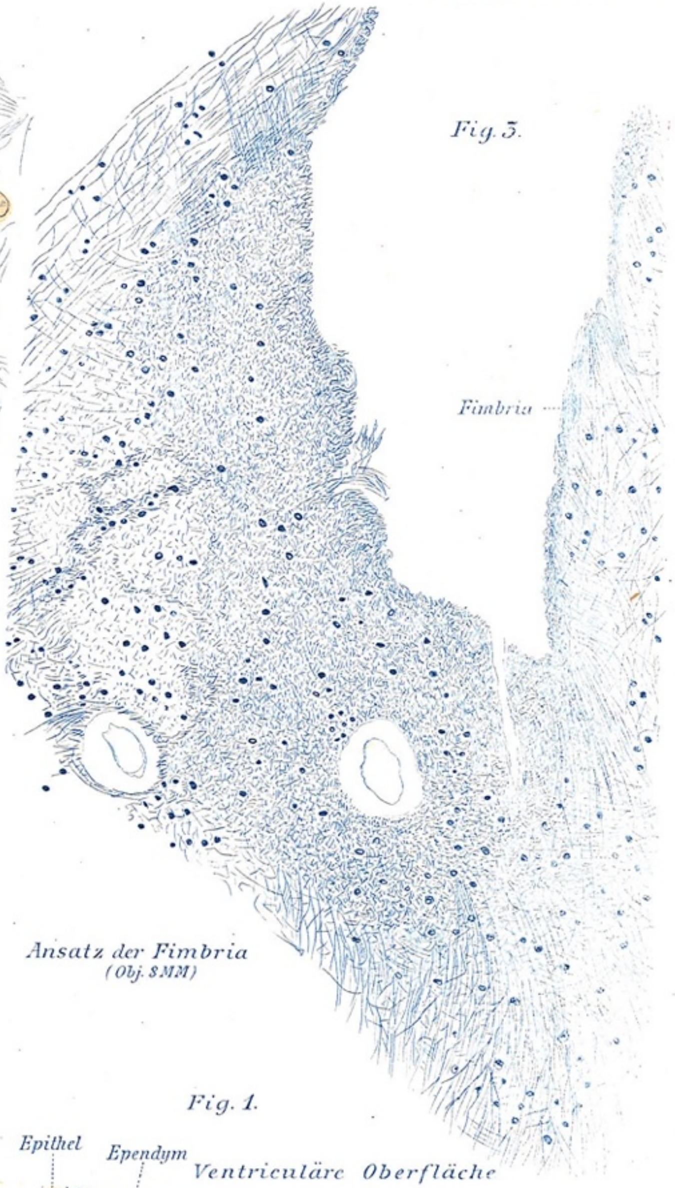

Ansatz der Fimbria (Obj. 8MM)

Fig. 1.

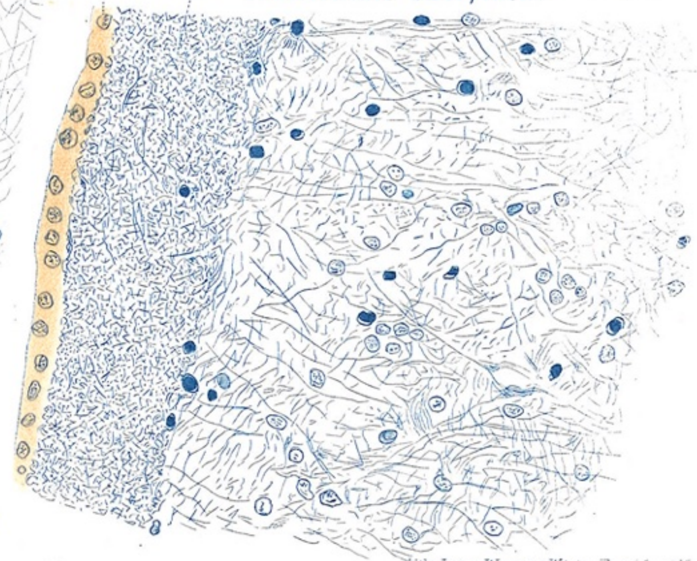

Weigert del.

Lith. Anst. v. Werner & Winter, Frankfurt a/M.

Ammonshorn

Fig. 1.

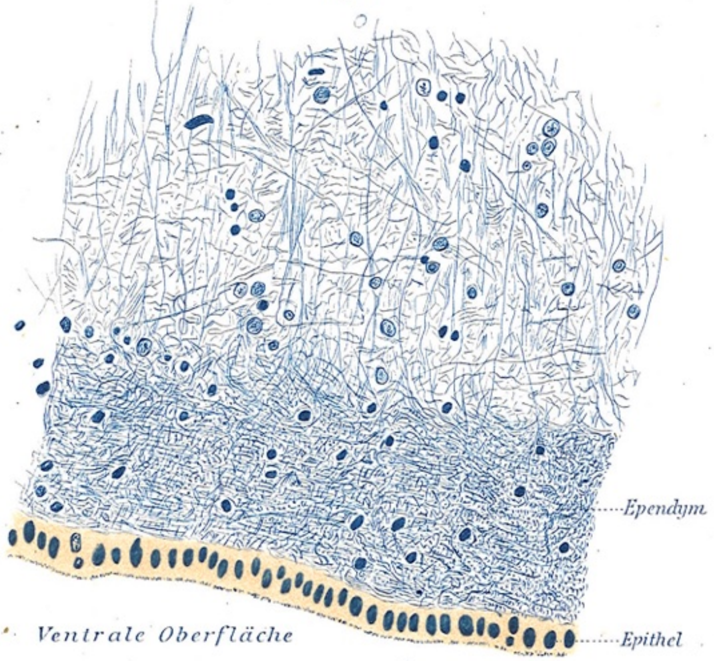

Ventrale Oberfläche

Fig. 2.

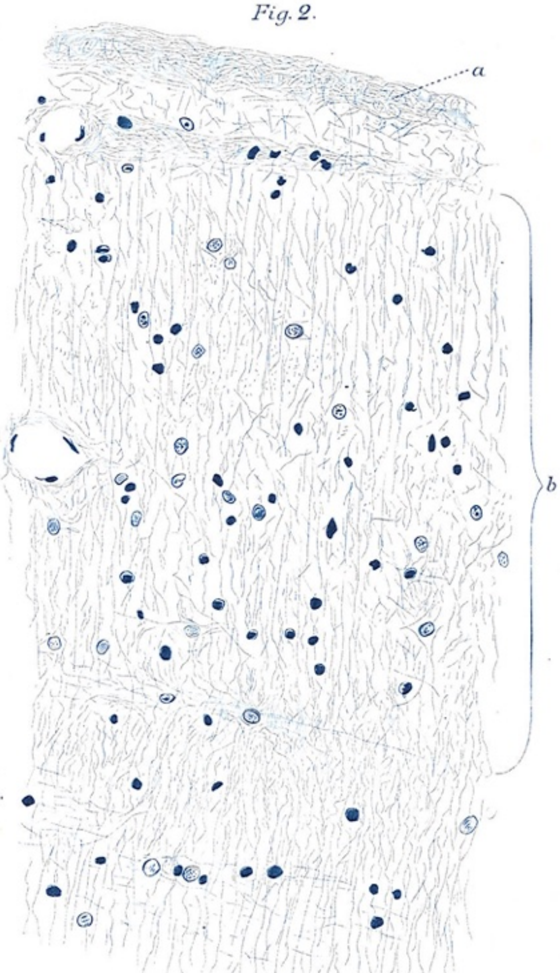

Dorsale Oberfläche

Fig. 4.

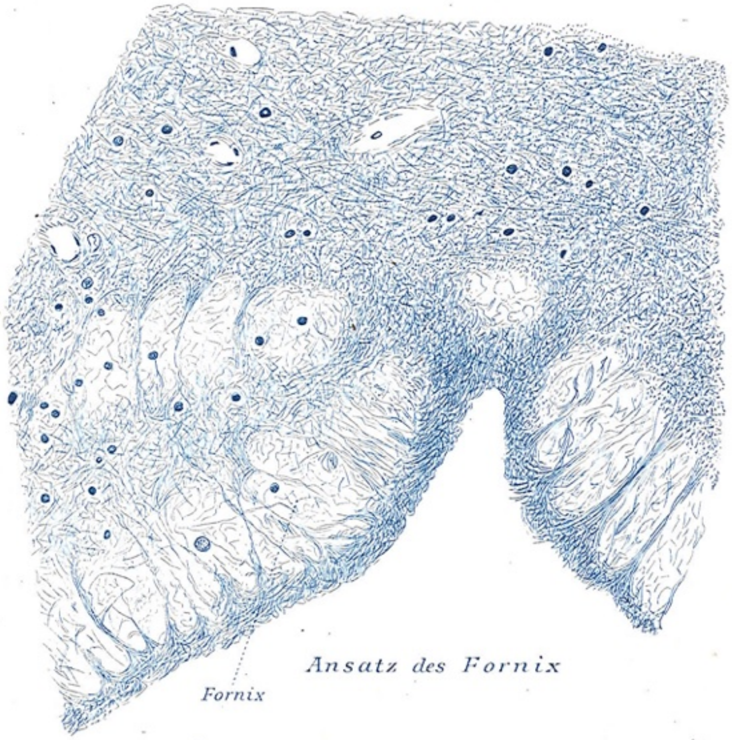

Ansatz des Fornix

Fig. 3.

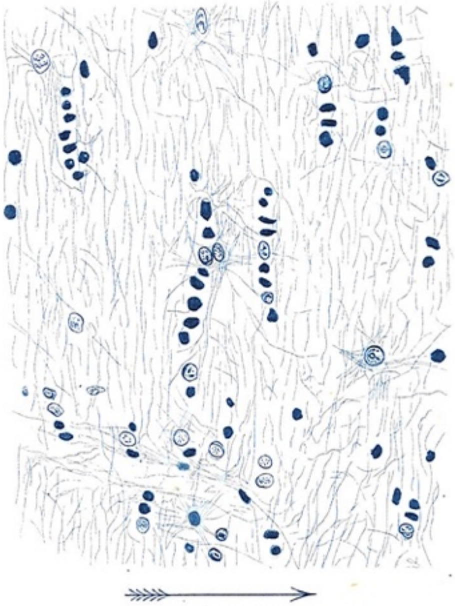

Tiefe Markschicht.

Weigert del.

Lith. Anst. v. Werner & Winter, Frankfurt a/M.

Balken

Fig. 1.

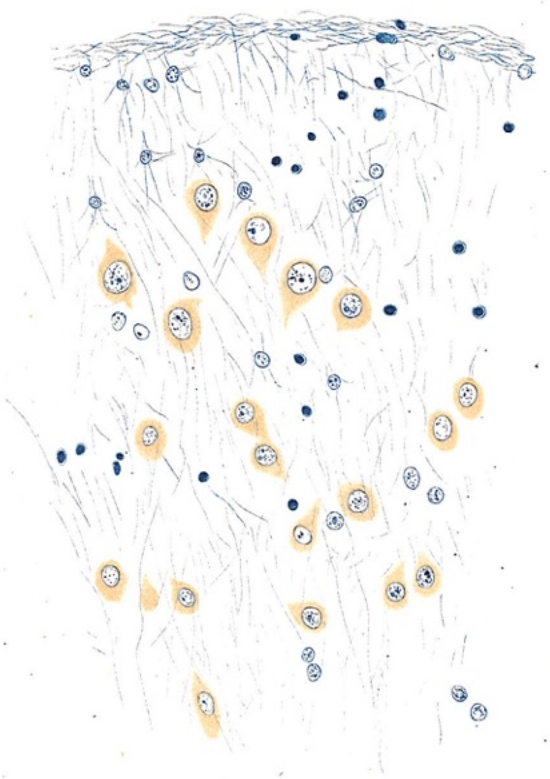

Ganglienzellenhaufen
(Balken)

Fig. 3.

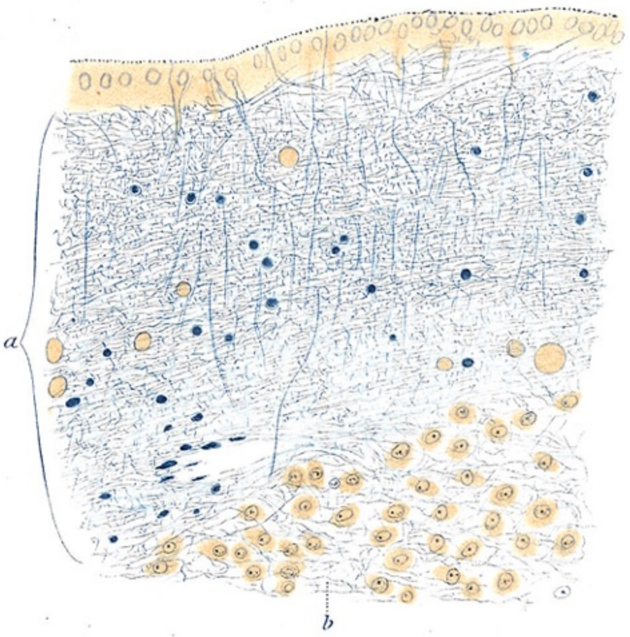

Sehhügeloberfläche mit Epithel
bekleidet

Fig. 2.

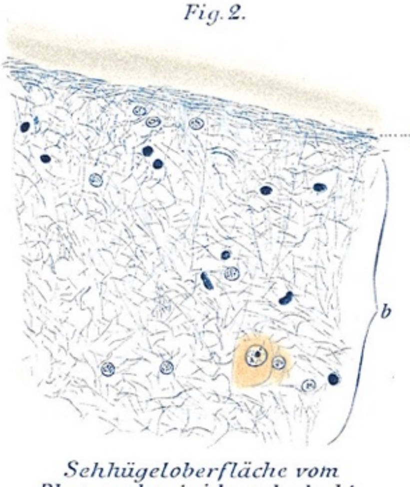

Sehhügeloberfläche vom
Plexus chorioideus bedeckt

Fig. 4.

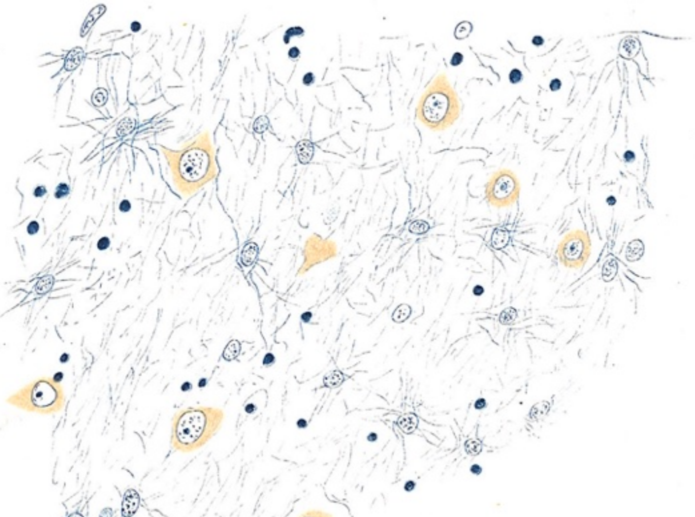

Pulvinar

Weigert del. Lith. Anst. v. Werner & Winter, Frankfurt a/M.

Fig. 1 Balken. Fig. 2 3,4. Sehhügel

Fig. 1.

Fimbria mit Plexus chorioideus

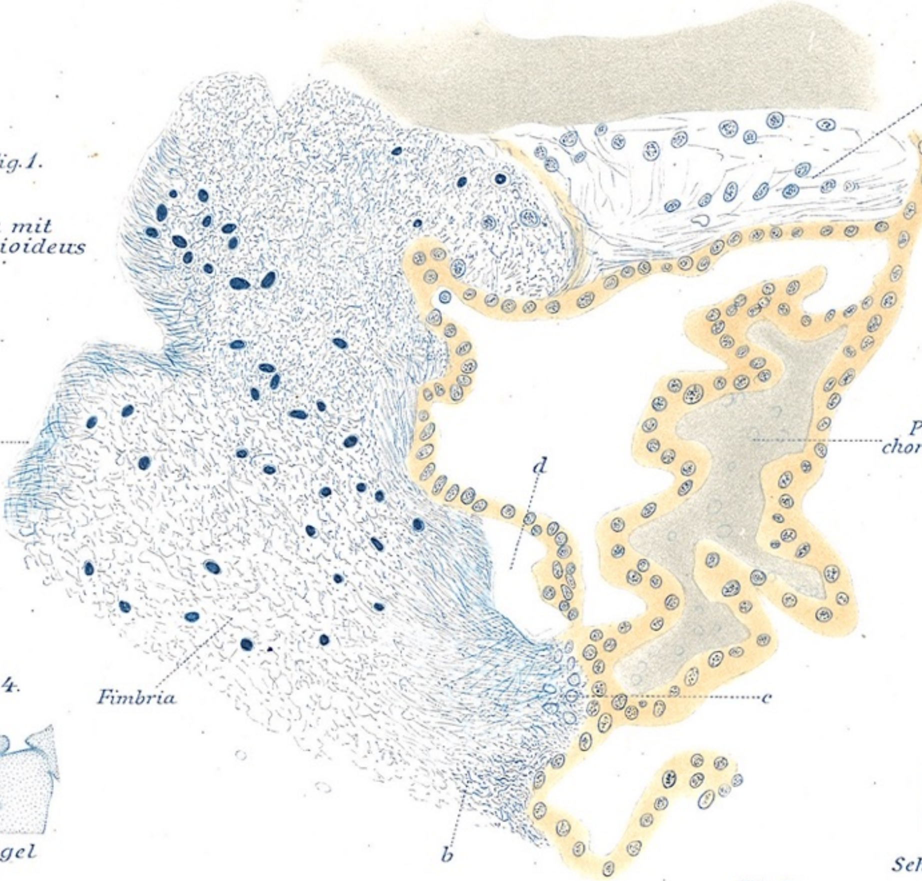

Fig. 4.

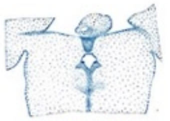

Vierhügel
1:1.

Fig. 5.

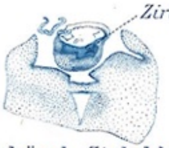

Sehhügel Zirbeldrüse
1:1

Fig. 2.

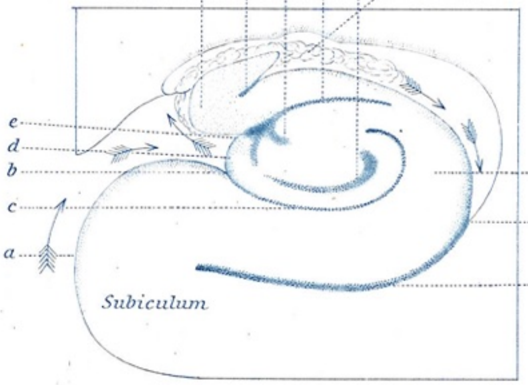

Ammonshorn 3½:1

Fig. 3.

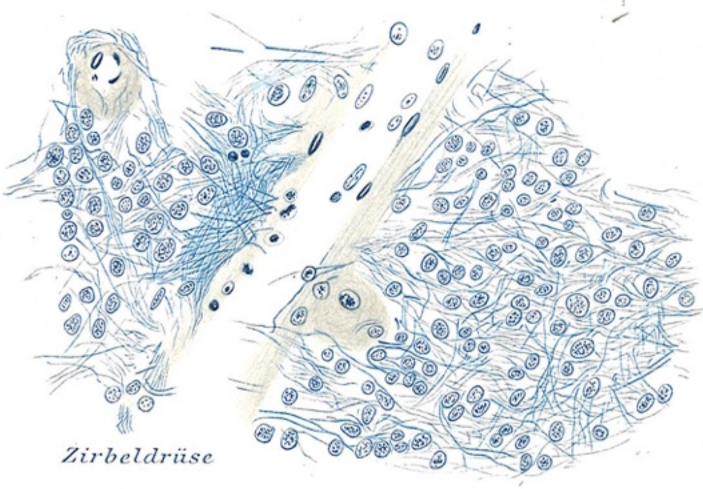

Zirbeldrüse

Fig. 6. Fig. 7. Fig. 8. Fig. 9.
