# Supplementary material for: The Concept of Neuroglia ‐ the State of the Art Circa 1900
Source: Glia. 2025 Feb 4;73(5):890–904. doi: 10.1002/glia.24678 (PMC11920685; doi:10.1002/glia.24678)
Supplement: Supplementary file 8 — Data S8. Translated text by Weigert with single figures inserted into the text. [file GLIA-73-890-s010.pdf]

# Commemorative publication

For the

fiftieth anniversary of the medical association

of

Frankfurt on the Main

November 3, 1895.

# Contributions

To

# The understanding of normal human neuroglia

By

Carl Weigert

With XIII panels

Commemorative publication

For the

fiftieth anniversary of the medical association

of

Frankfurt on the Main

November 3, 1895

Published simultaneously as issue II of volume XIX of "Papers published by the Senckenberg

Natural History Society"          Issue commissioned by

Moritz Diesterweg.

## Foreword

Det gik mig som En, der vandrer i en Labyrinth. Gang for Gang havde jeg fundet, og jeg stod an lige ved det Sted, hvor den endte. Men her fandtes en Muur, hvis Dör ingen Nögle formaatte at aabne, og som jeg mod meine Kräfter var for svag til at spränge. Hvergang jeg tog et nyt Udgangspunkt, kom jeg dog tilbage til den samme noverstigelige Muur. Indenfor den Iaa Skatten, men jeg syntes ikke at väre den, der skulde häve den.

*[I felt like I was walking through a maze. I had taken path after path, and finally found the end. But the end was a wall with a door which no key could open, and I was too weak to break it down. Every time I started over from a different starting point, I came back to the same insurmountable wall. The wall harbored a hidden treasure, but it seemed that I was not destined to lift it up"]*

Vilhelm Bergsöe: Fra den gamle Fabrik.

[From the old factory]

Anden Deel 8. 82. [Part two 8. 82.]

The following work is based on results, obtained using a new method. I have worked from December 5, 1888, to the present day to develop this method to its current version and I have devoted all my spare time, outside of my official duties, almost exclusively to this method.

It was during the first few years of this period of work that I visited a scholar who was an equally distinguished teacher, researcher and human being and spoke to him about the fact that I could not see myself finishing my work at all. During the course of our conversation, the revered scholar made something like the following comment: "Have you ever thought about why theorists like us actually work as scientists? We don't profit from it; because not a single penny is paid for even the greatest discoveries in our fields, and you have experienced for yourself that scientific work is of no use for one's career. So why do we persist this work? For the fame? Fame is flimsier and more ephemeral than spider webs, and in our fast-moving times one might borrow the words of Baccalaureus in Faust[1] to say: Even if you were famous for a brief time, soon no one will know what to say about you. So why do we do this sort of work? Simply because *we derive enormous and absolute joy from science research*.

[1] This is a reference to the play Faust by the famous German writer Wolfgang Goethe

If this had indeed been my motivation for working all of these years, I would have had almost seven reasonably happy years behind me, — but unfortunately my experience was quite different. If I only count from the very first time when I believed I saw a new and fruitful domain in front of me, and when I thought that I simply had to stretch out my hand to own this domain, — if I only consider this short period of time, my work on the new method was just the opposite of pleasure and joy. It was a succession of ever new hopes and ever new disappointments, a succession of perpetual agonizing tests of patience. At times I had to wait several weeks, usually it was of the order of several days, before I knew whether my new attempt was successful or not.

A Danish natural science researcher, also an outstanding poet, gives an excellent account of what such times conjure up in one's mind. I am referring to Vilhelm Bergsöe. In his novel "Fra den gamle Fabrik" (From the old factory), he recounts the plight of a chemist, in pursuit of a new discovery, but who cannot get beyond the "next", the "almost there" step. For my esteemed Scandinavian friends, I have included a pertinent passage of the *original* text at the beginning of this foreword; its English *translation* [1]follows:

> "I felt," says Olsen, the chemist in the novel, "like I was walking through a maze. I had taken path after path, and finally found the end. But the end was a wall with a door which no key could open, and I was too weak to break it down. Every time I started over from a different starting point, I came back to the same insurmountable wall. The wall harbored a hidden treasure, but it seemed that I was not destined to lift it up."]

I have taken the liberty of changing the verbs from the present to the past tense, both in the Danish citation, as well as its translation. Whether I have the right to do this, must be decided by those who try the new method.

But why did I continue the work if it was so unedifying to work on this method? Why did I not follow the advice of my friends and do something "worthwhile"? *Well, it just wouldn't let me go.* The work was somehow particularly gripping. I was always faced with the hideous "almost there", but I always believed that the next attempt would have to succeed, — again, just as Bergsöe describes it in his Chemist (Vol. 11 p. 146). It seemed that the long struggle would be rewarded in only a few more days or weeks! But days and weeks turned into months, months into many years, time passed without me noticing, until eventually a reasonably acceptable successful result was achieved after all.

[1] Weigert translated the text into German. The English translation of this sentence was therefore adapted accordingly.

As the saying goes: Tantae molis erat romanam condere gentem ! ["Oh how difficult it was to build the Roman Empire!"] but in my case it was not about the difficulty of building the roman empire but the difficulty of finding a simple histology method. Had I not wasted time and trouble[1], had I not squandered my efforts, or to use the words of Bergsöe's chemist, "scattered the gold grains of life like sand," ("Livets Guldkorn jeg spredte som Sand")?
Only time will tell. A new method is simply a key to open a door of the insurmountable wall that harbors all science treasures. The key, which I hand over to the science community, does not unlock the door very smoothly, it still has to be carefully filed down and refined, but it unlocks the door nonetheless, to allow everyone to make use of the treasures inside. I will present a few samples of the treasure in this book. If as a result of this (and excluding my modest share) *many additional treasures are unearthed by others who use this new method*, I will be completely satisfied and I can then confidently say: Oleum et tempus non perdidi. [I have not lost both time and trouble [2]]
I am, of course, well aware that some people consider the invention of a new method as an inferior scientific achievement, and one might even say look down on its inventors. But upon closer inspection, these same people use the methods devised by the inventors they hold in such low esteem with the greatest of zeal, to execute their own science projects with the *greatest of skills*. Many a mason's apprentice may even despise the architect, whose plans need to be followed, because the architect does not place the bricks one on top of the other himself/herself. These oddballs also have a place in this world!

---

Along with the new method, I am also publishing a series of observations. For work that took almost seven years to accomplish, this may seem a little meagre to some, and at best very incomplete. But I ask you to remember, that I continue to struggle with the imperfections of the method to this day, and that as long as this is the case, the mind is not free enough to engage in any other *intensive* evidence-based research. It is *only recently* that I have been able to harvest the crop that I had sown so long ago. Under the circumstances, it might have been better if I had followed the "nonum prematur in annum" ["put your parchment in the closet and keep it there till the ninth year."] literally, but that was not an option. I had committed to publish this work as a commemorative publication for the medical association in Frankfurt on the Main on November 3, 1895, and it was no longer possible to defer the publication date any further. I therefore ask the readers to excuse the unfinished and imperfect aspects of this book.

[1] Literally "Had I not wasted oil and time".
[2] Literally "Not lost oil and time".

Although I had committed somewhat hastily, the desire to present a token of my gratitude to my colleagues in Frankfurt in this publication made me overlook the difficulties that lay ahead. It had now been ten and a half years since my appointment to the Senckenberg Medical Institute, which not only provided me with a place to escape to, but also opened up an almost enviable range of effective reach. During this entire time, my colleagues at the institute have shown me so much kindness, and I have derived so much intellectual stimulation from our conversations, both in a learning and teaching capacity, that my eagerness to contribute in some way to the jubilee celebration of their association was irrefutable. May the spirit of collaboration and genuine scientific endeavor, which brought together its founding physicians some 50 years ago, always remain true to the medical association, may it flourish and prosper far into the future!

Frankfurt on the Main.

The Senckenberg Institute of Pathology and Anatomy.

The author.

Table of Contents

| | Page |
|---|---|
| Chapter 1: Historical background | 1 |
| Chapter 2: Relationship of neuroglial fibers to cells | 30 |
| Chapter 3: On the nature of neuroglia using the new fiber staining method | 43 |
| Chapter 4: Relationship of neuroglial fibers to other neuroglial components and to connective tissue. Chemistry. | 54 |
| Chapter 5: A discussion about the histogenetic status of neuroglia | 58 |
| Chapter 6: Other histologic features of neuroglial fibers | 67 |
| Chapter 7: General neuroglial fiber topography | 72 |
| Chapter 8: Specific neuroglial fiber topography | 81 |
| 1) Spinal cord | 81 |
| 2) Medulla oblongata | 98 |
| 3) Pons | 103 |
| 4) Pedunculus cerebri | 103 |
| 5) Quadruplet bodies | 104 |
| 6) Pineal gland | 105 |
| 7) Cerebellum | 106 |
| 8) Cerebrum | 108 |
| 9) Gyrus hippocampi. Cornu Ammonis | 110 |
| 10) Corpus Callosum and Fornix | 114 |
| 11) Opticus and Chiasm | 116 |
| 12) Corpora mammillaria | 117 |
| 13) Thalamus | 118 |
| 14) Corpus striatum and capsula | 120 |
| Chapter 9: The physiologic function of neuroglia | 121 |
| Chapter 10: Method | 128 |
| Figure legends | 146 |

# Contributions

To

# The understanding of normal human neuroglia

By

**Prof. Dr. C. Weigert.**

## Chapter 1:

## Historical background

Many people believe that we still know "nothing at all" about the natural sciences. There are many questions that remain unresolved, and many more questions that are yet to be asked; for it is a peculiarity of science that the *answer* to every question is *followed* by another question, one that was previously unexpected, that every "therefore" generates many "whys " that each in turn require their own "therefores" and so on and so forth ad infinitum. The sequence is indeed infinite and applies to the small as much as to the large scale, to space as well as time, and if we consider that we only dispose of finite means, we understand why someone like Faust despairs about being unable to understand the forces of nature all around him. Compared to the *infinite*, which is what we *ought* to know, the *finite*, which corresponds to what we actually *could* know, equates to *zero* in all contexts. Considered from that perspective, people who believe that we "still know nothing at all" in the natural sciences are undoubtedly right. But there is an alternative approach to demanding the impossible, and to striving to attain the impossible like Faust. That is to focus on comparing what we know today, not with what we *ought* to know, but with what we *did* to know yesterday. If we compare what we know today with what we knew yesterday we know *quite a lot* and *Goethe* would be wise to refrain from mocking those amongst us who draw great pleasure in immersing ourselves in the spirit of the age, to subsequently rejoice in the fact "that we got so gloriously far in the end after all."

As natural scientists, we can indeed allow ourselves this satisfaction, — as we are in any case always shielded from arrogance, when we remember how much still remains to be researched, even though we do not demand the impossible, the infinite.

We can also indulge in this gratification as regards the central nervous system, because the sheer abundance of questions that still need to be resolved serves as a constant reminder to keep us modest. We do not need to go as far back as Hippocrates, Rhazes and others, because even at the beginning of 1800s our understanding about the more intricate structures of the brain and the spinal cord, about the functions of their different components, was still very poor. Age-old questions, such as whether or not the brain is derived from the spinal cord, and whether or not the spinal cord is a nerve, were still keenly debated, and it was the latter question that gave rise to the eminent study entitled "About the Spinal Cord" by Keuffel which dutifully figures at the beginning of every historical account about neuroglia.[1]

Keuffel, admittedly, did not believe that the spinal cord as a whole was a nerve. At that time grey matter[2], which is not found in peripheral nerves, had already been described, and one spoke of "hydrogenic and oxygenic contrast" in the central nervous system[2] , where grey matter corresponded to the "hydrogenic", and white matter to the "oxygenic" component, but the appearance of the oxygenic component was so similar to the nerves that Keuffel looked to see whether there was not an oxygenic analogue in nerves too, much like his teacher Reil had previously identified around peripheral nerves, namely the neurolemma.

Keuffel was, of course, not the first "to observe the same strand-like structure that Reil had discovered in the nerve bundles" of the spinal cord, since Villars from Strasbourg had previously described it, as Keuffel himself reported. Villars was also the first to cut "thin slices" of spinal cord, as opposed to the conventional coarse preparation methods previously used to study the spinal cord. The advance of cutting thinner sections was however not sufficient to provide clarity about the potential existence of "neurolemma", which meant that Villars did not make much progress on answering the question and this then led Keuffel to start applying chemical agents in his research.

[1] "Reils und Authenrieds Archiv" [Archive of Reil and Authenried], Vol. X, p. 161 ff.
[2] "Reils und Authenrieds Archiv" [Archive of Reil and Authenried], Vol. IX, p. 485

Keuffel had already used sublimate solutions and diluted nitric acid to harden the spinal cord, but he used a different method to detect a similar neurolemma component. He placed small pieces of spinal cord in a potassium hydroxide solution for one week or more (one half to 1 dram[1#] to one ounce of water i.e., 2 to 4 grams of potassium hydroxide to 30 grams of water). Then he cut the pieces of tissue into fine sections, floated them on water, brushed them flat and examined them with the naked eye or under a "very sharp" microscope.

*Admittedly, what he [Keuffel] saw was probably not what are now referred to as "neuroglia"*. He was most likely looking at vascular networks of the spinal cord; for, as *Henle* and *Merkel* had previously indicated, in the method used by Keuffel the true neuroglia disappear, while the vessels and the actual connective tissue are preserved. One can easily convince himself when one treats frozen spinal cord sections with the above [mentioned] potassium hydroxide solution and then placed in plenty of water. Keuffel indeed states that the fibers were composed of small spheres which, at the low magnifications available to researchers at the time, could only be red blood cells trapped in the vessels. It is also evident from his drawings that he was not looking at actual neuroglia per se, because it is precisely in the region with the densest accumulation of neuroglia, near the central canal, that his drawings are quite light[2#].

Ascribing the discovery of neuroglia to Keuffel is therefore quite unjustified, but this does not underestimate the fact that his insight into the packing of nerve fibers, embedded in spaces characterized by "compacted cellular material", in other words the "neurilemma", was already a huge step forward. He compared white matter to a Spanish tube[1] in which the longitudinal cavities were filled with nerve fibers. If one considers that 14 years later Rolando (Sulla struttura del midollo spinale, Torino 1824) was still of the opinion that white matter consisted "of a folded medullary membrane whose folded edges lay alternately in the center and in the periphery", it is quite obvious that Keuffel was far ahead of his time. Even though the microscopes of the time were still inadequate to discern the true facts. Friedrich Arnold[2], whose book the quote about Rolando is taken from, agreed with Keuffel's accounts without adding any new findings of his own, and Keuffel's insight was then followed by a 30 year pause until the emergence of the next independent researcher in the field.

[1] Weigert specifies "mit einem spanischen Rohre" (Spanish tube) which refers to reet.
[2] "Bemerkungen über den Bau des Hirns und Rückenmarks" [Observations about the structure of the brain and spinal cord] Zurich, 1839.

It was not until 1846 that Virchow came up with some new observations that really proved the existence of a specific non-nerve tissue component in the central nervous system. **It was only at this point in time that neuroglia were discovered.**

Virchow identified the tissue which he later (1853) called "neuroglia" by starting from the ependyma of the brain ventricles and not from spinal cord tissue. As early as 1846[1] he noted "a completely structureless membrane, which often appeared to be composed of quite regular, very fine and pale fibrils (folds?) arranged parallel to each other" underneath epithelial cells of the ventricle. After adding acetic acid, he sometimes also observed that this membrane included nuclei, but they were usually not present. By "stimulating" the ependyma the familiar pearl-like granulations of this state became apparent, which he likened to the "similar formations" of the Pacchionic granulations, the nodular thickening of the serous membrane. *The ependyma is therefore an independent structure and not, as was argued at that time, an extension of the pia mater or the arachnoid membrane or both.*

Four years after that Virchow even believed that he was able to isolate this ependymal membrane with the scalpel[2] and he later[3] also told Henle that the existence of this skin on a *macroscopic* scale was incontestable — assumptions which of course were soon to be proven untenable.

During the following year he [Virchow] states that the ependyma extends, without a definite boundary, between the nerve tissue components of the central nervous system including the higher sensory nerves, with a "soft general mass of the connective component" that penetrates the nerve tissue components everywhere in this area and holds it all together, so that the ependyma is in fact only the portion of this connective mass which emerges freely on the surface.

He *first* mentions a pathological neoplasm of the connective tissue of the central nervous system in a case of tabes [dorsalis] two years later[4]. In these masses of neoplasm he observed, after hardening in chromic acid, instead of the otherwise fine-grained component, very densely packed, often matted, extremely fine but coarse fibrils. But Virchow did not consider these fibrils as essential components of neuroglia. Even in his 1859 publication (Cellularpathologie [Cellular Pathology] 2nd edition 1859, p. 252 ff.) he still mentions that in some regions, "neuroglia" looked like connective tissue, but that in other regions they have "a very soft texture, so that it is exceedingly difficult to describe their appearance".

[1] "Über das granulierte Ansehen der Wandungen der Gehirnventrikel" [On the granular appearance of the brain ventricle walls] "Zeitschrift für Psychiatrie" [Journal of Psychiatry], 1846, collated publications, p. 885.
[2] "Virchows Archiv" [Archive of Virchows], Vol. 3, p.246.
[3] "Virchows Archiv" [Archive of Virchows], Vol. 5, p. 592.
[4] "Virchows Archiv" [Archive of Virchows], Vol 8, p 540.

He also describes the actual cells as only sometimes appearing star-shaped or spindly, much like actual connective tissue, but otherwise as appearing as very soft and fragile roundish structures.

Virchow was already quite definite about the fact that this connective component would have to be distinguished from normal connective tissue and it is for this reason he coined it "nerve glue". He wanted this name to specifically highlight the more homogeneous nature of this component, in contrast to the typical characteristic fibrous appearance of ordinary connective tissue. It is also notable that Virchow had already observed how easily neuroglia were subject to cadaveric changes, and he had also described the absence of this typical nerve glue in peripheral nerves.

He also already mentioned "that the vessels run within the neuroglia, which are therefore almost everywhere still separated from the mass of nerves by a thin intermediate layer and are not in direct contact with it" (Cellularpathologie [Cellular Pathology] 3rd edition 1859, p. 255[3#]). He also emphasized the association of the central canal to the "central ependymal filament", — *it is simply astonishing what he had already correctly recognized at that time*, even though he only clearly perceived the *typical fibrous* nature of the neuroglia in pathological outgrowths. In spite of all this, one cannot but agree with Deiters[1] who says that all of this early research on neuroglia was "more an intellectual divination than an assertion backed by stringent evidence". Virchow did not provide "stringent proof" that the general mass of the ependyma had characteristic connective matter properties, or that the cells, which he referred to as neuroglial cells, had non-neuron characteristics, indeed no-one at the time could provide this type of proof, for both the methods available at that time as well as the understanding were still very much inadequate. Even though large neurons had already been described and coarse medullary fibers could be detected at the time, neither small neurons, the finer medullary nor the many non-medullary nerve fibrils of the central nervous system could be recognized. That is why Henle was at the time also right to claim that as far as "stringent proof" was concerned, epithelial cells in the brain ventricles did not sit on a connective component but sat directly on nerve tissue.

[1]"Untersuchungen über Hirn und Rückenmark des Menschen und der Säugetiere" [Studies on the brain and spinal cord of humans and mammals], Braunschweig, 1857.

Virchow was however intellectually superior to Henle or let us rather say more "ingenious" in his divination on this issue.

Given the shortfalls of the methods at that time it is understandable that the subsequent period was not marked by significant progress as far as neuroglia were concerned. Bidder and Kupffer were at least conscious that methodological progress was needed and tried to identify features which could help distinguish what should be considered as connective tissue from nerve tissue — whereas Virchow had not yet found any reason to search for such features — but the technical aspects had not yet sufficiently progressed to provide an answer to that question. Bidder and Kupffer[1] initially assumed that neuroglial cells could be distinguished from neurons because neurons turned a yellow to reddish color with chromic acid, while the connective tissue cells remained unstained — a distinction that was recognized as unsound even back then (e.g., by Kölliker). Bidder and Kupffer further sought to highlight the intercellular nature of the connective tissue by proving that the fibers of this intracellular component were associated with other definitely non-nerve tissue components.

Based on this, they considered that the filamentous extensions of epithelial cells in the central canal discovered by Hanover (1844)[2] were in fact connective tissue fibers, which Hanover had still referred to as nerve fibers. These fibers were associated with other fibers which emanated from angular cells that did not stain with chromic acid, and whose fiber extensions also communicated with each other, and this resulted in images "reminiscent of the anastomosing processes of bone corpuscles in thin sections" (p. 45). (An epithelial cell – connective tissue cell association was not considered as strange at the time. In the case of the villous epithelia of the intestine, other researchers in fact also believed that this type of interaction existed).

A second type of association of neuroglia with definite non-nerve tissue components they [Bidder and Kupffer] considered at the transition of fibers from the pia mater to the central nervous system. They believed such fibers to enter the spinal cord on the whole free surface, but then also through the pia process of the posterior and anterior columns. These pia mater fibers pass into the grey matter without distinct delimitations and from there through the processus reticulares into white matter (p. 48). With the exception of neurons, grey matter therefore appeared to them to consist entirely of connective tissue. They described the connective tissue as an either formless, hyaline or granular mass, or a mass containing spiral and elastic fibers, much like ordinary connective tissue (p. 93).

[1]"Untersuchungen über die Textur des Rückenmarks und die Entwicklung seiner Formelemente" [Studies on the texture of the spinal cord and the development of its form elements], 1857.

[2]Hanover is misspelled. The reference is: A. Hannover, Recherches microscopiques sur le système nerveux. Copenhague 1844.

The efforts of these authors to clarify the differences between connective tissue and nerve tissue components of the spinal cord, although very commendable, suffered from flawed technique and gave rise to errors: the pia fibers do not continue into the neuroglia fibers at all, and the grey matter, outside of the "neurons", which was referred to as bodies at the time, still consisted of large masses of nerve tissue.

This grey matter and neuroglia question was until more recently the source of much frustration and caused many unfruitful disputes, extending from the features of the smaller cells contained in it, to the "molecular" spongy intermediate mass. The confusion on the issue was further exacerbated by also including the cortex layer of the spinal cord in "grey" matter, and by attributing the intermediate mass between the nerve fibers of the white matter to the grey matter. When Max Schultze subsequently claimed to have discovered, in the molecular layers of the retina, a network analogous to that of the lymph glands (also with embedded nuclei, which he claimed was typical of grey "molecular" masses (even in the brain), the concept may well have been transferred to all neuroglia masses in general, even to those present in white matter. Kölliker[1] in particular consistently upheld this point of view. He stated that white matter, and grey matter in particular, contained dense networks with embedded nuclei. The nuclei, analogous to the situation in lymph glands, belonged to cells with numerous branched extensions. The network in the grey matter of the cerebrum is particularly dense. The reticulum is connected to extensions of the ependymal cells as well as to the connective tissue of the pia mater, as Bidder and Kupffer had previously assumed to be the case for their intermediate component. The granular layer of the cerebellum and of the hippocampus is particularly rich in nuclei. Kölliker also firmly favored the idea that this reticulum, even if it is related to the pia, is not ordinary connective tissue, and that in general, with the exception of the adventitia of the larger vessels, etc., there was no ordinary connective tissue inside of the central nervous system. He also emphasized, as did Virchow, the correlation between the network and the vessels, for smaller vessels the adventitia only consisted of this type of network "and only rarely did it also additionally contain fibrillar connective tissue".

[1] "Gewebelehre des Menschen" [Human tissue science], citation from the 4th edition (1883), p. 303 ff.

As correct as Kölliker's description may have been, he obviously did not see the actual structure of neuroglia with the method he used and certainly confused all kinds of artificially generated networks, particularly within the molecular mass, with neuroglia. This is not concluded based on the fact that neuroglia form a reticulum i.e., made up of anastomosing strands, because if he had seen the correct structure, the assumption of anastomosing strands would have been a very minor issue, but his illustrations clearly prove that he was not looking at pure forms of neuroglia at all. Figs. 166 and 167 are images that do not occur in white matter, because the spaces between the nerve fibers are filled with a completely diffuse mass. Fig. 168 is also an illustration that could never be obtained for a neuroglial structure, but rather represents the structure of some artificial product. The networks which he noted in grey matter can also be considered as artifacts, since neuroglia are extraordinarily sparse in the very places where he found the reticula to be particularly dense and abundant (cerebral cortex, granular layer of the cerebellum). In general, at that time (in Germany at least) nobody had seen the real structure of neuroglia. Stilling indeed denied the presence of a "connective tissue" component in the central nervous system altogether.

In contrast, J. L. Clarke[1] had at least approximately observed them in the spinal cord by 1859, in England. This was certainly the case for the cortex layers of the spinal cord, which he quite correctly described as containing groups of interwoven fibers mainly running parallel to the surface (p. 441). He also states that the fibers bend into the white matter, which they penetrate to join a similar network in grey matter. According to Clarke, connective tissue cells have differently shaped nuclei, and are either surrounded by a granular component or the nuclei are directly attached to the connective tissue fibers (p. 442): "*in adults the cell bodies have disappeared and only the nuclei remain*".

But Clarke was quite aware the time was not yet right to clearly differentiate between nerve tissue and connective tissue components, he therefore concluded (p. 442) with the following words: "These observations render it apparently impossible, to print out the exact distinction between the connective and the nerve tissue, and might suggest the question, whether there is any actual and essential difference between them or whether the connective tissue of the cord be intermediate in its nature passing on the one hand into nerve-tissue and on the other into the pia mater.[2] —

[1]Philosophical transactions, 1859, p. 437 ff.
[2]We will discuss some of Clarke's interesting and correct observations about the epithelium of the central canal in the specific topography chapter.

Proper pictures of the neuroglia of the spinal cord, at least in white matter and around the central canal, were then described by a researcher who (with Clarke) opened up a new era in the history of neuroglia, namely *Frommann*[1], whose work is treated as quite trivial in almost all historical accounts. The reason for this probably lies in the fact that it is sheer torture to *wade* through the horribly verbose descriptions of the most minimal details, through the author's immensely unclear long-winded discussions, which means that only a few have possibly managed to read Frommann's works at all or even to separate the wheat from the chaff in the process. But having read Frommann's work, one finds that this apparently excellent observer, who admittedly only worked on the spinal cord, had actually described everything that could be seen using the quite unreliable carmine method. *His descriptions and illustrations of neuroglia in white matter and around the central canal are positively exemplary for the time.*

For the question at issue here, it is all but irrelevant whether Frommann considered the fibers to be hollow or solid, branched or not branched, anastomosing or non-anastomosing, independent structures, or cell extensions: because he *saw the correct fibers*, described them *first* (perhaps with the only exception of Clarke) and *observed them in their most complete form*.

With the carmine method used by Frommann, the fibers appear as cell processes and Frommann is quite clear about this. He says (p. 45 f., part I): "It is not possible to directly prove that the cell extensions continue into the fibers; it is possible to follow some of them that do not split and do not decrease in thickness over longer distances, but nothing can be determined about their further fate. But because the fibers and cell processes appear the same and respond the same way to carmine, with the thicker ones staining, and because the same differences in size exist between the finer and coarser fibers as between their extensions and their ramifications,

[1] "Untersuchungen über die normale und pathologische Anatomie des Rückenmarks" [Investigating of the normal and pathological anatomy of the spinal cord], Part I, Jena, 1864. Part II, Jena, 1877.

*I believe that the fibers all emerge from cell extensions* and, are hollow like them, and that the whole connective component of white matter therefore consists of an interconnected network of variously-sized tubules, for which the numerous intercalated cells act as collecting and central points."

Frommann is even more specific in Part II, p. 9.

If we disregard the ambiguous arguments of Clarke, then Frommann was the first to consider the *right neuroglia fibers as cell extensions* (given that Kölliker was looking at neuroglia artifacts).

He argues quite pertinently that neuroglia are independent of the pia mater, even at the pia processes.

He also describes the region around the central canal, not only as correctly as it was possible at that time, but he was also the first to describe that *neuroglial fibers radiate out between the cells of the central canal*.

He was also the first to clearly observe the cadaveric disintegration of neuroglial fibers into granules (Part I, p. 49).

Admittedly, he was not as happy with the grey matter. He complained that he could not distinguish the fine axes cylinders [axons] from the fibers of the connective component. This does not however in any way diminish his great achievement of having seen a lot of things correctly for the first time, because his imperfect methodology was surely to blame. In *our* description of neuroglia, we will often refer to *Frommann's* results for comparison. —

Our understanding of neuroglia was further advanced by the famous investigations of Deiters, which lose nothing of their value by the fact that they were only published as fragments posthumously.

Deiters,[1] like Bidder and Kupffer, initially addressed the important preliminary question of what components of the central nervous system should be considered as nerve tissue components and what should be considered part of the non-nerve intermediate tissue or connective tissue mass.

He said quite genuinely that one must not start with a simplistic preconceived perception of connective tissue when assessing these associations. He writes for instance that "whoever considers connective tissue to be a fibrous mass and expects to find star-shaped cell bodies between the fibers regardless of the context, will expose themselves to two dangers, either to doubt the widespread presence of connective tissue altogether, or to seek to recognize this characteristic everywhere, by labelling any star-shaped ganglion cell as connective tissue for example." (p. 28)

[1] "Untersuchungen über Gehirn und Rückenmark des Menschen und der Säugetiere" [Studies on the brain and spinal cord of man and mammals], Braunschweig, 1865.

Deiters quite rightly did not expect the supporting components of the central nervous system to have the same characteristics as ordinary (what we now call collagenous) connective tissue and believed that in some instances one should be content with just proving that specific components cannot be nerve tissue, but should instead be considered as interstitial tissue components, which may differ from ordinary connective tissue.

Like Bidder and Kupffer, Deiters initially assumed that real connective tissue can also penetrate into central organs i.e., like the connective tissue which seems to be associated with the pia mater. He believed that this type of interstitial tissue component does not occur everywhere, but only in specific regions, like the Müllerian fibers in the retina for example. "It occurs most prominently and adopts its most characteristic appearance, in the white matter of the outer periphery of the spinal cord. Here a tissue extraneous to nerve fibers is known to pass through nerve fiber bundles in dense masses, and more or less closes up almost every primitive nerve fiber." (p. 36.) Deiters also allowed the fibers of the pia to enter grey matter at the surface of the cerebellum, like Bidder and Kupffer had done for the spinal cord, where they considered pia processes of the anterior and posterior fissure to radiate out into the grey matter. Located here are the radial fibers which Bergmann discovered in Greifswald as well, and which are now referred to as "Bergmann's fibers" in his honor[1]. It must be noted, that although Bergmann's paper was published prior to Deiters' work, Deiters must not have been aware of it, and should therefore be considered to have co-discovered these fibers with Bergmann, even though Deiters does erroneously consider these fibers as pia processes. As previously mentioned, Deiters also included central canal epithelial cell processes and the ependyma in the pia processes category.

In terms of what has been discussed so far, Frommann's work, which Deiters also must not have been aware of, was much more advanced.

[1] Deiters was obviously looking at the right fibers, but I still doubt whether Bergmann was really looking at true "Bergmann's fibers". Refer to the cerebellum section of our chapter on the specific topography of neuroglia. Bergmann's work is published in the "Zeitschrift für rationelle Medizin" [Journal of Rational Medicine], new series, Vol. 8, p. 360.

Frommann had correctly recognized that neuroglial fibers in the white matter of the spinal cord were independent from fibers in the pia mater, and he had already expressed the opinion that these fibers were also identical to the so-called cell extensions, which are facts that completely eluded Deiters.

As we now know, Deiters also erroneously believed to have excluded all doubt about the association of the fibers with the connective tissue of the pia. He found the question of other potential interstitial tissue components more difficult to address but figured out a way to handle this as well. According to Max Schultze's teachings, all modified protoplasmic masses which had become independent from cells and could therefore no longer be considered to belong to any cell, had to be regarded as interstitial tissue components. Max Schultze's view is quite correct per se, but its application to the issue at hand was premature, — here, too, the methods were inadequate to guard against errors. Deiters therefore also succumbed to these kinds of errors by classifying the "spongy-porous", molecular component (previously discussed on p. 39 and which we will discuss again later) as a secondary interstitial tissue component. The "spongy-porous" component was considered as the "main mass" of grey matter, in which neurons and isolated nerve fibers are embedded, but this "spongy-porous" component was also said to occur in white matter. According to Deiters, the mass could not be classed as nerve tissue at all, because it did not isolate nerve fibers; but rather, as an interstitial tissue component, because in its mature state it is completely independent of the cell bodies, a view which was consistent with Max Schultze's definition. As both neurons and free nuclei could give rise to the mass, which will be mentioned again later, from a purely developmental perspective the mass was therefore considered as something neutral which separated neurons from connective tissue. By gradually becoming independent from both neurons and connective tissue cells, it eventually developed into a true intercellular tissue component, a binding substrate in its own right.

Results obtained using the Golgi method have of course proven this assumption to be quite wrong. The "spongy-porous mass" is not spongy-porous at all, it is not independent from cells at all, but is an enormous tangle of cell dendrites and axis cylinders, which may insulated connections are possible.

In addition to true "connective tissue" fibers and the porous general mass, Deiters also included cells as a third connective tissue component.

Here, too, Deiters adhered to the teachings of Max Schultze, and also used the isolation method developed by Schultze. This method consists of concomitantly slightly hardening and macerating pieces of central nervous system in weak chromium solutions.

Max Schultze had already published his acclaimed work on the cell at that time and had reported that connective tissue cells had a rudimentary, protoplasm-poor appearance. This of course also holds true for ordinary, normal, non-pathologically modified connective tissue today, but Deiters went one step further. He not only assumed that connective tissue cells had small protoplasms, but also assumed that cells with small protoplasms were connective tissue cells. He coined the term "cell equivalents" to describe cells with little or no apparent protoplasm i.e., cells with no "distinct character". Deiters considered these "cell equivalents" to be connective tissue cells, as he believed to have observed (refer to p. 48) that all central nervous system cells associated with nerve tissue components definitely had more developed, more solid protoplasm. Based on these assumptions, he mistook the "granules" in the cerebellum and in the hippocampus, as well as all the other "free nuclei" of the central nervous system as they were called at that time, to be connective tissue. He did, however, correctly identify one type of cell as "connective tissue" i.e., *those structures which are still referred to as Deiters cells today*.

Deiters describes these cells as cell equivalents, with only sparse protoplasm (i.e., true granular protoplasm) surrounding the nucleus, with long, more or less modified smooth processes, which gives them the general appearance of fibrous structures in specific contexts (p. 38). These emerging processes have a firm, albeit delicate, appearance, a quite sharp, smooth contour, and considerable luster right from the start. They radiate out, in all directions, to form large masses, and branch out in the most diverse ways, but always as fork-like bifurcations (p. 45). Deiters observed these cell equivalents in both grey and white matter and considers most apparent central nervous system fibers to be "extensions" of these cells (with the exception of the large radiating masses mentioned above). He also erroneously reported them to be particularly abundant in the substantia gelatinosa of Rolando.

Although the author of the current account cannot confirm that these strange radiating formations, the Deiters cells, were interpreted entirely correctly by Deiters himself, it is undeniable that their *discovery represented a significant advance* in the field. Irrespective of how they were interpreted, when detected with due diligence, the presence of these characteristically shaped cells allowed one to identify neuroglia even in complex regions such as in the white matter of the spinal cord. The true topographic distribution could not be determined from picked and fragmented tissue. Whether they could even have been detected with better methods from tissue sections will be discussed later. —

At this point we also wish to add Henle's views. His work with Merkel[1] is interspersed with controversial arguments that are in part even difficult to confirm today, which means that the authors' views are not very clear. We will nevertheless revisit the important chemistry aspects of this work later. What follows is the account by Henle from the first edition of his renowned "Handbuch der systematischen Anatomie" [Handbook of Systematic Anatomy] and the section entitled "Nervenlehre" [Neuroscience][2].

Henle first of all defines the diffuse, finely granulated mass as an interstitial tissue component. This component forms the outer layer of the cortex of the cerebrum and the cerebellum, as well as the thin cortical layer of the spinal cord, which sparsely surrounds the central canal and represents the peripheral part of the posterior grey columns of the spinal cord (Substantia gelatinosa of Rolando). Nowhere does it appear independently, and besides neurons, it also contains "granules" that are similar to lymph corpuscles. *It appears in its purest form in the substantia gelatinosa of Rolando*, connective tissue fibers are also present, but difficult to distinguish from naked axis cylinders, since incubation in potassium hydroxide solution dissolves both of these structures. These connective tissue fibers are very prominent in the outermost layers of the cerebrum and spinal cord. But they belong to a different type of connective tissue fiber than, for example, the connective tissue of the pia mater, which they merely have contact with. They belong to the type of connective tissue fibers that are matted and whose stiff fibrils emanate in manifold directions from small multipolar cells.

[1] "Über die sogenannte Bindesubstanz der Zentralorgane des Nervensystems" [About the so-called connective tissue components of the central organs of the nervous system], "Zeitschrift für rationelle Medizin" [Journal of Rational Medicine], Series 3, Vol. 34.
[2] Braunschweig, 1871.

Henle thus apparently identified the right fibers in only very few places, in many places, such as around the central canal, etc., the fibers appeared to him as a diffuse finely granulated mass, which is otherwise not present at all in these regions. —

The next researcher who essentially confirmed Deiters' data and who expanded on Deiters' fragmented communications was Golgi.[1]

In terms of the actual distribution of cells, he deviates from Deiters on only a few, rather insignificant points. Golgi observed more processes per cell, but very little branching, and short branches (p. 8), as opposed to the multitude of branches attributed by Deiters. Gogli also disagreed with the anastomoses, which, by the way, Frommann already doubted, etc.

But he agrees with Deiters about the main point that such isolatable cells endowed with long processes are characteristic of neuroglia. Golgi admittedly only mentions Deiters in a note (p. 31), where he refers to deviations from his views that are not further specified.

In other respects, however, Golgi saw more than Deiters. Most notably he succeeded in identifying the characteristic "Deiters cells" from tissue sections and not only from isolated tissue samples. Golgi considered the structures that Deiters referred to as "cell equivalents", to be real cells. This was a similar interpretation to that of Frommann, who had however not yet noticed their characteristic "branched" forms.

He also investigated the relationship of these "cells" to the vessels in more detail. Virchow had already demonstrated that the vessels have a neuroglial sheath, but Golgi confirmed it in a very distinctive way, noting in particular that more distant cells send their "extensions" towards the vessels. Golgi also correctly emphasized that neuroglia in the cerebral cortex of *older individuals [humans] is much more strongly developed than in younger individ*uals.

At that time, however, the methods he used were not yet robust enough to guard against misinterpreting the distribution of neuroglia. Even his illustrations of white matter of the spinal cord, as far as the fibers ("cell extensions") are concerned, are very much inferior to Frommann's.

[1]"Beitrag zur feineren Anatomie des Zentralnervensystems" [Contribution to the finer anatomy of the central nervous system], Bologna, 1871. Cited from the "Untersuchungen über den feineren Bau des zentralen und peripherischen Nervensystems" [Studies on the finer structures of the central and peripheral nervous system], Jena, 1894, p. 1. ff. In the interest of space, we will hereafter refer to these "investigations" as "collated papers".

His description of spinal cord grey matter was equally flawed because he considered the substantia gelatinosa of Ronaldo to consist almost exclusively of neuroglia (p. 34).

He was the first to quite correctly observe a series of more vertical fibers which descend from the more tangential neuroglial fibers of the superficial layer, in the cerebral cortex. He also quite correctly identified that many more neuroglial cells are present on the surface and progressively fewer deeper in the cerebral cortex (p. 7), from osmium preparations. Golgi already states in Paragraph 2 of the following page "that cells sharing the same characteristics are scattered in considerable *numbers over all layers*[1] *of the cerebral cortex, where they form a support tissue*". He emphasizes this point even more poignantly (p. 9 f.) when describing sections treated with his own modified bichromate method. And I quote: "...at the edges of the sections and at their thinnest part, the interstitial stroma appears to be *distinctly fibrous even in the deepest layers of the cerebral cortex*, not reticular as Schultz and Kölliker had reported. This is not intended to dispute the concomitant presence of an amorphous, finely granulated intercellular tissue component in all the preparations I have described, for I have always found traces of such a component, and some of this component must been removed from my specimens. However it seems obvious to me that the multitude of names given to this component: finely granulated, reticulated, spongy, or dotted molecular, amorphous, or gelatinous, reflect changes in the cadaver examined or the tissue preparation. . . . *This all seems to indicate that the interstitial stroma of the cerebral cortex largely consists of connective tissue cells and their processes*."

This may suggest that the connective tissue cell processes decay just like the very fine protoplasmic processes of neurons and that both decay products give rise to the molecular component.

Even Golgi is still haunted by the specter of the interstitial nature, at least of a large part of the "spongy" component of the cerebral cortex, a specter which he exposed with the method later invented by him, although Golgi himself still maintained in 1885 that the deepest layers of the cerebrum appear as he described them in 1871.[2]

[1] Meaning not merely over the surface which he mentions in Paragraph 1.
[2] Collated papers, p. 162.

Golgi's assertions about the molecular layer of the cerebellum were also partially incorrect, although he did of course confirm the presence of the Bergmann fibers, and described them correctly, as did Deiters (as opposed to Bergmann), etc. But Golgi also assumed that the molecular layer of the cerebellum consisted of stroma made up of associated connective tissue cells with numerous projections. He believed that all nuclei scattered in the molecular layer belonged to these connective tissue cells (p. 17).

Like all other parts of the central nervous system, he also considered the granular layer of the cerebellum to be associated with a stroma consisting of connective tissue cells with numerous long processes, which never or rarely branch. Golgi even believed that the granules themselves were connective tissue components, whose processes frequently extended into the true connective tissue cells. (p. 21.)

Golgi himself later corrected some of his errors concerning the cerebellum by applying his new method, but he did not change his description of the rich neuroglial scaffold in the granular layer, even in 1885.[1]

But most influential, and perhaps most disastrous, were Golgi's views on the relationships of the fibers to the cells. It is true that Frommann had already expressed similar views — but these received very little attention (Golgi also only mentioned Frommann in passing), Deiters' results were also too vague, because he was cautious not to mention cells, but used the term "cell equivalents" — and so it was Golgi, whose work mentioned here and more specifically his later work that paved the way for the acceptance of Deiters cells together with their extensions as real cells, and the dismissal of the concept of secreted fibers as well as the rejection of the entire neuroglial scaffold as nothing more than a network of extensions of these cells. The extent to which the enormous authority of Golgi has come to the fore is also evident from the fact that "Deiters cells" have recently been renamed "Golgi cells". — Jastrowitz[2] incidentally gives a similar description of Deiters cells as Golgi (and independently of the latter), without mentioning Deiters.

[1] Collated papers, p. 167.

[2] "Über Encephalitis und Myelitis im ersten Kindesalter" [On encephalitis and myelitis in early infancy], "Archiv für Psychiatrie" [Archives of Psychiatry] (Vol. 2, p. 389 ff. and Vol. 3, p. 162 ff.)

Jastrowitz coined the term "*spider cells*" to describe Deiters cells, but he also assumed that the *square and rectangular cells arranged in rows belonged to neuroglia*. According to him, these types of cells are found in the white matter of the brain (he only worked on the brain), and he believed them to be rudimentary spider cells. He did not consider the "molecular" component of the cerebral cortex to be neuroglia but believed it to be "*more similar to nerve tissue than to connective tissue*." He therefore also separated it quite correctly from the spinal cord support layer. His description of the relationship of the ependyma to the neuroglia is very strange and given our current understanding, extraordinarily paradoxical. The relevant passage is quoted verbatim:

"These (spider) cells are more and more abundant towards the ventricle cavity; they are densely packed one behind the other, with their processes generally extending in the posterior and lateral direction, eventually, they *rest next to each other one by one, to form the epithelium of the ependyma*. Thereby they undergo a modification insofar as the processes at the free end vanish and are replaced by a dual contoured, often rather broad and mostly unstained hem. The sparse lateral and posterior extensions are drawn out towards the third layer (of the columns) and are particularly delicate, readily breaking off. One type of extension is notable, because of its *vigor, and is often the only one preserved, with the cup-like (cylindrical) epithelial cells clinging on to it, as to a stalk*."

"Every detail of the identity of glial cells and their relationship to the ventricle epithelium is described here, and the *connective tissue nature of glial cells appears undeniable. It is therefore quite rightly referred to as an epithelium spurium or endothelium*."

Jastrowitz consequently interprets the relationship of the ependyma epithelium to the neuroglia exactly inversely to the current convention. Whereas neuroglia are to date attributed epithelial characteristics owing to their close developmental association with the ependyma epithelium, Jastrowitz, on the contrary, considered the ependyma to be a connective tissue entity, an endothelium: this was an altogether new concept, because the distinction between epithelium and connective tissue had not even dawned on histologists before that time. —

The work of Boll[1] we will mention now took account of the publications from Deiters and Golgi. Boll, like Deiters, argued even more strongly for the "differentiated nature" of the fibers. When comparing the cells seen by Deiters with those of (embryonic) connective tissue, Boll said (p. 8):

"In both cases, the cell, the central histological part, is nothing but *a center* for a multitude of *differentiated* fibers projecting to all sides, to two sides, or to one side. The nucleus is in both cases in the center of this cell and surrounded by a larger or — as in the vast majority of cases — smaller amount of the granular component. Both cases need to be investigated to address whether this multitude of granular aggregates which surround the center of these convoluted fibers and include nuclei, should be considered as viable, efficient protoplasm or amorphous protein components."

This concept, as we will see later, represents a substantial advance with respect to Golgi, who still considered the entire structures as real cells and in his later work even went as far as to criticize Deiters for using the carefully chosen "cell-equivalents" term to refer to these structures. The key step on this issue, as we shall see later, was provided by Ranvier.

Boll, by the way, was quite right to make the distinction between neuroglia and ordinary connective tissue, undeterred by the similarities between these two types of tissue both in terms of development and chemistry.

Like Jastrowitz, Boll also assumed that besides the cells described for the first time here as "Deiters cells", there were in addition rectangular cells arranged in rows[4#]. Boll also described that there are transitions between the two types of neuroglial cells — but his description of white matter is incomplete. Boll considers 50-60 nerve fibers in white matter of the brain and 5-6 nerve fibers in white matter of the spinal cord to rest together in a neuroglia sheath, he does not however truly believe that the transverse fibers in white matter of the spinal cord are really neuroglial fibers, etc. Boll was quite right to be cautious to avert any confusion with free axis cylinders, which are indeed present (as collaterals) in this region.

[1]"Die Histologie und Histogenese der nervösen Zentralorgane" [Histology and histogenesis of the nervous central organs], "Archiv für Psychiatrie" etc. [Psychiatry archive], Vol. 4, 1874, p. 1 ff.

His description of neuroglia in the cerebral cortex is more accurate than Golgi's interpretation. He correctly emphasized the multitude of Deiters cells which only occur on the surface, but that they are much scarcer in the deeper layers and generally appear only along with the vessels. He was therefore not aware of the "composite" neuroglia network in the deep cerebral cortex layers that Golgi had suggested. Boll also assessed the granular layer of the cerebellum more accurately than Golgi, by saying the only thing that could be surmised at that time, namely that nothing was known about the nature of the "granules". —

The work from Gierke[1] rates a mention as it is the latest work from this group. Gierke's work could actually be omitted from our historical overview, since it does not contribute anything substantially novel that was confirmed to be correct. With the exception of relatively few correct data, all of which pertain to previously established issues, the work consists of virtually entirely erroneous assertions. It is therefore incomprehensible that the most outstanding authors invariably praise the work as "thorough", and "excellent" etc. It is indeed one of the ironies of historical accounts that the work of Frommann is consistently mentioned in passing, and that Gierke's work is always highlighted as excellent. The following are but a few examples of his work.

Gierke gives a similar description of Deiters cells as Golgi, Jastrowitz and Boll with the minor difference that he lets the "cell processes" be branched, and that he considers them as "horny" (like Kühne and Ewald). In addition to suggesting that the bodies and nuclei of Deiters cells may atrophy in old age, Gierke also considers that neuroglia contain a "general component", which is not granular as the older research described, but is as translucent as glass. According to Gierke this vitreous general component forms the basis of grey matter. This component is particularly abundant and well-developed in the outer sheaths of the central nervous system, in the cerebral cortex and in the substantia gelatinosa centralis. It occurs more parsimoniously in white matter (p. 459) — all these are quite arbitrary and unsubstantiated claims. According to Gierke, the "general component" exhibits a not entirely insignificant elasticity (p. 464), but only in fresh tissue.

[1]"Die Stützsubstanz des Zentralnervensystems" [The support component of the central nervous system], "Archiv für mikroskopische Anatomie" [Archives of Microscopic Anatomy], Vol. 25, p. 441.

It starts to soften a few hours postmortem, which, according to Gierke, causes softening of the central nervous system — otherwise it is assumed that this cadaverous softening is due to the softening of myelin.

According to Gierke, the entire support component (the "general component" plus the neuroglia) is so widespread that it occurs everywhere in the central nervous system and no region, no matter how small, is found to lack it" — this is also an unsubstantiated assertion. Gierke's description of the white matter of the spinal cord is mostly, though not completely, correct, but it comprises nothing that Frommann had not already described better. Gierke's description of the grey matter suffers from the same misconceptions as earlier accounts. His description of the situation at the medulla oblongata is quite obscure and misleading, and he omits to discuss the olivary body, which are quite striking; he only describes the ependymal layer better than his predecessors.

As for the brain, all his new descriptions are quite wrong, the cerebellum is so flawed that even his illustration (Fig. 21) fails to show what he actually saw. He also did not even recognize the true neuroglia in the cerebral cortex. His illustrations (Fig. 19 a) show a molecular component that has shrunk down to a meshwork. This fact is not only evident from his own illustration, but also from the fact that he refers to a similar image by Stricker as a "very accurate" representation. —

This figure is included in two papers, one co-authored by Stricker and Unger "Untersuchungen über den Bau der Großhirnrinde" [Investigating the structure of the cerebral cortex][1] and the other authored by Unger alone ("Histologische Untersuchungen der traumatischen Hirnentzündung" [Histological analysis of traumatic encephalitis]). As far as this work is concerned, it probably suffices to quote the concluding remarks of the co-authored Stricker and Unger paper (p. 156):

I. The ganglion cells and their axis cylinder processes (!) bear extensions, which continuously project into a network of connective tissue components.

II. There are transitional variants between the connective tissue component cells and ganglion cells.

Those among you who have still not had enough of these types of statements may like to refer to the two previously mentioned papers, as well as the 32nd lecture in Stricker's "Vorlesungen über allgemeine und experimentelle Pathologie" [Lectures on General and Experimental Pathology] for themselves. —

[1]"Wiener Sitzungsberichte" [Vienna conference proceedings], Vol. 80, 1879.

The concepts, already expressed by Boll, could now be bestowed a much stronger foundation in the important and downright era-changing work of Ranvier[1]. What Ranvier reported was considerably more important than debating the details about more or less abundant extensions and whether these extensions branch or not, etc.

Ranvier was almost predestined to clarify the true nature of Deiters cells, as his prior work on ordinary connective tissue had all but compelled him to assume that a similar relationship of cells and fibers existed in the support tissue of the central nervous system. He did not simply make an assumption but provided concrete evidence which supported the fact that the Deiters cell was an artifact. Although the fibers of Deiters cells are independent of the cell and project out from the cell with the cell as a central focal point, they only appeared to originate from the protoplasm, whereas in reality the fibers lean against the protoplasm.

His achievement was facilitated by a specific method and, as we will see in a moment, by a carmine solution with a very favorable effect.

This method consisted of placing pieces of spinal cord in one-third alcohol for 24 hours, breaking them up and shaking the fragments in a test tube of distilled water, then staining them with picrocarmine, and allowing them to settle. He subsequently collected the pellet with a pipette and transferred it to a new test tube containing a very dilute osmic acid solution prepared from a saturated stock. He waited for the solids to settle out again, collected them and examined them under the microscope. This allowed Ranvier to first dissociate, stain and subsequently to definitively fix the dissociated components.

Spinal cord specimens prepared in this manner allowed him to observe that the "cell processes" were not real protoplasmic body elongations, as all authors since Frommann believed (except for Boll), but that they were in fact real fibers that are independent of the protoplasmic body, and that penetrate the cell body, or lean against it. They radiate out from the center of the cell body in all directions (and do not branch further). But the cell body itself does not simply merge into them, instead it exists as a chemically and morphologically separate body.

This notably only applies to neuroglial cells of the adult spinal cord.

[1] 1)"De la névroglie" [About neuroglia], Comptes rendus [Proceedings], June 5, 1892. 2) "De la névroglie" [About neuroglia], "Archive de Physiologie normale et pathologique" [Archive of Normal and Pathological Physiology], February 15, 1883. Citations in the text were taken from the published work, the verbal quote was merely a preliminary communication.

In the embryo, the cells are really star-shaped, and the processes simply elongations of the cell body. Differentiation of the embryonic fibers only occurs later, as is the case for ordinary connective tissue. —

This provided a whole new insight of the neuroglial scaffold. According to Ranvier, this scaffold did not consist of cells alone, but of cells as well as fibers. Ranvier also correctly pointed out that the previous results inevitably led to the misleading notion that cells and fibers were one and the same, because in specimens prepared with Mueller's solution the refraction indices of the fibers and the cell body are so similar that a distinction of the former from the latter was not possible.

Admittedly, the method of fragmenting the specimen was not adequate to clarify the topography of neuroglia, and it even led Ranvier astray in other regions of the central nervous system, so that he expressed the quite erroneous view that the neuroglial fibers of the adult brain did not appear to arise from embryonic differentiation i.e., from undifferentiated cell processes. (p. 182.)

There was absolutely no appreciation of Ranvier's view. With the exception of some scholars affiliated with Ranvier, only the author of the current work has spoken out completely in favor of Ranvier's view. The merits of Ranvier's contributions were only to be fully appreciated later.

Schwalbe[1], whose work we wish to present here, occupies, or at least used to occupy, a special place as regards the neuroglia question. He makes a distinction between (p. 393) a mesodermal and an ectodermal portion of the support component in the central nervous system. Apart from the odd elastic fiber (or fibers in proximity to the elastic fibers), Schwalbe considered the mesodermal portion to primarily include neuroglial cells, which he positioned adjacent to migratory cells. According to Schwalbe, neuroglial cells have no extensions, but also no connection with glial intercellular components, which means that his view is quite different to the interpretations of Frommann, Deiters, Golgi, etc., as well as Ranvier. What Schwalbe refers to as the intercellular component he considers to be of ectodermal origin, just like the epithelial cells of the central canal. This intercellular component is found in two forms. The first is nerve glue (true neuroglia). This is a completely homogeneous, soft component and contains no fibers in its natural state. The fibers observed by other authors are artifacts produced by cadaveric coagulation or by coagulating agents e.g., alcohol.

[1]1) "Handbuch der Augenheilkunde" [The handbook of ophthalmology] by Gräfe and Sämisch, Vol. I, p. 342, Leipzig, 1874. 2) "Lehrbuch der Neurologie" [Textbook of neurology], Erlangen, 1881 p. 393 ff.

This component is very similar to an epithelial glue. It also turns brown when stained with silver nitrate, like epithelial cells. An additional ectodermal support component, which Schwalbe referred to as the "granulated component", consisted of very fine, tightly interwoven filaments, which he therefore assumed to simulate granulation. It is found in specific layers, in various parts of the spinal cord, on the surface of the cerebrum and cerebellum, and in the retina. This compound defines the horn spongiosa as reported by Ewald and Kühne.

It hardly rates a mention here, and we will prove in the next few sections, that these views cannot be upheld. Schwalbe himself may have changed his mind about these issues by now. After all, it is remarkable that like Ranvier, he considered neuroglial cells to be devoid of processes. Schwalbe did however not see the filaments that Ranvier had illustrated.

Of the new generation of authors who have worked with methods other than the previously mentioned ones (with the exception of the Golgi method), Luigi Maria Petrone should be mentioned first. He was the first to use acid fuchsin and picric acid to stain neuroglia, a stain that was later (1889) further developed by van Gieson[1]. Van Gieson's method was subsequently very slightly modified by Kultschitzky[2]. Petrone[3] also worked with the Golgi impregnation and with carmine picric acid.

[1] Laboratory notes of technical methods for the nervous system, New York medical Journ., 1889.

[1] "Über eine Färbungsmethode der Neuroglia" [On the neuroglia staining method], "Anatomischer Anzeiger" [Anatomical index], Vol 8, 1898.

[3] "Gazzetta degli Ospidalii", 1886—1886, "Gazzetta Lombarda", 1886—1887. (Preliminary communications, not amenable to me). "Sulla struttura della nevroglia dei centri nevrosi cerebro-spinali, Gazzeta degli Ospidali", 1888. The title page of this latest work reads: Senckenberg's Pathological Institute of Frankfurt a. M., Prof. Weigert, and is dated in Breslau, where Petrone was staying at the time. One may conclude from this that I would have some merit in this work, particularly because Petrone claimed to have clarified the structures of the medulla oblongata, the isthmus of the brain and all parts of the brain himself, which may lead some readers to believe that I at least contributed my share to the results on the spinal cord etc. But that is also not quite true. Not only was I not involved in the work, but I also did not even see the preparations in question. Yes, I must expressly emphasize that I have only very recently become aware of Petrone's paper, otherwise I would certainly have given him priority in my "Technique" article which describes the Merkel-Bonnet's "Results" as regards the fuchsin acid-picric acid staining. The reasons for these most peculiar-appearing circumstances were regrettable, but eluded general public knowledge.

For his actual staining, Petrone used specimens that were prepared in the same way as for my copper hematoxylin method.

Petrone distinguished between two types of neuroglial cells, the actual Deiters cells and "lamellae", flat rectangular cells without extensions, which occur primarily at nerve fiber junctions. He disagreed with Ranvier about Deiters cells, because he erroneously believed that Ranvier mistook Deiters cells for the flat cells associated with neuroglial fibers. Petrone disputed that neuroglial fibers in white matter anastomose, but believed that the "Schultz-Kölliker network" was present in grey matter. Admittedly, he was aware that his method, which does not stain selectively at all, is unsatisfactory to investigate grey matter "on account of the technical inability to distinguish neuroglia from the other surrounding components".

Petrone in fact also considered the substantia gelatinosa of Rolando to contain more neuroglial cells than the remaining grey matter, which is quite erroneous. He did not identify any actual neuroglia in the most superficial layer of the cortex of the cerebellum and cerebrum but did find "lamellae" and connective tissue descending from the pia mater. Petrone missed the dense accumulation of neuroglia at the ependyma.

Curiously, he observed the dense neuroglia mass in the olivary body, which no one else had reported prior to my 1890 publication, and I regret that I was not aware of his work in 1890 (see note p. 24), otherwise I would have already acknowledged it at that time.

Although Petrone may have described white matter quite correctly, what he actually observed is somewhat uncertain, as he does not include any illustrations of it.

I published a preliminary communication[1] about my new staining technique in 1890, which was at the time still in its "nearly finished" stage, and results were far too preliminary for a detailed publication. But I was nevertheless able to disclose some pertinent results at that time. My results certainly allowed me to agree with Ranvier's view, discussed above, that neuroglial fibers are not "cell processes". My data on the topographic distribution in the grey matter of the spinal cord, namely the reduced numbers of neuroglia in the substantia gelatinosa of Rolando, was completely novel.

[1]"Bemerkungen über das Neurogliagerüst des menschlichen Zentralnervensystems" [Observations about the neuroglia scaffold of the human central nervous system], "Anatomischer Anzeiger" [Anatomical index], 1890, p. 543 ff.; and: "Zur pathologischen Histologie des Neurogliafäsergerüsts" [On the pathological histology of the neuroglial fiber scaffold], "Zentralblatt für allgemeine Pathologie und pathologische Anatomie" [Reference journal of general pathology and pathological anatomy], 1890, p. 729 ff.

The granulations at the free edge of the epithelia had also not been described before, likewise (except for the note in Petrone's publication, which I was not aware of at the time) the extraordinary abundance of neuroglial fibers in the olivary body. I briefly described the characteristics of the cortex layers, the substantia grisea centralis, the obliterated central canal of the spinal cord, and also emphasized, as earlier authors had done, that the "processes" projecting into the pia were not connective tissue but neuroglia and described a novel stain which highlighted the differences and expanded on the previously appreciated differences between connective tissue and neuroglia. I also published sketches of the "basket" [cells] surrounding Purkinje's cells and anterior horn cells, as well as sketches of the opticus, etc.

Lavdowsky[1], whose work appeared the following year, used an extensive number of methods (including the Golgi method), which predominantly used "acid" aniline stains. Lavdowsky must have been unhappy with the quality of the sections because he only partially illustrates the topography. He considered neuroglial fibers to be hollow and believed them to be true cell extensions (in contrast to Ranvier). Lavdowsky reported neuroglia to form veritable "networks" in grey matter, but he did not observe such structures in white matter, which means that his views are similar to Petrone's. He missed the differences in the topographic dispersion of neuroglia in the different parts of grey matter. He also laments the fact that neuroglial fibers cannot be distinguished from neurons and nerve fibers where they intermingle with neuronal processes (p. 239).

Popoff's work[2] using the van Gieson method modified by Kultschitzky, also needs to be mentioned, although I am only familiar with his short paper published in the "Revue neurologique" [Neurological review], Vol. 1, 1893, p. 557. He considered neuroglia to exist as branched or unbranched bodies ("corpuscules ramifiés et non ramifiés"). The ramifications do not divide and do not anastomose, they correspond to ordinary protoplasmic cell divisions ("divisions protoplasmatiques ordinaires des cellules") (which deviated from Ranvier's view) and they are not hollow, as Lavdowsky believed. Popoff also observed free fibers.

[1]"Vom Aubau des Rückenmarks" [On the structure of the spinal cord], "Archiv für mikroskopische Anatomie" [Archives of Microscopic Anatomy], Vol. 38 (1891) p. 263 ff.

[2]"De la névrologie et de sa distribution dans les régions du bulbe et de la protubérance chez l'homme adulte" [Interstitial nerve tissue and its distribution in the bulb and protuberance regions of the adult male brain], "Arch. de psych., de neurologie et de médecine légales" [Arch. of psych., neurology and forensic medicine], 1893. vol. 11, p. 1.

In grey matter, the strands of the neuroglial network between the nerve tissue components are spaced further apart than in white matter, (which is not generally true), but the density of neuroglia varies. It is most dense in the olivary body (which confirms my own observation) of the "gelatinous" substance, in the hypoglossus, vagus and facialis nuclei, less so in the nucleus of the acusticus, abducens and the scattered grey masses in the pons, in the trigeminal nucleus, etc. Some further comments about this paper will be mentioned later.

Concluding remarks

This concludes our historical overview which does not claim to be a complete account by any means. Firstly, because I certainly missed a number of publications, secondly because I could not access some specific references, and thirdly because a number of publications had at the time already dated too much, the Jacubowitsch papers, for instance. We have also omitted everything related to chemistry and evolutionary concepts, since this work will be discussed in more appropriate places later.

Results obtained with the Golgi method are also best discussed in detail and in relation to our own results at a later stage. We will however reflect on the more general repercussions of the Golgi method on neuroglia research in our concluding remarks here.

The success of the Golgi method, and more particularly its application to neuroglia, has been grossly overestimated. The advances in our appreciation of neuroglia we owe to the Golgi method pale into insignificance compared to the immense progress the same method facilitated in terms of our understanding of nerve tissue components. Although the Golgi method truly opened up a new era in terms of our perception of nerve tissue components, a number of scholars consider that it has also opened up a new era in neuroglia research, making the times prior to Golgi impregnation seem prehistoric, — this latter comparison is immensely exaggerated.

The Golgi method was perhaps most significant for our understanding of development. However, it yielded extremely poor results and, in some cases, completely erroneous results in terms of the distribution of neuroglia in adults. The extensive overestimation of these results can only be explained by the fact that the limits of the Golgi method had yet to become apparent. It is only very recently that von Lenhossek, Greeff and Retzius began to acknowledge the method's shortcomings, but they still substantially overestimated the significance of silver-stained images.

The rationale for the Golgi method's poor results in terms of the most important question, namely the topography of neuroglia, are obvious. The method does not fulfill the primary requirement of any method investigating support components: because it was not able to visualize all integral associations of the scaffold. This shortcoming, which has fundamental deleterious repercussions for the investigation of support tissue components, is not only insignificant in case of nerve tissue components, where the relative distribution of the different individual components is of fundamental importance and where the technique could almost be considered advantageous because it simplifies certain aspects of nerve tissue that would be confusing if visualized all together. One of the fundamental prerequisites of any useful method for investigating support component structures is that it should at least in some areas preserve the overall integrity of all components. The Golgi method does not satisfy this criterion. Apart from the fact that it invariably impregnates neuroglia components only here and there, it also only impregnates the cells and the fibers radiating directly from them ("processes of the cells"). This means that any fibers that are not directly associated with cells can no longer be identified as neuroglia components at all.

Even a reasonably completely stained specimen may convince that the majority of neuroglial fibers elude detection, despite the great thicknesses of tissue that may be impregnated with the Golgi method allowing to follow many a fiber to a cell in sequential sections.

The Golgi method presented an additional disadvantage for the research of support tissue components. As previously mentioned, it only preserves cells and the fibers adjacent to them. In addition, quite apart from the fact that the resulting silhouettes no longer reflect chemical and physical differences between the fibers and the cells, thereby producing mirages of cells with "processes", which we will revisit in more detail later, the one-sidedness of the method totally diverted the attention from the fibers ("cell processes") and concentrated it on the "cells". Although it is certainly interesting to investigate the morphology of neuroglia (sham) cells produced with the Golgi method, any functional investigation of neuroglia, as is the case for bone, elastic and connective tissue masses, needs to place particular emphasis on the scaffold components of the neuroglial fibers (what most authors refer to as "cell processes"), their massive assemblies, their orientation and the forms of their interconnections.

These questions could hardly be addressed using the Golgi method, or if they were addressed the attention was quickly deviated to answer completely insignificant questions, and the important issues as regards topography neglected, if not completely ignored. —

Given these constraints, it was highly desirable to develop a method that would allow us to explore the topography of the neuroglia. This method had to fulfill many requirements if it were not to fail in its purpose. It had to stain the support scaffold clearly and selectively i.e., without staining nerve tissue components, and more specifically without staining the axis cylinders. It had to depict the scaffold in its entirety and reproducibly stain appropriately treated tissue samples.

This was a difficult task that required many, long years of steadfast work, and is perhaps yet to be completed. Whether this new method offers advantages over previous protocols that make the tedious work worthwhile is a matter for readers to decide after considering the following sections. At this point, it is important to mention the shortcomings of the method right from the start.

The method is not able to significantly retrace the embryonic development of neuroglia. Apart from neuroglial cell nuclei, the method only stains fibers that have differentiated in a particular manner, as we shall see later. It is therefore completely a priori feasible that interstitial components which exist in the central nervous system, but which lack such differentiated fibers may go entirely undetected with the new method.

However important these defects may be to the embryologist and the ordinary histologist, they are of little consequence to the pathological anatomist. Although the new method was specifically intended to address pathological anatomy questions, it first needed to be tested on the normal topography of neuroglia. The current account should therefore be viewed as preliminary results, although the author will gladly admit that the investigation of the normal topography of neuroglia was particularly important to him.

## Chapter 2:

## Relationship of neuroglial fibers to the cell

When specimens are stained according to the new method described at the end of this paper, a large number of blue-stained fibers are observed. In addition to these fibers, (and possibly red blood cells in the vessels) the nuclei of all other cells are also stained.

Of the cell bodies, those of the larger ganglion cells stain yellow and very neatly confirm the drawings of Nissl, because they stand out clearly from the darker, more brownish stained background and the remaining, lighter protoplasms (Panel II, Fig. 1 a). The coarser cell extensions and axis cylinders also stain yellowish, but the finer ones are not visualized. Likewise, the cell bodies of the small ganglion cells are only slightly yellowish or not stained at all; the bodies of those cells which are considered to be neuroglial cells are also unstained i.e., invisible.

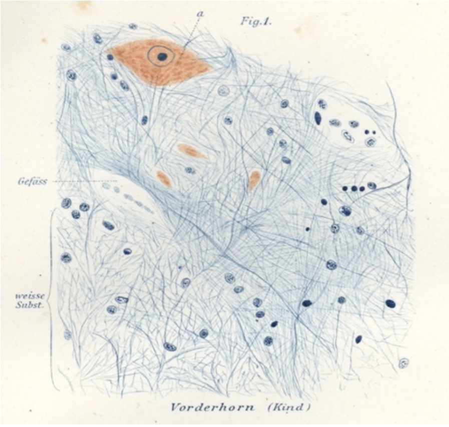


*Fig. 1a from Panel II: Peripheral region of the anterior horn in a newborn infant. Magnification C. (a) A ganglion cell with Nissl granulations. Bottom, white matter.*

For the time being, we are only interested in the relationship of those blue fibers to the nuclei, which are likewise blue in color; other subordinate histological peculiarities of the former will be discussed in a separate chapter later. Among the nuclei are those which, according to the current view, can only be regarded as nuclei of glial cells, because they are located in regions where, as far as we know, ganglion cells do not occur e.g., in the white matter of the spinal cord. There are two main types of these nuclei: larger vesicular nuclei with granular-looking chromatin and smaller ones in which the chromatin is a homogeneous dark mass. As distinct as these two types of nuclei may seem, there are cases where it is difficult to assign a particular nucleus to one or the other category, such that it is feasible to consider the existence of "transitional" forms in between these two nuclei subtypes. From the two nuclear forms there are numerous lighter, vesicular, punctate structures which are spatially associated with the fibers.

These structures are only exceptionally, if at all, associated with smaller darker nuclei.

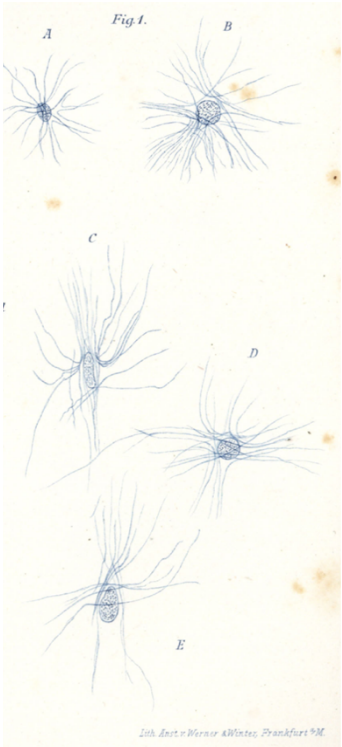


*Fig. 1 from Panel I: Astrocyte forms. Magnification A.*

In many cases the fibers are close to the (bright) nuclei or are separated from them by only a small space, which is thought to be filled by (unstained, therefore invisible[1]) protoplasm. They sometimes continue on past the nucleus to the other side in a fairly straight line, with some of fibers deflecting from nuclei with a more or less sharp bend to also extend beyond the nuclei (Panel I, Fig. 1 A-E). A part of the fibers, which in tissue sections, run above or below the nuclei (not like the ones mentioned so far laterally from the nuclei), (e.g., Panel I, Fig. 1 A, D, E), also behave the same way, except that the focus of the microscope must be changed to notice the sharp separation between individual fibers and nuclei. Other fibers still can only be traced up to the proximity of nuclei (see Panel I, Fig. 1 D), where they end sharply without continuing beyond the nucleus; but these are rarer than the fibers that can be traced further into the tissue beyond the nucleus. Whether these fibers, which only reach into the area of the nucleus, really end there, or whether we are only dealing with those whose (bent) continuation has been interrupted by sectioning, remains to be decided.

Very characteristic images are formed when, as is very often the case, the fibers are arranged in whole clusters around the nucleus, forming a spider-, brush- or star-shaped structure, in the center of which lies the nucleus with its invisible assumed protoplasm (cf. Panel I, Fig. 1[2]). Transitions of the fibers into this invisible protoplasm are not apparent. They would have to manifest in such a way that the fibers gradually became paler near the nucleus and disappeared in its vicinity. But this never occurs.

It needs very little imagination to identify these nuclear centers with the radiating fibers attached to them as Deiters cells, neuroglial cells, spider cells, brush cells, astrocytes, gliacytes etc.

[1] The protoplasm can be visualized using other methods e.g., with neutral carmine, in our preparations: it is therefore not missing.

[2] Similar images are often implied in our illustrations. However, since these latter images are drawn without adjusting the microscope focus as far as is possible, the spider shape, etc., is not as evident as in illustrations, which are drawn by intermittently adjusting the focus.

This perception is particularly pronounced where the space between the fiber clusters and the nucleus is very small, so that it is necessary to look more closely in order to perceive the sharp separation of the fibers. If the microscope lens is not in focus under such conditions, it is easy to believe that one is looking at an "astrocyte". Also, in photographs of such preparations that are not extremely sharp, the nuclei with their apposed fibers look exactly like Deiters cells. —

In many other places, however, the relationship of the fibers to the nuclei does not manifest with quite the same characteristic appearance. Some nuclei are located in such a tangle of fibers that it is difficult to assign them to a specific group; some nuclei do not display a clear relationship to the fibers even in regions where the tangle is not as pronounced. Less extensive tangles of fibers can easily persuade to include hidden "astrocyte images" via a change in the sectional direction, particularly if the fibers project out of the plane of the section. This had previously already been mentioned by Golgi[1], with a greater abundance of such "astrocyte images" occurring in vertical plane sections of the spinal cord compared to horizontal plane sections.

These "astrocyte images" may also be further revealed by counterstaining the neuroglial cell bodies with neutral carmine for instance — this double staining approach is, of course, not favorable for visualizing the finer fibers. Counterstaining is helpful when the space between the nucleus and the fibers is too large to clearly reveal their association i.e., when the cell body, which is not visualized without counterstaining, is too large.

But in spite of all this, many nuclei between the fibers (particularly all small, dark colored ones perhaps) cannot be considered to be in any way the center of the projection systems. Conversely, it is not surprising that not all fibers can be traced to the nuclei centers, given the extensive length of the fibers which means that they cannot be visualized in their entirety in the same plane of the section, since the point of contact with the nuclei always only represents a very small part of their path.

Notwithstanding the fact that so many nuclei have no characteristic associations with the fibers at all, and the fact that most fibers display no association with nuclei, one cannot but conclude that all fibers that stain with our method are identical to the structures that have, since Frommann, been considered to be the extensions of neuroglial cells[2].

[1] Collated papers, p. 168.

[1] Whether these cells and their extensions may really be classified as "lower" neuroglial cell fibers will be discussed in detail later. We will, in the meantime, refer to these cells as neuroglial cells, which reflects the current prevailing interpretation.

The resemblance of the fibers, which project around nuclei in ray-, spider- or brush-like clusters, to the overall aspect of typical astrocytes, as we previously mentioned, is so striking that it leaves no doubt as regards the identity of the extensions and fibers. Indeed, when the small space between the nucleus and the fibers is considered to be filled, the resulting image is very similar to a Deiters cell obtained by isolation or to a Golgi silhouette.

The remaining fibers, which do not appear to be radiating from a nucleus, may be assumed to be identical to the aforementioned fibers grouped around the nuclei in their characteristic configurations, based on the reflections formulated by Frommann more than thirty years ago (refer to the historical background chapter). The fibers that are not associated with nuclei are so similar to those radiating from specific nuclei in terms of their general appearance, uptake of stain, etc., that it would be difficult to consider them to be a distinct type of fiber. The similarity of the free fibers to the astrocyte fibers is also supported by another feature.

Indeed, the new method detects "fibers", albeit in greater abundance, but in identical arrangements, wherever "extensions of neuroglial cells" had been shown to exist in the given arrangement using the old methods. This applies primarily to the cortex layer, the white matter, and the vicinity of the central canal in the spinal cord. It is also worth recalling Bergmann's description of such fibers in the cerebellum, the superficial connective layer in the cerebrum, and the opticus. Also, the Golgi method, which admittedly only highlights fragments of the rich "cell extension networks", does not cast doubt on the uniformity of the arrangement, it does not provide a reliable assessment of the diversity of the network.

Accordingly, we can draw the following conclusion with some degree of certainty:

The fibers we have described are not a novelty, nor a hitherto unheard-of structural component, rather they are identical to what has previously been described as extensions of Deiters cells.

The initial fibers that stained with our method were identified to be identical to the structures that had previously been described using the older methods a long time ago. But of the nuclei, or the cells that those nuclei belong to, that stained with our method, only those with radiating fiber arrangements can be ascertained to correspond to the nuclei and cell bodies of "Deiters cells". We do however observe that (in addition to ganglion cell nuclei) a large number of nuclei are located between our fibers, with fiber strands running very irregularly in the vicinity of these nuclei: based on the current views, we must certainly consider these nuclei to be neuroglia nuclei in many regions, for they lie, as already mentioned, mostly in regions previously determined to be devoid of neurons.

How should such neuroglia nuclei or cells be interpreted?

There are two possible options. The first is that a large proportion of neuroglial cells in human adults have lost their typical astrocyte characteristics. A similar interpretation had already been suggested by Jastrowitz and Boll, and in more recent times, by Petrone and Popoff. Also, Kölliker explicitly states[1]: "Furthermore, I must say that such free cell bodies are too abundant and appear in the presence of very specific structures to be considered as accidentally detached components of Golgi cells" (i.e., of astrocytes).

Most other researchers that use the Golgi method do not believe that these cells without processes exist, — but it should be recalled that the Golgi method does not reveal these types of cells, which means that they are not observed.

If we were to entertain this possibility, we would have to assume that even very abundant neuroglial cells are not part of the astrocyte category, which had indeed already been previously suggested by Jastrowitz and Boll, in particular.

[1] "Handbuch der Gewebelehre des Menschen" [Handbook of human tissue science], 6th edition, Leipzig, 1833, Vol. 2. p. 150.

Alternatively, many of these cells may be astrocytes in the old sense of the word i.e., not associated with differentiated fibers and simply leaning against the fibers in the context of radiating centers, but these cells may in fact consist of non-fibrous i.e., protoplasmic extensions. Such protoplasmic i.e., true extensions, however, as we shall see later, cannot be visualized with our method; we cannot, therefore, comment on their presence or absence, and are compelled to leave the alternative option open to interpretation.

We have just contrasted "astrocytes in the old sense of the word" with our astrocyte-like fiber arrangements around the nuclei and specified protoplasmic extensions (the former case) to be true, relative to the differentiated, only leaned on fibers.

As we saw in the historical background, almost all authors denied the existence of such a distinction and thought that astrocytes only occurred with real extensions (in human adults which is what we are referring to all along here, with the exception of the discussion about the cells without processes). Only Ranvier had expressed the view (admittedly only in relation to the spinal cord) that such neuroglial cells with real processes occur in the embryo, but that "astrocytes" otherwise represent structures with only few differentiated fibers[1].

So, who is right? Ranvier (in terms of the spinal cord) and the author of the current account (in relation to the whole central nervous system) or all other authors since Frommann[2]?

A number of objections have been raised about Ranvier's theories, among them Golgi[3] even went as far as disputing the accuracy of Ranvier's evidence.

[1] Boll in some respects had similar views to Ranvier but was not as explicit as Ranvier about the fundamental differences between fibers and cell extensions. Boll still referred to the fibers as "cell processes".

[2] For Lloyd Andriezen refer to the footnote on p. 38. It must be emphasized that in the case of Ranvier, as well as in our own case, the nuclei of these cells leaning against the fibers radiating from the centers of these structures were clearly visible. This comment is necessary because Paladino recently inferred ("Bolletino della R. Academia medica di Roma", 1801, 2nd edition, p8, Sept issue) that Ranvier and I had looked at old cells which had lost their nuclei. Paladino's comment is all the more bizarre, because Ranvier's drawings clearly depict large and distinct nuclei, which proves that Paladino's statement is obviously incorrect.

[1] "Über die feinere Anatomie des Centralnervensystems" [On the more detailed anatomy of the central nervous system] (1885), in the collated papers, Jena, 1884, p. 157.

Golgi claimed that he had prepared samples according to Ranvier's specifications and never found anything different in terms of "astrocytes" than the same cell extensions he had seen in his own preparations when using other methods.

However, our method provides evidence that Ranvier was correct after all, which means that we can dismiss Golgi's negative results in this instance. But what led such an outstanding researcher to miss Ranvier's correct images? In any case, Golgi must have done something different to Ranvier when he attempted to confirm Ranvier's results. As there was no real room for deviation, we may assume that the difference between the Golgi and Ranvier methods is possibly due to the picrocarmine stain[5#]. "Picrocarmine" and "Picrocarmine" can be quite different. Apart from the nuclear staining, which can be achieved with some degree of confidence, picrocarmine stains vary greatly depending on the preparation, and this is why this particular stain has been phased out or has been superseded with other carmines that are better adapted for nuclear staining. Ranvier's carmine was probably optimized to stain the fibers but not, or hardly stain the cell bodies, while Golgi's picrocarmine stained both unselectively, making them impossible to distinguish.

Ranvier's method was nevertheless still inadequate. This can be inferred from the fact that he claimed that the Deiters "cells" in the cerebrum were different than in spinal cord. Ranvier believed that spinal cord Deiters cells were not associated with distinct fibers, but were cells with protoplasmic extensions, much like earlier authors had assumed to be the case for all Deiters cells, and as Ranvier himself had reported in embryonic structures. Kölliker was therefore absolutely right to turn the Ranvier spinal cord Deiters cell data against Ranvier himself, because there was no reason at all why Deiters cells in the brain should have remained in their "embryonic" state in the adult, while they acquired a different characteristic in the spinal cord. But Kölliker's argument simply drops to the wayside because exactly the same types of differentiated fibers, which only lean against the cell body, are also found in the cerebrum, as they are in the spinal cord and as they are in general in the whole adult central nervous system. —

But criticisms of Ranvier's work went even further than contesting his evidence by attempting to rationalize his apparent errors according to the prevailing views of the time. Thus, Golgi[1] and Kölliker[2] after him pointed out that what Ranvier had described as continuations of the fibers in the interior and at the edge of the protoplasmic body were simply folds, giving the impression of fibers. Our method immediately revealed the fallacy of this interpretation. It would just have been wonderful if these folds not only overwhelmingly followed the lines that connected them to the corresponding presumed cell extensions, but also followed the respective curvature of these extensions, thus yielding an overall picture of a uniform fiber formed from the two extensions and the "fold", and if the two extensions on the one hand and the fold on the other, did not seem separate. However, using our method, the darkly stained fibers cannot be mistaken for real folds that sometimes occur randomly or as artifacts. Moreover, if the fibers run perpendicularly to the sectioned surface of the cell, they appear as dots — and a dot cannot be a fold.

It could still be possible that Ranvier's method and our own method produce separate filaments instead of extensions, but this would amount to some kind of artificial product. Since Ranvier's method was still unreliable and in the case of the cerebrum left its inventor and other outstanding researchers, like Golgi, stranded, the assumption of folds was certainly an obvious one, and one cannot begrudge the researchers for holding on to their old established views despite Ranvier's publications. Although it is undeniable that, in terms of the question we are addressing here, our method may otherwise leave a lot to be desired, but in this case, it is reliable, and at this point we have to assert that the images obtained using the old methods (but not those from Ranvier) were in fact mirages.

[1] Collated papers, p. 158.

[2] "Handbuch der Gewebelehre des Menschen" [Handbook of human tissue science], 6th edition, Vol. 2, p. 149 f.

Images obtained with the old methods, as well as the Golgi method[1], simply cannot distinguish fibers from cell bodies because both structures refract light in the same way (Ranvier) or stain to the same extent, so that the two structures appear to share identical chemical (and morphological) properties. Our preparations however prove that this is in fact an illusion, because we are able to demonstrate that the chemical composition of fibers and cell bodies is quite distinct. This, of course, is the crux of the whole question, and perhaps we may be excused for going into a little more detail here in light of the importance of this particular issue.

The same facts apply here as they do in all other chemical and physical reactions. Two compounds e.g., one with potassium and the other with sodium bonds, may have as many reactions in common as they like, but it takes only one single reaction to be different to prove in the most definitive way that the two compounds are different from one another. These reactions do not need to be of a chemical nature in the true sense of the word. Organic chemistry, for example distinguishes two substances, which otherwise have who knows how many chemical properties in common, by differences in boiling point or by their different properties with regard to polarized light.

The microscopic staining of tissues is also a reaction, which we always call a chemical reaction, although it may at least in some cases also be a physical reaction. But this distinction is irrelevant for us histologists. In these marginal areas, the chemical and physical differences become blurred, and in addition, the physical reaction also always depends on the specific properties of the tissue to be stained, — and that is ultimately all that is involved. If staining and impregnation etc. are indeed reactions, then having two tissue samples that stain identically does not necessarily reflect that the stained materials are equivalent, as is the case for chemical and physical reactions in a strict sense.

[1] Only one researcher has obtained images equivalent to ours using the Golgi method, at least when used under particularly favorable conditions. His name is Lloyd Andriezen (The Neuroglia elements of the human Brain, British medical Journal, July 29, 1883). On p. 4 of the Sep issue, he states that: "With a wide angle of light perfectly focused and free from chromatid aberration and with equally good lenses the best preparations will show a very small quantity of protoplasm in the cell body, which, however, is mainly constituted of the meeting and intercrossing of fibers. Many of these neuroglial fibers pass right through the cell body." Those familiar with Golgi's images will have all due respect for a researcher who was able to make this subtle observation.

Two structural components, with one or more common staining reactions, may be considered to be chemically (or physically, see above) distinct, if they react differently to another stain or if each structural component stains differently with the same stain (provided that the staining method yields reliable and reproducible results). Our method stains both nuclei and neuroglial fibers, but a chromatin nucleus would be unmistakable in the neuroglial fibers, because counterstaining with any other nuclear stain would stain chromatin in the nucleus but leave neuroglial fibers unstained.

In contrast carmine, nigrosine and Golgi impregnation all stain neuroglial fibers as well as cell bodies. However, both neuroglial fibers and cell bodies may still be considered chemically distinct (see above), if just one other method stains them differently. In this particular case there are even two methods that stain these structures selectively: Ranvier's method and ours. In our scenario, not only is there a graduation of light to dark, but the cell body cannot be discerned at all and can only be assumed to be located in the vicinity of the nucleus, and this all the more readily because the nucleus can also be counterstained in the same preparations. If Lenhossék[1] criticizes my method for not making the cell body apparent, it is in fact a very fortuitous opportunity to point out that it is exactly the absolute absence of staining (in preparations from normal organs) that very distinctly highlights the chemical difference between the cell body and the fiber.

Let us further expand on this important issue by considering in more detail the methods which stain both the cell body and the fiber in the same way.

There is no need to add anything else about the unreliability of light refraction[2] to evaluate the more subtle structural relationships in a tissue section, but with regard to the particular stain considered here, it must be expressly pointed out that all stains which suggest a connection of the fibers to the cell body are extraordinarily non-selective, not only with regard to protoplasm and fibers, but in general.

[1]"Der feinere Bau des Nervensystems" [The finer structure of the nervous system], 2nd edition, Berlin, 1825, p. 186 f.

[2] It should however be recalled that Boll, despite the prevailing difficulty to make the distinction, had already noted a difference between the fibers and the protoplasm. (Refer to the historical background chapter)

Indeed, with the exception of the myelin sheaths, neutral carmine, nigrosine, etc. stain everything in the central nervous system. There are admittedly minor differences in stain intensity with coarser axis cylinders, and the like, appearing darker. But these can hardly be considered significant.

To date, Golgi impregnation is perhaps the least selective of these staining methods, even less selective than the now despised carmine stain. Golgi impregnation will not even reproducibly show up differences in stain intensity, with everything either staining uniformly dark or light. With the exception of myelin sheaths, the Golgi method stains all components of the central nervous system: neurons with their dendrites and axis cylinder processes, neuroglial cells and fibers, ependymal cells, even vessels, of course and depending on the whim of the stain each of these components sometimes stains on its own, or in the most diverse, quite unpredictable combinations with one or more of the other components. A method could hardly be more non-selective, if one may use that word. The Golgi method has another big disadvantage compared to carmine staining etc. The more generic carmine type stains still allow to visualize the actual structural image of the cell as far as it can be discerned by differences in light refraction and slight differences in staining intensity. This is in contrast to the Golgi method, which entirely eliminates or virtually eliminates this structural image of the cell, because of the opacity of the silver deposition, and the entire impregnation system makes cells simply appear as silhouettes. There are only hints of nuclei here and there as lighter spots, and even the vessels often do not appear as hollow tubes but as solid strand[1].

The question then arises, how would you counter someone arguing that a large number of the cells in the central nervous system do not have nuclei based on results from the Golgi method? Most researchers would vigorously reject this claim, because nuclei can simply be visualized with other methods. But what is true for nuclei should also apply to fibers. Even though the new methods considered in this current account are not as simple as nuclear staining, they are just as reliable, and allow to clearly visualize differences between fibers and protoplasm.

[1] This is similar for the biliary capillaries etc... While these appear as a hollow tube with an amazingly clear membrane using our method, the Golgi stain makes them appear as a solid vesicle. At the beginning of 1889, when the other staining methods on biliary capillaries had just been published, I was able to send specimens stained using my method to Councillor Heidenhain in Breslau.

In order to avoid committing the same type of error as claiming that most cells in the central nervous system are devoid of nuclei, we are compelled to admit that neuroglial fibers and cell bodies are distinct entities. It has therefore become untenable to substantiate that any results obtained with the Golgi method are contrary to the findings based on Ranvier's and our own methods. This has nevertheless not dissuaded a number of outstanding researchers to uphold that Golgi's images contradict our own clear-cut findings, even though the Golgi method itself when applied under particularly favorable conditions confirms our findings (Lloyd Andriezen, see above p. 38 note[1]).

That having been said, we can state the following with the utmost certainty:

1. Neuroglial fibers, previously interpreted as Deiters cell extensions, are chemically distinct from protoplasm.
2. The chemical difference of the "processes" does not appear gradually at a greater or lesser distance from the cell body, it is apparent right from the immediate vicinity of the cell nucleus.
3. Most of the so-called cell extensions are not extensions at all because two apparent extensions can form a common filament which runs past the cell.

[1] Golgi's comment that the "cell extensions" are intimately and intricately connected with the vessel walls (Figure on p. 168) to rationalize his disagreement with Ranvier's view remains completely incomprehensible to me. Why should "fibers" not themselves be connected to the vessels or gravitate towards them in an equally intimate and complicated manner? We do after all observe something very similar with elastic fibers, particularly with respect to vessels, but also with other parts of the tissue. Incidentally, the apposition of neuroglia to the vessels is somewhat elusive. It is only a part of a phenomenon of a more general topographic principle, which we will discuss in more details later.

> The filament is not in any way disrupted by the cell body, as would have to be the case for "extensions", which would each arise individually from the cell body. To summarize: **We are not dealing with processes or extensions of cells at all, but with fibers that are completely distinct from the protoplasm.**

If Frommann and later Golgi, followed by pretty much all of the newer authors have claimed that in humans, neuroglia only consisted of cells and their processes, this only holds true in embryonic tissue. In their fully differentiated state, neuroglia consist of cells and also of fibers, with the fibers so colossally predominant in their spatial distribution that they have to be considered as the more essential component of neuroglia.

## Chapter 3:

## On the nature of neuroglia using the new fiber staining method

In the previous chapter, we sought to demonstrate that the differentially stained fibers corresponded to what has been erroneously considered to be extensions of Deiters cells. In the interest of consistency with other authors, we provisionally designated the fibers as "neuroglia" fibers. But for this nomenclature to be adopted definitively, the proof that we are dealing with neuroglia here i.e., with a non-nerve tissue intermediate component, would either need to have been previously established, or will need to be provided herein.

We initially wish to demonstrate that there is so far no real evidence to support the neuroglial origins of the fibers nor the existence of neuroglial cells per se in normal histology.

The older arguments based on proving the existence of direct connections between specific fibers of the central nervous system and fibers in the pia mater i.e., with real connective tissue fibers, can be disregarded without further ado, because investigations from Frommann onwards have demonstrated that such connections simply do not exist. Deiters' evidence also cannot be considered stringent, however commendable his efforts, making him almost the only one to search for new criteria to characterize the nature of the connective tissue component of specific parts of the central nervous system.

We do not even need to mention his argument that the "spongy-porous" mass in grey matter is made up of neuroglia, based on his observation that the mass was independent of the cells, since we now know that this mass is neither independent from cells nor does it contain neuroglia. — Furthermore, he considered the structures that bear his name to be connective tissue cells because they were devoid of typical protoplasmic bodies and were therefore not actual cells but cell equivalents.

Apart from the fact that this view has not been widely accepted, as most authors do consider Deiters cells to represent true cells, the inadequacy of Deiters' proof to answer our question is already evident from the fact that Deiters, in the same vein, declared real neuron structures to be connective tissue components (the granules in the cerebellum and cerebrum).

If we disregard the pathologic-anatomic considerations, the only evidence which supports the existence of specific neuroglia characteristics in certain parts of the central nervous system is proof by elimination (an approach already employed by Virchow), which is applied arbitrarily i.e., one considered anything that cannot be classed as nerve tissue, for whatever reason, as neuroglia. This type of evidence may be sufficient under favorable circumstances. However, there are so many pitfalls, particularly in the case of the central nervous system, that one has to be extraordinarily careful about any conclusions drawn by way of eliminations.

Previously, some authors for instance considered the absence of nerve tissue components between the white matter myelinated fibers of the spinal cord as a given and argued that anything lying between these fibers must therefore be a connective tissue component. We now know that there are masses of axis cylinder collaterals in that region, which means that not all fibers observed here are necessarily neuroglia, as previously surmised. Likewise, the posterior commissure, near the central canal of the spinal cord, which was previously considered to be completely devoid of nerves, in fact contains an abundance of nerve fibers. Even the "molecular mass" in grey matter, which was also considered devoid of nerve tissue, and thus regarded as some form of "connective tissue", has been proven to be so rich in nerve tissue components that there is hardly any room left for a "molecular mass" in higher order creatures.

The same procedure applied to the evaluation of fibers and granular masses was also followed to evaluate Deiters cells, after disregarding the general points put forward by Deiters himself. None of the authors of that time even attempted to prove the non-nerve nature of Deiters cells, as it was considered self-evident that these cells were not nerve tissue given their overall appearance. But this conclusion based on elimination was purely supported by the fact that these cells did not look like the large neurons known at the time.

How unreasonable this conclusion was is already evident because Deiters and others, based on the same methodology, simply declared unmistakable nerve tissue components to be neuroglia. In 1871, for instance, Golgi still considered cerebellum granules to be non-neuronal structures.

Owing to the extraordinary success of Golgi's method, the true nature of these, previously completely unsuspected, ganglion cells has been readily acknowledged, and these ganglion cells were even represented with all their extensions. So now, after so many types of cells being formerly identified as neuroglia only to be subsequently acknowledged as nerve tissue, Deiters cells purely based on their shape after staining with silver chromate, were thought to surely not be ganglion cells i.e., by elimination they were therefore classed as neuroglia.

This conclusion would be valid if the criteria used to exclude the nerve tissue characteristics of Deiters cells simply because of the shape of the cell outline after staining with the Golgi method were really robust and there were no exceptions.

Since silver chromate-stained ganglion cells and astrocytes both appear to consist of a cell body as well as extensions, a fundamental difference between the two cell types could either be argued to apply to the extensions, to the cell body, or to both.

1. If we first compare the extensions of Deiters cells and of ganglion cells, there is an essential characteristic difference between the two or a simple difference in the shape of the processes.

a) As far as extensions are concerned, we know that neurons should possess two well-characterized types of extensions: dendrites and axis cylinder processes. Deiters cells do not appear to have such distinct processes, and if each individual ganglion cell were to invariably possess these two very different types of extensions, this would in itself already constitute a fundamental difference between these two cell types which could be inferred from the shape of the cell outline alone. But although this marked distinction between dendrites and axis cylinder processes is probably found in the most common type of ganglion cells, it is not a common feature of all ganglion cells.

α) Some neurons, such as spinal ganglia cells[1] and cells in the peripheral plexuses of the sympathetic nervous system, have no dendrites at all. These only have axis cylinder processes.

β) Conversely, some neurons without axis cylinder processes may only have dendrites e.g., in the granule layer of the olfactory bulb and in the peripheral sensory apparatus[2].

γ) Then there are neuron processes that even the most astute experts cannot agree on how to interpret. These types of neurons may include the cells described in Paragraph β), as well as the Ramón y Cajal cells of the cerebral cortex, whose processes are characterized so ambiguously that Retzius did not want to classify these cells as neurons at all initially[3]. Even once he acknowledged their true nature, he still stated that[4]: "Among the processes of the cellular component" concerned, it is difficult to find characteristic differences; thicker and thinner processes are found, but they do not exhibit any characteristics that distinguish axis cylinders from protoplasmic processes. The precariousness of the evaluation of these processes is also evident from the fact that, of the two other authorities on the matter, Ramón y Cajal and van Gebuchten respectively ascribe numerous axis cylinder processes or just one single process to the same cell type.

To sum up, even though the vast majority of ganglion cells possess two types of well-characterized projections, there are exceptions which prove that true neurons need not all possess these typical forms of projections.

[1] Ramón y Cajal considered one axis cylinder process, the cellulipetal one, as a dendrite, but this can only refer to its functional nature, because in the histological sense, which is the only perspective we are concerned with here, a process which turns into an axis cylinder of myelinated neurons remains an axis cylinder process irrespective of its context.

[2] cf. Kölliker, "Gewebelehre" [Histology], 6th edition, Vol. 2, p. 43. It is of course not yet clear how these cells should be interpreted. They can therefore in the interim be classified in the γ) category. Compare Golgi's, "Gesammelte Abhandlungen" [Collated papers], p. 51, and Ramón y Cajal, "Notas preventivas sobre la retlna y gran simpatico", Barcelona, 1891, p. 4 f.

[3] "Über den Bau der Oberflächenschicht der Großhirnrinde beim Menschen und bei den Säugetieren" [On the structure of the superficial layer of the cerebral cortex in man and mammals], "Verhandlung des Biologischen Vereins in Stockholm" [Proceedings of the Biological Society in Stockholm], Vol. 1. , March 15, 1891.

[4] "Biologische Untersuchungen" [Biological investigations], New series, Vol. V, No. 1 and 2, Stockholm, 1893, p. 7.

But once this has been established, there is a priori not the slightest reason why Deiters cells should then not represent a further exception among neurons. There is no need to get upset about the fact that Deiters cell processes are so diverse and may either be interpreted as (very numerous) axis cylinder processes, as dendrites, or even as both, because such indeterminate extensions also occur in real ganglion cells.

b) But even if there are no fundamental differences in terms of the extension's characteristics (axis cylinder processes and dendrites) between the two types of cells, the mere shape of the (indeterminate) extensions of Deiters cells may nevertheless still be sufficient grounds to separate them from neurons in general. Since the extensions of neurons branch, an obvious distinction would be to observe that anything that appears as processes on astrocytes stained with the Golgi method and on all so-called neuroglial cells is absolutely unbranched.

Conversely if one assumes that cells attributed to neuroglia have extensions that also branch, this would no longer suffice to confirm an ineluctable difference between neurons and astrocytes. However, all authors[1] agree that "neuroglial cells" stained with the Golgi method show sparser or more abundant branching.

[1] Lenhossék seems to think that Golgi still assumes that extensions almost absolutely do not branch. But this is not so. He had earlier (1871) assumed that such branching only occurred (and then only rarely) at the beginning of the processes. But he changed his mind somewhat later. From the isolation preparations he used to check Ranvier's work, he found (Collated papers p. 157 f.) that they branch more frequently near their origins, "but that they also often branch at a great distance from their origins". Colella, from Golgi's laboratory, recently published work which describes very abundant branching occurring in embryonic cells ("de nombreux ramuscules secondaires longs et courts naissent sur le trajet... ils se tenninent librement par une riche arborisatiom en patte d'oie" . . [numerous long and short secondary branches arise along the way... they terminate in freely branching goose feet patterns.]). "Archives ital. de Biologie" [Italian Biology Archives], Vol. 20, p. 214.

Profusely branched "neuroglia" are described as "short branched“ and are also found to associate with long branched "neuroglia” through a multitude of different types of "connections"[1], — and this is sufficient to state that the presence or absence of branches in itself does not constitute a principle difference between neurons and neuroglial cells.

Even the type of branching cannot be used to define "neuroglia” given the great variety of neuron branching: because one may always be able to find analogies between "neuroglial cells" branches and neuron branches.

2. The bodies of both cell types also exhibit no major differences. Neuroglial cell bodies are generally quite small. Although some of the neuroglial cells in the double pyramid-shaped lobus piriformis[2] also exhibit such small bodies, one also finds, as van Gehuchten depicts, neuroglial cells with such powerful protoplasmic abdomens that every ganglion cell would be envious of[3].

3. Secondary tools used to establish fundamental differences between neuroglial cells and neurons yield even more ambiguous results. When stained with the Golgi method, some authors report that neuroglial cells turn more rusty brown instead of the black appearance of neurons, that they impregnate faster than neurons, and the like. It is quite obvious that these differences are very variable. Neuroglial cells also often stain black, and ganglion cells rust-brown, and alongside astrocytes, which predominantly stain earlier, there are always some neurons which stain as well. This approach is therefore unhelpful.

4. The pivotal argument which most clearly proves that specific differences attributed to these two types of cells do not exist, is that the foremost authorities in the field may in some instances be ambivalent whether to class a specific cell as a neuroglial cell or as a neuron, and this may lead to errors in their diagnosis.

[1] Kölliker, "Gewebelehre" [Histology]. 6th edition Vol. 2, p. 144 ff.

[2] Kölliker, "Über den Fornix longus von Forel und die Riecht[?unclear]strahlungen im Gehirn des Kaninchens" [On Forel's fornix longus and the olfactory tract in the rabbit brain], "Anatomische Gesellschaft" [Anatomical Society], 1894, Fig. 1.

[3] "La moelle épinière et le cervelet" [The spinal cord and the cerebellum], "La Cellule" [The cell], Vol. 7, 1891, Figs. 38 and 42.

We have previously mentioned this in relation to Ramón y Cajal cells of the cerebral cortex; it equally applies to the basket cells of the Purkinje cell layer. Even Kölliker,[1] when discussing these basket cells, resigned himself to the following conclusion: "Because both neuroglia components as well as neurons take up the silver staining using Golgí's method, it is not easy to differentiate between these two cell types and the decision is primarily dependent on the individual's knowhow and experience."

5. Histogenesis also fails to find crisp differences, because both types of cells have exactly the same origin.

Altogether, we can surmise that: The criteria used to exclude the neuronal origin of Deiters cells are not reliable i.e., they do not allow for exceptions. Although the process of elimination may have correctly determined the characteristics of neuroglia, it did so only "as a result of a lucky divination, and not because the assertion was backed by stringent evidence", to paraphrase Deiters.

We have so far only discussed the possibility of attributing neuronal characteristics to Deiters cells as an intellectual "problema". It is therefore very timely that the recently published work of Colella tries to tackle this problem in the real world, and no less significant that this work comes out of the Golgi laboratory. Since this work is from that laboratory, it deserves our attention. It should be specifically highlighted that the following quotes from Colella[2] relate to the embryonic origins of neuroglia and not whether they have neuronal origins. This is clearly demonstrated in his opening words. Colella says and I quote:

"Leur" (meaning Deiters cells) "mode d'origine n'est pas un argument decisif pour juger de leur nature et le champ reste ouvert à de nouvelles recherches pour savoir, si les éléments de la névroglie de la moelle Epinière sont de nature nerveuse, épitheliale ou connective.

[1] Das Kleinhirn [The cerebellum], Zeitschrift für wissenschaftliche Zoologie [Journal of scientific zoology], Vol. 49, 1890, p. 676.

[2] Sur l'histogenèse de la névrologie dans la moelle épinière [On the histogenesis of neural systems in the spinal cord], Archives ital. de Biologie [Italian Biology Archives], Vol. 20, p. 212 ff.

Pourtant les recherches de Magini sur les systèmes de filaments Epithéliaux se colorant comme les fibres nerveuses à myéline et celles de Caporaso et Sgotto sur la propriété de l'épithélium du canal central chez les tritons et les larves des grenouilles tendrent à assigner à la névroglie embryonnaire une nature nerveuse."[The developmental origin of Deiters cells is not a decisive argument about their neuronal or non-neuronal nature and future research will need to determine whether spinal cord neuroglia have neuronal, epithelial and connective tissue components which arise from neuronal or non-neuronal precursors. However, Magini's research on epithelial filament systems which stain similarly to myelinated nerve fibers, and the work of Caporaso and Sgotto about the characteristics of the epithelium of the central canal in newts and frog larvae tend to support the neuronal origin of embryonic neuroglia.]
The function attributed to neuroglia in the nervous system is however not expanded on in the very short essay; one would possibly have to ascribe neuroglia a very specific, perhaps sympathetic function. But there is no need to worry about that. We performed the future research that Colella so rightly called for, which demonstrates that there can no longer be the slightest doubt that at least the neuroglia, meaning the Deiters cell processes, including the versions with long branched extensions that occur in vivo, are in fact true intercellular tissue components i.e., can be attributed "connective characteristics" in the morphological sense.

If we consider the results provided by Ranvier and ourselves, then the ground which we have under our feet is no longer shaky as it has been up to now.

1. Our investigations also allow us to draw a number of conclusions by the process of elimination: If we disregard the cell nuclei which are not considered here, our dye does not stain anything at all that can be considered a nerve tissue component based on our current understanding i.e., neither ganglion cells, nor their protoplasmic processes, nor axis cylinders. The evidence supporting our conclusion is much more reliable than prior evidence because it is not based on the ever-changing shapes of cells, but on a chemical reaction. A chemical reaction which results in differential staining as opposed to the earlier attempts to identify differences in a background of sameness. As previously mentioned on p. 38 (and here too the investigations of Magini[1] mentioned by Colella should serve as a warning), an identical histochemical reaction can only be used as a criterion to a very limited extent.

[1]I am not aware of Magini's work. The above statement was prompted by the comment in Colella's book.

However, a conclusion based on a difference produced by a chemical reaction, which after all corresponds to a material difference, is all the more reliable. —

2. But we need no longer contend with drawing conclusions by elimination, we can provide unambiguous supportive evidence that we are dealing with a true intercellular tissue component.

What is a true intercellular tissue component?

As previously mentioned in the historical background, Deiters also asked this question. He answered the question with a definition derived from Max Schultze. Accordingly, intercellular substances are "modified cellular tissue components that are distinct from cellular bodies and can therefore no longer be considered as directly related to them."

It should be recalled that, based on this definition, Deiters declared that the molecular tissue component of grey matter, which he called spongy-porous, was an intercellular tissue component. As this assumption has since been found to be erroneous, there must be an error in Schultze's definition or in Deiters' use of it, which we must of course avoid.

Deiters had omitted to prove that the spongy-porous mass was in fact a modified cell component. He was content assuming that these masses no longer appeared to be directly associated with the cell bodies, — a perception which did not correspond to reality and was influenced by the methodology used at that time. Although this association cannot be demonstrated even using our current methods, it did not follow by any means that the spongy-porous mass was in fact an intercellular tissue component because this would have also required proof of a modification of the "independent" cell component.

Furthermore, Schultze's requirements to define a truly intercellular tissue component can be formulated even more strictly. We need not contend with the nondescript "modification" word either, because truly modified components are no longer considered as genuine protoplasm.

Are the two requirements of modification to a non-protoplasmic component and separation from the cell body fulfilled in our fibers? This question can be answered in the affirmative with the greatest certainty.

a) The modification of the cell component from which these fibers originate based on the embryological investigations[1] is quite striking. The fibers respond to the new stain, the protoplasm does not respond at all. Indeed, not only do the Deiters cell protoplasms not stain with our tinction, but all normal protoplasms do not stain either, or if they do they stain they take up the counterstain. A fundamental contrast is apparent here between the cell protoplasms and typical filaments which certainly do not include any protoplasm.

b) However, this feature on its own does not suffice. This is evident from the fact that the axis cylinder is also a modified cell protoplasm, which can be distinguished at least gradually from the protoplasm of the body of the cell via the stain. But nowhere is this axis cylinder "separate" from the cell protoplasm, it certainly enters the protoplasm and loses its peculiarities quite gradually.

To prove the existence of an intercellular tissue component, separation from the cell body needs to be evident. This is indeed quite perfectly the case for the fibers in question, in contrast e.g. to the axis cylinders. The fibers are only contiguous to the cell body, they are not connected with it, like the axis cylinders, as extensions, but much like the leading and trailing parts of a thread, if one may use this expression, are so intimately united with each other that they represent something coherent, a common fibril, which runs smoothly over the adjacent cell.

In this regard, neuroglia finally rejoins the series of connective tissue components, but only in terms of their morphology.

Just as in typical mesodermal (mesenchymal, parablastic) connective tissue types, this peculiar connective tissue of the central nervous system is composed of cells and of independent fibrous interstitial tissue components, much like the collagenous connective tissue.

[1]If one would surmise that the fibers did not originate from a cell at all, but were of intercellular origin right from the start, we would of course not need any evidence supporting a modification of the cell component because the fibers would then be simply classed as "intercellular tissue components". But as far as I can gather this does not apply to either neuroglia or connective tissue.

3. Furthermore, not only does it behave morphologically absolutely like real, albeit a specialized type of connective tissue component, it also reacts pathologically it in exactly the same way as typical connective tissue. It is well established that typical connective tissue always proliferates where the specific parenchyma perishes, the same is true in the case of neuroglia. Our method furthermore allows us to prove that neuroglia always proliferate when the specific tissue of its organ i.e., the nerve tissue, has perished.

To summarize:

The fibers we have stained are non-nerve intercellular tissue components,

1. because our staining turns the fibers dark blue whilst everything of nerve tissue origin does not take up the stain (conclusion by elimination),
2. because the fibers are modified, they are no longer protoplasmic, and are separate from the cell body,
3. because the fibers (and their corresponding cells) behave like a connective tissue component in the pathological sense i.e., the fibers proliferate when the specific, nervous tissue perishes.

**Chapter 4:**

## Relationship of neuroglial fibers to other neuroglial components and to connective tissue. Chemistry

The evidence we have provided to prove that neuroglial fibers are true fibrous intercellular components, at the same time serves to at least prove that the typical Deiters cells are not neurons but glial cells. Deiters cells are nothing else than real cells with closely lying neuroglial fibers radiating from them as from a center. They are fragments of neuroglial scaffold in which the fibers contiguously meet the cells.

This evidence is also valid for the embryonic precursor cells which give rise to the filamentous intercellular component of the typical long branched variants, as far as can be reliably determined. It also applies in specific contexts to the long, branched variants which have retained their embryonic characteristics in their later life, a possibility which we raised on p. 34 f. One could of course assume that these embryonic variants in adult tissue sometimes retain the potential to yield filamentous intercellular components.

Our evidence thereby captures the entire nature of neuroglia. The short, branched variants and all other similar forms do not belong to the tissue components which one would assume with any degree of certainty to produce an interstitial component that is separate from the cell body. The same could be said of the structures which Ranvier and Lloyd Andriezen described as protoplasmic glial cells of the "cerebral cortex". All of these cells defy not only detection with our method, but they also do not satisfy all of the criteria we identified as necessary to stringently prove that a given component exhibits neuroglia characteristics.

The short, branched variants and the protoplasmic cells may be neuroglial components, but they equally may not be. There is a lack of supportive evidence for either possibility, and we must therefore absolutely forgo to evaluate them.

We must moreover absolutely refrain from any judgment about all other kinds of interstitial components along the same line of thinking. After all, we do not observe any molecular, reticular or vitreous "general component" in our preparations, nor a spongioblastic neuroglia as His described and no horny spongiosa. It cannot be emphasized enough that the latter has nothing to do with our "neuroglial fibers". Apart from the fact that the outer appearance of the scaffold of this horn spongiosa is completely different from our images, it has nothing in common with our neuroglial fibers already for the reason that it is also found within the myelin sheaths (even of the peripheral nerves), which appear completely empty with our method. Our neuroglial fiber structures have nothing in common with the "nevroglio mielinico"[1] described by Paladino, about which we likewise decline to pass any judgment, for the same reason.

Following on from the question whether neuroglial fibers are horn components, the question about other potential chemical relationships of these fibers should also be settled here at the same time.

First of all, it must be stated that our fibers are not at all chemically similar to the composition of the glue-producing connective tissue.

Henle and Merkel already previously established the chemical differences between true connective tissue and what they referred to as molecular masses[2]. But the molecular masses as defined by Henle and Merkel do pretty much correspond to what we now consider as fibrous neuroglia.

Boiling water dissolves glue-producing connective tissue but does not dissolve the "molecular mass"; the latter is however destroyed by successive incubations in a potassium hydroxide solution followed by water, which is not the case for true connective tissue. Boll also observed that acetic acid makes the fibers somewhat pale but does not cause them to swell into invisible masses, as is the case with connective tissue fibers[3].

[1] Dei limiti precisi tra il nevroglio e gli elementi nervrosi del midollo spinale, R. acad. di Roma, XIX. Fasc. 2, 1893.

[1] "Über die sogenannte Bindesubstanz der Zentralorgane des Nervensystems" [About the so-called connective tissue components of the central organs of the nervous system], "Zeitschrift für rationelle Medizin" [Journal of Rational Medicine], 3rd edition, Vol 34 (1863), p. 59.

[1] "Archiv für Psychiatrie" [Archives of Psychiatry], Vol. 4, p. 20.

Connective tissue and neuroglia also exhibit different properties with our staining method.

We can further say that the widely accepted assumption of Gerlach that neuroglial fibers are elastic is quite erroneous, however enticing this view may be given the rigid curved appearance of neuroglial fibers. Our staining method does not stain elastic fibers at all. Our fibrils conversely do not stain with the methods used to stain elastic fibers. Finally, the low resistance of neuroglial fibers to postmortem influences and to a solution of potassium hydroxide speaks without further ado against their identification as elastic fibers. —

Consideration should also be given to the fibrils' correlation with filamentous fibrin. Arndt[1] said this about it: "......On its own, all connective tissue is what presents itself in this way" (as described by Jastrowitz) "and the nuclei in particular, which lie in the myelinated canal between the nerve fibers, I dare even today dispute...... The columns and fibers in and on which those nuclei are found, among which there is certainly also many a white blood cell, I therefore consider to be lymph clots, of which after death and during the preparation a not inconsiderable amount of myelin, which detached itself from the sheaths, has mixed with."

Our method also stains the fibrin if it is present in the vessels. If, however, one draws a conclusion from this same reaction to a chemical equality, one would again commit the error we have so often criticized. Then one would have to surmise that the membranes of bile capillaries, the dual light refracting muscle component and the cell nuclei etc. are also fibrin. Neuroglial fibers and fibrin stain differently.

If the correct fibrin staining is performed on an ordinary alcohol preparation, the fibrin is stained, but not the neuroglia.

[1] "Zur Histologie des Gehirns" [On the histology of the brain], "Archiv für Psychiatrie" [Archives of Psychiatry], Volume III, p. 470f.

But one does not even need this staining reaction to dismiss that our fibers or what are known as Deiters cells could simply be coagulation products, because of the great regularity of the network, which is reproducible for every specific region of the central nervous system, this fact is not consistent right from the start with a coagulation event in the sense of Arndt and also, as we wish to add in a moment, in the sense of Schwalbe. Coagulation of previously liquid masses always have something variable, random about them, which is quite incompatible with the regularity of our fibers.

Schwalbe had arrived at his conclusion through injection results. According to Schwalbe, injected masses penetrate between the nerve fibers without any problems, so in his opinion no solid cement can unite the fibers. This is certainly true, but neuroglial fibers are not a solid glue at all, but insulated fibers, between which there is still enough space for the injected mass, and between which it can therefore easily penetrate. —

Neuroglial fibers also cannot correspond to unchanged protoplasm, as we have pointed out several times previously — but we cannot yet convincingly say anything about the nature of protoplasm as yet. The most important conclusion from all these negative results is that the neuroglial fibers are quite different from all the fibers in ordinary connective tissue.

## Chapter 5:

## A discussion about the histogenetic status of neuroglia.

We have observed that neuroglia, as far as they appear in a reliably detectable form, are similar to the ordinary type of connective tissue i.e., neuroglia consists of fibers and of cells, which are only contiguous in the mature state. Despite this organizational similarity, neuroglia deviates morphologically and chemically so significantly from ordinary connective tissue that they hold a uniquely specialized status. It maintains this specialized status and displays it even more markedly in pathological conditions: neuroglia never turn into "connective tissue" or vice versa.

Neuroglial fibers may grow into connective tissue (e.g., the pia mater), but the connective tissue does not change to become neuroglia, although neuroglial fibers may directly contact connective tissue fibers from adjacent nerve tissue, in this instance the neuroglia have only grown beyond their natural limit, and the connective tissue behaves quite passively towards them.

In addition to their chemical, morphological, and general biological differences, neuroglia and connective tissue are also different in terms of their histogenesis. Vignal[1] was the first to state that neuroglia is derived from ectodermal tissue. This notion gained increasing traction among his peers, particularly since the Golgi method sparked an interest in embryonic development. Almost all authors have come to accept Vignal's views.

The Golgi method has been used to investigate the development of neuroglia both in terms of phylogeny and ontogeny and has generally concluded that Deiters cells are ectodermal cells that have migrated to the medullary plate and adopted epithelial characteristics.

[1] "Archives de Physiologie" [Physiology Archives]. 1884.

The most primitive stage in this developmental series, which extends to the higher mammals, and which is in some respects maintained in the fully developed adult state, consists of epithelial cells of the central canal or of the ventricular walls sending out long processes towards the periphery, which penetrate the entire area of the central nervous organ concerned and reach as far as the pia mater. The central nervous system has been known to include shorter epithelial cell processes for a long time now. They were observed as far back as Hannover, but the interpretation that these short epithelial cell processes function as support components, which is based on the evidence of the processes extending to the pia mater, is a more recent concept. This discovery is usually attributed to Golgi, but Lenhossék[1] pointed out that Hensen had already traced epithelial cell processes to the pia in 1876. But Hensen was also not the first to make the observation and is also not the first who concluded that these epithelial cell processes were support components. Both achievements are credited to Mauthner alone and were described briefly and succinctly in 1861 (Vienna academy conference proceedings). In order to give due respect to the deceased scholar's observations, the relevant passage is reproduced below verbatim:

"The epithelial cells lining the central canal and the processes emanating from them, which individual researchers, such as Stilling, are inclined to believe are nerve tissue structures, must definitely be classified, along with the processes of the pia mater, as spinal cord support tissue. I was particularly fortunate to observe, colossal processes emerging from the posterior epithelial cells of the central canal in the uppermost part of the pike spinal cord, which were not associated with any other cellular components, but reached the periphery of the spinal cord and became submerged in the fibers of the pia mater."

The Golgi method was instrumental in identifying these epithelial processes which extended to the pia and easily substantiated that they were an entirely normal feature during earlier stages of ontogeny and phylogeny. The method also detected remnants of epithelial cells with such extensions in mature mammals.

[1]"Der feinere Bau des Nervensystems" [The finer structure of the nervous system], 2nd edition, 1895, p. 210.

The fact that cell structures could be interpreted as transitional forms of actual Deiters cells opened the way to consider that ontogenetically and phylogenetically these cells with extensions may have developed from epithelial cells of the medullary plate i.e., from epithelial cells of the ventricles and the central canal.

Most readers of the current work will be familiar with the facts of the matter. For those readers not familiar with the issue, however, we will briefly summarize the current state of understanding. The work of Sala y Pons: La Neuroglia de los Vertebrados (Madrid 1894) is a good starting point. We choose to present this particular work because it is a very clear account, and also because some readers may not be familiar with Sala y Pons' work as it was only published in Spanish and might therefore not be accessible to everyone. The Sala y Pons report is printed in a reduced font so that readers familiar with his work can skip this next part.

Sala y Pons explains that neurons originating from the ectoderm actually follow the old family traditions and like their brothers, the epithelial cells, they have direct relationships with each other or are at most separated from each other by a sparse glue-like component. But neurons had not able to fulfill their purpose under these conditions, because the conditions do not permit the insulated transmission of nerve currents. Enlisting the help of mesodermal components was excluded: on account that these could not provide the necessary insulative properties, and so right from the very beginning, while a portion of the precursor central nervous system cells differentiated into higher order neurons, other precursors differentiated into neuroglial cells. Although neuroglial cells sacrificed their ambitions, they nevertheless became a modest but very useful tissue component, without which the nerve machine would be unable to function properly. (p. 6.)

Sala y Pons then goes on to summarize the insights obtained by him, Lenshossék, Ramón y Cayal etc. He initially (p. 38) states that, in terms of ontogenesis, the primitive cells, which insert themselves as support components between the nerve tissue elements, are in fact epithelial cells. Their bodies form a wall that delimits the inner cavities of the nervous central organs (ependyma). They are ciliated and send out a thin process that penetrates the whole organ and attaches "with the characteristic conus" under the pia mater (p. 37). After a while, these bodies undergo a transition, as the radial, peripherally running process divides and acquires thorny appendages in specific areas.

The branching in the peripheral part of the process results in a tighter attachment to the pia mater and allows the processes to pull the cell body more towards the outer periphery, using amoeboid movements. This means that fewer and fewer elements delimit the inner surface, and the inner surface therefore becomes smaller as development progresses, so that in the adult, the cavities are very much reduced in size.[1]

As development progresses, the cell body approaches the outer surface and becomes more irregular and shaggier in appearance. Only the attachment to the pia mater and the radial orientation remains indicative of the epithelial origin of the cell. The connection with the pia finally disappears, the cell body now rests freely in the midst of the nerve tissue component, sending out its delicate and curved (flexuous) processes in all directions and thereby displaying its true spider cell characteristics. Spider cells therefore do not arise from mesodermal cell migration, nor develop from undifferentiated ectoderm, but are the product of the epithelial cell differentiation.

(p. 38) This succession of ontogenetic stages is also reflected in the phylogenetic analysis; even within the same central nervous system compartment of one and the same species, higher-order species exhibit more differentiated types of epithelial cells compared to lower-order species. Birds, for example, have true spider cells in their spinal cord and cerebellum, but only transitional forms in the cerebrum. Amphibians and reptiles have transitional forms in their spinal cord; but only epithelial cells as support components in the cerebral cortex and in the Lobus opticus. There are even differences within the same organ. Neuroglial cells in fish are for example found in the actual cerebellum, they are similar to those in mammals, but in their valvula cerebelli are primitive forms, which is also consistent with the fact that the valvula cerebelli of fish generally exhibits more embryonic characteristics.

Sala y Pons also showed that both cell types (epithelial cells and Deiters cells), can perform the same functions and can be substituted for each other. The support component in less ontogenetically or phylogenetically developed species is exclusively made up of epithelial cells (e.g., in the spinal cord of fish, the cerebral cortex of amphibians and reptiles, the central nerve organs of mammals at the beginning of development), conversely, spider cells are predominantly found in the more highly developed species (e.g., in the spinal cord of birds and mammals, and the mammalian cerebral cortex and cerebellum), in intermediate regions (such as the cerebral cortex and lobus opticus of birds) epithelial cells coexist with components that are similar to spider-type cells. This is also a reflection of fact that as the organs increase in thickness there is a decrease in the number of epithelial support cells and their processes which reach the periphery. —

[1] Sala missed that the narrowing is only relative. In absolute terms, the surface of the ventricular cavities of an adult human is significantly larger than that of a human embryo. Therefore, there is no reduction but a considerable *increase* in ependymal cells.

This concludes the Sala section. A number of authors do not agree that the issue is that straightforward, of course. Even those who agree that neuroglia is of ectodermal origin deviate in some aspects from the views of Sala. Lenhossék,[1] for instance, who contributed meticulous work on neuroglia during embryologic development, does not entirely agree with Sala y Pons' position. Lenhossék believed that astrocytes originated from ependymal cells with long processes that allow them to move outward, interspersed with ependymal cells that are identical to the ones found as the only support components in very early embryos i.e., with cilia ("little hairs") and peripheral radial processes that extend right to the pia. Herein lies an essential difference with the Sala y Pons view, only some Deiters cells, at least in higher mammals, arise in this way, the origins of the other part of the Deiters cells cannot be ascertained, but these other Deiters cells do arise in a less direct ependymal cell-dependent manner. "Spider cells already emerge in their characteristic form quite abruptly in 20 cm long human embryos. Many of the researchers completely miss that spider cells evolve from the radial [glial] cells. The sheer number of spider cell precursors in human spinal cord is also far too large for them to be all traced back to former radial [glial] cells, of which there are much fewer." (p. 234.) Lenhossék therefore believed that these spider cells arise by a truncated form of caenogenesis, which does not involve a radial-fibrous stage, but that these types of spider cells instead differentiate from germ cells which are initially without processes, but soon acquire processes on all sides.

Vignal and Kölliker go even further. They let all neuroglial cells arise from undifferentiated cells, some of which go on to generate neuroblasts, some ependymal fiber cells, and others still spider cells.

According to the opinion of the authors mentioned so far, which is currently shared by most, including Retzius, (and including those researchers who consider spider cells to arise from undifferentiated cells) the spider cell precursors originate from the ectoderm which forms the medullary plate, and not from the migration of mesoblasts.

[1]"Der feinere Bau des Nervensystems" [The finer structure of the nervous system], 2nd edition, 1895.

Schrader, whose views will be particularly relevant to us below, also shared this position.

But as widespread as this notion was at the time, it was not entirely without its opponents. Lacchi and Valenti for instance assumed a mixed origin of Deiters cells i.e., partially arising from ectoderm and partially from mesodermal migration. The position of His deviated even further. Although he believed like Vignal, Kölliker, Ramón y Cajal, Lenhossék, Retzius etc. that some of the cells which originated from the ectoderm which forms the medullary plate do not go on to form neurons (neuroblasts), but instead form a scaffold component, His did not think that this type of cell had anything to do with Deiters cells but considered them to be "spongioblasts". According to His, Deiters cells originated from the migration of mesoblastic structures, which therefore have no relation at all to the ectoderm giving rise to the central nervous system i.e., that neuroglia, which we are alone to prove the existence of in our preparations, are also real connective tissue components in the histogenetic sense, if we follow His' argument.

Having briefly discussed the current histogenetic understanding of neuroglia, we must now examine how to reconcile our own views with the embryo development data. First of all, a dual origin of neuroglia seems highly improbable.[1] The neuroglial fibers in our preparations have such a uniform and unique morphology and chemistry that it seems difficult to believe that one part of them originated from the mesoderm and the other from the ectoderm i.e., from two different origins. The only feasible option would be to suggest that our neuroglia as a whole either arose from the mesoderm or ectoderm.

Unfortunately, our method is not suitable for embryological analyses, since in the earlier stages of development, which are the only ones considered here, there are no separate fibers. We can therefore only rely on a theoretical argument to reconcile our results with the views of other authors.

[1] When we refer to neuroglia here, we are only talking about the blue fibers that are apparent in our preparations.

Our results are consistent with the notion expressed by His, as we have already indicated above. If Deiters cells are assumed to be of mesodermal origin, then these cells are connective tissue cells, and the fibers produced by them are connective tissue fibers, which always behave independently of the cells i.e., in their terminally differentiated state, they do not present with protoplasmic cell processes.

This assumption would not be inconsistent with the fact that neuroglia (based on our meaning of the word) is different from real collagenous connective tissue, because other tissues of the same group also reflect such deviations e.g., the elastic tissue. One would have to surmise that the migration of those components took place very early during development, when connective tissue precursor cells are still not terminally differentiated and could therefore give rise to a specialized connective tissue variant only intended for the central nervous system and thereby deviate from the remaining types of connective tissues.

Unfortunately, we have to do without this convenient way out. The evidence supporting the ectodermal origin of neuroglia is so compelling, particularly since it was obtained using different methods, when compared to the unsubstantial evidence supporting the mesodermal origin of Deiters cells,[1] that the conclusions drawn by His cannot be considered very helpful.

The same could be said about Jastrowitz. The reader may remember from the historical background chapter that Jastrowitz simply cut through the issue by declaring that the ependymal epithelium was an endothelium. This allowed Jastrowitz to assume a genetic connection between "spider cells" and ependymal cells, only he did not consider the latter to be the matrix of the former, but vice versa, in other words, he considered spider cells to be the matrix of the ependymal endothelial connective tissue. But (apart from the fact that ciliated endothelia were something unheard of at the time) the evidence supporting the ectodermal origin i.e., the epithelial nature of ependymal cells, has since become so compelling that Jastrowitz himself would have abandoned his old views by now.

Consequently, there is no other alternative but to assume a single ectodermal origin of Deiters cells i.e., in terms of our meaning of neuroglia.

[1] Refer to Kölliker, "Handbuch der Gewebelehre des Menschen" [Handbook of human histology], 6th edition, Vol. 2, p. 141.

There is however one other possibility to grant neuroglia an ectodermal origin and at the same time avoid the paradox of their epithelial nature. Schrader[1] namely observed that the epithelial scaffold is only a temporary embryonic structure in the cerebellum of teleosts, and that the definitive neuroglial scaffold arises from undifferentiated heterogeneous ectodermal cells. These cells would not need to be like the terminally differentiated epithelial cells (Schrader does not take a position on this) but could also just be connective tissue and arise from the epithelial-like endodermal layer similarly to mesodermal cells. Accordingly, the ectodermal cells of the medullary plate would have to have acquired "connective tissue determinants", just like embryonic stem cells in the germ layers.

But even if one can concede that particularly in the cerebellum the "caenogenesis" type of neuroglia structure in the sense of Lenhossék is the dominant structure, there can be no question of a generalization of the findings of Schrader. There are too many observations that very clearly show that the most diverse direct transitions from epithelial to neuroglial cells occur without the need for an interspersed heterogeneous cell component.

These transitional stages certainly remain in some animals, even in higher-order animals, even in humans, and they may even persist throughout adulthood, at least in the form of ependymal fibers. Neuroglia therefore is not only genetically related to the ectoderm in general, but more specifically to a real epithelium, in the postembryonic sense.

Since neuroglia are a type of connective tissue component, their epithelial origin is certainly very paradoxical. As the facts are not amenable to an alternative interpretation, the anguish of a paradox is not helpful, and it is best to just accept the facts.

It is perhaps a little easier to accept the paradoxical nature of neuroglia because the medullary plate epithelium exhibits entirely different properties which also deviate from the characteristics of all other epithelia. Quite apart from the fact that these epithelia become interspersed with interstitial fibers during older age which is different from the behavior of any other known epithelial mass, the histogenic behavior of the medullary plate in particular is very peculiar and paradoxical.

[1]"Die morphologische und histologische Entwicklung des Kleinhirns der Teleostier" [The morphological and histological development of the teleost cerebellum], "Morpholog. Jahrbucher" [Morpholog. yearbooks], Vol. 21, p. 625 ff.

The medullary plate epithelial cells after all give rise to profusely branched neurons i.e., (quite apart from their peculiar physiologic features) which diverge from any of the other types of cells that other epithelial cells differentiate into. In addition, early embryonic neurons acquire the ability to migrate, which is also quite unheard of in other epithelial structures. The morphology of embryonic neuroglial cells, with their multitude of long extensions, also differs from all other known epithelia. Among the sheer number of paradoxical properties of the medullary plate cell descendants, adding another one hardly makes a dent, but this characteristic deserves to be highlighted nevertheless: namely the generation of differentiated fibers, which is only very remotely analogous to the production of cuticular components in the other epithelia. With respect to these cuticular components, it is perhaps not entirely coincidental that our method also allows us to differentially stain these types of components.

Thus, nature achieves the same morphological and biological effect in two completely different ways: it generates connective tissue from mesoderm to function as a support component and produces neuroglia to function as a connective tissue component from ectoderm. If we have come to accept that the shape of embryonic neuroglial cells is very different from other epithelial cells, one may also come to terms with the modification and independent nature of the fibrous components in the adult.

Although most of this chapter presented arguments which were purely theoretical, embryologists will eventually have the final say on the matter. In the meantime, however, there is no escaping the fact that neuroglia behaves like connective tissue both in terms of morphology and biology.

## Chapter 6:

## Other histologic features of neuroglial fibers.

So far, the discussion has focused on the histologic peculiarities of "neuroglia" fibers, and their relationship to the cells. We deferred any further description of the microscopic behavior of the fibers because the nature of the fibers had to be elucidated first. Given that we are now in a position to make the necessary comparisons with the previously reported data, we can discuss other properties of these structures. Such comparisons were not amenable as long as the identity of our fibers which we have so far referred to as "Deiters cell extensions" etc. had not been definitively established.

Similar to the relationship of neuroglial fibers to the neuroglial cells discussed earlier, the fiber properties described in this chapter apply to the entire central nervous system, to the grey and white matter, to the cerebrum, cerebellum, spinal cord, and so on. Differences between these tissue types only pertain to the proportion and distribution of neuroglia, but these differences alone are significant enough to make very substantial distinctions between the different regions of the central nervous system. We will focus on these differences in the topography chapters. For now, let us focus on the common characteristics shared by the fibers.

1. The fibers are more or less straight (not in the mathematical sense, of course), or they run in rigidly curved bends. They are never tightly curled. If, they do form tight multiple turns, this reflects that the specimen has shrunk. It is even possible to artificially recreate these tortuous fibers, by subjecting central nervous system tissue to a vigorous oxalic acid treatment.

If tissue shrinkage is already apparent macroscopically, more or less tight tangles of fibers will also be apparent microscopically and reflect the degree of shrinkage.

2. The fibers appear quite solid; they are not hollow. They all appear as filled blue dots on transversal sections, and not as rings, as would be expected if the fibers were hollow. We therefore cannot agree with the observation of Frommann and that of Lavdowsky in more recent times.

3. The fibers are quite smooth, they do not have a "granular texture", nor circumscribed buds or thickenings. But this is only apparent from freshly embedded and meticulously hardened tissue samples. Conversely, a spinal cord sample cut in a non-hardened state with white matter protruding from its cut surface i.e., which already displays cadaverous swelling of the myelinated masses, almost certainly will contain fibers showing granular decay (or fibers that will no longer take up stain at all, see below). This cadaveric decay of the fibers was first described by Frommann, but Virchow had already noted more generally that "neuroglia" are destroyed by postmortem effects.

The cadaveric decay granules are initially small and follow the direction of the fibers; with more severe postmortem damage, the granules become larger, with the small droplets literally flowing together, and the resulting larger droplets lie farther apart and are distributed in an irregular fashion.

The granule formations eventually seem to dissolve; very badly affected samples certainly no longer take up any stain. Granules of tissue samples in the earlier stages of decay stain more heavily than normal fibers.

The varicose neuroglial fibers (cell extensions), which some authors (using the Golgi method) depict or describe, are probably nothing other than structures already altered by cadaveric changes.

It is unclear what causes this granular decay. I already pointed out that the cadaverous swelling of myelin seems to play a role here, in my 1890 preliminary communication. What seems sure is that, as I already stated at that time, white matter appears to decay first. It is not all together inconceivable that neuroglial fibers softened by the cadaveric changes may be shattered by the swelling of the myelin sheaths.

4. Just like varicosities, neuroglial fibers in our preparations have not acquired any moss-like or other attachments. Ramón y Cajal described such structures in embryonic neuroglial cells and in lower-order animals. For these types of cell extensions, the one and same extension may appear smooth and subsequently acquire a moss-like texture depending on the surrounding tissue layers. If one does not want to assume that these extensions are artifacts, then one has to perhaps consider that they correspond to a transient phylogenetic or ontogenetic developmental stage, which has left no residual traces in the developed central nervous system of humans.

5. Finally, our fibers never exhibit any of those conical or flask-like extensions so often described with the Golgi method. According to these descriptions, the attachment of "cell extensions" to the vessel boundaries where the surfaces are unobstructed, should always become wider like this at their end. In our specimens, these attachments are not thickened in any way; the fiber is as slender and uniform to its end as it was in its earlier trajectory. As our staining is selective, results obtained with our method are in any case the most definitive ones. It can therefore be surmised that, in this instance, the Golgi method stains some other component which is not a fiber or has a different chemical composition to that of the fiber.

It is difficult to determine the exact nature of this "something". It may be a glue component (which is not visualized with our selective stain, of course). It may also be that the silver stain forms deposits between the surface of the organ and the terminal ends of the fibers (that often appear to be crookedly bent), which simply results in an artifact.

6. The fibers occur in a variety of thicknesses, ranging from the very finest, which are only visible on well stained slides, to 1.5-micron fibers. The larger diameter fibers only occur in pathological samples, most notably in progressive paralysis of the cerebral cortex. However, somewhat thinner but still quite thick fibers are sometimes also observed under apparently normal conditions in humans, particularly in the dorsal horn of the spinal cord and the corresponding parts of the medulla oblongata. These fibers also radiate from the centers containing the nuclei, so that if one wants to refer to these structures as "cells" based on the old meaning of the word, one may call them "monster-cells", as I did in my 1890 preliminary communication.

These very striking structures in apparently normal tissue seem to have been hitherto missed altogether. The thick fibers in progressive paralysis, on the other hand, have been seen and imaged many times (as "cell extensions", of course).

We now come to two questions concerning neuroglial fibers, which are very much of secondary importance but have been highlighted as important issues by histologists in more recent times.

7. The first issue is whether neuroglial fibers divide, or not. This question is important enough in its own right because it would help differentiate ganglion cell extensions from neuroglial fibers, and also ganglion cells from the neuroglial cells themselves (n. b. in Golgi preparations). However, as we previously indicated in Chapter 3, data contributed by various authors do not allow to make this kind of distinction, such that this aspect bears no interest. Nor should the importance of this question be remotely compared with the identical question in relation to ganglion cell extensions. In nerve tissue, the branching of cellular extensions is of primary importance in terms of physiology, because it bestows the ability to make an immense number of neuron connections, — a feature which is not even considered possible for an intercellular component.

We shall therefore only briefly mention that we have not observed any division of the fibers in our preparations. The divisions observed in Golgi preparations can (if embryonic tissue samples are excluded) perhaps be explained by the fact that silver impregnation may merge the contours of two fibers that are close to each other, making them appear as one single fiber, much like Ranvier suggested in his example of a co-stained glue component, but the question is far too trivial to investigate in more detail.

8. A second equally subordinate question is whether or not neuroglial fibers anastomose. Here, too, the importance of the question in terms of nerve tissue has been quite wrongly transferred to interstitial tissue. In the case of nerve tissue, the proof of the absence of anastomoses is very significant in terms of physiology because the absence of anastomoses between neurons is consistent with neurons existing as insulated structures.

The absence or presence of anastomoses between interstitial tissue components is in contrast something absolutely insignificant, — and yet histologists have even gone as far as to accredit Golgi for being the first to state that the fibers do not anastomose, for essentially showing that the fibers formed a "network of touching strands" and not a "mesh of interconnected strands"!!!

Readers that are not familiar with modern neurohistology nomenclature will be surprised that the words "network" and "mesh" are conceptually different since a mesh is also a net. Afterall, a wire mesh is a specific type of wire network. A "mesh" is an intertwining of filaments, cell extensions and the like, where these structures fuse or anastomose at the points of contact, as opposed to a "network" which does not include any anastomoses.

Given the marginal significance of this question, it suffices to say that we did not observe any anastomoses, as far as the tangle of fibers would allow us to determine.

To guard against any potential misinterpretation, however, it should be noted that both the absence of divisions and of anastomoses could often only be observed by altering the focus of the microscope, consequently our drawings which reproduce images obtained without changing focus do not reflect tissue level differences. Divisions or anastomoses that did in fact not exist may however be inferred in our drawings.

## Chapter 7:

## General neuroglial fiber topography

The topographic arrangement of neuroglial fibers is very diverse, although quite distinct for individual regions of the central nervous system. This diversity does however follow some specific principles that at least allow us to establish some general rules about the distribution of neuroglia

1. The first overriding principle which really has no exceptions is the presence of a thick layer of a very closely interwoven network of neuroglial fibers under the epithelium of the ventricles and the central canal. It is the densest such network to normally occur in the central nervous system (Compare Panel III Figs. 2 and 3, Panel X Fig. 1, Panel XI, Fig. 1 etc.).

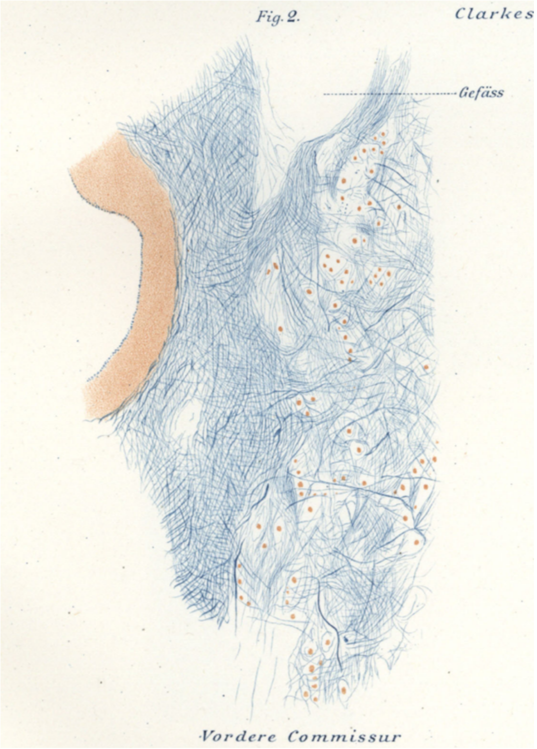


*Fig. 2 from Panel III. Central canal with anterior commissure in a child. Magnification B. (axis cylinder outlined).*

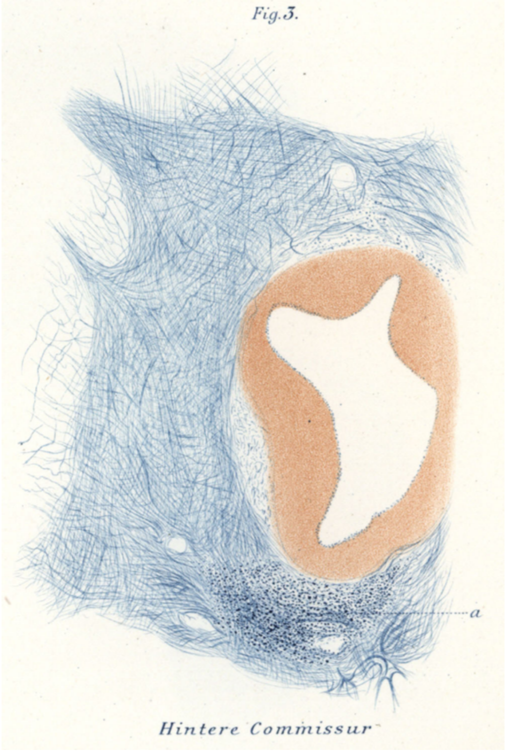


*Fig. 3 from Panel III. Central canal with posterior commissure in a child. Magnification B. Bottom, vertical fibers (a).Nuclei have not been included in this panel.*

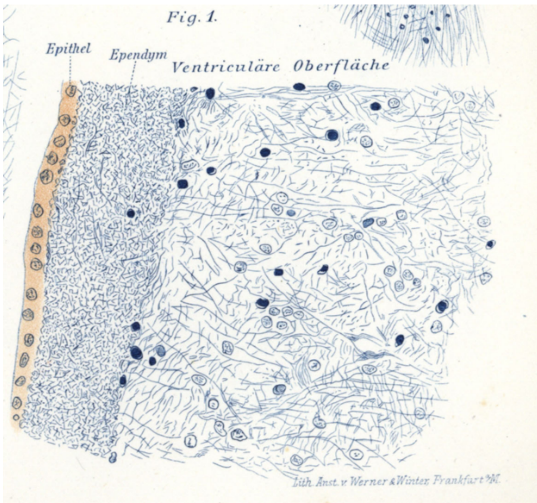


*Fig. 1 from Panel X: Ependymal surface of the hippocampus. Magnification C. Left ependymal layer, right radial fiber layer.*

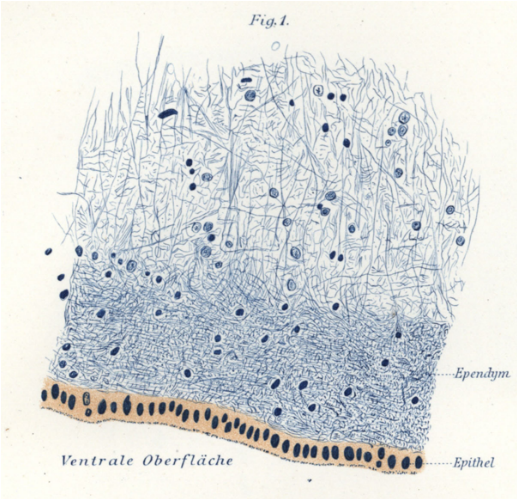


*Fig. 1 from Panel XI: Ventral (ependymal) surface of the column. Magnification C. Bottom, ependyma.*

An apparent exception to this rule occurs only at the choroid plexus. The choroid plexus is also covered with ventricular epithelium, but the ependymal neuroglial mass under this epithelium is only found in places where the plexus connects to the nervous masses (e.g., at the fimbria). In these parts, neuroglia continues on for some distance through the attachment of the choroid plexus. All other parts of the choroid plexus, however, do not have neuroglia but connective tissue under the epithelium; no neuroglia can be detected in the deeper layers of the plexus either (with the exception of the nerve tissue attachment sites).

The density of the ependymal neuroglial masses has been appreciated, to some extent, for a long time. Virchow already highlighted these regions some 50 years ago.

Our staining method particularly emphasizes the bulkiness of neuroglia since it distinctly stains each individual fiber. The network is so dense that there is hardly room for anything else besides the lymph (or whatever else fills the space between the strands), and yet we know, for instance, that masses of nerve fibers are embedded in the posterior commissure of the spinal cord.

If you look more closely though, you will notice that there is still enough room for the finer nerve fibers. The initial impression when looking at these dense neuroglia masses is rather the result of a purely psychological process. Any tissue part that is completely stained appears somewhat overbearing. When stained components are arranged very close together, it is all too easy to get the impression that they dominate the space all by themselves.

2. Another principle, but not entirely without exceptions, is that the outer surfaces of the central nervous system also contain compacted neuroglia, which is however not generally as tightly woven and as thick as the ependymal accumulations (compare Panel I Figs. 2 and 3, Panel VII Fig. 4, Panel IX Fig. 1, Panel X Fig. 2 and Panel XI Fig. 2).

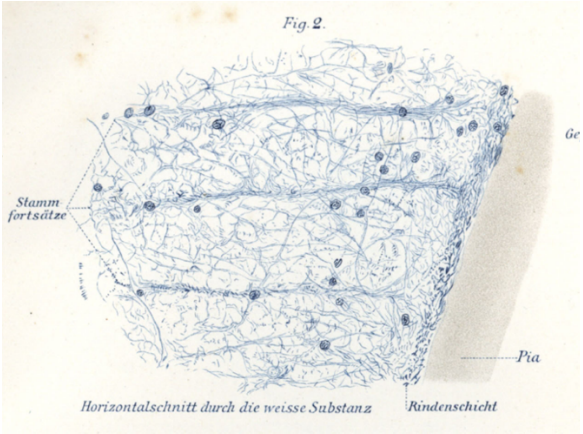


*Fig. 2 from Panel I: Transverse section through spinal cord white matter. Lateral strands, peripheral region with pia. Magnification C.*

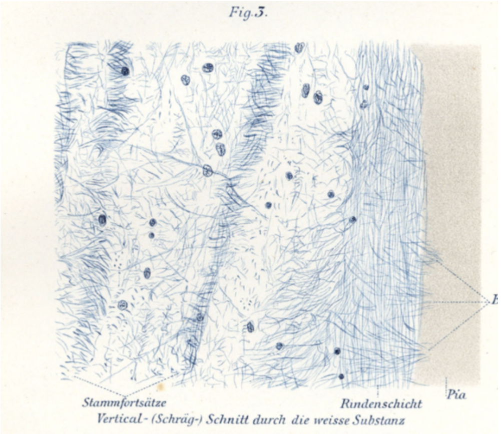


*Fig. 3 from Panel I: Oblique vertical section through spinal cord white matter. Magnification C. Right, pia, followed by cortex layer with bundle formation towards the pia. In the middle and left several trunk processes.*

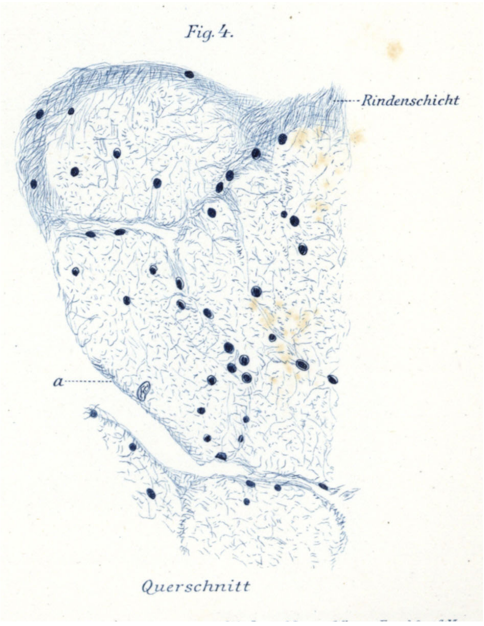


*Fig. 4 from Panel VII: Opticus, transverse section. Magnification C. Top, the compacted outer cortex layer. (a) Peripheral layer of a bundle.*

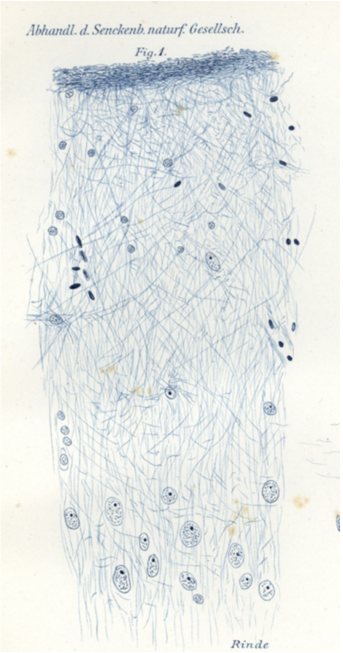


*Fig. 1 from Panel IX: Cerebrum cortex. Temporal lobe. Magnification C. (a) Cortex layer. (b) Radial fiber layer.*

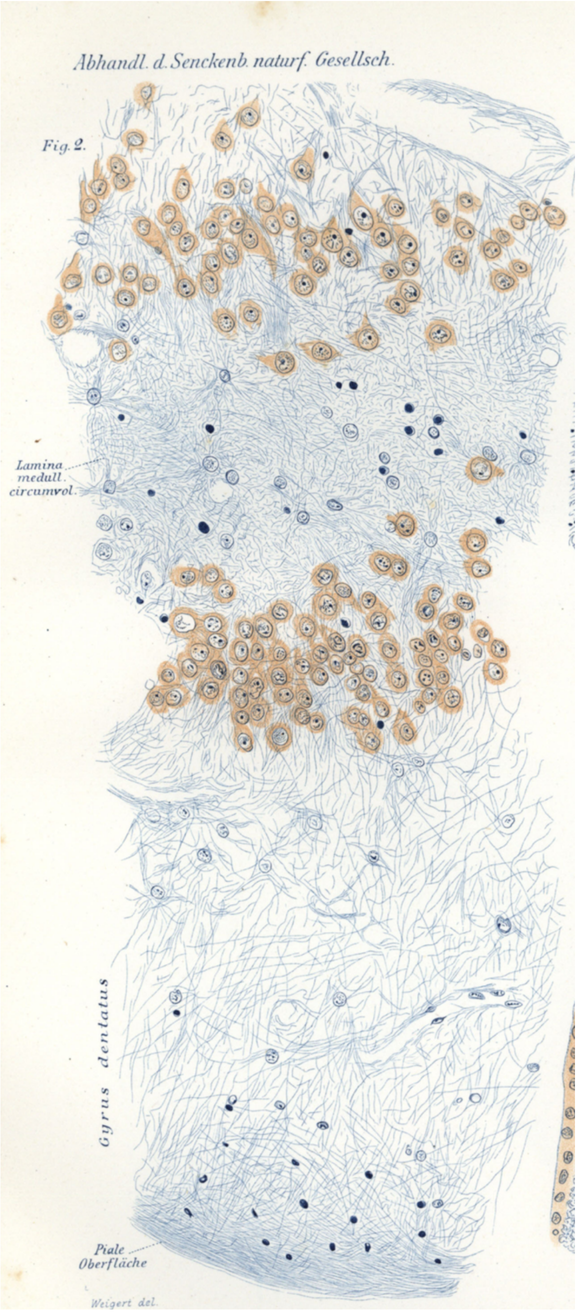


*Fig. 2 from Panel X: Gyrus dentatus. Magnification C. Bottom pial surface. This figure is composed of two drawings, the top and bottom parts of the same image. The drawings fit together perfectly. Their boundary is marked in the figure by the constriction of the center.*

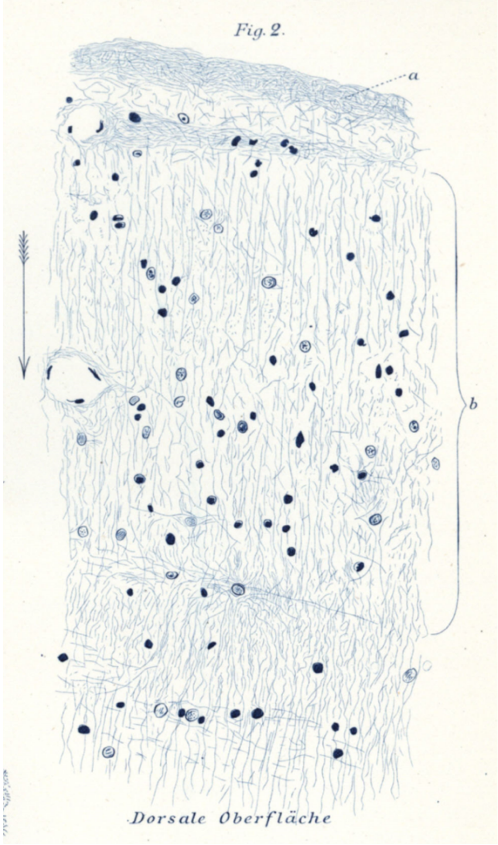


*Fig. 2 from Panel XI. Dorsal surface of the column. Magnification C. (a) Cortex layer. (b) Radial fiber layer.*

This fact has been known for a very long time in the case of the spinal cord; the spine's cortex layer has long been regarded as a particularly dense neuroglial accumulation and was at some stage even believed to not contain any nerve tissue components at all. Clarke and Frommann were the first to described this in more detail. The cortex layer of the cerebrum was first described by Golgi, and our staining method readily convinces that nearly all parts

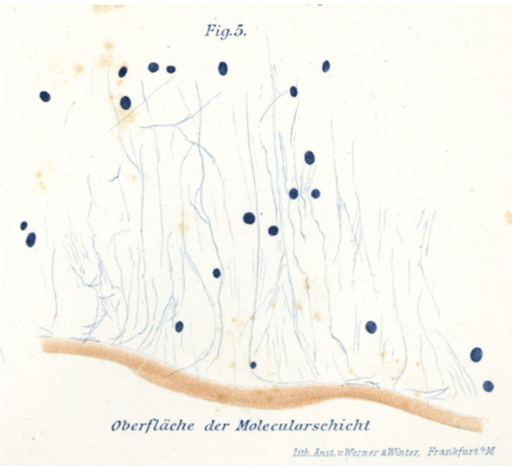

*Fig. 5 from Panel IX: Superficial region of the molecular layer. Magnification B. Bottom, pia mater.*

of the central nervous system contain such compact cortex layers, with the single exception, also first reported by Golgi (1871), namely the surface of the cerebellum, as I also pointed out in 1890 (Panel IX Fig. 5). Admittedly, the picture changes in pathological cases, and in progressive paralysis, for example, a typical dense "cortex layer" is often observed on the surface of the cerebellum.

3. These first two principles not only apply to the adult central nervous system, but also to a certain extent to the inner and outer surfaces, which existed in the embryo, but disappeared during development. Much like the surface of the ocean still bears the traces of an earlier passage of a ship's keel, the former inner and outer surfaces that have disappeared in the adult still contain residual traces of neuroglial accumulation that are more or less thick, more or less long, and more or less dense.

Since we will make several references to this compact neuroglia in relation to the topography (e.g., at the hippocampus), and considering that a new word is needed to describe these structures, we will refer to them as "keel stripes" to reflect the simile mentioned above and "stripes" because neuroglia predominantly appear as stripes. These keel stripes may perhaps in future be used to answer specific developmental questions. These keel stripes still maintain contact with an inner or outer region of the surface at one end, the other end is in the depth of the corresponding parts of the central nervous system.

4. The outer surfaces and the subepithelial parts are not the only regions characterized by compacted neuroglia, other areas with surface-like demarcations are also found internally i.e., in the depth of the nerve tissue. These compactions are also observed:

a) Where white matter nerve fibers form detached bundles. In this context, the surface of the bundles often forms a compacted peripheral layer. These compactions are not only less pronounced than the actual cortex layers of the free surfaces, or even the ependymal masses, but they also only occur on coarser bundles, and are not always easy to discern. These peripheral thickenings are observed at the pyramidal junction (Panel V, Fig. 3) and at the optic bundles (Panel VII, Fig. 4a) for example.

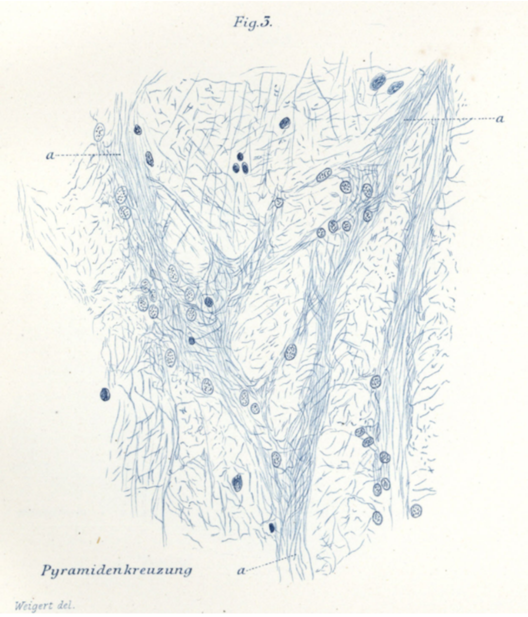


*Fig. 3 from Panel V: Pyramidal junction Magnification C. (a) Compacted peripheral layers.*

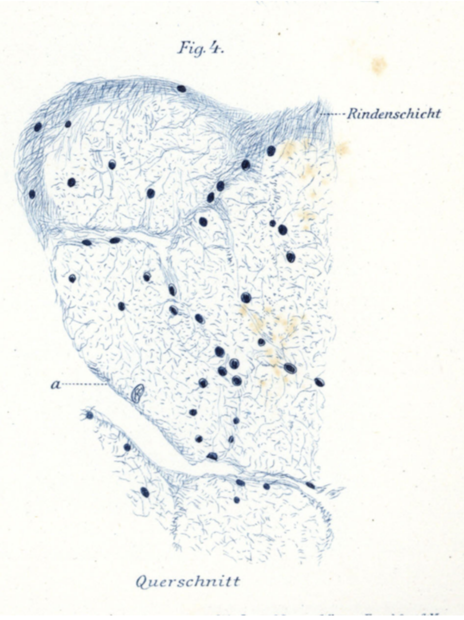


*Fig. 4 from Panel VII: Opticus, transverse section. Magnification C. Top, the compacted outer cortex layer. (a) Peripheral layer of a bundle.*

b) Also, minor and not at all regular are the neuroglia compactions around the large ganglion cells (e.g., Panel II, Fig. 2 and others), the "neuroglial baskets".

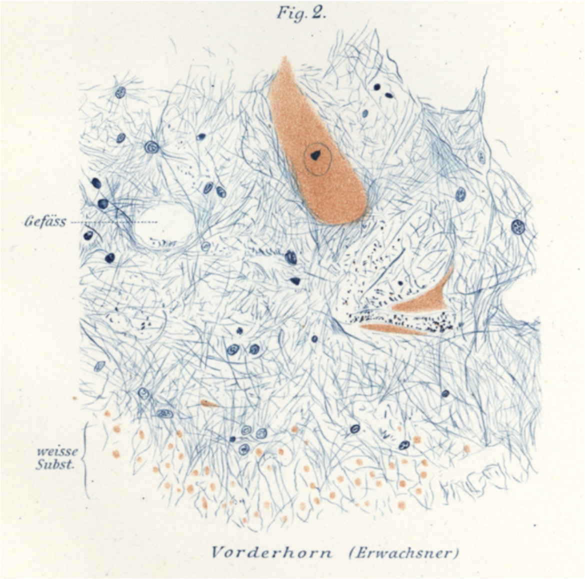


*Fig. 2 from Panel II: Peripheral region of the anterior horn in an adult. Magnification C. At the bottom, axis cylinders are marked in this case.*

These are particularly abundant around the large cells of the anterior horns of the spinal cord, as well as around the analogous structures of the medulla oblongata and the pons. They are often found around the scattered unicellular ganglion cells in the medulla oblongata and the pons. But they are completely missing or only implied as isolated filaments, where neuroglial fibers are very sparse or not present at all in the broader surroundings of the ganglion cells. This is the case in the deeper layers of the cerebral cortex, for example. Apart from that, not all of these principles can be visualized.

c) Neuroglia masses may become very dense at the edges of the spaces containing the vessels, they are even more dense there than on the outer surface.

This has also been known for a long time and was already emphasized by Virchow. The extensive thickening of neuroglia is only observed in the vicinity of larger vessels; these neuroglia thickenings are much more insignificant around smaller vessels, where they are sometimes simply implied (compare Panel IX, Figs. 2 and 3). Just like neuroglial fibrils are completely or almost completely missing in the broader surroundings of the vessels, I have also observed that neuroglial accumulation around the vessels is either completely missing or only implied by fine sparse fibrils. This is the case in the depth of the cerebral cortex.

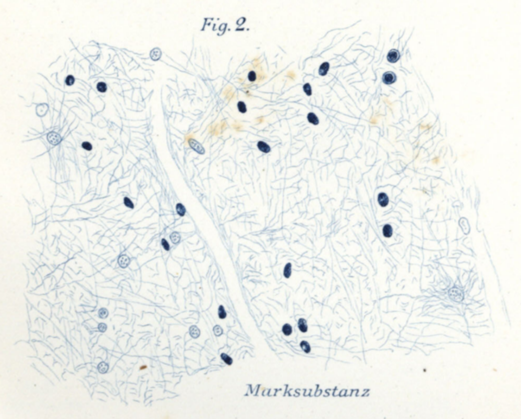


*Fig. 2 from Panel IX: White matter of the cerebrum. Magnification C.*

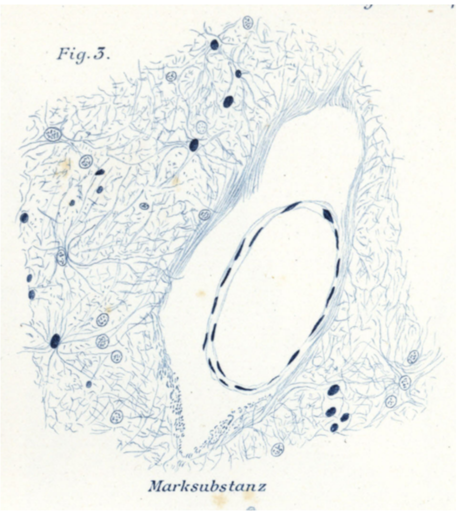


*Fig. 3 from Panel IX: White matter of the cerebellum. Magnification C. In the middle, a vessel. At the bottom edge a vessel with transversely sectioned fibers.*

Neuroglial fibers surrounding the vessels are mostly oriented parallel to the vascular axis (intrinsic fibers of Lloyd Andriezen[1]). But Lloyd Andriezen quite rightly draws attention to the fact that this seemingly parallel arrangement is actually spiral-like. Consequently, pure longitudinal sections, as well as transversal sections, allow to visualize neuroglia in the vicinity of the vessels as fibers and not as dots. But they still appear as dots when the fibers are sectioned perpendicular to their trajectory (see the bottom of Fig. 3, Panel IX, which illustrates fibers as dots). The coils of the spirals can sometimes be so narrow, that even on pure longitudinal sections of vessels, neuroglia appear as long fibers.

But spirals, that are more parallel to the axis with respect to the concentric trajectory of the neuroglial fibers, are not the only arrangements found around blood vessels. Some fibers are also arranged in a radial direction, often further away, but traveling towards the vessel where they join the other fibers, bending obliquely (extrinsic fibers of Lloyd Andriezen). Very characteristic images arise when these extrinsic fibers are traced to a center of nuclei. These fibers were first described, but not quite accurately, by Roth[2]. He had used paraffin preparations, a technique which was still in its infancy at that time, and therefore obtained peculiar shrinkage artifacts. This led Roth to believe that radial fibers passed through an empty (lymphatic) space before they reached the vessel.

[1]On a system of fiber-cells surrounding the blood-vessels of the brain of man and mammals, "Internationale Monatsschrift für Anatomie" [International Monthly Journal of Anatomy], 1893.

[2]"Zur Frage der Bindesubstanz in der Großhirnrinde" [Investigating the connective tissue component of the cerebral cortex], "Virchows archiv" [Archives of Virchows], Vol. 46 (1869), p 243.

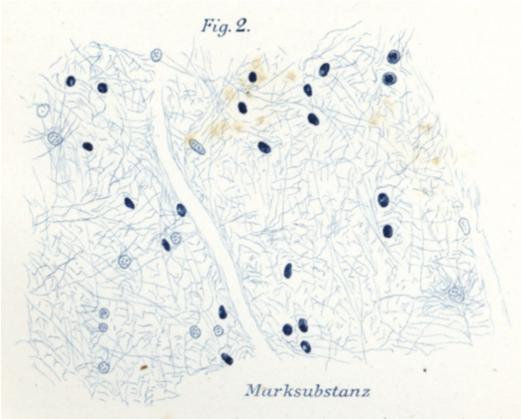


*Fig. 2 from Panel IX: White matter of the cerebrum. Magnification C.*

Golgi[1] subsequently corrected the error and was thus the first to describe extrinsic fibers more factually (as "cell extensions", of course). Such fibers can often be seen in our preparations (e.g., Panel IX top of Fig. 2).

They are often very abundant around somewhat larger vessels, as specifically illustrated in Panel VIII Fig. 2 which shows a vessel boundary in the pedunculus cerebri. The vascular space, whose content has been omitted in the drawing, is shown to the right. Very abundant extrinsic fibers, which can be traced to nuclei centers, radiate toward the glial sheath of the vessel from the left and disappear in it. It is worth pointing out again (see. p. 69) that conical thickenings at the site of attachment are not observed in our preparations but are quite commonly described in Golgi preparations. These radial fibers are observed particularly clearly and regularly in the cerebral cortex in cases of progressive paralysis i.e., a region where they are normally rarely found using our method. In progressive paralysis the cerebral cortex often features a large number of newly formed "astrocytes", some of which appear to be the "normal" type, and others that are so-called "monster cells" (Refer to p. 69).

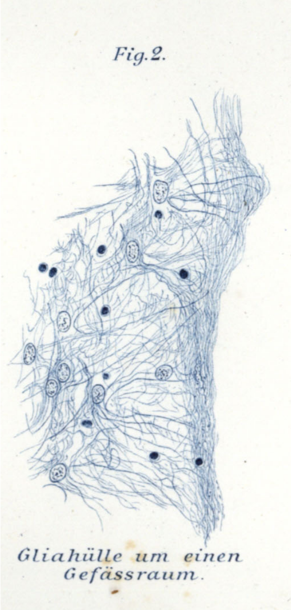


*Fig. 2 from Panel VIII: Glial sheath surrounding a vessel region from the crus cerebri. Magnification C. Right, the vessel region. The vessel itself was not drawn.*

The often very thick radiating fibers have the pronounced tendency to extend towards the vessels in a more or less vertical-radial direction, and eventually insert themselves there (always without conus). —

As the reader may recall, Golgi (refer to the note on p. 41) believed to have found something so strange in this close and intricate connection of the neuroglia with the vessels that he used his observations to argue against Ranvier's evidence which supported the fibrous nature of the "cell processes". We previously (in 1. c.) pointed out that the neuroglia to vessel relationship is no more intimate or complex than that of elastic fibers, for example, and also justified why Golgi's objection was incorrect. In addition to what we said in 1. c. however, the whole neuroglia compaction around the vessels is only a part of the widespread "cortex layer structures".

[1]Collated papers p. 6 f., Panel I, Fig. 4.

The vessels are something just as foreign to the central nervous system as the actual pia mater, which means that the nerve tissue to vessel boundary is nothing other than an internal surface, which corresponds to the outer surfaces of the brain and spinal cord. — If we also consider that neuroglial fibers are a support component, and that the arrangement of such support components in many locations has been shown to be based on mechanical principles, the intricate arrangements of neuroglial sheaths around vessels seems a little less perplexing. We will by the same token be less surprised by the outer cortex layers often exhibiting quite analogous arrangements, even though they do not entirely mirror those of the innermost cortex layers i.e., at the vessel boundaries, because of the deviating mechanical requirements.

We also observe an actual cortex layer i.e., a dense neuroglial mass, on the outer boundary, which corresponds to the intrinsic fibers of the vessels. The dispersed fibers that radiate in or out from this cortex layer perpendicular to its surface are analogous to the extrinsic fibers. The mechanical significance of the neuroglial sheath for the vessels in particular will be discussed in more detail towards the end, when we discuss the physiologic function of neuroglia in general.

5. As far as the general topography of white matter in the central nervous system is concerned, as a general rule virtually all myelinated nerve fibers in white matter are separated from each other by neuroglial fibers (See Panel I, Fig. 2, Panel VI, Fig. 1, Panel VII Figs. 3 and 4, Panel IX, Figs. 2 and 3 etc.). This forms a whole network of more widely spaced-out strands in the myelinated mass.

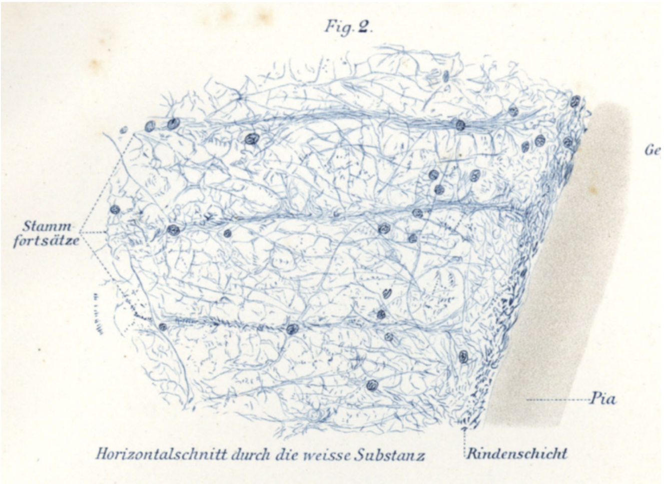


*Fig. 2 from Panel I: Transverse section through spinal cord white matter. Lateral strands, peripheral region with pia. Magnification C.*

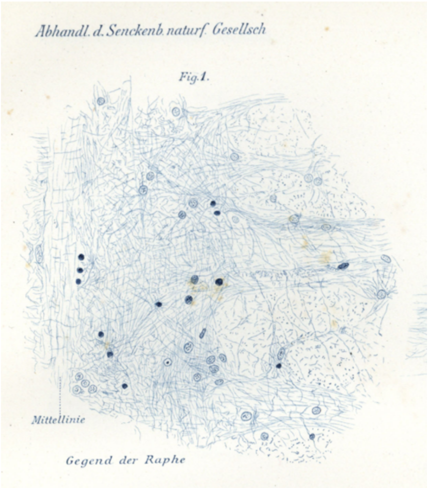


*Fig. 1 from Panel VI: Raphe of the medulla oblongata with adjacent tissue. Magnification C.*

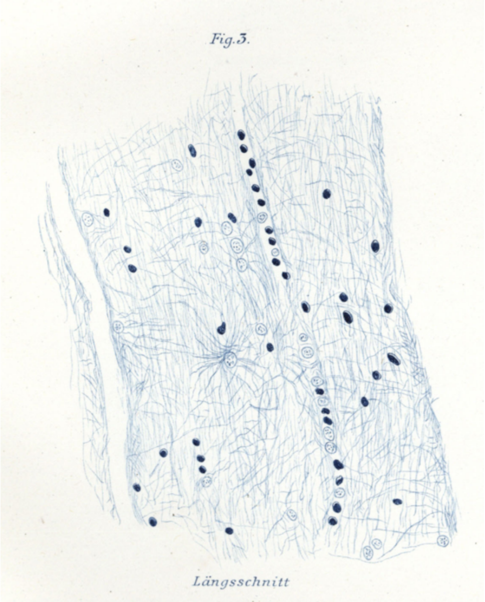


*Fig. 3 from Panel VII. Opticus, longitudinal section. Magnification C.*

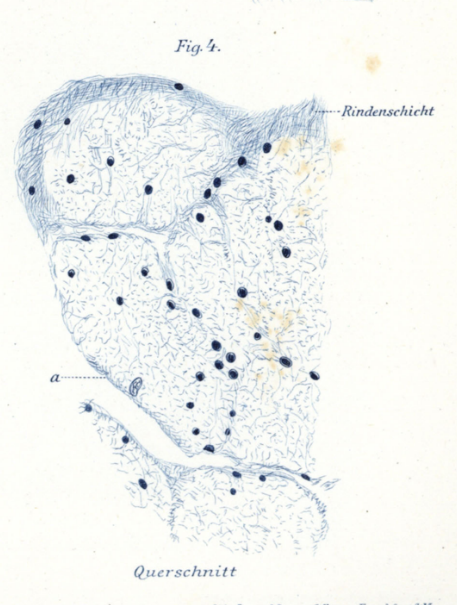


*Fig. 4 from Panel VII: Opticus, transverse section. Magnification C. Top, the compacted outer cortex layer. (a) Peripheral layer of a bundle.*

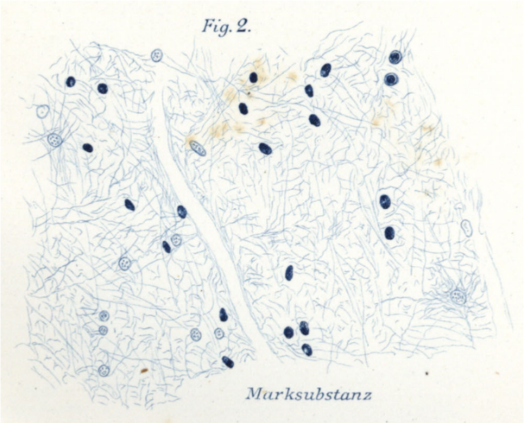


*Fig. 2 from Panel IX: White matter of the cerebrum. Magnification C.*

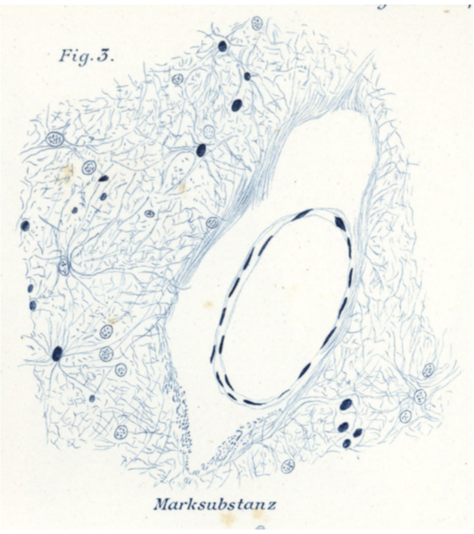


*Fig. 3 from Panel IX: White matter of the cerebellum. Magnification C. In the middle, a vessel. At the bottom edge a vessel with transversely sectioned fibers.*

But this principle only applies to the actual white mass. Where grey matter occurs between the individual myelinated fibrils, it may well be (very abundantly) interspersed with neuroglia, but it need not be present either, this is notably the case for the dense radial fibers which fan out in the deep layers of the cerebral cortex. This region does not contain white matter, but grey matter, and as we will show in Paragraph 6, does not abide by any general principles. The neuroglia network is also not uniform in white matter.

Although in general nerve fibrils are separated from the other by neuroglia, the number of neuroglial fibers between each pair of nerve fibers varies greatly. In the inner parts of the medulla oblongata, in the cerebrum and cerebellum, etc., pairs of nerve fibers are often separated by a single neuroglial fiber or very sparsely juxtaposed neuroglial fibers. In other cases, e.g., in specific regions of the medulla oblongata or the spinal cord (particularly in its upper regions) in the outer periphery, whole bundles of neuroglial fibers are inserted between every pair of nerve fibrils. The same applies to the upper regions of the spinal cord near gray masses which themselves contain very abundant neuroglial fibers e.g., near the anterior horns.

In general, the location of the white strands greatly influences the number of neuroglia that it contains. Extraordinarily dense neuroglial masses often occur where white masses pass tightly underneath the ventricular epithelium or even close to the ependyma.

This type of fiber configuration is observed in the striae acusticae, which is directly covered by epithelium (refer to Panel VII, Fig. 2), in the anterior commissure of the spinal cord, and where the medullary component of the cerebellum and cerebrum meets the ependyma, etc.

Likewise, the white masses adjacent to an outer surface or to an immediately adjacent cortex layer contain more neuroglia than more distant sites. Outer parts of spinal cord white matter etc. likewise contain more neuroglia than deeper-lying parts. — Keel stripes behave in the same manner as the corresponding surfaces, which they emanate from. — The formation of bundles was discussed on p. 74. —

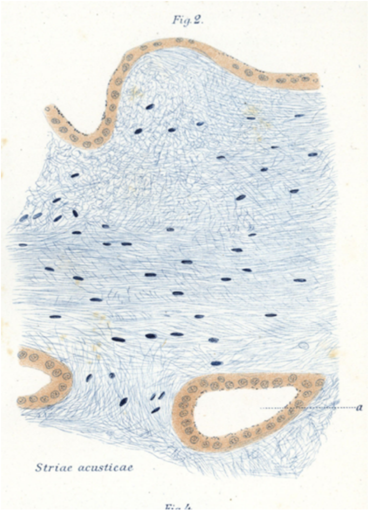

*Fig. 2 from Panel VII: Striae acusticae. Magnification C. In a cyst-like space.*

The orientation of the fibers in white masses is never entirely uniform, but invariably one orientation significantly dominates over the other. In the cerebrum and cerebellum, the orientation of neuroglial fibers follows that of nerve fibers. In the spinal cord, neuroglial and nerve fibers are perpendicular to each other. The influence of the outer and inner cortex layers is also striking here. If there are white masses in the vicinity, very often abundant radial i.e., perpendicular to the surface, potentially also perpendicular to the orientation of the nerve fibers, neuroglial fibers extend from the cortex layers into the white masses (Refer to Panel XI, Fig. 2). At the inner cortex layers i.e., at the ependyma masses, this is suggestive of an ependyma fiber effect, since these fibers must run in this direction. Given the same phenomenon is also observed on the outer layers of the cortex, this assumption is however not tenable, but must be due to some as yet unidentified reason, possibly of a mechanical consideration (as is the case for the intrinsic fibers of the vessels).

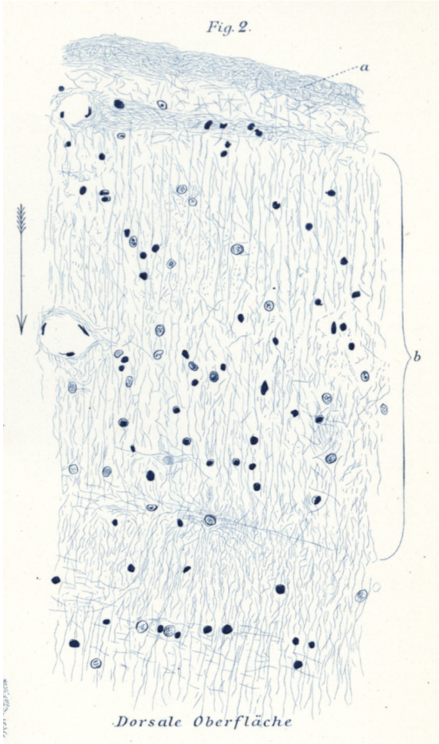


*Fig. 2 from Panel XI. Dorsal surface of the column. Magnification C. (a) Cortex layer. (b) Radial fiber layer.*

6. Grey matter, excluding the ependymal masses of course, does not abide by any general principles. This is indicative of the shortcomings of past investigation methods which have been unable to identify or have only been able to partially identify many of the differences present in this region. Popoff's claims that the strands of the neuroglial network are generally further apart in grey matter (see p. 27) relative to white matter is therefore incorrect (assuming that the reference has been faithfully reproduced). It is also not feasible to establish a general rule that neuroglia is more or less abundant in grey matter compared to white matter: neuroglia are simply more abundant in some grey matter than in other grey matter. Sala y Pons' attempt to substantiate differences in the distribution of neuroglia in the grey matter must also be considered unsuccessful. Sala y Pons believed that grey masses which contain extensive myelinated nerve fibers also contain more neuroglia compared to grey masses with less myelinated nerve fibers. This is however not the case as we will see later in the specific topography chapter. The cerebellar layer and the deepest layers of the cerebral cortex contain an abundance of myelinated nerve fibers, yet only very few neuroglia, to mention just two examples.

In the great diversity of neuroglia networks in grey matter, we can at least say that there are a number of very different types of network: among these the Stilling nucleus, the cerebral cortex nucleus etc. But it is probably somewhat premature to differentiate these specialized types of neuroglial networks in more detail.

7. There is no evidence of even the slightest transition between neuroglial fibers and nerve tissue structures. Nerve tissue components, such as ganglion cell bodies and their coarser protoplasmic processes, as well as the thicker myelinated nerve fibers, are clearly visualized in our specimens, because they take up the counterstain. Neuroglia is invariably sharply separated from these very apparent nerve tissue components. The intracellular neuroglial elements of the ganglion cells described by Rohde in lower-order animals[1] are completely absent in humans. Although neuroglial fibers are often found in close proximity to the bodies of ganglion cells and form the previously mentioned denser networks in some regions,

[1] "Ganglienzellen und Neuroglia" [Ganglion cells and neuroglia], "Archiv für mikroskopische Anatomie" [Archives of Microscopic Anatomy], Vol. 42, p. 423 ff.

not a single neuroglial fiber has ever been observed to penetrate the body or the apparent process of a ganglion cell[1].

As far as the myelinated fibers are concerned, Paladino (and also Colella) claimed that a neuroglial scaffold could also be identified within myelin sheaths. There is no evidence of this in our preparations either. The functional significance of these scaffold components identified by Paladino is of course a separate question. But these components are not neuroglia in our sense of the term. These structures may well be artifacts as Kölliker previously suggested, but resolving that particular question is beyond the scope of the current account. —

Golgi of course also postulated a specific, alternative association of neuroglia to the neurons. Golgi thought that the protoplasmic processes connected with the neuroglia. Admittedly our preparations do not detect finer dendrite extensions. Only the coarser dendrite extensions can be identified because they stain with the counterstain, while the finer ones appear as the previously extensively discussed "molecular mass". We have never observed these structures to be "connected" with neuroglial fibers; in all grey masses where neuroglial fibers can be identified at all, there is an absolute and sharp contrast; the sides and ends of the fibers absolutely and sharply contrast against their surroundings. Based on our preparations, we can therefore only conclude that dendrites are located in close proximity to neuroglia. This fact has certainly not been disputed by anyone. A more intimate connection as descried by Golgi cannot be observed in our preparations using the new staining method. The question of whether this type of connection can be proven using another technique must of course be left unanswered.

[1] To avert any misconceptions, it should be specifically noted that a number of images from our drawings, such as Fig. 4 in Panel IX, are not consistent with the Rohde, Paladino, Colella and Golgi descriptions discussed above. By adjusting the microscope focus, it is even more convincing to observe that neuroglial fibers are superimposed on top of nerve tissue cells and do not penetrate into them. Our drawings cannot reproduce this aspect because images are captured at the same focus.

## Chapter 8:

## Specific neuroglial fiber topography

Preliminary comments.

The following description of the specific topography of neuroglial fibers is only a sketch. A very, very long study will be needed to complete this sketch. As the reader would have gathered from the foreword, the author had insufficient time to provide any more than the following account.

The extensive interlacing of neuroglial fibers is an almost esthetically pleasing sight, "che l'occhio contempla sempre con sommo incanto[1]", to quote Petrone. The late Hermann v. Meyer, to whom I often showed the preparations, was perhaps right when he used to say: "These are very dangerous preparations. You fall in love with their beautiful shapes and forget to study them". It is a slight exaggeration — because one does actually study the specimens, but it is very difficult to give a good description of the networks. Although illustrations best convey the diversity of the interweaving of the fibers, mine only provide a weak reflection of reality. I am very inexperienced at drawing, so I was limited to reproducing images as I saw them and adjusting the microscope focus as little as possible. We are so used to focusing through tissue sections and merging images from multiple tissue planes in our minds, which gives the impression that the fibers are much more abundant throughout the tissue than they perhaps are in reality when simply considering one single plane of the tissue.

### 1. Spinal cord.

#### A. Cortex layer.

The spinal cord "cortex layer", with its varying thicknesses, has been recognized since ancient times. This layer is not entirely devoid of nerves, as was once believed, but consists predominantly of neuroglial fibers.

[1]Italian in the original: which the eye always contemplates with great enchantment

Frommann arguably provided the best description of this layer, and this is a good starting point to convey our own observations, if we bear in mind that our perceptions deviate from the generally accepted views and if we correct for those deviations accordingly. Frommann described the spinal cord cortex layer to consist of (I, p. 28):

"a dense, tight meshwork of fibers and branching cells, forming a sheath of alternating thicknesses over the entire surface of the spinal cord. The diameter of the cortex layer varies between 0.01 and 0.06 mm, but is mostly between 0.02-0.03 mm, as Goll also indicated. It is most dense near the posterior and the stronger anterior roots, at the entrance to the posterior fissure and often in the vicinity of regions where there is a retraction of the surface, and the entire thickness of the cortex layer sinks into the white matter". The spaces between the strands of the neuroglial network are often so small that they are hardly larger than the diameter of the strands themselves. I did not observe a common recurring arrangement of the fibers relative to each other but rather a more general tendency of the fibers to align themselves in two directions, either in the longitudinal or transverse direction. Fibers that are aligned sometimes cross over at acute angles or run parallel, and the gaps between them are filled with a meshwork of extremely delicate fibers." "The larger fibers are 0.001-0.002-0.003 mm in diameter[1], they are bright, shiny, have sharp outlines, and stain pale red with carmine; the smaller fibers are one half to one third of a diameter thinner, and do not appear to stain with carmine."

The only thing that needs to be added to Fromman's description is that the fibers in our preparations, the smaller as well as the larger ones, stain dark blue, which means that the direction of the fibers, as far as the fiber tangles allow, can be followed much further than Frommann was able to in his carmine preparations. The main fiber mass usually extends more or less obliquely and tangentially, with radial fibers frequently occurring in addition to vertical ones, and where the stronger processes of the cortex layer penetrate into the depth of white matter, they often form inwardly converging bundles that continue on to disperse in ways that will be described in a moment. (Panel I, Figs. 2 and 3)

[1]I have never seen fibers as thick as Frommann described in any of the normal specimens I examined. Refer to the author's comment on p. 69.

The cortex layer is normally clearly separated from the pia mater, but it happens often enough (Panel I, Fig. 3) that fiber tufts protrude like the hairs of a brush above the otherwise smooth surface of the cortex layer, as Frommann had also noted. — The cortex layer yields thicker fiber bundles in the larger nerve roots which generally run parallel to the nerve fibers, but only some of the way, — also previously described by Frommann (I. p. 30). (Also compare to my 1890 communication).

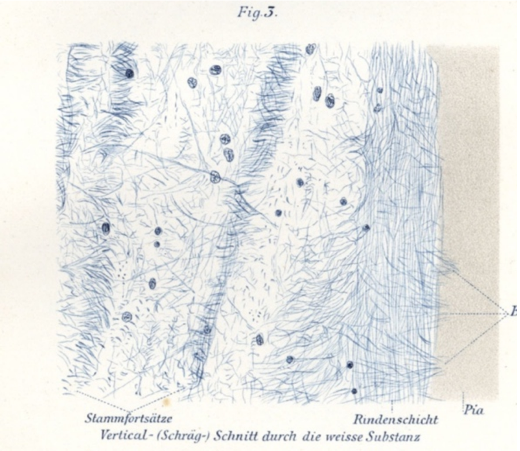


*Fig. 3 from Panel I: Oblique vertical section through spinal cord white matter. Magnification C. Right, pia, followed by cortex layer with bundle formation towards the pia. In the middle and left several trunk processes.*

The cortex layer is also interspersed with nuclei, around which one can hardly ever find spider-like fiber structures that extend out of the dense mesh. The nuclei are usually of the smaller type, with dense chromatin masses, and generally without surrounding astrocyte-like fiber structures. It is also noteworthy, as already pointed out by Golgi (See Fig. on p. 159), that nuclei in the cortex layer are quite sparse compared to the dense tangle of fibers, — evidence which supports that it would be quite wrong to draw any conclusions about the number of neuroglial fibers from the number of nuclei i.e., cells apparent in a tissue section.

**B. White matter.**

Fibers and fiber tracks, which seem to radiate from the cortex layer, are attached to it. Sometimes they form thicker masses, which could be considered direct continuations of the entire cortex layer in a more or less tapered form, sometimes only individual fibers or groups of fibers that radiate into the internal regions (Panel I, Figs. 2 and 3). Frommann referred to these denser neuroglial masses, which penetrate from the cortex layer into the depth of the spinal cord, as "trunk processes". They surround the vessels penetrating the cortex layer from the pia and entering the white matter, mainly in a semi-radial direction. With the exception of the predominantly minor adventitial connective tissue masses around the vessels (the adventitia next to the central canal, where the vessels are vertical, are often strikingly large) no connective tissue penetrates into the white matter, as Frommann already knew and as it is now more generally accepted. The vessels divide white matter into very imperfectly separated coarser bundles, which in terms of their shape approximately resemble sections of a circle.

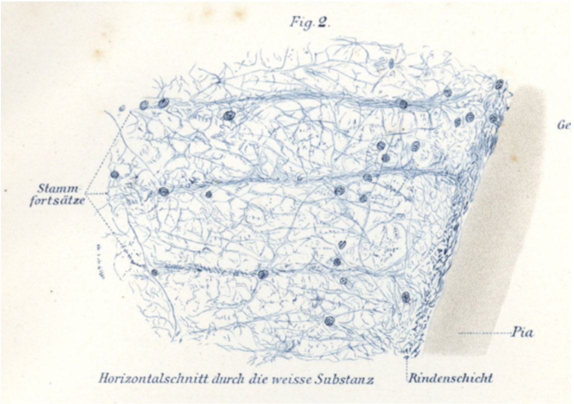


*Fig. 2 from Panel I: Transverse section through spinal cord white matter. Lateral strands, peripheral region with pia. Magnification C.*

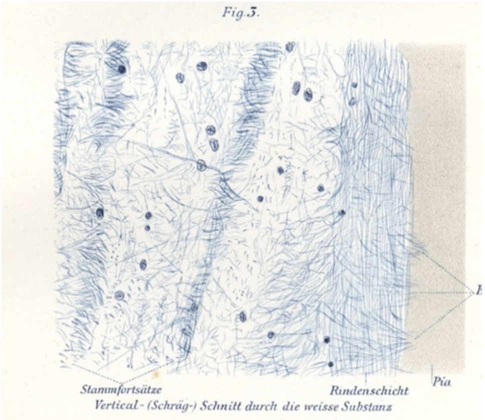


*Fig. 3 from Panel I: Oblique vertical section through spinal cord white matter. Magnification C. Right, pia, followed by cortex layer with bundle formation towards the pia. In the middle and left several trunk processes.*

A particularly large and long vessel radiates into the spinal cord at the sulcus longitudinalis posterior, and this vessel is associated with a connective tissue adventitia. These radiating structures recur in successive overlying planes, so that a series of consecutive transverse sections from the sulcus longitudinalis posterior to the posterior commissure may identify a "pia process" (vessel with adventitia).

One would presume that this process is a typical fissure, naturally surrounded by an abundance of neuroglia. In other places, however, this fissure becomes incomplete, as it were. The vessel and its connective tissue adventitia are missing in various areas on the transverse section. The midline, however, generally contains a more or less compact neuroglial layer, which symmetrically divides the posterior strands bilaterally.

Frommann described the proportions of the "septum posterius" as follows (I, p. 31): "The thickness of the septum varies from 0.001 to 0.024 mm. It is wider in the cervical and lumbar parts than in the dorsal part, where it often contains only a few fibrils." "Although it is rare, the septum is completely missing here and there, and the two posterior strands merge into each other without any interruptions. Sometimes it splits into two septa, which rejoin." "The septum's posterior region is usually wider than it is towards the commissure, only regaining width just before its transition into the commissure."

More recently, Lenhossék[1] in particular dealt with the characteristics of what is erroneously referred to as the septum posterius. I have to agree with his data, as indicated above. Lenhossék further states (p. 222): "This cleft formation is a secondary phenomenon; I believe it to be invariably associated with the entry of blood vessels into the posterior midline, and even if there are no apparent blood vessels in the transverse section, this may be because the cleft still extends somewhat further in the longitudinal direction beyond the vessels' point of entry."

There are of course multiple plausible interpretations of the vessel-free glial compaction in the midline of the posterior strands. As Lenhossék seemed to think, the radiating vessels may follow one another so densely from plane to plane that the glial vessel sheaths invariably fuse together in the vertical direction. Alternatively, the posterior strands may be considered as two large "bundles", with a common peripheral layer analogous to other such masses of strands (see Paragraph a on p. 74).

[1]In Vienna, Schaffer ("Archiv für mikroskopische Anatomie" [Archives of Microscopic Anatomy], Vol. 44) also investigated the cortex and the trunk processes mentioned above but did not contribute any new data.

But it seems to me that they are more likely to be "keel stripe structures". During early embryonic development, the spinal cord is a tissue mass extending over a large region, which closes to form a tube when the two lateral parts fuse together dorsally (the rear). Consequently, one could very well imagine that this seam in the midline of the adult spinal cord might still be a remnant of a keel stripe (p. 73 f.).

With the exception of the denser neuroglial masses that accompany the radiating vessels, the white matter is now interspersed with a loose framework of neuroglia that, in accordance with the general topographic principles, penetrate between each individual nerve fiber and its adjacent fiber. All individual nerve fibers are therefore separated from each other by neuroglial fibers.

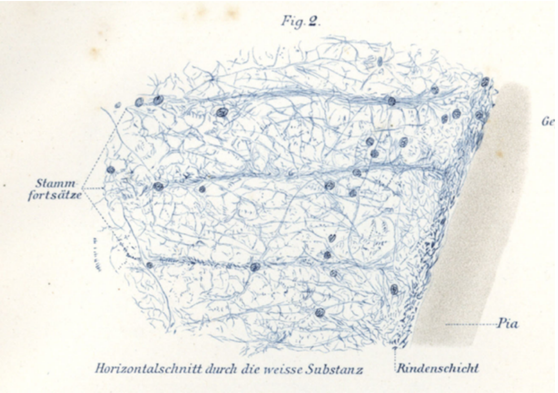


*Fig. 2 from Panel I: Transverse section through spinal cord white matter. Lateral strands, peripheral region with pia. Magnification C.*

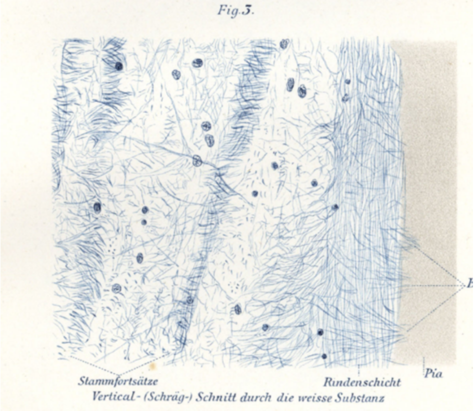


*Fig. 3 from Panel I: Oblique vertical section through spinal cord white matter. Magnification C. Right, pia, followed by cortex layer with bundle formation towards the pia. In the middle and left several trunk processes.*

In terms of the direction of these fibers, if one examines the fibers on transverse sections of a spinal cord, the initial impression is that, at least as far as the anterior and lateral strands are concerned, there are virtually only rather horizontally running scaffold fibers. But there are also vertical or oblique fibers, which are not as obvious because they are sparser and appear as dots or in short sections. Longitudinal sections are more convincing (Panel I, Fig. 3) and show that such fibers are also present. The non-horizontal fibers of the posterior strands are more abundant, at least in older people, and therefore more apparent, even on transverse sections. Pathological conditions are characterized by a tremendous predominance of vertical fibers. In young children, however, the mesh of neuroglial fibers in white matter is an incredibly regular radial system with very few fibers oriented in different directions. The

appearance is remarkably reminiscent of the primary neuroglial scaffold formed by the ependymal fibers in the embryo.

This uniformity is lost in adults i.e., the radial horizontal fibers are joined by many fibers running more or less obliquely to them, but also fairly horizontally, quite independently of the direction of the previously mentioned vertical fibers (see Panel I, Fig. 2). Neuroglial masses located between the nerve fibers are more abundant in regions that are in close proximity to the cortex layer and in regions near the anterior horns than in the intermediate areas (p. 78). —

The region which includes the posterior strands deserves more detailed investigation. It is striking how often in adults, particularly the cervical cord (but also other parts of the spinal cord), it is not only characterized by the presence of vertical fibers, but also by the presence of thicker, grouped accumulations of vertical fibers, which may be suggestive of degenerative processes. These foci of dense neuroglial masses tend to be most abundant in Goll's strands. They are absent in small children. I am not quite sure whether this is part of the normal processes in adults. Lichtheim was the first to report that neuroglial growths in the posterior strands are associated with pernicious anemia. Consequently, it seems feasible that such "neuroglial growths" may also occur in other chronic diseases, such as phthisis, nephritides, carcinoses, etc. but which would have eluded detection by Lichtheim's methods at the time. Alternatively, these less prominent "neuroglial growths" may be something quite normal; this cannot currently be excluded. Unfortunately, I have not been able to access sufficiently fresh spinal cord tissue from suddenly deceased individuals lately, such that this issue must remain unresolved for now. Finally, it should be noted that the anterior roots, which radiate into the white matter as distinct bundles for some way, feature a peripheral layer, albeit delicate, with bundle-like characteristics (Panel I, Fig. 4 a).

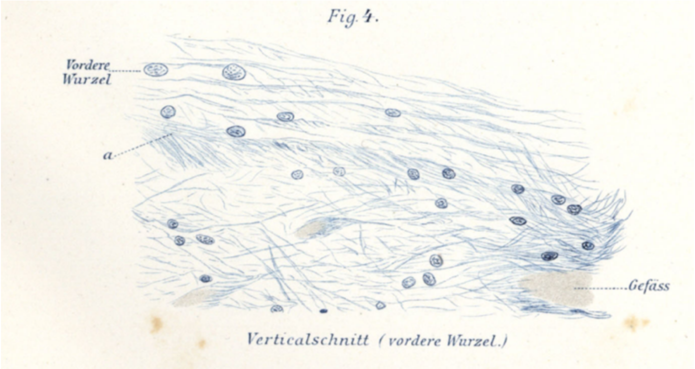


*Fig. 4 from Panel I: Entry of the anterior root. Magnification C. Vertical section. On top anterior root, (a) peripheral layer of the anterior root, at the bottom several vessels.*

Neuroglial nuclei in the white matter itself are either large vesicular structures with granular-appearing chromatin or smaller compact nuclei. "Astrocytes" are more apparent on longitudinal than on transverse sections, but not as abundantly as elsewhere in the central nervous system.

**C. Grey matter.**

While the neuroglia distribution in white matter is readily apparent, so that even Frommann described it correctly, it is quite a different story in grey matter. With the exception of the central canal region and the top of the dorsal horn, which Frommann also described fairly well and has not been surpassed by more recent work, the topographic aspects in grey matter have been depicted quite inadequately, and to a large extent downright incorrectly. As well as upholding past errors, studies based on the Golgi method also added some new misconceptions

The new edition of Lenhossék's textbook includes the only confirmation of my descriptions, which I had already published in 1890.

The Golgi method caused or confirmed the general and quite fundamental error, that the topography of neuroglia in grey matter is entirely uniform, and that there was fewer neuroglia in grey than in white matter. Both observations are wrong. Individual grey matter regions need to be considered separately, since they contain different types of neuroglial networks, and neuroglia are more abundant in most of these grey matter regions than in white matter. It is nevertheless very difficult to describe the specific features of neuroglial fibers in words, particularly in grey matter, and even the panels included herewith only provide a meager representation of the diversity and the elegance of the mesh.

A) Anterior horn.

The neuroglial network of the anterior horn has a much more uniform structure in newborns than in adults. The fibers extend primarily in the horizontal direction and form fan-shaped bundles, whose tips radiate into the extensions of the anterior horns, while the wider parts of the fans are turned inwards. The image is all the more

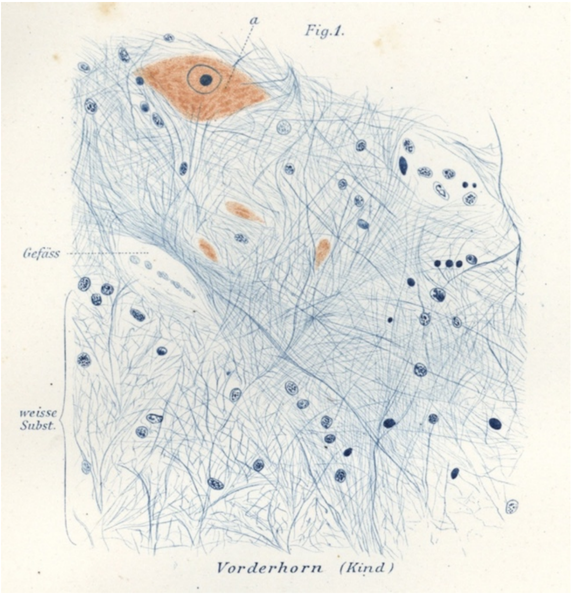


*Fig. 1 from Panel II: Peripheral region of the anterior horn in a newborn infant. Magnification C. (a) A ganglion cell with Nissl granulations. Bottom, white matter.*

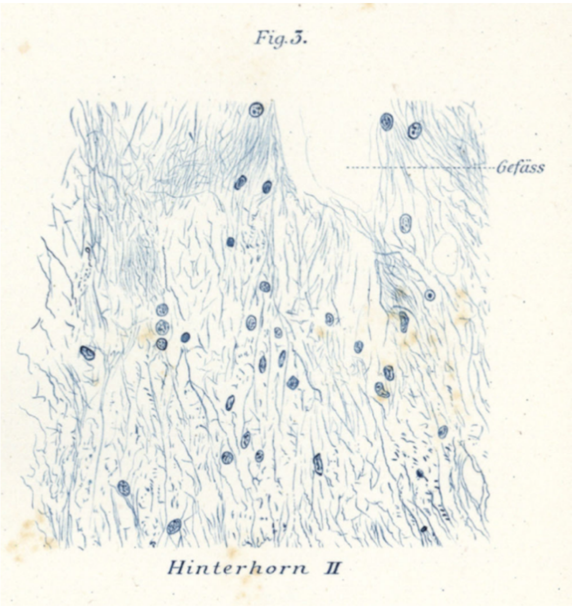


*Fig. 3 from Panel II: Anterior portion of the substantia spongiosa of the dorsal horn. Magnification C. On the top, boundary with the substantia gelatinosa of Rolando.*

elegant because the bases of the pyramids frequently overlap (Panel II, Fig. 1). Although remnants of these uniform structures are still apparent at the tips of the extensions of the anterior horn in adults, these are no longer observed in close proximity to this location nor in the whole internal region (Panel II, Fig. 3). Instead, the entire grey matter of the anterior horns is permeated with abundant meshes of fibers, which run in such different directions that, if the root entry points are disregarded, transverse and longitudinal tissue sections of the anterior horn hardly reveal any differences. — Here the vessels comply with the general topographic principles and the neuroglia appear compacted, but in proportion to their smaller size consequently the structures they form are not as thick as the vessels associated with the "trunk processes". The organization of the large motor ganglion cells has already been discussed in the general topography chapter. The minor compressions (Panel II, Fig. 3) also extend to the thicker processes of the ganglion cells in the form of accompanying fibers, whose trajectory is generally parallel to the direction of the processes.

(Panel II, Fig. 3, on the right. Here the accompanying fibers are sectioned perpendicular to their lengths and therefore appear as dots).

The anterior horn neuroglial fiber mass is quite substantial, and (with the exception of the cortex layer and the trunk processes) larger than its analogue in the white matter itself. The masses are particularly prominent at the anterior and lateral margins, where they often appear as somewhat darker hazy-looking masses to the naked eye. The density of the anterior horn neuroglial network is not at all comparable to that of the substantia grisea centralis or that of the tip of the dorsal horn. Neuroglial nuclei are found interspersed among the fibers, sometimes with, but usually without a radiating ring of fibers.

Incidentally, the fibers do not differ in any way from those found in white matter. Even though studies using the Golgi method often report the presence of short, branched fibers[6#] (cells) in the anterior horn as opposed to long branched fibers (cells) i.e., which correspond to the true Deiters cells, I find absolutely no evidence of different neuroglial components other than the typical (long branched) fibers in the anterior horns. Considering the abundance of these fibers as well as the very developed anterior horn nerve tissue material, it is difficult to conceptualize that there would be any room left for a second different "extension" type of neuroglial network, which our method also does not detect. We have of course previously mentioned (p. 73) that the argument of "not having enough space for something" is very dubious, but the issue is quite different in this particular case.

The authors that report on these "short branched" neuroglial fibers state that they should be at least as abundant as the long, branched forms, and that the number of extensions on the short-branched forms should be even more significant compared to the long branched fibers.

### b) Dorsal horn.

α. In terms of neuroglia, the tip of the dorsal horn, the Lissauer marginal zone, has already been described by Frommann, and he quite rightly complains that his Carmine stain yields very ambiguous results, because it does not allow to differentiate neuroglial fibers from axis cylinders that are particularly abundant in this region. This is even more evident with the Golgi method. The Lissauer tract stained using our method appears as an immensely dense neuroglial network, which is admittedly not as tightly woven as that of the substantia grisea centralis in the spinal cord.

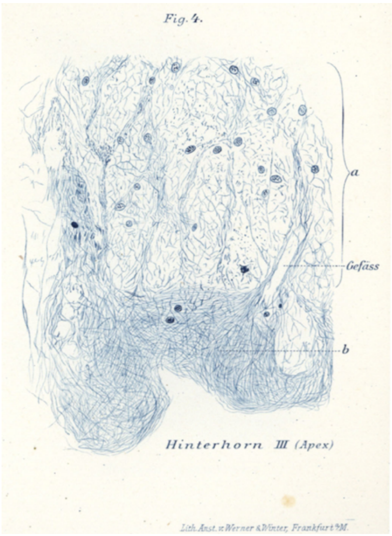


*Fig. 4 from Panel II: Lissauer marginal zone (bottom b) and substantia spongiosa (a) of the posterior horn. Magnification C.*

The fibers are partly intertwined horizontally (Panel II, Fig. 4b), partly vertically, sometimes predominantly in the latter direction. β. Substantia spongiosa. The Lissauer marginal zone is quite sharply defined against the substantia spongiosa towards the front (Panel II, Fig. 4a). The neuroglial network of the substantia spongiosa is not as dense by far. This loose neuroglial network sometimes appears broader, sometimes narrower, sometimes longer, sometimes shorter, sometimes only implied, and reflects the extraordinarily variable nature of the substantia spongiosa both in terms of shape and dimension[1]. The same can be said about the orientation of the fibers. Although radial bundles occur throughout, they do not necessarily exist in isolation, and they can sometimes fill in the spaces between the network strands with differently aligned fibers (Panel II, Fig. 4a). Further forward, towards the substantia gelatinosa, the radial fibers however generally appear as the main mass (besides sparsely otherwise oriented ones) (Panel II, Fig. 3). These then settle, often in a slightly compacted form, rather sharply against adjacent zones of the posterior horn, the substantia gelatinosa of Rolando.

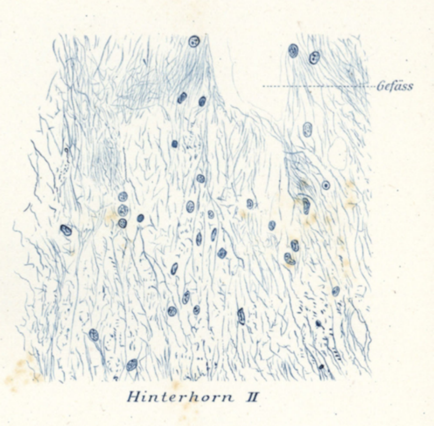


*Fig. 3 from Panel II. Anterior portion of the substantia spongiosa of the dorsal horn. Magnification C. On the top, boundary with the substantia gelatinosa of Rolando.*

γ. Substantia gelatinosa of Rolando (Panel I, Fig. 5). All descriptions of the neuroglial scaffold in the substantia gelatinosa of Rolando up to 1890 confirm the existence of a very diverse neuroglial mesh, indeed most authors claimed that apart from a few passing nerve fibers and ganglion cells the region was pretty much pure neuroglia. I was the first to assert in 1890 that the reverse was true.

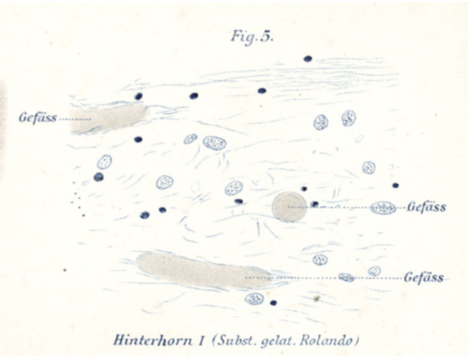


*Fig. 5 from Panel I: Substantia gelatinosa of Rolando. Magnification C.*

The substantia gelatinosa of Rolando has extraordinarily few neuroglial fibers, it is unlike any other region of the spinal cord in this regard. I was delighted that Lenhossék, contrary to all the other researchers who use the Golgi method, agreed with me on that point in the second edition of his book. The substantia gelatinosa of Rolando is of course not completely devoid of fibers, there are some fibers that are predominantly oriented radially, with additional small fibers oriented in other directions. There are relatively large empty spaces between the fibers here, which is very characteristic of this region.

[1] Lissauer, contribution towards the fiber trajectory in the posterior horn of the human spinal cord and corresponding changes induced by the tabes dorsalis. "Arch. für Psych. " [Psychology archives], Vol. 17, Book 2., p. 12, Sept. ed.

The radial fibers are partly continuations of parallel fibers from the substantia spongiosa, whose above-mentioned rather sharp contrast with the substantia gelatinosa of Rolando allows the fibers to quickly become sparser and finally disappear from the largest part of the region. Further forward, the zone with very few neuroglia gradually changes into a much more densely woven neuroglial mass, which forms the transition to Clarke's columns, respectively to the anterior horn and to the substantia grisea centralis. γ. Clarke's columns (Panel III, Fig. 1) Clarke's columns contain a meshwork of neuroglial fibers that are oriented in different directions, but these are, at least in the dorsal regions, somewhat sparser than in the anterior horns, but still by far more abundant than in the substantia gelatinosa of Rolando, as I already communicated in 1890.

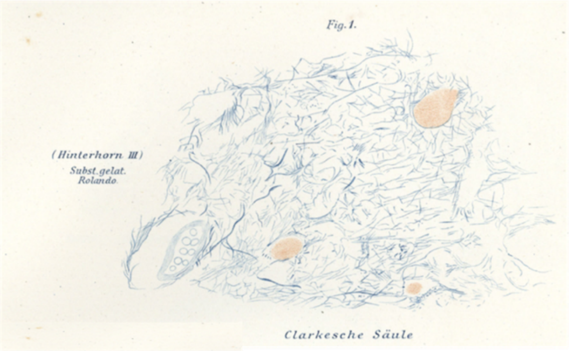

*Fig. 1 from Panel III. Clarke column. Magnification C.*

c) The central canal region

α. Substantia grisea centralis. We already pointed out in the general topography chapter that the area surrounding the central canal of the spinal cord, like all ependymal layers, is incredibly abundant in neuroglia. Neuroglial fibers are so immensely abundant in this area that in every transverse section of the spinal cord stained with our method, the area surrounding the central canal already appears as a dark blue spot to the naked eye.

This abundance of neuroglia fibers is apparent along the entire region surrounding the central canal. There is no evidence of a clearly distinct "ring commissure" intercalated between the anterior and posterior commissures, as older researchers had assumed, nor is the abundance of fibers only limited to the posterior commissure (Panel III, Fig. 3, in the child), although there are of course spaces in the dense neuroglia masses of the anterior commissure for the coarse myelinated nerve fibers (Panel III, Fig. 2, in the child). In between these individual nerve fibers, however, there is an equally dense neuroglial mesh as is normally found around the central canal, i.e., a mesh whose density cannot be compared at all with that found in the other white matter. The massive central glial heaps gradually merge laterally into the less dense anterior horns, in such a gradual manner that the majority of fibers are still visible at the sides. Towards the posterior, the contrast against the dorsal strands is rather sharp. In newborn children, horizontal obliquely intersecting fibers predominate in this fiber mass, but they are not universally present (Panel III, Fig. 3).

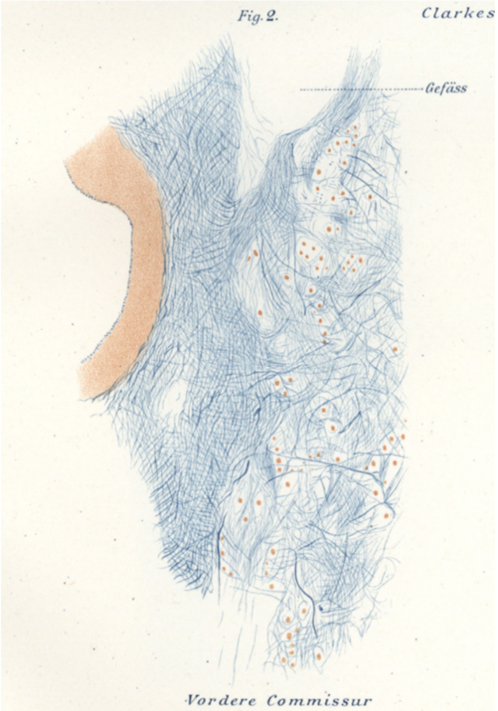


*Fig. 2 from Panel III. Central canal with anterior commissure in a child. Magnification B. (axis cylinder outlined).*

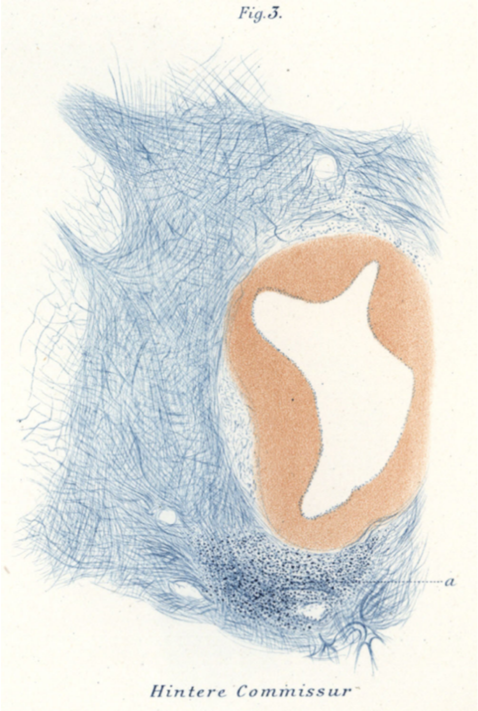


*Fig. 3 from Panel III. Central canal with posterior commissure in a child. Magnification B. Bottom, vertical fibers (a). Nuclei have not been included in this panel.*

In older individuals, there are more and more vertical fibers. Transverse sections are increasingly predominated by vertical fibers (which appear as dots and lines) even though they are still interspersed with fibers oriented in different directions.

This extraordinary abundance of neuroglia in the substantia grisea centralis, which I first described in 1890, as well as my data indicating that the distribution of neuroglia vary in the different parts of grey matter in general, have been contested in more recent times, by none other than Kölliker, on the basis of his experience with Golgi preparations. He specifically states (vol. 2, p.153)[1] that neuroglia is fairly evenly distributed in all parts of the grey matter, in the substantia gelatinosa centralis as well as in the ventral and dorsal columns and in the grey commissure, and he continues on to say that: "I emphasize this quite deliberately because Weigert draws different conclusions from his new staining of the glial fibers.

He found an immense number of blue staining fibers in the Substantia gelatinosa centralis. I interpret this result to mean that in the grey commissure both the processes of Golgi cells as well as the numerous extensions of ependymal cells take up the stain."

I have the following response to Kölliker's comment: My previous and current data solely refer to the neuroglial fibers. The Golgi method cannot accurately determine whether neuroglial fibers are present in abundance or not. Not only does the Golgi method only allow to detect cells and their associated fiber stumps, i.e. only a small portion of the fibers, but it also stains these "astrocytes" to such varying degrees, depending on the stain's fickle whims, that even if a small number of astrocytes were to be detected, this cannot be interpreted to mean that only a few astrocytes are actually present in any particular region. Furthermore, as we have already noted above in response to a comment by Golgi, the number of cells is by no means directly proportional to the number of fibers observed. This is also evident from our preparations. Although our preparations only clearly visualize the nucleus of the cell, because each individual nucleus corresponds to a cell, the number of nuclei observed directly corresponds to the number of cells present in the section. Our preparations shows that the number of fibers is not at all representative of the number of cells present in the section.

[1] "Handbuch der Gewebelehre des Menschen" [Handbook of human tissue science], 6th edition, 1893.

A priori we do not have the slightest objection against Kölliker's assumption that there are also ependymal fibers in the tangle of fibers in the substantia grisea centralis (and not only in the grey commissure, as he mentions). But there are several provisos to Kölliker's assumption: firstly, that ependymal fibers do not atrophy with age even in the elderly, as is often claimed, because particularly in very early childhood there are not as many fibers around the central canal as there are in older adults and in the elderly.
This would lead us to presume that at least in older adults the formation of neuroglial fibers follows exactly the same pattern of differentiation and separation from the cell body, as that of actual neuroglial cells, because in older adults the detached epithelial cells lie quite freely between the newly formed neuroglial fibers, without any organic connection (see Paragraph c ß, p. 94 f.).
Finally, one would have to assume that developing as well as mature ependymal fibers exhibit identical characteristics, have the same appearance, trajectory, and stain exactly the same way as real neuroglial fibers: one would simply have to presume that ependymal fibers and astrocyte fibers (neuroglial fibers) were completely identical. Accordingly, even accepting Kölliker's interpretation does not entail the slightest change to our description or interpretation of our data.
Admittedly, our method does not allow us to elicit the involvement of ependymal fibers in the generation of the human neuroglial network. We will have to leave other authors to provide the direct evidence supporting an epithelial cell involvement in the generation of neuroglial fibers. We can, however, ascertain this: if epithelial cells are involved in fiber development around the central canal, they generate typical neuroglial fibers.

The lack of understanding about glial compaction around the central canal has caused great confusion in the investigation of pathological anatomy of the spinal cord. It notably prompted the myth of "softened central gliosis" in syringomyelia. The confusion was further compounded by interchanging the terms "gliosis", i.e. the pathological proliferation of neuroglial fibers, with "glioma".

Gliomas are characterized by the proliferation of glial cells as opposed to glial fibers. Moreover, most glioma glial cells lose the ability to produce distinct fibers and remain in their original protoplasmic state. It is therefore not surprising to observe true Deiters cells in gliomas, just like those found in the embryo. The difference between glioma and gliosis is therefore comparable to that of sarcoma and inflammatory connective tissue proliferation, or to fibroma. The doctrine of "softened central gliosis" (erroneously called "softened central glioma") is now said to be a proliferation of characteristic fibrous neuroglia with softening. Neuroglia is normally very abundant around the central canal as compared to their density in normal tissue. They may appear to be less dense (in some cases at least) in syringomyelia or hydromyelia. Conversely, it is not uncommon to find truly pathological "glioses" beyond the limits of the central ependymal filum in multiple sclerosis. But these never soften, just like it has never been proven that true glioses soften — to put it simply, the perception of syringomyelia as a softened central gliosis is not supported by even a shred of evidence. I have already briefly discussed elsewhere why there may be a secondary increase in the number of glia in syringomyelia.[1]

β. Central canal. In juveniles, the epithelium rests smoothly on top of the dense neuroglial mass. The extensions of epithelial cells into this mass are not apparent with our method.

The epithelium itself is a uniform, uninterrupted row of large nuclei, with their corresponding protoplasm, which stain a yellowish color with our method.

(See Panel III, Figs. 2 and 3. note that the nuclei are not represented in these figures). Each internal epithelial cell wall contains groups of small, blue-colored granules, which I was the first to observe and described as early as 1890. The existence of these granules has now also been confirmed by Lenhossék. At the time I also speculated that these were probably cuticular deposits, and not deformed cilia hairs.

[1] "Zur pathologischen Histologie des Neurogliafasergerüsts" [On the pathological histology of the neuroglial fiber scaffold], "Centralblatt für allg. Path. und path Anat. " [Central Journal of General Pathology and Pathological Anatomy], 1890, p. 736 f.

I can now provide definitive evidence which supports this hypothesis.

The cilia hair of the third ventricle of a 15 cm crown-rump length embryo were found to be perfectly preserved. Each cell contained multiple cilia with several of the tips of these cilia turning inwards and connecting to form a pyramid structure (similarly to a rifle pyramid), but still allowing each individual cilium to be clearly defined. Since the attachment of cilia to the (embryonic) ependymal epithelium is still met with skepticism, I am including a sketch of this particular specimen (Panel IV, Fig. 1). The granules now stained underneath the attachment of the cilia. There was not much neuroglial staining apart from that, only the peripheral part of the radial fiber sections of the spinal cord were stained.

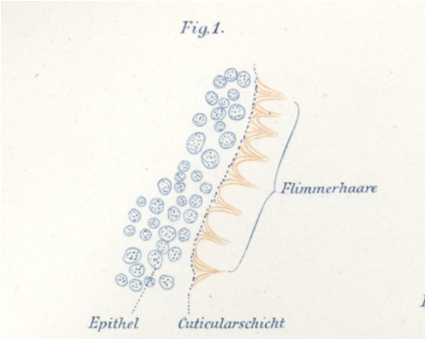


*Fig. 1 from Panel IV: Fetal ependymal epithelium with cilia. Underneath peripheral strip with dots. Magnification B.*

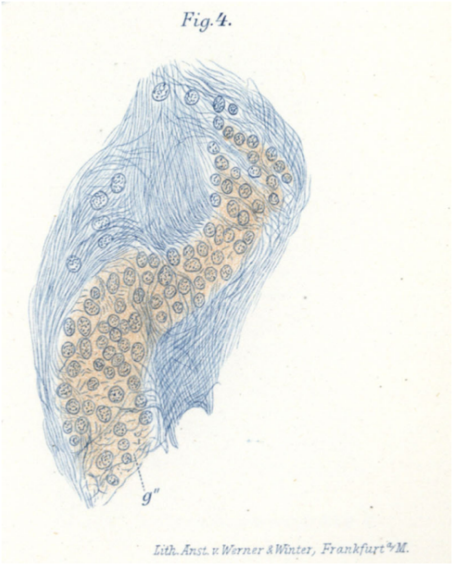


*Fig. 4 from Panel IV: The same. Magnification C. Irregular epithelial heap.*

I have not observed any other distinctly staining cilia in newborns and certainly not in older children, etc., and the integrity of the epithelium is completely maintained over the first few decades of life, at least as far as the lumen of the central canal is concerned. But the picture gradually changes, although it is not possible to precisely specify when it this change occurs. Like many of the aging process changes, they may occur earlier or later in life. One of the low-grade changes that occurs in the central canal with age is that the epithelial cells move further away from each other in some places and the spaces that are thereby created between individual epithelial cells become interspersed with isolated neuroglial fibers, mostly in the radial direction. Higher-grade changes involve the detachment of epithelial cells not only from each other, but also from their basal support, resulting in more or less large spaces that are denuded of epithelium. These spaces are infiltrated by distinct bundles of fairly parallel neuroglial fibers, which are directly connected to those of the substantia grisea centralis. Detached epithelial cells are not lost but are instead randomly scattered within neuroglial masses. Frommann had already illustrated one case of this observation. The aging process can follow multiple different pathways from this point onwards:

1. The detached epithelial cells form one or more irregular confused heaps, which together with the proliferated neuroglial masses occupy the central canal which is now devoid of lumen (Panel IV, Fig. 4).

2. A portion of the detached epithelial cells converge to form a proper simple luminal ring with a dotted inner margin.

Although the central canal appears quite normal on superficial inspection (Panel IV, Figs. 3 and 5), its lumen is considerably smaller than the corresponding lumen from a juvenile. In addition, all the other changes mentioned below (Paragraph 4) are also always present in this particular case.

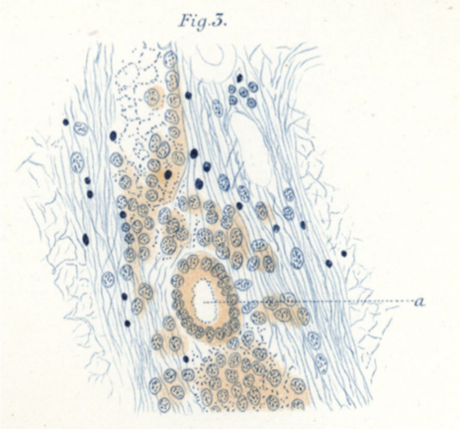


*Fig. 3 from Panel IV: The same. Magnification C. (a) Single new lumen.*

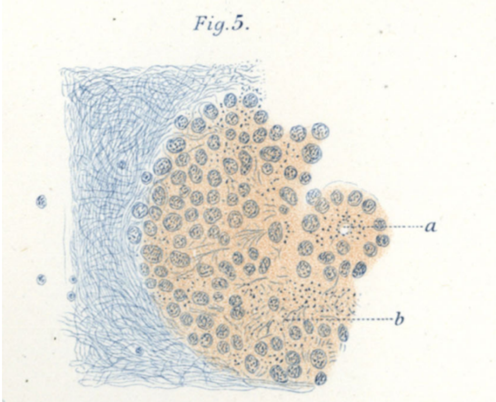


*Fig. 5 from Panel IV: Irregular epithelial heap from the lower regions of the medulla oblongata. Magnification B. (a) Newly formed lumen, (b) irregular epithelial mass interspersed with neuroglial fibers.*

3. The third possibility is as follows: Not a single lumen is formed but two or more correspondingly smaller lumina surrounded by a ring of epithelium (with dotted inner margins) (Panel IV, Fig. 2). All the small lumina are separated by immense neuroglial masses.

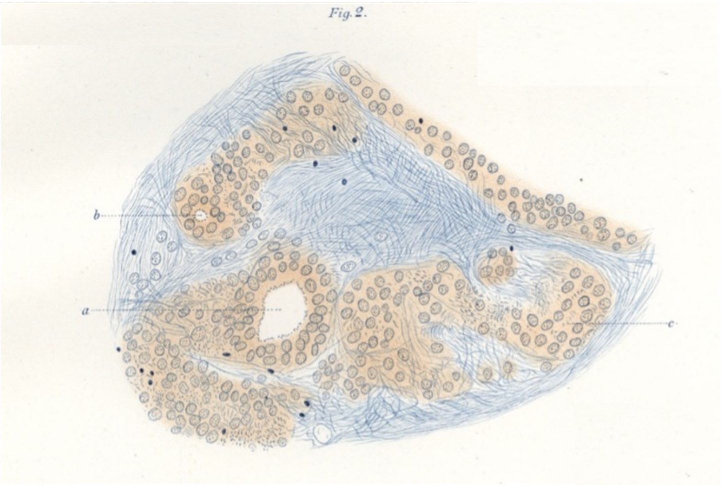


*Fig. 2 from Panel IV: Central canal in older age. Magnification C. (a), (b) Two newly formed lumina, (c) irregular epithelial heap.*

The rows of detached epithelial cells surrounding the lumen described under 2. and 3. could be held together because the epithelial cells detach together as cohesive shreds, whose ends then only have to join to form a lumen or several lumina. An alternative mechanism would be that epithelial cells are detached as individual cells, but, that when given the necessary space they migrate and adhere to each other biotactically, as Roux had first demonstrated in artificially separated embryonic cells.

4. Among all these different assemblies of detached epithelial cells, additional isolated epithelial cells are always apparent in the middle of the neuroglial mass proliferation. Sometimes they appear as completely solitary entities and are only reasonably reliably identified with our method if their protoplasmic bodies are large enough to stain a yellowish color (Panel IV, Figs. 2, 3 and 4). Some also retain remnants of their dots (possibly from the surface). At other times they appear in small heaps or in rows (Panel IV, Fig. 2). These are often joined to the ends of the (central) larger heaps or the (secondary) lumina-like keel stripes, but are separated from them by neuroglial fiber trains, or are at least surrounded and interspersed by such on all sides (Panel IV, Fig. 3). At other times they appear more parallel with the larger central assemblies, forming what could be considered as concentric epithelial stacks.

It should however be noted that in the absence of any of the previously mentioned characteristics, it is often difficult to differentiate isolated, atrophic epithelial cells from glial cells in our staining (compare Panel IV, Fig. 3 and Fig. 4).

Based on these observations, we consider the primary mechanism in the so-called obliteration of the central canal to be passive, consisting of a loosening and later the detaching of epithelia, as opposed to Brissaud[1], who considered it to be primarily a proliferation of the epithelium. Our interpretation is supported by the fact that these changes occur at a more advanced age, when the idioplastic potential of cells generally decreases and at the very least is certainly not inclined to increased cell activity i.e., to proliferation. It is further supported by the fact that even in relatively normal central canals, glial fibers can be seen growing into the spaces between the epithelial cells with age, which indicates that epithelial cells must therefore be diverging. These changes are in addition always associated with a reduction in the space occupied by the central canal, not an increase in space, as would be the case with active proliferation.

The fact that in addition to these passive processes and as a consequence of them, active processes also occur, is based on the biological principle that I have demonstrated numerous times over the last twenty years and longer, that the abolition of tissue resistance (by passive forces), is followed by proliferation processes. In this particular case, the abolition of tissue resistance is due to the detaching of epithelia (and resorption of spinal fluid?). The proliferation processes certainly primarily consist of the proliferation of neuroglial fibers beyond the barriers otherwise imposed by epithelial structures, and possibly also the secondary proliferation of the detached epithelial cells released from their reciprocal tissue pressure (and the pressure of the spinal fluid?).

Whether these new neuroglial fibers are generated solely by typical neuroglial cells or whether the central canal epithelium also contributes to the process must be left unanswered to be consistent with what was previously said on p. 92.

In contrast to Frommann's first (and only?) description of neuroglia radiating into the space of the original central canal, the "obliteration" of the central canal, an umbrella terms for all these different processes, has been known for a long time. The fragmentation of the epithelial mass on the one side and the formation of several lumina on the other was already very well described by Clarke in 1859.

[1]"Revue neurologique" [Journal of neurology], Vol. 2, p. 545 ff.

Clark says[1]: “In the human spinal cord the canal is often completely filled up, [with] what would appear to be the debris of the epithelium; for nothing is to be seen but a confused heap of nuclei, which are here mostly large and round: but sometimes in the midst of this heap there remains a small opening or canal, which strange to say is still lined or surrounded at its margin by the usual regular layer of columnar cells, and what is still more curious I occasionally find particularly in the cervical region two such secondary canals, each lined in the ordinary way.”
This description of events seems to have been completely forgotten. Even Brissaud, who generally confirms Clarke's data, also does not seem to have been aware of it.

The Golgi method might also have been inadequate to detect this phenomenon, which had been known to the older researchers, even though it was only correctly described by Frommann. This is at least what I deduce from the fact that such a thorough expert in this method as Lenhossék could even entertain the possibility that the obliteration of the central canal could have been caused by mishandling the spinal cord during the extraction, i.e. much in the same way as van Gieson demonstrated in his famous work with regard to so many other issues. But this is not even conceivable. Quite apart from the extraordinarily typical neuroglial proliferation, which could not be reconciled at all with an accidental injury sustained during extraction of the spinal cord, quite apart from the fact that this change is found in otherwise quite well-preserved spinal cords extracted with the greatest of care, it would be all too remarkable if the mechanical damage to the spinal cord were never observed in juvenile samples, but always in older specimens, while the other artifacts described by van Gieson arise in all the different age groups.
We can therefore confidently include obliteration of the central canal among the natural ageing changes of the human body. Brissaud was also opposed to considering this to be an artifact.

[1] Philosophical transactions, 1859, p. 455.

## 2. Medulla oblongata.

A macroscopic examination of a transverse section of medulla oblongata prepared using the new method reveals the following. As long as the central canal is still intact, its periphery is visible as a dark blue spot, similar to the spinal cord. As the olivary body emerges, a new feature becomes apparent, even to the naked eye; transverse medulla oblongata tissue sections treated using our method adopt a very characteristic staining pattern. Indeed, the olivary body are clearly contrasted against all the surrounding tissue as dark blue spots. The upper parts of the medulla oblongata i.e., where the central canal gives way to the ventricle, the margin of the ventricle starts out as a dark blue strip, that is washed out at its lower end, and also a darker line which corresponds to the raphe. The upper side features washed-out bluish patterns.

For the microscopic analysis, we will start with the cortical layer again.

### A. Cortex layer.

The cortex layer of the medulla oblongata is similar to that of the spinal cord, with one notable exception. Where nerve bundles run parallel to the surface i.e., tangentially, there is only a hint that the cortex layer as a distinct layer. The cortex layer is nevertheless not entirely absent in this region but has, as it were, been relocated to the interior of the tangential nerve masses, which exhibit a very rich neuroglial network, particularly with respect to their superficial features, with radial fibers also evident (Panel VI, Fig. 2, fibrae arciformes extemae).

### B. White matter.

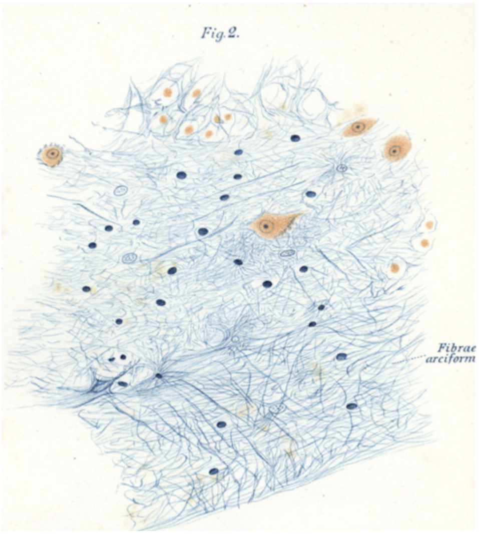


*Fig. 2 from Panel VI: Fibrae arciformes externae and pyramid nucleus. Magnification C.*

Every nerve fiber, throughout white matter, is separated from other nerve fibers by neuroglia.

But compared with spinal cord transverse sections, the neuroglial fiber arrangements appear to be much more intricate. This is because the nerve fibers in the medulla oblongata no longer extend as uniformly in the vertical direction as is the case for the main mass of myelinated fibrils in the spinal cord.

Instead, nerve fibers in the medulla oblongata are arranged as multiple interlaced bundles, and because neuroglial fibers are predominantly oriented parallel to the nerve fibrils, the neuroglial fibers also crisscross in these bundles.

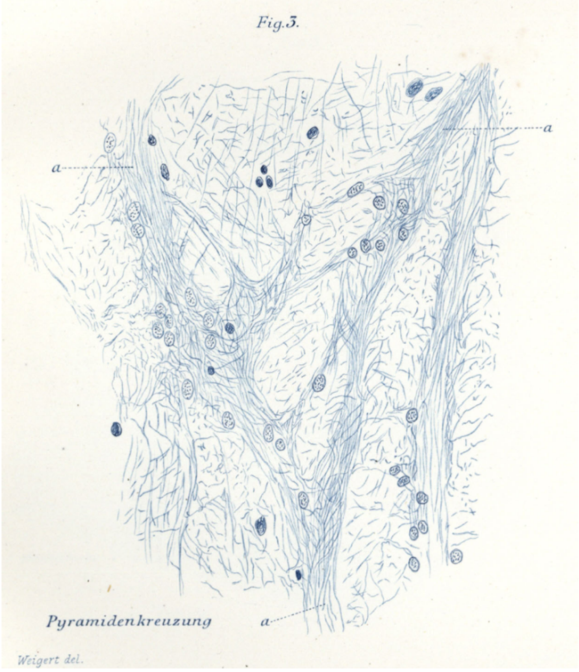


*Fig. 3 from Panel V: Pyramidal junction Magnification C.*

*(a) Compacted peripheral layers.*

Compacted peripheral layers can also be found here and there on the coarser bundles. This is specifically the case for the intersecting pyramid bundles (Panel V, Fig. 3). It should be remembered that the stacking of pyramidal fibers into bundles begins in the upper part of the spinal cord before the junction.

We already mentioned on p. 78 that this white matter region in the medulla oblongata adjacent to the periphery is particularly abundant in neuroglia.

The bundles become increasingly dispersed into individual nerve fibrils in the raphe region. These crisscross and are separated from each other by abundant, divergently oriented neuroglial fibers (Panel VI, Fig. 1). These numerous interweaving neuroglial fibers already make the raphe appear darker to the naked eye compared to its surroundings. But there is something more to it.

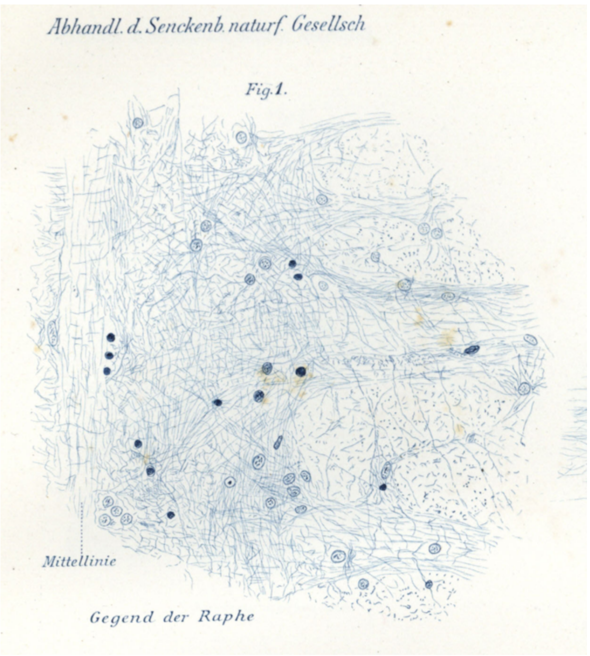


*Fig. 1 from Panel VI. Raphe of the medulla oblongata with adjacent tissue. Magnification C.*

Two more prominent compactions penetrate the raphe from the ventricular side as well as from the surface of the pia. The compacted neuroglia arising from the floor of the ventricle occupies approximately the upper third of the raphe. It is not only directly connected to the ependyma, but at first also completely coincides with this structure. This association gradually becomes looser and looser and eventually subsides towards the start of the middle third of the raphe. The second (ventral) compaction takes up approximately the lower third of the raphe. It is directly connected to the outer cortex layer, conforms to its structure, and thus does not feature such dense masses as the dorsal (ventricular) compaction strip. It also tapers off towards the middle third.

Both compactions of the upper and lower thirds of the raphe should unquestionably be considered as "keel stripes" (p. 74).

**C. Grey matter.**

The substantia gelatinosa of Rolando and the remains of the anterior horns behave like in the spinal cord. The delicate (Panel VI, Fig. 4) and wedge-shaped strand structures form a very irregular meshwork, often with slight compactions around ganglion cells. The neuroglial mass is smaller compared to that in each ventricular nucleus. Each ventricular nucleus reflects the influence of the ependyma and the dorsal keel stripe insofar as their corresponding substantial neuroglial masses very gradually recede into the depths of the nerve structure. However, even parts of the ventricular (dorsal) nucleus that are further away from the ependyma still have an abundance of neuroglial fibers (Panel V, Fig. 4: portion of the hypoglossal nucleus that is further away from the ependyma).

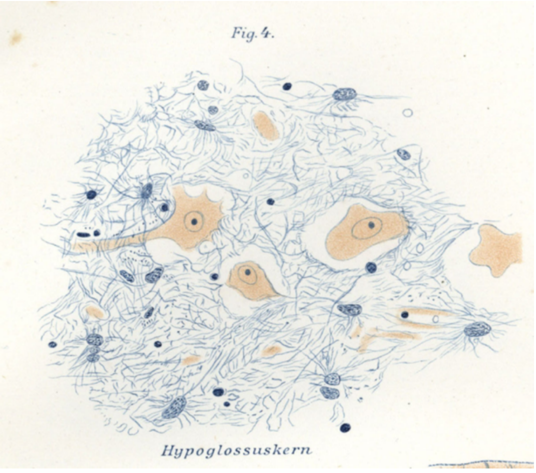


*Fig. 4 from Panel V: Hypoglossus nucleus, more distal from the ependyma. Magnification B.*

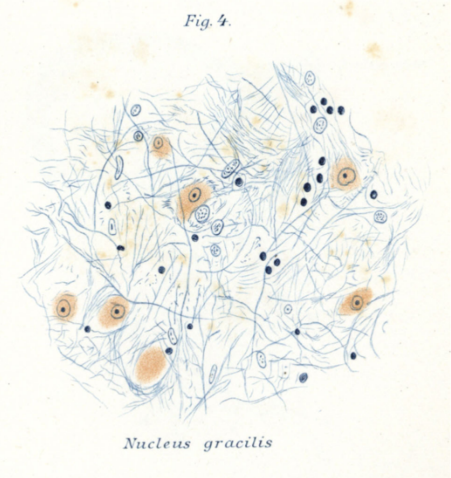


*Fig. 4 from Panel VI: Nucleus gracilis. Magnification C.*

There appear to be variations between the arrangements of the networks associated with the different dorsal nerve nuclei, but these will require further study to define.

The pyramid nucleus (Panel VI, Fig. 2), the ambiguous nucleus (Panel VII, Fig. 1) as well as other interspersed ganglion cell heaps include very irregular neuroglial networks, which are quite densely woven, although not nearly as densely as those of the olivary body. The ganglion cells of these groups usually have baskets around their bodies and along their coarser processes (Panel VII, Fig. 1). These baskets are commonly found around single solitary ganglion cells that are scattered in the medulla oblongata, as we have already mentioned on p. 74, Paragraph b. —

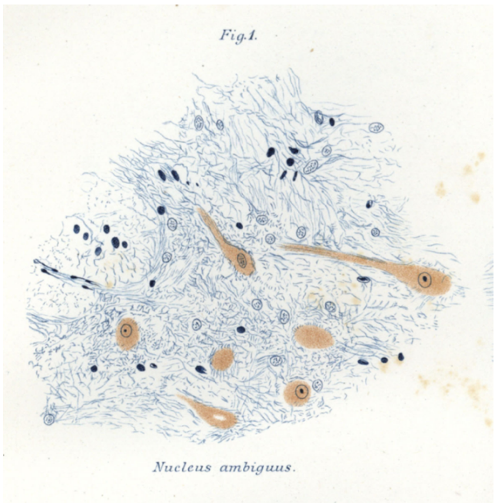


Fig. 1 from Panel VII: Ambiguous nucleus Magnification C.

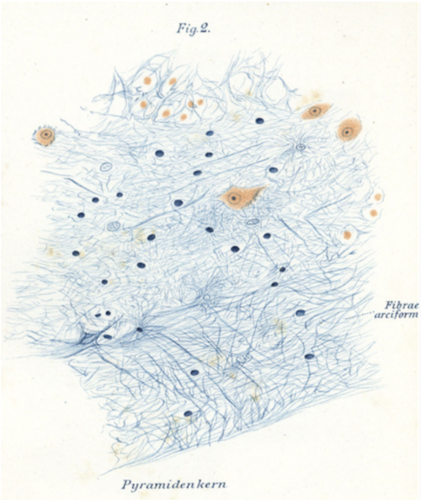


Fig. 2 from Panel VI: Fibrae arciformes externae and pyramid nucleus. Magnification C.

The olivary body however contains the densest neuroglial structure (always apart from the ependyma) in the medulla oblongata, as evidenced by the macroscopic appearance described above. The olivary body belongs to those portions of the central nervous system characterized by an exceptionally dense neuroglial mesh. The olivary body admittedly does not have such a lavish density of neuroglial networks as Petrone ascribed to it. He believed it to have the densest network in the entire central nervous system and exclaimed in awe: “Chi non lo vede, non lo crede!“ [He who does not see it, does not believe it!]. Petrone was unable to unravel the denser ependymal networks with his less selective staining methods, although it is remarkable that he did manage to observe the denseness of the neuroglial network in the olivary body, which had previously escaped everyone's attention.

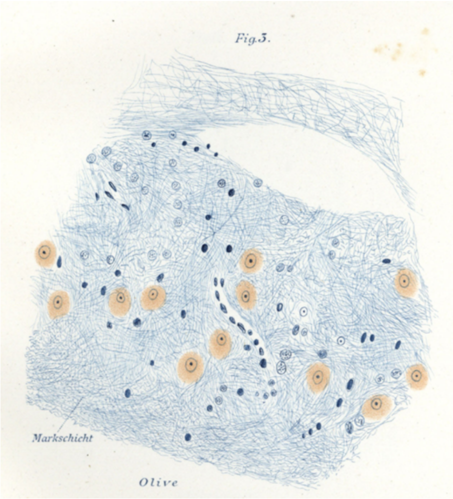


*Fig. 3 from Panel VI: Olivary body. Magnification C. Bottom, myelinated layer.*

The Golgi method has again proven so inadequate to evaluate topographic features such as the density of the neuroglial network in the olivary body — even though this characteristic is visible to the naked eye and I had described it in a communication (as had Petrone), nor was this particular feature confirmed thereafter, in reports that specifically examined the neuroglia of the medulla oblongata. The compaction of neuroglia is particularly evident in the white (myelinated) fibrous masses (Panel VI, Fig. 3), which surround and extend through the olivary body. The structures are a little looser, but still very dense, in the grey masses. The fibers crisscross in a wide variety of directions, but predominantly in the frontal plane, and form very small (0.002-0.005 mm in diameter) spaces between the strands. The fibers are generally very fine, but coarser ones are also observed traversing the field.

Even in this tangle, "astrocytes" with abundant star-shaped processes are often apparent when focusing through the section. Our images are however drawn with as little adjustment of the microscope focus as possible, consequently these astrocytes are not as evident. However, in addition to the light-colored, larger nuclei, often bearing radiating fibers, there are also many darker, smaller nuclei with no apparent surrounding grouped fibers.

**D. Ependyma.**

The abundance of neuroglial compactions is generally clearly apparent within the ependyma, but specific peculiarities should be highlighted. In some places, prominent myelinated fiber bundles, the striae acusticae, run just underneath the epithelium. There is no distinct glial ependymal layer here; rather, the epithelium sits directly on top of the myelinated nerve fibers. Instead, these myelinated nerve fibers are interspersed with a dense neuroglial mass (analogous to the Fibrae arciformes extemae), quite differently as is more commonly the case in white masses (Panel VII, Fig. 2: on the right, the fibers run longitudinally on the left, obliquely). The fibers are predominantly oriented parallel to the nerve fibers, but there are also a few perpendicular ones. It is also noteworthy that the bundles leave gaps i.e., to some extent they overhang over short distances. The epithelium extends into these gaps, giving the appearance of cyst-like spaces in the sections (Panel VII, Fig. 2 a).

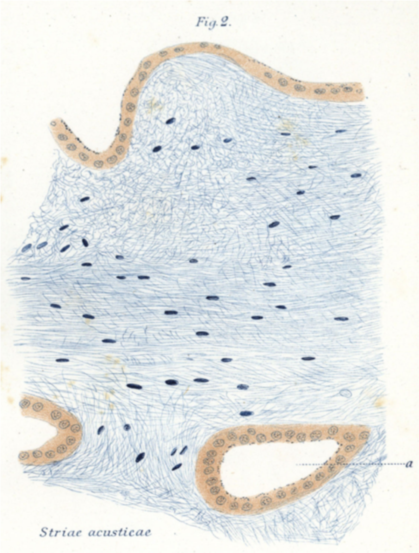

*Fig. 2 Panel VII: Striae acusticae. Magnification C. In a cyst-like space.*

Another distinctive feature is formed by the chorioid plexus. Not only do tapered extensions originating from the epyndyma extend towards the chorioid plexus and also include neuroglia over a short stretch (compare with top of p. 72), but the chorioid plexus also lies flat on the lateral parts of the fourth ventricle, so that the ventricular floor is no longer directly covered by epithelium, but by connective tissue. I have never noticed an actual ingrowth of the connective tissue into the medulla oblongata per se, as Gierke described. —

Another peculiarity here (and the ependyma more generally) involves the "ependymal proliferations" discovered by Virchow, which may probably still be considered borderline normal, as far as age-related changes on this borderline are concerned. Admittedly, these ependymal proliferations also appear quite abundantly as a direct result of pathologies. I was recently able to access one such case with apparent "vitreous granules" in the ependyma which was fresh enough to see these structures, and on which the following discussion is based.

While the medulla oblongata epithelium, as long as the central canal remains closed, behaves similarly to the spinal cord epithelium, detaching during old age, and with neuroglia ingrowths, etc., these changes also occur (Panel IV, Fig. 5), in the open ventricle. Although the epithelium is generally well preserved as associated layers in this open ventricle region (and even includes a granular seam), here and there where the cells diverge a little, a neuroglial filament is apparent between them.

In the case of "ependymal proliferations," however, the excrescences, which protrude like mounds above the level of the ventricular surface, reveal an epithelial defect on the crests of these mounds (see Panel V, Fig. 1). The epithelium only reappears on the lower parts of the slopes. Initially, the cells were a little shorter, but adopted their normal shape soon thereafter. When two such nodules are located close to each other, the epithelium-depleted upper parts merge, while the basal slope part, which is covered with epithelium, is prevented from doing the same. This forms epithelium-lined cavities that appear as closed cysts but may in fact be tunnel-shaped (Panel V, Fig. 2).

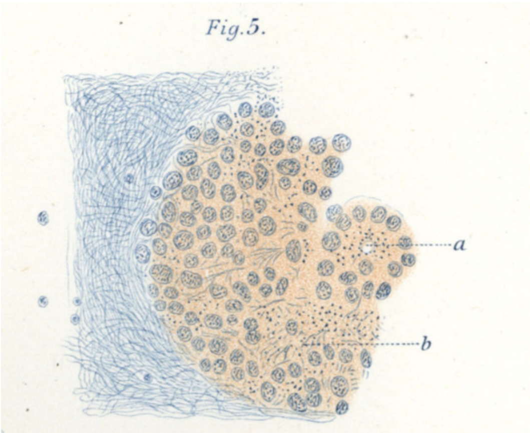


*Fig. 5 from Panel IV: Irregular epithelial heap from the lower regions of the medulla oblongata. Magnification B. (a) Newly formed lumen, (b) irregular epithelial mass interspersed with neuroglial fibers.*

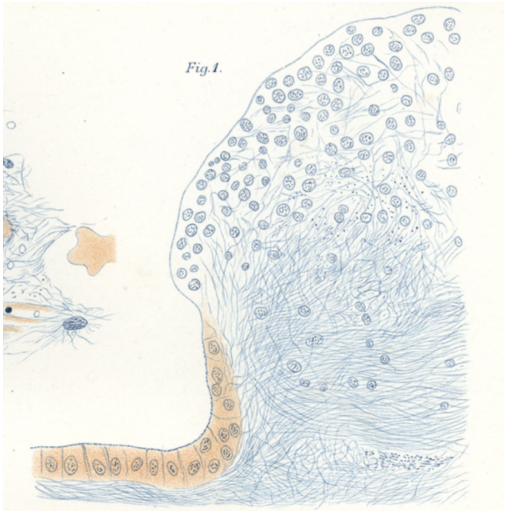


*Fig. 1 from Panel V: Ependymal proliferation in the 4th ventricle. Magnification B.*

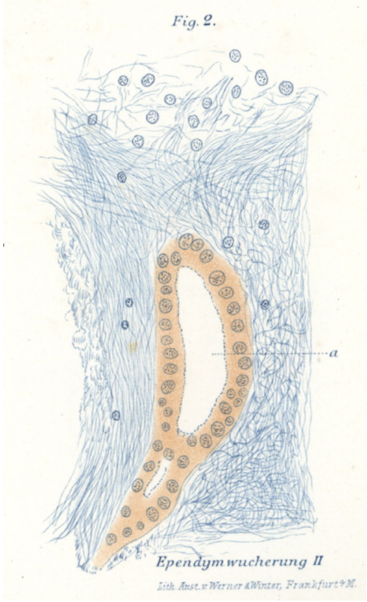


*Fig. 2 from Panel V: Cyst-like space between two adjacent ependymal proliferations. Magnification B.*

If this epithelial loss also occurred in other such cases, the evidence supporting the pathogenic nature of these proliferations would be quite convincing. It was just a matter of considering the loss of the epithelium as the primary issue. Loss of the epithelium would then be the initial step which abolishes the tissue resistance acting on the underlying neuroglial mass, and awakens the dormant idioplastic potential of neuroglial cells, i.e. which has thus far been kept within its natural limits, but has the potential to endow neuroglial cells with a vital force in the true sense of the word, thereby inducing neuroglial cell proliferation exceeding its physiologic constraints.

These types of interactions would in principle be quite similar to those described earlier for the central canal of the spinal cord. The central canal of the spinal cord also showed evidence of detachment of the epithelium which induces neuroglial proliferation. Notwithstanding this concordance in principle, there are differences between our case of ependymal proliferation and the phenomena which lead to obliteration of the central canal.

Firstly, the ependymal proliferations do not occur as frequently as the analogous processes at the central canal of the spinal cord. Secondly, there are other differences, which can be easily attributed to the different localizations.

The epithelium in the ventricles, does after all, not delimit a very long and very narrow cavity, but a wide cavity. In the narrow central canal, detached epithelial cells remain in place and are only infiltrated by the neuroglia. If cells detach from the perimeter of the ventricle, they are however not held in place by the constrictions of the space, but fall into the wide cavity and disappear in an as yet undetermined manner.

If the data gathered from our one individual case were to apply more generally, it may clarify another matter. Ependymal proliferations are often characterized by their color, which is not simply gray, but has a dewdrop-like translucent appearance. This was indeed also observed in this particular case and allows us to observe that neuroglial fibers located at the crests of the proliferations (Panel V, Fig. 1) are very sparse in contrast to their distribution in the valleys of the mounds. This "hyaline" transformation (with the word "hyaline" only used in its morphological sense and not how Recklinghausen defined it) is likely to be explained in a similar way to the hyaline transformation observed in syringomyelia of the spinal cord[1], namely, by the pressure imparted by the cerebrospinal fluid, which is not neutralized by the protective epithelium adapted to the pressure. I have similarly been able to attribute "hyaline formation" to pressure effects for other cases years ago[2].

### 3. Pons.

The cell nuclei of white matter and the ventricles are comparable to those in the medulla oblongata. Neuron nuclei that are interspersed everywhere in these regions are intertwined with an abundance of neuroglial fibers. These neuroglial fibers are predominantly of the ambiguous nucleus type (Panel VII, Fig. 1). I cannot confirm the gradual differences in the density of the neuroglial mesh between the different nerve nuclei reported by Popoff (p. 27).

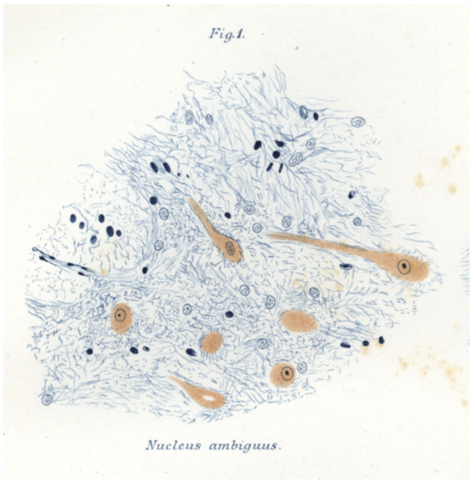


*Fig. 1 from Panel VII: Ambiguous nucleus Magnification C.*

## 4. Pedunculus cerebri.

Among the features to be highlighted in the pedunculus cerebri are the substantia nigra and the nucleus ruber. The substantia nigra (Panel VIII, Fig. 1) includes an abundant neuroglial network with similar characteristics to that found in the anterior horn but also with some unique differences.

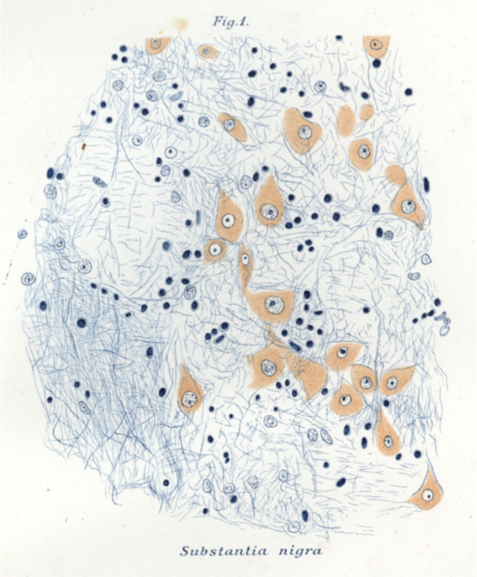


*Fig. 1 from Panel VIII: Crus cerebri. Substantia nigra. Magnification C.*

[1] Weigert, "Centralblatt für allgemeine Pathologie und pathologische Anatomie" [Central Journal of General Pathology and Pathological Anatomy], 1890, p. 737.
[2] „Deutsche medizinische Wochenschrift" [German Medical Weekly], 1885, p. 814.

Ganglion cells in the substantia nigra are often endowed with delicate baskets.

Neuroglia in the red nucleus is completely different. It is an extremely delicate network, with immensely numerous large astrocyte forms that send off long fine fibers into the tissue. The neuroglial network of the red nucleus occurs as an intermediate mass between two myelinated fibers, it delivers delicate baskets to the ganglion cells and, of course, also surround the vessels. Overall, the type of network is most similar to the white matter of the cerebrum with more numerous and more developed astrocytes forms. The interspersed ganglion cells are another difference of course.

**5. Quadruplet bodies**

The quadruplet bodies feature an abundant neuroglial mesh of almost esthetic beauty. Even to the naked eye, the blueness of the organ is more pronounced than in other similar large sections of the central nervous system, and a macroscopic inspection is already suggestive of the basic arrangement of the neuroglial network.

A frontal section considered with the naked eye (Panel XIII, Fig. 4) or with a magnifying glass, discerns a dark blue connecting strip in the midline which joins the surface with the aquaeductus sylvii. This connecting strip is approximately 2 mm wide at the top, and narrows somewhat toward the base, where it approaches the upper margin of the aquaeductus sylvii. Whereas the upper halves of the lateral margins of the aquaeductus sylvii are not characterized by a strong bluing of the adjacent parts with the naked eye, the lower half exhibits a very dark base on both sides. The upper edge of this dark base slopes somewhat obliquely outward and downward, reaching about a millimeter on either side, then ending in the formation of a relatively sharp apex. From this apex, the outer edges of the blue medial field descend with slight lateral convexity, and below the aquaeductus Sylvii there is thus a uniform generally dark blue strip, which increasingly narrows the whole substance of the quadruplet bodies into right and left halves. The reason we generally describe this strip as dark blue, is because immediately at the lower edge of the aquaeductus in the strip that is already shared here, a somewhat lighter field is apparent.

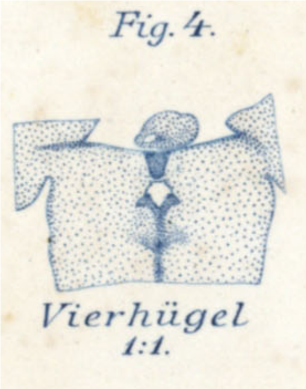


*Fig. 4 from Panel XIII: Quadruplet bodies. Actual size.*

The central parts of the two quadruplet bodies i.e., those distant from the lateral edge and the midline, appear as a very slightly lighter blue to the naked eye when compared to the rest of the area.

Microscopic examination confirms the colossal abundance of neuroglia in the dark blue areas. Under the microscope, these regions are however not as sharply defined as one might expect from their appearance with the naked eye. Rather, the immensely dense fiber network in the midline or at the edge of the aquaeductus disperses quite gradually into the larger surroundings. The upper edge of the aquaeductus, whose lateral sections are not so dark to the naked eye, also has an ependymal thickening that does not extend as far into the depths and is too narrow to be apparent macroscopically.

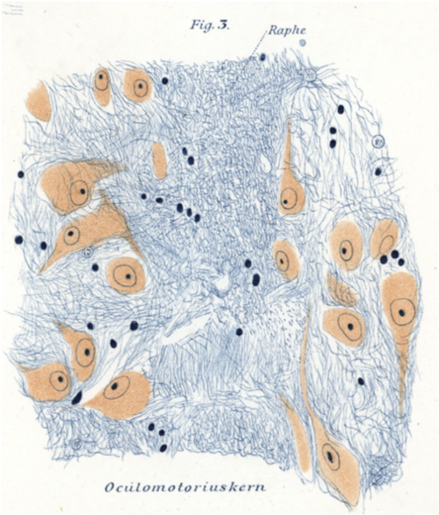


*Fig. 3 from Panel VIII: Oculomotor nucleus. Magnification C.*

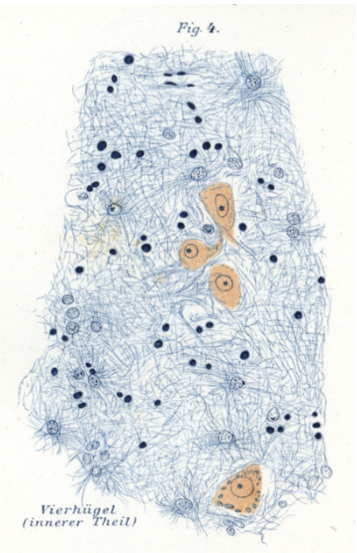


*Fig. 4 from Panel VIII: Quadruplet bodies. Section of the inner region. Magnification C.*

The remaining regions of the corpora quadrigemina include a generally rather uniform (Panel VIII, Fig. 4) densely woven neuroglial network without a predominating orientation. The spaces between the strands are irregular, either triangular, square, polyhedral, or roundish. It is only where the bundles of nerve fibers are intercalated that the spaces between the neuroglial strands take on their distinct appearance typical of white matter with nerve fibers oriented in a more parallel fashion. Single nerve fibers do not appear to be particularly disruptive. The circumferences of numerous ganglion cells show hints of basket formations. "Astrocytes" are very abundant. —

The oculomotor nucleus is very close to the midline, and its medial part is embedded in the very dense neuroglial network of the midsection, but even the lateral regions are still immensely abundant in neuroglial fibers (Panel VIII, Fig. 3). —

The upper surface of the quadruplet bodies, which is not covered with epithelium like the aquaeductus sylvii, also features a compacted cortex layer approximately 0.075 mm thick, which dissipates rather rapidly into a loose network towards the core. The previously mentioned connection of the middle of the surface with the upper margin of the aquaeductus sylvii, as well as the downward extension of the dense ependymal neuroglial accumulation, are to be interpreted as keel stripes.

### 6. Pineal gland.

The inner lower region of the pineal gland contains incredibly massive neuroglial stacks. It is so massive that it appears as a large blue spot to the naked eye (Panel XIII, Fig. 5). A small cavity is apparent above this spot.

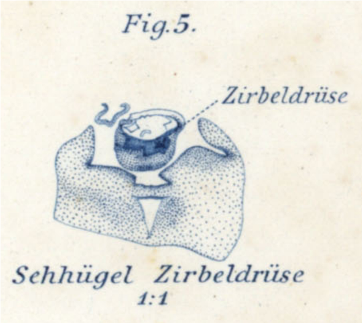


*Fig. 5 from Figure XIII: Thalamus, posterior commissure, pineal gland. Actual size.*

Microscopic examination shows this region to be composed of a dense network of strong neuroglial fibers, more distinctive than anywhere else in the central nervous system. From this dense mass, similarly, constituted thinner, dense fiber tracks are interspersed between the cell accumulations of the pineal gland (Panel XIII, Fig. 3). The cells themselves are pervaded by an abundant but loose network of vigorous neuroglial fibers (Panel XIII, Fig. 3).

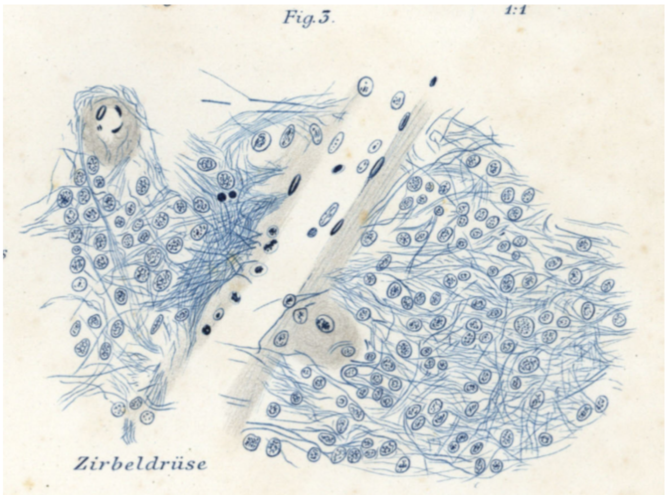


*Fig. 3 from Panel XIII: Pineal gland. Magnification C.*

### 7. Cerebellum.

The cerebellum lacks a denser cortex layer, as we already mentioned on p. 73, Paragraph 2. This makes it rather unique in the whole central nervous system, much like its other characteristic, which is perhaps associated with the first, in that it is also the only region of the central nervous system that does not have a substantial number of myelinated fibers (e.g., as tangential fibers) near its surface.

The molecular layer contains radial fibers that radiate from the surface into the depths at intervals of about 0.01 mm, but sometimes also closer, or further apart, and which eventually lose themselves in the Purkinje cell region (Panel IX, Fig. 5). Here and there these radial fibers are bent over at the surface and then rest flush against it. In regions where this is repeated many times over, a rudimentary cortex layer develops, which admittedly consists of only one fiber layer. However, this may already be a sign of age[1].

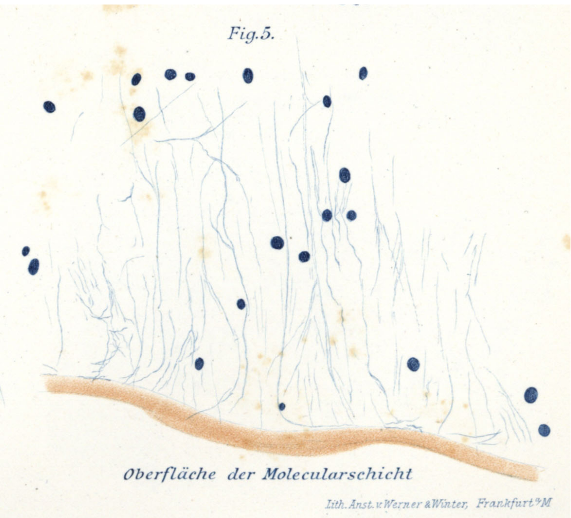


*Fig. 5 from Panel IX: Superficial region of the molecular layer. Magnification B. Bottom, pia mater.*

These are the infamous "Bergmann fibers". I am however not convinced that Bergmann actually saw these fibers. Indeed, he described them[2] as "reticulated", whereas they are in fact radial fibers that are not associated with any mesh formation (or, as we now say, network formation) In one of his later papers[3] he even expressly criticizes Kupffer,[4] "who describes the inwardly penetrating fibers as predominantly traveling straight inwards, thereby bearing more close similarity to the radial fibers of the retina."

[1] Conversely, a multilayered sheet of cells is found here in embryos, which is even more clearly apparent in newborns. Based on the generally accepted information, this was first described by Hess (De cerbelli textura. Dorpat Dissertation) in 1858, but his work is not accessible to me. In recent times, the layer has often been described as the "outer granular layer" and by Retzius as the "Vignal layer".

[2] "Zeitschrift für rationelle Medizin" [Journal of Rational Medicine], New series, Vol. 8.

[3] The same journal, Series 3, Vol. 11, p. 264.

[4] In Stephany's contributions to "Histologie der Rinde des großen Gehirns" [Histology of the Cortex of the cerebrum], Dorpat, 1860. Not accessible to me.

Based on this comment, Bergmann cannot have seen these fibers clearly at all, he may even have described something totally different to the fibers that we now attribute his name. Kupffer is more likely to have actually seen the correct fibers, but, as Bergmann indicates, he mistook them as nerve fibers. Of course, Deiters described the fibers quite correctly, independently of Bergmann and others (see p. 11), which means that he should be ultimately acknowledged for their discovery.

But the similarities between Bergmann's fibers and the Müller fibers in the retina, which are generally highlighted by almost all authors, do not seem valid to me. First of all, I would like to argue, with all due reservations (because my research on the retina is not yet complete), that Müller's fibers are chemically distinct from neuroglia, they are much thicker and both their extremities split into bundles, consequently all the similarities of Bergmann's and Muller's fibers are solely based on the radial trajectory of the fibers.

Bergmann's fibers are sparser in juvenile than in older individuals. They become very abundant in some regions in progressive paralysis and even more abundant in multiple sclerosis. We have already discussed cortex layer changes associated with pathologies on p. 73, Paragraph 2.

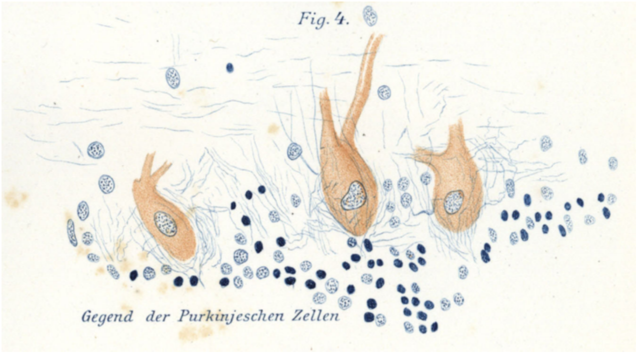


*Fig. 4 from Panel IX: Purkinje cells from an old woman. Magnification B. Bottom, start of the granular layer.*

In addition to the actual radial fibers, there are very sparse transverse fibers in the superficial regions of the molecular layer, which become more abundant towards the base, but are always very scattered, particularly in the vicinity of the Purkinje cells (Panel IX, Fig. 4). Sparse fibers are evident around the Purkinje cells themselves in juvenile individuals, and more abundant basket-like accumulations of fibers in older individuals (Panel IX, Fig. 4 taken from an old woman). These fibers proliferate dramatically in progressive paralysis and multiple sclerosis.

I am very skeptical as to whether all that has been designated as neuroglia in the cerebellum based on Golgi preparations is actually truly neuroglia, because the Golgi "cell silhouettes" are not easy to reconcile with my own images. —

I have virtually not found any neuroglial fibers in the granular layer in normal individuals, but have found them to be plentiful in progressive paralysis, etc.

Even around the vessels, only very rarely were neuroglial fibers apparent, in contrast to Golgi's data (refer to p. 17). —

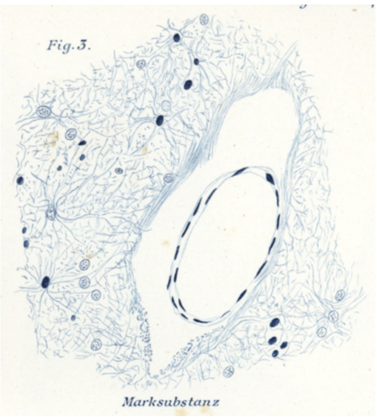

*Fig. 3 from Panel IX: White matter of the cerebellum. Magnification C. In the middle, a vessel. At the bottom edge a vessel with transversely sectioned fibers.*

On the other hand, the medullary component, as all authors state, shows a very beautiful neuroglial network, which is identical to that in white matter: with fibers predominantly, but not exclusively, arranged parallel to the nerve fibers, with abundantly embedded beautiful "astrocytes". (Panel IX, Fig. 3).
Where the cerebellum forms the roof of the fourth ventricle, it is of course covered with ependyma. In accordance with general topographic principles, the influence of the ependyma is also evident in the adjacent myelinated mass of the cerebellum. Here it is interspersed with a much denser neuroglial network as compared to regions that are further away from the ependyma.

**8. Cerebrum**

The cortex layer of the cerebrum has been quite incorrectly likened to that of the cerebellum by several authors. The cerebrum rather has a typical true cortex layer which is located just below the pia mater and consists of tightly interwoven fibers (Panel IX, Fig. 1 a). This type of structure is lacking on the cerebellum. The thickness of this cortex layer is highly variable and varies from approximately 0.003 to 0.03, depending on the specific cerebrum region and on the age of the individual. The cerebrum cortex becomes increasingly thick and its fibers coarser with age, as Golgi had already reported. The direction of the fibers in this actual cortex layer is very variable, but they are generally oriented obliquely tangential.

The Golgi method is inadequate for investigating this particular layer as well as the next layer we will mention. It only yields very incomplete images, as shown in the illustrations of numerous publications which used the method. The Golgi method yields particularly poor results in older individuals, as previously noted by Retzius[1]. From the denser, actual cortex layer, predominantly loose (but again, by no means exclusively) obliquely radial fiber masses radiate downwards. At first, these fibers are still quite numerous and discreet (Panel IX, Fig. 1 b), they then gradually become more and more sparse and are finally lost completely.

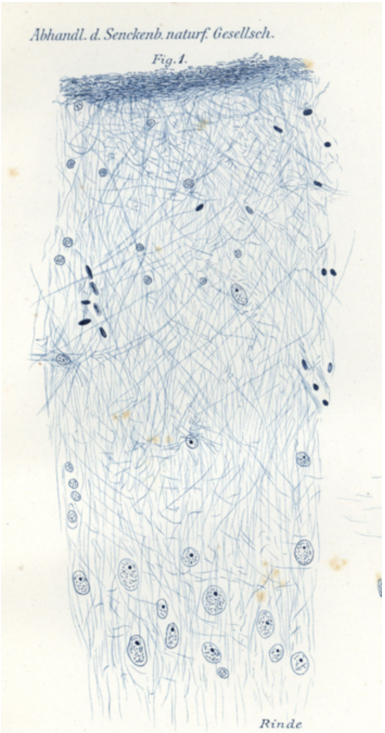


*Fig. 1 from Panel IX: Cerebrum cortex. Temporal lobe. Magnification C. (a) Cortex layer. (b) Radial fiber layer.*

[1]Die Neuroglia des Gehirns beim Menschen und bei Säugetieren" [The neuroglia of the brain in man and mammals], Jena, 1894, p. 11.

This second layer also reaches more or less far down in various places, deeper in the case of older individuals. The fibers from this second layer can often be traced right down to the lower boundary of the small pyramid cells. Lloyd Andriezen[1] reported that they "reached into the middle of the pyramid cells". Vessels located in the zone containing these radial neuroglial fibers either have thinner or thicker glial sheaths depending on the size of the vessel.

I have only observed neuroglia as quite scattered fibrils, which completely disappear over long distances, in the deeper layers of the cerebral cortex and the radial myelinated fibers, which means that I absolutely cannot confirm the existence of an associated connective tissue component, which Golgi still reported in 1885.

The medullary component, on the other hand, again contains a rich neuroglial network of the type observed in white matter in general, which is very similar to the network in the cerebellum. However, the fibers in the cerebrum are somewhat finer and the network tighter (compare Panel IX, Fig. 2).

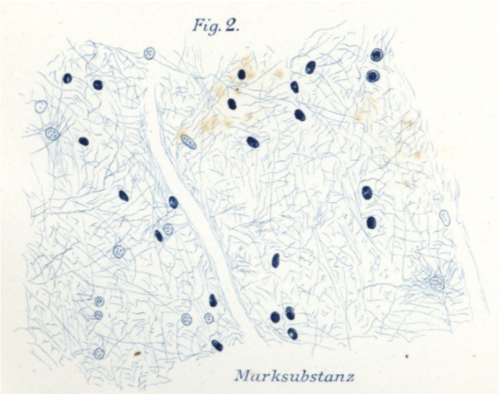


*Fig. 2 from Panel IX: White matter of the cerebrum. Magnification C.*

It needs to be specifically reiterated that the protoplasmic cells which Golgi, Ranvier, Lloyd Andriezen, and Retzius referred to as neuroglial cells are not visualized with the new method. If it were really true, as Lloyd Andriezen argued, that these "protoplasmatic elements" were of mesoplastic origin, as opposed to the epiplastic "fiber-elements", this would reflect such a fundamental discrepancy with the actual astrocytes that this reason alone would be enough to differentiate the "protoplasmatic elements" from the actual neuroglia. Our evidence neither supports nor rejects the justification for this assumption. On the other hand, Lloyd Andriezen is certainly mistaken when he reports (British medical Journal 1893. July 29) that: "the protoplasmic glia elements are really the elements, which exhibit a morbid hypertrophy in pathological conditions (alcoholism, G. P.) and which may show further morbid activities, in the last stage of which their protoplasm will deposit numerous organised fibrillae, in the act of doing which the protoplasm proper is used up except a scanty remnant, which may persist, ghost-like, to mark the position of what was once a protoplasmic cell body."

[1] "Internationale Monatsschrift für Anatomie" [International Monthly Journal of Anatomy], 1893, p. 537.

It is precisely in pathological cases, particularly in general paralysis (G.P.), that enormous numbers of typical "astrocytes" i.e., not protoplasmic cells, start to appear, with real, very thick neuroglial fibers, it is therefore simply not true that the protoplasm is consumed with only a "ghost of the cell body" being left behind. The cell bodies are unusually large in progressive paralysis, one can even see thick protoplasmic extensions against which the fibers lean on for some stretches (admittedly the fibers are sharply separated from the protoplasmic extensions).

## 9. Gyrus hippocampi. Cornu ammonis

The hippocampus is such a complex organ that it is not surprising that its neuroglial structures are very intricate. The development of the hippocampus is characterized by all kinds of invaginations and folding processes, and also by keel stripe formations which are apparent in several regions.

To make our investigation of this brain structure easier to understand, we have included a drawing of a tissue section observed under a magnifying glass in Panel XIII, Fig. 2 (1: 3½ magnification). This image, however, only represents neuroglial characteristics which can actually be seen at this magnification. Some details are shown in Panel X, Figs. 1 to 3 and Panel XIII, Fig. 1 at a high magnification. The following description follows the direction of the arrows in Fig. 2, Panel XIII.

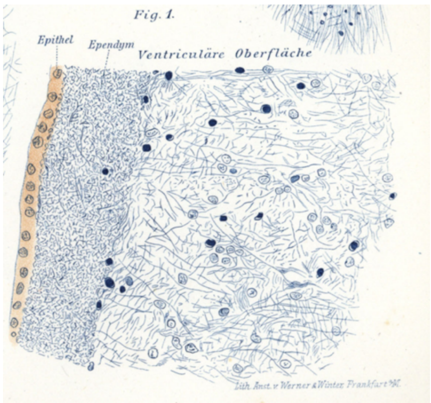


*Fig. 1 from Panel X: Ependymal surface of the hippocampus. Magnification C. Left ependymal layer, right radial fiber layer.*

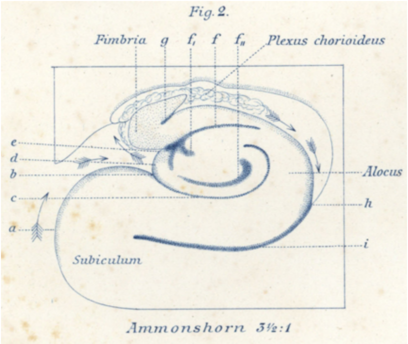


*Fig. 2 from Panel X: Gyrus dentatus. Magnification C. Bottom pial surface. This figure is composed of two drawings, the top and bottom parts of the same image. The drawings fit together perfectly. Their boundary is marked in the figure by the constriction of the center.*

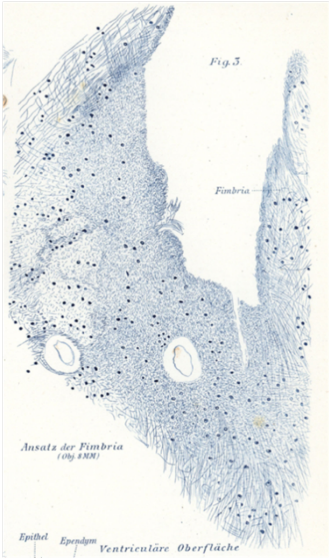


*Fig. 3 from Panel X: Base of the fimbria. Magnification D.*

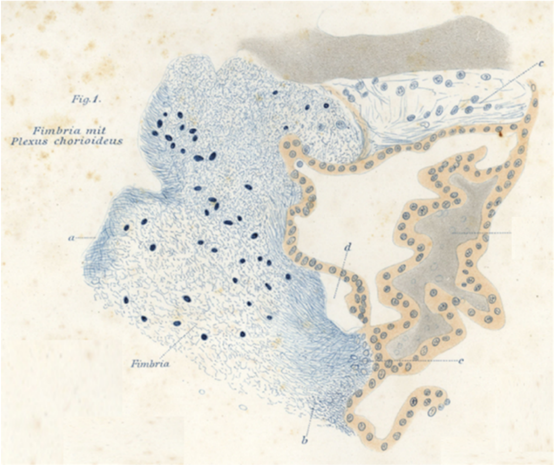


*Fig. 1 from Panel XIII: Fimbria with plexus chorioideus Magnification C. (a) Pial surface, (b) Ependyma. (c) Plexus chorioideus (d) Epithelial papillae.*

### A. Gyrus hippocampi.

We start with the turning point of the gyrus hippocampi to the hippocampus (Panel XIII, Fig. 2 a). It is well established that myelinated fibers lie freely on the surface of the gyrus hippocampi. These correspond to the usual transversal fibers but are more powerful than these and are not embedded in grey matter. The white layer is not continuous, but consists of reticulated strands, with the surface of the cortex appearing grey between the strands. (Substantia reticularis alba Arnoldi.)

Because the myelinated tracts are freely exposed on the surface here, the characteristics of neuroglia in this region deviate somewhat from the rest of the cerebral cortex.

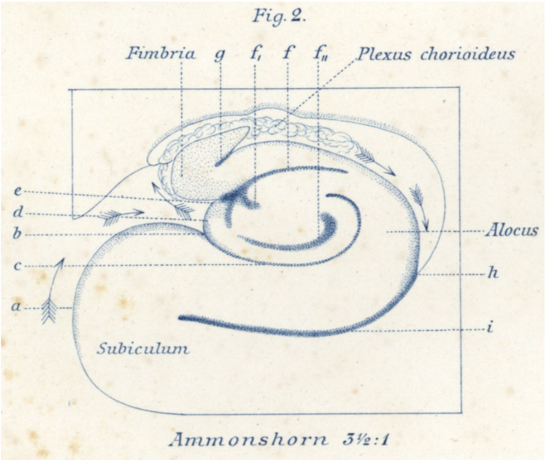


*Fig. 2 from Panel XIII: Hippocampus. 3 ½ x magnification. Explanation in text.*

Although the gyrus hippocampi is also covered by a cortex layer of densely interwoven fibers, approximately 0.02 mm thick, this is not directly followed by the (predominantly) radial neuroglial fibers radiating into the cortex. Instead, there is first a layer, approximately 0.2 mm wide, which consists of a rather dense network of neuroglial fibers oriented in all different directions. "Astrocytes" are observed within this fiber network. The strands of fibers are far enough apart to accommodate a single myelinated fiber. It is only from this particular layer, with reasonably sharply defined top and bottom margins, that predominantly radially oriented neuroglial fibers that are typically found in the cerebral cortex project into the depths. The spaces between the strands of the network gradually decrease in size (about 0.4 mm between individual fiber strands) and project into the underlying cerebral cortex, whose deepest layers again exhibit an extraordinary sparseness of neuroglia which is a defining feature of this particular region. Apart from its deviation, in terms of the superficially lying powerful tangential fiber zone prompting the intercalation of a distinct neuroglial layer, the distribution of neuroglia in the cortex of the gyrus hippocampi is identical to that found in other parts of the cortex. The regions of this gyrus without exposed tangential fibers, and which therefore already appear gray to the naked eye, indeed contain neuroglia that share the same characteristics as neuroglia in other parts of the cortex.

When sectioning through the gyrus hippocampi, the two types of neuroglial distribution alternate several times. Near the fissura hippocampi (Panel XIII, Fig. 2 b), where the hippocampus proper begins, however, the superficial white layer always appears to be present, along with its corresponding neuroglial configuration.

**B. Fissura hippocampi.**

From the fissura hippocampi, a continuation or fusion of the surfaces of the gyrus hippocampi and the hippocampus extends far into the depths. It is therefore not surprising to find a long 0.15-0.25 mm wide keel stripe of neuroglia in this region. This keel stripe is already visible with the naked eye or with a magnifying glass (Panel XIII, Fig. 2 c). It consists of a dense network of fibers that are oriented predominantly parallel to the myelinated nerve fibers here, but in such a way that secondary fibers can still be observed oriented in the other two directions. The strands of the fiber network are spaced 0.002 to 0.006 mm apart. Since we are dealing with the fused tangential fibers from the gyrus hippocampi and the surface of the hippocampus, we also find the expected cortex type of neuroglial fibers radiating from the neuroglia network of tangential fibers to both sides, predominantly oblique or perpendicular to the orientation of the nerves (radial fibers), and which are gradually lost in the depths of the adjacent cortex layer.

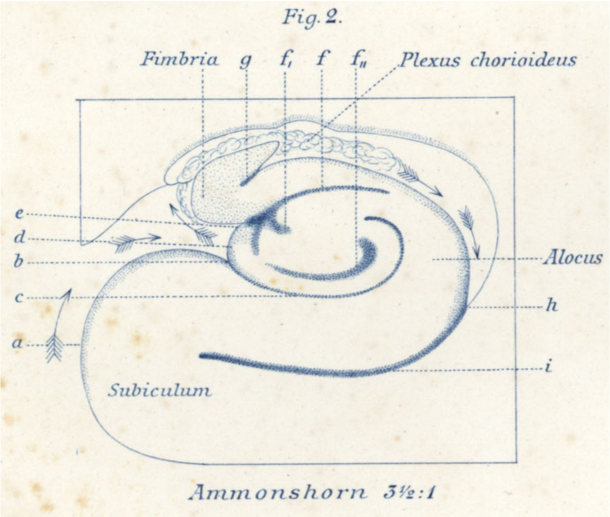


*Fig. 2 from Panel XIII: Hippocampus. 3 ½ x magnification. Explanation in text.*

The tangential neuroglial fibers of the fissura hippocampi, as well as the continuations of these fibers as far as they can be followed into the depths, exhibit the same dense structures as the fibers at the surface of the gyrus hippocampi, i.e. as opposed to the tangential fibers of the cerebral cortex. —

The roundish protrusion of the gyrus dentatus that follows (Panel XIII, Fig. 2d) has no ependyma at this point. It is covered by an ordinary superficial cortex layer. —

### C. Hippocampus and fimbria.

We encounter another inflection, which corresponds to the lower edge of the fimbria that attaches here (Panel XIII, Fig. 2e). The cortex layer is immensely well-developed at this bend (Panel X, Fig. 3) with several features, which are visible with the naked eye or the magnifying glass, emanating from it (Panel XIII, Fig. 2 f, $f_1$ and $f_{11}$). These neuroglia tracks are between 0.2-0.5 mm wide, depending on how they are sectioned, and consist of a very delicate, dense and relatively uniform network of fiber strands forming polyhedral shapes. This network is one of the densest and yet most delicate neuroglial fiber networks found in white matter of the central nervous system. In fact, it consists of white matter, to which specific hippocampus cells attach laterally (Panel X, Fig. 2, at high magnification).

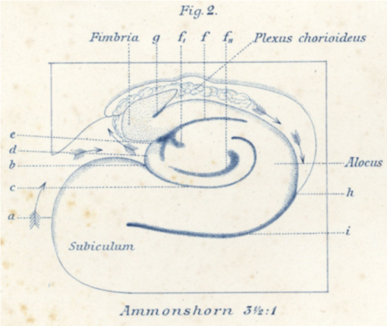


*Fig. 2 from Panel XIII: Hippocampus. 3 ½ x magnification. Explanation in text.*

I

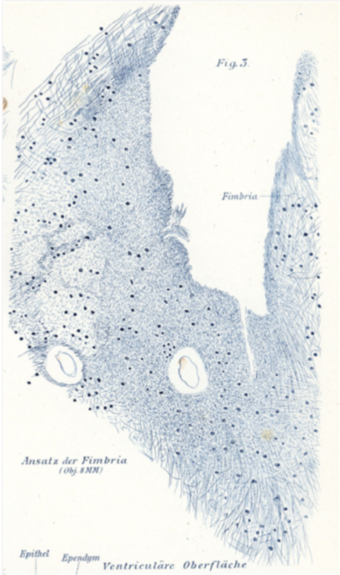


*Fig. 3 from Panel X: Base of the fimbria. Magnification D.*

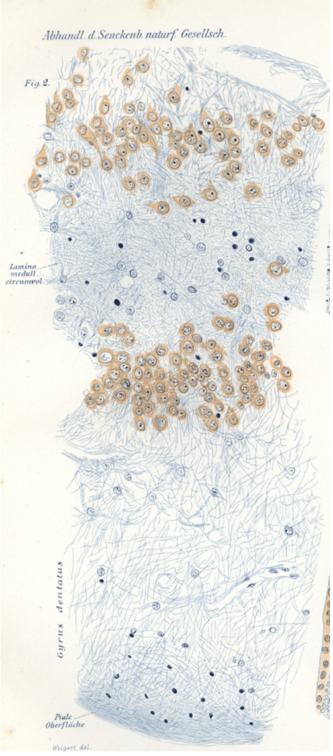


*Fig. 2 from Panel X. Gyrus dentatus. Magnification C. Bottom pial surface. This figure is composed of two drawings, the top and bottom parts of the same image. The drawings fit together perfectly. Their boundary is marked in the figure by the constriction of the center.*

One of these neuroglial tracks is oriented towards the upper surface, and runs parallel to it (Panel XIII, Fig. 2f), a second track which is intermittently oriented parallel to the lateral and ventral boundary between the hippocampus and the gyrus hippocampi is also apparent (Panel XII, Fig. 2 $f_{11}$), and there is a third track between these first two tracks (fi).

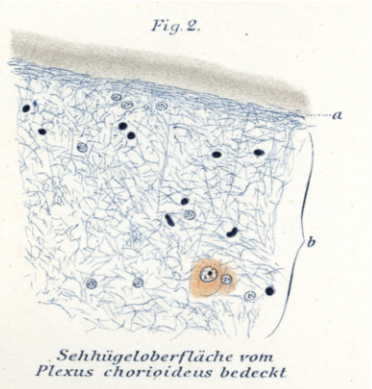


*Fig. 2 from Panel XII: Thalamus surface covered by plexus chorioideus. Magnification C. (a) Cortex layer. (b) Radial fiber layer.*

The characteristic hippocampus cells are arranged on one side or both sides of these fiber tracks. A loose neuroglial fiber network, whose individual fibers run predominantly perpendicular to the myelinated fibers but are also frequently interwoven with fibers running in other directions particularly in the vicinity of the myelinated fibers, extends into these fiber tracks (Panel X, Fig. 2, at high magnification). Among the hippocampus cells, the fibers located near the exposed surface or the deep tangential fibers, which extend through the hippocampus cell layer, mix with other fibers that extend radially into the depths from the exposed surface or the deep tangential fibers. The radial fibers do not reach specific hippocampus cells that are further removed from this layer (as represented in $f_1$), and the neuroglial fibers are lost in the deep cortex layers, which are also immensely depleted of fibers.

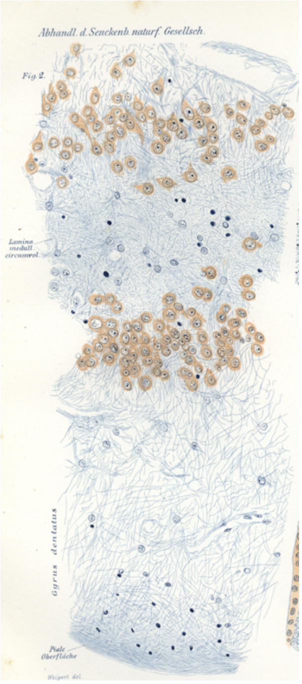


*Fig. 2 from Panel X. Gyrus dentatus. Magnification C. Bottom pial surface. This figure is composed of two drawings, the top and bottom parts of the same image. The drawings fit together perfectly. Their boundary is marked in the figure by the constriction of the center.*

We now follow the surface of the hippocampus back to where we started from, to reach the fimbria. The fimbria contains an abundance of neuroglial fibers, intertwined with the nerve fibers (Panel X, Fig. 3, at lower magnification; Panel XIII, Fig. 1, at high magnification). The lateral surface at the start of the fimbria is composed of a thinner superficial cortex layer (Panel XIII, Fig. 1 a), its medial side reveals a thicker ependymal layer (Panel XIII, Fig. 1 b). Attached to the fimbria is the choroid plexus, into which neuroglia only extend for a short distance (Panel XIII, Fig. l c).

The ependymal epithelium of the fimbria and of the hippocampus forms cups in some places (Panel XIII, Fig. 1 d), and slender papillae in others, — these may be interpreted as the first hints of plexus formation.

At the point where the fimbria curves towards the choroid plexus, there is an indentation, from which a keel strip (Panel XIII, Fig. 2 g) extends into the interior.

The hippocampus (ventricular) surface that follows is covered with epithelium, and encompasses a thick ependymal neuroglial accumulation, which is followed by loose, more radial fibers (Panel X, Fig. 1, at high magnification).

If we continue along the ventricular side of the hippocampus, we eventually reach the junction of the alveus and the dorsal ventricular wall (Panel XIII, Fig. 2 h). A keel stripe of neuroglia also extends out from this point (Panel XIII, Fig. 2 i). It originates from the fusion of two surfaces, which is supported by the fact that epithelium is still apparent near the ventricle, first as contiguous layers, then as intermittent tracks, until it finally disappears completely.

This keel stripe is also visible to the naked eye. It consists of a dense neuroglia network which progressively thins out at increasing depths before finally completely disappearing. Neuroglial fibers radiate out either side of the keel stripe, predominantly in oblique directions with the angles between the fibers becoming less acute toward the ventricular side.

The keel stripe is surrounded by white matter with its corresponding network of neuroglial fibers which is configured like the prototypic network associated with the medullary component of the cerebrum.

## 10. Corpus callosum and fornix.

The corpus callosum consists of two different surfaces as far as the configuration of neuroglia is concerned. An upper surface devoid of epithelium and a lower surface which is partially fused to the fornix and partially covered by epithelium. This epithelium forms part of the lining of the lateral ventricles.

The upper surface reveals a 0.01-0.03 mm thick compacted cortex layer (Panel XI, Fig. 2 a). Depending on the depth (Panel XI, Fig. 2 b), longitudinal nerve bundles that are contiguous with the surface of this cortex layer are associated with a neuroglial network, which, although it does not have the same density as the cortex layer, nevertheless features a closer interwoven fiber structure than the deeper transversely directed nerve fiber layer. The main fibers are predominantly perpendicular to the surface, with other fibers connecting these main fibers either oriented parallel or obliquely relative to the surface. This denser layer, which is located below the even denser cortex layer, is about 1/3 mm thick and fades into the depths of the neuroglial masses of the transverse myelinated fiber layer. This denser neuroglial layer is not dissimilar to the fiber configuration found in white matter in that it also consists of fibers predominantly oriented parallel to the nerve fibrils, with the usual secondary fibers oriented in other directions (Panel XI, Fig. 3. Please note that this drawing has been rotated by 90 degrees to save space. The right edge of the panel needs to be at the bottom of the panel).

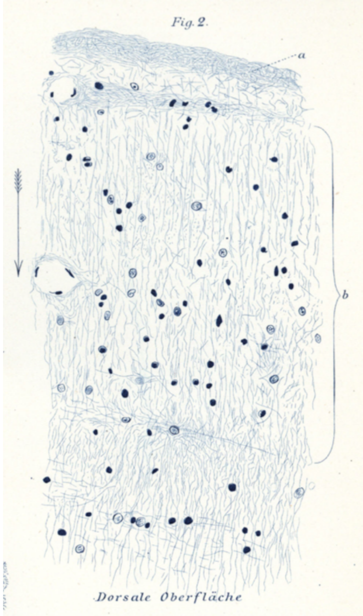


*Fig. 2 from Panel XI. Dorsal surface of the column. Magnification C. (a) Cortex layer. (b) Radial fiber layer.*

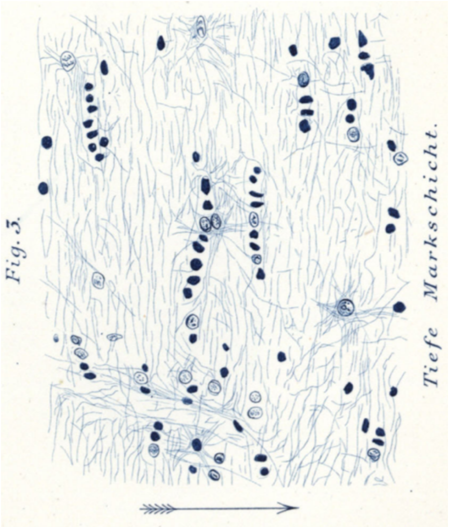


*Fig. 3 from Panel XI: Deep myelinated layer of the column. Magnification C. The figure needs to be rotated by 90 degrees to correspond to the orientation of the other figures on this panel.*

However, the denser neuroglial layer below the cortex layer, which is predominantly made up of radial fibers, is totally absent in those places where ganglion cell masses are present at the surface of the column or is only apparent as a residual hint underneath the ganglion cell masses. As far as I know, the ganglion cell masses were first discovered by Jastrowitz[1], Golgi[2] described them again much later, albeit without being aware of Jastrowitz's data. These ganglion cell accumulations appear as dome-shaped projections on transverse sections. The neuroglial stain allows these regions to stand out from the background even at lower magnification.

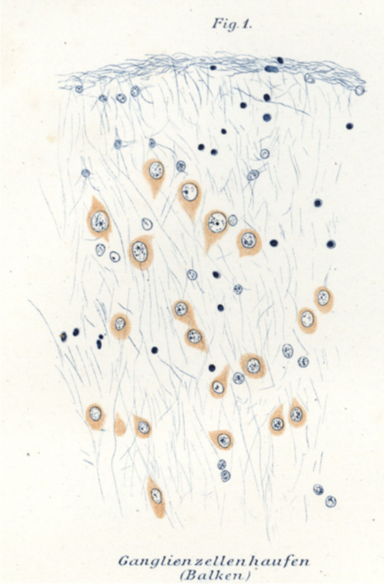

*Fig. 1 from Panel XII: Ganglion cell heap on dorsal surface of the column. Magnification C.*

At higher magnifications (Panel XII, Fig. 1), there is a region with very few neuroglial fibers, which corresponds to the ganglion cell accumulations, situated directly below the compacted cortex layer which is quite thin here.

[1] "Studien über Encephalitis und Myelitis im ersten Kindesalter" [On encephalitis and myelitis in early infancy], Final paper, "Archiv für Psychiatrie" [Archives of Psychiatry], Vol. III, 1872, p. 167 f.

[2] "Über die feinere Anatomie der Zentralorgane des Nervensystems" [About the finer anatomy of the central organs of the nervous system], 1885, Collated papers, p. 135 ff. and Panel 268.

Fibers between the ganglion cells form a loose network which predominantly consists of fibers oriented perpendicular to the surface. They extend from the cortex layer to the underlying myelinated fiber layer and are associated with both types of neuroglial networks.

"Astrocytes" are found throughout the column. The depths of the myelinated mass throughout the column, however, also contains neuroglial cells that appear square, as previously described by Jastrowitz, and that are often arranged in small longitudinal rows[7#]. Fibers are often observed to radiate out from these longitudinal rows of fibers, more specifically from the side edges of the whole cell rows. —

The lower part of the column, which is covered with epithelium or fused with the fornix, exhibits different characteristics compared to the upper surface (Panel XII, Fig. 1). There is a 0.1 to 0.2 mm thick layer beneath the epithelium which consists of very tightly interwoven neuroglia fibers, that gradually and without any apparent sharp margin, fade upward (i.e., toward the deep nerve fiber layer) into a loose network which is 0.3 mm thick and which in turn fades away into the even looser neuroglia stacks of the deeper myelinated mass. The fibers from the zone that is located directly above the dense ependymal layer are again predominantly perpendicular to the surface and thereby differ from the fibers of the deeper white masses, which are mainly oriented parallel to the surface. The transverse secondary fibers that run between the fibers of the second layer contrast with the layer of fibers that run perpendicular to the surface to create a very delicate network. At times, the epithelium is absent here as well. Neuroglia appears to proliferate as flat bulges over the free surface (ependymal proliferation) of these regions, but these growths are not as extensive as those observed in the fourth ventricle, not even in hyaline degeneration tissue. —

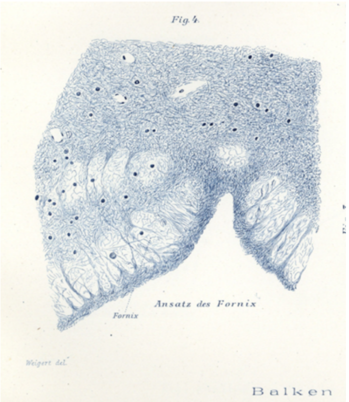

*Fig. 4 from Panel XI: Attachment of the fornix to the column. Magnification C. Bottom, fornix.*

The dense ependymal layer increases in thickness until it reaches a thickness of approximately 1/2 mm where the fornix connects to the corpus callosum (Panel XI, Fig. 4). Past this junction boundary, that is, between the fornix and the corpus callosum, it then continues on as a thick but short keel strip, which leaves an immensely large bulk of neuroglia at this junction. —

The fornix itself has an epithelial coating on its lateral side (bordering the lateral ventricle) with its corresponding compact ependymal neuroglia. Its fibers are separated from each other by quite an abundance of neuroglia for

white matter, and in many cases are arranged into smaller bundles separated by peripheral layers (Panel XI, Fig. 4).

This is also apparent on the medial surface close to the keel stripe formation of the midline. —

The medial surface of the fornix (ventriculus septi pellucidi) has no epithelium and only a thin compacted cortex layer. Once again, "astrocytes" are everywhere here.

## 11. Opticus and Chiasm.

Virchow was already aware that the opticus contains neuroglia. Leber had also identified Deiters cells here. I had already reported on the essential neuroglial features of the opticus that could be appreciated with the new method, back in 1890. Various communications using the Golgi method have been published since that time, but they failed to add anything new to our current understanding. The description and illustration by Ramón y Cajal[1] must nevertheless be given commensurate consideration because it represents the technical pinnacle of what could be achieved with the Golgi method. I had already stated in my preliminary 1890 communication that the opticus features a rich neuroglia network, which is much thicker on the surface of the whole nerve than on the surface of the individual bundles, in other words: in terms of its neuroglia, the opticus behaves completely like white brain matter divided into smaller bundles and assembled into a whole bundle. There is no need to say any more (refer to Panel VII, Figs. 3 and 4.)

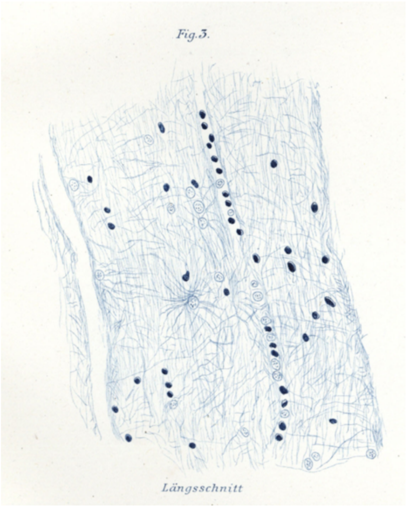


*Fig. 3 from Panel VII: Opticus, longitudinal section. Magnification C.*

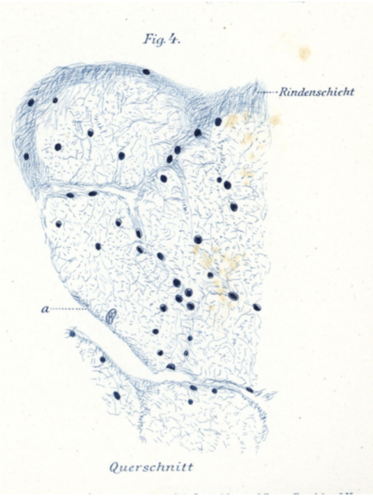


*Fig. 4 from Panel VII: Opticus, transverse section. Magnification C. Top, the compacted outer cortex layer. (a) Peripheral layer of a bundle.*

The errors, which the Golgi method may induce in terms of topography, can be inferred from Greeff's comment[2]. Greeff indeed considered the fact that neuroglia would be at their densest just below the surface of the optic nerves to be an illusion. The illusion could only have been brought about if specimens were not incubated long enough in the Golgi mixture, because this would only allow the reagent to penetrate into the outer layers of the tissue. If tissue samples are incubated in stain for longer, only a few poorly stained cells are found at the edges, while a dense tangle of cells and fibers is found in the center.

[1] "Notas preventivas sobre la retina y gran simpático de los mamiferos", Barcelona, 1891.

[2] "Die Spinnenzellen — Neurogliazellen — in Sehnerv und der Retina" [Spider cells — neuroglia cells — in the optic nerve and the retina.]. Archiv für Augenheilkunde [Archives of Ophthalmology], Vol. 29, p. 11 of the separate publication.

Greeff himself admits that it is difficult to get an accurate idea of neuroglia density with the Golgi method (p. 10) — but this did not prevent him from erroneously arguing that the compact cortex layer at the opticus was an illusion. —

The configuration of neuroglia in the chiasm is very much analogous to that in the opticus, except that the interweaving of bundles in the opticus produces a more complex neuroglial fiber picture, because each bundle must have a different main direction from adjacent bundles that intersect it.

The lateral and anterior cortex layer of the chiasm is approximately 0.04 mm thick, but the back of the chiasm is covered by a thicker layer consisting of very densely woven fibers, which is about 1/4 mm thick and thereby also visible to the naked eye as a dark blue strip. The considerable thickness and density of the layer is somewhat remarkable because there is no epithelium-covered ependyma in this case, instead the surface is merely covered by connective tissue.

This connective tissue isolates the chiasm until the middle portion of the funnel. The funnel features a cortex layer on the side facing the chiasm, that is as thick as the connective tissue layer, and which fades into the depths as scattered fibers. Both parts of the brain merge in the median line as the connective tissue falls away but remain separated from each other by a thick dense neuroglia layer, a keel stripe, which fills all the free spaces. The lateral regions of this dense neuroglial mass are interspersed with large ganglion cells.

## 12. Corpora mammillaria.

The lateral side of the external surface of the corpora mammillaria exhibits of a very thick, densely woven cortex layer (approx. 0.1 mm thick). This narrows down to 0.02 mm as it bends into the medial surface of the body, which is the approximate thickness it coats this body. The dense masses disperse into looser networks toward the depths, but they still contain very abundant fibers. Discrete fibers project out from these main fibers into the ganglion cells and ganglion cell groups located in the substance of the corpora mammillaria in the most diverse directions.

The ependymal surface again exhibits a subepithelial neuroglial compaction approximately 0.1 mm in diameter, which at its greatest thickness stands out very starkly against the much thinner narrow tissue bridge which separates the outer cortex layer from the medial surface of the two bodies. The ependymal compactions are again followed by a somewhat thicker layer of loosely woven, but still quite abundant fibers, which gradually fade into the depths. However, in the larger ganglion heaps of the substantia grisea centralis that lie here, the fibers are again more abundant, and run in different directions.

The end section of the fornix also reveals neuroglial features of white matter.

## 13. Thalamus.

I found it very difficult to study neuroglia in the large central ganglia, because the solutions used to fix and perform the metallic impregnation of the tissue have great trouble penetrating this structure. The neuroglia configuration descriptions that follow in this chapter and the next will therefore require much more additional research to elucidate.

The surface of the thalamus is characterized by three distinct features:

1. The choroid plexus region. The choroid plexus seems to rest loosely on the surface of the thalamus. In very fresh brain tissue, one can however observe that the connection is not quite so loose, but rather that vessels from the lower surface of the plexus penetrate into the surface of the thalamus. The thalamus is not covered with epithelium at this point, or rather there is real connective tissue, i.e. the plexus, inserted between the epithelium and the nerve tissue, whose free surface is covered by epithelium. The configuration is therefore similar to that found in the lateral parts of the ventricular floor at the medulla oblongata.
2. The second type of thalamus surface is characterized by the presence of superficial myelinated nerve fibers.
3. The third type, beyond the Sulcus Monroi, is characterized by exposed grey masses.

The differences in ependymal neuroglial masses are conditioned by these three differently constituted surface regions. The first region, the choroid plexus, encompasses a strikingly thin (0.01-0.02 mm thick) compact surface layer. The interposition of the connective tissue plexus is perhaps the reason why this tightly woven neuroglial layer is so thin. Looser, but still fibrous, partially radially oriented neuroglial masses are projected into the depths from this compacted layer (Panel XII, Fig. 2a).

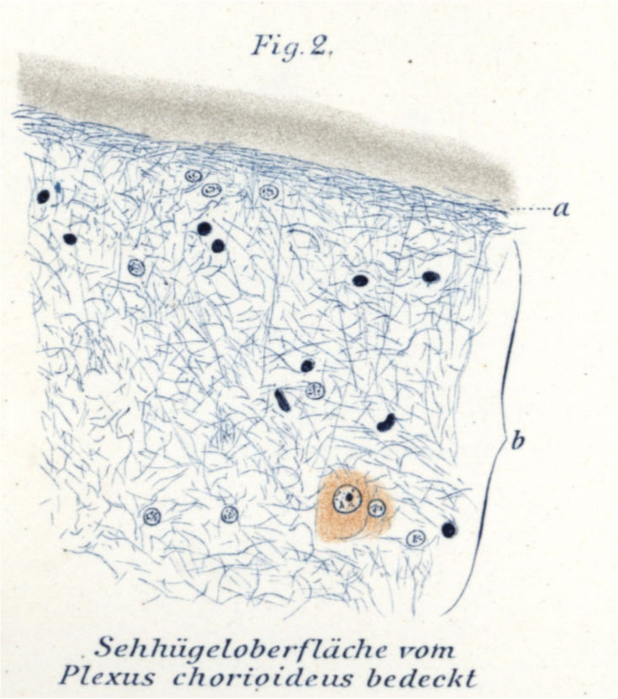


*Fig. 2 from Panel XII: Thalamus surface covered by plexus chorioideus. Magnification C. (a) Cortex layer. (b) Radial fiber layer.*

But the ependymal layer in other thalamus surface regions also exhibits immensely variable thicknesses which range from 0.025 to 0.17 mm in diameter. I cannot offer any explanations for these variations. In places within the superficial layer that contain somewhat larger vessels, the mass of the ependymal compaction greatly increases.

Where the white fiber tracks lie directly on top of the surface, they may either be covered by a discontinuous ependymal layer, or it is as is the case in the striae acusticae of the medulla oblongata i.e., the epithelium may lie directly on top of the nerve bundles. In the latter case, the nerve fibrils are then interspersed with very dense neuroglial masses and long conspicuous radial fibers, while the remaining fibers run in the other two directions. The densely packed layer, however, lacks the interstices for the myelinated nerve fibers. In cases where a distinct neuroglial compaction is sandwiched between the epithelium and the nerve bundle, the nerve bundles still reveal abundant fibers, but the networks are not as dense as when the ventricle boundary is only defined by epithelium.

The ependymal layer in the grey regions of the thalamus surface also vary in thickness, but not in composition (Panel XII, Fig. 3 a). —

Looser, but still fiber-rich neuroglial masses are then found below the compacted thalamus surface neuroglial masses. Where the layer adjacent to this second zone is depleted of neuroglia, there is often a tendency for the fibers to project in a radial direction (pl. XII, Fig. 2 b), but in other cases the ependymal neuroglia or the associated

interfibrillary neuroglial mass is directly followed by an irregular plexus (pl. XII, Fig. 3 b), which is similar to the configuration found in Stilling's ventricular nerve nuclei.

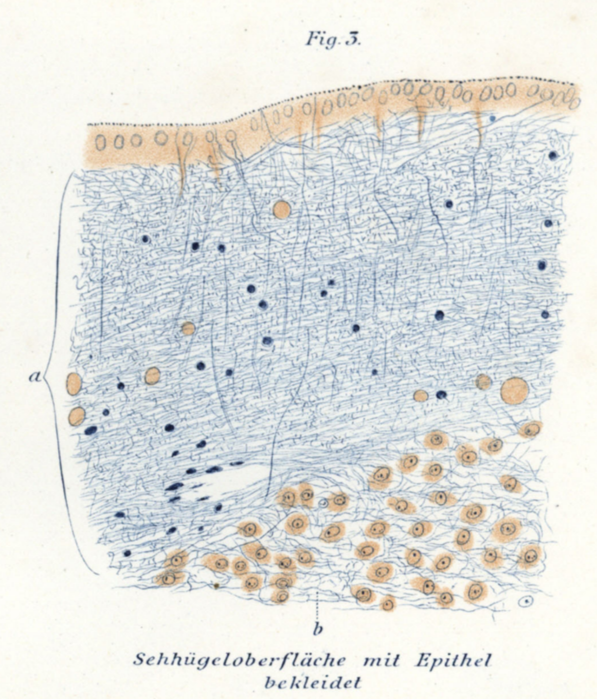


*Fig. 3 from Panel XII: Thalamus surface covered with epithelium. Magnification C. (a) Ependymal layer. (b) Ganglion cell layer.*

The distribution of neuroglia in the deeper regions of the thalamus opticus seems to be very different, because the ganglion cells in these regions are also arranged in many different groups (Nissl).

It is precisely these regions that require further study and presuppose a detailed understanding of the “thalamic nucleus". I am therefore only including an illustration of a particularly characteristic type of network found in the pulvinar in this section (Panel XII, Fig. 4). In this particular case there are coarse astrocyte structures from which abundant but loosely lying fibers radiate outwards, and also produce slight compactions around the ganglion cells. The image is very reminiscent of neuroglia found in the red nucleus, albeit that the red nucleus seemed to contain a greater abundance of neuroglia. The mesh of nerve fibrils in the thalamus is not as clearly visualized, as in the red nucleus.

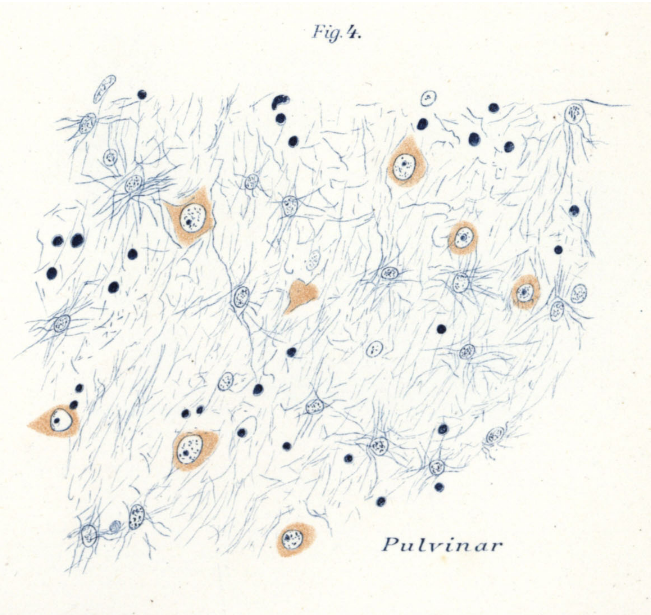


*Fig. 4 from Panel XII: From the depths of the pulvinar. Magnification C.*

## 14. Corpus striatum and capsula.

The head of the corpus striatum is covered with an ependymal layer of highly variable thickness, which is also conspicuously thin in places i.e., very similar to that observed in the thalamus.

This layer is then followed by the more typical loose fiber mass with many radial fibers. In all other respects, the deeper layers of the caudate nucleus and lentiform nucleus nevertheless reveal quite different configurations of neuroglia compared to the thalamus. With the exception of the vicinity of somewhat coarser vessels, there are otherwise incredibly few neuroglia in the corpus striatum and the lenticular nucleus, which is reminiscent of the cerebral cortex.

The capsula and the white tracks in the corpus striatum, etc., feature delicate, tight neuroglia scaffolds which are similar to the type found in white matter.

## Chapter 9:

## The physiologic function of neuroglia.

It would be very useful to deduce a fundamental physiologic principle from the previously described topographic features of neuroglia. It is incontestable that neuroglial fibers represent an intermediate tissue component, and that intermediate tissue components can be attributed a number of passive functions. But which of these functions specifically apply to neuroglia?

It is safe to say that neuroglia serves a space-filling function. This is particularly evident from pathological histology, because wherever space is freed by the demise of nerve tissue, neuroglia is observed to proliferate and fill the vacant space with their fibers. Irrespectively of whether this destruction affects only the myelin sheath, as in multiple sclerosis, or the entire nerve fiber, as in tabes [dorsalis] or secondary degenerations, or whether it results in the destruction of entire neurons, as in poliomyelitis anterior, or only parts of neurons, as in progressive paralysis, or whether all neuronal components are damaged (i.e., cells and fibers), as in ischemic necroses, a correspondingly smaller or larger number of neuroglial fibers are always observed to fill the vacant space. With regard to this, I wish to emphasize that, contrary to a view I expressed in earlier times, the new method readily identifies that even solid scars resulting from ischemic necrosis do not contain connective tissue but consist of densely woven, colossal neuroglial masses.

In addition to serving a filler function, neuroglia may perform other functions as well, and many hypotheses have been suggested along these lines. A preeminent example is Golgi attributing a nutrient support function to ganglion cell dendrites. Ganglion cell dendrites were presumed to fulfill this function by establishing associations with neuroglial cell extensions, which would somehow provide nutrients to ganglion cells.

We have already stated on p. 80 that our preparations do not allow us to identify any potential connections between dendrites and neuroglial fibers, although this type of interaction may well be detected using other methods. Our method does however allow us to confidently state that: irrespective of the nature of these yet to be proven interactions, Golgi's notion that dendrites have protoplasmic nutritional processes precisely because they are associated with "neuroglial cells" cannot be correct. Golgi's argument is only valid if the "extensions" of Deiters' cells are considered to be true protoplasmic cell processes. But we can now confidently confirm that Deiters cell "extensions" are not protoplasmic extensions and indeed that they are not cell extensions at all. Consequently, the concept that these fibers might be important for neuroglial cell metabolism and moreover that they may be indirectly involved in ganglion cell metabolism must definitely be dismissed.

We also reject the notion that neuroglial fibers function as capillary (sit venia verbo[1]) serous vessels, if only to transfer their nutrient fluids. This possibility could not be discarded as long as one agreed with Frommann, Lavdowsky and others that the fibers are hollow. We have also refuted this idea on p. 68, Paragraph 2. —

P. Ramón, as well as S. Ramón y Cajal and Sala y Pons suggested an alternative hypothesis. P. Ramón et al. believed that neuroglia mainly functioned to insulate the transmission of nerve impulses i.e., to prevent the formation of harmful secondary conduction of nerve currents (corrientes nerviosos).

Sala y Pons summarizes his reasoning as follows: "The assumption that neuroglia simply function as a support component does not in itself explain the peculiarity that the fibers ("cellular processes") are dense in some places, less dense in others, and almost completely absent in other regions still. It also does not explain the fact that the "cell processes" are smooth when passing through one region and shaggy when passing into another, which after all increases their surface area. These two characteristics appear to be more consistent with another function of neuroglia, namely, to prevent any arbitrary and harmful nerve tissue contacts.

[1] Latin idiom meaning "pardon my expression".

This hypothesis also accounts for the fact that neuroglia is very abundant everywhere in white matter, but absent or very scarce in grey matter where there are no transiting fibers. The shaggy fiber appendages may also serve to specifically reduce contacts because they are observed in zones where contacts need to be reduced but are absent in regions where this function is not required i.e., where axis cylinder ends and collaterals contact ganglion cell bodies and dendrites, which presumably results in a collision of nerve currents. Conversely, there is an abundance of neuroglia when this transmission of nerve currents from one entity to another needs to be impeded."[1]

This ends Sala y Pons' account. As far as the hypothesis of P. Ramón is concerned, we should stress that neither the supporting evidence, nor the theoretical justification are correct. The very first assumption that neuroglia are very abundant in white matter compared to grey matter is absolutely wrong. Quite to the contrary, we found the most abundant neuroglia masses in specific regions of grey matter. Furthermore, there is no evidence of frequent contacts of dendrites and axis cylinders occurring in grey matter regions that are depleted of neuroglia. Indeed, we observed an immensely rich neuroglial scaffold, which was much more abundant than in white matter, in regions where such contacts are certainly prolific such as in the olivary body, the quadruplet bodies, the Stilling's nucleus, etc. Finally, it is not true as we discussed earlier (p. 79, Paragraph 6) that those grey masses which contain many transiting myelinated fibers should also contain a greater neuroglial mass.

The eminent Spanish researchers were led astray because they relied solely on the Golgi method to investigate neuroglia. The Golgi method is unsuitable for evaluating the topographic features of neuroglia, for reasons that we have surely become sufficiently acquainted with by now.

Not only are the facts that P. Ramon based his hypothesis on incorrect, but his theoretical justification can also be contested. The need for an insulating function of neuroglia seems particularly superfluous in white matter, where axis cylinders are encased in thick, insulating myelin sheaths.

[1] "Sala y Pons, La Neuroglia de los Vertebrados" (Spanish in the Original), Madrid, 1804, p. 40.

This makes an additional insulating layer, like neuroglia, absolutely unnecessary.

Instead, it would be much easier to argue for the requirement of an insulating component in grey matter precisely at the sites where axis cylinders end and collaterals contact dendrites.

These contacts also cannot be allowed to transmit the corrientes nerviosos in an uncontrolled manner from one pathway to the other. No arbitrary nerve branch should be able to transmit an impulse from any dendrite it randomly encounters on its passage, or from any other nerve branch in its surroundings. Instead, there needs to be provisions that allow the conduction of nerve currents exclusively along prescribed pathway and that turn off all alternative secondary pathways. Even though the extensive branching and interweaving of dendrites and axis cylinders present an immense potential for harmful secondary conduction of nerve currents — when contrasted with white matter with its much less intricate pathways and with its thick myelin sheaths, P. Ramon still managed to argue that white matter requires more insulating components!

If we also consider that in contrast to myelin sheaths, neuroglia fibers are organized as networks and are not solid masses, as would be expected for an insulating layer, we cannot but drop the hypothesis of P. Ramón all together, — at best, one may perhaps ascribe insulating properties to the neuroglia that form coherent layers, in regions that are devoid of or almost devoid of nerve tissue components. This would perhaps be plausible for the outer and inner surfaces.[1]

To avert any misunderstandings, it should nevertheless be emphasized that the presumed requirement for an insulation component to avoid unrequited dendrite and axis cylinder current transmissions for the previously mentioned reasons, may not actually require a "general component" or another yet to be identified type of neuroglia.

[1] Curiously, Schleich attributes sleep to an "irritation of neuroglia" ("Schmerzlose Operationen" [Painless surgical procedures] Berlin 1894. p. 78 ff.) Although the irritation of an intercellular component is a very peculiar notion, Schleich does provide an illustration of neuroglia in the cerebral cortex on p. 89. He does depict a rich web of neuroglia fibers around a ganglion cell, but it amounts to pure fantasy.

Perhaps it is enough that the finer tendrils bathe in connective tissue fluid, which may serve as an insulator analogous to the oil used in the transformers for high-voltage currents. This assumes of course that connective tissue fluids do in fact function to insulate the minimal current voltages that prevail in dendrites etc., — but who knows what solutions nature resorts to? —

If we ascribe neuroglia a space-filling function, the very observation that led the Spanish researchers to their hypothesis does not seem so far-fetched. It was the changes in the abundance of neuroglia in the internal organs that these researchers found odd and identified as the specific issue that needed to be addressed. But it is conceivable that in some regions of the central nervous system, where nerve tissue components are sometimes in such close proximity to each other, there may simply be no space left for any other component, while in other regions the components are more loosely packed, and the interstitial spaces filled by a more or a less abundant "connective tissue component" depending on the size of the space. The deep layers of the cerebral cortex may therefore contain such densely interwoven dendrites and axis cylinders that there may be no room left for neuroglia, whereas in the olivary body the more loosely woven dendrites and nerve fibers may leave enough space for neuroglia. Changes in the abundance of neuroglia within organs may well be explained without resorting to the untenable insulation hypothesis.

What is perhaps more striking and in urgent need of clarification is not the abundance of neuroglia inside the nerve tissue regions, but rather the extremely diverse and yet characteristic organization of neuroglia in specific regions of the central nervous system, as well as changes in the abundance of neuroglia in the upper surfaces of organs, which cannot simply be reconciled with the neuroglia filler function. It is particularly the frequently recurring types of neuroglia in the upper surfaces of the organs which inevitably forces us to concede that the space-filling function of neuroglia under normal physiological as well as pathological conditions does not proceed in an arbitrary manner. The various types of neuroglial networks must follow specific structural principles, analogous to those that have long been demonstrated for other connective tissue components, notably: for the organization of the normal bone trabeculae by Culmann, Hermann v. Meyer and others, for the pathological bone features by Julius Wolff, for the fibers in dolphin flippers, and even for the branching of the blood vessels by Wilhelm Roux, etc..

The dense networks present on the inner and outer surfaces, in particular, must be conditioned by such mechanical or structural principles. Lloyd Andriezen already drew attention to the fact that the glial sheath, which is often quite prominent, may function to protect brain matter against "undue expansions" of the vessels[1]. Cerebral vessels only possess a weak adventitia and are very thin walled in general, which means that their own wall would not provide sufficient resistance to fluctuations in blood pressure. Consequently, they are in dire need of structural support from the neuroglial sheath. Lloyd Andriezen further noted that this type of protective defense, although characterized by a dense network, is also based on a mesh construction, to not impede the flow of fluids into and out of the blood.
Other neuroglial compactions identified on the surfaces could also represent similar protective configurations to ward against some, as yet unidentified mechanical influences, — because as discussed on p. 76, compactions around the vessels may be considered as a type of surface compaction too. But this is not the whole story.
The typical, so often repeated configuration of neuroglia needs to be explained first. We have already drawn attention to the fact that both the compaction of glia around the vessels and the compaction in the outer surfaces share organizational similarities (refer to p. 77). Both feature a particularly dense network of more or less transverse fibers, followed by a less dense, but still fiber-rich network of predominantly radial fiber strands — this observation cannot be incidental. Changes in the composition of these surface compactions (in a broader sense) must also be significant. Why is there such a variation in the ependymal layer thickness of the central ganglia? Why does the lateral side of Corpora candicantia have such a pronounced cortex layer, but the medial side such a thin one? Why is this structure completely absent on the surface of the cerebellum, etc., etc.? Why are radial fibers absent in much of the grey matter under the ependymal layer, but are present in other regions of grey matter and why are they so evenly distributed throughout white matter? Why are myelinated nerve fibers sometimes covered by an intermittent ependymal cortex layer or by an external cortex layer, but sometimes only by an interfibrillar layer?

[1] On a system of fiber-cells surrounding the blood-vessels of the brain of Man and Mammals, "Internationale Monatsschrift für Anatomie und Physiologie" [International Monthly Journal of Anatomy and Physiology], 1893, p. 539.

What are the enigmatic compressive, tensile, and shear strength constraints that give rise to these characteristic trajectories?

There are many more questions that could be asked, and no doubt even more that will be asked as our understanding of neuroglia topography improves.

This proves yet again, as we saw in the introduction, that the answer to the question concerning the topographic distribution of neuroglial fibers, like the answer to any other natural science question, is succeeded by always new, previously unforeseen questions, indeed that every "therefore" gives rise to many "why's", — and this will likely continue on with no end in sight. —

## Chapter 10:

## Method.

We already summarized the most important requirements that need to be fulfilled by a method for staining neuroglia in the concluding remarks of our historical overview. But we can go into a bit more detail in this chapter about the specific criteria that a usable method needs to meet.

1. The primary criterion is that the staining needs to be selective i.e., the method should not stain anything that may be mistaken for neuroglia fibers or that may prevent the fibers from clearly standing out from the background.

It is most interesting to see how, over time, the expectations in terms of this criterion have gradually increased. I still remember my admiring amazement when, in my first semesters of study at the Berlin Physiological Institute, when I looked at the spinal cord charts drawn, if I remember correctly, based on Goll's illustrations. Everything in these pictures was colored red with the exception of the myelin sheaths. And yet even this technical achievement of Goll's was considered a major advancement. Kölliker must have looked at similar images because the figures in the 4th edition of his "Gewebelehre" [Histology], also depict the interstices between the myelinated nerve fibers of the spinal cord with the same diffuse, undifferentiated masses as they appeared on those panels.

Consequently, it was already pleasing at that time if the myelin sheaths remained unstained and everything else stained, preferably in different shades of red.

Regarded as the second stage of development, neuroglia fibers present in the white matter of the spinal cord were subsequently stained with carmine so that the component (tissue fluid?) in the spaces between the fiber strands, which stained in the Goll and Kölliker specimens, was either excluded from the staining or was stained in a very pale color.

Obtaining this type of differential staining was largely a matter of luck because the standard carmine solutions of the time could not be trusted at all, as I recall from my own youth. Frommann must have studied specimens that were already more differentially stained like that.

One could now discern a feature of neuroglia that had eluded detection earlier, namely, that neuroglia had fibrous characteristics, which is why Frommann always called neuroglia "fibers," even though he believed that they were actually cell extensions.

At least, this new development allowed to study neuroglial fibers in the white matter of the spinal cord and in similarly favorable regions. As we now know, and as Boll had already suspected, this type of staining was not reliable even for the better-suited location i.e., for the white spinal cord matter, since here, too, axis cylinders run collaterally, the existence of which was ignored at that time, — and axis cylinders also stain in this improved carmine protocol.

In the less favorable regions, in particular the grey masses, the degree of uncertainty was so high that it became unreasonable to make even very modest claims, and so all authors, from Clarke and Frommann to Petrone and Lavdovsky, complained about the difficulty of determining what could be counted as neuroglia when using carmine and similar staining methods. —

We now needed a neuroglia staining method that did not stain the myelin sheaths, the (presumed) tissue fluid, the axis cylinders, or the dendrites of the ganglion cells. Any methods in which axis cylinder and ganglion cell staining cannot be definitively excluded should be discarded without any reservations.

A recent example illustrates just how misleading methods which also stain axis cylinders can be, particularly for pathological anatomists. Popoff[1] published a preliminary communication from Flechsig's laboratory reporting results of his investigation of disseminated sclerosis. He used a triple staining method for these experiments, which included "patent acid rubin" (aka acid fuchsin). Popoff now claimed to have found the following:

[1] "Zur Histologie der disseminierten Sklerose" [On the histology of disseminated sclerosis.], "Neurologisches Zentralblatt" [Reference journal of neurology], 1894. p. 321.

"Furthermore, based on my own investigations, I cannot agree with the generally held view that these are connective tissue proliferations. My microscopic preparations clearly show that what most observers thought were connective tissue tracks lying between the nerve fibers are only products of changes in the nerve fibers themselves" (p. 322). It is supposed to relate to proliferation and alterations more specifically in axis cylinders.

Regardless of the results from older observations, the inaccuracy of this so uncommonly paradoxical statement is most strikingly shown by our staining. Even in acute cases of multiple sclerosis, and even more so in chronic forms, one is almost convinced that these are indeed colossal "connective tissue" i.e., neuroglia proliferations. A method that invites such misconceptions as those drawn by Popoff is absolutely useless under all conditions. —

Needless to say, any new method must not stain ganglion cells and their protoplasmic extensions. Methods that stain neurons in the same or a similar tone as neuroglia not only run the danger of mistaking dendrites for neuroglial fibers, but also have the great disadvantage of not making fine neuroglial fibers stand out sufficiently from the rich network of dendrites, which means that they may not be detected reliably. Given this and given that Deiters cell bodies generally stain even more heavily than ganglion cells, it would be fair to say: that all the methods which stain Deiters cell bodies in the same color tone as neuroglia fibers i.e., all the methods in which the fibers appear as real extensions of the said cells, are not useful for studying neuroglial topography. Also of little use are those methods which only produce a slight difference in the intensity of the color between the cell body and the fiber. —

It is generally less important for the method not to stain the connective tissue as well. Firstly, because connective tissue is also not a nerve tissue component, but is instead an intermediate mass, like the neuroglia, and secondly because the structure of connective tissue is so different from the structure of neuroglia that it is unlikely to lead to any confusion.

Virchow, who discovered neuroglia, had already recognized the difference between neuroglia and true connective tissue using his primitive methods.

It may however be desirable to keep the connective tissue unstained under some circumstances, particularly for pathological anatomists, and it would be advantageous to at least have the option to exclude collagenous masses from the staining. As far as elastic fibers are concerned, it is not particularly difficult to prevent them from staining. In contrast to collagenous tissue, elastic fibers only stain with very specific methods. —

In terms of the requirements of a neuroglia staining method discussed, our method so far satisfies each and every one of them.

2. A second key requirement is the reliability of the method i.e., each preparation should show every single neuroglia fiber present in any region of the tissue. This prerequisite is less important for the normal tissue anatomist than for the pathologist. The normal tissue anatomist is content just to see a single area of any specimen completely stained. This allows the anatomist to identify the type of neuroglial network to always expect to find in this specific region. The pathological anatomist needs to be more demanding for reasons I elucidated earlier.[1]

So far, I have not yet fulfilled the requirement discussed in the strictest sense of the term, despite many years of effort. I still get empty spots inside the regions that should contain neuroglia networks, but the method is nonetheless quite reliable.

However modest expectations about the reliability of a method may be, one thing can be demanded under all circumstances, namely that the success of the method is not predicated by the fickleness of a very short incubation time in any steps of the protocol. Any protocol whose success is determined by whether the tissue is incubated for one second more or for one second less needs to be discarded.

[1] “Merkel and Bonnets "Ergebnisse der Anatomie und Entwicklungsgeschichte" [Results from anatomy and developmental history], 1891, Vol 3, p. 19 f.

3. For neuroglia staining it is also desirable to recognize other tissue components, at least for orientation purposes. First of all, it is important to see the nuclei, this is essential for pathological processes. Nuclei may even stain the same color as neuroglial fibers without causing any problems. No one will mistake a nucleus for a neuroglia fiber, and the clarity of the images is in no way affected by the nuclei also staining; if anything, the opposite is true. This prerequisite was very easy to fulfill.

What was more difficult was to visualize the nerve tissue components at least to the extent necessary to not lose orientation of tissue in the preparations. It was not necessary to make more exacting demands, but nervous tissue components had to stain with a contrasting color to neuroglia i.e., not in a similar color to the neuroglia fibers, for reasons elucidated in Paragraph 1 above.

This caused great difficulty because all the dyes I tried could not reliably avoid damaging neuroglial staining. I finally came up with another substance that not only does not damage neuroglial staining, but also increases the intensity of the color. It is true that my method does not stain ganglion cells as well as the Golgi method, but we do not wish to study ganglion cells, we simply want to see where they are located. Besides, it turned out that at least the coarser Nissl granulations stood out quite nicely. That was more than was actually necessary, although it was of course a very good feature to have.

4. I struggled for a long time with the fact that although the fibers stained, the staining was pale and hardly strong enough to identify the fibers as fibers at low magnification. I therefore pursued greater intensity of staining and achieved it for relatively modest requirements. There is no need to demand the fibers appear as black as in the Golgi method, it is sufficient to get a good overview of the networks at low magnifications. After all you can still study the networks in detail at high magnifications.

5. A grievance long felt by pathological anatomists and particularly by clinicians is that the preparations take such a tremendously long time to harden in dichromate salts.

It is quite true that as far as histology protocols are concerned the "tuto" (reliably) precedes by far the "cito et jucunde" (swiftly and sweetly), but within reasonable limits. If it takes months to sufficiently harden the tissue and perform the metallic impregnation, this is an imposition that can only be endured if there is no other way to achieve the "tuto" (reliability). I tried to address this a long time ago. I initially dragged Erlick's fluid [1]out of obscurity, — but it penetrates too unevenly to yield useful results. I then tried adding heat, but even with this, weeks went by and there was no way of protecting preparations from becoming brittle. We will see that specimens for myelin sheath staining can now be prepared without applying heat in as little as four days. These types of preparations could also be used for neuroglia staining, but this requires a different hardening method which only takes a little bit longer.

6. I also struggled for a long time with the fact that the hardening and any subsequent treatments caused the tissue to shrink, become brittle, or the like.

I therefore abandoned entire procedures and tried to find alternatives, because it is essential to ensure that any manipulations necessary to prepare the tissue do not damage the tissue. This objective has also been achieved to my satisfaction.

7. Finally, it was also desirable, although not strictly necessary, to ensure the staining was preserved over time. My first preparations have held up quite well, they still look very nice after five or six years now. But when I reworked the method to fulfill the other more important requirements, the results suffered a lot because the staining faded with time. It barely lasted 8-14 days. The current preparations seem to be holding up, but I cannot guarantee that they will last long.

As with all methods established empirically, and the neuroglia staining method is no different, it was difficult to really understand the basic principle of the method, and yet it was absolutely necessary to have a clear idea of this principle to be able to perfect the method.

[1] Erlick's fluid is 2.5 g potassium bichromate, 0.5 g copper sulfate in 100 ml of distilled.

I was still on the wrong track in 1890. I was at the time convinced, as I also published in my preliminary communication, "that the preparations had to be impregnated with metal salts containing an organic acid." I searched for a long time afterwards to find the right organic acid and the right metal salt, until I finally found out that the metal salt and the organic acid had to be associated completely differently than by a simple bond. The metal salt had to be incorporated into the preparations in a highly oxidized state, with the organic acids that I had used successfully only playing the part of a reducing agent.

We could perhaps explain this empirically derived principle in theoretical terms, as follows: The dye only adheres to neuroglia if these contain a strongly reduced metal compound. But such a strongly reduced metal compound cannot be directly attached to neuroglia. The metal only binds to neuroglia in its highly oxidized state, or possibly, as we shall see, as a mixture of higher and highest oxidation states. To realize its staining potential, the metal first had to be added to neuroglia in a more highly oxidized state, and only then can the strong reduction be carried out. This is, of course, only a hypothesis. It would also be conceivable that the metal compound only caused a change in the neuroglia themselves. The first hypothesis however seemed more likely to me, because we also know from other technical staining applications that basic aniline dyes adhere better to very fine precipitates (e.g., methyl green to very finely distributed sulfur). —

After I had figured out the basic staining principle, I tried a large variety of different metal compounds and reductions, hoping to finally obtain a reliable, selective stain. But I could not get beyond a certain point. After many wrong turns I realized that the error lay somewhere else entirely: namely, in the very first part of the procedure that needs be performed for preparations from the central nervous system.

Although I knew from the beginning (refer to my 1890 communication) that only very fresh tissue that had "a good consistency" should be used, I believed that the conventional hardening methods also fixed the tissue quite reliably, all the more so since this reliable fixation method had been proven successful for staining myelin sheaths.

With the advent of 10% formaldehyde, which allowed to quickly fix central nervous system tissue, the thought that fresh brain or spinal cord tissue, cut in the conventional way, would not be well preserved, took a while to cross my mind. But I eventually found that neuroglia is incredibly sensitive to 10% formaldehyde fixation.

If the hardening liquid had not completely penetrated and fixed the preparation within 24 hours, the innermost regions of the specimen became unsuitable for (neuroglia) staining. This generally applied to any parts of the tissue the liquid has not immediately penetrated.

This phenomenon is different to simple cadaveric softening. In the latter, neuroglia disintegrates into granules, but can be stained for quite some time, whereas in the case of decomposition within the hardening liquids, neuroglia loses their ability to take up stain right from the start. It is presumably the water component of the hardening liquids that causes this difference. Unfortunately, the problem could not be overcome by using absolute alcohol. Alcohol is indeed very detrimental for unstained preparations as far as neuroglia are concerned. It also does not help to dissolve metal compounds in the alcohol. This always results in very unreliable, often tremendously poor neuroglia staining.

We eventually found a very simple, but admittedly also very inconvenient way around this problem (see Paragraph 1 a below).

The neuroglia staining method therefore comprises 3 — 4 main steps: I a. Fixation of tissues collected from the central nervous system, I b. Impregnation with highly oxidized metal compounds. These two steps may also be combined into one single step. 2. Reduction of the metal compound 3. Staining.

1. Fixing and metallic impregnation.

   a) These two steps can be performed separately or combined into one single step as previously mentioned. The two procedures are performed separately to allow tissue specimens to be treated using methods other than our new one e.g., the March, Golgi or Nissl method or for staining myelin sheath. In this case, the tissue is in 10% formaldehyde. Beware of more dilute solutions; they do not fix the tissue well enough. Fixing tissue in more concentrated solutions did not achieve better staining. But to obtain decent neuroglia staining, it is absolutely essential to cut tissue into small pieces, no more than half a centimeter thick, before placing them in the fixing agent.

It goes without saying that the tissue specimen must be very fresh i.e., no cadaverous softening.

Although 10% formaldehyde may harden larger pieces of tissue, these will not yield reliable neuroglia staining.

The tissue size restriction for fixing is very inconvenient, but for the time being I have not yet found a way around it.

The hardening step can be done in large flat dishes with lids e.g., like the plates used to store bacterial plate cultures. The standard procedure is to line the bottom of the dish with blotting paper. This prevents thin tissue pieces from distorting. The 10% formaldehyde solution should be changed after the first day, but this is not necessary thereafter. When the tissue pieces have hardened (after about four days) and the tissue is unlikely to distort any further, preparations can be moved to tall, less space-consuming jars. They can be stored like this for many years and still stain well.

b) the metallic impregnation can be performed on 10% formaldehyde fixed or on fresh tissue. I have already reported this in my article "Technique" published in Merkel and Bonnets Ergebnisse der Anatomie und Entwicklungsgeschichte, 1894 [Results from anatomy and developmental history]. Combining fixing with the metallic impregnation has for a long time been the preferred method for staining central nervous tissue. Bichromate treatment fulfills the dual purpose of hardening the tissue and impregnating it with metal. Pieces of tissue impregnated with chromates are also suitable for neuroglia staining, as long as they were not hardened or impregnated with the original Müller solution (2 ½ % potassium bichromate with or without I % Glauber's salt[1]), which is still being used here and there. Neuroglia stainability is completely lost when using such weak solutions. But pieces hardened in the (now probably most commonly used) saturated (approx. five percent) solution of potassium dichromate yield good neuroglial staining when using the appropriate methods and small enough pieces of tissue. I have however completely abandoned chromium hardening altogether, because one is never sure that axis cylinders do not also stain.

[1] Glauber's salt is the decahydrate form of sodium sulfate.

This is such a fundamental flaw that I would not even be discussing chromium hardening, and many other methods that I left by the wayside if I had not found something of great use for myelin sheath staining in my experiments.

I have succeeded in significantly shortening the time required to obtain suitable hardening and metallic impregnation of the specimens for myelin sheath staining to 4-5 days.

Theoretical reflection led me to uncover a feature about how myelin sheaths bind chromate, which is an interaction that is required for the color coating, namely that the reaction proceeds much faster when a suitable amount of a chromium oxide salt is added to the strong bichromate solution. Too little chromium oxide salt is not recommended because the hardening and the impregnation steps will take too long, and too much should also be avoided because the solution will penetrate too much and cause the preparations to become brittle too quickly.

The type of dichromate used is not important, potassium, sodium or ammonium bichromicum all work well. Sodium bichromicum it the easiest to dissolve and the cheapest. The type of chromate salt used is also quite arbitrary, you can use chromate acetic acid or oxalic acid reactions or any other version commonly used in technology are all suitable; but I would recommend the very cheap chromium(III) potassium sulfate (potassium chromate and sulfuric acid), which is easy to obtain in crystallized form.

The solution consists of 5% potassium (sodium or ammonium) bichromicum and 2% chromium(III) potassium sulfate dissolved in water by boiling. If precipitates form upon cooling, remove them by decanting or filtering the solute, as these precipitates form a fine sludge around the tissue pieces, which makes it difficult for the liquid to penetrate.

Do not place tissue pieces that are too thick into this mixture because the solution will not penetrate quickly enough. The metallic impregnation and hardening should take 4-5 days to complete. Tissue pieces can be left in the chromium alum bichromate solution for up to 8 days, but no longer, otherwise they will become brittle. The tissue pieces are then rinsed with water and treated with alcohol as usual.

The necessity to use thinner tissue pieces when using this method is not an inconvenience. Because larger tissue pieces can be hardened in 10% formaldehyde beforehand.

The thinner tissue pieces can easily be cut out of such hardened masses without worrying about distorting the tissue.

The hardening step can of course also be performed directly in the chromium(III) potassium sulfate, bichromate solution (on small tissue pieces, of course), by adding 10% formaldehyde to the solution.

I do not use such bichromate-hardened tissue pieces for neuroglia staining any more but would like to, for the time being, recommend a different metallic impregnation for the standard neuroglia staining method. It is a copper impregnation which (as is the case for the copper plating used to stain myelin sheaths) includes a neutral copper oxide acetate as its main component. The most important consideration, however, was to eliminate the precipitate that is so troublesome in the conventional aqueous solution of this copper salt, and to find a compound that also adhered well to neuroglia. This objective can be met in a number of ways.

I will only mention one mixture which has proven quite successful: it consists of 5 % copper oxide acetate, 5 % conventional acetic acid and 2 ½ % chromium(III) potassium sulfate in water. A few precautions must be followed when preparing this solution. If copper and acetic acid were simply added to the chromium(III) potassium sulfate solution prepared cold, or vice versa, this would produce a voluminous greenish precipitate. The precipitate does not form when the chromium(III) potassium sulfate is boiled in water and then mixed with copper and acetic acid.

My explanation is that the green modification, which the chromium(III) potassium sulfate forms when boiled in water, behaves differently to the acetic acid copper solution than the violet modification, which is formed when dissolved cold. It is however important to vigorously boil the chromium(III) potassium sulfate solution and not to just heat it, because only then will all the violet salt be converted to the green salt.

This is why the chromium(III) potassium sulfate is first boiled in water (in an enamel pot with a lid). Once it comes to a full boil, turn off the flame, add the acetic acid first, and then the finely powdered neutral copper oxide acetate. Stir diligently until the copper salt has all but dissolved and one can, using the glass rod, feel only a very small remnant of sediments. Then leave to cool. The fluid always remains clear.

This solution is also recommended for myelin sheath staining, as it does not precipitate on the chrome plated pieces, and on the other hand, it has the advantage over the Seignette salt solution[1] that further copper plating with a simple aqueous solution of the copper salt is unnecessary.

[1] Seignette salt is a potassium sodium tartrate tetrahydrate (KNaC4H4O6 x 4 H2O).

If tissue pieces have been previously treated with 10% formaldehyde (at least 4 days), they are placed in the copper-oxide-acetate and chromium(III) solution for at least 4—5 days at hatching incubator temperature[1], or at room temperature for at least 8 days. For single neuroglia staining it is best to omit the separate 10% formaldehyde step and instead incubate fresh tissue pieces (no more than 1/2 cm thick), directly in the copper-oxide-acetate, chromium(III) solution, and add formaldehyde to 10%. The solution is changed on the second day of incubation. Another change later is perhaps desirable, but not necessary.

Tissue pieces treated in this way (not chrome plated) are unsuitable for myelin sheath staining as are tissue pieces treated with copper oxide acetate alone. I disagree with van Gieson on this point. The differentiating solution removes excess staining from tissue sections far too quickly and unevenly.

Tissue pieces treated in the single step copper, chromium(III) solution, 10% formaldehyde solution should also be incubated at room temperature for at least 8 days. Prolonged incubations do no harm, tissue pieces never become brittle.

The pieces intended for sectioning are rinsed with water, dehydrated in the usual way in alcohol and embedded in celloidin.

2. Reduction. The reduction of the chrome plated preparations is performed differently for neuroglia staining than for copper plated samples. Since neuroglia staining obtained with the chromium preparations currently does not satisfy the staining requirements for these structures, I will refrain from going into detail about the reductions that are possible in this particular case. But the reduction does need to be discussed for all the other types of staining.

Many people, especially ophthalmologists, have regarded the fact that fibrin and microorganisms do not stain in chromium preparations using the method I described as a shortcoming. This can nevertheless be achieved by placing sections from chromium preparations in reducing solutions. It is sufficient to incubate sections in 5 % oxalic acid for some time, preferably a few hours. Fibrin staining etc. can then also be performed on preparations hardened in potassium dichromate.

[1] Weigert says "Brütofen Temperatur" [hatching oven temperature]. The incubator temperature for hatching chicken eggs is between 37 to 39 degrees Celsius.

This method is unsatisfactory for neuroglia staining. We will therefore discuss the reduction for the copper plated sections.

There are several reduction options for copper plated sections, but there are fewer options if the finer fibers need to be stained more reliably and for the sections not to become too brittle. All the various reducing agents recommended for photographic purposes, all of which I have tried, are unsuitable. This is particularly true for those used in alkaline solutions since they damage the sections. Other reducing agents are again too weak, the reduction must rather be a very energetic one. The best method is to use potassium permanganate and sulfuric acid, which has long been used in technology but was first used in histology by Lustgarten. Lustgarten first used this reduction (independently) in the Leipzig Pathological Institute in 1884. He brought it to Vienna, where it was adapted by Pal (with very few changes) and used as a modification for my myelin sheath staining. The Lustgarten method can be used directly. However, a small modification which uses a compound that is an effective contrast stain and an intensifier, works even better.

This compound was first used in technology under the name "Chromogen" by the Höchster Farbwerke [Hoechster dyeworks] and, like so many other reagents, was a very kind gift, for which I would like to express my sincere thanks. This substance, which is not a dye itself, is used in technology as a dye, but serves a different purpose in our case.

Chromogen is a naphthalene compound, namely the sodium acid salt of the 3—6 disulfonic acid of 1—8 dioxynaphthalene, thus:

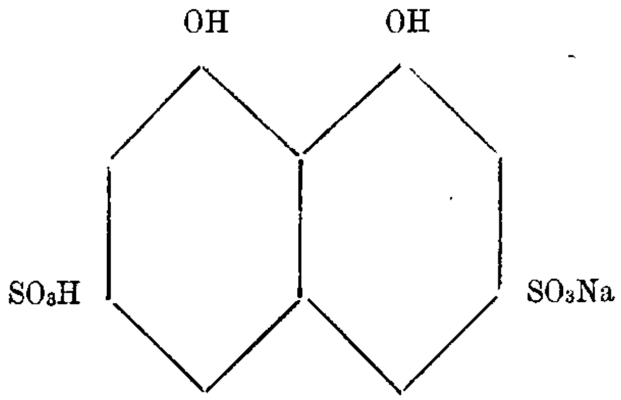

The solution reacts as an acid and has a reducing effect, changing the hydroquinone-like compound into a quinone-like one.

But the reducing capacity of the simple-aqueous solution is not strong enough to stain the finest fibers; the reducing capacity of the reagent therefore needs to be considerably strengthened using the principle of Lustgarten's method of staining histology.

To this end, dissolve 5% chromogen and 5% formic acid (the one I used had a specific weight of 1.20) in water. Filter carefully. Before use add 10 ml of a 10% solution of sodium sulfite (simply sodium bicarbonate and sulfuric acid), which is commonly used in photography, to 90 ml of the chromogen solution.

First, add an approximately 1/3 percent solution of potassium hypermanganate to the section and incubate for about 10 minutes. Pour off the solution and wash sections with water. Then pour off the water and add the previously discussed reduction solution to the sections. After only a few minutes, the sections, which turned brown due to the potassium permanganic acid have lost their color, but it is still better to incubate them in the solution for another 2—4 hours.

When the sections are stained as described below, the neuroglia fibers are blue, but the connective tissue is colorless. If the connective tissue in the section does not need to be stained, the staining procedure can be completed at this stage.

Most applications however do not specifically require connective tissue to appear colorless (refer to p. 130 f.) it is therefore recommended to follow the actual reduction reaction with a procedure which stains collagenous tissue blue with a tinge of violet. This subsequent procedure has the great advantage of staining neuroglial fibers even darker and of making the finer fibers also clearly stand out. Its second advantage is that is makes ganglion cells, ependymal and the coarser axillary cylinders appear yellowish as indicated earlier (p. 132, Paragraph 3). So do not let the minor effort and the small loss of time detract from it.

This procedure consists of pouring off the reduction solution and washing sections in water twice before adding a simple (i.e., not acidified) saturated aqueous solution of Chromogen. The Chromogen solution is prepared by dissolving 5 % Chromogen in distilled water. Filter carefully.

Incubate sections in this solution overnight. The longer the incubation, the more the nerve tissue components will stain in the contrasting color.

Then pour off the solution and wash sections in water twice. The color of the sections is now ready to be developed. — There are instances where the color cannot be developed straight away after finishing the previous step. If sections are left in water for a long time at this stage of the protocol, they produce weaker colors. Even pure alcohol (diluted, of course, because of the celloidin) is not suitable. But a solution of alcohol with oxalic acid (90 ml 80 % alcohol with 10 ml 5 % oxalic acid) has proven to be effective. Sections can be left in this alcohol mixture for days without compromising their ability to take up the stain, which means that the staining can be completed when convenient. This alcohol treatment also seems to preserve the color of stained sections for longer.

3. Staining. In the early days, I believed I could improve the reliability of neuroglia staining by modifying the different procedures of the fibrin method (which I used from the beginning). But it became apparent that only extremely slight modifications of the fibrin method were in fact necessary, and that any additional changes did not improve the reliability of the staining method. The reliability was most notably improved by variables already discussed in Paragraphs 1 and 2 above.

I am presuming that the fibrin method is generally well-known. I will therefore only mention minor deviations from my original method and add a few comments, that are in my experience not always given sufficient consideration.

Fibrin staining using my method requires the following three solutions: 1. a methyl violet solution, 2. A potassium triiodide solution. 3. a xylene aniline oil mixture. The second solution is borrowed from Gram's method for staining microorganisms, the third solution is one that I developed.

But because fibrin never successfully stained using Gram's original method i.e., when using the alcohol instead of the aniline oil, I devised a new protocol for staining fibrin which uses the third solution. In terms of the bacterial staining, the xylene aniline oil makes the methyl violet iodine method more reliable. This means that the fibrin method is only a modification of Gram's method for staining bacteria.

Neuroglia staining uses an unmodified potassium triiodide solution ("saturated solution of iodine in five percent potassium iodide"). The other solutions do however need to be modified a little. Instead of an aqueous methyl violet solution, an alcohol-based (70-80 % alcohol) solution is used (only use the liquid portion of the heat saturated and decanted solution after cooling).

To every 100 ml of this solution add 5 ml of a five percent aqueous oxalic acid solution. While adding oxalic acid is not necessary for the staining itself, a small amount of oxalic acid does seem to preserve the preparations better. Note that aniline oil is not added to the alcohol-based methyl violet solution.

The xylene aniline oil solution is not used at a 2 to 1 ratio of aniline oil to xylene, as in the typical fibrin method, but at equal parts of both compounds mixed together.

Apart from this modification, the procedure for neuroglia staining is identical to the fibrin staining procedure. Sections should not be too thick i.e., no thicker than 0.02 mm. Tissue can be easily sectioned to this thickness. Staining and all subsequent procedures are performed using tissue sections mounted on glass slides. Tissue sections should not contain wrinkles or folds. This is quite simply done by floating sections in a large bowl of water and then lifting them up with a slide that has been wiped with alcohol beforehand. Generally, tissue sections will stick to slides cleaned in this way without forming wrinkles. If wrinkles do start to form, dip the slide back into the bowl of water on its side so that the wrinkle is horizontal to the surface of the water. This will allow the wrinkle to resolve on its own. The dye is then carefully applied to sections that have been wiped dry. The staining occurs almost instantaneously. It does no harm, but it also does no good if the solution is left on the section for longer.

The potassium triiodide solution is also carefully applied to the section (stained and wiped dry) and poured off again immediately. A longer incubation in the iodine is not recommended in this case either. A very long incubation in the iodine solution will deteriorate the color rather than improve it.

When washing out the iodine solution with the xylene aniline oil, do not be afraid to proceed quite thoroughly. The finer fibers only start fading after a 15 to 30 min incubation in this solution.

The xylene aniline oil must be washed off very carefully several times with pure xylene before placing the sections in balm, otherwise the preparations will not keep. Neuroglia is more sensitive to this step than fibrin, which I only realized much later.

It is also very strange that the sections keep better if you do not put them in the dark right away but leave them exposed to diffuse daylight for 2—5 days first.

Finally, a comment about drying the sections with blotting paper. Not every type of blotting paper is suitable for this purpose, blotting papers with a grainy surface are particularly unsuitable. We have been using filter paper no. 1116 from the Ferdinand Flinsch company, Großer Kornmarkt 12 in Frankfurt a. M., for many years.

It is also important that the wad of blotting paper does not slip onto the tissue section, otherwise the preparation will tear. Hold the wad of blotting paper between the two fingers of the left hand and firmly dab the (empty) portion of the slide with it.

The new neuroglia-only staining procedures can be summarized as follows:

1. Fixation and impregnation in the acetic acid copper oxide chromium(III) potassium sulfate solution made up to 10% formaldehyde: 8 days.
2. Embedding of tissue for sectioning (celloidin method): 3 days.
3. Preparation of sections.
4. Reduction with potassium hypermanganate and the chromogen solution with sulfuric acid.
5. Amplification of staining for neuroglia and contrast staining of nerve tissue components with the simple aqueous chromogen solution.
6. (Modified) fibrin method.

Altogether, 3—6 require one day. Total time required: 12 days.

As I already partially reported in 1890, a very similar staining method can in principle be used for many other tissue components: to visualize the biliary capillaries, the cuticular components of the renal epithelia and other epithelial cells, to stain splenic structures, the dual light refracting component of striated (and smooth) muscles or (with a modification) to visualize the intermediate discs in muscle fibers, etc. Many colleagues have viewed specimens stained using these methods in my laboratory over the years.

I reserve the right to make further disclosures about any of these issues.

I can only consider the end of the new method, which I have now reached, as a provisional result. If one accepts the inconvenience of the hardening step, then the requirements we have listed are partially fulfilled, in such a way that the method is at least usable. However, one very important requirement, namely the reliability of the method, has not yet been totally optimized. Until the method yields almost mathematically consistent results, it cannot be described as complete.

There is also another shortcoming, which is inconsequential to the pathological anatomist and the "human histologist": **neuroglia staining can so far only be applied to human tissue. The method is not yet recommended for animals.** Neuroglia of rabbit brains consistently only stain very faintly and do not quite stain selectively. I do not yet know why. In time, I hope, I will be able to eliminate this as well as other deficiencies.

Now that it has been shown to be at least a selective and complete staining method for neuroglia, with some degree of reliability, perhaps other, fresher forces, which have not been hindered by many years of narrow thinking, will find a perfect method based on a completely novel approach. Based on my experience so far though, many researchers will also try to walk further in my footsteps to improve the paths I have opened up. I also firmly believe that, as was the case with my earlier published methods, this method will also yield a rich harvest for the modification specialists.

**Comments:**

[1#] Drachme was a medieval weight unit, about 4 gram, Unze corresponds to 27 to 31 g (from Wikipedia)
[2#] with light he means without much structures.
[3#] The third edition appeared in 1862.
[4#] It maybe that he distinguished between astrocytes and oligodendrocytes - the cells in rows are probably the oligodendrocytes.
[5#] He means that the stain can greatly vary.
[6#] The original German word Kurzstrahler describes actually a cell with short branches. Lenhossek used that. In contrast Langstrahler are cells with long processes.
[7#] I guess he is describing oligodendrocytes.

## Figure legends.

### Preliminary comments.

Almost all figures are reproduced with the assistance of the Abbe device, most with the new model (No 44a) and on the Bernhard drawing table. The coarser shapes, the fibers that were not too close together, and all nuclei were reproduced exactly as they appeared, one stroke at a time. This was not possible for the denser fiber masses. For these only the general network characteristics are reproduced as closely as possible. The very fine fibers also appeared too blurred in the drawing apparatus to be traced directly by pen or pencil.

The new drawing table (Zeiss, no. 105a of the 1895 catalog) is well known for being adjustable. The no. 90, 45 and 0 settings respectively correspond to the base of the microscope, the microscope stage, and the highest position above the stage.

All drawings, with the exception of Figures 2, 4 and 5 on Panel XIII, reproduce images visualized with Zeiss apochromatic lenses in a 3 mm homogeneous immersion with a 1.30 aperture (except for Figure 3 on Panel X). Figure 3 on Panel X reproduces images visualized with the 8 mm apochromatic lens.

Most of the figures are also drawn with the stage set at 0, some at 45. Figure 1, Panel I, and Figure 3, Panel X, used the 90 setting.

The bottom of Panel XIII includes a micrometer scale for the different objectives, magnifications and table positions used (each at 5 hundredths of a millimeter):

| | | | | |
|---|---|---|---|---|
| Fig. 6: | Homogeneous immersion, | Drawing table setting | 90, | Magnification A. |
| Fig. 7: | Homogeneous immersion, | Drawing table setting | 45, | Magnification B. |
| Fig. 8: | Homogeneous immersion, | Drawing table setting | 0, | Magnification C. |
| Fig. 9: | Apochromatic lens, | Drawing table setting | 90, | Magnification D. |

Magnification A was only used for Figure 1 Panel I, magnification D only for Figure 3 Panel X.

Ganglion cells and reliably stained ependymal cells are drawn in yellow, which corresponds to their appearance in the preparations, axis cylinders are only represented with yellow dots in some of the figures.

## Panel I.

Fig. 1: Astrocyte forms. Magnification A.

Fig. 2: Transverse section through spinal cord white matter. Lateral strands, peripheral region with pia. Magnification C.

Fig. 3: Oblique vertical section through spinal cord white matter. Magnification C. Right, pia, followed by cortex layer with bundle formation towards the pia. In the middle and left several trunk processes.

Fig. 4: Entry of the anterior root. Magnification C. Vertical section. On top anterior root, (a) peripheral layer of the anterior root, at the bottom several vessels.

Fig. 5: Substantia gelatinosa of Rolando. Magnification C.

## Panel II.

Fig. 1: Peripheral region of the anterior horn in a newborn infant. Magnification C. (a) A ganglion cell with Nissl granulations. Bottom, white matter.

Fig. 2: Peripheral region of the anterior horn in an adult. Magnification C. At the bottom, axis cylinders are marked in this case.

Fig. 3: Anterior portion of the substantia spongiosa of the dorsal horn. Magnification C. On the top, boundary with the substantia gelatinosa of Rolando.

Fig. 4: Lissauer marginal zone (bottom b) and substantia spongiosa (a) of the posterior horn. Magnification C.

## Panel III.

Fig. 1. Clarke column. Magnification C.

Fig. 2. Central canal with anterior commissure in a child. Magnification B. (axis cylinder outlined).

Fig. 3: Central canal with posterior commissure in a child. Magnification B. Bottom, vertical fibers (a).

Nuclei have not been included in this panel.

## Panel IV.

Fig. 1: Fetal ependymal epithelium with cilia. Underneath peripheral strip with dots. Magnification B.

Fig. 2: Central canal in older age. Magnification C. (a), (b) Two newly formed lumina, (c) irregular epithelial heap.

Fig. 3: The same. Magnification C. (a) Single new lumen.

Fig. 4: The same. Magnification C. Irregular epithelial heap.

Fig. 5: Irregular epithelial heap from the lower regions of the medulla oblongata. Magnification B. (a) Newly formed lumen, (b) irregular epithelial mass interspersed with neuroglial fibers.

## Panel V.

Fig. 1: Ependymal proliferation in the $4^{th}$ ventricle. Magnification B.

Fig. 2: Cyst-like space between two adjacent ependymal proliferations. Magnification B.

Fig. 3: Pyramidal junction Magnification C. (a) Compacted peripheral layers.

Fig. 4: Hypoglossus nucleus, more distal from the ependyma. Magnification B.

## Panel VI.

Fig. 1: Raphe of the medulla oblongata with adjacent tissue. Magnification C.

Fig. 2: Fibrae arciformes externae and pyramid nucleus. Magnification C.

Fig. 3: Olivary body. Magnification C. Bottom, myelinated layer.

Fig. 4: Nucleus gracilis. Magnification C.

## Panel VII.

Fig. 1: Ambiguous nucleus Magnification C.

Fig. 2: Striae acusticae. Magnification C. In a cyst-like space.

Fig. 3: Opticus, longitudinal section. Magnification C.

Fig. 4: Opticus, transverse section. Magnification C. Top, the compacted outer cortex layer. (a) Peripheral layer of a bundle.

## Panel VIII.

Fig. 1: Crus cerebri. Substantia nigra. Magnification C.

Fig. 2: Glial sheath surrounding a vessel region from the crus cerebri. Magnification C. Right, the vessel region. The vessel itself was not drawn.

Fig. 3: Oculomotor nucleus. Magnification C.

Fig. 4: Quadruplet bodies. Section of the inner region. Magnification C.

## Panel IX.

Fig. 1: Cerebrum cortex. Temporal lobe. Magnification C. (a) Cortex layer. (b) Radial fiber layer.

Fig. 2: White matter of the cerebrum. Magnification C.

Fig. 3: White matter of the cerebellum. Magnification C. In the middle, a vessel. At the bottom edge a vessel with transversely sectioned fibers.

Fig. 4: Purkinje cells from an old woman. Magnification B. Bottom, start of the granular layer.

Fig. 5: Superficial region of the molecular layer. Magnification B. Bottom, pia mater.

## Panel X.

Fig. 1: Ependymal surface of the hippocampus. Magnification C. Left ependymal layer, right radial fiber layer.

Fig. 2: Gyrus dentatus. Magnification C. Bottom pial surface. This figure is composed of two drawings, the top and bottom parts of the same image. The drawings fit together perfectly. Their boundary is marked in the figure by the constriction of the center.

Fig. 3: Base of the fimbria. Magnification D.

## Panel XI.

Fig. 1: Ventral (ependymal) surface of the column. Magnification C. Bottom, ependyma.

Fig. 2: Dorsal surface of the column. Magnification C. (a) Cortex layer. (b) Radial fiber layer.

Fig. 3: Deep myelinated layer of the column. Magnification C. The figure needs to be rotated by 90 degrees to correspond to the orientation of the other figures on this panel.

Fig. 4: Attachment of the fornix to the column. Magnification C. Bottom, fornix.

## Panel XII.

Fig. 1: Ganglion cell heap on dorsal surface of the column. Magnification C.

Fig. 2: Thalamus surface covered by plexus chorioideus. Magnification C. (a) Cortex layer. (b) Radial fiber layer.

Fig. 3: Thalamus surface covered with epithelium. Magnification C. (a) Ependymal layer. (b) Ganglion cell layer.

Fig. 4: From the depths of the pulvinar. Magnification C.

## Panel XIII.

Fig. 1: Fimbria with plexus chorioideus Magnification C. (a) Pial surface, (b) Ependyma. (c) Plexus chorioideus (d) Epithelial papillae.

Fig. 2: Hippocampus. 3 ½ x magnification. Explanation in text.

Fig. 3: Pineal gland. Magnification C.

Fig. 4: Quadruplet bodies. Actual size.

Fig. 5: Thalamus, posterior commissure, pineal gland. Actual size.

Fig. 6-9: Figure scales. Explanation on p. 146.

## Corrections[1].

Page 9 line 1 from bottom reads 1867 instead of 1877.

Page 22 line 3 from bottom reads 1882 instead of 1892.

Page 24 line 14 from bottom reads 1885-1886 instead of 1886- 1886.

Page 62 line 9 from top reads "darstellen" instead of "darsellen".

Page 63 line 1 from top,

Page 65 line 3, 6, 15 from top reads "Schaper" instead of "Schrader".

Page 78 line 5 from top reads "spärlicher" instead of "spärlich".

Page 89 line 12 from top reads "spärlicher" instead of "spärlich".

Page 89 line 15 from top insert (Panel I, Fig. 5) after "Rolando".

[1] All these points had already been corrected in the original German text, and therefore do not affect the English translation. The last correction was however added to the translation of the main text on p. 89.
