# Supplementary material for: The Concept of Neuroglia ‐ the State of the Art Circa 1900
Source: Glia. 2025 Feb 4;73(5):890–904. doi: 10.1002/glia.24678 (PMC11920685; doi:10.1002/glia.24678)
Supplement: Supplementary file 9 — Data S9. Scanned original publication by Held. [file GLIA-73-890-s011.pdf]

H. HELD ÜBER DEN BAU DER NEUROGLIA ...

ÜBER DEN BAU DER NEUROGLIA UND ÜBER DIE WAND DER LYMPHGEFÄSSE IN HAUT UND SCHLEIMHAUT

VON

HANS HELD.

Des XXVIII. Bandes der Abhandlungen der mathematisch-physischen Klasse
der Königl. Sächsischen Gesellschaft der Wissenschaften

Nº IV.

MIT 3 FIGUREN IM TEXT UND 4 LITHOGRAPHIERTEN TAFELN.

LEIPZIG
BEI B. G. TEUBNER.
1903.

Einzelpreis: 6 Mark 50 Pf.

ÜBER DEN BAU DER NEUROGLIA UND ÜBER DIE WAND DER LYMPHGEFÄSSE IN HAUT UND SCHLEIMHAUT

VON

HANS HELD.

Des XXVIII. Bandes der Abhandlungen der mathematisch-physischen Klasse der Königl. Sächsischen Gesellschaft der Wissenschaften

Nº IV.

MIT 3 FIGUREN IM TEXT UND 4 LITHOGRAPHIERTEN TAFELN.

LEIPZIG
BEI B. G. TEUBNER.
1903.

Vorgetragen für die Abhandlungen am 11. Januar 1903.
Das Manuskript eingeliefert am 18. März 1903.
Der letzte Bogen druckfertig erklärt am 14. Juni 1903.

ÜBER DEN BAU DER NEUROGLIA UND ÜBER DIE WAND DER LYMPHGEFÄSZE IN HAUT UND SCHLEIMHAUT

VON

HANS HELD.

I. Bau der Neuroglia.

Als *Elemente der Neuroglia* gelten seit VIRCHOW[1]) diejenigen Zellen der grauen und weißen Substanz, welche allgemein den *Nervenzellen und ihren Fortsätzen zwischengelagert sind und sie einhüllen.* Sie sind, wie heute wohl allgemeiner angenommen wird, ebenso wie diese *ectodermaler Natur* und werden ebenfalls wie sie im Wege der Entwicklung zu komplizierten *multipolaren Zellformen.* Sie unterscheiden sich aber einmal dadurch, daß alle ihre Fortsätze gleichartiger sind und zum Teil auch bleiben und nie in *Nervenfasern übergehen,* sondern zu einem Teil vielmehr besondere *Scheiden um dieselben, sowie um ihre Nervenzellen* ausprägen; daß sie *zweitens keine* NISSL-*Körper* in ihrem Protoplasma produzieren und anhäufen; daß sie *drittens* zum großen Teil einen *Filz* von besonders beschaffenen *Neurogliafasern* entstehen lassen, der einen gewissen festeren Halt für die nervösen Elemente des Zentralnervensystems abzugeben vermag; daß sie *viertens* endlich die *Bildung bestimmter Grenzhäute* ermöglichen, die für den völligen Abschluß des Zentralnervensystems im engsten Sinne des Wortes entscheidend ist.

Von diesen Eigenschaften der Neuroglia will ich hier näher und zuerst diejenige besprechen, welche das *Verhältnis der Neurogliafasern zu den Neurogliazellen* umfaßt und als ein noch strittiger Punkt in der heutigen Lehre vom inneren Bau der Neuroglia gilt, um dann in späteren Abschnitten außer anderem den *Begriff der gliösen Grenzmembran* zu entwickeln, der bisher für eine Lehre vom Abschluß der eigenen Masse des Zentralnervensystems gegenüber den es durchsetzenden Blutgefäßen bereits zum Teil bekannt geworden ist.

---

1. Neurogliazellen und Neurogliafasern.

Gegenüber der älteren von KÖLLIKER[2]), DEITERS[3]) und GOLGI[4]) aufgestellten Lehre, daß die Neurogliazellen als stern- oder spinnenförmig verzweigte Zellgebilde eine Menge von Ausläufern besitzen, die schließlich und bald faserdünn werden und sich zwischen den nervösen Elementen der grauen und weißen Substanz verschränken, ist von RANVIER[5]) und WEIGERT[6]) die Meinung aufgestellt worden, daß die Neurogliafasern keine Zellfortsätze sind, sondern von der Neurogliazelle gesonderte und zu ihr unabhängig gewordene Faserbildungen bedeuten. RANVIER ist durch eine besondere Isolationsmethode mit nachfolgender Picrocarminfärbung zu diesem Ergebnis gekommen; WEIGERT hat eine neue eigenartige Färbungsmethode von dünnen Schnitten hinzugefügt, welche am menschlichen Zentralnervensystem mit großer Schönheit diese besondere Faserung der Neuroglia und ihre verschiedene topographische Anordnung und Wertigkeit gezeigt hat. Auf Grund seiner differenzierten Färbung, die *außer den Gliafasern nur noch Kerne sichtbar* macht, kommt WEIGERT zur allgemeinen Ansicht, daß 1) die Neurogliafasern zwar mit den bisher so aufgefaßten Ausläufern der DEITERSschen Spinnenzellen oder der GOLGIschen Gliazellen identisch sind, aber 2) trotzdem keine eigentlichen Zellfortsätze bedeuten, weil sie von ihrem Protoplasma stofflich durchaus verschieden sind. Damit kommt dann WEIGERT zu dem besonderen Schluß, daß die bekannten Bilder von verästelten Neurogliazellen bei gewöhnlichen Färbungen und vor allem bei der GOLGIschen Silbermethode nur Trugbilder wären und also die Neuroglia morphologisch nach dem Typus der Bindesubstanz gebaut sei und zweierlei enthalte, Zellen und von ihr *total emanzipierte, rein zwischengefügte Faserbildungen*, die nur noch in „Kontiguität mit dem Zellleib“ ständen.

Ist wirklich diese WEIGERTsche Behauptung, daß die *Neurogliafasern* eine *Interzellularsubstanz* im Sinne von MAX SCHULTZE seien, richtig? Kann die WEIGERTsche Methode überhaupt beweisen, daß die Neurogliafasern eine derartig modifizierte Zellsubstanz vorstellen und daß sie also nicht mehr unmittelbar mit den Neurogliazellen zusammengehören?

Bei der WEIGERTschen Färbung ist außer den dunkelblau sichtbaren Neurogliafasern nur noch der Kern der Zelle gefärbt, während das Protoplasma total entfärbt und unsichtbar geworden. Eine derartige Restfärbung kann nach meiner Meinung wohl den Schluß zulassen, daß jene Fasern etwas besonderes in ihrer Masse bedeuten und auch verschieden vom übrigen Protoplasma sein können, aber nie eine Berechtigung geben, zu entscheiden, daß die einzelnen Gliafasern als echte Interzellularsubstanz auch wirklich außerhalb des Protoplasmas jener Zellen liegen, da ja von den Zellen selber bei derartigen Präparaten außer ihrem Kern nichts mehr zu sehen ist. Wie kann man also mit irgend welcher Sicherheit ausschliessen, daß die Gliafasern nicht im Protophasma drinliegen? Daß sie, wie die WEIGERTschen Bilder zuerst gezeigt, am Kern vorbei und unter sich übereinander gekreuzt verlaufen, hat doch für diese Frage nach einer interzellulären Lage gar keine Bedeutung. Hiermit stimme ich ähnlichen Ausführungen von LENHOSSÉKS bei.

Im Folgenden werde ich deshalb in der *Hauptsache zunächst untersuchen müssen*, ob die bei *starker oder reiner Differenzierung vom Gliazellenprotoplasma vollständig unabhängig erscheinenden Gliafasern auch wirklich interzellulär* liegen, wie WEIGERT aus einem an und für sich unzureichenden Grunde geschlossen hat, oder noch irgendwie *innerhalb jener Zellen*. Vorausschicken will ich kurze Betrachtungen über das, was inzwischen für oder gegen die RANVIER-WEIGERTschen Behauptungen angegeben worden ist.

Zuerst hat PELLIZZI[7]) gegen WEIGERT gesagt, daß nur seine Methode zellenabhängige Gliafasern zeige; in Wirklichkeit gingen sie aus dem Zellkörper hervor. Dann hat v. KÖLLIKER[8]) sich gegen die neu-alte RANVIER-WEIGERTsche Ansicht von der Neuroglia ausgesprochen. Von seiner Beobachtung ausgehend, daß sehr viele GOLGIsche Gliazellen wie aus zwei Teilen bestehen, einem Zellenkörper und einer demselben einseitig ansitzenden Platte, von welcher die Ausläufer abgehen, stellt er hier zur Erklärung jener Ansicht die Hypothese auf, „daß die GOLGIschen Zellen aus einem Teile ihres Protoplasma *einseitig* eine mit Ausläufern versehene Platte erzeugen, welche anfänglich und so lange die Ausläufer noch sich verlängern mit dem kernhaltigen Teile des Zellprotoplasma innig und unmittelbar zusammenhängt, später jedoch in vielen Fällen eine andere Dichtigkeit und vielleicht auch eine etwas abweichende chemische Konstitution gewinnt und von diesem Zeitpunkte an unter gewissen Umständen von dem Zellen-

körper sich trennen läßt". Daß diese Hypothese nicht richtig ist, werde ich weiter unten zeigen. Dann hat REINKE[9]) ausgeführt, daß die Neuroglia der weißen Substanz des Rückenmarks aus zahlreich verästelten Zellen bestände, deren Ausläufer zum Teil rein protoplasmatisch sind. Als Produkte der Gliazellen müssen die Gliafasern gelten; sie liegen aber nur zum Teil in ihrem Protoplasma, während andere nur noch äußerlich anliegen und eine dritte Art ganz emanzipiert geworden ist. Protoplasmafortsätze und Neurogliafibrillen sollen verschiedene ja entgegengesetzte Richtung haben. Zur gleichen Zeit habe ich[10]) gezeigt, daß nur auf total differenzierten Präparaten die WEIGERTschen Gliafasern selbstständig erschienen. Bei Anwendung andersartiger Methoden, die beides, Fasern und Gliazellenprotoplasma zugleich und verschieden gefärbt erkennen ließen, seien die Gliafasern durchaus noch mit dem Protoplasma bestimmter Zellen verbunden. Deshalb wären sie als intrazelluläre Bildungen zu betrachten, weil sie z. T. aus spongioplasmatischen Zellfäden hervorgingen, die sich zwar weiterhin zu stärkeren und auch freieren Fasern anscheinend vereinigen könnten, aber nicht den Zusammenhang mit ihren Zellen verlören. Weiter hat ROBERTSON[11]) gegen die WEIGERTsche Ansicht einen rein zelligen Bau der Neuroglia betont. Nach ihm sind wohl im Sinne der v. KÖLLIKERschen Hypothese von einer ausläuferreichen Zellplatte der GOLGIschen Gliazellen die Zellkörper der Gliazellen mit einer dünnen, leicht schrumpfenden und besonders färbbaren Membran versehen, die mit den anscheinend selbständig an der Zelle vorbeiziehenden Gliafasern aber noch verbunden ist. Auch nach v. GEHUCHTEN sind die WEIGERTschen Bilder so zu erklären, daß die Gliafasern nur als chemisch differente Zellausläufer allein gefärbt bleiben, während sie in Wirklichkeit mit dem Zellkörper fest verbunden sind. Auch EURICH[12]) gibt von den Neurogliafasern an, daß sie keineswegs alle von den Zellen unabhängig sind.

WHITWELL[13]) dagegen behauptet im Sinne WEIGERTS, daß die Neurogliafibrillen keine Zellfortsätze sind, sondern ein von ihnen unabhängiges Flechtwerk bilden, in dessen Maschen von zugleich lymphatischer Funktion Neurogliazellen und Nervenzellen und Blutgefässe eingefügt sind und wie der Inhalt eines Korbes getragen werden. Seine ferneren Angaben sind, daß diese Fibrillen stark lichtbrechend sind, nicht aus Neurokeratin noch Elastin bestehen, aber beträchtliche elastische Eigenschaften haben, was

letzteres aus ihren Krümmungen unter gewissen Bedingungen hervorgehen soll.

Die folgenden Arbeiten sprechen zum größten Teil wiederum gegen RANVIER-WEIGERT. Unter diesen gehen die Untersuchungen von BRODMANN[14]) und YAMAGIVA[15]) von der Ausbildung der Astrocyten in Gliomen aus. BRODMANN kommt allerdings zu dem Resultat, daß seine histologischen Befunde „eine Hauptstütze für die von WEIGERT begründete neue Lehre von der normalen menschlichen Neuroglia" bilden. An und für sich zeigen aber seine Befunde Zellen mit strahlig verzweigten und konisch aus dem Zellprotoplasma entspringenden Fortsätzen, deren „Ränder jedoch dunkler tingiert" sind. Peripheriewärts gehen dann derartig konturierte Zellausläufer in eine oder mehrere Fasern über, die Gliafasern vorstellen und nicht mehr unter einander anastomosieren. Nur besondere, breite und fibrillenstreifige Fortsätze gehen zur „perivaskulären Gliascheide". Bei diesem Befund kommt aber BRODMANN auf dem Wege einer komplizierteren Schlußfolgerung zu dem obigen Resultat, weil er erstens meint, daß im normalen Gliagewebe die Gliafasern keine Zellbeziehungen mehr haben können, da die WEIGERTsche Methodik nur im pathologischen Material derartige Bilder von faserdifferenzierten Astrocyten zeige, und weil er sich zweitens die Vorstellung gebildet hat, daß die Astrocyten als Bildungszellen der Neurogliafasern „verschwinden", sobald der „Prozeß der Fibrillation" zu Ende ist.

YAMAGIVA hat mit einer eigenen Methode ebenfalls ein Thalamusgliom untersucht und danach auch normales Gehirn berücksichtigt. Seine Methode zeigt außer anderem eine „gleichzeitige Kontrastfärbung der Gliafasern (rot) und des Protoplasmas der Gliazellen (schwachviolett)". Er sieht „feine, rote Punkte als Querschnitte der feinen Gliafasern in der peripherischen Schicht des Zellleibes" und „Zellkomplexe, welche verschiedentlich von den Fasern begrenzt sind" und kommt zu dem einfachen Resultat, „daß die *Gliafasern differenzierte Interzellularsubstanz* darstellen, welche aber *nicht ganz oder nicht überall von den Zellen getrennt*" sei. Er kommt also damit zu einem Resultat, welches schon früher STRÖBE[16]) so formuliert hatte, daß ein *„organischer Zusammenhang" zwischen dem Zellleib und den Gliafasern* vorhanden wäre.

Umfangreicher hat STORCH[17]) die pathologischen Vorgänge am Gewebe der Neuroglia beobachtet; er meint, daß die Glia-

fasern unabhängige Interzellularsubstanz seien und will nur für die Monstrezellen gelten lassen, daß um den Kern herum eine deutliche oder größere Protoplasmamasse angehäuft sei, die in faserhaltige Ausläufer gegliedert ist und auch sonst im Zellleib mit passierenden Gliafasern verbunden ist. Gewisse Rückbildungsvorgänge an diesen krankhaft geschwollenen Gliazellen sollen dann erst zu jener Form führen, die KÖLLIKER für normale Gliazellen gehalten und an denen er seine Grundlage für die Aufstellung jener Ansicht über die Entstehung der Gliafasern aus einer Zellplatte gefunden habe. Auf einige andere Angaben STORCHS, die zum Teil auch noch die Gefäßbegrenzung durch Gliafasern betreffen, werde ich weiter unten zurückkommen.

In ausführlicheren und tiefgehenden Untersuchungen hat dann E. MÜLLER[18]) von dem Neurogliagewebe der niederen Wirbeltiere nachgewiesen, daß die Gliafasern „sowohl morphologisch wie physikalisch-chemisch als Differenzierungsprodukte der Gliazellen“ zu betrachten sind, daß aber noch zum Unterschied von den WEIGERTschen Behauptungen, *Anfänge wie Enden von Gliafasern* da sind; und zwar finden sich letztere an den Gefäßen und an einer subpialen Schicht, erstere in den Gliazellen, aus denen sie teils einzeln, zum Teil auch als eine Summe einzelner konvergierender Fäden in meinem früheren Sinne hervorgehen und auslaufen. Im übrigen meint E. MÜLLER, daß „die Neuroglia einen Übergang zwischen dem rein epithelialen Gewebe und dem Bindegewebe“ bilde, insofern sich eine Anzahl von Zellen aus dem primären Ependym loslösen und zu relativ freien Gliazellen werden, welche dann ebenso wie jene Stützfasern produzieren, die aber zum *Unterschied von denen des Bindegewebes nicht selbständig werden, sondern mit ihren Zellen verbunden bleiben.*

Von den neuesten Arbeiten über die Neuroglia kommen dann noch für unsere Frage nach dem Verhältnis zwischen Neurogliafasern und Neurogliazellen hauptsächlich die Untersuchungen von STUDNIČKA[19]), OBERSTEINER[20]), MARINESCO[21]), DIMITROVA[22]), HUBER[23]) und HARDESTY[24]) in Betracht.

Die Beobachtungen von STUDNIČKA zeigen einen Zusammenhang der gliösen Ependymfasern mit dem Protoplasma der Ependymzellen und zwar auch am menschlichen Nervensystem, wo WEIGERT einen solchen Zusammenhang nicht hatte beobachten können.

Ebenso spricht sich OBERSTEINER für die Gliazellen an der Oberflächenzone der grauen Großhirnrinde *gegen* die WEIGERTsche Lehre aus. Auch in den Gliaelementen der Zirbeldrüse findet DIMITROVA dieselben vielfach nur als *differenzierte Zellfortsätze*, während *andere frei oder den Zellen nur äußerlich angelagert* sein können.

MARINESCO und HUBER stehen dagegen mehr auf dem Boden der RANVIER-WEIGERTschen Lehre. Vor allem betont ersterer, daß die *faserige Glia unabhängig von der zelligen Glia sei*, wenn er auch zugibt, daß *es außerdem protoplasmatische Fortsätze der Neurogliazellen* gebe, welche *in der Gefäßwand* endigten und besonders erst in *pathologischen Prozessen* der Glia deutlicher würden.

HUBER rechnet ebenfalls die Gliafasern zu den Interzellularsubstanzen aber aus *dem Grunde*, weil die *größte Anzahl derselben keine gleichmäßigen Beziehungen zur Masse der Gliazellen* habe, hebt aber außerdem hervor, daß es bei gleichzeitiger Beobachtung und Färbung des Gliazellenprotoplasmas Zellen gäbe, die protoplasmatische Fortsätze erkennen ließen, *von denen die Gliafasern nicht vollständig getrennt wären*, und mit deren *Seitenflächen sie irgendwie kontinuierlich verbunden* wären. HUBER hat bei Hund, Katze, Kaninchen, Taube, Schildkröte und Frosch die allgemeinen Verhältnisse von Gliafasern und Gliazellen studiert. Die chemische, d. h. in färberischer Differenzierung sich geltend machende Unabhängigkeit der Faserung ist nach ihm bei diesen verschiedenen Tieren ungleich; am stärksten ist sie bei Hund, Katze und Schildkröte, am wenigsten bei der Taube ausgeprägt.

Fasse ich kurz die *Resultate der obigen Literatur über den Bau der Neuroglia* zusammen, so erscheint als ein unbestrittenes Verdienst der RANVIER-WEIGERTschen Untersuchungen über die Elemente dieses zentralen Stützgewebes die Lehre, daß die *Neurogliafasern ihrer chemisch-physikalischen Natur nach besondere Faserbildungen* im Gewebe der Neuroglia bedeuten; die *andere Behauptung* aber, daß die *gliöse Fasermasse* eine reine und von den Gliazellen *unabhängige Interzellularsubstanz* sei, die nur lose dem Protoplasma jener Zellen „per contiguitatem“ anliege, erscheint dagegen bereits unrichtig.

Zur weiteren Begründung aller jener Beobachtungen, die dieser letzteren Ansicht von einer Interzellularsubstanz in der Neuroglia entgegenstehen, und zur Fortsetzung jener früher von

mir kurz erhobenen Einwände, komme ich also hier auf meine obige Fragestellung zurück, um zu untersuchen, ob, wie WEIGERT *speziell für die Neuroglia des Menschen* angegeben, die *Gliafasern auch wirklich ausschließlich interzellulär liegen, oder ob sie noch irgend eine Verbindung mit dem Gliazellenprotoplasma haben.*

Eigene Beobachtungen und eine allgemeine Einteilung der Neuroglia.

Meine folgenden Angaben betreffen in der Hauptsache das zentrale Nervensystem des Menschen, das ich am neugeborenen und einige Monate alten Kinde sowie am Erwachsenen von Anfang und Mitte der zwanziger Jahre untersucht habe. Daneben habe ich noch Kaninchen und Hund und stellenweise das Rückenmark von Kalb und Rind berücksichtigt. Bei der Erklärung der einzelnen Figuren auf Tafel I—IV habe ich zu ihrer besonderen Begründung kurze methodische Angaben gemacht. Sonst gehe ich aber an dieser Stelle auf die Methodik der Untersuchung der Neuroglia nicht ein, weil ich noch nicht über eine glatte und überall ausreichende Färbung der Neuroglia verfüge, die vor allem als eine spezifische Färbung für diese zentrale Stützsubstanz sicher wäre. Immerhin habe ich meine abschließenden Beobachtungen aus solchen Stellen meiner Schnitte gezogen, die irgend eine Verwechselung von Gliafasern mit Nervenfasern infolge von verschieden großer Entfärbung beider Elemente oder auch totaler Entfärbung der Achsencylinder verhindern konnten. Auf die architektonische Gliederung der gliösen Stützsubstanz werde ich ebenfalls bei der Schilderung meiner Befunde im folgenden nicht eingehen, da ich hier nur das elementare Verhältnis von Zelle und Faser zu berücksichtigen habe. Erst im dritten Abschnitt, wo ich auf die *marginale Neuroglia* näher eingehe, werde ich einige der WEIGERTschen Angaben über die Topographie der Neurogliafaserung zu kritisieren haben.

Um eine handliche Gliederung für die Besprechung der an den verschiedenen Regionen des Zentralnervensystems gemachten Beobachtungen zu haben, will ich kurz eine solche hier entwickeln, die zugleich eine *allgemeine Einteilung der Neuroglia* erlaubt. Ihre Begründung wird aus den folgenden Abschnitten klar werden; sie stützt sich auf das regionäre und zugleich bestimmte Ver-

hältnis von Neurogliazellen und ihren Gliafasern als einem genetischen und dauernden Anteil an der allgemeinen Masse der Neurogliazellen selber.

Ich unterscheide zunächst eine *Substantia Neurogliae centralis.* Sie entspricht dem, was bisher als Substantia gelatinosa centralis bezeichnet worden ist. Seitdem durch WEIGERT für die Zone um den Zentralkanal des Rückenmarks resp. für diejenigen an den Wandungen der Gehirnhöhlen der außerordentliche Reichtum an Neurogliafasern bekannt geworden ist, geht es nicht mehr an, jenen früheren Namen zu gebrauchen, der aus einer mangelnden Erkennung der elementaren Zusammensetzung dieser Gegend stammt und außerdem noch für eine andere Gegend, die substantia gelatinosa des Hinterhirns angewandt wird, die gerade umgekehrt, wie WEIGERT erkannt, sehr arm an Gliafasern ist. Ich schlage deshalb die Bezeichnung Substantia Neurogliae centralis für jenen obigen Abschnitt von Gehirn und Rückenmark vor. Unterscheiden will ich ferner an ihr *zwei Anteile*, die *ependymäre Neuroglia*, welche von den Ependymzellen selber und ihren gliafaserhaltigen Fortsätzen gebildet erscheint, und die *subependymäre Neuroglia*, die von einer mehr zerstreuten Masse allseitig verzweigter Neurogliazellen und ihrer Fasermenge zusammengesetzt ist. Im weiteren Umkreis würde die subependymäre Neuroglia in *die Summe von Gliazellen* sich fortsetzen, welche *im allgemeinen in weiter und also diffuser Anordnung bereits den nervösen Elementen, den Nervenfasern resp. den Nervenzellen zwischengefügt* ist, sonst aber in örtlich wechselnder Weise eine verschiedene und besonders angepaßte Formung ihrer stützenden Teile zu jenen nervösen Zellen und ihren Fortsätzen zeigt. Ich will sie als *diffuse Neuroglia* bezeichnen; sie gliedert sich allgemein in eine solche der *grauen* und in eine solche der *weißen Substanz*. Zu *trennen* bleibt endlich eine *Masse von Gliafasern*, die weniger scharf auch einer bestimmten Zone von Gliazellen entspricht, insofern sie sowohl von den Zellen der diffusen als auch der subependymären und ependymären Glia geliefert wird; sie ist durch ihre *engen und wichtigen Beziehungen zur äußeren Oberfläche* des Zentralnervensystems oder zu den *inneren Blutwegen* desselben charakterisiert. Ich will sie als *marginale Neuroglia* bezeichnen. Wie ich im dritten Abschnitt auseinandersetzen werde, ist sie es, die in *besonderer Weise die HISschen Räume des Gehirns der Eigenmasse des Zentralnervensystems*

gegenüber abschließt und natürlich begrenzt; sie liegt dementsprechend teils an der freien, teils an der inneren Oberfläche des Gehirns und bestimmt somit entweder den *Epicerebralraum* resp. die *perivaskulären Räume*.

Nach dieser Einteilung der Neuroglia habe ich auf die elementare Zusammensetzung derselben aus Zellen und Fasern in der obigen Fragestellung einzugehen.

a) Substantia Neurogliae centralis.

Die die Substantia Neurogliae centralis der Ventrikelhöhle oder dem Zentralkanal zu begrenzenden *Ependymzellen* sind *ungefähr epithelartig* gestellt. Ein erheblicherer Unterschied besteht nur insofern, als durch späte postembryonale Verschiebung der einzelnen Zellelemente bei einzelnen Tieren und insbesondere beim Menschen der reine Charakter einer geschlossenen, flächenhaft verklebten Reihe länglicher Zellen, wie er z. B. beim erwachsenen Kalb und Rind zu beobachten ist, verloren oder verändert werden kann. Beim neugeborenen und einige Monate alten Kind, das ich habe untersuchen können, sind noch reine Epithelauskleidungen zu beobachten. Beim 21 Jahre alten Hingerichteten dagegen ist bereits durch Proliferation der Ependymzellen an der dorsalen und ventralen Ecke des Zentralkanales eine Vermehrung und eine entsprechende periphere Verschiebung und Formänderung der einzelnen Zellen zu unregelmäßig eckigen Gebilden eingetreten. Bei einem 24 Jahre alten Hingerichteten habe ich bereits den ganzen Zentralkanal obliteriert und von faserreichen Gliazellen ausgefüllt gefunden, was auch noch bei einem zweiten, wenige Jahre älteren Mann der Fall war. Die von WEIGERT auf Tafel IV, Fig. 4 abgebildeten Verhältnisse, die auch nach ihm zu den natürlichen Altersveränderungen gehören, habe ich hier wiedergefunden. Beim erwachsenen Kaninchen (Fig. 1 auf Tafel 1) und beim Hund sind dagegen Verhältnisse ausgeprägt, die eine erheblichere Asymmetrie und gegenseitige Entfernung der einzelnen Zellelemente sowohl in ihrer seitlichen Stellung als auch in ihrer Tiefenausdehnung von einander zeigen. Ein gleiches fand sich am Ependym des IV. Ventrikels vom Hingerichteten (Mitte der 20er Jahre), sodaß ich also die umfangreichen Angaben von STUDNIČKA bestätigen kann, die als Eigentümlichkeiten derartiger Ependymzellen gegenseitige seitliche Anastomosen rein protoplasmatischer Natur zeigen. Eine zweite Frage, auf die ich hier noch kurz eingehen will, ist kürzlich von FUCHS[25]) wieder hervorgeholt worden, der das innere Ende der Ependymzellen auf Basalkörperchen und Flimmerhaare bei Embryonen von Meerschwein, Schwein, Maulwurf, Kaninchen, Ratte sowie von jungen und alten Katzen untersucht hat und jene Charakteristika echter Flimmerzellen hier vermißt hat. Gegen die FUCHSsche Behauptung sprechen die bekannten älteren Angaben von der am frischen Ependym sichtbaren Flimmerung (s. KÖLLIKER, Gewebelehre II. 1896. S. 144). Was speziell das Vorkommen von Basalkörperchen an den Flimmerhaaren anbetrifft, so hat das bekanntlich zuerst WEIGERT (1890) für den Menschen und zwar am embryonalen Ependym behauptet gehabt und später hinzugefügt, daß er sie am neugeborenen und einige Monate alten Kinde dagegen nicht mehr gesehen habe. Daß sie aber auch beim erwachsenen Mensch vorkommen, hat STUDNIČKA vom Ependym des IV. Ventrikels angegeben. Im übrigen hat bereits in Halle BENDA hervorgehoben, daß er an den Ependymzellen des Seitenventrikels überall die für Flimmerepithel charakteristischen Basalkörperchen gefunden habe. Ich will hier hinzufügen, daß ich selber *stellenweise oder umfangreicher* am Boden des IV. Ventrikels beim reifen Katzenfötus, der neugeborenen Katze, beim 4 Wochen alten Hund, ferner im Zentralkanal und der Rautengrube vom neugeborenen Kind, dem erwachsenen Kaninchen und Hund deutliche Basalkörperchen und steife Flimmerhaare habe beobachten können. Ich rechne sonach ebenfalls das Ependym wenigstens teilweise zum Flimmerepithel, wenn ich auch nicht hier entscheiden will, ob regelmäßig und immer eine konstante Ausbildung des Flimmerbesatzes eintritt oder mitunter ausbleibt.

Nach STUDNIČKA soll eine normale Flimmerung nicht überall bestehen, sondern auch eine mit eigentümlichen starren Stiftchen versehene Zellformation eintreten können. Im Centralkanal des Kaninchens finde ich überall Flimmerhaare und Basalkörperchen, beim Hund vereinzelte Ependymzellen, die das nicht mehr zeigen; beim einige Monate alten Kinde habe ich dagegen umgekehrt nur vereinzelte Flimmerzellen mit deutlichen Zeichen gefunden. Das spräche für eine *partielle Ausbildung des Ependyms zum Flimmerepithel.*

Auch am erwachsenen Ependym zeigen nun die einzelnen *Ependymzellen periphere* und für den Zentralkanal oder auch den

Aquaeductus Sylvii ziemlich rein radiär angeordnete *protoplasmatische Fortsätze*, die denjenigen entsprechen dürften, welche durch die GOLGIsche Methode im embryonalen Gehirnrohr bekannt geworden sind. Nur die der vorderen Rückenmarksspalte, dem Septum posterius oder der Raphe des Hirnstammes zustrebenden Fortsätze der Bündel von solchen sind durch eine geringe bogenförmige Zusammendrängung ein wenig vom rein radiären Verlauf auf dem Querschnitt abgelenkt.

Beim Kaninchenrückenmark sind diese beiden Anteile aus sehr groben Fortsätzen zusammengesetzt. Beim Hund sind sie schon weniger stark. Bei Kalb und Rind finde ich dieselben dagegen gleichmäßiger aus mehreren feineren Fortsätzen gebildet, sodaß sie sich auch wenig von der rein seitlichen und zur grauen Substanz beiderseits gekehrten Gruppe von Ausläufern durch Kaliber unterscheiden. Beim Menschen und auch bei der Katze sind wiederum Verhältnisse, die an diejenigen des Kaninchens erinnern, wenn sie auch nicht so einseitig stark entwickelt worden sind. Für den Boden des IV. Ventrikels will ich hinzufügen, daß auch der „Ependymkeil der Raphe" am erwachsenen Nervensystem von stärkeren und bogenförmig zum dorsalen Anfang der Raphe orientierten Fortsätzen der angrenzenden Gruppe von Ependymzellen zusammengesetzt ist. Für die seitlichen Abschnitte finde ich wiederum feinere Fortsätze, die entweder in Einzahl aus jeder Zelle hervorgehen, um sich dann öfters zu teilen, oder gleich zu mehreren entstehen, wie es für den Menschen bereits STUDNIČKA auf Tafel 32 und 33, Fig. 5—10 seiner Abhandlung (19) abgebildet hat.

Alle die peripheren Fortsätze derartiger Ependymzellen des Zentralkanals oder der Rautengrube sind weiter durch *feinere oder gröbere Seitenzweige verästelt*, welche innerhalb der Substantia neurogliae centralis ein zierliches und bald feiner oder gröber gewebtes Maschenwerk bilden. Besonders grob und deutlich maschig ist es unmittelbar unter dem Ependym des IV. Ventrikels zu beobachten, während es in der gleichen Substanz des Rückenmarks feiner zusammengesetzt ist. Sonst ist aber die *Maschengröße an denselben Stellen durchaus abhängig von der Art des Fixierungsmittels und der Einbettungsmethode;* sie fällt geringer aus bei der MÜLLERschen Lösung und nachfolgender Celloïdeinbettung, größer bei Paraffinschnitten derselben Fixierung. Bei Salpetersäurekonservierung ist

die Dicke der Balken gleichmäßiger, während sie bei Alkohol oder Pikrinschwefelsäure feiner und zarter resp. zerrissener erscheinen. Ich vermag deshalb ein wirkliches Kriterium über eine dem *natürlichen Zustand entsprechende Weite dieser Formation* nicht anzugeben. Über die Art dieser Maschung werde ich weiter unten erst einiges anzugeben haben.

Die *peripheren Fortsätze* der Ependymzellen sind nun zum Teil reich an inneren Fasern, welche dieselben aussteifen, wie es im Rückenmark der Fall ist; andere sind jedoch rein protoplasmatisch, wie die meisten in den seitlichen Abschnitten des Ependyms im IV. Ventrikel. Im Zentralkanalependym von Rind, Kalb, Hund und Mensch ist mehr im ganzen Umkreis eine ungleiche Ausbildung von solchen feineren Stützfasern vorhanden. Statt einzelner Fasern können auch *ganze Bündel von Fasern* gebildet sein, welche dann im *inneren Abschnitt des Zelleibs kelchartig den Kern der Zelle umgeben.* Bei den genannten Tieren sind solche *Bündel* jedoch im allgemeinen nur spärlich ausgeprägt und im wesentlichen auf jene *Rapheanteile der Ependymfortsätze* resp. auf *diejenigen der reinen dorsalen oder ventralen Radiärfortsätze des Rückenmarkependyms* beschränkt. Beim Menschen sind bald die dorsalen, bald die ventralen Bündel stärker ausgeprägt, was ich auch mitunter bei der Katze und bei dem Hund gesehen; am wenigsten habe ich sie bei Kalb und Rind ausgebildet gefunden.

Beim Kaninchen dagegen sind zum Unterschied hiervon allgemein die peripheren Fortsätze des Zentralkanalependyms reich an Bündeln von Fasern (Fig. 1), besonders sind die zur vorderen Spalte strebenden übermächtig entwickelt. Entsprechend stark ist natürlich dann der intrazelluläre Anfang dieser Bündel in Form eines *perinukleären Kelches* ausgeprägt (Fig. 2). Bis zur Kernzone lassen sich aber auch zum Teil die *Einzelfasern* vom Fortsatz her verfolgen, wobei sie mitunter *außerordentlich eng der Wand anliegen* oder auch als *Membranversteifungen* selbst erscheinen können. Stellenweise reichen sie aber auch nur bis zum Abgang des peripheren Fortsatzes, oder hören auch schon in einiger Entfernung vorher als deutlich gefärbte Fasern auf, sodaß, wie es mitunter z. B. bei den seitlichen Abschnitten des Ependyms am IV. Ventrikel zu sehen, das Anfangsstück des peripheren Fortsatzes faserfrei sein kann. Vielleicht ist hierbei ein Differenzierungsfehler der Methode beteiligt. Handelt es sich um eine geringere Anzahl

von 2—4 Einzelfasern, so finde ich dieselben ungleichmäßig im Zellumfang verteilt, sodaß sie also auch mitunter schon im Beginn ihrer deutlichen und dunklen Tingierung zu einem dünnen Bündel zusammenliegen. Im übrigen erscheinen auf meinen Präparaten die Anfänge aller dieser intrazellulären Fasern, die ich zweckmäßig als *ependymäre Gliafasern* bezeichnen will, als matter gefärbte Spitzen, die ich aber nie bis zur Höhe der Basalkörperchen habe verfolgen können. Nur im *Ependym von Petromyzon* habe ich fast durchweg die in den peripheren Fortsätzen vorhandenen *Einzelgliafasern in einen inneren Faserkegel verfolgen können,* welcher nach dem Modus der von APATHY bei der Flimmerzelle nachgewiesenenen intrazellulären *Faserstruktur* bis zur Basalkörpergruppe reicht. Von APATHY ist dieser Faserkegel der Flimmerzellen für eine der Nervenleitung dienende Zellstruktur erklärt worden. Bei Lumbricus terr. habe ich umfangreicher im Flimmerepithel derartige Fasern gefärbt erhalten; da ich bisher sie nie in „Neurofibrillen“ habe sich fortsetzen gesehen, sondern immer am Boden der Flimmerzelle aufhörend gefunden, möchte ich sie nur für einen inneren Stützapparat der Flimmerzelle ansprechen. Ob er mit dem von APATHY beschriebenen System identisch ist, kann ich nicht angeben. Auf meinen Präparaten ist das untere Ende der Hauptfaser nicht zugespitzt, mitunter sogar ein wenig verbreitert. Auch erscheint am anderen Ende der Kegel feinerer Fibrillen nicht so gleichmäßig.

Die *ependymären Gliafasern* setzen sich nun im allgemeinen und in der Hauptsache zunächst in den *peripheren Ausläufern* der Ependymzellen fort; einzelne jedoch gelangen, was besonders bei den faserbündelhaltigen Exemplaren zu beobachten ist, in die *Seitenzweige* ein (Fig. 2), wobei sie auf Querschnitten durch Rückenmark und Hirnstamm sehr bald in eine *Längsrichtung* umbiegen, sodaß sie der Verfolgung ihrer weiteren Richtung sich entziehen. Zum Teil biegen aber auch die peripheren Hauptfortsätze aus einer anfänglich queren in eine Längsrichtung um, und mit ihnen natürlich auch ihre Gliafasern.

Auf diese Weise entsteht wenigstens zu einem großen Teil jenes *Lager von Gliafasern,* welches auf Rückenmarksquerschnitten dicht unterhalb der eigentlichen Ependymzellen in je einem halben Umkreis jene quergetroffene Faserzone bildet, welche bei Kaninchen nur schwach entwickelt, bei Hund, Kalb, Rind und Mensch da-

gegen zu einer stärkeren und charakteristischen Masse der Substantia Neurogliae centralis geworden ist. Ein Gleiches läßt sich für die ganzen seitlichen Bezirke des Ependyms der Rautengrube behaupten. Beim neugeborenen und einige Monate alten Kinde ist dieses Lager von Fasern nur schwach entwickelt, während es bei dem Rückenmark aus den zwanziger Jahren schon sehr erheblich geworden ist. Ich kann also hiermit die WEIGERTsche Behauptung, daß „bei älteren Individuen immer mehr und mehr vertikale Fasern auftreten“, bestätigen. Daß zu dieser Fasermasse eine solche hinzukommt, welche von gewissen Gliazellen der nächsten Umgebung geliefert wird, soll weiter unten gezeigt werden.

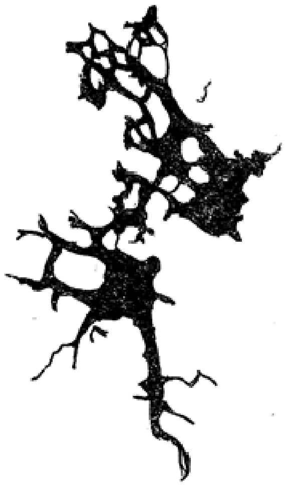

Fig. 20a. Aus dem Rückenmarksquerschnitt eines wenige Wochen alten Hundes. Silbermethode nach GOLGI. Zwei Gliazellen der weißen Substanz des Vorderstranges sind in netzförmigem Zusammenhang, der im Niveauunterschied von 60 μ erfolgt, imprägniert worden. Es soll diese Abbildung nachweisen, daß die GOLGIsche Methode keineswegs immer frei verästelte Gliazellen wie auf den folgenden Figuren 20 b und c (S. 293—294) demonstriert; woraus hervorgeht, daß sie für die Lehre vom Neurogliafilz keine eindeutigen Resultate gibt.

Zwischen diese Fasermassen der Substantia Neurogliae centralis dringen nun jene peripheren Hauptfortsätze der Ependymzellen ein, indem sie sie entsprechend ihrer Zahl und Anordnung septieren, wobei ein Teil derselben in seinem weiteren Verlauf in die *äußeren Abschnitte* der *Substantia Neurogliae centralis* eindringt, welche bereits durch zahlreichere *Gliazellen* und ringförmig oder wellenartig angeordnete Gliafasern charakterisiert wird und außerdem schon durch die Nervenfaserzüge der Kommissuren beherrscht erscheint. Hierbei sind jedoch die meisten jener ependymären Gliafasern resp. Bündel von solchen auf Querschnitten schräg abgeschnitten, dementsprechend, daß ein rein querradiärer Verlauf jener Fortsätze der Ependymzellen nicht mehr im ausgewachsenen Nervensystem vorhanden ist. Infolgedessen gelingt es nicht mehr, wie es z. B. E. MÜLLER bei Amphioxus oder Myxine gelungen ist darzustellen, den Verlauf der ependymären Gliafaserung bis zur Rückenmarksoberfläche oder bis zur ventralen Hirnstammfläche direkt nachzuweisen. Hinzukommt, daß im Rückenmark sehr bald die seitlichen Ependymzellenfortsätze sehr

fein werden, sodaß sie nicht mehr im Gewirr der übrigen Gliafasern verfolgt werden können. Ob außerdem, wie vielfach behauptet worden, auch infolge einer späteren *Reduktion* von ursprünglich durchreichenden Fortsätzen solches am ausgewachsenen Rückenmark unmöglich ist, kann ich nicht mit Sicherheit entscheiden. *Ein Teil endet* jedenfalls nicht an der Oberfläche, sondern an den *Gefäßbäumen in der nächsten oder weiteren Umgebung des Zentralkanals resp. der angrenzenden grauen Substanz* (Fig. 39a, b, c). Von einem *andern Teil* aber kann ich behaupten, daß er noch bis zur *Oberfläche* sich hin erstreckt. Es ist dies der aus starken Gliabündeln gebildete Zug, welcher zur vorderen Spalte des Rückenmarks geht und z. B. beim Kaninchen fast noch in rein querem Verlauf hingelangt, um an der in der Tiefe derselben befindlichen Membrana limitans gliae superficialis (siehe weiter unten) zu inserieren. Auch jener zweite Zug, der schon in mehr schräg abgeschnittenen Bündeln *die gleiche* Grenzhaut des Septum posterius erreicht, gehört noch einem durchreichenden Stützsystem an. Bei den übrigen von mir untersuchten Säugetieren ist der rein radiäre Verlauf viel weniger vorhanden; doch lassen sich solche Richtungen und solcher Endverlauf auch hier und auch beim Menschen aus jenen stärkeren Bündeln erschließen, welche wie beim Kaninchen als schräg abgeschnittene, in Wirklichkeit also in der vertikalen Richtung wellenförmig verlaufende Züge sich aus dem übrigen Gewirr der Gliafaserung der Substantia centralis deutlicher abheben. Auch für die Raphe des Hirnstammes möchte ich ein Gleiches behaupten, wobei ich mich auf den ähnlichen Verlauf und die besondere Stärke der entsprechenden Fortsätze der der Raphe unmittelbar angrenzenden Lager von höheren Ependymzellen stütze.

Was nun die Hauptfrage anbetrifft, die das Verhältnis derartiger *Gliafasern* zum Ependym entscheidet, so zeigen meine obigen Angaben einen *intrazellulären Anfang*, wodurch es auch gerechtfertigt erscheint, derartige Anteile der faserigen Neuroglia hier als *ependymäre Neurogliafasern* zu bezeichnen. Es stimmen also diese Eigentümlichkeiten des Ependyms der höheren Wirbeltiere und des Menschen im allgemeinen mit den von E. MÜLLER bei den niederen Vertebraten gefundenen Verhältnissen überein, was auch bereits von diesem Forscher (18. S. 50) kurz dahin mitgeteilt worden ist, daß sich „die Neuroglia in struktureller Hinsicht bei diesen höheren Vertebraten (Rana, Bombinator, Lacerta, Kaninchen, Katze) ganz ebenso wie bei den niederen verhält“. Im besonderen scheinen jedoch derartig volle und kelchartig den Kern der Ependymzelle umgreifende Anfänge von ependymären Gliafasern bei den niederen Vertebraten nicht vorzukommen, wenigstens nicht in der von E. MÜLLER untersuchten Reihe (Amphioxus, Myxine, Acanthias vulg., Salus merlangus und Pleuronectes platessa). Größere oder völlige Übereinstimmung zeigen dagegen mehr jene Arten von Ependymzellen, deren periphere Protoplasmafortsätze nur wenige oder auch nur eine einzige Gliafaser führen. Der *weitere Verlauf* dieser intrazellulär beginnenden Einzelfasern oder Bündeln von solchen liegt auch noch zunächst im betreffenden protoplasmatischen Fortsatze selber drin, da sich längs jener intensiv schwarz gefärbten Fasern eine schmälere oder breitere und rot gefärbte Masse als direkte Fortsetzung des kernhaltigen Zellprotoplasmas hinzieht, welche ebenfalls wie jenes Protoplasmakörnchen enthalten kann. Auch weiterhin ist an vielen Stellen, z. B. bei jenen starken Faserbündeln, welche zur ventralen Spalte des Rückenmarks oder zum dorsalen Septum hinziehen, sowie an den von ihnen abgezweigten und bald quergetroffenen Seitenfasern eine ebenfalls rot gefärbte und stellenweise auch protoplasmatisch granulierte *Hülle* zu erkennen. Da sie bald stärker und deutlich, bald nur schwach und unscheinbar wie ein feines Häutchen ist, stellenweise aber auch überhaupt nicht zu sehen ist, so erscheint es zweifelhaft, ob diese *Hülle der Gliafasern kontinuierlich ist.* An den feineren Fortsätzen, die nur Einzelfasern enthalten, ist dagegen sehr bald, in geringer Entfernung von der Ependymzelle schon eine deutliche Hülle nicht mehr mit Sicherheit zu beobachten. Ob damit auch eine *intrazellulär beginnende und so sich auch zunächst fortsetzende Faser* weiterhin völlig frei und nackt wird, ist eine schwierige Frage. Da ich weiter unten in anderem Zusammenhange noch hierauf zurückkommen werde, so genügt es, hier soviel hervorzuheben, daß an den nackt und frei verlaufenden Gliafasern oder Bündeln von solchen dort mit einem Mal eine *Hülle oder ein Häutchen wieder sichtbar werden* kann, wo an der *Membrana limitans gliae superficialis oder perivascularis* das *Ende einer Gliafaser* gegeben ist.

Von den *ependymären Gliafasern* lassen die WEIGERTschen Abbildungen (Fig. 2, 3 Tafel III, Fig. 1 Tafel V, Fig. 2 Tafel VII

und Fig. 1 Tafel XI) nichts derartiges erkennen. Nur Fig. 3 Tafel XII zeigt eine Ausnahme. Im Text gibt WEIGERT allgemein zu dieser Frage an (6. S. 156), daß er gegen die Annahme KÖLLIKERS („daß an dem Fasergewirr der Substantia grisea centralis — auch Ependymfasern teilnehmen") a priori nicht das geringste einzuwenden habe. Seine Methode gestatte nur nicht, solche Beteiligung beim Menschen zu eruieren. WEIGERT meint dann weiter, daß, „wenn die Epithelzellen an der Faserbildung um den Zentralkanal einen Anteil haben, so erzeugen sie typische Neurogliafasern". Auch ich meine in Übereinstimmung hiermit, daß die von mir am menschlichen Ependym des Zentralkanals resp. der Rautengrube beobachteten Einzelfasern in den peripheren Fortsätzen jener Zellen typische Neurogliafasern sind und zwar *intrazellulär beginnende*. Und weiter halte ich von jener Fig. 3 Tafel XII (Sehhügeloberflächenepithel), daß sie, mit starker Vergrößerung und bei entsprechender Kontrastfärbung des Zellprotoplasmas gesehen, auch einen Zusammenhang der in der Tiefe des Sehhügels markierten, senkrecht verlaufenden Faserbündel mit jener ependymären Zellage in meinem Sinne zeigen würde.

Für die Frage nach einer ependymären Gliafaserung kommt weiter in Betracht, daß die *Gliafasern selber nicht immer mit der Richtung der radiären Fortsätze übereinstimmen*. In den seitlichen Umfängen des Zentralkanals, in den gleichen Abschnitten der Seite der Rautengrube (Hund, Mensch) stehen die *Gliafasern annähernd senkrecht zu ihren Fortsätzen*, sodaß sie zum Unterschied von den längsfaserigen Raphebündeln der letzteren Gegend oder den mehr ventral resp. dorsal gerichteten Fortsätzen der Zentralkanalzellen als quere und in Anfangsstücken der *Fortsätze selber zum Teil drin gelegene Faserpunkte* erscheinen. Es ist klar, daß auf rein differenzierten Gliafaserpräparaten eine derartige intrazelluläre Beziehung dem Beobachter leicht entgehen wird. Umsomehr wird das unmittelbare, subependymäre Lager von Gliafasern vom Ependym selber völlig unabhängig erscheinen müssen. Es sind das Verhältnisse, die auch in der Beurteilung des elementaren Zusammenhanges der subependymären und diffusen Neurogliafaserung und ihrer Verbindung oder Nichtverbindung mit den eigentlichen Gliazellen von großer Bedeutung sein werden. Wenn deshalb auf den angegebenen WEIGERTschen Abbildungen der ersten Gruppe nur ein Gewirr von schräg oder quer unter der Ependymfläche hinweg

verlaufenden Gliafasern zu sehen ist, so vermag also eine derartige Anordnung der Faserverflechtung noch keineswegs jede obige Beziehung zu gewissen Ependymzellfortsätzen auszuschließen.

Auf Grund dieser Beobachtungen und ihrer Folgerungen unterscheide ich also als *erste Zone in der Substantia Neurogliae centralis* eine *ependymäre Neuroglia*, die somit von den Ependymzellen und ihren faserreichen oder faserärmeren Fortsätzen gebildet wird. Die *Ausdehnung dieser Zone* ist dem entsprechend natürlich *abhängig von der Länge der Ependymzellfortsätze*, was beim erwachsenen Nervensystem aus den oben angegebenen Verhältnissen schwierig oder gar unmöglich ist. Ich kann also nur von der Gegend der ventralen Rückenmarksspalte, dem dem hinteren Septum zugekehrten Teile, sowie von der Raphe des Hirnstammes behaupten, daß die *Substantia Neurogliae centralis mit ihrer ersten Zone, der ependymären Neuroglia, streifenförmig bis zur pialen Oberfläche des Zentralnervensystems hindurchreicht*. Wie weit die *vertikalen Fasern als Anteil dieser Zone* reichen, kann ich aus leicht erklärlichen Gründen nicht angeben.

Vervollständigt wird nun die *ependymäre Neuroglia* durch eine *Reihe von Zellen* und ihre Faseranteile, welche bereits in ihrer Form typische, strahlenförmige und *eigentliche Neurogliazellen* sind und hier nur eine *unmittelbare Schicht von subependymärer Neuroglia* ausprägen, welche in ihrer groben Anordnung gar nicht oder wenig durch die Einlagerung nervöser Elemente bestimmt ist. Auch nach innen kann stellenweise, sobald das Ependym jene obige nicht mehr streng geschlossene Reihe flächenhaft gefügter Epithelzellen zeigt, wie beim Zentralkanal des Kaninchens oder Hundes oder des erwachsenen Menschen, die Abgrenzung der subependymären Gliazellen schwieriger sein, weil dann im erwachsenen Nervensystem Bilder entstehen, die an die embryonale Auswanderung der Ependymzellen und ihrer Umwandlung zu Astrocyten erinnern, und gewissermaßen eine späte Entstehung der subependymären Neurogliazellen aus dem Ependym dokumentieren. Es sind das Formationen, die auf Fig. 2 zum Teil zu sehen sind und darin bestehen, daß geringere oder beträchtliche Tiefenverschiebungen einzelner Ependymzellen oder Gruppen von solchen erfolgt sind, die ihre länglich-cylindrische bis kubische Form verloren haben und mehr vieleckig geworden sind. Sie können hierbei mit der inneren Fläche des Höhlenepithels noch stielartig verbunden sein,

teils auch schon soweit herausgeschoben sein, daß sie nur noch eine geringe Verklebung oder Verbindung mit benachbarten Ependymzellen besitzen. Im ersteren Fall würde eine solche Zelle noch als verschobene Ependymzelle zu gelten haben, im letzteren Fall aber muß man sie bereits den Zellelementen der subependymären Neuroglia zurechnen. Für die Lehre von der Entstehung der eigentlichen und sternförmigen Gliazellen aus dem Ependym können also in dieser Hinsicht auch die späten postembryonalen Erscheinungen am Ependym verwertet werden. Hierher gehören endlich in letzter Linie die Wucherungsvorgänge an dem menschlichen Ependym, die am Zentralkanal des Rückenmarks häufiger eintritt und jetzt zu den regelmäßigen Altersveränderungen gerechnet wird. Sie führt in gewisser Weise zu der Bildung eines dichteren Kranzes faserreicher Gliazellen von eckiger Gestalt und in allen Stadien der Lösung aus dem ursprünglichen die Höhle rein begrenzenden Ependym. So finde ich z. B. an dem Lenden-Rückenmark eines Hingerichteten vom Anfang der zwanziger Jahre unter der eigentlichen aber noch geschlossenen Epithellinie der Seitenwand zahlreiche und faserreiche Gliazellen, während an der dorsalen und ventralen Kante des Längsspaltes schon eine beträchtliche und zur völligen Auflösung der eigentlichen Ependymzellenschicht vorgeschrittene Zellvermehrung eingetreten ist. Dadurch ist also an diesen beiden Stellen die ependymäre Neuroglia durch eine solche von subependymärem Charakter ersetzt, insofern sie von unregelmäßig vielseitigen und vielfach mit einander durch schräge und kürzere Anastomosen verbundenen Zellformen zusammengesetzt ist, welche zugleich eine Menge typischer Gliafasern ausgebildet haben.

Was die allgemeine *Form der subependymären Gliazellen* anbetrifft, sowie das *gröbere Verhältnis von Gliafasern zu ihr*, so finde ich im Rückenmarksquerschnitt des Menschen und jener Tiere teils solche, welche durch eine Anzahl von Gliafasern *radiär gefasert* sind, teils andere, bei denen in der Hauptsache die betreffenden Fasern *quer getroffen* sind oder auch ein *wenig schräg* zu der *angeschnittenen Zellebene stehen.* Letztere Elemente überwiegen vor allem im Boden der Rautengrube. Von den radiärfaserigen Gliazellen, die also in den Begriff der „Astrocyten“ gehören, gelingt es, ihre Gliafasern weiter zu verfolgen. Sie sind es, welche teils denjenigen sich beigesellen, die im Rückenmarksquerschnitt der inneren Ependymoberfläche ein wenig parallel laufen, also ungefähr Bogenformen zu dem Zentralkanal resp. zu der Richtung des IV. Ventrikels beschreiben. Eine andere Gruppe schließt sich jenen Fasern oder Faserbündeln an, welche aus den Ependymzellen hervorgehen, um mit ihnen an der oberflächlichen Grenzhaut der vorderen Spalte usw. zu inserieren. Eine dritte Gruppe endet an den perivasculären Grenzhäuten der unmittelbar die zentrale gliöse Substanz durchsetzenden und weiterhin in der Substantia grisea centralis sich verzweigenden Blutgefäßen, wobei sie in rein radiärer Richtung und geradem Verlauf ein Blutgefäß erreicht und hier oft senkrecht zur Gefäßoberfläche endet oder auch seinen Längsverlauf weiter begleitet. Hauptsächlich aber bilden die Neurogliafasern dieser Gruppe mit jene vertikale Faserzone. Daß sie aus querlaufenden subependymären Gliafasern mit hervorgeht, läßt sich öfters beobachten. Immerhin aber ist dieser Zuwachs auf den einzelnen Querschnitten nicht sehr groß, so daß ich schließen muß, daß jene Längsfasern eine beträchtliche Länge haben, welche die meiner dünnen Rückenmarksquerscheiben überschreitet, so daß ich genauere Angaben über ihren derartigen Verlauf nicht machen kann. Was endlich das *Verhältnis* aller dieser *Gliafasern zu dem Protoplasma der subependymären Gliazellen* anlangt, so zeigen mir geeignete dünnere Paraffinschnitte, daß 1) jene *radiären Fasern im Protoplasma der betreffenden Astrocytenformen* liegen, wobei sie teils *unmittelbar neben der Oberfläche* (vielleicht in der Zellmembran selber) oder *dicht am Kern* das Protoplasma des Zellleibes durchsetzen, weiterhin aber auch *noch eine Strecke weit von einer feinen protoplasmatischen Hülle begleitet* sind, und daß 2) *ein großer Teil* jener *schief oder rein quer getroffenen Gliafasern in einem protoplasmatischen Netzwerk* zum Teil darin liegt, welches nach meiner Meinung aus der zahlreichen Verästelung aller jener Gliazellen wie der Seitenfortsätze der Ependymzellen entsteht und wie das Protoplasma jener Zellen selber eine wechselnde Menge von intensiv färbbaren Protoplasmakörnchen enthält. Immerhin aber erscheint auch an meinen Präparaten *ein Teil der Gliafasern ganz oder wenigstens eine Strecke weit* vollständig frei zu sein. Frei insoweit, als sie aus dem Bereich jener Netzbalken austreten können und dann frei in den Maschenräumen der Glia liegen. Daß sie nach weiterem Verlauf wieder in jene granulierte Masse zurücktreten können, habe ich aber auch anderwärts beobachten können, so

daß ich also für derartige Gliafasern nur eine partielle Emanzipation vom Gliazellenprotoplasma und seiner netzartigen Verzweigung zugeben kann. Was jene rein vertikalen Gliafasern anlangt, so finde ich sie beim Kaninchen zum größten Teil in der netzigen Gliasubstanz deutlich eingeschlossen (Fig. 2), ein kleinerer Teil scheint nur völlig frei zu liegen. Beim Menschen habe ich bisher keine umfangreichen Beobachtungen darüber machen können, inwieweit eine solche netzige Substanz der Neurogliazellen entwickelt ist, da ich aus Mangel an völlig frischem Material bisher nicht die zu diesem Nachweis nötige Art der Fixierung und Färbung für das Rückenmark habe anwenden können. Was ich am Lendenmark jenes Hingerichteten von 21 Jahren gesehen habe, ist, daß teilweise auch jenes die Gliafasern einschließende Netzwerk vorhanden ist. Ob es überall so wie beim Kaninchen und auch beim Hund, Kalb, Rind ausgeprägt ist, kann ich nicht angeben, da auf den mir vorliegenden Präparaten eine zu enge Zusammenpressung der einzelnen Fasern jener Schicht erfolgt ist. Dagegen kann ich mit Sicherheit behaupten, daß an vielen Gliafasern hier eine dünne, ein wenig mattgekörnte und stellenweise eckig geformte Substanz als Hülle für jene Faserelemente vorhanden ist.

b) Diffuse Neuroglia.

Beim erwachsenen Menschen, bei Kalb und Rind habe ich diese Elemente der diffusen Neuroglia hauptsächlich im Rückenmark untersucht; einige Betrachtungen habe ich auch an der weißen und grauen Substanz des Großhirns und Kleinhirns des Menschen angestellt. Bei Kaninchen und Hund habe ich außerdem ausgiebig den Hirnstamm beobachtet.

Abgesehen von der besonderen Anordnung der Neuroglia zu den nervösen Elementen der weißen und grauen Substanz ist ein elementarer Unterschied zwischen der subependymären und der diffusen Glia nicht vorhanden; auch eine wechselnde Menge von Gliafasern, ihre größere oder geringere Beziehung zum Protoplasma der Gliazellen und ein ungleicher Reichtum an protoplasmatischen Granulis können einen durchgreifenden Unterschied nicht bedingen, da dieses alles sowohl an den Elementen der diffusen wie der subependymären Glia selber an verschiedenen Stellen wechseln kann. Auch die von AGUERRE[26]) allgemein betonte außerordentlich

große Variation der Kernformen nach Größe (kleine, mittelgroße und große Kerne) und Krümmung ihrer allgemeinen Gestalt, oder nach Gehalt und Verteilung des Chromatins (gekörnte und homogene) ist keine, die für diese Zonen der Glia unterschiedlich wäre. Eine Sonderstellung muß ich vorläufig nur gewissen in unmittelbarer Nähe der Nervenzellen der grauen Substanz gelegenen Gliazellen geben, da sie, wie weiter unten noch besonders besprochen werden soll, *einseitige Beziehungen zu den perizellulären Stütznetzen der Nervenzellen*, ihren GOLGInetzen, erkennen lassen.

Im übrigen erscheint nun die äußere Gestalt und Verzweigung des Gliazellenprotoplasmas und zweitens die Gestalt *der deutlich in ihnen gelegenen Menge von Gliafasern* außerordentlich wechselnd. Folgende Einteilung wird hiervon ein Bild geben können. Ich unterscheide zunächst *protoplasmagroße* und *protoplasmakleine Formen.* Unter den letzteren sind die Extreme derartig, daß man die *protoplasmakleinsten* Neurogliazellen bei schneller Betrachtung und auf nicht sehr kräftig gefärbten Schnitten für protoplasmafreie Kerne halten könnte und versteht, weshalb früher in der Lehre von der Neuroglia so viel von den *„freien Kernen"* in der Grundsubstanz gesehen und gesprochen worden ist. Oft sind diese Kerne homogen, oft aber auch noch deutlich chromatinkörnig gebaut; es ist da schwer, ein abschließendes Urteil zu geben, weil erstens die Art der Fixierung (MÜLLERsche Lösung gibt viel mehr homogene Kerne wie z. B. ZENKERsche Lösung) und zweitens die Differenzierung und Art der Färbung große Fehlerquellen enthält. Die Kerne können ebenfalls sehr klein sein, oft aber eine etwas größere und deutlich bläschenförmige Gestalt besitzen. Immer aber ist die Protoplasmamenge wie eine dünne Schale nur angeordnet, die weiterhin in spärliche und sehr feine, mattgekörnte Ausläufer im Umkreis der ganzen Zelle sich fortsetzt (Fig. 8b). Irgend welche Schrumpfungen, die der MÜLLERschen Lösung eigen sind, und auch bei Formalinlösungen oder Alkohol von 96% mit nachfolgender Paraffineinbettung häufig entstehen, zerreißen leicht diese dünnen und feinen Fortsätze. Alle Übergänge gibt es von dieser protoplasmakleinsten oder kleinen Form der Gliazellen zu denjenigen, die durch eine große und sehr deutliche Menge von Protoplasma im Zellleib begrenzt sind (Fig. 6, Fig. 5b). Zwei Hauptarten können mit Leichtigkeit und Sicherheit unterschieden werden 1) *häutchenartige* (Fig. 4a, b) und 2) *protoplasmadichte* oder *proto-*

plasmakörnige Zellen (Fig. 5 u. 9). Zwischenstufen bestehen insofern, als das Protoplasma nur partiell körnig und dicht gebaut sein kann. Letztere Formen geben dann der ganzen Gestalt der Zelle eine leichte und deutliche Färbung. Die *häutchenartigen* Gliazellen sind durch das fast völlige Fehlen von körnigem Protoplasma zunächst charakterisiert, wenn auch mitunter einzelne Granula vorhanden sein können. Sonst aber scheint der Zellleib in der Hauptsache nur noch aus der vielleicht metamorphosierten oder verdickten Zellmembran allein zu bestehen, welche wie eine dünne Haut oder Hülle die betreffende Menge von mit der Zelle verbundener Gliafasern verbindet und zusammenhält (Fig. 4b). In anderen Fällen ist innerhalb dieser Haut noch eine eigentümliche spongioplasmatische Struktur vorhanden, welche in Form eines feineren oder gröberen Balkennetzes geordnet ist und einem durch umfangreichere und gröbere Vakuolisation entstandenen und veränderten Protoplasmarest entspricht. Derselbe ist zu den die Zelle durchsetzenden Gliafasern so angeordnet, daß er sie in seinen Wänden oder auch in seinen Balken während ihres Verlaufes durch den Zellleib führt und einkleidet. Bei bestimmter Differenzierung erscheint dann das innere Balkenwerk dunkelrot, während das allgemeine Häutchen mehr blaß gefärbt ist und die Gliafasern selber dunkelschwarz aussehen (Fig. 6 u. 7). Bei geringerer Entfärbung kann man das Netzwerk noch graudunkel darstellen, wodurch es dann weniger different von den Gliafasern selber erscheint, wie ich an einer derartigen Zelle vom Kaninchen abbilde (Fig. 4a).

Mag nun auch eine derartige Einteilung von Gliazellen in Rücksicht auf die protoplasmatische Beschaffenheit und die äußere Form des Zelleibes begründet sein, so sind doch alle darin einander ähnlich, daß sie die Art und der Anfang ihrer Verzweigung zu *protoplasmatischen Fortsätzen*, die dicker und feiner sein können, zu *sternförmigen* Gebilden macht. Im übrigen finde ich den Zellleib rund, wie bei den kleinsten Formen, sonst oval oder zu einem längeren und flachen Gebilde entwickelt, welches z. B. in der weißen Substanz den eingehüllten Nervenfasern entsprechend konkav geformt ist.

Berücksichtigt man nur die *Anordnung* der *Gliafasern zu diesen verzweigten Gliazellen* oder auch nur die alleinige Gruppierung ausschließlich gefärbter *Gliafasern zum Gliazellenkern*, so scheint, wie

WEIGERT gemeint hat, eine Astrocytenform nur noch für diejenigen Gliazellen zutreffend zu sein, bei der allein eine *radiäre Anordnung der Fasern zum Zellkern* vorherrscht. Hierbei ist vorausgesetzt resp. noch zu unterscheiden, daß eine derartige Figurierung der Fasern auch eine *allseitige* ist und also *in jeder Ansicht der Zelle* zur Anschauung kommt. Nun zeigen meine Abbildungen (Fig. 4—10 auf Tafel I), daß die *Anordnung der Gliafasern zu der jedesmal* (auf Querschnitten und Längsschnitten der weißen Rückenmarkssubstanz) *angeschnittenen verzweigten Zellfläche eine sehr wechselnde* ist. Im folgenden gehe ich hierauf näher ein, da sie durch die genauere Beurteilung der Gliafaserung in ihrer Richtung zur verästelten Zelle eine zweite Einteilung der Gliazellen ermöglicht, die dem für das ausgewachsene Zentralnervensystem so wichtigen Reichtum der Glia an besonderen Fasern gerecht wird. Was zunächst die *Lage der Gliafasern zum Protoplasma* der Gliazellen anbetrifft, die natürlich dort am schärfsten zu beurteilen ist, wo *reine Querschnitte der Gliafasern* vorliegen, so ergibt sich, daß 1) *freie Gliafasern* vorkommen, die den *Zellen oder ihren Fortsätzen nur anliegen* und sich mit ihnen kreuzen können. Ihre Menge ist insofern ungenau abzuschätzen, als der ganze Teil schief oder lang angeschnittener Fasern, die über die angeschnittene Fläche der Gliazelle hinweglaufen, unsichere Beobachtungen geben müssen. Immerhin gibt es eine Anzahl von Fasern, die wellenförmig verlaufend optische Querschnitte anzulegen gestatten und dann zeigen, daß sie nirgends auf dem betreffenden Schnitt, weder durch einen Zellleib, noch durch einen protoplasmatischen Gliazellenfortsatz hindurchgehen, also auch nicht von einem Protoplasma auch nur eine Strecke weit deutlich eingehüllt sind, sondern überall eine, wenn auch nur minimale Entfernung von demselben einhalten. Solcher Fasern finde ich eine ganze Anzahl. Berücksichtige ich zugleich, daß meine Schnitte 5—10 μ dick sind, und daß man überhaupt noch gar keine irgendwie genaue Vorstellung von der Länge einer Gliafaser hat, wohin man also eine Gliafaser, die man irgendwo auf einer Schnittstelle als völlig freie Faser findet, als Zellprodukt zu verlegen hat, so ist der Schluß auf eine *vollständig freie, zellunabhängige Faser* nicht sehr weitgehend. Dazu kommt noch folgende Beobachtung.

2) Finde ich solche Gliafasern, die nach *einigem Verlauf eine Strecke weit von einem Protoplasmafortsatz einer Gliazelle oder einer*

wie dieser auf meinen Präparaten rot gefärbten und etwas matt gekörnten oder häutchenartigen Masse gefaßt und eingeschlossen sind oder mit einander dort *verbunden* sind, wo sich *divergierende Gliafasern* eines geringeren oder stärkeren Gliafaserbündels von einander trennen (Fig. 2, 4b, 6, 7, 8a, 11, 12).

3) Endlich kann ich solche Beziehungen konstatieren, wo die Gliafasern deutlich *mitten im Zelleib* liegen, wobei sie als quergetroffene Fasern entweder *dicht der Kernmembran anstoßen* oder *unmittelbar in dem Protoplasma* der Gliazelle oder sogar in der Zellmembran selber drinstecken, welche bei gewissen häutchenartigen Gliazellen, die noch einen gekörnten Protoplasmarest fassen und zum Teil jenes oben beschriebene spongioplasmatische Fachwerk haben, als etwas dunkler gefärbte Randlinie sich abhebt. Im letzteren Fall würden derartige, also *unmittelbar in der Zelloberfläche laufende*, nicht ihr bloß außen anliegende Gliafasern, als *Membranversteifungen* aufzufassen sein.

4) Endlich finde ich an solchen Gliazellen, welche *außerordentlich grobe und konisch entspringende Fortsätze* haben und zugleich sehr dichtes Protoplasma im Leib wie Fortsatz besitzen, und zweitens durch abgehende *Bündel starker Gliafasern* gezeichnet sind, die weiterhin der allgemeinen Zelloberfläche folgend verlaufen, diese *Gliafaserbündel durch rot gefärbtes Protoplasma zusammengehalten und ihrer ganzen Länge nach* bis zu einem Gefäß z. B. oder bis zur Membr. lim. gliae superficialis bekleidet. Untersucht man Querschnitte dieser starken Fortsätze, so findet man die *Einzelfaser kreisartig* gestellt und der Mitte zu von rot gefärbtem Protoplasma vereinigt oder auch den ganzen Fortsatz von Fasern durchsetzt, in deren geringen Zwischenräumen die rote Farbe des Protoplasmas durchschimmert. Am Ende eines solchen Fortsatzes geht dann die Protoplasmamenge in den gleichsinnig gefärbten Gliafuß über, worauf ich noch näher bei der Besprechung der Eigentümlichkeiten der marginalen Glia zurückkomme.

Nach allen diesen Befunden kann ich also WEIGERT *nicht Recht geben, daß die Gliafasern vom Gliazellenprotoplasma völlig emanzipierte Gebilde* seien und einer *reinen Interzellularsubstanz gleich zu achten.* Und wenn ich auch hinzufüge, daß auf meinen Präparaten, die zum Unterschied von den nur rein fasergefärbten Gliapräparaten gleichzeitig die beiden zu bestimmenden Dinge verschieden gefärbt erkennen und demgemäß auch zu beurteilen

erlauben, ein Teil der Gliafasern an einer betreffenden Schnittstelle frei zwischen dem Gliazellenprotoplasma und seinen Fortsetzungen liegt, so folgt daraus noch keineswegs, daß nicht an *anderer Stelle für diese Gliafasern dieselben Beziehungen* bestehen, die an der ersten Gruppe von Gliafasern deutlich eine *intrazelluläre Lage* erkennen lassen. Weiter unten werde ich nochmals hierauf zurückkommen, wo ich über die Formation der Neuroglia und die Entwickelung der Glia einige Bemerkungen anzufügen habe.

Nach dieser allgemeinen Schilderung der Verhältnisse zwischen Gliafaserung und dem Protoplasma von Gliazellen habe ich auf eine zweite Einteilung letzterer einzugehen, die die wechselnde Faserhaltigkeit von Gliazellen auszudrücken ermöglicht. Ich unterscheide auf Grund der angegebenen Figuren 1) *faserreiche,* 2) *faserarme* und 3) *faserlose Gliazellen.*

Die letztere Gruppe besteht also aus Zellen, die von GOLGI, RANVIER usw. als „protoplasmatische Neurogliazellen" angesprochen worden sind. Ob solche Zellen dazu gehören, welcher (Fig. 10) nur überkreuzte Gliafasern äußerlich angelagert sind, ist nach dem Obigen fraglich. In der marginalen Glia der Großhirnrinde habe ich häufiger derartige Elemente gefunden als in der weißen Substanz des Rückenmarks. Über ihre Anzahl vermag ich keine Angaben zu machen. Doch kann ich hierzu ebenso wie zu der zweiten Gruppe der faserarmen Gliazellen bemerken, daß insofern eine gewisse Fehlerquelle dieser Klassifizierung anhaftet, als sich schwer der Verästelungsbezirk solcher Gliazellen auf Schnitten feststellen läßt, wozu hinzukommt, daß nach meiner Meinung überhaupt eine regionäre Abgrenzung für den protoplasmatischen Verzweigungsbezirk einer Neurogliazelle gegenüber benachbarten Gliazellen sehr unscharf oder überhaupt nicht besteht, sodaß es oft unmöglich wird anzugeben, welchen Zellen einzelne Gliafasern angehören. Das geht unzweifelhaft aus den angegebenen Figuren von der weißen Rückenmarkssubstanz wie vom Ependym des Zentralkanals wie von der marginalen Neuroglia der Großhirnrinde hervor. Es berührt diese *eigentümliche Beziehung von Gliafasern zu mehreren Gliazellen* schon die Frage nach der Formation des Neurogliagewebes. Es muß deshalb genügen hier hervorzuheben, daß jene obige Einteilung nur mit Rücksicht auf die *unmittelbare Beziehung von Gliafasern zu dem Zelleib und den Anfangs-*

stücken der abgehenden Fortsätze gerechtfertigt ist, und daß zweitens der *auffallende* Unterschied zwischen faserreichen und faserarmen Gliazellen wohl durch Berücksichtigung weiterer Verästelungszonen und hinzukommender Faseranteile eingeschränkt aber nicht ganz wird beseitigt werden können. Für die Trennung der 2. und 3. Gruppe mag vielleicht eine vollkommenere Untersuchungsmethode späterhin nicht mehr ausreichende Momente finden. Da außerdem beim Menschen mit dem Alter die Produktion an Gliafasern wächst, so würde sich also streng genommen die Grenze zwischen faserlosen und faserarmen Gliazellen verschieben, ebenso wie diejenige zu den faserreichen sich ändern müßte. Faserarme Gliazellen zeigen Fig. 5d, 8, 8b, 9. Zu unterscheiden wären zwei Arten; solche, bei denen die Gliafasern quer die Fortsätze passieren und andere, denen sie in leichtem Bogen oder mehr längs folgen. Zu den faserarmen Gliazellen gehören in letzter Linie natürlich auch die meisten der Ependymzellen, sofern sie nicht wie jene oben besprochenen Gruppen einseitig orientierte Bündel von Gliafasern produzieren.

In den *faserreichen Gliazellen* unterscheide ich 3 Gruppen 1) *radiärgefaserte*, 2) *radiärgebündelte* und 3) *quer-* oder *längsgefaserte*.

Bei den *radiärgefaserten Gliazellen folgen* im allgemeinen die *Fasern den entsprechenden Fortsätzen des Protoplasmas*, sodaß sie wie diese natürlich auch eine zum *Kern ungefähr radiäre Anordnung* zeigen müssen, ebenso wie die faserhaltigen Ependymzellen entsprechend ihrem Hauptfortsatz nur einseitig orientierte Gliafasern oder Bündel von solchen haben können. Nur kommt bei den radiärgefaserten eigentlichen Gliazellen hinzu, daß die den Zellleib tief oder oberflächlich durchsetzenden, langentwickelten Fasern nicht immer in gleichem Niveau in einen zweiten Fortsatz übergehen, sondern erst nach mehr oder weniger starkem Bogenverlauf denselben erreichen. Dem entsprechend findet man auf gewissen Schnittfiguren der Gliazellen außer lang angeschnittenen, von Fortsatz zu Fortsatz ausgespannten und gerade oder mehr bogenförmig am Zellkern passierenden Gliafasern solche, die als kürzere Bogenstücke der Tiefe des Schnittes zu und von ihr weg arrangiert sind, sowie endlich auch solche, die nur als reine Querschnittspunkte erscheinen (Fig. 6, 4a, 7). Aus derartigen Figuren wird man den Schluß ziehen dürfen, daß die betreffende Gliazelle nicht nur in der angeschnittenen Fläche radiärgefasert

ist sondern es auch in Rücksicht auf die vollständige Zelle sein wird. Man muß demgemäß derartige Gliazellen als *allseitig radiärgefaserte* Formen bezeichnen. Von diesen zu unterscheiden sind solche, wo außer einigen lang getroffenen Fasern, die noch dazu an einem Zellrande vorwiegend verlaufen, nur noch rein quergetroffene oder rein schief angeschnittene Fasern zu sehen sind; woraus zu folgern ist, daß bei diesen die Radiärfaserung hauptsächlich nur in einer oder einer zweiten Ebene entwickelt ist. Den Einwand, daß letzterer Schluß nur eine Folge eines zu dünnen Schnittes ist, kann ich nicht gelten lassen, da bei Steigerung der Schnittdicke von 5 μ zu 10 μ keine Bogenstücke hinzukommen. Derartige Formen müssen also als *einseitig radiärgefaserte* Gliazellen unterschieden werden. (Fig. 6.)

Von den radiärgefaserten Gliazellen unterscheiden sich die *radiärgebündelten* dadurch, daß *statt der einzelnen Gliafasern Bündel* von solchen entwickelt sind (Fig. 7a). Die einzelnen Fasern der Bündel brauchen dabei nicht immer stärker wie jene in der ersteren Zellart zu sein. Zahlreicher habe ich die radiärgebündelten Gliazellen in dem Lendenmark eines 21 Jahre alten Hingerichteten beobachtet und zwar besonders in der Substantia Neurogliae centralis. Ihre Faserbündel gingen teils an Gefäße, teils in die quergetroffene Innenzone, teils zu den der Fissura interior zustrebenden Faserzügen. Bei demselben Hingerichteten sowie auch an dem Rückenmark eines etwas älteren Menschen, habe ich noch eine Formvariante gefunden, die eine Zwischenstufe zwischen den radiärgefaserten und radiärgebündelten Gliazellen darstellt und einen oder zwei faserbündelreiche Fortsätze besitzt. Bei den oberflächlichen Gliazellen der weißen Substanz waren sie vorwiegend der *Membrana limitans superficialis* zugekehrt, wo sie mit einem dickeren Gliafuß inserierten.

Die merkwürdigsten Formen der Beziehung zwischen Zellprotoplasma und Gliafasern geben endlich die *quergefaserten Gliazellen* (Fig. 8b, 5) deren radiär abgehende und bald dünn verzweigte Fortsätze *hauptsächlich* außer einigen schief angeschnittenen *rein quergetroffene Gliafasern* enthalten. Daß dieselben wirklich im Protoplasma drin stecken, läßt sich sicherer und leichter entscheiden, wie bei den lang oder schief verlaufenden; sie lassen sich ebenso genau unterscheiden von solchen, die ihnen nur angelagert sind.

Eine andere Frage ist natürlich, wie derartige Zellen in der entgegengesetzten Ansicht aussehen würden. Sie kann natürlicherweise und leider nicht genau entschieden werden. Einen *kleinen Teil* von ihnen halte ich in Folge der Vergleichung von Querschnitten und Längsschnitten, die ich aus der Region der Hinterstränge des Rückenmarks daraufhin untersucht habe, für Schnittbilder von radiärgefaserten Gliafasern. Ich schließe mich damit einer zuerst von GOLGI und dann auch WEIGERT ausgesprochenen Ansicht an, wonach auf Vertikalschnitten durch die weiße Rückenmarkssubstanz die Astrocytenbilder zahlreicher erscheinen. Von einem zweiten Teil dagegen meine ich, daß er nicht von solchen Gliazellen herkommt, sondern von mehr lang entwickelten und in der Richtung der Nervenfasern gestreckten flachen Zellformen, welche ihnen gebogen anliegen und hauptsächlich eine reiche Menge von längsverlaufenden, teils auch unter sich gekreuzt angeordneten Gliafasern produziert haben. Insofern sind also die *quergefaserten Gliazellen* in entgegengesetzter Ansicht *längsgefaserte*. Auch auf Querschnitten oder Schiefschnitten vom Rückenmark lassen sich solche Zellen z. B. an den vorderen Wurzeln beobachten, sofern dieselben während ihres Verlaufes durch die weiße Substanz mehr der Länge nach getroffen sind (Fig. 12). Sie sind oft mehrkernig, also Riesenzellen (Fig. 5d vom Kalb). Bemerkenswert erscheint, daß ihre Gliafasern teils gekreuzt arrangiert sind, was man auch auf Querschnitten beobachten kann und aus ihrer entgegengesetzten Bewegung abzuleiten hat, die bei wechselnder Tiefendurchmusterung des Schnittes erfolgt. Diese *verschränkte Anordnung der Gliafasern*, sowie die *Umbiegung von Gliafasern* radiärgefaserter Zellen in den Rückenmarkssträngen ist es, die auch auf Vertikalschnitten ein Verfolgen der Gliafasern unmöglich macht, sodaß es auch hier nicht entschieden werden kann, ob die auf einem Schnitt beziehungslos und frei von Gliazellenprotoplasma erscheinenden Fasern es auch wirklich sind oder nur abgeschnittene Fasern, die irgendwo anders aus dem Leib oder dem Fortsatze einer Gliazelle hervorkommen. Zweitens betone ich aus der Beobachtung von Längsschnitten der weißen Rückenmarkssubstanz sowie derjenigen des Kleinhirns und Großhirns, daß *stellenweise eine deutlich netzartig angeordnete Masse zwischen den verschieden laufenden Gliafasern ausgespannt ist und dieselben einhüllt*, welche *von dem Protoplasma der Gliazellen irgendwie gebildet oder*

geliefert wird, sodaß auch in Rücksicht auf diese Zwischenmasse eine *totale Emanzipation der Gliafasern von den Gliazellen von mir nicht zugegeben werden kann.* (Siehe hierzu den folgenden Abschnitt und die Figg. 12, 14, 15, 16, 17).

Auf Grund aller dieser Angaben über die *protoplasmatische Form und Verzweigung von Gliazellen* sowohl wie über *die intrazelluläre Einfügung einer großen Anzahl von Gliafasern* ist es verständlich, daß auf reinen Faserfärbungspräparaten die wirkliche Beziehung der Gliafasern zum Gliazellenprotoplasma verborgen bleiben muß. Es ist andererseits klar, daß gewisse allgemeine Verhältnisse der Gliafaserung, der radiären und der queren, jedoch noch als solche zum mitgefärbten Kern der Gliazelle heraustreten müssen, die auffallender bei der ersten Gruppe sein werden, während sie bei der zweiten Form, mag sie nun eine scheinbare oder eine wirklich quergefaserte Gliazelle repräsentieren, mehr verborgen bleiben, zumal wenn bei einem *größeren Protoplasmaleib die Fasern hauptsächlich in seiner oberflächlichen Schicht* liegen. Ich citiere hierzu die Angaben WEIGERTS, auf Grund deren er eine freie, zellunabhängige Gliafaserung behauptet (6, S. 95—97): „Sehr charakteristische Bilder entstehen nur dann, wenn, wie sehr häufig, die Fasern in ganzen Büscheln um den Kern gelagert sind, sodaß eine spinnen-, pinsel- oder sternförmige Figur entsteht, in deren Mitte der Kern mit seinem zu supponierenden, unsichtbaren Protoplasma liegt. *Übergänge in dies unsichtbare Protoplasma sind nicht zu bemerken.* Sie müßten sich in der Weise geltend machen, daß die Fasern allmählich in der Nähe des Kerns blasser würden und sich dann in dessen Umgebung verlören. Das ist aber niemals der Fall.“ WEIGERT führt dann hierzu aus, daß „in diesen Kernzentren mit den strahlenförmig an sie angelagerten Fasern“ jene DEITERSschen Spinnenzellen oder Neurogliazellen ohne weiteres zu erkennen sind, daß in andern Fällen aber, wo die Kerne schon in einem mehr oder minder großen „Gewirr von Fasern“ liegen, solche Gruppierung entweder nicht besteht oder nur durch die betreffende Schnittrichtung verdeckt sein kann („verlarvte Astrocytenbilder“). „Endlich gelingt es auch diese „Astrocytenbilder“ noch manchmal herauszubekommen, wenn man die Seiten der Neurogliazellen z. B. durch neutrales Carmin färbt — eine Doppelfärbung, die freilich für die feineren Fasern nicht günstig ist. Dieses Mittel hilft dann, wenn der Raum zwischen Kern und

Faser zu groß ist, um die Beziehung beider hervortreten zu lassen d. h. wenn der Zellleib, der ohne Doppelfärbung unsichtbar bleibt, zu umfangreich ist. Aber trotzdem kann man wohl sagen, daß sehr viele *Kerne* zwischen den Fasern (namentlich vielleicht sämtliche kleine, dunkelgefärbte) sich in keiner Weise als Zentren zu Strahlensystemen erkennen lassen.“ WEIGERT kommt dann schließlich zu folgendem Resultat: „Trotzdem so viele Kerne ohne charakteristische Beziehung zu den Fasern sind, trotzdem die weichen Fasern keine Beziehung zu den Kernen erkennen lassen, wird man doch nicht umhin können, *alle* die nach unserer Methode gefärbten *Fasern für identisch mit den Gebilden zu halten, die man seit FROMMANN für Ausläufer der Neurogliazellen hält.*

Vergleicht man diese Angaben WEIGERTS mit meinen Befunden so zeigt sich erstens, daß ich in dem, was ich oben über eine quergefaserte oder eine radiärgefaserte Gliazelle angegeben, mit WEIGERT übereinstimme, daß aber zweitens WEIGERT jene *tiefe oder oberflächliche intrazelluläre Lage der betreffenden Gliafasern bei seiner Doppelfärbungsmethode entgangen ist.*

Außerdem ist klar, wie im besonderen WEIGERT dazu gekommen ist, die *Neurogliafasern mit den früheren Ausläufern der Gliazellen zu identifizieren und zu gleicher Zeit behaupten zu können, daß diese keine Ausläufer der Gliazellen sind. Weil WEIGERT an jenen radiärgefaserten Gliazellen keine „Übergänge der Fasern“ gefunden hat, sondern nur passierende, nie anfangende oder in der Kernzone aufhörende, deshalb sind zweitens die Neurogliafasern keine Zellfortsätze.* Man sieht, daß für WEIGERT die radiärgefaserten Gliazellen den Hauptausgangspunkt seiner Argumentation geliefert haben, die aber *einseitig ist, da WEIGERT gar nicht mit der Möglichkeit gerechnet hat, daß diese Fasern*, soweit sie nicht durch angelagerte von anderen Zellen her vermehrt sind, *intrazellulär liegen können*, daß sie also im Zellleib *oder auch in ihrem nächsten von der Zelle fortgehenden Verlauf von Protoplasma resp. einer Zellhaut bekleidet sein können*, wie meine obigen Beobachtungen zeigen. WEIGERTS Argumentation ist hierbei folgende: (S. 101) „Aber es wäre immerhin möglich, daß durch RANVIER und unsere Methode zwar abgesetzte Fäden statt der Ausläufer dargestellt werden, daß aber diese Darstellung auf irgend ein Kunstprodukt hinausliefe. Da die RANVIERsche Methode noch eine sehr unsichere war, die ihren Erfinder für das Großhirn, andere hervorragende Forscher, wie GOLGI, überhaupt im Stiche ließ, so lag diese Vermutung gewiß nahe, und man kann es den Untersuchern nicht übel nehmen, wenn sie trotz der Veröffentlichungen von RANVIER an ihren altgewohnten Auffassungen festhielten. Aber *unsere* Methode mag sonst gar manches zu wünschen übrig lassen, in der uns hier beschäftigenden Frage ist sie *ganz sicher*, und da müssen wir denn sagen, *nicht die Bilder von RANVIER*, sondern die nach *den alten Methoden erhaltenen Bilder waren Trugbilder.* Die nach den alten Methoden und nach der GOLGIschen erhaltenen Bilder bedeuten nämlich nur, daß bei diesen die Fasern und Zellleiber wegen ihrer gleichen Lichtbrechung (RANVIER) oder gleichen Färbbarkeit *nicht differenziert* werden, sodaß beide in chemischer (und morphologischer) Beziehung eins zu sein *scheinen.* Daß dies aber in der Tat nur Schein ist, das beweisen eben unsere Präparate, welche ganz sicher zeigen, daß Fasern und Zellleib im *chemischen Sinne von einander durchaus verschieden sind. Das ist aber der Kernpunkt der ganzen Frage.*“

Ich habe hierzu zu bemerken, daß mir das nicht richtig zu sein scheint, und zwar insofern, als in der WEIGERTschen Ansicht von der Neuroglia *zwei* Hauptpunkte den Kern seiner eigentümlichen Lehre bilden, *erstens, daß die Neurogliafasern chemisch verschieden vom Protoplasma der Gliazellen sind*, und zweitens, daß sie eine echte *Interzellularsubstanz* bilden. Das erstere bestreite ich WEIGERT keineswegs; es ist ihm auch nicht, wie die oben citierte Literatur gezeigt, irgendwie bisher bestritten worden. Diese besondere Beschaffenheit der Neurogliafasern bestätigt zu haben, und auch vor allem die wichtige, topographisch verschiedene Verteilung der Gliafasern zum ersten Male aufgedeckt zu haben, bleibt als großes Verdienst WEIGERTS bestehen.

Was aber den zweiten Hauptpunkt anlangt, so muß ich ihn als unrichtig bezeichnen. WEIGERT behauptet im Sinne der Definition von MAX SCHULTZE, daß bei den Neurogliafasern *außer jener Modifikation* ihrer Substanz auch eine *vollkommene Emanzipation vom Zellleib der Gliazellen vorhanden* sei. „Die Fasern stehen nur in Kontiguität mit dem Zellleib, sie sind mit ihm nicht als Ausläufer verbunden, sondern die zu- und abführenden Teile des Fadens, wenn man diese Ausdrücke brauchen darf, sind mit einander so innig vereinigt, daß sie etwas Zusammenhängendes, eine gemeinschaftliche *Fibrille* darstellen, die glatt über die anliegende

Zelle hinwegläuft. *Auf diese Weise tritt die Neuroglia endlich wieder durchaus in die Reihe der Bindesubstanzen*, aber wohlgemerkt nur vom *morphologischen Standpunkte aus.* (l. c. S. 116.)

Gegen diesen *zweiten Teil der WEIGERTschen Lehre* wende ich zuerst ein, daß er mit der von WEIGERT gegebenen *methodischen Unterlage überhaupt nicht begründet* worden ist. Daß die *Neurogliafasern den Gliazellen ausschließlich* auf- oder anliegen, oder über sie hinweglaufen, also die Zelle *nur berühren*, kann die WEIGERTsche Methode überhaupt nicht beweisen, da sie das Protoplasma ungefärbt läßt und also kein Urteil erlaubt, ob die Gliafaserung Interzellularsubstanz ist oder nicht. Zweitens halte ich dafür, daß jene Behauptung über die morphologische Natur der Neuroglia auch wirklich unrichtig ist, da meine obigen Angaben zeigen, daß die *Astrocyten radiärgefaserte Gliazellen* sind, deren eigene Gliafasern durch ein sie vereinigendes Protoplasma oder auch ein Zellhäutchen zellulär zusammengeschlossen sind, wovon sie nicht nur im Zellleib, sondern auch noch in ihrer Abgangsstelle oder noch weiter umhüllt sind. Wenn WEIGERT also gegen die Astrocytenlehre behauptet, daß ihre Bilder Trugbilder wären, da sie den Unterschied zwischen Gliafasern und Gliazellprotoplasma nicht gäben, sondern jene schlechthin als seine Ausläufer hinstellten, so würde dieser Vorwurf nach meiner Meinung jedenfalls dahin zu verändern sein, daß nur nicht die im Protoplasma des Zellleibs resp. dem der abgehenden Fortsätze die betreffenden Gliafasern als intrazelluläre Teile unterschieden worden wären. Womit ich also zugleich WEIGERT vorwerfen muß, daß er die Grenzen seiner Methode überschritten, als er die Gliafasern als reine nur noch den Zellen äußerlich angelagerte Interzellularsubstanz bezeichnete. Wenn ich hiermit wiederhole, was bereits früher LENHOSÉK (Feiner Bau des Nervensystems. 1895. S. 187) gegen die WEIGERTsche Methodik und Lehre hervorgehoben hat, daß sie „leicht zu der irrtümlichen Vorstellung führen kann, als wären die „Gliafasern“ etwas Selbständiges, wie etwa die Fasern des fibrillären Bindegewebes, als wären dann die „Gliazellen“ nur als sekundäre, davon unabhängige Gebilde in dieses Fasergewirre eingeschlossen“, so geschieht es auch aus dem Grunde, weil WEIGERT diesen Tadel dadurch pariert hat, daß er ihn nur auf seinen methodischen Nachweis der chemischen Verschiedenheit von Zellprotoplasma und Faser bezieht. Daß mit dem letzteren aber die Sachlage nicht entschieden ist, dürfte klar sein. Im übrigen unterscheiden sich in gewisser Weise die Beobachtungen RANVIERS von denen WEIGERTS, soweit sie für die zweite Frage nach den *Zellbeziehungen der Gliafasern* in Betracht kommen. So gibt RANVIER an (5, S. 180): „Ses fibres ne paraissent plus être de simples prolongements des cellules, car on peut les suivre maintenant au sein des cellules elles-mêmes. Elles sont simplement noyées dans le protoplasma“ — und weiter unten „Dans les préparations faites après l'action de liquide de MÜLLER, on voit la masse de protoplasma envoyer sur les fibres, qui s'en dégagent des expansions qui, généralement, s'étendent entre elles comme une membrane interdigitale, d'autres fois les entourent d'une sorte de manchon.“ Ich habe an diesen Angaben RANVIERS nichts auszusetzen, da ich dasselbe habe beobachten können. *Jedenfalls geht daraus hervor, daß die Gliafasern für gewisse Zellteile intrazellulär liegen und also hier auch keine Interzellularsubstanz bilden, wie WEIGERT behauptet.* Es ergibt sich aber auch andererseits der *allgemeine Schluß*, daß es sich überhaupt bei dem Streit um die Auffassung der Gliafaserung keineswegs um die WEIGERTsche Alternative — die Gliafasern liegen bei den Astrocyten dort, wo sonst die Ausläufer liegen sollen, sie sind aber keine Ausläufer, weil sie chemisch different sind — handelt, sondern um eine Frage, *die viel weiter gefaßt werden muß.*

Bekanntlich gibt es nach den Angaben von RANVIER und ANDRIEZEN z. B. in der Großhirnride rein protoplasmatische Gliazellen. Auch meine Beobachtungen zeigen auch sonst vielfach rein protoplasmatische Ausläufer von Gliazellen. Und da nun nach RANVIER die embryonalen Gliazellen überhaupt nur protoplasmatische Fortsätze haben, so wird solche Frage zu entscheiden haben, *wie viele von den ursprünglichen Protoplasmafortsätzen der Gliazellen faserhaltig werden oder nicht*, im besonderen wird hierbei zu untersuchen sein, wieso es kommt, daß die Gliafaserung so unregelmäßig erfolgt, daß sie einmal von Fortsatz zu Fortsatz in dem Zellleib einer Gliazelle ausgespannt ist, im anderen Fall von solcher Richtung erheblich abweicht. Als letzter Punkt bliebe aber dann immer noch zu wissen, ob die so aus dem Gliazellenprotoplasma herausziehenden besonderen Fasern auch in ihrem weiteren, langen Verlauf nackt werden oder nicht, und wie sie sich dann zu anderen Teilen der zentralen Substanz, den weiteren

Gliazellen oder auch den fremden Elementen der Blutgefäße verhalten.

Im folgenden Abschnitt schließe ich deshalb an diese allgemeinen Betrachtungen über das Verhältnis von Gliazellen und Gliafasern Beobachtungen an, welche einiges zur Entwicklung der Neurogliafaserung bringen und außerdem die feinere Bildung von Gliafüßen demonstrieren, um nochmals hieran die WEIGERTsche Lehre von einer faserigen gliösen Interzellularsubstanz zu kritisieren.

2. Die Entstehung der Neurogliafasern und die Enden derselben in den Fußstücken der Gliazellen.

Es ist klar, daß die Entstehungsgeschichte der faserigen Neuroglia nichts darüber an Beweis wird erbringen können, ob später im definitiven und funktionsreifen Gewebe die zu den anfänglichen Neurogliazellen hinzukommenden Neurogliafasern eine Interzellularsubstanz für sie bedeuten oder nicht. Immerhin wird aber eine Beobachtung ihrer Entwicklung zunächst die *Frage entscheiden müssen,* welche in den ersten Angaben von RANVIER bereits im Keime vorhanden ist, *was denn eigentlich aus den Fortsätzen der embryonalen Neurogliazellen wird, wenn es die Gliafasern später nicht mehr sein sollen und auch wegen ihrer „besonderen Beschaffenheit" in diesem engsten Sinne des Wortes wirklich nicht mehr sind.* Die Beobachtung von RANVIER ist folgende (de la Névroglie S. 182): Chez les embryons plus âgés (j'ai examiné des embryons de boeuf de o,m 75 et de o,m 95) un grand nombre de cellules de la nevroglie étaient étoilées et présentaient de longs prolongements; mais ces prolongements n'étaient pas encore des fibres véritables: ils avaient la constitution du protoplasma de la cellule et se fondaient avec lui."

Ich kann diese Angabe von RANVIER durchaus bestätigen nach dem, was ich an Embryonen von Maus, Meerschwein, Kaninchen, Katze, Hund und Mensch gesehen habe. Ich finde außerdem, daß auch auf etwas älteren Stadien, die bereits die erste Entwicklung von färberisch darstellbaren Gliafasern zeigen, die Zellen der embryonalen Neuroglia alle noch mehr oder weniger sternförmig sind und eine Anzahl von protoplasmatischen Fortsätzen besitzen. Der Reichtum an solchen Fortsätzen ist allerdings noch nicht derjenige wie später im erwachsenen Nervensystem.

In der zeitlichen Ausprägung sowie in der örtlichen Entstehung der faserigen Glia bestehen nun beträchtliche Unterschiede für Rückenmark, Kleinhirn und Großhirn, die im allgemeinen mit der sonstigen Reifung dieser verschiedenen Abschnitte des Zentralnervensystems zusammenfallen. Die faserige Glia entsteht zuerst im Rückenmark, und zwar zeigt hier durchweg die weiße Substanz ein Vorauseilen vor der grauen. Weitere und feinere Unterschiede, die noch die marginale Glia der Oberfläche betreffen, werde ich jedoch hier noch unberücksichtigt lassen, da ich die für die menschliche Neuroglia angefangenen und noch nicht abgeschlossenen Entwicklungsstadien später im Zusammenhang darzustellen beabsichtige. Ich werde also jetzt nur gewisse Hauptpunkte hervorheben, die auf jene obige Frage zurückgreifen und also für die zu behandelnde *allgemeine Frage nach der Entstehung der Gliafasern* überhaupt von Interesse sind.

In der Figur 28a, b, c habe ich die Verhältnisse dargestellt, welche z. B. für den Nervus opticus der Maus die Entstehung seiner Neuroglia zeigen. Von diesen drei Figuren demonstriert Fig. 28a (von einem Embryo von 1,2 cbm Länge) ein *rein zellige Glia,* die aus einer *Summe einfach verzweigter* und, wie hier sehr deutlich und sicher zu beobachten ist, auch *noch vielfach durch ihre Protoplasmaausläufer zusammenhängender Gliazellen* gebildet ist. *Im Innern* des nervus opticus anastomosieren diese protoplasmatischen Fortsätze nach Art eines *retikulären Gewebes,* in dessen Lücken die einzelnen Bündel des Sehnerven laufen; an seiner Oberfläche wiederum sind sie zu einer feinen Grenzhaut vereinigt, die noch alle bindegewebigen Elemente und insbesondere alle Blutgefäße von der eigenen Masse des Sehnerven und seiner zelligen Neuroglia fernhält. Durch *konisch verbreiterte protoplasmatische Füße* gehen die oberflächlichen Gliazellenfortsätze in die äußere, abschließende Grenzhaut über. Daß ich diese embryonalen Zellen des Sehnerven als Gliazellen bezeichnen kann, ist darin begründet, daß hier eine Verwechslung mit Nervenzellen ausgeschlossen ist, weil nach allen bisherigen Erfahrungen im Sehnerven Ganglienzellen nicht vorkommen, seine Masse also den *reinen Typus einer zentralen weißen Substanz* darstellt. Es kommt hinzu, daß, wie dieses Präparat weiter erkennen läßt, die *Entstehung der sternförmigen oder eigentlichen Gliazellen* aus der Masse der *HISschen Spongioblasten* erfolgt, worauf ich in weiterem Kapitel unten noch

einzugehen habe. Der dritte Grund endlich, der allgemein mit der WEIGERTschen Erkenntnis von der sicheren Beurteilung der Glia aus ihrer Faserung gegeben worden, ist der, daß die *Bildung der Gliafasern innerhalb dieser Zellen* erfolgt.

Wie Fig. 28b von der neugeborenen Maus erkennen läßt, geht sie in der Weise vor sich, daß die *feinen protoplasmatischen Fortsätze* einer embryonalen Gliazelle zu einer anderen Zelle hin oder zu jener Grenzhaut durch eine *dünne intrazelluläre Faser angefüllt und versteift werden.* Also *innerhalb des Protoplasmas jener Zellfortsätze* entseht zunächst die *faserige Glia* als ein *besonderes fädiges Produkt des betreffenden Fortsatzes.* Die feine intraprotoplasmatische Gliafaser erscheint auf meinen Präparaten dunkelschwarz, mehr dunkelgrau das umgebende Protoplasma des Fortsatzes selber, während die Nervenbündel des N. opticus mehr hellgrau entfärbt sind. Mit diesem Stadium habe ich noch ein etwas früheres, den Nervus opticus eines *reifen Mäusefötus*, verglichen; er zeigt zum Unterschied nur *ganz vereinzelte Gliafasern* in jenen Zellfortsätzen, welche noch nicht den Zellleib durchsetzen. Auch ist hier die *Substanz des sich bildenden Gliafäserchens* noch nicht so fest und homogen, sondern noch etwas *körnig,* sodaß er wie ein matter Strich im Zellprotoplasma erscheint.

Ich füge hinzu, daß dieser Bildungsprozeß der faserigen Glia nicht nur für den Sehnerven gilt; ich habe ihn allgemein in gleicher Weise z. B. auch im Rückenmark gefunden. Im besonderen erscheinen hier *vielfach,* wenn auch nicht immer, als *erste faserhaltige Protoplasmafortsätze* der sternförmigen Gliazellen der weißen Substanz diejenigen, welche zur *oberflächlichen* oder *perivaskulären Grenzhaut* rein radiär ausgespannt sind.

Die *weitere Veränderung der zelligen Neuroglia* durch die in ihr auftretende Gliafaserung betrifft die *Menge ihrer Fortsätze* und dann das *Wachstum der Gliafasern.*

Fig. 28c zeigt an einem Sektor vom Optikus einer fünf Tage alten Maus, daß die *Gliazellen fortsatzreicher* geworden sind. Auch ist die *Richtung der neuen Fortsätze* nicht mehr rein quer, sondern allseitig, wodurch auf dieser Stufe das allgemeine Bild einer früher vorwiegend quer zur Faserrichtung ausgespannten und deshalb leichter auf Querschnitten darstellbaren Gesamtmasse von Gliazellen undeutlicher und komplizierter erscheint. Ich kann deshalb auf diesem Entwicklungsstadium nicht mehr entscheiden, ob die

späteren Protoplasmafortsätze mit anderen Gliazellen sich verbinden oder frei bleiben. Daß aber auch hier einzelne und sichere Anastomosen noch vorkommen, zeigt die Fig. 28b. Ob diese die alten und ursprünglichen, vielleicht nur etwas ausgezogenen und gedehnten Verbindungen sind, ist deshalb ebenfalls zu sagen unmöglich. Mit diesem Stadium habe ich dann noch den Optikus von der 18 Tage alten Maus verglichen; er zeigt noch eine beträchtliche Zunahme seiner Gliazellen an faserhaltigen Fortsätzen.

Was nun die Hauptfrage nach der *weiteren Entwicklung* der intrazellulären und zwar in den Fortsätzen enthaltenen Gliafasern anbetrifft, so zeigen mir meine Präparate, daß die *Gliafasern sowohl in den Zellleib* wie in jene *oberflächlich gelegenen Enden* gewisser Protoplasmafortsätze der Gliazellen vordringen. Durch ein *Längenwachstum der Gliafasern* erfolgt also eine ausgiebigere Aussteifung der embryonalen und protoplasmatisch weichen Gliazelle. Speziell folgen die wachsenden Gliafasern den *oberflächlichsten Zonen des Zellleibes,* was vielleicht zum Teil damit zusammenhängen kann, daß überhaupt um den Kern herum nur eine geringe Protoplasmamenge stellenweise vorhanden ist. Insofern nun in der weiteren Entwicklung *außer den anfänglichen und vereinzelten Fortsätzen auch die weiteren faserhaltig* werden und zugleich *im Zellleib durchquerende Fasern* beobachtet werden können, wird man schließen müssen, daß die in einem Fortsatz z. B. nach beiden Richtungen hin fortwachsende Faser bald den Zellleib erreicht und durchsetzt und schließlich auch in einen *zweiten Fortsatz hineinwächst.* Auf diese Weise entsteht also aus einer reifenden Gliazelle mit zunächst noch mehreren, rein protoplasmatischen Fortsätzen und einzelnen, bereits faserhaltigen das Bild einer *allseitig radiär gefaserten* und für die spätere Zeit des fertigen Zentralnervensystems *typischen Gliazelle,* deren *Fasern natürlicherweise, da sie den vorgezeichneten Bahnen radiär gestellter Protoplasmafortsätze folgen, in Bogenform den Kern passieren* und von ihm divergieren müssen. Zweitens folgt aus diesen Beobachtungen über Entstehung und Wachstum der Gliafasern, daß bei *diesen sternförmigen Gliazellen* späterhin von irgend einem *Anfang der Gliafasern in der Zelle* nicht mehr gut die Rede sein kann, da sie ja bald von einem Fortsatz zu einem anderen hindurchreichen und den Zellleib nur durchqueren. Eine Ausnahme machen hiervon nur die Ependymzellen, welche als *unipolare* und mit ihrem Fort-

satz auch *einseitig orientierte Gliazellen auch später im Zellleib das eine Ende ihrer Gliafasern,* was man für gewöhnlich als Anfang zu bezeichnen pflegt, erkennen lassen. Drittens geht aus dieser Entstehungsweise der faserigen Neuroglia hervor, daß die KÖLLIKERsche Hypothese von einer oberflächlichen Zellplatte der Gliazellen und einer aus ihr erst sich sondernde Gliafaserung nicht richtig ist. Viertens endlich zeigt sich, daß eine *regionäre Abgrenzung* und eine *Beziehung von entstehenden und weiter wachsenden Gliafasern auf einzelne Gliazellen sich vielfach überhaupt nicht beschränken läßt.* Da jene Fasern in den *protoplasmatischen Anastomosen von Gliazellen* entstehen, wird schon von Anfang an eine *Unterscheidung unmöglich, welcher Zelle* sie zugeordnet werden soll. Aber auch in der folgenden Zeit des Wachstums der Gliafasern wird eine Verteilung einer gewissen Summe von ihnen auf einzelne Gliazellen als ihren Bildungszellen dadurch illusorisch, daß die schließlich lang gewordene Gliafaser bei ihrem Vordringen aus einem *ersten Zellleib auch einen zweiten,* wenn nicht noch weitere, erreicht.

Mit dieser *Bildungsgeschichte der faserigen Neuroglia* stimmen Beobachtungen überein, welche mir auch am *fertigen Nervensystem* zeigen, daß *Neurogliafasern mehr als einer Gliazelle gemeinsam* sein können. Im 6. Kapitel über die Formation des Neurogliagewebes werde ich näher hierauf eingehen.

Es bleibt eine Frage und zwar die, ob nicht irgendwie die wachsenden Gliafasern an irgend einer Stelle einfach den Zellleib und die Zellhaut hier durchstoßen und dann frei interzellulär vordringen, statt in die Bahn eines Protoplasmafortsatzes einzutreten. Gesehen habe ich bisher etwas derartiges nicht. Was *dagegen* spricht ist, daß auf diesen jüngeren Stufen der Faserreifung der Neuroglia die *einzelnen Neurogliafasern noch in ihrer ganzen Länge von einer dünnen und mattgekörnten Protoplasmahülle umgeben sind.* Dann vorzüglich ist dieses zu beurteilen und zu beobachten, wenn man *jene Fortsätze* verfolgt, welche als *oberflächliche* zur allgemeinen Grenzhaut hin ausgespannt sind und hier mit einem verbreiterten und konischem Ende, ihrem Fußstück, in sie übergehen. Zuerst erscheint dieses *Fußstück* nur als eine einfache und kegelförmige Ausbreitung eines vorher dünneren Protoplasmafortsatzes (Fig. 28a); auf der weiteren Stufe der Gliafaserung (Fig. 28b) erscheinen dann jene *Stiele der Fußstücke als faserhaltige* durch die in ihnen entstehenden und fortwachsenden Gliafasern, denen Enden als solche dann im Fuß selbst als freie erscheinen müssen, sofern man die protoplasmatische Masse des Fußes selber unberücksichtigt läßt oder auf radikal entfärbten Schnitten beobachtet, auf denen nur noch die Gliafasern als allein gefärbte Teile sichtbar zurückgeblieben sind. Dagegen halte ich es für vielleicht möglich, daß bei Dehnungen in Folge des allgemeinen Wachstums der nervösen Zwischenmasse jene anfänglich kontinuierliche Hülle der Gliafasern durchreißt oder sich wenigstens soweit zurückbildet, daß sie nicht mehr deutlich gesehen werden kann, weil ich an mehreren Stellen gesehen, daß am Zellleib und andererseits am Fußstück eine dickere Protoplasmamasse die betreffende Gliafaser umhüllte, in ihrem Zwischenstück aber anscheinend nicht mehr.

Im übrigen gehen die Fußstücke gewisser Fortsätze der Gliazellen noch weitere Veränderungen im späteren Lauf der Entwicklung ein. Ihre erwachsene Form, ihre Bedeutung für die morphologische Charakterisierung der marginalen Neuroglia und ihren Anteil an der Funktion dieser Grenzschicht des Zentralnervensystems wird das folgende Kapitel zeigen.

3. Marginale Neuroglia, ihre Grenzmembranen und ihre Entwicklung.

Eigentümlich für den *Begriff des Zentralnervensystems* ist, daß *seine Grenze* gegenüber den anderen Körperorganen *scharf* bestimmt werden kann, sowie man die *Ausbreitung und Entfaltung seines inneren Stützgewebes, der Neuroglia,* einer solchen Grenzbestimmung zu Grunde legt, daß dagegen solche Grenzen verloren gehen und z. B. in die Körpermuskulatur hineinreichen, sowie man die Endbezirke seiner ausführenden Nerven berücksichtigt, die doch nur die gewissen Ausläufer zentraler Nervenzellen sind. Daß die Neurogliazellen für das Zentralnervensystem der Wirbeltiere charakteristisch sind und normaler Weise an keiner anderen Stelle der Organismus ausgeprägt sind, ist allgemein bekannt und angenommen.

Daß nun das *zusammen mit den Nervenzellen im embryonalen Gehirnrohr entstehende Gewebe der späteren Glia auch noch im fertigen Organ streng auf den ursprünglichen Ort seiner Entstehung beschränkt bleibt,* wird allgemein aus der am *ectodermalen Epithel-*

gewebe bekannten *Eigenschaft* erklärt werden müssen, an ihrer *freien, d. h. dem mesodermalen Bindegewebe zuliegenden Oberfläche* eine *besondere Grenzfläche auszubilden*, die eine *fremde Gewebsart* scheidet.

Somit betrachte ich als *Keimlinge der Neuroglia* diejenigen *Zellelemente des embryonalen Gehirnrohres*, welche von Anfang an in *epithelartiger Stellung ausschließlich durchreichende Elemente* seiner Wand sind und es zum Unterschied von den *bald* sich lösenden und wandernden Neuroblasten auch noch für die *nächste Zeit der Entwicklung* bleiben.

Durch MAUTHNER (1861) ist das vom Zentralkanalepithel her durchreichende Stützsystem entdeckt worden, welches allerdings noch „in den Fasern der Pia mater" an der Rückenmarksoberfläche untergehen soll. Eine ectodermale Natur der Neuroglia hat dann zuerst HIS behauptet ([27]) S. 16): „Die Neuroglia selbst erscheint darnach auch in einem anderen Lichte; da sie nämlich mit der Pia und den von dieser austretenden Blutgefäßen *nirgends* zusammenhängt, so wird man genötigt sein, sie überhaupt vom Bindegewebe zu trennen, mit der sie ja auch histologisch nicht recht stimmen will, und man wird sie als ein Gewebe eigener Art anzusehen haben". Ebenso hat BOLL[28]) hervorgehoben, daß die Neuroglia mit den Nervenzellen an gleichem Orte entstände. Dann hat GOETTE[29]) ein das „ganze Rückenmark einhüllendes Häutchen" beschrieben, „welches keine Pia ist, „da die Gefäße erst außerhalb jenes Häutchens entstehen", sondern eine „Cuticula, die, sobald eine weiße Substanz angelegt worden ist, durch Schrumpfung jener Substanz abstehen kann. Sie bleibt aber durch feine Fäden, die mit breiterer Basis aus jener Fasernmasse hervorgehen, mit ihr verbunden. Später vermehren sich diese Verbindungen nicht nur, sondern gehen in membranartige Bildungen über, welche zwischen die Fasern der weißen Masse mehr oder weniger tief eindringen und so ein Fachwerk von zarten Scheidewänden bilden".

Im folgenden Jahr zeigte HENSEN[30]) beim Kaninchen, daß das Epithel des Zentralkanals als ein Radiärfasersystem von Fortsätzen durch die Masse des Markes hindurchreicht und mit fußförmig gestalteten Enden an einer „oberflächlichen Membrana prima" inseriere. Am menschlichen Embryo zeigte HIS[31]), daß sich ein System von radiären Fasern durch die gesamte Dicke

des Markes erstreckt", welches aus Verlängerungen von Zellen hervorgeht, die selber bis zur inneren Oberfläche des Medullarrohres reichen. Die äußeren Enden der Fasern „bilden eine selbstständige Belegschicht als erste Anlage weißer Rückenmarkssubstanz", sie verbreitern sich dann trompetenartig und liefern schließlich als „eine Art von Grenzschicht" die *Membrana limitans medullaris*. Dieselbe ist keine „ununterbrochene Haut" und zu unterscheiden von der *M. limitans meningea**), die als eine glatte Grenzschicht von den *netzförmigen Bindesubstanzzellen* geliefert wird, welche das Medullarrohr außen abgrenzen. Weiter[32]) hat HIS gezeigt, daß aus den Ausläufern jener Rückenmarkszellen ein Gerüst, das *Myelospongium* hervorgeht, „das späterhin zum Träger der Nervenfasern wird und das man als Neuroglia zu bezeichnen pflegt". Im übrigen meint HIS, daß zu diesem primären Teil der Neuroglia noch ein sekundärer hinzukomme, welcher aus den mit einwachsenden Gefäßen vordringenden Bindegewebszellen stamme und die DEITERSschen Zellen der weißen Substanz bilde. In der folgenden Abhandlung[33]) hat HIS die gerüstbildenden Zellen des embryonalen Gehirns als *Spongioblasten* bezeichnet, die aber wahrscheinlich mit der Entstehung jener DEITERSschen Stützzellen nichts zu tun hätten.

VIGNAL[34]) und GIERKE[35]) waren inzwischen für eine rein ectodermale Natur der gesamten Neuroglia eingetreten. Einen *definitiven Beweis* aber hat erst GOLGI[36]) an Hühnerembryonen erbracht. Seine Beschreibung ist folgende (S. 169 der deutschen Übersetzung „Untersuchungen über den feineren Bau des centralen und peripheren Nervensystems" Jena 1894): Die Cylinderzellen des Epithels des Zentralkanals „verlieren sich nicht in größerer oder geringerer Entfernung in der grauen Substanz, sondern durchziehen radial die ganze Schnittebene des Rückenmarks und erstrecken sich bis an den äußersten peripherischen Rand des Organs, bis zur Pia mater. Hier enden die fadenförmigen Ausläufer der Cylinderzellen, indem sie bald eine kegelförmige Anschwellung, bald eine

*) Es ist also ein Mißverständnis der HISschen Nomenclatur, wenn v. LENHOSSÉK folgendes behauptet (Feinerer Bau des Nervensystems, II. Auflage 1895 S. 204): „Diese Knötchen treten auf der freien Oberfläche des Markes mosaikartig zu einer offenbar lückenlosen, kompleten, äußerst feinen *Grenzmembran* zusammen, einer Art Cuticula (Membrana limitans meningea, HIS), die gegen die Pia mater hin das ectodermale Rückenmark vollkommen abschließt."

dünne Ausbreitung bilden, mit denen sie sich entweder an die Pia, oder an die dort verlaufenden Gefäße ansetzen. Auf diesem langen Wege bilden die mehr oder weniger kräftigen Fasern, welche die peripherischen Fortsätze des Körpers der einzelnen Epithelzellen darstellen, gewöhnlich Verzweigungen, welche bisweilen sparsam, bisweilen zahlreich und kompliziert sind. Die sekundären Verzweigungen ihrerseits gelangen zum Teil bis zur Peripherie des Rückenmarks und endigen hier auf die angegebene Weise; zum Teil verlieren sie sich auf dem Wege, ohne daß man das Wie? genau bestimmen könnte, zum Teil setzen sie sich an die Gefäßwände an." „Aus dieser Beobachtung geht offenbar hervor, daß der epitheliale Teil des Rückenmarks, welcher ohne Zweifel vom äußeren Keimblatte abstammt, an der Bildung des zwischen den nervösen Elementen liegenden Gewebes (der Neuroglia) unmittelbaren Anteil hat." GOLGI fügt hinzu, daß er sich aus einer „Reihe von embryologischen, chemischen und histologischen Beobachtungen" für berechtigt halte, dem Epithelium auch die eigentlich so genannten Zellen der Neuroglia gleichzustellen". NANSEN[37]) kam dann für Myxine ebenfalls zu dem Schluß, daß die Neurogliazellen aus dem Epithel des Zentralkanals sich entwickeln.

Die Form des Entwicklungsprozesses selber, die Auswanderung der Epithelzellen und ihre Formänderung zu Astrocyten hat dann zuerst R. y CAJAL[38]) demonstriert. Durch die Untersuchungen von v. KÖLLIKER[39]), van GEHUCHTEN[40]), von LENHOSSÉK[41]), RETZIUS[42]), Cl. SALA y PONS[43]) sind dann diese Beobachtungen CAJALS bestätigt und erweitert worden.

Gegen die Lehre von der einheitlichen ectodermalen Entstehung des Neurogliagewebes haben sich im Sinne der späteren Angaben von HIS die Untersuchungen von LACHI[44]), VALENTI[45]), CAPOBIANCO[46]), HATAI[47]) ausgesprochen, auf die ich hier nicht näher eingehen will. Ich selber meine, daß die *Neuroglia nirgends eine Beimischung von mesodermalen Elementen* erhält. Die Gründe hierfür werde ich in folgenden Beobachtungen ausführlicher erörtern.

Ich teile die Entstehung der Neuroglia und ihre Entwicklung zum fertigen Stützgewebe in 3 Perioden. Die erste Periode umfaßt die Bildung der *primären Glia;* sie ist dadurch charakterisiert, daß das *zentrale Stützgerüst ausschließlich* von den *Ausläufern* der Zentralkanalepithelzellen des embryonalen Gehirnrohres oder

der *embryonalen Ependymzellen* zusammengesetzt wird. Beim Amphioxus bleibt auch dauernd die Neuroglia auf dieser primären Stufe stehen. Bei den übrigen Wirbeltierklassen aber ist sie wie bekannt nur für die erste Zeit der Gehirnentwicklung typisch. Die zweite Periode führt zur *sekundären zelligen Glia*, die *außer* den reinen und später reduzierten *Ependymzellen noch jene umgewandelten Ependymzellen* und ihre *weiteren Vermehrungen*, die *Astrocyten* enthält. Die dritte Periode würde dann die *Entstehung der Gliafasern* umfassen, welche nach der oben skizzierten Weise in jenem Gewebe der sekundären, noch rein zelligen Glia vor sich geht und die Ausbildung einer *sekundären zellig-faserigen Neuroglia* bedeutet.

Ich komme jetzt auf die *Begrenzung des embryonalen Zentralnervensystems durch die primäre Glia* zurück. Nach HIS bilden die *Spongioblasten* als durchreichende Elemente der embryonalen Gehirnwand mit ihrer *inneren Oberfläche* die *Membrana limitans interna*. Sie begrenzt den Zentralkanal des Rückenmarks resp. die Gehirnventrikel und zeigt eine Felderung, die auch sonst für eine durch Kittlinien vereinigte Masse von Epithelzellen an der Oberfläche charakteristisch ist und als solche auch für das fertige Ependym des reifen Zentralnervensystems gilt. Der epithelartige Charakter dieser inwendigen Grenzhaut kommt auch weiter auf meinen Präparaten dadurch zum Ausdruck, daß in den einzelnen Feldern und zwar dicht unter der feinen, kutikularen Endfläche je ein Doppelkorn vorkommt, welches auch sonst für das innere Ende von Cylinderepithelien als ihr Zentralkörper gilt. Derartige Präparate besitze ich vom Meerschweinembryo (2,3 cm), Kaninchenembryo (3,7 cm) und einem gleich weit entwickelten Embryo vom Hund. (Bei beiden letzteren zeigt der Plexus chorioïdeus bereits durchweg Basalkörper mit Flimmerhaaren.)

An der *äußeren Oberfläche* entsteht nun nach HIS aus den entsprechenden Teilen der Spongioblasten ein grobgerüstiges Balkenwerk (Myelospongium), welches dann als *ein Randschleier* die periphere Zone des Gehirnrohres bildet. „Die Hauptbestandteile des Randschleiers sind zahlreiche blattartig geformte Radiärbälkchen, die an ihrem äußeren Ende verbreitert zu sein pflegen, und die untereinander durch kurze Quersprossen zusammenhängen. Je jünger die Entwicklungsstufe, um so schmaler ist der Randschleier, und um so dichter sein Gefüge. In vorgerückteren Perioden lockert

sich das Maschenwerk; aber auch in einer Zeit, da schon reiche Mengen von nervösen Längsbahnen vorhanden sind, ist die primäre Anordnung des Markgerüstes in der radiären Gliederung der weißen Substanz immer noch zu erkennen" (Abhandlung über die Neuroblasten S. 239). *Ob die einzelnen Lücken des Randschleiers selber offen oder nach außen geschlossen sind,* hat HIS unentschieden gelassen. In seiner Arbeit von 1883 sagt HIS hierüber, daß der Name *M. limitans medullaris*, die also die *äußere Grenzhaut des Randschleiers* bilden würde, nicht den Begriff einer „ununterbrochenen Haut" enthalte.

Ich finde nun, daß der HISsche Randschleier durch eine *feine, späterhin sich noch verdickende Membran kontinuierlich bedeckt* ist, welche als *dauernder Rest einer von vornherein nach außen abgeschlossenen* Epithelfläche persistiert und durch *Vermehrung* der von *innen her* zu ihrer Masse zusammenkommenden Elemente, *Spongioblasten* und *spätere Gliazellen*, der ganzen Organvergrößerung entsprechend wächst und sich ausdehnt. Ich will sie als *Membr. limitans medullaris superficialis* bezeichnen.

Bei *Flachschnitten* zeigt sie eine *Gliederung*, welche den durch HIS bekannt gewordenen Feldern der M. lim. interna durchaus entspricht. *Senkrechte Durchschnitte* lassen erkennen, daß zahlreiche und im allgemeinen konisch geformte *Füße jener Spongioblasten* in ihr zusammenkommen (Fig. 21 vom Rückenmark des Embryo EB der HISschen Sammlung). Der Randschleier zeigt auf dieser Stufe noch seine ersten Stadien der Ausbildung; beim Embryo Br 3, dessen Untersuchung ich ebenfalls der Freundlichkeit des Herrn Geheimrat HIS verdanke, ist der Randschleier bereits breiter entfaltet; seine Räume sind auch hier durch eine Grenzhaut eingefaßt, welche dicker wie beim Embryo EB. geworden ist. Ihre Entstehung aus den fußartigen Endstücken der Spongioblaste tritt auch hier klar hervor (Fig. 22). Hinzufügen muß ich, daß allgemein und, wie auch bei Br 3 zu beobachten, unter dem Einfluß der Fixierung und Einbettung ein Zerreißen der oberflächlichen und also jene Fußteile der Spongioblasten enthaltenden Schicht des Randschleiers stellenweise eintreten kann, wodurch die genetische Zusammengehörigkeit jener Grenzhaut mit dem HISschen Randschleier unklarer werden kann. Nennt man nach HIS das an der Oberfläche des embryonalen Gehirnrohres sich zusammendrängende mesodermale Gewebe die *M. limitans meningea*, so würde also erst die *M. limitans medullaris superficialis* die genaue Scheidewand des ectodermalen Gehirnrohres darstellen. *Überall* ist nach meinen Beobachtungen jene *Membrana limitans superficialis* ausgeprägt; sie bedeutet somit auch eine *vollständige Scheidewand für das die Gefäße vorbereitende Mesoderm*. Denn man findet anfangs noch *alle Gefäßsprossen* **ausschließlich** in jener *Schicht der M. limitans meningea zusammengedrängt* und eine Zeit hindurch auch *vollständig von den oberflächlichen Zonen des Randschleiers* durch die *M. limitans superficialis zurückgehalten und abgedrängt* (Fig. 22).

Diesem *ersten Stadium einer oberflächlichen Anhäufung neugebildeter Blutgefäße* folgt dann das *Stadium* ihres *Eindringens* in das embryonale Gehirnrohr. Es geschieht in der Weise (Fig. 23 bis 25), daß eine *keilförmig* gestaltete *junge Gefäßsprosse* jene *M. limitans superficialis spaltet*, in der sie, wie Fig. 23 offenbar zeigt, längs eines Kittstreifens zwischen zwei benachbarten Spongioblastenfeldern eindringt und die entsprechend tieferen Anteile des noch in einzelne Epithelzellbezirke gegliederten Randschleiers auseinanderlegt.

Die weiteren Stadien des Eindringens geben Fig. 24 und 25 von derselben Stelle eines Kaninchenembryo wieder, die vor allem zeigen, daß es sich auch bei dem *weiteren Einwachsen der ersten Gefäße ins Gehirn* zugleich um eine *entsprechend vorschreitende Neubildung einer M. limitans aus den tieferen Abschnitten des Randschleiers* handelt, die in *gleicher Weise* wie jene M. lim. an der äußeren Oberfläche hier *innerhalb des Zentralnervensystems selber das eindringende gefäßhaltige Bindegewebe von vorne herein von der eigenen epithelialen Masse des Gehirns abgrenzt und abschließt*. Ich will diese im Innern des Gehirnrohres aus Seitenzweigen der Spongioblasten entstehende Grenzhaut als *M. limitans perivascularis* bezeichnen. Sie umgibt also als eine *röhrenartige Scheide das einzelne Gefäß* und hängt naturgemäß an jener oberflächlichen Einbruchspforte mit der oberflächlichen und allgemeinen Grenzhaut des Gehirns, der M. limitans superficialis kontinuierlich zusammen. Nicht nur für den Embryo des Menschen und des Kaninchens gelten diese Angaben; umfangreicher habe ich auch an Embryonen von Ringelnatter, Frosch, Salamander, Forelle, Zitterrochen, Huhn, Meerschwein, Maus, Katze, Hund die Ausbildung jener oberflächlichen und inwendigen Grenzhaut des Zentralnervensystems beobachten können. Daß ich dieses vergleichende Material in relativ kurzer

Zeit habe untersuchen können, verdanke ich Herrn Geheimrat HIS und Herrn Professor KÄSTNER, die mir freundlichst ihre Sammlungen zur Verfügung gestellt haben.

Die *Grenzhäute des erwachsenen Zentralnervensystems* sind dieselben wie beim Embryo; sie liegen zu *einem Teil unmittelbar unter der Pia mater* und entsprechen jener *M. limitans superficialis* als dem ersten Abschluß des noch nicht von Gefäßen durchsetzten Gehirnrohres, zum *anderen Teil umhüllen* sie die *bindegewebigen und gefäßhaltigen Einsenkungen oder Septen der Pia* als *perivaskuläre Membranen*.

Für das *fertige Nervensystem* will ich nun die Bezeichnungen von *Grenzhäuten der Neuroglia* gebrauchen, da auch nach meinen Beobachtungen, welche durchaus die zuerst von GOLGI angedeuteten und später von RAMON y CÁJAL an Silberpräparaten demonstrierten Tatsachen bestätigen können, daß die Glia *rein epithelialer Herkunft* ist. Daß die *Entstehung der sekundären zelligen Glia* wirklich auf der Auswanderung und weiteren Formänderung von ursprünglich rein im Ependym gelegenen Zellen beruht, kann ich auch mit einer anderen Methode der Schnittfärbung als der GOLGIschen Silbermethode am Nervus opticus demonstrieren und damit die Resultate eines heute vielfach angegriffenen Imprägnationsverfahrens unterstützen und sichern.

Die Fig. 28a zeigt am n. opticus eines zwölf Tage alten Mäuseembryo die *Entstehung einer sekundären zelligen Glia aus dem Ependym*, welches noch am unteren Rand in seiner *primären Form* den Rest des hohlen Augenblasenstieles bildet, während der übrige Umfang des Sehnerven bereits *zahlreiche Nervenbündel von einer zusammenhängenden Masse sternförmig verzweigter Zellen* umgeben und begrenzt zeigt. Diese *innere Menge von Stützzellen* bildet mit jenem Nervenbündel zusammen eine *weiße Substanz*, welche für diesen Teil des Zentralnervensystems bekanntlich von Anfang an und auch dauernd diesen *reinen Charakter* behält und somit auch klar die Bildungsgeschichte der zelligen Neuroglia beweisen muß, insofern zum Unterschied von anderen Abschnitten des embryonalen Gehirnrohres keine Beimischung von Nervenzellen die Deutung der Zellverschiebungen erschweren kann.

Daß diese Summe von inneren Astrocyten ependymärer Natur ist, zeigt ohne weiteres die angegebene Figur; daß sie weiter auch *aus sich selber herauswächst und sich vermehrt*, zeigen vereinzelte, überall vorhandene *Mitosen*; daß endlich auch *keine Mesodermzellen beigemischt sein können*, geht aus der *geschlossenen Grenzmembran* hervor, welche die *Oberfläche des Sehnerven einfaßt und scheidet*. Erst nach dem Wurf erfolgt das Eindringen von Gefäßen in dieses bis dahin völlig abgeschlossene und rein gebliebene epitheliale Gewebe und damit die innere Ausbildung von perivaskulären Grenzhäuten, zu einer Zeit also, wo bereits die Produktion von Gliafasern begonnen hat. Auch die ersten Stadien der beginnenden Randschleierbildung zeigt die Fig. 28a; sie kommt an den zum unteren Rand reichenden Ependymzellen zum Ausdruck, welche eine oberflächliche *Lakunenbildung* erkennen läßt, wie sie HIS als *vorbereitendes Stadium zur Formation des Randschleiers* erkannt hat. (W. HIS, Histogenese und Zusammenhang der Nervenelemente. Referat in d. anat. Sektion des internationalen med. Kongr. zu Berlin. 7. Aug. 1890).

Ihrer Entwickelung entsprechend sind also die *Grenzhäute des erwachsenen Zentralnervensystems solche der Neuroglia;* ich unterscheide sie als *Membrana neurogliae superficialis* und *perivascularis*. Beobachtet habe ich sie an solchen Präparaten, die *sowohl die Neurogliafasern als auch das Protoplasma der Gliazellen und die Masse der Membran selber gleichzeitig gefärbt* erkennen lassen. Bei der Beschreibung stütze ich mich also ausschließlich auf solche Präparate.

Ich schicke voraus, daß bei der WEIGERTschen Neurogliafärbung auch diese Beziehungen der Glia zur M. limitans Gliae verborgen bleiben. Diese nur für die Erkennung der normalen oder pathologischen Menge und Anordnung der Neurogliafasern so bedeutungsvolle und wichtige Methode bedingt eine so radikale Entfärbung aller der anderen Gewebsteile der Glia, daß es, wie aus dem folgenden klar werden wird, nicht verwunderlich ist, wenn die WEIGERTsche Lehre von der Neuroglia so einseitig geworden ist. Daß sie andererseits in der Unterscheidung von Gliazellenfortsätzen und Gliafasern als etwas Besonderem aufklärend gewirkt hat, verkenne ich hierbei keineswegs.

In Übereinstimmung mit der im 2. Kapitel nachgewiesenen intrazellulären Entstehung von Gliafasern sind es also im erwachsenen Zentralnervensystem zum großen Teil nicht mehr allein einfache und locker gebaute Fortsätze von Astrocyten, sondern *Gliafasern*, welche in einer gleich näher zu beschreibenden Weise

an jene *Grenzfläche* herantreten und in sie übergehen, welche noch an jene *Fußbildungen* erinnert, die für die *primäre oder auch sekundäre zellige Glia* gilt. Daß *daneben* auch *rein protoplasmatische Gliafüße* vorkommen, zeigen auch meine Präparate. Die Kapillaren der großen Rindensubstanz im Kleinhirn und Großhirn zeigen hauptsächlich einen solchen Aufbau ihrer perivasculären Grenzhaut. Auch an der M. lim. Gliae superficialis der Großhirnrinde finden sich vielfach derartige Verhältnisse, die aber schon häufiger mit faserhaltigen Füßen gemischt sind, sodaß also hier jene Verhältnisse wiederkehren, die ich oben einer Einteilung der Gliazellen in faserfreie und faserhaltige zu Grunde gelegt. In der weißen Substanz, in der oberflächlichen Gliahaut des Kleinhirns, in der entsprechenden Grenzmembran des Rückenmarks dagegen, sowie in der Substantia Neurogliae centralis sind durchweg und hauptsächlich derartige Beziehungen der Gliazellen durch Gliafasern vermittelt. Zwei Typen unterscheide ich hauptsächlich in der Bildung derartiger Gliafüße, wobei ich von der Variation absehe, daß eine Einzelfaser oder zwei und mehrere zu einem Bündel vereinigte zu den Füßen herangehen.

Die *eine Hauptform* besteht darin, daß die *Gliafaser* selber oder das betreffende Bündel (Fig. 39b, c) von ihnen als dunkel gefärbte Teile aufhören und in *blasser, häufig noch feiner zerfaserter Substanz in den Fuß selber* übergehen. Ob es sich hier um eine Substanzänderung oder nur eine Substanzlockerung handelt, die jene Färbungsunterschiede hervorruft, mag unentschieden bleiben. Fein und regelmäßig aufgefaserte Füße sind z. B. die Enden der BERGMANNschen Fasern im Kleinhirn (Fig. 33a, b).

Die *zweite Hauptform* zeigt eine Gliafaser oder einzelner bei einem Bündel, welche als solche *durch den Fuß selber hindurchziehen* und in der betreffenden Grenzhaut flach weiterlaufen. Meistens können sie weil abgeschnitten nicht weiter in ihrem Verlauf verfolgt werden; mitunter aber habe ich doch beobachtet, wie sie nach kürzerer Strecke blaß in derselben aufhören. Es entsprechen also diese Angaben denjenigen von RETZIUS, wonach hakenförmige Umbiegungen der Gliafasern an der Intima Piae vorkommen.

Dieser zweite Typus der Gliafüße interessiert noch insofern als er einen Beweis dafür enthält, daß bei der *Bildung der Gliafüße noch etwas anderes und Besonderes* in Betracht kommen und auch beim ersten Typus versteckt vorhanden sein muß, bei dem

der betreffende Fuß mehr als die direkte Ausbreitung einer substanzveränderten oder substanzgelockerten Gliafaser erscheinen kann. Dieses Besondere erscheint von vorneherein nur da, wo es sich deutlich von der dunkel gefärbten Faser abhebt und die es einhüllt, als eine feine, mitunter etwas gefältelte Haut, welche der Grenzmembran zu immer mehr kegelförmig oder auch pyramidenförmig um die in ihr laufende Faser ausgespannt ist und schließlich mit breiter Basis in jener Gliamembran selber aufhört. In Rücksicht auf jene embryonalen Fußformen ist dieses Häutchen zum mindesten also der einer *Zellmembran* gleichwertige und oberflächliche Teil einer Gliazelle. Was auch dadurch kenntlich wird, daß oft außer jener Faser *protoplasmatische Granula in ihr eingefügt* sein können, wie mir besondere Präparate zeigen. Woraus ich weiter folgere, daß dieses Häutchen des Gliafußes nicht ein einfacher protoplasmatischer Rest oder eine umgeformte, veränderte Substanz ist, die als Produkt der Gliazelle der Gliafaser etwa gleich zu achten sei.

Ich füge hinzu, daß bei dem Typus einer endständig aufgefaserten Gliafaser die einzelnen divergierenden Strahlen sich innerhalb jenes Häutchens als ein dasselbe ausstützendes System feiner Fäserchen bis zur Grenzhaut als ihrer Insertionsstelle verfolgen lassen (Fig. 33a, b).

Daß die *Grenzhaut der Glia* resp. der *Anteil eines jeden sie zusammensetzenden Gliafußes* in den Begriff einer *Zellmembran* zu bringen ist, dafür sprechen auch die zahlreichen Variationen, die man an der marginalen Glia beobachten kann, die zugleich eine Beurteilung des *Fußstückes* selber erlauben.

Am einfachsten liegt die Sache da, wo der *Zellleib einer Gliazelle selber breit in der Grenzhaut eingepaßt ist*, was keineswegs selten ist. Hier fällt also die Fußfläche direkt in den Bereich der *Zellleibsmembran*. Fig. 29 zeigt dies an der M. lim. gliae superficialis des Kaninchenrückenmarkes. Auch beim Menschen habe ich dies oft gesehen, sowohl an der oberflächlichen wie perivasculären Grenzhaut. Die früheren Angaben von GOLGI, wonach die „Zellkörper dicht den Gefäßwänden anliegen", sodaß diese „oft auf lange Strecken ihres Laufes von einer dichten Reihe strahliger Zellen umgeben" sind, „deren Körper fast einen Teil der Gefäßwand auszumachen scheint", gehören hierher. Ich deute sie als *Reihen von Zellleibern*, die nach meinen Beobachtungen an

der weißen Substanz des Großhirns vom Menschen entsprechend *große und lange Abschnitte einer perivasculären Grenzhaut* aufbauen. Auch dort, wo eine Gliazelle mit einem breiten und eine Gliafaser enthaltenden Fortsatz in eine perivasculäre Grenzhaut z. B. übergeht (Fig. 15), wird man nicht darüber im Zweifel sein, daß der betreffende Gliafuß im obigen Sinne zu deuten ist. Ebenso wird solche Entscheidung noch möglich, wenn nur eine schmale eben sichtbare Hülle wie ein blasser Saum die Gliafasern umgibt oder als eine feine verbindende Masse das Gliafaserbündel von dem Zellleib fort bis zum Gliafuß begleitet. Nun gibt es aber viele Gliafasern, die das nicht mehr hinreichend deutlich und sicher zeigen. Zwar finde ich an ihnen auf solchen Präparaten, die die Fasern dunkel gefärbt erkennen lassen, und die außerdem mit alkoholischer Erythrosinlösung oder dem VAN GIESONschen Picrocarmingemisch zur Sichtbarmachung des Protoplasmas der Gliazellen u. s. w. nachgefärbt sind, oft einen gleichmäßig glatten, roten Saum, der sie auf längere Strecken hin einhüllt. Von diesem Saum will ich nicht ohne weiteres behaupten, daß er eine protoplasmatische Hülle oder nur eine die Faser bedeckende Zellhaut sei, da für die *Fasern* natürlich auch eine *Differenzierungsform* und eine kontrastliche Rindenfärbung gelten muß, wie sie bei *Granulis* A. FISCHER als *Spiegeldifferenzierung* nachgewiesen hat. Dagegen ist bei einer *anderen Gruppe von Gliafasern die Existenz einer dünnen vielleicht auch nicht ganz kontinuierlichen Hülle dadurch zu erweisen*, daß eine solche nicht gleichmäßig dünn und homogen ist, sondern *eckig und zackig sowie stellenweise matt gekörnt* erscheint. Derartige Hüllen oder Besätze an Gliafasern fasse ich also als *reduzierte Reste* eines ursprünglich vollständig vorhanden gewesenen Protoplasmastückes auf, wie es die embryonale Gliazelle im Beginn ihrer Faserbildung noch erkennen läßt. Bei den völlig glatt aussehenden Fasern wäre die Reduktion bis auf den Fuß selber eingetreten, während sie bei der ersteren Form überhaupt nicht erfolgt ist, wo ein kurzer, zwischen Zellleib und Grenzhaut ausgespannter Fortsatz eine dünne, aber kontinuierliche Hülle um seine Fasern oder sein Faserbündel erkennen läßt. Von ihr endlich würde sich ein rein protoplasmatischer Fortsatz nur noch durch das Ausbleiben einer Faserbildung unterscheiden.

In diesen Beobachtungen sind die engeren Gründe dafür enthalten, daß ich die das fertige Zentralnervensystem abschließenden

Grenzhäute *Membranae limitantes Gliae superficialis et perivascularis* benannt habe. Daß ich sie den *Zellmembranen gleichwertig* erachten und ihre Gesamtfläche also aus einer Summe von Gliafüßen im obigen Sinne zusammengesetzt bezeichnen kann, wird aus jener Analyse der Gliafüße selber hinreichend begründet erscheinen müssen. Hinzukommen Beobachtungen von Flächenbildern, die bei geeigneter Fixierung und Färbung die ganze Grenzmembran als solche kenntlich machen. Bevor ich dieselben näher schildere, gebe ich eine kurze Übersicht, in welcher Weise bisher der *Begriff einer gliösen Grenzmembran* gewürdigt worden ist.

Identisch ist die *Membrana Neurogliae* mit der subpialen resp. perivasculären *Endothelmembran GIERKES*[48]), der sie nur irrtümlich der Pia mater zurechnete und für homogen, strukturlos und aus platten Zellen zusammengesetzt erklärte, obgleich er sie doch mit vielen feinen Fortsätzen der Gliahülle resp. der Gliazellen durch eine fußartige Verdickung verbunden sah. Ebenso entspricht ihr die *BERGMANNsche Membrana limitans*[49]) am Kleinhirn, deren Zusammenhang mit den Füßen der BERGMANNschen Fasern bereits gegen eine piale Natur sprach. Für die Erkenntnis der Membr. gliae perivascularis kommen die Beobachtungen GOLGIS (1885) in Betracht, welcher sie zuerst aus der *Vereinigung von Gliazellansätzen abzuleiten* versuchte. Seine Angaben sind[50]) (S. 161): „Der Ansatz geschieht in der Regel mit einer Ausbreitung, welche bisweilen von kegelförmiger, wohl begrenzter Gestalt, bald sehr dünn und ohne deutliche Grenze, so daß man fast sagen möchte, sie bilde ein perivasculäres Häutchen. An den Kapillaren und kleinen Arterien, welche keine bestimmte Adventitia besitzen, scheint der Ansatz direkt an die Endothelwand der ersteren und an die dünne Muskelhaut der zweiten zu geschehen. Auch in diesen Fällen scheint aus der Gesamtheit der Ausbreitungen der Ansätze der Zellfortsätze eine zusammenhängende Bekleidung hervorzugehen, welche der Gehirnwand unmittelbar aufliegt und eine Art von weiterer Adventitia darstellen könnte.“ Zwar haben schon HENLE und MERKEL[51]) unter der Pia eine feine, aus gekreuzten Bindegewebsfasern bestehende Grenzmembran beschrieben gehabt, welche beim Kleinhirn die BERGMANNsche Membran u. s. w. bilden soll, und von welcher feine Fäserchen in der Gehirnmasse eindringen; zwar hat auch ROTH (1867) an der Pia mater und an Blutgefäßen des Gehirns überall feinste Radiärfortsätze mit

trichterförmiger Abgangsstelle beschrieben gehabt und sie am Kalbshirn als derbere Fäden mit sternförmigen Zellen verbunden gesehen, ebenso wie BOLL (1873) die adventitiellen Scheiden der Hirngefäße mit den zottenartig sie bedeckenden, gestielten Ansätzen gewisser DEITERSscher Zellen (der BOLLschen Pinselzellen) im Sinne der ersten kurzen Angabe GOLGIS (1870 und 1871—2) besetzt gefunden, — zu der Auffassung, daß hier eine *besondere Grenzhaut* vorliegen könne, sind alle zusammen nicht vorgedrungen. Insbesondere gebührt also GOLGI außer GIERKE das Verdienst, die *Erkenntnis der außerordentlich wichtigen Beziehungen der Glia zum Gefäßapparat des Gehirns* angebahnt zu haben. Was dagegen die Bemerkungen GOLGIS über die entsprechende Oberflächenhaut des Gehirns anbetrifft, die speziell auch gegen die BERGMANNsche Membran gerichtet waren, so halte ich sie für unrichtig. Sein Hinweis z. B., daß die Oberfläche des Kleinhirns nur von einer dünnen Schicht leicht erkennbarer Gliazellen gebildet werde, die im Großhirn nur eine besondere Stärke habe, enthält doch zunächst keine Gründe, ob eine Grenzhaut vorhanden ist oder nicht. Dann hat u. a. J. SCHAFFER[53]) dargelegt, daß *von der Gliahülle* GIERKES, die aus cirkulären und longitudinalen Fasern bestehe, eine *radiäre Faserschicht* unterschieden werden müsse, welche mit radiären Faserenden in eine *Grenzmembran* übergehe, die die oberflächliche Pia sowohl wie die Adventitia der eindringenden Gefäße abschließe. Dadurch sei „in der Tat Glia und Nervensubstanz, die ursprüngliche ectodermale Anlage, scharf gegen die sekundär eingewucherten mesodermalen Elemente abgesondert." In besonderer Weise hat dann RENAULT[53]) den Nachweis einer Oberflächenhülle des Rückenmarkes bei Petromyzon geführt, indem er nach dem Vorgange von SCHELSKE, der an der Retina des Menschen mit Silber die Zusammensetzung der inneren Grenzhaut aus den Fußenden der MÜLLERschen Stützformen nachwies, die Oberfläche jenes Rückenmarks mit einem Pikrin-Osmium-Silbergemisch behandelte und zahlreiche abgegrenzte Felderchen beobachtete. Jedes Feld ist mit den benachbarten verkittet und entspricht dem Ansatze einer Neurogliafaser. Die zahlreichen kleinen Felder sind durch die entsprechenden Neurogliafasern wie kleine Basalplatten mit zahlreichen Neurogliazellen in Verbindung. RENAULT schließt, daß erstens die Neuroglia ectodermal ist, ihre Zellen wie Epithelzellen sich verhalten, und die WEIGERTsche

Lehre von der Unabhängigkeit der Gliafasern nicht richtig sein könne. Auf dem internationalen medizinischen Kongreß zu Paris (1900) hat dann RENAULT in der Diskussion zu dem MARINESCOschen Vortrag (Du rôle de la nevrologie dans l'évolution des inflammations) noch hierzu hervorgehoben, daß er gleiche Verhältnisse auch bei Säugetieren gefunden habe und weiter überzeugt sei, daß die sogenannte Lymphscheide der Gefäße eine gleiche Bildung besitze und nur als eine Umbiegung jener oberflächlichen Lamina des Zentralnervensystems zu bezeichnen sei. Endlich hat E. MÜLLER[18]) mit einer eigenen Methode ebenfalls nachgewiesen, daß den WEIGERTschen Befunden entgegen die Gliafasern ein besonderes Ende haben. Seine Befunde bei Myxine sind: „Die Gliafasern endigen, ganz wie die Ependymfasern, an der Peripherie des Markes mit kleinen, ungefärbten Füßen, die dicht an einander gestellt eine geschlossene Grenzschicht gegen die Pia bilden. Solche Endigungen findet man noch in dem Innern des Markes, indem die kleinen Füßchen der Gliafasern sich an den hier vorhandenen Gefäßen befestigen. Aus diesen sich regelmäßig zeigenden Bildern der Endigungsweise einer Menge in jedem Schritt verfolgbarer Gliafasern kann man wohl mit Recht schließen, daß alle Gliafasern in der gleichen Weise mit ungefärbten Füßen an der Peripherie oder an den Gefaßen endigen." Hierher gehört endlich auch die Angabe von KURE[14]), wonach die Gliazellen durch protoplasmatische Ausläufer mit der Gefäßwand resp. dem Endothel direkt verbunden sind (nach d. Referat im Neurol. Centralbl. 1902 S. 1017).

Meine folgenden Beobachtungen bestätigen zunächst jene verschiedenen Angaben über Grenzflächen und Gliafüße im Sinne GOLGIS; ich erweitere sie für jene Säugetiere und den Menschen insbesondere, für welche bekanntlich WEIGERT auf Grund seiner einseitigen Methodik zu einem die GOLGIschen Anschauungen völlig ablehnendem Resultat gekommen war. Wenn also WEIGERT auf seinen Präparaten konstatierte, daß die Neurogliafasern „niemals etwas von jenen konischen oder flaschenförmigen Erweiterungen" wie auf den GOLGIpräparaten erkennen liessen, und zu dem Schluß kam, daß seine elektiven Färbungen als die „maßgebenden" jene Füße nur als eine „Kittsubstanz" oder als ein „Kunstprodukt" nachwiesen, so ist dies unrichtig und unbegründet. Daß außerdem WEIGERT noch jene aufgesplitterten Gliafaserenden übersehen resp.

bei seiner radikal entfärbenden Methode nicht dargestellt bekommen hat, geht aus seiner Bemerkung hervor, daß „die Gliafaser bis zu ihrem Ende so schlank und gleichmäßig" bleibt wie früher. Einen Einwand, daß jene feineren Endfäserchen in Folge einer *anderen chemischen Natur* entfärbt würden, und also nicht mehr zum Begriff einer Gliafaser gehören, lasse ich nicht gelten, seitdem ich weiß, wie leicht und schnell solche dünneren Fadenbildungen bei differenzierten Färbungen verschwinden.

In Fig. 33b und Fig. 35 resp. 36 (linker Rand) habe ich *Flächenbilder der gliösen Grenzmembran der Oberfläche* dargestellt. Fig. 33b zeigt ihre Felderung am Kleinhirn des Kaninchens, Fig. 35 am menschlichen Großhirn. Ich füge hinzu, daß auch das menschliche Rückenmark und das menschliche Kleinhirn mir gleiche Ansichten gegeben haben. Fig. 33b zeigt besonders jene aufgesplitterten Gliafaserenden (Enden der Bergmannschen Fasern) und ihre ungefähr radiäre Anheftung an die freie Grundfläche des Gliafußes. Die einzelnen Fußflächen sind durch Kittlinien verbunden, welche hier infolge der Heidenhainschen Methode (bei bestimmter Fixierung) schwarz gefärbt geblieben sind. Die *Grenzhaut selber*, welche im Durchschnitt wie eine feine glatte Linie erscheint, die nach innen in zahlreiche konische Zapfen sich verlängert, sieht im Flächenbild mattgekörnt aus, wenn man sehr wenig oder gar nicht differenzierte Präparate untersucht. Auch an den großen Gefäßen in den oberflächlichen Hirnschichten habe ich eine gleiche Felderung gesehen. Fig. 41 zeigt sie an einer Vene aus der menschlichen Hirnrinde. An den Kapillaren habe ich deutliche Flächenbilder bisher nicht darstellen können. Die Grenzhaut und ihre Kittlinien sind offenbar so fein, daß sich keine sicheren Differenzierungsbilder durch solche Methode gewinnen lassen. Den Übergang der oberflächlichen Felderung in diejenige der perivasculären Gliamembran zeigt Fig. 35 an der menschlichen Großhirnrinde in Flächenansicht.

Wie oben angegeben hat Renault aus seinen Beobachtungen über die Bildung seiner Lamina vitrea zwei Schlüsse gezogen, daß erstens die Gliazellen epithelialer Natur sind, und daß zweitens die Neuroglia allgemein deshalb auch ein rein ectodermales Gewebe bedeute. Gegen den ersten Satz habe ich keine Bedenken; aber für den zweiten ließe sich einwenden, daß zu irgend einer Zeit der Entwickelung der Gliagrenzhaut ein Durchbruch vom

mesodermalen Gewebe erfolgen könne. Dies auszuschließen, sind also besondere embryologische Beobachtungen notwendig.

Wie ich Anfang dieses Kapitels über die marginale Glia und ihre Entwickelung gezeigt, *dringen mit den einwachsenden Gefäßen mesodermale Zellen in die eigentliche Gehirnsubstanz im engsten Sinne des Wortes nicht ein*, da längs des Blutgefäßweges *sofort eine Ausbildung von feinsten Grenzen mit radiär gespannten Zellfäden einsetzt*, wodurch also auch im Inneren des Hirnparenchyms seine eigene Masse durch eine innere Oberfläche von Anfang an gewahrt bleibt. Außerdem habe ich nirgends eine eigentliche Vermischung von Zellen der wachsenden Gefäßsprossen mit den eigenen des Gehirns finden können. Man könnte einwenden, daß dann vielleicht die *Durchbruchsstelle der Nerven an der äußeren Oberflächengrenzhaut* Bedingungen für das Einschlüpfen von mesodermalen Elementen herbeiführten. Daß dies nicht der Fall, zeigen Fig. 26 und Fig. 27, welche an der Durchbruchsstelle eines *motorischen wie sensiblen Nerven eine Zurückhaltung, eine Art von Aufstauung der mesodermalen Zellelemente* erkennen lassen. Bemerkenswert ist hierbei, daß die Neuroblastenfortsätze mit ihrer ectodermalen Eigenschaft glatt die Grenzhaut durchsetzen können oder siebartig durchlöchern, ohne jene Reaktion herbeizuführen, die das Mesoderm beim Eindringen der Gefäße verursacht. Jenen primären Durchbruchsstellen entsprechend zeigt nun auch am fertigen Zentralnervensystem das *betreffende Stück eines Gehirn- oder Rückenmarksnerven* eine *deutliche Grenze*, an der erst die *periphere Bildung von Schwannschen und Henleschen Scheiden* ansetzt, mit dem Unterschied nur, daß diese mesodermale Einhüllung und Verbindung der einzelnen Nervenfasern *nicht mehr genau wie beim Embryo in der unmittelbaren Höhe der Membrana limitans superficialis* liegt, sondern ein wenig in den *peripheren Teil* des betreffenden Nerven verschoben ist. Eine Tatsache, die bereits Schaffer und Weigert hervorgehoben haben, indem sie eine Neuroglia im Anfang eines abgehenden Nerven fanden. Auf ein *späteres Nachdrängen der zentralen Neuroglia* durch jene frühen Durchbruchsstellen infolge von stärkerem Wachstum und der besonderen Ausbildung von steiferen Fasern wird diese *periphere Vorlagerung der Gliascheiden* zurückgeführt werden müssen. Grenzt man allgemein das Zentralnervensystem nach seiner Membrana limitans Neurogliae ab, so würde also nach allen diesen Ausführungen sein wirkliches Ende

keineswegs mit der glatten und ebenen Oberfläche zusammenfallen, die man als solche sieht. Sie reicht einerseits überall in die Tiefe längs seiner Blutgefäßwege und reicht andererseits dort vielfach wie kleinste Buckel nach außen, wo seine Nervenwurzeln liegen. Beim N. acusticus des Meerschweins und der Maus ist sie z. B. so weit ausgedehnt, daß erst innerhalb des Meatus acusticus internus selber das Ende des Zentralnervensystems durch eine quere Grenzhaut gegeben ist.

Die *marginale Neuroglia* ist der Tiefe des Parenchyms zu noch durch eine *besondere Schicht der Glia charakterisierbar*, welche bereits in jenen Beobachtungen von HENLE und MERKEL, ROTH, GOLGI, BOLL, GIERKE, SCHAFFER u. a. enthalten ist. Es ist dies jene Zone, welche den *Zusammenhang der tieferen Gliaelemente mit der Grenzhaut selber* bedingt und durch eine ungefähr *radiäre Anordnung von Gliafüßen und Gliafasern* oder auch reinen Protoplasmafortsätzen resp. einem Gliareticulum mit radiär gespannten oder gefaserten Balken ausgezeichnet ist. Durch jene oberflächlichen Gliazellen, die direkt der Grenzhaut eingelagert sind, wird dieser allgemeine Charakter der Grenzschicht nicht geändert. Ich will sie als *Grenzschicht der Glia* bezeichnen. Mit gewissen Ausnahmen, die z. B. am Kleinhirn stellenweise bestehen, bildet sie sonst eine deutlich gegliederte und besonders von einer erst unter ihr gelegenen *Rindenschicht* (FROMMAN, WEIGERT, Gliahülle GIERKES) unterschiedliche Zone. Fig. 29—32a zeigen diese Verhältnisse an der Oberfläche vom Rückenmark (Kaninchen und Mensch), Fig. 33 beim Kleinhirn (Kaninchen), Fig. 34—38 beim Großhirn, Fig. 38—43 im Bereich der inneren Gefäßoberfläche.

Auf den von WEIGERT gegebenen Abbildungen der Neuroglia und des von ihr bedingten Abschlusses der zentralen Gehirnmasse ist *weder von einer Gliagrenzhaut oder von jenen Gliafüßen noch von einer Grenzschicht der Neuroglia* etwas zu sehen. WEIGERT hat nur eine *Rindenschicht* abgebildet, die schon FROMMANN[65]) und GIERKE (Gliahülle) bekannt war. WEIGERT faßt sie als letzte und äußerste Grenze des Gehirns zur Pia und der von ihr ins Innere eindringenden gefäßführenden Septen auf. Daß dies kein wirklicher Abschluß sein kann, sondern nur ein einseitiges Resultat starker Entfärbung zeigen meine Beobachtungen. Über jene *Rindenschicht* gibt WEIGERT folgendes an (S. 146): „Die Hauptmasse der Fasern pflegt mehr oder weniger schief tangential zu verlaufen, es kommen aber noch außer vertikalen auch radiäre Fasern vor, und da, wo stärkere Fortsätze der Rindenschicht in die Tiefe der weißen Substanz eindringen, bilden sie oft nach innen konvergierende Bündel, die sich in bald zu erwähnender Weise weiterhin wieder auflösen. Gegen die Pia mater zu ist normaler Weise die Rindenschicht scharf abgesetzt, doch kommt es oft genug vor (Tafel I Fig. 3), daß Faserbündel wie die Haare einer Bürste über die sonst glatte Oberfläche der Rindenschicht herausragen, wie dies auch FROMMAN bermerkt hat."

Diese „Faserbündel" entsprechen nach meiner Meinung entweder einzelnen stark abgehobenen und auch gefärbt gebliebenen Teilen meiner Grenzschicht oder gewissen hernienartigen Wucherungen der marginalen Glia in das Piagewebe hinein, wie es von SCHAFFER z. B. gesehen worden und auch auf meinen Rückenmarksquerschnitten eines 25jährigen Hingerichteten vereinzelt vorhanden ist. In einem zweiten Fall eines jüngeren Hingerichteten waren stellenweise im unteren Lendenmark die Piabündel förmlich eingefangen von solchen flächenhaften Hervorquellungen der Glia. Ob dies vielleicht auf unvorsichtiges Herausnehmen des Rückenmarkes zurückzuführen, vermag ich nicht zu entscheiden. Im übrigen aber führe ich sonst die „scharfe Absetzung" jener Rindenschicht mehr als ein einseitiges Resultat auf die WEIGERTsche Methode zurück, da nach meinen Erfahrungen bei starker Differenzierung sich die radiäre Grenzschicht u. s. w. leichter entfärbt wie die Rindenschicht. Vielleicht aber stellt sich noch heraus, daß auf den WEIGERTschen Präparaten eine starke Anpressung der Grenzschicht samt der Membrana limitans Gliae an die Rindenschicht als eine Folge seiner Fixierungsmethode entstanden und deshalb von WEIGERT übersehen worden ist. Auf meinen Präparaten erscheint die gliöse Grenzhaut ein wenig segelartig abgehoben und von innen her durch die Füße jener Grenzschicht gehalten.

In ähnlicher Weise wie beim Rückenmark ist auch am Hirnstamm und an der Oberfläche des Großhirns die Abgrenzung der Neuroglia gegeben. Nur an der Kleinhirnrinde fehlt zum großen Teil eine oberflächlichere Lage von Gliafasern und Gliazellen und damit die deutliche Anordnung einer Rindenschicht. Es hängt hier die oberflächliche Grenzhaut vorwiegend mit den *tief aus der Molekularzone der Kleinhirnmasse emporsteigenden Gliafasern*, den sogenannten BERGMANNschen Fasern (Fig. 33a, b) zu-

sammen, deren zugehörige Gliazellen hauptsächlich in der Höhe der PURKINJEschen Zellreihe gelegen sind. Die von GOLGI seinerzeit gegen solche Grenzeinrichtungen geltend gemachten Gründe können dieselbe nach meiner Meinung nicht umstoßen. Aber es kommt hinzu, daß die vielfach bei jungen Tieren von einigen Wochen noch als eine unmittelbar unter der Grenzhaut gelegene Zellreihe in reduzierter Form bestehen bleibt und zu Gliazellen reift, die an der Oberflächenvergrößerung dieser Grenzhaut beim Wachstum des Kleinhirns sich beteiligt. Beim erwachsenen Menschen (Hingerichteter von 25 Jahren) finden sich solche Zellen nur noch ganz vereinzelt; wenige von ihnen zeigen eine Entwicklung spärlicher Gliafasern, die flächenhaft verlaufen und nach einiger Strecke zur Grenzhaut umbiegen, wo sie mit einem Gliafuß endigen.

Zum Unterschied von der Kleinhirnoberfläche ist also die *Grenzhaut des Rückenmarks und Hirnstammes viel fester gehalten und gebaut*, infolge zahlreicherer Gliafüße sowohl als auch durch Ausbildung einer besonderen und mächtigen Rindenschicht, *die jenen tieferen und sie radiär durchsetzenden Gliafasern vielfach umliegende Fasern aus ihrer eigenen Zone beigibt.*

Zu der marginalen Glia dieser Regionen gehört auch noch außer jenen Teilen etwas, was auf den angegebenen Figuren vom Rückenmark des Menschen nicht dargestellt worden ist, und was auf so stark differenzierten Präparaten nur noch durch eine ausreichende Kontrastfärbung wieder zum Vorschein zu bringen ist. Es ist dies eine *netzartig geformte Masse*, welche den Gliafasern resp. den reinen Protoplasmafortsätzen von Gliazellen dieser Zone wie *breitere Balken oder dünnere und mehr oder weniger kugelig gewölbte Membranen zwischengespannt ist.*

Auf den Fig. 34a bis c und Fig. 36 kommt dies zur Ansicht. Zwar stellen diese Abbildungen die Eigentümlichkeiten der kortikalen Grenzglia bei bestimmter Differenzierungsgröße dar. Ich füge hinzu, daß ich andererseits Präparate von der Großhirnrinde besitze, wo die Differenzierung soweit getrieben ist, daß sie wie die Rückenmarksbilder jene einfache aber unvollständigere Anordnung der einzelnen Elemente geben.

Jene Figuren (34 und 36) zeigen also, daß es an der marginalen Glia *innerhalb der eigentlichen Grenz- und Rindenschicht (beim Cortex kommt auch noch die unmittelbar tiefer gelegene Zone hinzu)* zu einer Ausbildung von zahlreichen Netzbalken oder auch mehr und weniger abgeschlossenen und längs der einzelnen Gliafasern und der reinen Gliazellfortsätze ausgespannten Membranen kommt. Es besteht mit anderen Worten bereits unter der Grenzhaut selber gewissermaßen ein teilweise *kammerartiger Bau*, in dessen Wänden zahlreiche, teils flächenhaft, teils zur Grenzhaut emporstrebende Gliafasern wie innere Stützen enthalten sind. In welchem Umfang dieser eigentümliche Bau ausgeprägt ist, kann ich nicht angeben; vor allem auch nicht, wie weit er der Tiefe zu vorherrscht, und ob er in eine andere Formation hier übergeht.

Dagegen habe ich eine Reihe von Beobachtungen darüber gemacht, wie *die Größe oder Weite der Maschen* oder auch der Kammern innerhalb gewisser Bezirke der marginalen Glia *allgemein zu beurteilen* ist. Durch verschiedenartige Fixierung von Serien dünner Scheiben bin ich zu dem Resultat gekommen, daß es *größtenteils Wirkung eines bestimmten Fixierungsmittels ist, wenn man jene Maschen weit und deutlich* oder *ganz eng und kaum als solche zu erkennen* erzielt. Dazu kommt, daß *dasselbe Fixierungsmittel an verschiedenen Regionen eine ungleiche Wirkung* hat. So preßt z. B. das Alkohol-Chloroform-Eisessiggemisch die marginale Glia des Rückenmarks eng zusammen, während sie diejenige an der Großhirnrindenoberfläche deutlich maschig konserviert. Dasselbe Gemisch gibt endlich bei beiden Abschnitten gute Konservierung der Grenzhäute und Endfüße, während es beim Kleinhirn häufig eine Zerstörung der BERGMANNschen Membran verursacht. Für die Oberfläche des Kleinhirns wirkt wiederum besser konservierend meine Chrom-Formalin-Eisessigfixierung, die auch am Großhirn brauchbare Bilder erzeugt. Alkohol, MÜLLERsche Lösung geben gleichmäßig schlechte Resultate, Salpetersäure dagegen bessere. Zu den Fixierungsmitteln endlich, die *sehr eng zusammengepreßte Bilder* erzeugen, gehört die ALTMANNsche Chromosmiumfixierung, die außerdem die Grenzhaut ungenügend konserviert, sowie die WEIGERTsche Chromalaun-Kaliumbichromicumlösung. Am Rückenmark habe ich engere, aber noch deutliche Bilder an der Oberfläche durch das Sublimat-Osmiumgemisch erhalten. Welche Weite in den Teilen der marginalen Neuroglia dem normalen Zustand am meisten entspricht, vermag ich also nicht zu entscheiden, zumal da ich nicht genauer anzugeben weiß, ob und wie sehr örtliche Gewebsverschiedenheiten oder ungleiche Flüssigkeits-

füllungen innerhalb des Parenchyms hierbei beteiligt sind. Bei der Besprechung der perivasculären Räume komme ich zum Schluß dieses Kapitels nochmals hierauf zurück.

Wie die marginale Glia der äußeren Oberfläche ist nun auch vielfach diejenige der Tiefe im *Umkreis der Gefäße* zusammengesetzt. So finde ich im Rückenmark überall an den *Septen der Pia*, mögen sie feiner oder gröber sein, sowohl eine *Grenzschicht* wie eine durch fußartige Ansätze von zahlreichen Gliafasern gebildete *gliöse Grenzhaut* ausgeprägt, welche *perivasculärer* Natur ist, da in diesen bindegewebigen Septen fast überall gröbere oder feinere Gefäße liegen. Erst in etwas weiterer Entfernung liegt zu *ihren beiden Seiten je eine Rindenschicht* (s. Fig. 32), mit denen zusammen erst ein FROMMANscher *„Stammfortsatz“* gebildet wird. Ähnlich schließt sich das Neurogliagewebe im *Septum posterius* ab, sodaß je nach der wechselnden Breite dieser pialen Fortsetzung und seiner Tiefe eine M. limitans Gliae ausgeprägt ist oder fehlt. Wodurch es sich erklärt, daß die *Hinterstränge* des Rückenmarks ungleichmäßig in Länge und Tiefe von jener Grenzhaut getrennt sind. Zum Unterschied hiervon erscheinen die *Vorderstränge* des Rückenmarks in seiner vorderen Längsspalte auch der ganzen Länge nach durch eine gleichmäßiger tief und breit eingetriebene Masse von Gefäßen auseinandergedrängt. In der Tiefe der vorderen Spalte, teilweise auch dem hinteren Septum zu, läßt sich zudem noch etwas beobachten, was an anderen Stellen des Gehirns oder auch des Rückenmarks nicht so leicht oder überhaupt nicht mehr gelingt, daß nämlich der Grenzhaut die Füße der an Gliafaserbündeln reichen Fortsätze der Ependymzellen angeheftet sind. Vom Rückenmark des Kaninchens zeigt Fig. 1 und 2 den Ursprung dieser Gliafasern aus dem Ependym. In der Abbildung der gleichen Gegend eines menschlichen Rückenmarkes (25jähriger Hingerichteter mit obliteriertem Zentralkanal) stammen sie aus den faserreichen, gewucherten Ependymzellen (Fig. 31). Im übrigen finde ich an den *Blutgefäßen* des *Hirnstammes*, sowie der weißen Substanz von *Großhirn und Kleinhirn* mehr *schwächere* Rindenschichten. Nur stellenweise zeigen die groben Gefäße bei ihrem Eindringen in die Tiefe eine ihnen folgende stärkere Rinde von verfilzten Gliafasern, die an die gleiche Bildung längs der pialen Septa im Rückenmark erinnert. Weiter aber finde ich oft nur noch eine radiäre *Grenzschicht*. Die gröberen Kapillaren der Rinde zeigen solche Begrenzungen. Die

feinsten Kapillaren sind nur noch durch eine sehr zarte Grenzschicht abgesetzt, die außerdem zum größten Teil nicht mehr von *Gliafasern*, sondern *von feinen Protoplasmafäden*, den Ausläufern der Gliazellen resp. den Anteilen des Gliareticulum, gebildet werden (Fig. 41). Insofern ist also allgemein die *radiäre Grenzschicht* eine *konstante* perivasculäre Zone. Im Vergleich mit ihr bildet eine von einzelnen Gliafasern (Fig. 37, 32a) zu groben Faserzügen wechselnde Rindenschicht nur eine *accessorische* Einrichtung, welche die Grenzschicht der Gefäße mit ihren radiären Balken und ihren Gliafüßen verstärkt. Somit sind auch die *letzten Beziehungen der Neuroglia zu den Blutgefäßen der grauen oder weißen Substanz* durch eine Reihe von rings um das Gefäß gestellten Gliafüßen vermittelt, deren Form mit denen in der oberflächlichen Grenzschicht beschriebenen übereinstimmt. Zum Teil hängen diese Füße mit *radiären Gliafasern* zusammen, zum anderen Teil auch mit solchen, welche *der Länge nach das betreffende Gefäß* begleiten und dann unter mehr oder weniger starker Umbiegung erst ihren Gliafuß abgeben, was infolgedessen schwieriger und weniger umfangreich zu beobachten ist. Aus einer Summe von Gliafüßen entsteht dann rings um das Blutgefäß resp. um seine adventielle Hülle eine besondere Scheide, eben jene *M. lim. Gliae perivascularis*, die nach meinen Beobachtungen eine *vollständige Grenze für das betreffende Gefäßrohr samt seiner adventiellen Scheide zu der eigentlichen Masse des Zentralnervensystems bedeutet*. Die von LLOYD ANDRIEZEN[56]) und von WEIGERT beschriebenen, aus *verfilzten Gliafasern bestehenden Begrenzungen der Blutgefäße* (OBERSTEINER nennt sie geradezu „Begrenzungsmembranen“) geben somit den *wirklichen Abschluß des Gehirns zu seinen Gefäßen* noch nicht an. Denn es fehlt die letzte Analyse der *radiären Fasern*, die *keineswegs ausschließlich in eine Längsrichtung umbiegen*, sondern im *Sinne* GOLGIS *mit Gliafüßen jene besondere perivasculäre Grenzhaut bilden*. Sonst ist die allgemeine WEIGERTsche Bemerkung, daß die Blutgefäße „für das Zentralnervensystem etwas genau so Fremdes, wie die eigentliche Pia mater“ sind, und daß „die Grenze des Nervengewebes gegen ein Gefäß nichts anderes als eine *innere* Oberfläche“ bedeutet, durchaus zutreffend, deren Wert auch durch die Entwickelungsgeschichte der Vascularisierung der Gehirnmasse, wie oben gezeigt, in besonderer Weise beleuchtet wird.

Die Untersuchung und Darstellung der *Membrana limitans*

Gliae perivascularis stößt auf noch größere Schwierigkeiten als diejenige der oberflächlichen Grenzhaut, da sie sehr leicht infolge ihrer Feinheit bei der Fixierung zerstört oder von den Gliafäden abgerissen wird. Hinzukommt, daß die verspätete Tiefenwirkung einer Fixierungsflüssigkeit im Innern eines Stückes Hirnsubstanz eine ungenügende Konservierung aller perivasculären Grenzhäute bedingt. Für das menschliche Zentralnervensystem leistet noch das Alkohol-Chloroform-Eisessiggemisch die gleichmäßigste Fixierung, wenn man die Stücke klein nimmt und vorsichtig einbettet; doch ist auch sie nicht frei von gewissen Verzerrungen, auf die ich unten noch eingehen werde. Alkohol und MÜLLERsche Lösungen geben durchweg schlechte Konservierungen. Fig. 43 zeigt die Alkoholwirkung. MÜLLERsche Lösung oder Chromsäurelösungen erhalten wenigstens nur Reste jener Grenze. Durch den Zusatz von Eisenalaun zur Lösung von Kal. bichromic. habe ich den Vorzügen dieser Flüssigkeit für die Markkonservierung eine Eigenschaft verleihen können, wenigstens für die Oberfläche und die weiße Substanz, die Gliafüße und ihre Endhaut gut zu konservieren. Doch hat es den Nachteil von Einbettungsschrumpfungen. Für die graue Substanz ist sie dagegen unbrauchbar, weil solche Schrumpfungen im Bereich der Nervenzellen (perizelluläre Lymphräume) zu groß ausfallen. Zum Unterschied von Fixierungsmitteln, welche zwar geringe Schrumpfungen und eine besonders deutliche Erkennung der Gliafüße und ihrer Grenzmembran geben, habe ich auch solche angewandt, welche vollkommen frei von Schrumpfungen sind und also *alle Elemente der weißen und grauen Substanz in enger Pressung* zeigen, sodaß sie in dieser Hinsicht vielleicht den besseren Situs zeigen. Vorläufig aber haben sie den Nachteil, die hier in Frage stehenden Gewebsgrenzen der Glia nicht sichtbar zu machen, da es an einer *elektiven Färbung der Gliafüße und ihrer Endhaut* fehlt, was bei ihrer dünnen Masse ganz hervorragende Schwierigkeiten hat. Ich gehe auf solche Fixierungsmethoden deshalb hier nicht näher ein, und begnüge mich zu konstatieren, daß auch bei ihnen stellenweise und *angedeutet* jene Endbeziehungen zu sehen sind, daß sie also wenigstens diese wichtigen Abschnitte der Neuroglia nicht von vornherein zerstören.

Nach diesen allgemeinen Auseinandersetzungen über den ungleichen Wert verschiedener Fixierungen für die radiäre Grenzschicht der Neuroglia gehe ich auf die besondere Frage nach den

Lymphgefäßeinrichtungen im Innern des Zentralnervensystems ein. Nach der einen Meinung enthält die *perivasculäre Zone* der Gehirnmasse die besonderen und eigentlichen *Lymphwege des Gehirns* (HIS), nach der anderen Auffassung (GOLGI, BOLL, RETZIUS) handelt es sich nur um *Schrumpfungsräume*. Hinzukommt, daß nach HIS *diese Räume nicht von „fädigen Bildungen“ durchsetzt* sind, während andere (ROTH, HENLE, MERKEL, GIERKE, SCHAFFER, BINSWANGER und BERGER) sie von einem *Faserwerk ausgestützt* finden und damit eine Parallele zu dem Lymphsinus mit seinem reticulären Gewebe finden. Eine Kritik dieser Anschauungen, die wohl nicht stärker von einander abweichen können, gebe ich im folgenden, wobei ich mich *hauptsächlich auf jene Beobachtungen einer Gliagrenzhaut*, ihrer *entwickelungsgeschichtlichen Bedeutung* und die von der *Fixierungsweise abhängige Darstellung einer radiärgefaserten Grenzschicht der Glia* stütze. Auf eine Diskussion der Resultate einer Injektion dieser Lymphwege werde ich dagegen hier nicht eingehen, da meine Versuche hierüber noch nicht abgeschlossen sind und vor allem auch noch nicht den Teil ganz erledigt haben, der von BINSWANGER und BERGER durch Versuche über die resorbierenden Eigenschaften der Neuroglia und den Transport unlöslicher Bestandteile innerhalb ihrer Maschen angefangen worden ist.

Meine obigen Ausführungen über den Bau der marginalen Neuroglia zeigen zunächst, daß man in *Rücksicht auf jene Grenzhaut und ihre fußartige Befestigung am Gewebe der Glia* nicht mehr *ohne weiteres* jene klaren Löcher oder Kanäle, in denen die Blutgefäße des Gehirns oder Rückenmarks bei Schnittbetrachtung drinliegen, als natürliche resp. *natürlich weite* Räume für die Zirkulation einer lymphatischen Flüssigkeit auffassen darf. Ist der *Raum um das Gefäß* von *radiären Fasern oder groben Balken und Netzwerken durchsetzt*, so würde die *Grenzmembran der Glia*, sofern sie überhaupt erhalten ist, nach meinen Erfahrungen und Beobachtungen dem *Gefäß resp. seiner adventiellen Scheide angepreßt anliegen* (Figg. 38, 39a, b, c, 42). Finden sich dagegen *keine radiären Faserzüge* oder wandständige Reste von Füßen, so braucht nicht immer die Grenzhaut der Glia als eine aufgeweitete Hülle der *äußeren Wand* der klaren Lichtung anzuliegen, wie wohl mitunter zu sehen ist. Denn es kann auch bei bestimmter Fixierung als eine reine Folge derselben der *ganze Apparat von Radiärfasern, Füßen und Grenzhaut zerstört* sein. Ein Mittelding zwischen beiden

illustriert Fig. 43, wo nach Alkoholfixierung (und sekundärer Paraffineinbettung) die Grenzhaut dem Gefäß angedrückt und nur ihre Haltefasern zerrissen und zerstört sind. In jedem Fall wird also erst durch eine genaue mikroskopische Beobachtung entschieden werden müssen, welche Fixierungswirkung eingetreten ist, d. h. welcher *Anteil* bei der Fixierung die *verschiedene Schrumpfungsgröße der Blutgefäße und der umgebenden zentralen Substanz an der Bildung solcher Zonen und Figuren* hat. Unentschieden muß aber bleiben, ob vielleicht in diesen Abschnitten der marginalen Glia ein größerer Gehalt von transsudierter Flüssigkeit vorhanden ist als in der Tiefe und dadurch eine weitere und besondere Ursache für jene oben gekennzeichneten Unterschiede der Fixierungsflüssigkeiten bedingt ist oder nicht. Alle bisherigen Beobachtungen und Überlegungen reichen hierzu nicht aus. Einer künstlichen Schrumpfung kann die ebenfalls künstliche Wirkung einer stärkeren Zusammenpressung entgegengehalten werden.

Immerhin neige ich der Ansicht zu, daß den engeren Bildern eine größere Wahrscheinlichkeit zukommt, weil nach meinen früheren Erfahrungen die nervöse Substanz wasserreicher ist und sich infolge dessen bei der Schrumpfungswirkung der Fixierung und Einbettung gewissermaßen aus den Maschen ihres Stützgewebes retrahiert. Weshalb ich insofern der Meinung bin, daß die von GOLGI, BOLL u. a. behauptete Ansicht begründet ist, daß die von ROTH und GIERKE als *perivasculäre Lymphräume* angesprochenen Loch- und Kanalbildungen *Schrumpfungsräume* sind. So habe ich sie auch auf meinen Abbildungen bezeichnet. Ob sie andererseits vielleicht noch eine Bedeutung von virtuellen Lymphspalten haben können, darüber halte ich mit meiner Meinung noch zurück.

Jedenfalls *unterscheide ich von ihnen diejenigen Spalten*, welche *zwischen der Gefäßadventia und der Membrana limitans Gliae* liegen, und meine von ihnen, daß sie Flüssigkeitswege sind, welche von der adventitiellen Gefäßscheide her z. B. gefüllt werden können oder auch in diese hinein wieder sich entleeren, sofern die Grenzhaut der Glia eine weitere Diffusion in das eigentliche Gewebe der zentralen Substanz verhindern oder nur erschweren kann.

Ich *identifiziere diese Spalträume*, die also an der *genauen Grenze* zwischen dem *ectodermalen Gehirn* und seiner *mesodermalen Pia mater mit ihren Blutgefäßen und deren inneren Verzweigungen*

liegen und so entstanden sind, mit den *HISschen Räumen*, und behaupte weiter, daß die gegen diese *perivasculären resp. epizerebralen Räume* geltend gemachten Gründe, wonach sie nur künstlich entstandene Schrumpfungsräume sind, nicht stichhaltig sind, weil sie die von ROTH, GIERKE u. s. w. dargestellten Retraktionslücken betreffen.

Daß ich diese *Grenzräume* des Gehirns in meinem obigen Sinne mit den HISschen Räumen identifizieren kann, wird aus Folgendem klar sein. Durch die Freundlichkeit von Herrn Geheimrat HIS habe ich seine Originalpräparate mit den meinigen daraufhin vergleichen können. Auf den HISschen Schnitten erscheint nun zunächst sein Kanalsystem vollkommen klar und *glattwandig*, sodaß es mit den ROTHschen Räumen nicht identisch sein kann. Man könnte auf den Gedanken kommen, daß es sich trotz dieses Unterschiedes nur um künstlich erweiterte und glatt gewordene Retraktionsräume handele, in denen nur infolge der Fixierung das radiäre Gewebe der marginalen Glia u. s. w. vollständig zu Grunde gegangen sei, so wie ich es oben gezeigt habe, zumal da HIS[57]) solche Schnitte seinen Beobachtungen zu Grunde gelegt hat, welche von in Alkohol oder in Chromsäure gehärtetem Material angefertigt worden sind. Nun hat aber HIS angegeben, daß „seine Spalten stets vollkommen glatt begrenzt und von einer nachweisbar verdichteten Substanzschicht eingesäumt" sind. Wenn also jener Einwand richtig sein sollte, müßten doch irgendwo faserige Reste von Glia zu sehen sein. Ich habe vergeblich darnach auf den HISschen Präparaten gesucht. Dazu kommt, daß ich an einigen *dünnen Stellen jener Schnitte*, die eine *Immersionsvergrößerung* zulassen, *eine den betreffenden perivasculären Kanal der Gehirnmasse zu begrenzenden*, ein wenig abgehobenen feinen Saum gesehen habe, von dem ich glaube, daß er eine hier erhaltene Grenzhaut bedeutet. Warum aber zum Unterschied auf meinen Präparaten diese fraglichen Spalträume nur eng erscheinen, vermag ich nicht irgendwie zu entscheiden.

Ist nun überhaupt dieser *Spaltraum zwischen der bindegewebigen Scheide eines Blutgefäßes und der Membrana limitans Gliae perivascularis* oder derjenige, welcher als *HISscher Epicerebralraum zwischen der oberflächlichen Gliahaut und dem Gewebe der Pia mater liegt*, ein Saftraum, der von einer wechselnden Flüssigkeitsmenge ungleich geweitet wäre oder mindestens jederzeit durch das glatte Aus-

einanderweichen der bindegewebigen Gefäßscheide und jener Grenzhaut sich bilden würde? Ein solcher Einwand führt zur Frage, ob nicht das der *Grenzhaut anliegende Gewebe mit ihr selber verklebt* ist, sodaß nicht einmal eine leichte Lösung, wie sie zur schnellen Bildung eines sonst kollabierten Spaltraumes nötig wäre, erfolgen könnte. Damit wäre dann mehr oder weniger auch das *Einheitliche jener Räume* in Frage gestellt.

Ich führe hierzu zunächst von meinen Injektionsversuchen einen am Kleinhirn des Kaninchens an, welcher außer aus jenem Grund auch den Charakter der gliösen Grenzschicht (der BERGMANNschen Membran) als einer *geschlossenen* Haut bestimmen sollte.

Durch *Einstichinjektion einer dünnen Tuschelösung gegen die Unterfläche der M. limitans superficialis habe ich ihre Durchlässigkeit und Festigkeit* zu prüfen versucht. Natürlich wird auf ihrem Wege dahin die Tuschelösung mehr oder weniger künstliche Wege laufen, worauf es aber in diesem Fall zunächst nicht ankommt. Mit einer PRAVAZschen Spritze und einer besonderen konischen Kanüle habe ich unter möglichst leichtem und geringem Druck injiziert und mit der Injektion aufgehört, sobald sich an der Oberfläche eine geringe Menge von Tuschelösung ausbreitete. In anderen Fällen habe ich noch weiter injiziert und dann jene Beobachtung von HIS wieder machen können, daß, sobald einmal eine gewisse Menge von Injektionsmasse an die Oberfläche gedrungen, sie sich plötzlich und auf einmal auf eine weitere Strecke hin ausfließend verbreitet. Die betreffenden herausgeschnittenen Stücke habe ich dann in Alkohol-Chloroform-Eisessig fixiert, welcher sowohl jene Grenzhäute und Gliafüße einigermaßen vollständig zu fixieren vermag als auch die Tusche selbst schnell niederschlägt. Durch Untersuchung einer Reihe von Schnitten habe ich dann die Wege der Injektionsmasse bestimmt. Sie hat sich in dem einen Fall zum Teil in seitlicher Abzweigung von der Injektionsrichtung *unter der M. lim. Gliae superfic.* ausgebreitet gehabt und wie Fig. 45 mit starker Vergrößerung zeigt, sich überall unter jener Grenzhaut und längs jener Gliafüße der emporsteigenden Gliafasern gefangen, während sie *nirgends hier durch die Grenzhaut selber gedrungen* ist. An einer *Stelle*, welche direkt in der *Richtung der Injektion* gelegen ist und jedenfalls unter einem *größeren Druck* gestanden haben wird, war dagegen ein *Platzen der Grenzhaut* erfolgt, wodurch sich erst in umfangreicher

Weise ein *oberflächlicher, epicerebraler Raum* zwischen M. lim. Gliae und Pia gefüllt hatte. Fig. 46 gibt bei starker Vergrößerung eine Stelle wieder, welche weit von jener Durchbruchsstelle entfernt an der konvexen Oberfläche des Kleinhirns lag; sie zeigt die *glatte Injektion dieses Raumes*, von dem aus ebenfalls in *umgekehrter Richtung nun keine Tuschemasse in jene Gebiete der Gliafüße unterhalb der M. limitans vorgedrungen ist.* In beiden Figuren ist auch eine geringe Strecke weit die *Injektion perivasculär* geworden. Während Fig. 45 die Injektion eines *künstlichen, von Gliafasern durchzogenen Raumes* darstellt, handelt es sich andererseits nach meiner Meinung bei der Fig. 46 um die Füllung eines natürlichen Spaltes im HISschen Sinne, der dadurch entsteht, daß die *Pia mater nirgends mit jener M. lim. Gliae flächenhaft verwachsen ist.*

Untersuche ich nun den Situs der Pia mater und ihrer inneren Fortsetzungen zur Membrana limitans Gliae auf Schnitten von fixiertem Material, so finde ich vielfach außer jenen Stellen, wo das piale Gewebe oder einzelne Teile desselben gegen die Grenzhaut angepreßt sind, *eine glatte Lösung beider Schichten.* Ebenso führt nach meinen Erfahrungen eine *vorsichtige Entfernung der Pia keineswegs zu einer Läsion der Grenzhaut*, sodaß ich Präparate erhalten habe, wo diese Grenzhaut des Gehirns als eine freie und von Pia nicht mehr bedeckte Linie die Eigengrenze des Gehirns direkt erkennen läßt. Ich verweise hierzu ferner auf die Angaben von KEY und RETZIUS[58]) S. 145, wonach die Pia mater mit ihrer *Intima piae* sich *leicht* z. B. vom Rückenmark abtrennt, „ja beinahe zu leicht, weshalb es gar schwer ist, feine Querschnitte von Pia und Rückenmark in beibehaltener Lage zu erhalten.“ Die spätere Bemerkung (S. 146): daß die Intima piae „mit einer dünnen Flächenschicht von Neuroglia leicht vereinigt ist, aber doch so innig, daß kein freier Zwischenraum (HIS, Epimedullärraum) hier vorhanden ist“, stimmt dazu nicht so recht. Ebenso habe ich an der folgenden Angabe („Die Neuroglia befestigt sich, ohne Modifikation ihres Gewebes zu erleiden, an der gegen sie übrigens scharf und gut als Haut begrenzten Intima piae, und die steifen Fasern der Mittelschicht treten keineswegs in die Neuroglia ein“) nur auszusetzen, daß die Bezeichnung einer „Befestigung“ nach meiner Meinung für die an *dieser Stelle nur lose aufliegende Pia* nicht richtig ist. Im übrigen meine ich, daß natürlich jener Raum nicht immer offen und weit sein wird,

aber in sich die Eigenschaft hat, jederzeit bei bestimmten Bedingungen offen zu werden und sich zu füllen. Die erwähnte Gegenpressung pialer Fasern an die Grenzhaut betrachte ich also als eine durch Fixierung entstandene. Wozu ich weiter die frühere Bemerkung von HIS citiere, daß die leichte Isolation von „ganzen Gefäßbäumen des Gehirns durch einfachen Zug mit der Pincette“, sich daraus erkläre, daß die „Gefäßadventitia in durchaus keiner Verbindung“ mit der Wand der perivasculären Kanäle stehe.

Zu allen diesen Gründen kommt nun endlich noch ein *weiterer Umstand* hinzu, welcher in der *Struktur* der *Intima piae* und ihrer *perivasculären Fortsetzung* längs der inneren Blutgefäße und ihrer adventiellen Scheide enthalten ist.

Die *Intima piae* zeigt nach KEY und RETZIUS eine eigentümliche dünne Schicht, eine *„Häutchenzellenschicht“*, welche „zerstreute Kerne und um sie herum eine dünne protoplasmatische Zone enthält, die diffus in das körnige Häutchen übergeht“. Unter ihr liegt ein *feines elastisches Fasernetz*. Diese Beschreibung trifft auch nach meinen Betrachtungen vollkommen die Wirklichkeit. In Fig. 33b und c habe ich eine kurze Strecke weit am Kleinhirn des Kaninchens das Gegenüber dieser tiefsten Schicht der Pia und der oberflächlichen, eigentlichen Grenzhaut des Gehirns im Flächenbild und in der dazu senkrechten Schnittansicht dargestellt. *Zellgrenzen* habe ich zum *Unterschied von der M. lim. Gliae* an dem *pialen Zellhäutchen nicht darstellen können*. Ich halte sie für eine *dünne syncytiale Zellmasse*. Der HISsche Epicerebralraum ist also nach meiner Auffassung von zwei wohlcharakterisierten Zellflächen verschiedener Abstammung und Wertigkeit, die eine glatte Lösung von einander zulassen, natürlich begrenzt.

Für die *Grenzen der perivasculären Räume* sind meine Beobachtungen weniger vollständig, insofern sie für das Gebiet der *feinsten Kapillaren* die hier interessierende Frage nach der *Gefäßscheidenbegrenzung durch eine jener oberflächlichen „Häutchenzellenschicht“ entsprechende Bekleidung* offen lassen müssen.

Bekanntlich hat zuerst VIRCHOW[59]) angegeben, daß an den Hirngefäßen überall eine „homogene Schicht“ vorkommt, „die sehr expansibel ist und schon durch einfache Wasserimbition zuweilen in so großen Säcken abgehoben wird, wie es sonst durch Blut geschieht“. Nach VIRCHOW geht diese Schicht auch „zuweilen auf

Gefäße von kapillarem Charakter“ über. Dann haben ROBIN[60]) und HIS[61]) unabhängig von der VIRCHOWschen Angabe und von einander eine feine und besondere Hülle beschrieben, welche nachher als „VIRCHOW-ROBINsche Scheide“ bekannt geworden ist. Nach ROBIN ist diese Scheide „d'une substance homogène ou à peine striée“. Zum Unterschied von der ROBINschen Textfigur und seiner Fig. 3 Pl. VI zeigen die HISschen Figuren 10—13 auf Taf. XXVIII eine mit länglichen Kernen besetzte adventielle Hülle, deren Zellelemente nach HIS als „überlagerte“ eine sichere Unterscheidung von den ebenfalls länglichen der Kapillarwand selber ermöglichen.

An meinen Präparaten vom *Kaninchen*, das im allgemeinen durch wenig bindegewebsfaserreiche Hüllen des Gehirns u. s. w. ausgezeichnet ist, finde ich nicht nur die feineren Kapillaren, sondern auch die *größeren Gefäßstämme* (s. Fig. 39a von einer aufsteigenden Arterie) von einer vorwiegend zelligen Hülle eingescheidet, die *stellenweise dem Gefäßrohr selber anliegt* und dann einen *weiten perivasculären Raum* zeigt, bald aber auch der M. limitans Gliae perivasc. *eng* angedrückt ist und dann von jenem *perivasculären Raum nichts* mehr angibt, sondern nur eine *offene VIRCHOW-ROBINsche Scheide* erkennen läßt. Beim *Menschen* dagegen finde ich an den *größeren Gefäßen*, z. B. an denen in der vorderen Rückenmarksspalte oder an solchen, welche eben in die graue Großhirnrinde eingedrungen sind (Fig. 37), auch eine stattliche *Menge von faserigem Bindegewebe* im Umkreis des eigentlichen Gefäßrohres mit *spärlich* angefügten adventitiellen Kernen. An den *Kapillaren* finde ich in Übereinstimmung mit HIS ausschließlich jene *rein zellige Hülle*, die im Bereich *der feinsten Kapillare nur noch ganz vereinzelte und in größeren Abständen von einander gestellte Kerne* zeigen, welche dann eine entsprechende *konkave Einbuchtung* der *feinen M. lim. Gliae* verursachen (Fig. 41). Ich kann aber nicht mehr angeben, ob das *zugehörige Protoplasma der verschiedenen und so weit gestellten Kerne*, wie es bei jener abgebildeten Arterie des Kaninchens z. B. noch zu beobachten war, *eine einheitliche, nirgend unterbrochene Zellhaut bilden*. Auch die früheren Angaben von EBERTH[62]), wonach die Hirn- und Rückenmarksgefäße bis auf die feinsten Kapillaren mit einem äußeren, aus *kernhaltigen Plättchen* zusammengesetzten *Perithel* überzogen sind, lassen diese Frage ungelöst. Eine Verwechselung etwa mit

Resten der M. lim. Gliae halte ich dadurch für ausgeschlossen, daß EBERTH *in den einzelnen Plättchen Kerne* gesehen und sie außerdem an den mittleren Gefäßästen der Art. fossae Sylvii beobachtet hat, die bereits völlig außerhalb der Grenzhaut liegen. Im übrigen führe ich seine andere Angabe, wonach kernlose Fädchen aus der circumvasculären Verdichtungsschicht der Gliafasern am Perithel verbreitert inserieren, auf eine zu enge Anpressung der feinen Gliagrenzhaut an jene äußere Schicht der Adventitia zurück. Von RIEDEL[62a]) sind übrigens die EBERTHschen Perithelzeichnungen auf kleinsten Arterien bestätigt worden. Für seine Angabe, daß eine direkte Verbindung der adventiellen Lymphscheiden verschiedener Gefäße vorkommen, welche als reinste Vasa serosa anzusprechen wären, habe ich auf meinen Schnitten keine Anhaltspunkte gewinnen können. Das einzige, was ich ihr zur Seite stellen könnte, sind *weitere Einscheidungen von verästelten Gefäßbäumen durch die Membrana limitans Gliae*, wie es die Figg. 32a u. 39b zeigen, die auf Schnitten natürlich *stellenweise leer von Gefäßröhren oder auch von Bindegewebsmassen* sein können. Da es sich hier aber nicht um adventielle Räume handelt und außerdem RIEDEL an Isolationspräparaten jene Gebilde gesehen hat, so finde ich keine eigentlichen Vergleichspunkte. Dasselbe muß ich von den Angaben KRONTHALS (Neurol. Zentralblatt 1890) sagen; seine Lymphkapillaren habe ich auf meinen Schnittpräparaten nicht gefunden. Was die *Übergangsstelle* der adventitiellen Gefäßscheide in das Gewebe der oberflächlichen Pia mater anbetrifft, so würde also an der Stelle der sogenannte *Piatrichter*, jenes Zellhäutchen der Intima piae, in diese besprochene *adventielle Wandschicht der perivasculären Räume* sich fortsetzen. KEY und RETZIUS geben hierfür an (S. 146), daß „die steifen Fasern der Circulärschicht der Intima mit dem Trichter und der Scheide ebenso steif und gerade laufen, wie in der Pia selbst" und sich späterhin „im allgemeinen etwas von dem Zellhäutchen trennen und sich dicht an die Wände des Gefäßes legen, oft dasselbe mit ihren Zweigen kreuzend". Womit meine bisherigen Beobachtungen durchaus übereinstimmen.

Zwei Fragen muß ich also hierbei offen lassen, die für die Vorstellung von der Saftbewegung innerhalb des Gehirns und ihrer besonderen Räume, dem ROBINSchen und dem HISschen Raum, von großer Bedeutung sind. Die erste Frage ist die, ob in der Tiefe des Gehirns, d. h. im *Gebiet seiner feinsten Kapillaren die beiden Spalten,* welche konzentrisch das Blutgefäß bis dahin begleitet haben, *mit einander zusammenhängen oder nicht.* Sie würden in einander einfach und breit übergehen, sobald sich präzis und sicher nachweisen ließ, daß jenes von der Pia herstammende Zellhäutchen der Tiefe zu irgendwie aufhörte. Die andere Frage ist, ob der zwischen dem pialen Zellhäutchen und der M. limitans Gliae gelegene Spaltraum mit den Lymphgefäßen der Pia mater zusammenhängt. HIS selber hat angegeben, daß die perivasculären Räume mit jenen Lymphwegen kommunizieren, was teils bestritten, teils bestätigt worden ist. So führen z. B. GOLGI und KEY und RETZIUS jene Füllung auf eine Mitverletzung der adventiellen Gefäßscheide bei der Einstichinjektion zurück. Ich selber habe noch keine abgeschlossenen Erfahrungen hierüber. Bei anderer Gelegenheit werde ich hierauf zurückkommen. Soviel will ich hier noch bemerken, daß bei der erschwerten Deutung alleiniger Injektionsresultate die Frage nach den *weiteren Verbindungswegen der HISschen Räume* auch dann lösbar werden muß, sobald im Sinne meiner Definition, wonach die HISschen Räume als dehiscierende Spalten zwischen der Intima piae und der M. limitans Gliae liegen, irgendwelche Lücken in dem Zellhäutchen der Pia mater nachweisbar werden.

Ich habe zum Schluß dieses Kapitels noch kurz meine Stellung zu der von BEVAN LEWIS[63]) ausgeführten Ansicht von einem besonderen, den perivasculären Lymphscheiden zwischengeschalteten „Lymphkonnektivsystem" der Hirnrinde zu präzisieren. Dasselbe soll nach ihm und auch nach ANDRIEZEN aus denjenigen Gliazellen bestehen, welche mit bestimmten Gefäßfortsätzen versehen sind und dadurch eine Saftzirkulation bewirken sollen. Für ein solches von gewissen Gliazellen nur, welche morphologisch durch Größe und Mehrkernigkeit charakterisiert sein sollen, gebildetes System haben sich BINSWANGER und BERGER[64]) ausgesprochen, welche bei Untersuchung der feinen Rindenveränderung beim Menschen nach einer ausgedehnten und aus dem Seitenventrikel stammenden subarachnoidalen Blutung, die „wohl auch an Zahl vermehrten Gliazellen der Molekularzone der Rinde massenhaft mit feinkörnigem Blutpigment erfüllt" fanden. Die perivasculären Räume waren weit, aber frei von Blut, ebenso die „perizellulären" Räume.

BINSWANGER und BERGER haben nun den Schluß gezogen, *„daß die Gliazellen der Molekularschicht mit ihren Fortsätzen bis an den Subarachnoidalraum reichen und tatsächlich ein Lymph-Konnektiv-*

system im Sinne BEVAN LEWIS' *darstellen, und zweitens, daß eine Verlegung der intraadventitiellen subarachnoïdalen Lymphräume eine Drucksteigerung in dem anderen — dem extraadventiellen — perizellulären Saftbahnsystem zu Folge hat."* Aus diesem dreiteiligen Schluß scheint mir die *Erklärung der Pigmentierung jener Gliazellen* im Sinne von LEWIS richtig zu sein, was auch noch beide Autoren durch *Karmininjektionen* in den *Subarachnoïdalraum eines lebenden Hundes* (nach gewisser Zeit fanden sich solche *Karminkörnchen in den Gliazellen*) bekräftigt haben. Nur haben BINSWANGER und BERGER noch *keinen definitiven Beweis für die feineren Wege* erbracht, auf welchen diese beiden Substanzen in den Leib der Gliazellen gelangt sind. Das Problem bleibt also dahin ungelöst, ob ausschließlich in den sogenannten *Saftspalten der Glia, zwischen dem Filz ihrer Fasern* u. s. w. jener Transport vor sich gegangen ist, oder ob er in der Hauptsache durch eine *aktive Beteiligung des Gliazellenprotoplasmas von jenen Gliafüßen her* besorgt worden ist. Für letzteres spräche ihre Angabe (S. 538), daß „einzelne Karminkörnchen sich auch streifenförmig in den Gliafäden, welche von den Zellen abgingen", abgelagert fanden. Dagegen meine ich, daß ihre Behauptung, wonach die Gliazellen bis an den Subarachnoïdalraum resp. seine Fortsetzung, den ROBINschen oder ihren intraadventitiellen Raum heranreichen, nach meinen obigen Darlegungen unrichtig ist. Dasselbe halte ich von dem letzten Teil ihrer ganzen Schlußfolgerung, da der von ihnen so bezeichnete Raum (extraadventitieller oder HISscher Raum) nach meiner Meinung bereits *innerhalb der marginalen Glia* liegt. Da außerdem bisher für diese Zone der Neuroglia eine von der Wirkungsweise der Fixierung als unabhängig erkannte Maschenweite noch nicht festgestellt worden ist, so muß natürlich auch ihre Erklärung aus einer *alternierenden Drucksteigerung* nur als eine Annahme erscheinen, die noch weitere Experimente und Untersuchungen nötig hat.

Für die Diffusion von Flüssigkeiten und den Transport von Stoffen, soweit sie aus dem Blutgefäßrohr stammen oder in die begleitenden Lymphwege zurückführen, sind also allgemein nach meinen obigen Ausführungen *zwei Filtrationsflächen in einer zu den Nervenzellen radiären Richtung angeordnet* zu unterscheiden, wobei ich von der eigenen Endothelfläche des Blutgefäßes selber absehe. Die *erste* würde in dieser Richtung das *Zellhäutchen der Intima piae*, rsp. die gleiche Schicht der *adventiellen Blutgefäßscheide* repräsentieren; die *zweite* würde *in der Membrana limitans Gliae* als der parenchymatösen Grenze der HISschen Räume gegeben sein. Zugleich würde hier das *Lymphkonnektivsystem* von LEWIS in Form jener Gliafüße ansetzen. Nur rechne ich dazu nicht nur seine Gliazellen, sondern ohne weiteres und mindestens die ganze Menge der marginalen Glia, wobei aber nach dem heutigen Umfang unserer Kenntnis verborgen bleiben muß, ob es überhaupt Gliazellen gibt oder wie viele es sind, die sich *nicht* an der Bildung einer oberflächlichen und tiefen Grenzhaut beteiligen. Berücksichtigt man nämlich die Gesamtfläche der oberflächlichen Gliagrenzhaut, die Kleinheit der einzelnen Felder der Gliafüße, sowie den Reichtum der zentralen Substanz an Blutgefäßen und der für ihre Einscheidung nötigen Summe von Gliafüßen, so erscheint meine Rechnung nicht unbegründet. Zu unterscheiden wird aber bei den weiteren diesbezüglichen Untersuchungen sein, ob jener Transport von Stoffen ausschließlich innerhalb der Gliazellen und ihres Protoplasmas oder innerhalb ihrer Maschenräume vor sich geht. Erst dann würde die Frage nach dem Anschluß der Nervenzellen an jene extragliösen Lymphräume einer Lösung sich nähern können. Von OBERSTEINER ist behauptet worden, daß es für die zentralen Nervenzellen noch besondere Lymphräume gibt, die er als *perizelluläre Räume* bezeichnet hat und von denen er meint, daß sie in *direkter Verbindung mit den perivasculären Räumen* stünden. Bereits HIS hat sich von vornherein gegen solche Einrichtung ausgesprochen gehabt und das damit begründet, daß er an gut injizierten Präparaten nie eine „Verbindung dieser ringförmigen Höfe mit dem System der perivasculären Räume" gesehen habe. Ich führe gegen jene Ansicht den von mir oben bereits entwickelten Begriff einer Gliagrenzhaut an. Im folgenden Kapitel werde ich außerdem hierzu nachweisen, daß die Entstehung freier Räume um die Nervenzellen im OBERSTEINERschen Sinne auf einem bestimmten Artefakt beruht.

4. Über netzartig verzweigte Gliazellen.

Wie ich im vorigen Kapitel für die *marginale Neuroglia* bereits angedeutet habe, sind die vorherrschenden Anschauungen von einer rein *frei verästelten Form der Gliazellen*, wie sie die RANVIERsche

Isolationsmethode oder die GOLGIsche Imprägnationsmethode demonstrieren, *nicht richtig*. Eine Erklärung jener eigentümlichen und deutlichen Formung, die auf *einer netzigen oder mehr membranartigen Verbindung* von mehreren Gliazellen mit eingefügten, überkreuzten Gliafasern beruht, aus einer *reinen Verfilzung* ist nach meiner Meinung nicht möglich. Eine solche trifft nach meiner Meinung nur für die Menge der Gliafasern zu. *Jene übrige Masse*, welche erst die *wirkliche Form des Gliagewebes* ausmachen würde, scheint im allgemeinen diesen beiden Methoden zu entweichen. Hiermit schließe ich mich also in gewisser Weise PALADINO an, der von einzelnen Gliazellen der grauen Substanz und von denjenigen der weißen eine netzförmige Ausbreitung angegeben hat. Seine Behauptungen von dem Zusammenhang der Glia mit dem Mark der Nervenfasern, sowie mit dem Gewebe der Pia, kann ich jedoch nicht unterstützen.

Jene obigen Befunde an dem netzförmigen Bau der marginalen Glia will ich hier durch Beobachtungen erweitern, welche ich vereinzelt auch an den *Elementen der weißen und grauen Substanz* im unmittelbaren Bereich der Nervenfasern und Nervenzellen selber habe gewinnen können. Daß ich an der marginalen Neuroglia der äußeren Oberfläche umfangreicher und leichter eine netzartige Formation habe darstellen und beobachten können als in der Tiefe der zentralen Substanz, wird, abgesehen von der anfänglichen Wirkung des noch reinen Fixierungsmittels, vor allem wohl darauf zurückzuführen sein, daß hier zum Unterschied von jenen Zonen *keine oder eine nur geringe Beimischung oder Einfügung von nervösen Elementen* besteht.

Zunächst beschreibe ich hier genauer jene obigen Angaben über den netzförmigen Bau der Glia marginalis. Wie ich auf der Fig. 34a—c, 36 u. 37, sowie in Fig. 39a dargestellt, erscheinen mir die Gliazellen in dem Grenzgebiete zur Pia oder auch zu deren tiefen, gefäßhaltigen Fortsetzung bei nicht zu stark differenzierter Färbung und auf dünnen Schnitten als *netzförmig verzweigte und jedenfalls zum Teil mit einander zusammenhängende Elemente*. Die *netzartige Verzweigung entsteht zunächst durch solche des Protoplasmas der betreffenden Gliazellen*, wobei vielleicht noch eine eigentümliche und *modifizierte Zellsubstanz* eine gewisse Rolle spielen mag. Diese netzartige Masse zeigt weiter eine *wechselnde und verschieden laufende Summe von Gliafasern*, welche *teils in den Netzbalken als quer oder längs getroffene feinere und intensiv dunkel gefärbte Fasern liegen, teils im Protoplasmaleib der Gliazellen eingeschlossen sind*. Führt man unter ausgiebiger Anwendung der Mikrometerschraube genaue Tiefenbeobachtungen aus, so zeigt sich dieses Netz als ein allgemeines, die betreffenden Schnitttiefen durchsetzendes, dreidimensionales Netz- oder Maschenwerk. Der Oberfläche zu, d. h. gegenüber dem Bindegewebe der Pia resp. der adventiellen Scheide der inneren Blutgefäße ist dieses Maschenwerk durch eine Membran abgeschlossen, wie ich oben nachgewiesen habe. Daß es stellenweise auch *in sich selber geschlossene* Maschen- oder Hohlräume ausbilden kann, schließe ich aus *undifferenzierten Präparaten*. Zum Teil sind allerdings diese Räume durchbrochen, zum kleinen Teil aber auch wirklich geschlossen, sodaß ihr Schnittbild erst jene erstere Formation geben würde. Deren gröberen Balken resp. den dickeren Wandabschnitten entsprechen die sonst als Fortsätze der Gliazellen bezeichneten Zellteile, wie sie also bei einer gröberen Isolation oder einer unvollständigen Silberimprägnation naturgemäß entstehen müssen, da sie zu einer Zerreißung der übrigen Anteile resp. zu einer optischen Unsichtbarkeit dieser Netzwerke führen. Ebenso ist klar, daß endlich eine reine Faserfärbung wie sie die WEIGERTsche Methode gibt oder differenzierte Hämatoxylinfärbungen, die ich an den unmittelbar folgenden Kontrollschnitten ausgeführt habe, entstehen lassen, *statt jener eigentümlichen Gewebsform nur einen mehr oder weniger dichten Filz von Fasern* zeigen. *Von freien Neurogliafasern als einer zellunabhängigen Interzellularsubstanz kann hier natürlich nicht die Rede sein, da sie ja in der angegebenen Weise intrazelluläre Anteile* dieses von Zellen und Zellaufzweigungen zusammengesetzten Gewebes der marginalen Neuroglia sind.

An den *Gliazellen der weißen Substanz* und zwar derjenigen des Rückenmarks, des Kleinhirns und des Großhirns habe ich bei gleicher differenzierter Hämatoxylinfärbung gefunden, daß *außer den Gliafasern*, welche teils längs, teils in mehr querer Richtung oder auch in reiner Überkreuzung die faserigen Gliascheiden der zentralen markhaltigen Nervenfasern bilden, noch eine *Masse* vorhanden ist, welche *körnig oder gerinselig erscheint*, mehr oder weniger vollständig und oft nur sehr dünn die einzelnen Gliafasern umhüllt oder breiter dieselben dort verbindet, wo sie sich überkreuzen oder dichter nebeneinander laufen (Fig. 11 und 12).

Diese Masse ist im allgemeinen nur sehr dünn ausgebreitet und wie ein feines Netz angeordnet, wo zwischen den Gliafasern kernfreie Strecken vorhanden; sie wird aber sofort gröber und deutlicher, sowie man in die Nähe von kompakterem Gliazellenprotoplasma und seiner Ausläufer kommt, wobei man beobachten kann, daß jene Substanz mit dem Protoplasma der Gliazellen zusammenhängt resp. aus ihr hervorgeht. Es sind mit anderen Worten die *weiteren* und vielleicht noch irgendwie besonders beschaffenen *Verzweigungen jener Gliazellenfortsätze netzartig angeordnet; sie begleiten zugleich die Gliafasern, sie mehr oder weniger einhüllend oder auch verbindend.* (Fig. 14 und 15 aus der weißen Substanz des Großhirns). Ich erinnere hierbei, daß ich auch in der *Substantia Neurogliae centralis* einen derartigen netzförmigen Bau finde, der hier mit jenen Fortsätzen der Ependymzellen und ihren Seitenzweigen, sowie denen der subependymären Gliazellen zusammenhängt. Er erscheint mir nur gröber und enger, sowie aus dickeren und dichter gekörnten Balken oder Lamellen zusammengesetzt.

Die *Nervenfasern der weißen Substanz* sind also in einem *maschigen oder netzförmig* gestalteten Gewebe gefaßt, welches sie *scheidenartig* begleitet und zu *einem Teil aus Fasern* besteht, welche als *Gliafasern* nur *überkreuzt* sind, im übrigen aber durch jene mit dem Protoplasma der Gliazellen zusammenhängende Zwischenmasse netzartig oder auch mehr flächenartig im Bereich der Gliazellenleiber selber vereinigt erscheinen. Man könnte mir einwenden, daß diese von mir beobachtete und zunächst als netzförmig verzweigte Protoplasmamasse von Gliazellen bezeichnete Zwischensubstanz der Gliafasern nur ein Kunstprodukt sei, welches aus einer lymphatischen Flüssigkeit, die man sich ja zwischen den Gliazellräumen vorstellt, durch den Fixierungsvorgang niedergeschlagen sei und an jene Zwischenwinkel der Gliafasern angeklebt und festgehalten sei. Man könnte auch behaupten, daß es Gerinnungsreste oder Lösungsreste aus der zentralen Markmasse seien, die solches nur vortäuschten. Gegen die letzte Meinung führe ich an, daß außer den Fixierungslösungen, welche wie das Alkohol-Chloroform-Eisessiggemisch mit nachfolgender Härtung in 96% oder 100% Alkohol die Markmasse zum großen Teil lösen, auch solche Fixierungen gleiche Resultate geben, welche wie die MÜLLERsche Lösung z. B. oder eine Mischung von Kal. bichrom. und Eisenalaun die Markmassen konservieren, wenn sie sie auch aufblättern.

Wozu ich außerdem anführe, daß die netzartige Substanz anders aussieht und anders sich entfärbt wie jene fixierten oder partiell gelösten Markscheiden oder Markscheidenreste. Sehe ich außerdem davon ab, daß die *direkte Zugehörigkeit einer netzig geformten Gliascheide zu einer Gliazelle* von mir wiederholt beobachtet worden ist, so spricht auch das noch vor allem für meine Behauptung, daß bei Färbungen, die den granulären Bau der Gliazellen demonstrieren, *protoplasmatische Körnchen längs der Gliafasern verteilt zu sehen sind,* welche mit den Körnchenmengen des Zelleibes oder eines dickeren Fortsatzes selbst in Farbe und Größe übereinstimmen und außerdem durch eine feine, matt entfärbte Masse stellenweise mit jenen Zellabschnitten verbunden erscheinen. (Fig. 4c; Fig. 3 zeigt dasselbe an der subependymären Glia des IV. Ventrikels).

Zu diesen Beobachtungen kommen andere, welche ich auf Präparaten der BETHEschen Molybdänmethode angestellt habe. Unter den Bildern, welche diese Methode in wechselnder Weise von den Elementen des Zentralnervensystems liefert, sodaß sie auch in gewisser Weise zu ihrer Deutung eine Erfahrung an anders gefärbten Präparaten nötig hat, habe ich einige gefunden, die mit meinen obigen Befunden eine weitgehende Übereinstimmung erkennen lassen.

So besitze ich Rückenmarkspräparate vom Hund und vom Rind, die mir für die Substantia Neurogliae centralis sehr deutlich eine allgemeine und durchgreifende Netzform der gliösen Protoplasmamasse in blaß violettem Farbton zeigen. Dunkelblau sind zugleich und zufällig eine Anzahl von Gliafasern gefärbt, welche aus einzelnen Zelleibern von subependymären Gliazellen hervorgehen und weiterhin in den Balken jenes Netzwerkes verlaufen, nicht etwa in ihren Zwischenräumen, wie man nach der WEIGERTschen Lehre von einer faserigen Interzellularsubstanz erwarten sollte. Es stimmen also diese Präparate mit solchen überein, die ich oben beschrieben habe.

Von BETHE[65]) ist seinerzeit im Zentralnervensystem von Wirbeltieren ein *„Füllnetz"* beschrieben worden, welches an eine frühere Beschreibung GOLGIS (ges. Abhandl. 1894, S. 249) von einem allgemeinen diffusen Netzwerk erinnert. Es kommt nach ihm nicht nur in der grauen Substanz vor, sondern „geht auch auf die weiße Substanz über und füllt hier ähnlich wie die WEIGERTsche Glia Spalten zwischen den Markscheiden aus, indem es die

Markscheiden umspinnt." Es hat weiterhin auch „Beziehungen" zu Blutgefäßen, Gliakernen und der Pia mater. „Mit den WEIGERTschen Gliafasern hat das Füllnetz direkt nichts zu tun; es besteht nicht aus verklebten derartigen Fasern, wie man aus übermolybdänierten Schnitten ersehen kann, an denen die WEIGERTschen Gliafibrillen (allerdings nicht schön) zutage treten. Es scheint mir nicht ausgeschlossen, daß das Füllnetz ein Gerinnungsprodukt ist, ich will aber nichts Bestimmtes darüber aussagen." Als etwas *Besonderes*, was mit dem Füllnetz nichts zu tun hat, hat BETHE die *GOLGInetze* bezeichnet. Er meint von ihnen, daß sie *nervös* sind, aus *Neuritenenden* wahrscheinlich hervorgehen und wahrscheinlich auch durch die in ihren Netzbalken laufenden Fibrillen mit den *Neurofibrillen* der von ihnen umsponnenen Nervenzellen zusammenhängen. Ich habe[46]) dann dazu ausgeführt, daß das BETHEsche Füllnetz ein *allgemeines Gliareticulum* vorstelle, welches an *zwei Stellen nur eine besondere Modifikation* zeige, an der *Oberfläche der Ganglienzellen* der grauen Substanz, wo es die *GOLGInetze* unter Verdichtung seiner Substanz bilde, und an den Marksegmentgrenzen der Nervenfasern, wo die Gliascheiden zu den *Gliaschnürringen* sich zusammenschließen. Als Gründe habe ich u. a. damals mitgeteilt, daß ein *kontinuierlicher Zusammenhang* zwischen den Balken des Füllnetzes und denen der GOLGInetze bestehe, und daß zweitens erst mit dem GOLGInetz als seinem besonderen Stütznetz dasjenige Netz alterniere, welches aus den Endflächen von Neuriten als nervöses perizelluläres Terminalnetz hervorgehe. Ich füge jetzt weitere Beobachtungen hinzu, die mir zum großen Teil schon damals bekannt waren, und die als einen II. Teil über diese besonderen Formbeziehungen der Neuroglia hinzuzufügen mich nur äußere Gründe damals abgehalten haben. Diese Beobachtungen enthalten die *direkten Beziehungen des „Füllnetzes" oder des Gliareticulum zu der Masse der Gliazellen.*

Sucht man nämlich auf solchen Präparaten der BETHEschen Molybdänmethode, auf denen die Füllnetze zur blassen Darstellung nur gekommen sind, in der weißen Substanz z. B. nach irgend welchen sonstigen Beziehungen derselben, so wird bald auffallen, zumal wenn man Quer- und Längsschnitte derselben Gegend miteinander vergleicht, daß die blasse und bald dichter, bald enger gekörnte Masse des Füllnetzes mitunter deutlich eigentümlich hellen und ziemlich ungefärbten Straßen folgt, welche mit der

Richtung der Nervenfasern mehr oder weniger stark gekreuzt sind und oft deutlich zu den Stellen der Gliakerne hinführen, wobei jedoch die Masse des Füllnetzes von dem Gliazellkern selber durch eine noch blässere Substanz in geringem Abstande stets entfernt gehalten wird. Untersucht man hierzu Präparate, die möglichst wenig alkoholdifferenziert worden sind (Fig. 16, 17), oder solche, bei denen durch alkoholdifferenzierte Erythosinnachfärbung eine gewisse, wenn auch nicht sehr elektive und elegante Darstellung jener blassen Stellen erfolgt ist, so sieht man, daß das Füllnetz eine bestimmte Beziehung zu einer ebenfalls netzartig verzweigten Substanz der Gliazellen selber haben muß.

Daß das Füllnetz keine „verklebten Gliafasern" bedeutet, gebe ich BETHE zu; aber es hat insofern nach meiner Meinung mit ihnen zu tun, als es ihren Wegen folgt, resp. sie in seiner Masse mit enthält. Wie ich in Fig. 16 und 17 aus der weißen Substanz des Kleinhirns vom Kaninchen dargestellt habe, hängt das Füllnetz, welches auf dem Längsschnitt die ziemlich ihrer Länge nach getroffenen Nervenfasern mit Gliascheiden und Gliaschnürringen zeigt, mit mehreren Gliazellen zusammen, welche in ihrer protoplasmatischen Ausbreitung selber blasser gefärbt sind. Am Zelleib und an ihren Fortsätzen, welche radiär abgehen und zum Teil sicherlich den Gliafasern der Zelle entsprechen dürften, zeigt sich dagegen eine dunkler gefärbte Masse, welche zu einem Teil aus Körnchen und Streifen oder auch kurzen Stäbchen zusammengesetzt ist und in wechselnder Verteilung die Balken des Füllnetzes besetzt und in dichterer Zusammenfügung endlich die Substanz des Schnürringes mit bilden hilft. Im Querschnittsbild zeigt das Gleiche die Fig. 17, wobei besonders bei der flach angeschnittenen Gliazelle eine Flächenansicht jener dunkler gefärbten eigentümlichen Masse zu sehen ist, welche hier, etwas undeutlich netzig verteilt, die Oberfläche des Zelleibes einnimmt, weiterhin aber spärlicher in den Netzbalken selber resp. in ihren Knotenpunkten angeordnet ist. Derartige Präparate können demonstrieren, daß diese *Masse des Füllnetzes den Gliazellen und ihren Fortsätzen folgt*; daß sie in der Oberflächenschicht dieser Stützzellen aber selber enthalten ist resp. dieselbe zum Teil ausmacht, geht noch nicht unmittelbar daraus hervor. Deutlicher wird solche Beziehung schon an mit Erythrosin nachgefärbten Präparaten, die mir zeigen, daß jene bei der Molybdänmethode *dunkel gefärbten Substanzteile*

in der *Oberfläche* der nun rot gefärbten Zelle enthalten sind und *in den Fortsätzen geringe seitliche Verdickungen* bilden. Dazu kommt, daß meine Hämatoxylinfärbungen mir Bilder geben, welche sich direkt hiermit vergleichen lassen, da sie jene eigentümliche Substanz des Füllnetzes als *eine vom übrigen Protoplasma des Gliazellenleibes durch etwas dunklere Färbung und oberflächliche Lage unterschiedene Masse* zeigen, welche weiterhin längs der Gliafasern sich fortsetzt und hier jene Verteilung hat, die ich oben auf *faserdifferenzierten Präparaten* als partielle Hüllen derselben resp. Verbindungsbalken beschrieben habe. Ich verweise weiter hierzu auf Fig. 14, welche die *Bildung eines Gliascheidenanteiles aus einer Gliazelle der weißen Substanz des Großhirns* vom Menschen wiedergibt, die ich durch eine etwas andere Entfärbung erzielt habe, sodaß nur die allgemeine Verästelungsform der betr. Zelle, weniger bereits die in ihr enthaltene Gliafaser, zum Ausdruck kommt.

Von BETHE ist angegeben worden, daß sein *Füllnetz überall an den Blutgefäßen und der Pia mater mit seinen Balken inseriere.* Wie ich im 3. Abschnitt gezeigt habe, sind solche Beziehungen ein *Charakteristicum der marginalen Glia.* Ihre *allgemeine Form* ist ebenfalls die eines *Netzes,* welches von Gliazellen geliefert wird und reicher oder ärmer an Gliafasern sein kann, wodurch sein gliöser Charakter im Sinne WEIGERTS entschieden wird, der ja zuerst eine Sicherheit in der Erkennung des Gliagewebes durch ihren Gehalt an Gliafasern geschaffen hat. Fig. 34—38 zeigen diesen Charakter der marginalen Glia an Hämatoxylingefärbten Präparaten; was ich an solchen mit der BETHEschen Methodik gesehen, entsprach ihrer Formation, nur daß jene Beziehungen zu dem Gliazellenprotoplasma sowie die Gliafasern mit ihren Gliafüßen und außerdem noch der häutchenartige Abschluß durch eine Grenzmembran nicht zum Ausdruck kamen.

Ich habe endlich noch Beobachtungen an *gewissen Gliazellen der grauen Substanz gemacht.* Im Bereich des Vorderhorns des Rückenmarks finde ich an den gliösen *Begleitzellen der großen Nervenzellen* eine Form und Verästelungsweise, die meine vor kurzem geltend gemachten Gründe in hohem Maße verstärken können, sodaß ich jetzt die perizellulären GOLGInetze an den zentralen Nervenzellen als *gliöse Stütznetze* bezeichnen kann. Wie Fig. 18 und 19 zeigen, sind es *Gliazellen, welche die GOLGInetze aus sich hervorgehen lassen.* Fig. 18 zeigt zunächst einen Kalottenschnitt

durch die einer Nervenzelloberfläche anliegende Zone der grauen Substanz. Die Nervenzelle ist selber nicht getroffen; nur ganz vereinzelte Neurosomenhaufen ihrer nervösen Endfläche zeigen ihre Nähe an. Dagegen sind zwei Gliazellen angeschnitten, die zusammen ein Netzwerk liefern, in dem auch einzelne sehr feine Gliafasern enthalten zu sein scheinen. Die Formation dieses Netzes gleicht genauer derjenigen eines GOLGInetzes, wenigstens spricht für *solche Idenfizierung der Umstand, daß hier einzelne Neurosomenhaufen in ihren Maschen liegen,* von denen ich früher solche charakteristische Lage an einer Nervenzelloberfläche angegeben habe. Da aber an diesem abgeschnittenen Netz keine direkte Lagebeziehung zu einer Nervenzelle selber zu sehen ist, so brauchte in BETHEscher Bezeichnungsweise das Netz erst ein solches zu sein, welches als „Füllnetz" die *weiteren Umkreise* des eigentlichen und von ihm verschiedenen GOLGInetzes einnimmt, das hier selber dann noch nicht getroffen wäre. Fig. 19 zeigt nun von einer anderen Stelle desselben Präparates, wo zugleich zwei Nervenzellen und ihr Belag von Neurosomenhaufen angeschnitten ist, mit Sicherheit, daß erstens es die die Nervenzellen begleitenden Gliazellen sind, welche ihre GOLGInetze liefern und daß zweitens solche Hüllnetze kontinuierlich in das diffuse Netz übergehen, welches als „Füllnetz" z. B. den jenen beiden Nervenzellen zwischengelagerten Zug markhaltiger Nervenfasern mit Gliascheiden versieht. Die *substanticilen Unterschiede,* die die BETHEsche Methode *zwischen GOLGInetz und Füllnetz* gezeigt, kommen hier allerdings nicht zum Ausdruck; dafür aber zeigt dieses Präparat, daß *beide nur zonale Anteile eines allgemeinen, weil von Gliazellen gebildeten, Gliareticulum bedeuten.* Bei der BETHEschen Methodik kommen im allgemeinen solche Beziehungen zwischen den Gliazellen und den GOLGInetzen oder auch den Füllnetzen nicht zum Ausdruck, da außer ihren Kernen weder ihr eigentliches Protoplasma noch ihre Gliafasern gleichzeitig mitgefärbt werden. Die BETHEschen Abbildungen zeigen alle die betreffenden Netze erst in einiger Entfernung von Gliakernen einsetzend. Ich glaube hierfür eine Erklärung geben zu können, welche einen solchen Grund zum Teil in der Methodik nachweist. Derselbe ist in der Wirkungsweise eines Alkalis auf das Gliagewebe enthalten, welches bei der BETHEschen Methode in Form der alkoholischen Ammoniaklösung zur Anwendung gelangt. Solche Wirkungen sind mir seit

meinen Fixierungsversuchen mit Laugenalkohol aufgefallen und speziell für meine neueren Versuche, mit Hilfe von molybdänisierten Hämatoxylinlösungen die fraglichen Netze zu differenzieren, als besondere Veränderungen wieder bekannt geworden, wenn ich eine Behandlung der bereits fixierten und aufgeklebten Schnitte mit Laugenalkohol der Beizung und Färbung vorausschicke. Es zeigt sich hierbei, daß die den *Gliakern zunächst umgebende protoplasmatische Substanz irgendwie verändert* oder gelockert, vielleicht auch partiell gelöst werden muß, sodaß sie künstlich bei der nachträglichen Färbung und Differenzierung einen *mehr freiliegenden Gliakern* entstehen läßt. Wodurch es verständlich erscheint, daß bei der BETHEschen Methodik erst im Umkreis der Gliakerne und nicht mehr durch eine irgendwie deutliche Protoplasmamasse mit ihnen verbunden das allgemeine Gliareticulum anfängt. Weiter geht aber aus diesen Beobachtungen hervor, daß die Substanz der Netze, welche als dunkel gefärbte Masse im Gliareticulum durch die BETHEsche Methodik hervorgehoben wird und im besonderen Abschnitt des „Füllnetzes" lockerer und weiter verteilt, in der Zone der GOLGInetze und Gliaschnürringe dagegen, wie ich früher ausgeführt, kompakter und enger gehäuft ist, nicht *einfaches Gliazellenprotoplasma* der angegebenen Herkunft ist, sondern *eine irgendwie beschaffene und besondere Substanz* bedeutet. Mit den im Gliazellenprotoplasma sonst noch vorkommenden Granulis, deren Verteilung in Zellleib und Fortsätzen ich oben angegeben (siehe Fig. 4c), halte ich sie nicht für identisch, schon aus morphologischen Gründen, wozu solche hinzukommen, daß Granulafärbungen nie eine Andeutung jener Netzsubstanz geben. Positive und sichere Anhaltspunkte über die Art dieser Masse habe ich bisher nicht finden können.

Damit komme ich zu dem allgemeinen Schluß, daß, abgesehen natürlich von der Entstehung einer protoplasmatischen Granulierung, die *Gliazellen* sonst im Laufe der Entwicklung zwei verschiedene Substanzen ausbilden, die auch in der Form unterschiedlich sind, 1. die WEIGERTschen *Gliafasern* und 2. die in dem *Gliareticulum* mit seinen beiden Zonen enthaltene besonders färbbare Substanz.

Meine obige Auffassung von der *Natur der Begleitzellen der Nervenzelle* als besonders und enger verzweigte, netzförmige Gliazellen, welche wegen ihrer gehäuften Lage an der Oberfläche dieser Elemente eine besondere Hülle und Stütze für jene Zellen

sowohl wie für ihr perizelluläres nervöses Terminalnetz bedeuten müssen, gibt mir einen Anlaß, eine begründete Erklärung für die Entstehung jener OBERSTEINERschen Räume zu geben. Ich stütze mich hierbei außerdem auf eine *Reihe von verschiedenen Fixierungen*, die nicht nur alle *Übergänge von der Enge zur Weite* bei diesen angeblichen perizellulären Lymphräumen demonstrieren, sondern vor allem auch eine *Zerreißung der netzförmigen Verästelung jener Gliazellen* beweisen. Ich stimme also durchaus den ersten Angaben GOLGIS zu, welcher die OBERSTEINERschen Räume für einfache Retraktionslücken als Folge ungeeigneter Fixierung erklärt und bereits jene angeblichen Lymphkörperchen hier für wahrscheinliche „Bindegewebskörperchen" (die damalige Bezeichnungsweise GOLGIS für die heutigen Gliazellen) des umgebenden Stromas gehalten hat. Die Angabe von ANDRIEZEN, wonach die im „perizellulären" Raum befindlichen Zellen kurze Fortsätze haben, betrifft also durchgerissene Zellformen. Erst recht nicht stimmt jene Behauptung FRIEDEMANNS mit der Wirklichkeit, daß diese „Randzellen" eine Endothelauskleidung des perizellulären Lymphraumes wären. Von PALADINO[7]) ist nun beobachtet worden, daß jener perizelluläre Raum von einem mit dem übrigen Stützwerk der umgebenden Substanz zusammenhängenden Netzgewebe ausgefüllt sei. Ich identifiziere meine obigen Angaben mit dieser Beobachtung und meine, daß die Vermutung OBERSTEINERS (Bau der nervösen Zentralorgane 1901 S. 221) von einer „Verwechselung mit Nervenenden" die Sache nicht erledigt.

Zum mindesten ergibt sich also, daß die noch von einem Netzwerk durchzogenen Höfe um die Nervenzellen einigermaßen die normalen Verhältnisse wiedergeben; wobei aber jener Unterschied unberücksichtigt bleiben muß, der zwischen *nur locker oder stärker angespannten Netzteilen* besteht, und von dem man ebensowenig bisher wie vom ROTHschen Raum genau sagen kann, ob solche Unterschiede *außer auf Fixierungswirkung* auch noch auf eine *ungleiche Füllung von Gliaspalten mit lymphatischer Flüssigkeit wirklich zurückgeführt werden dürfen.*

5. Elemente der Neuroglia bei Wirbellosen.

Als einen kurzen Anhang nur zum vorigen Kapitel beschreibe ich hier von Hirudo officinalis zunächst einige Beobachtungen, welche

eine auffallende Ähnlichkeit gewisser Zellelemente und ihrer Produkte mit den *netzförmig verzweigten Gliazellen* der Wirbeltiere in ihren *allgemeinen Beziehungen zu Nervenzellen und Nervenfasern* erkennen lassen.

Auf den Figg. 47a, b, c und Figg. 48a und b habe ich die *Gliascheiden um die Nervenfasern eines Konnectivs* von Hir. off. abgebildet, wie sie meine Hämatoxylinpräparate zeigen. Sie bilden ein enges Gitter, welches je nach der *gedehnten oder verkürzten Leibeslänge* des Tieres eine *wechselnd weite Maschung* zeigt (Fig. 48a zum Unterschied von Fig. 48b). Die Balken des Gitters, die bald gröber, bald sehr fein sein können, zeigen nicht überall eine reine Überkreuzung sondern auch eine richtige Verlötung ihrer Substanz. Diese maschigen Gliascheiden sind nun, wie die Figg. 47a—c zeigen, die *außerordentlich lang gewachsenen Differenzierungsprodukte einer einzigen Zelle*, welche in der Mitte eines Konnektivs gelegen ist. Als radiäre Septen in der Quere des Konnektivs und zu Röhren in seiner Länge sind diese maschig geformten Gliabalken von der Zelle fort entwickelt, mit der sie übrigens durch ein *in ihrem Protoplasma ausgespanntes Gitter* direkt zusammenhängen. Das *intrazelluläre Gitterwerk* ist in der Kernzone feiner, um an der Oberfläche der ganzen Riesenzelle sowohl in ihrer Kernhöhe (47b) wie an beiden Polen (47a u. c) in grobe Balken überzugehen, die sich dann wiederum und vorwiegend in der Länge des Konnectivs erst in jene gleichmäßigeren Gliascheiden der einzelnen Nervenfasern fortsetzen. Es ist klar, daß eine derartige Gitterformation eine beträchtliche Scheerfestigkeit abgibt, die den Nervenfasern des Konnektivs bei den Längenverkürzungen des Tieres einen ausreichenden Schutz und Halt verleiht. Diese *Riesengliazelle*, welche durch ihre gitterförmigen Gliamassen zum mindesten die *Nervenfasern eines Konnektivs einhüllt*, entspricht dem, was APÁTHY[68]) als *Konnektivspindel* bezeichnet hat; meine Beschreibung ihrer Gliaprodukte stimmt mit derjenigen APÁTHYS überein. HOLMGREEN[69]) hat inzwischen eine Abbildung (Fig. 73 auf Tafel XIV) dieser Zelle gegeben, die diese Verhältnisse im Längsschnitt beurteilen läßt. Auf seine Auffassung komme ich unten zurück.

Vergleiche ich die Nervenfasern des Konnektivs mit der weißen Substanz eines Wirbeltieres, so erscheint hier von einer *einzigen Zelle* gebildet, was dort das faserige Produkt einer *Summe kleinerer Gliazellen* ist. Wie deren Gliafasern mit dem Protoplasma der Zellen noch vereinigt sind, so ist bei Hirudo der Anfang jener Gliascheiden in einem von Protoplasma umhüllten *intrazellulären Gitterwerk* enthalten, wozu ich hervorhebe, daß auch bei den *Astrocyten der Wirbeltiere* (siehe Fig. 4a vom Kaninchen, 6 vom Menschen) ein *schwaches intrazelluläres Netzwerk in Verbindung mit ihren Gliafasern* vorkommen kann. Sonst erscheinen die Gliafasern in den Gliascheiden der weißen Substanz mit einander unverbunden; ihre stellenweise sehr deutlich geordneten *Überkreuzungen* (Fig. 12 vom Menschen, Fig. 5b vom Kalb) haben also nur eine äußere Ähnlichkeit mit jener netzmaschigen Formation, die den erwähnten beträchtlicheren Körperveränderungen zu folgen geeigneter erscheint, während jene nur die Druckschwankungen der Gehirnmasse und deren Eigengewicht auszugleichen haben.

Mit den *Gliazellen der grauen Substanz* vergleiche ich in der Hauptsache diejenigen Zellen, welche APÁTHY als *mediane Sternzellen* bezeichnet hat; hinzukommen solche, welche von ihm *Sternzellen der Ganglienzellenpakete* genannt werden. Beide halte ich mit APÁTHY für gliaproduzierende Zellen, welche unter anderem die besonderen Hüllen der Ganglienzellen bilden. Daß auch hier meine Befunde mit denen APÁTHYS ziemlich vollständig übereinstimmen, wird meine folgende Beschreibung zeigen.

Die zwei *medianen Sternzellen eines Bauchganglion*, an dessen ventraler Längsfurche sie liegen, bilden für die zentrale Fasermasse und die ihr aufsitzenden Ganglienzellen zwei *Riesengliazellen*, die sich derjenigen eines Konnektivs vergleichen lassen. Sie sind in ihrem Protoplasmahof wie diese von einem Gliagitter durchsetzt (Fig. 50), welches an der Oberfläche der ganzen Zelle grobbalkig wird (in Fig. 49 ist dieser Anteil allein gefärbt geblieben). Im Umkreis der Zelle gehen dann weit verzweigte Gliafasern oder Bündel von solchen hervor, die die ganze zentrale Fasermasse als einzelne Züge durchweben, und nur stellenweise maschig geformte Gliascheiden um ihre Nervenfasern bilden. Sie scheinen mir mit der vom Konnektiv her einstrahlenden Glia zusammenzuhängen. Aus den Gliafasern jener Fasermasse entstehen an ihrer ganzen Oberfläche wieder dickere Bündel, die bald ringförmig sich ordnen und nun die *Stielfortsätze und die Ganglienzellleiber* mit einem *netz-maschigen, zum Teil auch rein faserverkreuztem Gliagewebe*

umhüllen, welches APÁTHY als „innere Gliazone" bezeichnet hat. Seiner *Form* und *allgemeinen Beziehung* wegen betrachte ich es als ein *Analogon zum stützenden* GOLGI*netz der Ganglienzellen einer grauen Substanz* der Wirbeltiere (Fig. 50). Während aber dort eine kolossal große Gliazelle viele Ganglienzellen umhüllt, sind es zum Unterschied hier mehrere kleinste Gliazellen, welche als Begleitzellen die GOLGInetze ihrer zugehörigen Ganglienzellen liefern (Figg. 18 u. 19). Hinzukommt als Unterschied, daß in den GOLGI-netzen nur vereinzelte Gliafasern laufen; auch sonst wird die Substanz der GOLGInetze eine andere sein.

Die *Sternzellen der Ganglienzellenpakete* liefern nach APATHY eine *äußere Gliahülle* für die ihnen zugehörige Anzahl von Ganglienzellen. Sie haben nach ihm eine Anzahl von „Radiärfortsätzen", welche „aus einem Bündel von größtenteils sehr feinen und einigen etwas stärkeren Gliafibrillen" bestehen. Nur die stärkeren und auf meinen Präparaten dunkel gefärbten Fasern, die im Innern der Sternzelle weitmaschig und spärlich zusammenhängen, möchte ich mit den Gliafaserungen bei Wirbeltieren vergleichen; die übrigen erscheinen mir zu weich dafür. Sie sind außerdem dicht mit *Körnchen* besetzt, welche an der frisch untersuchten Zelle als *glänzende Tröpfchen* hervortreten. Sie scheinen mir weniger die Ganglienzellen zu stützen als zu ernähren geeignet zu sein.

Nun hat HOLMGREEN über die Natur der radiären Sternzellen einige Deutungen gemacht, welche sie als nervöse Zellen und als „große Assoziationszellen" der Ganglienzellen hinstellen, Angaben, die ich an dieser Stelle nicht unwidersprochen lassen kann. Die HOLMGREENschen Beobachtungen selber weichen von denjenigen APÁTHYS ebenso wie die meinigen nicht wesentlich ab. Meine Zeichnungen unterscheiden sich von denjenigen in der HOLMGREENschen Arbeit in der Hauptsache nur dadurch, daß die Fasern und Balken des Glianetzes besonders im intrazellulären Abschnitt ihrer Zellen mehr gleichmäßiger, fester und starrer erscheinen. Besonders in den Figg. 69 und 73, schon weniger in Fig. 70 der HOLMGREENschen Abhandlung sind diese Fasern mehr wellenförmig verlaufend und bald dick und bald dünner in der Masse und auch mehr überkreuzt zu sehen, was vielleicht nur Art des Zeichnens sein wird (siehe hierzu meine Zeichnungen Figg. 47—50).

Das Gitterwerk der medianen Sternzellen und seine Fortsetzung auf die Oberfläche der Ganglienzellen hält nun HOLMGREEN für nervös und für identisch mit den GOLGInetzen der Wirbeltiere, wobei er sich auf die früher von mir, SEMI MEYER und BETHE vertretene Meinung stützt, daß diese perizellulären GOLGInetze nervöse Terminalnetze bedeuten. Ich selber halte nun heute die GOLGInetze als solche nicht mehr für nervöse Netze, wie ich vor kurzem und in dieser Abhandlung weiter nachgewiesen habe.

HOLMGREEN hält sogar auch das *Fasergitter in den APÁTHYschen Konnektivspindeln* für wahrscheinlich identisch mit demjenigen an der Oberfläche der unipolaren Ganglienzellen und jener medianen Sternzellen, die er als „multipolare Nervenzellen" bezeichnet, während er nur die LEYDIGschen Zellen (Seitenkerne von APÁTHY), die ich hier als weitere Gliazellen unberücksichtigt lassen will, und die Sternzellen der Ganglienzellenpakete für große Gliazellen halten will.

Gegen HOLMGREEN führe ich nun aus, daß die *Art der oberflächlichen Ausbreitung jenes anfänglich intrazellulär in den betreffenden Riesenzellen beginnenden Gitterwerkes auch für seine Auffassung und Natur entscheidend* sein muß. Da dieses Gitter *oberflächliche Scheiden um die Nerven eines Konnektivs* ausbildet und trennende Faserbündel oder stellenweise auch mehr gitterförmige Hüllen zwischen und um die Nerven der *zentralen Fasermasse* aussendet und endlich auch die *Stielfortsätze jener Ganglienzellen* und sie selber mit einem Netz von Fasern einfaßt, so ist das alles ein *Zeichen einer äußerlichen Stützhülle* und einer trennenden und zusammenhaltenden *Scheidewand*, nicht aber einer nervösen Einrichtung, wenn nicht ganz besondere Gründe vorliegen.

Der vergleichende Grund aus den GOLGInetzen fällt also jetzt weg, da dieselben im ersten Sinne GOLGIS von APÁTHY und von mir als ein Stützwerk bezeichnet werden, wofür ich meinerseits die obigen Beobachtungen an den Bildungszellen der GOLGInetze, den gliösen Begleitzellen, hier hervorhebe. Die Berufung HOLMGREENS auf die Resultate einer vitalen Methylenblaufärbung kann ich nicht gelten lassen, da diese Methode alles mögliche, auch vieles nicht Nervöse unter Umständen färbt, und ich außerdem ohne weiteres die SIMONschen Beschreibungen und Abbildungen [70]) für nicht identisch mit den hier diskutierten Gitterbildungen halten kann.

Es bleibt ein Grund übrig, der mir seit langem zu schaffen gemacht hat, und der mir vor den diesbezüglichen Arbeiten und

Ansichten Holmgreens bekannt war. Es handelt sich um das *radiäre Eindringen* von *faserigen Anteilen jener inneren Gliazone in das Protoplasma der von ihr umhüllten Ganglienzelle*, welches nach Apáthy an der Entstehung einer äußeren Alveolarzone des Ganglienzellleibes selber beteiligt ist. Holmgreen gibt an, daß von jenem Oberflächennetz „intrazelluläre und in radiärer Richtung verlaufende Fasern" *sehr wahrscheinlich* mit einem tieferen und perinucleären Faserring zusammenhängen, welcher nach Apáthy ein Teil des endozellulären Neurofibrillengitters („Perinucleargitter") im Typus K der Ganglienzellen bildet. Die *Voraussetzung einer Kontinuität* zwischen jenem Oberflächennetz und diesem Apáthyschen Perinucleargitter mit seiner ableitenden dickeren und axialen Primitivfibrille, die Holmgreen gleichzeitig mit jenem gefärbt bekommen hat, hat also für ihn einen Grund abgegeben, jenes Oberflächennetz nicht für gliös sondern für nervös zu schätzen und damit auch jene medianen Sternzellen für sehr wahrscheinlich als riesige Assoziationszellen anzusprechen. Im übrigen hat Holmgreen, wie er angibt, nicht ganz sicher jene fragliche Verbindung sehen können; auch seine Abbildung (Fig. 71) zeigt keine Kontinuität.

Ich selber habe nun bereits 1896 derartige Präparate von Hirudo gehabt und zufälligerweise auch mit der gleichen Methode (Fixierung in Alkohol-Chloroform-Eisessig-Eisenhämatoxylin nach M. Heidenhain) erhalten, wie sie Holmgreen in seiner Arbeit von 1900 beschrieben und abgebildet hat. Merkwürdig ist nur, daß ich zu einer entgegengesetzten Auffassung kam. Da ich auf meinen früheren Schnittfärbungen eine umfassende Färbung von der Glia des Blutegels und ihrem ausgiebigen Eindringen in die Ganglienzellen erhielt und außerdem eine Anzahl von Fibrillen im Innern von Ganglienzellen und Nervenfasern gleichzeitig gefärbt bekam, die den Abbildungen Apáthys entsprachen und mir direkt und unverändert mit einander zusammenzuhängen schienen, so hielt ich mich damals für berechtigt, Apáthy eine grobe Verwechslung seiner Neurofibrillen mit Gliafasern vorzuwerfen. Dieser Zusammenhang scheint mir nun heute *nicht mehr so einfach* zu sein, weil ich jetzt auf etwas anders gefärbten und differenzierten Präparaten die *Gliazone entfärbt* erhalte, jene *Neurofibrillen dagegen noch gefärbt* sehe. Nur auf Präparaten mit gleichzeitiger Färbung beider Anteile finde ich noch jetzt die Schwierigkeit, beide zu unterscheiden. Da ich aber andererseits heute nicht absolut sicher angeben kann, daß beide Teile in einander wirklich übergehen, so habe ich kein Recht mehr jenen Vorwurf länger aufrecht zu erhalten, weshalb ich ihn zurücknehme. Ich kann nur noch von der Möglichkeit eines Zusammenhangs sprechen, der aber von einer Änderung der Substanz begleitet wäre; wobei noch zu berücksichtigen bleibt, daß nach Apáthy ein Teil jener Riesenzellen nicht nur im erwachsenen Zustand einige wirkliche und sie durchsetzende Neurofibrillen außer ihren Gliafasern enthält, sondern überhaupt die Bildung von Neurofibrillen besorgt hat. Ich hoffe in einiger Zeit hierauf nochmals näher eingehen zu können und genauer meine Gründe für eine Entscheidung dieser Dinge zu entwickeln, die für die Auffassung der Nervenleitung wesentlich erscheinen.

Wenn ich nun nochmals auf das Analoge zwischen jenem gliösen Oberflächennetz an der Ganglienzelle eines wirbellosen Tieres mit dem Golginetz um die betreffende Zelle der grauen Substanz zurückkomme, so geschieht es, weil nach Apáthy die Ganglienzellen des Rückenmarkes der Wirbeltiere „keine der inneren Gliazone bei Hirudineen entsprechende Gliazone besitzen, sondern bloß vom mehr oder weniger dichtem interstitialen Gliageflecht umgeben werden."

Ich bemerke hierzu, daß sich mein Vergleich auf die *Netzform der betreffenden Hülle* stützt, die also bei der Wirbeltierganglienzelle von besonderen Begleitzellen als Teile des allgemeinen und interstitiellen Gliagewebes nur gebildet werden. Im übrigen ist natürlich die Masse und innere Form eine andere, die ja bei Hirudo eine mehr grob- und derbfaserige ist und insofern als weiter differenziertes Protoplasmaprodukt jener medianen Sternzellen erscheint. Daß aber auch die Substanz der Golginetze eine besondere Bildung jener Gliazellen ist, dürfte aus dem 4. Kapitel dieser Abhandlung außer meinen früheren Angaben hervorgehen. Im übrigen beteiligen sich auch diese Zellen bei ihrer allseitigen Netzbildung an der Bildung benachbarter Anteile des allgemeinen Gliareticulums der Nervenfasern u. s. w., wodurch sie wiederum im kleinen jenen medianen Sternzellen gleichen, nur daß diese mit viel weiter verzweigten und besonders groben, derbfaserigen Protoplasmaprodukten, die Gliabalken der zentralen Fasermasse liefern.

Was dagegen jene BETHEschen Angaben anbetrifft, wonach die GOLGInetze mit inneren Fibrillen der Ganglienzelle zusammenhängen, so habe ich bisher keine sichere Entscheidung erhalten können, ob hier Verhältnisse vorliegen können, wie sie bei Hirudo vorkommen und einen für ihre dortige Bedeutung noch strittigen Punkt enthalten. Jedenfalls kann ich nach den Gründen, welche ich für eine gliöse Natur der GOLGInetze vorgebracht habe, nicht der von BETHE entwickelten Lehre zustimmen, wonach die GOLGInetze als mit *Neuritenzweigen direkt zusammenhängende Bildungen* auch die *Kontinuität der Neurofibrillen bei Wirbeltieren* vermitteln.

6. Formation der Neuroglia und ihre Bedeutung.

Unter der Formation der Neuroglia verstehe ich die *allgemeine und gegenseitige Beziehung der Neurogliazellen zu einander.* Wodurch die Frage entsteht, ob die *Ausläufer der Neurogliazellen,* mögen sie nun faserhaltig oder faserarm sein oder auch nur aus nackten und stellenweise protoplasmaunbedeckten Gliafasern allein bestehen, unter *einander zusammenhängen* oder nur sich gegenseitig *überkreuzen.*

Es ist bemerkenswert, daß sich die Ansichten hierüber in kurzer Zeit vollständig in ihr Gegenteil geändert haben. Von v. KÖLLIKER ist früher eine allgemein netzige Verbindung der Gliazellen behauptet worden und (Handbuch der Gewebelehre 1867, S. 266) als ein allgemeines Reticulum des zentralen Nervensystems bezeichnet worden, das wie ein zartes Skelett die ganze weiße und graue Substanz durchzieht. Während sich u. a. FROMMAN und GIERKE dieser Meinung gefügt haben, ist von DEITERS[12]) und GOLGI und dann später von RANVIER und WEIGERT jedes Anastomosieren von Gliazellen bestritten worden, wodurch dann die Lehre vom *Neurogliafilz* herrschend geworden ist, wozu ich wiederum auf die Darstellung verweise, die v. KÖLLIKER in der 6. Auflage seiner Gewebelehre (1896) S. 151 von der Einfügung der Nervenfasern der weißen Substanz, z. B. in trügerischen Maschen des Glianetzes gegeben hat. Auch ERIK MÜLLER gibt im Sinne von DEITERS und GOLGI an, daß bei den niederen Vertebraten die Ausläufer von Neurogliazellen diejenigen der anderen nur überkreuzen oder auch am Zellleib fremder Gliazellen in firstenartigen Erhebungen und Rinnen derselben sich anlegen.

Bei der Untersuchung der Formation des Gliagewebes wird *zweierlei* zunächst zu trennen sein, die *faserige Glia* und die übrige, auf ihre *Fortsätze verteilte Protoplasmamenge der Neurogliazellen.*

Darin stimme ich WEIGERT bei, daß die Gliafasern „mehr oder weniger gerade" sind oder in „starr geschwungenen Biegungen verlaufen", daß sie ferner solide sind und „ganz glatt" in frisch fixierten Präparaten. Das kommt auch auf gewissen GOLGIpräparaten nach meiner Meinung dadurch zum Ausdruck, daß, wie Fig. 20c bei einem Langstrahler zeigt, durchweg die sogenannten Ausläufer glatt imprägniert sind. Ich erkläre diese Form daraus, daß die *Imprägnation vom Zellleib auf die „Manschette der Faser"* (RANVIER) und dann auf die *Gliafasern allein* übergegangen ist, woraus sich zugleich bei der Länge der Gliafasern überhaupt der *Typus von Langstrahlen* erklären würde. Daß sich unter Umständen auch einmal die *Gliafasern einer Zelle allein imprägnieren* können, hat ANDRIEZEN gezeigt. Dann kenne ich eine Form von Imprägnation, die an demselben Typus einer Gliazelle *moosige und rauhe Fortsatzflächen* zeigt (Fig. 20b). Hier hat die Imprägnation nach meiner Meinung *zugleich* auch etwas von der *unvollständigen protoplasmatischen Hülle der Gliafasern* sichtbar gemacht, da ich an meinen *gefärbten Präparaten gleiche Rauhigkeiten* in der Protoplasmafarbe des Zellleibes als angesetzte Ecken der schwarz tingierten Gliafasern sehe. Die bekannte Imprägnationsweise, daß die Gliazellen embryonaler oder jugendlicher Gehirne bei der GOLGImethode häufiger rauh imprägniert werden, die erwachsenen und älteren Individuen dagegen mehr glatt, dürfte also vielleicht auf die *Zunahme der Gliafasern einer Zelle,* sowie der Reduktion des zunächst breiteren Protoplasmanetzes und einem leichteren Überspringen der Reaktion vom Zellleib auf die gleichmäßigere

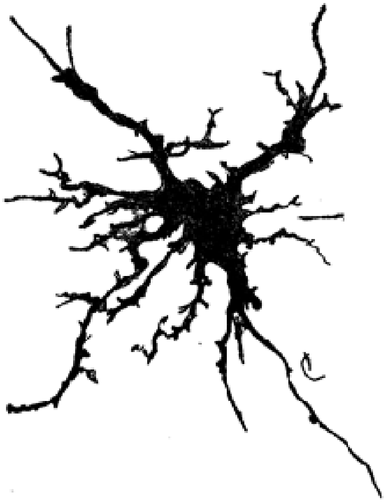

Fig. 20b.

Masse der Gliafasern zurückzuführen sein. Was endlich die Frage nach den *Teilungen von Gliafasern* anbetrifft, so stimme ich wiederum WEIGERT zu, der eigentliche Teilungen nicht gesehen hat. Wozu ich andererseits bemerke, daß *scheinbar einheitliche Fasern aus feineren und eng gepreßten Einzelfasern* bestehen können, die dann an gewissen Stellen (ich erinnere nur an die Form strahliger Gliafüße) eine *Aufbündelung in divergierende Fäserchen* zeigen. Die WEIGERTsche Erklärung von Teilungsstellen der Gliazellfortsätze auf Silberpräparaten („daß — zwei sehr nahe an einander liegende Faserteile zu einer gemeinschaftlichen Silhouette verschmelzen, etwa wie RANVIER es annahm durch Mitfärbung einer verkittenden

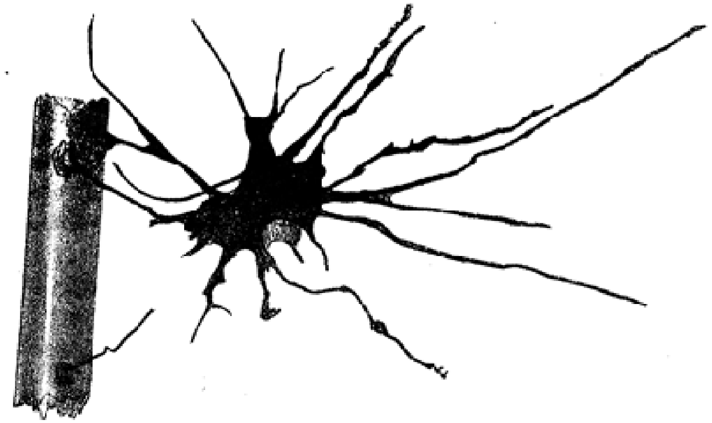

Fig. 10 c.

Substanz") halte ich nur insofern für richtig, als jene Kittsubstanz eine *beide Fasern einschließende Protoplasmahülle* ist, wie ich sie an meinen Präparaten häufiger sehe, ganz abgesehen davon, daß sich teilende, faserfreie Protoplasmafortsätze an Gliazellen vorkommen (siehe hierzu Fig. 5a).

Dazu ist noch etwas für die Gliafaserung Wichtiges hervorzuheben, was bisher unbekannt geblieben und erst vor kurzem von HARDESTY angegeben und auch von mir wiederholt beobachtet worden ist, daß nämlich viele *Gliafasern von einer Zelle in die andere reichen.* Es wäre dies also eine *Anastomose zweier Gliazellen durch eine beiden gemeinsame Gliafaser.* Bei der oben von mir angegebenen Entstehungsweise der Gliafasern in protoplasmatischen

Anastomosen embryonaler Gliazellen ist dies nichts Wunderbares. Mit Sicherheit wird natürlich eine *plurizelluläre Gliafaser* nur dann als solche angesehen werden dürfen, wenn dieselbe so getroffen ist, daß sie in *beiden Zellen* einen vom Protoplasma umgebenen optischen Querschnitt anzulegen erlaubt.

Daß es außerdem eine ganze Anzahl von Gliafasern gibt, die sich nur kreuzen und auch fremden Gliazellleibern nur angelehnt sind, ist etwas, was ich damit nicht bestreite. Ich unterscheide also durchaus an der *reifen* Glia *eigene und fremde* (angelehnte) *Gliafasern,* was aber nur eine Schnittunterscheidung ist.

Nun gibt es aber an der relativ reifen und ausgebildeten Glia (insofern ihre Faserhaltigkeit beim Menschen noch fortwährend mit dem Alter zunimmt, wird dieselbe immer nur relativ fertig genannt werden dürfen) außer jenen Fasern noch Teile, um die man bei der Definition der Gliaformation nicht herum kann. Ich meine weniger hierbei die allgemeine Vereinigung der Gliazellen durch ihre Ausläufer und Gliafüße in der Grenzhaut der Glia, sondern jene *netzige Zwischenmasse,* welche aus den fortsatzartigen oder mehr flächenhaften *Protoplasmaausbreitungen der Gliazellen hervorgeht und zu einem Teil die Gliafasern resp. nur Abschnitte derselben maschig zusammenschließt.* Für die *marginale Glia, die subependymäre Region* und endlich für jene *Begleit- oder Hüllzellen der Nervenzellen* kann ich das mit Bestimmtheit behaupten.

Damit spreche ich ein Resultat aus, zu welchem vor kurzem J. HARDESTY in der erwähnten Arbeit gelangt ist, indem er die Glia als ein *Syncytium* von ektodermaler Natur bezeichnete. Ich füge hinzu, daß ich unabhängig hiervon auf Grund der angegebenen Beobachtungen jener Regionen der Neuroglia dieselbe Vorstellung mir gebildet habe. Für die übrige noch sehr beträchtliche Masse der diffusen Glia kann ich Formationsangaben dagegen bisher nicht machen. Ich verweise aber für die *Auffassung der Neuroglia überhaupt als eines syncytialen Gewebes* auf das, was ich oben über ihre *Entwickelung* angegeben.

HARDESTY operiert für seine Gliadefinition mit *der inneren Form* des *HISschen „Randschleiers"*, indem bekanntlich eine *Abgrenzung einzelner Ependymzellenanteile* nicht mehr möglich und vorhanden ist. Auch mir ist dieser Begriff infolge meiner Untersuchung der marginalen Neuroglia geläufig geworden.

Zu dieser im *Randschleier ausgeprägten syncytialen Form der ependymären Glia* kommt hinzu, daß, wie meine obigen Beobachtungen zeigen, auch die *sekundäre zellige Neuroglia* sich von vorneherein infolge zahlreicher in ihrem Gewebe sich abspielender mitotischer Prozesse aus einem durch *Zellfortsätze im Zusammenhang bleibenden Zellmaterial* aufbaut (Fig. 28a).

Auf dieser einfachen Stufe bleibt nun allerdings die sekundäre zellige Neuroglia nicht stehen. Wie Fig. 28b am Opticus einer neugeborenen Maus demonstriert, sind die Gliazellen *fortsatzreicher* geworden, wobei sie zum Teil nicht mehr anastomosieren, sondern *freie Überkreuzungen* ihrer neuen Fortsätze zeigen, eine Formation, die später bei Schnittbetrachtung noch beträchtlich zuzunehmen scheint (Fig. 28c). Auch in der weißen Substanz des Rückenmarks finde ich Gleiches. In wie weit solche Überkreuzungen auf dieser Entwickelungshöhe in ihrer Zahl noch zu reduzieren sind, da es sich um eine *Durchschneidung gedehnter Anastomosen entfernterer Zellen handeln kann,* vermag ich nicht anzugeben.

Die mitotische Vermehrung der sekundären zelligen Glia aus sich selber wird weiter einige Eigentümlichkeiten der erwachsenen Stützsubstanz der zentralen Substanz erklären können. Hierzu gehören 1) die *Mehrkernigkeit von Gliazellen* (s. Figg. 5b, 9, 18), 2) *breite hautartige Brücken benachbarter Zellen* (Figg. 6, 7), 3) *dünnere* und seltener zu beobachtende *Anastomosen*, die teils rein protoplasmatisch, teils faserhaltig sein können (Figg. 5b, c), 4) jene schon erwähnten *plurizellulären Neurogliafaern.*

Vergleiche ich endlich das Anfangsstadium der sekundären zellig-faserigen Glia, jenes *Stadium*, auf dem *Gliafasern schon als dunkel färbbare, homogene und intrazelluläre Fadenbildungen* in den Fortsätzen wie am Rand des Zellleibes oder auch in seiner Tiefe darzustellen sind, mit dem *erwachsenen Gewebe*, so ist keine Frage, daß eine *protoplasmatische Reduktion* insofern eingetreten ist, als eine feine und kontinuierliche Hülle für die Fasern überall nicht mehr zu beobachten ist. Bei der *hypertrophischen Glia* scheint dagegen wiederum eine *größere Protoplasmavermehrung* einzutreten und umfangreicher jene Gliafasern wieder einzuschließen, was ich in Fig. 13 aus der weißen Rückenmarkssubstanz eines Mannes abbilde, bei dem auch sonst die Gliafasern vermehrt, das Ependym gewuchert und vielfach in der grauen Substanz verteilte Monstregliazellen mit oberflächlich differenzierter Zellplatte und dicken

Gliafaserbildungen, sowie eine beträchtlich verdickte oberflächliche Rindenschicht zu sehen waren.

Ich führe hierzu die Bemerkung STORCHS[73]) an, daß bei „Abnahme der Lebensenergie innerhalb der nervösen Bestandteile der spärliche Anteil von Protoplasma“ um die Gliakerne sich vermehrt und „schwimmhautartig“ die Fasern „vielleicht bis zu ihren Endigungen überzieht“.

Die für die Definition des Neurogliagewebes wesentlichen Dinge fasse ich nun dahin zusammen, daß 1) *die embryonalen Gliazellen intrazellulär die Gliafasern bilden* und daß 2) *die Gliafasern später wohl aus dem manschettenartigen Protoplasma des Zellleibes oder seines Fortsatzes frei heraustreten können, aber auch wieder in dasjenige eines anderen Fortsatzes und Zellleibes eintreten*, wobei sie im Zwischenstück ihres Verlaufes *reine Überkreuzungen* geben können. *Eine Interzellularsubstanz, die zellunabhängig geworden, ist somit die Gliafaserung keineswegs.* Infolge jener netzig geordneten feinsten und modifizierten Verzweigungen des Gliazellenprotoplasmas, die aber mit jenen früheren und gröberen Vorstellungen eines Reticulum nicht verwechselt werden dürfen, die z. B. die einzelnen Nervenfasern der weißen Substanz schon mit echten Maschen umschlossen sein ließen, sind die Protoplasmafortsätze der Gliazellen *nicht einfach verfilzt*, sondern in der Form eines *sehr fein verästelten und netzigen Gewebes* arrangiert. *Was also die Gliazellen syncytial vereinigt*, sind *teils jene mehr protoplasmatischen Anteile*, die in feinster Aufzweigung die *Anteile jenes* BETHE*schen Füllnetzes oder meines Gliareticuium* enthalten, teils jene *Gliafasern*, insofern sie teils nackt, teils wieder von Protoplasma umhüllt überall in die *Zellen und ihre Fortsätze eintauchen. Hinzu kommt die besondere und letzte Vereinigung von faserhaltigen und faserlosen Gliafüßen* zur *Membrana limitans Gliae superficialis et perivascularis*, in welcher durch *Kittlinien eine Vereinigung der hautartigen Fußflächen jener letzten Gliaansätze erfolgt.*

Die *Bedeutung der Neuroglia* liegt, wie allgemein und mit Recht angenommen worden ist, zunächst darin, eine *durchgreifende Stützsubstanz des Zentralnervensystems*, ein besonderes „Bindegewebe“ für Nervenzellen und Nervenfasern zu geben. Zu dieser begründeten Ansicht hat R. Y CAJAL[74]) eine unzureichende hinzugefügt, welche besagt, daß die *Neuroglia kontraktil* sei und mit ihren *wechselnden Stadien einer Erschlaffung oder Ausdehnung* ungleiche Bedingungen

für die nervösen Kontaktverhältnisse gebe, da die Neuroglia sich überall als stromisolierende Masse zwischen die freien Endbäume einschiebe und somit auseinanderziehen müsse. Hinzu komme bei dem Stadium ihrer Kontraktion eine Gefäßerweiterung, welche durch jene am Gefäßrohr angreifenden Gliaansätze die Weite seiner Lichtung bestimme. Zu den ausführlichen Gründen, welche WEIGERT in seiner Neurogliaarbeit (1895)*) und v. KÖLLIKER (Handbuch d. Gewebelehre 1896 II) entwickelt haben, füge ich hier nur hinzu, daß die erwähnte Hypothese auch damit fällt, daß jene Behauptung freier Kontakte, die sich nähern oder entfernen ließen, immer noch eine unbewiesene Annahme ist, die auf einseitig verwerteten Resultaten der GOLGIschen und EHRLICHschen Färbungsmethoden beruht und außerdem bereits eine Summe besonderer Angaben und Gründe gegen sich hat, die in jener Gegenschrift R. Y CAJALS nicht berücksichtigt worden sind.

Es bleibt also bei der Definition, daß die *Neuroglia* eine *stützende Zwischensubstanz für die Nervenzellen und ihre Ausläufer* bedeutet, welche besonders durch die *später erfolgende Faserhaltigkeit* geeignet ist, zunächst das *Eigengewicht der Nervenzellen und ihrer Fortsätze, sowie dasjenige der Gefäße zu tragen.* Es ist das Verdienst WEIGERTS, mit seiner Methode die *Anordnungen der Gliafasern zu architektonischen Geflechtsformen* an der äußeren und inneren Oberfläche des Zentralnervensystems nachgewiesen und erkannt zu haben. Von WEIGERT ist diese Eigenschaft der Glia auch dahin besonders noch gefaßt worden, daß sie eine *raumausfüllende Aufgabe* haben, die zur *Wucherung der Glia* führe, wo *nervöse Substanz zu Grunde* gegangen sei. Es mag hier unerörtert bleiben, ob diese Wucherungsfähigkeit und die Wachstumsgröße der Glia überhaupt erst sekundär eintritt, wenn schon die Nervenzellen lädiert, oder ob auch eine direkte primäre Veränderung und Steigerung jener Energie zu einer Erstickung der nervösen Substanz führt. Jedenfalls wird diese Natur der Neuroglia in ihrer *syncytialen Zusammensetzung aus außerordentlich zahlreichen Kernzentren* mit ihrem *Reichtum an protoplasmatischen Anteilen* und ihrer *Eigenschaft zu ausgedehnten Faserbildungen* sowie in ihrer eigentümlichen und *flächenhaften Beziehung zu den Gefäßwegen des Zentralnervensystems durch ihre Gliafüße* gesucht werden müssen.

*) Die Gegengründe R. Y CAJALS sind in: Algo sobre la signification fisiologica de la Neuroglia. Rev. trimestral micrograph. II. 1. 1897.

Jene Entdeckung GOLGIS von den *Ansätzen der Gliazellen an den Gehirngefäßen* halte ich somit für eine der wichtigsten in der Lehre von den feineren Einrichtungen im Bau des Gehirns, insofern sie die Möglichkeit gab, die Ernährungswege der Nervenzellen überhaupt einem Verständnis zu erschließen. Sie hat ihn bekanntlich zu der Lehre geführt, daß die *Protoplasmafortsätze der Nervenzellen* zum Unterschied von ihren Neuriten *nur nutritive Funktionen* haben, da sie sich ebenfalls mit Gliazellfortsätzen „verbinden". Man sieht, daß in diesen Sätzen GOLGIS bereits die Lehre von BEVAN LEWIS enthalten ist, welche von einem durch die Glia gebildeten Lymphkonnektivsystem spricht, nur daß GOLGI kritischer war und jene „perizellulären Lymphräume" OBERSTEINERS für Retraktionslücken erklärte, während sie bei BEVAN LEWIS (siehe hierzu Tafel V seines Textbuches) eine große Rolle spielen. Daß im übrigen aber die Lehre GOLGIS von der alleinigen nutritiven Funktion der Dendriten nicht richtig ist, dürfte wohl allgemeiner entschieden worden sein. Präzisiert man aber jene Behauptung GOLGIS von einer Verbindung der Gliazelle mit jenen Protoplasmafortsätzen dahin, daß außer anderem jene gliösen Begleitzellen der Nervenzellen (s. oben und hierzu Figg. 18 u. 19) z. B. die perivasculären Räume vorbeistreichender Kapillaren begrenzen und andererseits das Protoplasma von Nervenzellen in Leib und Dendriten netzig einfassen, so erscheinen allgemein diese lokalen Abschnitte der Glia als Bindeglieder zwischen dem Gefäßweg und dem nervösen Protoplasma. Dazu kommt, daß, wie HOLMGREEN gezeigt, das Protoplasma zentraler, also von Glia begrenzter Nervenzellen nicht nur oberflächlich eingefaßt, sondern auch inwendig von einem „Trophospongium" durchsetzt und zerklüftet wird. Also nicht die *Protoplasmafortsätze der Nervenzellen haben als solche eine nutritive Bedeutung für den übrigen Teil der Nervenzelle, sondern es erscheint die Glia, die mit ihrer marginalen Fläche die lymphatischen Räume bestimmt* und auch *andererseits die freien Flächen des Nervenzellprotoplasmas* erreicht, als besondere Ernährerin der nervösen Substanz, die sie zu gleicher Zeit mit ihrem Maschenwerk auch stützt. Für diese erste Funktion der Glia führe ich noch an die Untersuchungen von WLASSAK[75]) welche der primären und sekundären Glia auch die Bedeutung eines aufspeichernden „Übertragungsapparates" der für die Markumhüllung wichtigen Stoffe aus dem Blut zuschreiben, und die Beobachtungen von

NISSL[76]), der den Gliazellen außer ihrer Funktion, den Stoffumsatz in der nervösen Substanz zu besorgen, auch phagocytäre Eigenschaften zuschreibt, wie sie sonst dem Körper der Leucocyten eigen ist, die aber nach ihm in die eigentliche Masse der Großhirnrinde nie eindringen sollen. Die letztere Angabe NISSLS ist mir außerordentlich wertvoll, weil ich erstens selber nie deutliche und sichere Leucocyten in Gehirn und Rückenmark innerhalb ihrer Grenzhaut gesehen habe und deshalb mit NISSL meine, daß jene verschiedenen Angaben wohl auf Verwechselung mit Gliazellen beruhen werden (s. hierzu die Kritik NISSLS über das KRONTHALsche Buch von der Nervenzelle im Zentralbl. f. Nervenheilk. u. Psychiatrie 1902). Außerdem meine ich zweitens, daß erst jene *Grenzhaut der Glia eine Sperre* für die Leucocyten zu der nervösen zentralen Substanz bedeuten kann, und so jene eigentümliche Tatsache eine Erklärung finden würde.

Einige Bemerkungen über die *Neuroglia des Sehnerven* und der *Retina* füge ich hier an. Ihrer Entwickelung entsprechend zeigt die Retina in den *MÜLLERschen Zellen Faserbildungen*, die von der *inneren zur äußeren Grenzhaut durchreichen und sie stützen* müssen. Das innere Ende dieser starken und aus mehreren feinsten Fäserschen zusammengesetzten Stäbe zeigt Fig. 44 vom Meerschwein. Die Membrana limitans Retinae interna mit ihrer Felderzeichnung entspricht durchaus auch nach meiner Meinung den Fußflächen der faserhaltigen Stützzellen und ist ein Analogon zu den Gliahäuten des Gehirns, ebenso wie die MÜLLERschen Stützzellen den faserhaltigen Gliazellen des Gehirns an die Seite zu stellen sind. Der M. lim. Ret. externa zu finde ich die Fasern sehr fein und schließlich in deutlicherer Ringstellung zu den Zapfen und Stäbchenelementen der äußeren Körnerschicht; sie scheinen mir an der Lim. externa aufzuhören, die sie mit einer Anzahl feiner und kleiner Füße aufbauen. Im übrigen finde ich auch an den *Gefäßen der Retina* eine *feine, ihre perivasculären Räume begrenzende Haut,* wie sie für die Hirnkapillaren charakteristisch ist. Für den *N. opticus* füge ich hinzu, daß seine *Nervenbündel gegenüber dem durchsetzenden Bindegewebe* genau so durch eine *Rinden- und Grenzschicht mit Grenzhaut* abgeschlossen sind, wie es im Rückenmark in der weißen Substanz z. B. der Fall ist.

Von LLOYD ANDRIEZEN ist die circumvasculäre Gliahülle als ein Schutz des Hirnparenchyms gegen die Pulsationen seiner

Gefäße bezeichnet worden, die zugleich infolge ihrer nur verfilzten Natur der Saftbewegung von und zu den Gefäßräumen freie Strömung gewähre. Von STORCH ist hinzugefügt worden, daß für die Bewegungen der aus den Blutkapillaren diffundierten Lymphe innerhalb der Gliafaserzwischenräume die Gesetze der Kapillarität gelten müßten, und daß insbesondere vielleicht die Pulsation der Gefäße zu Strömungen der innerhalb des perivasculären Gliagerüstes verteilten Saftmenge führen und ihren weiteren Abfluß zu den subarachnoïdalen Lymphräumen begünstigen können. Von beiden ist aber hierbei übersehen worden, daß die sogenannten Zwischenräume der Glia nach außen zu nicht offen, sondern durch eine *Grenzhaut abgeschlossen* sind, welche den Bewegungen einer lymphatischen Flüssigkeit zum mindesten den *Widerstand einer Filtrationsfläche* aufdrückt. Insofern nun die Membr. lim. Gliae perivascularis als eine feine und zweite Röhre, die dazu von allen Seiten durch das Radiärsystem der marginalen Glia gestützt und gehalten wird, das Gefäßrohr mit seiner adventiellen und ebenfalls röhrenförmigen Hülle umzieht, werden also die Pulsationen der Gefäße im Bereich der muskelhaltigen Arterien *Saugbewegungen* in ihren *perivasculären Räumen* abgeben müssen, welche nicht ohne *einen Einfluß auf die Verteilung einer intramarginalen Flüssigkeit* bleiben kann. Insofern stimme ich also jenen Äußerungen von ANDRIEZEN und STORCH zu.

Besonderen Untersuchungen muß es vorbehalten bleiben, den *Anteil der Glia für eine Saftbewegung in ihrem Protoplasma oder in ihren Maschen* noch näher festzustellen, sowie zu prüfen, in wie weit *besondere und gesonderte Längsströmungen in jenen extramarginalen Räumen*, dem HISschen und dem VIRCHOW-ROBINschen, für den weiteren Abfluß einer lymphatischen Gehirnflüssigkeit nach außen dienen.

II. Die Wand der Lymphgefäße in Haut und Schleimhaut.

Daß ich ein so heterogenes Thema meinen Beobachtungen über den Bau der Neuroglia anhänge, hat seinen Grund in *allgemeinen Verhältnissen*, welche die *Wand der Lymphgefäße in Haut und Schleimhaut zum elastischen Gewebe* zeigen. Ob dies Einrichtungen sind, welche bestimmten morphologischen *Beziehungen der Neuroglia zu den extramarginalen Lymphwegen des Zentralnervensystems* an die Seite gestellt zu werden verdienen, werden meine folgenden Beobachtungen und Ausführungen zeigen. Eine Bemerkung WEIGERTS (l. c. S. 140) schicke ich denselben voraus: „Wie sich der Leser vielleicht erinnert, hatte GOLGI in dieser innigen und verwickelten Verbindung der Neuroglia mit den Gefäßen etwas so Merkwürdiges zu sehen geglaubt, daß er diesen Befund gegen RANVIERS Ansicht von der Fasernatur der 'Zellfortsätze' verwerten zu können meinte. Wir haben l. c. bereits darauf hingewiesen, daß die Verhältnisse der Neuroglia zu den Gefäßen garnicht inniger und komplizierter sind, als die der elastischen Fasern z. B., und wir haben schon daraus entnommen, daß der Einwand GOLGIS nicht berechtigt war. Dazu kommt aber, was wir l. c. nur erst vermuten konnten, daß die ganze Art der Neurogliaverdichtung um die Gefäße herum nichts ist, als eine Teilerscheinung der so verbreiteten 'Rindenschichtbildungen'."

Oben habe ich nachgewiesen, daß die sogenannte Rindenschicht der Neuroglia noch gar nicht den letzten Abschluß der äußeren wie inneren Gehirnoberfläche gibt, welche auch nicht direkt jene Blutgefäße, sondern erst ihre perivasculären lymphatischen Räume begrenzt. Der WEIGERTsche Vergleich zwischen der gliösen Rindenschicht des Zentralnervensystems und der elastischen Faserung der Wände der Blutgefäße hinkt also bereits. Will man überhaupt diese ganze Einrichtung mit solchen in peripheren Organen vergleichen, so wird man die Lymphwege derselben studieren müssen und danach suchen, ob etwa irgend eine ihre Wand begrenzende Gewebsart in ähnlicher Beziehung zu ihr steht, wie die Neuroglia zu den HISschen Räumen. Dann wird man demonstrieren können, daß auch die Beziehungen des elastischen Gewebes zu den Lymphgefäßen der Haut und Schleimhaut ebenfalls wie bei der Glia kompliziertere sind.

Auf in Sublimat-Eisessig fixierten Schnitten von Haut und Schleimhaut (Augenlid vom Menschen, Zunge vom Hund, Darm vom Menschen), die mit meiner auch für die Neurogliafaserung angewandten Hämatoxylinfärbung tingiert waren, fielen mir, noch bevor ich meine Beobachtungen über die gliöse Grenzhaut irgendwie abgeschlossen hatte, Anordnungen von bindegewebigen Zellen und Zellverbänden sowie von Fasern und Fasernetzen auf, welche zu diesen Zellen sowohl wie zu der dünnen Wand von Lymphgefäßen eigentümlich orientiert waren. In Fig. 51 (aus der Mucosa und Submucosa der Zunge vom Hund) und der Fig. 54 (aus der Hautseite des Augenlides vom Menschen) habe ich diese Beobachtungen abgebildet. Beide Abbildungen zeigen ohne weiteres, daß hier der Längsschnitt resp. der Querschnitt von Lymphgefäßen enthalten sind, deren Lichtung durch eine Reihe von Zellen mit vorspringenden Kernen endothelartig begrenzt sind. Der Lymphgefäßlängsschnitt zeigt außerdem eine fast geschlossene Klappe sowie die charakteristischen Erweiterungen des ganzen Rohres.

Zu der Gefäßwand sind nun dunkelgefärbte Fasern orientiert, welche *zu einem Teil* dicht unter seinem Endothel eine *quergetroffene oder auch längsgeschnittene Lage von Fasern*, zu einem andern Teil jedoch eine ungefähr *radiäre Anordnung* zeigen, indem sie sich allseitig aus dem Netz von Zellen und Fasern der Umgebung loslösen, welches in seinen Zwischenräumen entfärbte Bündel kollagener Fasern aufnimmt. Bei dem Lymphgefäß aus der Schleimhaut der Zunge kommt die Besonderheit hinzu, daß auch von den submucösen Enden der Zungenmuskulatur her resp. von den ihr anliegenden Zellen her eine reichliche Menge von Fasern diesem *Radiärsystem des Lymphgefäßes* beigefügt werden. Diesen entgegengesetzt ziehen Radiärfasern von den oberflächlichen Zonen der Tunica propria her zum Gefäß, Fasern also, die von den Zellagern herkommen, die unmittelbar unter dem Epithel, seinen Zapfen und Tälern gelegen sind (Fig. 51 u. 54).

Es ist keine Frage, daß diese Fasern, welche die Lymphgefäße begleiten oder sie radiär erreichen, zum großen Teil *elastische Fasern* sind, also *Anteile des allgemeinen elastischen Netzes der Haut resp. der Schleimhaut*. Die Fig. 55—58 begründen dies; sie sind nach Präparaten gezeichnet, die nach der von PRANTER[17])

angegebenen Vorschrift gefärbt waren, welche mir bei der Sublimat-Eisessigfixierung regelmäßigere Resultate gegeben hat wie die WEIGERTsche Originalmethode. Die Fig. 58 stammt von einem Schnitt, der außerdem noch mit einer alkohol-differenzierten alkoholischen Erythrosinlösung nachgefärbt war, um die Zellbeziehungen der elastischen Fasern zu beobachten, wie weiter unten gezeigt werden wird. Fig. 59 ist ebenfalls nach einer solchen Doppelfärbung gesehen worden.

Die Beziehungen des elastischen Gewebes zum Lymphgefäßsystem in Haut und Schleimhaut sind zuerst von HIS[78]) näher gewürdigt worden, insofern er in dem „reichlichen Vorhandensein elastischer Fasern" eine Einrichtung erkannt hat, „welche die Anfüllung der in bindegewebigen Häuten verlaufenden Lymphgefäßwurzeln wesentlich befördern muß", da ihre Fasern jene Gefäße bei Gewebsdehnungen „klaffend erhalten". Sonst hat HIS „keine bestimmtere anatomische Beziehung der elastischen Fasern zu den Lymphkanälen" gefunden, sondern nur eine Anzahl dem Gefäß längsfolgender Fasern gesehen, die sich aus dem übrigen Gewebe sondern und wieder in dasselbe zurücktreten. Sonst zeigt aber seine Fig. 6 auf Tafel XXIV eine Anzahl von radiärabstehenden Fußstücken, welche auch schon KÖLLIKER (1846) gesehen und als „spitze Ausläufer der Membran" der Lymphkapillare gedeutet hatte. Von GASKELL[79]) sind dann zuerst genauer die anatomischen Anordnungen des elastischen Gewebes zu den Lymphgefäßen an der oberen Wand vom Kehldeckel (Hund und Schaf) dargelegt worden.

Seine Abbildungen zeigen zum ersten Mal das elastische Radiärsystem der Lymphgefäße. Dann hat noch RIEDER[80]) in einer vorläufigen Mitteilung mit Hilfe der WEIGERTschen Elastinfärbung jene *allseitigen Verbindungen der elastischen Fasern der Haut* mit der Wand der Lymphgefäße beobachtet. Er gibt als wichtigen Unterschied von den der Umgebung zu mehr abgeschlossenen elastischen Strukturen der Blutgefäßwand an, daß die „Lymphgefäße mit den elastischen Fasern der Umgebung, ja selbst mit dem elastischen Gefüge der benachbarten Arterien und Venen zusammenhängen."

Meine angegebenen Abbildungen und Beschreibungen bestätigen also jene Beobachtungen von GASKELL und RIEDER. Ich erweitere sie hier noch für den Darmtractus des Menschen, wo ich auch

an den Lymphgefäßen der Subserosa, der Submucosa und des Muscularis solches Radiärsystem gefunden. Bei den subserösen Lymphgefäßen gehen solche elastische Fasern besonders von der Fläche der Längsmuskulatur aus, für diejenigen der Submucosa hebe ich hervor, daß das Radiärsystem durch außerordentliche grobe aber auf dünnen Längsschnitten vom Jejunum außerordentlichen sparsamen Balken elastischer Natur repräsentiert ist. Die durch die Muscularis mucosae endlich zur Mucosa hindurchtretenden Lymphkapillaren sind außerordentlich arm an elastischen Wandelementen; ich finde nur sehr zarte und wenige der Wand folgende Fäserchen. Auch das Radiärsystem ist geringfügig aber noch dort angedeutet zu erkennen, wo ganz vereinzelte und feinste Fäserchen sich von denjenigen der Muscularis mucosae loslösen. Ich erwähne endlich noch, daß das Radiärsystem der Lymphgefäße, sofern es nur deutlich und kräftig überhaupt entwickelt, eine vorzügliche Diagnose gewährt, völlig kollabierte Lymphgefäße zu erkennen. An den tiefen und intrastitiellen Lymphgefäßen innerhalb der Muskulatur der Zunge vom Hund habe ich z. B. derartige für die leichte Erkennung ihrer Wandung außerordentlich wichtige Beobachtungen machen können.

Zweierlei habe ich jetzt näher zu untersuchen, das *Insertionsverhältnis der radiären elastischen Fasern resp. der Ausläufer von Bindegewebszellen an der Lymphgefäßwand* und die *allgemeine Beziehung der elastischen Fasern überhaupt zu Bindegewebszellen.* Was das letztere anbetrifft, so fällt zunächst auf, daß die Anteile der elastischen Netze und insbesondere jene radiären Züge, wo sie aus einem anderen Verlauf in jene Richtung zu einem Lymphgefäß umbiegen, mit den Scheitelpunkten ihrer Kurven oder auch sonst der *Oberfläche von den Zellleibern der Bindegewebszellen und ihrer Fortsätze eng folgen.* Ob an solchen Stellen nur eine engste Anpressung vorhanden ist oder eine Verbindung durch eine irgendwie angeordnete verkittende Masse, die sie zusammenhält, habe ich bisher nicht sicher entscheiden können. Zum anderen Teil aber sind die elastischen Fasern, wie ich an jenen Hämatoxylinfärbungen oder jener Doppelfärbung sehe, mit bestimmten Bindegewebszellen dadurch *vereinigt*, daß eine der Zellmembran *an Feinheit gleichkommende Haut jene elastischen Fasern faßt,* wo sie einer Zellfläche anliegen oder die dünneren und auch mehr lamellenartigen Fortsätze zwischen den Bindegewebszellen erreichen.

In Fig. 60 habe ich diese *hautartige Vereinigung von elastischen Netzteilen mit einer zugehörigen Bindegewebszelle* aus der Mucosa der Hundezunge dargestellt. Fig. 59 zeigt dasselbe aus der Cutis des menschlichen Augenlides auch für jene Anastomosen der Bindegewebszellen. Ich füge hinzu, daß ich in letzterem Präparat auch vielfach solche Zellbeziehungen gesehen, wo die geringen Zwischenräume divergierender elastischer Fasern von einer Bindegewebszelle mit ihrem Protoplasma vollständig ausgefüllt sind, wodurch eine Art von radiärer Anfügung elastischer Fasern zu solchen Zellen bedingt wird. Endlich habe ich bei einem Teil von Bindegewebszellen eine deutliche und sichere *Einfügung von feineren und auch etwas dickeren elastischen Fasern in das Protoplasma eines Zellleibes oder eines Fortsatzes* gesehen (Fig. 59 vom Menschen). Es ist klar, daß differenzierte Färbungen der elastischen Fasernetze derartige Beziehungen zu Bindegewebszellen auslöschen, sodaß *in dieser Hinsicht die summarische Bezeichnung der elastischen Fasern* als einer *reinen Interzellularsubstanz* eine *nicht richtige Vorstellung* gibt. Wie groß der Anteil solcher intrazellulären Vereinigung von elastischen Faserteilen mit Bindegewebszellen ist, vermag ich bisher umfangreicher nicht zu entscheiden. Ich begnüge mich darauf hinzuweisen, daß REINKE[81]), SPULER[82]), FLEMMING[83]) und GARDNER[83]) insbesondere für das elastische Gewebe eine *intrazelluläre Entstehung* der kollagenen wie elastischen Fasern beobachtet haben. Wie schon DEUTSCHMANN sah auch GARDNER die Bildung elastischer Fasern aus Körnchenreihen, die im Zellleib oder in den mit einander verbundenen Ausläufern derselben sich ordneten und zu feinen Fäden verschmolzen, die wiederum mit einander sich zu dickeren Fasern vereinigen können. Hiernach sind also die elastischen Fasernetze intrazelluläre und plurizelluläre (syncytiale) Bildungen. Meine Beobachtungen lassen erkennen, daß auch im *erwachsenen Bindegewebe* eine totale Emanzipation der elastischen Fasern von solchen Zellen wenigstens zum Teil nicht vorhanden ist, sodaß man ebenfalls auch hier derartige Bindegewebszellen als Elastinzellen bezeichnen kann.

Die radiären Beziehungen des Bindegewebsnetzes von Zellen und Fasern zur Wand von Lymphgefäßen sind einmal solche von *gewöhnlichen Ausläufern.* Sie erscheinen auf meinen Doppelfärbungen als verschieden breite oder schmale und dann mehr hautartige *Brücken zwischen den benachbarten Bindegewebszellen und den Endo-*

thelzellen der Lymphgefäßwand selber. Sie sind dort, wo sie am Gefäß ansetzen, fußartig verdickt und gleichen somit in ihrer allgemeinen Form den Bildungen, die HIS früher im Froschlarvenschwanz „als zackige Ausläufer“ der die Gefäßwand bildenden Zellkörper beschrieben hat, die er allerdings „nie mit sternförmigen Bindegewebszellen zusammenhängen“ sah. Ihre Zahl ist verschieden groß, je nachdem die *elastischen Radiärfasern der Lymphgefäße* überwiegen. Die *elastischen Fasern* gehen in besonderer Weise in die Wand der Lymphgefäße über. Es sind nicht einfache Umbiegungen einer radiären Richtung in einen dem Gefäß folgenden Längsverlauf, wie mitunter auch zu sehen ist, sondern es handelt sich hierbei um *Fußbildungen,* die an stark entwickelten Stellen besonders auffallen und an jene Ansätze von Gliafasern erinnern, die ich oben an der Grenzhaut des Gehirns beschrieben habe. Figg. 52a, b und Fig. 58 zeigen dies bei stärkerer Vergrößerung; sie lassen erkennen, daß es *Aufsplitterungen elastischer Fasern in feinste Fibrillen oder Streifen* sind, welche *konisch oder auch trichterförmig* arrangiert sind und schließlich in jene unmittelbar dem Endothel angefügte elastische Faserschicht übergehen, welche als ein sehr feines elastisches Häutchen von enger Maschung jene Endothelzellen trägt. Die Größe dieser Ansätze variiert erheblich; mitunter sind es nur zwei bis vier feinste Fäserchen, die divergierend aus feineren elastischen Fasern hervorgehen; in jenen stärkeren elastischen Füßen, wie sie z. B. Fig. 58 unten zeigt, habe ich gegen 20 zählen können, die aber bald sehr fein und netzig sich verbinden. Es spricht diese Aufsplitterung durchaus für die von GARDNER betonte Zusammensetzung dickerer elastischer Fasern aus feinsten Fibrillen. Der Beschreibung jener elastischen Füße an den Wandungen der Lymphgefäße füge ich noch hinzu, daß ihnen mitunter die protoplasmatische Ausbreitung einer Bindegewebszelle eingesetzt oder angefügt sein kann, die dann weiter mit den Endothelzellen verbunden erscheint. Überhaupt scheint mir die Vereinigung des Endothels mit der elastischen Unterhaut und jenen Füßen eine sehr enge zu sein, worauf ich aber hier nicht näher eingehen will.

Es ist klar, daß die *anatomische Einrichtung an den Lymphgefäßen der Haut- und Schleimhaut* es erklärt, warum die aus den Blutgefäßen transsudierte und unter höherem Druck stehende Flüssigkeit in die geschlossenen Lymphgefäße filtrieren muß, welche

ihr Radiärsystem öffnet. „Es wird sich daher“, wie es HIS bezeichnet hat, „niemals darum handeln können, daß die elastischen Fasern dem Eindringen der Parenchymflüssigkeit in die Abzugsröhren einen Widerstand entgegensetzen; sie werden vielmehr dazu beitragen müssen, die letztere klaffend zu erhalten, und bei einer Dehnung des Gewebes durch Transsudatflüssigkeit, werden sich ihre elastischen Kräfte zu Gunsten einer Entleerung der Flüssigkeit in jenes Kanalsystem geltend machen.“ Durch v. RECKLINGHAUSEN wurde dann eine allgemeine Zellauskleidung, das jetzige Endothel, in den Lymphgefäßen und Lymphkapillaren nachgewiesen, was HIS seinerzeit bis auf jene vorspringenden Kerne noch nicht beobachtet hatte. Daß die Existenz eines Lymphgefäßepithels an der Filtrationstheorie nichts ändert, ist dann von HIS[85]) ausführlich begründet worden, und damit konnte dann GASKEL und besonders auf Grund seiner Beobachtungen über die nach allen Richtungen hin angeordneten elastischen Netze jene allgemeinen Bedingungen für die Filtration einer entstandenen Gewebsflüssigkeit in die Lymphgefäße dahin formulieren, daß der von den elastischen Fasern allseitig auseinandergezerrte und also in geringeren Druck gebrachte Endothelschlauch mechanisch sich mit jener Flüssigkeit anfüllen müsse.

Es ist bekannt, daß die Lymphe reichlicher fließt unter dem Einfluß der Muskelkontraktionen. Für eine Erklärung dieser Tatsache kommen außer den allgemeinen Gewebsdehnungen, die ohne weiteres ansaugend wirken müssen, nach meinen obigen Beobachtungen auch noch jene direkten Verbindungen in Betracht, welche die Muskulatur der betreffenden Organe durch besondere elastische Faserzüge mit ihren Lymphgefäßen verbindet.

Vergleiche ich nun mit den histologischen Verhältnissen an der Wand der Lymphgefäße in Haut und Schleimhaut jene Einrichtungen, welche im Zentralnervensystem um ihre Blutgefäße herum oder an seiner Oberfläche als lymphatische Räume liegen und dem Gehirnparenchym zu durch die gliöse Grenzhaut abgeschlossen sind, so zeigt sich eine gewisse morphologische Übereinstimmung. Wie dort eine *Filtrationsfläche* durch das *Endothel der Lymphgefäße* gegeben und auf einem *Apparat von verästelten Bindegewebszellen* und *einem Netzwerk von elastischen Fasern* befestigt ist, so erscheint am Gehirn eine ähnliche Einrichtung im *System der marginalen Neuroglia mit ihrer geschlossenen Grenzhaut*

enthalten zu sein. Wie die elastischen Fasern mit ihren fußartigen Ansätzen insbesondere dem *Offenhalten* des Lymphgefäßes dienen, so werden auch die *radiären und zum Teil von Neurogliafasern gelieferten Abschnitte der gliösen Grenzschicht* eine gleiche Wirkung auf jene Räume ausüben können, indem sie sie vor einem dauernden Schluß durch den intrakraniellen Druck bewahren. Insofern nun die pulsatorischen und respiratorischen Druckschwankungen sowie die rhythmischen, vom Gefäßzentrum abhängigen Veränderungen in der Füllung der Gehirngefäße auch auf die Umgebung wirken müssen, wird man von Saugwirkungen jener Räume sprechen dürfen, die sich denen vergleichen lassen, welche in den bindegewebigen Häuten des Körpers durch ihre Gewebsspannungen entstehen. Daß jene marginale Glia in ihrer Grenz- und Rindenschicht zugleich eine Schutzhülle für das nervöse Parenchym gegen die Pulsationen der Gehirngefäße oder auch für die dünnwandigen Blutgefäßröhren selber mit ihrer geringeren Adventia bedeuten mag, wie ANDRIEZEN ausgeführt, wird an jener andern Funktion nichts ändern können. Sonst aber werden die Bedingungen für eine Ansaugung und Filtration von Gewebsflüssigkeit in die Lymphräume des Gehirns und ihre weitere Bewegung besondere sein und in ihrer allgemeinen Anordnung natürlich unterschieden werden müssen von denen in Haut und Schleimhaut. Das geht daraus hervor, daß jene Räume im Innern des Gehirns *perivasculär* angeordnet sind, wodurch die Druckschwankungen in den Gehirngefäßen sich unmittelbar in eine Bewegung der in ihren perivasculären Räumen befindlichen Flüssigkeit umsetzen müssen.

Dadurch endlich, daß zwei verschiedene Gewebsarten zu den lymphatischen Räumen des Gehirns zusammenkommen, sind, vielleicht von den feinsten Kapillaren abgesehen, *zwei Filtrationsflächen zwischen das Endothel der Blutgefäße und der nervösen zentralen Substanz* eingesetzt, einmal die *Zellhaut der Intima piae* mit ihrer perivasculären Tiefenfortsetzung und dann die besondere *Grenzhaut der Neuroglia*. In wie weit dieser engsten und eigensten Grenze des Gehirns ein *besonderer osmotischer Wert* zukommt, der für den Stoffwechsel des Zentralnervensystems bestimmend ist, werden erst besondere Untersuchungen zeigen können.

Literatur.

1) R. Virchow, Über eine im Gehirn und Rückenmark gefundene Substanz mit der chemischen Reaktion der Cellulose. Arch. f. path. Anat. u. Phys. 1853.

2) Kölliker, Handbuch der Gewebelehre. 1863.

3) Deiters, Untersuchungen über Gehirn und Rückenmark. 1865.

4) Golgi, Contributione alla fina anatomia degli organi centrali del sistema nervoso. 1871.

5) Ranvier, De la névroglie. Archives des physiologie normale et pathologique. 1883.

6) Weigert, Beiträge zur Kenntnis der menschlichen Neuroglia. Frankfurt 1895.

7) S. B. Pellizzi, Sulla struttura e sull' origine delle granulazioni ependimali. Contributo all istologia e patologia della nevrologia. Riv. sper. Frenatria 22. 1896.

8) A. v. Kölliker, Handbuch der Gewebelehre. 1897. S. 148—153.

9) F. Reinke, Beiträge zur Histologie des Menschen II. Arch. f. mikr. Anatomie. 50. 1897.

10) H. Held, II. Abhandlung über Nervenzellenstrukturen. Arch. f. Anatomie 1897, Suppl. S. 275 Anm.

11) W. F. Robertson, Note on Weigerts theory regarding the structure of the Neuroglia. Journ. of Mental Science 1897.

12) Eurich, Studies on the Neuroglia, Brain 1897.

13) Whitwell, On the Structure of the Neuroglia. British med. Jour. 12.

14) Brodmann, Über den Nachweis von Astrocyten mittelst der Weigertschen Gliafärbung. Jenaische Zeitschr. f. Naturw. XXXIII. 1899. 1. S. 181.

15) Yamagiva, Eine neue Färbung der Neuroglia. Virchows Archiv 160. S. 358. 1900.

16) Ströbe, Über Struktur pathologischer Neurogliawucherungen. Zentralblatt f. allg. Path. u. path. Anat. 1896. S. 864.

17) Storch, Über die pathologisch-anatomischen Vorgänge am Stützgerüst des Zentralnervensystems. Virchows Archiv. 157. 1899.

18) E. Müller, Studien über Neuroglia. Arch. f. mikr. Anatomie. 55. 1899.

19) Studnička, Untersuchungen über den Bau des Ependyms der nervösen Zentralorgane. Anatomische Hefte. Bd. XV. 1900.

20) Obersteiner, Zur Histologie der Gliazellen in der Molekularschicht der Großhirnrinde. Arb. a. d. Institut. 1900.

21) Marinesco, Du rôle de la nevroglie dans l'évolution des inflammations. 2. internat. med. Congress. Paris 1900. (n. d. Jahresbericht v. Schwalbe. VI. 1900.)

22) Z. Dimitrova, Recherches sur la structure de la Glande pinéale. Le Nevraxe. II. 1901.

23) G. Carl Huber, Studies of Neuroglia. The American Journal of Anatomy. I. 1901.

24) Irving Hardesty, The Neuroglia of the Spinal Cord of the Elephant with some preliminary Observation upon the Developement of Neuroglia Fibres. American Journal of Anatomy II. Nr. I.

25) H. Fuchs, Über das Ependym. Verh. d. anatom. Gesellschaft zu Halle 1902.

26) Aguerre, Untersuchungen über die menschliche Neuroglia. Arch. f. mikr. Anat. 56. 1900.

27) W. His, Über ein perivasculäres Kanalsystem in den nervösen Centralorganen 1865.

28) F. Boll, Die Histiologie und Histiogenese der nervösen Centralorgane. Berlin 1873.

29) A. Goette, Die Entwicklungsgeschichte der Unke. Leipzig 1875.

30) Hensen, Zeitschrift für Anatomie und Entwicklungsgeschichte 1876.

31) W. His, Über das Auftreten der weißen Substanz und der Wurzelfasern am Rückenmark menschlicher Embryonen. Arch. f. Anatomie 1883.

32) W. His, Zur Geschichte des menschlichen Rückenmarks und der Nervenwurzeln. Abh. d. math. phys. Klasse d. Kgl. sächs. Ges. d. W. 1886.

33) W. His, Die Neuroblasten und deren Entstehung im embryonalen Mark. Abh. d. math. phys. Kl. d. Kgl. s. Ges. d. W. 1889.

34) Vignal, Sur le développement des éléments de la moelle des mammifères. Arch. de physiologie normale et path. 1884.

35) H. Gierke, Die Stützsubstanz des Centralnervensystems. Arch. f. mikr. Anatomie 25, 26 1885, 1886.

36) C. Golgi, Sulla fina anatomia degli organi centrali del sistema nervoso. Milano 1885.

37) F. Nansen, The Structure and Combination of the Histological Elements of the Central Nervous System. Bergen 1887.

38) R. y Cajal, Sur l'origine et les ramifications des fibres nerveuses de la moelle embryonnaire. Anat. Anzeiger 1890.

Nuevos observationes sobre la estructura de la médula espinal de les mamiferos. Barcelona, 1890.

39) A. v. Kölliker, Zur feineren Anatomie des centralen Nervensystems, II. das Rückenmark. Zeitschrift f. wiss. Zool. 1890.

40) v. Gehuchten, La structure des centres nerveux. La cellule 1891.

41) M. v. Lenhossék, Zur Kenntnis der Neuroglia des menschl. Rückenmarks. Verhandl. der anat. Gesellsch. 1891.

Feinerer Bau des Nervensystems 1892.

42) G. Retzius, Ependym und Neuroglia. Biol. Unters. 1893.

43) Cl. Sala y Pons, La Neuroglia de los Vertebrados. Barcelona 1894.

44) P. Lachi, Contribution à l'histogenèse de la nevrologie dans la moelle épinière du poulet. Arch. it. biol. XV. 1891.

45) G. Valenti, Contribution à l'histogenèse de la cellule nerveuse et de la nevrologie du cerveau de certains poissons chondrostéiques. Arch. it. biol. XVI. 1891.

46) Capobianco, Della partecipazione mesodermica nelle genesi della neuroglia cerebrale. Monit. zool. ital. XII. 1901 u. Arch. ital. biol. 1902.

47) Shinkishi Hatai, On the origin of nevrologia tissue from the mesoblast. Journ. of comp. neurology XII. 1902.

48) H. GIERKE, Die Stützsubstanz des Centralnervensystems Arch. f. mikr. Anatomie 1885—1886.

49) BERGMANN, Zeitschrift f. rat. Medicin. N. F. VIII.

50) C. GOLGI, Gesammelte Abhandl. deutsche Ausgabe 1894.

51) J. HENLE und F. MERKEL, Über die sogenannte Bindesubstanz der Centralorgane des Nervensystems. Zeitschr. f. ration. Medicin 1868.

52) J. SCHAFFER, die oberflächliche Gliahülle und das Stützgerüst des weißen Rückenmarksmantels. Anat. Anzeiger 1894. Beiträge zur Kenntnis des Stützgerüstes im menschlichen Rückenmarke. Arch. f. mikr. Anat. 1894.

53) RENAULT, Insertion sous forme de revêtement épithélial continu des pieds des fibres neurogliques sur la limitante marginale d'un nevraxe adulte. Comptes rend. hebdom. des sc. T. 126.

54) S. KURE, Über die Beziehungen der Glia zu den Gefäßen. Neurologia I.

55) FROMMAN, Untersuchungen über die normale und pathologische Anatomie des Rückenmarkes. Jena 1864 und 1877.

56) L. ANDRIEZEN, On a system of fibre-cells surrounding the blood-vessels of the Brain of Man and Mammals, and its Physiological Significance. Intern. Monatsschr. f. An. und Phys. X. 1893.

57) W. HIS, Über ein perivasculäres Kanalsystem in den nervösen Zentralorganen und über dessen Beziehungen zum Lymphsystem. Leipzig 1865.

58) AXEL KEY und G. RETZIUS, Studien in der Anatomie des Nervensystems und des Bindegewebes. Stockholm 1875.

59) R. VIRCHOW, Über die Erweiterung kleinerer Gefäße. VIRCHOWS Archiv III. 1851.

60) CH. ROBIN, zuerst in Segond, Le systeme capillaire sanguin., Paris 1853, dann in Comptes rendus et mém. de la Soc. de Biol. Paris 1855 u. Recherches sur quelques Particularités de la Structure des Capillaires de l'encéphale. Journal de la Physiol. 1859 (S. 543—545).

61) W. HIS, Beiträge zur Kenntnis der zum Lymphsystem gehörigen Drüsen. Zeitschr. f. wiss. Zool. X. 1859. S. 340.

62) C. J. EBERTH, Über die Blut- u. Lymphgefäße des Gehirns u. Rückenmarks. VIRCHOWS Archiv 49. 1870.

62a) RIEDEL, Die perivasculären Lymphgefäße im Zentralnervensystem und der Retina. Arch. f. mikr. Anatomie 1870.

63) BEVAN LEWIS, A text book of mental diseases with special reference to the pathological aspects of insanity. London 1889.

64) BINSWANGER und BERGER, Beiträge zur Kenntnis der Lymphzirkulation in der Großhirnrinde. VIRCHOWS Archiv 152. 1898.

65) ALBRECHT BETHE, Über die Neurofibrillen in den Ganglienzellen von Wirbeltieren und ihre Beziehungen zu den GOLGInetzen. Archiv für mikrosk. Anat. 55. 1900.

66) HANS HELD, Über den Bau der grauen und weißen Substanz I. Arch. f. Anatomie. 1902.

67) S. PALADINO, Sur les limites précises entre la névroglie et les éléments nerveux dans la moelle épinière, et sur quelques-unes des questions histophysiologi ques qui s'y rapportent. Ach. ital. de Biologie XXII. 1895.

68) STEPHAN APÁTHY, das leitende Element des Nervensystems und seine topographischen Beziehungen zu den Zellen. Mitt. d. zool. Station XII. 1897.

69) EMIL HOLMGREEN, Studien in der feineren Anatomie der Nervenzellen. Anat. Hefte XV. 1900.

70) CH. SIMON, Recherches sur la cellule des ganglions sympathiques des Hirudinées. Internationale Monatsschr. f. An. u. Phys. XIII. 1896.

71) A. BETHE, Über die Neurofibrillen in den Ganglienzellen der Wirbeltiere und ihre Beziehungen zu den GOLGInetzen. Arch. f. mikr. Anatomie 55. 1900.

72) O. DEITERS, Untersuchungen über Gehirn und Rückenmark. 1865.

73) E. STORCH, Über die pathologisch-anatomischen Vorgänge am Stützgerüst des Zentrelnervensystems. VIRCHOWS Arch. 157. 1899. S. 233.

74) R. Y CAJAL Algunas conjeturas sobre el mecanismo anatómico de la Ideacion, Asociation y Atencion. Madrid 1895 und Arch. f. Anat. 1895.

75) R. WLASSAK, Die Herkunft des Myelins, Archiv f. Entwicklungsmechanik 1895.

76) FR. NISSL, Über einige Beziehungen zwischen Nervenzellerkrankungen und gliösen Erscheinungen bei verschiedenen Psychosen. Arch. f. Psychiatrie 1899.

77) V. PRANTER, Zur Färbung der elastischen Fasern. Zentralbl. für allg. Path. u. path. Anat. XIII. 1902.

78) W. HIS, Über die Wurzeln der Lymphgefäße in den Häuten des Körpers und über die Theorien der Lymphbildung. Zeitschr. f. wiss. Zool. XII. 1862.

79) W. H. GASKELL, Über die Wand der Lymphkapillaren (C. LUDWIG, Arbeiten aus d. phys. Institut zu Leipzig. 1877 S. 143).

80) RIEDER, Beiträge zur Histologie und pathol. Anatomie der Lymphgefäße und Venen. Zentralbl. f. allg. Path. 1898.

81) REINKE, Zellstudien, Arch. f. mikr. Anatomie 1894.

82) A. SPULER, Beiträge zur Histologie und Histogenese der Binde- und Stützsubstanz. Anat. Hefte 1896.

83) W. FLEMMING, Über die Entwicklung der kollagenen Bindegewebsfibrillen bei Amphibien und Säugetieren. Arch. f. Anat. u. Phys. anat. Abt. 1897.

84) M. GARDNER, Zur Frage über die Histogenese des elastischen Gewebes, Biol. Zentralbl. 1897.

85) W. HIS, Über das Epithel der Lymphgefäßwurzeln und über die v. RECKLINGHAUSENschen Saftkanälchen. Zeitschr. f. wissensch. Zoologie. 1863. S. 470.

Tafelerklärung.

Tafel I, Figg. 1—19.
Tafel II, Figg. 21—36.
Tafel III, Figg. 37—52.
Tafel IV, Figg. 53—60.
Figg. 20a—c im Text. S. 215 und 293, 294.

Mit Ausnahme der Fig. 49 und teilweise auch der Fig. 47a, welche vor einigen Jahren Herr Lithograph SCHINDLER angefertigt hat, habe ich sonst selber die übrigen Abbildungen mit Hilfe der ABBÉschen Camera gezeichnet. Die Figg. 21 u. 22 betreffen Präparate von Herrn Geheimrat W. HIS, die Fig. 27 ein solches von Herrn Professor S. KÄSTNER, die Figg. 23—26 entstammen einer ALTMANNschen Serie. Die übrigen Abbildungen sind nach eigenen Präparaten gezeichnet. Die Figg. 15—19 sind in Originalgröße, die anderen auf $^2/_3$ reduziert worden.

Tafel I.

Fig. 1. Aus einem Querschnitt durch das Lendenrückenmark vom erwachsenen Kaninchen, der den Zentralkanal und seine Umgebung zeigt. Fixierung in einer erwärmten Lösung von Kal. bichrom. und Amm. molybd. Differ. Alsol-Hämatoxylinfärbung. LEITZ Obj. 4. Oc. 6.

Fig. 2. Aus demselben Schnitt; ventraler Teil des Ependyms. HARTNACK homog. Imm. $^1/_{12}$. Oc. 6.

Fig. 3. Ependym des IV. Ventrikels vom Kaninchen in der Höhe des Facialiskerns. Fixierung in MÜLLERscher Lösung mit sec. Osmierung. Auf Granula differ. Hämatoxylinfärbung. HARTNACK homog. Imm. $^1/_{12}$. Oc. 6. Außer dem Ependym sind zwei subependymäre Gliazellen angeschnitten. Gliafasern dunkelschwarz, Protoplasmakörnchen dunkelgrau.

Fig. 4a und b. Gliazellen aus dem Querschnitt des Kaninchenrückenmarks (weiße Substanz des Vorderstranges).

Fig. 4a eine spongioplasmatische, Fig. 4b eine häutchenartige Gliazelle. HARTNACK $^1/_{12}$. Oc. 6.

Fig. 4c. Oberflächliche Gliazelle aus der ventralen Hirnstammfläche vom Kaninchen, welche mit ihrer Zellleibsfläche direkt die M. lim. Gliae superf. bildet. Gliafasern schwarz, Protoplasmagranula (z. Teil Ringkörner) mattgrau. Fixierung in MÜLLERscher Lösung mit sec. Osmierung. Auf Granula differ. Alsol-Hämatoxylinfärbung.

Figg. 5a—c, 6—10. Gliazellen aus dem menschlichen Rückenmarksquerschnitt (Fig. 7a von einem 21jährigen, die übrigen von einem 24jährigen Hingerichteten). Figg. 5a—c, 5, 8—10 aus der weißen Substanz. Kal. bichrom. Eisenalaunfixierung. HARTNACK $^1/_{12}$. Oc. 6.

Fig. 5d. Aus einem Längsschnitt durch die weiße Substanz vom Rückenmark des Rindes.

Fig. 5a. Quergefaserte Gliazelle; auf den nächsten Schnitten hängt der unten angeschnittene Fortsatz mit einer Gliazelle zusammen. Fig. 5b. Die

obere Gliazelle ist eine faserarme Form von großem und granuliertem Protoplasmaleib. Fig. 5c. Zwei Gliazellen, bei denen die Gliafasern hauptsächlich in den Fortsätzen liegen.

Fig. 5d. Vom Rind; breit anastomosierendes Syncytium von Gliazellen, dessen Protoplasma flächenhaft mehrere Nervenfaserräume umscheidet und zahlreiche Gliafasern enthält.

Fig. 6. Zwei zusammenhängende Zellen; die untere zur Hälfte spongioplasmatisch und radiärgefasert, die obere protoplasmagroß, granuliert und quergefasert.

Fig. 7. Zwei zusammenhängende radiärgefaserte Gliazellen; beide spongioplasmatisch.

Fig. 7a. Radiärgebündelte Gliazelle aus d. Subst. Neurogliae centralis.

Fig. 8a. Protoplasmakleine Gliazelle, radiärgefasert.

Fig. 8b. Protoplasmakleinste Zelle, quergefasert.

Fig. 9. Zweikernige Gliazelle, die stark granuliert ist.

Fig. 10. Protoplasmakleine Gliazelle mit dicken Fortsätzen, die aber rein überkreuzt sind von Gliafasern.

Fig. 11. Querschnittsbild aus der weißen Substanz des menschlichen Lendenrückenmarks (Gegend der hinteren Wurzelzone). Hingerichteter von 24 Jahren. Fixierung in Kal. bichrom. und Eisenalaun. Differ. Hämatoxylinfärbung nach M. HEIDENHAIN. HARTNACK $^1/_{12}$. Oc. 6. (Kein Netz zwischen den Nervenfasern.)

Fig. 12. Aus demselben Schnitt; Durchtrittszone vorderer Wurzeln durch den Vorderstrang.

Fig. 13. Aus dem Lumbalmark vom Menschen; Querschnittsbild aus dem Vorderstrang. Breitere Protoplasmamassen zwischen den Gliafasern. HARTNACK $^1/_{12}$. Oc. 6.

Fig. 14. Netzförmig verzweigte Gliazelle aus der weißen Substanz der menschlichen Großhirnrinde (Hingerichteter von 24 Jahren). Alkohol-Chloroform-Eisessiggemisch. HARTNACK $^1/_{12}$. Oc. 8.

Fig. 15. Aus demselben Schnitt.

Fig. 16. Aus der weißen Substanz des Kleinhirns vom Kaninchen. Molybdänmethode nach BETHE. HARTNACK $^1/_{12}$. Oc. 8.

Fig. 17. Aus demselben Schnitt, der die netzförmigen Gliascheiden im Längsschnitt und zwei Gliaschnürringe zeigt. HARTNACK $^1/_{12}$. Oc. 8.

Fig. 18. Aus der grauen Substanz des Vorderhirns vom erwachsenen Kaninchen; Flachschnitt durch zwei netzförmig verzweigte Gliazellen an der Oberfläche einer großen Vorderhirnzelle. Chrom-Formalin-Eisessig. Differ. Alsol-Hämatoxylinfärbung. HARTNACK $^1/_{12}$. Oc. 8.

Fig. 19. Aus demselben Schnitt.

Textfiguren 20a—c. Gliazellen aus dem Rückenmark eines wenige Wochen alten Hundes. Silberfärbung nach GOLGI. HARTNACK $^1/_{12}$. Oc. 6.

Tafel II.

Fig. 21. Teil des Rückenmarksquerschnittes des menschlichen Embryo EB. Präparat von Herrn Geheimrat W. HIS. HARTNACK $^1/_{12}$. Oc. 6.

Fig. 22. Teil des Rückenmarksquerschnittes des menschlichen Embryo Br 3. Präparat von Herrn Geheimrat W. HIS. HARTNACK $^1/_{12}$. Oc. 6.

Figg. 23—26. Vom Kaninchenembryo. Präparate von R. ALTMANN. Figg. 23—25 aus der seitlichen Oberfläche des verlängerten Markes, Fig. 26 des Rückenmarkes. HARTNACK $^1/_{12}$. Oc. 6.

Fig. 27. Von einem Hähnchenembryo aus dem 5ten Tag der Bebrütung. Präparat von Herrn Professor S. KÄSTNER. Durchschnittsstelle des sensiblen Trigeminusnerven durch die M. limitans Gliae superficialis. HARTNACK $^1/_{12}$. Oc. 6.

Figg. 28a—c. Drei Stadien aus der Entwicklung des N. opticus der Maus im Querschnitt. Fixierung im Chrom-Formalin-Eisessig. Differ. Alsol-Hämatoxylinfärbung. HARTNACK $^1/_{12}$. Oc. 4.

Fig. 28a von einem 12 Tage alten Embryo, Fig. 28b von der neugeborenen, Fig. 28c von der 5 Tage alten Maus.

Fig. 29. Oberfläche des Lendenrückenmarks vom erwachsenen Kaninchen (Bereich des Seitenstrangs). Kal. bichrom. und Amm. molybd. HARTNACK $^1/_{12}$. Oc. 4. Im Innern der weißen Substanz ist die Glia nicht vollständig ausgezeichnet worden. Bei x eine in der M. lim. Gliae superf. mit ihrem Zellleib eingefügte Gliazelle. Der links vom äußeren Blutgefäß befindliche Streifen von dichterer Glia gibt auf den nächsten Schnitten eine marginale und perivasculäre Glia für das dann eingedrungene Blutgefäß ab.

Fig. 30. Dorsale Oberfläche des menschlichen Rückenmarks (Umbiegung zum Septum post.) auf dem Querschnitt vom Lendenmark (Hingerichteter von 24 Jahren). Die Pia mater hat sich glatt von der M. lim. Gliae superf. abgehoben. HARTNACK $^1/_{12}$. Oc. 2.

Fig. 31. Aus demselben Schnitt. Tiefe der Fissura long. ant.

Fig. 32. Aus demselben Schnitt. Begrenzung eines Septum der Pia mater aus dem Hinterstrang, welches nur an dieser Stelle gefäßlos erscheint.

Figg. 33a, b. Kleinhirnoberfläche vom erwachsenen Kaninchen. Chrom-Formalin-Eisessig. HARTNACK $^1/_{12}$. Oc. 6.

Fig. 33a. Senkrechter Durchschnitt durch die Rindensubstanz. Fig. 33b. Flächenbild der M. limitans Gliae superficialis (links) und der Intima piae (rechts).

Figg. 34a—c. Senkrechte Durchschnitte durch die oberflächliche Zone der Großhirnrinde vom Menschen (Hingerichteter von 24 Jahren). Alkohol-Chloroform-Eisessig. Eisenalaunhämatoxylinfärbung nach M. HEIDENHAIN. HARTNACK $^1/_{12}$. Oc. 6.

Fig. 34a. Wenig differenzierter Schnitt. Kammerartiger Bau der marginalen Glia; die Gliafasern erscheinen stellenweise als tiefdunkle Verstärkungsstreifen; Fig. 34c zeigt Differenzierung auf Gliafasern und ihre gefaserten Füße. Fig. 34b zeigt die glatte Lösung einer frisch abgezogenen Pia, die in Fig. 34c dagegen eng und gleichmäßig anliegt. In Fig. 34a ist die Intima piae blasenartig abgehoben, wodurch die Bildung eines epicerebralen Spaltraumes erfolgt ist. HARTNACK $^1/_{12}$. Oc. 6.

Fig. 35. Flächenbild der M. lim. Gliae superficialis an der Großhirnrinde vom Menschen. Gleiches Präparat. Eintrittsstelle eines kleineren Blutgefäßes. Von der Pia sind einzelne verlagerte Bündel angeschnitten.

Fig. 36. Schiefer Flachschnitt von einem gleichen Präparat. Links ist die Grenzhaut angeschnitten, rechts die Gliaräume unter ihr.

Tafel III.

Fig. 37. Querschnitt durch eine kleine Arterie in der oberflächlichen Gliazone der Großhirnrinde vom Menschen. Oben ist ein geringer perivasculärer Raum offen; die adventitielle Scheide ist eng zusammengepreßt. HARTNACK $^1/_{12}$. Oc. 6.

Fig. 38. Eintritt zweier Gefäße in die Substanz der Großhirnrinde; Übergang der oberflächlichen in die perivasculäre Grenzhaut der Neuroglia. Von demselben Gehirn. SEITZ Obj. 4. Oc. 2.

Figg. 39a—c. Gliöse Gefäßeinscheidungen vom Kaninchen. b und c aus der Substantia Neurogliae centralis des Rückenmarks, a aus derjenigen des IV. Ventrikels. HARTNACK $^1/_{12}$. Oc. 6.

Fig. 40. Flächenbild der perivasculären Grenzhaut an einer Vene des verlängerten Markes vom Kaninchen. Die Punkte resp. Striche in den einzelnen Feldern geben die Stellung von Gliafasern unter ihr an. HARTNACK $^1/_{12}$. Oc. 6.

Fig. 41. Gliagrenzhaut an einer Kapillare aus der grauen Rinde vom Menschen (Hingerichteter von 24 Jahren). Links ist stellenweise das Gliareticulum sichtbar geworden. Am linken Ende der Kapillare ein adventitieller Kern, der eine schmale, sehr feine Protoplasmamasse als stellenweise sichtbare Scheide zwischen dem Endothelrohr und der perivasculären Gliahaut umgibt.

Fig. 42. Aus der weißen Substanz einer Großhirnwindung vom Menschen. Alkohol-Chloroform-Eisessig. Stark differenzierte Eisenalaunhämatoxylinfärbung nach M. HEIDENHAIN. Am Gefäßrohr teils parallele, teils radiäre und ansetzende Gliafasern. Infolge der starken Entfärbung und der engen Anpressung ist weder von der Gliagrenzhaut, noch den das Gefäß begleitenden Räumen etwas zu sehen, sodaß der Anschein einer direkten Verbindung von Gliafaserfüßen mit dem Gefäßrohr selber entsteht. HARTNACK $^1/_{12}$. Oc. 6.

Fig. 43. Querschnitt eines Blutgefäßes aus der grauen Rindensubstanz vom Kaninchen. Alkoholfixierung. Die radiäre Grenzschicht der Glia ist zerstört, ein breiter Schrumpfungsraum ist entstanden, in dem nur die Reste von Gliafüßen samt der Gliagrenzhaut am Gefäß geblieben sind. Diese Stelle ist noch eine der besseren, insofern sonst auch die Gliafüße und ihre M. limitans umfangreicher zerstört sind. HARTNACK $^1/_{12}$. Oc. 6.

Fig. 44. Senkrechter Durchschnitt durch die innere Hälfte der Retina eines Meerschweins. Alkohol-Chloroform-Eisessig. Differenzierte Hämatoxylinfärbung. Stützfaserbündel in den MÜLLERschen Stützzellen; außerdem um die Ganglienzellen des Sehnerven und seinen Bündeln ein sehr feines und enges Netzwerk, welches mit den MÜLLERschen Stützfaserzellen zusammenzuhängen scheint. HARTNACK $^1/_{12}$. Oc. 6.

Figg. 45 und 46. Vom Kleinhirn des Kaninchens. Einstichinjektion in die Tiefe. Alkohol-Chloroform-Eisessig.

Fig. 45. An der unteren Windungsoberfläche hat die Injektionsmasse die Pia abgedrängt und einen epicerebellaren Raum gefüllt, der durch die Grenzhaut mit ihren Gliafüßen von einem Schrumpfungsraum getrennt ist. Oben liegt die Injektion auch subarachnoidal, was wahrscheinlich durch Verletzung einer adventitiellen Blutgefäßscheide im Innern der Hirnsubstanz bedingt ist.

Fig. 46. Aus einer anderen Stelle von demselben Präparat. Die Tusche hat sich in dieser Windung unter der Membrana limitans Gliae superficialis gefangen.

Figg. 47a—c. Längsschnitt und zwei Querschnitte durch ein Konnectiv vom Blutegel. Gliazelle und ihre Ausbreitung zu Gliascheiden. a von einem gestreckten, b und c einem verkürzten Tier. Alkohol-Chloroform-Eisessig. Eisenalaun-Hämatoxylin nach M. HEIDENHAIN. HARTNACK $^1/_{12}$. Oc. 6.

Figg. 48a, b. Aus demselben Schnitt; Gliascheide von einem gestreckten Tier, b von einem verkürzten.

Fig. 49. Mediane Sternzelle vom Blutegel; starke Differenzierung, sodaß nur noch der Kern und das Gliagitter als ein von der Zelle scheinbar unabhängiges Netzwerk sichtbar geblieben sind. HARTNACK $^1/_{12}$. Oc. 6.

Fig. 50. Aus einem Längsschnitt durch das Bauchganglion eines Blutegels. Mediane Sternzelle mit ihren Gliaausbreitungen. Das Gliagitter liegt im Zellprotoplasma; seine Ausbreitungen in die zentrale Fasermasse sind nicht ausgezeichnet, sondern nur ihre Fortsetzung als netzförmige Scheide von Ganglienzellen. HARTNACK $^1/_{12}$. Oc. 6.

Fig. 51. Aus der Mucosa und Submucosa der Zunge vom Hund. Längsschnitt eines Lymphgefäßes. Sublimat-Eisessig. Differ. Hämatoxylinfärbung. HARTNACK $^1/_{12}$. Oc. 4.

Figg. 52a und b. Aus demselben Schnitt. Fußansätze elastischer Fasern an der Lymphgefäßwand. In b die Verbindung elastischer Fasern mit einer Elastinzelle. HARTNACK $^1/_{12}$. Oc. 8.

Tafel IV.

Fig. 53. Querschnitt einer Lymphkapillare aus dem gleichen Präparat. HARTNACK $^1/_{12}$. Oc. 4.

Fig. 54. Hautseite des menschlichen Augenlids (Hingerichteter von 24 Jahren). Epithel oben. Sublimat-Eisessig. Differ. Hämatoxylinfärbung.

Figg. 55, 56. Aus den nächsten Schnitten desselben Präparates. Elastische Faserfärbung nach V. PRANTER.

Fig. 57. Aus der Hundezunge. Färbung der elastischen Fasern nach V. PRANTER.

Fig. 58. Aus der Hundezunge. Gleiches Präparat, aber Nachfärbung mit alkoholischer Erythrosinlösung. Epithel unten. HARTNACK $^1/_{12}$. Oc. 6.

Fig. 59. Gleiche Färbung wie bei Fig. 58. Elastinzellen aus dem menschlichen Augenlid. HARTNACK $^1/_{12}$. Oc. 8.

Fig. 60. Elastinzelle aus der Submucosa der Hundezunge. Differ. Hämatoxylinfärbung. HARTNACK $^1/_{12}$. Oc. 8.

Inhaltsübersicht.

Seite

I. Bau der Neuroglia . . . . 201—302
Neurogliazellen und Neurogliafasern . . . . . . 202—235
Allgemeine Einteilung der Neuroglia . . . . . . 208—210
Substantia Neurogliae centralis . . . . . . 210—222
Diffuse Neuroglia . . . . 222—235
Entstehung der Neurogliafasern und die Enden derselben in den Fußstücken der Gliazellen . . . . 236—241
Marginale Neuroglia, ihre Grenzmembranen und ihre Entwicklung 241—275
Netzartig verzweigte Gliazellen . . . . . . 275—285
Elemente der Neuroglia beim Blutegel . . . . . 285—292
Formation der Neuroglia und ihre Bedeutung . . . . 292—301
II. Wand der Lymphgefäße in Haut und Schleimhaut . . . 302—309

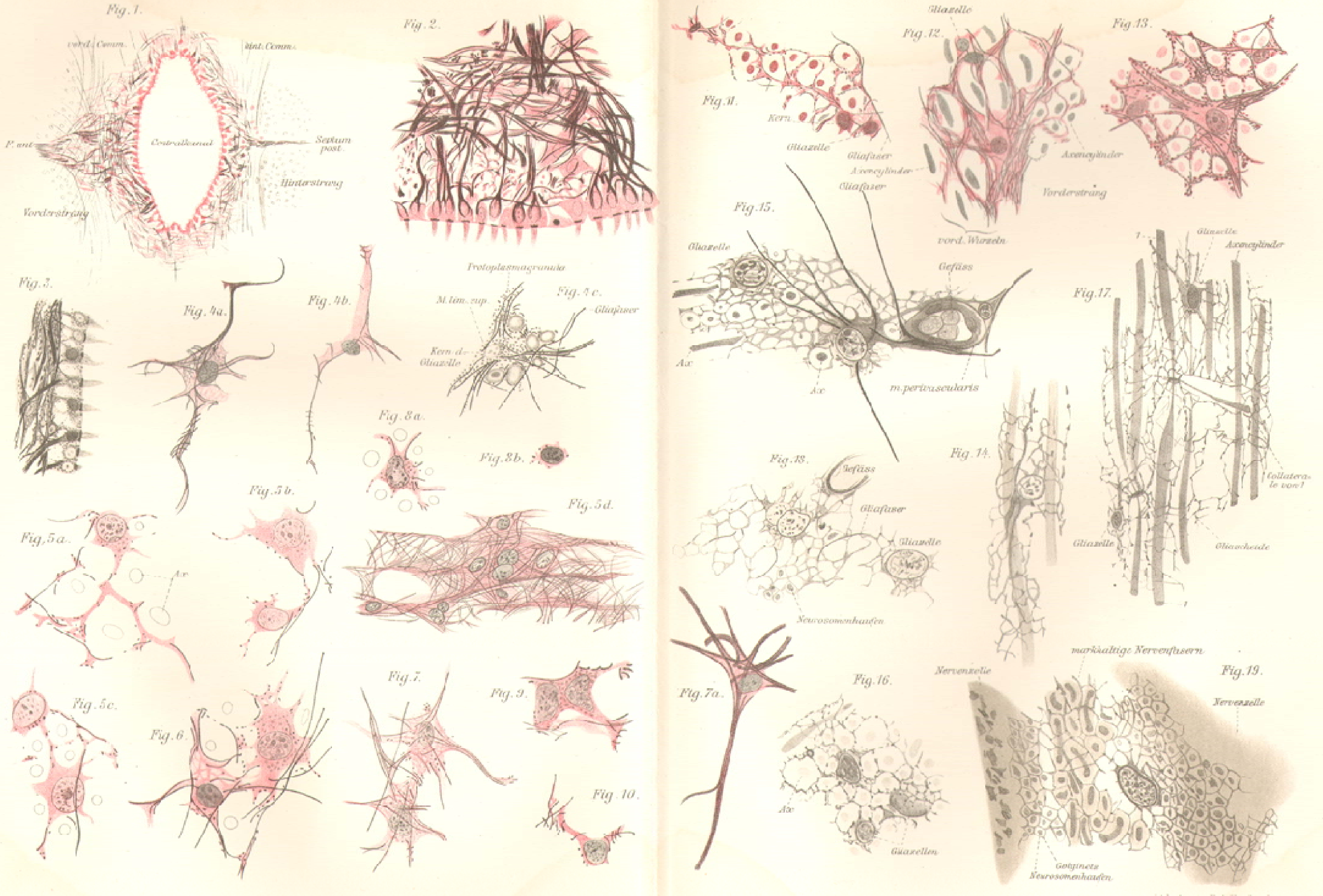

Lith. Anst. v. E. A. Funke, Leipzig.

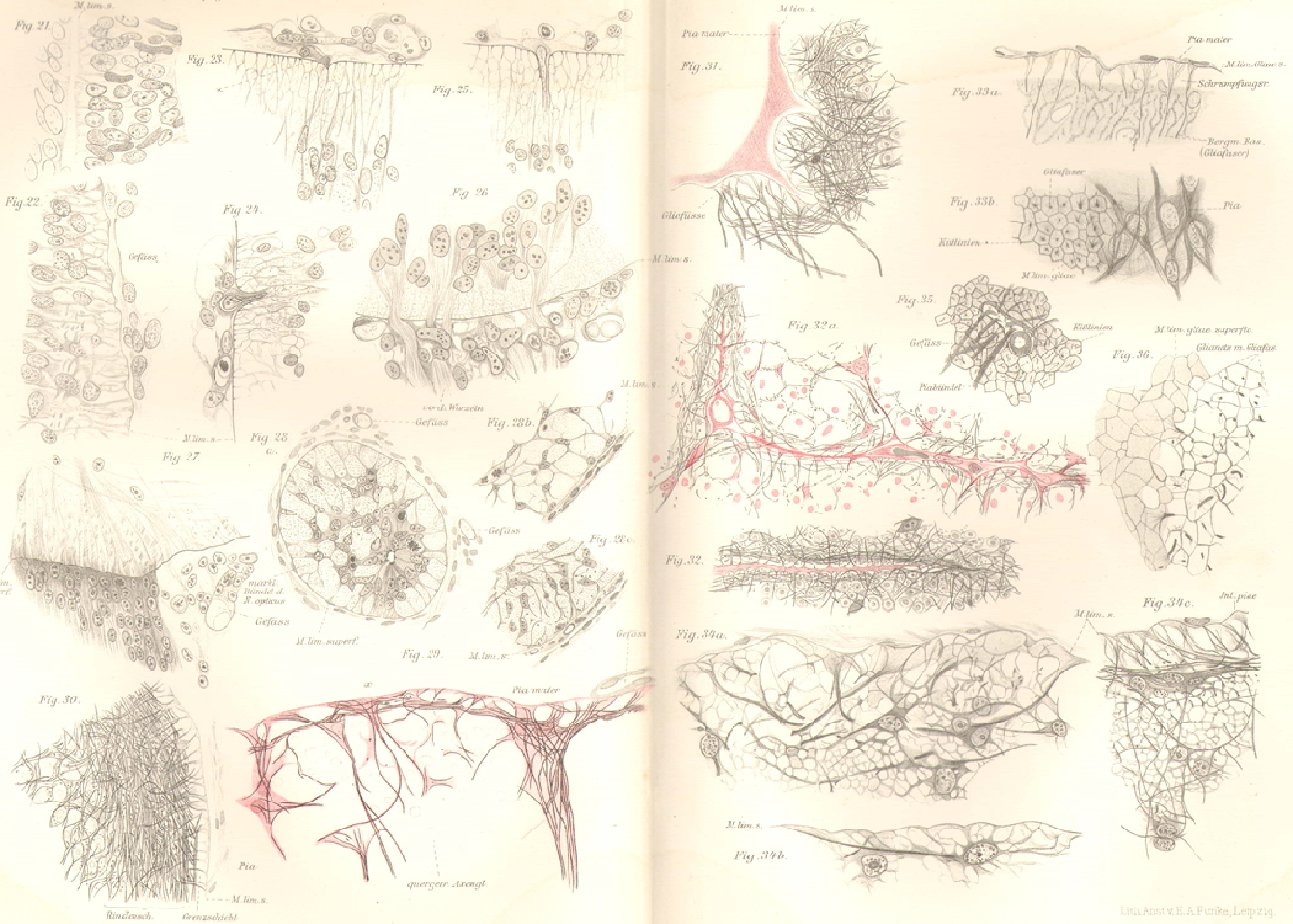

Lith. Anst. v. E. A. Funke, Leipzig.

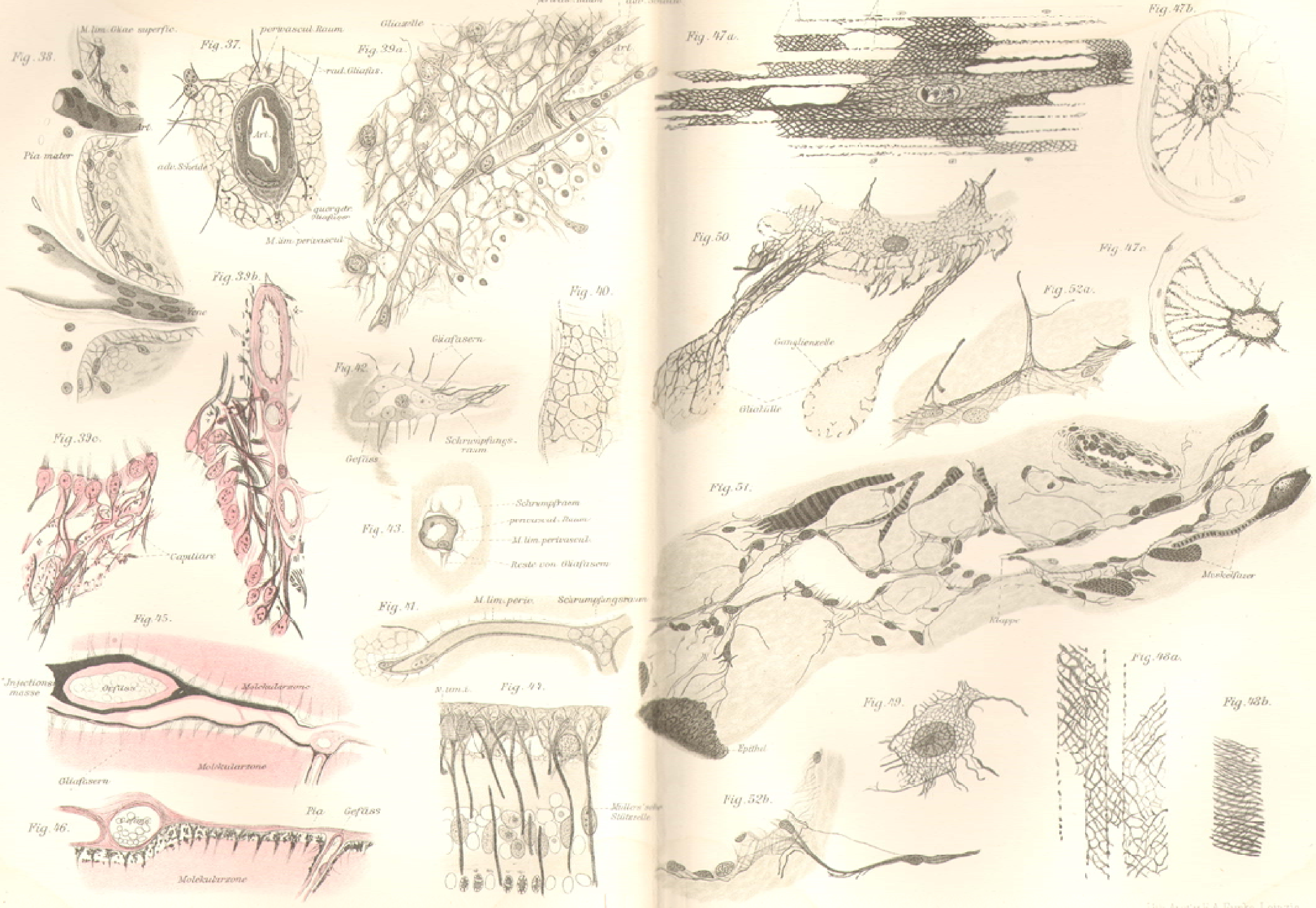

Lith. Anst. v. E. A. Funke, Leipzig

Fig. 53.

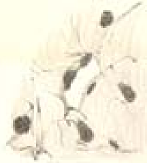

Fig. 54.

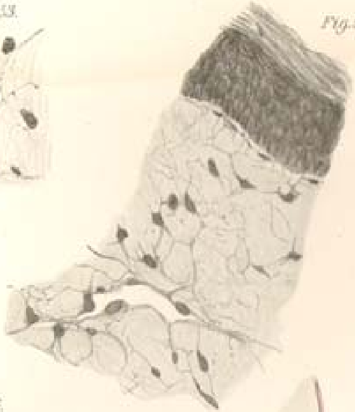

Fig. 55.

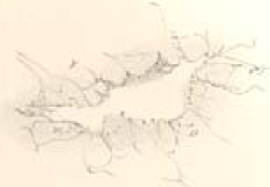

Fig. 58.

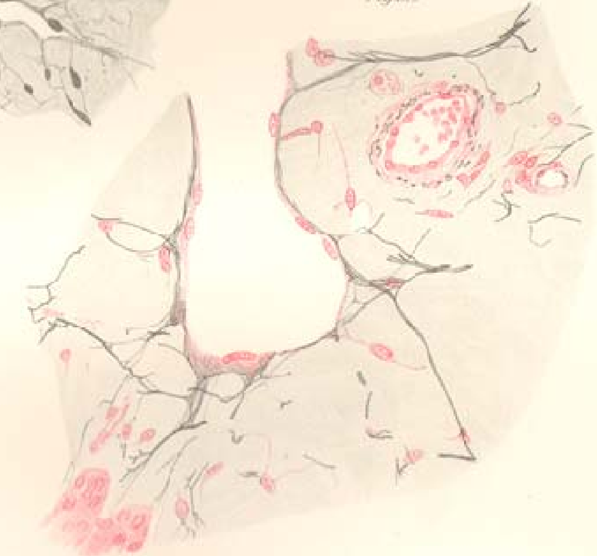

Fig. 56.

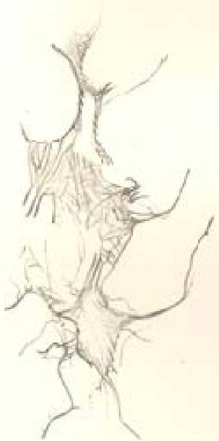

Fig. 59

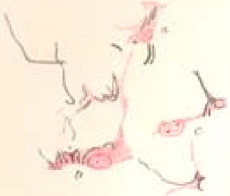

Fig. 60.

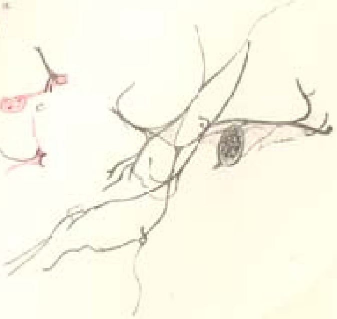

Fig. 57.

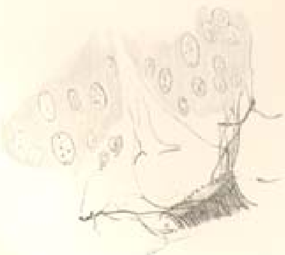

Lith. Anst. v. E. A. Funke, Leipzig
