# Supplementary material for: The Concept of Neuroglia ‐ the State of the Art Circa 1900
Source: Glia. 2025 Feb 4;73(5):890–904. doi: 10.1002/glia.24678 (PMC11920685; doi:10.1002/glia.24678)
Supplement: Supplementary file 10 — Data S10. Translated text by Held with single figures inserted into the text. [file GLIA-73-890-s005.pdf]

On the structure of neuroglia and on the wall of the lymphatic vessels in skin and mucosa

by Hans Held

XXVIII volume of the proceedings of the mathematical-physical class of the Royal Saxonian Society of Science

With 3 figures in the text and 4 lithographic tables

Leipzig

At B. G. Teubner

1903

Lectured for the paper on January 11, 1903

Manuscript submitted on March 18, 1903

Last page printer-ready on June, 14, 1903

I. Structure of the Neuroglia

The elements of neuroglia, cells of the grey and white matter, according to Virchow[1], are commonly placed between the nerve cells and their processes, which they enwrap. Similar to nerve cells, they are of ectodermal origin, as is generally accepted today, and transform during development into complex, multipolar forms of cells. They are distinct in that all of their processes are homogeneous and stable and never transform into nerve fibers. In contrast, they partially form an ensheathment around them (the nerve fibers) and the nerve cells. Second, they neither produce nor accumulate Nissl bodies in their protoplasm. Third, they largely produce a felt of characteristic neuroglial fibers, which serve to stabilize the nervous elements of the central nervous system. Fourth, they enable the formation of border membranes, which are responsible for the complete enclosure of the central nervous system.

I will provide more details about the properties of neuroglia, particularly those which concern the relationship between neuroglial fibers and neuroglial cells, which is still disputed today. In the following chapters, I will develop the concept of the glial border membrane, which has already partly become known as the segregation of the intrinsic mass of the central nervous system from the penetrating blood vessels.

1. Neuroglial cells and neuroglial fibres

While Kölliker[2], Deiters[3], and Golgi[4] postulated that neuroglial cells are star- or spider-type cells with many processes that intermingle with the nervous elements in gray and white matter, Ranvier[5] and Weigert[6] opined that neuroglial fibers do not represent cell processes, but a fiber formation segregated from neuroglial cells, obtaining an independent state. Ranvier came to that conclusion based on an isolation procedure followed by picrocarmin staining; Weigert developed a novel staining method on thin slices, which illustrates, in a beautiful fashion, the particular fibers of neuroglia in the human central nervous system in its topographic arrangement. Based on his staining method, which highlights, besides the fibers, only nuclei, he concludes that:

1. The neuroglial fibers are indeed identical to the processes of Deiters' spider cells or Golgi glial cells.
2. They are not proper cellular processes since they are distinct from the protoplasm based on their material composition.

Weigert therefore concludes that the known images of branched neuroglial cells, in ordinary stainings and particularly when using Golgi’s silver method, are an illusion and that the neuroglia is morphologically similar to connective tissue and contains two components: cells and totally independent, inserted fibers, which are only in contiguity with the cell body.

Is Weigert’s claim that the neuroglial fibers are an intercellular substance in the sense of Max Schultze correct? Can Weigert’s technique verify at all that the neuroglial fibers are such a modified cellular substance and that they are not part of the neuroglial cells?

Weigert’s stain labels, besides the dark blue neuroglial fibers, only the nucleus of a cell, while the protoplasm is completely unlabeled and invisible.

From such a residual staining, one can conclude that these masses of fibers are something special and that they could be distinct from other parts of the protoplasm, but it can never be concluded that the single glial fibers are a true intercellular substance based outside the protoplasm of those cells, since the cells cannot be recognized in these preparations except for their nucleus. How can one exclude with certainty that these glial fibers are not within the protoplasm? That they pass the nucleus, as Weigert's images demonstrate, and that they criss-cross is not relevant for concluding on their intercellular position. Here, I support the conclusion by von Lenhossék.

Therefore, in the following, I will mainly focus on analyzing whether these glial fibers are truly in between cells, as Weigert concluded, or whether they are within the cell. I start with a short discussion on observations that are the basis of the pros and cons of the Ranvier-Weigert postulate.

First, Pellizzi[7] argued against Weigert that only his method shows cell-dependent glial fibers; in reality, they originate from the cell body. Then von Kölliker[8] argued against the new-old Ranvier-Weigert view of the neuroglia. Based on his observation that many Golgi glial cells apparently consist of two parts, namely a cell body and the attached plate from which the processes originate, he formulates the hypothesis: "that the Golgi cells generate out of a part of the protoplasm a unilateral plate which is equipped with processes. Initially, as long as the processes still elongate, this plate is closely connected with the cell protoplasm containing the nucleus. Later, however, it often acquires a different density, maybe even a slightly different chemical constitution, and then it may, under certain circumstances, become separated from the cell body."

That this hypothesis is not correct, I will show later. Then Reinke[9] has argued that the neuroglia of the white matter of the spinal cord consists of many branched cells, and their processes are partially purely protoplasmic. The glial fibers are the products of the glial cells, but they are only partially within the protoplasm, while some others are attached on the outside, and a third class is completely emancipated. Processes of the protoplasm and the fibrils of the neuroglia are supposed to have different or even opposite directions. At the same time, I could demonstrate[10] that only in completely differentiated (adult) preparations, the glial fibers of Weigert appear independent. By using other methods, which show both fibers and glial cell protoplasm, even differently stained, it can be recognized that the glial fibers are indeed connected to the protoplasm of certain cells. Thus, they can be viewed as intracellular formations since they originate partially from the spongioplasmic cellular threads, which might unify to stronger and even free fibers, but they would not lose their contact with their cells.

Furthermore, Robertson[11] emphasized, against Weigert's view, that neuroglia is a purely cellular entity. In line with the hypothesis of von Kölliker, assuming a process-rich cell plate of Golgi glial cells, according to him, the cell bodies of glial cells are equipped with a thin, somewhat shrinking, and particularly stainable membrane, which is still connected with the apparently independent passing glial fibers. Also, von Gehuchten interprets Weigert's images in such a way that the glial fibers only remain stained as chemically distinct cellular processes but are, in reality, connected to the cell body. Eurich[12] also states that the neuroglial fibers are not at all independent of cells.

In contrast, Whitwell[13] argues in the sense of Weigert, namely that the neuroglial fibrils are not cellular processes but form an independent meshwork and that neuroglial cells, nerve cells, and blood vessels are embedded into this meshwork, having a lymphatic function and carried in it like the content of a basket. Moreover, he states that these fibrils are strongly light-refractive, that they do not consist of neurokeratin or elastin, but that they have considerable elastic properties, which he concludes based on their couverture.

The following studies again argue against the Ranvier-Weigert view. Among these are the studies by Brodmann[14] and Yamagiva[15], which investigate the formation of astrocytes in glioma. Brodmann actually states that his histological results support the hypothesis of Weigert regarding the normal human neuroglia. His studies, however, show cells being ray-like branched and having processes originating in a conic form from the cellular protoplasm, which are darker stained at the edges. Towards the periphery, these processes transform into one or more fibers to represent glial fibers, which no longer anastomose among each other. Only particular fibril-striped and broad processes extend to the "perivascular glia sheath." However, through a complex conclusion, this finding brings Brodmann to the above result since, first, he thinks that in the normal glial tissue the glial fibers no longer have connections to the cells, since Weigert's technique shows only in pathological material images of fiber-differentiated astrocytes. Secondly, he came to that result because he has formed the idea that the astrocytes "disappear" as the generator cells for the glial fibers as soon as the process of "fibrillation[(a)16] has ended.

Yamagiva has used his own method to study glioma in the thalamus and also normal brain tissue. His method shows, among others, "simultaneous contrast staining of the glial fibers (in red) and the protoplasm of glial cells (weakly violet)." He sees "tiny red dots representing cross sections of the thin glial fibers in the peripheral region of the cell body" and "cell complexes which are bordered by the fibers." He concludes that the "glial fibers represent differentiated intercellular substance which, however, are not completely or not everywhere separated from the cells." He therefore comes to the conclusion, already previously formulated by Ströbe[16], that there is an "organic connection" between the cell body and glial fibers.

Storch[17] has more extensively analyzed the pathological process in the tissue of neuroglia.

He considers that the glial fibers are an independent intercellular substance and will only admit, for the monstre cells, that there is an obvious and considerable accumulation of protoplasmic substance around the nucleus, which is divided into fiber-containing processes and connected with passing fibers within the cell body. Certain regressive events in those pathologically swollen glial cells should finally lead to the form that Kölliker considered as normal glial cells and on which he had based his assumption that glial fibers originate from a cell plate. I will later return to other findings by Storch related to the border formation of vessels by the glial fibers.

In detailed and profound investigations, E. Müller[18] has demonstrated in neuroglial tissue of lower vertebrates that the glial fibers should be considered, “both on a morphological as well as a physical-chemical basis, as differentiation products of glial cells.” In distinction from the Weigert claim, he argues that there exist beginnings and ends of the glial fibers; the latter are at the vessels and at the subpial border, the former in the glial cells from which they originate and propagate either individually or as a sum of single, converging threads, which is compatible with my assumption. Moreover, Müller expressed the opinion that “the neuroglia forms a transition between purely epithelial tissue and connective tissue,” as a number of cells detach from the primary ependyma and transform into relatively free glial cells, which then produce support fibers, which, however, in contrast to connective tissue, do not become independent but remain connected to the cells.

From the most recent work on the relation between neuroglial cells and neuroglial fibers, we mainly have to consider the studies by Studnička[19], Obersteiner[20], Marinesco[21], Dimitrova[22], Huber[23], and Hardesty[24]. The observations by Studnička show a relationship between the glial ependymal fibers and the protoplasm of the ependymal cells, including in the human nervous system, where Weigert has not observed such a relationship.

Similarly, Obersteiner argues against the Weigert doctrine with respect to glial cells in the superficial zone of the gray matter in the cortex. Additionally, in the glial elements of the pineal gland, Dimitrova finds that some are often only differentiated cell processes, while others are free or attached to the cells at the surface.

Marinesco and Huber, however, support the Ranvier-Weigert hypothesis. The former emphasizes that fibrous glia is independent of the cellular glia, even though he also admits that, additionally, there are protoplasmic processes of the neuroglial cells that terminate in the vessel wall and become particularly apparent only under pathological conditions.

Huber also attributes the glial fibers to the intercellular substance, but mainly based on the observation that the majority of those do not have a regular relation to most of the glial cells. In addition, he emphasizes that there are cells, revealed by combined observation and staining of the glial protoplasm, where one can recognize protoplasmic processes that are not completely separated from the glial fibers and are laterally somehow continuously connected. Huber studied the general relationship between glial fibers and glial cells in dogs, cats, rabbits, pigeons, turtles, and frogs. According to him, the chemical distinction in staining, which emphasizes the independence of the fibers, seems to vary among these different animals. It is most evident in dogs, cats, and turtles, and least evident in pigeons.

If I briefly summarize the results of the above-described literature on the structure of the neuroglial cells, an uncontested merit of Ranvier-Weigert's studies on the elements of the central support tissue is the doctrine that the neuroglial fibers, based on their chemical and physical nature, are a distinct fiber formation within the neuroglial tissue. The other statement, however, that the glial fiber ensemble is an intercellular substance isolated from the glial cells and only loosely adherent to the protoplasm of those cells, seems to be incorrect.

For further justification of those observations that oppose the latter vision of intercellular substances in the neuroglia, and to continue my previous objection, I will now return to my above question and investigate whether the glial fibers are really exclusively intercellular, as Weigert has reported for humans, or whether they still have any connection to the protoplasm of the glial cells.

Own observations and a general classification of neuroglia

My following results refer mainly to the central nervous system of humans, which I have studied in newborns or children only a few months old and in adults in their early and mid-twenties. Moreover, I have studied rabbits and dogs, and partially the spinal cords of calves and cattle. In the explanation of the figures in Tables I-IV, I have added brief methodological information. Apart from that, I will not discuss the methodology of investigating neuroglia since I do not yet have a method for a general staining of glial cells that is specific to this central support substance. At the very least, I have based my conclusions on those regions of the slices that exclude confusion between glial fibers and nerve fibers due to the different bleaching of both elements or the complete bleaching of axons. I will also not describe the architectonic structure of the glial support substance but will instead focus on the elementary relationship between cell and fiber. Only in my third chapter, where I focus on the marginal neuroglia, will I critique some of Weigert's reports related to the topography of the neuroglial fibers.

To obtain a practical classification of the observations made in different regions of the central nervous system, I will develop one here, which also allows for a general classification of neuroglia. Its rationale will become clear in the following chapters; it is based on the regional and, at the same time, distinct relationship between neuroglial cells and their fibers, as a genetic and permanent part of the general mass of the neuroglial cells.

I initially distinguish the Substantia Neurogliae centralis. It corresponds to the previously described Substantia gelatinosa centralis. Since it has been established by Weigert that the area around the central canal of the spinal cord, as well as the walls of the ventricles, are highly enriched with neuroglial fibers, it is no longer appropriate to use this earlier name. This name originates from a limited understanding of the elementary composition of this area and is also used for another region, the Substantia gelatinosa of the hindbrain, which has a low density of glial fibers. Therefore, I propose the name Substantia Neurogliae centralis for the above-mentioned region of the brain and spinal cord.

I will furthermore distinguish two parts of it, namely the ependymal neuroglia, which is formed by ependymal cells and their glial fiber-containing processes, and the subependymal neuroglia, which is formed by a dispersed group of all-around branched neuroglial cells and their fiber mass. At a larger distance, the subependymal neuroglia will continue as glial cells intermingled in a diffuse order between the nervous elements, the nerve fibers, and the nerve cells. In a locally changing manner, the subependymal neuroglia forms distinct and especially adapted support elements for the nerve cells and their processes.

I will describe this as diffuse neuroglia; it is generally subdivided into the gray and the white matter. Finally, one can separate a mass of glial fibers, which more or less corresponds to a defined area of glial cells since it originates from cells of the diffuse as well as the ependymal and subependymal glia. This mass of glial fibers is characterized by a close and important relationship to the outer surface of the central nervous system or to the internal blood vessels. I will describe this as marginal neuroglia. As I will describe in the third chapter, it is this marginal neuroglia that particularly closes off and delimits the His space of the brain from the central nervous system.

it is correspondingly placed partially at the free and partially at the internal surface of the brain and thereby determines either the epicerebral space, respective the perivascular spaces.

After this classification of neuroglia, I will describe their elementary composition out of cells and fibers.

a. Substantia Neurogliae centralis.

The ependymal cells that delineate the Substantia Neurogliae centralis of the ventricular walls or the central canal are considered epithelial. A significant difference lies in the fact that, due to a late postembryonic displacement of individual cellular elements in some animals and particularly in humans, the pure character of a continuous, bonded row of elongated cells, as observed in adult cattle, is lost. In a newborn or a few-months-old child, as I observed, this pure epithelial formation can still be seen.

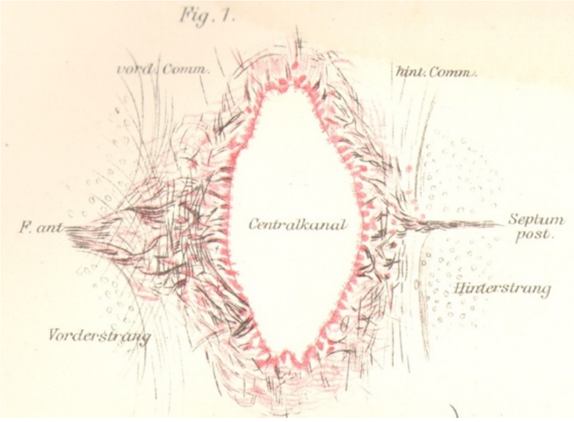

Fig. 1 from Table I. From a cross section through the lumbar spinal cord of an adult rabbit, showing the central canal and its environment. Fixation in a warmed solution of potassium bichromium and ammonium molybdanium. Differential also hematoxylin staining. [illegible]

In a 21-year-old executed human, however, proliferation of the ependymal cells at the dorsal and ventral corners of the central canal caused an increase, a peripheral transfer, and a morphological transformation of single cells into irregular, angled forms. In a 24-year-old executed human, I found that the entire central canal was obliterated and filled with fiber-rich glial cells. This was also the case in a second human who was only a few years older. I observed these conditions as age-related progression, similar to those depicted by Weigert in Table IV, Fig. 4.

In contrast, in the adult rabbit (Fig. 1) and in the dog, there is considerable asymmetry and mutual distance between the single cellular elements with respect to their lateral position and extension into depth.

A second question I would like to address has been raised by Fuchs[25]. He analyzed the inner end of the ependymal cells with respect to basal bodies and cilia in embryos of guinea pig, pig, mole, rabbit, rat, and in young and old cats, and found that they are devoid of true cilia. Opposing Fuchs's statements are older reports that fresh ependyma clearly shows cilia (see Kölliker, *Gewebelehre II,* 1896, p. 144). Regarding the presence of basal bodies at the cilia, this was reported by Weigert (1890) in human embryonic ependyma. Later, he added that he no longer observed them in newborns and in a few-month-old child. However, Studnička reported that basal bodies are also present in adult humans, in the ependyma of the fourth ventricle. Furthermore, Benda in Halle already reported that he found typical basal bodies in ciliated epithelium.

I will add here that I have found basal bodies and cilia, in parts or more extensively, at the floor of the fourth ventricle in a cat fetus, a newborn cat, a 4-week-old dog, at the floor of the fourth ventricle (*Rautengrube*) of a newborn child, and in adult rabbits and dogs. I therefore consider the ependyma, at least partially, as a ciliated epithelium, but I will not decide whether this is regularly or always present.

According to Studnička, classical cilia are not always present, but sometimes strange, stiff spikes can be found at some cell formations. In the central canal of the rabbit, I found cilia and basal bodies everywhere; in a dog, I found scattered ependymal cells without cilia and basal bodies. Conversely, in a few-month-old child, I found only scattered ciliated cells. This indicates that the ependyma is only partially a ciliated epithelium.

Also, in the adult ependyma, one can find single ependymal cells with peripheral protoplasmic processes. These can be observed in the central canal or in the Aquaductus Sylvii, where they are radially arranged and correspond to those known in the embryonic canal based on the Golgi method. Only those process bundles extending to the frontal spinal cord canal, the septum posterius, or the raphe nucleus of the brainstem show a slight curved condensation and deviation from the radial course.

In the spinal cord of the rabbit, these two parts are composed of very coarse processes. In a dog, they are less well pronounced. In contrast, in calves and cattle, I found them more evenly formed out of multiple fine processes, and they are somewhat distinct from the purely lateral and gray matter-oriented group of processes in terms of size and diameter. In humans and cats, they are similar to those in rabbits, although less unilaterally developed.

For the floor of the fourth ventricle, I would like to add that the “ependymal wedge of the raphe” in the adult nervous system is also composed of strong and curved processes oriented towards the dorsal beginning of the raphe of an adjacent group of ependymal cells. For the lateral parts, I found fine processes that either originate as a single process from a cell and branch frequently, or, in some cases, multiple processes are observed, as reported by Studnička in Table 32 and 33, Figures 5–10, in his report[19].

All the peripheral processes of these ependymal cells in the central canal or on the floor of the fourth ventricle show finer or coarser branches that form a fine or coarser meshwork in the Substantia Neurogliae centralis. This meshwork is coarser and more obviously meshed immediately underneath the ependyma of the fourth ventricle, while it is finer in the spinal cord. The size of the mesh also depends on the type of fixation material and the method of embedding; it is less pronounced using Müller’s solution and subsequent embedding in celloidin, and coarser in paraffin slices with the same fixation.

When using nitric acid as a fixative, the size of the mesh is more homogeneous, but it appears finer, more delicate, and even ruptured when using alcohol or picric acid. I therefore cannot provide a definitive criterion for the natural condition of this formation. I will provide more information below.

The peripheral processes of the ependymal cells contain multiple internal fibers, as is the case in the spinal cord. Others, however, are purely protoplasmic, which is the case for most of the processes in the lateral parts of the ependyma of the fourth ventricle. In the ependyma of the central canal of cattle, calves, dogs, and humans, one can find an uneven formation of such fine support fibers throughout the entire field. Instead of single fibers, there can be whole bundles of fibers that calyx-like surround the nucleus of the cell within the cell body. However, in the animals mentioned above, such bundles are usually rare and restricted to the raphe parts of the ependymal processes, or to the pure dorsal and ventral radial processes of the spinal cord ependyma. In humans, the dorsal and ventral bundles are more pronounced. Occasionally, I have also observed them in cats and dogs, but they are least developed in calves and cattle.

In rabbits, this is different, as the peripheral processes of the central canal ependyma commonly contain many bundles of fibers (Fig. 1). In particular, those running towards the frontal cleft are strongly developed. Correspondingly well-developed, of course, is the intracellular origin of those bundles, which form a perinuclear calyx (Fig. 2). The single fibers of the processes can be followed to the nuclear zone, where they are closely associated with the wall or appear as membranous reinforcements. Sometimes, however, they extend only to the origin of the peripheral process, or they terminate even earlier as prominently stained fibers, so that the origin of the peripheral process is occasionally fiber-free, as is sometimes evident in the lateral parts of the ependyma of the fourth ventricle. This could potentially also be an artifact of the method.

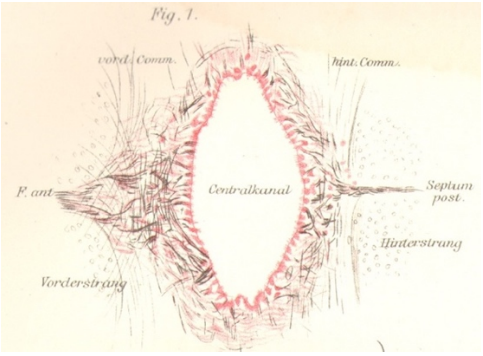

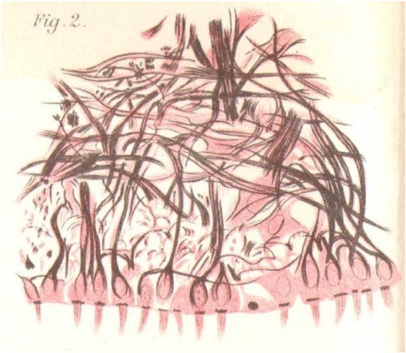

Fig. 1 from Table I. From a cross section through the lumbar spinal cord of an adult rabbit, showing the central canal and its environment. Fixation in a warmed solution of potassium bichromium and ammonium molybdanium . Differential alsol hematoxylin staining. Leitz Obj. 4, Oc.6.

Fig. 2 from Table I. From the same slice, ventral part of the ependyma. Hartnack homogeneous immersion 1/12. Oc. 6.

If there are only 2–4 single fibers, I find them irregularly distributed in the cell body, and they are sometimes attached as a thin bundle with obvious and dark staining. In my preparations, the origins of these intracellular ependymal glial fibers, as I would name them, appear with dull tips, but I could never follow them up to the basal bodies. Only in the ependyma of the Petromyzon[b] could I almost always follow these single glial fibers in an inner fiber cone within the peripheral processes, which reach the basal body as described by Apathy for the fiber structures of the ciliated cells. Apathy describes this fiber cone of the ciliated cells as a cell structure responsible for nervous conduction.

In Lumbricus terrestris[c], I could stain these fibers more extensively in the ciliated epithelium. Since I have never observed that they continue as “nervous fibrils” but are present only at the floor of the ciliated cells, I will therefore consider them solely as an internal support structure of the ciliated cells. Whether it is identical with the system described by Apathy, I cannot decide. In my preparations, the lower end of the main fibers is not pointed, sometimes even somewhat enlarged. At the other end, the cone of the fine fibrils appears less uniform.

The ependymal glial fibres continue mainly into the peripheral processes of the ependymal cells; single ones, however, extend into the lateral processes what can be observed particularly in those which contain many fibres (Fig. 2). In cross sections of the spinal cord and brain stem they bend into longitudinal direction so that they can no longer be followed. Sometimes the peripheral main processes also bend from an initially transverse into a longitudinal direction and the glial fibres follow this path.

This is the origin of a large part of the glial fibres which are found in cross sections of the spinal cord slightly below the ependymal cells and form the fibre zone of the cross sections. They are less developed in rabbit while they form a strong and characteristic mass of the substantia neurogliae centralis in dog, calf, cattle and human.

The same can be stated for the whole lateral area of the ependyma in the floor of the fourth ventricle (rhomboid fossa). In a new born or few months old child, these fibres are only weakly developed while they are more extensive in the spinal cord of humans in their twenties. I can therefore support Weigert's claim that "in older individuals there are more and more vertical fibres". In addition to this fibre mass there are ones which originate from glial cells in the close vicinity as I will show below.

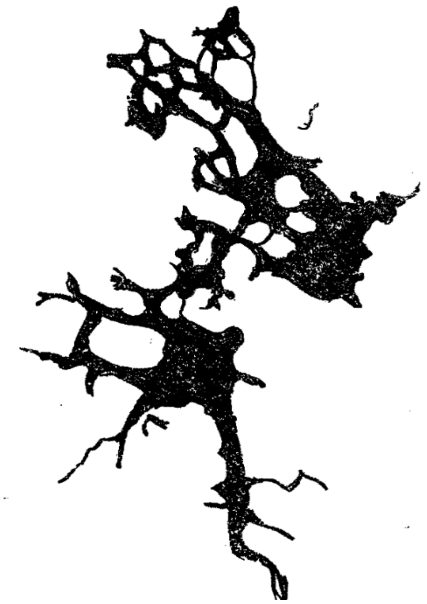

Fig. 20a. From a cross section of the spinal cord of a few week-olds dog. Silver method according to Golgi.
Two glial cells of the white matter of the anterior funiculus are connected in a net form, in a level difference of 60 microns. This figure should demonstrate that the Golgi method does not always show single, branched glial cells as shown in the figures 20b and e (p., 293 – 294); this illustrates that for the doctrine of glial cells the method does not always gives unambiguous results.[ee)]

Within this fiber mass of the Substantia Neurogliae centralis, peripheral main processes of the ependymal cells infiltrate and thereby separate them depending on their number and arrangement. Some of these processes penetrate further into the outer regions of the Substantia Neurogliae centralis. This area is already characterized by numerous glial cells and glial fibers arranged in a ring or in waves. Moreover, many nerve fibers of the commissure are found in the Substantia Neurogliae centralis.

Most of the ependymal glial fibers, or respective bundles, are cut off in cross-sections, indicating that a purely cross-radial course of the processes of the ependymal cells is not present in the adult nervous system. Therefore, it is not possible to directly illustrate, as E. Müller demonstrated in Amphioxus[(d)] or Myxine[(e)], the course of the ependymal glial fibers up to the surface of the spinal cord or the ventral brainstem. Additionally, it is evident that in the spinal cord, the lateral processes of the ependymal cells become very fine and can no longer be followed within the mass of the other glial fibers.

I cannot determine with certainty whether this is absent in the adult spinal cord, also due to the later reduction of penetrating processes, as has often been stated by others. In any case, some processes do not terminate at the surface but rather at the vessels in the vicinity of the central canal or the adjacent gray matter (Fig. 39 a, b, c). However, for another part, I can confirm that it still extends to the surface. This bundle is formed by strong clusters of glial processes that extend to the frontal cleft of the spinal cord.

In rabbits, for instance, it extends in a purely cross course and inserts into the *Membrana limitans Gliae superficialis* (see below). A second strand, consisting of more diagonally oriented bundles, extends to the border layer of the septum posterius and also belongs to this support system. In the other mammals I studied, this radial course is much less pronounced, but these directions and terminal courses can also be assumed in humans based on the strong bundles that are diagonally cut in rabbits. These strands, which actually extend vertically in humans in a wave-like form, are apparent within the crisscross of the glial fibers in the Substantia centralis. I would like to state this also for the raphe of the brainstem, based on the similar course and strength of these processes bordering the ependymal cells near the raphe. With respect to the main question, namely the relationship between glial fibers and ependyma, my statements above show an intracellular origin, and it can thus be concluded that these parts of the fibrous neuroglia can be named ependymal neuroglia fibers. The characteristics of the ependyma of higher vertebrates and humans are, in general, similar to those described for lower vertebrates by E. Müller. This researcher (E. Müller[18], p. 50) has already briefly noted that “the neuroglia is in structural aspects similar in these higher (rana[f], bombinator[g], lacerta[h], rabbit, cat) and lower vertebrates.”

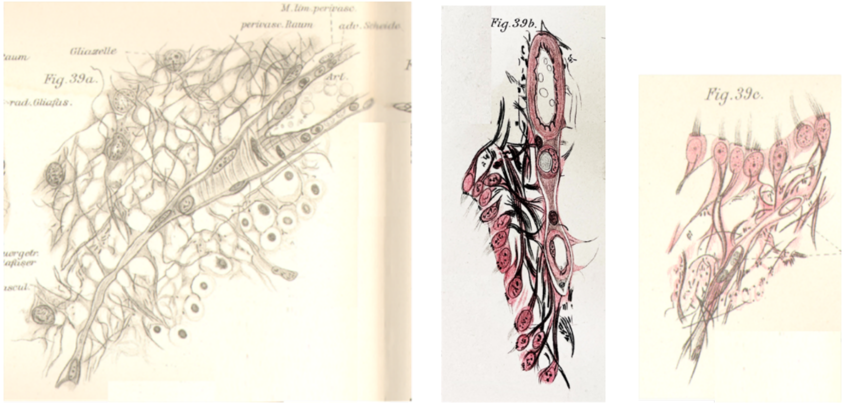

Fig. 39 from Table III. Glial vessel insertion in rabbit.
a from the IV. ventricle.
b from the Substantia Neurogliae centralis of the spinal cord.
c from the Substantia Neurogliae centralis of the spinal cord, Hartnack 1/12 Oc. 6.

However, such strong ependymal glial fibers that surround the nucleus of the ependymal cells in a cup-like fashion are obviously not present in lower vertebrates, at least not in the series studied by E. Müller (Amphioxus[d], Myxine[e], Acanthias vulgaris[i], Salus merlangus[j], Pleuronectes platessa[k]). Greater or total agreement is observed in those types of ependymal cells that have protoplasmic processes with only a few or a single glial fiber.

The subsequent course of these intracellularly originating single fibers or bundles is initially still located within the corresponding protoplasmic process. Along the intensely black-labeled fibers, a reddish-stained mass runs, which is a direct extension of the nucleus-containing cellular protoplasm and can also contain protoplasmic granular bodies. One can also often recognize a reddish-colored, protoplasmic granulated cover, for instance, in those strong fiber bundles that extend to the ventral cleft of the spinal cord, the dorsal septum, or the branching lateral fibers cut crosswise.

Sometimes this cover appears stronger and more obvious; other times, it is weak and unimpressive, appearing as a thin cover. In some cases, it cannot be recognized at all, which raises doubts about whether this is a continuous cover of the glial fibers. At the fine processes containing only single fibers, this cover cannot be recognized with certainty at some distance from the ependymal cell. It remains a difficult question whether these fibers originating from the intracellular space continue completely free or naked.

Since I will return to this issue later, it is enough to state here that a cover becomes apparent again on those free glial fibers or bundles that terminate at the *Membrana limitans Gliae superficialis or perivascularis*.

This feature cannot be recognized in the figures of ependymal glial fibers provided by Weigert (Fig. 2, 3, Table III; Fig. 1, Table V; Fig. 2, Table VII; and Fig. 1, Table XI).

Only Fig. 3, Table XII shows an exception. In the text, Weigert mentioned (6, p. 156) that he has no objection to Kölliker's statement "that the fiber meshwork of the Substantia Grisea centralis also includes contributions from ependymal fibers." His method simply does not allow determination of such contributions in humans. Weigert also states that, "if the epithelial cells contribute to the fiber formation of the central canal, then they produce typical neuroglial fibers."

I agree with this, namely that the single fibers I observed in the human ependyma of the central canal, as well as in the floor of the fourth ventricle, are typical neuroglial fibers located in the peripheral processes of those cells and that they have an intracellular origin. I also consider that Fig. 3, Table XII (surface epithelium of the thalamus), studied with high magnification and proper staining of the cellular protoplasm, would reveal a connection between the fiber bundles at the depth of the thalamus and the ependymal cell layer.

Regarding the question of ependymal glial fibers, it must be noted that the glial fibers are not always aligned with the radial processes. In the lateral parts of the central canal, and in the ventral part of the floor of the fourth ventricle (dog, human), the glial fibers are perpendicular to their processes. As a result, they appear as transverse fibers or as fiber points within the initial parts of the processes, contrasting with the long-fiber raphe bundles or the more ventral and dorsal-oriented processes of the cells in the central canal. It is clear that such an intracellular relationship can be easily overlooked in differentiated glial fiber preparations.

This makes it even more likely that the subependymal layer of glial fibers will appear independent of the ependyma itself. These relationships are crucial for assessing the subependymal and diffuse neuroglial fibers with respect to their connection, or lack thereof, to the actual glial cells. If one observes in Weigert's figures only a crisscross of glial fibers running obliquely or transversely beneath the ependymal surface, it cannot be excluded that this fiber meshwork is connected to ependymal processes.

Based on these observations and conclusions, I distinguish, as a first zone within the Substantia Neurogliae centralis, an ependymal neuroglia, which is formed by the ependymal cells and their fiber-rich or fiber-sparse processes. The extension of that zone naturally depends on the length of the ependymal processes, which is impossible to determine in the adult nervous system based on the conditions described above. Therefore, I can only conclude that in the area of the ventral spinal cord cleft, the parts oriented towards the posterior septum, and the raphe of the brainstem, the Substantia Neurogliae centralis, with its first zone, including the ependymal neuroglia, reaches the pial surface of the central nervous system in a band-formed fashion. For obvious reasons, I cannot determine how far the vertical fibers extend as part of that zone.

The ependymal neuroglia is completed by a series of cells and their fibers, which are typical neuroglial cells, characterized by their radial shape, forming a layer of subependymal neuroglia that rarely contains nervous elements. The demarcation of the subependymal glial cells toward the interior can be partially difficult to determine, especially when the ependyma is no longer composed of a continuous row of attached ependymal cells, as observed in the central canal of rabbits, dogs, or adult humans.

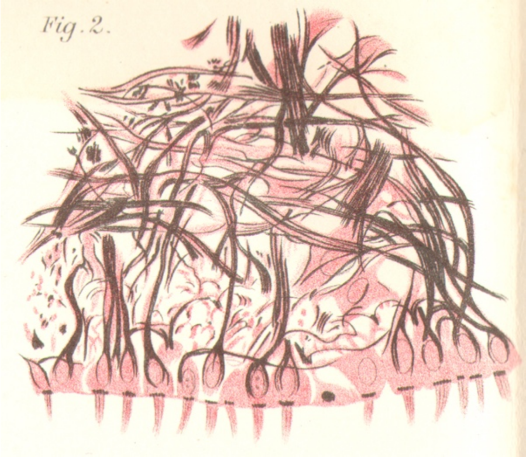

Fig. 2 from Table I. From the same slice (From a cross section through the lumbar spinal cord of an adult rabbit, showing the central canal and its environment), ventral part of the ependyma. Hartnack homogeneous immersion 1/12. Oc. 6.

In the adult nervous system, one finds formations reminiscent of the embryonic emigration of ependymal cells and their conversion into astrocytes, documenting the late formation of subependymal neuroglial cells from the ependyma. These formations, shown partially in Fig. 2 of Table I, consist of single or grouped ependymal cells that have moved deeper into the tissue and have lost their elongated cylindrical or cubical form, becoming polygonal. They may still be connected to the inner epithelium by a shaft or may have pushed so far outward that they retain only minimal connection to the ependymal cells.

In the first case, one could consider such a cell as a displaced ependymal cell, but in the latter case, it must be regarded as a cellular element of the subependymal neuroglia. For the doctrine of the formation of true, star-shaped glial cells from the ependyma, one can also consider their late post-embryonic appearance at the ependyma. This includes the growth events in the human ependyma, which are often found in the central canal of the spinal cord and can be attributed to a regular age-related process. This process results in a dense crest of fiber-rich glial cells with an angular appearance, separating from the area of the ependyma.

In the lower spinal cord of a person executed in their early 20s, I found multiple fiber-rich glial cells beneath the still intact epithelial layer of the lateral wall, while there was a proliferation of cells at the dorsal and ventral edges of the longitudinal cleft. This proliferation resulted in the disintegration of the ependymal cell layer. Consequently, the ependymal neuroglia is replaced in these areas by cells with a subependymal characteristic. These subependymal cells are composed of irregular cell forms, connected by short and inclined anastomoses, and they have formed a mass of typical glial fibers.

With respect to the common forms of subependymal glial cells, I observe in cross-sections of the spinal cord in humans and some animals two types: those with radial glial fibers and others where the fibers are cross-sectioned or slanted relative to the cellular orientation. The latter elements are found at the bottom of the fourth ventricle. It is possible to follow the fibers of the radial-fibered glial cells, which can be considered astrocytes. These are the cells close to those that, in spinal cord cross-sections, run parallel to the inner ependymal surface and follow a wave-like form along the central canal or in the direction of the fourth ventricle.

Another group is attached to the fibers or fiber bundles that originate from the ependymal cells and insert, along with these cells, at the surface of the frontal cleft. A third group ends at the perivascular border of the blood vessels, which penetrate the central glial substance and extend further into the Substantia Grisea centralis. These fibers extend in a radial and straight orientation to reach a blood vessel, ending perpendicularly to the surface of the vessel or running alongside it. Predominantly, the neuroglial fibers of that group form the vertical fiber zone.

It is often observed that they originate from transversely oriented subependymal glial fibers. The gain on single cross-sections is not substantial, and I therefore conclude that these fibers are so long that they extend beyond the thickness of my spinal cord sections, making it impossible to draw a definitive conclusion about their full extension.

Considering the relationship of all these glial fibers to the protoplasm of the subependymal glial cells, one can conclude, based on appropriate thin paraffin sections, that:

1. The radial fibers are rooted in the protoplasm of astrocytes and sometimes lie close to the surface (or even within the cell membrane), or they penetrate the protoplasm near the nucleus, accompanied over a certain distance by a thin protoplasmic sheath.
2. A large portion of the inclined or transversely cut glial fibers is embedded in a protoplasmic network, which, in my view, is composed of branches of glial cells or peripheral processes of ependymal cells. These contain, similar to the protoplasm of the cells, intensely stained protoplasmic granules.

In my preparations, a portion of the glial fibers appears to be entirely free, or at least free over some distance. By "free," I mean that they extend beyond the area of the net beams and are unbound within the mesh spaces of the glial cells. I have also observed that, in their further course, these fibers can reenter the granulated mass. Therefore, I can only attribute a partial isolation of these glial fibers from the protoplasm of the glial cells and their net-like branches.

Referring to the vertical glial fibers of the rabbit, I found them mostly enclosed in the net-formed glial substance (Fig. 2), with only a small portion appearing to be completely free. In humans, I could not make extensive observations regarding whether such a net-like structure of glial cells is developed, as I could not obtain fresh spinal cord material to stain and fix appropriately. However, in the lumbar spinal cord of a 21-year-old executed individual, I observed the existence of a network enclosing glial fibers.

Whether this network is similar to that observed in rabbits, dogs, calves, or cattle, I cannot determine, as the fibers in the preparations are too compressed. Nevertheless, I can state with certainty that many glial fibers here are associated with a thin, lightly granulated, and partially angled substance that appears to serve as a cover for these fiber elements.

b) Diffuse neuroglia

In adult humans, calves, and cattle, I have studied the elements of diffuse neuroglia primarily in the spinal cord. Additionally, I have made some observations in the white and gray matter of the human cortex and cerebellum. In rabbits and dogs, I have also extensively studied the brainstem.

Apart from the defined arrangement of the neuroglia with respect to the nervous elements of white and gray matter, there is no fundamental difference between subependymal and diffuse glia. A variable mass of glial fibers, their differing relationships to the protoplasm of glial cells, and the unequal abundance of protoplasmic granules are not significantly distinct, as these features can vary across different regions of the diffuse or subependymal glia.

The large variation in the form of the nuclei, with respect to size (small, medium-sized, and large nuclei), their curvature or general appearance, and the content and distribution of chromatin (granular and homogeneous), as emphasized by Aguerre[26], is not different for these zones of glia. An exceptional position is occupied only by glial cells in close proximity to nerve cells in the gray matter, as they have unilateral relationships with the peripheral support nets of the nerve cells, the Golgi nets, as discussed later. Additionally, the form and branching of the glial cell protoplasm, as well as the shape of the clearly intrinsic mass of glial fibers, is highly variable. The following classification provides a framework. I first distinguish protoplasm-large and protoplasm-small forms. In the latter case, these can be so extreme that the protoplasm-smallest forms, upon superficial inspection of poorly stained slices, may be mistaken for protoplasm-free nuclei. This explains why earlier studies of neuroglia often described “free nuclei” embedded in a basic substance. These nuclei are often homogeneous but may also contain granular chromatin, making it difficult to provide a definitive judgment. Many potential artifacts arise due to fixation techniques (e.g., Müller solution results in more homogeneous nuclei compared to Zenker’s solution) or the type of staining used. The nuclei can also vary greatly in size, with some being very small, while others are larger and bubble-like in appearance. However, the plasma mass is always arranged as a thin hull that continues into very fine, slightly granulated processes around the entire cell (Fig. 8b).

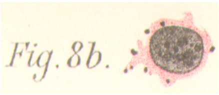

Fig. 8b from Table I. Cell with very small protoplasm, cross-fibered

Contractions caused by Müller solution, formalin, or 96% alcohol followed by paraffin embedding can easily disrupt these thin and fine processes. There are many transitional forms between the smallest or small glial cells and those with a larger, more substantial mass of protoplasm forming the cell body (Fig. 6, Fig. 5b). Two main forms can be easily and reliably distinguished: 1. Skin-like forms (Fig. 4a, b) and 2. Protoplasm-dense or protoplasm-granulated cells (Fig. 5 and Fig. 9).

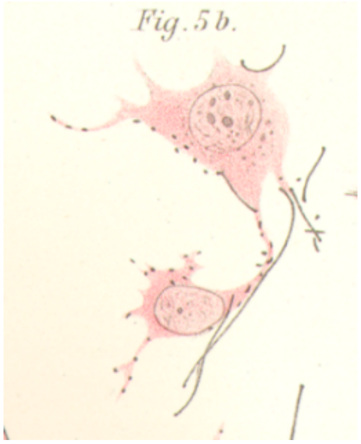

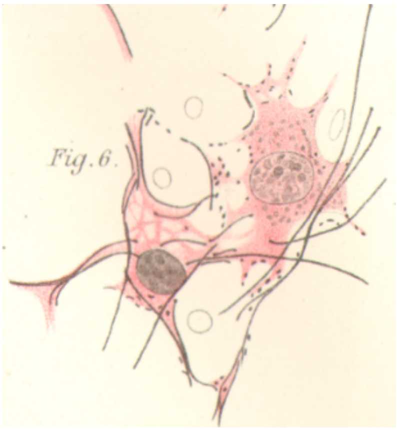

Fig. 5b from Table I. The upper glial cell is a form with few fibers and a large and granulated cell body.

Fig. 6 from Table I. Two connected cells; the lower one is partially spongioplastic and radial fibered, the upper one with a large protoplasm, granulated and cross-fibered.

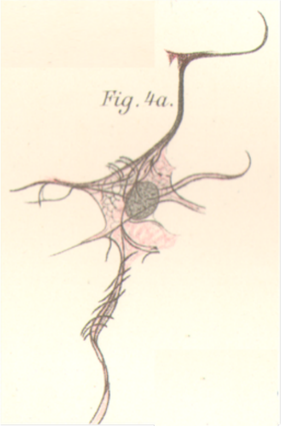

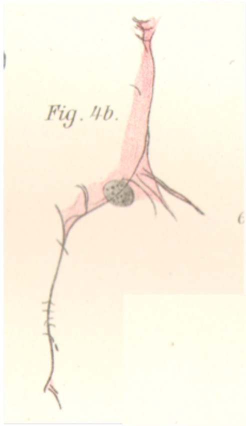

Fig. 4a and b from Table I. Glial cells at a cross section of the rabbit spinal cord (white matter of the ventral columns). Fig. 4a a spongioblastic, Fig. 4b a film-type glial cell. Hartnack 1/12, Oc. 6.

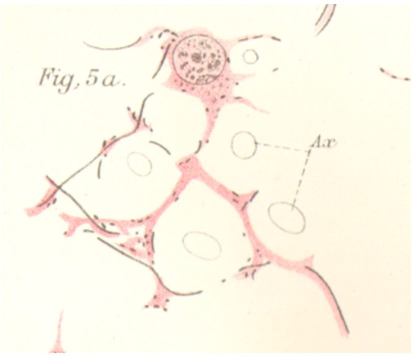

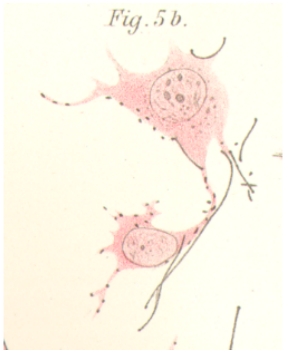

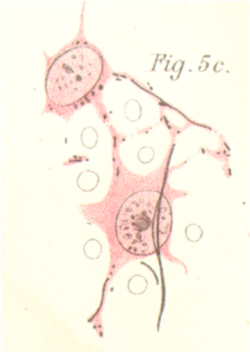

Figs. 5a-c from Table I. From a 24-year-old executed). Figs. 5a-c, 6, 10 from white matter. Potassium bichromium, iron alaun fixation. Hartnack 1/12 Oc. 6. Fig. 5a. cross-fibered glial cell; on the following slices the process cut below is connected to a glial cell. Fig. 5b. The upper glial cells is a form with few fibers and a large and granulated cell body. Fig. 5c. Two glial cells with glial fibers mainly in the processes. Fig. 5d. From a longitudinal section of bovine white matter.

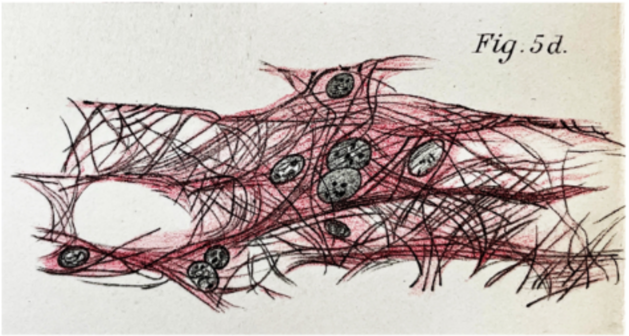

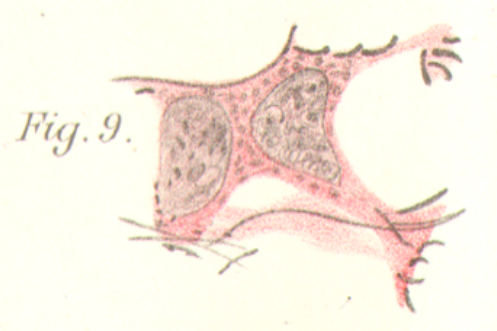

Fig. 5d. From bovine; a broadly anastomized syncytium of glial cells. Its protoplasm enwraps several nerve fiber spaces in an arial fashion and contains several glial fibers.
Fig. 9. Glial cell with two nuclei which is strongly granulated.

Intermediate stages exist in which the protoplasm can be partially granular and densely constructed. The latter forms result in light and clear staining of the cell structure. Skin-like glial cells are characterized by being almost completely devoid of granular protoplasm, although single granules may be present. Otherwise, the cell body appears to consist mainly of a metamorphosed and thickened cell membrane, which connects the glial fibers to the cell via a thin skin or cover (Fig. 4b).

In other cases, there remains a peculiar spongy-plasmatic structure within this cover, arranged in the form of a fine or coarse net of beams. This can be considered a remnant of modified protoplasm generated by coarse vacuoles. It is organized in relation to the glial fibers within the cell, guiding them through the cell along its walls and beams. In certain differentiations, the internal beam system appears dark red, while the cover is pale, and the glial fibers are stained dark black (Fig. 6 and 7). With low bleaching, the network appears grayish and less distinct from the glial fibers, as I observed in a rabbit cell (Fig. 4a). Such a classification of glial cells based on their protoplasmic constitution or the outer form of the cell body may be relevant, but they all share similarities in the form and origin of their protoplasmic processes, which can vary in thickness but are responsible for generating the star-shaped forms. The cell body may be round, as in the smallest forms, oval-shaped, elongated, or flattened. In the white matter, it may also exhibit a concave shape, similar to the enwrapped nerve fibers.

Considering only the arrangement of the glial fibers in relation to the branched glial cells, or simply the arrangement of labeled glial fibers with respect to the nucleus of the glial cells, it appears that the astrocyte form, as proposed by Weigert, refers exclusively to those glial cells that exhibit a radial arrangement of fibers in relation to the cell nucleus.

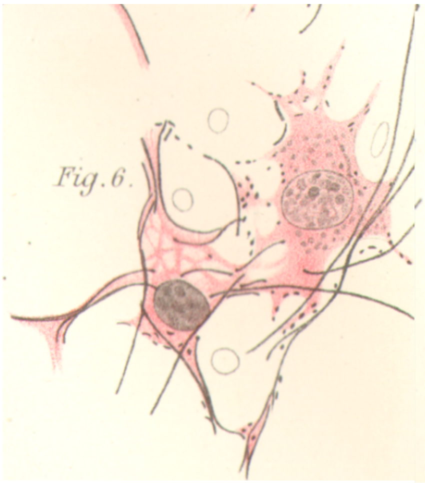

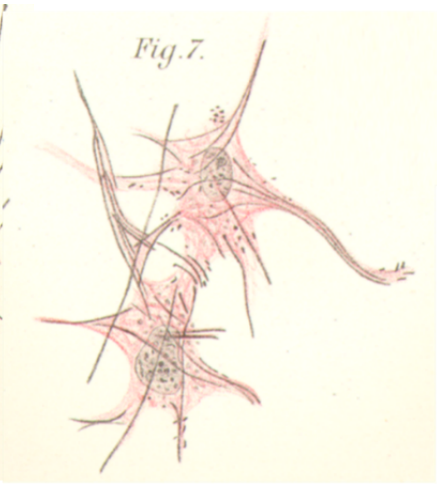

Fig. 6 from Table I. Two connected cells; the lower one is partially spongioplastic and radial fibered, the upper one with a large protoplasm, granulated and cross-fibered.
Fig. 7 from Table I. Two connected radial-fibered glial cells; both spongioplastic

Here, it is presumed that such a shape of the fibers applies to all locations and is visible in all images of the cell. My illustrations (Fig. 4–10, Table I) demonstrate that the arrangement of glial fibers can vary depending on how the branched cell is cut (e.g., in longitudinal or cross-sections of the spinal cord white matter). I will address this further in the following sections, as it allows for a secondary classification of glial cells based on the assessment of glial fibers with respect to the direction of the cell branches. This classification is justified due to the abundance of specific glial fibers in the adult central nervous system.

Considering the position of glial fibers in relation to the protoplasm, which can best be observed in cross-sections of glial fibers, one can state the following: 1) Free glial fibers exist, which are only attached to or cross over cells or their processes. Their number is difficult to estimate, as inclined or longitudinally cut fibers are challenging to quantify. However, there are several fibers running in wave-like forms that allow for the preparation of optical cross-sections. These cross-sections reveal that the fibers neither penetrate the cell body nor the protoplasmic process; that is, they are never enwrapped by protoplasm but remain at a minimal distance from it. I have observed many such fibers.

Considering that my slices are 5–10 microns thick, and given the lack of information about the actual length of these fibers, the conclusion regarding the existence of completely free, cell-independent fibers is not very substantial. Furthermore, the following observation adds to this understanding.

2) I find glial fibers embedded for some distance within a protoplasmic process of a glial cell or within a skin-like mass that is stained red and slightly granulated in my preparations. These fibers are sometimes connected to each other in regions where glial fibers diverge into slight or strong bundles (Fig. 2, 4b, 6, 7, 8a, 11, 12 of Table I). 3) Finally, I can identify relationships where glial fibers are clearly located within the cell body. In these cases, they directly contact the nuclear membrane as cross-sectioned fibers or are situated in the protoplasm of the glial cell, or even embedded in the cell membrane. In certain skin-like glial cells, which contain a granulated remnant of protoplasm and occasionally exhibit the previously described spongy-plasmatic network, the fibers are silhouetted as a dark-labeled border line. These glial fibers, running directly along the surface of the cell rather than merely being attached, can be interpreted as a structural stiffening of the membrane. 4) Lastly, I observe glial cells with extraordinarily coarse, conically originating processes that also contain dense protoplasm in both the cell body and processes. These cells are further characterized by outgoing bundles of strong glial fibers that follow the cell surface. These glial fiber bundles are adhered together by red-stained protoplasm and extend to structures such as blood vessels or the *Membrana limitans Gliae superficialis*.
When studying cross-sections of these strong processes, the individual fibers are arranged in a circular fashion, united in the middle of the red-stained protoplasm. The entire process is densely filled with fibers, and in the narrow interspaces between them, the red color of the protoplasm is visible. At the end of such a process, the protoplasm terminates in a similarly labeled glial endfoot, which I will describe later in the context of the peculiarities of marginal glia.
Based on all these results I cannot agree with Weigert, that the glial fibres are a structure completely distinct from the glial cell protoplasm and should be considered as a purely intercellular substance. I should add that in my preparations, in contrast to the pure fibre labelled preparations, one can recognize both issues distinctly stained and one can conclude

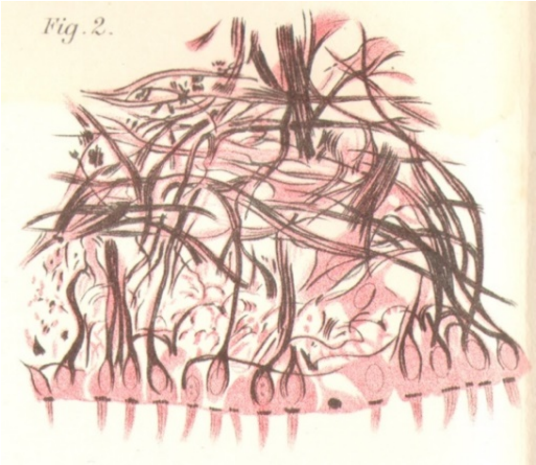

Fig. 2 from Table I. From the same slice, ventral part of the ependyma. Hartnack homogeneous immersion 1/12. Oc.6

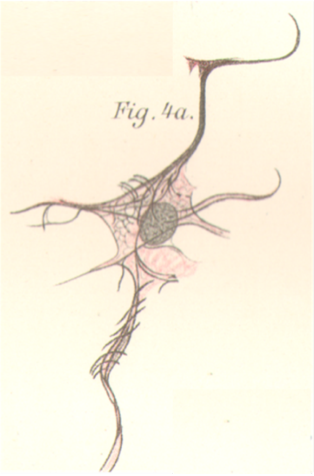

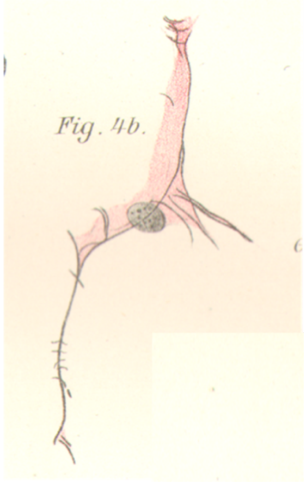

Fig. 4a and b from Table I. Glial cells at a cross section of the rabbit spinal cord (white matter of the ventral columns).Fig. 4a a spongioblastic, Fig. 4b a film-type glial cell. Hartnack 1/12, Oc. 6.

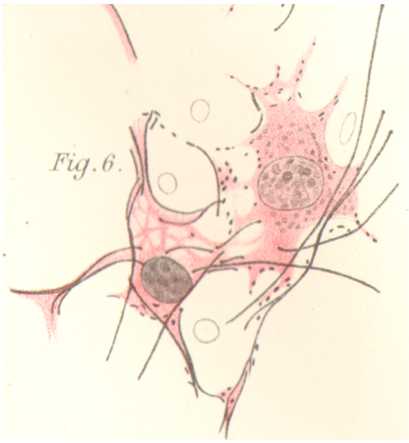

Fig. 6 from Table I. Two connected cells; the lower one is partially spongioplastic and radial fibered, the upper one with a large protoplasm, granulated and cross-fibered.

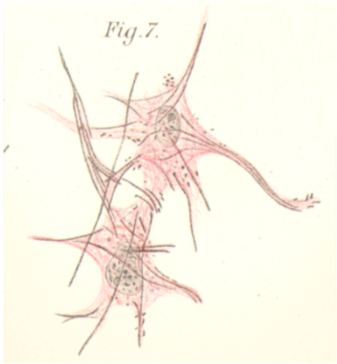

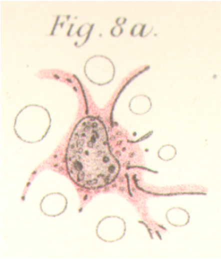

Fig. 7 from Table I. Two connected radial-fibered glial cells; both spongioplastic.

Fig. 8a from Table I. Glial cell with small protoplasm, radial fibered.

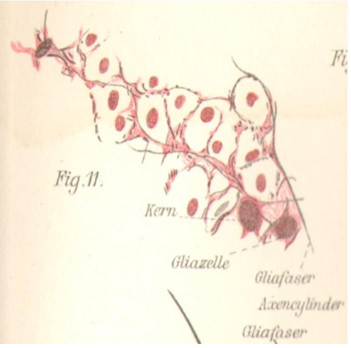

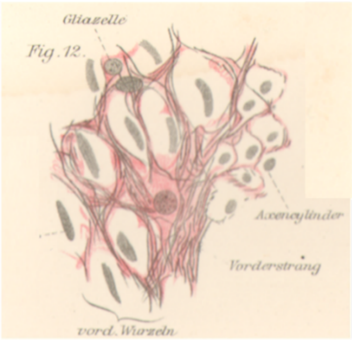

Fig. 12 from Table I. From the same slice; Penetration area of the ventral roots at the ventral horn.

Fig. 11 from Table I. Cross-section image from the white matter of the human lower spinal cord (area of the dorsal horn). 24-year-old executed. Fixation in potassium bichromium. Differential hematoxylin staining according to M. Heidenhain. Hartnack 1/12 Oc. 6. (No net between the nerve fibers).

Some of the glial fibers are located in regions free of glial cell protoplasm and its processes, but one cannot conclude that they maintain the same relationship in other regions, as a clear intracellular position can also be recognized. I will revisit this topic later when I discuss the formation of neuroglia and the development of glia.

After this general description of the relationship between glial fibers and protoplasm, I propose a second classification based on the variable fiber content of glial cells. Referring to the figures, I distinguish between: 1) Fiber-rich glial cells, 2) Fiber-sparse glial cells, and 3) Fiber-devoid glial cells. The latter group consists of cells termed protoplasmic neuroglial cells by Golgi, Ranvier, and others. Whether cells with crossing glial fibers attached to their surface (Fig. 10) also belong to this group remains questionable (cf. above). Among the marginal glia of the cortex, I have found these cells more frequently compared to the white matter of the spinal cord. I cannot provide specific information regarding their number.

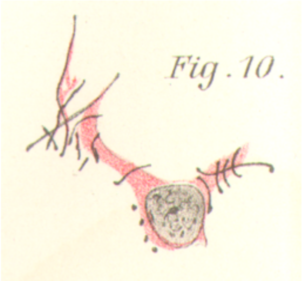

Fig. 10 from Table I. Glial cell with small protoplasm with thick processes, which are crossed-over by glial fibers.

For the second class, the fiber-sparse glial cells, there is also a potential for error in this classification. This arises because it is challenging to determine the full extent of the branches of these glial cells within the slices. Furthermore, it is difficult to ascertain the boundaries of these cells and distinguish them from their neighboring ones, making it hard to decide to which cells the individual fibers belong. This is particularly evident in the figures from the white matter of the spinal cord, the ependyma of the central canal, and the marginal glia of the cortex.

The complex relationship between glial fibers and several glial cells is closely tied to the question of the formation of neuroglial tissue. It should be emphasized that this classification refers only to the immediate relationship of glial fibers to the cell body and the initial portion of their outgoing processes.

and that secondly, the apparent distinction between fiber-rich and fiber-sparse glial cells may be restricted by branching zones and the incoming fiber parts. For distinguishing between the second and third classes, a more precise method of investigation may no longer provide sufficient clarity. Additionally, since the production of glial fibers in humans increases with age, the distinction between fiber-sparse, fiber-devoid, and fiber-rich glial cells may shift over time. Fiber-sparse glial cells are illustrated in Fig. 5d, 8, 8b, and 9.

Two types of fiber-sparse glial cells can be distinguished: 1)Those with fibers crossing the processes transversely. 2)Those with fibers following the processes longitudinally or in a slight curve. Most ependymal cells belong to the class of fiber-sparse glial cells, as long as they do not produce unilaterally oriented bundles of glial fibers, as previously discussed. Among the fiber-rich glial cells, I distinguish three types: 1) Radial-fibered cells, 2) Radial-bundled cells, and 3) Cross- or longitudinal-fibered cells.

In radial-fibered glial cells, the fibers follow the course of the corresponding protoplasmic processes, resulting in a radial orientation relative to the nucleus. This arrangement is similar to that of fiber-containing ependymal cells, in which the main process contains unilateral fibers or fiber bundles. Additionally, in radial-fibered true glial cells, the long fibers that penetrate the cell body do not extend into a second process but terminate after following a more or less arched course. As a result, in some preparations of glial cells, fibers can be observed as short, bowed sections within the depth of the slice, or as pure cross-sectional points. In addition to these, long fibers can be seen extending from process to process and passing near the nucleus (Fig. 6, 4a, 7). Based on these figures, one can conclude that this type of glial cell is not only radially fibered in the visible section but is radially fibered throughout the entire cell.

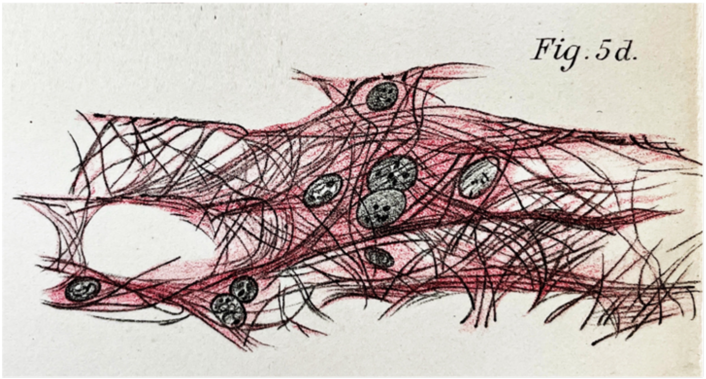

Fig. 5d from Table I. From bovine; a broadly anastomized syncytium of glial cells. Its protoplasm enwraps several nerve fiber spaces in an arial fashion and contains several glial fibers.

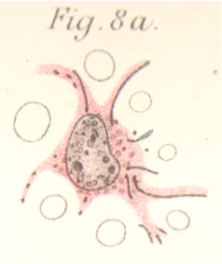

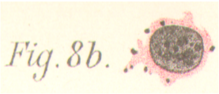

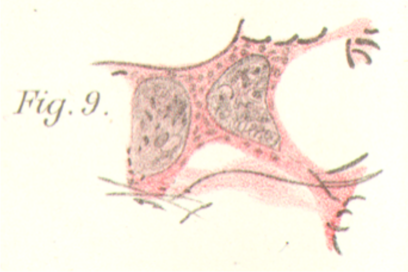

Fig. 8a from Table I. Glial cell with small protoplasm, radial fibered.
Fig. 8b from Table I. Cell with very small protoplasm, cross-fibered
Fig. 9 from Table I. Glial cell with two nuclei which is strongly granulated.

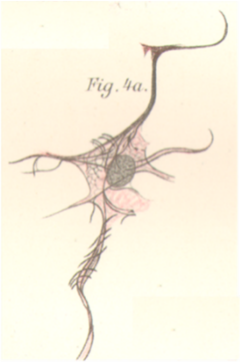

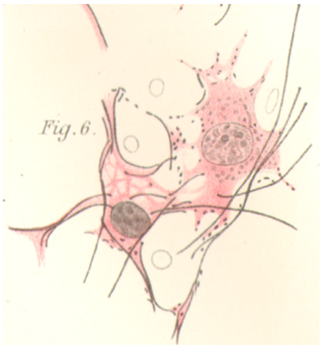

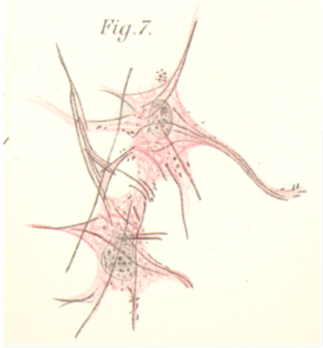

Fig. 4a from Table I Glial cells at a cross section of the rabbit spinal cord (white matter of the ventral columns). Fig. 4a a spongioblastic, Hartnack 1/12, Oc. 6.
Fig. 6 from Table I. Two connected cells; the lower one is partially spongioplastic and radial fibered, the upper one with a large protoplasm, granulated and cross-fibered.
Fig. 7 from Table I. Two connected radial-fibered glial cells; both spongioplastic.

These glial cells must be described as all-round radial-fibered. They are distinct from those where only purely cross or inclined-cut fibers can be observed, along with a few longer visible fibers. One can conclude that the radial fibers in these cells are developed only in one or two planes. I cannot accept the argument that this is due to overly thin sections, as increasing the thickness from 5 to 10 micrometers did not reveal additional bowed sections. This form must therefore be distinguished as unilateral radial-fibered glial cells (Fig. 6).

The radial-fibered glial cells are distinct from the radial-bundled ones, as the former possess single fibers instead of bundles of fibers (Fig. 7a). The individual fibers within the bundles are not necessarily thicker than those of the first cell type described above. I observed radial-bundled glial cells quite abundantly in the lumbar spinal cord of a 21-year-old executed individual, particularly in the Substantia Neurogliae centralis. Their bundles extended partially to blood vessels, partially into the cross-sectioned internal zone, and partially as fiber tracts extending to the Fissura Interior.

In this individual, and additionally in an older human, I identified a variant that represents an intermediate form between radial-fibered and radial-bundled glial cells, characterized by one or two fiber-bundle-rich processes. Among the superficial glial cells of the white matter, these were predominantly oriented towards the *Membrana limitans superficialis*, where they inserted with a thick glial endfoot.

The most peculiar relationship between the cellular protoplasm and the glial fibers is observed in the cross-fibered glial cells (Fig. 8b, 5). Their radially extending and thin processes primarily contain cross or inclined-cut glial fibers. It can be assumed with certainty that these fibers are indeed located within the protoplasm, more clearly than the long or inclined fibers. Additionally, they can be clearly distinguished from fibers that are merely attached.

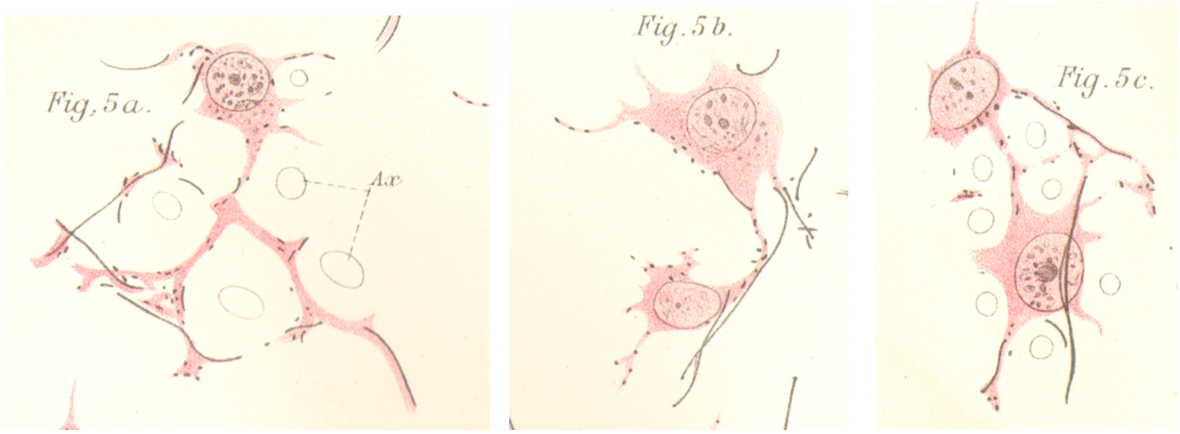

Figs. 5a-c, from a 24-year old executed). Figs. 5a-c, 6, 10 from white matter. Potassium bichromium, iron alaun fixation. Hartnack 1/12 Oc. 6. Fig. 5d. From a longitudinal section of bovine white matter
Fig. 5a from Table I. cross-fibered glial cell; on the following slices the process cut below is connected to a glial cell.

Fig. 5b from Table I. The upper glial cells is a form with few fibers and a large and granulated cell body.
Fig. 5c from Table I. Two glial cells with glial fibers mainly in the processes

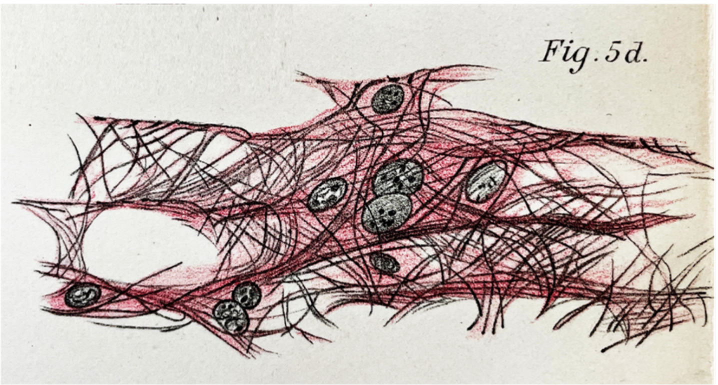

Fig. 5d from Table I. From bovine; a broadly anastomized syncytium of glial cells. Its protoplasm enwraps several nerve fiber spaces in an arial fashion and contains several glial fibers.

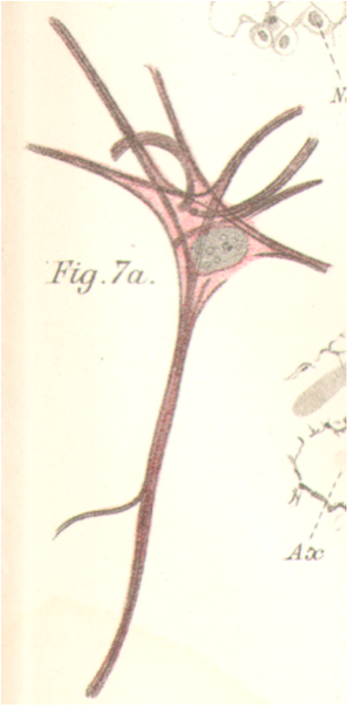

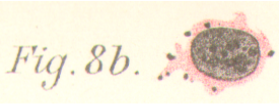

Fig. 7a. Radial-bundled glial cell from the substantia neurogliae centralis.
Fig. 8b. Cell with very small protoplasm, cross-fibered.

It is obviously another question how these cells would look like in the opposite view. This can obviously and unfortunately not precisely be decided. I consider a small fraction of them as cut images of radial fibred glial cells, based on a comparison of longitudinal and cross sections which I studied in the dorsal root of the spinal cord.

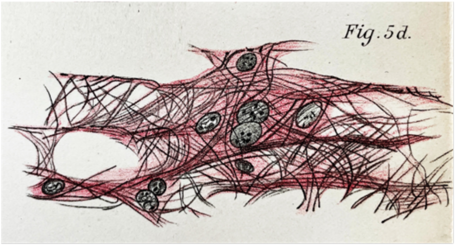

Fig. 5d from Table I. From bovine; a broadly anastomized syncytium of glial cells. Its protoplasm enwraps several nerve fiber spaces in an arial fashion and contains several glial fibers.

I support the view of Golgi and Weigert, who state that in vertical sections of spinal cord white matter, astrocytes appear more numerous. Additionally, I consider that they do not originate from those glial cells but instead from long, flat cell forms oriented along the direction of the nerve fibers. These fibers are attached to the cells in a curved form and produce a large number of longitudinally and cross-oriented glial fibers. Consequently, cross-fibered glial cells may, in the opposing view, be classified as longitudinal-fibered glial cells.

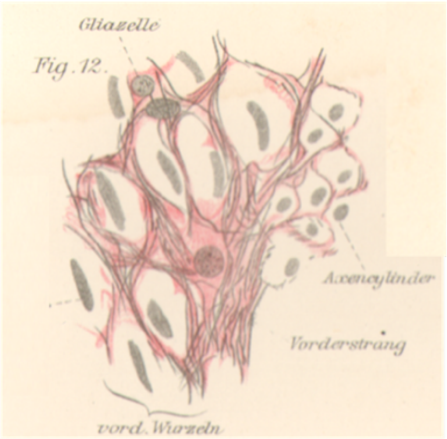

Fig. 12 from Table I. Cross-section image from the white matter of the human lower spinal cord. Penetration area of the ventral roots at the ventral horn.

These cells can also be observed in cross or inclined sections of the spinal cord, such as at the ventral root, provided they are sectioned along the longitudinal course of the white matter (Fig. 12). They are often multinucleated, forming giant cells (cf. Fig. 5d from calf). Remarkably, their glial fibers are partially arranged in a cross orientation, which can be observed in cross sections due to the fibers' opposite movement when viewed in different focal planes.

The entangled arrangement of glial fibers, as well as the bending of fibers in radial-fibered glial cells within the spinal cord tracts, makes it impossible to trace the glial fibers in cross sections. It cannot be determined whether fibers that appear free from glial protoplasm are independent entities or whether they are cut-off fibers originating from another glial cell or process.

Furthermore, based on observations of longitudinal sections of the white matter in the spinal cord, cerebellum, and cortex, I emphasize that there is often a distinct net-like mass between the different glial fibers. This net-like structure embeds the fibers and appears to be produced or delivered in some way by the protoplasm of the glial cells.

Thus, I cannot accept the notion that glial fibers are completely emancipated from glial cells (see also the following section and Fig. 12, 14, 15, 16, 17).

Based on all the information regarding the protoplasmic form and ramification of glial cells, as well as the intracellular location of a large number of glial fibers, it is clear that in preparations with pure fiber staining, the true relationship between fibers and glial cell protoplasm remains obscured. On the other hand, it is evident that certain general properties of glial fibers—whether radial or cross-fibered—still become apparent in co-stained preparations. This is more noticeable in the radial group, where the glial nucleus is more visible, while in the cross-fibered group, it tends to remain hidden. This is particularly true in cells with large somas, where fibers are located at the surface, whether the cell represents an apparent or true cross-fibered glial cell. In this context, I refer to statements by Weigert, who claimed that glial fibers are a free and cell-independent structure ([6], pages 95–97): “Characteristic images are only generated when, which is often the case, the fibers are arranged in bundles around the nucleus, generating a spider-, brush-, or star-shaped figure with the nucleus in the center, including the invisible protoplasm. Transitions to the invisible protoplasm cannot be noticed. These transitions should become noticeable since the fibers approaching the nucleus should become paler, and close to the nucleus, they should disappear. This never occurs.” Weigert further elaborates: “In these nuclear centers with the beam-like attached fibers, the spider cells of Deiters or neuroglial cells can be well recognized, while in other cases, where the nuclei are embedded in a tangle of fibers, such an arrangement does either not exist or is obscured by the cutting direction (disguised astrocyte images). Sometimes it is possible to get such 'astrocyte images' by staining the neuroglial cells with neutral carmine red—a double staining which is not favorable for the delicate fibers.” This procedure is only helpful when the space between nucleus and fiber is too big

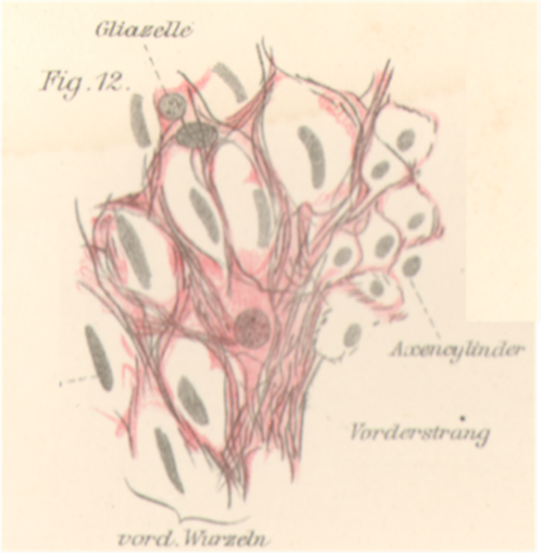

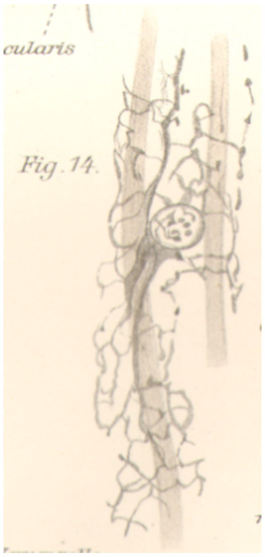

Fig. 12 from Table I. From the same slice (Cross-section image from the white matter of the human lower spinal cord (area of the dorsal horn). 24-year-old executed); Penetration area of the ventral roots at the ventral horn.

Fig. 14 from Table I. Net-shaped branched glial cell from the white matter of the human cerebral cortex (24-year-old executed). Alcohol-chloroform, acidic acid mix. Hartnack 1/12 Oc. 8.

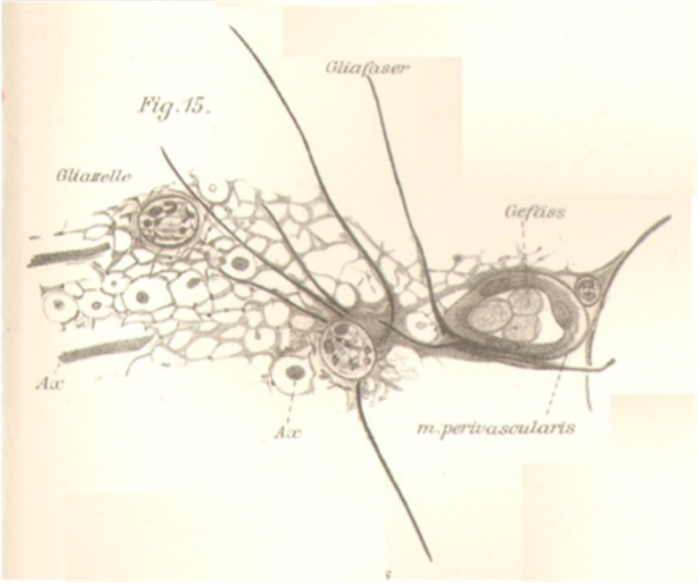

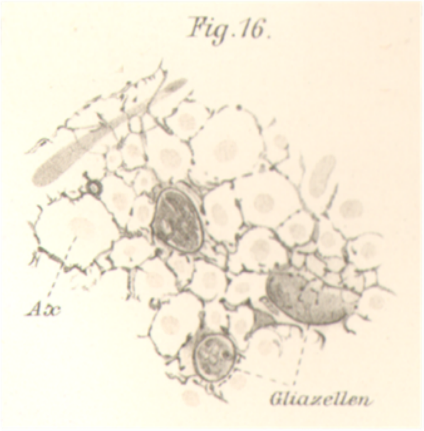

Fig. 15 from Table I. From the same slice.
Fig. 16 from Table I. From the white matter of the rabbit cerebellum. Molybdenum method according to Bethe.

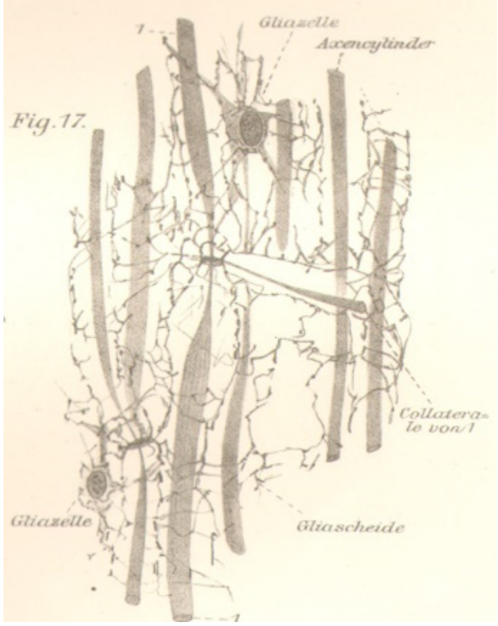

Fig. 17 from Table I. From the same slice which shows the net-shaped glial sheath and two glial nodes of Ranvier in a longitudinal section. Hartnack 1/12 Oc. 8.

to reveal the relationship among the two, which means that the cell body, which remains invisible without double staining, is too extensive. Nevertheless, one can state that many nuclei among the fibers (particularly the small, dark-labeled ones) cannot, in any way, be recognized as centers of beam-like systems." Weigert then arrives at the following conclusion:

"Despite the fact that so many nuclei have no relationship to the fibers and that the soft fibers seem unrelated to the nuclei, one must conclude that all the fibers stained with our method are identical to those formations which, since Frommann**(s)**, have been considered processes of glial cells."

When comparing Weigert's report with my findings, it is first evident that I agree with Weigert regarding the longitudinal or cross-fibered glial cells. However, I must also point out that Weigert overlooked the deep or superficial intracellular location of the glial fibers in question in his double staining.

Furthermore, it becomes clear how Weigert concluded that neuroglial fibers could be identified as former glial processes while simultaneously claiming that they are not processes of glial cells. Since Weigert did not observe "transitions of fibers" in radial-fibered glial cells—only passing fibers that neither began nor ended at the nucleus—he concluded that glial fibers are not cellular processes. It is evident that the radial-fibered glial cells served as the main foundation for Weigert's argumentation.

This conclusion is biased, as Weigert did not consider the possibility that these fibers—so long as they are not augmented by attached fibers from other cells—could be intracellularly located, within the cell body, or within the protoplasm covered by a cell membrane, as my observations demonstrate. Weigert's arguments are as follows (page 101): "It could still be possible that Ranvier's method and our own method produce separate filaments instead of extensions, but this would amount to some kind of artificial product." Since Ranvier's method was still unreliable and in the case of the cerebrum left its inventor and other outstanding researchers, like Golgi,

stranded, the assumption of folds was certainly an obvious one, and one cannot blame the researchers for holding on to their old established views despite Ranvier's publications. Although it is undeniable that, in terms of the question we are addressing here, our method may otherwise leave a lot to be desired, but in this case, it is reliable, and at this point we have to assert that the images obtained using the old methods (but not those from Ranvier) were in fact mirages. Images obtained with the old methods, as well as the Golgi method, simply cannot distinguish fibres from cell bodies because both structures refract light in the same way (Ranvier) or stain to the same extent, so that the two structures appear to be one with regard to their chemical (and morphological) properties. Our preparations however prove that this is in fact an illusion, because we are able to demonstrate that the chemical composition of fibres and cell bodies is quite distinct. This, of course, is the crux of the whole question."

I have to remark, that this does not sound right to me, since in Weigert´s view on neuroglia there are two main issues in his strange doctrine, first that the neuroglial fibres are chemically distinct from the protoplasm of the glial cells and second that they form a true intercellular substance. The first I will not dispute with Weigert, and as the above cited literature shows, it has not been disputed. To have established the special nature of the neuroglia fibres and having for the first time revealed their important topographic distribution, remains the big merit of Weigert.

With respect to the second main point, I have to state that it is wrong. Weigert claims in the sense of the definition of Max Schultze that the glial fibres, except for the modification of their substance, represent a complete emancipation from the cell body. "The fibres are only contiguous to the cell body, they are not connected with it, but the leading and trailing parts of a thread, if one may use this expression, are so intimately united with each other that they represent something coherent, a common fibril,

which runs smoothly over the adjacent cell.

In this regard, neuroglia finally rejoins the series of connective tissue components, but only in terms of their morphology" (l. c. S. 116[(n))]).

Against that second part of Weigert´s doctrine, I first counter that it is not substantiated by the methods used by Weigert. The Weigerts method cannot prove that the glial fibres are exclusively attached or run over the glial cells, in other words only touch the cell, since the protoplasm has not been stained. Thus, it cannot be decided whether the glial fibres are an intercellular substance. Secondly, I must note that the statement on the morphological nature of neuroglia is truly wrong. My data, described above, demonstrate that astrocytes are radial-fibred glial cells and their own glial fibres are embedded in the protoplasm or enwrapped by a cell membrane. Not only the cell body is enwrapped, but also the onset of the process. Weigert, with respect to the astrocyte definition, claims that their images are mirage, showing no distinction between glial fibres and glial cell protoplasm and the former being simply its processes. According to my opinion this reproach is to be amended to the effect that the glial fibres are intracellular within the protoplasm of the cell body and in the branching off processes. I must thus accuse Weigert, that he has overstepped the limits of his method by claiming that the glial fibres are an intercellular substance merely attached to cells. I repeat here Lenhossek´s earlier objection (Feiner Bau des Nervensystems 1895, page 187) against Weigerts method and doctrine that "it easily leads to an erroneous vision that the glial fibres are something autonomous, such as the fibres of the fibrillary connective tissue and that the glial cells are only secondary, independent formations embedded in the fibre crisscross". I emphasize this since Weigert has parried this blame by stating that it refers only to his methodical proof of the chemical distinction between fibre and cell protoplasm. That this does not provide a final proof, should be obvious.

The observations of Ranvier differ from those of Weigert regarding the second question: the relationship between cells and glial fibers. Ranvier states (5, page 180): *"Ses fibres ne paraissent plus être de simples prolongements des cellules, car on peut les suivre maintenant au sein des cellules elles-mêmes. Elles sont simplement noyées dans le protoplasma"* (*"Its fibers no longer appear to be simple extensions of the cells, because they can now be traced within the cells themselves. They are simply embedded in the protoplasm."*)

Further:

"Dans les préparations faites après l`action de liquide de Müller, on voit la masse de protoplasma envoyer sur les fibres, qui s'en dégagent des expansions qui, généralement, s'étendent entre elles comme une membrane interdigitale, d'autres fois les entourent d'une sorte de manchon"

(*"In the preparations made after the action of Müller's liquid, we see the mass of protoplasm send expansions onto the fibers, which emerge from it. These expansions generally extend between them like a sort of interdigital membrane; other times, they surround them like a kind of sleeve."*) I have no objections to Ranvier's statements, as I have observed the same phenomena. This demonstrates that glial fibers are, in part, intracellularly located and do not form an intercellular substance, as Weigert claims.

One can generally conclude in this dispute that Weigert's alternative—the claim that glial fibers are located in astrocytes at positions where processes are typically found, but that they are not processes because they are chemically distinct—is not correct. This issue is of broader significance.

It is well established, as shown in the reports of Ranvier and Andriezen, that purely protoplasmic glial cells exist in the cortex. I have also observed many purely protoplasmic processes in glial cells. According to Ranvier, embryonic glial cells possess only protoplasmic processes. This raises the question of how many of these originally protoplasmic processes become, or do not become, fiber-containing processes.

Additionally, it is essential to determine why glial fiber formation occurs so irregularly—sometimes extending within the cell body from one process to another, while in other cases deviating significantly from this pattern. One would also like to find out, as a last point, whether or not the fibers extending from the glial cell protoplasm become naked in their further course and how they are related to other parts of the central substance,

to the other glial cells, or to the blood vessels as foreign elements.

In the following chapter I will add observations to this general relationship between glial cells and glial fibers, which deal with the development of glial fibers and in addition demonstrate the formation of glial end feet. This will add further criticism to the Weigert-doctrine of the fibrous glial intercellular substance.

2. The development of the neuroglial fibers and their endings in the endfeet of glial cells

It is evident that the developmental history of fibrous neuroglia does not provide evidence that, in mature tissue, neuroglial fibers associated with neuroglial cells form an intercellular substance. Observations of their development, however, can address the question of the fate of the processes of embryonic glial cells—whether they later transform into glial fibers, as distinct in their composition, as already posed by Ranvier.

Ranvier made the following observation (*De la Névroglie*, page 182):

"Chez les embryons plus âgés (j'ai examiné des embryons de bœuf de 0,75 m et de 0,95 m), un grand nombre de cellules de la névroglie étaient étoilées et présentaient de longs prolongements; mais ces prolongements n'étaient pas encore des fibres véritables: ils avaient la constitution du protoplasma de la cellule et se fondaient avec lui."

(*"In older embryos (I examined beef embryos of 0.75 m and 0.95 m), many of the neuroglia cells were stellate and had long processes. But these extensions were not yet true fibers: they had the constitution of the protoplasm of the cell and fused with it."*)

I can confirm this statement by Ranvier based on my observations in embryos of mice, guinea pigs, rabbits, cats, dogs, and humans. Furthermore, I found that, at later stages, where labeled glial fibers are already present, the cells of embryonic neuroglia are all more or less star-shaped and possess several protoplasmic processes. However, these processes are not as numerous as those observed later in the adult nervous system.

With respect to the temporal characteristics and the differences across regions such as the spinal cord, cerebellum, and cortex, there are considerable variations in the generation of fibrous glia. These differences also reflect the general development of the central nervous system. Fibrous glia first arises in the spinal cord, initially in the white matter and later in the grey matter. I will not elaborate on detailed differences concerning superficial glia, as I will later discuss the developmental stages of human neuroglia in a broader context. Here, I will focus on the main points addressing the general issue of the genesis of glial fibers.

In Fig. 28a, b, and c, I have illustrated the development of neuroglia in the optic nerve of the mouse. Among these, Fig. 28a (from an embryo measuring 1.2 cm in length) depicts a purely cellular glia composed of single, branched glial cells connected by their protoplasmic processes, which are clearly visible. Within the optic nerve, these protoplasmic processes anastomose, forming a reticular tissue. Between these structures are bundles of the optic nerve (fibers), which at the surface fuse to form a delicate border membrane. This membrane isolates the optic nerve with its cellular neuroglia from connective tissue and blood vessels.

At the border membrane, superficial glial processes terminate with conically enlarged protoplasmic endfeet. These embryonic cells can be identified as glial cells because they cannot be mistaken for nerve cells, as it is well established that there are no ganglion cells in the optic nerve. This structure represents a type of purely white matter. Furthermore, it is evident that the star-shaped, proper glial cells originate from the mass of His-type spongioblasts, which I will address in a subsequent chapter.

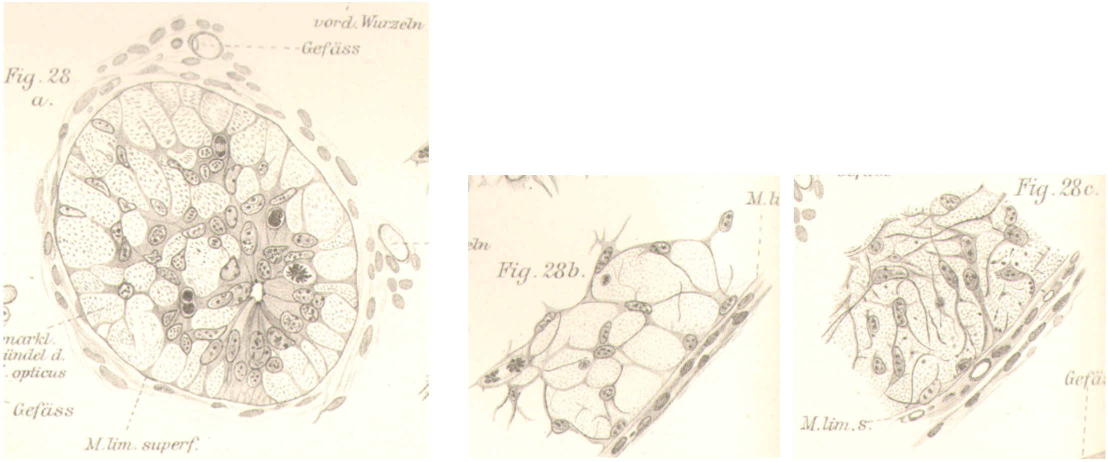

Figs. 28a-c from Table II. Three developmental stages of the Nervus opticus of the mouse in cross-section. Fixation in chromium-formalin , acidic acid. Also hematoxylin staining. Hartnack 1/12 Oc. 4.
Fig. 28a from a 12-day old embryo, Fig. 28b from a new-born, Fig. 28c from a 5-day old mouse.

It is evident, in relation to Weigert's perception, that the formation of glial fibers occurs within these cells.

As shown in Fig. 28b, obtained from a newborn mouse, the delicate protoplasmic processes of an embryonic glial cell projecting to another cell or to the border membrane are filled and stiffened with intracellular fibers. Within the protoplasm of these processes, fibrous glia develops as a peculiar thread-like product of the respective process. In my preparations, the delicate intraprotoplasmic glial fiber appears dark black, the surrounding protoplasm appears dark gray, while the nerve bundles of the *Nervus opticus* appear light gray.

I also compared this stage with a slightly earlier one—a late-stage mouse embryo—which shows, as a distinction, only a few glial fibers within those processes, and these fibers are not yet visible in the cell body. At this earlier stage, the substance of the newly forming glial fibril is not yet firm or homogeneous but rather granular, appearing as a dull line within the protoplasm.

I would like to add that the formation of fibrous glia is not restricted to the optic nerve; I have observed the same process in other regions, such as the spinal cord. Quite often, though not always, the first fiber-containing protoplasmic processes of the star-shaped glial cells are those extending to the superficial or perivascular border membrane in a radial fashion.

The subsequent changes in cellular neuroglia, due to its internal glial fibrillation, involve an increase in the number of processes and the growth of the glial fibers. Fig. 28c from a section of the opticus of a 5-day old mouse illustrates that the glial cells have developed more processes. The generation of the new processes is no longer purely crosswise, but into all directions. Therefore, at this stage, the total mass of glial cells appears cloudy and more complex as compared to the previous stage, which is more oriented transverse to the fiber tract. At this stage I can no longer decide

whether the later protoplasmic processes are connected with other glial cells or remain independent. Single but distinct anastomoses still occur, as shown in Fig. 28b. It is impossible to determine whether these are the original connections, perhaps slightly extended and stretched.

In this stage, I also studied the *Nervus opticus* of an 18-day-old mouse, which shows a significant increase in glial cells at fiber-containing processes.

Regarding the main question of the further development of intracellular glial fibers located within the processes, my preparations reveal that the fibers extend into the cell body as well as into the protoplasmic processes of the glial cells that terminate at the surface. Due to the growth in length of the glial fibers, the embryonic and protoplasmic soft glial cell becomes stiffer. The growing glial fibers follow the most superficial zones of the cell body, which may partially be due to the minimal amount of protoplasm surrounding the nucleus.

In subsequent development, in addition to the few initial fiber-containing processes, all processes eventually contain fibers, and one can observe fibers crossing through the cell body. This indicates that the fibers penetrate the cell body and extend into a second process. In this manner, an immature glial cell with multiple, primarily protoplasmic and a few fiber-containing processes develops into a typical glial cell of the mature central nervous system. These cells are all-around radial-fibered, with fibers following the processes and surrounding the nucleus.

Secondly, based on these observations of the genesis and growth of glial fibers, it is not possible to define a specific origin for the fibers of these star-shaped glial cells, as they extend from one process to another and only traverse the cell body. An exception to this are the ependymal cells, which are unipolar and have a single process.

Here, one could define the endpoint of its glial fibers within the cell body.

Thirdly, it can be concluded regarding the development of fibrous neuroglia that Kölliker's hypothesis is incorrect in stating that glial fibers emerge from a superficial plate of glial cells.

Fourthly, it is evident that the regional restriction and relation of emerging and growing glial fibers cannot be confined to a single glial cell. Since these fibers emerge from the protoplasmic anastomoses of glial cells, it is impossible to attribute them to a specific glial cell. Moreover, during the subsequent growth of glial fibers, it is an illusion to assign a number of these fibers to individual glial cells as their sole cells of origin, since these long fibers emerging from one glial cell often reach a second cell body or even more.

This description of the formation of fibrous neuroglia aligns with observations in the adult nervous system, where it is evident that neuroglial fibers are part of more than one glial cell. I will elaborate further on the formation of neuroglial tissue in Chapter 6.

An open question remains as to whether the growing glial fibers pierce into the cell body, penetrating the cell membrane and advancing freely intercellularly instead of being part of the protoplasmic bundle. I have never observed such an occurrence. Evidence against this hypothesis is that, in the earlier phases of fiber development, single neuroglial fibers are surrounded along their entire length by a thin, dull, granulated protoplasmic cover.

This can be clearly observed when following those processes that stretch along the surface toward the common border membrane, terminating there with an enlarged, conical ending called the endfoot. Initially, these endfeet appear as simple, cone-shaped enlargements of previously thin protoplasmic processes (Fig. 28a). At a later stage of glial fibrillation (Fig. 28b), the stalks of these endfeet exhibit fibrous structures formed by the emerging and growing glial fibers.

These endings appear in the endfoot as free elements if one does not consider the protoplasmic content of the endfoot or examines them in radically bleached slices where only the glial fibers remain as stained and visible elements. I consider it possible that, during stretching due to the general growth of the nervous intermediate mass, the initially continuous cover of the glial fibers ruptures or degenerates, rendering it unrecognizable. In many locations, I have observed that close to the cell body and at the endfoot, the fiber is covered by a protoplasmic mass, while in intermediate areas this cover is not apparent.

The endfeet of certain glial cells undergo further changes during later development. In the following chapter, I will elaborate on their mature form, their relevance for the morphological characterization of the marginal neuroglia, and their contribution to the peripheral border of the central nervous system.

3. Marginal neuroglia, its peripheral border and its development

The central nervous system is characterized by its sharp border separating it from other organs, a feature that arises from the development and expansion of its internal support tissue, the neuroglia. These sharp borders are absent when studying peripheral nerves, such as those extending into muscles, even though these nerves are extensions of central nerve cells. It is generally recognized and accepted that neuroglial cells are specific to the central nervous system of vertebrates and are not found in any other region of an organism. The tissue of the neuroglia is emerging from the embryonic neural tube together with the nerve cells and is restricted to the original location of its development. This can be explained due to the properties of ectodermal tissue

which forms a special boundary surface facing the mesodermal connective tissue and separates it from foreign tissue. I therefore consider those cellular elements of the embryonic neural tube as the seedlings of neuroglia. These elements are exclusively pass-through components of the epithelial wall from the beginning and remain in that position during subsequent stages of development, in contrast to the detaching and migrating neuroblasts. Mauthner (1861) discovered the support system of the epithelium of the central canal, which is believed to merge into the fibers of the pia mater at the surface of the spinal cord. He was the first to postulate the ectodermal origin of neuroglia ([27] page 16): *"The neuroglia appears in a new light; since it is nowhere connected to the pia or the extending blood vessels, one has to consider that it is separate from the connective tissue. Moreover, it is distinct based on histology and must be considered as a tissue of its own."*

Boll[28] also emphasized that neuroglia arises in the same location as nerve cells. Subsequently, Goette[29] described "a membrane enwrapping the entire spinal cord, which is distinct from pia, since the vessels emerge only outside this membrane." He regarded it as "a cuticula which becomes separated from white matter when it shrinks. However, it still remains connected with delicate threads which originate from the fiber mass. These connections not only become more abundant at later stages but also merge into membrane-type formations that penetrate between the fibers of the white matter to varying depths, thus forming a lattice of dividing walls."

Hensen[30] showed in the following year in rabbit that the epithelium of the central canal as a radial fiber system of processes penetrates the entire mass of the cord and terminates with food-formed ends at the “superficial membrana prima”. His[31] demonstrated on human embryos that a system of radial fibers extends through the entire thickness of the cord,

which originates from prolongations of cells extending to the surface of the medullary tube. The outer endings of these fibers "form an independent layer as a first arrangement of spinal cord white matter." They enlarge in a trumpet-like fashion and eventually form the *Membrana limitans medullaris*, a type of limiting layer. This is not an uninterrupted layer and should be distinguished from the *M. limitans menigea*[*)], which is derived from net-like connective tissue cells as a smooth border layer and confines the spinal tubes at their outer border.

His further demonstrated[32] that a scaffold, the *Myelospongium*, is formed by the processes of spinal cord cells, "which will later become the support structure for nerve fibers and is now termed neuroglia." Additionally, His considered that this primary part of the neuroglia is supplemented by a secondary component originating from connective tissue cells that intrude along with ingrowing vessels. These cells form the Deiters cells of the white matter.

In a subsequent paper[33], His referred to the scaffold-forming cells of the embryonic brain as spongioblasts. However, these cells most likely have no connection to the origin of the Deiters supportive cells.

Vignal[34] and Gierke[35] meanwhile proposed a pure ectodermal origin of the entire neuroglia. However, a final proof was only provided by Golgi[36] on chicken embryos. His description is as follows (on page 169 of the German translation of "Untersuchungen über den feineren Bau des centralen und peripheren Nervensystems" Jena 1894): The cylindrical cells of the epithelium of the central canal "are not lost in larger or smaller distance to the grey matter, but extend radially through the entire cross-section of the spinal cord and extend to the outmost peripheral border of the organ, up to the pia mater. Here the thread-like processes of the cylindrical cells end, by forming a cone-like swelling or a thin extension,

[*)] it is a misunderstanding of the nomenclature by His when Lenhossek claims (Feinerer Bau des Nervensystems, II. Edition, Auflage 1895, S. 204): "The nodules convene, at the free surface of the cord, in a mosaic-like manner, to an apparently complete and very delicate border membrane, a form of a cuticula (*Membrana limitans meningea*, His), which entirely locks the ectodermal spinal cord against the pia mater".

with which they contact either the pia or the vessels running along there. On this long path, there are more or less stout fibers representing the peripheral processes of individual epithelial cell bodies. These fibers usually branch—sometimes sparsely, other times multiple times and in a complex manner. These secondary branches partially reach the periphery of the spinal cord and terminate there as described. Some, however, are lost along the way, and their fate cannot be determined, while others attach to the vessel walls.

"Based on these observations, it is evident that the epithelial part of the spinal cord, which originates from the outer blastodermic layer, contributes significantly to the tissue (neuroglia) embedded between the nervous elements."

Golgi added that, based on embryologic, chemical, and histologic observations, he concluded that the so-called neuroglial cells are synonymous with the epithelium. Similarly, Nansen[37] concluded for the myxine that neuroglial cells develop from the epithelium of the central canal.

The form of this developmental process—the emigration of epithelial cells and their metamorphic conversion into astrocytes—was first demonstrated by Cajal[38]. These observations were later confirmed and expanded upon by v. Kölliker[39], van Gehuchten[40], von Lenhossek[41], Retzius[42], and Cl. Sala y Pons[43].

Lachi[44], Valenti[45], Capobianco[46], and Hatai[47] opposed the doctrine of a uniform ectodermal origin of neuroglia as later reported by His. However, I will not elaborate further on this. In my opinion, neuroglia contains no components of mesodermal origin. I will discuss this in more detail with the following observations.

I divide the development of neuroglia into the final support tissue into three periods. The first period represents the formation of the primary glia. It is characterized by the fact that the central scaffold is composed exclusively of the processes of the central canal epithelial cells of the embryonic tube or by the embryonic ependymal cells.

In *Amphioxus*, the glial cells remain at this first stage. In other classes of vertebrates, however, this stage is typical only during the initial phase of brain development. The second stage leads to the development of secondary cellular glia and its further progeny, which include, in addition to the pure and later reduced ependymal cells, transformed ependymal cells and astrocytes. The third stage comprises the formation of glial fibers, which occurs within the tissue of the previously described purely cellular glia and results in the development of a secondary cellular-fibrous neuroglia.
I now return to the boundary of the embryonic central nervous system formed by the primary glia. According to His, the spongioblasts extend through the embryonic brain and form, with their inner surface, the *Membrana limitans interna*. This structure confines the central canal of the spinal cord and the brain ventricles. It is characterized by a distribution typical of masses of surface epithelial cells united by putty lines, which are considered to constitute the mature ependyma of the central nervous system.
The epithelial nature of this internal border membrane is evident in my preparations by the presence of a double granule located directly beneath the delicate, cuticular end plane. This structure is considered a central body at the inner end of the cylindrical epithelium. My preparations include samples from guinea pig embryos (2.3 cm), rabbit embryos (3.7 cm), and a similarly developed dog embryo. In the latter two, the *plexus chorioideus* is already characterized by basal bodies with cilia.
On the outer surface, a coarse-scaffolded structure (*Myelospongium*) emerges from the corresponding parts of the spongioblasts, as described by His. This forms the peripheral zone of the brain tube, creating a border haze. *"The main components of this border haze are numerous, sheath-like radial bars, which are broadened at the outer end and interconnected by crosswise rungs."* This border haze is smaller and denser during early development. At later stages

this meshwork gets looser; but still at a time when numerous nervous longitudinal projections are present, the primary arrangement of the cord scaffold in its radial structure can be recognized" (section on neuroblasts, page 239). His remained undecided whether the single gaps of the border haze are open or closed to the outside. In his publication from 1883, His states that the name *M. limitans medullaris* which forms the outer border membrane of the border haze, would not mean an "uninterrupted membrane".

I conclude that the border haze described by His is continuously covered by a delicate membrane, which later thickens and persists as a permanent remnant of an epithelial surface sealed off from the exterior. It grows through the addition of elements from the interior, namely spongioblasts and later glial cells, leading to organ enlargement. I would designate this structure as the *Membrana limitans medullaris superficialis.*In flat slices, one observes a structure corresponding to the fields of the *Membrana limitans interna* described by His. Coronal sections demonstrate that numerous cone-shaped endfeet of the spongioblasts converge at this point (Fig. 21) of the spinal cord of the embryo EB from the His collection). At this stage, the border haze is still in its initial developmental phase. In the embryo Br 3, which I had the opportunity to study thanks to the courtesy of Privy Councilor[(p)] His, the border haze is already more extensively developed. Its spaces are framed by a border membrane that is thicker compared to the one observed in embryo EB. Its origin from the endfeet of the spongioblasts is evident (Fig. 22). I should note that, both generally and particularly in the case of Br 3, the fixation and embedding processes could result in a rupture of the superficial layer of the border haze containing the endfeet of the spongioblasts. This disruption can obscure the genetic association between the border membrane and the border haze described by His. If one terms the compacting mesodermal tissue, according to His, *M. limitans meningea*

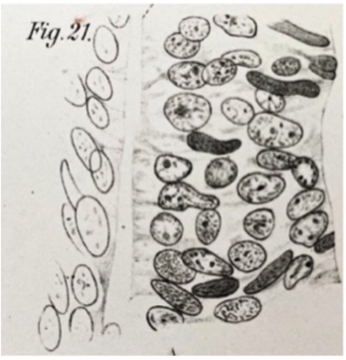

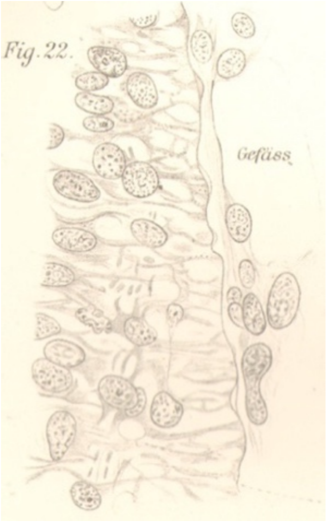

Fig. 21 from Table II. Part of a spinal cord cross-section of a human embryo E B. Preparation from Geheimrat W. His. Hartnack 1/12 Oc. 6.

Fig. 22 from Table II. Part of a spinal cord cross-section of a human embryo Br 3. Preparation from Geheimrat W. His. Hartnack 1/12 Oc. 6.

then the *M. limitans medularis superficialis* would represent the partition of the ectodermal brain tube. According to my observations, the *Membrana limitans superficialis* is formed everywhere, serving as a complete diaphragm for the mesoderm, which later develops the vessels. Thus, in the beginning, all vessel sprouts are exclusively condensed within the layer of the *M. limitans meningea* and, for some time, are completely restrained and pushed away from the superficial zones of the border haze by the *M. limitans superficialis* (Fig. 22).

This initial stage of superficial accumulation of newly formed blood vessels is followed by a stage of penetration into the brain tube. This process occurs in the manner illustrated in Fig. 23 to 25. A shim-shaped young vessel sprout intrudes and splits the *M. limitans superficialis*, as shown in Fig. 23, by advancing along a putty stripe between two neighboring fields of spongioblasts. This intrusion separates the deeper parts of the border haze, which remain grouped into individual areas of epithelial cells. The subsequent stages of penetration are depicted in Fig. 24 and 25 at the same position in a rabbit embryo. These figures show that the continuous ingrowth of the first vessels into the brain simultaneously leads to the progressive new formation of a *M. limitans* from the deeper layers of the border haze. This newly formed membrane isolates, similar to the *M. limitans* at the outer surface, the intruding vessel-containing connective tissue from the genuine epithelial mass of the brain.

I propose naming this internal border membrane the *M. limitans perivascularis*. Located within the brain tube, it is formed from branches of the spongioblasts. This membrane enwraps individual vessels in a tube-like septum and is continuously connected at the superficial entry zone with the *M. limitans superficialis* of the brain.

These findings are not limited to human and rabbit embryos. Even more pronounced formations of the superficial and interior border membranes of the central nervous system were observed in embryos of the grass snake, frog, salamander, trout, electric ray, chicken, guinea pig, mouse, cat, and dog.

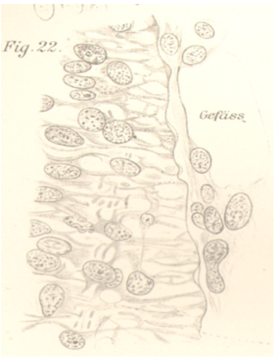

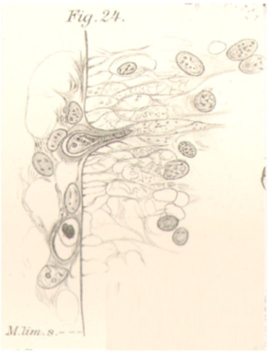

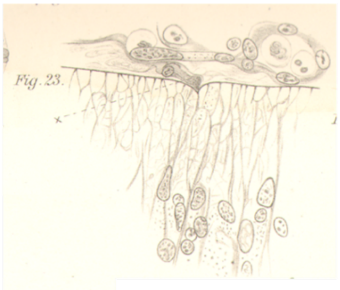

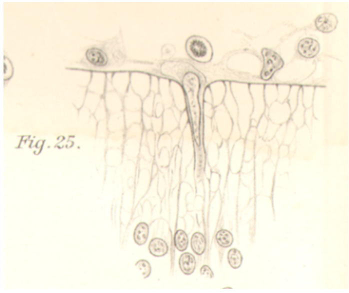

Fig. 22 from Table II. Part of a spinal cord cross-section of a human embryo Br 3. Preparation from Geheimrat W. His. Hartnack 1/12 Oc. 6.

Figs. 23-25 from Table II. From a rabbit embryo. Preparations from R. Altmann. Figs. 23-25 from the lateral surface of the prolonged cord.

That I could study this comparative material within a short time, I owe to Privy Councilor His(p) and Professor Kästner, who kindly provided me access to their collections. The border membranes of the adult nervous system are the same as in the embryo; they are partially positioned directly under the pia mater and correspond to the *M. limitans superficialis* as the first closure of the brain tube, which is not yet penetrated by vessels. The other part enwraps the connective tissue-like and vessel containing depressions or septa of the pia as perivascular membranes.

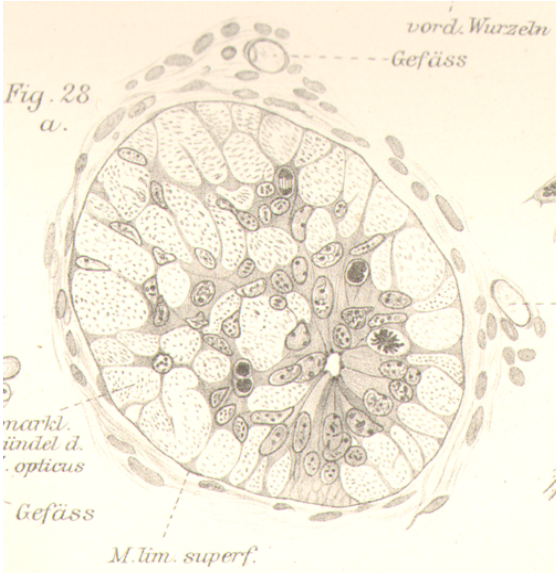

Figs. 28a-c from Table II. Three developmental stages of the Nervus opticus of the mouse in cross-section. Fixation in chromium-formalin , acidic acid. Also hematoxylin staining. Hartnack 1/12 Oc. 4.
Fig. 28a from a 12-day old embryo.

For the adult nervous system, I will now use the term "border membrane of neuroglia," as my observations that glia is of purely epithelial origin support the findings first indicated by Golgi and later confirmed by Ramón y Cajal through silver impregnations. The formation of secondary cellular glia results from the emigration and subsequent morphological modification of cells originally residing in the ependyma.

I can corroborate this with a method other than the Golgi silver impregnation in the optic nerve, thereby supporting the results of this technique, which have recently been questioned. *Fig. 28a* illustrates the formation of secondary cellular glia from the ependyma in the optic nerve of a 12-day-old mouse embryo. The ependyma still forms the remnant of the hollow eye shaft at its lower edge, while in the remaining part of the optic nerve, multiple nerve bundles are already surrounded and enclosed by a coherent mass of star-shaped, branched cells.

This interior mass of support cells, together with the nerve bundles, constitutes the white matter. This region of the nervous system exhibits this pure character from the outset and retains it. This observation confirms the development of cellular neuroglia, as in contrast to other areas of the brain tube, cell migration in this region is not confounded by nerve cells.

That all internal astrocytes are of ependymal nature is demonstrated by the quoted figure; that it grows and multiplies internally show single, but ubiquitous mitoses;

that no mesodermal cells are mixed in is indicated by the closed border membrane which enwraps the surface of the optic nerve and separates it. The invasion of vessels into this completely enclosed and purely epithelial tissue occurs only after birth, marking the formation of the internal border membranes. This period coincides with the onset of glial fibre production. The earliest stages of border haze formation are shown in Fig. 28a. It is evident that the ependymal cells extend to the lower edge, where the formation of lacunas can be observed—an initial phase in the development of the border haze, as reported by His (W. His, *Histogenese und Zusammenhang der Nervenelemente*. Lecture at the anatomical section of the International Medical Congress in Berlin, August 7, 1890).

In alignment with this developmental process, the border membranes of the adult nervous system are of neuroglial origin. I classify them as the *Membrana Neurogliae superficialis* and the *Membrana Neurogliae perivascularis*. I have observed these membranes in preparations where the neuroglial fibres, the protoplasm of the glial cells, and the membrane mass are simultaneously stained and distinguishable. My description is based exclusively on such preparations.

To begin, I must state that in Weigert's neuroglia staining, the relationship between glia and the *Membrana limitans Gliae* remains obscured. This method, although pivotal for identifying the normal and pathological arrangement of glial fibres, involves radical bleaching of all other glial components. It is therefore unsurprising that the Weigert doctrine has led to a biased interpretation. Nonetheless, I acknowledge its importance in distinguishing between glial cell processes and glial fibres.

In accordance with the intracellular origin of glial fibres discussed in Chapter 2, the adult nervous system no longer contains simple or loosely formed astrocytic processes. Instead, glial fibres approach and integrate into the border membrane, which I will now describe in greater detail.

This process resembles the endfoot formation characteristic of primary and secondary cellular glia. My preparations also reveal the presence of purely protoplasmic glial endfeet. The capillaries of the large cortical layers in the cerebellum and cortex predominantly exhibit this formation of the perivascular border membrane. Similar formations are often observed in the *Membrana limitans Gliae* of the cortex, though they are more frequently interspersed with fibre-containing endfeet. This indicates conditions similar to those described above, distinguishing between fibre-containing and fibre-free glia.

In contrast, in the white matter, the superficial layer of the cerebellum, the border membrane of the spinal cord, and the *Substantia Neurogliae centralis*, such relationships among glial cells are primarily mediated by glial fibres. I classify two types of glial endfoot formation, without considering variations where one or more fibres access the endfeet in a unified manner. In one primary form, the glial fibres or their corresponding bundle (Fig. 39b, c) terminate as dark-stained structures and transition into a pale, often frayed substance forming the foot. Whether this represents a change in substance or simply a loosening caused by staining differences remains uncertain. Delicate and generally frayed endfeet are observed, for instance, at the terminals of the Bergmann fibres in the cerebellum (Fig. 33a, b).

The second primary form involves a glial fibre or single fibres within a bundle that penetrate the endfoot and continue in a flattened manner into the corresponding border membrane. These fibres are typically truncated in the sections, but occasionally I have observed them terminating after a short course. This observation aligns with Retzius's description of hook-shaped bends of glial fibres at the *Intima Piae*.

This second type of glial endfeet is interesting since it is proof that there must be something else, special considered for the formation of the glial endfeet, which is also present, but hidden in the first form,

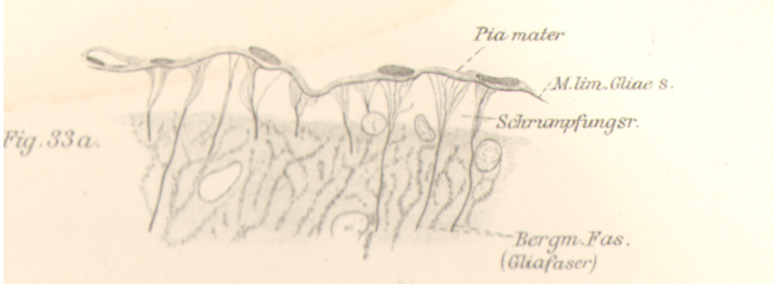

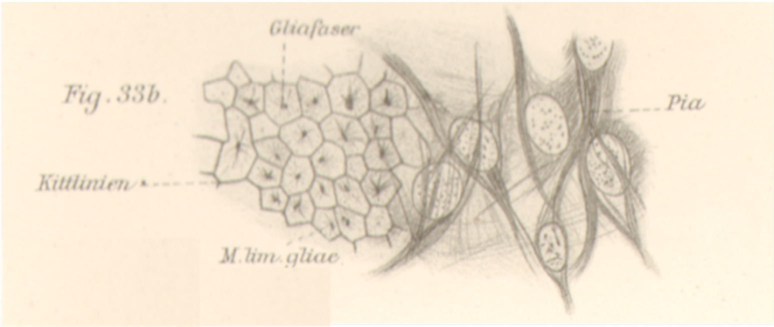

Fig. 33a from Table II. Vertical cut through the entire cerebral cortex.
Fig. 33b. Plane image of the M. limitans Gliae superficialis (left) and the Intima piae (right).

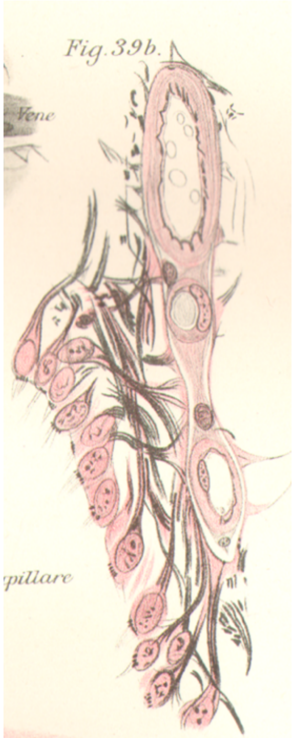

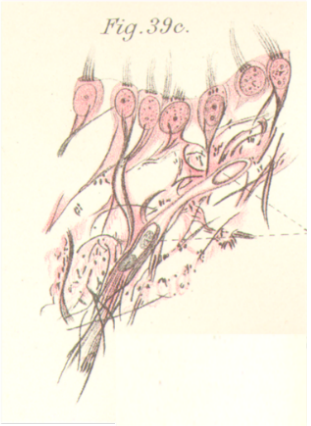

Fig. 39b-c from Table III. Glial vessel insertion in rabbit. b and c are from the Substantia Neurogliae centralis of the spinal cord, a from the IV. ventricle. Hartnack 1/12 Oc. 6.

and which represents more than an enlargement of a changed or loosened substance of the glial fibers. This distinct feature is found only at locations where it becomes clearly differentiated from the dark-labeled fibre, which it enwraps as a delicate, slightly folded membrane. The structure increasingly assumes a cone- or pyramidal shape, extending around the fibre it encloses, and terminates with a broad base as a glial membrane. With regard to these embryonic foot forms, this membrane is equivalent to a cell membrane or the superficial part of a glial cell.

Additionally, embedded protoplasmic granules can be observed alongside the fibres, as demonstrated in some of my preparations. I conclude that this glial foot membrane is not merely a residual protoplasmic structure or a transformed substance but rather a product of the glial cell, holding the same significance as the glial fibres themselves.

Moreover, in a terminal frayed glial fibre, the diverging fine beams form a support system within the membrane, which can be traced to the terminal membrane as its insertion site (Fig. 33a, b, see above). This terminal glial membrane and its associated foot can thus be regarded as a functional cell membrane.

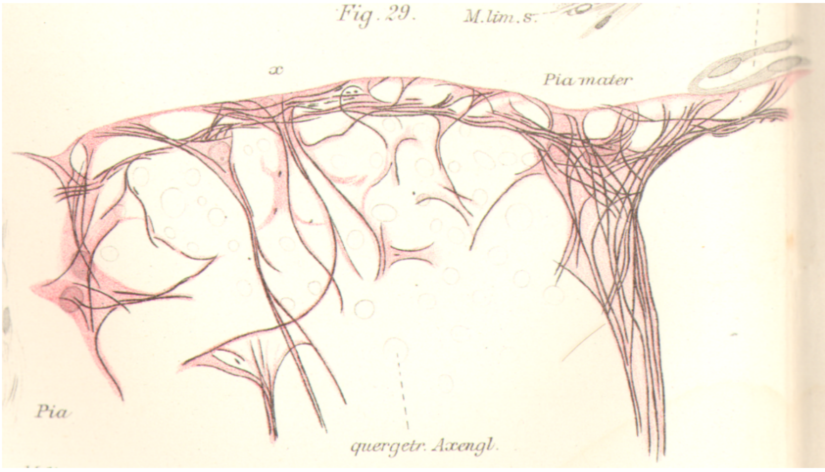

Fig. 29 from Table II. Surface of the lumbal spinal cord of an adult rabbit (area of the lateral streak). Potassium bichromium and ammonium molybdenum. Hartnack 1/12 Oc. 4.
In the interior of the white substance the glia is not completely marked. At x is a glial cell inserted with their cell body into the M. lim. Gliae superfic. The band of dense glia left to outer blood vessel will provide in the subsequent slices a marginal and a perivascular glia corresponding to the inserted blood vessel.

This is supported by the multiple variations which can be observed in the marginal glia which also allow an evaluation of the foot.

It is most obvious when the cell body is directly inserted into the border membrane which is not rare. Here the plane of the foot is directly located within the membrane of the cell body. Fig. 29 shows this at the *M. lim. Gliae superficialis* of the spinal cord of a rabbit. I have also observed this in human, both on the superficial as well as on the perivascular border membrane. Also, the earlier reports by Golgi are relevant here stating that “the cell bodies are closely attached to the vessel walls” so that “on their long course they are often surrounded by a tight row of radiant cells and their cell body

seems almost to be a part of the vessel wall." I consider them as a row of cell bodies which, according to my observations

in the white matter of the human brain build up large and long segments of the perivascular border membranes. At the region where a glial cell transitions into the perivascular border membrane through a broad, glial fibre-containing process (Fig. 15), the glial foot can similarly be considered as described above. This remains evident even when the glial fibre is surrounded by a slim, barely visible cover resembling a pale seam, or when it is accompanied by a fine, connecting mass extending from the cell body to the endfoot. However, there are many glial fibres where this characteristic is less apparent.

In some preparations, I observe a continuous red seam enwrapping the fibres when they are darkly labeled and the protoplasm is visualized using an alcoholic erythrosin solution or the van Gieson picrocarmin mix. However, I do not claim that this seam represents a protoplasmic sheath or merely a cell membrane covering the fibre. For the fibre itself, one might hypothesize a differentiated form or contrast staining of the cortical layer, as A. Fischer demonstrated with mirror differentiation in granular structures. In another group of glial fibres, there appears to be a thin, incomplete ensheathment that is sometimes angled, ragged, or dull granulated.

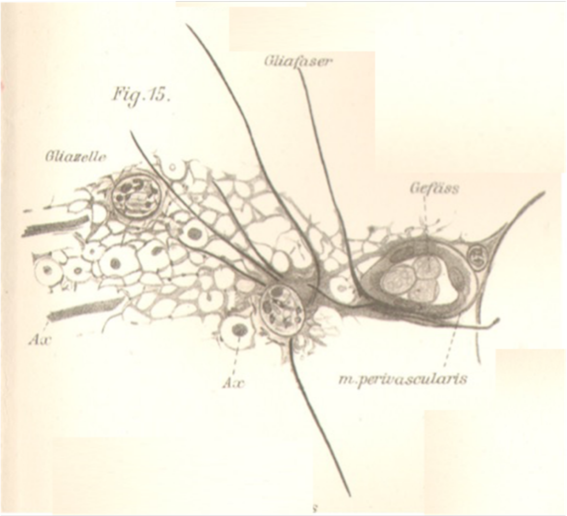

Fig. 15 from Table I. Net-shaped branched glial cell from the white matter of the human cerebral cortex (24-year-old executed). Alcohol-chloroform, acidic acid mix. Hartnack 1/12 Oc. 8.

These ensheathments or coverings on a glial fibre can be interpreted as remnants of an originally complete protoplasmic element, as seen in embryonic glial cells during the onset of fibre formation. For fibres that appear completely smooth, a reduction to just the foot is likely, whereas in the first form, this reduction does not occur. Here, a short process between the cell body and the border membrane remains enwrapped by a thin, continuous cover around its fibre or fibre bundle.

Such a structure would differ from a purely protoplasmic process only in the absence of fibre formation. Based on these observations, I identified and named the border membranes that cover the adult central nervous system

Membranae limitantes Gliae superficialis et perivascularis. I consider these equivalent to cell membranes, and it is evident that their entire surface is formed from the multiple glial endfeet, as revealed through analysis of these structures. This conclusion is further supported by observations from flat mounts, in which the entire border membrane is visualized after appropriate fixation and staining. Before describing these in detail, I will provide a brief overview of how the concept of the glial border membrane has been appreciated thus far.

The *Membrana Neurogliae* is identical to the subpial or perivascular endothelial membrane described by Gierkes (48). Gierkes incorrectly classified it as part of the pia mater, describing it as homogeneous, structureless, and composed of flat cells, even though he noted its connection to many fine processes of the glial envelope and glial cells via foot-like enlargements. It is also equivalent to the *Membrana limitans* of the cerebellum, as described by Bergmann (49). The connection of this structure to the endfeet of the Bergmann fibres already argued against its classification as part of the pia.

Golgi's (1885) observations are particularly relevant for recognizing the *Membrana Gliae perivascularis*. He first described it as an accumulation of glial processes. His report[50] states the following (page 116): "The attachment usually starts with a spreading, sometimes in a cone-formed, limited form, sometimes very thin and without obvious borders, so that one may almost say it forms a perivascular membrane. At the capillaries and small arteries, which do not have adventitia [(r)], the attachment seems to be directly at the endothelial wall of the former, and at the thin muscular membrane of the latter. Also, in those cases, the entire spread of cellular processes seems to form a complete coverage, which is directly attached to the brain wall and which can be considered an additional kind of adventitia."

Henle and Merkel[51] previously described a border membrane composed of delicate, crossing connective tissue fibres located beneath the pia. They suggested that this forms the Bergmann membrane in the cerebellum, with delicate fibres protruding into the brain mass. Similarly, Roth (1867) described fine radial processes with cone-shaped excrescences at the pia mater and throughout the blood vessels of the brain

and found that in the calf brain they were connected with coarse threads to star-shaped cells; also, Boll (1873) observed that the adventitial borders of brain vessels were covered by villus-like, stalked extensions from Deiters cells (referred to as "brush cells" by Boll), which were briefly mentioned by Golgi (1870 and 1871–1872). However, none of these researchers concluded that this structure constituted a distinct border membrane. In particular, Golgi should be credited, alongside Gierke, for recognizing the significant relationship between glia and the vascular system of the brain.

In contrast, I find Golgi's criticisms of the surface membrane of the brain, particularly against the Bergmann membrane, to be incorrect. His assertion that the surface of the cerebellum is composed only of a thin layer of easily recognizable glial cells, which are more prominent in the cortex, does not definitively prove the absence of a border membrane.

Schaffer[59] later argued that one must differentiate the glial cover described by Gierke, consisting of circular and longitudinal fibres, from a radial fibre layer that terminates in radial fibre endings within the border membrane. This border membrane completes the superficial pia and the adventitia of the penetrating vessels, thereby ensuring that "glia and nervous substance of originally ectodermal origin are sharply separated from intruding mesodermal elements."

Renault[53] provided evidence for a superficial covering of the spinal cord in *Petromyzon*, composed of multiple distinct fields, using a staining mixture of picric acid, osmium, and silver. This method was based on the procedure developed by Schelske, who demonstrated, with a silver stain, the composition of the inner border membrane of the human retina formed by the terminal feet of Müller cells.

Renault described each of these fields as being cemented to its neighbors and representing the attachment of a neuroglial fibre. These small fields, resembling basal plates, are connected via the corresponding glial fibres to multiple neuroglial cells. Renault concluded that the neuroglia is of ectodermal origin and that these cells possess epithelial characteristics, further challenging Weigert's doctrine of the independence of glial fibres.

At the international medical congress in Paris (1900), Renault emphasized during the discussion following Marinesco's presentation (*Du rôle de la nevrologie dans l'évolution des inflammations*) that he found similar conditions in mammals and that he was furthermore convinced that the so-called lymph border of the vessels has a similar formation and can be considered an extension of the superficial lamina of the central nervous system.

Finally, E. Müller[18] demonstrated with his own method that glial fibers have a distinct endpoint, contrary to Weigert's findings. His observations in *Myxine* are as follows: “The glial fibers terminate, similar to the ependymal fibers, at the periphery of the cord with small, unlabeled feet, which form a continuous border layer against the pia. Such endings can also be found in the interior of the cord, where these endfeet of the glial fibers are attached to the vessels. Based on the regular pattern of the endings of traceable glial fibers, one can conclude with confidence that all glial fibers end in a similar fashion with unstained endfeet at the periphery or on vessels.”

This conclusion is further supported by the reports of Kure[54], who stated that glial cells are directly connected by protoplasmic processes to the vessel wall or the endothelium (*Neurol. Centralbl.*, 1902, page 1017).

My subsequent observations align with the various statements about the border membranes and glial endfeet in the context of Golgi's findings. I extend these observations to mammals and humans, where, as is well known, Weigert—due to his biased method—opposed Golgi's doctrine. It is entirely incorrect and unsupported when Weigert claims that, based on his preparations, neuroglial fibers "never show the conic- or bottle-like enlargements" that are evident in Golgi's preparations. Furthermore, when Weigert concludes that his efficient staining method is definitive and that the feet are merely putty substance or artifacts, this assertion is without merit. Weigert also failed to notice those fragmented glial fiber endings, respective

could not label them with his radical destaining method, which results from his remark that the “glial fibers remain slim and uniform up to their end”. A remark suggesting that those fine endfeet are destained as a consequence of a distinct chemical nature, and that they are therefore not part of the glial fiber, I cannot support, since I have observed how easily and rapidly those thin fiber formations can disappear under defined staining conditions.

In Fig. 33b and Fig. 35, respectively Fig. 36 (left side), I have illustrated flat images of the surface of the glial border membrane. Fig. 33b shows the partitions at the cerebellum of the rabbit, while Fig. 35 depicts the human cortex. I should add that I observed similar arrangements in the human spinal cord and human cerebellum. Fig. 33b, in particular, highlights the frayed ends of the glial fibers (ends of the Bergmann fibers) and their approximate radial attachment at the free glial endfoot plate. These individual foot plates are connected by putty lines, which remain labeled in black due to the Haidenhain method (with distinct fixation).

The border membrane itself, which appears in cross-sections as a fine, smooth line that elongates into its interior in multiple cones, looks matte and granular in flat mounts if one studies little or undifferentiated preparations. I have also observed such fields at the large vessels in the superficial brain layers. Fig. 41 shows them at a vein in the human cortex. Unfortunately, I could not generate good flat-mount pictures from the capillaries. Apparently, the border membrane and its putty lines are so fine that it was not possible to obtain clear images. Fig. 35 illustrates the transition from the superficial partitions to the perivascular glial membrane in the human cortex in flat mounts.

As mentioned above, Renault reached two conclusions regarding the formation of the lamina vitrea. First, the glial cells are of epithelial nature; second, the neuroglia is therefore a pure epithelial tissue. I do not have any objections against the first conclusion. For the second, however, one might argue that at some stage during the development of the glial border membrane, an invasion of mesodermal tissue could occur.

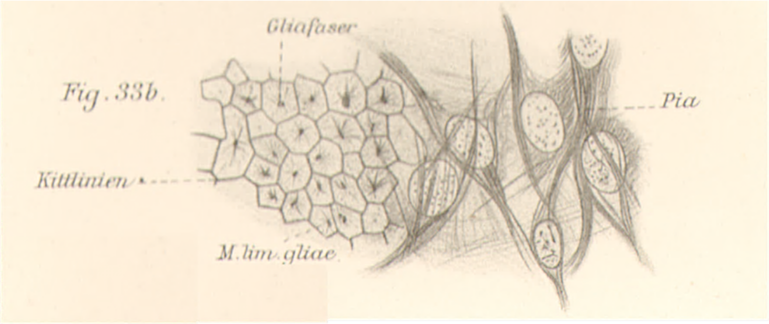

Fig. 33 b from Table II. Surface of the cerebellum of an adult rabbit. chromium-formalin, acidic acid. Hartnack 1/12 Oc. 6. Fig. 33b. Plane image of the M. limitans Gliae superficialis (left) and the Intima piae (right).

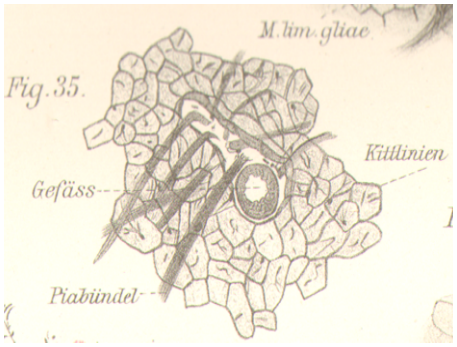

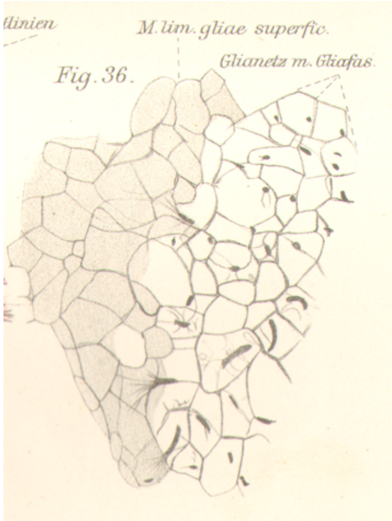

Fig. 35 from Table II. Surface image of the M. lim. Gliae superficialis at the human cerebral cortex. Same preparation. Entry location of a small blood vessel. Single bundles of the pia are cut.
Fig. 36 from Table II. Skewed cut of the same preparation. On the left, the border membrane is cut, on the right the glial space below.

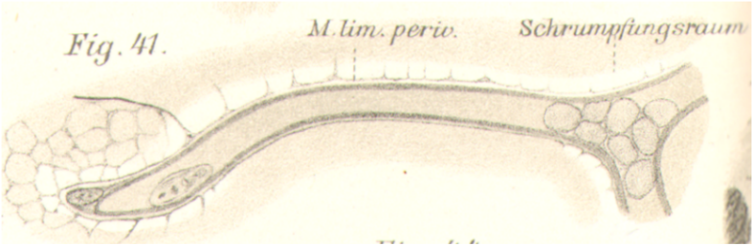

Fig. 41 from Table III. Glial border membrane of a capillary from the human cortical grey matter (24 year old-executed). On the left, the glial reticulum is partially visible. At the left end of the capillary is an adventitial nucleus which is surrounded by a small, very delicate mass of protoplasm as a partially visible sheath between the endothelial tube and the perivascular glial membrane.

To exclude this, embryological studies are required.

As I have described at the beginning of this chapter on the marginal glia and its development, it is evident that mesodermal cells do not intrude together with the ingrowing vessels into the brain substance, since instantly fine borders are formed with radial fibres along the lines of the blood vessels. This ensures that inside the brain parenchyma its properties remain isolated by an internal surface. Moreover, I have nowhere found that the cells of the invading vessels mix with the intrinsic brain cells. One could speculate that at the breakthrough of the nerves at the superficial border membrane, conditions for the invasion of mesodermal cells could be created. This is not the case as shown in Fig. 26 and 27, demonstrating that mesodermal cellular elements are retained at the penetration point of motor and sensory nerves. It is remarkable that the processes of the ectodermal neuroblasts can penetrate the border membrane or mesh-like perforate it, without leading to a response of the mesoderm seen upon intrusion of vessels. Corresponding to the primary breakthrough positions, it is also evident in the adult nervous system that there is a defined border at brain or spinal cord nerves, at which first the peripheral formation of the Schwann and then the Henle sheaths start. However, distinct from the situation in the embryo, these mesodermal ensheathments of the single nerve fibres are not located at the level of the *Membrana limitans superficialis*, but moved towards the peripheral part of the nerve. A fact, which was already emphasized by Schaffer and Weigert, by finding neuroglia at the beginning of a dispatching nerve. This peripheral pre-emplacement of the glial sheath is based on the pushing of central neuroglia through the penetration points due to growth and the formation of stiff fibres. If one confines in general the central nervous system towards its *Membrana limitans Neurogliae*, its real end would not coincide with the smooth and flat surface as apparent based on all the previous observations.

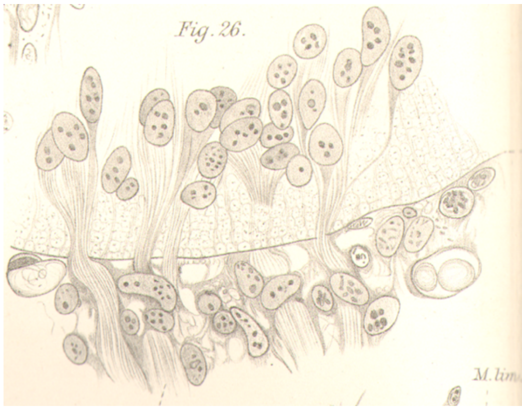

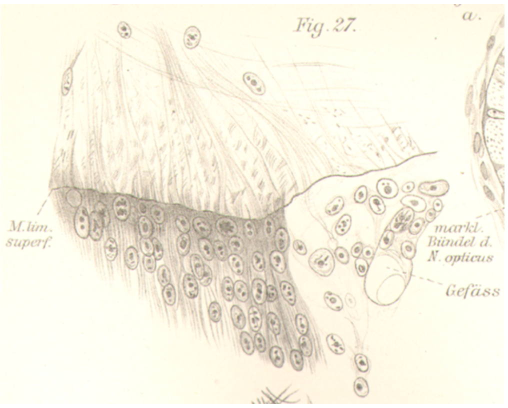

Fig. 26 from Table II. From a rabbit embryo. Preparations from R. Altmann. Fig. 26 of the spinal cord. Hartnack 1/12 Oc. 6.

Fig. 27 from Table II. From a chicken embryo on the 5th day of incubation. Preparation from Professor S. Kästner. Transit position of the sensible trigeminal nerve through the M. limitans Gliae superficialis. Hartnack 1/12 Oc. 6.

On one side, it extends into depth along the course of the blood vessels; on the other side, it protrudes with small humps to the outside where the nerve roots are located. At the *N. acusticus* of the guinea pig and mouse, it is expanded so far that it terminates only within the *Meatus acusticus internus*, through a cross-border membrane at the end of the nervous system.

The marginal glia is in the depth of the parenchyma characterized by a particular layer, which was already observed by Henle and Merkel, Roth, Golgi, Boll, Gierke, Schaffer, and others. It is the zone that connects the deeper glial elements with the border membrane proper and is characterized by a radial orientation of glial feet and fibres or by pure protoplasmic processes, respectively, in a glial reticulum oriented as radially arranged or fibrous bars. These superficial glial cells, directly embedded in the border membrane, do not change the general characteristic of the latter. I will term it the border membrane of the glia. With certain exceptions, such as partially in the cerebellum, it forms a structured layer distinct from the cortical layers below (Frommann[55], Weigert, the glial cover of Gierke). Fig. 29–32a show this relationship at the surface of the spinal cord (rabbit and human), Fig. 33 at the cerebellum (rabbit), Fig. 34–38 at the cortex, and Fig. 38–43 at the area of the internal vessel surface.

In Weigert's figures of neuroglia and the termination of the central brain mass resulting from it, one cannot recognize the glial border membrane, or the glial endfeet nor the border membrane of neuroglia. Weigert depicted only one cortical layer, which was already known by Frommann[55] and Gierke (glial cover). Weigert considers it as the last and outmost border of the brain towards the pia and its intruding vascularized septa. My observations show that this is not a real termination but only the biased result of strong destaining. Weigert reports about this cortical layer (page 146): "the main mass of the fibers seems to run more of less askew tangential,

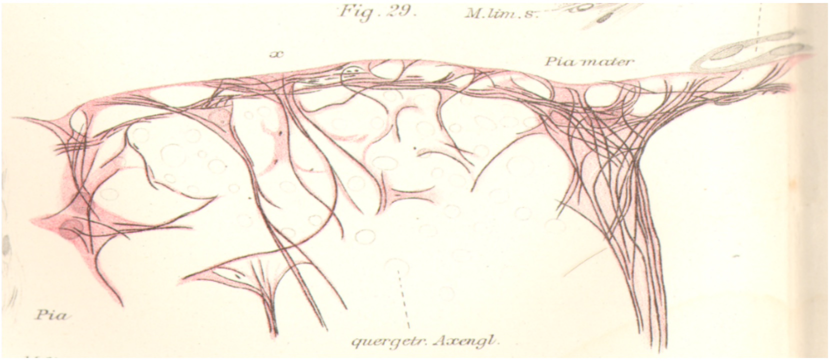

Fig. 29 from Table II. Surface of the lumbal spinal cord of an adult rabbit (area of the lateral streak). Potassium bichromium and ammonium molybdenum. Hartnack 1/12 Oc. 4.

In the interior of the white substance the glia is not completely marked. At x is a glial cell inserted with their cell body into the M. lim. Gliae superfic. The band of dense glia left to outer blood vessel will provide in the subsequent slices a marginal and a perivascular glia corresponding to the inserted blood vessel.

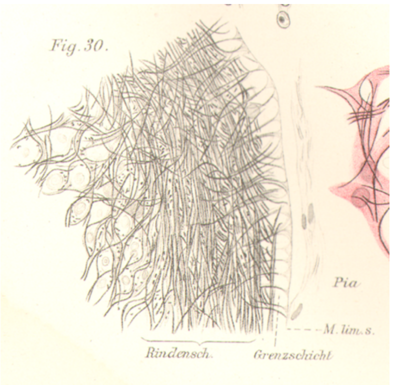

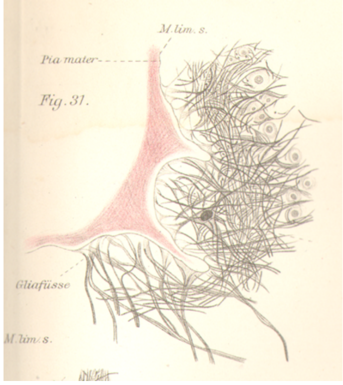

Fig. 30 from Table II. Dorsal surface of the human spinal cord (bend towards the septum posterior) at a cross-section of the lumbar cord (24-year-old executed). The pia mater has been plainly taken off from the M. lim. Gliae superficialis. Hartnack 1/12 Oc. 2.
Fig. 31 from Table II. From the same slice. Depth of the Fisura long. ant.

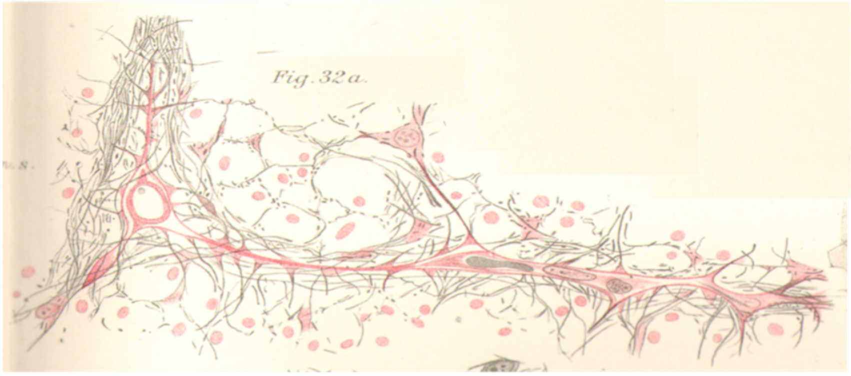

Fig. 32 from Table II. From the same slice. Boundary of a septum of the pia mater from a dorsal root which appears vessel-free.

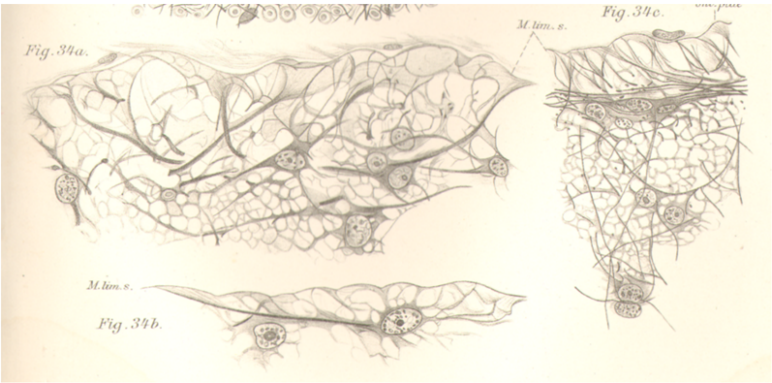

Fig. 34a from Table II. Less differentiated slice. Chamber-like structure of the marginal glia; the glial fibers appear at some locations as deeply dark enforcement bands; Fig. 34c shows the differentiation of glial fibers and their fibrillate endfeet. Fig. 34b

shows the smooth isolation of a detached pia, which in contrast is closely and uniformly attached in Fig. 34c. In Fig. 34a, the Intima piae is bleb-like detached resulting in a epi-cerebral cleft. Hartnack 1/12 Oc. 6.

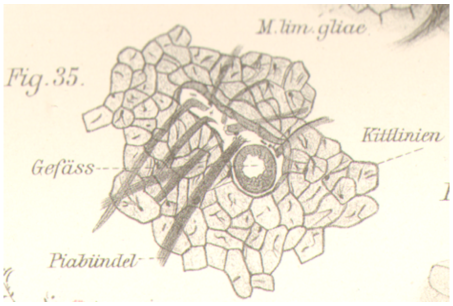

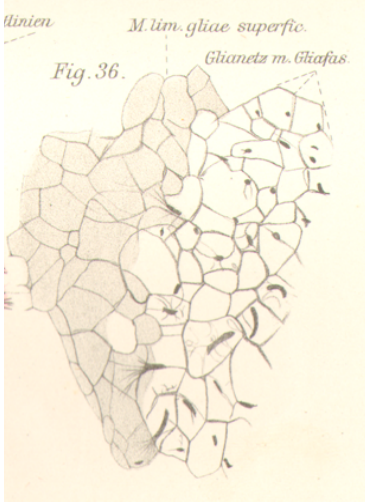

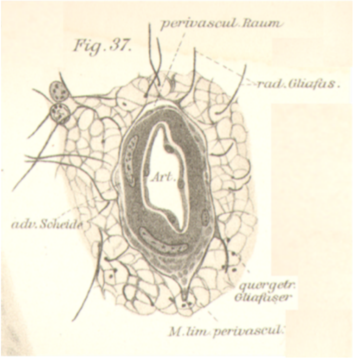

Fig. 35 from Table II. Surface image of the M. lim. Gliae superficialis at the human cerebral cortex. Same preparation. Entry location of a small blood vessel. Single bundles of the pia are cut.
Fig. 36 from Table II. Skewed cut of the same preparation. On the left, the border membrane is cut, on the right the glial space below.
Fig. 37 from Table III. Cross-section through a small artery in the superficial glial zone of the human cerebral cortex. On top a small perivascular space is open; the adventitial sheath is tightly compressed. Hartnack 1/12 Oc. 6.

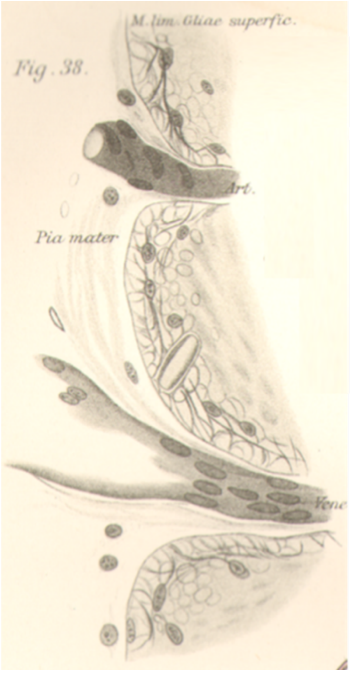

Fig. 38 from Table III. Entry of two vessels into the substance of the cerebral cortex. Transition of the superficial into the perivascular border membrane of neuroglia. From the same brain. Seitz Obj. 4 Oc. 2.

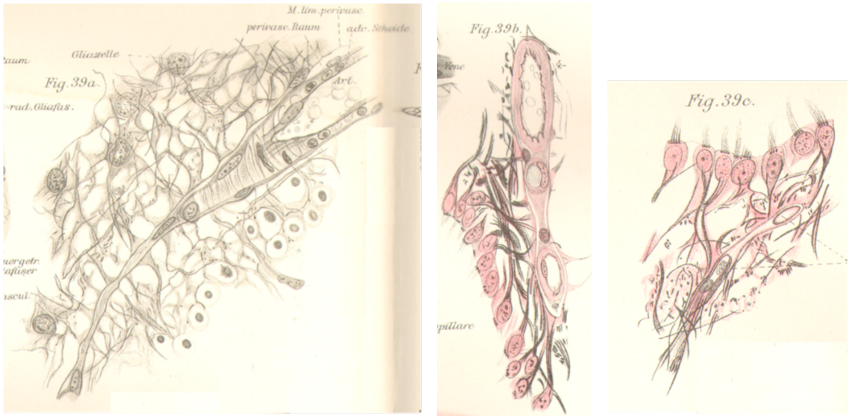

Fig. 39a-c from Table III. Glial vessel insertion in rabbit. b and c are from the Substantia Neurogliae centralis of the spinal cord, a from the IV. ventricle. Hartnack 1/12 Oc. 6.

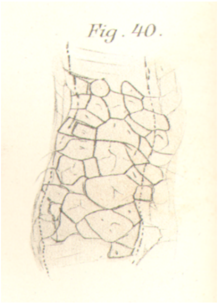

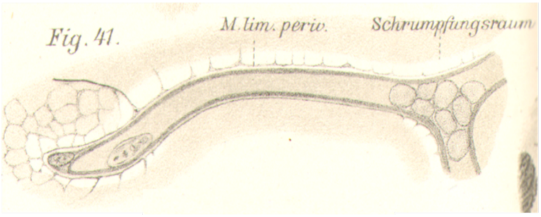

Fig. 40 from Table III. Arial image of the perivascular border membrane of a vein of the rabbit elongated cord. The dots, respective lines in the single fields indicate the position of the glial fibers below it. Hartnack 1/12 Oc. 6.
Fig. 41 from Table III. Glial border membrane of a capillary from the human cortical grey matter (24 year old-executed). On the left, the glial reticulum is partially visible. At the left end of the capillary is an adventitial nucleus which is surrounded by a small, very delicate mass of protoplasm as a partially visible sheath between the endothelial tube and the perivascular glial membrane.

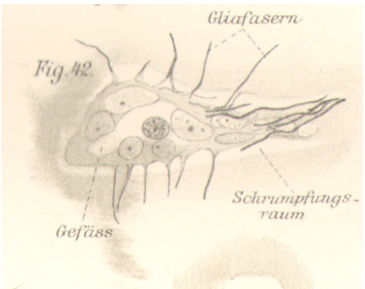

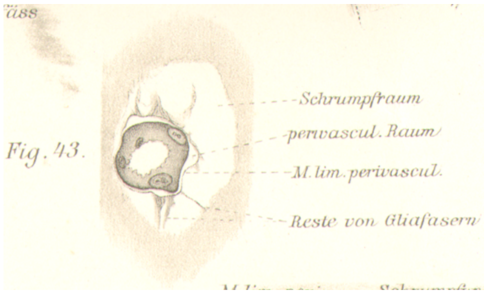

Fig. 42 from Table III. From the white matter of the human cerebral cortex. Alcohol-chloroform-acetic acid. Highly differentiated iron alaun hematoxylin staining according to M. Heidenhain. At the vessel tube are parallel, partially radial, attached glial fibers. Due to the strong de-staining and contact pressure, the space at the glial border membrane or at the vessel is not visible resulting in the appearance of a direct connection between glial endfeet and the vessel tube. Hartnack 1/12 Oc. 6.

Fig. 43 from Table III. Cross-section of a blood vessel from the rabbit cortical grey matter. Alcohol fixation. The glial radial border layer is destroyed resulting in a broad shrinkage space in which only the glial endfeet and the glial border membrane remained at the vessel. This is a better location, since often the glial endfeet and its M. limitans is largely destroyed. Hartnack 1/12 Oc. 6.

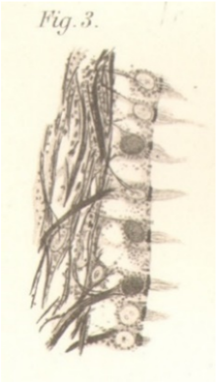

but there are also vertical and radial fibers and, in the region, where strong processes of the cortical layer protrude into the depth of white matter, they form internal converging bundles which dissolve as later described. Usually, the cortical layer is sharply demarcated against the pia mater, but it may often happen that some fibers bundles extend beyond the smooth surface of the cortical layer like hairs of a brush (Fig. 3) as was also noted by Frommann[55]." These fiber bundles correspond, according to my opinion, to parts of my border membrane which are either strongly differentiated and also remained stained.

Fig. 3 from Table I. Ependyma of the IV. Ventricle of a rabbit at the level of the nucleus facialis. Fixation with Müller´s solution with secondary osmication. On granula differential hematoxylin staining. Hartnack homogeneous immersion 1/12. Oc. 6. Except of the ependyma, two subependymal glial cells are cut. Glial fibers dark black, protoplasmic granules dark grey.

Or they represent hernia-like proliferations of the marginal glia into the pial tissue, as, for instance, observed by Schaffer or by me in spinal cord cross sections of a 25-year-old executed. In a second case of a younger executed individual, the pial bundles were partially trapped by such extensive glial swellings at the lumbar cord. Whether this was caused by careless preparation, I cannot decide. In general, however, I consider the sharp border of the cortical layer as a biased result of the method by Weigert, since, in my experience, this radial border membrane is more easily destained compared to the cortical layer. It may also be possible in the Weigert preparations that the border membrane, including the *Membrana limitans Gliae*, is pressed onto the cortical layer due to his fixation method. This is why Weigert overlooked it. In my preparations, the glial border membrane is elevated like a sail and is supported internally by the feet of the border membrane.

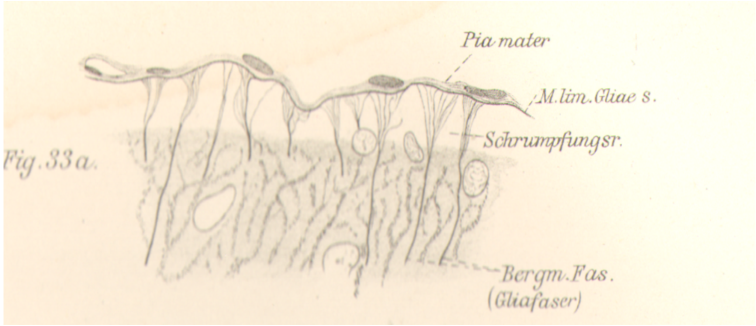

Fig. 33a, b from Table II. Surface of the cerebellum of an adult rabbit. chromium-formalin, acidic acid. Hartnack 1/12 Oc. 6. Fig. 33a. Vertical cut through the entire cerebral cortex. Fig. 33b. Plane image of the M. limitans Gliae superficialis (left) and the Intima piae (right).

In a similar fashion as in the spinal cord, one finds the boundary of the neuroglia at the brain stem and at the surface of the cortex. Only in the cerebellum, the superficial layer of glial fibers and glial cells, and thus a clear arrangement of

cortical layers, is mostly missing. Here, the superficial border membrane is connected to the glial fibers which extend from the deeper molecular zone, namely the Bergmann fibers (Fig. 33a)

and its corresponding glial cells are mainly positioned in the layer of the Purkinje cell layer. The reasons against such a border layer, as formulated by Golgi, are not valid in my opinion. It should be added that in young, a few-week-old animals, the cell layer below the border membrane still exists in a reduced form and, during the growth of the cerebellum, develops into glial cells which take part in the surface enlargement of the border membrane. In the adult human (executed at the age of 25), one only finds very few of such cells; few of them show sparse glial fibres, which proceed in a laminar fashion and move towards the border membrane, terminating with an endfoot.

In contrast to the surface of the cerebellum, the border membrane of the spinal cord and hindbrain is built more strongly and compactly due to the multiple glial endfeet and the formation of a strong cortical layer, which adds fibres from its own zone to the deeper and radially penetrating glial fibres. In addition to the marginal glia, there is something extra which is not illustrated in the images of the human spinal cord and can only be visualized by high-contrast staining. It is a net-like mass located between the glial fibres or the pure protoplasmic processes of the glial cells, resembling a broad bar or a more or less ball-shaped membrane. It can be recognized in Figs. 34a–c and 36. These images show the peculiarities of the cortical border glia. I should add that I have preparations of the cortex where the differentiation has advanced, resembling the spinal cord, and showing incomplete arrangements of these single elements.

Thus, these figures (34 and 36) demonstrate that there is a formation of multiple net bars at the marginal glia within the proper border or cortical layer (in the cortex in addition also in the adjacent deeper layer),

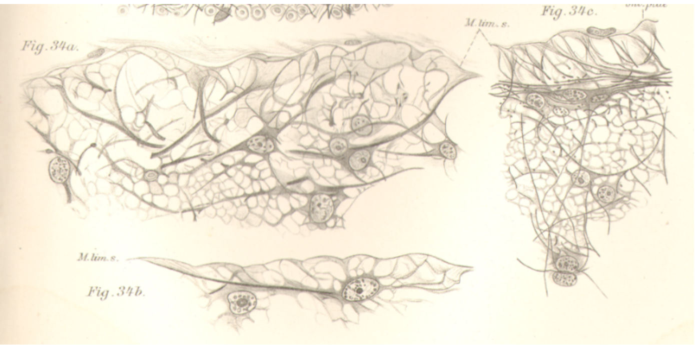

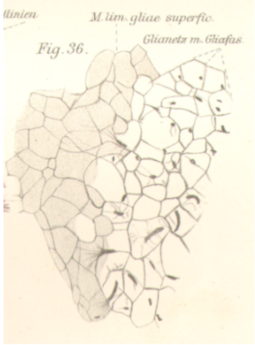

Figs. 34a-c from Table II. Vertical cut through the superficial area of the human cerebral cortex (24-year-old executed). alcohol-chloroform-acetic acid. Iron alaun hematoxylin staining according to M. Heidenhain. Hartnack 1/12 Oc. 6.

Fig. 34a. Less differentiated slice. Chamber-like structure of the marginal glia; the glial fibers appear at some locations as deeply dark enforcement bands; Fig. 34c shows the differentiation of glial fibers and their fibrillate endfeet. Fig. 34b shows the smooth isolation of a detached pia, which in contrast is closely and uniformly attached in Fig. 34c. In Fig. 34a, the Intima piae is bleb-like detached resulting in a epi-cerebral cleft. Hartnack 1/12 Oc. 6.

Fig. 36 from Table II. Skewed cut of the same preparation (Surface image of the M. lim. Gliae superficialis at the human cerebral cortex). On the left, the border membrane is cut, on the right the glial space below.

or also formation of more or less completed and stretched-out membranes along the single glial fibers and the pure glial processes. In other words, there is a chamber-type compartment below the border membrane. In its walls are multiple, laminar glial fibers extending to the border membrane, like internal supports. To what extent this peculiar arrangement has taken shape, I cannot finally conclude and, in particular, how far it extends into the depth or whether it transitions into another formation. However, I have made a series of observations on how the size and extent of the loops or chambers within the areas of the marginal glia should be considered in general. By using different fixations on series of thin slices, I concluded that it is mainly the effect of these fixations whether these loops are wide and obvious or narrow and hard to recognize.$

In addition, the same fixation has a different impact depending on the region. For example, the alcohol-chloroform-pure acetic acid mix compacts the marginal glia of the spinal cord while preserving those at the surface of the cortex in loop form. This mix results in good preservation of both tissues, while in the cerebellum it often causes the destruction of the Bergmann membranes. For the surface of the cerebellum, my chrome-formalin-pure acetic acid fixation results in good preservation and also yields good results for the cortex. Alcohol and the Müller solution yield poor results, whereas nitric acid produces better ones.

A fixation solution that results in very condensed images is the Altmann chrome-osmium fixation, which also insufficiently preserves the border membrane, as does Weigert's chromealaum(t)–potassium bichrome solution. In the spinal cord, I have observed narrow but clear images at the surface with a sublimate osmium mix. I cannot determine which width in the parts of the marginal neuroglia reflects the true condition. In particular, I cannot specify whether and to what extent local differences in tissue or uneven fluid filling within the parenchyma contribute.

I will come back to this when discussing the perivascular spaces at the end of this chapter.

The marginal glia at the depth surrounding vessels is similarly composed as those at the outer surface. In the spinal cord at the septa of the pia, may it be finer or coarser, I found everywhere both a border membrane and a glial border layer formed by foot-like attachments of multiple glial fibers. It is of perivascular nature since in these connective tissue septa are everywhere larger or smaller vessels. Only at a larger distance is at both sides a cortical layer (see Fig. 32) together forming a Frommann- ´stem process´. Similarly terminates the neuroglial tissue in the septum posterius. Depending on the varying size of this pial extension and its depth, there is an *M. limitans Gliae*, or it may be absent. This explains why the dorsal horn of the spinal cord is irregularly separated in length and depth from the border membrane. In contrast, the ventral roots of the spinal cord at its frontal cleft are divided along their entire length by a deep and broad mass of vessels.

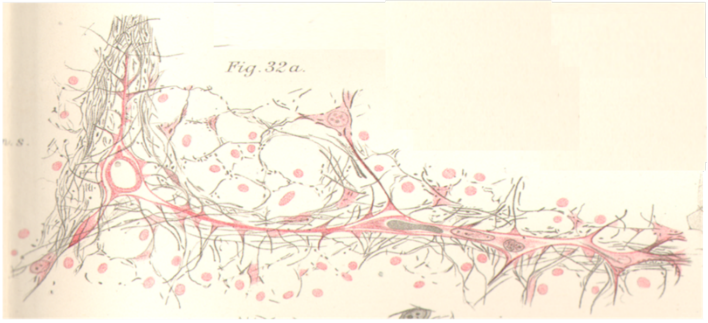

Fig. 32 from Table II. From the same slice. Boundary of a septum of the pia mater from a dorsal root which appears vessel-free.

At the depth of the frontal cleft, and partly also at the posterior septum, one can observe something special that is hard to recognize in other areas of the brain or spinal cord: at the border membrane, the feet of the processes (rich in glial fibre bundles) of the ependymal cells are attached. Figs. 1 and 2 from the spinal cord of the rabbit show the origin of these glial fibres from the ependyma. In an image of the same region of the human spinal cord (from a 25-year-old executed individual with an obliterated central canal), they originate from the fibre-rich, proliferated ependymal cells (Fig. 31).

I find weaker cortical layers at the blood vessels of the brainstem and in the white matter of the cortex and cerebellum. One finds only partially felt-like glial fibres along the large vessels extending into the depths. It is reminiscent of a similar formation along the pial septa of the spinal cord. Furthermore, I often find only a radial border layer. The coarse capillaries of the cortex exhibit such limitations.

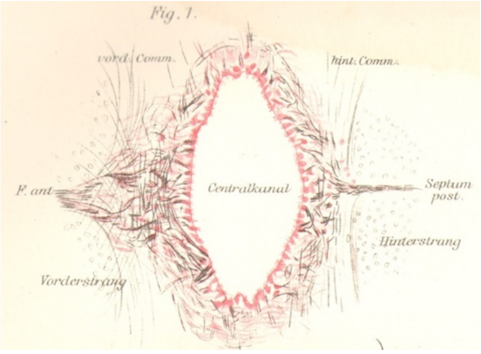

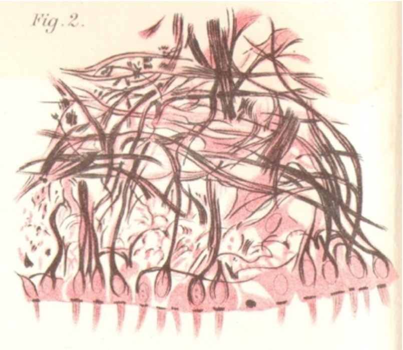

Fig. 1 from Table I. From a cross section through the lumbar spinal cord of an adult rabbit, showing the central canal and its environment. Fixation in a warmed solution of potassium bichromium and ammonium molybdanium . Differential also hematoxylin staining. Leitz Obj. 4, Oc.6.

Fig. 2 from Table I. From the same slice, ventral part of the ependyma. Hartnack homogeneous immersion 1/12. Oc. 6.

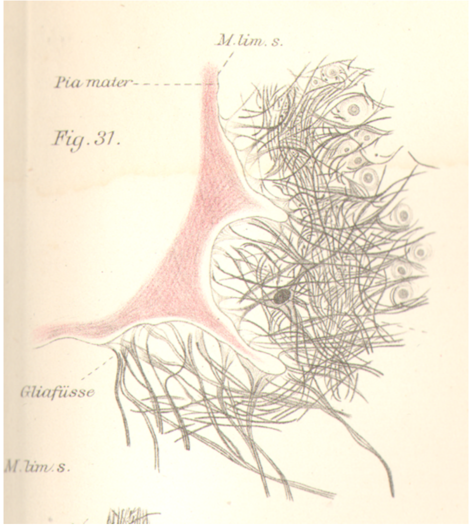

Fig. 31 from Table II. From the same slice (Dorsal surface of the human spinal cord (bend towards the septum posterior) at a cross-section of the lumbar cord (24-year-old executed)). Depth of the Fisura long. ant.

The finest capillaries are only separated by a delicate border layer, which is largely not formed by the glial fibres but by thin protoplasmic threads, the processes of glial cells, or parts of the glial reticulum (Fig. 41). In general, the radial border membrane is, therefore, a constant perivascular zone. Compared to that, a cortical layer with single glial fibres and coarse fibre tracts (Fig. 37, 32a) forms only an accessory arrangement that reinforces the border layer of the vessels with its radial bars and glial endfeet. Thus, the final connections of the neuroglia with the blood vessels of the gray or white matter are mediated by glial endfeet surrounding the vessel, which are similar to those at the superficial border layer.

Some of these endfeet are connected to radial fibres, while others are connected to fibres that run along the respective vessel and terminate, after bedding, in an endfoot. The latter is more difficult and, thus, rarely observed. In summation, all these endfeet form a special ensheathment around the blood vessel and its adventitious cover, namely the *M. lim. Perivascularis*, which, according to my observations, represents a complete border, including its adventitious sheath, between the vessel tube and the central nervous system proper. The isolation of the vessels, based on felt-like glial fibres as described by Andiezen[56] and Weigert (termed 'border membranes' by Obersteiner), does not yet reflect the true border between vessels and brain. Their description lacks the final analysis of the radial fibres, which do not exclusively bend in the longitudinal direction but form, with their glial endfeet, this special perivascular border membrane in the sense of Golgi. Otherwise, the general remark by Weigert is correct: "the blood vessels are something foreign to the nervous system, similar to the pia mater proper," and that "the border of the nervous system to the vessel represents nothing but an internal surface." Its value is particularly illustrated by the development of the vascularization of the brain mass, as described above. The investigation and illustration of the *Membrana limitans Gliae perivascularis*

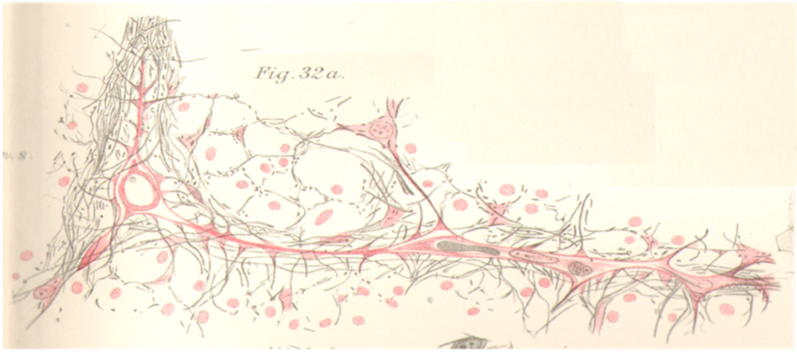

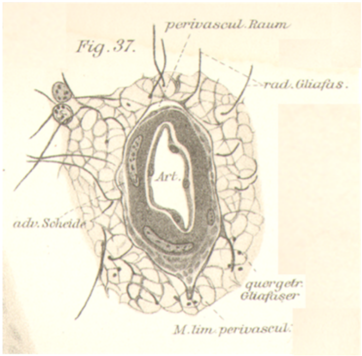

Fig. 37 from Table III. Cross-section through a small artery in the superficial glial zone of the human cerebral cortex. On top a small perivascular space is open; the adventitial sheath is tightly compressed. Hartnack 1/12 Oc. 6.

Fig. 32 from Table II. From the same slice (. Dorsal surface of the human spinal cord (bend towards the septum posterior) at a cross-section of the lumbar cord (24-year-old executed)). Boundary of a septum of the pia mater from a dorsal root which appears vessel-free.

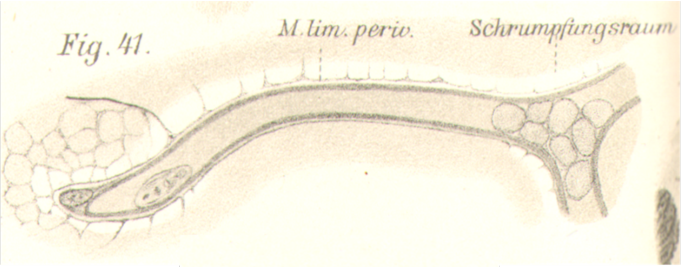

Fig. 41 from Table III. Glial border membrane of a capillary from the human cortical grey matter (24 year old-executed). On the left, the glial reticulum is partially visible. At the left end of the capillary is an adventitial nucleus which is surrounded by a small, very delicate mass of protoplasm as a partially visible sheath between the endothelial tube and the perivascular glial membrane.

is more difficult compared to the superficial border layer since it can be easily destroyed during fixation or ripped off from the glial fibers due to its delicate structure. In addition, the delayed effect of the fixation solution in the center of a piece of brain tissue results in an insufficient conservation of all the perivascular border membranes. For the human central nervous system, the alcohol-chloroformic pure acetic acid mix results in the most uniform fixation if one takes small pieces and embeds them carefully; but it is also not free from distortions which I will address later. Alcohol and the Müller solution generally result in a bad conservation. Fig. 43 shows the result of alcohol.

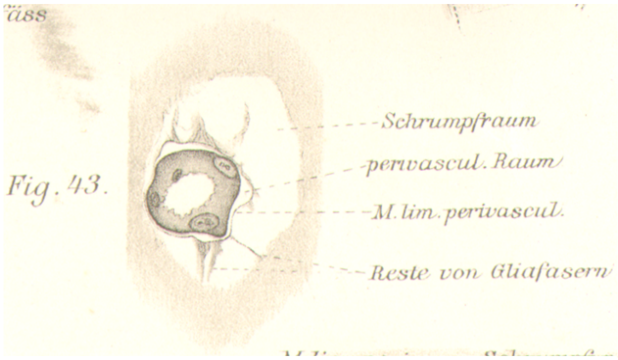

Fig.43 from Table III. Cross-section of a blood vessel from the rabbit cortical grey matter. Alcohol fixation. The glial radial border layer is destroyed resulting in a broad shrinkage space in which only the glial endfeet and the glial border membrane remained at the vessel. This is a better location, since often the glial endfeet and its M. limitans is largely destroyed. Hartnack 1/12 Oc. 6.

Müller solution and chromic acid solution which preserve at least rests of the border. By the addition of ironalaun[u] to a solution of potassium bichromic, useful for cord conservation, I obtained a solution with properties resulting in a good conservation at least for the surface and the white matter, the glial endfeet and their border membrane. Yet, it has the disadvantage of shrinking. In contrast, it is useless for the gray matter, as the shrinking in the region of the nerve cells (pericellular lymphatic space) is too significant. Unlike fixation solutions that result in limited shrinking and proper recognition of glial endfeet and their border membrane, I have also used those that do not cause any shrinking and show all elements of the white and gray matter highly condensed, revealing a better image in that respect. However, they have the disadvantage that the tissue borders of the glia are not visible, as they lack efficient labeling of the endfeet and their border membrane, resulting in significant difficulties due to the delicate structure of the latter. Therefore, I will not further address these fixations and maintain the statement that such relationships can be partially observed and that they do not destroy these important parts of the neuroglia from the beginning. After these general remarks on the different results of fixation of the radial border membrane of neuroglia,

I will address the lymphatic vessels in the interior of the central nervous system. According to one opinion, the perivascular zone of the brain contains the special and real lymphatic ways (His), while according to another opinion (Golgi, Boll, Retzius) these are spaces due to shrinking. Moreover, according to His, these spaces are not filled with fibrous formations, while others (Roth, Henle, Merkel, Gierke, Schaffer, Binswanger, and Berger) describe them as supported by a fiber meshwork and therefore draw a parallel to the lymphatic sinus with its reticular tissue. I will provide a critical reflection of these opinions, which cannot be more different. I will restrict myself to observations of the glial border membrane, its importance for development, and the fixation-dependent appearance of the radial-fibred glial border membrane. I will not discuss the results of injections into the lymphatic pathways, as I have not completed my experiments. Moreover, I have not completed the work initiated by Binswanger and Berger regarding experiments on the resorbing properties of neuroglia and the transport of insoluble material within their meshwork.

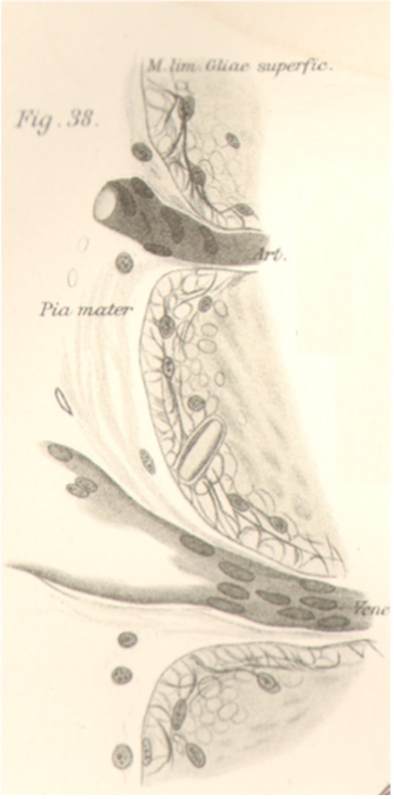

Fig. 38 from Table III. Entry of two vessels into the substance of the cerebral cortex. Transition of the superficial into the perivascular border membrane of neuroglia. From the same brain. Seitz Obj. 4 Oc. 2.

My description above on the formation of the marginal neuroglia shows that, based on the border membrane and the foot-type fixation at the tissue of the glia, one can no longer consider the clear holes or canals in which the blood vessels of the brain or spinal cord are embedded, as natural wide spaces for the circulation of the lymphatic fluid. When the space around the vessels is penetrated by radial fibers or coarse bars and networks then, in my experience, the border membrane of the glia (if preserved) is very closely pressed on the vessel or its adventitious sheath (Fig. 38, 39a, b, c, 42). If one finds no radial fibers or rest of endfeet at the wall, then the glial border membrane may not always be a widened cover of the outer wall. Due to certain fixations, the entire arrangement of radial fibers, endfeet and border membrane may be destroyed. An intermediate stage

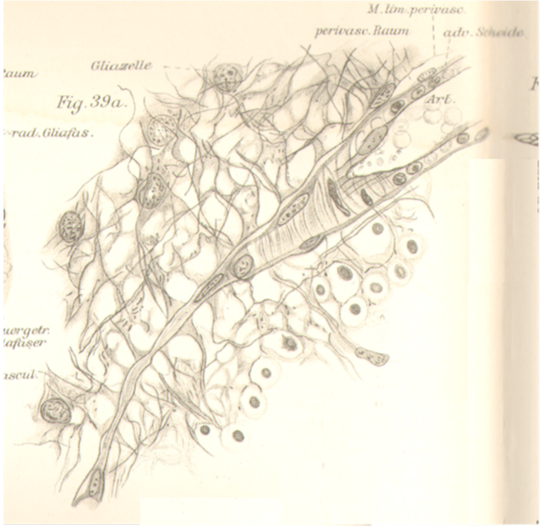

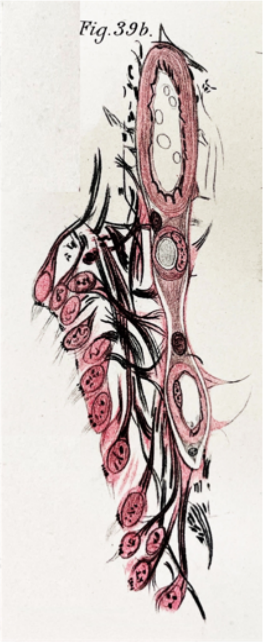

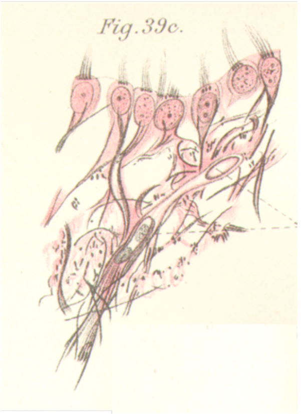

Fig. 39a-c from Table III. Glial vessel insertion in rabbit. b and c are from the Substantia Neurogliae centralis of the spinal cord, a from the IV. ventricle. Hartnack 1/12 Oc. 6.

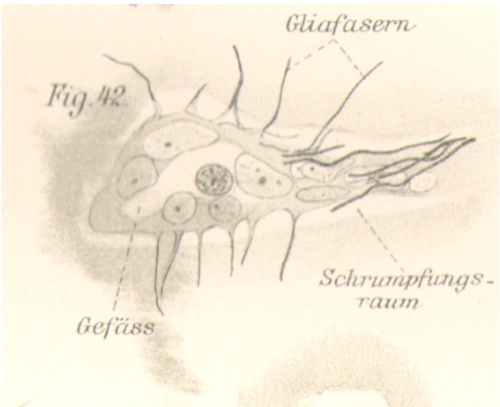

Fig. 42 from Table III. From the white matter of the human cerebral cortex. Alcohol-chloroform-acetic acid. Highly differentiated iron alaun hematoxylin staining according to M. Heidenhain. At the vessel tube are parallel, partially radial, attached glial fibers. Due to the strong de-staining and contact pressure, the space at the glial border membrane or at the vessel is not visible resulting in the appearance of a direct connection between glial endfeet and the vessel tube. Hartnack 1/12 Oc. 6.

is illustrated in Fig. 41, where after alcohol fixation (and secondary embedding in paraffin) the border membrane is pressed on the vessel, while its holding fibers are ruptured and destroyed. In any case, one can only decide by precise microscopic inspection how fixation impacted or how those zones and figures are influenced by the shrinking of blood vessels and their surrounding substance. It remains undecided whether, in the region of the marginal fluid, there may be a larger content of transudated fluid compared to deeper layers, which may be responsible for the differences in the impact of fixation fluids as discussed above. All current observations and considerations are not sufficient for this decision. One could consider artificial shrinking as a result of the artificial impact of strong compression.

I lean toward the opinion that the condensed images are more likely, as, based on my previous experience, the nervous substance contains more water and withdraws from the regions of the support tissue due to shrinking after fixation and embedding. I therefore agree with the view of Golgi, Boll, and others that the hole and canal formations, considered perivascular lymphatic spaces by Roth and Gierke, are shrinking artifacts. This is how I labeled them in my figures. I remain cautious about the importance of virtual lymphatic clefts in this context.

In any case, from those I distinguish those clefts which are in between the vessel adventitia and the *Membrana limitans Gliae* and I assume that these are fluid pathways which are filled and emptied from the adventitious vessel sheath since the border membrane of the glia prevents diffusion into the tissue of the central substance or at least restricts it.

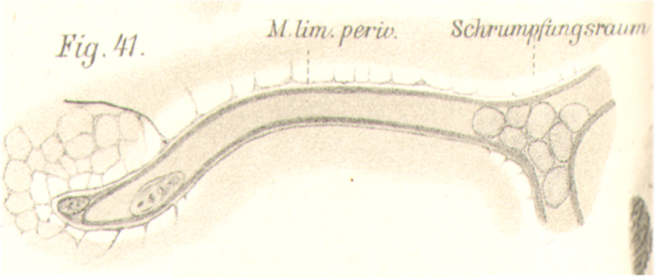

Fig. 41 from Table III. Glial border membrane of a capillary from the human cortical grey matter (24 year old-executed). On the left, the glial reticulum is partially visible. At the left end of the capillary is an adventitial nucleus which is surrounded by a small, very delicate mass of protoplasm as a partially visible sheath between the endothelial tube and the perivascular glial membrane.

I identify those cleft spaces as those defined by His which are exactly located at the border between the ectodermal brain and the mesodermal pia mater with its blood vessels and its internal branching.

I postulate further that the arguments against these perivascular or epicerebral spaces being shrinking spaces are incorrect, as they correspond to retraction holes in the illustrations by Roth, Gierke, and others. That I can identify these border spaces, as described above, as His spaces will become clearer in the following. Due to the courtesy of Councilor His, I could compare his original preparations with mine. In the slices by His, his canal system appears clear and with napless walls, and they are therefore not identical to the spaces of Roth.

One might consider that, despite these differences, it might be artificially enlarged napless retraction spaces and that the radial tissue of the marginal glia is completely deteriorated due to fixation, as I have shown above. This is particularly relevant as His[57] based his observations on material hardened by alcohol or chromic acid. However, His stated that "his clefts are always completely bounded by a napless, compressed border layer." Thus, if the above objection were true, one would expect to see fibrous remnants of glia. I have searched in vain for that in the preparations by His.

In addition, in some thin areas of the slices, which permitted me to use immersion optics, I observed a slightly detached seam bordering the corresponding perivascular canal of the brain mass, and I assume that it represents the border membrane. I cannot explain why, in contrast to my preparations, these cleft spaces appear tight.

May the cleft space positioned between the connective sheath of a blood vessel and the *Membrana limitans Gliae*, or that which as epicerebral space of His is located between the superficial glial membrane and the tissue of the pia, represent a fluid space, which is widened by variable amount of fluid, or at least anytime formed by the smooth separation

of the connective tissue vascular sheath and the border membrane? Such an objection leads to the question, whether the adjacent tissue sticks together with the border membrane, so that it cannot easily be detached, which would be necessary for generating a rapidly collapsing cleft. This would also take the unity of these spaces in question.

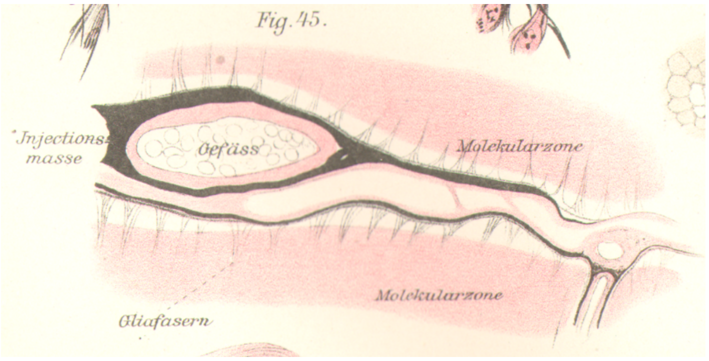

Figs. 45 from Table III. From the rabbit cerebellum. Injection into the depth. Alcohol-chloroform-acetic acid.
At the lower curved surface, the injection mass has pushed away the pia and has filled a epi-cerebral space which is separated by the border membrane with its glial endfeet from the shrinkage space. On top, the injection is also subarachnoid most likely due to the damage of the adventitial blood vessel sheath in the interior of the brain substance.

I now introduce my injection experiments on the cerebellum of a rabbit, which should determine the character of the glial border membrane (the Bergmann membrane) as a closed membrane.

By injection of a thin China ink solution into the lower surface of the *M. limitans superficialis* I have tried to determine their permeability and closeness. Certainly, the ink solution will travel some artificial course, but that is here not so important. With a Paravaz[v] syringe and a particular conic needle I have injected the ink with a very low pressure and have stopped as soon a small amount of ink solution spread on the surface. In other cases, I continued to inject and made the observation, similar than His, that, as soon as some injection mass appeared on the surface, it spreads rapidly and over a large distance. The respective pieces (of tissue) I have then cut out, fixed in alcohol-chloroform- pure acetic acid which fixates well both border membrane and glial endfeet, and also rapidly precipitates the ink deposits. By studying a series of slices, I determined the path of the injection material. In the one case it spread partially into lateral branches from the injection direction below the *M. lim. Gliae superfic*. And as shown in Fig. 45 with high magnification, it was everywhere trapped below the border membrane and along the glial endfeet of the ascending glial fibers, while it nowhere permeated the border membrane. At one location in the direction of the injection, where it was obviously exposed to higher pressure, the border membrane cracked and thereby an extensive superficial, epicerebral space between

M lim. Gliae and *pia* was filled. Fig. 46 shows an area at high magnification, which is far away from the cracked location, at the convex surface of the cerebellum; it shows the smooth injection into this space, while there was no ink in the area of the glial endfeet below the *M. limitans*. In both figures, the injection became over a short distance perivascular. While Fig. 45 shows the injection of an artificial space filled with glial fibers, Fig. 46 shows the filling of a natural space, as defined by His, which appears due to the fact that the pia mater is nowhere grown together with the *M. lim. Gliae*.

Fig. 46.

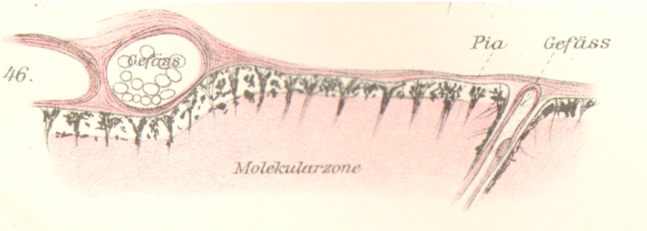

Figs. 45 and 46 from Table III. From the rabbit cerebellum. Injection into the depth. Alcohol-chloroform-acetic acid.
Fig. 46 from Table III. At a different location of the same preparation. The ink has been caught in the windings below the Membrana limitans Gliae superficialis.

If I study the situs of the pia mater and its internal continuum towards the *Membrana limitans Gliae* in slices of fixed material, I often find a smooth separation of both layers, except at locations where the pial tissue or parts of it are pressed against the border membrane. According to my experience, careful removal of the pia does not result in a lesion of the border membrane. I have obtained specimens where the border membrane of the brain, as a free line no longer covered by the pia, directly reveals the intrinsic border of the brain. I further refer to the reports by Key and Retzius[58]), page 145, stating that the pia mater, with its intima piae, can be easily separated from the spinal cord: "actually too easily so that it is very difficult to obtain cross sections of pia and spinal cord with the position remaining intact." A later remark (page 146): that the intima piae "is slightly connected with a thin layer of neuroglia, but so intense that there is no free space present (His, ependymal space)" is not consistent with that.To the following report: ´the neuroglia is attached, without modification of the tissue, at the Intima piae and the stiff fibers of the middle layer do not enter the neuroglia´, I have only to criticize that the term ´attachment´ is not correct, since, according to my opinion, the pia just loosely rests at this position. Furthermore, I express the opinion that this space must not always be wide and open,

but has the property to become open and to fill anytime under certain conditions. The mentioned pressing of the pial fibres to the border membrane is according to my opinion a fixation artefact. I would further like to cite a former remark by His that the easy isolation of ´entire vessel trees of the brain by simply puling with the forceps´ can be explained that ´the vessel adventitia have no connection´ to the walls of the perivascular canals.

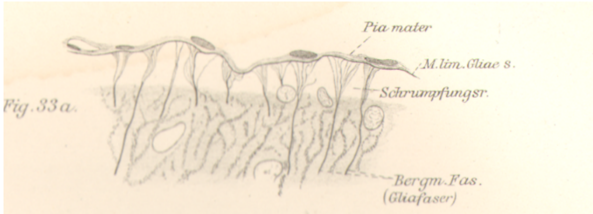

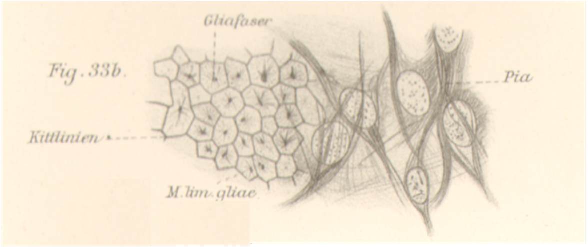

Fig. 33a, b from Table II. Surface of the cerebellum of an adult rabbit. chromium-formalin, acidic acid. Hartnack 1/12 Oc. 6.
Fig. 33b from Table II. Plane image of the M. limitans Gliae superficialis (left) and the Intima piae (right).

To all these considerations there is an additional point which is based on the structure of the Intima piae and its perivascular continuation along the internal blood vessels and their adventitious sheath.

According to Key and Retzius, the Intima piae is characterized by a peculiar thin layer, the ´membranous cell layer´ which ´contains "dispersed nuclei and around a thin protoplasmic zone which diffusely transits into the granular membrane´. Beneath it lies a fine elastic fiber net. This description is correct, in my opinion. In Fig. 33b and c [(w)], I have illustrated, in the cerebellum of a rabbit, a short section of the deepest layer of the pia and the superficial, actual border membrane of the brain, both as a flat mount and as a cross-section. I did not find cell borders in the pial membrane, in contrast to the *M. limitans Gliae*. I consider it a syncytial thin cell mass. The epicerebral space of His is, therefore, naturally limited by two well-characterized cell layers of different origin and properties, which can be smoothly separated. For the borders of the perivascular spaces, my observations are less complete, and particularly for the fine capillaries, the interesting question of the vessel boundary by a membranous cell layer as a cover remains open. It is well known that Virchow[59] first noted that there is everywhere a homogenous layer at the brains vessels which can expand and can be removed after water embedding as big sacs as found in blood. According to Virchow, this layer can transit

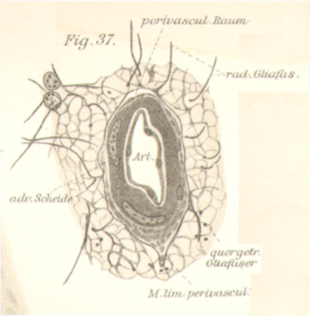

Fig. 37 from Table III. Cross-section through a small artery in the superficial glial zone of the human cerebral cortex. On top a small perivascular space is open; the adventitial sheath is tightly compressed. Hartnack 1/12 Oc. 6.

to vessels with capillary character. Independently from Virchow and from each other, Robin[60] and His[61] have described a thin and particular cover which was later known as ´Virchow-Robin space´. According to Robin this space is ´of a homogeneous or barely streaked substance).[(x)]´ In contrast to the figure in the text and Fig. 3, plate VI by Robin, the Fig. 10-13 on table XXVIII by His show an adventitious cover embedded with elongated nuclei. According to His, these cellular elements provide a clear distinction to the elongated nuclei in the capillary wall.

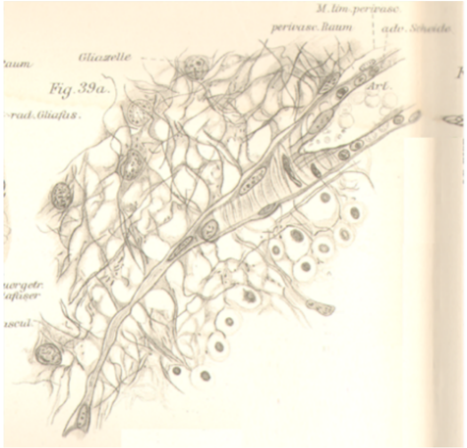

Fig. 39a-c from Table III. Glial vessel insertion in rabbit. b and c are from the Substantia Neurogliae centralis of the spinal cord, a from the IV. ventricle. Hartnack 1/12 Oc. 6.

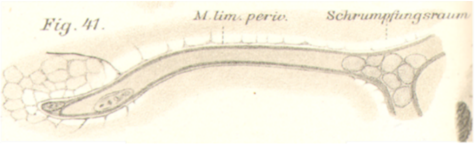

Fig. 41 from Table III. Glial border membrane of a capillary from the human cortical grey matter (24 year old-executed). On the left, the glial reticulum is partially visible. At the left end of the capillary is an adventitial nucleus which is surrounded by a small, very delicate mass of protoplasm as a partially visible sheath between the endothelial tube and the perivascular glial membrane.

My preparations of the rabbit are characterized by only a few covers of the brain made of fibre-rich connective tissue. I found that not only the thin capillaries but also the larger vessels (see Fig. 39a of an ascending artery) are embedded in a cellular cover, which is partially attached to the vessel tube and then shows a wide perivascular space. It can be tightly pressed onto the *M. limitans Gliae perivascularis* without a perivascular space, leaving only a Virchow-Robin space visible. In humans, in contrast, I found at the large vessels—for instance, in those of the ventral spinal cord or those intruding into the gray matter of the cortex (Fig. 37)—a significant mass of fibrous connective tissue in the vicinity of the actual vessel tube, with sparsely added adventitious nuclei. In concordance with His, I find at the capillaries a pure cellular cover, which shows at the fine capillaries only sparse and widely separated nuclei, which accordingly cause a concave indentation of the delicate *M. limitans Gliae* (Fig. 41). However, I can no longer state whether the corresponding protoplasm of the different and separated nuclei forms a continuous cell membrane, as could be observed at the illustrated artery of the rabbit. Additionally, the earlier statement by Eberth[62]) that the vessels of the brain and spinal cord, except for the fine capillaries, are covered by an outer perithelium composed of nuclei-containing pads leaves that question unresolved.

A mix-up with the rests of the *M. lim. Gliae*, I find unlikely since Eberth recognized nuclei in the single pads and moreover, he observed it at the middle vessel branches of the Art. fossae Sylvii which is already located completely outside the border membrane. Moreover, his other statement that nuclei-free filaments from the circum-vascular compression layer of glial fibers would insert in a broadened fashion at the perithel, is, in my opinion, due to fact that the delicate border membrane is pressed against the outer layer of the adventitia. Riedel[62a] confirmed the drawings of perithel on smallest arteries by Eberth. I cannot support his statement based on my preparations that there is a direct connection of the adventitious lymphatic sheaths of different vessels, which would refer to a true Vasa serosa. The only observation which I could add as support, are insertions of branched vessel through the *Membrana limitans Gliae*, as shown in Fig. 32a and 39b, which of course in some preparation can be lacking vessel tubes or connective tissue. I find nothing comparable, since these are not adventitious spaces, and moreover Riedel had seen these formations on isolated preparations. The same I have to say with respect to the statement of Kronthals (Neurolog. Zentralblatt 1890); I could not find his lymphatic capillaries in my preparations. With respect to the transit point of the adventitious vessel sheath into the superficial pia mater, the so-called pia-funnel, that thin membrane of the Intima piae would continue into the wall of the perivascular space, as discussed. Key and Retzius report (page 146) that `the stiff fibers of the circular layer of the Intima run similarly with the funnel and the sheath as within the pia itself´ and later ´separate from the cellular membrane and attach closer to the vessel walls, often crossing it with their branches´. This is compatible with my observations. Two questions remain open, which are of great importance for the fluid movement within the brain and its special spaces, the Robin and the His space. The first question is whether these two spaces in deeper layers of the brain, i.e. in the region of fine capillaries

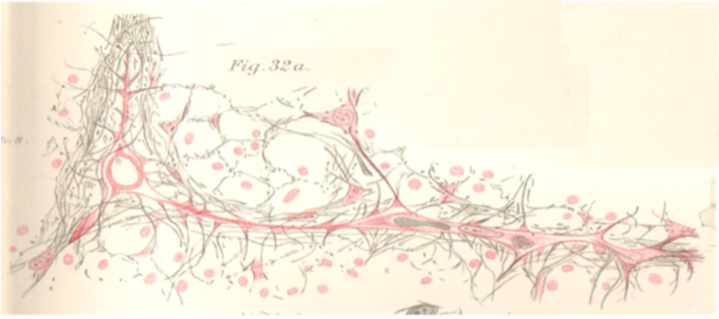

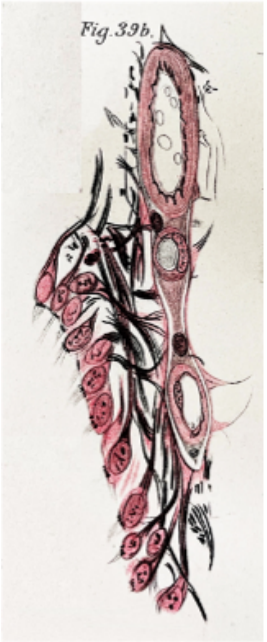

Fig. 32 from Table II. From the same slice (Dorsal surface of the human spinal cord (bend towards the septum posterior) at a cross-section of the lumbar cord (24-year-old executed)). Boundary of a septum of the pia mater from a dorsal root which appears vessel-free.

Fig. 39b from Table III. Glial vessel insertion in rabbit. b is from the Substantia Neurogliae centralis of the spinal cord, a from the IV. ventricle. Hartnack 1/12 Oc. 6.

are connected with each other or not, after following the blood vessel to this point. They would simply transit into each other as soon as it is precisely and reliably established that the membrane derived from the pia ends in the depth. The other question is whether the space between the pial membrane and the *M. limitans Gliae* is connected with the lymphatic vessels of the pia mater. His reported that the perivascular space communicates with the lymphatic paths, which has been partially disputed or supported. Golgi, Key, and Retzius, for instance, base the filling on damage to the adventitious vessel sheath at the injection site. I myself have no final conclusion. I will come back to this later. I would like to remark that, with all the difficulties in interpreting the injection results, the question of further connections of the His spaces should be resolved as soon as any gaps can be found in the pia mater, according to my definition that the His spaces are located between the Intima piae and the *M. limitans Gliae.*

At the end of this chapter, I would like to position myself to the view of Bevan Lewis[63] with respect to a particular ´lymphatic connective system` of the cortex, which is interconnected to the perivascular lymphatic sheath. According to him and Andriezen, it should consist of those glial cells which have a certain vessel process and with it should mediate fluid circulation. Such a system, promoted by Binswanger and Berger[64)], should be formed by certain glial cells which are characterized morphologically by size and being multi-nuclear. They found an increased number of such glial cells in the molecular zone of the cortex abundantly filled with fine-grained blood pigments in investigations of cortical changes in humans after an extensive subarachnoid bleeding originating from the lateral ventricles. These perivascular spaces were large, but free of blood, like the ´pericellular´ spaces.

Binswanger and Berger concluded ´that the glial cells of the molecular layer come up to the subarachnoid space with their processes and represent indeed a lymphatic connective system

in the sense of Bevan Lewis, and second that a relocation of the intra-adventitious, subarachnoid lymphatic spaces results in a pressure increase in the other – the extra- adventitious - pericellular fluid system.” Based on this three-part conclusion the explanation of the pigmentation of those glial cells in the sense of Lewis seems to be correct to me and it was substantiated by both authors by a carmine injection into the subarachnoid space of a live dog (after a certain period carmine granules were found in glial cells). Yet Binswanger and Berger could not find evidence for the delicate routes which resulted in the presence of these substances in the body of the glial cells. The issue remains unresolved whether this transport occurred exclusively in the so-called fluid cleft of the glia, between the felt of their fibers, or by an active contribution of the glial protoplasm via their feet. The latter is supported by their finding (page 538) that “single carmine granules were found in the glial processes originating from the cell.” In contrast, I hold the opinion that it is incorrect to claim that the glial cells reach the subarachnoid space or its continuation, the Robin or intra-adventitial space. I also believe that their final conclusion is incorrect, as the space they named (extra-adventitious or His space) is, in my opinion, already within the marginal glia. For this zone of the neuroglia, a mesh width independent of fixation has not been established, and their explanation of alternating pressure increases is merely an assumption that requires further experiments and investigations. The first would represent the cell membrane of the Intima piae

respective the same layer of the adventious blood vessel sheath; the second is the *Membrana limitans Gliae* as a parenchymal border of the His space. In addition, here the lymphatic connective system of Lewis in the form of glial endfeet would attach. Although I do not consider only its glial cells, but at least the entire mass of marginal glia. Yet, it remains unresolved whether glial cells exist that do not participate in the formation of the superficial or deeper border membrane. Considering the total surface of the superficial glial border membrane, the small area occupied by the glial endfeet, the richness of the central substance of the blood vessels, and their requisite number of glial endfeet, my assumption seems reasonable. Future studies must determine whether this transport of matter occurs exclusively within the protoplasm of glial cells or within their mesh spaces. Only then can one approach the question of whether the nerve cells are connected to the extra-glial lymphatic space.

Obersteiner claimed that there is a special lymphatic space for the central nerve cells, which he termed the pericellular space. He suggested that they are in direct contact with the perivascular spaces. His opposed this view, justifying it by noting that he never found a “connection between these ring-shaped courts and the perivascular spaces” in well-injected preparations. I counter this former view with my above definition of a glial border membrane. I will demonstrate below that the development of free spaces around nerve cells, as stated by Obersteiner, is based on an artifact.

4. On the net-like branched glial cells

As I have already mentioned in the previous chapter on the marginal neuroglia, the assumption of exclusively freely branched glial cells as shown by Ranvier´s

isolation method or by Golgi´s impregnation method is not correct. It is not possible; in my opinion, to explain this formation of a net-like or membranous connection of several glial cells with inserted, crossing glial fibers, with pure felting. This refers, in my opinion, only to the mass of glial fibers. The remaining mass, which represents the true form of the glial tissue, is not reflected by these two methods. However, I do not support his claim of a connection between glial cells and the core of the nerve fibers. The fact that I could observe a net-shaped formation of the marginal glia much better in the outer superficial layers, compared to the deeper areas of the central matter, is due to the complete or partial lack of nervous elements (apart from the initial impact of the pure fixation solution).

In the following, I will describe in more detail the net-shaped formation of the Glia marginalis. As I have shown in Fig. 34a-c, 36, and 37, and also in Fig. 39a, the glial cells in the border layers to the pia, or at its deeper vessel-rich extension, appear as net-shaped, branched, and partially connected elements in less-strong staining and thin slices. This net-shaped branching occurs by the protoplasm of the concerned glial cells whereupon a strange modified cell substance may play a certain role. This net-shaped mass shows further a changing sum of glial fibers with different orientation, which are partially

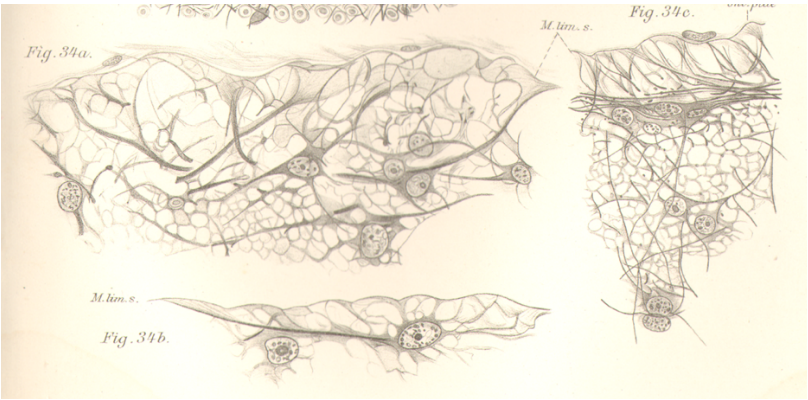

Figs. 34a-c from Table II. Vertical cut through the superficial area of the human cerebral cortex (24-year-old executed). alcohol-chloroform-acetic acid. Iron alaun hematoxylin staining according to M. Heidenhain. Hartnack 1/12 Oc. 6.
Fig. 34a from Table II. Less differentiated slice. Chamber-like structure of the marginal glia; the glial fibers appear at some locations as deeply dark enforcement bands; Fig. 34c shows the differentiation of glial fibers and their fibrillate endfeet. Fig. 34b shows the smooth isolation of a detached pia, which in contrast is closely and uniformly attached in Fig. 34c. In Fig. 34a, the Intima piae is bleb-like detached resulting in a epi-cerebral cleft. Hartnack 1/12 Oc. 6.

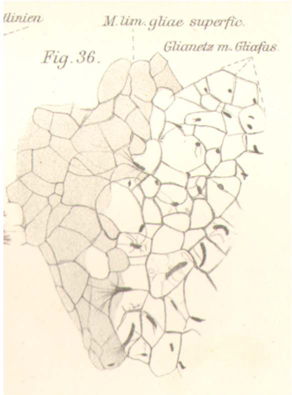

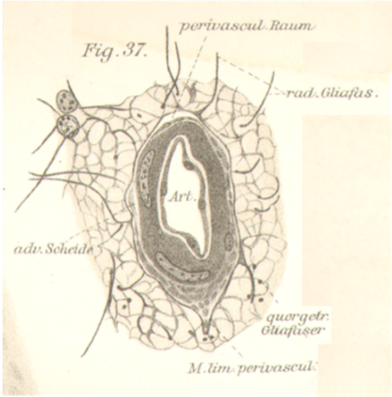

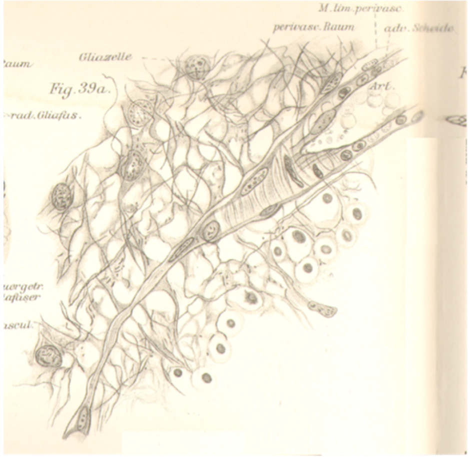

Fig. 36 from Table II. Skewed cut of the same preparation (Surface image of the M. lim. Gliae superficialis at the human cerebral cortex). On the left, the border membrane is cut, on the right the glial space below.

Fig. 37 from Table III. Cross-section through a small artery in the superficial glial zone of the human cerebral cortex. On top a small perivascular space is open; the adventitial sheath is tightly compressed. Hartnack 1/12 Oc. 6.

Fig. 39a-c from Table III. Glial vessel insertion in rabbit. b and c are from the Substantia Neurogliae centralis of the spinal cord, a from the IV. ventricle. Hartnack 1/12 Oc. 6.

based in the net bars as cross or longitudinal intensively dark labelled fibers or partially included in the protoplasm of the glial cells. If one observes this in three-dimensions by varying the focal plane using the micrometer screw[y], one finds this net as a three-dimensional net or mesh-work penetrating the respective cutting depth. Towards the surface, i.e. the connective tissue of the pia respective the adventitious sheath of the inner vessels, this meshwork is locked by a membrane as I have shown above. I conclude based on undifferentiated preparations that it can itself form mesh or hollow spaces. These spaces are partially interrupted, but partially truly closed showing this form in sections. Its crude bars, respective its thicker wall segments, correspond to the cell components otherwise termed as processes of the glial cells, as they originate due to a coarse isolation or an incomplete silver impregnation since it results in a rupture of the other parts making the neuronal networks invisible. It is also evident that a pure fiber labelling as accomplished by the Weigert stain or a differential hematoxylin staining (which I did at the subsequent control sections) will show a more or less dense felt of fibers instead of these peculiar tissue form. Certainly, one cannot speak of free neuroglial fibers as cell-independent intercellular substance since they are intracellular parts of the tissue composed of cells and cell branches of the marginal neuroglia.

Using hematoxylin staining of spinal cord, cerebellum and cortex, I found a mass which appears granular and clotting enwrapping more or less completely and often very thin the single glial fibers or connects them where they cross or run closely together (Fig. 11 and 12). This is in addition to the glial fibers which form the fibrous glial sheath of the central nerve fibers of the marrow and which run longitudinal, partially crosswise and crossing over.

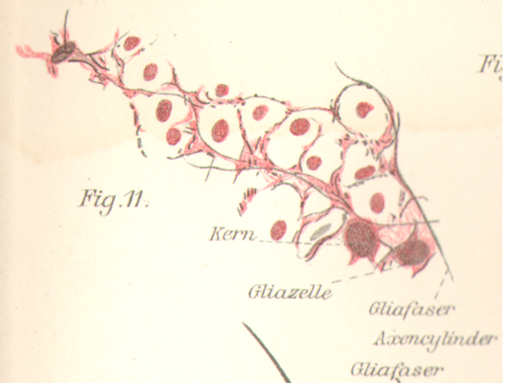

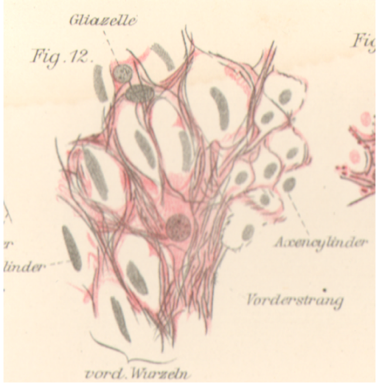

Fig. 11 from Table I. Cross-section image from the white matter of the human lower spinal cord (area of the dorsal horn). 24-year-old executed. Fixation in potassium bichromium. Differential hematoxylin staining according to M. Heidenhain. Hartnack 1/12 Oc. 6. (No net between the nerve fibers).
Fig. 12 from Table I. From the same slice; Penetration area of the ventral roots at the ventral horn.

This mass is generally very thin, outstretched, and arranged like a delicate net, with nucleus-free spaces between the glial fibers. It becomes rapidly coarser and more obvious as it approaches the compact protoplasm of the glial cells and their processes. One can observe that this substance is connected to, or originates from, the protoplasm of the glial cells.

In other words, the specially branched glial processes are arranged in a net-type structure; they accompany the glial fibers to varying extents, thereby enwrapping or connecting them (Fig. 14 and 15 from the white matter of the cortex). I would like to note that I also found such a net-shaped arrangement in the *Substantia Neurogliae centralis*, which is connected here to the processes and branches of the ependymal cells and the subependymal glial cells. To me, it seems only coarser and narrower, composed of densely granulated bars or lamellae.

Thus, the nerve fibers of the white matter are embedded in a mesh-type, net-formed tissue that accompanies them in a sheath-like manner and is partially composed of crossing glial fibers. Moreover, it is united by this intermediate mass, connected to the protoplasm of the glial cells in a net-type fashion, or more broadly in the region of the glial somata.

One could argue that the net-shaped, branched mass of glial cell protoplasm, as observed by me, is an artifact, namely a lymphatic fluid present between the glial cell spaces and condensed due to the fixation process, being deposited at the interface of the glial fibers. Against the last argument, I note that, except for fixation solutions that dissolve most of the marrow, such as the alcohol-chloroform-acidic acid mix with subsequent hardening in 96% or 100% alcohol, fixations like the Müller solution or the mix of potassium bichromate and iron alaun preserve the marrow mass while delaminating.

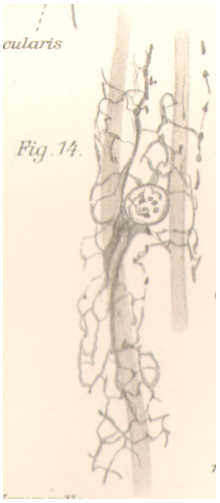

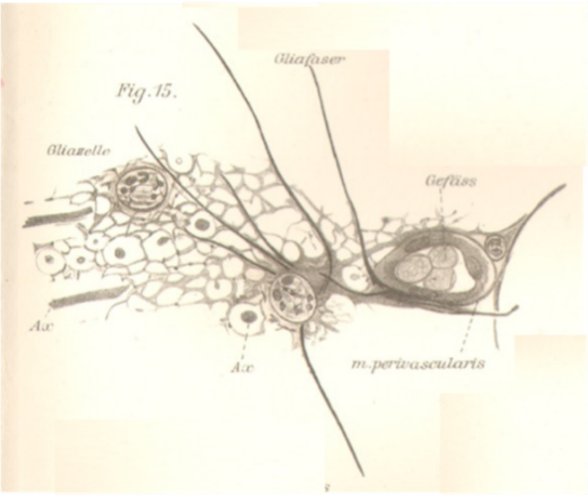

Fig. 14 from Table I. Net-shapedbranched glial cell from the white matter of the human cerebral cortex (24-year-old executed). Alcohol-chloroform, acidic acid mix. Hartnack 1/12 Oc. 8.

Fig. 15 from Table I. Net-shaped branched glial cell from the white matter of the human cerebral cortex (24-year-old executed). Alcohol-chloroform, acidic acid mix. Hartnack 1/12 Oc. 8.

Moreover, I state that this net-like substance looks different and is destained differently than those fixed or partially dissolved marrow sheaths or their remnants. Furthermore, if I refrain from stating that I have often observed a direct affiliation of a net-formed glial sheath to a glial cell, it supports my assumption, based on stainings illustrating the granular form of the glial cells, that protoplasmic granules are distributed along the glial fibers. They match the number of granules in the cell body or a thick process, even in color and size, and seem to be partially connected via a delicate, dull, destained link to those cell compartments (Fig. 4c; Fig. 3 shows this in the subependymal glia of the IV ventricle). These observations are complemented by others which I obtained on preparations using the molybdenum method by Bethe. Among the images this method delivers, which variably depict elements of the central nervous system, I have found several that are generally in accordance with my previous findings.

Thus, I have spinal cord preparations from dogs and cattle that clearly show a common and penetrating net-form of the glial protoplasm mass in a pale violet color in the *Substantia Neurogliae centralis*. At the same time, and randomly, a number of glial fibers stained dark blue originate from individual cell bodies of subependymal glial cells, extend into the bars of that network, and do not run within the interspace as expected from Weigert's doctrine of a fibrous intercellular substance. Thus, these preparations are in concordance with those I have described above.

Bethe[65] described a so called ´filled net´[z] in the central nervous system of vertebrates which reminds on the general diffuse network described by Golgi (ges. Abhandlungen 1894, page 249)[aa]. According to him this is not only present in grey matter, but also “transits into white matter and here fills, similar to Weigert´s glia, clefts between the myelin sheaths by rewinding them.”

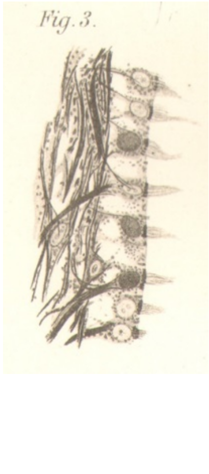

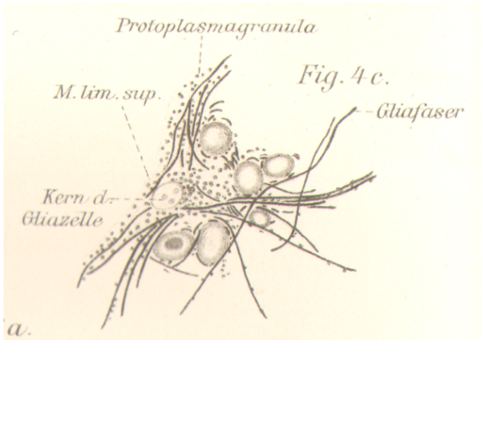

Fig. 3 from Table I. Ependyma of the IV. Ventricle of a rabbit at the level of the nucleus facialis. Fixation with Müller´s solution with secondary osmication. On granula differential hematoxylin staining. Hartnack homogeneous immersion 1/12. Oc. 6. Except of the ependyma, two subependymal glial cells are cut. Glial fibers dark black, protoplasmic granules dark grey.

Fig. 4c from Table I. Superficial glial cell from the ventral brain stem of the rabbit, which directly forms the M- lim. Gliae with its cell body. Glial fibers black, protoplasmic granula (partially ring bodies) dim grey. Fixation in Müller solution with secondary osmium treatment. For granula differentiated alsol

It furthermore has connections to the blood vessels, glial nuclei, and the pia mater. “This filled net is not related to the Weigert glial fibers; it does not consist of clotted fibers, as one can see in saturated molybdenum-labeled slices, in which the Weigert glial fibers are apparent (though not well-defined). It cannot be ruled out that the filled net is a coagulation product, but I will not draw a conclusion.”

As something particularly unrelated to the filled net, Bethe described the Golgi nets. He considered them of nervous origin, emanating from nerve endings and probably associated via the fibrils of their net bars to the neurofibrils of the nerve cells, which they enwrap. I have suggested[66] that the filled net of Bethe represents a general common glial reticulum, which shows a particular modification in two regions: at the surface of the ganglion cells of gray matter, where it forms the Golgi nets by condensation of its substance, and at the marrow segment borders of the nerve fibers, where the glial sheaths consolidate into the nodes of Ranvier.

As reasons, I reported at that time, among others, that there is a continuous connection between the bars of the filled net and those of the Golgi net, and second, that only with the Golgi net as a particular support net does an alternative net appear, which originates from the terminal areas of the neurites as a nervous pericellular terminal net. I now add further observations, which I knew earlier but did not present at that time. These observations include the direct connection of the filled net or glial reticulum to the mass of glial cells.

If one searches for further relationships in the white matter on the preparations of the Bethe molybdenum method where the filled nets are shown only pale, so it is obvious that the pale, sometimes denser granulated mass of the filled net follows strangely light and unstained routes in cross and longitudinal sections.

Those (routes) cross the direction of the nerve fibers more or less and lead obviously towards the locations of the glial nuclei, yet while the mass of the filled net is kept apart at a short distance from the glial nuclei by an even fainter substance. If one investigates preparations which are only slightly exposed to alcohol (Fig. 16, 17) or those with erythosin post alcohol staining, which reveals these fainter areas, yet not very well and elegant, so one can recognize that the filled net has a relationship to the net-shaped structure of the glial cells.

I agree with Bethe that the filled net does not reflect clotted glia fibers; yet it is related to the latter in that way that it follows its course, respective does contain it. As I have shown in Fig. 16 and 17 from the white matter of the rabbit cerebellum, the filled net is connected to several glial cells with their protoplasm being slightly stained in a longitudinal section showing the nerve fibers with glial sheaths and glial (Ranvier) nodes. At the cell body and at its radial processes, probably corresponding to the glial fibers of the cell, there is a dark stained mass which is partially composed of granules stripes or short rods and makes up in varying distribution the bars of the filled net and in tight composition finally helps forming the substance of the node (of Ranvier). The same shows Fig 17 in a cross section, while in the flat cut glial cell, an area view of the dark labelled strange mass is visible. It is net-formed distributed and includes the surface of the cell body and is sparser arranged in the net bars, respective the nodes. Those preparations demonstrate that the mass of the filled net follows the glial cells and their processes; it does not allow concluding that the net is contained in the superficial layer of the support cells respective being part of it. More obvious is such relation in preparations post-stained with erythrosine, which showed me that those dark labelled substance parts revealed with the molybdenum method are contained in the surface of the now red stained glial cells.

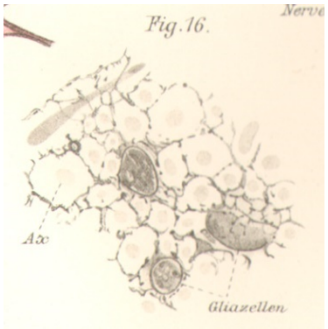

Hartnack 1/12 Oc. 8.

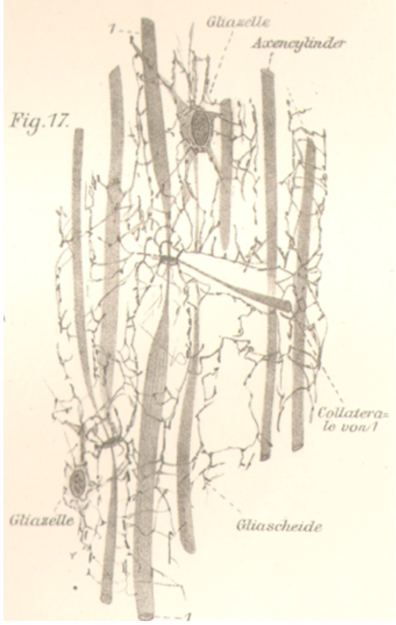

Fig. 16 from Table I. From the white matter of the rabbit cerebellum. Molybdenum method according to Bethe.

Fig. 17 from Table I. From the same slice which shows the net-shaped glial sheath and two glial nodes of Ranvier in a longitudinal section.

They form slight lateral compactions in the processes. Additionally, my hematoxylin staining resulted in images that can be compared to those, as it shows the peculiar substance of the filled net as a mass distinct from the protoplasm of the glial cell body by its darker staining and more superficial position. It continues along the glial fibers and has the distribution I described above in fiber-differentiated preparations, forming partial sheaths and respective connective bars.

Furthermore, I refer to Fig. 14, which illustrates the formation of a part of a glial sheath from a glial cell in the white matter of the human cortex. This was obtained through a different destaining method that visualizes only the general branches of the cell rather than the glial fiber contained within it. Bethe reported that his filled net inserts at various points on the blood vessels and the pia mater with its bars. As I described in Chapter 3, such relationships are characteristics of the marginal glia. Their general form is also a net formed by glial cells, which can be more or less rich in glial fibers. This defines its glial character in the sense of Weigert, who defined glial tissue by its content of glial fibers. Fig. 34-38 show the character of the marginal glia on hematoxylin-stained preparations. What I observed on the preparations using the Bethe method corresponds to their formation, except that relationships to the glial protoplasm, glial fibers, their endfeet, and the sheath-type termination by a border membrane could not be resolved.

I also obtained observations on defined glial cells in the grey matter. In the area of the ventral horn of the spinal cord, I found accompanying glial cells to the large neurons with a form and branch pattern which strongly support my previous arguments. I thus term the pericellular Golgi nets at the central nerve cells as a glial support nets. As shown in Fig. 18 and 19, it is the glial cells from which the Golgi nets originate. Fig. 19 shows a calvarium slice

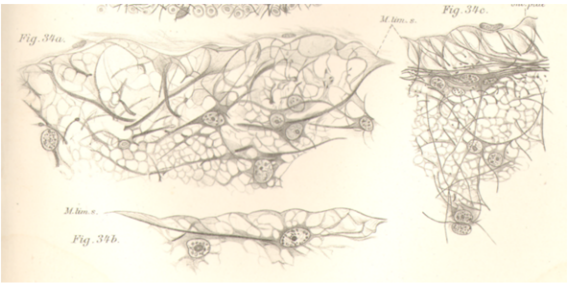

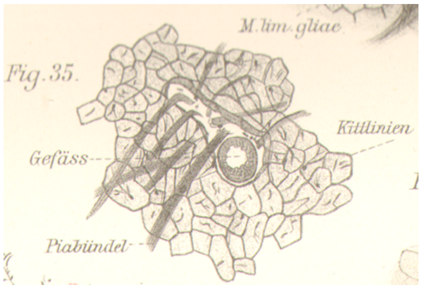

Fig. 34a from Table II. Less differentiated slice. Chamber-like structure of the marginal glia; the glial fibers appear at some locations as deeply dark enforcement bands; Fig. 34c shows the differentiation of glial fibers and their fibrillate endfeet. Fig. 34b shows the smooth isolation of a detached pia, which in contrast is closely and uniformly attached in Fig. 34c. In Fig. 34a, the Intima piae is bleb-like detached resulting in a epi-cerebral cleft. Hartnack 1/12 Oc. 6.

Fig. 35 from Table II. Surface image of the M. lim. Gliae superficialis at the human cerebral cortex. Same preparation. Entry location of a small blood vessel. Single bundles of the pia are cut.

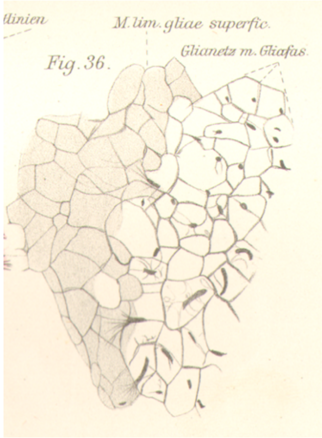

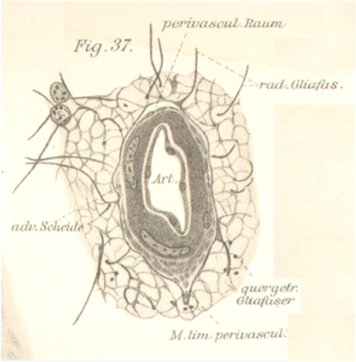

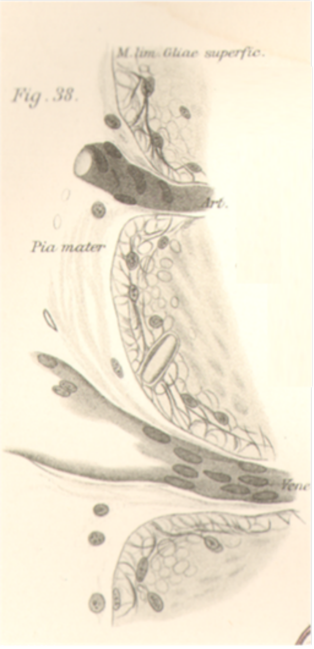

Fig. 36 from Table II. Skewed cut of the same preparation. On the left, the border membrane is cut, on the right the glial space below.

Fig. 37 from Table III. Cross-section through a small artery in the superficial glial zone of the human cerebral cortex. On top a small perivascular space is open; the adventitial sheath is tightly compressed. Hartnack 1/12 Oc. 6.

Fig. 38 from Table III. Entry of two vessels into the substance of the cerebral cortex. Transition of the superficial into the perivascular border membrane of neuroglia. From the same brain. Leitz Obj. 4 Oc. 2.

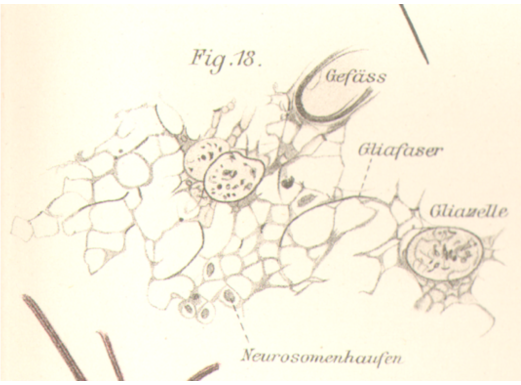

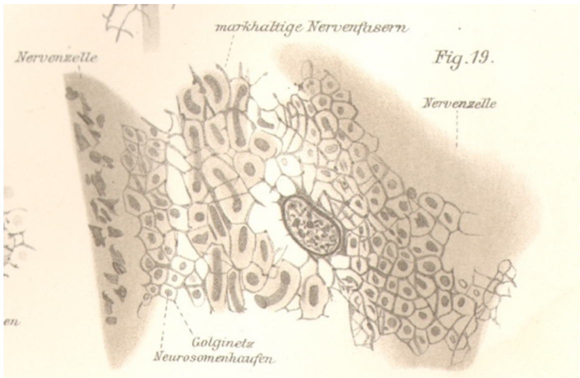

Fig. 18. From the grey matter of the frontal brain of an adult rabbit; Section through two net-shaped branched glial cells at the surface of a large frontal brain cell . Chromium-formalin , acidic acid. Differential alsol hematoxylin staining. Hartnack 1/12 Oc. 8.

Fig. 19. From the same slice

through the zone close to the surface of a nerve cells in the grey matter. The nerve cell itself is not hit; only rare single clusters of neurosomes[(bb)] of their nervous ending area indicate its presence. Yet, two glial cells are cut in the slice, which together form a network that also contains single, very fine glial fibers. The formation of that net resembles that of a Golgi net, as supported by the presence of single neurosome clusters within its mesh, an arrangement I previously observed at a nerve cell surface. However, since there is no obvious correlation to a nerve cell in this truncated net, the net in Bethe's sense can only be defined as a filled net if it incorporates an area distinct from the Golgi net, which is not shown here.

Fig. 19 shows another location of the same preparation, where two nerve cells and their associated clusters of neurosomes are visible. It clearly shows that, first, the glial cells associated with the nerve cells provide their Golgi nets, and second, these cover nets transit continuously into the diffuse net, which, as a filled net, provides the glial sheaths for the myelinated nerve fibers located between the two nerve cells. The substantial differences between the Golgi net and the filled net, as shown by the Bethe method, are not apparent here. However, this preparation shows that both are part of a more common glial reticulum formed by the glial cells.

Using the Bethe method, the correlation between the glial cells and the Golgi net or the filled net is generally not apparent, as, except for the nuclei, neither the glial protoplasm nor the glial fibers are simultaneously co-stained. The Bethe images display the nets only at some distance from the glial nuclei. I believe I can provide an explanation for this based on the methodology. This is due to the effect of an alkali on the glial tissue which is used in Bethe´s method in the form of an alcohol ammoniac solution. Such an effect

Fig. 19, see page above

I have observed during my experiments with fixations using alkali alcohol. Especially in my new experiments to differentiate the relevant nets with molybdenum hematoxylin solution, I could recognize these changes when fixation and mounting of the section was preceded by a treatment with alkali alcohol. This showed that the protoplasm surrounding the nuclei must be modified or untightened so that a subsequent staining results in freely placed nuclei. This explains why with the Bethe method, the general glial reticulum only appears in the surrounding of the nuclei and is not connected by the mass of the protoplasm. Furthermore, one can conclude that the substance of these nets, which is highlighted as dark-stained mass of glial reticulum by the Bethe method, is not simply a glial protoplasm, but a special, distinct substance. In the region of the filled net it is more loose and more broadly distributed, in the area of the Golgi net and at the nodes of Ranvier it is more compact and more dense. It is not identical with the granules occurring in the glial cell protoplasm as I have described above (see Fig. 4c), also based on morphological reasons and in addition because the staining of granules never indicates a net substance. Clear and definitive guiding principles I could not yet find for the characteristics of this mass. Thus, I come to the general conclusion that, apart of course from the formation of the protoplasmic granulation, the glial cells produce during development two distinct substances, which are also distinct in form: 1. the Weigert glial fibers and 2. a distinctly stainable substance contained within the two zones of the glial reticulum.

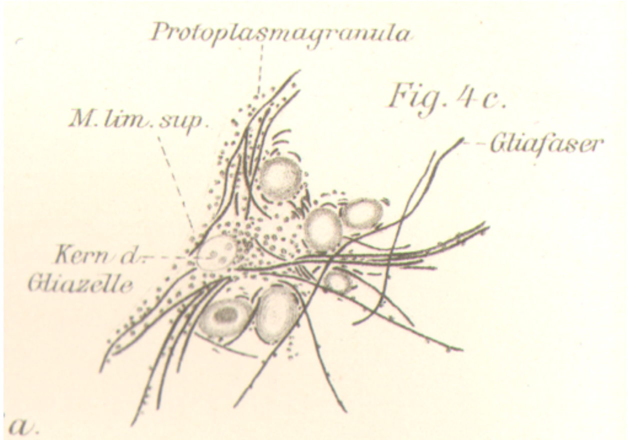

Fig. 4c from Table I. Superficial glial cell from the ventral brain stem of the rabbit, which directly forms the M- lim. Gliae with its cell body. Glial fibers black, protoplasmic granula (partially ring bodies) dim grey. Fixation in Müller solution with secondary osmium treatment. For granula differentiated alsol hematoxylin stain.

My above perception of the nature of the cells accompanying nerve cells as particularly and densely branched, net-shaped glial cells, enriched at the surface of these elements to provide a particular cover and support for the former cells

and for their pericellular nervous terminal net, inspires me to give an explanation for the generation of the spaces according to Obersteiner. I base my observations on a series of different fixations that not only demonstrate all transitions from close to wide of these putative lymphatic spaces but also prove the rupture of the net-formed branches of the glial cells. I agree with the first observations by Golgi, who considered the Obersteiner space a retraction due to improper fixation and the presumed lymphatic bodies as actually 'connective tissue bodies' (a term used by Golgi for the glial cells) of the surrounding stroma. The report by Andriezen, showing that the cells in the pericellular space have short processes, reflects severed cell forms.

Friedemann's statement certainly does not reflect reality, namely that these 'border cells' form an endothelial lining of a pericellular lymphatic space. Paladino[67] observed that this pericellular space is filled with a net-type tissue connected to the support structure of the surrounding substance. This observation agrees with my previous findings, and I think that Obersteiner's speculation (Bau der nervösen Zentralorgane, 1901, page 221), namely a 'mix-up with nerve endings,' does not resolve the issue.

At least, it is evident that the net-filled corona around the nerve cells represents somewhat normal conditions; yet, a distinction between loose or more strongly stressed net parts remains unconsidered, and one cannot determine, as with the Roth space, whether these differences are solely due to the impact of fixation or also to the uneven filling of the glial clefts with lymphatic solution.

5. Elements of Neuroglia in Invertebrates

As a short addition to the previous chapter, I describe some observations on Hirudo officinalis[(cc)]

which reveal an obvious similarity of certain cellular elements and their products with the net-shaped branched glial cells of the vertebrates and their general relationship to nerve cells and nerve fibers.

In Fig. 47a, b, c, and Fig. 48a and b, I have depicted the glial sheaths around the nerve fibers of a connective of *Hir. off.*, as seen in a hematoxylin preparation. They form a narrow grid showing a differently wide mesh depending on whether the body of the animal is stretched or contracted (Fig. 48a compared to Fig. 48b). The bars of the grid, which can be coarse or delicate, not only show a cross-over everywhere but also a true fusion of their substance.

These mesh-like glial sheaths, as shown in Fig. 47a-c, are the long-grown differentiation products of a single cell, which is located at the center of the connective. As radial septa transverse to the connective and tubes, these mesh-type glial bars develop away from the cell and are directly connected to a grid extended within their protoplasm. This intracellular grid structure is more refined around the nucleus but transitions at the level of the nucleus (47b) and the poles (47a and c) into gross bars, which then mainly extend along the connective into more uniform glial sheaths of the individual nerve fibers. It is evident that such a grid formation results in significant shear stability, which provides protection and support for the animal when it shortens.

This giant glial cell which enwraps at least the nerve fibers of a connective, corresponds to the connective spindle as defined by Apathy[68]; my description of the glial products is in accordance with Apathy. Holmgreen[69] has provided a figure (Fig. 73 on table XIV) which shows these relationships in a longitudinal section. I come back to his consideration later.

If I compare the nerve fibers of the connective with the white matter of a vertebrate, it appears that what is formed here (in the leech) by one single cell, is there (in the vertebrate) the fibrous product of a sum of smaller glial cells.

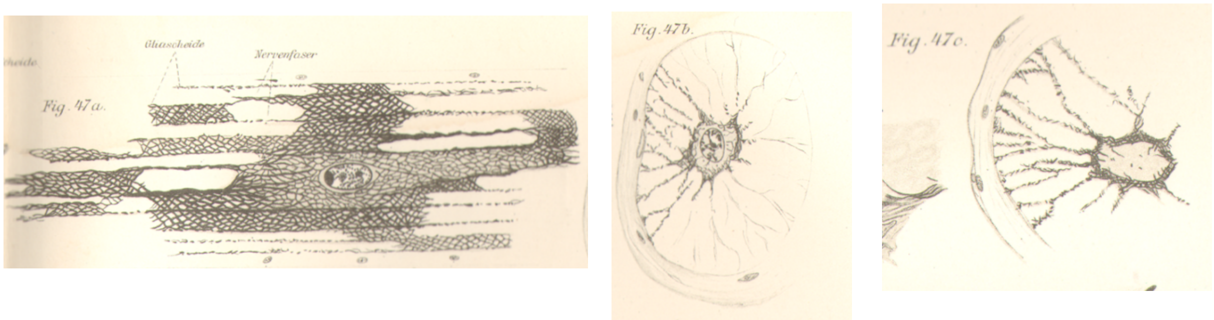

Figs. 47a-c from Table III. Longitudinal section and two cross-sections of a leech connective. Glial cell and its extension to the glial sheath. ´a´ from a stretched, ´b´and ´c´ from a contracted animal. Alcohol-chloroform-acetic acid. Alaun hematoxylin staining according to M. Heidenhain. Hartnack 1/12 Oc. 6.

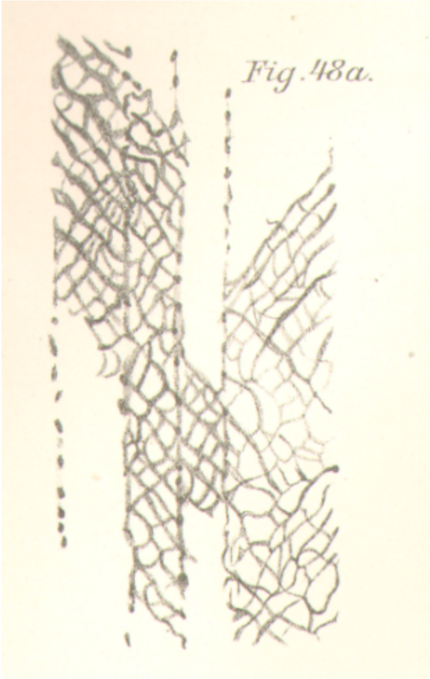

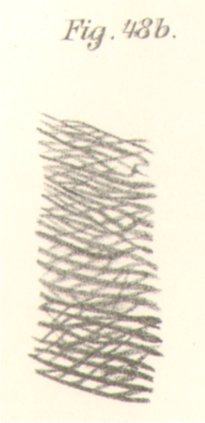

Fig. 48a, b from Table III. From the same slice; glial sheath from a stretched animal, ´b´ from a contracted.

While the glial fibers (of the vertebrates) are connected to the protoplasm of the cells, in Hirudo the beginning of the glial sheaths is included in an intracellular grid-formation enwrapped by protoplasm. I should emphasize that such a weak intracellular network in connection with its glial fibers can also be found in astrocytes of vertebrates (see Fig. 4a in rabbit and 6 in human). Otherwise, the glial fibers in the glial sheaths are not connected to one another. Their partly very obvious cross-overs (Fig. 12 in human, Fig. 5b in calf) have only an external similarity with the net-type formation, which is better compatible with the significant body changes mentioned, while those in vertebrates only need to adapt to the pressure changes of the brain and its weight.

The glial cells of the gray substance in vertebrates correspond to the cells that Apathy described as median star cells, as well as those he described as star cells of the ganglion cell clusters. Like Apathy, I consider both types to be glia-producing cells that, among other functions, form the peculiar ensheathments of the ganglion cells. My observations are almost completely compatible with those of Apathy, as I will show below.

The two median star cells of a ventral ganglion are positioned at its ventral longitudinal groove and form two giant glial cells for the central fiber mass and its attached ganglion cells, similar to those in the connective. Similar to the latter, their protoplasm is penetrated by a glial grid (Fig. 50) that becomes coarser at the surface of the entire cell (in Fig. 49, this part is exclusively stained). Highly branched glial fibers or bundles originate around the cell, penetrating the entire central fiber mass and only partially forming mesh-like glial sheaths around the nerve fibers. These appear to be related to the glial cells of the connective.

The glial fibers form at the whole surface thick bundles, which are organized in a ring-form and enwrap the processes and the ganglion cell bodies with a net-meshed and partially purely fibrous glial tissue

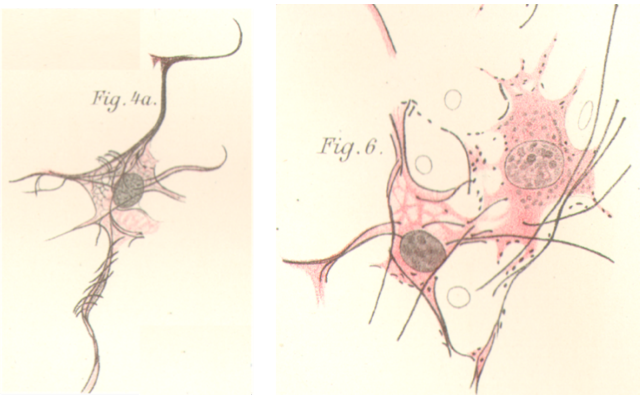

Fig. 4a and b from Table I. Glial cells at a cross section of the rabbit spinal cord (white matter of the ventral columns) Fig 4a. a spongioblastic, Fig. 4b a film-type glial cell. Hartnack 1/12, Oc. 6.

Fig. 6. Two connected cells; the lower one is partially spongioplastic and radial fibered, the upper one with a large protoplasm, granulated and cross-fibered.

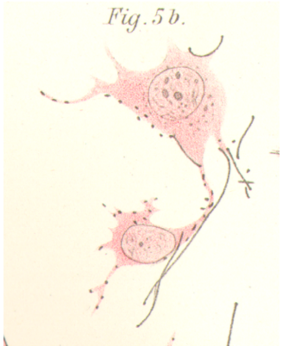

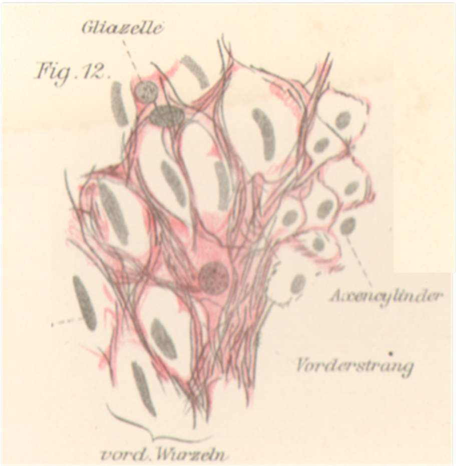

Figs. 5a-c, 6-10 from Table I. Glial cells from the human cross section of the spinal cord (Fig. 7a from a 21-year-old, the others from a 24-year old executed). Figs. 5a-c, 6, 10 from white matter. Potassium bichromium, iron alaun fixation. Hartnack 1/12 Oc. 6.
Fig. 12 from Table I. From the same slice (Cross-section image from the white matter of the human lower spinal cord (area of the dorsal horn). 24-year-old executed); Penetration area of the ventral roots at the ventral horn.

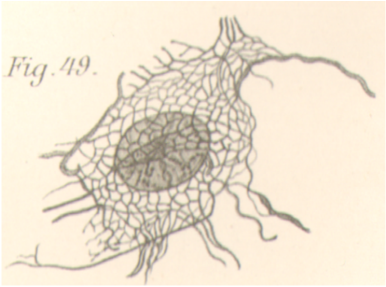

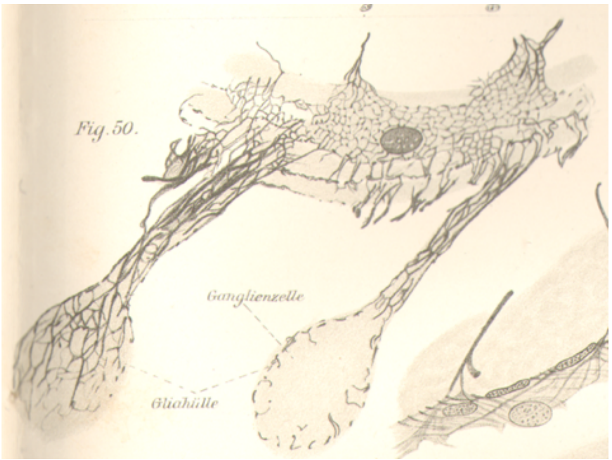

Fig. 49 from Table III. Median star cell from leech; strong differentiation, so that only the nucleus and the glial grid as an apparently independent network remains visible. Hartnack 1/12 Oc. 6.

Fig. 50 from Table III. From a longitudinal section through the ventral ganglion of a leech. Median star cell with glial extensions. The glial grid is within the cell protoplasm; its extension into the central fiber mass is not visible, but only its continuation as net-shaped sheath at ganglion cells. Hartnack 1/12 Oc. 6.

which Apathy termed the ´inner glial zone´. Based on its form and its general relationship I consider it as an analog to the supporting Golgi net of the ganglion cells in the grey matter of vertebrates (Fig. 50). While there (in the leech) a giant glial cell enwraps many ganglion cells, it is here (in vertebrates) many small glial cells which as accompanying cells provide the Golgi nets for the corresponding ganglion cells (Figs. 18 and 19). An additional difference is that the Golgi nets contain only few glial fibers and also otherwise the substance of the Golgi nets should be different.

The star cells of the ganglia packages provide an outer glial cover for the corresponding ganglion cells according to Apathy. According to him, they have a number of radial processes which consist "of a bundle of mainly very delicate, and few stronger glial fibrils." Only the stronger fibers, which are dark labelled in my preparations, which in the interior of the star cell are widely and sparsely linked, are comparable to the glial fibers of the vertebrates; the others seem to be too weak according to my opinion. In addition, they are densely occupied with granules, which appear as shining drops in the freshly studied cell. They seem to me less as supporting elements for the ganglion cells, but rather feeding ones.

Holmgreen has interpreted the nature of the radial star cells as nervous cells and as large association cells of the ganglion cells. I have to object this. The observations by Holmgreen are not much different from mine and those by Apathy. My drawings are distinct from those of Holmgreen mainly by the fact that the fibers and bars of the glial nets are more stiff and more uniform in the intracellular compartments of the cells. Particularly in Figs. 69 and 73, less in Fig. 70 of Holmgreens report are the fibers running in a wave form, sometimes thicker, sometimes thinner in its mass and more crossing over. It may be due to the type of drawing (see in this context my drawings in Figs. 47-50).

Holmgreen considers the grid-formation of the median star cells and its extension to the surface of the ganglion cells as nervous

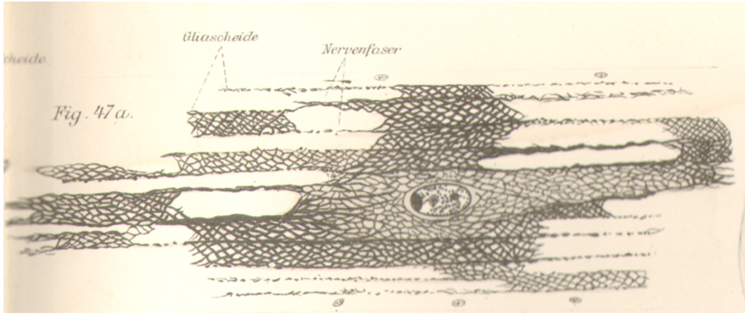

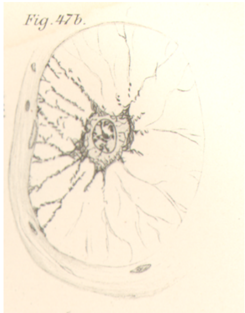

Figs. 47a-c. Longitudinal section and two cross-sections of a leech connective. Glial cell and its extension to the glial sheath. ´a´ from a stretched, ´b´and ´c´ from a contracted animal. Alcohol-chloroform-acetic acid. Alaun hematoxylin staining according to M. Heidenhain. Hartnack 1/12 Oc. 6.

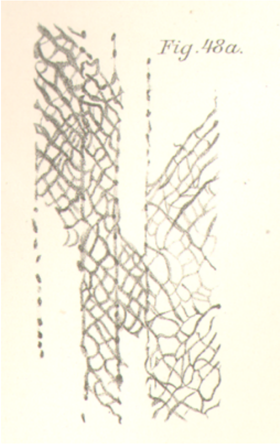

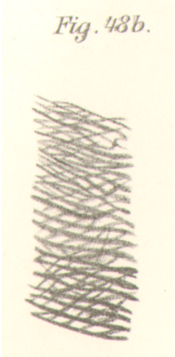

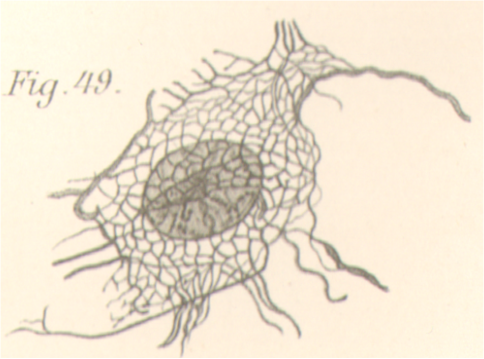

Fig. 48a, b. From the same slice; glial sheath from a stretched animal, ´b´ from a contracted.
Fig. 49. Median star cell from leech; strong differentiation, so that only the nucleus and the glial grid as an apparently independent network remains visible. Hartnack 1/12 Oc. 6.

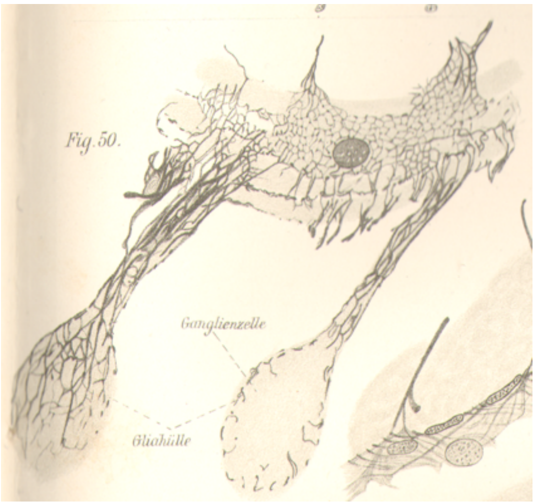

Fig. 50. From a longitudinal section through the ventral ganglion of a leech. Median star cell with glial extensions. The glial grid is within the cell protoplasm; its extension into the central fiber mass is not visible, but only its continuation as net-shaped sheath at ganglion cells. Hartnack 1/12 Oc. 6.

and identical to the Golgi nets of the vertebrates. He based this on my previous opinion, and also those of Semi Meyer and Bethe, that these pericellular nets are nervous terminal nets. Today, I no longer consider the Golgi nets as nervous nets, as I have recently, and also in this report, shown.

Holmgreen even considers the fiber grid in Apathy's connective spindles identical with the one at the surface of the unipolar ganglion cells and those median star cells which he describes as ´multipolar nerve cells´, while he considers only the Leydig cells (side nuclei of Apathy), which I disregard here, and the star cells of the ganglion cell packages as large glial cells.

In opposition to Holmgreen, I report that the features of the superficial extension of the grid, which initially forms within the intracellular compartment of the respective giant cells, determine its nature. This grid forms superficial sheaths around the nerves of a connective, sends separating fiber bundles, or partially forms grid-shaped covers between and around the nerves of the central fiber mass, and additionally enwraps those ganglion cells, including their processes, with a net of fibers. This is all indicative of an external support structure and a separating, coherent sheath structure, but not of a nervous element.

The comparison with Golgi nets is not relevant here, as these are described as support elements by me and Apathy in the sense of Golgi, while I emphasize the above observations regarding the cells forming the Golgi nets as glial accompanying cells. I cannot accept Holmgreen's reference to his methylene blue staining, as this method stains all kinds of structures, including non-nervous ones. I also find the description and images of Simon[70] inconsistent with the grid structures discussed here. One point remains that has concerned me for quite some time and is known to me from the corresponding work and views of Holmgreen.

It refers to the radial insertion of the fibrous parts of the inner glial zone into the protoplasm of the enwrapped ganglion cell, which, according to Apathy, takes part in the formation of the outer alveolar zone of the ganglion cell body. Holmgreen states that, from this superficial net, “intracellular fibers oriented in radial direction” are very probably connected to a deeper and perinuclear fiber ring. According to Apathy, this fiber ring forms part of the endocellular neurofibril grid (the “perinuclear grid”) in the type K ganglion cells.

The assumption of a continuity between the superficial net and the perinuclear grid of Apathy, with its extending thick and axial primitive fibril, which Holmgreen observed as stained together, was the basis for him to consider this superficial net as nervous and not glial. He, therefore, also considered the median star cells as giant associative cells. Furthermore, Holmgreen admits that he was not sure about that connection; moreover, his images do not show this continuity (Fig. 71).

I myself obtained such preparations from *Hirudo* as early as 1896 using the same method (fixation in alcohol, chloroform, acidic acid, and iron hematoxylin, according to M. Heidenhain) that Holmgreen described and depicted in his work from 1900. My earlier staining revealed comprehensive labeling of the glia in leeches and their insertion into the ganglion cells. I also obtained co-labeling of fibrils in the interior of the ganglion cells with nerve fibers corresponding to the images of Apathy, which seemed to me to be linked. Based on that, I found it justified to accuse Apathy of having confused his neurofibrils with glial fibers.

Today, this relationship seems less clear to me, as in differently stained slices, the glial zone can be destained while the neurofibrils remain stained. Only in preparations with simultaneous staining of both components do I still find it difficult to distinguish between the two.

However, since today I cannot be absolutely sure whether these two components merge, I can no longer support my accusation and therefore withdraw it. I can only discuss the possibility of a relationship that would be accompanied by a change in the substance. It should be considered that some of the giant cells, not only in adults, contain passing neurofibrils apart from the glial fibers, but they are also responsible for the formation of neurofibrils according to Apathy. I return to the analogy of the glial superficial net at the ganglion cell of the invertebrate and the Golgi net around the corresponding cell in grey matter. According to Apathy, the ganglion cells in the spinal cord of vertebrates "do not have an internal glial zone corresponding to the one in leech, but are rather embedded in a dense, interstitial glial network." I note that my comparison is based on the net shape of the corresponding cover, which, in vertebrate ganglion cells, is formed by special accompanying cells as part of the general interstitial glial tissue.

Moreover, the mass and internal form are, of course, different, since in *Hirudo*, it is coarser and more grossly fibered, appearing as a more differentiated protoplasmic product of the median star cells. The substance of the Golgi nets being a particular formation of those glial cells is evident from the fourth chapter of this publication and my previous reports. Additionally, these cells participate, during their general net formation, in the generation of neighboring parts of the general glial reticulum of the nerve fibers. Thus, on a small scale, they are similar to the median star cells, except that the latter provide, with their coarse and grossly fibered protoplasmic products, the glial bars of the central fiber mass.

Bethe reported that the Golgi nets are associated with the internal fibrils of the ganglion cells. I cannot decide whether this is the case, similar to the conditions in Hirudo. Based on the arguments which I brought forward for the glial nature of the Golgi nets, I cannot agree with the doctrine of Bethe that the Golgi nets are a common formation with the branched neurites and that they mediate a continuity of the neurofibrils of vertebrates.

6. Formation of Neuroglia and its Significance

As formation of neuroglia, I consider the general and interdependent relationship of the neuroglial cells among each other. The question arises whether the processes of the neuroglial cells are interconnected among each other or only cross over, be they fiber containing or fiber poor or consisting only of nude, partially protoplasmic covered glial fibers.
It is remarkable that the views on that question have recently changed to the opposite. Von Kölliker earlier postulated a general net-type connection of the glial cells and considered it as a general reticulum of the central nervous system (Handbuch der Gewebelehre, 1867, page 266) that penetrates like a delicate skeleton the entire white and grey matter. While Fromman(n) and Gierke supported hat view, Deiters[72)] and Golgi and later Ranvier and Weigert objected against an anastomosis of glial cells resulting in a dominance of the doctrine of neuroglia felt. I refer in this context to the statement of von Kölliker in the 6th edition of his ´Gewebelehre´ (1896), page 151, describing the insertions of the nerve fibers of the white matter into the deceptive meshes of the glial net. Also, Erik Müller reports in the sense of Deiters and Golgi that in lower vertebrates the processes of neuroglial cells only cross each other over or only attach to the cell body of other glial cells in ridge-like elevations or grooves.

In the studies of the formation of the glial tissue, one has to distinguish between the fibrous glia and the reminder protoplasmic mass of the neuroglial cells distributed on their processes.

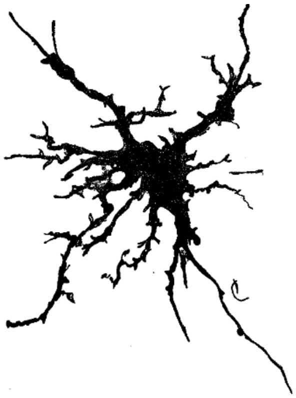

Fig. 20b from Text Figures. Glial cell from the spinal cord of a few week-old dog. Silver staining according to Golgi. Hartnack 1/12 Oc. 6.[ee)]

I agree with Weigert that the glial fibers "are more or less straight" or run in "stiffly bend curves", and are solid and "completely smooth" in freshly fixed preparations. According to my opinion that is evident in certain Golgi preparations, where the so called processes are smoothly impregnated[(dd)] as shown in Fig. 20c for a ´Langstrahler[(ee)]´. I explain this form due to the fact that the impregnation transits from the cell body to the ´collar of the fiber´ (Ranvier) and subsequently to the glial fiber. Due to the length of the glial fibers, the type of Langstrahler is defined. Andriezen demonstrated that under certain conditions only the glial processes of a cell can be impregnated. Then I know a form of impregnation which shows both mossy and rough process areas (Fig. 20b). In my opinion, here the impregnation simultaneously visualized part of the incomplete protoplasmic cover of the glial fibers, since I can observe in my stained preparations a similar roughness in the protoplasm labelling of the cell body as attached corners of the black stained glial fibers. It is an established observations that the glial cells of embryonic or juvenile brain are often roughly impregnated with the Golgi method, while in the adult they are smoother. This may be due to the increase in glial fibers of a cell with aging and a reduction of the of the originally broad protoplasmic network and an easier transition from the cell body to the mass of the glial fibers.

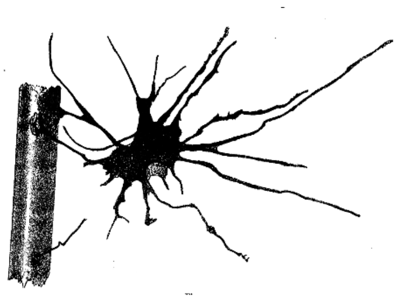

Fig. 20c from Text Figures. Glial cell from the spinal cord of a few week-old dog. Silver staining according to Golgi. Hartnack 1/12 Oc. 6.[ee)]

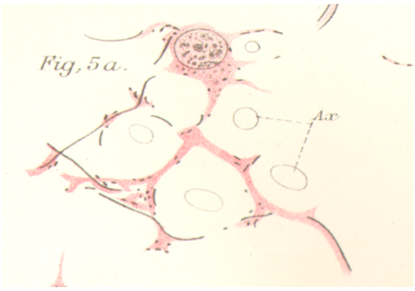

Fig. 5a from Table I. cross-fibered glial cell; on the following slices the process cut below is connected to a glial cell.

With respect to the question of a division of glial fibers, I support Weigert's view who has never seen such a division. I would, however, remark that apparently uniform fibers could be composed out of very

delicate, highly condensed individual fibers. At certain locations they could divide into diverging small fibers (I refer to the form of radial glial endfeet). Weigert´s explanation with respect to divisions of glial processes in silver preparations (namely that two closely attached fiber parts melt to a common silhouette due to a co-labelling of a sticky substance, as Ranvier considered) is only partially true. I consider this sticky substance as the protoplasmic cover of the fibers as often observed in my preparations. Moreover, there are dividing, fiber-free protoplasmic processes at glial cells (see fig. 5a).

There is another important issue with respect to the glial fibers recently reported by Hardesty and also observed several times by me: Many glial fibers reach from one cell to the next. This would therefore be an anastomosis of two glial cells by a common glial fiber. This is not surprising considering the development of the glial fibers in the protoplasmic anastomoses of embryonic glial cells as I have reported.

Certainly, a pluricellular glial fiber can only be regarded as such if a protoplasmic connection between both cells can be recognized in the section. I do not deny that there is a large number of glial fibers that only cross each other and are attached only to other glial cell bodies. Thus, in mature glia, I distinguish intrinsic and foreign (attached) glial fibers, which, however, is only a distinction based on slices.

In fairly mature and developed glia (since the fiber content in humans increases with age, it will always be considered fairly mature), there are parts beyond the fibers that cannot be neglected. I refer less to the association of glial cells by their processes and glial endfeet in the border membrane of the glia and more to the net-shaped intermediate mass, which originates from the process-like or laminar protoplasmic extensions of the glial cells and partially embeds the glial fibers or only segments of them in a net-like manner. I can postulate this with certainty for the marginal glia, the subependymal region, and the accompanying or covering cells of the nerve cells.

With this, I refer to a finding by J. Hardesty, who describes glia as a syncytium of ectodermal nature. I would like to add that I independently arrived at the same opinion based on my observations. For the remaining considerable mass of diffuse glia, I cannot make that statement. However, regarding the general concept of neuroglia as a syncytial tissue, I refer to my previous arguments regarding its development.

Hardesty relates his glial definition to the inner form of the ´Randschleier´ (border haze), defined by His, in which a separation of single ependymal cell parts is neither possible nor present. I have also become familiar with this definition during my studies on marginal neuroglia.

In addition to the pronounced syncytial form of the ependymal glia at the border haze, the secondary cellular glia is also built up, based on mitotic processes, out of cellular processes forming a consistent, connected material (Fig. 28a). The secondary cellular glia does not remain at this simple stage. As shown in Fig. 28b at the optic nerve of a newborn mouse, the glial cells have developed more processes, which partially do not anastomose, but show cross-over of their new processes. This formation will further increase at later stages (Fig. 28c). A similar arrangement, I find in the white matter of the spinal cord. Whether these cross-overs of processes at this developmental stage should be considered lower due to the cut of stretched anastomoses of distant cells, I cannot decide.

The mitotic proliferation of the secondary cellular glia out of its own pool could explain some peculiarities of the adult support substance of the central substance. This includes 1) the multinuclearity of glial cells (see Figs. 5b, 9, 18), 2) broad, skin-like bridges between neighboring cells (Figs. 6, 7), 3) thinner and more rare anastomoses which can be purely protoplasmic or partially fiber-containing (Figs. 5b, c), 4) the already mentioned pluricellular neuroglial fibers.

If I compare the early stage of the secondary cell-fibrous glia (a stage at which the glial fibers can already be recognized as dark-labelled, homogeneous and intracellular thread formations in the processes and at the rim of the cell body or in its depth) with the adult tissue, so it is obvious that a protoplasmic reduction has occurred so that a continuous cover of the fibers can no longer be recognized. In contrast, in the hypertrophic glia there seems to be a large increase in protoplasm, now including those glial fibers to a larger extent. I show this in Fig. 13 in the white matter of spinal cord of a man, which shows an increase in glial fibers, a proliferation of the ependymal, and many monster glial cells in the grey matter with superficially differentiated cell plates and thick glial fiber formations, as well as a considerably thickened superficial cortical layer.

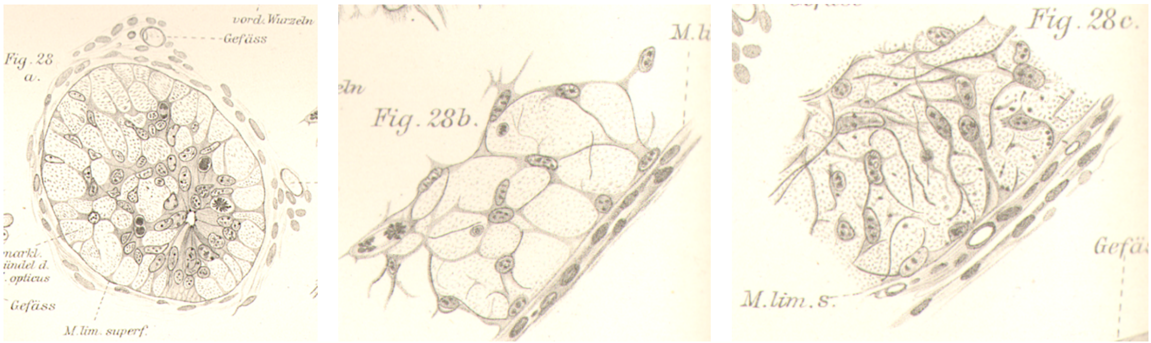

Figs. 28a-c from Table II. Three developmental stages of the Nervus opticus of the mouse in cross-section. Fixation in chromium-formalin , acidic acid. Also hematoxylin staining. Hartnack 1/12 Oc. 4.

Fig. 28a from Table II. from a 12-day old embryo, Fig. 28b from a new-born, Fig. 28c from a 5-day old mouse.

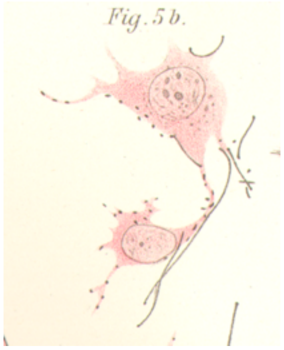

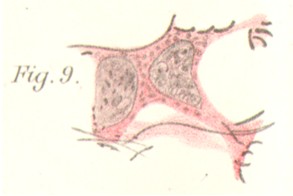

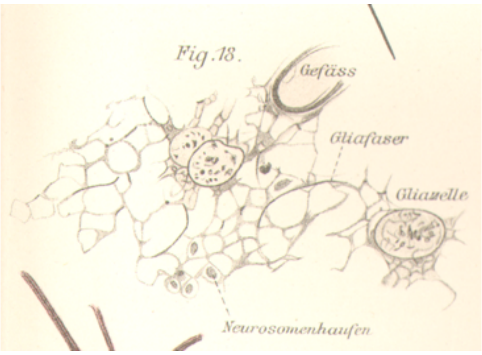

Fig. 5b from Table I. The upper glial cells is a form with few fibers and a large and granulated cell body. Fig. 5c. Two glial cells with glial fibers mainly in the processes.
Fig. 9 from Table I. Glial cell with two nuclei which is strongly granulated.
Fig. 18 from Table I. From the grey matter of the frontal brain of an adult rabbit; Section through two net-shaped branched glial cells at the surface of a large frontal brain cell . Chromium-formalin , acidic acid. Differential alsol hematoxylin staining. Hartnack 1/12 Oc. 8.

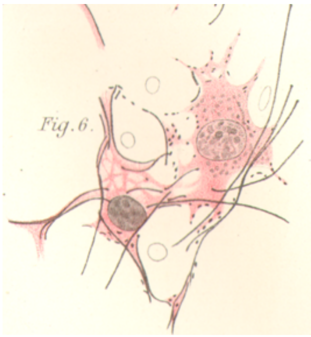

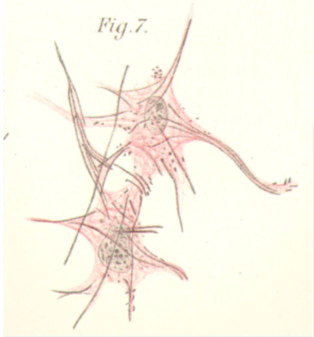

Fig. 6 from Table I. Two connected cells; the lower one is partially spongioplastic and radial fibered, the upper one with a large protoplasm, granulated and cross-fibered.
Fig. 7 from Table I. Two connected radial-fibered glial cells; both spongioplastic.

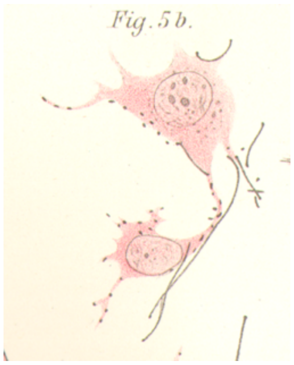

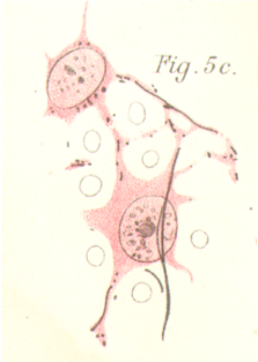

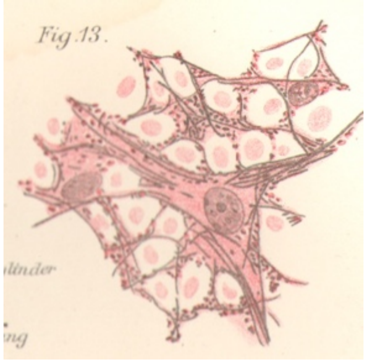

Fig. 5a from Table I. cross-fibered glial cell; on the following slices the process cut below is connected to a glial cell. Fig. 5b. The upper glial cells is a form with few fibers and a large and granulated cell body. Fig. 5c. Two glial cells with glial fibers mainly in the processes.
Fig. 13 from Table I. From the lumbal cord of human; Cross-section from the ventral cord. Broader protoplasm mass between the glial fibers. Hartnack 1/12 Oc. 6.

I refer here to the statement of Storch[73)] that upon a "reduction in live energy within the nervous compartments the protoplasmic compartment" around the glial nuclei increases and "covers the fibers with in a webbing fashion, possibly up to their endings."

I now conclude that the important issues for the definition of glial tissue are that (1) embryonic glial cells form the glial fibers intracellularly and (2) the glial fibers later probably emerge from the collar-type protoplasm or its process in a free fashion but could also re-enter other processes or cell bodies and cross over in their course. The glial fibers are by no means a cell-independent intercellular substance. Based on the delicately arranged and modified branching of the glial cell protoplasm, the processes of the protoplasm are not simply felted but are arranged as a delicately branched, net-type tissue.

The branches should not be confused with an earlier and coarser conception of a reticulum, which, for example, enwraps the individual nerve fibers of the white matter with true meshes. What unites the glial cell as a syncytium are partly their protoplasmic parts, which, in their finest branching, contain portions of Bethe's filled net or my glial reticulum. It also partly includes those glial fibers which, either partially naked or enwrapped by protoplasm, insert into the cells and their processes. It also includes the combination of fiber-containing and fiber-free glial endfeet with the *Membrana limitans Gliae superficialis et perivascularis,* in which the skin-like foot areas of the final glial appendages are connected through putty lines.

The importance of the glial cells is generally considered as to provide a profound support substance of the nervous system, a special connective tissue for nerve cells and nerve fibers. To this justified view R. y Cajal[74] added an insufficient one, which states that the neuroglia is contractile and that it gives, with their different stages of relaxation or expansion, uneven conditions for the nervous contacts.

This is because the neuroglia can insert itself as a current-isolating mass between the dendrites, pulling them apart. During contraction, it results in an enlargement of the vessels, influenced by the glial attachments to the vessel tubes. In addition to the elaborations by Weigert in his neuroglia publication (1895) and by von Kölliker (*Handbuch der Gewebelehre,* 1896 II), I would like to add that this hypothesis collapses because it is an unproven assumption that free contacts approach or disconnect. It is based on biased results from the staining methods of Golgi and Ehrlich, and there are many arguments against it that were not considered by R. y Cajal in his refutation.

It thus remains defined that the neuroglia represents a supporting intermediate substance for the nerve cells and their processes. In particular, due to their later-developing fiber content, they are suited to support the weight of the nerve cells, their processes, and the vessels. Weigert's merit lies in recognizing the arrangement of the glial fibers as architectonic networks at both the inner and outer surfaces of the nervous system, based on his method. Weigert also recognized the role of glia in providing a space-filling function through expansion at sites of neuronal degeneration.

It remains undecided whether the expansion and growth of glia is a secondary event following nerve cell damage, or whether their activity is primary, resulting in the suffocation of nervous substance.

The nature of the glia in the nervous system is based on their syncytial composition out of multiple core centers, its richness of protoplasmic components, its property of extensive fiber formation and its particular and extensive connections to the vessels with their glial endfeet.

*) The counterarguments of R. Y Cajal are in: Algo sobre la signification fisiologica de la Neuroglia. Rev. trimestral micrograph. II. I.

1897

I consider Golgi's discovery that glial cells attach to brain vessels as one of the most important for understanding the finer details of brain structure, particularly regarding the nourishment of nerve cells. This led him to propose that the protoplasmic extensions of nerve cells, in contrast to neurites, have only a feeding function as they connect to glial processes. It is evident that Golgi's statement already incorporates Bevan Lewis's concept, which claims that glia forms a lymphatic connective system. However, Golgi remains more critical, considering the "pericellular lymphatic spaces" coined by Obersteiner as retraction gaps, whereas they play an important role for Bevan Lewis (see Table V in his textbook).

Golgi's concept of the sole nourishing function of dendrites is now generally considered incorrect. However, focusing Golgi's claim on the connection between glial cells and the protoplasmic extensions of nerve cells, one can define these local sections of glia as a link between the vessel system and nervous protoplasm. This is evident as glial accompanying cells (see above and Figs. 18 and 19) confine the capillaries at the perivascular space and, on the other hand, enwrap the protoplasm of nerve cell bodies and dendrites in a net-like manner.

Additionally, as Holmgreen demonstrated, the protoplasm of central nerve cells is not only surrounded by glial cells but also internally penetrated, intermingled, and fissured by a "trophospongium." Thus, not only are the protoplasmic extensions of nerve cells relevant for feeding the rest of the nerve cell, but so is the glia, which determines lymphatic spaces with its marginal side while contacting the free areas of nerve cell protoplasm. Therefore, it can be considered a unique feeder of nervous substance in addition to its role as a support structure. Regarding this primary function of glial cells (nutrition), I would like to mention the studies by Wlassak[75], who attributes primary and secondary glia with importance as storage and transfer structures, providing substances for the myelin cover from the blood.

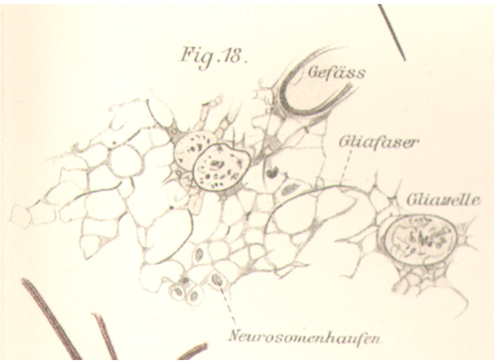

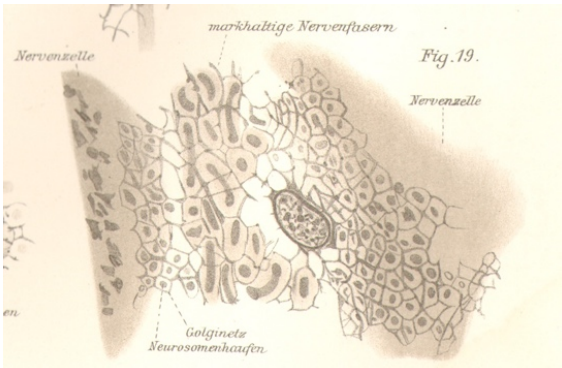

Fig. 18 from Table I. From the grey matter of the frontal brain of an adult rabbit; Section through two net-shaped branched glial cells at the surface of a large frontal brain cell . Chromium-formalin , acidic acid. Differential alsol hematoxylin staining. Hartnack 1/12 Oc. 8.

Fig. 19 from Table I. From the same slice

Moreover, I mention the observation by Nissl[76)] that the glial cells, besides their function as providing nutrients for the nervous substance, also have phagocytic properties similar to the body leukocytes which never enter into the cerebral cortex. This latter observation by Nissl seems to me very important since, first, I have never observed clearly identified leukocytes in brain and spinal cord within the border membrane. I agree with Nissl that those contrary claims are based on a confusion with glial cells (see the criticism of Nissl of the book on the nerve cell by Kronthal in Zentralbl. f. Nervenheil. und Psychiatrie 1902). Furthermore, second, I consider only the border membrane as a barricade for the leukocytes to the central nervous substance, which explains this peculiar fact.

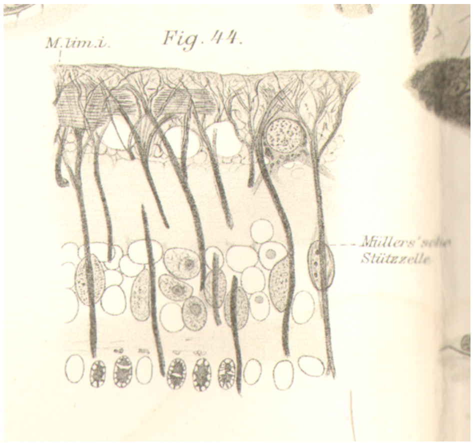

Fig. 44 from Table III. Vertical cross-section through the inner half of a guina pig retina. Alcohol-chloroform-acetic acid. Differentiated hematoxylin staining. Support fiber bundles of the Müller support cells; moreover, around the ganglion cells of the optic nerve and its bundles is a delicate and fine network which seems to be connected to the Müller support cells. Hartnack 1/12 Oc.

During its development, the retina shows fibrous formations in the form of Müller cells, which span from the inner to the outer border membrane and provide support. The internal end of these strong rods, composed of multiple delicate fibers, is shown in Fig. 44, obtained from a guinea pig. In my opinion, the *Membrana limitans Retinae interna*, with its field pattern, corresponds to the endfeet of the fiber-containing support cells, representing an analog to the glial threads of the brain.

Towards the *M. lim. Ret. externa*, I find delicate fibers arranged in a ring-like fashion at the rods and cones of the outer granule cell layer; they seem to terminate at the *Lim. externa*, which they form with numerous delicate and small endfeet. Moreover, I observe a delicate membrane at the vessels of the retina, which limits the perivascular spaces, similar to the capillaries of the brain.

For the N. opticus, I would like to add that its nerve bundles are delimited by a cortical border layer with a border membrane towards the penetrating connective tissue, similar to the white matter of the spinal cord.

Lloyd Andriezen considered the circumvascular glial cover as a protection of the brain parenchyma against the pulsation of the vessels,

which in addition permits a free stream of the solution from and towards the vessel space, due to its felt nature. Storch has added the notion that for the diffusion of the lymphatic solution from the blood capillaries within the glial fiber intermediate spaces, the laws of capillarity must be applied. Possibly, the pulsation of the vessels triggers flows within the fluid of the perivascular glial structure and could promote drainage to the subarachnoid lymphatic spaces. Both have overlooked that the so-called intermediate spaces of the glia are not open to the outside but are restricted by a border membrane, which would at least impose filtration resistance to the movement of lymphatic fluid. The *Membr. lim. Gliae perivascularis*, as a delicate and second tube, circumvents the vessel tube with its adventitious and tube-like cover, additionally supported by the radial system of the marginal glia. Thus, pulsations of the vessels in regions of the muscle-type arteries will generate suction movements into the perivascular spaces and influence the distribution of the intramarginal fluid. Thus, I support the statements of Andriezin and Storch.

It remains for future investigations to determine the extent to which glia participate in substance movement within their protoplasm or meshwork. It needs to be determined to what extent the longitudinal drainage via the extramarginal spaces, namely the His and Virchow-Robin spaces, serves the outward efflux of lymphatic brain fluid.

Comments

a) Not clear what he means with fibrillation

b) The sea lamprey (Petromyzon marinus)

c) Earth worm

d) Lancelet

e) Hagfish

f) Frog

g) Toad

h) Lizzard

i) Dogfish

j) Whiting

k) Plaice

l) It is actually on page 37/38 in the Weigert book. I have used the translation from the Weigert book

m) Weigert terms the fibres as filaments, the processes extensions

n) Citation on page 52 of Weigert, not 116 as stated in the Held text

o) He is obviously refering here to the radial glial cells in early development

p) The original German title is Geheimrat

q) Plural of granulum, granule. It is not clear what he actually means

r) The adventitia is the outer layer of fibrous connective tissue surrounding an organ

s) Frommann is several times misspelled in the original text by missing the last character. We have used the correct name.

t) Chromealaun is a double salt of potassium- and chromesulfate

u) Ironaluminumsulfate ($Fe^{2+}Al_2[SO_4]_4$)

v) In 1850, Charles-Gabriel Pravaz (1791–1853) developed a syringe for subcutaneous injection which is considered as the prototype of all injection syringes

w) There is obviously a mistake in the original text. There is no Fig. 33c, but it refers to 33a and b.

x) In the original text of Held the French term is cited: D´une substance homogene ou a peine striee

y) refering to the focus screw of the microscope

z) What is described as filled net is probably myelin

aa) It is referred here to the German translation of Golgi´s omnia opera published in 1894.

bb) Old term for mitochondria in nerve cells, the nerve ending area is most likely the neurite

cc) European medicinal leech

dd) Impregnated refers to stained

ee) the term Langstrahler refers to a cell with long rays (=long processes). This terminology was originally introduced by Retzius.

Explanation of Tables

Table I Fig. 1-19

Table II Fig. 21-36

Table III Fig. 37-52

Table IV Fig. 51-60

Fig. 20a-c is in the text page 215 and 293, 294

With exception of Fig.49 and partially also Fig. 47a which were made by the lithograph Schindler, I have drawn all other images with the help of the Abbe camera[1]. The preparations corresponding to Figs 21 and 22 are from Geheimrat W. His, Fig. 27 from Professor S. Kästner, Fig. 23-26 from a Altmann series. All other images are drawn based on my own preparations. Figs 15-19 are of original size, all others reduced to 2/3.

[1] I guess he means a Camera Lucida

Table I

Fig. 1. From a cross section through the lumbar spinal cord of an adult rabbit, showing the central canal and its environment. Fixation in a warmed solution of potassium bichromium and ammonium molybdanium . Differential also hematoxylin staining. Leitz Obj. 4, Oc.6.

Fig. 2. From the same slice, ventral part of the ependyma. Hartnack homogeneous immersion 1/12. Oc. 6.

Fig. 3. Ependyma of the IV. Ventricle of a rabbit at the level of the nucleus facialis. Fixation with Müller´s solution with secondary osmication. On granula differential hematoxylin staining. Hartnack homogeneous immersion 1/12. Oc. 6. Except of the ependyma, two subependymal glial cells are cut. Glial fibers dark black, protoplasmic granules dark grey.

Fig. 4a and b. Glial cells at a cross section of the rabbit spinal cord (white matter of the ventral columns)

Fig. 4a a spongioblastic, Fig. 4b a film-type glial cell. Hartnack 1/12, Oc. 6.

Fig. 4c. Superficial glial cell from the ventral brain stem of the rabbit, which directly forms the *M- lim. Gliae* with its cell body. Glial fibers black, protoplasmic granula (partially ring bodies) dim grey. Fixation in Müller solution with secondary osmium treatment. For granula differentiated alsol hematoxylin stain.

Figs. 5a-c, 6-10. Glial cells from the human cross section of the spinal cord (Fig. 7a from a 21-year-old, the others from a 24-year old executed). Figs. 5a-c, 6, 10 from white matter. Potassium bichromium, iron alaun fixation. Hartnack 1/12 Oc. 6.

Fig. 5d. From a longitudinal section of bovine white matter

Fig. 5a. cross-fibered glial cell; on the following slices the process cut below is connected to a glial cell. Fig. 5b. The upper glial cells is a form with few fibers and a large and granulated cell body. Fig. 5c. Two glial cells with glial fibers mainly in the processes.

Fig. 5d. From bovine; a broadly anastomized syncytium of glial cells. Its protoplasm enwraps several nerve fiber spaces in an arial fashion and contains several glial fibers.

Fig. 6. Two connected cells; the lower one is partially spongioplastic and radial fibered, the upper one with a large protoplasm, granulated and cross-fibered.

Fig. 7. Two connected radial-fibered glial cells; both spongioplastic.

Fig. 7a. Radial-bundled glial cell from the substantia neurogliae centralis.

Fig. 8a. Glial cell with small protoplasm, radial fibered.

Fig. 8b. Cell with very small protoplasm, cross-fibered.

Fig. 9. Glial cell with two nuclei which is strongly granulated.

Fig. 10. Glial cell with small protoplasm with thick processes, which are crossed-over by glial fibers.

Fig. 11. Cross-section image from the white matter of the human lower spinal cord (area of the dorsal horn). 24-year-old executed. Fixation in potassium bichromium. Differential hematoxylin staining according to M. Heidenhain. Hartnack 1/12 Oc. 6. (No net between the nerve fibers).

Fig. 12. From the same slice; Penetration area of the ventral roots at the ventral horn.

Fig. 13. From the lumbal cord of human; Cross-section from the ventral cord. Broader protoplasm mass between the glial fibers. Hartnack 1/12 Oc. 6.

Fig. 14. Net-shaped branched glial cell from the white matter of the human cerebral cortex (24-year-old executed). Alcohol-chloroform, acidic acid mix. Hartnack 1/12 Oc. 8.

Fig. 15. From the same slice.

Fig. 16. From the white matter of the rabbit cerebellum. Molybdenum method according to Bethe.

Fig. 17. From the same slice which shows the net-shaped glial sheath and two glial nodes of Ranvier in a longitudinal section. Hartnack 1/12 Oc. 8.

Fig. 18. From the grey matter of the frontal brain of an adult rabbit; Section through two net-shaped branched glial cells at the surface of a large frontal brain cell . Chromium-formalin , acidic acid. Differential alsol hematoxylin staining. Hartnack 1/12 Oc. 8.

Fig. 19. From the same slice

Textfigures 20a-c. Glial cells from the spinal cord of a few week-old dog. Silver staining according to Golgi. Hartnack 1/12 Oc. 6.

Table II

Fig. 21. Part of a spinal cord cross-section of a human embryo E B. Preparation from Geheimrat W. His. Hartnack 1/12 Oc. 6.

Fig. 22. Part of a spinal cord cross-section of a human embryo Br 3. Preparation from Geheimrat W. His. Hartnack 1/12 Oc. 6.

Figs. 23-26. From a rabbit embryo. Preparations from R. Altmann. Figs. 23-25 from the lateral surface of the prolonged cord, Fig. 26 of the spinal cord. Hartnack 1/12 Oc. 6.

Fig. 27. From a chicken embryo on the 5th day of incubation. Preparation from Professor S. Kästner. Transit position of the sensible trigeminal nerve through the *M. limitans Gliae superficialis.* Hartnack 1/12 Oc. 6.

Figs. 28a-c. Three developmental stages of the Nervus opticus of the mouse in cross-section. Fixation in chromium-formalin , acidic acid. Also hematoxylin staining. Hartnack 1/12 Oc. 4.

Fig. 28a from a 12-day old embryo, Fig. 28b from a new-born, Fig. 28c from a 5-day old mouse.

Fig. 29. Surface of the lumbal spinal cordof an adult rabbit (area of the lateral streak). Potassium bichromium and ammonium molybdenum. Hartnack 1/12 Oc. 4.

In the interior of the white substance the glia is not completely marked. At x is a glial cell inserted with their cell body into the *M. lim. Gliae superfic*. The band of dense glia left to outer blood vessel will provide in the subsequent slices a marginal and a perivascular glia corresponding to the inserted blood vessel.

Fig. 30. Dorsal surface of the human spinal cord (bend towards the septum posterior) at a cross-section of the lumbar cord (24-year-old executed). The pia mater has been plainly taken off from the *M. lim. Gliae superficialis*. Hartnack 1/12 Oc. 2.

Fig. 31. From the same slice. Depth of the Fisura long. ant.

Fig. 32. From the same slice. Boundary of a septum of the pia mater from a dorsal root which appears vessel-free.

Fig. 33a, b. Surface of the cerebellum of an adult rabbit. chromium-formalin, acidic acid. Hartnack 1/12 Oc. 6.

Fig. 33a. Vertical cut through the entire cerebral cortex. Fig. 33b. Plane image of the *M. limitans Gliae superficialis* (left) and the Intima piae (right).

Figs. 34a-c. Vertical cut through the superficial area of the human cerebral cortex (24-year-old executed). alcohol-chloroform-acetic acid. Iron alaun hematoxylin staining according to M. Heidenhain. Hartnack 1/12 Oc. 6.

Fig. 34a. Less differentiated slice. Chamber-like structure of the marginal glia; the glial fibers appear at some locations as deeply dark enforcement bands; Fig. 34c shows the differentiation of glial fibers and their fibrillate endfeet. Fig. 34b shows the smooth isolation of a detached pia, which in contrast is closely and uniformly attached in Fig. 34c. In Fig. 34a, the Intima piae is bleb-like detached resulting in a epi-cerebral cleft. Hartnack 1/12 Oc. 6.

Fig. 35. Surface image of the *M. lim. Gliae superficialis* at the human cerebral cortex. Same preparation. Entry location of a small blood vessel. Single bundles of the pia are cut.

Fig. 36. Skewed cut of the same preparation. On the left, the border membrane is cut, on the right the glial space below.

Table III

Fig. 37. Cross-section through a small artery in the superficial glial zone of the human cerebral cortex. On top a small perivascular space is open; the adventitial sheath is tightly compressed. Hartnack 1/12 Oc. 6.

Fig. 38. Entry of two vessels into the substance of the cerebral cortex. Transition of the superficial into the perivascular border membrane of neuroglia. From the same brain. Seitz Obj. 4 Oc. 2.

Fig. 39a-c. Glial vessel insertion in rabbit. b and c are from the Substantia Neurogliae centralis of the spinal cord, a from the IV. ventricle. Hartnack 1/12 Oc. 6.

Fig. 40. Arial image of the perivascular border membrane of a vein of the rabbit elongated cord. The dots, respective lines in the single fields indicate the position of the glial fibers below it. Hartnack 1/12 Oc. 6.

Fig. 41. Glial border membrane of a capillary from the human cortical grey matter (24 year old-executed). On the left, the glial reticulum is partially visible. At the left end of the capillary is an adventitial nucleus which is surrounded by a small, very delicate mass of protoplasm as a partially visible sheath between the endothelial tube and the perivascular glial membrane.

Fig. 42. From the white matter of the human cerebral cortex. Alcohol-chloroform-acetic acid. Highly differentiated iron alaun hematoxylin staining according to M. Heidenhain. At the vessel tube are parallel, partially radial, attached glial fibers. Due to the strong de-staining and contact pressure, the space at the glial border membrane or at the vessel is not visible resulting in the appearance of a direct connection between glial endfeet and the vessel tube. Hartnack 1/12 Oc. 6.

Fig. 43. Cross-section of a blood vessel from the rabbit cortical grey matter. Alcohol fixation. The glial radial border layer is destroyed resulting in a broad shrinkage space in which only the glial endfeet and the glial border membrane remained at the vessel. This is a better location, since often the glial endfeet and its *M. limitans* is largely destroyed. Hartnack 1/12 Oc. 6.

Fig. 44. Vertical cross-section through the inner half of a guina pig retina. Alcohol-chloroform-acetic acid. Differentiated hematoxylin staining. Support fiber bundles of the Müller support cells; moreover, around the ganglion cells of the optic nerve and its bundles is a delicate and fine network which seems to be connected to the Müller support cells. Hartnack 1/12 Oc. 6.

Figs. 45 and 46. From the rabbit cerebellum. Injection into the depth. Alcohol-chloroform-acetic acid.

Fig. 45. At the lower curved surface the injection mass has pushed away the pia and has filled a epi-cerebral space which is separated by the border membrane with its glial endfeet from the shrinkage space. On top, the injection is also subarachnoid most likely due to the damage of the adventitial blood vessel sheath in the interior of the brain substance

Fig. 46. At a different location of the same preparation. The ink has been caught in the windings below the *Membrana limitans Gliae superficialis*.

Figs. 47a-c. Longitudinal section and two cross-sections of a leech connective. Glial cell and its extension to the glial sheath. ´a´ from a stretched, ´b´and ´c´ from a contracted animal. Alcohol-chloroform-acetic acid. Alaun hematoxylin staining according to M. Heidenhain. Hartnack 1/12 Oc. 6.

Fig. 48a, b. From the same slice; glial sheath from a stretched animal, ´b´ from a contracted.

Fig. 49. Median star cell from leech; strong differentiation, so that only the nucleus and the glial grid as an apparently independent network remains visible. Hartnack 1/12 Oc. 6.

Fig. 50. From a longitudinal section through the ventral ganglion of a leech. Median star cell with glial extensions. The glial grid is within the cell protoplasm; its extension into the central fiber mass is not visible, but only its continuation as net-shaped sheath at ganglion cells. Hartnack 1/12 Oc. 6.

Literature

1) R. Virchow, Über eine im Gehirn und Rückenmark gefundene Substanz mit der chemischen Reaktion der Cellulose. Arch. f. path. Anat. u. Phys. 1853.

2) Kölliker, Handbuch der Gewebelehre. 1863.

3) Deiters, Untersuchungen über Gehirn und Rückenmark. 1865.

4) Golgi, Contributione alla fina anatomia degli organi centrali del sistema nervoso. 1871.

5) Ranvier, De la névroglie. Archives des physiologie normale et pathologique. 1883.

6) Weigert, Beiträge zur Kenntnis der menschlichen Neuroglia. Frankfurt 1895.

7) S. B. Pellizzini, Sulla struttura e sull' origine delle granulazioni ependimali. Contributo all istologia e patologia della nevrologia. Riv. sper. Frenatria 22. 1896.

8) A. v. Kölliker, Handbuch der Gewebelehre. 1897. S. 148-153.

9) F. Reinke, Beiträge zur Histologie des Menschen II. Arch. f. mikr. Anatomie. 50. 1897.

10) H. Held, II. Abhandlung über Nervenzellenstrukturen. Arch. f. Anatomie 1897, Suppl. S. 275 Anm.

11) W. F. Robertson, Note on Weigerts theory regarding the structure of the Neuroglia. Journ. of Mental Science l897.

12) Eurich, Studies on the Neuroglia, Brain 1897.

13) Whitwell, On the Structure of the Neuroglia. British med. Jour. 12.

14) Brodmann, Über den Nachweis von Astrocyten mittelst der Weigertschen Gliafärbung. Jenaische Zeitschr. f. Naturw. XXXIII. l899. 1. S. 181.

15) Yamagiva, Eine neue Färbung der Neuroglia. Virchows Archiv 160. S. 358. l900.

16) Ströbe, Über Struktur pathologischer Neurogliawucherungen. Zentralblatt f. allg. Path. u. path. Anat. 1896. S. 864.

17) Storch, Über die pathologisch-anatomischen Vorgänge am Stützgerüst des Zentralnervensystems. Virchows Archiv. 157. 1899.

18) E. Müller, Studien über Neuroglia. Arch. f. mikr. Anatomie. 55. 1899.

19) Studnička, Untersuchungen über den Bau des Ependyms der nervösen Zentralorgane. Anatomische Hefte. Bd. XV. 1900.

20) Obersteiner, Zur Histologie der Gliazellen in der Molekularschicht der Großhirminde. Arb. a. d. Institut. 1900.

21) Marinesco, Du rôle de la nevroglie dans l'évolution des inflammations. 2. internat. med. Congress. Paris 1900. (n. d. Jahresbericht v. Schwalbe. VI. 1900.)

22) Z. Dimitrova, Recherehes sur la structure de la Glande pinéale. Le Nevraxe. II. 1901.

23) G. Carl Huber, Studies of Neuroglia. The American Journal of Anatomy. I. 1901.

24) Irving Hardesty, The Neuroglia of the Spinal Cord of the Elephant with some preliminary Observation upon the Developement of Neuroglia Fibres. American Journal of Anatomy II. Nr. I.

25) H. Fuchs, Über das Ependym. Verb. d. anatom. Gesellschaft zu Halle 1902.

26) Aguerre, Untersuchungen über die menschliche Neuroglia. Arch. f. mikr. Anat. 56. 1900.

27) W. His, Über ein perivasculäres Kanalsystem in den nervösen Centralorganen 1865.

28) F. Boll, Die Histiologie und Histiogenese der nervösen Centralorgane. Berlin 1873.

29) A. Goette, Die Entwicklungsgeschichte der Unke. Leipzig 1875.

30) Hensen, Zeitschrift für Anatomie und Entwicklungsgeschichte 1876.

31) W. His, Über das Auftreten der weißen Substanz und der Wurzelfasern am Rückenmark menschlicher Embryonen. Arch. f. Anatomie 1883.

32) W. His, Zur Geschichte des menschlichen Rückenmarks und der Nervenwurzeln. Abh. d. math. phys. Klasse d. Kgl. sächs. Ges. d. W. 1886.

33) W. His, Die Neuroblasten und deren Entstehung im embryonalen Mark. Abh. d. math. phys. Kl. d. Kgl. s. Ges. d. W. 1889.

34) Vignal, Sur le développement des éléments de la moelle des mammifères. Arch. de physiologie normale et path. 1884.

35) H. Gierke, Die Stützsubstanz des Centralnervensystems. Arch. f. mikr. Anatomie 25, 26 1885, 1886.

36) C. Golgi, Sulla fina anatomia degli organi centrali del Sistema nervoso. Milano 1885.

37) F. Nansen, The Structure and Combination of the Histological Elements of the Central Nervous System. Bergen 1887.

38) R. y Cajal, Sur l'origine et les ramifications des fibres nerveuses de la moelle embryonnaire. Anat. Anzeiger 1890.

Nuevos observationes sobre la estructura de la médula espinal de les mamiferos. Barcelona, 1890.

39) A. v. Kölliker, Zur feineren Anatomie des centralen Nervensystems, II. das Rückenmark. Zeitschrift f. wiss. Zool. 1890.

40) v. Gehuchten, La structure des centres nerveux. La cellule 1891.

41) M. v. Lenhossék, Zur Kenntnis der Neuroglia des menschl. Rückenmarks. Verhandl. der anat. Gesellsch. 1891.

Feinerer Bau des Nervensystems 1892.

42) G. Retzius, Ependym und Neuroglia. Biol. Unters. 1893.

43) Cl. Sala y Pons, La Neuroglia de los Vertebrados. Barcelona 1894.

44) P. Lachi, Contribution à l'histogenèse de la nevrologie dans la moelle épinière du poulet. Arch. it. biol. XV. 1891.

45) G. Valenti, Contribution à l'histogenèse de la cellule nerveuse et de la nevrologie du cerveau de certains poissons chondrostéiques. Arch. it. biol. XVI. 1891.

46) Capobianco, Della partecipazione mesodermica nelle genesi della neuroglia cerebrale. Monit. zool. ital. XII. 1901 u. Arch. ital. biol. 1902.

47) Shinkishi Hatai, On the origin of nevrologia tissue from the mesoblast. Journ. of comp. neurology XII. 1902.

48) H. Gierke, Die Stützsubstanz des Centralnervensystems Arch. f. mikr. Anatomie 1885-1886.

49) Bergmann, Zeitschrift f. rat. Medicin. N. F. VIII.

50) C. Golgi, Gesammelte Abhandl. deutsche Ausgabe 1894.

51) J. Henle und F. Merkel, Über die sogenannte Bindesubstanz der Centralorgane des Nervensystems. Zeitschr. f. ration. Medicin 1868.

52) J . Schaffer, die oberflächliche Gliahülle und das Stützgerüst des weißen Rückenmarksmantels. Anat. Anzeiger 1894. Beiträge zur Kenntnis des Stützgerüstes im menschlichen Rückenmarke. Arch. f. mikr. Anat. 1894.

53) Renault, Insertion sous forme de revêtement épithélial continu des pieds des fibres neurogliques sur la limitante marginale d'un nevraxe adulte. Comptes rend. hebdom. des sc. T. 126.

54) S. Kure, Über die Beziehungen der Glia zu den Gefäßen. Neurologia I.

55) Fromman(n), Untersuchungen über die normale und pathologische Anatomie des Rückenmarkes. Jena 1864 und 1877.

56) L. Andriezen, On a system of fibre-cells surrounding the blood-vessels of the Brain of Man and Mammals, and its Physiological Significance. Intern. Monatsschr. f. An. und Phys. X. 1893.

57) W. His, Über ein perivasculäres Kanalsystem in den nervösen Zentralorganen und über dessen Beziehungen zum Lymphsystem. Leipzig 1865.

58) Axel Key und G. Retzius, Studien in der Anatomie des Nervensystems und des Bindegewebes. Stockholm 1875.

59) R. Virchow, Über die Erweiterung kleinerer Gefäße. Virchows Archiv III. 1851.

60) Ch. Robin, zuerst in Segond, Le systeme capillaire sanguin., Paris 1853, dann in Comptes rendus et mém. de la Soc. de Biol. Paris 1855 u. Recherches sur quelques Particularités de la Structure des Capillaires de l'éncephale. Journal de la Physiol. 1859 (S. 543-545).

61) W. His, Beiträge zur Kenntnis der zum Lymphsystem gehörigen Drüsen. Zeitschr. f. wiss. Zool. X. 1859. S. 340.

62) C. J. Eberth, Über die Blut- u. Lymphgefäße des Gehirns u. Rückenmarks. Virchows Archiv 49. 1870.

62a) Riedel, Die perivasculären Lymphgefäße im Zentralnervensystem und der Retina. Arch. f. mikr. Anatomie 1870.

63) Bevan Lewis, A text book of mental diseases with special reference to the pathological aspects of insanity. London 1889.

64) Binswanger und Berger, Beiträge zur Kenntnis der Lymphzirkulation in der Großhirnrinde. Virchows Archiv 152. 1898.

65) Albrecht Bethe, Über die Neurofibrillen in den Ganglienzellen von Wirbeltieren und ihre Beziehungen zu den Golginetzen. Archiv für mikrosk. Anat. 55. 1900.

66) Hans Held, Über den Bau der grauen und weißen Substanz I. Arch. f. Anatomie. 1902.

67) S. Paladino, Sur les limites précises entre la névroglie et les éléments nerveux dans la moelle epiniere, et sur quelques-unes des questions histophysiologi ques qui s'y rapportent. Ach. ital. de Biologie XXII. 1895.

68) Stephan Apáthy, das leitende Element des Nervensystems und seine topographischen Beziehungen zu den Zellen. Mitt. d. zool. Station XII. 1897.

69) Emil Holmgreen, Studien in der feineren Anatomie der Nervenzellen. Anat. Hefte XV. 1900.

70) Ch. Simon, Recherches sur la cellule des ganglions sympatbiques des Hirudinées. Internationale Monatsschr. f. An. u. Phys. XIII. 1896.

71) A. Bethe, Über die Neurofibrillen in den Ganglienzellen der Wirbeltiere und ihre Beziehungen zu den Golginetzen. Arch. f. mikr. Anatomie 55. 1900.

72) O. Deiters, Untersuchungen über Gehirn und Rückenmark. 1865.

73) E. Storch, Über die pathologisch-anatomischen Vorgänge am Stützgerüst des Zentralnervensystems. Virchows Arch. 157. 1899. S. 233.

74) R. y Cajal Algunas conjeturas sobre el mecanismo anatómico de la Ideacion, Asociation y Atencion. Madrid 1895 und Arch. f. Anat. 1895.

75) R. Wlassak, Die Herkunft des Myelins, Archiv f. Entwicklungsmechanik 1895.

76) Fr. Nissl, Über einige Beziehungen zwischen Nervenzellerkrankungen und gliösen Erscheinungen bei verschiedenen Psychosen. Arch. f. Psychiatrie 1899.

77) V. Pranter, Zur Färbung der elastischen Fasern. Zentralbl. für allg. Path. u. path. Anat. XIII. 1902.

78) W. His, Über die Wurzeln der Lymphgefäße in den Häuten des Körpers und über die Theorien der Lymphbildung. Zeitschr. f. wiss. Zool. XII. 1862.

79) W. H. Gaskell, Über die Wand der Lymphkapillaren (C. Ludwig, Arbeiten aus d. phys. Institut zu Leipzig. 1877 S. 143).

80) Rieder, Beiträge zur Histologie und pathol. Anatomie der Lymphgefäße und Venen. Zentralbl. f. allg. Path. 1898.

81) Reinke, Zellstudien, Arch. f. mikr. Anatomie 1894.

82) A. Spuler, Beiträge zur Histologie und Histogenese der Binde- und Stützsubstanz. Anat. Hefte 1896.

83) W. Flemming, Über die Entwicklung der kollagenen Bindegewebsfibrillen bei Amphibien und Säugetieren. Arch. f. Anat. u. Phys. anat. Abt. 1897.

84) M. Gardner, Zur Frage über die Histogenese des elastischen Gewebes, Biol. Zentralbl. 1897.

85) W. His, Über das Epithel der Lymphgefäßwurzeln und über die v. Recklinghausenschen Saftkanälchen. Zeitschr. f. wissensch. Zoologie. 1863. S. 470.
