# Supplementary material for: The Concept of Neuroglia ‐ the State of the Art Circa 1900
Source: Glia. 2025 Feb 4;73(5):890–904. doi: 10.1002/glia.24678 (PMC11920685; doi:10.1002/glia.24678)
Supplement: Supplementary file 11 — Data S11. Reference list of citations from the articles by Retzius, Lenhossek, Weigert and Held and combined into one list either sorted alphabetically or chronologically. [file GLIA-73-890-s001.pdf]

This list provides all references from the 5 chapters by Held, Retzius, Weigert and Lenhossek separately. Subsequently all references were combined into one list, duplicates were removed and either sorted alphabetically or chronologically.

References Held

Aguerre, Untersuchungen über die menschliche Neuroglia. Arch. f. mikr. Anat. 56. 1900.

Andriezen, L. On a system of fibre-cells surrounding the blood-vessels of the Brain of Man and Mammals, and its Physiological Significance. Intern. Monatsschr. f. An. und Phys. X. 1893.

Apáthy, Stephan. Das leitende Element des Nervensystems und seine topographischen Beziehungen zu den Zellen. Mitt. d. zool. Station XII. 1897.

Bergmann, Zeitschrift f. rat. Medicin. N. F. VIII.

Bethe, Albrecht. Über die Neurofibrillen in den Ganglienzellen von Wirbeltieren und ihre Beziehungen zu den Golginetzen. Archiv für mikrosk. Anat. 55. 1900.

Binswanger und Berger, Beiträge zur Kenntnis der Lymphzirkulation in der Großhirnrinde. Virchows Archiv 152. 1898.

Boll, F. Die Histiologie und Histiogenese der nervösen Centralorgane. Berlin 1873.

Brodmann, Über den Nachweis von Astrocyten mittelst der Weigertschen Gliafärbung. Jenaische Zeitschr. f. Naturw. XXXIII. I899.

Capobianco, Della partecipazione mesodermica nelle genesi della neuroglia cerebrale. Monit. zool. ital. XII. 1901 u. Arch. ital. biol. 1902.

Deiters, O. Untersuchungen über Gehirn und Rückenmark. 1865.

Dimitrova, Z. Recherehes sur la structure de Ia Glande pinéale. Le Nevraxe. II. 1901.

Eberth, C. J. Über die Blut- u. Lymphgefäße des Gehirns u. Rückenmarks. Virchows Archiv 49. 1870.

Eurich, Studies on the Neuroglia, Brain 1897.

Flemming, W. Über die Entwicklung der kollagenen Bindegewebsfibrillen bei Amphibien und Säugetieren. Arch. f. Anat. u. Phys. anat. Abt. 1897.

Frommann, C. Untersuchungen über die normale und pathologische Anatomie des Rückenmarkes. Jena 1864 und 1877.

Fuchs, H. Über das Ependym. Verb. d. anatom. Gesellschaft zu Halle 1902.

Gardner, M. Zur Frage über die Histogenese des elastischen Gewebes, Biol. Zentralbl. 1897.

Gaskell, W. H. Über die Wand der Lymphkapillaren (C. Ludwig, Arbeiten aus d. phys. Institut zu Leipzig. 1877 S. 143).

Gierke, H. Die Stützsubstanz des Centralnervensystems. Arch. f. mikr. Anatomie 25, 26 1885, 1886.

Goette, A. Die Entwicklungsgeschichte der Unke. Leipzig 1875.

Golgi, C. Sulla fina anatomia degli organi centrali del Sistema nervoso. Milano 1885.

Golgi, Contributione alla fina anatomia degli organi centrali del sistema nervoso. 1871.

Hardesty, lrving. The Neuroglia of the Spinal Cord of the Elephant with some preliminary Observation upon the Developement of Neuroglia Fibres. American Journal of Anatomy II. Nr. I.

Hatai, Shinkishi. On the origin of nevrologia tissue from the mesoblast. Journ. of comp. neurology XII. 1902.

Held, H. II. Abhandlung über Nervenzellenstrukturen. Arch. f. Anatomie 1897, Suppl. S. 275 Anm.
Held, Hans Über den Bau der grauen und weißen Substanz I. Arch. f. Anatomie. 1902.
Henle, J. und Merkel, F. Über die sogenannte Bindesubstanz der Centralorgane des Nervensystems. Zeitschr. f. ration. Medicin 1868.
Hensen, Zeitschrift für Anatomie und Entwicklungsgeschichte 1876.
His, W. Beiträge zur Kenntnis der zum Lymphsystem gehörigen Drüsen. Zeitschr. f. wiss. Zool. X. 1859..
His, W. Die Neuroblasten und deren Entstehung im embryonalen Mark. Abh. d. math. phys. Kl. d. Kgl. s. Ges. d. W. 1889.
His, W. Über das Auftreten der weißen Substanz und der Wurzelfasern am Rückenmark menschlicher Embryonen. Arch. f. Anatomie 1883.
His, W. Über das Epithel der Lymphgefäßwurzeln und über die v. Recklinghausenschen Saftkanälchen. Zeitschr. f. wissensch. Zoologie. 1863.
His, W. Über die Wurzeln der Lymphgefäße in den Häuten des Körpers und über die Theorien der Lymphbildung. Zeitschr. f. wiss. Zool. XII. 1862.
His, W. Über ein perivasculäres Kanalsystem in den nervösen Zentralorganen und über dessen Beziehungen zum Lymphsystem. Leipzig 1865.
His, W. Zur Geschichte des menschlichen Rückenmarks und der Nervenwurzeln. Abh. d. math. phys. Klasse d. Kgl. sächs. Ges. d. W. 1886.
Holmgreen, Emil. Studien in der feineren Anatomie der Nervenzellen. Anat. Hefte XV. 1900.
Huber, G. Carl. Studies of Neuroglia. The American Journal of Anatomy. I. 1901.
Key. Axel und Retzius, G. Studien in der Anatomie des Nervensystems und des Bindegewebes. Stockholm 1875.
Kölliker A. v. Zur feineren Anatomie des centralen Nervensystems, II. das Rückenmark. Zeitschrift f. wiss. Zool. 1890.
Kölliker, A. v. Handbuch der Gewebelehre. 1897.
Kure, S. Über die Beziehungen der Glia zu den Gefäßen. Neurologia I.
Lachi, P. Contribution à l'histogenèse de la nevrologie dans la moelle épinière du poulet. Arch. it. biol. XV. 1891.
Lenhossék M. v. Feinerer Bau des Nervensystems 1892.
Lenhossék, M. v. Zur Kenntnis der Neuroglia des menschl. Rückenmarks. Verhandl. der anat. Gesellsch. 1891.
Lewis, Bevan. A text book of mental diseases with special reference to the pathological aspects of insanity. London 1889.
Marinesco, Du rôle de la nevroglie dans l'évolution des inflammations. 2. internat. med. Congress. Paris 1900. (n. d. Jahresbericht v. Schwalbe. VI. 1900.)
Müller, E. Studien über Neuroglia. Arch. f. mikr. Anatomie. 55. 1899.
Nansen, F. The Structure and Combination of the Histological Elements of the Central Nervous System. Bergen 1887.
Nissl, Fr. Über einige Beziehungen zwischen Nervenzellerkrankungen und gliösen Erscheinungen bei verschiedenen Psychosen. Arch. f. Psychiatrie 1899.
Obersteiner, Zur Histologie der Gliazellen in der Molekularschichtder Großhirminde. Arb. a. d. Institut. 1900.

Paladino, S. Sur les limites précises entre la névroglie et les éléments nerveux dans la moelle epiniere, et sur quelques-unes des questions histophysiologi ques qui s'y rapportent. Ach. ital. de Biologie XXII. 1895.
Pellizzini, S. B. Sulla struttura e sull' origine delle granulazioni ependimali. Contributo all Histologia e patologia della nevrologia. Riv. sper. Frenatria 22. 1896.
Pranter, V. Zur Färbung der elastischen Fasern. Zentralbl. für allg. Path. u. path. Anat. XIII. 1902.
Ranvier, De la névroglie. Archives des physiologie normale et pathologique. 1883.
Reinke, F. Beiträge zur Histologie des Menschen II. Arch. f. mikr. Anatomie. 50. 1897.
Reinke, Zellstudien, Arch. f. mikr. Anatomie 1894.
Renault, Insertion sous forme de revêtement épithélial continu des pieds des fibres neurogliques sur la limitante marginale d'un nevraxe adulte. Comptes rend. hebdom. des sc. T. 126.
Retzius, G. Ependym und Neuroglia. Biol. Unters. 1893.
Riedel, Die perivasculären Lymphgefäße im Zentralnervensystem und der Retina. Arch. f. mikr. Anatomie 1870.
Rieder, Beiträge zur Histologie und pathol. Anatomie der Lymphgefäße und Venen. Zentralbl. f. allg. Path. 1898.
Robertson, W. F. Note on Weigerts theory regarding the structure of the Neuroglia. Journ. of Mental Science I897.
Robin, H. zuerst in Segond, Le systeme capillaire sanguin., Paris 1853, dann in Comptes rendus et mém. de la Soc. de Biol. Paris 1855 u. Recherches sur quelques Particularités de la Structure des Capillaires de l'éncephale. Journal de la Physiol. 1859 (S. 543-545).
Sala y Pons, Cl. La Neuroglia de los Vertebrados. Barcelona 1894.
Schaffer, J. Beiträge zur Kenntnis des Stützgerüstes im menschlichen Rückenmarke. Arch. f. mikr. Anat. 1894.
Schaffer, J. die oberflächliche Gliahülle und das Stützgerüst des weißen Rückenmarksmantels. Anat. Anzeiger 1894.
Simon, Ch. Recherches sur la cellule des ganglions sympatbiques des Hirudinées. Internationale Monatsschr. f. An. u. Phys. XIII. 1896.
Spuler, A. Beiträge zur Histologie und Histogenese der Binde- und Stützsubstanz. Anat. Hefte 1896.
Storch, E. Über die pathologisch-anatomischen Vorgänge am Stützgerüst des Zentralnervensystems. Virchows Arch. 157. 1899.
Storch, E. Über die pathologisch-anatomischen Vorgänge am Stützgerüst des Zentralnervensystems. Virchows Archiv. 157. 1899.
Ströbe, Über Struktur pathologischer Neurogliawucherungen. Zentralblatt f. allg. Path. u. path. Anat. 1896.
Studnička, Untersuchungen über den Bau des Ependyms der nervösen Zentralorgane. Anatomische Hefte. Bd. XV. 1900.
v. Gehuchten, La structure des centres nerveux. La cellule 1891.
Valenti, G. Contribution à l'histogenèse de la cellule nerveuse et de la nevrologie du cerveau de certains poissons chondrostéiques. Arch. it. biol. XVI. 1891.
Vignal, Sur le développement des éléments de la moelle des mammifères. Arch. de physiologie normale et path. 1884.

Virchow, R. Über die Erweiterung kleinerer Gefäße. Virchows Archiv III. 1851.
Virchow, R. Über eine im Gehirn und Rückenmark gefundene Substanz mit der chemischen Reaktion der Cellulose. Arch. f. path. Anat. u. Phys. 1853.
Weigert, C. Beiträge zur Kenntnis der menschlichen Neuroglia. Frankfurt 1895.
Whitwell, On the Structure of the Neuroglia. British med. Jour. 12.
Wlassak, R. Die Herkunft des Myelins, Archiv f. Entwicklungsmechanik 1895.
y Cajal R. Algunas conjeturas sobre el mecanismo anatómico de la Ideacion, Asociation y Atencion. Madrid 1895 und Arch. f. Anat. 1895.
y Cajal, R. Nuevos observationes sobre la estructura de la médula espinal de les mamiferos. Barcelona, 1890.
y Cajal, R. Sur l'origine et les ramifications des fibres nerveuses de la moelle embryonnaire. Anat. Anzeiger 1890.
Yamagiva, Eine neue Färbung der Neuroglia. Virchows Archiv 160. S. 358. I900.

References Lenhossek

Arnold, Fr. Bemerkungen über den Bau des Hirns und Rückenmarks. Zürich 1838.
Arnold, Fr. Handbuch der Anatomie. Bd, 1, Freiburg i. Br. 1844.
Balfour, F. M. Handbuch der vergleichenden Embryologie. Übersetzt von C. Vetter. Jena 1881, II. Bd.
Barnes, On the Development of the posterior fissure of the Spinal cord and the Reduction of the Central Canal in the Pig. Proc. Amer. Acad. arts and sc. 1884.
Bechterew W., Über einen besonderen Bestandteil der Seitenstränge des Rückenmarks. Arch. f. Anat. und Physiol. Anat. Abt., 1886, p. 4.
Beneke, Über eine Modifikation des Weigert´schen Fibrinverfahrens. Anat. Anz. Jahrg. VIII, 1893, Suppl. p165.
Bidder, F. and Kupffer, C. Untersuchungen über die Textur des Rückenmarks, Leipzig, 1857.
Boll, F. Die Histologie und Histogenese der nervösen Centralorgane. Archiv f. Psychiatrie u. Nervenkrankh. Bd. IV, 1874.
Burckhardt, R. Histologische Untersuchungen am Rückenmarke der Tritonen. Archiv f. mikrosk. Anat., Bd. XXXIV, 1889.
Corning, H. K. Über die Entwicklung der Substantia galatinosa Rolandi beim Kaninchen. Archiv f. mikrosk. Anatomie, Bd. 31, 1888.
Deiters, O. Untersuchungen über Gehirn und Rückenmark, 1865.
Edinger, L. Vorlesungen über den Bau der nervösen Centralorgane, 4. Aufl., 1893.
Falzacappa, E. Ricerche istologiche sul midollo spinale. Rendiconto della R. Accademia dei Lincei, Vol. V. 1889.
Frommann, Untersuchungen über die normale und pathologische Anatomie des Rückenmarkes. Jena 1864.
Gierke, H. Die Stützzellen des Centralnervensystems. Archiv f. mikrosk. Anat. Bd. 25, 1885, p. 441 and Bd. 26, 1886.
Gieson, Ira van. A study of the Artefacts of the Nervous System. New York medical Journal, 1892.
Golgi, C. Contribuzione alla fina Anatomia degli organi centrali del sistemia nervosa, Rivista clinica die Bologna, 1871-1872,.
Golgi, C. Sui gliomi del cervello. Rivista sperim. di Freniatria, 1872. S. Sammelwerk.

Götte, A. Entwicklungsgeschichte der Unke. Leipzig, 1875.
Henle, J. and Merkel, Fr. Über die sog. Bindesubstanz der Centralorgane des Nervensystems. Zeitschr. f. ration. Medizin. Bd. 34, 1868.
Hensen Zeitschrift für Anatomie und Entwicklungsgeschichte, Band I, 1876.
His, W. Histogenese u. Zusammenhang der Nervenelemente. Archiv. f. anat. u. physiol. Anat. Abt. 1890.
His, W. Über ein perivaskuläres Kanalsystem in den nervösen Centralorganen und über dessen C. Beziehungen zum Lymphsystem. Zeitschr. f. wiss. Zoologie, Bd. XV, 1865.
His, W. Zur Geschichte des menschlichen Rückenmarkes und der Nervenwurzeln. Abh. d. math.-phys. Klasse d. Kgl. Sächs. Ges. d. Wiss., Bd. XIII, 1886, p. 479.
Hoche, A. Beitrag zur Kenntnis des anatomischen Verhaltens der menschlichen Rückenmarkswurzeln etc. Habilitationsschrift, Heidelberg 1891.
Hoffmann, J. Zur Lehre von der Syringomyelie. Deutsche Zeitschr. f. Nervenheilkunde, Bd. 8, 1893, p. 1.
Jastrowitz, Studien über die Encephalitis und Myelitis des ersten Kindesalters. Archiv f. Psychiatrie, Bd. III, 1871,
Keuffel, G. G. Th. Über das Rückenmark. Reil´s Archiv, Bd. 10, 1811.
Key, A. und Retzius, G. Studien in der Anatomie des Nervensystems und des Bindgewebes. I. Hälfte, Stockholm 1875.
Kölliker v., Handbuch der Gewebelehre des Menschen. 4. Auflage, 1862.
Kölliker, A. Handbuch der Gewebelehre, 6. Aufl., Bd. II, 1893.
Kölliker, A. Zur feineren Anatomie des centralen Nervensystems. Zweiter Beitrag: Das Rückenmark. Zeitsch. für wissensch. Zoologie. Bd. LI, 1890.
Krause, W. Handbuch der menschlichen Anatomie, Bd. I, Allgemeine Anatomie, Hannover 1876.
Lachi, P. Contributo alla istogenesi della nevroglia nel midollo del pollo. Memoria della Soc. Toscana di Scienca natur., Vol. 11, Pisa 1890.
Lawdowsky, M. Vom Aufbau des Rückenmarkes. Archiv f. mikrosk. Anatomie, Bd. 38, 1891, p. 264.
Lenhossék, M. v. Beobachtungen an den Spinalganglien und dem Rückenmarke von Pristiurus embryonen. Anat. Anz., Jahrg. VII, 1892,.
Lenhossek, M. v. Untersuchungen über die Entwicklung der Markscheiden und den Faserverlauf im Rückenmark der Maus. Arch f. mikrosk. Anat., Bd. 33, 1889.
Lenhossek, M. v. Zur Kenntnis der Neuroglia des menschlichen Rückenmarkes. Verhandl. d. anat. Gesellsch, 5. Versamml., 1891, Anatom. Anz.
Lenhossék, M. v. Zur Kenntnis des Rückenmarkes der Rochen. In: Beitr. z. Histolog. d. Nervensystems u. d. Sinnesorgane. Wiesbaden 1894.
Meynerth, Th. Vom Gehirn der Säugetiere. Stricker´s Handbuch der Lehre von den Geweben. Wien 1870, Bd. II,.
Nansen, Fr. Structur and Combination of the Histiological Elements of the Central Nervous System. Bergen's Museums Aarsberetning for 1886 Bergen 1887.
Obersteiner, H. Anleitung beim Studium des Baues der nervösen Centralorgane. 2. Aufl., Wien 1892.
Obersteiner, H. Über einige Lymphräume im Gehirne. Sitzungsberichte der kais. Akad. d. Wissensch. zu Wien, Bd. 61, Abt. I, 1870.
Prenant, A. Critériums histologiques pour la détermination de la partie persistante du canal épendymaire primitif. Internat. Monatsschrift f. Anatn. u. Physiol., Bd. XI, 1894.
Ramon, P. El encefalo de los reptiles. Barcelona, 1891.

Ranvier, L. De la névroglie. Comptes rendus de l´Acad. des Sc. Tome 94, 1882.
Retzius, G. Studien über Ependym und Neuroglia. Biolog. Untersuchungen, N. F., V, 1893.
Retzius, G. Zur Kenntnis der Ependymzellen der Centralorgane. Verhandl. d. Biol. Vereins in Stockholm, 1891.
Retzius, G. Zur Kenntnis des centralen Nervensystems von Myxine glutinosa. Biolog. Untersuchungen, N. F. II, Stockholm 1891.
Rohde, E. Histologische Untersuchungen über das Nervensystem vom Amphioxus lanceolatus. Schneider's Zoolog. Beitg., Bd. 2., H. 2, Breslau 1888.
Sala y Pons, Cl. Estructura de la médula espinal de los batracios. Barcelona 1892.
Sala y Pons, Cl. La Neuroglia de los Vertebrados. Barcelona 1894.
Schaffer, J. Die oberflächliche Gliahülle und das Stützgerüst des weißen Rückenmarkmantels. Anat. Anz., Bd. IX, 1894.
Schaffer, J. Beiträge zur Kenntnis des Stützgerüstes im menschlichen Rückenmarke. Arch. f. mikr. Anat., Bd. 40, 1894.
Schwalbe, G. Lehrbuch der Neurologie. Erlangen 1881.
Simon, Th. Das Spinnenzellen- und Pinselzellengliom. Archiv für pathol. Anat. u. Physiol. 1874.
Staderini, Contributo allo studio del tessuto interstiziale di alcuni nervi craniensi dell' uomo. Monitore zoolog. italiano, Anno I, 1890.
Stilling and Wallach, Untersuchungen über die Textur des Rückenmarks. Leipzig 1842.
Van Gehuchten, A. La Structure des centres nerveux. La moëlle épinière et le cervelet. La Cellule, T. VII, 1891.
Van Gehuchten, A. Le système nerveux de l'homme. Lierre 1893.
Vignal, W. Sur le développement des éléments de la moëlle des mammifères. Archives de Physiologie normale et pathologique, Tome 1884.
Virchow, H. Über Zellen in der Substantia gelatinosa Rolando. Reported in Neurol. Centralbl., 1887, p. 263.
Virchow, R. Gesammelte Abhandlungen. Frankfurt, 1856.
Virchow, R. Über eine im Gehirn und Rückenmark gefundene Substanz mit der chemischen Reaktion der Cellulose. Archiv f. pathol. Anat. u. Physiol. Bd. VI, 1853,.
Wagner, R. Neurologische Bemerkungen. Göttinger Nachrichten, 1854
Waldeyer, W. Über die Entwicklung des Centralkanals im Rückenmark. Archiv f. path. Anat., 1876, Bd. LXVIII,.
Weigert, C. Bemerkungen über das Neurogliagerüst des menschlichen Centralnervensystems. Anat. Anz. Jahrg. V, 1890.
Wilson, J. T. On the Closure of the central canal of the spinal cord in the foetal lamb. Transact. Intern. med. Congress Sydney 1892.
y Cajal, S. R. Pequeñas comunicaciones al conocimiento del sistema nervioso. La médula espinal de los reptiles. Barcelona 1891.
y Cajal, S.R. Sur l'origine et les remifications des fibres nerveuses de la moëlle embryonnaire. Anat. Anz. Jahrg. V, 1890.
y Cajal, S.R. Nuevos observaciones sobre la estructura de la médula espinal de los mamiferos. Barcelona, 1890.
y Cajall S. R., Significacion fisiologica de las expansions protoplasmaticas y nerviosas de la celulas de la sustancia gris. Rivista de ciencias medicas de Barcelona, 1891, Nr. 22 and 23.

References Retzius V

Gierke, H. Die Stützsubstanz des Centralnervensystems. Archiv f. mikrosk. Anatomie. Bd. 25, 1885.

Golgi, Camillo Sulla fina anatomia degli organi centrali del sistema nervosa. Reggio Emilia, 1885. I have used the German translation provided by the author, which was published in Anatom. Anzeiger, Jahrg. V, No13 & 14, Juli 1890.

Kölliker, A. von. Zur feinen Anatomie des centralen Nervensystems. Zweiter Beitrag: Das Rückenmark. Zeitschr. f. wissensch. Zoologie. Bd 51, 1890-

Lachi, Pilade. Contributo alla istogenesi della Nevroglia nel midillo spinale del pollo. Atti della Societa toscana di scienze naturali, resid. In Pisa. Memorie. Vol. 11, 1891.

Lenhossek, M. von. Beobachtungen an den Spinalganglien und dem Rückenmark von Pristiurusembryonen. Anat. Anzeiger, 7. Jahrg., 1892.

Lenhossek, M. vo.n Der feinere Bau des Nervensystems im Lichte neuster Forschungen. Fortschritte der Medizin, 1892.

Lenhossek, Mich. von. Zur Kenntnis der Neuroglia des menschlichen Rückenmarks. Verhandl. d. Anatomischen Gesellschaft auf d. fünften Versamml. in München. 18.-19- Mai 1891.

Lenhossek. Mich. Von. Zur ersten Entstehung der Nervenzellen und Nervenfasern bei dem Vogelembryo. Mittheil. Aus dem anatom. Institut im Vesalianum zu Basel, 1890.

Martinotti, Carlo. Beitrag zum Studium der Hirnrinde und dem Centralursprung der Nerven. Internat. Monatsschrift f. Anat. und Phys. Bd 7, 1890.

Nansen, Fridthof. The Structure and Combination of the Histological Elements of the Central Nervous System. Bergens Museums Aarsberetning for 1886. Bergen 1887.

Oyarzun, A. Über den feineren Bau des Vorderhirns der Amphibien. Arch. f. mikrosk. Anatomie. Bd35, 1890.

Ramon, Pedro. El encefalo de los Reptiles. Trab. d. Laborat. histologia de la facultad de medicina de Zaragoza. Sept. 1891.

Retzius, Gustav. Ueber den Bau der Oberflächenschicht der Grosshirnrinde beim Menschen und bei den Säugethieren, ebenda (same reference).

Retzius, Gustav. Zur Kenntnis des Nervensystems von Myxine glutinosa. Biol. Unters. N. F. II, 2. 1892.

Retzius, Gustav. Zur Kenntnis der Ependymzellen der Centralorgane. Verhandl. d. Biolog. Vereins in Stockholm. Bd. 3, 1890-91 (1. März 1891)

Sala, Cl. Estruetura de la medulla espinal de los batracios. Trab. d. Laborat. histologia de la facultad de medicina de Barcelona. Febr. 1892.

Van Gehuchten, A. La structure des centres nerveux. La Moelle epiniere et le cervelet. La Cellule, t. 7, dep. 1. 20 avril 1891.

y Cajal, Ramon Sur l´origine et les ramifications des fibres nerveuses de la mole embryonaire. Anatom. Anzeiger, V. Jahrg. No 4. Febr. 1890.

y Cajal, S. Ramon Pequenas Contribuciones al Conecimiento del sisterna nervioso. Trab. d. Laborat. histol. de la facultad de medicina de Barcelona. Aug. 1891.

References Retzius VI

Andriezen, W. Lloyd. On a system of fiber-cells surrounding the blood vessels of the Brain of Man and Mammals, and its Physiological Significance. Intern. Monatsschr. f. Anat. u. Physiol. Bd10, 1893 – British Medical Journal, July 1893.
Azoulay, L. Note sur le aspects des cellules nevroglique dans les organs nerveux centraux de l´enfant, Comptes rend. Hebd. D. s. de la Soc. De Biol., 1894, No 9.
Berkley, Henry J. The Neuroglia Cells of the Walls of the middle Ventricle in the adult Dog. Anatom. Anzeiger, Bd 9, No 24 and 25, Aug. 1894.
Dogiel, A. S. Neuroglia der Retina des Menschen. Arch. F. mikrosk. Anat. Bd 41, 1893.
Gierke, Hans. Die Stützsubstanz des Centralnervensystems, Archiv f. mikrosk. Anatomie, Bd 25, 1885 and Bd 26, 1896.
Golgi, Camillo. Contribuzione alla fina anatomia degli organi centrali des Sistema nervosa, Rivista clinica di Bologna. Ser. 2 A, 1, fasc. 11, novembre 1871 and A., 2, fasc. 12, dic. 1871.
Golgi, Camillo. Sulla fina anatomia degli organi centrali del Sistema nervosa, Reggio Emilia, 1885, see also the above mentioned edition of Golgi´s reports in German language, 1894.
Golgi, Camillo. Untersuchungen über den feineren Bau des centralen und peripherischen Nervensystems. Aus dem Italienischen übersetzt von Dr. R. Teutscher. Verlag von Gustav Fischer. Jena, 1894.
Greppin, L. Ueber die Neuroglia der menschlichen Rinde. Anatom. Anzeiger, Bd9, No 3, Nov. 1893.
Kalius, E. Ueber Neurogliazellen in peripherischen Nerven, Nachrichten von d. k. Gesellsch. d. Wiss. etc. In Göttingen 1892.
Kalius, E. Untersuchungen zur Netzhaut der Säugetiere. Anatom. Hefte, herausgeg. Von Merkel Und Bonnet, 1894.
Key, Axel and Retzius, Gustav Studien in der Anatomie des Nervensystems und des Bindegewebes. Bd. I. 1875.
Kölliker, A. von Handbuch der Gewebelehre des Menschen. 6. Auf., II Bd, 1, 1893.
Lenhossek, M. v. Zur Kentnis der Neuroglia des menschlischen Rückenmarkes. Verh. D. Anatom. Gesellsch. Auf d. fünften Versamml. In München, 1891.
Lothringer, S. Untersuchungen an der Hypophyse einiger Säugetiere und des Menschen. Archiv f. Mikrosk. Anatomie, Bd 28, 1886.
Lugaro, Ernst. Über die Histogenese der Körner der Kleinhirnrinde. Anatom. Anzeiger, Bd 9, No 23, Aug. 1894.
Magini, Guiseppe. Neuroglia e cellule nervosa cerebrali nei feti. Att d 12. Congresso della Assoc. med. Ital. in Pavia, sett. 1887, Vol1, Pavia 1888.
Martinotti, Carlo. Beitrag zum Studium der Hirnrinde und dem Centralursprung der Nerven. Inernat. Monatschr. für Anatomie und Physiologie, Bd 7, 1890.
Michel, Sitz, Ber. D. Würzb. Med. naturwiss. Gesellsch. 14. Jan, 1893.
Mondino, Casimiro. Richerche macro e microscopiche sui centri nervosa, Torino 1887.
Petrone, Louis Sur la structure des nerfs cerebro-rachidiens. Intern. Monatsschr. F. Anat. Physiol. Bd 5. 1888
Rauber, A. Lehrbuch der Anatomie des Menschen, 4. Aufl., II, 2, I, 1894.
Retzius, Gustav. Über den Bau der Oberfächenschicht der Grosshirnrinde beim Menschen und bei einigen Säugetieren. Verhandl. d. Biololg. Vereins in Stockholm. Bd 3, März 1891.
Retzius, Gustav. Studien über Ependym und Neuroglia. Biolog. Untersuchungen von Gustav Retzius. N. F. Bd V, 2, 1883.

Sala y Pons, Claudio. La Neuroglia de los Vertebrados. Barcelona 1894.
Schäfer, E. A. Quain´s Elements of Anatomy, Vol. 3, P. 1; 10. Edition, 1893.
Schwalbe, G. Lehrbuch der Neurologie, 2. Bd, 2. Abth. Von Hoffmann´s Lehrb. D. Anat. D. Menschen, 1881.
Toldt, C. Lehrbuch der Gewebelehre, 3. Aufl., 1888.
Van Gehuchten, A. La Neuroglie dans le cervelet de l´homme. Bibiolgraphie anatomique, Année 2, No 4, 1894.
Van Geruchten, A. La structure des centres nerveux. La moelle epiniere et le cervelet. La Cellule. T. 7, 1, 1891.
Van Geruchten, A. Le systeme nerveux de l´homme, Lecous professes a l´Universite de Louvain, 1893
y Cajal, S. Ramon Estructura del asta de Amon y fascia dentata. Trabajos leidos ante la Sociedad espanola de historia natural, 1893.
y Cajal, S. Ramon La retine des vertebres, la Cellule t. 9, 1, 1893 (Dep. 1892).
y Cajal, S. Ramon Les novelles idees sur la structure du systeme nerveux chez l´homme et les vertebres. Paris 1894.
y Cajal, S. Ramon Sur la structure de l´ecorce cerebrale de quelque mammiferes. La Cellule t. 7, 1891.
y Cajal. S. Ramon A propos de certaius elements bipolaires du cervelet, avec quelques details sur l´evolution des fibres cerebelleuses. Internat. Monatschr. für Anatomie und Physiologie, Bd7, 1890.

References Weigert

Andriezen, Lloyd. On a system of fiber-cells surrounding the blood-vessels of the brain of Man and Mammals, Internationale Monatsschrift für Anatomie und Physiologie, 1893, p. 539.
Arndt. Zur Histologie des Gehirns, Archiv für Psychiatrie, Volume III, p. 470 f.
Bidder and Kupffer Untersuchungen über die Textur des Rückenmarks und die Entwicklung seiner Formelemente, 1857
Boll. Die Histologie und Histogenese der nervösen Zentralorgane, Archiv für Psychiatrie, Vol. 4, 1874, p. 1 ff.
Brissaud. neurologique Vol. 2, p. 545 ff.
Clarke , J. L. Philosophical transactions, 1859, p. 437 ff.
Colella. Sur l'histogenèse de la névrologie dans la moelle épinière, Archives ital. de Biologie, Vol. 20, p. 212 ff.
Deiters, O. Untersuchungen über Gehirn und Rückenmark des Menschen und der Säugetiere. Braunschweig, 1865.
Frommann, C. Untersuchungen über die normale und pathologische Anatomie des Rückenmarks", Part I, Jena, 1864. Part II, Jena, 1877.
Gierke Die Stützsubstanz des Zentralnervensystems, Archiv für mikroskopische Anatomie, Vol. 25, p. 441.
Golgi, C. Beitrag zur feineren Anatomie des Zentralnervensystems, Bologna, 1871. Cited from the "Untersuchungen über den feineren Bau des zentralen und peripherischen Nervensystems"], Jena, 1894
Greeff Die Spinnenzellen — Neurogliazellen — in Sehnerv und der Retina. Archiv für Augenheilkunde, Vol. 29, p. 11

Henle, J. and Merkel Über die sogenannte Bindesubstanz der Zentralorgane des Nervensystems, Zeitschrift für rationelle Medizin3rd edition, Vol 34 (1863).
Keuffel Über das Rückenmark Reils und Authenrieds Archiv" [Archive of Reil and Authenried], Vol. X.
Kultschitzky Über eine Färbungsmetbode der Neuroglia, Anatomischer Anzeiger, Vol 8, 1898.
Lavdowsky Vom Aufbau des Rückenmarks, Archiv für mikroskopische Anatomie Vol. 38 (1891)
Lissauer, Arch. für Psych. Vol. 17, Book 2., p. 12, Sept. ed.
Paladino. Dei limiti precisi tra il nevroglio e gli elementi nervrosi del midollo spinale, R. acad. di Roma, XIX. Fasc. 2, 1893.
Popoff. De la névrologie et de sa distribution dans les régions du bulbe et de la protubérance chez l'homme adulte, Arch. de psych., de neurologie et de médecine légales, 1893. vol. 11, p. 1.
Ranvier. De la névroglie, Archive de Physiologie normale et pathologique, February 15, 1883.
Ranvier. De la névroglie, Comptes rendus, June 5, 1892.
Rohde. Ganglienzellen und Neuroglia, Archiv für mikroskopische Anatomie], Vol. 42.
Roth. Zur Frage der Bindesubstanz in der Großhirnrinde, Virchows archiv, Vol. 46 (1869).
Sala y Pons. La Neuroglia de los Vertebrado, Madrid, 1904,.
Schwalbe. Handbuch der Augenheilkunde by Gräfe and Sämisch, Vol. I, p. 342, Leipzig, 1874
Stricker and Unger. Untersuchungen über den Bau der Großhirnrinde, Wiener Sitzungsberichte" Vol. 80, 1879.
van Gieson. Laboratory notes of technical methods for the nervous system, New York medical Journ., 1889.
Vignal. Archives de Physiologie. 1884.
Virchow, R. Über das granulierte Ansehen der Wandungen der Gehirnventrikel, Zeitschrift für Psychiatrie 1846
Weigert, C. Zur pathologischen Histologie des Neurogliafäsergerüsts, Centralblatt für allg. Path. und path Anat, 1890, p. 736 f.
Weigert, C. Bemerkungen über das Neurogliagerüst des menschlichen Zentralnervensystems, Anatomischer Anzeiger, 1890, p. 543 ff
Weigert, C. Zur pathologischen Histologie des Neurogliafäsergerüsts, Zentralblatt für allgemeine Pathologie und pathologische Anatomie, 1890, p. 729 ff.

All references in alphabetical order (178)

Aguerre. Untersuchungen über die menschliche Neuroglia. Arch. f. mikr. Anat. 56. 1900.
Andriezen, Lloyd. On a system of fiber-cells surrounding the blood-vessels of the brain of Man and Mammals, Internationale Monatsschrift für Anatomie und Physiologie, 1893
Apáthy, Stephan. Das leitende Element des Nervensystems und seine topographischen Beziehungen zu den Zellen. Mitt. d. zool. Station XII. 1897.
Arndt Zur Histologie des Gehirns, Archiv für Psychiatrie, Volume III,.
Arnold, Fr. Bemerkungen über den Bau des Hirns und Rückenmarks. Zürich 1838.
Arnold, Fr. Handbuch der Anatomie. Bd, 1, Freiburg i. Br. 1844.
Azoulay, L. Note sur le aspects des cellules nevroglique dans les organs nerveux centraux de l´enfant, Comptes rend. Hebd. D. s. de la Soc. De Biol., 1894, No 9.
Balfour, F. M. Handbuch der vergleichenden Embryologie. Übersetzt von C. Vetter. Jena 1881, II. Bd.

Barnes, On the Development of the posterior fissure of the Spinal cord and the Reduction of the Central Canal in the Pig. Proc. Amer. Acad. arts and sc. 1884.

Bechterew W., Über einen besonderen Bestandteil der Seitenstränge des Rückenmarks. Arch. f. Anat. und Physiol. Anat. Abt., 1886.

Beneke, Über eine Modifikation des Weigert´schen Fibrinverfahrens. Anat. Anz. Jahrg. VIII, 1893, Suppl.

Bergmann, C. Zeitschrift f. rat. Medicin. N. F. VIII. 1857

Berkley, Henry J. The Neuroglia Cells of the Walls of the middle Ventricle in the adult Dog. Anatom. Anzeiger, Bd 9, No 24 and 25, Aug. 1894.

Bethe, Albrecht. Über die Neurofibrillen in den Ganglienzellen von Wirbeltieren und ihre Beziehungen zu den Golginetzen. Archiv für mikrosk. Anat. 55. 1900.

Bidder, F. and Kupffer, C. Untersuchungen über die Textur des Rückenmarks und die Entwicklung seiner Formelemente, 1857

Binswanger und Berger, Beiträge zur Kenntnis der Lymphzirkulation in der Großhirnrinde. Virchows Archiv 152. 1898.

Boll, F. Die Histologie und Histogenese der nervösen Zentralorgane, Archiv für Psychiatrie, Vol. 4, 1874.

Boll, F. Die Histiologie und Histiogenese der nervösen Centralorgane. Berlin 1873.

Brissaud, neurologique Vol. 2.

Brodmann, K. Über den Nachweis von Astrocyten mittelst der Weigertschen Gliafärbung. Jenaische Zeitschr. f. Naturw. XXXIII. I899. 1.

Burckhardt, R. Histologische Untersuchungen am Rückenmarke der Tritonen. Archiv f. mikrosk. Anat., Bd. XXXIV, 1889.

Capobianco. Della partecipazione mesodermica nelle genesi della neuroglia cerebrale. Monit. zool. ital. XII. 1901 u. Arch. ital. biol. 1902.

Clarke , J. L. Philosophical transactions, 1859.

Colella. Sur l'histogenèse de la névrologie dans la moelle épinière, Archives ital. de Biologie, Vol. 20.

Corning, H. K. Über die Entwicklung der Substantia galatinosa Rolandi beim Kaninchen. Archiv f. mikrosk. Anatomie, Bd. 31, 1888, p. 594.

Deiters, O. Untersuchungen über Gehirn und Rückenmark des Menschen und der Säugetiere. Braunschweig, 1865.

Dimitrova, Z. Recherehes sur la structure de Ia Glande pinéale. Le Nevraxe. II. 1901.

Dogiel, A. S. Neuroglia der Retina des Menschen. Arch. F. mikrosk. Anat. Bd 41, 1893.

Eberth, C. J. Über die Blut- u. Lymphgefäße des Gehirns u. Rückenmarks. Virchows Archiv 49. 1870.

Edinger, L. Vorlesungen über den Bau der nervösen Centralorgane, 4. Aufl., 1893

Eurich, Studies on the Neuroglia, Brain 1897.

Falzacappa, E. Ricerche istologiche sul midollo spinale. Rendiconto della R. Accademia dei Lincei, Vol. V. 1889, p. 696.

Flemming, W. Über die Entwicklung der kollagenen Bindegewebsfibrillen bei Amphibien und Säugetieren. Arch. f. Anat. u. Phys. anat. Abt. 1897.

Frommann, C. Untersuchungen über die normale und pathologische Anatomie des Rückenmarks", Part I, Jena, 1864. Part II, Jena, 1877.

Fuchs, H. Über das Ependym. Verb. d. anatom. Gesellschaft zu Halle 1902.

Gardner, M. Zur Frage über die Histogenese des elastischen Gewebes, Biol. Zentralbl. 1897.

Gaskell, W. H. Über die Wand der Lymphkapillaren (C. Ludwig, Arbeiten aus d. phys. Institut zu Leipzig. 1877.
Gierke, Hans. Die Stützzellen des Centralnervensystems. Archiv f. mikrosk. Anat. Bd. 25, 1885 and Bd. 26, 1886.
Gieson, Ira van. A study of the Artefacts of the Nervous System. New York medical Journal, 1892.
Golgi, C. Sui gliomi del cervello. Rivista sperim. di Freniatria, 1872. S. Sammelwerk.
Golgi, Camillo. Sulla fina anatomia degli organi centrali del sistema nervosa. Reggio Emilia, 1885.
Golgi, Camillo. Untersuchungen über den feineren Bau des centralen und peripherischen Nervensystems. Aus dem Italienischen übersetzt von Dr. R. Teutscher. Verlag von Gustav Fischer. Jena, 1894.
Götte, A. Entwicklungsgeschichte der Unke. Leipzig, 1875.
Greeff Die Spinnenzellen — Neurogliazellen — in Sehnerv und der Retina. Archiv für Augenheilkunde, Vol. 29.
Greppin, L. Ueber die Neuroglia der menschlichen Rinde. Anatom. Anzeiger, Bd9, No 3, Nov. 1893.
Hardesty, lrving. The Neuroglia of the Spinal Cord of the Elephant with some preliminary Observation upon the Developement of Neuroglia Fibres. American Journal of Anatomy II. Nr. I.
Hatai, Shinkishi. On the origin of nevrologia tissue from the mesoblast. Journ. of comp. neurology XII. 1902.
Held, H. II. Abhandlung über Nervenzellenstrukturen. Arch. f. Anatomie 1897, Suppl. S. 275 Anm.
Held, Hans Über den Bau der grauen und weißen Substanz I. Arch. f. Anatomie. 1902.
Henle and Merkel Über die sogenannte Bindesubstanz der Zentralorgane des Nervensystems, Zeitschrift für rationelle Medizin3rd edition, Vol 34 (1863).
Hensen Zeitschrift für Anatomie und Entwicklungsgeschichte, Band I, 1876.
His, W. Beiträge zur Kenntnis der zum Lymphsystem gehörigen Drüsen. Zeitschr. f. wiss. Zool. X. 1859.
His, W. Die Neuroblasten und deren Entstehung im embryonalen Mark. Abh. d. math. phys. Kl. d. Kgl. s. Ges. d. W. 1889.
His, W. Histogenese u. Zusammenhang der Nervenelemente. Archiv. f. anat. u. physiol. Anat. Abt. 1890, p. 103.
His, W. Über das Auftreten der weißen Substanz und der Wurzelfasern am Rückenmark menschlicher Embryonen. Arch. f. Anatomie 1883.
His, W. Über das Epithel der Lymphgefäßwurzeln und über die v. Recklinghausenschen Saftkanälchen. Zeitschr. f. wissensch. Zoologie. 1863.
His, W. Über die Wurzeln der Lymphgefäße in den Häuten des Körpers und über die Theorien der Lymphbildung. Zeitschr. f. wiss. Zool. XII. 1862.
His, W. Über ein perivaskuläres Kanalsystem in den nervösen Centralorganen und über dessen Beziehungen zum Lymphsystem. Zeitschr. f. wiss. Zoologie, Bd. XV, 1865.
His, W. Zur Geschichte des menschlichen Rückenmarkes und der Nervenwurzeln. Abh. d. math.-phys. Klasse d. Kgl. Sächs. Ges. d. Wiss., Bd. XIII, 1886.
Hoche, A. Beitrag zur Kenntnis des anatomischen Verhaltens der menschlichen Rückenmarkswurzeln etc. Habilitationsschrift, Heidelberg 1891.
Hoffmann, J. Zur Lehre von der Syringomyelie. Deutsche Zeitschr. f. Nervenheilkunde, Bd. 8, 1893.
Holmgreen, Emil. Studien in der feineren Anatomie der Nervenzellen. Anat. Hefte XV. 1900.

Huber, G. Carl Studies of Neuroglia. The American Journal of Anatomy. I. 1901.
Jastrowitz, Studien über die Encephalitis und Myelitis des ersten Kindesalters. Archiv f. Psychiatrie, Bd. III, 1871.
Kalius, E. Ueber Neurogliazellen in peripherischen Nerven, Nachrichten von d. k. Gesellsch. d. Wiss. etc. In Göttingen 1892.
Kalius, E. Untersuchungen zur Netzhaut der Säugetiere. Anatom. Hefte, herausgeg. Von Merkel Und Bonnet, 1894.
Keuffel, G. G. Th. Über das Rückenmark. Reil´s Archiv, Bd. 10, 1811.
Key, A. und Retzius, G. Studien in der Anatomie des Nervensystems und des Bindgewebes. I. Hälfte, Stockholm 1875.
Kölliker A. v., Handb. d. Gewebelehre, 6. Aufl., Bd. II, 1893,.
Kölliker, A. Zur feineren Anatomie des centralen Nervensystems. Zweiter Beitrag: Das Rückenmark. Zeitsch. für wissensch. Zoologie. Bd. LI, 1890.
Krause, W. Handbuch der menschlichen Anatomie, Bd. I, Allgemeine Anatomie, Hannover 1876.
Kultschitzky. Über eine Färbungsmetbode der Neuroglia, Anatomischer Anzeiger, Vol 8, 1898.
Kure, S. Über die Beziehungen der Glia zu den Gefäßen. Neurologia I.
Lachi, P. Contribution à l'histogenèse de la nevrologie dans la moelle épinière du poulet. Arch. it. biol. XV. 1891.
Lachi, P. Contributo alla istogenesi della nevroglia nel midollo del pollo. Memoria della Soc. Toscana di Scienca natur., Vol. 11, Pisa 1890.
Lachi, Pilade. Contributo alla istogenesi della Nevroglia nel midillo spinale del pollo. Atti della Societa toscana di scienze naturali, resid. In Pisa. Memorie. Vol. 11, 1891.
Lawdowsky, M. Vom Aufbau des Rückenmarkes. Archiv f. mikrosk. Anatomie, Bd. 38, 1891.
Lenhossek, M. v. Untersuchungen über die Entwicklung der Markscheiden und den Faserverlauf im Rückenmark der Maus. Arch f. mikrosk. Anat., Bd. 33, 1889.
Lenhossek, M. v. Zur Kenntnis der Neuroglia des menschlichen Rückenmarkes. Verhandl. d. anat. Gesellsch, 5. Versamml., 1891, Anatom. Anz.
Lenhossék, M. v. Zur Kenntnis des Rückenmarkes der Rochen. In: Beitr. z. Histolog. d. Nervensystems u. d. Sinnesorgane. Wiesbaden 1894.
Lenhossek, M. v. Beobachtungen an den Spinalganglien und dem Rückenmark von Pristiurus embryonen. Anat. Anzeiger, 7. Jahrg., 1892.
Lenhossek, M. v. Der feinere Bau des Nervensystems im Lichte neuster Forschungen. Fortschritte der Medizin, 1892.
Lenhossek. M. v. Zur ersten Entstehung der Nervenzellen und Nervenfasern bei dem Vogelembryo. Mittheil. Aus dem anatom. Institut im Vesalianum zu Basel, 1890.
Lewis, Bevan. A text book of mental diseases with special reference to the pathological aspects of insanity. London 1889.
Lissauer, Arch. für Psych. Vol. 17, Book 2., p. 12, Sept. ed.
Lothringer, S. Untersuchungen an der Hypophyse einiger Säugetiere und des Menschen. Archiv f. Mikrosk. Anatomie, Bd 28, 1886.
Lugaro, Ernst. Über die Histogenese der Körner der Kleinhirnrinde. Anatom. Anzeiger, Bd 9, No 23, Aug. 1894.
Magini, Guiseppe. Neuroglia e cellule nervosa cerebrali nei feti. Att d 12. Congresso della Assoc. med. Ital. in Pavia, sett. 1887, Vol1, Pavia 1888.

Marinesco, Du rôle de la nevroglie dans l'évolution des inflammations. 2. internat. med. Congress. Paris 1900. (n. d. Jahresbericht v. Schwalbe. VI. 1900.)
Martinotti, Carlo Beitrag zum Studium der Hirnrinde und dem Centralursprung der Nerven. Inernat. Monatschr. für Anatomie und Physiologie, Bd 7, 1890.
Meynerth, Th. Vom Gehirn der Säugetiere. Stricker´s Handbuch der Lehre von den Geweben. Wien 1870, Bd. II.
Michel. Sitz, Ber. D. Würzb. Med. naturwiss. Gesellsch. 14. Jan, 1893.
Mondino, Casimiro. Richerche macro e microscopiche sui centri nervosa, Torino 1887.
Müller, E. Studien über Neuroglia. Arch. f. mikr. Anatomie. 55. 1899.
Nansen, Fridthof. The Structure and Combination of the Histological Elements of the Central Nervous System. Bergens Museums Aarsberetning for 1886. Bergen 1887.
Nissl, Fr. Über einige Beziehungen zwischen Nervenzellerkrankungen und gliösen Erscheinungen bei verschiedenen Psychosen. Arch. f. Psychiatrie 1899.
Obersteiner, H. Anleitung beim Studium des Baues der nervösen Centralorgane. 2. Aufl., Wien 1892.
Obersteiner, H. Über einige Lymphräume im Gehirne. Sitzungsberichte der kais. Akad. d. Wissensch. zu Wien, Bd. 61, Abt. I, 1870.
Obersteiner, H. Zur Histologie der Gliazellen in der Molekularschichtder Großhirminde. Arb. a. d. Institut. 1900.
Oyarzun, A. Über den feineren Bau des Vorderhirns der Amphibien. Arch. f. mikrosk. Anatomie. Bd35, 1890.
Paladino, S. Dei limiti precisi tra il nevroglio e gli elementi nervrosi del midollo spinale, R. acad. di Roma, XIX. Fasc. 2, 1893.
Paladino, S. Sur les limites précises entre la névroglie et les éléments nerveux dans la moelle epiniere, et sur quelques-unes des questions histophysiologi ques qui s'y rapportent. Ach. ital. de Biologie XXII. 1895.
Pellizzini, S. B. Sulla struttura e sull' origine delle granulazioni ependimali. Contributo all Histologia e patologia della nevrologia. Riv. sper. Frenatria 22. 1896.
Petrone, Louis Sur la structure des nerfs cerebro-rachidiens. Intern. Monatsschr. F. Anat. Physiol. Bd 5. 1888
Popoff De la névrologie et de sa distribution dans les régions du bulbe et de la protubérance chez l'homme adulte, Arch. de psych., de neurologie et de médecine légales, 1893. vol. 11.
Pranter, V. Zur Färbung der elastischen Fasern. Zentralbl. für allg. Path. u. path. Anat. XIII. 1902.
Prenant, A. Critériums histologiques pour la détermination de la partie persistante du canal épendymaire primitif. Internat. Monatsschrift f. Anatn. u. Physiol., Bd. XI, 1894.
Ramon, Pedro El encefalo de los Reptiles. Trab. d. Laborat. histologia de la facultad de medicina de Zaragoza. Sept. 1891.
Ranvier, L. De la névroglie, Archive de Physiologie normale et pathologique, February 15, 1883.
Ranvier, L. De la névroglie. Comptes rendus de l´Acad. des Sc. Tome 94, 1882, p.1536.
Reinke, F. Beiträge zur Histologie des Menschen II. Arch. f. mikr. Anatomie. 50. 1897.
Reinke, Zellstudien, Arch. f. mikr. Anatomie 1894.
Renault, Insertion sous forme de revêtement épithélial continu des pieds des fibres neurogliques sur la limitante marginale d'un nevraxe adulte. Comptes rend. hebdom. des sc. T. 126.
Retzius, Gustav. Studien über Ependym und Neuroglia. Biolog. Untersuchungen, N. F., V, 1893,.

Retzius, Gustav. Zur Kenntnis des centralen Nervensystems von Myxine glutinosa. Biolog. Untersuchungen, N. F. II, Stockholm 1891.
Retzius, Gustav. Ueber den Bau der Oberflächenschicht der Grosshirnrinde beim Menschen und bei Säugetieren. Verhandl. D. Biolog. Vereins in Stockholm. Bd 3, 1891.
Retzius, Gustav. Zur Kenntnis der Ependymzellen der Centralorgane. Verhandl. d. Biolog. Vereins in Stockholm Bd 3 (1890-1891); 15 März 1891.
Retzius, Gustav. Zur Kenntnis des Nervensystems von Myxine glutinosa. Biol. Unters. N. F. II, 2. 1892.
Riedel, Die perivasculären Lymphgefäße im Zentralnervensystem und der Retina. Arch. f. mikr. Anatomie 1870.
Rieder. Beiträge zur Histologie und pathol. Anatomie der Lymphgefäße und Venen. Zentralbl. f. allg. Path. 1898.
Robertson, W. F. Note on Weigerts theory regarding the structure of the Neuroglia. Journ. of Mental Science I897.
Robin, H. first in Segond, Le systeme capillaire sanguin., Paris 1853, then in Comptes rendus et mém. de la Soc. de Biol. Paris 1855 u. Recherches sur quelques Particularités de la Structure des Capillaires de l'éncephale. Journal de la Physiol. 1859 (S. 543-545).
Rohde, E. Histologische Untersuchungen über das Nervensystem vom Amphioxus lanceolatus. Schneider's Zoolog. Beitg., Bd. 2., H. 2, Breslau 1888.
Rohde, Ganglienzellen und Neuroglia, Archiv für mikroskopische Anatomie], Vol. 42.
Roth. Zur Frage der Bindesubstanz in der Großhirnrinde, Virchows Archiv, Vol. 46 (1869),.
Sala y Pons, Cl. Estructura de la médula espinal de los batracios. Barcelona 1892.
Sala y Pons, Cl. La Neuroglia de los Vertebrados. Barcelona 1894.
Sala y Pons,Cl. La Neuroglia de los Vertebrado, Madrid, 1904.
Sala y Pons, Cl. Estruetura de la medulla espinal de los batracios. Trab. d. Laborat. histologia de la facultad de medicina de Barcelona. Febr. 1892.
Schäfer, E. A. Quain´s Elements of Anatomy, Vol. 3, P. 1; 10. Edition, 1893.
Schaffer, J. Beiträge zur Kenntnis des Stützgerüstes im menschlichen Rückenmarke. Arch. f. mikr. Anat. Bd. 40, 1894.
Schaffer, J. Die oberflächliche Gliahülle und das Stützgerüst des weißen Rückenmarkmantels. Anat. Anz., Bd. IX, 1894.
Schwalbe, G. Lehrbuch der Neurologie, 2. Bd, 2. Abth. Von Hoffmann´s Lehrb. D. Anat. D. Menschen, 1881.
Schwalbe, G. Lehrbuch der Neurologie. Erlangen 1881.
Schwalbe, Handbuch der Augenheilkunde by Gräfe and Sämisch, Vol. I, p. 342, Leipzig, 1874.
Simon, Ch. Recherches sur la cellule des ganglions sympatbiques des Hirudinées. Internationale Monatsschr. f. An. u. Phys. XIII. 1896.
Simon, Th. Das Spinnenzellen- und Pinselzellengliom. Archiv für pathol. Anat. u. Physiol. 1874.
Spuler, A. Beiträge zur Histologie und Histogenese der Binde- und Stützsubstanz. Anat. Hefte 1896.
Staderini, Contributo allo studio del tessuto interstiziale di alcuni nervi craniensi dell' uomo. Monitore zoolog. italiano, Anno I, 1890, p. 232.
Stilling and Wallach. Untersuchungen über die Textur des Rückenmarks. Leipzig 1842, p. 23.
Storch, Über die pathologisch-anatomischen Vorgänge am Stützgerüst des Zentralnervensystems. Virchows Archiv. 157. 1899.

Stricker and Unger. Untersuchungen über den Bau der Großhirnrinde, Wiener Sitzungsberichte" Vol. 80, 1879.
Ströbe, Über Struktur pathologischer Neurogliawucherungen. Zentralblatt f. allg. Path. u. path. Anat. 1896.
Studnička. Untersuchungen über den Bau des Ependyms der nervösen Zentralorgane. Anatomische Hefte. Bd. XV. 1900.
Toldt, C. Lehrbuch der Gewebelehre, 3. Aufl., 1888.
Valenti, G. Contribution à l'histogenèse de la cellule nerveuse et de la nevrologie du cerveau de certains poissons chondrostéiques. Arch. it. biol. XVI. 1891.
Van Gehuchten, A. La Neuroglie dans le cervelet de l´homme. Bibiolgraphie anatomique, Année 2, No 4, 1894.
Van Gehuchten, A. La structure des centres nerveux. La Moelle epiniere et le cervelet. La Cellule, t. 7, dep. 1. 20 avril 1891.
Van Geruchten, A. Le systeme nerveux de l´homme, Lecous professes a l´Universite de Louvain, 1893
van Gieson Laboratory notes of technical methods for the nervous system, New York medical Journ., 1889.
Vignal, W. Sur le dévelopment des éléments de la moëlle des mammiféres. Archives de Physiol. normale et pathol. T. 1884.
Virchow, R. Über das granulierte Ansehen der Wandungen der Gehirnventrikel, Zeitschrift für Psychiatrie, 1846
Virchow, H. Über Zellen in der Substantia gelatinosa Rolando. Reported in Neurol. Centralbl., 1887.
Virchow, R. Gesammelte Abhandlungen. Frankfurt, 1856.
Virchow, R. Über die Erweiterung kleinerer Gefäße. Virchows Archiv III. 1851.
Virchow, R. Über eine im Gehirn und Rückenmark gefundene Substanz mit der chemischen Reaktion der Cellulose. Arch. f. path. Anat. u. Phys. 1853.
Wagner. R. Neurologische Bemerkungen. Göttinger Nachr. 1854, Nr. 3.
Waldeyer, W. Über die Entwicklung des Centralkanals im Rückenmark. Archiv f. path. Anat., 1876, Bd. LXVIII.
Weigert, C. Zur pathologischen Histologie des Neurogliafäsergerüsts, Centralblatt für allg. Path. und path Anat, 1890.
Weigert, C. Bemerkungen über das Neurogliagerüst des menschlichen Zentralnervensystems, Anatomischer Anzeiger, 1890.
Weigert, C. Beiträge zur Kenntnis der menschlichen Neuroglia. Frankfurt 1895.
Whitwell, On the Structure of the Neuroglia. British med. Jour. 12.
Wilson, J. T. On the Closure of the central canal of the spinal cord in the foetal lamb. Transact. Intern. med. Congress Sydney 1892.
Wlassak, R. Die Herkunft des Myelins, Archiv f. Entwicklungsmechanik 1895.
y Cajal S. R. Algunas conjeturas sobre el mecanismo anatómico de la Ideacion, Asociation y Atencion. Madrid 1895 und Arch. f. Anat. 1895.
y Cajal, S. R. A propos de certains elements bipolaires du cervelet avec quelques details noveaux sur l´evolution des fibres cereblleuses. Inter. Monatsschr. F. Anat. U. Phys., Bd 7, 1890.
y Cajal, S. R. Estructura del asta de Amon y fascia dentata. Trabajos leidos ante la Sociedad espanola de historia natural, 1893.
y Cajal, S. R. La retine des vertebres, la Cellule t. 9, 1, 1893 (Dep. 1892).

y Cajal, S. R. Les novelles idees sur la structure du systeme nerveux chez l´homme et les vertebres. Paris 1894.
y Cajal, S. R. Pequeñas comunicaciones al conocimiento del sistema nervioso. La médula espinal de los reptiles. Barcelona 1891.
y Cajal, S. R. Pequenas Contribuciones al Conecimiento del sisterna nervioso. Trab. d. Laborat. histol. de la facultad de medicina de Barcelona. Aug. 1891.
y Cajal, S. R. Sur l´origine et les ramifications des fibres nerveuses de la mole embryonaire. Anatom. Anzeiger, V. Jahrg. No 4. Febr. 1890.
y Cajal, S. R. Sur la structure de l´ecorce cerebrale de quelque mammiferes. La Cellule t. 7, 1891.
y Cajal, S.R. Sur l'origine et les remifications des fibres nerveuses de la moëlle embryonnaire. Anat. Anz. Jahrg. V, 1890, p. 115.
y Cajal. S. Nuevos observaciones sobre la estructura de la médula espinal de los mamiferos. Barcelona, 1890.
y Cajal. S. R. A propos de certaius elements bipolaires du cervelet, avec quelques details sur l´evolution des fibres cerebelleuses. Internat. Monatschr. für Anatomie und Physiologie, Bd7, 1890.
y Cajal S. R., Significacion fisiologica de las expansions protoplasmaticas y nerviosas de la celulas de la sustancia gris. Rivista de ciencias medicas de Barcelona, 1891, Nr. 22 and 23.
Yamagiva, Eine neue Färbung der Neuroglia. Virchows Archiv 160. S. 358. I900.

All references in chronological order (178)

1811
Keuffel, G. G. Th. Über das Rückenmark. Reil´s Archiv, Bd. 10, 1811.

1838
Arnold, Fr. Bemerkungen über den Bau des Hirns und Rückenmarks. Zürich 1838.

1842
Stilling and Wallach. Untersuchungen über die Textur des Rückenmarks. Leipzig 1842, p. 23.

1844
Arnold, Fr. Handbuch der Anatomie. Bd, 1, Freiburg i. Br. 1844.

1846
Virchow, R. Über das granulierte Ansehen der Wandungen der Gehirnventrikel, Zeitschrift für Psychiatrie, 1846

1851
Virchow, R. Über die Erweiterung kleinerer Gefäße. Virchows Archiv III. 1851.

1853

Robin, H. first in Segond, Le systeme capillaire sanguin., Paris 1853, then in Comptes rendus et mém. de la Soc. de Biol. Paris 1855 u. Recherches sur quelques Particularités de la Structure des Capillaires de l'éncephale. Journal de la Physiol. 1859 (S. 543-545).
Virchow, R. Über eine im Gehirn und Rückenmark gefundene Substanz mit der chemischen Reaktion der Cellulose. Arch. f. path. Anat. u. Phys. 1853.

1854
Wagner. R. Neurologische Bemerkungen. Göttinger Nachr. 1854, Nr. 3.

1856
Virchow, R. Gesammelte Abhandlungen. Frankfurt, 1856.

1857
Bergmann, C. Zeitschrift f. rat. Medicin. N. F. VIII. 1857
Bidder, F. and Kupffer, C. Untersuchungen über die Textur des Rückenmarks und die Entwicklung seiner Formelemente, 1857

1859
Clarke , J. L. Philosophical transactions, 1859.
His, W. Beiträge zur Kenntnis der zum Lymphsystem gehörigen Drüsen. Zeitschr. f. wiss. Zool. X. 1859.

1862
His, W. Über die Wurzeln der Lymphgefäße in den Häuten des Körpers und über die Theorien der Lymphbildung. Zeitschr. f. wiss. Zool. XII. 1862.

1863
Henle and Merkel Über die sogenannte Bindesubstanz der Zentralorgane des Nervensystems, Zeitschrift für rationelle Medizin3rd edition, Vol 34 (1863).
His, W. Über das Epithel der Lymphgefäßwurzeln und über die v. Recklinghausenschen Saftkanälchen. Zeitschr. f. wissensch. Zoologie. 1863.

1865
Deiters, O. Untersuchungen über Gehirn und Rückenmark des Menschen und der Säugetiere. Braunschweig, 1865.
His, W. Über ein perivaskuläres Kanalsystem in den nervösen Centralorganen und über dessen Beziehungen zum Lymphsystem. Zeitschr. f. wiss. Zoologie, Bd. XV, 1865.

1869
Roth. Zur Frage der Bindesubstanz in der Großhirnrinde, Virchows Archiv, Vol. 46 (1869).
Eberth, C. J. Über die Blut- u. Lymphgefäße des Gehirns u. Rückenmarks. Virchows Archiv 49. 1870.

1870
Meynerth, Th. Vom Gehirn der Säugetiere. Stricker´s Handbuch der Lehre von den Geweben. Wien 1870, Bd. II.
Obersteiner, H. Über einige Lymphräume im Gehirne. Sitzungsberichte der kais. Akad. d. Wissensch. zu Wien, Bd. 61, Abt. I, 1870.
Riedel, Die perivasculären Lymphgefäße im Zentralnervensystem und der Retina. Arch. f. mikr. Anatomie 1870.

1871
Jastrowitz, Studien über die Encephalitis und Myelitis des ersten Kindesalters. Archiv f. Psychiatrie, Bd. III, 1871.

1872
Golgi, C. Sui gliomi del cervello. Rivista sperim. di Freniatria, 1872. S. Sammelwerk.

1873
Boll, F. Die Histiologie und Histiogenese der nervösen Centralorgane. Berlin 1873.

1874
Boll, F. Die Histologie und Histogenese der nervösen Zentralorgane, Archiv für Psychiatrie, Vol. 4, 1874.
Schwalbe, Handbuch der Augenheilkunde by Gräfe and Sämisch, Vol. I, p. 342, Leipzig, 1874.
Simon, Th. Das Spinnenzellen- und Pinselzellengliom. Archiv für pathol. Anat. u. Physiol. 1874.

1875
Götte, A. Entwicklungsgeschichte der Unke. Leipzig, 1875.
Key, A. und Retzius, G. Studien in der Anatomie des Nervensystems und des Bindgewebes. I. Hälfte, Stockholm 1875.

1876
Hensen Zeitschrift für Anatomie und Entwicklungsgeschichte, Band I, 1876.
Krause, W. Handbuch der menschlichen Anatomie, Bd. I, Allgemeine Anatomie, Hannover 1876.
Waldeyer, W. Über die Entwicklung des Centralkanals im Rückenmark. Archiv f. path. Anat., 1876, Bd. LXVIII.

1877
Frommann, C. Untersuchungen über die normale und pathologische Anatomie des Rückenmarks", Part I, Jena, 1864. Part II, Jena, 1877.
Gaskell, W. H. Über die Wand der Lymphkapillaren (C. Ludwig, Arbeiten aus d. phys. Institut zu Leipzig. 1877.

1879
Stricker and Unger. Untersuchungen über den Bau der Großhirnrinde, Wiener Sitzungsberichte" Vol. 80, 1879.

1881
Balfour, F. M. Handbuch der vergleichenden Embryologie. Übersetzt von C. Vetter. Jena 1881, II. Bd.
Schwalbe, G. Lehrbuch der Neurologie, 2. Bd, 2. Abth. Von Hoffmann´s Lehrb. D. Anat. D. Menschen, 1881.
Schwalbe, G. Lehrbuch der Neurologie. Erlangen 1881.

1882
Ranvier, L. De la névroglie. Comptes rendus de l´Acad. des Sc. Tome 94, 1882, p.1536.

1883
His, W. Über das Auftreten der weißen Substanz und der Wurzelfasern am Rückenmark menschlicher Embryonen. Arch. f. Anatomie 1883.
Ranvier, L. De la névroglie, Archive de Physiologie normale et pathologique, February 15, 1883.

1884
Barnes, On the Development of the posterior fissure of the Spinal cord and the Reduction of the Central Canal in the Pig. Proc. Amer. Acad. arts and sc. 1884.
Vignal, W. Sur le dévelopment des éléments de la moëlle des mammiféres. Archives de Physiol. normale et pathol. T. 1884.

1885
Golgi, Camillo. Sulla fina anatomia degli organi centrali del sistema nervosa. Reggio Emilia, 1885.

1886
Bechterew W., Über einen besonderen Bestandteil der Seitenstränge des Rückenmarks. Arch. f. Anat. und Physiol. Anat. Abt., 1886.
Gierke, Hans. Die Stützzellen des Centralnervensystems. Archiv f. mikrosk. Anat. Bd. 25, 1885 and Bd. 26, 1886.
His, W. Zur Geschichte des menschlichen Rückenmarkes und der Nervenwurzeln. Abh. d. math.-phys. Klasse d. Kgl. Sächs. Ges. d. Wiss., Bd. XIII, 1886.
Lothringer, S. Untersuchungen an der Hypophyse einiger Säugetiere und des Menschen. Archiv f. Mikrosk. Anatomie, Bd 28, 1886.

1887
Mondino, Casimiro. Richerche macro e microscopiche sui centri nervosa, Torino 1887.
Nansen, Fridthof. The Structure and Combination of the Histological Elements of the Central Nervous System. Bergens Museums Aarsberetning for 1886. Bergen 1887.
Virchow, H. Über Zellen in der Substantia gelatinosa Rolando. Reported in Neurol. Centralbl., 1887.

1888
Corning, H. K. Über die Entwicklung der Substantia galatinosa Rolandi beim Kaninchen. Archiv f. mikrosk. Anatomie, Bd. 31, 1888, p. 594.

Magini, Guiseppe. Neuroglia e cellule nervosa cerebrali nei feti. Att d 12. Congresso della Assoc. med. Ital. in Pavia, sett. 1887, Vol1, Pavia 1888.
Petrone, Louis Sur la structure des nerfs cerebro-rachidiens. Intern. Monatsschr. F. Anat. Physiol. Bd 5. 1888
Rohde, E. Histologische Untersuchungen über das Nervensystem vom Amphioxus lanceolatus. Schneider's Zoolog. Beitg., Bd. 2., H. 2, Breslau 1888.
Toldt, C. Lehrbuch der Gewebelehre, 3. Aufl., 1888.

1889
Burckhardt, R. Histologische Untersuchungen am Rückenmarke der Tritonen. Archiv f. mikrosk. Anat., Bd. XXXIV, 1889.
Falzacappa, E. Ricerche istologiche sul midollo spinale. Rendiconto della R. Accademia dei Lincei, Vol. V. 1889, p. 696.
His, W. Die Neuroblasten und deren Entstehung im embryonalen Mark. Abh. d. math. phys. Kl. d. Kgl. s. Ges. d. W. 1889.
Lenhossek, M. v. Untersuchungen über die Entwicklung der Markscheiden und den Faserverlauf im Rückenmark der Maus. Arch f. mikrosk. Anat., Bd. 33, 1889.
Lewis, Bevan. A text book of mental diseases with special reference to the pathological aspects of insanity. London 1889.
van Gieson Laboratory notes of technical methods for the nervous system, New York medical Journ., 1889.

1890
His, W. Histogenese u. Zusammenhang der Nervenelemente. Archiv. f. anat. u. physiol. Anat. Abt. 1890, p. 103.
Kölliker, A. Zur feineren Anatomie des centralen Nervensystems. Zweiter Beitrag: Das Rückenmark. Zeitsch. für wissensch. Zoologie. Bd. LI, 1890.
Lachi, P. Contributo alla istogenesi della nevroglia nel midollo del pollo. Memoria della Soc. Toscana di Scienca natur., Vol. 11, Pisa 1890.
Lenhossek. M. v. Zur ersten Entstehung der Nervenzellen und Nervenfasern bei dem Vogelembryo. Mittheil. Aus dem anatom. Institut im Vesalianum zu Basel, 1890.
Martinotti, Carlo Beitrag zum Studium der Hirnrinde und dem Centralursprung der Nerven. Inernat. Monatschr. für Anatomie und Physiologie, Bd 7, 1890.
Oyarzun, A. Über den feineren Bau des Vorderhirns der Amphibien. Arch. f. mikrosk. Anatomie. Bd35, 1890.
Staderini, Contributo allo studio del tessuto interstiziale di alcuni nervi craniensi dell' uomo. Monitore zoolog. italiano, Anno I, 1890, p. 232.
Weigert, C. Bemerkungen über das Neurogliagerüst des menschlichen Zentralnervensystems, Anatomischer Anzeiger, 1890.
1890Weigert, C. Zur pathologischen Histologie des Neurogliafäsergerüsts, Centralblatt für allg. Path. und path Anat, 1890.
y Cajal, S. R. A propos de certains elements bipolaires du cervelet avec quelques details noveaux sur l´evolution des fibres cereblleuses. Inter. Monatsschr. F. Anat. U. Phys., Bd 7, 1890.

y Cajal, S. R. Sur l´origine et les ramifications des fibres nerveuses de la mole embryonaire. Anatom. Anzeiger, V. Jahrg. No 4. Febr. 1890.
y Cajal, S.R. Sur l'origine et les remifications des fibres nerveuses de la moëlle embryonnaire. Anat. Anz. Jahrg. V, 1890, p. 115.
y Cajal. S. Nuevos observaciones sobre la estructura de la médula espinal de los mamiferos. Barcelona, 1890.
y Cajal. S. R. A propos de certaius elements bipolaires du cervelet, avec quelques details sur l´evolution des fibres cerebelleuses. Internat. Monatschr. für Anatomie und Physiologie, Bd7, 1890.

1891

Hoche, A. Beitrag zur Kenntnis des anatomischen Verhaltens der menschlichen Rückenmarkswurzeln etc. Habilitationsschrift, Heidelberg 1891.
Lachi, P. Contribution à l'histogenèse de la nevrologie dans la moelle épinière du poulet. Arch. it. biol. XV. 1891.
Lachi, Pilade. Contributo alla istogenesi della Nevroglia nel midillo spinale del pollo. Atti della Societa toscana di scienze naturali, resid. In Pisa. Memorie. Vol. 11, 1891.
Lawdowsky, M. Vom Aufbau des Rückenmarkes. Archiv f. mikrosk. Anatomie, Bd. 38, 1891.
Lenhossek, M. v. Zur Kenntnis der Neuroglia des menschlichen Rückenmarkes. Verhandl. d. anat. Gesellsch, 5. Versamml., 1891, Anatom. Anz.
Ramon, Pedro El encefalo de los Reptiles. Trab. d. Laborat. histologia de la facultad de medicina de Zaragoza. Sept. 1891.
Retzius, Gustav. Ueber den Bau der Oberflächenschicht der Grosshirnrinde beim Menschen und bei Säugetieren. Verhandl. D. Biolog. Vereins in Stockholm. Bd 3, 1891.
Retzius, Gustav. Zur Kenntnis der Ependymzellen der Centralorgane. Verhandl. d. Biolog. Vereins in Stockholm Bd 3 (1890-1891); 15 März 1891.
Retzius, Gustav. Zur Kenntnis des centralen Nervensystems von Myxine glutinosa. Biolog. Untersuchungen, N. F. II, Stockholm 1891.
Valenti, G. Contribution à l'histogenèse de la cellule nerveuse et de la nevrologie du cerveau de certains poissons chondrostéiques. Arch. it. biol. XVI. 1891.
Van Gehuchten, A. La structure des centres nerveux. La Moelle epiniere et le cervelet. La Cellule, t. 7, dep. 1. 20 avril 1891.
y Cajal S. R., Significacion fisiologica de las expansions protoplasmaticas y nerviosas de la celulas de la sustancia gris. Rivista de ciencias medicas de Barcelona, 1891, Nr. 22 and 23.
y Cajal, S. R. Pequeñas comunicaciones al conocimiento del sistema nervioso. La médula espinal de los reptiles. Barcelona 1891.
y Cajal, S. R. Pequenas Contribuciones al Conecimiento del sisterna nervioso. Trab. d. Laborat. histol. de la facultad de medicina de Barcelona. Aug. 1891.
y Cajal, S. R. Sur la structure de l´ecorce cerebrale de quelque mammiferes. La Cellule t. 7, 1891.

1892

Gieson, Ira van. A study of the Artefacts of the Nervous System. New York medical Journal, 1892.
Kalius, E. Ueber Neurogliazellen in peripherischen Nerven, Nachrichten von d. k. Gesellsch. d. Wiss. etc. In Göttingen 1892.

Lenhossek, M. v. Beobachtungen an den Spinalganglien und dem Rückenmark von Pristiurus embryonen. Anat. Anzeiger, 7. Jahrg., 1892.
Lenhossek, M. v. Der feinere Bau des Nervensystems im Lichte neuster Forschungen. Fortschritte der Medizin, 1892.
Obersteiner, H. Anleitung beim Studium des Baues der nervösen Centralorgane. 2. Aufl., Wien 1892.
Retzius, Gustav. Zur Kenntnis des Nervensystems von Myxine glutinosa. Biol. Unters. N. F. II, 2. 1892.
Sala y Pons, Cl. Estructura de la médula espinal de los batracios. Barcelona 1892.
Sala y Pons, Cl. Estruetura de la medulla espinal de los batracios. Trab. d. Laborat. histologia de la facultad de medicina de Barcelona. Febr. 1892.
Wilson, J. T. On the Closure of the central canal of the spinal cord in the foetal lamb. Transact. Intern. med. Congress Sydney 1892.

1893
Andriezen, Lloyd. On a system of fiber-cells surrounding the blood-vessels of the brain of Man and Mammals, Internationale Monatsschrift für Anatomie und Physiologie, 1893
Beneke, Über eine Modifikation des Weigert´schen Fibrinverfahrens. Anat. Anz. Jahrg. VIII, 1893, Suppl.
Dogiel, A. S. Neuroglia der Retina des Menschen. Arch. F. mikrosk. Anat. Bd 41, 1893.
Edinger, L. Vorlesungen über den Bau der nervösen Centralorgane, 4. Aufl., 1893
Greppin, L. Ueber die Neuroglia der menschlichen Rinde. Anatom. Anzeiger, Bd9, No 3, Nov. 1893.
Hoffmann, J. Zur Lehre von der Syringomyelie. Deutsche Zeitschr. f. Nervenheilkunde, Bd. 8, 1893.
Kölliker A. v., Handb. d. Gewebelehre, 6. Aufl., Bd. II, 1893.
Michel. Sitz, Ber. D. Würzb. Med. naturwiss. Gesellsch. 14. Jan, 1893.
Paladino, S. Dei limiti precisi tra il nevroglio e gli elementi nervrosi del midollo spinale, R. acad. di Roma, XIX. Fasc. 2, 1893.
Popoff De la névrologie et de sa distribution dans les régions du bulbe et de la protubérance chez l'homme adulte, Arch. de psych., de neurologie et de médecine légales, 1893. vol. 11.
Retzius, Gustav. Studien über Ependym und Neuroglia. Biolog. Untersuchungen, N. F., V, 1893,.
Schäfer, E. A. Quain´s Elements of Anatomy, Vol. 3, P. 1; 10. Edition, 1893.
Van Geruchten, A. Le systeme nerveux de l´homme, Lecous professes a l´Universite de Louvain, 1893
y Cajal, S. R. Estructura del asta de Amon y fascia dentata. Trabajos leidos ante la Sociedad espanola de historia natural, 1893.
y Cajal, S. R. La retine des vertebres, la Cellule t. 9, 1, 1893 (Dep. 1892).

1894
Azoulay, L. Note sur le aspects des cellules nevroglique dans les organs nerveux centraux de l´enfant, Comptes rend. Hebd. D. s. de la Soc. De Biol., 1894, No 9.
Berkley, Henry J. The Neuroglia Cells of the Walls of the middle Ventricle in the adult Dog. Anatom. Anzeiger, Bd 9, No 24 and 25, Aug. 1894.
Golgi, Camillo. Untersuchungen über den feineren Bau des centralen und peripherischen Nervensystems. Aus dem Italienischen übersetzt von Dr. R. Teutscher. Verlag von Gustav Fischer. Jena, 1894.
Kalius, E. Untersuchungen zur Netzhaut der Säugetiere. Anatom. Hefte, herausgeg. Von Merkel Und Bonnet, 1894.

Lenhossék, M. v. Zur Kenntnis des Rückenmarkes der Rochen. In: Beitr. z. Histolog. d. Nervensystems u. d. Sinnesorgane. Wiesbaden 1894.
Lugaro, Ernst. Über die Histogenese der Körner der Kleinhirnrinde. Anatom. Anzeiger, Bd 9, No 23, Aug. 1894.
Prenant, A. Critériums histologiques pour la détermination de la partie persistante du canal épendymaire primitif. Internat. Monatsschrift f. Anatn. u. Physiol., Bd. XI, 1894.
Reinke, Zellstudien, Arch. f. mikr. Anatomie 1894.
Sala y Pons, Cl. La Neuroglia de los Vertebrados. Barcelona 1894.
Schaffer, J. Beiträge zur Kenntnis des Stützgerüstes im menschlichen Rückenmarke. Arch. f. mikr. Anat. Bd. 40, 1894.
Schaffer, J. Die oberflächliche Gliahülle und das Stützgerüst des weißen Rückenmarkmantels. Anat. Anz., Bd. IX, 1894.
Van Gehuchten, A. La Neuroglie dans le cervelet de l´homme. Bibiolgraphie anatomique, Année 2, No 4, 1894.
y Cajal, S. R. Les novelles idees sur la structure du systeme nerveux chez l´homme et les vertebres. Paris 1894.

1895
Paladino, S. Sur les limites précises entre la névroglie et les éléments nerveux dans la moelle epiniere, et sur quelques-unes des questions histophysiologi ques qui s'y rapportent. Ach. ital. de Biologie XXII. 1895.
Weigert, C. Beiträge zur Kenntnis der menschlichen Neuroglia. Frankfurt 1895.
Wlassak, R. Die Herkunft des Myelins, Archiv f. Entwicklungsmechanik 1895.
y Cajal S. R. Algunas conjeturas sobre el mecanismo anatómico de la Ideacion, Asociation y Atencion. Madrid 1895 und Arch. f. Anat. 1895.

1896
Pellizzini, S. B. Sulla struttura e sull' origine delle granulazioni ependimali. Contributo all Histologia e patologia della nevrologia. Riv. sper. Frenatria 22. 1896.
Simon, Ch. Recherches sur la cellule des ganglions sympatbiques des Hirudinées. Internationale Monatsschr. f. An. u. Phys. XIII. 1896.
Spuler, A. Beiträge zur Histologie und Histogenese der Binde- und Stützsubstanz. Anat. Hefte 1896.
Ströbe, Über Struktur pathologischer Neurogliawucherungen. Zentralblatt f. allg. Path. u. path. Anat. 1896.

1897
Apáthy, Stephan. Das leitende Element des Nervensystems und seine topographischen Beziehungen zu den Zellen. Mitt. d. zool. Station XII. 1897.
Flemming, W. Über die Entwicklung der kollagenen Bindegewebsfibrillen bei Amphibien und Säugetieren. Arch. f. Anat. u. Phys. anat. Abt. 1897.
Gardner, M. Zur Frage über die Histogenese des elastischen Gewebes, Biol. Zentralbl. 1897.
Held, H. II. Abhandlung über Nervenzellenstrukturen. Arch. f. Anatomie 1897, Suppl. S. 275 Anm.
Reinke, F. Beiträge zur Histologie des Menschen II. Arch. f. mikr. Anatomie. 50. 1897.

Robertson, W. F. Note on Weigerts theory regarding the structure of the Neuroglia. Journ. of Mental Science I897.

1898

Binswanger und Berger, Beiträge zur Kenntnis der Lymphzirkulation in der Großhirnrinde. Virchows Archiv 152. 1898.

Kultschitzky. Über eine Färbungsmetbode der Neuroglia, Anatomischer Anzeiger, Vol 8, 1898.

Rieder. Beiträge zur Histologie und pathol. Anatomie der Lymphgefäße und Venen. Zentralbl. f. allg. Path. 1898.

1899

Brodmann, K. Über den Nachweis von Astrocyten mittelst der Weigertschen Gliafärbung. Jenaische Zeitschr. f. Naturw. XXXIII. I899. 1.

Müller, E. Studien über Neuroglia. Arch. f. mikr. Anatomie. 55. 1899.

Nissl, Fr. Über einige Beziehungen zwischen Nervenzellerkrankungen und gliösen Erscheinungen bei verschiedenen Psychosen. Arch. f. Psychiatrie 1899.

Storch, Über die pathologisch-anatomischen Vorgänge am Stützgerüst des Zentralnervensystems. Virchows Archiv. 157. 1899.

1900

Aguerre. Untersuchungen über die menschliche Neuroglia. Arch. f. mikr. Anat. 56. 1900.

Bethe, Albrecht. Über die Neurofibrillen in den Ganglienzellen von Wirbeltieren und ihre Beziehungen zu den Golginetzen. Archiv für mikrosk. Anat. 55. 1900.

Holmgreen, Emil. Studien in der feineren Anatomie der Nervenzellen. Anat. Hefte XV. 1900.

Marinesco, Du rôle de la nevroglie dans l'évolution des inflammations. 2. internat. med. Congress. Paris 1900. (n. d. Jahresbericht v. Schwalbe. VI. 1900.)

Obersteiner, H. Zur Histologie der Gliazellen in der Molekularschichtder Großhirminde. Arb. a. d. Institut. 1900.

Studnička. Untersuchungen über den Bau des Ependyms der nervösen Zentralorgane. Anatomische Hefte. Bd. XV. 1900.

Yamagiva, Eine neue Färbung der Neuroglia. Virchows Archiv 160. S. 358. I900.

1901

Capobianco. Della partecipazione mesodermica nelle genesi della neuroglia cerebrale. Monit. zool. ital. XII. 1901 u. Arch. ital. biol. 1902.

Dimitrova, Z. Recherehes sur la structure de Ia Glande pinéale. Le Nevraxe. II. 1901.

Huber, G. Carl Studies of Neuroglia. The American Journal of Anatomy. I. 1901.

1902

Fuchs, H. Über das Ependym. Verb. d. anatom. Gesellschaft zu Halle 1902.

Hatai, Shinkishi. On the origin of nevrologia tissue from the mesoblast. Journ. of comp. neurology XII. 1902.

Held, Hans Über den Bau der grauen und weißen Substanz I. Arch. f. Anatomie. 1902.

Pranter, V. Zur Färbung der elastischen Fasern. Zentralbl. für allg. Path. u. path. Anat. XIII. 1902.

1904
Sala y Pons,Cl. La Neuroglia de los Vertebrado, Madrid, 1904.

Not dated in the reference list
Arndt Zur Histologie des Gehirns, Archiv für Psychiatrie, Volume III,.
Brissaud, neurologique Vol. 2.
Colella. Sur l'histogenèse de la névrologie dans la moelle épinière, Archives ital. de Biologie, Vol. 20.
Eurich, Studies on the Neuroglia, Brain 1897.
Greeff Die Spinnenzellen — Neurogliazellen — in Sehnerv und der Retina. Archiv für Augenheilkunde, Vol. 29.
Hardesty, lrving. The Neuroglia of the Spinal Cord of the Elephant with some preliminary Observation upon the Developement of Neuroglia Fibres. American Journal of Anatomy II. Nr. I.
Kure, S. Über die Beziehungen der Glia zu den Gefäßen. Neurologia I.
Lissauer, Arch. für Psych. Vol. 17, Book 2., p. 12, Sept. ed.
Renault, Insertion sous forme de revêtement épithélial continu des pieds des fibres neurogliques sur la limitante marginale d'un nevraxe adulte. Comptes rend. hebdom. des sc. T. 126.
Rohde, Ganglienzellen und Neuroglia, Archiv für mikroskopische Anatomie], Vol. 42.
Whitwell, On the Structure of the Neuroglia. British med. Jour. 12.
